# Therapeutic and immunomodulatory potential of pazopanib in malignant phyllodes tumor

**DOI:** 10.1038/s41523-022-00413-1

**Published:** 2022-04-01

**Authors:** Dave Yong Xiang Ng, Zhimei Li, Elizabeth Lee, Jessica Sook Ting Kok, Jing Yi Lee, Joanna Koh, Cedric Chuan-Young Ng, Abner Herbert Lim, Wei Liu, Sheng Rong Ng, Kah Suan Lim, Xi Xiao Huang, Jing Han Hong, Peiyong Guan, Yirong Sim, Aye Aye Thike, Nur Diyana Md Nasir, Shang Li, Puay Hoon Tan, Bin Tean Teh, Jason Yongsheng Chan

**Affiliations:** 1 Laboratory of Cancer Epigenome, National Cancer Centre, Singapore, Singapore; 2 Duke-NUS Medical School, Singapore, Singapore; 3 Division of Medical Oncology, National Cancer Centre, Singapore, Singapore; 4 Cancer Discovery Hub, National Cancer Centre, Singapore, Singapore; 5 Institute of Molecular and Cellular Biology, Singapore, Singapore; 6 Laboratory of Biodiversity Genomics, Genome Institute of Singapore, Singapore, Singapore; 7 Department of Breast Surgery, Division of Surgical Oncology, National Cancer Centre, Singapore, Singapore; 8 Department of Breast Surgery, Singapore General Hospital, Singapore, Singapore; 9 SingHealth Duke-NUS Breast Centre, Singapore, Singapore; 10 Division of Pathology, Singapore General Hospital, Singapore, Singapore

**Keywords:** Targeted therapies, Cancer models

## Abstract

Malignant phyllodes tumors (PT) are rare aggressive fibroepithelial neoplasms with high metastatic potential and lack effective therapy. We established a patient-derived xenograft (PDX) and cell line model (designated MPT-S1) of malignant PT which demonstrated clinical response to pazopanib. Whole exome sequencing identified somatic mutations in *TP53*, *RB1*, *MED12,* and *KMT2D*. Immunohistochemistry and genomic profiles of the tumor, PDX and cell line were concordant. In keeping with clinical observation, pazopanib reduced cell viability in a dose-dependent manner and evoked apoptosis, and led to significant abrogation of in vivo tumor growth. Whole transcriptomic analysis revealed that pazopanib decreased expression of genes involved in oncogenic and apoptosis signaling. We also observed decreased expression of *ENPP1*, with known roles in cancer invasion and metastasis, as well as STING pathway upregulation. Accordingly, pazopanib induced micronuclei formation, and evoked phospho-TBK1 and PD-L1 expression. In an additional cohort of malignant PT (*n* = 14), six (42.9%) showed comparable or higher levels of *ENPP1* relative to MPT-S1, highlighting its potential role as a therapeutic target. In conclusion, we established MPT-S1, a new PDX and cell line model, and provided evidence for the clinical efficacy of pazopanib in malignant PT.

## Introduction

Phyllodes tumors (PT) are rare fibroepithelial neoplasms, accounting for less than 1% of all breast tumors in Western countries and up to 7% amongst Asian populations^1,2^. Histologically, PTs are biphasic and are characterized by a leaf-like morphology where clefts are surrounded by a hypercellular stromal component. Histopathological characteristics including stromal cellularity, mitotic activity, atypia, stromal overgrowth, and tumor borders are used to classify PT into three categories: benign, borderline, and malignant^2^. Benign PT constitute the majority and harbor a lower risk of recurrence compared to malignant PT^3^.

On the other hand, malignant PTs are known to be aggressive in behavior and demonstrate clear metastatic potential^4^. Furthermore, malignant PTs may harbor heterologous elements resembling osteosarcoma, chondrosarcoma, or other sarcomas, which may confer a more aggressive phenotype^5^. For localized malignant PT, the primary modality of treatment is surgical wide excision with or without adjuvant radiation therapy. Despite adequate local treatment, however, distant metastasis has been reported to occur in approximately a quarter of malignant PTs^6,7^. Upon systemic dissemination, no established standard therapy currently exists, and malignant PTs are known to be generally unresponsive to conventional chemotherapy or radiation therapy. The employment of drug treatment for malignant PTs remains uncertain and requires examination by the basis of individual cases due to the lack of prospective trials for efficacious chemotherapy agents^8^.

Patient-derived tumor xenograft models and cell lines may be useful tools in the identification of effective therapies that mirror a patient’s treatment trajectory in real-time. To date, however, no PT xenografts and cell line models have been established for this purpose^9–13^.

In this study, we established a new patient-derived mouse xenograft and cell line model of malignant PT (MPT-S1). Furthermore, we characterized the genomic profile of this model via whole exome sequencing and transcriptomic profiling, and examined mechanisms underlying its therapeutic vulnerability to pazopanib.

## Results

### Clinical case presentation

**Fig. 1:**
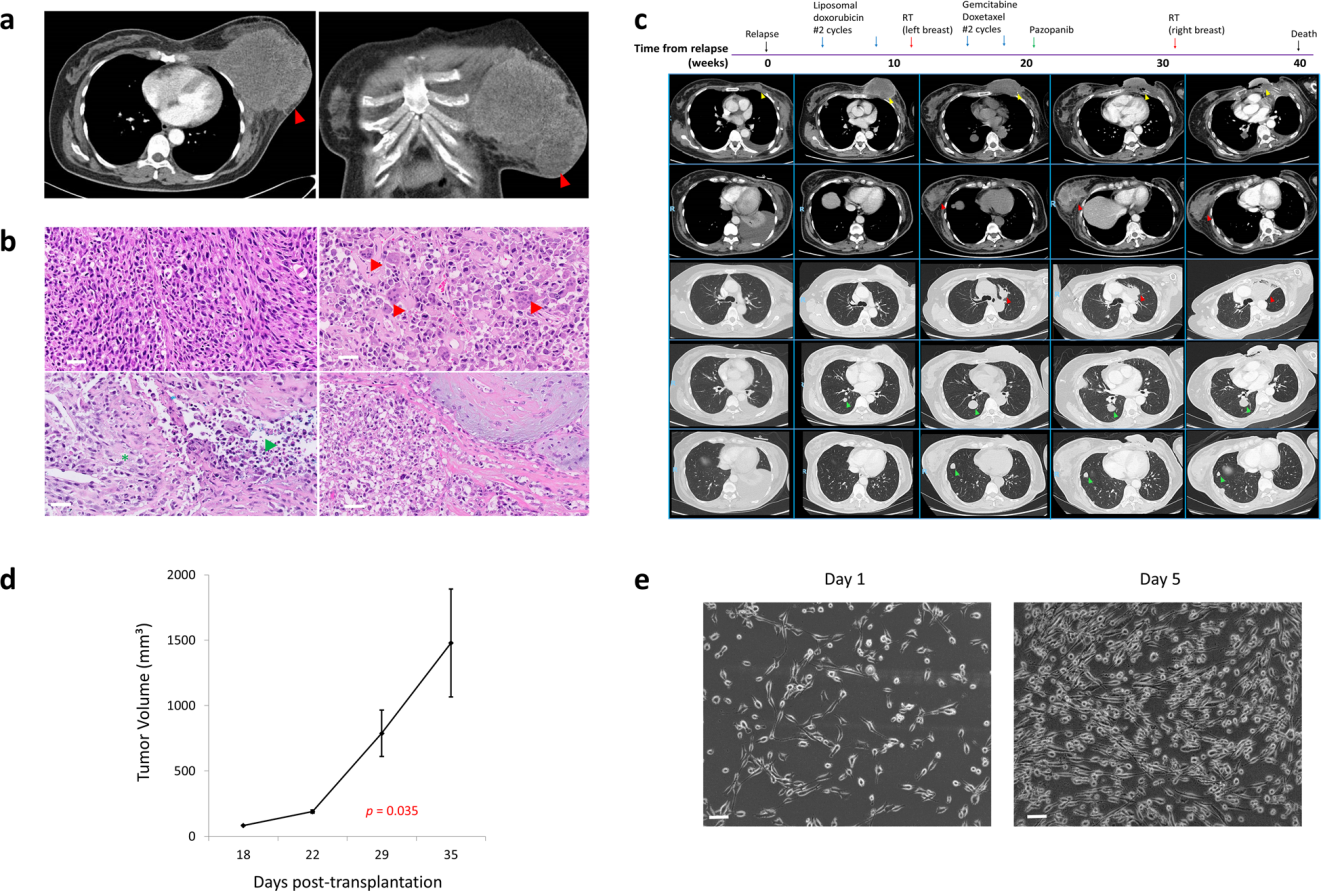
Establishment of patient-derived xenograft and cell line MPT-S1. **a** Images of breast tumor on CT imaging (red arrow). **b** Representative H&E images (20X magnification) of malignant PT with osteoclast-like multi-nucleated giant cells (red arrowhead), epithelioid areas (green asterisk), necrosis (green arrowhead), and myxoid components (bottom right) (scale bar: 50 µm). **c** Clinical course of the patient from time of relapse till death. **d** Growth characteristics of the xenograft in NSG mice. **e** Morphology and growth characteristics of MPT-S1 cell line (scale bar: 20 µm).

A 51-year old Chinese woman was diagnosed with malignant PT of the left breast. She had presented with an enlarging and painful lump on the left breast, which grew in size over a 1-year period. CT imaging showed a large heterogeneous lobulated soft tissue mass arising from the left breast and involving the underlying chest wall, without nodal or distant metastasis (Fig. 1a). She then underwent simple mastectomy & axillary clearance with chest wall reconstruction. Histology showed a high grade malignant tumor composed of markedly pleomorphic spindle cells. Prominent interspersed osteoclast-like multinucleated giant cells and eosinophilic osteoid-like material were seen. In addition, malignant cartilaginous foci were evident (Fig. 1b). Tumor cells stained positive for p63 and showed a high proliferative index on Ki-67 staining. Cytokeratin (AE1/3, MNF116), EMA and GATA3 stains were negative. A month from surgery, the patient developed local recurrence and new lung metastases. Biopsy of the chest wall mass revealed a high-grade malignant neoplasm with similar morphology to the original tumor, including the presence of scattered osteoclast-like multinucleated giant cells. The patient continued to progress despite receiving two cycles of liposomal doxorubicin (40 mg/m^2^) followed by two cycles of gemcitabine plus docetaxel. Palliative radiotherapy was administered in the course of treatment which led to good local responses and symptom relief. Extrapolating from data supporting the potential efficacy of pazopanib (a multitargeted tyrosine kinase inhibitor) in metastatic soft tissue sarcomas after failure of chemotherapy^14^, pazopanib was commenced at 400 mg and escalated to 800 mg daily. Remarkably, this achieved tumor shrinkage in most lung nodules as best response. Unfortunately, the patient developed progression of disease 18 weeks later and passed away at week 40 from relapse (Fig. 1c).

### Establishment of MPT-S1 xenograft

To establish an appropriate preclinical model, a tumor xenograft was established in NSG mice post-inoculation of the patient’s metastatic PT sample. The tumor volume in inoculated mice increased significantly from week 4 onwards (83.6 mm^3^ at week 3, 189.5 mm^3^ at week 4, 788.2 mm^3^ at week 5, 1479.2 mm^3^ at week 6; *p* = 0.035) (Fig. 1d).

### Establishment of MPT-S1 cell line

Tumor cells were derived via enzymatic dissociation of the xenograft. Morphologically, the cells were elongated and spindle-shaped, with doubling time of approximately 2–3 days (Fig. 1e). The cell line remained in continuous culture beyond 6 months from the time of first derivation and was authenticated using STR profiling (Supplementary Table 1).

### Morphologic and genome characterization of MPT-S1 xenograft and cell line

**Fig. 2:**
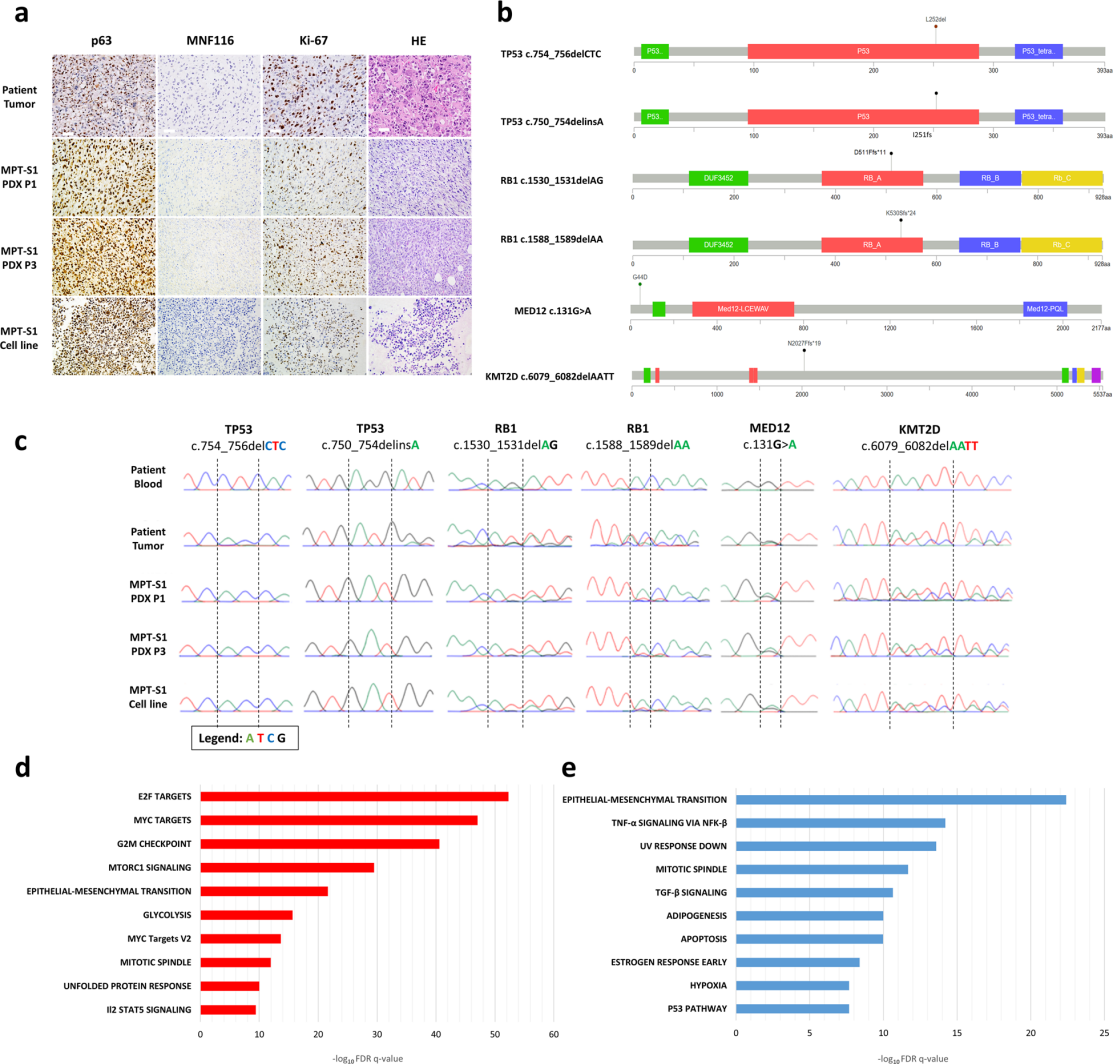
Morphologic and genomic characterization of patient-derived xenograft (PDX) and cell line. **a** Immunohistochemistry (IHC) profiles of the PDX and cell line were positive for p63 and Ki-67 staining which were concordant with the patient’s original tumor sample (scale bar: 50 µm). **b** Somatic mutations in *MED12*, *TP53*, *RB1*, and *KMT2D* identified on whole exome sequencing and (**c**) verified on Sanger sequencing. Whole transcriptomic sequencing and gene set enrichment analysis identified significant (**d**) upregulated and (**e**) downregulated pathways within the tumor as compared to matched normal component.

We then characterized the immunohistochemistry (IHC) profile of the PDX model and cell line using a panel of markers (p63, Ki-67, and MNF116). The IHC profiles of the PDX model and MPT-S1 had positive p63 and Ki-67 staining consistent with the patient’s tumor sample (Fig. 2a). To understand the genetic alterations in our phyllodes models, we performed whole exome sequencing of the tumor tissue sample and matched normal blood. A total of 41 missense, 1 nonsense, 13 silent, and 14 indel somatic mutations were observed (Supplementary Table 2). We further investigated and validated mutations in 4 genes, *MED12* (c.131 G > A), *TP53* (c.754_756delCTC and c.750_754delinsA), *RB1* (c.1530_1531delAG and c.1588_1589delAA) and *KMT2D* (c.6079_6082delAATT), using Sanger sequencing. These mutations were concordant across the patient’s tumor, PDX and cell line (Fig. 2b, c). In an exploratory analysis, whole transcriptomic sequencing and gene set enrichment analysis identified significantly deregulated genes and pathways within the tumor as compared to matched normal component. The top three upregulated pathways include E2F targets, MYC targets, and G2M checkpoint, while the top three downregulated pathways include epithelial-mesenchymal transition, TNF-α signaling via NFk-β and UV response (Fig. 2d, e and Supplementary Tables 3–7).

### In vitro and in vivo response to chemotherapeutic agents and tyrosine kinase inhibitors

**Fig. 3:**
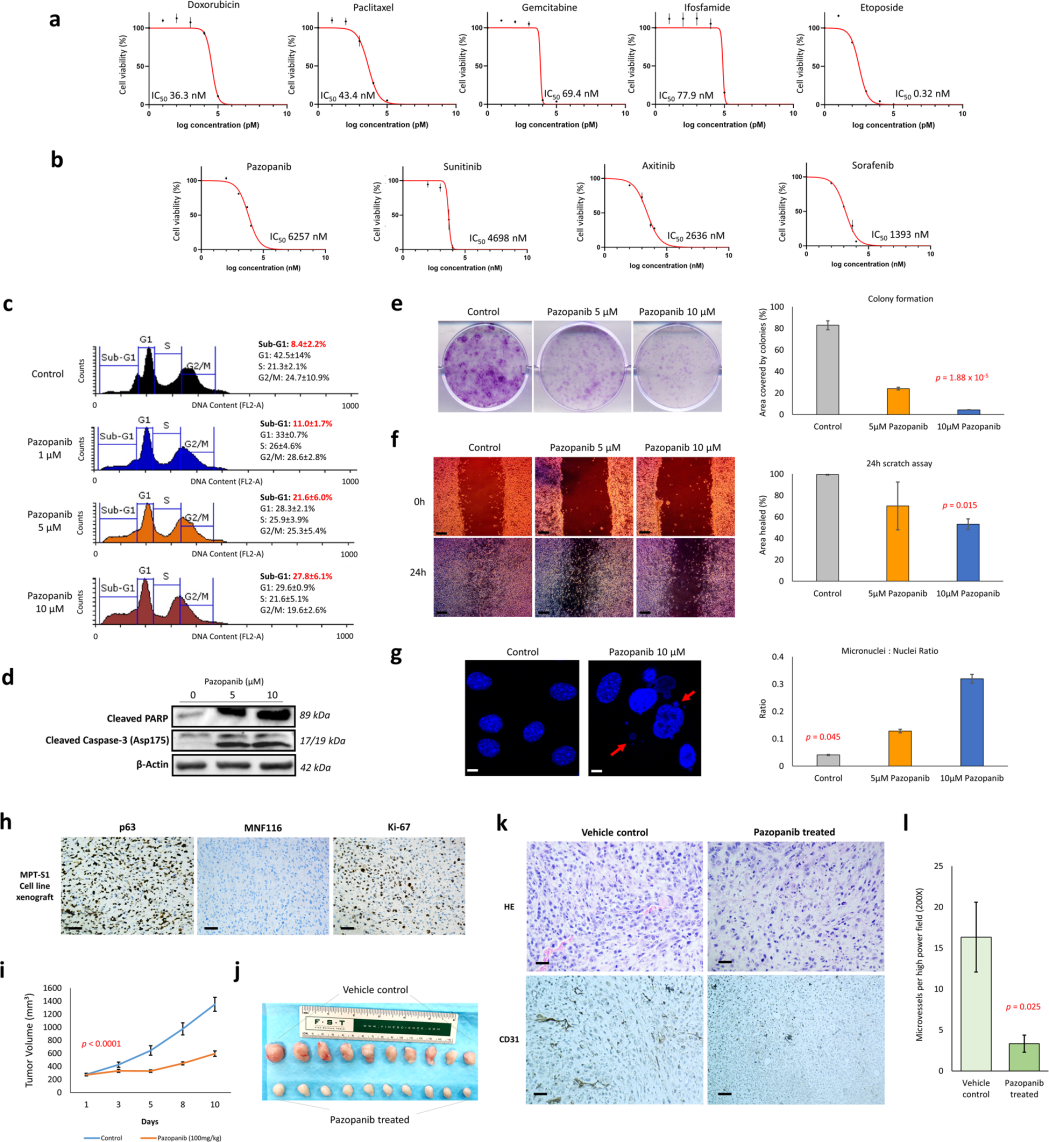
Response to chemotherapeutic agents and tyrosine kinase inhibitors. **a**, **b** In the patient-derived cell line, treatment with several chemotherapeutic drugs and tyrosine kinase inhibitors resulted in reduced viability in a dose-dependent manner. **c** Pazopanib evoked a dose-dependent increase in the sub-G1 cell fraction (*p* = 0.002), **d** induced cleavage of PARP and caspase-3, **e** reduced clonogenicity, **f** decreased cell migration (scale bar: 100 µm) and **g** induced the formation of micronuclei (scale bar: 5 µm). **h** The resultant re-transplanted cell line xenograft retained the immunophenotype of the original PDX and primary tumor (scale bar: 50 µm). **i**, **j** Pazopanib inhibited tumor growth in our PDX model and **k**, **l** reduced microvessel density (mean: 16.3 ± 3.3 versus 4.3 ± 1.0, *p* = 0.025) (scale bar: 50 µm). All drug treatments were performed in triplicate, and results are represented by mean values and standard deviations.

A panel of five chemotherapy drugs: doxorubicin, paclitaxel, gemcitabine, ifosfamide, and etoposide were evaluated for their in vitro effects on the MPT-S1 cell line. Interestingly, despite in vivo primary chemoresistance as observed in the patient, all the chemotherapeutic drugs including doxorubicin evoked a potent reduction in cell viability in a dose-dependent manner, with IC_50_s ranging from 0.32 nM to 77.9 nM (Fig. 3a). To investigate the effects of tyrosine kinase inhibitors (TKI), four different TKIs (pazopanib, sunitinib, axitinib, and sorafenib) used for the treatment of various soft tissue sarcomas were tested. Their known targets are listed in Supplementary Table 8. Similar to chemotherapeutic agents, TKIs decreased viability of the MPT-S1 cells in a dose-dependent manner, although they were less potent based on IC_50_ - pazopanib, sunitinib, axitinib, and sorafenib showed IC_50_ values of 6257 nM, 4698 nM, 2636 nM, and 1393 nM, respectively (Fig. 3b).

Activity of Pazopanib was similarly observed in four other patient-derived primary malignant phyllodes cell lines established using tumor samples from our previous study (morphological features and in vivo growth characteristics summarized in Supplementary Fig. 1)^15^. To further characterize the inhibitory effects of pazopanib, treatment of the cell line with the drug for 72 h at concentrations of 1 µM, 5 µM, and 10 µM resulted in an increasing sub-G1 population of 11.0%, 21.6%, and 27.8%, respectively (*p* = 0.002) (Fig. 3c). Pazopanib induced cleavage of PARP and caspase-3, indicating programmed cell death by apoptosis (Fig. 3d) and reduced clonogenicity (Fig. 3e). Cell migration was inhibited as evident from the scratch assay, as treatment with 5 µM and 10 µM pazopanib resulted in an average of 70.2% and 53.2% “healed” scratches, respectively, as compared to mock treatment control (99.6%, *p* = 0.015) (Fig. 3f). Furthermore, pazopanib induced the formation of micronuclei indicating a mode of action by causing chromosomal instability, with micronuclei-to-nuclei ratio scores of 0.04, 0.13, and 0.32 for mock treatment control, 5 µM, and 10 µM pazopanib treatment, respectively (*p* = 0.045) (Fig. 3g).

To evaluate the inhibitory effects of pazopanib in vivo, daily oral administration of pazopanib was performed in MPT-S1 PDX models (*n* = 10 per group) at a dosage of 100 mg/kg and tumor growth was measured thrice weekly for 10 days. The resultant re-transplanted cell line xenograft retained the immunophenotype of the original PDX and primary tumor (Fig. 3h). Pazopanib treatment led to a significant reduction in tumor growth with the resulting mean tumor volumes of the pazopanib treatment group at 594.51 mm^3^ and vehicle control group at 1350.27 mm^3^ (*p* = 1.85^−6^, Fig. 3i). Gross examination showed that the pazopanib-treated tumors were relatively pale and avascular (Fig. 3j). Histological examination demonstrated that pazopanib significantly reduced microvessel density (mean: 16.3 ± 3.3 versus 4.3 ± 1.0, *p* = 0.025) (Fig. 3k, l).

### Downregulation of genes involved in oncogenic and apoptosis signaling pathways by pazopanib

**Fig. 4:**
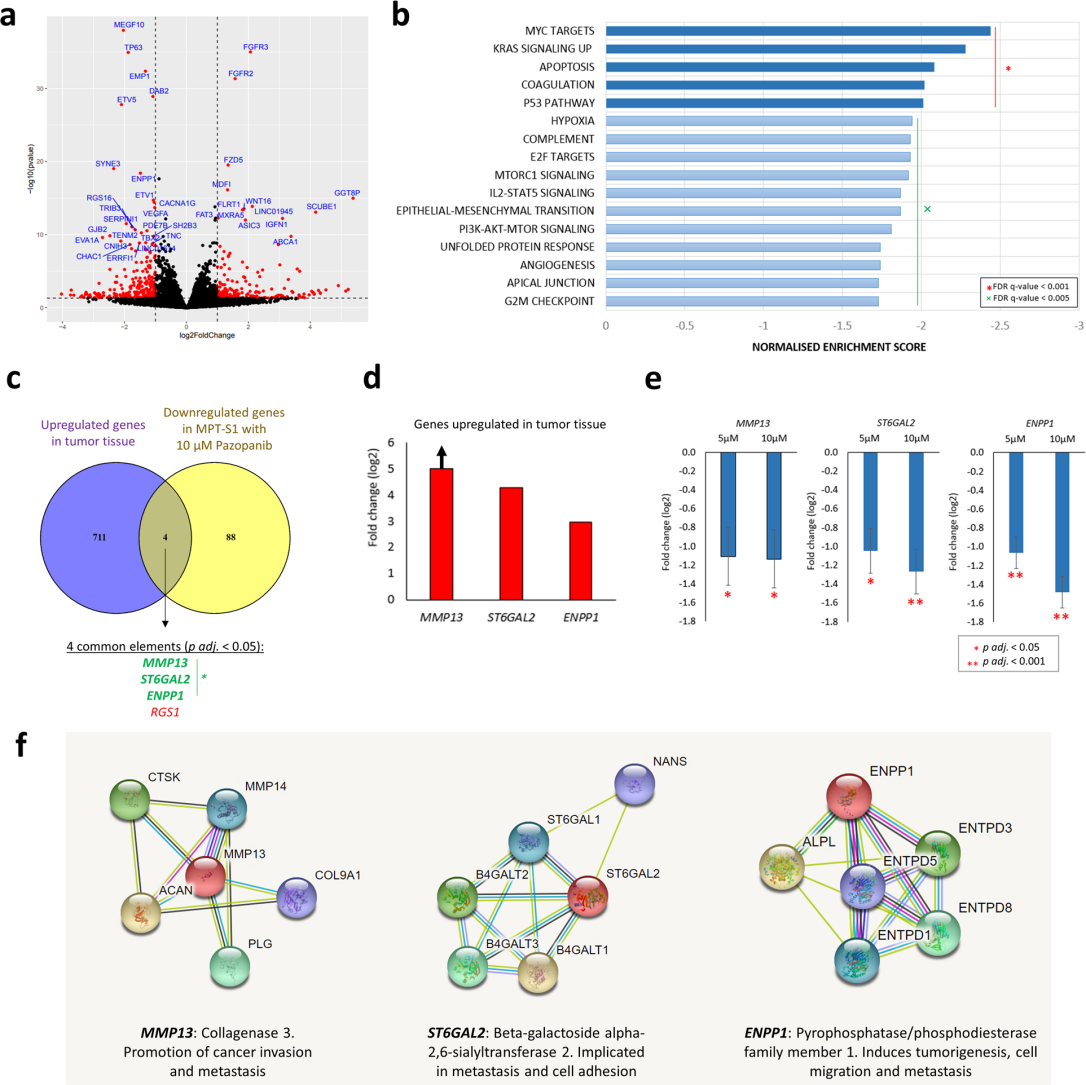
Downregulation of genes involved in oncogenic and apoptosis signaling pathways by pazopanib. **a** Volcano plot showing significant differentially-expressed genes after treatment of MPT-S1 cell line with pazopanib (10 µM). **b** Gene set enrichment analysis highlighted top-scoring downregulated gene sets in oncogenic and apoptosis signaling pathways. **c** Overlap of upregulated genes in the tumor tissue and downregulated genes in the cell line by pazopanib identified genes involved in cancer invasion and metastasis (*MMP13*, *ST6GAL2*, and ENPP1). **d** *MMP13*, *ST6GAL2*, and *ENPP1* genes are upregulated in tumor tissue compared to adjacent normal tissue. **e** Downregulation of *MMP13*, *ST6GAL2,* and *ENPP1* in the cell line following treatment with pazopanib. **f** Protein interaction network of MMP13, ST6GAL2, and ENPP1 and their putative role in cancer development.

Whole transcriptomic sequencing followed by gene set enrichment analysis demonstrated that treatment of the MPT-S1 cell line with pazopanib at 10 µM evoked significant downregulation of genes involved in oncogenic and apoptosis signaling pathways. Downregulation of angiogenesis and hypoxia signaling pathways supported the anti-angiogenic effects observed in vivo (Fig. 4a, b). Leading edge analysis was also performed to examine the genes that contributed the most to the enrichment signal of a given gene set’s core enrichment (leading edge) – this identified six significantly downregulated genes (*F3, TIMP1, VEGFA, CDKN1A, LDHA, CD44*) in response to pazopanib treatment, several of which are relevant to angiogenesis and hypoxia signaling pathways (Supplementary Table 9). Interestingly, *FGFR2* (log2-foldchange 1.57, adjusted *p* = 1.59^−28^) and *FGFR3* (log2-foldchange 2.07, adjusted *p* = 6.87^−32^) were among significantly upregulated genes following pazopanib treatment at 10 µM, though their clinical relevance remains unclear. Similar results were observed for pazopanib treatment at 5 µM (Supplementary Tables 10-11 and Supplementary Fig. 2). Overlap of upregulated genes in the tumor tissue with downregulated genes in the cell line treated with pazopanib identified three genes involved in cancer invasion and metastasis (*MMP13*, *ST6GAL2*, and *ENPP1*) (Fig. 4c). Of note, *MMP13* was specifically expressed within the tumor and not in the normal component; *ST6GAL2* and *ENPP1* were highly expressed in tumor tissue compared to adjacent normal tissue (log2-foldchange 4.26 and 2.94, respectively) (Fig. 4d). Treatment with pazopanib significantly reduced the expression of *MMP13* (10 µM: log2-foldchange −1.13, *p* = 1.45^−02^; 5 µM: log2-foldchange −1.11, *p* = 2.24^−02^), *ST6GAL2* (10 µM: log2-foldchange −1.27, *p* = 2.92^−05^; 5 µM: log2-foldchange −1.05, *p* = 1.71^−03^) and *ENPP1* (10 µM: log2-foldchange −1.49, *p* = 6.95^−16^; 5 µM: log2-foldchange −1.06, *p* = 8.89^−18^) (Fig. 4e). For these genes, their protein interaction network was generated from the STRING database^16^. In general, these genes have been implicated in cancer progression and metastasis (Fig. 4f).

### Upregulation of STING pathway and PD-L1 by pazopanib

**Fig. 5:**
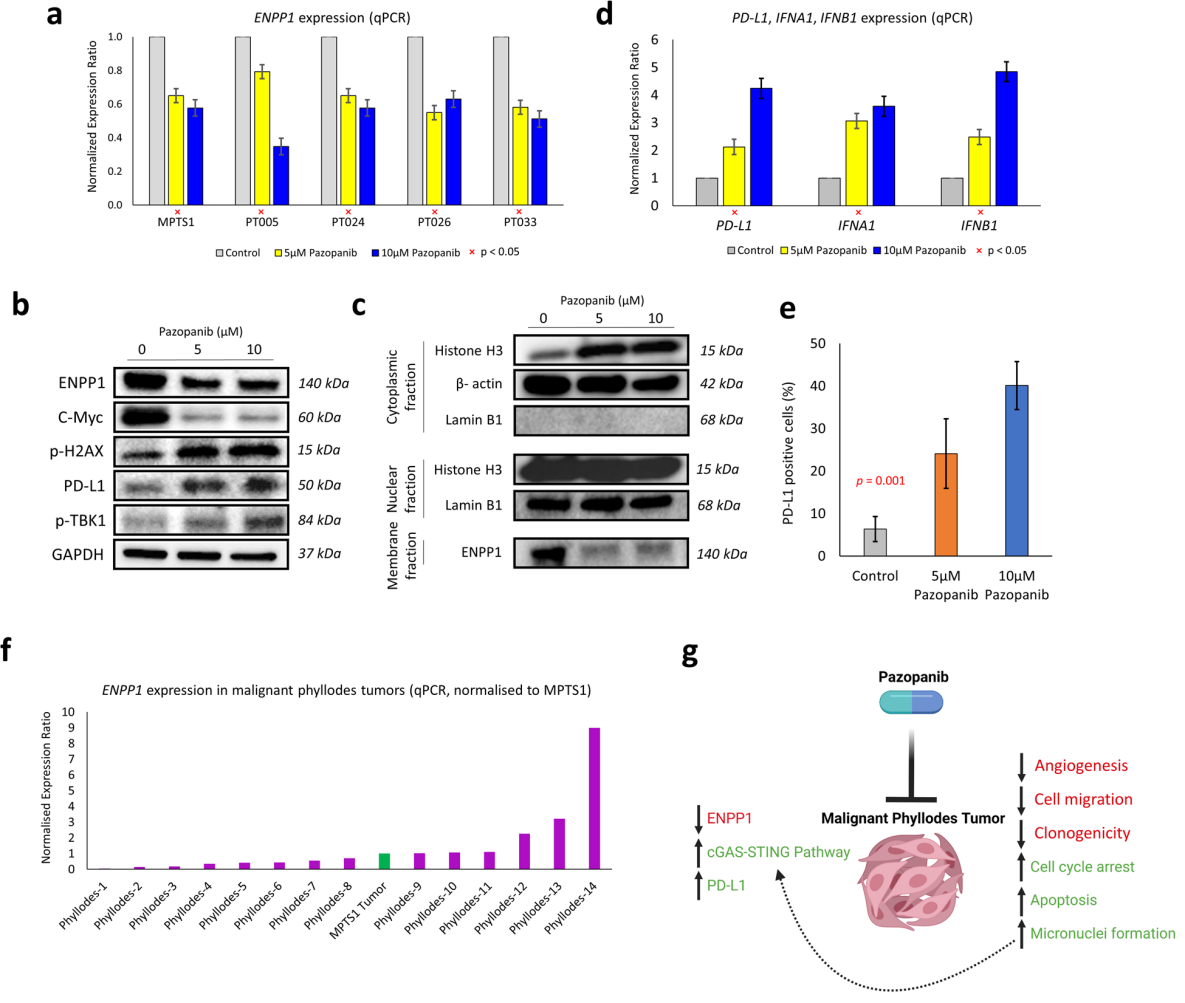
Upregulation of STING pathway and PD-L1 by pazopanib. **a** Downregulation of *ENPP1* expression by pazopanib across PT cell lines. **b** Western blot demonstrating decreased protein expression of ENPP1 and C-Myc, while phophos-H2AX, PD-L1, and phospho-TBK1 were increased. **c** On cellular fractionation, ENPP1 was shown to be significantly downregulated in the membrane fraction, while cytosolic DNA levels, detected using histone H3, increased following pazopanib treatment. **d** Upregulation of *PD-L1*, *INFA1*, and *IFNB1* gene expression by pazopanib. **e** Similarly on flow cytometry, PD-L1 positive cells increased after pazopanib treatment. **f** Relative expression of *ENPP1* across malignant phyllodes tumors, normalized against the MPT-S1 tumor sample. **g** Schematic depicting our main findings on the possible effects of pazopanib on malignant phyllodes tumors (created with BioRender.com).

We selected *ENPP1* for further downstream validation given its recently identified role in immune evasion through modulation of the STING pathway, and its ability to potentiate response to checkpoint immunotherapy when inhibited^17^. We confirmed the downregulation of *ENPP1* expression by pazopanib in MPT-S1 and the four other patient-derived primary malignant phyllodes cell lines using qPCR (Fig. 5a). Western blot analyses demonstrated decreased protein expression of ENPP1 (Fig. 5b and Supplementary Fig. 3). Additionally to corroborate this finding, Western blot analysis was performed on the cell plasma membrane fraction, where ENPP1 is specifically enriched, confirming striking downregulation in response to pazopanib treatment. Pazopanib decreased C-Myc protein expression levels, increased phophos-H2AX, PD-L1, and phospho-TBK1. Cytosolic DNA levels, detected using histone H3, increased following pazopanib treatment (Fig. 5b, c). Using qPCR, pazopanib treatment of MPT-S1 increased expression of *PD-L1*, *INFA1,* and *IFNB1* in a dose-dependent manner (Fig. 5d). Similarly using flow cytometry, PD-L1 positive cells were increased after pazopanib treatment (Fig. 5e).

Treatment of the MPT-S1 cell line with a commercially available ENPP1 inhibitor (ENPP1-IN-1) similarly decreased cell viability in a dose-dependent manner, and was accompanied by reduction of ENPP1 and C-Myc protein levels. We also observed an upregulation of phospho-TBK1 levels. Treatment with other TKIs (sorafenib 1.5 µM; sunitinib 5 µM; axitinib 2.5 µM) however, did not reduce ENPP1 levels (Supplementary Fig. 4).

Finally, we examined the gene expression of *ENPP1* in an additional cohort of malignant PT (*n* = 14) relative to the MPT-S1 tumor sample. Six samples (42.9%) showed comparable or higher levels of *ENPP1* relative to MPT-S1, suggesting that *ENPP1* may be an attractive therapeutic target in a larger cohort of malignant PT (Fig. 5l, m).

## Discussion

We examined in this study the prospects of employing PDX and cell line models to project patient response to therapy for malignant PT. Our MPT-S1 PDX and cell line model recapitulated the genomic profiles of the patient’s tumor as well as clinical response to pazopanib. Using whole transcriptomic sequencing, we identified the oncogenic pathways and genes that drove PT progression, which were suppressed by pazopanib treatment. In addition, in this model, functional assays demonstrated that pazopanib may promote STING pathway activation and PD-L1 expression, implying prospective synergies with checkpoint immunotherapy.

Cell line and xenograft models are valuable preclinical models for evaluating new drug candidates. However, such models for PT remain lacking to date. Takamoto et al. described a mouse model of human PT by transplanting syngeneic mammary gland cells expressing mutant *HRAS*. The choice of *HRAS* mutation is peculiar as the gene is not typically implicated in PT formation. Most importantly, the study did not provide data that the mouse model could reflect the therapeutic susceptibilities of human PT^18^. Comparatively, we managed to establish both patient-derived cell line and xenograft models representative of the malignant PT. Extensive characterization of the cell line and xenograft tumors carried out via IHC profiling, whole exome sequencing and whole transcriptomic analysis confirmed that our established models indeed reflected the characteristics of the actual human PT. Consistent with our previous study in which 22 matched tumor and normal pairs of human PT were whole-exome sequenced^15^, the patient’s tumor, xenograft, and cell line harbored mutations in *MED12*, *TP53*, *RB1,* and *KMT2D*. The MPT-S1 model may be useful for future studies on PT biology and therapy.

Efficacious systemic therapies for metastatic malignant PTs are currently lacking and contemporary guidelines typically follow that for soft tissue sarcomas. Typically regarded as a chemoresistant disease, responses to cytotoxic chemotherapy such as anthracycline and ifosfamide-based regimens have rarely been described^19–22^. The presence of metastatic disease thus portends a grim prognosis, with most patients not surviving beyond a year^8^. As expected, our patient demonstrated primary resistance to chemotherapy and rapid progression of lung metastases. Interestingly, following extrapolated evidence from the phase 3 PALETTE study, which showed efficacy of the multikinase inhibitor pazopanib in soft tissue sarcomas^23^, our patient demonstrated a significant albeit short-lived response to pazopanib. This is in line with prior observations in metastatic PTs, including a recent report by Conduit et al. of sustained response to pazopanib for over 4 years^24,25^. While the authors postulated that this response to pazopanib might be due to functional dependency on angiogenic pathways including upregulation of *HIF* and *VEGF*, we had additionally observed that genes involved in tumor progression and metastasis *MMP13, ST6GAL2,* and *ENPP1* were downregulated by pazopanib. Taken together, this suggests that predictive biomarkers for pazopanib response will need to be further investigated and individualized for use in the clinic.

Interestingly, we showed *ENPP1* was upregulated in the malignant PT described – ENPP1 is a transmembrane glycoprotein that is essential for purinergic cell signaling^26^. Recently, ENPP1 has received significant attention due to its new role in immune evasion and promotion of metastasis, through the selective degradation of extracellular 2′3′cyclic GMP–AMP (cGAMP), an immune-stimulatory metabolite activating the STING pathway. The loss of ENPP1 was shown to suppress metastasis, restore immune infiltration, and potentiate response to immune checkpoint inhibition^27^. Consequently, ENPP1 inhibitors have emerged as an attractive approach for cancer therapy^28^. In our study, we demonstrated that pazopanib downregulated ENPP1 and upregulated the STING pathway including its downstream type I IFN-signaling pathways and PD-L1, highlighting its potential utility as a novel treatment option for PT in combination with immune checkpoint blockade. This concept would need to be validated in future settings including the use of immunocompetent mouse models.

Our work demonstrates the practicability of generating PDX and cell line models combined with genomic and transcriptomic data to understand mechanisms underlying patient responses to specific therapies in rare cancers. While we have gained insight as to how pazopanib affects the transcription of genes in several oncogenic pathways in PT, our study is limited to the focus on a single patient and a small sample size of PT for comparison. Additionally, although our models mirror the observed patient response to pazopanib, results from our in vitro assays display a discordant susceptibility to chemotherapeutic agents. This phenomenon suggests that in vivo chemotherapy resistance may be due to factors extrinsic to the tumor cell such as paracrine signals from the tumor microenvironment, and remains to be investigated. Nevertheless, our successful establishment of PDX and cell line models of metastatic PT provided a workflow on which we will be improving on, to hasten the process for application in future real-time pre-clinical studies. Such models may also be useful for the study of treatment resistance mechanisms. Furthermore, we have identified three genes implicated in cancer metastasis – *MMP13, ST6GAL2, and ENPP1*, which pazopanib has demonstrated to downregulate in our study and could act as drug-response biomarkers or a druggable target with regards to the latter.

In conclusion, we established a new PDX and cell line model, and provided initial evidence for the clinical efficacy of pazopanib in malignant PT, with the potential for synergy with checkpoint immunotherapy. Larger scale efforts to develop PT models are required for the discovery of novel therapeutic targets and improvement of patient outcomes.

## Methods

### Patient data and biospecimen collection

All clinical information was retrieved from electronic medical records. Verification of demographic data including sex, age, and ethnicity of the affected patient was corroborated by National Registry Identification Card. All histological parameters were reviewed by pathologists. Written informed consent from patients for use of biospecimens and clinical data was obtained in accordance with the Declaration of Helsinki. Tissue collection and consent protocols were under ethics approval from the SingHealth Centralized Institution Review Board.

### Xenograft creation

The patient’s tumor samples were obtained from trucut biopsies of the recurrent chest wall mass and implanted onto 6-week-old female NSG mice. The tumor tissue was minced into 2–3 mm cores and inserted into a transplantation needle (3.5 × 9.5 mm). Each mouse was anesthetized before transplantation. A small incision was made, and tumor tissue was implanted subcutaneously into the flank of mice. Subcutaneous rather than orthotopic implantation was performed as the tumor sample was derived from the chest wall mass and not from the original primary breast tumor. Serial transplantation of xenografts was performed when tumor volumes reached approximately 1500–2000 mm^3^. After the third serial transplantation, the xenograft was harvested and dissociated for cell culture establishment. Xenograft studies were conducted in compliance with animal protocols approved by the SingHealth Institutional Animal Care and Use Committee (IACUC). Mouse and human CD45 staining were performed to exclude xenograft-associated lymphoma (Supplementary Fig. 5). Immunohistochemistry and genomic profiles of the xenograft and cell line were compared with the original tumor.

### In vivo drug treatment

For in vivo drug treatment with pazopanib, 6-week-old female NSG mice were inoculated with 1 × 10^6^ cells with daily oral administration of pazopanib at a dosage of 100 mg/kg or vehicle control (0.5% carboxymethylcellulose, 0.1% Tween-80), for 10 days starting after the tumors reach 200 mm^3^ in size^29^. Tumor measurements were recorded thrice weekly until the vehicle control tumor sizes reached approximately 1000 mm^3^, when the mice were euthanized following IACUC guidelines. Tumor sizes in experimental and control groups (*n* = 10 per group) were averaged at each time point (days 1, 3, 5, 8, 10) and compared statistically. Microscopic visual evaluation of tumor cells and microvasculature was also performed.

### Establishment of MPT-S1 cell culture

Single cell suspensions were prepared using the human Tumor Dissociation Kit using the gentleMACS™ Dissociator (Miltenyi Biotec, Bergisch Gladbach, Germany) following the manufacturer’s protocol. Any contaminating mouse cells were removed using the Mouse Cell Depletion Kit (Miltenyi Biotec, Germany). Cell line authentication was performed by standard short tandem repeat (STR) genotyping (Axil Scientific, Singapore). The cells were maintained in Dulbecco’s Modified Eagle Medium (DMEM) supplemented with 1% penicillin-streptomycin (Thermo Fisher Scientific, Waltham, MA, US) and 10% fetal bovine serum (HyClone, WA, US) in a humidified incubator at 37 °C with 5% CO_2._ Cell cultures were at an estimated 70% confluency when used for all experimental procedures.

### Whole exome sequencing and somatic variant calling

All tumor tissues were microscopically evaluated and assessed by a pathologist for tumor content. Genomic DNA was isolated from snap frozen tumor tissue and from matched blood prior to whole exome sequencing. Briefly, hybrid selection was performed with the Human All Exon kit SureSelect Target Enrichment System (Agilent Technologies, Santa Clara, CA, USA) version 6 on the Illumina NovoSeq platform as paired-end 150-base pair reads. Alignment of read pairs were performed using Burrows-Wheeler Aligner (BWA MEM, http://bio-bwa.sourceforge.net/) to the human reference genome NCBI GRC Build 37 (hg19)^30^. Using Picard (http://broadinstitute.github.io/picard/), we marked the optical duplicates prior to base score recalibration with GATK version 4.1.4 for post-alignment data processing^31^. To identify somatic variants from the normal and tumor BAM files, we used Mutect2 followed by annotating and prioritizing using VEP^32^.

### RNA isolation, whole transcriptome sequencing and gene set enrichment analysis

Total RNA was extracted from tumor, matched normal tissue, and cell lines using the RNeasy Mini Kit according to the manufacturer’s protocol (Qiagen, Valencia, CA, USA). The integrity of RNA was determined by electrophoresis using the 2100 Bioanalyzer (Agilent Technologies, USA). Whole transcriptome sequencing of cell lines was performed on the Illumina NovoSeq platform (Novogene, Singapore) using the standard Illumina RNA-seq protocol. The reads were aligned to the human genome hg19 RefSeq reference transcriptome by STAR^33^. Transcript abundance estimation was performed using RSEM^34^. Differentially expressed genes were identified using DESeq2^35^. For each gene, read counts were represented as “transcripts per million” (TPM) and were normalized for both sequencing depth and gene length. For tumor and matched normal tissue transcriptomic analysis was performed using the Illumina Ampliseq Transcriptome Human Gene Expression Panel (Illumina, San Diego, CA, US) following macrodissection of a representative section from formalin-fixed paraffin-embedded samples. Gene set enrichment analysis (GSEA) was performed using the Molecular Signatures Database (MSigDB) Hallmark gene set^36^. A gene set is significantly enriched if its Normalized Enrichment Score (NES) has a False Discovery Rate (FDR) *q*-value below 0.05.

### Immunohistochemistry

Sections (4 μm) were cut from the FFPE tissue blocks and mounted onto positively charged Bond Plus Slides (Leica Biosystems, Inc., Richmond, IL, USA) glass slides, and dried on a heating bench for at least 20 min. After deparaffinization and rehydration, tissue samples were subjected to antigen retrieval before incubation with the respective primary antibodies. Evaluation of microvessel density following CD31 staining was performed as previously described^37^. Antibodies used and IHC conditions are summarized in Supplementary Table 12. Slides were counter-stained with Mayer’s Haematoxylin (Dako, Glostrup, Denmark). Appropriate controls were run with each batch of slides. Negative controls consisted of the omission of primary antibody without any other changes to subsequent procedures.

### Quantification of cell viability

Cell viability was quantified using Promega CellTiter-Glo® 2.0 Cell Viability Assay (Promega, Madison, WI, US), following the manufacturer’s protocol as previously described^38^. Briefly, cells in the exponential growth phase were seeded at a concentration of 5 × 10^3^ cells in 90 μl media per well into 96-well plates and allowed to attach overnight. Each well was supplemented with 10 μl fresh medium along with increasing concentrations of drugs in parallel. After 96 h, Promega CellTiter-Glo® 2.0 Cell Viability Assay reagent was added to the wells and incubated at room temperature for 10 min before measuring for absorbance at 480 nm using Tecan M200 Infinite 96-well plate reader with IControl Software 1.6 (Tecan, Männedorf, Switzerland). Cell viability was assessed as the percentage of control absorbance. The growth inhibitory effects were analyzed by generating dose response curves as a plot of the percentage surviving cells versus drug concentration and their IC50s were estimated using GraphPad Prism version 8.0.2 (GraphPad Software). Doxorubicin, paclitaxel, gemcitabine, ifosfamide, etoposide, pazopanib, sunitinib, axitinib, sorafenib, and ENPP1-IN-1 were purchased from Selleck Chemicals LLC (Houston, TX, US) and were prepared as per the manufacturer’s recommendations. All reactions were performed in triplicate.

### Colony formation assay

Cells were seeded at 2000 cells per well in 6-well tissue culture dishes (Nunc, Roskilde, Denmark), treated on the following day and cultured for 2 weeks. Ice-cold methanol was used as a fixative for 15 min followed by crystal violet staining for 30 min. Scanned images were analyzed using the ImageJ plugin, *ColonyArea*^39^.

### Scratch assay

To assess cell migration, cells were seeded in 6-well tissue culture dishes (Nunc, Denmark) and cultured to visible confluency prior to a fresh medium change with the appropriate concentration of pazopanib. Three parallel scratches were then introduced using a pipette tip down each well. Photographs of the scratches at marked positions were taken at 0 h, 24 h, and 48 h for triplicate wells and analyzed using the ImageJ plugin, *Wound Healing Size Tool*^40^.

### Confocal microscopy and quantification of micronuclei

Cells were fixed using 4% paraformaldehyde in PBS for 15 min, permeabilized with 0.1% Triton X-100 in PBS for 15 min and stained with NucBlue® Live ReadyProbes® Reagent (Hoechst 33342) (Thermo Fisher Scientific, USA) for 20 min and imaged with Leica TCS SP8 STED 3X (Leica Biosystems, Richmond, IL, USA). Confocal micrographs at 40X magnification of Hoechst 33342-stained cells were assessed for the occurrence of micronuclei by manual counting using ImageJ^41^. The criteria used for identifying micronuclei are: (1) round in shape; (2) less than a third of the size of a nucleus; (3) boundary of micronuclei is distinguishable from nuclear boundary; and (4) micronuclei are adjacent to a nucleus. Scores for micronuclei are represented as the number of micronuclei over the number of nuclei counted. Criteria and ratio scoring were adapted from previous publications^27,42^.

### Fluorescence-activated cell sorting for cell cycle analysis

Fluorescence-activated cell sorting (FACS) analyses of sub-G_1_ fractions using propidium iodide staining for DNA fragmentation. Briefly, MPT-S1 cells were harvested in 15 mL tubes, fixed with 70% ethanol, washed twice with PBS and stained with propidium iodide (PI) (50 μg/ml) and RNase A (100 μg/ml) for 30 min at 37 °C. At least 10,000 events were analyzed by flow cytometry (Coulter EPICS Elite ESP; Beckman Coulter, Fullerton, CA, USA) with the excitation set at 488 nm and emission at 610 nm. Data were analyzed using Flowing Software version 2.5.1 (Turku Bioscience, Turku, Finland).

### Flow cytometry for CD45 and PD-L1 expression

Ba/F3 murine pro-B cell line and human peripheral blood mononuclear cells (PBMC) were used as positive and negative controls for mouse CD45 expression (VioGreen-CD45 130-110-665, Miltenyi Biotec, Gladbach, Germany), respectively. For PD-L1 staining, cells were incubated with PD-L1 antibody (1:100, 30 min at room temperature) (#PA5-28115, Thermo Fisher Scientific, MA, USA), washed and resuspended in PBS and marked with PE-conjugated secondary antibody (#31864, Thermo Fisher Scientific, MA, USA) before analysis (BD LSR Fortessa, BD Biosciences, San Jose, CA, USA). Data were analyzed using FlowJo version 10.8.0 (BD Biosciences, San Jose, CA, USA). FACS gating strategies are available in Supplementary Fig. 6.

### SDS-PAGE and western blot

Whole cell or cellular fraction lysates were obtained using Cell Fractionation Kit (#9038, Cell Signaling Technology, Danvers, MA, USA) following the manufacturers’ protocols. Cell lysates were subjected to separation using SDS-PAGE with 4-15% Mini-PROTEAN™ TGX Stain-Free™ Protein Gels (Bio-Rad Laboratories, Hercules, CA, USA) and then transferred onto 0.2 μm PVDF membranes (Bio-Rad Laboratories, Hercules, CA, USA). After blocking for 1 h with either 5% non-fat dry milk (Bio-Rad Laboratories, Hercules, CA, USA) or 5% bovine serum albumin (Sigma-Aldrich, Darmstadt, Germany) in TBST solution (50 mM Tris/HCl pH 7.4, 150 mM NaCl, 0.1% Tween-20), the membranes were exposed to primary antibodies (Supplementary Table 12) overnight at 4 °C with gentle shaking. Exposure to the appropriate HRP-conjugated anti-rabbit or anti-mouse antibodies (Cytiva, Washington, DC, USA) were performed for 1 h and eventually subjected to chemiluminescence detection using the SuperSignal Substrate Western Blotting Kit (Thermo Fisher Scientific, MA, USA) and imaged with ChemiDoc™ XRS + System with Image Lab™ Software (Bio-Rad Laboratories, Hercules, CA, USA). Bands were quantified as indicated with ImageJ^41^. Unedited Western blots are available in Supplementary Fig. 7.

### Validation of genetic pathways using qPCR

Total RNA isolated as described above were subjected to cDNA synthesis using the iScript cDNA Synthesis kit, (Bio-Rad Laboratories, USA), according to the manufacturer’s protocol. Primer sequences are shown in Supplementary Table 13. qPCR was performed using Maxima SYBR Green/ROX qPCR Master Mix (Thermo Scientific, USA). ΔCt for mRNA expression of genes was calculated in relation to Ct value of GAPDH, which was used as an internal control. ΔΔCt was calculated by subtracting treated group ΔCt values from that of untreated control group. The normalized expression ratio was calculated with the formula 2^−ΔΔCt^.

### Statistical analysis

Results are presented as mean ± standard deviation (SD) of data collected from replicate experiments. Statistical analysis of mean values was performed through *t*-test by ANOVA using MedCalc for Windows, version 19.0.7 (MedCalc Software, Ostend, Belgium). A *p* < 0.05 was considered to be significant.

### Reporting summary

Further information on research design is available in the Nature Research Reporting Summary linked to this article.

## Supplementary information

Supplementary Appendix

Reporting summary

## Data Availability

Whole exome sequencing data were deposited in the European Nucleotide Archive (ENA) under accession no. PRJEB48011. Whole transcriptomic data were deposited in the ENA under accession no. PRJEB48016. The phyllodes xenograft and cell lines that support the findings of this study are available from the corresponding author upon reasonable request.
