# Supplementary material for: Therapeutic and immunomodulatory potential of pazopanib in malignant phyllodes tumor
Source: NPJ Breast Cancer. 2022 Apr 1;8:44. doi: 10.1038/s41523-022-00413-1 (PMC8975864; doi:10.1038/s41523-022-00413-1)
Supplement: Supplementary file 2 — Reporting summary [file 41523_2022_413_MOESM2_ESM.pdf]

nature portfolio

Corresponding author(s): Bin Tean Teh
Jason Yongsheng Chan
Puay Hoon Tan

Last updated by author(s): Dec 5, 2021

Reporting Summary

Nature Portfolio wishes to improve the reproducibility of the work that we publish. This form provides structure for consistency and transparency in reporting. For further information on Nature Portfolio policies, see our Editorial Policies and the Editorial Policy Checklist.

Statistics

For all statistical analyses, confirm that the following items are present in the figure legend, table legend, main text, or Methods section.

n/a	Confirmed	
☐	☒	The exact sample size (*n*) for each experimental group/condition, given as a discrete number and unit of measurement
☐	☒	A statement on whether measurements were taken from distinct samples or whether the same sample was measured repeatedly
☐	☒	The statistical test(s) used AND whether they are one- or two-sided<br>*Only common tests should be described solely by name; describe more complex techniques in the Methods section.*
☐	☒	A description of all covariates tested
☒	☐	A description of any assumptions or corrections, such as tests of normality and adjustment for multiple comparisons
☐	☒	A full description of the statistical parameters including central tendency (e.g. means) or other basic estimates (e.g. regression coefficient) AND variation (e.g. standard deviation) or associated estimates of uncertainty (e.g. confidence intervals)
☐	☒	For null hypothesis testing, the test statistic (e.g. *F*, *t*, *r*) with confidence intervals, effect sizes, degrees of freedom and *P* value noted<br>*Give P values as exact values whenever suitable.*
☒	☐	For Bayesian analysis, information on the choice of priors and Markov chain Monte Carlo settings
☒	☐	For hierarchical and complex designs, identification of the appropriate level for tests and full reporting of outcomes
☒	☐	Estimates of effect sizes (e.g. Cohen's *d*, Pearson's *r*), indicating how they were calculated

Our web collection on statistics for biologists contains articles on many of the points above.

Software and code

Policy information about availability of computer code

Data collection	N/A
Data analysis	MedCalc for Windows, version 19.0.7 (MedCalc Software, Ostend, Belgium) used for statistical analyses. Bioinformatic tools cited in the Methods section.

For manuscripts utilizing custom algorithms or software that are central to the research but not yet described in published literature, software must be made available to editors and reviewers. We strongly encourage code deposition in a community repository (e.g. GitHub). See the Nature Portfolio guidelines for submitting code & software for further information.

Data

Policy information about availability of data

All manuscripts must include a data availability statement. This statement should provide the following information, where applicable:

- Accession codes, unique identifiers, or web links for publicly available datasets
- A description of any restrictions on data availability
- For clinical datasets or third party data, please ensure that the statement adheres to our policy

Whole exome sequencing data were deposited in the European Nucleotide Archive (ENA) under accession no. PRJEB48011. Whole transcriptomic data were deposited in the ENA under accession no. PRJEB48016. The phyllodes xenograft and cell lines that support the findings of this study are available from the corresponding author upon reasonable request.

Field-specific reporting

Please select the one below that is the best fit for your research. If you are not sure, read the appropriate sections before making your selection.

☒ Life sciences ☐ Behavioural & social sciences ☐ Ecological, evolutionary & environmental sciences

For a reference copy of the document with all sections, see nature.com/documents/nr-reporting-summary-flat.pdf

Life sciences study design

All studies must disclose on these points even when the disclosure is negative.

Sample size	No sample size calculation was performed in this study. Study was based on a single index case. Selected findings were validated in 4 additional cell lines and 14 additional patient samples.
Data exclusions	N/A
Replication	Orthogonal experiments performed
Randomization	N/A
Blinding	N/A

Reporting for specific materials, systems and methods

We require information from authors about some types of materials, experimental systems and methods used in many studies. Here, indicate whether each material, system or method listed is relevant to your study. If you are not sure if a list item applies to your research, read the appropriate section before selecting a response.

Materials & experimental systems

n/a	Involved in the study	
☐	☒	Antibodies
☐	☒	Eukaryotic cell lines
☒	☐	Palaeontology and archaeology
☐	☒	Animals and other organisms
☐	☒	Human research participants
☒	☐	Clinical data
☒	☐	Dual use research of concern

Methods

n/a	Involved in the study	
☒	☐	ChIP-seq
☐	☒	Flow cytometry
☒	☐	MRI-based neuroimaging

Antibodies

Antibodies used	Information supplied in Supplementary Data (Supplementary Table 1)
Validation	Antibody catalog number provided in Supplementary Data (Supplementary Table 1)

Eukaryotic cell lines

Policy information about cell lines

Cell line source(s)	In house developed cell-lines
Authentication	The main cell line under study (MPT-S1) was authenticated and the information is included in Supplementary Data.
Mycoplasma contamination	All cell lines tested negative for mycoplasma contamination
Commonly misidentified lines (See ICLAC register)	N/A

Animals and other organisms

Policy information about studies involving animals; ARRIVE guidelines recommended for reporting animal research

Laboratory animals	six-week-old female NSG mice

Wild animals	N/A
Field-collected samples	N/A
Ethics oversight	Animal protocols approved by the SingHealth Institutional Animal Care and Use Committee (IACUC).

Note that full information on the approval of the study protocol must also be provided in the manuscript.

Human research participants

Policy information about studies involving human research participants

Population characteristics	Patients diagnosed with phyllodes tumors of breast
Recruitment	Patients recruited serially and when available. No clear selection bias evident.
Ethics oversight	Ethics approval from the SingHealth Centralized Institution Review Board.

Note that full information on the approval of the study protocol must also be provided in the manuscript.

Flow Cytometry

Plots

Confirm that:

☒ The axis labels state the marker and fluorochrome used (e.g. CD4-FITC).

☒ The axis scales are clearly visible. Include numbers along axes only for bottom left plot of group (a 'group' is an analysis of identical markers).

☒ All plots are contour plots with outliers or pseudocolor plots.

☒ A numerical value for number of cells or percentage (with statistics) is provided.

Methodology

Sample preparation	Included in Methods section: Ba/F3 murine pro-B cell line and human peripheral blood mononuclear cells (PBMC) were used as positive and negative controls for mouse CD45 expression (VioGreen-CD45 130-110-665, Miltenyi Biotec, Gladbach, Germany) respectively. For PD-L1 staining, cells were incubated with PD-L1 antibody (1:100, 30 minutes at room temperature) (#PA5-28115, Thermo Fisher Scientific, MA, USA), washed and resuspended in PBS and marked with PE-conjugated secondary antibody (#31864, Thermo Fisher Scientific, MA, USA) before analysis (BD LSR Fortessa, BD Biosciences, San Jose, CA, USA). Data were analyzed using FlowJo version 10.8.0 (BD Biosciences, San Jose, CA, USA).
Instrument	BD LSR Fortessa, BD Biosciences, San Jose, CA, USA
Software	FlowJo version 10.8.0 (BD Biosciences, San Jose, CA, USA)
Cell population abundance	N/A
Gating strategy	Standard gating strategy, with control specimen included each experiment. Initial gates applied to FSC-A/SSC-A to discriminate cells from debris material. Cells were then gated in FSC-H/FSC-A to discriminate single cells. Single cells were gated in SSC-H/SSC-A to discriminate live cells. Resulted population were analyzed to find cells stained with the respective fluorophore.

☐ Tick this box to confirm that a figure exemplifying the gating strategy is provided in the Supplementary Information.
