# Supplementary material for: Therapeutic and immunomodulatory potential of pazopanib in malignant phyllodes tumor
Source: NPJ Breast Cancer. 2022 Apr 1;8:44. doi: 10.1038/s41523-022-00413-1 (PMC8975864; doi:10.1038/s41523-022-00413-1)
Supplement: Supplementary file 1 — Supplementary Appendix [file 41523_2022_413_MOESM1_ESM.pdf]

SUPPLEMENTARY APPENDIX

Supplement to: Therapeutic and immunomodulatory potential of pazopanib in malignant phyllodes tumor

CONTENTS

1.0 Supplementary Figures

Supplementary Figure 1. Characteristics of additional patient-derived malignant phyllodes tumor cell-lines

Supplementary Figure 2. Downregulation of genes involved in oncogenic and apoptosis signaling pathways by pazopanib

Supplementary Figure 3. Downregulation of ENPP1 protein expression on Western blot

Supplementary Figure 4. Effects of ENPP1 inhibitor (ENPP1-IN-1) on MPT-S1 cell-line

Supplementary Figure 5. Absence of human and mouse CD45 expression in MPT-S1 PDX

Supplementary Figure 6. FACS gating strategies for the representative FACS plots shown in figures

Supplementary Figure 7. Unedited Western blots

2.0 Supplementary Tables

Supplementary Table 1. Cell line authentication by standard short tandem repeat (STR) genotyping

Supplementary Table 2. List of somatic mutations in the case of malignant phyllodes tumor

Supplementary Table 3. Differentially expressed genes comparing tumor and matched normal components

Supplementary Table 4. Upregulated Hallmark gene sets in tumor compared to matched normal component

Supplementary Table 5. Specific genes in upregulated Hallmark gene sets in tumor compared to matched normal component

Supplementary Table 6. Downregulated Hallmark gene sets in tumor compared to matched normal component

Supplementary Table 7. Specific genes in downregulated Hallmark gene sets in tumor compared to matched normal component

Supplementary Table 8. Known targets of TKIs used in this study

Supplementary Table 9. Leading edge analysis of pathways downregulated by pazopanib

Supplementary Table 10. Differentially expressed genes comparing pazopanib 5 µM and control

Supplementary Table 11. Differentially expressed genes comparing pazopanib 10 µM and control

Supplementary Table 12. List of antibodies for IHC and Western blot used in the study

Supplementary Table 13. List of PCR primers used in the study

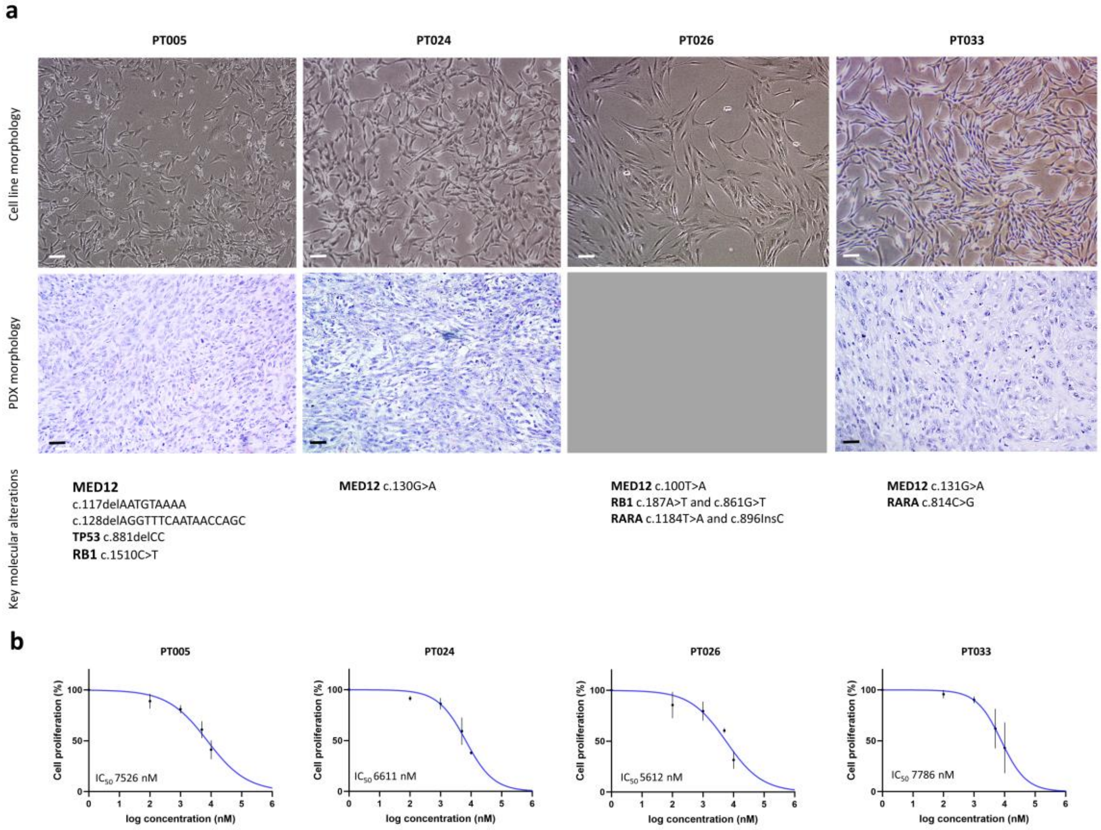

Supplementary Figure 1. Characteristics of additional patient-derived malignant phyllodes tumor cell-lines. a) Morphology of the cell-lines in active culture and in vivo cell line-derived xenografts, as well as their key molecular alterations are shown. Xenografting of PT026 was unsuccessful. Scale bar (upper panel: 20 µm; lower panel: 50 µm). b) In these additional cell-lines tested, pazopanib reduced cell viability in a dose-dependent manner.

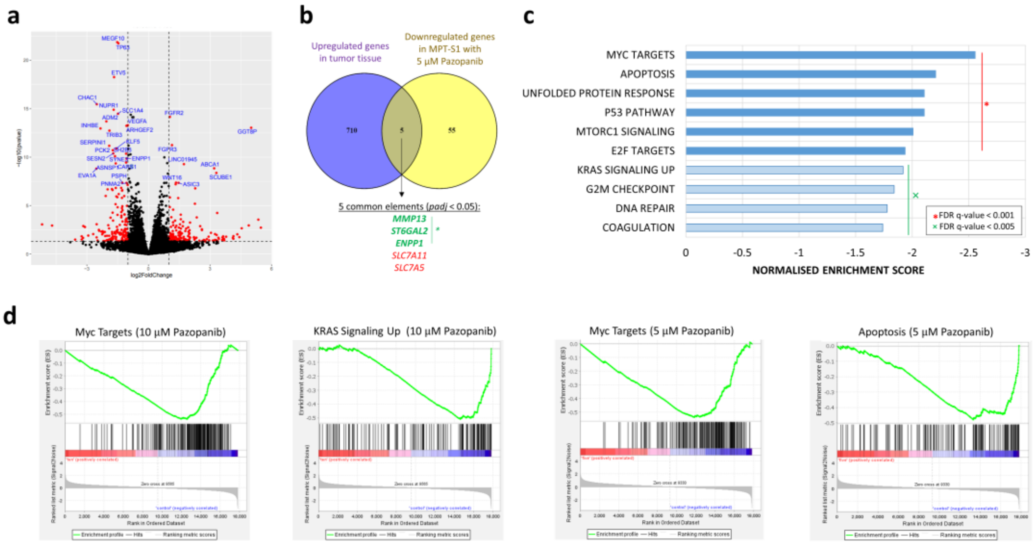

Supplementary Figure 2. Downregulation of genes involved in oncogenic and apoptosis signaling pathways by pazopanib. a) Volcano plot showing significant differentially-expressed genes after treatment of MPT-S1 cell line with pazopanib (5 µM). b) Overlap of upregulated genes in the tumor tissue and downregulated genes in the cell line by pazopanib. c) Gene set enrichment analysis highlighted top-scoring downregulated gene sets in oncogenic and apoptosis signaling pathways. d) Enrichment plots showing top 2 downregulated gene sets after pazopanib treatment.

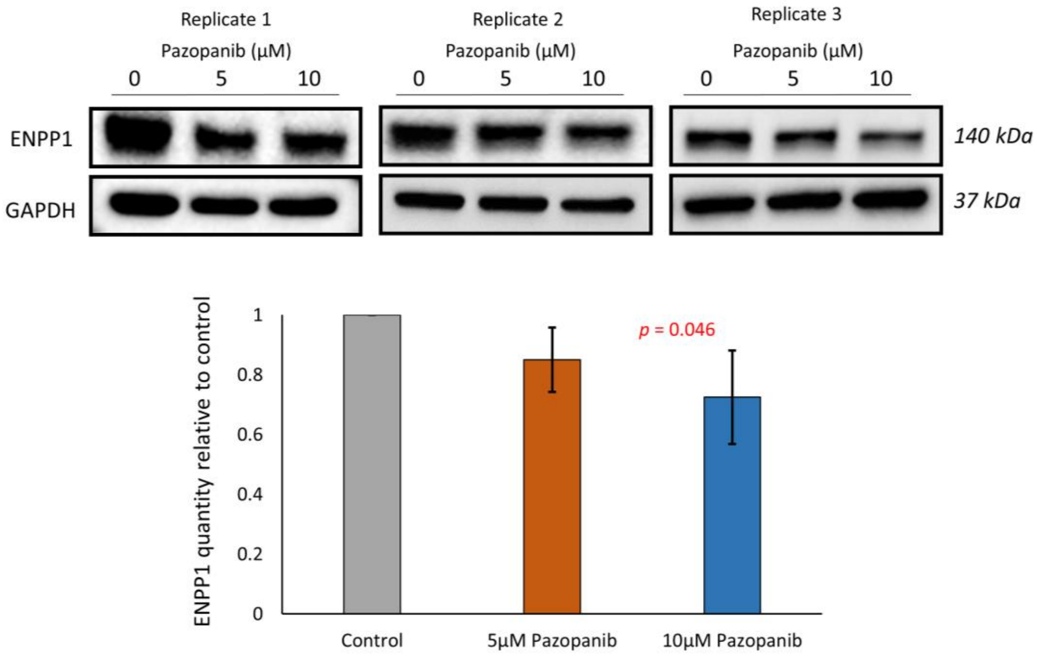

Supplementary Figure 3. Downregulation of ENPP1 protein expression on Western blot. Results from three independent experiments are shown. Band intensities were quantified with ImageJ version 1.8.0_172 and results are represented by mean intensity normalized to GAPDH, relative to untreated control. Error bars represent standard deviation and statistical analysis was performed through t-test by ANOVA.

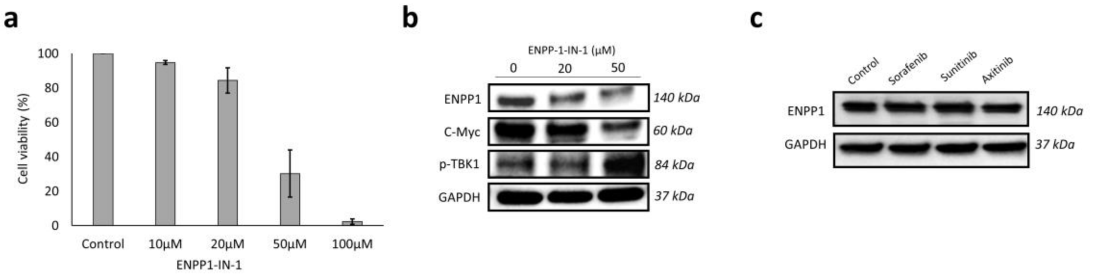

Supplementary Figure 4. Effects of ENPP1 inhibitor (ENPP1-IN-1) on MPT-S1 cell-line. a) ENPP1-IN-1 decreased cell viability in a dose-dependent manner. b) ENPP1-IN-1 decreased ENPP1 and C-Myc protein levels, and upregulated phospho-TBK1 levels. c) Treatment with other TKIs (sorafenib 1.5 µM; sunitinib 5 µM; axitinib 2.5 µM) did not reduce ENPP1 levels.

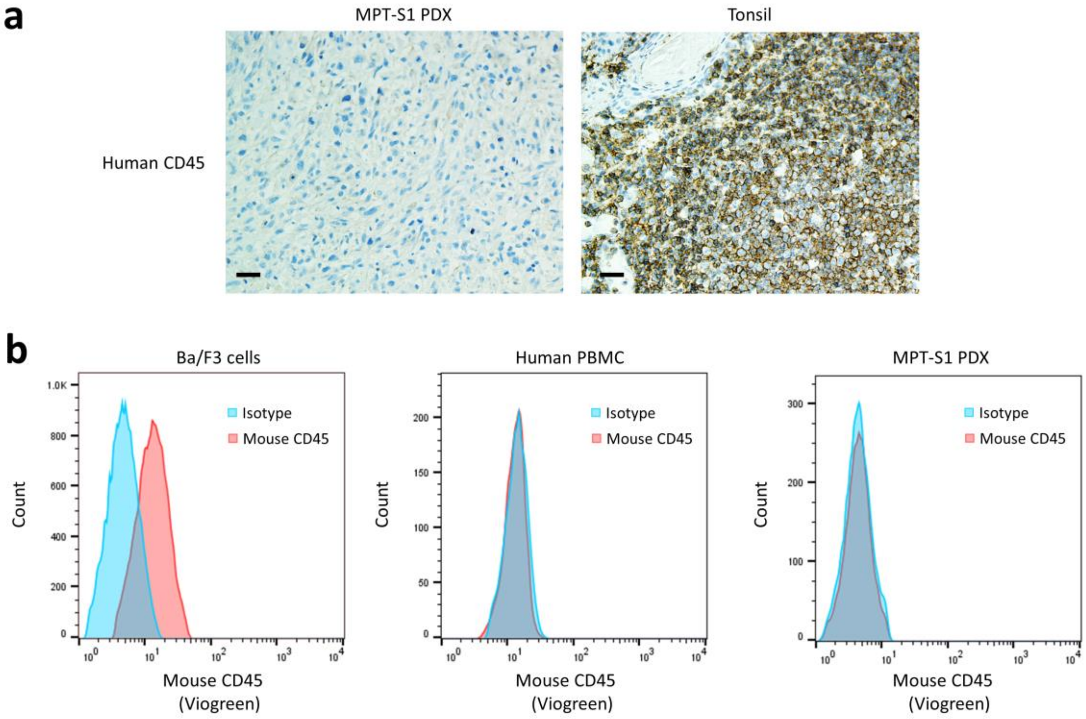

Gating strategy: Standard gating strategy, with control specimen included each experiment. Initial gates applied to FSC-A/SSC-A to discriminate cells from debris material. Cells were then gated in FSC-H/FSC-A to discriminate single cells. Single cells were gated in SSC-H/SSC-A to discriminate live cells. Resulted population were analyzed to find cells stained with the respective fluorophore.

Supplementary Figure 5. Absence of human and mouse CD45 expression in MPT-S1 PDX. a) Absence of human CD45 expression in MPT-S1 PDX by immunohistochemistry. A control (tonsil) specimen demonstrating positive CD45 staining in lymphocytes is shown (scale bar: 50 µm). b) Absence of mouse CD45 expression in MPT-S1 PDX by flow cytometry. Ba/F3 murine pro-B cell line and human peripheral blood mononuclear cells (PBMC) were used as positive and negative controls, respectively.

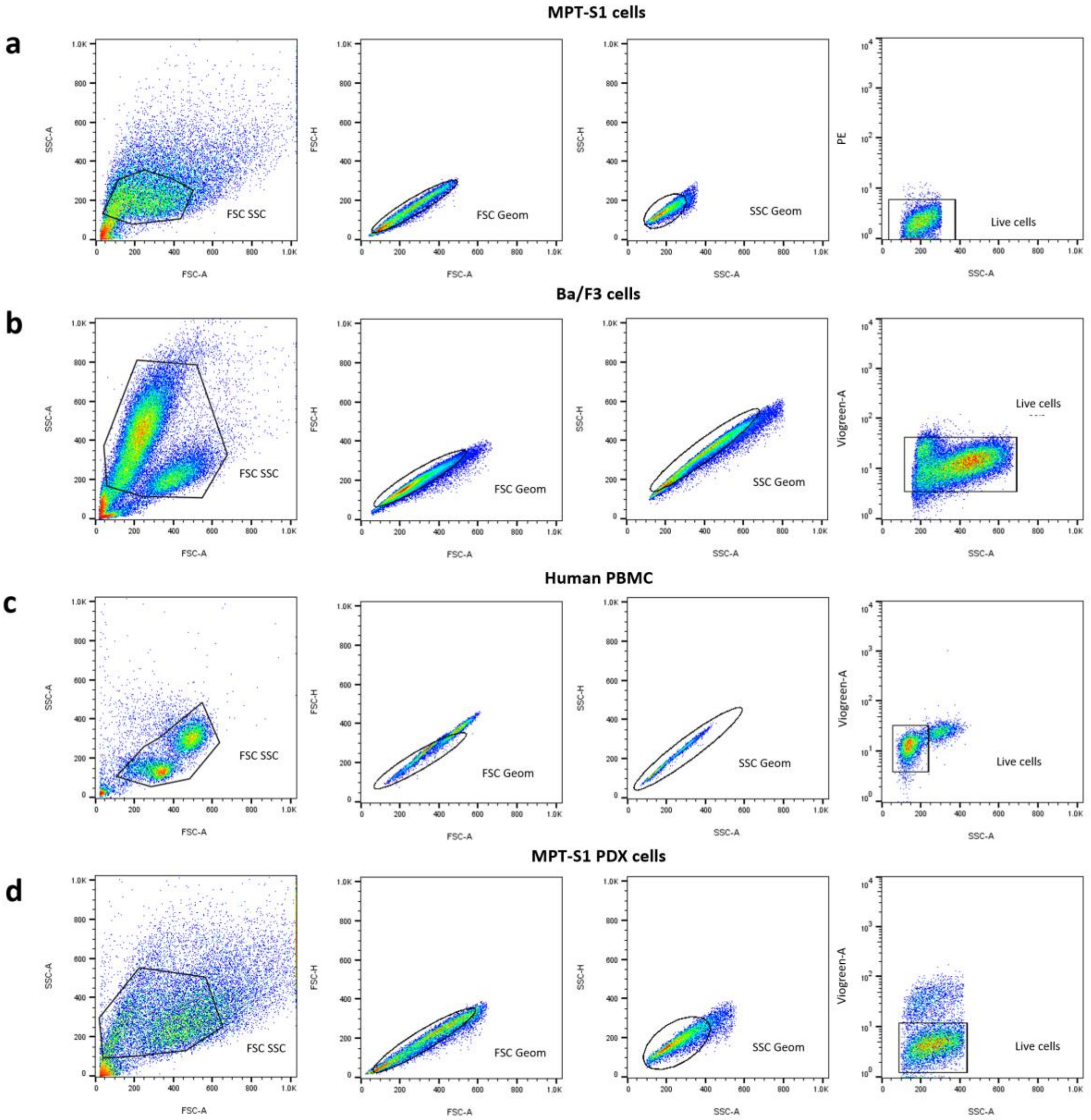

Supplementary Figure 6. FACS gating strategies for the representative FACS plots shown in figures. (a) MPT-S1 cells in figure 5e. (b) Ba/F3 cells, (c) human PBMC and (d) MPT-S1 PDX cells in supplementary figure 5b.

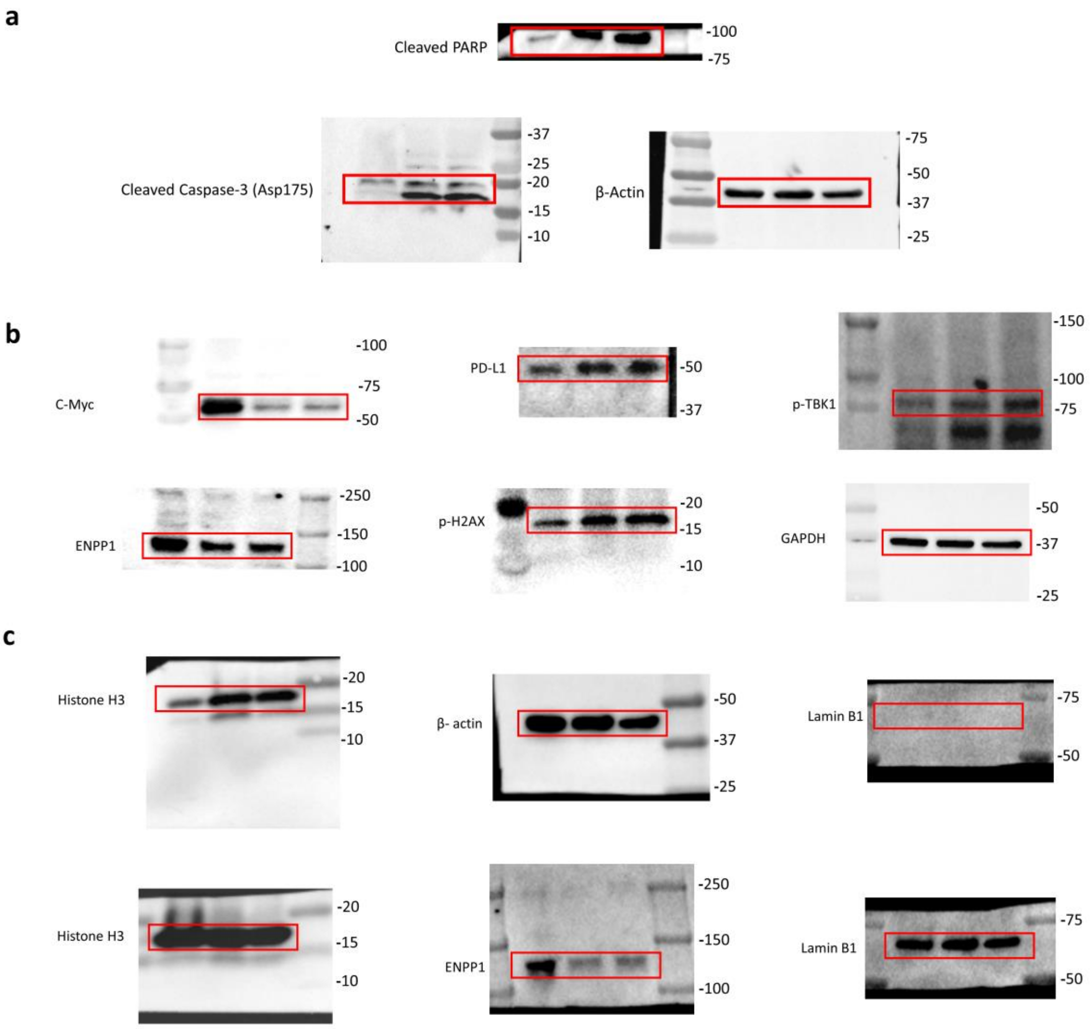

Supplementary Figure 7. Unedited Western blots. Immunoblot data shown in main figures (a) 3d, (b) 5b and (c) 5c. Boxes outline the areas cropped for use in figures. Molecular weight markers are indicated in kilodaltons.

Supplementary Table 1. Cell line authentication by standard short tandem repeat (STR) genotyping		
**LOCI**	**MPT-S1**	
Amelogenin	X	
D3S1358	15	16
D1S1656	11	16
D2S441	12	
D10S1248	13	14
D13S317	9	12
Penta E	11	19
D16S539	10	11
D18S51	18	
D2S1338	23	
CSF1PO	12	
Penta D	9	14
TH01	9	
vWA	14	16
D21S11	29	30
D7S820	12	
D5S818	11	
DYS391		9
TPOX	8	9
D8S1179	11	13
D12S391	21	
D19S433	13.2	
FGA	19	
D22S1045	14	

Supplementary Table 2. List of somatic mutations in the case of malignant phyllodes tumor

Hugo symbol	Chromosome	Start_Position	End_Position	Strand	Variant_Classification	Variant_Type	Reference_Allele	Tumor_Seq_Allele1	Tumor_Seq_Allele2	t_depth	t_ref_count	t_alt_count	vaf	n_depth	n_ref_count	n_alt_count	SIFT	PolyPhen
MED12	chrX	70339254	70339254	+	Missense_Mutation	SNP	G	G	A	187	119	68	0.363636364	141	141	0		probably_damaging(1)
KMT2D	chr12	49435899	49435902	+	Frame_Shift_Del	DEL	AATT	AATT	-	444	317	127	0.286036036	203	203	0		
RB1	chr13	48955414	48955415	+	Frame_Shift_Del	DEL	AG	AG	-	74	35	39	0.527027027	16	16	0		
RB1	chr13	48955472	48955473	+	Frame_Shift_Del	DEL	AA	AA	-	95	77	18	0.189473684	32	32	0		
TP53	chr17	7577525	7577527	+	In_Frame_Del	DEL	GAG	GAG	-	357	93	264	0.739495798	261	260	1		
UBR4	chr1	19439330	19439330	+	Missense_Mutation	SNP	T	T	C	175	134	41	0.234285714	77	77	0		probably_damaging(0.932)
ERI3	chr1	44778653	44778653	+	Intron	SNP	G	G	A	130	92	38	0.292307692	47	47	0		
CYP4X1	chr1	47497150	47497150	+	Missense_Mutation	SNP	T	T	C	265	211	54	0.203773585	95	95	0	deleterious(0)	benign(0.007)
ROR1	chr1	64643329	64643329	+	Missense_Mutation	SNP	T	T	G	591	456	135	0.228426396	232	232	0	deleterious(0.03)	possibly_damaging(0.814)
MTF2	chr1	93602314	93602316	+	In_Frame_Del	DEL	AAG	AAG	-	243	181	62	0.255144033	116	116	0		
DNTTIP2	chr1	94344616	94344616	+	Intron	SNP	A	A	C	527	387	140	0.265654649	284	284	0		
POGZ	chr1	151379754	151379754	+	Missense_Mutation	SNP	G	G	A	244	175	69	0.282786885	108	106	2	deleterious(0.01)	probably_damaging(0.987)
CRTC2	chr1	153925883	153925883	+	Intron	SNP	G	G	A	167	121	46	0.275449102	83	83	0		
OR6Y1	chr1	158517146	158517146	+	Silent	SNP	C	C	A	474	367	107	0.225738397	202	202	0		
PYHIN1	chr1	158914761	158914761	+	Missense_Mutation	SNP	A	A	G	224	161	63	0.28125	94	94	0	tolerated(0.34)	possibly_damaging(0.6)
AXDND1	chr1	179380448	179380448	+	Intron	SNP	A	A	T	217	162	55	0.253456221	70	70	0		
CACNA1E	chr1	181719531	181719531	+	Intron	SNP	T	T	G	85	60	25	0.294117647	30	30	0		
HMCN1	chr1	186157108	186157108	+	Missense_Mutation	SNP	G	G	C	476	382	94	0.197478992	184	184	0		probably_damaging(0.916)
TPR	chr1	186287736	186287736	+	Splice_Site	SNP	C	C	A	451	215	236	0.523281596	145	145	0		
IGFN1	chr1	201180370	201180370	+	Missense_Mutation	SNP	G	G	C	319	231	88	0.275862069	197	197	0		unknown(0)
PTPN7	chr1	202119482	202119482	+	Missense_Mutation	SNP	C	C	G	190	148	42	0.221052632	88	88	0	tolerated(0.37)	probably_damaging(0.991)
SERTAD4	chr1	210407337	210407339	+	Intron	TNP	TTT	TTT	AAA	74	65	9	0.121621622	47	47	0		
ROCK2	chr2	11332520	11332529	+	Intron	DEL	TGCTTAAAAT	TGCTTAAAAT	-	242	194	48	0.198347107	60	60	0		
LTBP1	chr2	33518190	33518190	+	Intron	SNP	C	C	G	228	157	71	0.311403509	104	104	0		
PNPT1	chr2	55863438	55863438	+	Silent	SNP	A	A	T	207	144	63	0.304347826	88	88	0		
ANKRD36BP2	chr2	89092053	89092053	+	Intron	SNP	T	T	A	131	100	31	0.236641221	36	36	0		
FSIP2	chr2	186657188	186657188	+	Silent	SNP	T	T	C	180	149	31	0.172222222	61	61	0		
ALS2CR12	chr2	202172178	202172178	+	Intron	SNP	T	T	A	270	190	80	0.296296296	99	99	0		
GRM7	chr3	7444844	7444844	+	Intron	SNP	T	T	C	170	112	58	0.341176471	77	77	0		
CCR8	chr3	39374237	39374237	+	Missense_Mutation	SNP	G	G	A	417	277	140	0.335731415	247	247	0	deleterious(0)	probably_damaging(0.994)
CELSR3	chr3	48688774	48688774	+	Intron	SNP	G	G	T	361	350	11	0.030470914	219	218	1		
STAB1	chr3	52557023	52557023	+	Intron	SNP	G	G	T	236	147	89	0.377118644	122	122	0		
FILIP1L	chr3	99569453	99569454	+	Frame_Shift_Del	DEL	TC	TC	-	171	111	60	0.350877193	66	66	0		
DPPA4	chr3	109052811	109052811	+	Missense_Mutation	SNP	C	C	G	147	48	99	0.673469388	127	127	0	tolerated(0.24)	benign(0.067)
PIGG	chr4	500670	500673	+	Intron	DEL	TTAA	TTAA	-	153	111	42	0.274509804	69	69	0		
OTOP1	chr4	4199528	4199528	+	Missense_Mutation	SNP	A	A	T	322	140	182	0.565217391	118	118	0	tolerated(0.28)	possibly_damaging(0.515)
SLC2A9	chr4	9889504	9889504	+	Intron	SNP	C	C	T	138	106	32	0.231884058	61	61	0		
ANAPC4	chr4	25379107	25379122	+	Frame_Shift_Del	DEL	CCGCAGGAGATTATTT	CCGCAGGAGATTATTT	-	210	108	102	0.485714286	95	95	0		
FRYL	chr4	48625213	48625213	+	Intron	DEL	C	C	-	118	105	13	0.110169492	33	33	0		
TERT	chr5	1279531	1279531	+	Missense_Mutation	SNP	G	G	A	71	53	18	0.253521127	29	29	0	tolerated(0.07)	possibly_damaging(0.649)
ADAMTS16	chr5	5222993	5222993	+	Missense_Mutation	SNP	A	A	C	275	202	73	0.265454545	87	87	0	deleterious(0.02)	benign(0.027)
DHX29	chr5	54552381	54552381	+	Intron	DEL	A	A	-	84	52	32	0.380952381	28	28	0		
SV2C	chr5	75594746	75594746	+	Missense_Mutation	SNP	A	A	T	129	89	40	0.310077519	55	55	0	deleterious(0)	possibly_damaging(0.583)
HIST1H4B	chr6	26027311	26027311	+	Missense_Mutation	SNP	C	C	G	777	638	139	0.178893179	293	293	0	tolerated(0.1)	benign(0.02)
HIST1H2BD	chr6	26157358	26157359	+	5'Flank	DEL	AG	AG	-	250	194	56	0.224	88	88	0		
ZFP57	chr6	29643738	29643738	+	Silent	SNP	C	C	T	272	222	50	0.183823529	122	122	0		
SLC26A8	chr6	35960331	35960331	+	Missense_Mutation	SNP	C	C	A	351	282	69	0.196581197	141	141	0	deleterious(0)	probably_damaging(1)
DAAM2	chr6	39836656	39836656	+	Nonsense_Mutation	SNP	A	A	T	449	366	83	0.184855234	140	140	0		
UBR2	chr6	42650772	42650772	+	Intron	SNP	C	C	T	102	79	23	0.225490196	34	34	0		
PNISR	chr6	99848898	99848898	+	Missense_Mutation	SNP	G	G	A	155	50	105	0.677419355	104	104	0	deleterious_low_confidence(0)	possibly_damaging(0.835)
Y_RNA	chr6	109627142	109627143	+	3'Flank	INS	-	-	C	293	190	103	0.351535836	117	117	0		
TIAM2	chr6	155497960	155497960	+	Missense_Mutation	SNP	T	T	A	186	140	46	0.247311828	145	145	0	tolerated(0.13)	benign(0.045)
ADCYAP1R1	chr7	31102912	31102912	+	Missense_Mutation	SNP	C	C	T	107	54	53	0.495327103	42	42	0	tolerated(0.08)	probably_damaging(0.993)
Unknown	chr7	54269173	54269173	+	IGR	SNP	T	T	G	121	85	36	0.297520661	84	84	0		
NSUN5	chr7	72721527	72721527	+	Intron	SNP	A	A	T	99	54	45	0.454545455	77	77	0		
AKR1B15	chr7	134264243	134264243	+	Intron	SNP	T	T	C	260	188	72	0.276923077	110	110	0		
SLC13A4	chr7	135375925	135375925	+	Intron	DEL	A	A	-	134	126	8	0.059701493	71	71	0		
ZNF705G	chr8	7220485	7220486	+	5'UTR	DEL	TC	TC	-	244	221	23	0.094262295	67	67	0		
CCAR2	chr8	22471911	22471912	+	Frame_Shift_Ins	INS	-	-	CT	585	349	236	0.403418803	189	189	0		
LINC00966	chr8	65290109	65290109	+	Intron	SNP	C	C	T	523	319	204	0.390057361	178	178	0		
CNBD1	chr8	87878861	87878861	+	Intron	SNP	C	C	T	325	201	124	0.381538462	107	107	0		
KCNQ3	chr8	133198737	133198737	+	Intron	SNP	G	G	A	67	40	27	0.402985075	24	24	0		
TMEM71	chr8	133759291	133759291	+	Silent	SNP	A	A	G	396	326	70	0.176767677	119	119	0		
COL22A1	chr8	139749795	139749795	+	Missense_Mutation	SNP	G	G	C	257	224	33	0.128404669	54	54	0		unknown(0)
CYP11B2	chr8	143997787	143997787	+	Intron	SNP	T	T	A	218	185	33	0.151376147	66	66	0		
RP11-87H9.3	chr9	69113607	69113607	+	5'Flank	SNP	A	A	C	268	205	63	0.235074627	112	112	0		
OSTF1	chr9	77748157	77748158	+	Intron	INS	-	-	A	89	67	22	0.247191011	37	37	0		
SUSD3	chr9	95846792	95846792	+	Intron	SNP	G	G	C	214	171	43	0.200934579	103	103	0		
DNM1	chr9	131015467	131015467	+	Intron	SNP	G	G	C	156	118	38	0.243589744	64	64	0		
CHST1	chr11	45671342	45671342	+	Missense_Mutation	SNP	G	G	C	76	51	25	0.328947368	50	50	0	tolerated(0.3)	benign(0.001)
FAM111B	chr11	58893069	58893069	+	Missense_Mutation	SNP	C	C	A	219	147	72	0.328767123	119	119	0	tolerated(0.08)	benign(0.42)
ATG2A	chr11	64658593	64658593	+	3'Flank	SNP	C	C	A	94	61	33	0.35106383	74	74	0		
USP35	chr11	77921060	77921062	+	In_Frame_Del	DEL	AGA	AGA	-	134	38	96	0.71641791	84	84	0		
CXCR5	chr11	118765021	118765021	+	Silent	SNP	G	G	A	213	134	79	0.370892019	176	176	0		
LRMP	chr12	25260930	25260930	+	Missense_Mutation	SNP	C	C	T	281	177	104	0.370106762	161	161	0	tolerated(0.91)	benign(0.01)
KMT2D	chr12	49418361	49418361	+	Missense_Mutation	SNP	C	C	T	332	186	146	0.439759036	175	175	0		unknown(0)
KCNH3	chr12	49949455	49949457	+	In_Frame_Del	DEL	AGA	AGA	-	187	181	6	0.032085561	65	65	0		
EEA1	chr12	93210062	93210062	+	Missense_Mutation	SNP	G	G	A	234	142	92	0.393162393	71	71	0	deleterious(0.02)	benign(0.032)

TBC1D4	chr13	75886954	75886954	+	Missense_Mutation	SNP	C	C	G	519	376	143	0.275529865	202	202	0	deleterious(0)	probably_damaging(0.977)
RNASE10	chr14	20978785	20978785	+	Missense_Mutation	SNP	A	A	T	305	226	79	0.259016393	130	130	0	deleterious(0.01)	benign(0.067)
RNASE10	chr14	20979121	20979121	+	Missense_Mutation	SNP	G	G	T	355	266	89	0.250704225	160	160	0	tolerated(0.06)	possibly_damaging(0.49)
LRRC16B	chr14	24529930	24529930	+	Missense_Mutation	SNP	C	C	T	319	244	75	0.235109718	136	136	0	deleterious(0)	probably_damaging(0.968)
VRTN	chr14	74825136	74825136	+	Silent	SNP	T	T	G	136	106	30	0.220588235	91	91	0		
RTL1	chr14	101348075	101348075	+	Silent	SNP	C	C	G	454	427	27	0.059471366	219	219	0		
STRA6	chr15	74481611	74481611	+	Missense_Mutation	SNP	A	A	T	81	54	27	0.333333333	39	39	0	deleterious(0)	probably_damaging(1)
LMF1	chr16	997490	997490	+	Intron	SNP	C	C	T	284	197	87	0.306338028	151	151	0		
WDR81	chr17	1631262	1631262	+	Silent	SNP	C	C	T	204	43	161	0.789215686	111	111	0		
TNK1	chr17	7290268	7290268	+	Intron	SNP	G	G	C	144	31	113	0.784722222	66	66	0		
SLC25A35	chr17	8193836	8193836	+	Intron	SNP	G	G	C	217	191	26	0.119815668	130	130	0		
MAP2K3	chr17	21201712	21201712	+	Intron	SNP	C	C	T	787	723	64	0.081321474	128	128	0		
DSC2	chr18	28654831	28654831	+	Missense_Mutation	SNP	T	T	C	309	232	77	0.249190939	153	153	0	deleterious(0.01)	possibly_damaging(0.754)
ASXL3	chr18	31322927	31322928	+	Frame_Shift_Ins	INS	-	-	CTAG	264	188	76	0.287878788	130	130	0		
ZNF407	chr18	72493342	72493361	+	Intron	DEL	AGTCAAGTTTTAGTTTTATA	AGTCAAGTTTTAGTTTTATA	-	193	43	150	0.777202073	130	130	0		
ADAT3	chr19	1912205	1912206	+	Frame_Shift_Del	DEL	AG	AG	-	111	91	20	0.18018018	98	98	0		
AC016586.1	chr19	4042445	4042447	+	In_Frame_Del	DEL	GAG	GAG	-	206	136	70	0.339805825	139	139	0		
SLC25A23	chr19	6454450	6454450	+	Missense_Mutation	SNP	C	C	A	542	522	20	0.036900369	299	299	0	deleterious(0)	probably_damaging(0.95)
COLGALT1	chr19	17691473	17691473	+	Intron	SNP	G	G	A	83	42	41	0.493975904	61	61	0		
ZNF728	chr19	23185789	23185789	+	Intron	SNP	C	C	A	169	129	40	0.236686391	79	79	0		
NLRP13	chr19	56424027	56424027	+	Missense_Mutation	SNP	A	A	G	298	157	141	0.473154362	118	118	0	tolerated(0.18)	probably_damaging(0.998)
ZNF552	chr19	58326312	58326312	+	5'Flank	SNP	A	A	T	104	45	59	0.567307692	45	45	0		
PTPRT	chr20	40899146	40899146	+	Intron	SNP	A	A	C	72	39	33	0.458333333	54	54	0		
DOK5	chr20	53208380	53208381	+	Intron	INS	-	-	TG	211	138	73	0.345971564	114	114	0		
COL20A1	chr20	61945524	61945524	+	Missense_Mutation	SNP	G	G	C	243	166	77	0.316872428	168	168	0	tolerated(0.08)	probably_damaging(0.992)
ZGPAT	chr20	62367212	62367214	+	In_Frame_Del	DEL	GCC	GCC	-	130	77	53	0.407692308	88	88	0		
TPTE	chr21	10943003	10943003	+	Missense_Mutation	SNP	C	C	T	54	48	6	0.111111111	11	11	0	tolerated(0.08)	possibly_damaging(0.899)
TRMT2A	chr22	20100641	20100641	+	Missense_Mutation	SNP	G	G	A	257	111	146	0.568093385	135	135	0	deleterious(0)	probably_damaging(0.992)
MED15	chr22	20922793	20922796	+	Intron	DEL	TCTT	TCTT	-	253	210	43	0.169960474	115	115	0		
EIF4ENIF1	chr22	31854490	31854490	+	Missense_Mutation	SNP	T	T	C	359	120	239	0.665738162	178	178	0	deleterious(0.04)	probably_damaging(0.995)
SFI1	chr22	32000276	32000277	+	Intron	DEL	CT	CT	-	173	73	100	0.578034682	108	108	0		
BPIFC	chr22	32849234	32849234	+	Intron	SNP	A	A	T	151	112	39	0.258278146	71	71	0		
WWC3	chrX	10094229	10094229	+	Silent	SNP	A	A	G	98	71	27	0.275510204	51	51	0		
CHDC2	chrX	36122690	36122690	+	Missense_Mutation	SNP	A	A	C	200	131	69	0.345	125	125	0	deleterious(0.02)	probably_damaging(0.937)
SHROOM4	chrX	50377450	50377450	+	Silent	SNP	A	A	G	415	255	160	0.385542169	283	283	0		
NOX1	chrX	100117309	100117309	+	Intron	SNP	A	A	C	281	189	92	0.327402135	162	162	0		
TAF7L	chrX	100533063	100533063	+	Missense_Mutation	SNP	C	C	T	309	209	100	0.323624595	215	215	0	tolerated(0.21)	benign(0.007)
ZNF449	chrX	134480901	134480901	+	Intron	SNP	T	T	C	144	95	49	0.340277778	80	80	0		
GPR112	chrX	135453407	135453407	+	Intron	SNP	C	C	T	89	58	31	0.348314607	45	45	0		
MAGEC3	chrX	140984762	140984762	+	Silent	SNP	T	T	A	76	50	26	0.342105263	47	47	0		
ABCD1	chrX	152991078	152991078	+	Silent	SNP	C	C	A	96	66	30	0.3125	84	84	0		
L1CAM	chrX	153134175	153134175	+	Missense_Mutation	SNP	C	C	T	279	171	108	0.387096774	223	223	0	tolerated(0.58)	benign(0.001)

Supplementary Table 3. Differentially expressed genes comparing tumor and matched normal components			
**Gene**	**Tumor read count**	**Normal read count**	**log2FoldChange**
LHX8	4158	0	>10
MGC45800	2090	0	>10
IGF2BP3	1493	0	>10
LOC728724	1692	0	>10
MMP13	1323	0	>10
ZIC5	1235	0	>10
IGF2BP1	2723	0	>10
SIGLEC15	2299	0	>10
PAGE2	2218	0	>10
MAGEC2	1816	0	>10
CSAG1	1009	0	>10
nan	1436	0	>10
HMGA2	10887	13	9.7099
RUFY4	1449	2	9.5008
FGF5	1327	2	9.3740
ATP6V0D2	2480	4	9.2761
LINC00619	1847	4	8.8510
TM4SF19	5168	16	8.3354
MMP9	130648	650	7.6510
EPYC	5463	32	7.4155
PYDC2	1347	16	6.3955
SMOC1	1132	14	6.3373
DCSTAMP	2288	32	6.1599
SLC6A15	3524	52	6.0826
TEX15	4223	76	5.7961
CXCL5	2647	48	5.7852
TNFSF11	2300	52	5.4670
TNFRSF9	1122	26	5.4314
HDAC9	2548	64	5.3151
RASL10B	1194	32	5.2216
AGMAT	1181	32	5.2058
SPP1	137080	3735	5.1978
AIM2	5272	160	5.0422
BCAT1	9382	296	4.9862
TEX29	1594	52	4.9380
BAALC	3171	104	4.9303
SEMA7A	4449	148	4.9098
INHBA	8610	308	4.8050
H19	456667	17257	4.7259
HIST1H2BJ	1629	62	4.7156
KIF4A	2907	120	4.5984
NUF2	3143	131	4.5845
RRM2	8897	377	4.5607
FOXM1	1120	48	4.5443
RAD51AP1	1021	44	4.5363

ACP5	19124	867	4.4632
CTHRC1	29878	1371	4.4458
COCH	7722	363	4.4109
CCNB2	3571	168	4.4098
TFRC	59576	2970	4.3262
HIST1H2BO	1234	62	4.3149
FAM72D	1472	74	4.3141
ST6GAL2	21513	1126	4.2559
TPX2	1377	76	4.1794
NCAPG	3654	208	4.1348
ARSI	1130	66	4.0977
ANLN	8915	523	4.0914
DLGAP5	5152	310	4.0548
IGF2BP2	2398	146	4.0378
KIF23	1102	69	3.9974
ORC6	1382	90	3.9407
BUB1	5894	392	3.9103
LIPG	2517	173	3.8629
FAM72A	1322	92	3.8449
BIRC5	2368	170	3.8001
PLAGL1	20490	1480	3.7913
MLLT11	4366	321	3.7657
DPY19L2P1	1238	92	3.7502
UBE2C	15964	1190	3.7458
nan	3770	308	3.6136
DEPDC1	4422	366	3.5948
TNIK	3479	290	3.5845
SLC16A3	3048	260	3.5513
MELK	3185	277	3.5233
OIP5	2209	194	3.5093
KIAA0101	6247	549	3.5083
DNM3	4402	395	3.4782
WNT5B	1170	108	3.4374
VEPH1	1020	96	3.4094
C17orf51	3430	328	3.3864
ARHGAP11A	3108	298	3.3826
FOXC2	1613	155	3.3794
PLAUR	12341	1211	3.3492
PBK	2964	295	3.3288
PRR11	1483	148	3.3248
APOC2	1308	132	3.3088
KCNJ15	4404	446	3.3037
KIF15	3375	343	3.2986
TTK	1325	137	3.2737
SLC9B2	4457	462	3.2701
SLC37A2	5687	592	3.2640
CDCA2	1165	122	3.2554

BUB1B	1300	138	3.2358
CCNE2	1652	177	3.2224
CKS2	5010	542	3.2084
ASF1B	1162	126	3.2051
SLC7A11	1554	172	3.1755
CREB3L1	3984	444	3.1656
MCM10	1148	128	3.1649
NEK2	3390	378	3.1648
ITGA10	1832	208	3.1388
SPHK1	4776	559	3.0949
CENPF	4386	516	3.0875
NT5DC2	20966	2489	3.0744
EPHA3	5097	607	3.0699
RFC4	2073	252	3.0402
CCDC152	1339	164	3.0294
RAC3	3806	474	3.0053
TOP2A	7992	998	3.0014
SNORA71C	155029	19471	2.9931
CENPK	2163	272	2.9914
LINC00152	1187	150	2.9843
CD84	5212	670	2.9596
KIF18A	1623	210	2.9502
ENPP1	12153	1582	2.9415
CCNA2	2938	383	2.9394
ITGAX	1052	138	2.9304
FAM81A	1238	163	2.9251
TENM3	1497	198	2.9185
nan	2390	318	2.9099
CCDC34	9480	1264	2.9069
RUNX2	5821	781	2.8979
SPAG5	1082	148	2.8700
PTTG1	2654	369	2.8465
HIST1H3B	2900	410	2.8224
CDK1	2607	369	2.8207
UBE2S	4777	681	2.8104
LAPTM5	13297	1897	2.8093
HJURP	1396	208	2.7466
WNT16	1760	263	2.7424
EIF4EBP1	6142	919	2.7406
ASPM	4736	711	2.7357
GINS2	1929	290	2.7337
HAS2	2605	396	2.7177
TYMS	3281	500	2.7141
PLAU	8240	1266	2.7024
GPC6	1796	276	2.7020
CEP55	4020	620	2.6969
KPNA2	19825	3088	2.6826

MTFP1	1412	224	2.6562
MMP14	20614	3328	2.6309
PTPRD	1608	260	2.6287
ITGB3	3830	622	2.6224
UCHL1	2051	336	2.6098
ATP6V0B	2365	391	2.5966
ADAMTS6	6428	1065	2.5935
CENPN	1868	310	2.5912
CKAP2	2381	396	2.5880
UBE2T	6778	1141	2.5706
TNFRSF10D	1826	308	2.5677
BRIP1	1741	296	2.5562
ARNTL2	7173	1220	2.5557
CA2	14291	2436	2.5525
FAM216A	2146	370	2.5361
TUBA1C	52338	9138	2.5179
LMNB2	6841	1201	2.5100
PPAT	6320	1124	2.4913
MTHFD1L	3788	674	2.4906
TCIRG1	6198	1112	2.4786
GOLIM4	26492	4754	2.4783
MATK	1609	289	2.4770
LRP8	2578	467	2.4648
CD68	5890	1069	2.4620
RACGAP1	4057	737	2.4607
ECT2	1999	364	2.4573
MRPL47	16433	3003	2.4521
TOMM34	4792	882	2.4418
CENPE	3325	624	2.4137
ATP13A3	8807	1656	2.4109
GPR180	1643	310	2.4060
SNORA71A	53503	10096	2.4058
BOP1	1567	296	2.4043
IFI30	12946	2456	2.3981
SGOL2	2860	544	2.3943
HIST1H4B	2529	482	2.3915
HIST1H4A	3793	731	2.3754
TMEM158	7939	1561	2.3465
MAD2L1	1768	349	2.3408
MCM8	1964	388	2.3397
PRDX4	16215	3213	2.3353
HIST1H2BN	3039	604	2.3310
MGC12916	2285	455	2.3283
RAC2	1035	208	2.3150
PTK7	12672	2548	2.3142
CKB	57683	11605	2.3134
ATP6V1B2	12917	2625	2.2989

TGFBI	20028	4086	2.2933
HIST2H3D	1629	333	2.2904
COL12A1	11558	2379	2.2805
PYCR1	1814	376	2.2704
EEF1E1	13577	2823	2.2659
B4GALT6	2026	424	2.2565
DCAF13	15944	3337	2.2564
SKA3	5251	1100	2.2551
SLC39A8	15115	3184	2.2471
HIST1H2AI	7599	1601	2.2468
FUCA2	16294	3434	2.2464
IKBIP	6057	1295	2.2257
PSMD2	7461	1603	2.2186
SLC7A5	3111	674	2.2066
PHLDA1	37022	8070	2.1977
GMNN	1364	298	2.1945
HIST1H2AH	16102	3527	2.1907
DNAJB11	11430	2510	2.1871
MKI67	3833	845	2.1815
PTPN22	3263	727	2.1662
ENO1	44728	9971	2.1654
NUSAP1	1405	314	2.1617
PDCD5	40596	9080	2.1606
ZWILCH	1443	323	2.1595
SCARNA9L	7322	1645	2.1542
C19orf10	1881	427	2.1392
CCNB1	2616	594	2.1388
L3HYPDH	2398	546	2.1349
DSEL	3971	906	2.1319
SNRPC	2244	512	2.1319
FN1	13370	3059	2.1279
WDR54	3543	813	2.1236
PRIM1	2117	490	2.1112
DACT1	2233	520	2.1024
SUPT3H	1854	432	2.1015
LOC339803	1074	251	2.0972
CENPP	2711	635	2.0940
TIPIN	1817	426	2.0926
SCD	12437	2919	2.0911
LMNB1	1248	294	2.0857
ATAD5	1540	363	2.0849
TERC	7828	1848	2.0827
PODXL2	1089	258	2.0776
RGS10	18659	4427	2.0755
nan	2122	516	2.0400
LTV1	9755	2374	2.0388
PFDN2	17927	4497	1.9951

EVI2A	7003	1762	1.9908
MTBP	1649	415	1.9904
POMP	4801	1210	1.9883
PSMG1	5466	1397	1.9682
ADAM19	1925	492	1.9681
HN1	4691	1199	1.9681
CCT5	20542	5251	1.9679
C12orf23	9559	2472	1.9512
CLEC4A	2604	674	1.9499
ERO1L	9562	2488	1.9423
SAE1	3981	1050	1.9227
SPDL1	2274	601	1.9198
MZT1	1283	340	1.9159
SF3B5	9392	2494	1.9130
POLR2H	11005	2927	1.9107
STRADB	3546	946	1.9063
POLD1	1031	277	1.8961
IGFBP3	15167	4097	1.8883
YARS	6536	1773	1.8822
SHMT2	1411	386	1.8700
FAM98A	6085	1665	1.8697
CDKN3	2919	801	1.8656
ITGA5	8231	2264	1.8622
TDRD3	17710	4876	1.8608
NETO2	5974	1646	1.8597
USP13	1485	410	1.8568
TXN	6388	1772	1.8500
MRPL14	2013	560	1.8458
FBXO22	1210	339	1.8356
HIST1H2BD	24376	6842	1.8330
XPOT	7663	2154	1.8309
HELLS	3777	1062	1.8305
POP5	2078	587	1.8238
SNRPB	10626	3015	1.8174
MTHFD2	1769	506	1.8057
RAD54B	1582	453	1.8042
HIST1H4C	20025	5748	1.8007
CSTB	30928	8893	1.7982
PFN2	3133	907	1.7884
NOC2L	1323	384	1.7846
LDHB	27425	8012	1.7753
LOXL2	1554	455	1.7720
BOLA3	12659	3708	1.7715
PLOD2	5031	1475	1.7701
CYCS	9131	2682	1.7675
MSANTD3	2066	607	1.7671
POLR3D	4120	1212	1.7653

ITGA4	5012	1476	1.7637
COTL1	3400	1003	1.7612
HSPD1	20718	6113	1.7609
E2F5	2431	722	1.7515
KDELR3	6562	1949	1.7514
SNRPD1	23491	6992	1.7483
RNASEH2A	1070	319	1.7460
FAM207A	15072	4529	1.7346
VEGFC	2492	750	1.7323
TNFRSF12A	3437	1036	1.7301
RPL22	4257	1285	1.7281
LOC100505687	1666	504	1.7249
SQLE	1041	315	1.7245
TTYH3	1364	413	1.7236
CSE1L	9684	2940	1.7198
XRCC3	2321	705	1.7191
FKBP14	6060	1849	1.7126
FMNL3	7097	2177	1.7049
GAS1	27960	8586	1.7033
CTSB	15476	4765	1.6995
LMAN1	6824	2107	1.6954
GLIPR1	3105	965	1.6860
TWISTNB	4158	1293	1.6852
DPP4	2834	882	1.6840
PSMA5	3033	944	1.6839
MTAP	2270	711	1.6748
TMEM48	3034	952	1.6722
PDIA6	45182	14266	1.6632
GSR	4258	1350	1.6572
MRPS23	1976	631	1.6469
FJX1	1568	503	1.6403
PRPF4	1147	368	1.6401
GLO1	8670	2783	1.6394
PHTF2	5590	1797	1.6373
GCNT1	3890	1251	1.6367
C6orf228	2691	870	1.6291
TXNDC5	10050	3258	1.6251
COL5A1	8978	2922	1.6194
SNORA84	41421	13485	1.6190
NUP160	1418	463	1.6148
SASS6	1132	370	1.6133
CAD	2672	877	1.6073
DNAJC11	2164	711	1.6058
SLC20A1	4143	1363	1.6039
PSMA6	2098	691	1.6023
MPP6	5711	1884	1.5999
MRPL12	2682	885	1.5996

MCM4	1539	508	1.5991
CCT2	4056	1339	1.5989
TOMM40	2047	677	1.5963
SENP2	5619	1879	1.5803
TGFB1	5491	1838	1.5789
SRM	6754	2261	1.5788
GPC4	4446	1490	1.5772
LRRC34	1058	355	1.5754
SLC35G1	1156	388	1.5750
N4BP2	4421	1485	1.5739
BAX	2408	809	1.5736
CDK8	3200	1077	1.5711
TMEM161A	1173	395	1.5703
TMED9	4146	1402	1.5642
SNORA79	6771	2292	1.5628
SGK196	1071	364	1.5569
NCAPD2	2497	850	1.5547
PCNA	6198	2112	1.5532
GTF2E2	4102	1402	1.5488
TBL1XR1	19509	6673	1.5477
CCT8	35735	12291	1.5397
TMEFF1	2280	786	1.5364
DKC1	2244	774	1.5357
SLC25A13	3086	1066	1.5335
MRPL3	13635	4718	1.5311
DOT1L	1887	653	1.5309
BORA	2068	716	1.5302
TUBG1	4974	1732	1.5220
SNRPF	16010	5580	1.5206
LRRC59	15032	5241	1.5201
KARS	3554	1242	1.5168
TRMT13	1015	356	1.5115
PRPS1	8204	2897	1.5018
BNIP3	4684	1656	1.5000
MAP2K3	1895	670	1.5000
NANS	3439	1216	1.4998
SPI1	1963	695	1.4980
nan	1032	367	1.4916
CHST7	1563	556	1.4912
EDIL3	5287	1886	1.4871
FXR1	46323	16535	1.4862
CCDC88A	1919	685	1.4862
PKIA	2607	931	1.4855
ALG8	4976	1780	1.4831
ATP6V1A	3270	1171	1.4815
AMPD2	2592	930	1.4788
YBX1	143345	51608	1.4738

STAR	2600	938	1.4709
nan	2994	1081	1.4697
PFDN4	22736	8222	1.4674
SNORA30	3459	1251	1.4673
HMGB3	1978	716	1.4660
PNO1	1877	680	1.4648
DNA2	1382	502	1.4610
LCP1	2856	1039	1.4588
GART	2858	1040	1.4584
RAN	21813	7939	1.4582
CERK	3543	1293	1.4542
PHACTR2	1716	628	1.4502
TGFB2	3144	1152	1.4485
MRPS7	5205	1911	1.4456
HIST1H2BK	3929	1443	1.4451
GOLT1B	3311	1218	1.4428
AP2M1	23911	8809	1.4406
GPR137B	5970	2201	1.4396
ODC1	5077	1874	1.4379
GNB4	2551	943	1.4357
LSM7	5923	2191	1.4347
RGS1	3331	1234	1.4326
MPLKIP	4930	1830	1.4297
GGH	2705	1005	1.4284
C12orf45	1227	457	1.4249
UCHL3	7564	2824	1.4214
TUBA1B	9939	3711	1.4213
MMD	2315	865	1.4202
SLC35D1	4785	1788	1.4202
PYGL	2618	979	1.4191
HCCS	1508	564	1.4189
MPI	2334	873	1.4188
USP1	3159	1183	1.4170
NCAPG2	1623	608	1.4165
VRK1	1358	509	1.4157
MANF	11221	4214	1.4129
LAPTM4B	22817	8587	1.4099
MND1	1513	570	1.4084
TFDP1	1656	624	1.4081
CCT6A	6618	2495	1.4074
OPA1	1705	643	1.4069
PGAM1	8705	3295	1.4016
HGF	1046	396	1.4013
GPX8	6131	2322	1.4008
PAPPA	1652	627	1.3977
MTIF2	2165	824	1.3937
WDR43	8173	3115	1.3916

TDG	3899	1487	1.3907
ANPEP	7030	2682	1.3902
CCT4	24385	9307	1.3896
EMP3	11015	4213	1.3865
NOP16	3746	1433	1.3863
MRPL52	1863	715	1.3816
AHSA1	6696	2575	1.3787
MRTO4	1263	486	1.3778
PEMT	1263	486	1.3778
PLEC	1339	518	1.3701
NCLN	1752	678	1.3696
SLC7A1	1664	644	1.3695
PSMD3	5594	2167	1.3682
MSH6	4363	1691	1.3674
SLC52A2	1272	493	1.3674
CKAP4	1977	767	1.3660
MRPS10	8075	3143	1.3613
RRP1B	2962	1153	1.3612
LYAR	8093	3157	1.3581
WDR76	3360	1315	1.3534
GOLM1	10820	4249	1.3485
nan	25453	9996	1.3484
CHAF1A	1064	418	1.3479
GJA1	51199	20157	1.3448
IARS	11892	4686	1.3436
TAF1A	1309	516	1.3430
C17orf79	5651	2228	1.3428
GBE1	1922	761	1.3366
CCDC138	1399	557	1.3286
GNPTAB	6095	2429	1.3273
PSMA2	3958	1578	1.3267
SKP2	1023	408	1.3262
MRGBP	2063	823	1.3258
RHOBTB3	14246	5692	1.3235
MAPK12	1754	704	1.3170
C9orf41	1436	577	1.3154
PDE3A	1719	692	1.3127
RANBP1	1994	803	1.3122
COX5A	4386	1768	1.3108
SERPINE1	5037	2031	1.3104
MLF1IP	1667	673	1.3086
C11orf83	1166	472	1.3047
STARD3NL	6398	2591	1.3041
PPIF	2344	951	1.3015
AKR1B1	1096	445	1.3004
EIF5A2	1192	484	1.3003
nan	1162	472	1.2998

CDK4	2786	1132	1.2993
CALU	36976	15058	1.2961
C12orf49	1275	522	1.2884
ACP1	5427	2223	1.2876
MTFR1	3041	1246	1.2872
KDELR1	37921	15539	1.2871
CCT7	1146	471	1.2828
CALR	77282	31802	1.2810
FCER1G	4632	1909	1.2788
RPL26L1	4716	1950	1.2741
SKIL	23967	9913	1.2737
MNF1	3204	1329	1.2695
PSMB7	4689	1950	1.2658
LSM1	5411	2251	1.2653
ASUN	15249	6349	1.2641
U2AF1	4577	1908	1.2623
CBX3	27889	11628	1.2621
AAAS	1393	581	1.2616
PGK1	7796	3252	1.2614
NUFIP1	1814	757	1.2608
SCARNA15	4602	1921	1.2604
MRPS17	1995	834	1.2583
ARSJ	1822	762	1.2577
SACS	5757	2413	1.2545
YKT6	5086	2132	1.2543
NCBP2	5836	2448	1.2534
LOC152217	14293	5996	1.2532
SLC1A5	4714	1981	1.2507
SNORA69	1676	705	1.2493
GLT25D1	6101	2568	1.2484
HRSP12	2802	1180	1.2477
RRP1	2356	994	1.2450
PRKDC	6191	2613	1.2445
INCENP	2687	1135	1.2433
PIGF	4006	1693	1.2426
MYBL1	2110	892	1.2421
EIF4G1	23651	9999	1.2420
RPIA	1612	682	1.2410
EIF5A	54350	23027	1.2390
DCTPP1	2643	1122	1.2361
HIST1H3A	1389	590	1.2353
DEK	63135	26826	1.2348
IVNS1ABP	5794	2464	1.2336
KPNA3	5892	2508	1.2322
KDELR2	43104	18351	1.2320
CHCHD3	2028	864	1.2310
UQCRH	4322	1843	1.2296

ATP6V1C1	4191	1788	1.2289
RFC3	2042	872	1.2276
TMEM106C	5244	2241	1.2265
PRMT1	13778	5889	1.2263
TMX1	5554	2374	1.2262
LEPRE1	2654	1135	1.2255
SNHG16	5473	2342	1.2246
BAZ1B	3628	1562	1.2158
E2F6	1607	692	1.2155
UCK2	1934	833	1.2152
PRKAR2A	2481	1069	1.2147
LACC1	3252	1403	1.2128
LPCAT1	3934	1700	1.2105
VDAC1	8260	3573	1.2090
MIS18A	2260	978	1.2084
IMPDH1	2214	959	1.2071
AP1M1	4984	2159	1.2069
CHM	3031	1316	1.2036
UTP18	1588	690	1.2025
PSMA7	8104	3522	1.2022
ME2	1901	828	1.1991
MRPL42	3127	1363	1.1980
RUVBL1	1919	837	1.1971
POFUT2	2543	1110	1.1960
EOGT	3664	1600	1.1953
TMEM45A	3297	1440	1.1951
ACTR3	20616	9006	1.1948
HIST1H1A	4229	1851	1.1920
OLFML2B	5092	2231	1.1905
GORAB	1149	504	1.1889
DPAGT1	2381	1045	1.1881
HIST1H2AK	1604	706	1.1839
RUVBL2	1224	539	1.1832
SURF4	11839	5218	1.1820
NUP155	1159	512	1.1787
C12orf65	1462	646	1.1783
HK2	1131	500	1.1776
NUS1	13347	5903	1.1770
RCN2	8245	3651	1.1752
RAVER1	1474	653	1.1746
CBR1	2548	1129	1.1743
FBXO5	1696	752	1.1733
SSRP1	3796	1685	1.1717
ISLR	2044	908	1.1706
NOP2	2190	974	1.1689
CDK6	5329	2371	1.1684
PLEKHA8	1139	507	1.1677

PAICS	2175	970	1.1650
AGPAT5	2246	1002	1.1645
NOLC1	7969	3565	1.1605
TPD52L2	2183	977	1.1599
GNPNAT1	13602	6088	1.1598
SEC23A	8711	3900	1.1594
SLC25A39	1542	691	1.1580
UTP6	8184	3668	1.1578
SSR3	30075	13497	1.1559
MDFIC	6129	2752	1.1552
POLD2	5285	2376	1.1534
TRIM65	1014	456	1.1530
CENPV	1082	487	1.1517
MKI67IP	2695	1213	1.1517
RPN1	34779	15656	1.1515
UBXN7	2368	1066	1.1515
HSP90AB1	81907	36925	1.1494
NIP7	1031	465	1.1487
SNRPA1	13554	6117	1.1478
BNC2	2860	1291	1.1475
SGTB	2020	912	1.1472
GTF2F2	3707	1675	1.1461
DCUN1D5	4945	2235	1.1457
nan	13923	6293	1.1457
RNASEH1	2874	1301	1.1434
DPY19L1	6438	2915	1.1431
PPP1R14B	3803	1722	1.1431
PSMB5	4769	2160	1.1427
USP22	18873	8553	1.1418
KHSRP	2044	927	1.1408
SUV39H1	1098	498	1.1407
MAPK6	9157	4154	1.1404
SWI5	2019	916	1.1402
CSPG4	3652	1657	1.1401
HSBP1	12592	5730	1.1359
RRAGD	1193	544	1.1329
COL6A1	29775	13591	1.1314
MTHFD1	2820	1288	1.1306
USP16	3041	1389	1.1305
NCEH1	1088	497	1.1304
PLOD1	6391	2922	1.1291
MRPL15	2548	1166	1.1278
ZNF480	1058	485	1.1253
GEMIN2	1614	740	1.1250
C1orf112	1628	748	1.1220
TTL	3169	1457	1.1210
EIF4A2	33571	15443	1.1203

ARCN1	12301	5661	1.1196
BZW2	6287	2895	1.1188
NUP43	2364	1089	1.1182
MSH2	3166	1462	1.1147
TMEM251	1255	580	1.1136
ZNF639	6410	2964	1.1128
EIF2S2	9968	4612	1.1119
GATAD2A	14968	6927	1.1116
GNL3	3589	1662	1.1107
HPRT1	1850	858	1.1085
NUDCD1	2464	1144	1.1069
TPGS2	2244	1042	1.1067
RPL23A	5954	2767	1.1055
GLRX	2042	949	1.1055
ATP5G1	3211	1493	1.1048
MRPL19	3701	1723	1.1030
TIMM44	3946	1838	1.1023
CMC2	1112	518	1.1021
SRSF1	23091	10779	1.0991
NDUFB5	30246	14136	1.0974
MRPS33	2477	1158	1.0970
FAM49B	5403	2527	1.0963
TBCCD1	1598	748	1.0952
PAK1IP1	7271	3405	1.0945
SUZ12	10554	4946	1.0935
CLN6	8319	3899	1.0933
RMI1	1622	761	1.0918
FAM92A1	2488	1168	1.0909
MRPL39	6428	3018	1.0908
PTP4A1	7044	3312	1.0887
NUDT15	1033	486	1.0878
TPI1	5257	2476	1.0862
WDR18	1634	770	1.0855
TXNL4A	6486	3060	1.0838
MRPL36	2475	1168	1.0834
CCDC41	2098	991	1.0821
HSPA8	48806	23100	1.0792
MFN1	9741	4614	1.0781
EMG1	4883	2318	1.0749
PGAM5	3482	1653	1.0748
CHCHD2	24468	11619	1.0744
CARM1	3392	1616	1.0697
MAK16	7540	3594	1.0690
ATP6AP1	6233	2972	1.0685
PSMB2	24853	11859	1.0674
DHRS7B	1552	741	1.0666
RRP36	2335	1116	1.0651

DCBLD2	1546	739	1.0649
IER3IP1	22032	10535	1.0644
SLC7A6	2155	1031	1.0636
RCN3	6249	2991	1.0630
PTGES3	41713	19992	1.0611
CENPH	1089	522	1.0609
HAX1	4713	2261	1.0597
PSMD11	8389	4026	1.0592
PLS3	19775	9492	1.0589
SMS	5065	2433	1.0578
PXMP2	1371	659	1.0569
C1orf53	1012	487	1.0552
SRPR	7130	3432	1.0549
PDIA4	1288	620	1.0548
UAP1	3971	1913	1.0537
COQ3	2068	997	1.0526
COPG1	7342	3548	1.0492
AP2S1	7992	3865	1.0481
CAPZA1	14388	6959	1.0479
FDX1L	1427	691	1.0462
ARF4	18104	8775	1.0448
SFXN1	2356	1142	1.0448
MTMR2	5986	2904	1.0436
TMEM208	2599	1261	1.0434
DNAAF2	1630	791	1.0431
SNORA58	2487	1208	1.0418
PSD3	2879	1401	1.0391
DNAJA1	17399	8470	1.0386
LOC100506844	2973	1448	1.0379
UBE2M	3260	1588	1.0377
DCLRE1B	2161	1055	1.0345
PVR	3024	1478	1.0328
BRMS1	2066	1010	1.0325
SMC1A	2285	1118	1.0313
COL5A2	13225	6471	1.0312
SLC35B4	1790	876	1.0310
MDH1	6754	3306	1.0307
CADM1	1205	590	1.0302
SUGT1	2548	1248	1.0297
KDM1A	1325	649	1.0297
SCARNA12	33765	16539	1.0297
ITGB1BP1	7335	3602	1.0260
KTI12	3217	1580	1.0258
MCTS1	6748	3317	1.0246
ACOT13	1043	513	1.0237
CEP170	2470	1215	1.0236
DDX18	2576	1268	1.0226

RNF34	1681	829	1.0199
AIMP2	1411	696	1.0196
PUS7	3634	1798	1.0152
PNP	1245	616	1.0151
COL7A1	5691	2816	1.0150
NANP	1010	500	1.0144
SSX2IP	1335	662	1.0119
SCUBE3	1270	630	1.0114
GARS	1505	747	1.0106
TUSC3	8308	4131	1.0080
ABL2	3368	1675	1.0077
LONP1	1698	845	1.0068
WHSC1	1018	507	1.0057
ORMDL2	1459	727	1.0050
MED8	3707	1850	1.0027
EPHB2	1084	541	1.0027
LACTB2	1180	589	1.0024
CCDC59	1250	624	1.0023
SENP5	4616	2306	1.0013
PCCB	1297	648	1.0011

Supplementary Table 4. Upregulated Hallmark gene sets in tumor compared to matched normal component

**Gene set [Total number of genes]**	**Genes in overlap**	**p-value**	**FDR q-value**
HALLMARK_E2F_TARGETS [200]	59	1.03 e^{-54}	5.17 e^{-53}
HALLMARK_MYC_TARGETS_V1 [200]	55	3.51 e^{-49}	8.78 e^{-48}
HALLMARK_G2M_CHECKPOINT [200]	50	1.59 e^{-42}	2.65 e^{-41}
HALLMARK_MTORC1_SIGNALING [200]	41	2.52 e^{-31}	3.15 e^{-30}
HALLMARK_EPITHELIAL_MESENCHYMAL_TRANSITION [200]	34	2.21 e^{-23}	2.21 e^{-22}
HALLMARK_GLYCOLYSIS [200]	28	3.46 e^{-17}	2.88 e^{-16}
HALLMARK_MYC_TARGETS_V2 [58]	16	3.07 e^{-15}	2.19 e^{-14}
HALLMARK_MITOTIC_SPINDLE [199]	24	1.82 e^{-13}	1.14 e^{-12}
HALLMARK_UNFOLDED_PROTEIN_RESPONSE [113]	17	1.73 e^{-11}	9.59 e^{-11}
HALLMARK_IL2_STAT5_SIGNALING [199]	21	7.61 e^{-11}	3.81 e^{-10}

Supplementary Table 5. Specific genes in upregulated Hallmark gene sets in tumor compared to matched normal component										
Gene symbol/Geneset	E2F TARGETS	MYC TARGETS V1	G2M CHECKPOINT	MTORC1 SIGNALING	EPITHELIAL-MESENCHYMAL TRANSITION	GLYCOLYSIS	MYC Targets V2	MITOTIC SPINDLE	UNFOLDED PROTEIN RESPONSE	Il2 STAT5 SIGNALING
NOLC1	X	X	X				X		X	
CDK4	X	X	X				X			
KPNA2	X	X	X							
MAD2L1	X	X	X							
SRSF1	X	X	X							
MCM4	X	X		X			X			
PCNA	X	X								
PRDX4	X	X								
POLD2	X	X								
RAN	X	X								
RANBP1	X	X								
USP1	X	X								
DEK	X	X								
CDK1	X		X			X		X		
CCNB2	X		X					X		
TOP2A	X		X					X		
CENPE	X		X					X		
KIF4A	X		X					X		
RACGAP1	X		X					X		
BIRC5	X		X					X		
LMNB1	X		X					X		
SMC1A	X		X					X		
CDKN3	X		X							
CKS2	X		X							
ORC6	X		X							
UBE2S	X		X							
HMGB3	X		X							
MKI67	X		X							
JPT1	X		X							
SUV39H1	X		X							
PTTG1	X		X							
MTHFD2	X			X					X	
TFRC	X			X						
TUBG1	X			X						
RRM2	X			X						
DEPDC1	X					X				
PRPS1	X					X				
DCTPP1	X						X			
DLGAP5	X							X		
POLD1	X									
RFC3	X									
SSRP1	X									
RNASEH2A	X									
MSH2	X									
PAICS	X									
SPAG5	X									
RAD51AP1	X									
CSE1L	X									
UBE2T	X									
HELLS	X									
TIPIN	X									
LYAR	X									
ASF1B	X									
PRKDC	X									
DCLRE1B	X									
SNRPB	X									
BUB1B	X									
MELK	X									
NCAPD2	X									
ODC1		X	X							X
SNRPD1		X	X							
TFDP1		X	X							
CCNA2		X	X							
PGK1		X		X		X				
HSPD1		X		X			X			

HPRT1		X		X						
EIF2S2		X		X						
CBX3		X					X			
GNL3		X					X			
NOP16		X					X			
SRM		X					X			
AIMP2		X					X			
DDX18		X					X			
XPOT		X							X	
IARS1		X							X	
PTGES3		X								
KARS1		X								
ACP1		X								
RFC4		X								
VDAC1		X								
COX5A		X								
NCBP2		X								
TYMS		X								
PSMA2		X								
PSMB2		X								
CCT7		X								
CCT4		X								
CCT2		X								
RUVBL2		X								
TXNL4A		X								
CCT5		X								
GLO1		X								
HSP90AB1		X								
LSM7		X								
PSMA6		X								
PSMA7		X								
PSMD3		X								
RPL22		X								
SNRPA1		X								
U2AF1		X								
CAD		X								
BUB1			X	X				X		
SLC7A5			X	X					X	
SQLE			X	X						
TGFB1			X		X					
NEK2			X					X		
TTK			X					X		
CENPF			X					X		
TPX2			X					X		
FBXO5			X					X		
INCENP			X					X		
NUSAP1			X					X		
KIF15			X					X		
KIF23			X					X		
DKC1			X						X	
UCK2			X							X
SLC7A1			X							
GINS2			X							
CHAF1A			X							
UBE2C			X							
HSPA8			X							
PBK			X							
NSD2			X							
H2BC12			X							
PLOD2				X	X	X				
HK2				X		X	X			X
ERO1A				X		X			X	
GLRX				X		X				
ENO1				X		X				
TPI1				X		X				
CALR				X					X	
SLC1A5				X						X
PNP				X						X
BCAT1				X						

GSR				X						
SHMT2				X						
YKT6				X						
RPN1				X						
GBE1				X						
ATP5MC1				X						
PSMG1				X						
SCD				X						
MAP2K3				X						
ACTR3				X						
SLC7A11				X						
IFI30				X						
TOMM40				X						
MLLT11				X						
NUFIP1				X						
PNO1				X						
PSMB5				X						
CCT6A				X						
EEF1E1				X						
IGFBP3					X	X				
TGFBI					X	X				
COL5A1					X	X				
PLOD1					X	X				
SPP1					X					X
PLAUR					X					
INHBA					X					
SERPINE1					X					
MMP14					X					
ITGB3					X					
EMP3					X					
ITGA5					X					
PVR					X					
FN1					X					
TNFRSF12A					X					
COL5A2					X					
GJA1					X					
CADM1					X					
GAS1					X					
EDIL3					X					
GLIPR1					X					
CTHRC1					X					
COL7A1					X					
COL12A1					X					
FOXC2					X					
ANPEP					X					
LOXL2					X					
PFN2					X					
P3H1					X					
VEGFC					X					
COLGALT1					X					
CALU					X					
KDELR3						X			X	
RRAGD						X				X
GPC4						X				
MDH1						X				
TXN						X				
HAX1						X				
CLN6						X				
SLC25A13						X				
NANP						X				
ME2						X				
MPI						X				
PGAM1						X				
PYGL						X				
SLC16A3						X				
NOP2							X			X
WDR43							X			
MRTO4							X			
NIP7							X			

SASS6								X		
ECT2								X		
ANLN								X		
CCDC88A								X		
EIF4A2									X	
PDIA6									X	
EIF4EBP1									X	
EIF4G1									X	
LSM1									X	
FKBP14									X	
SRPRA									X	
KHSRP									X	
CA2										X
TNFRSF9										X
PHLDA1										X
CKAP4										X
COL6A1										X
COCH										X
ENPP1										X
PLAGL1										X
PLEC										X
PHTF2										X
SLC39A8										X
TNFSF11										X
ADAM19										X

Supplementary Table 6. Downregulated Hallmark gene sets in tumor compared to matched normal component			
**Gene set [Total number of genes]**	**Genes in overlap**	**p-value**	**FDR q-value**
HALLMARK_EPITHELIAL_MESENCHYMAL_TRANSINSITION [200]	34	$7.8\ e^{-25}$	$3.9\ e^{-23}$
HALLMARK_TNFA_SIGNALING_VIA_NFKB [200]	26	$2.48\ e^{-16}$	$6.19\ e^{-15}$
HALLMARK_UV_RESPONSE_DN [144]	22	$1.59\ e^{-15}$	$2.65\ e^{-14}$
HALLMARK_MITOTIC_SPINDLE [199]	23	$1.69\ e^{-13}$	$2.11\ e^{-12}$
HALLMARK_TGF_BETA_SIGNALING [54]	13	$2.28\ e^{-12}$	$2.28\ e^{-11}$
HALLMARK_ADIPOGENESIS [200]	21	$1.23\ e^{-11}$	$1.02\ e^{-10}$
HALLMARK_APOPTOSIS [161]	19	$1.5\ e^{-11}$	$1.07\ e^{-10}$
HALLMARK_ESTROGEN_RESPONSE_EARLY [200]	19	$6.49\ e^{-10}$	$4.06\ e^{-9}$
HALLMARK_HYPOXIA [200]	18	$4.33\ e^{-9}$	$2.17\ e^{-8}$
HALLMARK_P53_PATHWAY [200]	18	$4.33\ e^{-9}$	$2.17\ e^{-8}$

Supplementary Table 7. Specific genes in downregulated Hallmark gene sets in tumor compared to matched normal component

Gene symbol/Geneset	EPITHELIAL-MESENCHYMAL TRANSITION	TNF-α SIGNALING VIA NFKB	UV RESPONSE DOWN	MITOTIC SPINDLE	TGF-β SIGNALING	ADIPOGENESIS	APOPTOSIS	ESTROGEN RESPONSE EARLY	HYPOXIA	P53 PATHWAY
CCN1	X	X	X						X	
ID2	X	X			X					
NOTCH2	X		X	X						
DAB2	X		X							
PMP22	X		X							
FLNA	X			X						
THBS1	X				X					
COL4A1	X					X				
DCN	X						X		X	
MMP2	X						X			
TGM2	X							X	X	
CXCL12	X							X		
CCN2	X								X	
FBN1	X									
LRP1	X									
HTRA1	X									
THY1	X									
VCAN	X									
MYL9	X									
COL4A2	X									
ITGB5	X									
TAGLN	X									
TPM2	X									
FSTL1	X									
FERMT2	X									
DPYSL3	X									
ELN	X									
MXRA5	X									
MATN2	X									
NNMT	X									
ACTA2	X									
SFRP4	X									
TPM1	X									
CALD1	X									
PDLIM5		X	X	X						
SMAD3		X	X		X					
BHLHE40		X	X					X	X	
DUSP1		X	X						X	
KLF10		X			X			X		
JUNB		X			X					
BCL6		X				X				
CCND1		X					X	X		
SOD2		X					X			
SQSTM1		X					X			
MCL1		X					X			
TIPARP		X						X	X	
BTG1		X							X	X
ZFP36		X							X	
IER5		X								X
CCL2		X								
PDE4B		X								
ETS2		X								
CEBPD		X								
NFAT5		X								
EGR1		X								
NFE2L2		X								
CCNL1		X								
TRIP10		X								
TJP1			X		X					
BMPR1A			X		X					
NR3C1			X						X	
ATP2B4			X							
NRP1			X							
ANXA4			X							
AKT3			X							
IGFBP5			X							
TFPI			X							
PIAS3			X							
RBPMS			X							
RND3			X							
MAP1B			X							
TGFBR2			X							
SPTBN1				X	X					
GSN				X			X			
SPTAN1				X			X			

EPB41L2				X						
WASL				X						
ABI1				X						
ABL1				X						
NUMA1				X						
HDAC6				X						
NET1				X						
CDC42EP1				X						
CSNK1D				X						
CTTN				X						
RAB3GAP1				X						
RABGAP1				X						
SUN2				X						
PKD2				X						
CLIP1				X						
PCGF5				X						
SSH2				X						
BMPR2					X					
WWTR1					X					
SMAD1					X					
ARID4B					X					
PPM1A					X					
PDCD4						X	X			
IFNGR1						X	X			
CAVIN1						X			X	
STOM						X				X
MGST3						X				
IDH1						X				
COL15A1						X				
ACADM						X				
HIBCH						X				
LPCAT3						X				
BAZ2A						X				
ESYT1						X				
DNAJC15						X				
APLP2						X				
LAMA4						X				
PPM1B						X				
RREB1						X				
CD151						X				
G3BP2						X				
F2R							X			X
H1-0							X			
ADD1							X			
CASP4							X			
LGALS3							X			
HSPB1							X			
WEE1							X			
CREBBP							X			
LMNA							X			
SLC2A1								X	X	
XBP1								X		
FKBP5								X		
JAK2								X		
MYOF								X		
AFF1								X		
CHPT1								X		
NRIP1								X		
LRIG1								X		
ELF1								X		
MED13L								X		
MAST4								X		
PODXL								X		
DDIT4									X	X
MXI1									X	
AKAP12									X	
ZNF292									X	
RBPJ									X	
IDS									X	
HSPA4L										X
CD81										X
ISCU										X
TRAFD1										X
VAMP8										X
VWA5A										X
ERCC5										X
XPC										X
RCHY1										X
TM4SF1										X

FBXW7										X
SP1										X
ZFP36L1										X

Supplementary Table 8. Known targets of TKIs used in this study

TKI	Targets	Reference
Pazopanib	Vascular endothelial growth factor receptor (VEGFR)-1, VEGFR-2, VEGFR-3<br>Platelet-derived growth factor receptor (PDGFR)-α and -β<br>Fibroblast growth factor receptor (FGFR)-1 and -3<br>Stem cell factor receptor (KIT)<br>Interleukin-2 receptor inducible T-cell kinase (ITK)<br>Leukocyte-specific protein tyrosine kinase (LCK)<br>Transmembrane glycoprotein receptor tyrosine kinase (c-FMS)	https://www.accessdata.fda.gov/drugsatfda_docs/label/2009/022465lbl.pdf
Sunitinib	VEGFR-1, VEGFR-2 and VEGFR-3<br>PDGFR-α and PDGFR-β<br>Stem cell factor receptor (KIT)<br>Fms-like tyrosine kinase-3 (FLT-3)<br>Colony stimulating factor receptor Type 1 (CSF-1R)<br>Glial cell-line derived neurotrophic factor receptor (RET)	https://www.accessdata.fda.gov/drugsatfda_docs/label/2011/021938s13s17s18lbl.pdf
Sorafenib	VEGFR-1, VEGFR-2, VEGFR-3<br>PDGFR-ß<br>KIT, FLT-3, RET, RET/PTC<br>c-CRAF, BRAF, mutant BRAF	https://www.accessdata.fda.gov/drugsatfda_docs/label/2018/021923s020lbl.pdf
Axitinib	VEGFR-1, VEGFR-2, VEGFR-3	https://www.accessdata.fda.gov/drugsatfda_docs/label/2012/202324lbl.pdf

Supplementary Table 9. Leading edge analysis of pathways downregulated by pazopanib

Name	F3	TIMP1	VEGFA	CD44	CDKN1A	LDHA	DDIT4	ATF3	CCND2	BMP2	DDIT3	PPP1R15A	MXD1	PLSCR1	CFH
HALLMARK_FATTY_ACID_METABOLISM_signal						-0.493451									
HALLMARK_OXIDATIVE_PHOSPHORYLATION_signal						-0.493451									
HALLMARK_APICAL_JUNCTION_signal															
HALLMARK_UV_RESPONSE_DN_signal	-0.781895														
HALLMARK_INTERFERON_ALPHA_RESPONSE_signal														-0.404085	
HALLMARK_INTERFERON_GAMMA_RESPONSE_signal					-0.389635									-0.404085	-0.658074
HALLMARK_PI3K_AKT_MTOR_SIGNALING_signal					-0.389635						-1.519636				
HALLMARK_ESTROGEN_RESPONSE_LATE_signal				-0.902079											
HALLMARK_IL2_STAT5_SIGNALING_signal				-0.902079					-0.583241	-0.895726			-0.574705	-0.404085	
HALLMARK_UNFOLDED_PROTEIN_RESPONSE_signal			-1.70087				-1.080224	-0.551294							
HALLMARK_INFLAMMATORY_RESPONSE_signal	-0.781895	-0.653016											-0.574705		
HALLMARK_TNFA_SIGNALING_VIA_NFKB_signal	-0.781895		-1.70087	-0.902079				-0.551294		-0.895726		-0.529344	-0.574705		
HALLMARK_MTORC1_SIGNALING_signal					-0.389635	-0.493451	-1.080224				-1.519636	-0.529344			
HALLMARK_GLYCOLYSIS_signal			-1.70087	-0.902079		-0.493451	-1.080224								
**HALLMARK_HYPOXIA_signal**	-0.781895		-1.70087		-0.389635	-0.493451	-1.080224	-0.551294			-1.519636	-0.529344			
HALLMARK_P53_PATHWAY_signal							-1.080224	-0.551294	-0.583241	-0.895726	-1.519636	-0.529344	-0.574705		
**HALLMARK_ANGIOGENESIS_signal**		-0.653016	-1.70087						-0.583241						
HALLMARK_EPITHELIAL_MESENCHYMAL_TRANSITION_signal		-0.653016	-1.70087	-0.902079											
HALLMARK_COMPLEMENT_signal	-0.781895	-0.653016												-0.404085	-0.658074
HALLMARK_COAGULATION_signal	-0.781895	-0.653016													-0.658074
HALLMARK_APOPTOSIS_signal		-0.653016		-0.902079	-0.389635			-0.551294	-0.583241	-0.895726	-1.519636				
HALLMARK_KRAS_SIGNALING_UP_signal									-0.583241	-0.895726		-0.529344			-0.658074
HALLMARK_DNA_REPAIR_signal															
HALLMARK_G2M_CHECKPOINT_signal															
HALLMARK_E2F_TARGETS_signal					-0.389635										
HALLMARK_MYC_TARGETS_V1_signal						-0.493451									
Counts	6	6	6	6	6	6	5	5	5	5	5	5	4	4	4

PLAT	ITGA2	TXNIP	IFITM3	FAS	PIK3R3	CCND1	KLF4	CAV1	VLDLR	TPBG	GLRX	STC1	DCN	BTG1	RETSAT	NRP1	RGS16	PLAUR	HPRT1	SQSTM1
															-0.556375					
															-0.556375					
	-0.598812				-0.389778															
					-0.389778			-1.266432	-0.846004							-0.522728				
		-1.891328	-0.77442																	
		-1.891328	-0.77442	-1.009396										-1.168773						
					-0.389778															-0.593687
						-0.752018	-0.88943	-1.266432		-0.533352									-0.508685	
			-0.77442													-0.522728	-2.172625			
										-0.533352							-2.172625			
						-0.752018	-0.88943							-1.168773						-0.593687
					-0.389778				-0.846004		-0.768456	-1.691949							-0.508685	-0.593687
									-0.846004	-0.533352	-0.768456	-1.691949	-0.430805							
								-1.266432	-0.846004	-0.533352	-0.768456	-1.691949	-0.430805	-1.168773				-0.417278		
		-1.891328		-1.009396			-0.88943							-1.168773	-0.556375		-2.172625			
												-1.691949				-0.522728				
	-0.598812			-1.009396									-0.430805					-0.417278		
-0.565557																		-0.417278		
-0.565557	-0.598812																			
-0.565557		-1.891328	-0.77442	-1.009396		-0.752018		-1.266432					-0.430805		-0.556375					-0.593687
-0.565557	-0.598812						-0.88943				-0.768456					-0.522728	-2.172625	-0.417278		
																			-0.508685	
						-0.752018														
																			-0.508685	
4	4	4	4	4	4	4	4	4	4	4	4	4	4	4	4	4	4	4	4	4

SLC2A3	MTHFD2	MCM2	XBP1	SLC7A5	PGK1	CDK4	H2AZ1	CMKLR1	TNFAIP6	LY6E	NMI	ATP2B1	TNFRSF9	PSMB9	SERPING1	SERPINC1	FYN	LTA4H	TIMP3	DUSP6
							-0.721915													
												-0.744319					-1.079494			
										-0.468445	-0.538057			-0.315609						
	-1.187865							-0.589795	-0.842775	-0.468445	-0.538057			-0.315609	-2.84887					
						-0.550489														
			-0.514419	-0.784116																
-1.555256			-0.514419										-0.509062			-0.571973				
	-1.187865		-0.514419	-0.784116																
								-0.589795	-0.842775	-0.468445	-0.538057	-0.744319	-0.509062							
-1.555256									-0.842775			-0.744319	-0.509062							
-1.555256	-1.187865	-0.669975	-0.514419	-0.784116	-0.439423													-0.542005		
					-0.439423															
-1.555256					-0.439423															
																			-0.639568	
														-0.315609	-2.84887	-0.571973	-1.079494	-0.542005		-1.322236
															-2.84887	-0.571973	-1.079494	-0.542005	-0.639568	-1.322236
																			-0.639568	
								-0.589795												-1.322236
		-0.669975		-0.784116		-0.550489	-0.721915													
	-1.187865	-0.669975				-0.550489	-0.721915													
		-0.669975			-0.439423	-0.550489	-0.721915													
4	4	4	4	4	4	4	4	3	3	3	3	3	3	3	3	3	3	3	3	3

FN1	LRP1	WARS1	PSME1	GPX4	CD86	PTEN	EGR3	TIAM1	MT2A	TP53	STMN1	RPA2	H2AX	TOP2A	PDGFRB	TGFBR3	DLD	SDHC	IRS2	SDC3
			-0.525038														-0.583284	-0.428605		
				-0.251756													-0.583284	-0.428605		
					-1.006988	-0.72549														-0.633041
						-0.72549									-0.374661	-1.688161				
		-0.800382	-0.525038																	
		-0.800382	-0.525038		-1.006988				-0.797903											
						-0.72549		-0.84895												
							-1.044661	-0.84895												
					-1.006988			-0.84895												
													-0.500683							
							-1.044661												-0.734389	
		-0.800382																		
											-0.709036						-0.583284	-0.428605	-0.734389	-0.633041
									-0.797903										-0.734389	-0.633041
										-0.500379										
-0.919935	-0.736021														-0.374661	-1.688161				
-0.919935	-0.736021																			
-0.919935	-0.736021																			
				-0.251756			-1.044661							-0.257458	-0.374661	-1.688161				
				-0.251756						-0.500379		-0.380357								
									-0.797903		-0.709036	-0.380357	-0.500683	-0.257458						
										-0.500379	-0.709036	-0.380357	-0.500683	-0.257458						
3	3	3	3	3	3	3	3	3	3	3	3	3	3	3	3	3	3	3	3	3

CITED2	CHST2	CTH	ENO1	TPI1	AK4	PLOD2	CASP6	SDC4	NT5E	LAMC2	S100A4	ITGAV	ENO3	TGM2	TNFRSF1B	FUCA1	EMP1	G0S2	DUT	PCNA
													-0.391907					-0.769834		
										-0.832478										
-0.456352																				
										-0.832478										
									-0.373381			-0.722594	-0.391907	-0.517	-0.824088		-2.145173			
	-0.737444														-0.824088					
								-0.79554										-0.769834		
		-1.320322	-0.426884	-0.444943	-0.393086	-0.531896														
-0.456352	-0.737444	-1.320322	-0.426884	-0.444943	-0.393086	-0.531896	-0.49126		-0.373381											
-0.456352	-0.737444		-0.426884	-0.444943	-0.393086		-0.49126	-0.79554			-1.857479		-0.391907	-0.517						
											-1.857479					-0.695884				
											-1.857479	-0.722594								
						-0.531896		-0.79554	-0.373381	-0.832478		-0.722594		-0.517		-0.695884				
		-1.320322					-0.49126										-2.145173			
															-0.824088	-0.695884	-2.145173	-0.769834		
																			-0.267855	-0.311284
																			-0.267855	-0.311284
																			-0.267855	-0.311284
3	3	3	3	3	3	3	3	3	3	3	3	3	3	3	3	3	3	3	3	3

NME1	TRIB3	FDXR	GPI	MCM4	DDX39A	SLC1A4	HSPA9	GOT2	PSMC4	ETF1	SRSF2	SRSF1	GSPT1	SYNCRIP	TRA2B	MYC	HNRNPD	MCM5	MCM6	MCM7
			-0.502274				-0.311232	-0.344875												
	-1.641899																			
						-1.618822														
						-1.618822	-0.311232													
	-1.641899	-0.524947	-0.502274	-0.339339	-0.505686	-1.618822	-0.311232		-0.339721	-0.719987										
								-0.344875	-0.339721											
			-0.502274																	
	-1.641899	-0.524947																		
																				-0.589866
		-0.524947								-0.719987										
-0.251778																				
					-0.505686						-0.271821	-0.303031	-0.338331	-0.353012	-0.314406	-0.281692	-0.371015	-0.616323	-0.560101	
-0.251778				-0.339339	-0.505686						-0.271821	-0.303031	-0.338331	-0.353012	-0.314406	-0.281692	-0.371015	-0.616323	-0.560101	-0.589866
-0.251778				-0.339339				-0.344875	-0.339721	-0.719987	-0.271821	-0.303031	-0.338331	-0.353012	-0.314406	-0.281692	-0.371015	-0.616323	-0.560101	-0.589866
3	3	3	3	3	3	3	3	3	3	3	3	3	3	3	3	3	3	3	3	3

DCBLD2	CD40	CD82	EMP3	CXCL8	HAS2	PLPP3	CD83	PHLDA1	PTGER4	KYNU	RNF19B	C1R	RABIF	CTSV	S100A13	PLG	APOC1	SRC	GNAI2	ADAM9
																		-0.272794	-0.315097	-0.401935
					-1.757834	-0.892432														
	-1.538316											-0.423358								
							-0.927176	-0.789059												
-0.585926	-1.538316	-0.590231	-0.593757	-0.775378	-1.757834				-0.550174											
						-0.892432	-0.927176	-0.789059	-0.550174	-0.525796	-0.713791									
		-0.590231									-0.713791									
			-0.593757	-0.775378																
										-0.525796		-0.423358	-0.462447	-0.854627	-0.989797	-0.710925	-0.867796	-0.272794	-0.315097	-0.401935
													-0.462447	-0.854627	-0.989797	-0.710925	-0.867796			
-0.585926																				
2	2	2	2	2	2	2	2	2	2	2	2	2	2	2	2	2	2	2	2	2

FDX1	ANXA1	GSN	PRSS23	CAPN5	MMP11	MMP3	THBS1	EPSTI1	PSME2	DHX58	TRIM25	HERC6	RSAD2	TRIM14	SAMD9L	MX1	IFIT3	HELZ2	PARP12	OASL
-0.558094																				
								-1.880452	-0.61589	-0.355821	-0.439659	-0.462296	-0.325634	-0.704393	-0.31165	-0.369451	-0.274763	-0.281368	-0.302904	-0.643192
								-1.880452	-0.61589	-0.355821	-0.439659	-0.462296	-0.325634	-0.704393	-0.31165	-0.369451	-0.274763	-0.281368	-0.302904	-0.643192
			-0.720156																	
				-1.477511																
						-0.453511	-0.544901													
-0.558094																				
	-0.916068	-1.115434	-0.720156	-1.477511	-0.589526	-0.453511	-0.544901													
	-0.916068	-1.115434																		
					-0.589526															
2	2	2	2	2	2	2	2	2	2	2	2	2	2	2	2	2	2	2	2	2

BCAP31	LGALS3	TIMP2	CASP9	CD38	PDE4B	PSMA2	EGFR	IRS1	DLG1	GTF2F1	JUP	PDCD4	SGK1	PTPN6	PRKAR2B	MKNK2	MYD88	GINS2	RAD51C	NUDT21
							-0.275839	-0.464792	-0.395679	-0.455535	-1.332826									
								-0.464792	-0.395679						-0.870068					
				-0.816502	-0.341296	-0.349465								-0.829671			-0.567762			
							-0.275839									-1.209746	-0.567762			
												-0.975482	-0.940111	-0.829671	-0.870068			-0.542298		
													-0.940111							
																-1.209746			-0.440113	
	-1.574338	-0.344653	-0.266724																	
-0.271773	-1.574338	-0.344653	-0.266724	-0.816502								-0.975482								
											-1.332826									
-0.271773					-0.341296					-0.455535										-0.466814
																		-0.542298		
																			-0.440113	-0.466814
						-0.349465														
2	2	2	2	2	2	2	2	2	2	2	2	2	2	2	2	2	2	2	2	2

RPA3	POLD1	SSRP1	POLE4	RFC3	RFC2	LIG1	CCNE1	CDKN3	CDC25B	BIRC5	TMPO	RACGAP1	KIF22	KIF2C	HMGA1	KIF4A	LMNB1	MYBL2	RAD21	BARD1
							-0.369464													
-0.591885	-0.246505	-0.380125	-0.447378	-0.417334	-0.577591	-0.303681														
								-0.248182	-0.228834	-0.300192	-0.257319	-0.338483	-0.329287	-0.207176	-0.577745	-0.313678	-0.79741	-0.288188	-0.258288	-0.417148
-0.591885	-0.246505	-0.380125	-0.447378	-0.417334	-0.577591	-0.303681	-0.369464	-0.248182	-0.228834	-0.300192	-0.257319	-0.338483	-0.329287	-0.207176	-0.577745	-0.313678	-0.79741	-0.288188	-0.258288	-0.417148
2	2	2	2	2	2	2	2	2	2	2	2	2	2	2	2	2	2	2	2	2

CDKN2C	SMC1A	MCM3	CHEK1	STAG1	BRCA1	HMGB2	WEE1	MGLL	SLC7A1	SMAD7	LAMC1	DAB2	FBLN5	ALDH9A1	ECHS1	DLST	MDH1	HSD17B10	ECI1	DECR1
								-0.497713						-0.699583	-0.758548	-0.28102	-0.274947	-0.323999	-0.458201	-0.51899
															-0.758548	-0.28102	-0.274947	-0.323999	-0.458201	-0.51899
								-0.497713	-0.33873	-0.320802	-0.374188	-1.794177	-0.904566							
														-0.699583						
											-0.374188	-1.794177	-0.904566							
					-0.310812	-0.275204	-0.514067			-0.320802										
-0.268879	-0.289331	-0.333525	-0.208787	-0.651579					-0.33873											
-0.268879	-0.289331	-0.333525	-0.208787	-0.651579	-0.310812	-0.275204	-0.514067													
2	2	2	2	2	2	2	2	2	2	2	2	2	2	2	2	2	2	2	2	2

ACAA2	ACAA1	SDHD	HADHB	IDH3G	CPT1A	UGDH	KMT5A	EXT1	GPC3	GPC4	GALK1	PDK3	MT1E	ADM	BNIP3L	HMOX1	EGLN3	GOT1	GCLC	QSOX1
-0.377885	-0.276878	-0.409955	-0.423484	-0.3688	-0.529093	-0.47716	-0.327978													
-0.377885	-0.276878	-0.409955	-0.423484	-0.3688	-0.529093															
													-0.890975							
						-0.47716														
														-0.946778						
																	-0.558911	-0.410739	-0.74473	
								-0.45232	-0.39704	-0.800853	-0.694076	-0.698857					-0.558911	-0.410739	-0.74473	-0.743946
								-0.45232	-0.39704	-0.800853	-0.694076	-0.698857	-0.890975	-0.946778	-0.367423	-0.37316				
																				-0.743946
															-0.367423	-0.37316				
							-0.327978													
2	2	2	2	2	2	2	2	2	2	2	2	2	2	2	2	2	2	2	2	2

TGFA	SOD1	PMEPA1	PTX3	TNC	ECM1	CAPG	APLP1	THY1	SLIT2	CXCL12	MEST	LGALS1	S100A10	PERP	POSTN	PLIN2	AHR	PTGER2	ABCB1	ETV4
												-0.797782	-0.751432							
								-0.873478	-1.278021											
										-0.654678	-1.028286			-0.505453						
					-0.457948	-0.846644	-0.381091									-0.541633	-0.832198	-0.467324	-1.034404	-1.928744
																	-0.832198	-0.467324		
		-0.752819	-1.343459	-1.787629																
-0.63527	-0.372975																			
																-0.541633				
-0.63527													-0.751432	-0.505453						
															-1.144053					
		-0.752819	-1.343459	-1.787629	-0.457948	-0.846644	-0.381091	-0.873478	-1.278021	-0.654678	-1.028286	-0.797782			-1.144053					
	-0.372975																			
																			-1.034404	-1.928744
2	2	2	2	2	2	2	2	2	2	2	2	2	2	2	2	2	2	2	2	2

CD81	ANKH	TRIB1	CIDEA	NGF	IMPDH2	TYMS	GTF2H1	UBE2D3	SLC7A11	NUPR1	GAPDH	TES	GBE1	UNG	RRM2	ASNS	PSAT1	SLC1A5	IARS1	NPM1
			-0.353596																	
				-0.876009				-0.592981												
-0.589371																		-0.58391		
																-1.080012	-0.966304		-0.529337	-0.385332
		-0.583994																		
							-0.324859	-0.592981	-1.07323	-1.289796	-0.379332	-0.931775	-0.477265	-0.487885	-0.549605	-1.080012	-0.966304	-0.58391		
											-0.379332	-0.931775	-0.477265							
-0.589371									-1.07323	-1.289796										
	-0.614948																			
	-0.614948	-0.583994	-0.353596	-0.876009																
					-0.281148	-0.247344	-0.324859													
														-0.487885	-0.549605					
					-0.281148	-0.247344													-0.529337	-0.385332
2	2	2	2	2	2	2	2	2	2	2	2	2	2	2	2	2	2	2	2	2

NHP2	XPOT	SHC1	SLC25A3	COX5A	PHB2	VDAC3	VDAC1	TOMM70	LDHB	PSMC6	EPRS1	PSMD14	EIF2S2	KPNB1	SMARCC1	HNRNPU	TFDP1	BUB3	XRCC6	POLD2
			-0.209679	-0.242006	-0.396563	-0.420831	-0.253267	-0.197756	-0.443324											
		-0.396513																		
-0.351326	-0.308326	-0.396513																		
										-0.306455	-0.695446	-0.364846	-0.818688							
									-0.443324											
														-0.276405	-0.393137	-0.29725	-0.244506	-0.264013		
																			-0.44006	-0.257648
-0.351326	-0.308326		-0.209679	-0.242006	-0.396563	-0.420831	-0.253267	-0.197756		-0.306455	-0.695446	-0.364846	-0.818688	-0.276405	-0.393137	-0.29725	-0.244506	-0.264013	-0.44006	-0.257648
2	2	2	2	2	2	2	2	2	2	2	2	2	2	2	2	2	2	2	2	2

RAN	PA2G4	USP1	EIF2S1	NAP1L1	RPL18	PTAFR	PCDH7	OSMR	RGS1	ADGRE1	SLC4A4	GPR132	STAB1	SLC31A2	SLC31A1	AXL	HRH1	NMUR1	EIF1	EGR1
						-0.916769	-1.095298	-0.95178	-1.298266	-0.880778	-0.778733	-0.52768	-0.963727	-1.378002	-0.471223	-0.834792	-0.663871	-0.467203		
																			-0.65578	-0.645451
					-0.425757															
-0.465536	-0.192919	-0.256688	-0.190046	-0.189192																
-0.465536	-0.192919	-0.256688	-0.190046	-0.189192	-0.425757															
2	2	2	2	2	2	1	1	1	1	1	1	1	1	1	1	1	1	1	1	1

EGR2	FJX1	RELB	TGIF1	IER2	SERPINB8	CLCF1	BCL6	KLF9	KLF10	HPCAL4	GATA3	PLA2G7	CASP10	DUSP5	SIRT6	MMP14	F8	MMP13	L3MBTL4	PRCP
-1.284239	-0.678013	-0.711787	-0.668105	-0.928532	-0.566717	-0.682278	-0.586613	-0.782723	-0.813102											
										-0.648587	-0.408516	-0.493499	-0.784238	-0.249016	-0.561232	-0.228696	-0.245649	-1.264447	-0.228063	-0.22419
1	1	1	1	1	1	1	1	1	1	1	1	1	1	1	1	1	1	1	1	1

SH2B3	PREP	APOBEC3F	CALM3	CALM1	CTSD	CTSB	KIF2A	GNAI3	CD55	GCA	VCPIP1	GNB2	GNG2	SPOCK2	PHEX	CSRP1	ANXA5	ZEB1	LAMP2	MAFF
-1.751245	-0.302686	-0.619968	-0.45667	-0.228756	-0.254307	-0.350832	-0.250569	-0.288095	-0.32527	-0.49106	-0.256954	-0.292604	-1.091771	-0.393063	-0.928205	-0.348228	-0.552697	-0.250235	-0.286857	-0.317368
1	1	1	1	1	1	1	1	1	1	1	1	1	1	1	1	1	1	1	1	1

CR2	CR1	KCNIP3	CBLB	ZFPM2	DOCK10	HSPA5	DOCK9	USP14	SH2B2	CTSK	CRIP2	GDA	DCT	MOV10	GMPR	IFI35	TRIM21	IL15	NCOA7	CD47
														-0.317883	-0.191712	-0.205989	-0.190643	-0.17492	-0.631896	-0.276551
-0.484954	-0.553466	-0.629851	-0.35474	-0.467121	-0.266258	-0.230347	-0.228395	-0.279015												
									-0.475108	-0.519894	-0.500993	-1.01149	-0.716941							
1	1	1	1	1	1	1	1	1	1	1	1	1	1	1	1	1	1	1	1	1

PROCR	LGALS3BP	MVB12A	NUB1	OGFR	CNP	IFITM1	IFITM2	PARP14	PARP9	TMEM140	SELL	FEZ1	H1-0	TSPO	DPYD	BID	CD14	PSEN2	IGF2R	ADD1
-0.222927	-0.176229	-0.203669	-0.249016	-0.228215	-0.301345	-0.265478	-0.183572	-0.183449	-0.282676	-0.201431	-0.269845									
												-1.388533	-0.580602	-0.376145	-0.612397	-0.336816	-0.303636	-0.244771	-0.25797	-0.487262
1	1	1	1	1	1	1	1	1	1	1	1	1	1	1	1	1	1	1	1	1

BCL10	PPP3R1	CYLD	PPT1	RARA	APP	GADD45A	SLC20A1	LMNA	KRT18	HGF	PMAIP1	TGFB2	HSPB1	PPP2R5B	GBP6	RBCK1	IDO1	NFKB1	ITGB7	LYSMD2
															-0.307199	-0.291814	-0.525825	-0.282972	-0.295355	-0.292889
-0.485627	-0.273184	-0.505696	-0.313395	-0.31189	-0.30683	-0.310085	-0.247785	-0.709299	-1.475235	-0.522962	-0.967974	-0.239834	-0.767448	-0.257057						
1	1	1	1	1	1	1	1	1	1	1	1	1	1	1	1	1	1	1	1	1

AUTS2	MVP	SAMHD1	ST3GAL5	APOL6	METTL7B	XAF1	EIF4E3	OAS2	OAS3	RSU1	CADM3	ADAM23	JAM3	DSC1	DSC3	TNFRSF11B	CD276	PTPRC	ARPC2	ITGB4
										-1.173884	-1.062197	-0.306661	-0.422263	-1.032536	-0.4228	-0.264956	-0.796501	-1.12124	-0.422205	-0.375534
-0.322242	-0.349502	-0.57265	-0.478994	-0.763572	-0.272208	-1.518479	-0.380048	-0.293003	-0.70251											
1	1	1	1	1	1	1	1	1	1	1	1	1	1	1	1	1	1	1	1	1

MDK	ITGA3	PARD6G	ACTN1	WASL	RAC2	CAP1	ARHGEF6	VCL	MSN	LAYN	SHROOM2	LAMB3	MAPK11	GAMT	LDLRAP1	CDH8	CDK8	ITGA10	NF1	SGCE
-0.42355	-1.842934	-0.337879	-0.45932	-0.38734	-1.528425	-0.407151	-0.583779	-0.573189	-0.334615	-0.393475	-0.590038	-0.355061	-0.599561	-0.375137	-0.552864	-0.271053	-0.266513	-1.013146	-0.304989	-0.451897
1	1	1	1	1	1	1	1	1	1	1	1	1	1	1	1	1	1	1	1	1

ADAMTS5	NLGN2	NFASC	TIAL1	VASP	PARVA	RHOF	CLDN11	AMIGO2	LIMA1	CTNNA1	SIRPA	PKD1	RASA1	EMP2	NAB2	HR	OLFM1	PKP3	PLAC1	FAM102A
-0.67419	-0.297313	-0.315058	-0.266307	-0.666033	-0.97848	-0.327009	-1.608925	-0.423546	-0.293293	-0.288027	-0.546824	-0.294176	-0.742652							
														-0.68395	-0.830534	-1.288463	-1.972688	-0.802894	-1.213618	-1.004975
1	1	1	1	1	1	1	1	1	1	1	1	1	1	1	1	1	1	1	1	1

ANXA9	CKB	ABHD2	BAG1	ASS1	IGSF1	IL17RB	DNAJC12	ATF1	SFN	PRKAG1	DUSP3	THEM4	ACTR2	RAC1	PPP1CA	UBE2N	GSK3B	YWHAB	RALB	MKNK1
								-0.750801	-0.29956	-0.454477	-0.504507	-0.884726	-0.268391	-0.231068	-0.959273	-0.234659	-0.229108	-0.571626	-0.414119	-0.249588
-1.094148	-0.754946	-0.486634	-0.763643	-0.843048	-0.774127	-0.532075	-0.824235													
1	1	1	1	1	1	1	1	1	1	1	1	1	1	1	1	1	1	1	1	1

MAPK10	CLTC	PLCB1	CFL1	RPS6KA3	RPS6KA1	PDK1	VAV3	RIPK1	MAPK1	EWSR1	ATF5	PAFAH1B1	SRSF10	SLC38A1	FOXN3	NDC80	NUMA1	TOP1	E2F2	SLC12A2
-0.254509	-0.326513	-0.961052	-0.261373	-0.404457	-0.308087	-0.287489	-1.128122	-0.233425	-0.333216											
										-0.51088	-0.558695	-0.587018	-0.393469	-0.481755	-0.212835	-0.394989	-0.232516	-0.770234	-1.185538	-0.324534
1	1	1	1	1	1	1	1	1	1	1	1	1	1	1	1	1	1	1	1	1

H2AZ2	CCNF	FBXO5	H2BC12	SFPQ	NUP50	HIF1A	CDC6	HMGN2	TLE3	CUL3	ORC5	CUL4A	RPS6KA5	TPX2	PRMT5	HIRA	PURA	RAD54L	EFNA5	STIL
-0.536228	-0.223042	-0.215799	-0.452865	-0.575266	-0.207197	-0.261282	-0.210141	-0.303485	-0.515001	-0.409654	-0.483125	-0.395466	-0.227299	-0.215729	-0.388627	-0.243659	-0.359575	-0.257772	-0.33501	-0.216836
1	1	1	1	1	1	1	1	1	1	1	1	1	1	1	1	1	1	1	1	1

RBL1	MEIS1	SHMT1	CDCA8	EZH2	CTCF	PMS2	ATAD2	NCAPD2	MSH2	RNASEH2A	PPM1D	E2F8	PAICS	RPA1	SPC24	SPC25	MXD3	PSIP1	EED	BRMS1L
-0.217282	-0.558935																			
		-0.246009	-0.270872	-0.134004	-0.166477	-0.347675	-0.15261	-0.14794	-0.270199	-0.127564	-0.212133	-0.175861	-0.339583	-0.236956	-0.152554	-0.304274	-0.354046	-0.315596	-0.147362	-0.532384
1	1	1	1	1	1	1	1	1	1	1	1	1	1	1	1	1	1	1	1	1

SMC3	ASF1A	ASF1B	PRKDC	PLK1	ING3	TK1	TCF19	CIT	CDKN2A	MRE11	ILF3	MELK	SNRPB	DNMT1	AK2	RAD51AP1	UBR7	RFC1	RBBP7	MKI67
-0.142828	-0.226399	-0.734243	-0.301229	-0.131027	-0.568408	-0.818695	-0.14267	-0.148678	-0.358271	-0.164098	-0.166045	-0.314557	-0.474026	-0.156547	-0.125423	-0.13723	-0.521288	-0.156498	-0.41092	-0.194864
1	1	1	1	1	1	1	1	1	1	1	1	1	1	1	1	1	1	1	1	1

ANP32E	GINS1	RANBP1	GINS3	GINS4	MLH1	CBX5	DIAPH3	DCK	DEK	NIPBL	EFEMP1	ATXN1	CELF2	LPAR1	ARHGEF9	SCN8A	CACNA1A	RND3	ACVR2A	FZD2
										-0.421361	-0.543539	-0.461303	-0.648775	-0.336729	-0.563258	-0.72258	-0.460827	-0.308573	-0.307508	-0.623168
-0.216944	-0.211446	-0.1353	-0.461816	-0.381894	-0.458062	-0.158151	-0.35151	-0.239902	-0.126298											
1	1	1	1	1	1	1	1	1	1	1	1	1	1	1	1	1	1	1	1	1

NFIB	SYNE1	CDON	RUNX1	INPP4B	PTGFR	APBB2	DLC1	AMPH	LTBP1	DBP	TGFBR2	GABARAPL1	UROD	SMS	ACAT2	ALAD	HSD17B11	TP53INP2	PCBD1	PTPRG
												-0.277396	-0.634575	-0.378876	-0.374134	-0.503853	-0.407011	-0.339032	-0.956175	-0.877577
-0.540879	-0.305438	-0.342123	-0.34019	-1.226671	-0.807637	-0.416753	-0.321965	-0.411239	-0.988616	-0.521982	-0.382327									
1	1	1	1	1	1	1	1	1	1	1	1	1	1	1	1	1	1	1	1	1

ACADS	CPT2	HSDL2	CRYZ	ACOT8	ACOT2	ERP29	REEP6	ADSL	BPHL	ACSS1	SUCLG2	MAOA	CBR3	BMPR1B	ALDH1A1	LTC4S	SRPX	NAGK	ERRFI1	CAVIN3
-0.461097	-0.571829	-0.552452	-0.386562	-0.311304	-0.585389	-0.286883	-1.244554	-0.270032	-0.672899	-0.553187	-0.45251	-0.360614	-1.126565	-0.394531	-0.87013	-0.825246				
																	-1.386321	-0.471902	-1.632961	-0.491907
1	1	1	1	1	1	1	1	1	1	1	1	1	1	1	1	1	1	1	1	1

CCNG2	KIF5A	KLHL24	VHL	HOXB9	PLAC8	SELENBP1	AMPD3	NDST3	TSTA3	PGAM1	CHST1	AGRN	SLC16A3	PFKFB1	HAX1	MERTK	PC	CLN6	PAM	PYGB
								-0.378555	-0.311366	-0.401687	-0.534857	-0.719615	-0.685019	-0.761827	-0.509038	-0.408302	-0.333809	-0.328925	-0.310053	-0.373348
-0.812615	-0.886092	-0.465084	-0.362125	-0.457517	-0.470711	-0.876393	-0.448471													
1	1	1	1	1	1	1	1	1	1	1	1	1	1	1	1	1	1	1	1	1

PYGL	LHX9	PKM	HS6ST2	GYS2	CTHRC1	ANPEP	ECM2	FOXC2	VEGFC	LOXL1	PPIB	SPOCK1	COL7A1	VIM	COL12A1	TPM2	IGFBP2	SGCD	SGCB	GPX7
-0.31151	-0.318992	-1.084757	-0.854485	-0.455022																
					-0.997302	-0.877915	-0.533497	-0.421955	-0.381033	-1.263742	-0.389308	-0.429945	-0.808677	-0.895422	-0.402442	-0.437154	-0.899441	-0.577546	-0.391691	-0.61318
1	1	1	1	1	1	1	1	1	1	1	1	1	1	1	1	1	1	1	1	1

COL8A2	FBLN1	COL6A2	COL6A3	THBS2	PMM1	H1-2	DRAM1	TP63	CTSF	INHBB	MXD4	TRIAP1	SLC3A2	PLK3	TCN2	DEF6	COQ8A	PLXNB2	CEBPA	PTPN14
					-0.544371	-0.485557	-0.44174	-2.864743	-0.712251	-1.232062	-0.86518	-0.576173	-0.800353	-0.566185	-1.631011	-0.587675	-0.535954	-0.437632	-0.511954	-0.869282
-0.426657	-1.255227	-0.363813	-0.645333	-0.585862																
1	1	1	1	1	1	1	1	1	1	1	1	1	1	1	1	1	1	1	1	1

NHLH2	H2AW	ZFP36L1	STOM	VTN	FGFR1	GSTO1	ITGA6	LRRC8C	MYO1E	IKZF2	TNFSF11	SYNGR2	AHCY	ENPP1	ADAM19	PRNP	PLEC	GALM	PRKCH	ST3GAL4
						-0.721691	-0.432624	-0.589492	-0.451574	-0.420911	-0.430705	-1.046004	-0.458413	-2.368104	-1.096868	-0.919013	-0.413219	-0.356474	-1.042224	-0.395025
-0.92213	-0.809642	-0.563321	-0.573936																	
				-0.449282	-1.155157															
1	1	1	1	1	1	1	1	1	1	1	1	1	1	1	1	1	1	1	1	1

AHNAK	SWAP70	COCH	GLIPR2	CDCP1	TLR7	SPRED2	COL6A1	LRIG1	SPRY4	C3AR1	SPARCL1	ZNF277	EPB41L3	AVL9	ADGRL4	EVI5	LCP1	FBXO4	ST6GAL1	BTC
-0.626173	-0.551238	-0.498447	-0.961429	-0.359303	-0.371874	-0.923449	-0.375279	-0.535535	-0.837695											
										-0.338433	-0.726293	-0.36151	-0.795212	-0.366045	-1.035199	-0.50735	-0.356144	-0.40524	-0.342869	-0.621779
1	1	1	1	1	1	1	1	1	1	1	1	1	1	1	1	1	1	1	1	1

TRIB2	KCNN4	ITGBL1	CROT	MAP3K1	PRDM1	KIF5C	ETV1	ETV5	ATG10	PLEK2	ACE	GPNMB	LAPTM5	TMEM158	BTBD3	MAFB	PRKG2	HDAC9	GYPC	AMMECR1
-1.548207	-0.517783	-0.492632	-0.410866	-0.351707	-0.712275	-0.369254	-1.770105	-2.978507	-0.454432	-0.690943	-0.476074	-0.512403	-0.607647	-0.470707	-0.718771	-0.508245	-0.521091	-0.842521	-1.10412	-0.692141
1	1	1	1	1	1	1	1	1	1	1	1	1	1	1	1	1	1	1	1	1

GNG11	TLR8	CBR4	GUCY1A1	ALDH1A2	SPRY2	DDB1	TARBP2	SRSF6	SAC3D1	VPS37D	VPS37B	NT5C3A	EIF1B	CCNO	SUPT4H1	POLA1	CETN2	MPG	TAF9	TAF10
-0.423276	-2.510542	-0.621392	-0.758027	-0.338486	-1.319135															
						-0.364617	-0.225686	-0.401543	-0.522177	-0.495972	-0.653719	-0.348351	-0.2217	-0.285184	-0.492529	-0.522032	-0.251306	-0.426669	-0.617904	-0.307232
1	1	1	1	1	1	1	1	1	1	1	1	1	1	1	1	1	1	1	1	1

ERCC8	TAF6	ITPA	GSDME	TK2	RNMT	SF3A3	GTF2H3	EDF1	POLR1C	POLR1D	POLR2D	POLR2E	POLR2G	RAD51	POLR3C	DGUOK	ZWINT	FEN1	PRIM1	SDCBP
-0.25173	-0.307598	-0.566487	-0.454497	-0.389564	-0.351549	-0.257823	-0.564655	-0.291645	-0.316462	-0.237102	-0.25262	-0.435584	-0.228048	-0.225147	-0.225775	-0.528178	-0.22223	-0.43165	-0.683486	-0.319311
1	1	1	1	1	1	1	1	1	1	1	1	1	1	1	1	1	1	1	1	1

SMAD5	GMPR2	SHMT2	DHFR	PSMC2	CORO1A	BCAT1	PPA1	PHGDH	TCEA1	NIBAN1	ARPC5L	PRDX1	SERPINH1	SORD	RAB1A	CYP51A1	PITPNB	PSPH	ABCF2	ATF6
																				-0.405894
		-0.935328	-0.745269	-0.402785	-0.602208	-0.841548	-0.313451	-0.970974	-0.499577	-1.232035	-0.306117	-0.522983	-0.314938	-0.33057	-0.568508	-0.465335	-0.318451	-1.234828	-0.503066	
-0.228898	-0.22335																			
1	1	1	1	1	1	1	1	1	1	1	1	1	1	1	1	1	1	1	1	1

ATF4	SDAD1	EIF4EBP1	NOP14	EEF2	EIF4A3	HERPUD1	EXOSC5	KHSRP	YWHAZ	PARN	TARS1	CNOT2	CEBPG	DNAJA4	CHAC1	ATP6V0D1	NFYB	PDIA6	PDIA5	UQCRC1
																				-0.24562
-0.863111	-0.279781	-0.785691	-0.306012	-0.415255	-0.290902	-0.764394	-0.653309	-0.429296	-0.287821	-0.534155	-0.548202	-0.30262	-0.792265	-0.636333	-1.655429	-0.490964	-0.388198	-0.276441	-0.281726	
1	1	1	1	1	1	1	1	1	1	1	1	1	1	1	1	1	1	1	1	1

UQCRC2	ABCB7	MRPL15	CS	OAT	UQCRH	ACAT1	COX4I1	SLC25A5	SLC25A12	SLC25A4	NDUFB8	NDUFB7	NDUFB3	NDUFA9	NDUFA8	NDUFA5	IMMT	ATP6V1C1	MRPS30	COX5B
-0.199324	-0.359523	-0.267959	-0.193494	-0.284649	-0.415714	-0.387168	-0.327032	-0.46618	-0.798534	-0.213034	-0.206602	-0.370243	-0.262499	-0.337153	-0.232057	-0.510527	-0.279889	-0.449267	-0.324875	-0.249306
1	1	1	1	1	1	1	1	1	1	1	1	1	1	1	1	1	1	1	1	1

COX6C	HTRA2	ATP5MC3	ATP5MC1	NDUFV1	BCKDHA	NDUFS8	NDUFS3	NDUFS2	NDUFS1	TIMM8B	GLUD1	HADHA	TIMM9	LRPPRC	IDH3B	PDHB	PDK4	POR	COX7A2L	SUCLG1
-0.202417	-0.31449	-0.598709	-0.223704	-0.264323	-0.463564	-0.247426	-0.287941	-0.348067	-0.509595	-0.222328	-0.634411	-0.520169	-0.267248	-0.371318	-0.201849	-0.199353	-0.291027	-0.207236	-0.278746	-0.227585
1	1	1	1	1	1	1	1	1	1	1	1	1	1	1	1	1	1	1	1	1

MGST3	ACADSB	NNT	ATP6V0B	ATP5F1A	ATP5F1B	ATP5PD	ATP5PB	ATP5F1C	ATP5F1D	ATP5PO	PHYH	OXA1L	ATP6V1D	ATP5MF	BAX	PSMD8	CLNS1A	SET	MRPL23	RPLP0
-0.346455	-0.723636	-0.312404	-0.297319	-0.375519	-0.340766	-0.420528	-0.455353	-0.230902	-0.759865	-0.192599	-0.378822	-0.33471	-0.201734	-0.218683	-0.201187					
																-0.323487	-0.181337	-0.186035	-0.371543	-0.235246
1	1	1	1	1	1	1	1	1	1	1	1	1	1	1	1	1	1	1	1	1

PSMA6	PSMA7	PSMB3	APEX1	RUVBL2	EIF1AX	UBE2E1	SRSF3	MRPL9	ACP1	PRPS2	EIF4H	HNRNPA1	SSB	CNBP	EIF3J	EIF3D	EIF3B	UBE2L3	SNRPD2	SNRPD3
-0.34788	-0.32142	-0.279229	-0.199993	-0.350786	-0.294639	-0.334851	-0.27291	-0.195937	-0.515469	-0.493967	-0.342389	-0.321371	-0.239355	-0.409074	-0.227889	-0.530782	-0.420485	-0.448199	-0.462748	-0.523199
1	1	1	1	1	1	1	1	1	1	1	1	1	1	1	1	1	1	1	1	1

PCBP1	DHX15	CCT3	PABPC4	CANX	PABPC1	CCT7	CCT4	HDGF	EEF1B2	PPIA	YWHAE	RRM1	POLE3	RPL34	HNRNPR	PRDX3	HNRNPC	RACK1	AP3S1	RPL14
-0.513453	-0.224433	-0.411581	-0.286722	-0.262027	-0.517767	-0.220069	-0.259854	-0.498546	-0.357203	-0.285457	-0.24975	-0.267621	-0.295252	-0.278283	-0.338106	-0.180227	-0.230375	-0.201899	-0.545903	-0.198126
1	1	1	1	1	1	1	1	1	1	1	1	1	1	1	1	1	1	1	1	1

RPS3	RPS2	SF3A1	RPS5	ILF2	ERH	SNRPA	GLO1	PHB	COPS5	EIF4G2	HDAC2	MRPS18B	C1QBP	PTGES3	RSL1D1	PRPF31	FBL	TUFM	CBX3	FAM120A
-0.374177	-0.386144	-0.497885	-0.476977	-0.373582	-0.546631	-0.214723	-0.344138	-0.317869	-0.185523	-0.319099	-0.20575	-0.438535	-0.356357	-0.235117	-0.384919	-0.185738	-0.440552	-0.345365	-0.265012	-0.317953
1	1	1	1	1	1	1	1	1	1	1	1	1	1	1	1	1	1	1	1	1

ABCE1
-0.216458
1

Supplementary Table 10. Differentially expressed genes comparing pazopanib 5 µM and control						
**Gene**	**baseMean**	**log2FoldChange**	**lfcSE**	**stat**	**pvalue**	**padj**
MEGF10	1370.809837	-1.520507013	0.155437097	-9.78213724	1.34E-22	1.57E-18
TP63	1564.528244	-1.463462732	0.150026281	-9.754709095	1.76E-22	1.57E-18
ETV5	937.0777055	-1.674222481	0.188046851	-8.903219992	5.42E-19	3.22E-15
CHAC1	284.0626018	-2.517096296	0.308569995	-8.15729442	3.43E-16	1.53E-12
NUPR1	351.4683748	-1.694150785	0.211842131	-7.997232556	1.27E-15	4.54E-12
SLC1A4	854.7501036	-1.483659658	0.188304148	-7.879059875	3.30E-15	9.80E-12
EMP1	3891.672358	-0.86353001	0.110026149	-7.848407087	4.21E-15	1.07E-11
FGFR2	1256.82837	1.043201283	0.134004723	7.784809839	6.98E-15	1.35E-11
DAB2	899.6037513	-0.733526194	0.094315387	-7.77737561	7.40E-15	1.35E-11
ANKRD28	1255.30046	-0.783794983	0.100821789	-7.774063439	7.60E-15	1.35E-11
ADM2	99.77572532	-2.050719428	0.268136389	-7.648045962	2.04E-14	3.31E-11
VEGFA	2681.437446	-1.006592975	0.133661388	-7.530918172	5.04E-14	7.48E-11
ARHGEF2	2100.333163	-1.070551459	0.142504995	-7.512378462	5.81E-14	7.96E-11
GGT8P	67.41487044	5.005995116	0.671753448	7.45213163	9.18E-14	1.17E-10
INHBE	581.8869342	-2.336273441	0.314438904	-7.429975777	1.09E-13	1.29E-10
TRIB3	446.0245042	-1.899889968	0.258085956	-7.361462038	1.82E-13	2.03E-10
FGFR3	1199.154428	1.147287632	0.166584658	6.887114619	5.69E-12	5.97E-09
ENOX1	386.832367	-0.970441068	0.141321858	-6.866885875	6.56E-12	6.32E-09
SERPINI1	220.668581	-1.909990435	0.27829674	-6.863143403	6.74E-12	6.32E-09
KLF5	170.646536	-1.56448958	0.231011877	-6.77233395	1.27E-11	1.13E-08
DDR2	6647.2306	-0.985716981	0.145939252	-6.754296493	1.44E-11	1.22E-08
PCK2	1114.806254	-1.737042749	0.25887492	-6.709969224	1.95E-11	1.58E-08
SESN2	485.0802846	-1.714873077	0.259862054	-6.599166932	4.13E-11	3.20E-08
SH2B3	1069.582903	-1.089212137	0.165268899	-6.590545121	4.38E-11	3.25E-08
ETV1	508.5627055	-0.874241519	0.133171937	-6.564757866	5.21E-11	3.71E-08
SARS1	2492.479659	-0.973988052	0.148895565	-6.541417463	6.09E-11	4.18E-08
SYNE3	175.8323816	-1.629662885	0.250752442	-6.499090786	8.08E-11	5.33E-08
ACACA	7817.710493	0.775615099	0.120068599	6.459766404	1.05E-10	6.67E-08
ENPP1	6751.73869	-1.064568179	0.166054903	-6.410940948	1.45E-10	8.89E-08
CARS1	1189.601795	-1.089466801	0.173746473	-6.27043981	3.60E-10	2.14E-07
FZD5	701.8429258	0.916630294	0.146726011	6.247224273	4.18E-10	2.40E-07
ASNSP1	104.0703474	-1.592649448	0.255348539	-6.237159031	4.46E-10	2.48E-07
LINC01945	105.4452877	1.729310181	0.278431137	6.210908023	5.27E-10	2.84E-07
ABCA1	1661.332255	3.202403329	0.529093713	6.052620269	1.43E-09	7.47E-07
EVA1A	29.20459275	-2.538225217	0.420350297	-6.038357133	1.56E-09	7.76E-07
CEBPG	1067.601958	-0.928134422	0.153734791	-6.037243853	1.57E-09	7.76E-07
SCUBE1	56.75381509	3.309181034	0.563384917	5.873748005	4.26E-09	2.05E-06
FASN	22164.87325	0.946059447	0.161830261	5.845998418	5.04E-09	2.36E-06
MTHFD2	3744.058769	-0.96143428	0.170129755	-5.651182403	1.59E-08	7.28E-06
KIF26B	1524.503285	-0.913548812	0.165523837	-5.51913749	3.41E-08	1.52E-05
WNT16	1005.059189	1.342467359	0.245005327	5.47933946	4.27E-08	1.74E-05
MKNK2	817.8653136	-0.889187877	0.162290273	-5.478996734	4.28E-08	1.74E-05
PSPH	515.7850652	-1.273657684	0.232627105	-5.475104387	4.37E-08	1.74E-05
MDFI	512.0464592	0.876482299	0.160173158	5.472092262	4.45E-08	1.74E-05
ASIC3	291.3354864	1.468416181	0.268407078	5.470854907	4.48E-08	1.74E-05
PNMA2	170.5524373	-1.070302713	0.195768033	-5.467198598	4.57E-08	1.74E-05
FAT3	476.5635213	0.776568733	0.142052353	5.46677837	4.58E-08	1.74E-05
VSNL1	442.0194419	0.76411795	0.139870184	5.463051026	4.68E-08	1.74E-05
TEAD1	3036.650945	-0.506302488	0.092892006	-5.450441978	5.02E-08	1.83E-05
PCDH18	3744.86059	-0.530387827	0.09743309	-5.44361086	5.22E-08	1.86E-05
TNRC18	2917.136574	-0.506832754	0.0935747	-5.416343872	6.08E-08	2.13E-05
FLRT1	148.7061083	1.325328013	0.244998248	5.40954077	6.32E-08	2.17E-05
TARS1	2298.96427	-0.603380762	0.112099843	-5.382529926	7.34E-08	2.47E-05
GARS1	4719.981164	-1.012246567	0.188174289	-5.379303265	7.48E-08	2.47E-05
FAM160A1	375.6116135	0.987022237	0.185112357	5.332016999	9.71E-08	3.15E-05
SLC4A7	1411.882539	0.645091729	0.121745602	5.298686094	1.17E-07	3.71E-05
AARS1	5226.100532	-0.834137764	0.158073652	-5.276893102	1.31E-07	4.11E-05
GPT2	958.9596843	-1.327400987	0.252712303	-5.252617181	1.50E-07	4.57E-05
CTH	409.4931994	-1.591558631	0.303108118	-5.250795133	1.51E-07	4.57E-05
IGFN1	67.19427082	2.279588526	0.435667116	5.232408966	1.67E-07	4.97E-05
SLC6A9	214.5459559	-1.777534106	0.341761159	-5.201100417	1.98E-07	5.79E-05
GJB2	384.0161695	-1.989425906	0.38342909	-5.188510626	2.12E-07	6.06E-05
PDE7B	588.4737113	-0.988998805	0.190685883	-5.186533934	2.14E-07	6.06E-05
CNIH3	94.77907642	-1.749990458	0.339250976	-5.158394768	2.49E-07	6.94E-05
DDIT3	112.6889206	-1.273042266	0.247058963	-5.152787208	2.57E-07	7.04E-05
ERRFI1	1293.11125	-1.284770759	0.250403038	-5.130811395	2.88E-07	7.79E-05
PHGDH	3666.170368	-0.960970824	0.187633402	-5.12153388	3.03E-07	8.06E-05
MLLT11	765.930871	0.585125666	0.114621937	5.104831426	3.31E-07	8.68E-05
MAP4K4	3367.654805	-0.473237967	0.093390378	-5.067309686	4.03E-07	0.000104202
INPP5B	900.2445346	-0.594261363	0.11914813	-4.987584486	6.11E-07	0.000155642
PSAT1	2659.641316	-1.448181355	0.291091847	-4.974997994	6.52E-07	0.000163764
ENDOD1	484.6659608	0.64937547	0.130910905	4.960438329	7.03E-07	0.000174077
C2orf27A	317.6805717	0.893342874	0.182536463	4.894051641	9.88E-07	0.000241134
ULBP1	38.8190568	-2.081827841	0.425949291	-4.887501604	1.02E-06	0.00024457
DGKH	2305.934337	0.778350762	0.159310885	4.885734979	1.03E-06	0.00024457
RGS16	128.7159726	-1.179494507	0.241534588	-4.883335824	1.04E-06	0.00024457
EPSTI1	121.3536458	-1.561477952	0.320895987	-4.865994007	1.14E-06	0.000261457
TMED8	1299.247249	0.558814572	0.114917352	4.862751897	1.16E-06	0.000261457
TRIB2	994.0410819	-0.821513913	0.168948704	-4.862504979	1.16E-06	0.000261457
FLNB	4439.144482	0.725693475	0.149395831	4.857521584	1.19E-06	0.00026477
FKBP9P1	40.23713148	-1.515534922	0.313855323	-4.828769212	1.37E-06	0.000302235
YOD1	809.9112752	0.555808538	0.11516449	4.826214575	1.39E-06	0.000302402
LGR4	2336.71897	1.044909185	0.216902871	4.817405968	1.45E-06	0.000312251
DDIT4	1455.799523	-1.290036837	0.268950697	-4.796555097	1.61E-06	0.000340175
ANKRD11	1201.381305	-0.756698902	0.157793188	-4.795510592	1.62E-06	0.000340175
LINC02593	162.1719556	1.450291558	0.302952773	4.787186935	1.69E-06	0.000350464
ATF4	3309.241169	-0.790231286	0.165253768	-4.781925968	1.74E-06	0.000352181
TCF4	1542.050349	-0.609225961	0.127410818	-4.78158739	1.74E-06	0.000352181

EPRS1	5918.541874	-0.666430839	0.1406546	-4.738066426	2.16E-06	0.000432019
BCAT1	5170.885937	-0.592668686	0.127351395	-4.65380599	3.26E-06	0.00064521
KHDRBS1	5176.940296	-0.416980005	0.089770224	-4.644970086	3.40E-06	0.000666048
EVI2A	254.0997233	-0.872084939	0.188112657	-4.635971606	3.55E-06	0.000688132
GCNT1	435.8747173	0.672184536	0.145191622	4.629637201	3.66E-06	0.000691696
SLC7A2	3779.857341	0.617805654	0.13347772	4.628530152	3.68E-06	0.000691696
RNF145	2079.511635	0.605322441	0.130788312	4.628260966	3.69E-06	0.000691696
ZNF521	602.3492601	-0.555958339	0.120309368	-4.621072727	3.82E-06	0.000708644
LPCAT4	728.7072973	0.678199347	0.146848902	4.618348093	3.87E-06	0.000710608
RBMS1	1446.53518	-0.415279634	0.090078104	-4.610217309	4.02E-06	0.000731435
MAPKBP1	1110.661473	0.606213506	0.131654285	4.604586218	4.13E-06	0.000743916
EIF2S2	1590.594532	-0.669912543	0.145660091	-4.599149568	4.24E-06	0.000755959
ALDH1L2	6178.704291	-0.757600674	0.165972424	-4.564617742	5.00E-06	0.000874945
RSL24D1	1971.417726	-0.712719377	0.156145799	-4.564447972	5.01E-06	0.000874945
LOC101929322	52.01762939	2.263613742	0.501876104	4.510303883	6.47E-06	0.001119975
PTX3	224.5853668	-1.601570461	0.356925125	-4.487132876	7.22E-06	0.001236915
FAM210B	332.9014007	-0.836272689	0.186576512	-4.482197039	7.39E-06	0.001253823
DIPK1A	298.7619803	0.637291572	0.142761715	4.464022942	8.04E-06	0.001352218
ABCB6	330.0623204	0.666630393	0.149460483	4.460245129	8.19E-06	0.001363413
DDHD1	891.2273202	0.523258419	0.117784335	4.442512837	8.89E-06	0.001467086
SHMT2	2851.223466	-0.651480683	0.147762607	-4.408968523	1.04E-05	0.001698035
NCALD	254.6390308	0.854316252	0.19386961	4.4066538	1.05E-05	0.001700672
ST6GAL2	242.0788132	-1.048432007	0.238122802	-4.402904717	1.07E-05	0.001714751
LGALS3	571.1511603	-0.852643577	0.194461408	-4.384641594	1.16E-05	0.001848457
CH25H	897.405334	-1.271159712	0.290123055	-4.381450173	1.18E-05	0.001859147
PIH1D1	394.7265245	-0.594128665	0.135703893	-4.378125417	1.20E-05	0.00187117
TBX2	704.9718108	-0.94052819	0.215359442	-4.367248462	1.26E-05	0.001949687
IGF2BP3	625.944898	-0.643030324	0.147939735	-4.346569385	1.38E-05	0.002124318
MXRA5	2672.739673	1.040097404	0.239665024	4.339796383	1.43E-05	0.002154222
CRISPLD1	1019.426589	-0.549906509	0.126713994	-4.339745702	1.43E-05	0.002154222
AP2A2	1517.721479	-0.367380715	0.084777789	-4.333454756	1.47E-05	0.002198111
AKNA	287.9461736	-1.501710193	0.34675656	-4.33073334	1.49E-05	0.002206911
RSU1	1397.232943	-0.646817557	0.149762384	-4.318958745	1.57E-05	0.002294083
SH2B1	1095.97054	0.496408657	0.114948023	4.318548882	1.57E-05	0.002294083
ASS1	150.2692757	-1.290678267	0.301325163	-4.283340479	1.84E-05	0.002667322
PTPN13	3029.582206	-0.607664824	0.14211371	-4.275905713	1.90E-05	0.002735683
PTGFR	385.7532271	-0.851246405	0.200491586	-4.245796149	2.18E-05	0.003105222
ZC3H4	1122.482013	-0.444607327	0.104911006	-4.237947419	2.26E-05	0.003190241
OTULINL	107.5133133	-0.784544634	0.185664434	-4.225605387	2.38E-05	0.003343695
SLCO5A1	456.4794914	0.555137787	0.131726611	4.214317685	2.51E-05	0.003487909
BEND4	280.7322132	0.705488062	0.167752279	4.205534884	2.60E-05	0.003598057
SPAG4	130.8429546	1.204048387	0.286583253	4.201391304	2.65E-05	0.003636371
NARS1	3267.746256	-0.656558382	0.156803346	-4.187145237	2.82E-05	0.00384266
SLC1A1	377.0456199	0.641114227	0.153302109	4.182031363	2.89E-05	0.003900367
MYLIP	840.7969809	0.649473381	0.155703748	4.171212238	3.03E-05	0.00405845
ARFGAP1	1782.99054	0.50950966	0.122246163	4.167898984	3.07E-05	0.00405845
SMILR	132.6328091	-1.260415585	0.302412357	-4.167870638	3.07E-05	0.00405845
RIN1	524.9199692	-0.509149237	0.122432387	-4.158615616	3.20E-05	0.004195227
H2AC20	1402.113432	-0.696365269	0.167518436	-4.15694706	3.23E-05	0.004195227
PDCD4	438.8731818	-0.495079825	0.119248559	-4.15166297	3.30E-05	0.004262184
CTDSP1	1047.490731	-0.525373642	0.126708534	-4.146316155	3.38E-05	0.004331506
NONO	3869.018038	-0.349504055	0.084674343	-4.127626427	3.67E-05	0.004665364
USP3	1443.28335	-0.432146851	0.104790519	-4.123911723	3.72E-05	0.004707661
LOC105369147	297.2162382	-0.691677567	0.168109246	-4.114452857	3.88E-05	0.004870367
WARS1	2508.283813	-0.98122224	0.239423003	-4.0982789	4.16E-05	0.005154074
HHIP	93.82828911	-0.921156385	0.224774487	-4.098135859	4.16E-05	0.005154074
DUSP7	1262.342581	-0.710599611	0.173671862	-4.091622005	4.28E-05	0.005239127
EPS8	933.5141678	-0.608309634	0.148689242	-4.091147598	4.29E-05	0.005239127
KIF21B	401.505248	-0.726451843	0.178121378	-4.078409064	4.53E-05	0.005496916
UBXN2B	950.7126521	-0.470251009	0.115395999	-4.075106715	4.60E-05	0.005537836
TES	851.8282849	-0.557335738	0.136912532	-4.070743041	4.69E-05	0.005604739
TXNIP	1328.404114	-1.320267606	0.324474147	-4.068945454	4.72E-05	0.005610496
HEATR5A	968.0536453	0.551236324	0.135868708	4.057124938	4.97E-05	0.005862958
EFHD1	80.46519182	0.922927191	0.227797948	4.051516698	5.09E-05	0.005961979
OSTM1	326.3998767	0.513869642	0.12689856	4.04945212	5.13E-05	0.005961979
HNRNPA1	9542.761656	-0.263026405	0.064967129	-4.04860747	5.15E-05	0.005961979
IMPA1	453.6143608	0.465623764	0.115100357	4.045372037	5.22E-05	0.006005926
HINT2	103.3884788	-0.933547328	0.230924169	-4.042657514	5.28E-05	0.006036958
RGS13	26.68883798	-1.745987915	0.432589333	-4.036132614	5.43E-05	0.006167707
PITPNM2	799.8747222	0.596718453	0.148040751	4.030771597	5.56E-05	0.006270164
LGALS1	3389.748428	-0.443319997	0.11011901	-4.025826197	5.68E-05	0.006322386
MARS1	3622.592781	-0.854405808	0.212243991	-4.02558302	5.68E-05	0.006322386
LINC01614	4926.718314	-0.740273157	0.183946346	-4.024397183	5.71E-05	0.006322386
KIT	14.95510558	3.349703026	0.833269183	4.019953089	5.82E-05	0.00640307
SMARCE1	690.5098271	-0.459849215	0.114705169	-4.00896682	6.10E-05	0.006667193
SH3KBP1	693.2882385	-0.569276776	0.142219246	-4.002811086	6.26E-05	0.006801403
PAWR	1792.741928	0.623034828	0.156891274	3.971124797	7.15E-05	0.007725682
LPCAT1	2431.957346	0.67071066	0.169866365	3.948460674	7.87E-05	0.008443603
SEC63	1459.738616	-0.422155859	0.107056771	-3.943289661	8.04E-05	0.008576167
KIF7	520.5179784	0.565583685	0.143551422	3.939937881	8.15E-05	0.008645109
ASNS	92.86641594	-1.168640191	0.296740601	-3.938255122	8.21E-05	0.008654436
HTR1F	380.407911	-0.652964987	0.165876839	-3.93644461	8.27E-05	0.008668664
ETV4	77.72926568	-0.960920417	0.244212265	-3.934775409	8.33E-05	0.008678083
SLC7A5	3856.887198	-1.159539953	0.295209953	-3.927848439	8.57E-05	0.008879871
BBOX1-AS1	43.37246849	1.573201391	0.401006933	3.923127666	8.74E-05	0.009003406
FAM241A	289.9879321	-0.707645345	0.180904878	-3.911698521	9.16E-05	0.009313661
FBXO32	448.8015592	-1.507181847	0.385304784	-3.911661393	9.17E-05	0.009313661
UHMK1	2206.804741	0.428275979	0.109510787	3.910810886	9.20E-05	0.009313661
KYAT1	168.5161346	0.758172179	0.193976461	3.90857826	9.28E-05	0.00934703
HSPG2	2691.317261	0.604140099	0.155100619	3.895149496	9.81E-05	0.009824845

LAMB1	9020.262989	-0.530182183	0.136305344	-3.889665405	0.000100383	0.009993389
ZNF74	460.5028743	0.556775495	0.143326092	3.884676461	0.000102466	0.010144159
RANBP9	1132.242207	-0.438478483	0.11335072	-3.868334332	0.000109581	0.010766547
ITGA3	66.21679946	-1.314771754	0.340028938	-3.866646647	0.000110342	0.010766547
IVNS1ABP	2186.069128	0.721817739	0.1867018	3.866153083	0.000110566	0.010766547
BICD1	288.66776	0.636856684	0.164790672	3.86464037	0.000111253	0.010774611
H3C13	594.0920824	-0.869136737	0.224981284	-3.863151281	0.000111934	0.010781932
CCDC152	218.8703233	-0.61320375	0.159527009	-3.843886711	0.000121101	0.011559861
PARVG	267.8867492	-0.802234742	0.208726721	-3.843469283	0.000121307	0.011559861
DOCK5	3356.944962	0.330101109	0.086069788	3.835272698	0.000125425	0.011888694
CUBN	1226.280583	-0.696112866	0.181621297	-3.832771141	0.000126708	0.011946737
H4-16	212.3001416	-0.735849236	0.192451752	-3.823551769	0.000131543	0.012337345
STXBP5	1037.544882	0.333999865	0.087456135	3.819055859	0.000133963	0.012498577
PLEKHA3	772.7889007	0.543750253	0.142721933	3.809857678	0.000139047	0.012905282
EIF4EBP1	2113.716083	-0.78482071	0.206134684	-3.807320018	0.000140481	0.012970828
PYROXD1	240.3147164	-0.607145954	0.159581823	-3.804605947	0.00014203	0.013046271
ANXA1	3273.458104	-0.4685603	0.123419535	-3.79648409	0.000146763	0.01341186
JDP2	982.9822375	-0.503192721	0.132679936	-3.792530632	0.00014912	0.013557733
ATP2A1	89.96353491	1.115520116	0.295531913	3.774618131	0.000160253	0.014495978
SEMA6A	234.1020627	-0.959173527	0.25429337	-3.771917161	0.000161998	0.014579824
SPTLC3	131.7782332	-0.896476175	0.237985497	-3.766936165	0.000165263	0.014798949
NIBAN1	265.5355598	-1.318567738	0.350242284	-3.764730301	0.000166729	0.014826196
PTAR1	1257.512734	0.495790952	0.131719934	3.76397815	0.000167232	0.014826196
CRISPLD2	389.969977	2.354062307	0.625647357	3.762602498	0.000168154	0.014834211
ARF5	792.0163921	-0.547372633	0.145966709	-3.749982703	0.000176847	0.015524184
SLC7A11	1270.91535	-1.845604298	0.492392318	-3.748239422	0.00017808	0.015555831
MYLK	501.5711404	0.575641385	0.153866074	3.741184592	0.000183155	0.015921077
EIF5A2	345.9103924	0.466668137	0.124971583	3.734194011	0.000188317	0.01629036
HERPUD1	948.7232052	-0.54410856	0.145969576	-3.727547729	0.000193352	0.016645081
PDE8A	1184.10221	0.53617372	0.144357589	3.714205296	0.000203843	0.01746388
PKDCC	310.6848366	0.947856353	0.255509301	3.709674562	0.000207526	0.017694308
SLC3A2	1611.156383	-0.781604503	0.211317989	-3.698712572	0.000216696	0.018231851
FIBIN	849.6524878	-1.070092643	0.289373723	-3.697960655	0.000217339	0.018231851
ELAC1	56.99137898	-0.91530374	0.247552857	-3.697407295	0.000217813	0.018231851
PTPN14	8160.046404	-0.326016867	0.088177513	-3.697278995	0.000217923	0.018231851
FGFR1	14803.61196	-0.711953247	0.192669177	-3.69521091	0.000219704	0.018295006
TRIM8	318.191239	-0.744993226	0.202580789	-3.677511721	0.00023552	0.019457775
S100A4	113.6606694	-0.915347043	0.248928207	-3.677152753	0.000235852	0.019457775
SPRED2	641.9328851	-0.468976548	0.128047335	-3.662524872	0.000249741	0.020508726
SLC6A6	801.1500921	0.521642108	0.142523074	3.660053738	0.000252162	0.020612538
ZNF483	488.5500294	0.528487787	0.144594376	3.654967784	0.000257214	0.020878639
PLCE1	908.554713	0.516642842	0.141377874	3.654340166	0.000257844	0.020878639
FAM53C	812.4047109	-0.343958589	0.094173805	-3.652380692	0.00025982	0.020878639
LINC02614	24.3532505	1.575619641	0.43142836	3.652100296	0.000260104	0.020878639
PRUNE2	1367.315534	0.923877921	0.253152711	3.649488559	0.000262763	0.020914018
RNF20	865.5514802	-0.446886818	0.122456144	-3.649362156	0.000262892	0.020914018
ALS2	560.754653	0.440734365	0.120995086	3.642580711	0.000269918	0.021349912
TSEN15	899.3800563	-0.584867187	0.16059961	-3.641772147	0.000270768	0.021349912
RBM3	2916.182372	-0.63057527	0.173516008	-3.634104291	0.000278948	0.021898034
LZTS1-AS1	5.310156048	5.355404356	1.475794656	3.628827584	0.000284711	0.022180558
SNW1	941.743185	-0.397143965	0.109450284	-3.628532976	0.000285036	0.022180558
MMP13	120.7100209	-1.106580729	0.305472098	-3.622526371	0.00029174	0.022439485
TMEM209	903.806185	-0.386891611	0.106804696	-3.622421358	0.000291858	0.022439485
MCM2	2532.458743	-0.376685606	0.103994443	-3.622170513	0.000292141	0.022439485
ERP29	2167.454379	-0.442679407	0.122490423	-3.613991978	0.000301518	0.023060334
PRPSAP2	581.1128456	-0.420583526	0.116758508	-3.602165997	0.000315577	0.024032382
GPR180	1042.801306	0.354843993	0.098595667	3.598981595	0.000319466	0.024225023
PHC3	3561.993217	0.297994374	0.082873213	3.595786432	0.000323413	0.024420426
PTER	377.6381666	-0.760987247	0.211940344	-3.590572857	0.000329952	0.024720386
DNAJB4	548.5918188	0.525929475	0.146562938	3.588420674	0.000332687	0.024720386
VLDLR	409.075782	-1.098083351	0.306046009	-3.587968208	0.000333265	0.024720386
CHD4	4991.576708	-0.328426172	0.091571257	-3.586563974	0.000335064	0.024720386
VPS26A	557.7013786	-0.415107822	0.115739702	-3.586563766	0.000335064	0.024720386
IARS1	13962.82168	-0.482407983	0.134523039	-3.586062176	0.000335709	0.024720386
LARS1	4734.020667	-0.36355269	0.101497266	-3.581896404	0.000341109	0.025014665
LOXL1	470.9982685	-0.615717527	0.172250026	-3.574556943	0.000350821	0.025621469
POSTN	114.8038642	-1.724078976	0.48256699	-3.572724643	0.000353286	0.025696163
NXPH3	181.5424473	1.297338854	0.363413412	3.569870597	0.000357158	0.025872149
AP5B1	434.5406844	0.49089977	0.137594527	3.567727437	0.000360091	0.025979019
KCTD15	892.3418966	-0.515289523	0.144558377	-3.564577395	0.000364443	0.026186986
RBM33	993.3361511	0.334397245	0.093917207	3.560553556	0.000370074	0.026448293
H4C5	674.8993257	-0.620658483	0.174348965	-3.559863305	0.000371048	0.026448293
TPM1-AS	670.9640049	0.976026186	0.274364018	3.557413229	0.000374525	0.026560938
ZNF680	205.5936775	0.613113789	0.172417268	3.555988301	0.000376561	0.026560938
TNFRSF21	381.5142439	0.599737735	0.168673543	3.555612361	0.0003771	0.026560938
PRR12	846.0102848	0.359600465	0.10119194	3.5536473	0.000379928	0.026651984
TRIOBP	500.0253158	-0.48433418	0.136387483	-3.551162972	0.000383533	0.026651984
PYGB	1476.428671	-0.377435018	0.106291218	-3.550952031	0.00038384	0.026651984
PLCB1	378.3087814	-0.517213264	0.14566984	-3.55058578	0.000384375	0.026651984
S100A6	3105.037912	-0.687031482	0.193641214	-3.547961031	0.000388226	0.026814656
FYN	1319.548862	-0.758597454	0.214069716	-3.543693474	0.000394564	0.027147201
RNF152	365.6281935	-0.805726917	0.227729848	-3.538082174	0.000403045	0.027624057
SLC1A5	5762.532029	-0.556275191	0.157706318	-3.527285398	0.000419844	0.028665208
RBMS3	356.3422634	-0.505846415	0.143498552	-3.525097705	0.000423327	0.028792673
MIR1915HG	335.7482316	0.524233369	0.14891896	3.520259392	0.000431125	0.029211584
HCN1	45.48958635	-1.262732261	0.359120156	-3.516183205	0.000437799	0.029551417
MICA	925.6977148	0.458617939	0.130566799	3.512515772	0.000443886	0.029724241
GRIK3	237.0380291	-0.715933127	0.203848314	-3.512087558	0.000444601	0.029724241
SPRED1	2034.152742	-0.577142188	0.164351529	-3.51163261	0.000445363	0.029724241
TMEM120B	963.4119186	0.427658858	0.121988824	3.505721619	0.000455371	0.030235714

ZMAT5	80.53977089	-0.919238506	0.262256743	-3.505109134	0.00045642	0.030235714
EIF2S3	8355.471997	-0.393028738	0.112217531	-3.502382671	0.000461117	0.03043371
CAV1	1868.366729	-0.496456334	0.141893423	-3.498797348	0.000467362	0.030551216
IRS1	4010.517298	-0.327455224	0.09359657	-3.498581477	0.00046774	0.030551216
TENM2	250.0358658	-0.762282465	0.217893958	-3.498410288	0.000468041	0.030551216
PCNX1	1997.085123	0.305260469	0.087337609	3.495177774	0.000473746	0.030810786
SERPINE1	89.4576001	1.692767094	0.484861246	3.491240246	0.000480784	0.030917421
SNRPD3	811.2542085	-0.422554321	0.121040723	-3.491009554	0.000481199	0.030917421
ADD1	1814.395028	-0.354707937	0.101640801	-3.489818381	0.000483349	0.030917421
CDT1	658.5674928	-0.405600412	0.116233598	-3.48952815	0.000483874	0.030917421
SPRY2	203.9648315	-1.02220013	0.292942273	-3.489425135	0.000484061	0.030917421
NR2F2	732.8847715	-1.112660102	0.319135972	-3.486476613	0.000489428	0.031148607
DSE	1594.29273	0.330248161	0.095068058	3.473807776	0.000513128	0.032540747
ARMT1	732.297584	-0.478343944	0.137862412	-3.469719828	0.000521002	0.032809902
PTEN	2692.146959	-0.507111243	0.146154533	-3.469692194	0.000521055	0.032809902
EMB	613.3941487	-0.397450667	0.11466106	-3.466309014	0.000527656	0.033108585
SBNO2	1019.04851	0.340730168	0.098341258	3.46477332	0.000530679	0.033181377
FER1L4	254.9419183	1.199299859	0.346249332	3.463688588	0.000532823	0.033198969
SOX13	326.7102832	0.556979515	0.161149345	3.456294007	0.000547658	0.033973319
SRPX	491.4454388	-0.468509449	0.135579658	-3.455602819	0.000549064	0.033973319
NRP2	1396.589798	0.883479456	0.255855231	3.453044329	0.000554298	0.034178508
RNF125	269.6067152	-0.514825731	0.149241226	-3.449621418	0.000561373	0.034495416
EYA2	320.9452286	2.027117185	0.588418849	3.445024217	0.000571008	0.034966896
PRNP	1402.824968	-0.593311349	0.172635032	-3.436795781	0.000588639	0.035923121
EPC2	321.2592615	-0.709629768	0.206587951	-3.435000754	0.000592552	0.036038498
ZNF692	669.9863501	0.550519554	0.160750887	3.424675059	0.000615535	0.037308967
INPP4B	509.6457161	-0.622299719	0.182236392	-3.414793894	0.000638303	0.038557823
VPS33A	632.1441486	-0.444399874	0.1302551	-3.411765644	0.000645436	0.038856978
EIF1	5325.214306	-0.537121922	0.15790397	-3.401573254	0.000669992	0.040199502
THOC5	820.8468142	-0.438243484	0.12890801	-3.39966061	0.000674695	0.040345881
PARVA	863.2764357	-0.534058187	0.157244013	-3.396365792	0.00068287	0.04069816
ST8SIA6	86.10200525	-0.78039648	0.230121114	-3.391242408	0.000695765	0.04132846
MCMBP	1247.019938	-0.307393798	0.090722721	-3.388277978	0.000703329	0.041638972
ZNF699	293.6609452	0.450354542	0.132992332	3.386319622	0.000708368	0.041798419
ANP32B	1868.044205	-0.38590459	0.114084045	-3.38263418	0.000717942	0.042223517
STAT6	1841.601495	-0.281561485	0.083363222	-3.377526428	0.000731409	0.042874055
LMO4	540.9916587	-0.55130173	0.163368489	-3.374590381	0.000739256	0.043191962
ZNF592	969.2089874	0.353225946	0.104798251	3.370532825	0.00075023	0.043689846
SLC6A10P	109.587393	0.863295664	0.256414056	3.366803192	0.000760449	0.044140742
GET3	567.6267111	-0.389469222	0.115849919	-3.361842837	0.000774242	0.044795417
KIAA1549	506.7318749	0.402021239	0.119706103	3.358402205	0.000783945	0.045016297
SLC25A30	219.6322441	0.595730052	0.177408427	3.357958022	0.000785205	0.045016297
LOC100506990	347.8354557	0.491082258	0.146273259	3.357293472	0.000787095	0.045016297
DECR1	631.8111303	-0.389891951	0.116145799	-3.356918249	0.000788164	0.045016297
GREM2	215.46277	0.59585143	0.177569356	3.355598298	0.000791935	0.045087166
CHIC2	122.2606197	-0.888344517	0.265672796	-3.343754155	0.000826529	0.046906863
FZD1	1853.888489	-0.517329207	0.154962471	-3.338416087	0.000842575	0.047665657
SEPTIN2	5133.305687	-0.330854588	0.099178104	-3.335964028	0.000850042	0.047899288
EPB41L3	40.66884059	-2.521903492	0.756125398	-3.335297953	0.00085208	0.047899288
TTC7A	411.8518357	0.46532975	0.139567717	3.334078688	0.000855824	0.047958463
GNAI1	516.339089	0.447145927	0.134168301	3.332724086	0.000860002	0.048041482
CXCL14	127.6096961	1.535808553	0.460983947	3.331587927	0.00086352	0.048087279
NEK4	431.8811387	-0.367376675	0.110427114	-3.3268702	0.000878273	0.048653975
CASK	919.2687054	-0.885187747	0.266159149	-3.325783654	0.000881703	0.048653975
SEMA3A	1643.056044	-0.440873237	0.132564531	-3.325725467	0.000881887	0.048653975
FLOT2	781.3359465	-0.478514701	0.143934485	-3.324531306	0.000885673	0.048712009
INHBB	4.605548129	-5.181721323	1.559066341	-3.323605409	0.000888618	0.048723626
FAM131A	718.1314833	0.316137301	0.095223071	3.319965404	0.000900286	0.049211955
EWSR1	2589.076006	-0.401685693	0.121219296	-3.313710813	0.000920667	0.050172114
CES2	481.1757853	-0.512064505	0.154741333	-3.309164366	0.000935749	0.050601805
PREB	1122.352877	0.300603316	0.090862395	3.308335816	0.000938522	0.050601805
GPR1	40.53230172	-1.149325002	0.347442519	-3.307957253	0.000939792	0.050601805
DHX40	1208.788068	-0.519520896	0.157074084	-3.307489588	0.000941362	0.050601805
YRDC	290.9147838	0.560899022	0.169629404	3.306614357	0.000944308	0.050601805
RHOJ	134.926763	-0.662615142	0.200413839	-3.30623446	0.000945589	0.050601805
TLN2	2015.443443	0.290825716	0.088029141	3.303743644	0.00095403	0.050794463
MOCOS	988.0578896	-0.550438287	0.166623213	-3.303491014	0.00095489	0.050794463
CHURC1	576.7836266	-0.396978772	0.120219366	-3.302119995	0.00095957	0.050891486
RBMX	3811.92545	-0.280858973	0.085119404	-3.299588116	0.000968268	0.051200416
TRPM7	3935.920886	-0.327311646	0.099280217	-3.296846609	0.000977769	0.051549825
KHDRBS3	31.68373364	-1.395939223	0.42414725	-3.291166504	0.000997728	0.052388671
CEP68	633.3203252	-0.383951243	0.116679428	-3.290650723	0.000999559	0.052388671
PRDX2	153.6221299	-0.832041222	0.253261813	-3.285300744	0.001018736	0.053237156
SRPK2	867.0585326	-0.405050437	0.123348945	-3.283777077	0.001024259	0.053328884
NEFL	764.3128026	-0.60151182	0.183210819	-3.283167579	0.001026476	0.053328884
ADGRL2	1204.484255	0.396739908	0.120883082	3.282013501	0.001030687	0.053391968
BORCS7	45.11378331	-1.070047988	0.326379395	-3.278540268	0.001043455	0.053896707
OLFML2A	938.3308291	-0.756017634	0.230658603	-3.277647677	0.00104676	0.053911145
KAT6B	491.6346123	-0.383363082	0.117005574	-3.276451457	0.001051204	0.053984012
DUSP9	39.61543978	1.208215031	0.369318699	3.271469961	0.0010699	0.054786235
CAMK2N1	15.80373607	-1.955963738	0.598379398	-3.268768517	0.001080166	0.055153478
OAZ2	665.5008633	-0.450536657	0.137954812	-3.265827784	0.001091446	0.055570196
PANK3	3826.11496	0.506640618	0.155405472	3.260120847	0.001113648	0.056539028
ESPNP	3.145084436	4.888460546	1.501133906	3.256511977	0.001127902	0.057100024
HS3ST3A1	403.0641183	0.479931979	0.147473078	3.254370124	0.001136441	0.05736935
TPGS1	121.3204522	-0.691415263	0.21264249	-3.251538594	0.001147822	0.057780188
C18orf25	656.667122	0.315124688	0.096980546	3.2493598	0.001156651	0.057906327
CALCOCO2	708.9607858	-0.50558911	0.155599291	-3.249302154	0.001156885	0.057906327
GFRA2	6.766163118	-4.695808998	1.4455232	-3.248518596	0.001160076	0.057906327
CHSY3	922.0821479	0.404570283	0.124774784	3.242404187	0.001185258	0.058998035

TWF2	443.3685221	-0.502015898	0.155018802	-3.238419419	0.00120194	0.059616578
GGNBP2	697.1367612	-0.415711338	0.128391488	-3.237841901	0.001204375	0.059616578
LOC101930085	223.9692617	-0.525281492	0.162296898	-3.236546724	0.001209854	0.059721888
CARNS1	37.94620322	1.358956618	0.420202945	3.234048295	0.001220488	0.060080384
SPATA5L1	461.5122164	0.369437862	0.114288215	3.232510572	0.001227076	0.060238274
PCBP2	9121.005679	-0.471083115	0.146156985	-3.223131035	0.001267975	0.062075057
PDLIM7	1230.22989	0.443449224	0.137752028	3.219184715	0.001285556	0.062763331
C2CD2	282.0059554	0.459837595	0.14293588	3.217090034	0.00129498	0.063008188
SIPA1	388.3021939	0.382159996	0.118812357	3.216500431	0.001297643	0.063008188
PLRG1	830.9149084	-0.497943345	0.154854577	-3.21555459	0.001301927	0.063044414
TMEM47	7.76767168	-3.171736651	0.987032609	-3.213406143	0.001311707	0.063345831
KIAA1958	299.4704467	0.682560962	0.212475793	3.212417537	0.001316229	0.063392448
DHRS3	286.0663181	2.519557414	0.785922399	3.205860296	0.001346593	0.064680043
SAMD11	850.5028747	1.673457373	0.522292155	3.20406377	0.001355025	0.064910049
TGFBR3	334.3579822	-0.667062234	0.208418676	-3.200587611	0.001371477	0.065522022
FAM3C	1107.426557	0.444971382	0.139231189	3.195917414	0.00139387	0.066413807
ECHS1	847.8611573	-0.410366688	0.128542672	-3.192454929	0.00141069	0.067035969
TRPV3	112.8907961	0.748084627	0.234520968	3.189841115	0.00142351	0.067404122
IBTK	3378.583405	0.254174665	0.079724988	3.188143035	0.001431897	0.067404122
PIK3CB	645.3664574	0.377316859	0.118355665	3.187991552	0.001432647	0.067404122
NUFIP2	5333.274301	0.266342366	0.083550369	3.187805985	0.001433567	0.067404122
GSN	2311.34055	-0.607073814	0.191272238	-3.173873114	0.001504194	0.070439188
HOXB9	346.5703921	-0.479982676	0.151246128	-3.173520426	0.001506023	0.070439188
TK1	792.6307398	-0.345141243	0.108862102	-3.170444416	0.00152206	0.070973376
TCF7L2	345.5159464	-0.46731213	0.147426119	-3.169805549	0.00152541	0.070973376
CLVS2	94.69705902	0.744429712	0.234930641	3.168721243	0.001531112	0.071053151
KDM4A	1243.516659	-0.443955357	0.140400219	-3.16207025	0.001566518	0.07250739
IQGAP3	1145.142498	-0.391521393	0.123924253	-3.159360535	0.001581158	0.072995409
SNAI1	250.706038	1.096167445	0.347155913	3.15756524	0.001590926	0.073032223
LURAP1L	32.11513772	-1.534134446	0.485885061	-3.157401964	0.001591818	0.073032223
GTF2E2	1085.566117	0.335262279	0.106197933	3.156956737	0.00159425	0.073032223
BNC1	202.0404297	0.547170955	0.173543616	3.152930476	0.001616403	0.073857183
GDA	8.339912901	-3.093955996	0.982946131	-3.147635357	0.001645969	0.075015765
DOT1L	1501.132806	0.30481323	0.096996078	3.142531512	0.001674937	0.076141261
GNAS	6321.963079	-0.521929756	0.16624737	-3.139476773	0.001692498	0.076743817
ECM2	343.2007051	-0.614965778	0.19611963	-3.135666618	0.00171464	0.077484033
CCDC134	293.9856139	0.414196169	0.132119337	3.135015501	0.00171845	0.077484033
YARS1	4441.203817	-0.537013355	0.171327128	-3.134432709	0.001721867	0.077484033
DGKD	835.2265609	0.339950799	0.108486786	3.133568708	0.001726945	0.077516768
BLOC1S1	179.5551866	-0.615224155	0.196446581	-3.13176311	0.0017376	0.077799082
LOC100129617	16.5834113	1.936169514	0.619355232	3.126105042	0.001771383	0.07911288
CFL2	669.3147231	0.432700432	0.138494707	3.124310249	0.001782224	0.079362547
SNX8	450.8368569	-0.419187729	0.134195584	-3.123707328	0.00178588	0.079362547
ATP2B1	3701.62405	-0.39061981	0.125177941	-3.120516352	0.001805343	0.079931669
WSB2	924.8655926	0.389715462	0.124903242	3.120138894	0.001807658	0.079931669
SLC17A9	905.5437617	0.708151697	0.227209835	3.116729947	0.00182869	0.080661526
SNX2	1066.454715	-0.378519292	0.121526536	-3.114704874	0.00184129	0.080901921
GTF2F1	779.2633958	-0.409254112	0.131472258	-3.112855264	0.001852869	0.080901921
FAM102A	600.324259	-0.597895016	0.192086632	-3.112632105	0.00185427	0.080901921
CALCB	374.5138914	1.311513709	0.421371606	3.112487149	0.001855181	0.080901921
ALAD	556.9765931	-0.414169415	0.133078309	-3.112223311	0.00185684	0.080901921
LOC102723753	187.301438	0.727822538	0.233990976	3.110472682	0.001867882	0.081184518
XPR1	5402.306926	1.482502228	0.476762605	3.109518681	0.001873924	0.081248982
CACNA1G	1212.546436	-0.408936968	0.131571188	-3.108104252	0.001882916	0.081440702
CPN2	5.929256367	5.47863501	1.765109179	3.103850502	0.001910198	0.082420661
SPICE1	314.4746119	0.59773031	0.19267333	3.102299152	0.001920238	0.082653723
LMNA	9665.518747	-0.51749272	0.166911763	-3.10039694	0.001932615	0.082749495
SLC2A13	376.3427101	0.50138963	0.161718058	3.100393582	0.001932636	0.082749495
GPC4	1088.579862	-0.321516428	0.103721056	-3.099818303	0.001936394	0.082749495
GK5	751.9966794	0.522963423	0.168776242	3.098560648	0.001944632	0.082848979
GABARAP	1136.496537	-0.62238259	0.200906048	-3.097878815	0.001949111	0.082848979
YBX3	1966.555392	-0.365310021	0.117943213	-3.097338207	0.00195267	0.082848979
PPP1CA	688.109512	-0.461270651	0.149076215	-3.094193463	0.001973488	0.08332387
NR2F1	249.5606659	-0.567075111	0.183278779	-3.094057673	0.001974391	0.08332387
OMD	142.7932608	0.797874298	0.257916907	3.093532355	0.00197789	0.08332387
ZFAND3	1014.607459	-0.423643303	0.137026342	-3.09169242	0.001990189	0.083599955
TMEM64	1676.383571	0.332019774	0.107409776	3.09115041	0.001993826	0.083599955
SREBF1	2637.663204	0.47756559	0.154620593	3.08862863	0.002010826	0.083921475
ZNF684	52.58973927	0.892455318	0.289012463	3.087947516	0.002015441	0.083921475
ANKRD50	862.2187207	0.425495733	0.137793607	3.087920711	0.002015622	0.083921475
LPCAT3	238.9420437	0.5081519	0.164850423	3.082502856	0.002052678	0.08526507
LZTFL1	189.5663425	-0.655616366	0.212977925	-3.078330139	0.002081642	0.086267102
H2AC15	267.8649638	-0.748961921	0.243509891	-3.07569404	0.002100132	0.086831455
NFKBIL1	210.5538158	-0.631880822	0.205556431	-3.074001713	0.002112082	0.086954593
NRN1	297.5882713	-0.62488739	0.203288761	-3.073890491	0.00211287	0.086954593
CD81	2557.346218	-0.30050912	0.0977841	-3.073190033	0.002117836	0.086958141
GGA3	800.6277946	0.326684587	0.106377653	3.070988862	0.002133511	0.087400386
KLHL14	77.15071549	-0.863830582	0.282188087	-3.061187277	0.002204612	0.090105912
ACADS	150.6995505	-0.495387559	0.161917893	-3.059498554	0.002217079	0.090408101
LINC02028	3.148936958	4.544772922	1.485943592	3.058509722	0.002224409	0.090499912
UHRF1BP1	1732.603344	-0.591277423	0.193607003	-3.054008441	0.002258057	0.091659637
FBLN1	3088.919114	-0.602168751	0.197260694	-3.052654536	0.002268269	0.091864896
PCM1	3998.802586	-0.32742101	0.107317862	-3.050946065	0.002281215	0.092065678
FAM78B	22.52770805	-1.916649678	0.628278371	-3.050637692	0.002283559	0.092065678
NT5DC2	2687.026336	-0.362270449	0.11895703	-3.045389155	0.002323794	0.093476326
SLC5A3	1433.163376	0.556273891	0.182842404	3.042368064	0.002347247	0.09407624
PRICKLE3	196.5525051	0.482538656	0.158619767	3.042109219	0.002349266	0.09407624
LRP4-AS1	36.40732927	1.581236836	0.520515554	3.037828218	0.002382897	0.095084211
RIOK3	825.7751562	-0.418144481	0.137658529	-3.037548651	0.002385109	0.095084211
PMAIP1	376.1919584	-0.644206562	0.212322259	-3.034098103	0.00241256	0.095963868

APH1A	1492.612972	-0.372437827	0.122778094	-3.033422449	0.002417969	0.095964808
SNORA75	18.19778103	1.835893847	0.605444738	3.032306223	0.002426929	0.096106374
ENTPD7	848.337753	0.437310381	0.144298277	3.030600152	0.002440682	0.096436714
S100A2	143.0908137	-0.859009667	0.284113016	-3.02347875	0.002498866	0.09851723
KIAA1143	341.7168038	-0.452098829	0.149641825	-3.021206332	0.002517697	0.099040543
PSTPIP2	110.3604038	-0.818300874	0.270959829	-3.020008087	0.002527679	0.099207204
DLG1	2228.404041	-0.323251408	0.10708239	-3.018716797	0.002538477	0.099207204
DDX50	902.3283849	-0.424841877	0.140736799	-3.018697883	0.002538636	0.099207204
TFAP2A	70.91699743	-1.0848652	0.359627409	-3.01663659	0.00255596	0.099665675
ARRDC4	75.30794994	-1.142041534	0.378694496	-3.015733122	0.002563588	0.099744838
EMC2	1214.588204	0.401923519	0.13340542	3.012797523	0.002588516	0.100495312
CRK	2039.914489	-0.321553881	0.106787012	-3.011170311	0.002602428	0.100815813
PLEKHN1	83.05643646	0.904283625	0.300389278	3.010372505	0.002609275	0.100861767
FKBP14	2128.718726	0.365251407	0.121475839	3.006782332	0.002640287	0.101788259
LHX8	126.4295598	-0.801305942	0.266544152	-3.006278459	0.002644667	0.101788259
VAV3	135.7947071	-1.035436497	0.344771611	-3.003253354	0.0026711	0.102584042
KIDINS220	3212.102421	0.255134941	0.085040678	3.00015177	0.002698451	0.103411611
RARB	101.4901105	0.783552248	0.261553367	2.995764338	0.002737579	0.104546425
UPRT	114.0934018	-0.714459905	0.238509685	-2.99551738	0.002739797	0.104546425
SNAPC3	468.504363	0.359543828	0.120271498	2.989435025	0.002794939	0.10629178
TPST1	496.9692497	0.702706651	0.235114498	2.988784854	0.002800893	0.10629178
PPFIBP2	17.13721498	2.603680083	0.87123073	2.98850809	0.002803431	0.10629178
MIER2	431.3336931	0.37479665	0.125515675	2.986054535	0.002826022	0.106920848
PNPLA3	426.3885225	0.600280646	0.201254246	2.98269804	0.002857197	0.107871306
NFIA	804.8146521	-0.435842901	0.146244418	-2.980236145	0.002880263	0.108512216
CUTALP	195.2831688	-0.60640487	0.20379109	-2.97562013	0.002923968	0.109926394
SUPT6H	2568.359087	-0.354528545	0.119216059	-2.973832129	0.002941059	0.110276568
COMMD4	545.3146762	-0.407028556	0.136892128	-2.97335253	0.002945659	0.110276568
ZFP36L2	568.6323716	-0.479616359	0.161465902	-2.970387873	0.00297424	0.111113102
LZTS1	346.9806601	0.485659047	0.163546238	2.969551925	0.002982344	0.111182785
PAFAH1B1	2832.576333	-0.433858119	0.146170957	-2.968155431	0.002995928	0.111456024
C16orf72	1322.799126	-0.422411657	0.142346417	-2.967490628	0.003002414	0.111464633
H2AC14	166.8443933	-0.843281782	0.284945937	-2.959444839	0.003081939	0.114179101
OGN	678.8917891	0.388123457	0.131223581	2.957726457	0.00309917	0.114497189
PHC2	1119.503212	-0.437426291	0.147913651	-2.957308454	0.003103375	0.114497189
ZNF627	202.7118431	-0.480916233	0.16269122	-2.956006072	0.00311651	0.114560191
PIK3C3	535.7753167	-0.408474898	0.138191346	-2.955864525	0.00311794	0.114560191
RABGGTB	1001.18999	0.453609967	0.153496122	2.955188442	0.003124781	0.114575313
UBE2Q1	577.7700678	-0.367699806	0.124512553	-2.953114342	0.003145854	0.115111131
BRD3OS	408.5105277	-0.38332599	0.129846501	-2.952147249	0.003155724	0.115235661
EIF2S3B	45.62656248	-1.505710235	0.510339574	-2.950408534	0.00317354	0.115455861
ZNF850	304.7676384	0.422836403	0.143320069	2.950294439	0.003174712	0.115455861
RHOQ	1263.268496	-0.298298968	0.101216525	-2.947137013	0.003207311	0.116403818
FGF7	37.88051612	-1.307345961	0.443881678	-2.945257768	0.003226857	0.11665302
NSMF	1194.661182	-0.404734066	0.137427748	-2.945068024	0.003228837	0.11665302
BEND5	13.69847778	-2.415533259	0.820328879	-2.944591273	0.003233815	0.11665302
TCEA1	3302.448511	-0.432330082	0.146894193	-2.943139366	0.003249021	0.116699847
WDR45	289.3701463	-0.520330694	0.176802784	-2.94300058	0.003250478	0.116699847
EPHA4	159.0860034	0.714770189	0.242904876	2.942593006	0.00325476	0.116699847
HNMT	113.3998173	-0.570924145	0.194125374	-2.941007309	0.003271468	0.117063378
SMARCC2	1182.177636	-0.264324255	0.089916313	-2.939669626	0.003285624	0.117334298
FRMD4B	175.063822	0.798266074	0.271632899	2.938768008	0.003295196	0.117342514
MATN1-AS1	44.11192816	0.957892357	0.325990229	2.938408189	0.003299024	0.117342514
MPV17L	29.98027745	-1.515914824	0.516289293	-2.936173272	0.003322887	0.117955854
STX16	1215.816276	0.298273715	0.101639465	2.934625009	0.00333951	0.11828926
C22orf46	233.9540818	-0.460857432	0.157082646	-2.933853256	0.003347824	0.11828926
ENTPD4	2324.336485	0.414834802	0.141427561	2.933196319	0.003354917	0.11828926
CSPG4	7808.818335	-0.431301254	0.147059536	-2.932834317	0.003358831	0.11828926
UBE2E3	1309.191544	-0.415750681	0.141800569	-2.931939449	0.003368524	0.118396648
CYP26B1	8.762906661	2.606849522	0.889652546	2.930188344	0.003387566	0.118831552
CGAS	370.6481457	0.396856873	0.135488242	2.929087194	0.00339959	0.11901906
TXNDC17	123.8875934	-0.523079435	0.178688227	-2.927330162	0.003418857	0.119458897
SERTAD4	485.264552	-0.496272174	0.169732992	-2.923840378	0.00345742	0.120145669
ZNF587	1442.615169	0.446536177	0.152760003	2.923122329	0.003465403	0.120145669
H3P6	431.0406809	-0.670163866	0.229282613	-2.922872601	0.003468184	0.120145669
PLEKHA8	784.3174574	0.327133011	0.111971375	2.921577151	0.00348264	0.120145669
MTHFD1L	2031.227823	-0.458384953	0.156900976	-2.921492037	0.003483592	0.120145669
PRKAB2	1287.813686	0.299777544	0.102652774	2.920306311	0.003496875	0.120145669
ITGA4	5332.991127	0.44318356	0.151762995	2.920234674	0.003497679	0.120145669
UNC79	11.69854807	2.003613013	0.686146421	2.920095408	0.003499242	0.120145669
ATP10D	372.6289547	0.435865073	0.149277016	2.919840477	0.003502106	0.120145669
SMARCC1	2593.101788	-0.320838342	0.109902224	-2.919307103	0.003508104	0.120145669
EBF2	379.7505912	-0.572075933	0.195990173	-2.918901107	0.003512676	0.120145669
HUNK	142.4106462	-0.594979822	0.203933041	-2.917525372	0.003528209	0.12044577
TMEM106A	248.1608529	0.636495413	0.218221401	2.916741486	0.003537088	0.120517988
NBEAL1	1112.603254	0.363145309	0.124573673	2.915104774	0.003555691	0.120776808
ARHGAP24	221.5296029	-0.489418558	0.167903431	-2.914881228	0.003558239	0.120776808
ZFHX3	974.3314864	-0.256851329	0.088224419	-2.911340545	0.003598815	0.121921846
TMEM33	1972.293172	0.283073641	0.097319687	2.908698623	0.003629365	0.122723515
CCDC171	357.9729953	-0.619705279	0.21312038	-2.907771083	0.003640147	0.122802384
THG1L	334.6527155	-0.429068016	0.147582318	-2.907313163	0.00364548	0.122802384
BCL3	152.2869808	1.353331009	0.465650068	2.906326235	0.003657	0.122851475
SEPTIN7	3065.133245	-0.47151495	0.162255237	-2.906007584	0.003660726	0.122851475
FPGS	764.2773907	0.28857085	0.099359635	2.904306659	0.003680675	0.12328876
IL17RD	567.9393484	-0.448328168	0.154405137	-2.903583246	0.003689189	0.12334211
FOXP4	554.6364918	-0.426888554	0.147054852	-2.90292057	0.003697004	0.123371928
TBX15	513.8527668	-0.649792197	0.223891699	-2.902261227	0.003704795	0.123400823
BMP2	906.6486015	-0.854356499	0.294959795	-2.896518486	0.003773284	0.125447617
AKAP6	283.8141879	-0.45901807	0.158564974	-2.894826391	0.003793683	0.125769729
NDUFS1	1358.302665	-0.273689451	0.094553541	-2.894544708	0.003797088	0.125769729

TMEM268	515.1378016	-0.456119762	0.157852082	-2.889539086	0.00385807	0.127552532
TMTC2	2385.01411	0.256864124	0.088946216	2.887858947	0.003878738	0.127685316
STRA6	144.2939175	0.960937103	0.332757911	2.887796414	0.003879509	0.127685316
CHST2	324.2799543	-0.485404515	0.168107424	-2.88746626	0.003883583	0.127685316
STING1	35.94941389	-1.18817952	0.411606466	-2.886688176	0.003893199	0.127765745
ZFP36L1	2903.222373	-0.478274598	0.165794948	-2.884735662	0.003917424	0.128311797
TNRC6C	473.4428793	-0.334429314	0.115952657	-2.884188447	0.003924238	0.128311797
TTF2	1061.544313	0.400756875	0.139048654	2.882134157	0.003949915	0.128914815
TATDN2	1230.888345	0.29303141	0.101750839	2.879891846	0.003978116	0.12959786
PITPNA	715.0358586	-0.358733767	0.124717571	-2.876369096	0.00402279	0.130814088
GCLC	1021.90463	-0.342031599	0.118938409	-2.875703503	0.004031282	0.130851445
ATP5F1D	616.8417829	-0.444838025	0.154754478	-2.87447595	0.004046986	0.131122343
LOC101927420	7.107195076	-2.399571373	0.835397238	-2.872371685	0.004074035	0.131759168
MXD4	1051.054406	-0.360878576	0.125699682	-2.870958536	0.004092292	0.132109861
ABR	995.7804362	-0.453345922	0.157959664	-2.870010682	0.004104579	0.132266914
MFSD2A	81.01786018	0.671988262	0.234315244	2.867881117	0.004132308	0.132701108
DIP2B	1244.785976	0.333419309	0.116277081	2.867455109	0.004137875	0.132701108
RFC2	419.5839994	-0.485815407	0.169442811	-2.867134961	0.004142063	0.132701108
RGS1	57.56034586	-1.139877154	0.397627795	-2.866693849	0.00414784	0.132701108
ALDH1B1	2483.213167	0.507113744	0.176981604	2.865347204	0.004165522	0.133027974
TMTC1	127.1573666	0.633830624	0.2212851	2.864316781	0.004179098	0.13322278
C15orf39	927.5311442	-0.402427682	0.140604761	-2.862119874	0.004208177	0.133910208
RALGPS2	704.6561652	0.555655204	0.194203178	2.861205514	0.004220334	0.134057661
SLITRK5	627.9534943	0.422967521	0.147879769	2.860212211	0.004233576	0.134239014
TUBB1	16.25907835	1.794985018	0.627961839	2.858430093	0.004257429	0.13475557
GMIP	257.1053232	0.544089904	0.190427356	2.857204536	0.004273903	0.135016794
SNORA33	29.11257806	1.184682608	0.41473468	2.85648311	0.004283628	0.135016794
SMG5	2832.32444	0.280817944	0.098327422	2.855947377	0.004290862	0.135016794
OTUD5	540.8717952	-0.396953776	0.139026495	-2.855238325	0.004300454	0.135016794
RAB3GAP1	1157.497061	-0.358211103	0.125467607	-2.855008656	0.004303566	0.135016794
SLC29A2	896.7329669	0.596134741	0.208990734	2.852445791	0.004338421	0.135871115
MON2	2723.610208	0.280074287	0.09830242	2.849108766	0.004384189	0.137063592
CAMKK1	364.5410064	0.533740043	0.187387967	2.848315462	0.004395133	0.137102301
PLXNB2	1985.249277	-0.388033205	0.136252189	-2.847904373	0.004400815	0.137102301
WNT2B	122.5417225	0.796129341	0.279736188	2.84600054	0.004427212	0.137363331
STC1	20.11290698	-2.230111198	0.783789575	-2.845293265	0.004437055	0.137363331
RUSC2	363.2147374	0.425042784	0.149396442	2.845066304	0.004440218	0.137363331
HYAL2	338.3183274	0.485923837	0.170831572	2.844461542	0.004448656	0.137363331
TSPO	1129.3536	-0.371159252	0.130493044	-2.844283801	0.004451138	0.137363331
FEZ1	18.97266973	-1.563688162	0.549898441	-2.843594463	0.004460778	0.137363331
MSRB3	1154.911701	-0.330994531	0.116406987	-2.843424943	0.004463152	0.137363331
RSPO2	866.70405	0.611309894	0.215056855	2.842550141	0.004475419	0.137503393
TNKS1BP1	1282.049191	-0.452958409	0.159420125	-2.841287501	0.004493179	0.137811431
SMG6	433.1983582	-0.474829095	0.167353809	-2.837276888	0.004550013	0.139314845
NPLOC4	3050.592032	0.263936797	0.093047709	2.83657493	0.004560028	0.139381979
SLC19A2	386.7681124	0.389260439	0.137425415	2.832521481	0.004618246	0.140919776
ANTXR2	370.309266	-0.531836526	0.187806287	-2.831835586	0.004628164	0.140980994
GLT8D2	746.2038255	-0.375693097	0.132832128	-2.828330038	0.004679154	0.142158162
BTG1	2867.681428	-0.562623433	0.198941612	-2.828083215	0.004682763	0.142158162
KLHL3	225.986074	0.737051177	0.260719614	2.82698784	0.004698811	0.142251893
PLPP1	206.7886559	0.469965492	0.166254528	2.826783106	0.004701816	0.142251893
STMN1	1551.331697	-0.382040361	0.13518562	-2.826042903	0.004712695	0.142339369
PAPPA2	1032.485776	-0.598271356	0.21176001	-2.825232937	0.004724626	0.142458251
MCM6	1285.001588	-0.395266745	0.140034283	-2.82264269	0.004762962	0.14332746
AGA	253.7956066	-0.54670316	0.193715228	-2.822200223	0.004769539	0.14332746
IGFBP7	630.3741592	-0.595426521	0.211021654	-2.821637065	0.004777922	0.143337646
ALG5	301.3700155	-0.402223297	0.142618092	-2.820282409	0.00479814	0.143702282
EMX2	32.8381595	-2.291652693	0.814076472	-2.815033689	0.004877212	0.145729187
NFIX	5559.169494	-0.328544299	0.116732155	-2.814514139	0.004885103	0.145729187
ATG3	318.4460553	-0.383706916	0.136348213	-2.814169012	0.004890351	0.145729187
MINAR1	210.8732251	0.677461369	0.241066145	2.810271711	0.004949969	0.147132549
COL2A1	81.89778464	1.546754725	0.550444077	2.810012481	0.004953958	0.147132549
ACSL3	2590.22767	0.495178393	0.176414205	2.806907717	0.005001957	0.147977335
LINC00467	25.45179788	-1.128315848	0.402040349	-2.806474156	0.005008693	0.147977335
INTS4P2	23.97314568	1.950266997	0.694930255	2.806421196	0.005009516	0.147977335
ING4	206.1597317	-0.62717695	0.223534105	-2.805732702	0.005020232	0.147977335
BUB1B	1499.48113	0.30146172	0.10745585	2.805447267	0.00502468	0.147977335
TUBB2B	505.4954533	0.346176597	0.123415717	2.804963624	0.005032226	0.147977335
SLC15A2	14.20261274	1.507500778	0.537955966	2.802275415	0.005074353	0.14884843
AAK1	1768.505216	0.327104434	0.116739276	2.802008424	0.005078555	0.14884843
EPHA6	89.62256474	0.827329587	0.295362892	2.801061369	0.005093483	0.149040838
INTU	384.8817459	0.458600049	0.163851205	2.798881158	0.005128001	0.149751604
PXMP2	306.5614127	-0.530573159	0.189594214	-2.798467048	0.005134581	0.149751604
KDM7A	957.2981727	-0.77336193	0.276666047	-2.795290345	0.005185312	0.150774365
SMTN	847.5359713	0.456532212	0.163326512	2.795211914	0.00518657	0.150774365
SSR3	6018.594519	0.258176611	0.09241294	2.793727926	0.00521043	0.151221263
NABP1	1308.298565	0.529882194	0.189828537	2.791372692	0.0052485	0.152078488
GNG2	463.8080589	-0.542848136	0.194547773	-2.790307633	0.005265798	0.152119132
NXPE3	1451.923155	0.291047824	0.104309421	2.790235254	0.005266976	0.152119132
LOC101928034	6.614049598	-2.12408606	0.761537282	-2.789208237	0.005283708	0.152355464
S100A13	312.7046418	-0.483140012	0.173310651	-2.787711017	0.005308187	0.152814047
BACH2	155.185147	-0.730807411	0.262275753	-2.786408587	0.005329565	0.153182006
TMTC3	1788.770745	0.24148207	0.086681292	2.785861469	0.005338568	0.153193694
PCBP3	10.25927375	-1.772074887	0.636322581	-2.784868775	0.005354939	0.153416419
PRR13	423.4685657	0.432257467	0.15532499	2.782922861	0.005387161	0.153978077
C12orf75	1023.061489	0.451332418	0.162195641	2.782642094	0.005391825	0.153978077
ZNF658	125.5504394	-0.581418188	0.209229288	-2.778856597	0.00545506	0.155453738
DNTTIP1	163.9806751	-0.488181119	0.175699121	-2.778506325	0.005460945	0.155453738
LINC00703	167.2881805	-0.66501495	0.23953798	-2.776240113	0.005499158	0.156291857
LOC441155	11.82787383	-1.648013661	0.594026322	-2.774310835	0.00553188	0.1567308

SRGAP1	441.3884363	0.392746238	0.141566273	2.77429242	0.005532193	0.1567308
CA9	101.4493269	-0.688765735	0.248494522	-2.771754205	0.005575512	0.157707338
PLAC1	58.57106021	-1.217776342	0.439639262	-2.769944467	0.005606585	0.158334939
ZBTB37	969.9493214	0.497205172	0.179540527	2.769320001	0.005617343	0.158387751
GCN1	10185.01896	0.235370772	0.085039527	2.767780829	0.00564394	0.158886264
PSME2	527.9375951	-0.42175501	0.15254757	-2.764744194	0.005696745	0.160119868
ACSF3	327.8733386	-0.345608832	0.125095713	-2.762755205	0.005731574	0.160845102
IFFO2	382.8522564	0.662051699	0.240272342	2.755422005	0.005861648	0.164236735
TMEM200B	94.64697367	-0.636544755	0.231103505	-2.75437084	0.00588051	0.164393115
IL6R	97.3766316	0.821948414	0.298459623	2.753968545	0.005887743	0.164393115
RWDD2B	355.8201519	-0.330583769	0.120056397	-2.753570646	0.005894905	0.164393115
SLC7A3	12.59941621	-1.500195478	0.545046974	-2.752415019	0.00591575	0.164716658
PABPC1	40165.33704	-0.366840426	0.133313841	-2.751705479	0.005928581	0.164816412
CARMN	128.7453564	0.931276204	0.338517822	2.7510404	0.005940632	0.164894168
DUSP6	32.67403046	-1.478456174	0.537951849	-2.748305777	0.005990411	0.166017311
COL21A1	57.5025247	-0.816953267	0.297365135	-2.747306829	0.006008689	0.166085596
FAS	1380.320272	-0.573145684	0.208698551	-2.746284921	0.006027439	0.166085596
EXOSC5	339.8145216	-0.465979921	0.169684535	-2.74615434	0.006029839	0.166085596
YWHAB	2774.140031	-0.366587072	0.133491905	-2.746137088	0.006030156	0.166085596
H3C10	902.3642591	-0.599150283	0.218261395	-2.745104247	0.006049168	0.166352116
HOMER1	389.132069	0.327778624	0.119449402	2.744079232	0.006068089	0.166590116
CCNG2	277.0839203	-0.452505404	0.164929849	-2.74362346	0.006076519	0.166590116
ERCC2	1185.753222	0.325343063	0.118627633	2.742557151	0.006096284	0.16687524
ACADSB	107.309777	-0.644910861	0.235202978	-2.741933232	0.006107876	0.166936108
FBXO11	1852.738276	-0.344529749	0.125794666	-2.738826375	0.006165893	0.168263717
ZBED3	250.3279599	-0.47403736	0.173131383	-2.73802098	0.006181013	0.168418435
COPS2	2240.408456	-0.349047881	0.127535062	-2.736877806	0.006202533	0.168746769
CROT	157.8176559	-0.489837049	0.179025605	-2.736128433	0.006216676	0.168873722
NCR3LG1	501.7124265	0.441095634	0.161324124	2.734219922	0.006252827	0.169597214
PDIA3P1	163.6084437	-1.088888352	0.398353004	-2.733475937	0.00626697	0.169618247
OGFRL1	564.1404282	-0.442027423	0.16172653	-2.73317818	0.006272639	0.169618247
BBS1	229.8724565	-0.476707417	0.174748085	-2.727969328	0.006372553	0.171801146
DLX6-AS1	63.76910486	0.935351346	0.342875188	2.727964514	0.006372646	0.171801146
NTRK1	61.36608569	-1.378073735	0.505637405	-2.725418893	0.006421995	0.172870024
TTC30A	210.5284268	-0.432096443	0.158644265	-2.723681463	0.006455874	0.173497415
GOLIM4	3624.967073	-0.291781778	0.10714562	-2.723226361	0.006464775	0.173497415
LSP1P5	149.3684991	0.530272423	0.194797837	2.722167917	0.006485518	0.173792375
TTLL13P	4.801068163	3.045299969	1.119899217	2.719262521	0.006542766	0.174491397
AMBRA1	667.00833	-0.311279735	0.114481926	-2.719029509	0.006547377	0.174491397
PDRG1	227.6679879	-0.466950475	0.171758225	-2.71864987	0.006554895	0.174491397
HS3ST3B1	557.5689754	0.419486929	0.154367592	2.717454641	0.006578617	0.174491397
CC2D2A	425.8029748	-0.305701548	0.11254757	-2.716198554	0.00660363	0.174491397
ZNF600	114.9153403	0.5248832	0.193247703	2.716116118	0.006605275	0.174491397
MSRB1	371.2319459	0.367970633	0.135480147	2.716048374	0.006606626	0.174491397
ZYG11A	395.3208806	0.49653762	0.182817795	2.716024559	0.006607102	0.174491397
FAT4	1859.251408	0.310313451	0.11425493	2.715974289	0.006608105	0.174491397
ZCCHC4	281.4718699	0.528813593	0.194710025	2.71590327	0.006609523	0.174491397
THOC2	1963.249919	0.275126505	0.101344491	2.714765281	0.006632276	0.17483307
LINC02208	8.427232713	-2.210368051	0.814772494	-2.712865331	0.006670421	0.175445728
COL11A2	32.56060258	1.462548263	0.539162939	2.71262759	0.006675208	0.175445728
SENP8	62.73869125	-0.664587001	0.245187963	-2.710520508	0.006717769	0.176304346
PCYT1A	1651.525926	-0.233042983	0.086038343	-2.708594494	0.006756887	0.176805984
MIR7-1	26.98494491	1.056430318	0.390075936	2.708268362	0.006763531	0.176805984
SLC25A19	169.2729121	0.645156657	0.238263938	2.707739421	0.006774319	0.176805984
ATF3	65.37350375	-0.972682176	0.359237597	-2.707629116	0.006776571	0.176805984
ZEB2	1328.407794	-0.466013084	0.172374169	-2.703497202	0.006861403	0.178757593
DDI2	1181.41493	0.268860325	0.099474749	2.702799728	0.006875816	0.178871596
HSPA1B	796.0142811	0.725871996	0.268751876	2.700900204	0.006915208	0.179415445
GREM1	213.8684929	0.807847311	0.29911177	2.700820872	0.006916858	0.179415445
LINC00972	57.72840026	-0.804137965	0.297918934	-2.699183812	0.006950977	0.179829886
VIM	51302.93921	-0.547781744	0.202980816	-2.698687265	0.006961356	0.179829886
TADA2A	373.0784532	-0.332552536	0.123231348	-2.698603411	0.00696311	0.179829886
RGS2	753.5811576	-1.015489161	0.376694905	-2.695786819	0.007022259	0.181095022
GARNL3	195.1142414	0.61415878	0.227980383	2.693910639	0.00706191	0.18150009
SH3BP1	132.8778792	-0.630844224	0.234204566	-2.693560743	0.007069326	0.18150009
PDE3A	314.6681304	-0.566570949	0.210406673	-2.692742303	0.007086702	0.18150009
MRPL33	303.1605649	0.573321428	0.212918958	2.692674398	0.007088145	0.18150009
UAP1	1910.028286	0.302872286	0.112481568	2.692639259	0.007088892	0.18150009
PRKAR1B	131.5027114	-0.675994028	0.251107333	-2.692052117	0.007101385	0.181559075
ARIH1	2224.557971	-0.311679079	0.115825332	-2.690940523	0.00712509	0.181749721
SUPT5H	1515.230024	-0.299826446	0.111428733	-2.690746247	0.00712924	0.181749721
MROCKI	13.65407938	1.998591462	0.743195492	2.689186739	0.007162634	0.182218152
GYPC	27.45586306	-1.331354271	0.495187961	-2.688583684	0.007175585	0.182218152
VSIR	59.91672311	1.589839363	0.591357381	2.6884578	0.007178291	0.182218152
TUB	624.861844	0.573626553	0.213533289	2.686356562	0.007223596	0.182988766
C1QTNF7	108.315581	-0.774629116	0.288384521	-2.68609811	0.007229186	0.182988766
HORMAD1	192.5558656	0.487290594	0.181513069	2.684603358	0.007261593	0.183381776
BBOX1	18.71485903	1.54590232	0.575876657	2.684433031	0.007265294	0.183381776
UBE2D3	2251.362429	-0.406187422	0.15139632	-2.682941184	0.007297782	0.183673469
ZNF708	129.5064533	0.625460828	0.23313199	2.682861446	0.007299523	0.183673469
AGPAT2	87.89627795	-0.625879027	0.233352274	-2.682120968	0.0073157	0.183673469
QPCTL	365.3050401	0.441255728	0.164629947	2.680288347	0.007355877	0.183673469
SUMO1	1023.92342	-0.3802531	0.141893458	-2.679849423	0.007365528	0.183673469
H4C9	595.735042	-0.816852242	0.304824103	-2.679749513	0.007367727	0.183673469
SLU7	500.9554032	-0.482252076	0.179966608	-2.679675304	0.00736936	0.183673469
SH3GL1P1	23.53172695	1.634477581	0.609981689	2.67955188	0.007372078	0.183673469
SNHG3	1068.005054	0.738230725	0.275540311	2.67921134	0.00737958	0.183673469
XBP1	1692.191847	-0.385208998	0.143798939	-2.678802772	0.00738859	0.183673469
GNAO1	34.0368875	-1.649028474	0.615601197	-2.678728505	0.007390229	0.183673469
TUBGCP4	775.6299486	0.35596423	0.132945605	2.677517846	0.00741699	0.184081844

ARG2	73.10174481	-0.721174199	0.269482847	-2.676141385	0.007447522	0.184582534
INTS8	1451.709691	0.305305316	0.11411833	2.675339849	0.007465353	0.18476749
SFRP2	577.1513427	-0.431142865	0.161244723	-2.673841714	0.007498784	0.185289862
YTHDF2	1567.231361	-0.321191298	0.120150307	-2.673245754	0.00751212	0.185289862
GPD2	1247.292956	0.428553318	0.160326792	2.672998768	0.007517653	0.185289862
FAM126B	605.747428	0.319189261	0.119471301	2.671681478	0.007547225	0.185761809
SEPTIN10	1254.968789	-0.294088474	0.110222425	-2.668136498	0.007627326	0.187474422
LMNB1	1755.555424	-0.396079159	0.148617054	-2.665098976	0.007696567	0.188634588
MSH6	2578.928218	-0.214287327	0.080434455	-2.664123579	0.00771892	0.188634588
NIBAN2	3421.597057	-0.344107539	0.129168121	-2.664028364	0.007721105	0.188634588
SLX4IP	287.4547765	-0.397047712	0.149045945	-2.663928311	0.007723402	0.188634588
ATP7B	491.4827212	0.333194888	0.125105819	2.663304484	0.007737736	0.188634588
FASTKD1	495.8719046	0.40443012	0.151854503	2.663273799	0.007738442	0.188634588
SLC35E2B	1077.545496	-0.251435268	0.094424034	-2.662831252	0.007748626	0.188634588
ECI1	485.8288604	-0.352696811	0.132561567	-2.660626446	0.007799544	0.189615097
C11orf58	1210.226555	-0.246973577	0.092861087	-2.659602483	0.007823293	0.189933342
SGSH	317.5426664	-0.357215062	0.134389219	-2.658063371	0.007859111	0.190543353
CREG1	544.071306	-0.314298006	0.118334505	-2.656013193	0.007907052	0.191445201
ADCY7	2937.695761	0.521505863	0.196448035	2.654675892	0.007938464	0.191924602
ADGRE5	284.6735974	-0.501686411	0.189055123	-2.653651505	0.007962602	0.191924602
ANPEP	613.4731463	-0.855724869	0.322483313	-2.653547753	0.00796505	0.191924602
SNRPA1	764.4936833	0.290139408	0.109348711	2.653340915	0.007969933	0.191924602
CCND2	7953.929828	-0.384176905	0.144906703	-2.651201752	0.008020592	0.192883861
C6orf163	17.78646688	1.613343457	0.608846048	2.649838103	0.008053035	0.193403078
TOP1	922.792555	-0.475646283	0.179612259	-2.648183844	0.00809255	0.193985478
SWAP70	1071.710054	-0.33135878	0.125139643	-2.647912145	0.008099057	0.193985478
EIF5AL1	54.90087781	-1.596959111	0.603584174	-2.645793547	0.008149954	0.194835581
MCM5	1653.480431	-0.324398365	0.122621528	-2.64552539	0.008156417	0.194835581
CLOCK	626.7737702	0.319604884	0.120884494	2.643886511	0.008196013	0.195519349
PTPRC	125.4331789	-0.575330302	0.217783786	-2.641749937	0.008247893	0.196493914
PTS	146.8806123	0.460259523	0.174374844	2.639483503	0.008303246	0.197548533
PLS1	162.5247051	0.501976659	0.19028518	2.638022887	0.008339095	0.198116658
KMT5B	437.8133099	-0.347679503	0.131816332	-2.637605668	0.008349361	0.198116658
ISLR	122.2306454	1.200196021	0.455223322	2.63649941	0.008376635	0.198499506
SOX4	2619.272674	-0.465936154	0.176782635	-2.635644358	0.00839777	0.198736063
COLGALT2	253.2390342	-0.516830298	0.196222381	-2.633900864	0.008441013	0.199494497
GRPEL2	992.5778192	-0.296131194	0.112452344	-2.633392806	0.008453652	0.199528571
SLC22A15	268.518407	-0.512938671	0.194914679	-2.631606172	0.008498231	0.200315444
IL20RB	16.84531316	-1.310352457	0.498148845	-2.630443633	0.008527351	0.200736319
POMK	1444.799171	0.26237835	0.099798653	2.629077079	0.008561695	0.201226146
ZNF678	420.6413065	0.334760119	0.127350363	2.62865461	0.008572338	0.201226146
TWIST1	4170.491253	-0.431011498	0.163990567	-2.628270058	0.008582035	0.201226146
HR	86.863478	-1.405091696	0.534734814	-2.627642074	0.008597893	0.201236549
ACTR10	751.9973081	-0.333866545	0.127073088	-2.627358407	0.008605065	0.201236549
RPL30	4180.7807	-0.39958391	0.152142927	-2.626371916	0.008630047	0.201556263
CDH10	1282.773007	0.303499942	0.115585397	2.62576373	0.008645481	0.201652439
PKM	21732.30581	-0.439334997	0.167385811	-2.624684824	0.008672921	0.202028036
MIR12136	239.6148212	1.123729962	0.428245272	2.624033553	0.008689522	0.202150509
TMCC2	111.5997189	0.630908944	0.240497851	2.623345451	0.008707094	0.202286139
CGREF1	248.9056445	-0.463494387	0.176737549	-2.622500924	0.008728703	0.202286139
LINC02709	14.06587464	-1.766651803	0.673658609	-2.622473429	0.008729407	0.202286139
HMGA2	960.2706392	-0.517679865	0.197447862	-2.621856012	0.008745237	0.202389771
LINC02454	5.37737427	-2.691150937	1.027661295	-2.618713919	0.008826193	0.203238344
LOC105374378	8.331536159	1.980604506	0.756328408	2.618709659	0.008826303	0.203238344
LINC01411	11.49203961	3.242362114	1.238368179	2.618253739	0.008838106	0.203238344
AREL1	1300.02078	0.263543198	0.100660517	2.618138737	0.008841085	0.203238344
HMGXB3	1822.167954	0.268287661	0.102499343	2.617457375	0.008858755	0.203238344
DDAH1	1537.818373	0.384781119	0.147021392	2.617177764	0.008866016	0.203238344
PLCB4	414.5717141	0.510284282	0.19500228	2.61681188	0.008875524	0.203238344
ZCCHC3	557.0811023	-0.314722344	0.120298841	-2.61617104	0.0088922	0.203238344
H2BC18	718.8054357	-0.63752445	0.243690765	-2.616120684	0.008893512	0.203238344
LARP6	425.8373347	-0.709071324	0.27104894	-2.616026917	0.008895954	0.203238344
ZNF512	301.1105861	-0.48290382	0.184665838	-2.615014364	0.00892237	0.203377789
ANGEL1	979.0417416	0.280485871	0.107266863	2.614841743	0.008926881	0.203377789
DLD	1547.450792	-0.333746151	0.127652896	-2.614481622	0.008936297	0.203377789
KNOP1	840.1965355	0.25629507	0.098058803	2.613687531	0.008957091	0.203417735
UBR7	1157.56185	-0.300352239	0.114925317	-2.613455814	0.008963167	0.203417735
ARHGAP19	436.6685327	0.366867072	0.14039492	2.613107888	0.008972297	0.203417735
SRRM2	8839.649887	0.450827364	0.172561511	2.612560363	0.008986682	0.203484974
NSA2	239.6704049	-0.415934694	0.159290528	-2.611170283	0.009023295	0.204054721
HAUS1	244.6935524	-0.368503189	0.141158938	-2.610555129	0.00903954	0.204162996
PRIM1	644.9733214	-0.362948767	0.139112564	-2.609029377	0.009079945	0.204815968
RFLNB	1071.468013	-0.304865055	0.116882282	-2.608308536	0.00909909	0.204988346
KATNBL1	598.6181702	0.324507611	0.124469775	2.607119768	0.009130742	0.205270874
TWISTNB	3541.277636	-0.283578423	0.108776913	-2.60697252	0.009134669	0.205270874
PLEKHA1	684.413524	0.755845284	0.290315825	2.603527674	0.009226982	0.207084151
CD109	4712.069088	-0.494587316	0.190142085	-2.601145961	0.009291291	0.208265171
ARSJ	450.7161989	-0.469243656	0.18059646	-2.598299305	0.009368679	0.20876276
AHR	989.283043	-0.949102497	0.36528722	-2.598236247	0.0093704	0.20876276
LINC02060	3.016209601	4.329943841	1.666523909	2.598188851	0.009371694	0.20876276
LINC00235	48.6055204	1.113350493	0.428511822	2.598179176	0.009371958	0.20876276
NLRC5	502.0070636	0.454030842	0.174767244	2.597917275	0.009379109	0.20876276
VLDLR-AS1	6.898830711	-2.252908187	0.867254904	-2.597746263	0.009383781	0.20876276
GRIN3A	100.232694	0.677173094	0.260733126	2.59718857	0.009399031	0.20884131
TMCO1	1191.829946	-0.333009679	0.12825172	-2.596531872	0.009417017	0.208914924
PAIP2	1100.544754	-0.337055941	0.129826051	-2.596211913	0.009425791	0.208914924
IMPAD1	7187.536185	0.24731017	0.095276363	2.595713809	0.009439465	0.208958102
PLEKHG1	280.6409209	-0.580711979	0.223762001	-2.5952216	0.009452995	0.208997979
PRRT2	95.00476461	0.815481574	0.314446591	2.593386598	0.009503587	0.20985617
INPPL1	1766.849591	0.296388077	0.114310695	2.592828926	0.009519011	0.209936598

RNF185	438.627509	-0.300936059	0.116094238	-2.592170494	0.009537249	0.209990389
CALM3	4737.889189	-0.279343994	0.10777616	-2.591890393	0.009545018	0.209990389
GTPBP2	1330.858211	-0.352271941	0.135999013	-2.590253654	0.009590524	0.210731358
SLC43A1	486.7186385	-0.340247156	0.131383968	-2.589715937	0.009605516	0.210800852
DDX28	235.205437	-0.450830849	0.174139202	-2.588910735	0.009628005	0.211034499
PMS2	819.9387203	-0.326565433	0.126162334	-2.588454282	0.009640774	0.21105479
PCDH7	98.57581724	-0.614380052	0.237442242	-2.587492633	0.009667726	0.211385132
EAPP	378.4315956	-0.44111773	0.170890157	-2.581293966	0.009843073	0.214835932
SSBP3	313.598922	-0.594639167	0.230387792	-2.581035919	0.009850433	0.214835932
HNRNPK	8630.327178	-0.3863108	0.149695695	-2.580640677	0.009861717	0.214835932
SAP30L	456.2952272	-0.407476239	0.158089421	-2.577504777	0.009951651	0.21653042
TP53	1004.796396	-0.40236192	0.156195351	-2.576017254	0.009994566	0.21719898
HOXB8	41.47706364	-1.221650308	0.474569175	-2.574230211	0.01004634	0.218058202
AHI1	592.661934	0.317957738	0.1236327	2.571793206	0.01011733	0.219322334
MARVELD1	999.6893587	-0.274502241	0.106758946	-2.571234089	0.01013368	0.219322334
CRNKL1	420.2933646	-0.32498679	0.12640645	-2.570966827	0.010141504	0.219322334
GSDMB	54.8238098	0.861360962	0.33514126	2.570142993	0.010165654	0.219578137
MAEA	652.0744792	-0.302315619	0.117672431	-2.569128704	0.010195458	0.219756311
MID1	1223.528825	0.570185091	0.221946273	2.569023051	0.010198567	0.219756311
MTMR2	969.0923557	0.325995261	0.126926481	2.568378621	0.010217549	0.219804388
KLF4	294.0127706	-0.85361426	0.332419505	-2.567882593	0.010232181	0.219804388
RND2	12.73570747	-1.369930952	0.533526164	-2.567692169	0.010237803	0.219804388
ATP2B4	3957.172664	0.305177766	0.118896803	2.566744922	0.01026581	0.220056079
SGCE	445.3779202	-0.321942748	0.1254423	-2.566460818	0.010274223	0.220056079
WNT9A	22.25672195	-1.104359604	0.430383632	-2.565988859	0.010288213	0.220091189
H2BC13	327.8027213	-0.468532378	0.182648044	-2.565219795	0.010311047	0.22020439
EIF3J	1350.734946	-0.292057713	0.11386361	-2.564978522	0.010318219	0.22020439
NUDT15	303.6016529	0.348664117	0.136001531	2.563677878	0.010356962	0.220699053
TNC	4007.305899	-0.4573958	0.178435379	-2.563369452	0.010366168	0.220699053
DEPDC7	86.50818215	0.619571859	0.241812803	2.56219626	0.010401252	0.221168251
LINC00472	87.61933145	1.108983553	0.432891769	2.561803278	0.010413028	0.221168251
PLEKHG3	1482.173703	0.697777074	0.272503012	2.560621515	0.010448512	0.221657712
RAMAC	167.1183497	-0.421913341	0.164852667	-2.559335851	0.010487237	0.222186673
SERINC4	91.66715953	1.172845617	0.458327833	2.558966603	0.010498383	0.222186673
THEM4	221.7282256	-0.538238096	0.210423488	-2.557880305	0.010531233	0.222266529
PPP3CC	299.7774809	0.427181358	0.167013744	2.557761696	0.010534826	0.222266529
SLC38A2	5060.714078	-0.491558737	0.192194942	-2.557604965	0.010539574	0.222266529
GBA2	665.5767477	0.381907306	0.149389007	2.556461902	0.010574265	0.222503673
MLXIP	2063.918391	0.460839434	0.180268085	2.556411666	0.010575792	0.222503673
H4C2	1652.991938	-0.461138666	0.180447007	-2.555535127	0.010602466	0.22257212
PAFAH2	152.7419299	-0.445916157	0.174493821	-2.555483945	0.010604025	0.22257212
CACNA2D4	35.19458495	-1.115178448	0.436468805	-2.555001495	0.010618734	0.222618639
SLC25A37	1823.402885	0.345228875	0.135161135	2.554202244	0.010643142	0.222868136
EPDR1	1862.905119	0.360127665	0.141064619	2.552926933	0.01068219	0.223423276
ZNF131	425.5619795	-0.327008957	0.12813232	-2.552119213	0.010706988	0.22367939
SMC1A	3913.918034	-0.219018588	0.085838121	-2.551530537	0.010725092	0.223795255
B3GNT10	55.75332847	-0.703199533	0.275788836	-2.5497752	0.01077924	0.224662053
GTF2E1	446.1398603	0.349987434	0.137493202	2.545489007	0.01091248	0.227173356
MEX3C	935.6973454	-0.397637481	0.156274707	-2.544477532	0.010944135	0.227283245
PNCK	5.332624893	3.322199926	1.305732183	2.544319554	0.010949087	0.227283245
VIM-AS1	55.128129	-0.887636734	0.348900316	-2.544098395	0.010956022	0.227283245
LOC107985911	284.0132524	0.428104936	0.16834184	2.54306913	0.010988349	0.227446206
ZDHHC3	514.8486787	-0.359795407	0.141482648	-2.543035567	0.010989404	0.227446206
ARHGAP28	1203.745686	-0.457816668	0.18008244	-2.542261571	0.011013772	0.22768609
LRRC1	577.6289964	0.570069008	0.224435024	2.540018038	0.011084675	0.22888634
COPS6	1004.391739	-0.385976271	0.152003514	-2.539258863	0.011108759	0.229081968
PODXL2	149.154302	-0.560109896	0.220610426	-2.538909454	0.01111986	0.229081968
BPHL	480.6595142	-0.388668152	0.153133033	-2.538107844	0.011145363	0.229153558
ZNF714	422.3231492	0.437123645	0.172232082	2.53799199	0.011149054	0.229153558
H2AC6	967.0823892	-0.489431755	0.192919037	-2.536980084	0.011181331	0.22955222
TCEAL4	116.9774554	-0.503837365	0.198769877	-2.534777269	0.011251884	0.230734841
VIPAS39	431.0007087	-0.320963552	0.126735408	-2.532548382	0.011323674	0.231524418
MCM7	2493.048243	-0.344780977	0.136146176	-2.532432331	0.011327423	0.231524418
RUNX1T1	718.8075641	-0.573158862	0.226332787	-2.532372219	0.011329365	0.231524418
DNHD1	859.5210131	0.79017143	0.312793565	2.526175469	0.011531187	0.235125081
CSDE1	10679.19729	-0.313645849	0.124159518	-2.526152277	0.011531948	0.235125081
ASCC1	387.3138086	-0.408231379	0.16171389	-2.524405166	0.011589427	0.235546901
TBC1D2B	1642.729061	-0.425437275	0.168533578	-2.52434725	0.011591337	0.235546901
HMGN3-AS1	38.62773955	1.077872286	0.427045761	2.524020571	0.011602114	0.235546901
ZBTB11	916.4603112	0.279636493	0.110794616	2.5239177	0.01160551	0.235546901
SNORD134	30.11079667	1.554900196	0.616671072	2.521441763	0.011687502	0.236941166
H2BC17	1389.948693	-0.462753003	0.183601879	-2.520415398	0.011721641	0.237283729
FAM107B	226.8682862	-0.442200079	0.175516974	-2.519414895	0.011755005	0.237283729
PNP	950.5425724	0.321334187	0.127610764	2.51808058	0.011799632	0.237283729
E2F2	187.1072087	-0.557369584	0.221348548	-2.518062984	0.011800222	0.237283729
PRRG1	364.6590524	0.359526301	0.14278294	2.517991991	0.0118026	0.237283729
TRIM25	1935.312378	-0.313518924	0.124523869	-2.517741593	0.011810994	0.237283729
JAGN1	362.2514289	-0.35898548	0.142586138	-2.517674475	0.011813244	0.237283729
C6orf120	890.0649857	-0.266608396	0.105921156	-2.517045747	0.011834346	0.237283729
TPRG1	207.9066016	-0.857692487	0.340766315	-2.516952084	0.011837493	0.237283729
HOXC13	29.26384912	-0.97219574	0.386301763	-2.516674357	0.011846827	0.237283729
MAST2	1779.432068	0.456551119	0.181441198	2.516248371	0.011861156	0.237283729
H2BC15	790.8732975	-0.568857545	0.226088865	-2.516079443	0.011866843	0.237283729
TIMP3	2611.288017	-0.779423261	0.309851046	-2.515477262	0.011887134	0.237283729
UFSP2	241.8878338	-0.414322262	0.164733676	-2.515103606	0.011899741	0.237283729
ACVR1C	10.93239995	1.467416952	0.583472133	2.514973497	0.011904133	0.237283729
ARHGEF19	195.507321	0.804297492	0.319882539	2.514352597	0.011925114	0.237436351
C11orf21	29.31419881	-1.122179327	0.446674263	-2.512299049	0.01199474	0.238556094
LOC105376114	14.94060154	-1.229282125	0.489503653	-2.511282841	0.012029327	0.23897727
H2AC13	374.6751008	-0.563819038	0.224665595	-2.509592254	0.012087064	0.239405776

ARHGAP18	172.4943227	-0.465834048	0.185623518	-2.509563734	0.01208804	0.239405776
SLC35A3	535.1639867	0.398645179	0.158856235	2.509471395	0.012091201	0.239405776
MCCC1	757.2661898	-0.242618005	0.096701524	-2.508936731	0.012109517	0.239502326
MAP2K3	2113.167195	0.302026063	0.120447109	2.507540982	0.012157448	0.240041679
ZNF598	1800.114388	-0.40628338	0.162036416	-2.507358472	0.012163728	0.240041679
H4C12	52.09214788	-0.952994625	0.380361828	-2.50549491	0.012228016	0.241043409
TANC2	2275.786691	0.390700353	0.156012862	2.50428297	0.012269986	0.241603476
CDC42	4332.800155	-0.301279023	0.120348268	-2.503393091	0.012300884	0.241888883
AGBL3	85.2285291	0.69126026	0.276163417	2.503084105	0.012311628	0.241888883
DNAJC12	103.924914	-0.584604088	0.233663128	-2.501909874	0.012352537	0.242425333
IER2	564.7527046	-0.436523854	0.174554039	-2.500794924	0.012391491	0.24276541
HPSE	225.6380845	0.425151	0.17003613	2.500356839	0.012406827	0.24276541
H2BC7	808.0302366	-0.478394122	0.191338876	-2.500245283	0.012410735	0.24276541
SDC4	182.0220817	-0.505177656	0.202097824	-2.49966895	0.012430941	0.242894043
ATP5PD	443.5937583	-0.377150139	0.150944021	-2.498609334	0.012468168	0.243354595
HSPA6	16.11289076	1.208501089	0.483993579	2.4969362	0.012527149	0.243727235
ISM1	38.21376439	-0.993330236	0.397844917	-2.496777498	0.012532757	0.243727235
GATA6	217.7265523	-0.44144161	0.176821301	-2.496540903	0.01254112	0.243727235
PDZD4	4.122488969	-2.932640482	1.174705149	-2.496490701	0.012542896	0.243727235
BCL7C	211.7927271	-0.432229424	0.173159797	-2.496130348	0.012555645	0.243727235
SPACA6	134.6556308	0.855429943	0.34279452	2.495459798	0.012579401	0.243922659
URM1	576.4322945	-0.318974286	0.127859101	-2.494732747	0.012605203	0.244157299
SPATA5	645.5866592	0.374929972	0.150369538	2.493390473	0.012652961	0.244341086
HDGFL2	407.8358795	-0.360475458	0.14457439	-2.493356239	0.012654182	0.244341086
NANP	471.679797	0.325044561	0.13036668	2.4933101	0.012655826	0.244341086
SMARCA1	717.1137106	-0.410051668	0.164492445	-2.492829796	0.012672958	0.244407045
BNC2	1171.767133	-0.251966344	0.101116488	-2.49184231	0.012708244	0.244822612
POM121C	559.6510454	0.2626693	0.105454244	2.49083669	0.012744268	0.245063569
RPS19	7915.769265	-0.413492055	0.166012695	-2.490725516	0.012748256	0.245063569
NUDT3	3553.294397	-0.292451864	0.117466752	-2.489656518	0.01278666	0.245536948
NEDD4	1327.593874	-0.39622452	0.159359673	-2.486353756	0.01290596	0.247561046
ZMYM4	1701.64178	-0.354019062	0.142427831	-2.485603135	0.012933211	0.247817003
UBE2S	813.8456019	0.384429268	0.154689124	2.485173214	0.012948841	0.24785
ANKFN1	31.10358819	-1.061313782	0.427387317	-2.48325989	0.013018607	0.248917997
EEF1B2	1625.144344	-0.423656168	0.170641191	-2.482730961	0.013037952	0.249020685
SCD	26100.65165	0.73390977	0.295758237	2.481451669	0.013084845	0.249648763
WNK3	313.241886	0.475265152	0.191576103	2.480816469	0.013108185	0.249826577
ANXA6	4447.617996	-0.249140654	0.100468583	-2.479786683	0.0131461	0.250196768
MORN4	46.21499365	-0.98019233	0.39531429	-2.479526683	0.013155689	0.250196768
FLJ22447	10.52123629	-1.912688633	0.771877808	-2.477968163	0.013213293	0.251024403
HSPA1L	7.847707405	-2.019596368	0.8151778	-2.477491864	0.013230943	0.251092008
WSB1	1857.747137	0.467442296	0.188714942	2.476975547	0.013250098	0.251188027
UVSSA	447.322862	0.495862873	0.200286845	2.47576357	0.013295159	0.251774421
HNRNPLL	634.9231016	-0.376015704	0.151961274	-2.474417959	0.013345347	0.252334565
H2AZ2	802.8494161	-0.259523723	0.104891482	-2.474211605	0.013353058	0.252334565
KIF21A	463.2312317	0.377039397	0.152573875	2.471192374	0.013466336	0.254205614
GTF2H3	1643.606542	-0.322969676	0.130720118	-2.470696024	0.013485039	0.254289307
MZT1	586.8028054	0.353437481	0.143115634	2.469593785	0.013526656	0.25475359
ELMOD1	45.22241075	0.768096394	0.311059968	2.469287184	0.013538252	0.25475359
SOBP	182.3959823	-0.469543559	0.190296678	-2.467429087	0.013608717	0.255607551
ZZEF1	1724.649473	0.227731352	0.09229854	2.467334282	0.013612321	0.255607551
ETF1	3277.688425	-0.462306602	0.187490868	-2.465755296	0.01367247	0.256002572
LINC01583	123.9485451	0.905471728	0.367229185	2.465685638	0.013675129	0.256002572
CLDN11	43.70536461	-0.919782054	0.373038235	-2.465650882	0.013676456	0.256002572
XPOT	5834.077569	-0.318349793	0.129228283	-2.463468401	0.013760002	0.257296162
UNKL	312.0813979	0.541380544	0.219900847	2.461930239	0.013819154	0.257942884
TMSB4X	4946.897322	-0.566941298	0.230293893	-2.461816468	0.013823538	0.257942884
THY1	1497.685992	-0.425932596	0.173132521	-2.4601536	0.013887756	0.258870105
APLP2	2542.540994	-0.363048643	0.147643052	-2.45896192	0.01393394	0.259459566
KLF9	818.3367305	-0.662416563	0.269511794	-2.457838867	0.013977587	0.259643115
HSDL2	748.9684596	-0.32328134	0.131537806	-2.457706654	0.013982734	0.259643115
CHRNG	144.7081041	0.791998977	0.322298337	2.45734739	0.013996727	0.259643115
PIM2	87.2324752	0.563914079	0.229507588	2.457060715	0.014007901	0.259643115
DCTN1	3997.328555	-0.247147413	0.100595794	-2.456836443	0.014016649	0.259643115
OSR1	12.81128841	-2.106094537	0.857595907	-2.455812253	0.014056657	0.260113847
ZIC5	311.6222704	0.499567968	0.203581989	2.453890793	0.014131989	0.261236564
MIR548AR	10.47552738	1.932167697	0.787580516	2.453295452	0.014155402	0.2613982
C1GALT1	469.6681289	0.31932984	0.130189844	2.452801457	0.014174855	0.261486459
PDIA3	7572.134361	-0.19011075	0.077526112	-2.452215718	0.014197952	0.261641676
AGTPBP1	458.4371484	-0.290278511	0.118420122	-2.451260022	0.014235708	0.26202418
XRCC1	279.2520772	-0.432251286	0.176360969	-2.450946416	0.014248116	0.26202418
OS9	2603.911106	-0.39305863	0.160524498	-2.448589685	0.014341672	0.263284541
PTMS	1516.114647	-0.290688054	0.118732097	-2.448268505	0.014354464	0.263284541
DLX5	252.9905379	0.770875531	0.314886614	2.448105121	0.014360975	0.263284541
DAXX	650.6037816	-0.326853749	0.133533432	-2.447729717	0.014375945	0.263288122
GRB10	734.4685733	-0.401389336	0.164033545	-2.446995439	0.014405266	0.263554254
KLHL20	385.090909	-0.328416823	0.134262967	-2.446071535	0.014442234	0.263702818
LOC344967	1.831612111	4.143606484	1.693997118	2.446052853	0.014442983	0.263702818
SLC25A32	3070.287509	0.287542115	0.11758114	2.44547821	0.014466019	0.263839703
OLFML3	634.3179528	0.403385669	0.164975306	2.445127569	0.014480091	0.263839703
PRKAB1	418.9849792	-0.3599842	0.147273196	-2.444329382	0.01451217	0.264154114
UNG	857.1885049	-0.316245975	0.12940053	-2.443931071	0.014528202	0.26417608
LTBP3	132.6056732	-0.704600922	0.288431734	-2.44286893	0.014571028	0.264684731
ABCC1	1948.828587	0.258449123	0.105814324	2.442477667	0.014586832	0.264701984
MYO1B	3108.869309	-0.306516304	0.125523626	-2.441901289	0.014610141	0.264855249
GRID2	21.32063636	1.093140153	0.447878396	2.440707481	0.014658523	0.265462274
WDR48	538.9262851	-0.393710044	0.161374054	-2.439735726	0.01469801	0.265907144
AGAP5	17.67962828	1.518579876	0.622737375	2.438555861	0.014746079	0.266506222
FGFR4	179.5671559	0.390631874	0.160388522	2.435535096	0.014869782	0.268469621
PPFIA4	145.1079546	0.867737327	0.356424392	2.434562134	0.01490982	0.268920029

LINC01050	49.5485124	-1.24275464	0.510621003	-2.433810269	0.014940824	0.269206765
ZFPM2-AS1	91.47306651	-0.821885834	0.337764523	-2.433310125	0.01496148	0.269306643
FUT1	3.280690437	-3.254494203	1.33838564	-2.431656546	0.015029952	0.27021559
CFAP54	24.80509861	-0.899123901	0.369803029	-2.431358938	0.015042304	0.27021559
TMEM135	590.9519379	0.447235432	0.184145086	2.428712282	0.015152552	0.271921925
TOP3B	356.0373286	0.535879126	0.22071136	2.427963498	0.015183871	0.272209847
MAP1LC3B	769.2339847	-0.405941092	0.167242952	-2.427253808	0.015213608	0.272468847
ST3GAL5-AS1	3.228268641	3.641082094	1.501102061	2.425605953	0.015282854	0.273275675
FHAD1-AS1	7.69589966	-1.737949358	0.716546532	-2.425452194	0.015289329	0.273275675
CSNK1A1	3845.750231	-0.316456692	0.130563677	-2.423772827	0.015360211	0.27402334
ELF4	1033.149704	0.440294268	0.18169649	2.42324036	0.015382745	0.27402334
CMTM3	665.6158481	-0.48572461	0.20044566	-2.423223382	0.015383464	0.27402334
ACAA2	1056.522198	-0.273123524	0.11272094	-2.423006078	0.015392669	0.27402334
AMN1	27.51905004	1.050430484	0.434414742	2.418035998	0.015604533	0.277517743
CBWD5	109.8684455	0.550175319	0.227740114	2.415803304	0.01570054	0.278946787
METAP2	2474.396408	-0.256665677	0.106292803	-2.414704187	0.015747993	0.279511199
LOC100289511	52.34117317	0.73745901	0.30553103	2.413695954	0.015791634	0.279977392
PAQR3	587.7037577	0.28947938	0.119948102	2.413371914	0.015805682	0.279977392
LOC729652	61.55261547	1.008963684	0.418158592	2.412873256	0.015827322	0.280082308
YPEL3	29.29495427	-1.644460862	0.681877823	-2.411664972	0.015879866	0.280567765
SMG1P7	100.691	0.739812109	0.306815021	2.411264311	0.015897323	0.280567765
SUPT16H	4952.450591	-0.239513896	0.099335658	-2.411157288	0.015901989	0.280567765
AP3S1	976.1445437	-0.433903053	0.180005991	-2.410492289	0.015931008	0.28066555
HECTD4	2035.306727	0.316661063	0.131377808	2.410308619	0.015939031	0.28066555
UNC119B	1618.253117	-0.283240485	0.117552211	-2.409486661	0.01597498	0.281020873
DPAGT1	438.2857028	0.303065817	0.125942427	2.406383801	0.016111329	0.282805412
NR4A1	140.801387	-0.399771333	0.166143758	-2.406177264	0.016120441	0.282805412
TMEM181	1821.583132	0.205895042	0.085572254	2.406095826	0.016124035	0.282805412
EIF3G	1258.253176	-0.305365784	0.126933445	-2.40571572	0.016140819	0.282821437
LENG8-AS1	98.4518624	0.653843805	0.271921205	2.404534078	0.016193096	0.28345872
LOC105372273	4.421173527	2.934774845	1.22156722	2.402466927	0.016284907	0.284563716
ALDH9A1	816.4373575	-0.422689171	0.175955141	-2.402255303	0.016294332	0.284563716
BTBD18	19.90363368	1.330242432	0.553816153	2.401956721	0.016307638	0.284563716
NHLRC2	895.6152036	0.29338153	0.12215693	2.401677341	0.016320096	0.284563716
EMP3	401.1900936	-0.411020532	0.171192184	-2.400930486	0.016353443	0.284593652
BMP6	370.4714704	0.662438104	0.27592399	2.400799239	0.016359309	0.284593652
STK17B	1585.138208	-0.439693762	0.183162515	-2.400566309	0.016369725	0.284593652
LRP8	1131.990132	0.573400567	0.238901019	2.400159572	0.016387926	0.284632401
LOC101929829	2.64878008	3.786355098	1.578249935	2.39908459	0.016436117	0.28507583
GART	2272.067023	-0.321819764	0.134154359	-2.398876686	0.016445452	0.28507583
MAP3K10	370.9131027	0.430173517	0.179464359	2.39698579	0.016530565	0.286272755
ABCC5	2685.293886	0.390351546	0.162924863	2.395899185	0.01657965	0.286619318
PARN	780.7367589	-0.338583383	0.141321912	-2.39583076	0.016582745	0.286619318
NUDT21	2283.356159	-0.281594672	0.117583548	-2.394847544	0.016627277	0.287110543
DENND4B	1919.763436	0.371066537	0.155114603	2.39220892	0.016747306	0.288698201
H2AC21	561.0355333	-0.461458995	0.192952561	-2.391567091	0.016776617	0.288698201
PMPCB	1304.64683	-0.254299137	0.106332258	-2.391552117	0.016777302	0.288698201
EFNB3	69.54779164	-0.969582156	0.405444558	-2.39140503	0.016784026	0.288698201
DNMT3A	403.9427433	-0.335646512	0.140414181	-2.390403234	0.016829883	0.289207833
FAP	483.6542261	0.316237513	0.132482535	2.387012836	0.016985898	0.291607616
PRKX	801.8872549	0.265524797	0.111254176	2.386650157	0.017002662	0.291614479
SLC26A5	12.02210819	1.719343088	0.72135319	2.383496896	0.017149029	0.293842018
SPACA9	32.8227268	-0.846475941	0.355257937	-2.382708037	0.017185818	0.294189514
SOGA1	2220.677994	0.303900945	0.127574299	2.382148648	0.017211948	0.294354045
SLFN11	1268.507378	0.216772397	0.091072036	2.38023005	0.017301833	0.2952158
BCAS2	363.5750697	-0.382141512	0.160554803	-2.380131302	0.01730647	0.2952158
PIAS1	743.6214975	0.281709226	0.118364585	2.380012779	0.017312038	0.2952158
ETS2	929.7543801	0.453206197	0.190486386	2.37920518	0.017350016	0.295580572
DCTN2	1074.441383	-0.345560315	0.145278826	-2.378600691	0.01737849	0.295782894
PTENP1	54.4868848	-1.569715184	0.660348455	-2.377101319	0.017449294	0.296704606
MTURN	366.7930654	-0.656130021	0.276073911	-2.376646239	0.017470835	0.296787676
CHST6	233.5443848	0.475345116	0.200037443	2.376280704	0.017488153	0.296798943
OGFR	331.4455256	-0.408203134	0.171820194	-2.375757622	0.017512962	0.296853664
SPSB2	79.96001274	0.550334338	0.231669926	2.375510487	0.017524694	0.296853664
SMARCA5	1469.553928	-0.316068708	0.133096518	-2.374733113	0.017561643	0.297197036
H2AC4	358.9226143	-0.512365399	0.215977838	-2.372305439	0.01767747	0.298868163
PEBP1	2513.78089	-0.290355854	0.122411706	-2.371961497	0.017693934	0.298868163
RPS6KA3	2087.70393	-0.26101019	0.110198266	-2.368550788	0.017857928	0.301073438
RRAGC	557.5464278	-0.441343481	0.186346408	-2.368403481	0.017865041	0.301073438
STARD4-AS1	223.2059093	0.747221622	0.315523875	2.368193601	0.017875179	0.301073438
NOTUM	41.21383618	2.00076862	0.845555767	2.366217225	0.017970897	0.302162168
ASF1B	540.7977675	-0.358374709	0.151458433	-2.36615883	0.017973732	0.302162168
OCIAD1	1548.197136	-0.297254508	0.125683814	-2.365097768	0.018025312	0.302514152
SDC1	2898.136945	0.303488583	0.128367783	2.364211454	0.018068496	0.302514152
COL8A1	125.9377746	-1.022612338	0.432629606	-2.363713263	0.01809281	0.302514152
MOCS2	242.5081757	-0.35236932	0.149094828	-2.363390633	0.018108571	0.302514152
MIEN1	113.3610155	0.489716522	0.207230772	2.363145771	0.01812054	0.302514152
MOB4	41.42917873	-0.743649544	0.314699299	-2.363047982	0.018125323	0.302514152
FBXL21P	434.2809253	0.518166567	0.219356845	2.362208335	0.01816643	0.302514152
SLC66A2	509.6661103	-0.360389415	0.152571934	-2.362095084	0.018171981	0.302514152
LHB	13.15568151	1.268737506	0.537238012	2.361592958	0.018196609	0.302514152
ZNF84	807.6682417	-0.241771563	0.102390244	-2.361275395	0.0182122	0.302514152
LRATD2	31.7360544	-0.946292724	0.400803123	-2.360991395	0.018226154	0.302514152
HAS2	2338.701007	-1.158183586	0.490571524	-2.36088629	0.01823132	0.302514152
SNORD146	2.108352091	4.430254898	1.876544014	2.360858506	0.018232686	0.302514152
FAM216A	249.6961036	0.316776059	0.134195229	2.360561267	0.018247304	0.302514152
SNORA64	5.832580406	2.219467257	0.940415108	2.360093151	0.018270347	0.302514152
ANKRD61	59.35908303	1.142153109	0.484266969	2.358519539	0.018347994	0.302514152
CKB	203.7688754	-0.474635084	0.201299767	-2.357852128	0.018381013	0.302514152
CLCN4	444.745731	0.471894057	0.200141999	2.357796263	0.018383779	0.302514152

PPRC1	1307.883874	0.330062716	0.140002885	2.357542245	0.018396362	0.302514152
HSPB8	251.3645613	0.410647268	0.174192202	2.357437714	0.018401542	0.302514152
SLIT2	1898.309619	-0.690899447	0.293083692	-2.357345244	0.018406125	0.302514152
NEAT1	11374.73893	0.785731675	0.333322485	2.357271739	0.01840977	0.302514152
HNRNPUL1	3786.202441	-0.283188903	0.120135427	-2.357247225	0.018410985	0.302514152
STAG1	804.0517664	-0.406906639	0.172656125	-2.356746042	0.01843585	0.302514152
RMND1	414.0190709	-0.374227213	0.15881702	-2.35634199	0.018455918	0.302514152
ZNF268	652.2114169	-0.268900534	0.114121873	-2.356257638	0.018460109	0.302514152
TMX3	1962.369245	-0.273079088	0.115899508	-2.356171242	0.018464404	0.302514152
THRA	746.788229	-0.543435921	0.230654663	-2.356058679	0.01847	0.302514152
LGI2	92.50730807	-0.538769479	0.228813236	-2.354625495	0.018541382	0.303404428
MACO1	343.6624932	-0.383998204	0.163140797	-2.353784036	0.018583404	0.303813087
DDX39A	1109.111866	-0.215374287	0.091549328	-2.352549073	0.018645229	0.304544443
MAX	691.498134	-0.347152871	0.147616824	-2.351716163	0.018687028	0.304947659
TCF24	43.1840077	1.044619866	0.444300809	2.351154544	0.018715259	0.305128924
FIP1L1	598.1189337	-0.313048267	0.133174568	-2.350661031	0.018740097	0.305254595
ASXL1	1704.878851	0.20978472	0.089286768	2.349561141	0.018795557	0.305878379
GLUD1	2306.781764	-0.348378743	0.148315093	-2.348909583	0.018828478	0.306134565
ARHGAP5	3141.402658	-0.24415323	0.104000361	-2.347619061	0.018893834	0.306917151
SLC39A10	1970.974634	0.469071968	0.199969916	2.345712689	0.01899074	0.308192878
STARD4	660.8141923	0.33235782	0.141706574	2.345394497	0.019006957	0.308192878
ACOT2	303.4767267	-0.31289449	0.13356826	-2.34258117	0.019150868	0.310244058
DLG3	123.7751421	-0.486080317	0.207580255	-2.341650057	0.019198707	0.310388832
HADHA	3556.55036	-0.283598397	0.121110917	-2.341641893	0.019199126	0.310388832
FOXP1	846.2034124	-0.372996041	0.159325887	-2.341088749	0.019227596	0.310388832
BRWD3	972.7036297	0.31144108	0.133034657	2.341052236	0.019229476	0.310388832
PRDM6	229.3412803	0.429197244	0.183438482	2.339733956	0.019297481	0.311204623
ZDHHC15	4.077181216	-2.523601764	1.078833206	-2.339195485	0.019325319	0.311371771
CDKN2C	339.8709485	-0.367116927	0.156998417	-2.338347955	0.019369205	0.311796962
KIAA0232	653.9601008	-0.241698666	0.10342235	-2.33700614	0.019438865	0.312635895
CYTH2	357.3916343	-0.337656483	0.144522562	-2.336358277	0.019472577	0.312802658
B4GALT3	549.2301867	0.312591248	0.133807161	2.336132428	0.019484341	0.312802658
SLC7A1	4708.33195	-0.318248638	0.136250411	-2.335762777	0.019503609	0.312830158
XDH	3.291686405	3.606501472	1.544466137	2.335112041	0.019537568	0.313092232
LOC101448202	88.32458995	1.026635854	0.439746611	2.334607767	0.01956392	0.313092232
DENR	1710.666463	-0.336560725	0.144185929	-2.334213378	0.019584552	0.313092232
GRIA2	80.16336108	0.653545837	0.280035334	2.333797772	0.019606314	0.313092232
CEP89	295.8851871	-0.421941043	0.180798082	-2.333769468	0.019607796	0.313092232
NIPAL1	91.84629467	0.688526349	0.295106418	2.333145971	0.019640485	0.313231549
H2AC11	3429.793492	-0.536819482	0.230133462	-2.332644179	0.019666828	0.313231549
LOC100996724	73.47816506	0.567987468	0.243499938	2.332598003	0.019669254	0.313231549
CDK4	1934.439941	-0.272333086	0.116773206	-2.332153885	0.019692597	0.313323287
ZNF252P	562.4097616	-0.305056249	0.130844542	-2.331440379	0.01973015	0.31364075
ANLN	3659.886764	0.298001325	0.127838624	2.331074242	0.019749445	0.31365189
SF3A1	1793.754424	-0.284324491	0.121987923	-2.330759336	0.019766053	0.31365189
ATF5	139.0872744	-0.769370728	0.330145906	-2.330396087	0.019785226	0.31367681
ACP1	1191.742638	-0.261442669	0.112205057	-2.330043539	0.01980385	0.313692985
PCBP1	4245.221323	-0.300178353	0.128857868	-2.329530643	0.019830972	0.313795634
FNIP1	1104.975441	-0.215024335	0.09231463	-2.329255226	0.019845549	0.313795634
NT5DC3	1263.073895	0.228684656	0.098253917	2.327486393	0.019939392	0.314889474
PRB4	8.114102444	2.211646412	0.95031155	2.327285627	0.019950068	0.314889474
RTTN	856.4249712	0.38900478	0.167240647	2.326018143	0.020017583	0.31567551
KLHL17	824.2358069	0.563919872	0.242555116	2.324914361	0.02007654	0.316325324
ULK1	607.1070293	-0.365121212	0.157070287	-2.324572132	0.02009485	0.316334129
PPM1E	90.17026635	-0.481782218	0.207347713	-2.323547295	0.02014977	0.316918713
SERPINB8	52.94167677	-0.721547553	0.310632622	-2.322832505	0.020188152	0.317242391
CAPNS1	1911.430936	-0.37471998	0.161432153	-2.321222716	0.020274827	0.318104307
ZBTB39	399.8733181	0.275290187	0.11860617	2.32104441	0.020284447	0.318104307
NEDD9	119.0459179	0.65203656	0.280950925	2.320820118	0.020296554	0.318104307
FAM214B	51.62487039	-0.764487605	0.329486352	-2.320240579	0.020327867	0.318121382
VCAN	1908.309439	0.227251822	0.097947494	2.320139215	0.020333348	0.318121382
NFATC3	836.720285	0.294918144	0.127137968	2.319670102	0.02035873	0.318239101
CAPG	652.2981902	-0.737404243	0.31811117	-2.318070892	0.020445469	0.319314855
TNRC6B	1804.511599	-0.353088226	0.152378517	-2.317178518	0.020494009	0.319448688
MEST	1577.546548	-0.490428498	0.211656323	-2.317098264	0.02049838	0.319448688
GBP5	218.2190623	-0.574971141	0.248231854	-2.316266556	0.020543719	0.319448688
SAP18	402.5944899	-0.315039595	0.136016676	-2.316183614	0.020548245	0.319448688
GCNT3	7.062323161	2.029260826	0.876237218	2.315880658	0.020564786	0.319448688
STEAP1	166.4165219	-0.409075242	0.176662976	-2.315568615	0.020581834	0.319448688
CNOT1	6011.798617	-0.25337356	0.109434876	-2.315290788	0.020597024	0.319448688
GPRIN3	24.41766095	1.099512462	0.474893332	2.315283007	0.020597449	0.319448688
RPL32P3	117.8636542	0.611187315	0.264187679	2.313458815	0.020697427	0.320720124
PSME4	3505.180287	0.194421469	0.084056931	2.312973686	0.020724086	0.320854224
DYNC2H1	2050.86933	-0.478076377	0.206751216	-2.312326795	0.020759681	0.321126318
UHRF1BP1L	2050.325183	0.342632657	0.148242202	2.311303074	0.02081612	0.321613327
ANXA5	5834.228392	-0.321855137	0.139265546	-2.311089467	0.020827914	0.321613327
SEPTIN6	362.2370169	-0.357403083	0.154668085	-2.310774599	0.020845308	0.321613327
DARS1	1062.262809	-0.260416825	0.112718278	-2.310333598	0.020869692	0.321710999
SEC16B	5.459699684	2.338152103	1.012529079	2.309219707	0.020931393	0.322281625
NOL6	984.3619144	0.383580844	0.166136382	2.308831097	0.020952956	0.322281625
ARPC4	921.7662633	-0.310063911	0.134303149	-2.308686835	0.020960965	0.322281625
RNF175	314.7411612	-0.394705109	0.171029472	-2.30781926	0.021009191	0.322550357
RBCK1	743.2519878	-0.269172258	0.116639848	-2.307721267	0.021014644	0.322550357
ERCC6L2	1277.877988	0.253832524	0.110046404	2.306595349	0.021077389	0.323235003
GRK3	428.4443805	0.407695623	0.176865574	2.305115763	0.021160091	0.324111841
DCN	11271.73387	-0.337007474	0.146239669	-2.304487394	0.021195299	0.324111841
LOC101927854	4.014171916	3.411076727	1.480310992	2.304297371	0.021205956	0.324111841
CDC42EP3	1680.427872	-0.641359802	0.278369008	-2.303991407	0.021223126	0.324111841
LPAR1	727.3363596	-0.35474776	0.153973789	-2.30394901	0.021225506	0.324111841
RPL10A	5127.823414	-0.311597813	0.135279164	-2.303368856	0.021258099	0.324276852

DUOX1	88.8213647	0.716454685	0.311142427	2.302658274	0.021298078	0.324276852
LRRC47	1153.948202	-0.23891656	0.103763242	-2.302516344	0.021306071	0.324276852
ZNF652	1089.951307	0.433138056	0.188129604	2.302338636	0.021316083	0.324276852
CPVL	564.0776505	-0.244776821	0.106333408	-2.301974751	0.021336596	0.324276852
HARS1	583.690627	-0.285613273	0.124081661	-2.301816963	0.021345497	0.324276852
BAIAP2L1	224.6896647	-0.410369072	0.178412199	-2.300117784	0.021441548	0.325458591
C6orf136	94.65840323	0.482044921	0.209605325	2.299774209	0.021461015	0.325476846
RAB26	121.7893417	0.700249343	0.304607082	2.298861005	0.021512833	0.325674154
CEBPZ	1428.846954	-0.230053821	0.100077048	-2.298767069	0.02151817	0.325674154
VCL	7802.592374	-0.212307684	0.092364751	-2.29857908	0.021528853	0.325674154
GPKOW	170.6263755	-0.395930656	0.17230525	-2.297844417	0.021570646	0.326029608
RAB1A	2600.471708	-0.33621458	0.146351606	-2.297307084	0.021601258	0.326215607
CEP85	550.1476927	0.271212759	0.118085206	2.296754753	0.021632764	0.32641478
LINC01252	32.76120002	1.074302327	0.467950656	2.29575985	0.021689616	0.326995738
KCNK15-AS1	10.05011485	1.568249938	0.683297763	2.295119382	0.021726284	0.32727166
EPHX4	42.54071335	-0.684061356	0.298109788	-2.294662517	0.021752472	0.327389408
NCOA5	516.9244219	-0.461504655	0.20115734	-2.294247156	0.021776306	0.327471539
MAF	102.2755601	-3.183997726	1.388063929	-2.293840837	0.021799643	0.327546066
UBE2E1	752.0735637	-0.234290101	0.102193067	-2.292622257	0.021869762	0.32783512
LINC01603	2.199659509	4.114512799	1.794695539	2.29259655	0.021871243	0.32783512
AGPAT4-IT1	29.54490753	1.163248577	0.507404358	2.292547469	0.021874072	0.32783512
SIRT3	217.8149371	-0.340132676	0.148556023	-2.289591971	0.022044981	0.329226568
RAP2C	860.5265942	-0.299597619	0.130859855	-2.289454004	0.022052987	0.329226568
APBB2	488.7802801	-0.381444945	0.166619357	-2.289319517	0.022060795	0.329226568
SPAG5	1330.520217	0.240340693	0.104985009	2.289285837	0.02206275	0.329226568
COL5A1-AS1	90.17949137	0.792809527	0.346336291	2.289132117	0.022071677	0.329226568
MIR762HG	15.01925004	1.358176698	0.593342277	2.289027345	0.022077764	0.329226568
CHN1	541.1079718	-0.53688902	0.234649557	-2.288046169	0.022134833	0.329776579
UBB	5329.184857	-0.325688589	0.142361513	-2.287757295	0.022151659	0.329776579
VAC14-AS1	9.290796524	1.607272683	0.702893225	2.286652689	0.022216103	0.3304599
LAMTOR3	308.2232479	-0.293838898	0.128546251	-2.285861286	0.022262375	0.330780562
EDA	268.9147112	-0.559210082	0.244681243	-2.285463632	0.022285657	0.330780562
RBM8A	920.230458	-0.337170783	0.147536871	-2.285332346	0.022293348	0.330780562
ERI3-IT1	47.29427319	1.191702551	0.521551539	2.284918099	0.022317631	0.330865371
ASB6	594.8793862	0.315197154	0.138274106	2.279509611	0.022636789	0.335318026
TIMM44	1078.365314	-0.533553812	0.234133286	-2.278846469	0.022676193	0.335622731
LMBR1L	305.3749509	0.420202941	0.184443342	2.278222328	0.022713335	0.335648947
INTS9	613.3614874	-0.246636018	0.108259935	-2.278183687	0.022715636	0.335648947
NOL7	340.0430292	-0.448777424	0.197037208	-2.277627809	0.022748762	0.335859935
KMT2E	1228.963807	-0.23650541	0.103866233	-2.277019237	0.022785077	0.336117608
TMEFF2	14.693007	-1.803248447	0.792155058	-2.276383176	0.022823086	0.336399824
YPEL5	729.5760629	-0.43606076	0.191688475	-2.27484078	0.022915483	0.337231093
SLC34A3	66.89737105	0.774354021	0.340453071	2.274480939	0.022937086	0.337231093
TMX4	1184.258715	-0.296831474	0.130508788	-2.274417524	0.022940895	0.337231093
LOC100268168	20.4439317	1.133376706	0.498367246	2.274179763	0.02295518	0.337231093
UBE2Q2	1207.760302	-0.222278397	0.097761955	-2.273669728	0.022985852	0.337403522
NPM1	13181.25583	-0.276086595	0.121453283	-2.273191703	0.02301463	0.337438534
SCFD2	592.0481543	0.315929748	0.138992338	2.273001182	0.023026109	0.337438534
ANO5	28.29331447	0.926666665	0.408066007	2.27087444	0.02315458	0.339042412
EGR3	57.44336366	-1.286770617	0.566925403	-2.26973533	0.023223646	0.339774532
TNFRSF8	262.5747491	0.522264616	0.230159092	2.269146146	0.02325944	0.339841725
APBB1	326.7538773	-0.369233861	0.162745057	-2.268786948	0.023281285	0.339841725
PLP2	428.1778671	-0.347039884	0.15297351	-2.26862732	0.023290999	0.339841725
FBXL4	932.9035423	0.31608479	0.139342298	2.26840518	0.023304522	0.339841725
PLXNA1	3322.309684	-0.252929844	0.111568696	-2.267032365	0.023388249	0.340580452
MYO5A	1478.286351	0.242934162	0.107163537	2.266947962	0.023393405	0.340580452
CBX4	160.5301935	-0.496003049	0.218956557	-2.265303475	0.023494063	0.341766695
CRACR2B	61.58456744	0.864815877	0.381946241	2.264234556	0.023559692	0.342441857
RALY-AS1	17.66993099	1.263048527	0.558142375	2.262950429	0.023638745	0.342956871
RPL3	14126.01296	-0.439315877	0.194146864	-2.262801817	0.023647909	0.342956871
H4C8	1405.937928	-0.511677552	0.226133684	-2.262721515	0.023652862	0.342956871
BPIFB4	347.6717101	0.770419601	0.340628154	2.261761373	0.023712151	0.343537011
PRKAA1	1193.274099	-0.204155831	0.090322764	-2.260292112	0.023803127	0.344574921
MRPL30	1667.810956	-0.226421671	0.10020983	-2.25947566	0.023853813	0.345028362
GCH1	116.8474125	0.476137746	0.210801032	2.258706908	0.023901622	0.345439508
KLF10	1560.979447	-0.41405819	0.183374543	-2.257991673	0.023946179	0.345692152
PAF1	357.0807933	-0.285613598	0.126509031	-2.257653826	0.02396725	0.345692152
DISP2	38.25964297	-0.839555044	0.371897113	-2.257492769	0.023977301	0.345692152
R3HDM4	436.8209218	-0.376005204	0.166737994	-2.255066141	0.024129178	0.347061169
TMEM63A	782.8075434	0.293638304	0.130223555	2.25487857	0.024140952	0.347061169
HCFC1	3105.235374	0.225061059	0.099813971	2.254805186	0.02414556	0.347061169
DRG1	521.9266205	-0.319182492	0.141561171	-2.254731934	0.02415016	0.347061169
CEP290	696.0444713	0.404039987	0.179224354	2.254381048	0.024172207	0.347098082
SAP30L-AS1	58.2778855	0.665992043	0.295486529	2.253882928	0.024203534	0.347268095
ABCA17P	40.32626731	1.016083854	0.451016071	2.252877266	0.024266889	0.347896988
ATP2A1-AS1	14.60110487	1.261040339	0.559850712	2.25245822	0.02429333	0.347996096
IK	363.9612212	-0.345505281	0.153468096	-2.251316648	0.024365489	0.34874941
COX10-AS1	210.3243265	0.552363698	0.245426048	2.25063192	0.02440886	0.349089795
GCSAM	23.39528704	1.152167013	0.512337781	2.248842572	0.024522514	0.350433998
TUBA1C	3265.132135	0.377716508	0.168007799	2.248208171	0.024562919	0.35073014
FKBP9	4596.539481	-0.317533768	0.141264179	-2.247801042	0.024588879	0.350819721
ZDHHC17	803.8138127	0.258575912	0.115079803	2.24692695	0.024644696	0.351334787
GLI1	34.95811911	-0.820914819	0.365428986	-2.246441442	0.024675746	0.351496243
OLA1	527.9627556	-0.292768761	0.13035971	-2.245853114	0.024713418	0.351751683
UBE2A	475.8812003	-0.312583598	0.139240359	-2.24492094	0.024773208	0.352321288
ZNF138	111.4986347	0.691077927	0.307931115	2.244261439	0.024815585	0.352612476
SMIM3	374.2654072	-0.620538426	0.276533916	-2.243986686	0.024833258	0.352612476
G2E3	1213.369732	0.293796185	0.130965183	2.243315187	0.024876497	0.3529452
H2AC8	525.1511552	-0.469382375	0.209542905	-2.240029908	0.025088981	0.355676728
CD44	4400.546799	-0.517463793	0.231109027	-2.239046222	0.025152909	0.356208234

EME2	194.6606433	0.864094815	0.38595681	2.238838111	0.025166452	0.356208234
LOC102723809	36.36169769	0.838317556	0.374843855	2.23644471	0.025322655	0.358134695
PIDD1	340.5600022	0.355217504	0.158889355	2.235628078	0.025376144	0.358606568
STRBP	213.284707	-0.40795842	0.182532741	-2.234987641	0.02541816	0.3589157
SLC24A1	223.578285	-0.397049032	0.177689873	-2.234505691	0.025449819	0.35901817
BUB1	1166.95636	0.270010571	0.120849898	2.23426395	0.025465711	0.35901817
DZIP1	1261.483511	0.283707151	0.127068967	2.232702108	0.025568595	0.359590021
GRB14	23.75709425	0.992075905	0.444341465	2.232688108	0.025569519	0.359590021
AKR1B1	1143.905613	-0.28796362	0.128978855	-2.232642085	0.025572556	0.359590021
PAGR1	682.3068062	0.269909855	0.120904416	2.232423453	0.025586989	0.359590021
SOD3	33.80888734	-0.971562986	0.435463261	-2.231102076	0.025674368	0.360533683
MRPS30-DT	10.16631393	1.473407947	0.660737913	2.229943097	0.025751221	0.360717954
REPS2	6.406458851	-1.715224503	0.769220004	-2.22982306	0.025759192	0.360717954
VPS35	2112.131382	-0.33806857	0.151614024	-2.22979749	0.02576089	0.360717954
UBAP2L	2572.73962	-0.285958938	0.128250908	-2.229683532	0.02576846	0.360717954
ARL6IP4	622.6031879	-0.322558484	0.14470704	-2.229044857	0.02581092	0.360840245
PARP11	124.8514676	-0.428909681	0.192436105	-2.228842043	0.025824416	0.360840245
PNMA1	940.1265065	-0.283420452	0.127188864	-2.228343299	0.025857631	0.360840245
REM2	15.51374361	1.247606341	0.559882789	2.228334871	0.025858193	0.360840245
TOX4	1340.079873	-0.248705997	0.111690349	-2.226745636	0.025964279	0.362037123
GNG13	6.131183768	2.272594069	1.020821685	2.22624	0.02599811	0.362225426
SH3RF2	170.2879297	0.659634499	0.296413884	2.225383275	0.026055519	0.362510972
OLFML2B	1450.456878	-0.667340375	0.299919285	-2.225066569	0.02607677	0.362510972
FBXL3	608.1987333	-0.24667479	0.110882412	-2.224652101	0.026104602	0.362510972
FLJ31356	131.0712506	0.54473447	0.24489389	2.224369378	0.026123602	0.362510972
SLC25A44	417.7849407	0.284057235	0.127714206	2.224163182	0.026137467	0.362510972
RNF138P1	45.30067508	1.067226278	0.479906598	2.223820806	0.026160503	0.362510972
RPS27	452.4290173	-0.561993414	0.25271609	-2.223813347	0.026161005	0.362510972
LRP4	3068.547081	0.695531687	0.313156014	2.221038893	0.026348327	0.364822992
HCFC1-AS1	25.28648115	0.896849074	0.404019847	2.219814399	0.026431369	0.365688662
TAF1D	1365.600784	0.393037018	0.177152911	2.218631438	0.026511809	0.366261504
ZDHHC18	515.6858009	0.238533054	0.107515075	2.218601023	0.02651388	0.366261504
ZNF385D	66.30643956	-0.860448024	0.387892241	-2.218265622	0.026536727	0.366293161
FZD3	692.1930931	0.306765858	0.138339601	2.217484047	0.026590032	0.366744869
OTUD7B	1246.389275	0.414825041	0.187104887	2.217072188	0.026618159	0.366824631
PURA	798.0011527	-0.232243069	0.104772806	-2.216635006	0.026648043	0.366824631
BMP3	10.58317163	-1.586521525	0.715779193	-2.21649573	0.02665757	0.366824631
LOC646903	40.72358523	0.860447104	0.388256724	2.216180814	0.026679121	0.366837918
TM4SF1	312.3440942	0.456362865	0.206012855	2.215215482	0.026745278	0.367320094
DCTN3	248.9070412	-0.432387103	0.195202653	-2.215067755	0.026755414	0.367320094
LOC730268	139.2006955	0.432933211	0.195495229	2.21454617	0.02679123	0.367359029
SLC37A3	875.9324761	0.219345207	0.099064495	2.214165699	0.026817382	0.367359029
PTPRG	1322.625367	-0.349511331	0.157870699	-2.213908807	0.026835053	0.367359029
HIPK2	1942.812985	0.24997281	0.112932914	2.213462843	0.026865752	0.367359029
SNORA72	54.9318138	0.735728901	0.332496049	2.212744792	0.026915245	0.367359029
ADAMTS7	141.5962832	0.545742091	0.246643264	2.212677863	0.026919863	0.367359029
NTN3	127.3503822	0.98852055	0.446770002	2.212593832	0.026925661	0.367359029
PHLPP2	412.6695353	0.373182698	0.168672389	2.212470573	0.026934167	0.367359029
SYAP1	847.6968505	-0.26324599	0.118995878	-2.212227804	0.026950929	0.367359029
CYP26A1	3.843477698	4.139118017	1.871212368	2.211998001	0.026966803	0.367359029
KSR1	32.3568976	-0.796599047	0.36018765	-2.21162232	0.026992772	0.367359029
CPT1C	39.4485493	-0.946466534	0.427987224	-2.211436421	0.02700563	0.367359029
LNX1	49.81628561	-0.669618712	0.303065209	-2.209487241	0.027140769	0.368571104
AMER1	572.7687915	0.267915495	0.121308886	2.208539728	0.027206672	0.368571104
YJU2	96.65840082	-0.492007872	0.222797615	-2.208317499	0.027222148	0.368571104
MINPP1	378.3937037	0.27667249	0.125296796	2.208136993	0.027234725	0.368571104
DCP2	1349.504186	0.27036599	0.122462556	2.207744145	0.027262114	0.368571104
CALCA	4.063669347	3.428090565	1.55278693	2.207701842	0.027265064	0.368571104
HCFC1R1	270.8272544	0.366650133	0.166111076	2.20725879	0.027295985	0.368571104
RNF187	1163.342091	-0.299614591	0.135740755	-2.207255968	0.027296182	0.368571104
SLFN5	1030.603503	-0.811928175	0.367984938	-2.206416871	0.027354825	0.368571104
TDG	1074.715185	0.305438047	0.138449978	2.206125641	0.027375204	0.368571104
WWC1	354.4003349	0.318000941	0.1441521	2.206009779	0.027383315	0.368571104
MPP4	9.8419818	1.476927977	0.669622529	2.205612734	0.027411127	0.368571104
LOC101927402	1.865669489	3.684575831	1.670582474	2.205563561	0.027414573	0.368571104
THADA	1645.284089	0.246226785	0.111638987	2.205562681	0.027414634	0.368571104
PIGC	310.3985884	-0.418980184	0.190029663	-2.204814643	0.027467104	0.368571104
ZNF22	476.1482992	-0.312405613	0.141696555	-2.204750945	0.027471576	0.368571104
PAQR4	427.3565339	-0.296420104	0.134448038	-2.2047187	0.02747384	0.368571104
HMBOX1	1010.391982	0.32084167	0.145530082	2.204641588	0.027479255	0.368571104
COMT	940.6481605	-0.313583307	0.142245539	-2.204521206	0.02748771	0.368571104
MEIS3P1	255.6681883	-0.378887316	0.17201762	-2.202607591	0.027622418	0.370098869
CAPZA2	1644.567672	-0.269524947	0.122426174	-2.201530433	0.027698495	0.37083935
SLC25A35	108.1734092	0.754850301	0.343086057	2.200177729	0.027794288	0.371726486
SESN3	3346.642427	0.668451321	0.303840694	2.200005903	0.027806476	0.371726486
SNORD45A	78.96258573	0.927720847	0.422116108	2.19778594	0.027964364	0.372878701
PHEX	16.46436401	-1.276147453	0.58065194	-2.197783844	0.027964513	0.372878701
ITGA10	1218.793545	-0.343534572	0.156319517	-2.197643512	0.02797452	0.372878701
FUT10	1013.448006	0.219442834	0.099854875	2.197617643	0.027976365	0.372878701
RNF207	302.851855	0.647540292	0.294722169	2.197121087	0.028011799	0.372970138
MTX3	1018.112563	0.236508641	0.107653907	2.196935052	0.028025085	0.372970138
EML4	3473.08123	-0.265177359	0.120737842	-2.196306932	0.028069982	0.373288864
HOMER2	365.7296715	0.406903975	0.185328752	2.195579316	0.028122068	0.37360119
ZNF596	48.4271959	-0.635396341	0.289454163	-2.195153578	0.028152583	0.37360119
EBF1	455.0874652	-0.58835642	0.268031611	-2.19510086	0.028156364	0.37360119
PWRN1	11.0112776	1.724200642	0.785913301	2.193881488	0.028243931	0.374484258
PALB2	364.6021054	-0.354585942	0.161685958	-2.193053409	0.028303531	0.374870812
NAGK	254.9087799	-0.338660732	0.154445934	-2.192746176	0.028325672	0.374870812
RPS9	3531.189562	-0.34114249	0.155593706	-2.192521147	0.028341898	0.374870812
H2AZ1	5878.551362	-0.315734582	0.144019223	-2.192308604	0.028357231	0.374870812

GLUD2	48.68563511	-0.717902297	0.327663941	-2.190971316	0.028453868	0.375362559
PTPRF	3391.14015	0.214521969	0.097922428	2.190733746	0.028471066	0.375362559
CLMAT3	106.4096463	0.725743571	0.331352608	2.19024554	0.028506434	0.375362559
NOTCH3	310.6853891	0.378805224	0.172972025	2.1899797	0.028525709	0.375362559
BRD7P3	20.62918757	1.263911471	0.577314002	2.189296408	0.028575303	0.375362559
METTL7A	45.11352191	-1.046848937	0.478204417	-2.189124357	0.028587803	0.375362559
PRPF31	892.4398832	-0.276395882	0.126259837	-2.189103745	0.0285893	0.375362559
HOGA1	22.19205152	-0.860970458	0.393327535	-2.188940214	0.028601186	0.375362559
MAD1L1	480.0312124	-0.335925909	0.153483461	-2.188678218	0.028620236	0.375362559
MAPK6	2319.167799	0.292868662	0.133820926	2.188511698	0.02863235	0.375362559
ADRB1	4.423164227	2.847560382	1.301183214	2.188439223	0.028637624	0.375362559
DNAJC15	26.74377769	-1.041185741	0.475794951	-2.188307669	0.028647199	0.375362559
DNASE2	854.0251847	-0.495474703	0.226490355	-2.187619439	0.028697335	0.375743206
AKAP5	154.8081726	0.695171454	0.317964062	2.186320835	0.028792141	0.376707746
TMEM81	61.05351705	0.683567203	0.312772015	2.185512674	0.028851278	0.377137262
ZNF16	189.3682766	-0.352939747	0.161506757	-2.185294007	0.028867297	0.377137262
FLNB-AS1	42.74803337	1.116288423	0.511091646	2.184125746	0.028953009	0.377440465
RETSAT	684.8748675	-0.244644186	0.11202023	-2.183928616	0.028967494	0.377440465
AARS2	1154.948772	0.229435188	0.105059388	2.183861832	0.028972402	0.377440465
NAPB	161.80277	0.616821884	0.282450442	2.183823398	0.028975228	0.377440465
ZBTB25	1155.447224	0.307694214	0.140964316	2.182780876	0.029051951	0.378163449
TMEM161B-AS1	55.42171053	0.747643454	0.342701079	2.181619782	0.029137606	0.378729867
FBXO8	155.9579814	-0.486839703	0.22318482	-2.18132982	0.02915903	0.378729867
RMC1	196.3530707	0.337661492	0.154796352	2.181327196	0.029159224	0.378729867
TAZ	418.219851	0.253826838	0.11640503	2.180548713	0.029216812	0.378730684
LRSAM1	528.3401051	0.239455726	0.109818047	2.180477013	0.029222121	0.378730684
SUB1	607.2857929	-0.308223031	0.141356591	-2.180464513	0.029223047	0.378730684
FKBPL	71.39578147	-0.544257782	0.249642827	-2.180145889	0.029246649	0.37876111
CCDC65	47.03112229	0.760597999	0.349021298	2.179230906	0.029314519	0.379364365
TEX261	1361.91454	-0.242589874	0.111346345	-2.178696338	0.029354234	0.379602649
CCDC14	959.9351916	0.361892235	0.166162976	2.177935449	0.029410843	0.379855025
TPBG	653.7761539	-0.281599293	0.129300853	-2.177861059	0.029416382	0.379855025
MMRN2	10.19635961	1.424548509	0.654359213	2.177012995	0.029479598	0.380124032
TXNL1	452.4096803	-0.278260373	0.127820326	-2.176964983	0.02948318	0.380124032
H2AC16	59.2261883	-0.976987341	0.448836605	-2.176710476	0.029502176	0.380124032
SEC23B	713.4461499	0.285663583	0.131252813	2.1764378	0.02952254	0.380124032
CAPN6	26.23248673	-1.04598927	0.480869671	-2.175203248	0.029614889	0.381037777
WASL	513.9749897	-0.255961874	0.117694995	-2.174789793	0.029645872	0.381125772
ADGRA2	8092.733155	-0.264234453	0.121512728	-2.174541355	0.029664503	0.381125772
EFEMP1	15.93810259	-1.105705042	0.508791549	-2.173198521	0.029765379	0.382146294
NDST3	34.73998717	-1.001725672	0.461349139	-2.171296286	0.029908783	0.383001087
DYRK2	1656.441787	0.18917921	0.087130678	2.171212426	0.029915119	0.383001087
NCOA7	378.3360832	-0.448777784	0.206723868	-2.170904547	0.029938389	0.383001087
NSUN3	241.834604	0.381184616	0.175596559	2.170797754	0.029946464	0.383001087
RGS19	105.4059443	0.512320852	0.236014878	2.170714219	0.029952782	0.383001087
DDX5	7154.493071	-0.388034581	0.178811686	-2.170073944	0.030001245	0.383001087
RNF130	622.168031	-0.342008757	0.157613841	-2.169915756	0.030013229	0.383001087
SNHG12	447.84818	0.605608476	0.279098348	2.169874817	0.030016331	0.383001087
TTC34	7.401726421	1.800540117	0.829835594	2.169755226	0.030025394	0.383001087
ITFG2-AS1	29.62358615	1.157744716	0.533991894	2.168094176	0.030151522	0.384194848
SNORD143	17.83354033	1.169963905	0.539662409	2.16795516	0.030162098	0.384194848
MEX3D	423.7811963	-0.330897777	0.15268759	-2.16715567	0.030222986	0.38469544
P4HA2	5635.027243	0.695682514	0.321281462	2.165336618	0.030361916	0.386187969
IQCG	146.1639344	-0.475176763	0.219553209	-2.164289766	0.030442118	0.386595003
PIAS3	544.89204	0.251596336	0.116257889	2.164122699	0.030454934	0.386595003
SPRY4	172.8634843	-0.789937257	0.365023943	-2.164069704	0.030459	0.386595003
LMO7	932.27668	0.507225019	0.234436574	2.16359167	0.030495701	0.386785337
PI4K2A	509.3942719	-0.282678515	0.130677437	-2.163177683	0.030527516	0.386816082
APP	6021.391692	-0.291223935	0.134639188	-2.162995326	0.030541539	0.386816082
TIAM1	12.47733197	-1.894561463	0.876104705	-2.162482923	0.030580972	0.386972984
DHFR2	169.6143348	-0.351140597	0.162394982	-2.162262604	0.03059794	0.386972984
HBP1	599.4999913	-0.439111988	0.20311857	-2.161850531	0.030629699	0.386972984
PSMD4	1462.89188	-0.256189402	0.1185151	-2.161660429	0.03064436	0.386972984
TARBP1	2000.502722	0.273633589	0.126598683	2.161425244	0.030662506	0.386972984
VPS13A	1507.819898	0.415342216	0.192312241	2.159728439	0.030793698	0.388159968
PHTF2	613.662691	0.28599355	0.132426155	2.159645501	0.030800123	0.388159968
AGT	7.464655143	-1.578032201	0.73108546	-2.158478438	0.030890653	0.389025751
SCUBE3	441.507247	0.534082812	0.247494761	2.157956036	0.03093125	0.389070782
TSPAN3	2144.31698	-0.246112643	0.114053477	-2.157870579	0.030937896	0.389070782
LETM2	278.7999534	-0.390076163	0.180792913	-2.157585473	0.030960075	0.389075134
RSRP1	705.5325954	0.422396823	0.195870515	2.156510505	0.031043824	0.389772302
DST	9074.072144	0.26028357	0.12070774	2.156312181	0.031059297	0.389772302
GOLPH3	976.3989398	-0.354309442	0.1643541	-2.155768807	0.031101722	0.390030044
LRRC8D	740.0984415	-0.24254093	0.112523117	-2.155476468	0.031124568	0.390042057
GOLGA4	652.3743653	0.331512491	0.153845989	2.154833503	0.031174865	0.390165988
PTGES	1430.695635	1.987518238	0.922388726	2.154751225	0.031181307	0.390165988
TFDP2	1050.447335	-0.394821687	0.183253526	-2.154510721	0.031200142	0.390165988
THRB	246.6778653	0.605993542	0.281313899	2.15415429	0.031228074	0.390241432
NDUFV1	892.9076546	-0.250549727	0.116354466	-2.153331425	0.031292641	0.390527822
HEATR4	64.22846655	0.748983819	0.347830097	2.153303651	0.031294822	0.390527822
NCOA3	1081.767336	0.338803041	0.157367	2.152948469	0.031322729	0.390602536
UBE2B	500.597396	-0.46993249	0.218433129	-2.151379196	0.031446282	0.391869052
LYRM2	571.7227342	-0.311971447	0.145039736	-2.150937774	0.031481112	0.392028936
SCAI	827.4891503	0.238135645	0.110803019	2.149180109	0.031620125	0.39348508
RPL5	7264.893921	-0.360021807	0.167571694	-2.148464336	0.031676887	0.393916344
USP31	932.7391057	0.245113094	0.11412114	2.147832512	0.031727063	0.39400481
FBXO31	706.0265082	-0.255731055	0.119074266	-2.147660138	0.031740764	0.39400481
ZMYND11	902.9306609	-0.283447662	0.131987151	-2.147539809	0.031750332	0.39400481
CASC15	32.42958038	0.974807984	0.454017016	2.147073676	0.031787417	0.394190511
RBM24	18.50297199	1.166826184	0.543618905	2.146404721	0.031840703	0.39433328

CMTM1	48.86460436	-0.654313826	0.304846201	-2.146373562	0.031843187	0.39433328
ZNF813	96.54338116	0.471654758	0.219808835	2.145749772	0.031892948	0.394632493
RPS27A	2942.828112	-0.386739263	0.180254691	-2.14551567	0.03191164	0.394632493
PSMG2	441.8674682	-0.292097209	0.136192447	-2.144738679	0.031973747	0.394762539
GPM6B	171.8078461	-1.035835078	0.48303578	-2.144427225	0.031998671	0.394762539
TFRC	13443.19377	0.438810994	0.204660184	2.144095572	0.03202523	0.394762539
PNPLA4	106.8856384	-0.546959702	0.255110778	-2.144008595	0.032032198	0.394762539
TGM4	2.308760609	-3.251402465	1.516512636	-2.143999587	0.03203292	0.394762539
POLR2J4	67.83340144	0.701408287	0.327255405	2.143305429	0.032088581	0.394851378
ERH	1123.360844	-0.299576071	0.139781171	-2.143179005	0.032098727	0.394851378
TSPAN14	593.0709475	0.269406265	0.125709796	2.143080911	0.032106602	0.394851378
LY6K	512.3480447	0.389257921	0.181688988	2.142440905	0.032158019	0.395036112
LPP-AS2	81.51749858	-0.514084859	0.239987752	-2.142129562	0.032183058	0.395036112
FIGNL1	153.7084532	-0.523808277	0.244534082	-2.142066544	0.032188128	0.395036112
LPAR2	65.45921184	0.70651634	0.329975383	2.141118328	0.032264497	0.395700852
ZNRF1	309.810768	-0.338882816	0.158427251	-2.139043728	0.032432127	0.397361364
USP27X-AS1	13.09968856	1.174078626	0.548919308	2.138891106	0.032444489	0.397361364
TEP1	1028.887105	0.235273114	0.110026349	2.138334286	0.032489622	0.397640837
CAND2	232.6361145	0.316779283	0.148163245	2.138042288	0.032513311	0.397657657
FOXD2-AS1	142.1056401	0.64623558	0.302321434	2.137577778	0.032551027	0.397845885
FMC1	21.91788647	0.949660287	0.44442427	2.13683264	0.032611606	0.398237603
HHIP-AS1	10.1014678	-1.752792897	0.820374879	-2.136575537	0.032632531	0.398237603
SARAF	3189.042117	-0.265583932	0.124319583	-2.136300046	0.032654965	0.398237603
METTL22	266.8692229	0.442910211	0.207346694	2.136085226	0.032672468	0.398237603
MAGOH	139.6886279	-0.481814109	0.225594641	-2.135751567	0.032699669	0.398296717
LOC107984341	8.960094495	1.457283009	0.6824382	2.13540656	0.032727815	0.39836726
ANKRD13D	164.262601	0.394081404	0.184607349	2.134700526	0.03278548	0.39879676
GSTM5	3.200667484	-3.56968039	1.672623222	-2.134180814	0.032827983	0.39891833
TSPOAP1	261.5288059	0.423900197	0.198662451	2.133771099	0.032861523	0.39891833
TRIM2	925.7951813	0.584254435	0.273826417	2.133667166	0.032870036	0.39891833
SRGAP3	71.55787921	-0.723548366	0.339366508	-2.132055904	0.033002251	0.39891833
AMMECR1	705.1077652	-0.296146412	0.138915719	-2.131842335	0.03301981	0.39891833
FDXR	105.424268	-0.453840226	0.212910848	-2.131597479	0.033039951	0.39891833
SLC5A12	10.88274437	2.242642877	1.052116967	2.131552809	0.033043627	0.39891833
SUGP2	1735.094899	0.397489302	0.18649041	2.131419531	0.033054595	0.39891833
LNX2	227.6844363	0.420626026	0.19735755	2.131289252	0.03306532	0.39891833
JMJD1C	1484.561806	-0.243795201	0.114390932	-2.13124587	0.033068892	0.39891833
LOC374443	101.1790329	-0.544777947	0.255635857	-2.131070162	0.033083362	0.39891833
SLCO3A1	75.10155274	-0.499030066	0.234182547	-2.130944735	0.033093695	0.39891833
CPSF3	1321.720294	-0.227463997	0.106749019	-2.130829857	0.033103162	0.39891833
FAM160B1	440.0219088	0.350696602	0.164587528	2.130760498	0.033108878	0.39891833
PGRMC2	647.4785718	-0.298083591	0.139962229	-2.129743098	0.033192829	0.3996596
HRH1	104.0951482	-0.622857448	0.292598392	-2.128711111	0.033278169	0.40040044
KLC3	162.0906658	0.428137799	0.201160994	2.128334075	0.033309395	0.40040044
RBM4B	151.2283975	-0.43387698	0.203871854	-2.128184794	0.033321765	0.40040044
LCA10	3.388712455	3.616391621	1.699560996	2.127838677	0.033350461	0.400421628
ZNF566	259.0475986	-0.308682057	0.145083156	-2.127621611	0.033368469	0.400421628
GDI1	818.719722	0.239073228	0.112386796	2.127235904	0.033400487	0.400536127
UGDH	716.2636371	-0.277221382	0.130476776	-2.124679886	0.033613333	0.40281748
MKS1	412.3155624	-0.275074664	0.129495488	-2.124202688	0.033653199	0.403024192
YIPF4	1218.449058	-0.232705263	0.109648242	-2.122289055	0.033813472	0.40467164
RTN4IP1	191.814266	0.34556867	0.162884367	2.121558235	0.033874853	0.405118338
MAP3K8	20.68810606	1.078827538	0.508578852	2.121259139	0.033900001	0.405118338
SNORD31	52.88303348	0.720033541	0.339472999	2.121033316	0.033918999	0.405118338
AP2A1	1489.656845	-0.252597947	0.119259144	-2.118059369	0.03417004	0.407650406
HYMAI	516.5804549	0.754572804	0.356270012	2.117980121	0.034176751	0.407650406
PABPC3	131.7431664	-1.780441361	0.840872288	-2.117374286	0.034228095	0.40768407
HIRIP3	214.7755215	-0.365898741	0.172840442	-2.11697411	0.034262045	0.40768407
ZBTB40	1010.84745	0.284904207	0.134602829	2.11662866	0.034291376	0.40768407
PPP1R7	307.9150064	-0.382353074	0.180664747	-2.116367914	0.034313529	0.40768407
ZNF493	49.36082752	0.704022487	0.332684268	2.116188094	0.034328813	0.40768407
CAMK2G	2357.066849	0.204025304	0.096416607	2.116080527	0.034337959	0.40768407
NIPBL	2421.164468	-0.278792628	0.131750824	-2.116059836	0.034339719	0.40768407
EDF1	385.1471209	-0.328014869	0.155047532	-2.115576211	0.034380867	0.407779296
LOC100130950	14.3448094	1.212495816	0.573168151	2.115427755	0.034393506	0.407779296
HIPK1-AS1	6.590147983	1.779609813	0.841463496	2.114898413	0.034438606	0.408042531
HINT1	992.1686972	-0.255651637	0.120901871	-2.114538298	0.034469317	0.40813504
SSRP1	3134.10754	-0.266667824	0.12618477	-2.113312284	0.034574048	0.408727553
ZNF839	293.8638333	-0.33429543	0.15821137	-2.112967165	0.034603578	0.408727553
ABHD16A	404.8925061	-0.264771622	0.125319277	-2.112776482	0.034619903	0.408727553
TBKBP1	359.8841995	0.306208042	0.144938288	2.112678749	0.034628273	0.408727553
KLHL15	430.3252079	0.303133031	0.143491277	2.112553713	0.034638984	0.408727553
INKA2	145.978821	-0.5734541	0.271477644	-2.112343735	0.034656977	0.408727553
COL15A1	13.50696273	-1.952319658	0.92451606	-2.111720653	0.034710416	0.409087051
SLC25A12	390.0530392	-0.330838183	0.156733362	-2.110834473	0.034786542	0.40971327
ERG	425.0228479	0.285394154	0.135231522	2.110411462	0.03482293	0.409870946
AAMP	756.4280255	-0.299686682	0.142077893	-2.109312553	0.034917612	0.41071409
REL	614.9762518	0.317408918	0.150570446	2.108042616	0.035027303	0.411721844
CLIC4	8251.012983	-0.295267983	0.140084593	-2.107783423	0.035049727	0.411721844
FAM72B	144.9804755	0.549395111	0.260683328	2.107519173	0.035072602	0.411721844
RGCC	1242.92137	1.444786552	0.685741129	2.106897912	0.03512643	0.412082276
ALG14-AS1	4.129353184	2.699837182	1.281666429	2.106505345	0.035160479	0.412210357
SMARCA2	798.3263511	-0.260488873	0.123731664	-2.105272529	0.035267592	0.413023964
SLC38A6	226.8096638	0.405149235	0.192454109	2.105173213	0.035276233	0.413023964
ZC3H14	1119.855602	-0.305651925	0.145283558	-2.103830122	0.035393268	0.413724136
RSL1D1	1858.711573	-0.340910435	0.162078903	-2.103360946	0.03543423	0.413724136
VPS41	1734.13273	-0.222162596	0.105631494	-2.10318522	0.035449582	0.413724136
HECW1	349.8731413	0.360002662	0.171183281	2.10302467	0.035463613	0.413724136
ING3	141.7350367	-0.420420687	0.199932148	-2.102816836	0.035481784	0.413724136
IGFBP7-AS1	2.648850962	3.242476468	1.542010718	2.10275871	0.035486867	0.413724136

EMC10	1053.636643	-0.317549877	0.151046879	-2.10232663	0.035524673	0.413724136
IFNAR2	162.0138222	0.55684683	0.264897406	2.102122624	0.035542535	0.413724136
CDC42BPG	17.59825128	-0.952651883	0.453191718	-2.102094643	0.035544986	0.413724136
PAK4	254.5117778	0.314361888	0.149640077	2.100786729	0.035659692	0.4147483
BTN2A3P	182.5494372	0.505450663	0.240626517	2.100560945	0.035679525	0.4147483
TRIR	641.6937232	-0.294592375	0.140273303	-2.100131438	0.03571728	0.414916517
GSPT2	101.656415	-0.522164238	0.248744482	-2.099199279	0.035799338	0.415176476
LOC100128568	9.446370063	1.853645536	0.883146682	2.09891015	0.035824822	0.415176476
ANXA2R	42.38885	0.63848359	0.304211894	2.098812051	0.035833472	0.415176476
EIF3D	2104.156078	-0.352149208	0.16779801	-2.09864949	0.03584781	0.415176476
DIPK2A	2535.458514	0.433616793	0.206626369	2.098554961	0.03585615	0.415176476
GLI4	238.6324555	0.399585926	0.190448013	2.098136487	0.03589309	0.41533433
S100A10	2208.231057	-0.331721782	0.158168042	-2.097274377	0.035969294	0.415739982
PRCC	678.508445	-0.243431661	0.116085705	-2.096999461	0.035993623	0.415739982
WRN	1707.459195	0.219066951	0.104477577	2.096784376	0.036012667	0.415739982
KEAP1	979.5958401	-0.26083761	0.124416291	-2.09649081	0.036038674	0.415739982
PCA3	13.57723734	1.945887915	0.928194879	2.096421731	0.036044796	0.415739982
GID8	960.8815686	-0.228490999	0.109006501	-2.096122677	0.03607131	0.415776676
ARHGEF9-IT1	2.162889447	3.595343568	1.715750054	2.095493781	0.036127121	0.416150803
VWA8	988.9415783	0.221340242	0.105656119	2.094911727	0.03617884	0.416477346
PCED1B	13.99283626	-1.02741207	0.490563166	-2.094352249	0.036228613	0.416781076
CLIC1	2710.711069	-0.254815782	0.121700134	-2.093800332	0.036277771	0.417077337
MDC1	1826.524386	0.237951332	0.113679777	2.09317205	0.036333799	0.417236359
ABI3BP	73.65395282	0.62691575	0.299540942	2.092921745	0.036356141	0.417236359
LRRTM3	79.21091745	0.779104647	0.372326294	2.092531898	0.036390961	0.417236359
ELK4	2453.580361	0.317071672	0.151534926	2.092399953	0.036402753	0.417236359
ATF6B	1258.472272	-0.256843317	0.122754469	-2.092333732	0.036408672	0.417236359
JADE2	258.6242126	-0.376273615	0.179897594	-2.091598927	0.036474409	0.417579845
DSC1	5.8231339	-2.024339309	0.967900348	-2.091474927	0.036485512	0.417579845
LGALS2	16.37579192	1.438137678	0.687763428	2.091035404	0.03652489	0.417762225
ZFPM1	95.16805279	0.813521664	0.389272065	2.089853696	0.036630944	0.418067458
CD2BP2	794.218441	-0.234415504	0.112172875	-2.089769952	0.03663847	0.418067458
POLK	550.0328686	0.23621727	0.113046072	2.08956636	0.036656771	0.418067458
DCST1	12.62619861	1.289003907	0.616905068	2.089468825	0.036665541	0.418067458
VAPB	1270.392628	-0.222939573	0.106698665	-2.089431705	0.03666888	0.418067458
RFXAP	179.642048	-0.362085996	0.17333212	-2.088972292	0.036710217	0.418271147
DNAJC8	1046.400817	-0.264207511	0.126601772	-2.086917946	0.036895552	0.420114213
APAF1	1619.022115	0.221150559	0.106023822	2.085857253	0.036991555	0.420615657
PRDM8	25.18623063	-0.813937612	0.39027496	-2.085549153	0.037019481	0.420615657
LARP1B	256.5587328	0.383604955	0.183944964	2.085433304	0.037029986	0.420615657
KBTBD11	42.19512549	-0.749343968	0.359372286	-2.085146788	0.037055978	0.420615657
SCML1	350.8360382	0.269373219	0.129205685	2.084840294	0.0370838	0.420615657
COL9A2	593.9313424	0.708936163	0.340047825	2.084813108	0.037086268	0.420615657
MIR5692C2	3.305583874	3.237433565	1.553039745	2.084578695	0.03710756	0.420615657
TNXB	15.72059295	1.125712428	0.540078626	2.084349156	0.037128419	0.420615657
TLK1	671.707181	-0.248218973	0.119127247	-2.083645667	0.03719241	0.421072899
PCBP4	162.8834371	-0.47390938	0.227644124	-2.081799309	0.037360805	0.422710821
ASB9	9.920574742	-1.300191942	0.624763159	-2.081095728	0.037425145	0.423170102
LTBP2	724.9520776	-0.655212413	0.314944505	-2.080405923	0.037488316	0.423472139
BTF3L4	702.5242083	-0.415162697	0.199575608	-2.080227644	0.037504658	0.423472139
CCT3	5830.774982	-0.285014596	0.137024535	-2.080025995	0.037523149	0.423472139
RAX2	1.936490706	3.300743062	1.58715612	2.079658718	0.037556847	0.423584187
DDX49	595.008454	-0.272042841	0.130856756	-2.07893616	0.037623219	0.424064362
UBASH3B	100.6810971	-0.492113752	0.236804874	-2.0781403	0.037696439	0.424497985
BTBD3	567.0286033	-0.238186383	0.11462646	-2.077935435	0.037715306	0.424497985
CYP20A1	646.6248339	-0.39307213	0.189182383	-2.07774172	0.037733154	0.424497985
TUBB	13666.05356	0.241933192	0.116456794	2.077450214	0.037760026	0.424532277
NUP62	1180.456863	-0.273652021	0.131785914	-2.076489155	0.037848733	0.425261302
RELB	42.57735286	-0.887083655	0.427288006	-2.076078994	0.037886646	0.425419046
MSS51	153.5472789	0.861575867	0.41520355	2.075068645	0.037980173	0.426200684
SOCS4	1506.967439	0.22095889	0.106526652	2.074212282	0.0380596	0.426791731
MCM3	5406.17918	-0.226765414	0.109341822	-2.073912896	0.038087401	0.426791731
SNORA74D	53.28891609	0.805313284	0.388341076	2.073726767	0.038104694	0.426791731
ATXN7	447.8504115	0.2699287	0.130201356	2.073163505	0.038157066	0.427109867
PROM2	34.07762573	0.827191471	0.399074464	2.072774744	0.038193248	0.427246502
MASP1	34.93051921	-0.762651801	0.368125181	-2.071718644	0.038291688	0.428078967
FAM189B	993.2696178	0.306375845	0.147954015	2.070750466	0.038382121	0.428710369
SNORD4A	82.85514133	0.639601128	0.308923868	2.070416675	0.038413342	0.428710369
H3C4	2850.144466	-0.445990703	0.215474377	-2.069808529	0.038470278	0.428710369
NAB2	442.2049703	-0.467550046	0.225948232	-2.069279511	0.038519865	0.428710369
UBXN8	364.561447	-0.410522822	0.198396437	-2.069204612	0.03852689	0.428710369
SLC25A23	527.9934259	-0.265526645	0.128343466	-2.068875449	0.038557776	0.428710369
LOC100288203	108.9276676	0.454743165	0.219816427	2.068740594	0.038570436	0.428710369
EIF3M	1496.392487	-0.260240003	0.125807204	-2.068562018	0.038587205	0.428710369
MATR3	6236.773243	-0.245198912	0.118537268	-2.068538573	0.038589407	0.428710369
TOP2B	2115.776417	-0.334041993	0.161500193	-2.068369002	0.038605338	0.428710369
AGO1	1468.703325	-0.203309435	0.098298342	-2.068289563	0.038612803	0.428710369
MIR1256	2.83913329	2.958138073	1.430906088	2.067318113	0.038704189	0.428764088
COG4	1105.898262	-0.245984023	0.118987375	-2.067311948	0.038704769	0.428764088
H3C1	259.721939	-0.434235937	0.210051968	-2.067278592	0.03870791	0.428764088
RAD21-AS1	15.80026006	1.352128661	0.65408221	2.067215161	0.038713884	0.428764088
LOC105747689	22.50515589	1.161937953	0.56261582	2.065242232	0.038900086	0.430522304
GDF5	5.869560949	1.899398515	0.919899676	2.064788763	0.038942992	0.430522304
MILR1	21.16836301	-0.842786311	0.408175153	-2.064766329	0.038945115	0.430522304
NYAP1	23.39174489	1.09449115	0.530275288	2.064005572	0.039017188	0.430810472
PCDH9-AS3	4.054570606	3.693752378	1.789625335	2.063980826	0.039019534	0.430810472
FBLN5	257.511706	-0.427674077	0.2072541	-2.063525288	0.039062748	0.430973152
PPP4R3B	1734.954787	-0.291862568	0.141468453	-2.063092947	0.039103798	0.430973152
ZNF367	405.7563451	-0.285607675	0.138438786	-2.063061105	0.039106823	0.430973152
CHRFAM7A	35.5640672	0.780756035	0.378693759	2.061708216	0.039235524	0.43199454

FAM9C	586.6767834	-0.46041343	0.223357686	-2.061327904	0.039271768	0.43199454
SNX12	682.600214	-0.315315836	0.152992427	-2.060989831	0.03930401	0.43199454
TSPAN18	2521.272895	0.980761681	0.475884998	2.06092162	0.039310518	0.43199454
PPFIBP1	1525.13772	-0.298549855	0.144869815	-2.060814767	0.039320715	0.43199454
CLK3	653.3951856	0.224429401	0.108947608	2.059975478	0.039400885	0.43260861
SLC37A2	173.5656003	0.859523149	0.41770143	2.057745284	0.039614591	0.434687198
MTMR4	1898.789964	0.19223113	0.093432996	2.057422312	0.039645621	0.434759977
SIK3	617.3051241	-0.277985372	0.135149364	-2.05687518	0.039698234	0.435069207
NUTM2B	3.908222495	2.742974285	1.333873867	2.056397049	0.039744261	0.43507063
MAFG	754.5454195	0.295995084	0.143940815	2.056366598	0.039747193	0.43507063
RNF213-AS1	33.92559992	0.675559709	0.328621048	2.055740841	0.039807503	0.435463293
NDC80	424.3171947	-0.310854341	0.151267813	-2.054993289	0.039879653	0.43559778
MSH5-SAPCD1	38.63029762	0.964728497	0.469477625	2.054897709	0.039888886	0.43559778
SF3A3	1248.593723	-0.216649496	0.105440221	-2.054713984	0.039906638	0.43559778
C8orf34-AS1	4.975921054	-1.95919376	0.953902453	-2.053872232	0.03998806	0.43559778
NPAS2	381.686967	-0.308313796	0.150124204	-2.053724775	0.040002337	0.43559778
TSPOAP1-AS1	55.50811926	0.680817187	0.331522516	2.053607687	0.040013678	0.43559778
SNHG32	974.4502364	-0.272080806	0.132492767	-2.053552143	0.040019058	0.43559778
SLC35E2A	896.6333546	-0.24095475	0.11733752	-2.053518345	0.040022332	0.43559778
PCSK7	393.6085414	0.311683538	0.151793577	2.053338123	0.040039796	0.43559778
MYH10	2035.220307	-0.218645634	0.10657158	-2.05163172	0.040205467	0.436517967
CORIN	7.343197658	1.551958134	0.756492306	2.051518728	0.040216458	0.436517967
FRMD6-AS1	86.86166085	0.657820045	0.320672434	2.051376966	0.04023025	0.436517967
CSRNP2	343.4207319	0.300181436	0.146355315	2.051045676	0.040262499	0.436517967
PTP4A1	8946.821123	-0.248064172	0.120949188	-2.0509784	0.04026905	0.436517967
AHSA2P	1309.282401	0.494326368	0.241022563	2.05095474	0.040271355	0.436517967
NT5C3B	433.4925788	-0.272196537	0.132786692	-2.049878137	0.040376324	0.437389724
MDH1B	2.955246831	-2.477589547	1.209398318	-2.048613356	0.040499938	0.438462265
PAG1	184.7900778	0.447377795	0.218493742	2.047554271	0.040603694	0.439260167
MCC	562.061212	0.342295712	0.167189589	2.047350644	0.040623669	0.439260167
PGPEP1	32.98184389	-1.020283195	0.498402495	-2.047106918	0.040647588	0.439260167
PLEKHM3	433.0120226	0.492318303	0.240593424	2.046266668	0.040730141	0.439570813
JUP	20.46628283	-1.132566368	0.553612659	-2.04577397	0.040778614	0.439570813
LDLRAD2	27.98600846	0.859079552	0.419965426	2.045595897	0.040796146	0.439570813
INHBC	14.49462169	-1.223710077	0.598227081	-2.045561153	0.040799567	0.439570813
VHL	1050.523321	-0.229875116	0.112377591	-2.045560102	0.04079967	0.439570813
TMEM104	422.5991275	0.238407781	0.116577324	2.045061363	0.040848809	0.439834304
ABHD17A	553.2728818	-0.213596883	0.104484661	-2.044289376	0.040924968	0.440291842
FMR1	743.4222916	-0.474451514	0.232104387	-2.044129887	0.040940717	0.440291842
MTFR1	1216.297504	0.230682128	0.112919517	2.042889787	0.04106335	0.441108841
GPBP1	1243.852283	-0.227673431	0.111448318	-2.042861066	0.041066193	0.441108841
GSPT1	3335.613546	-0.241709892	0.118334309	-2.04260196	0.041091857	0.441118609
SERTAD2	1234.758293	0.368936759	0.180646574	2.042312515	0.041120541	0.441160776
NFIB	846.795812	-0.485614828	0.237814677	-2.041988463	0.041152676	0.44123988
LINC01359	2.507344907	3.138531799	1.537400303	2.041453871	0.041205735	0.441440025
ADAMTS3	230.1453323	0.325570608	0.159496279	2.041242654	0.041226714	0.441440025
BAG1	173.578788	-0.391810863	0.191965155	-2.041051998	0.041245659	0.441440025
SLC26A6	299.8417092	0.446263153	0.218743757	2.040118353	0.041338539	0.441477657
ZFR	1635.625868	-0.253469288	0.124250038	-2.039993653	0.041350958	0.441477657
AVL9	1607.182059	-0.204915374	0.100456358	-2.039844749	0.041365791	0.441477657
PLXNA2	1245.387117	0.648362592	0.317856226	2.039798309	0.041370418	0.441477657
MTERF2	162.2683343	0.429948031	0.210782404	2.039771934	0.041373046	0.441477657
DTX2	95.56638095	0.435433266	0.213541773	2.039101108	0.041439938	0.441575207
HES7	26.74933916	1.026826126	0.503577829	2.039061425	0.041443897	0.441575207
PRPF19	1380.654515	-0.23271508	0.114135612	-2.038934877	0.041456528	0.441575207
MEF2A	1201.921453	-0.222635445	0.109228562	-2.038253003	0.041524637	0.442036461
CTHRC1	2241.122601	-0.354436288	0.173931548	-2.037791837	0.041570755	0.442147768
MED14	2089.17227	0.195529354	0.095958154	2.037652305	0.041584717	0.442147768
SMAD1-AS2	5.797960935	2.721503261	1.335933122	2.037155316	0.04163448	0.442412901
PSENEN	120.8285698	-0.453545398	0.222700138	-2.036574389	0.041692711	0.442567482
GAS5-AS1	85.82376946	0.551788591	0.270989776	2.036197085	0.041730569	0.442567482
ENY2	466.5082449	-0.443568254	0.21785583	-2.036063275	0.041744002	0.442567482
SAE1	2613.276236	-0.272782765	0.133978445	-2.03601978	0.041748369	0.442567482
ATP6V0D1	380.4249931	-0.339105003	0.166593223	-2.035526994	0.041797875	0.442828857
INSC	220.2332162	1.516513934	0.74512605	2.035244822	0.041826245	0.442866124
SIN3A	1918.787321	-0.220722499	0.10848197	-2.034646848	0.04188642	0.442931838
CEMIP2	227.2787467	0.444564859	0.21852351	2.034402888	0.041910991	0.442931838
SLC22A5	158.1251719	0.36130876	0.177610724	2.034273335	0.041924044	0.442931838
TRIM52-AS1	30.87079768	0.864708598	0.425086253	2.034195631	0.041931875	0.442931838
MED27	319.8441213	0.259942629	0.127843555	2.033286937	0.042023542	0.44354207
LOC101929130	18.6387578	-1.061438157	0.522117687	-2.032948095	0.042057767	0.44354207
HTRA3	599.2062638	-0.804359997	0.395674459	-2.032883293	0.042064315	0.44354207
EFCAB12	3.739645116	2.481804937	1.221393104	2.031946086	0.042159114	0.444233544
LCAT	87.9500533	0.737776162	0.363124871	2.031742306	0.042179751	0.444233544
STK26	900.3981663	0.259915685	0.12794533	2.031458938	0.042208461	0.444273345
ANP32A	1817.530944	-0.275816341	0.135828139	-2.030627401	0.042292806	0.444895328
H2AC17	1739.330524	-0.388373042	0.191280552	-2.030384362	0.042317485	0.444895328
PRSS53	99.24701946	0.615687814	0.303303723	2.029938199	0.042362822	0.445102008
MLEC	8451.97865	0.224298041	0.110508007	2.029699447	0.042387099	0.445102008
CAPN1	1056.871913	-0.257650359	0.126958291	-2.029409469	0.042416601	0.445149492
FSTL3	85.80840569	0.512736092	0.252709311	2.028956077	0.042462764	0.445371665
DBI	120.8517496	0.403798069	0.199088857	2.028230379	0.042536741	0.445885129
FJX1	304.1390853	-0.427213151	0.210668144	-2.027896311	0.042570832	0.445980142
ULBP2	52.64475043	0.661761964	0.326455618	2.027111581	0.042651003	0.446466514
DTWD2	200.8301778	-0.389825282	0.192359656	-2.026543869	0.042709082	0.446466514
KITLG	1159.960503	0.404374852	0.19954398	2.026494865	0.042714098	0.446466514
ARID4B	647.9766947	-0.265851543	0.131206527	-2.026206692	0.042743608	0.446466514
RALGAPA1	787.183356	0.271184786	0.1338711	2.02571568	0.042793929	0.446466514
PTP4A2	5076.896443	-0.29701082	0.146636632	-2.025488549	0.042817223	0.446466514
CENPU	352.0549363	-0.514666502	0.254109896	-2.025369772	0.042829409	0.446466514

FBXO4	160.7918041	-0.519845597	0.256682601	-2.025246722	0.042842036	0.446466514
CDH11	9088.433711	-0.245338803	0.121143254	-2.025195743	0.042847269	0.446466514
EFCAB2	40.2476853	0.701656186	0.346497609	2.024995751	0.042867801	0.446466514
WAPL	1186.385349	-0.236730722	0.116925733	-2.024624646	0.042905922	0.446602524
PHB2	1810.068177	-0.263872317	0.13035025	-2.024333032	0.042935897	0.44665364
TLNRD1	1160.473312	0.268992351	0.132938135	2.02344009	0.043027794	0.447348481
ARHGEF17	638.3710648	0.293835785	0.145237992	2.023133075	0.043059429	0.447392699
PRDM15	227.3171143	0.352842576	0.174429921	2.022832862	0.043090382	0.447392699
OXCT1	811.3580874	-0.242004167	0.119648468	-2.022626539	0.043111666	0.447392699
USP38	486.4369568	0.239662866	0.118502725	2.022424924	0.043132472	0.447392699
MMP11	54.5246755	-0.602969056	0.298364819	-2.020912043	0.043288871	0.448753741
DPRXP4	13.83972695	1.596163929	0.790025588	2.020395229	0.043342408	0.448872679
PPP1R15A	211.591368	-0.477963838	0.236578868	-2.02031501	0.043350723	0.448872679
EMP2	501.5849797	-0.347546922	0.17207634	-2.019725206	0.043411899	0.448899916
VGLL3	771.2246913	0.405430597	0.200748358	2.019596079	0.043425302	0.448899916
SIMC1	153.1777727	0.418147184	0.207048536	2.019561176	0.043428926	0.448899916
SQSTM1	1706.008506	-0.34028145	0.16854548	-2.018929542	0.043494543	0.449317542
LRP12	1098.74582	0.227042375	0.112489716	2.018338938	0.043555974	0.449691459
WHAMM	153.3124856	0.432811263	0.214488954	2.017872044	0.043604589	0.44986845
XIST	3574.246742	0.61750324	0.306045926	2.017681622	0.04362443	0.44986845
FBXL8	164.5274422	0.620255312	0.307445604	2.017447329	0.043648852	0.44986845
EIF3A	3365.655387	-0.193578878	0.095968405	-2.017110516	0.043683981	0.44997026
MMP16	1757.340069	0.205834808	0.102072317	2.016558603	0.043741597	0.450213307
H1-4	3360.085748	-0.290365224	0.144018615	-2.016164543	0.043782773	0.450213307
SPTBN1	5393.801135	-0.178380725	0.088476514	-2.016136461	0.043785708	0.450213307
PRKCSH	3666.068501	-0.24585876	0.121982554	-2.015523961	0.043849778	0.450213307
POLR1D	469.3283438	-0.365309696	0.181259158	-2.015399933	0.043862761	0.450213307
HK2	1121.468612	0.407918974	0.202448128	2.014930831	0.043911897	0.450213307
ASB3	52.63484195	0.560188419	0.278084613	2.014453127	0.043961981	0.450213307
INO80B	44.57473304	-0.827387524	0.410764565	-2.014262171	0.043982015	0.450213307
PLPP4	20.55491174	1.144597976	0.568343711	2.01391861	0.044018079	0.450213307
LOC101927021	76.76005896	-0.581328404	0.288665056	-2.013850972	0.044025182	0.450213307
HEXA	1661.591851	-0.240231853	0.119312857	-2.013461581	0.044066093	0.450213307
TNPO2	3069.589508	0.305709393	0.151845752	2.013289066	0.044084228	0.450213307
TMEM129	471.9028035	-0.281198775	0.139672411	-2.013273577	0.044085857	0.450213307
LOC100129203	12.62677246	1.397570071	0.694247262	2.013072499	0.044107003	0.450213307
HTR7	79.36817899	-0.490668441	0.243741217	-2.013071269	0.044107132	0.450213307
PSMG3	484.9346661	-0.238495674	0.118479373	-2.012972122	0.044117562	0.450213307
TRAM2-AS1	111.0103621	-0.497569427	0.24721552	-2.012694945	0.044146732	0.450213307
KCNJ2	71.54834464	0.768071068	0.381641362	2.012546711	0.044162338	0.450213307
PCLAF	865.4185687	-0.322573017	0.160311063	-2.012169413	0.044202082	0.450222417
LOC101928021	3.675856339	2.635479329	1.309856567	2.012036581	0.044216081	0.450222417
SLC9A3	149.2622368	0.392051263	0.194874029	2.011818941	0.044239027	0.450222417
ZNF808	361.3606292	0.308989512	0.153643821	2.011076718	0.044317354	0.450423055
TBC1D25	228.1917007	0.303432726	0.150883379	2.011041436	0.04432108	0.450423055
SET	10736.90827	-0.258099421	0.128351731	-2.010876039	0.044338552	0.450423055
CCSAP	336.1430403	0.268368143	0.133471698	2.010674528	0.044359846	0.450423055
MIIP	307.1207985	0.263557796	0.131133219	2.009847684	0.044447312	0.450725053
GPR155	154.0941808	0.602512584	0.299813475	2.009624767	0.044470918	0.450725053
RNF103-CHMP3	7.756556519	1.716898841	0.854404196	2.009469112	0.044487407	0.450725053
MED21	320.5878979	-0.269911274	0.134342318	-2.009130697	0.044523275	0.450725053
PSMC2	1242.723229	-0.267521047	0.133162707	-2.008978732	0.044539389	0.450725053
CEP250	642.1553947	0.223791934	0.111402241	2.008863841	0.044551576	0.450725053
GABPB2	129.2036309	-0.415558605	0.206877128	-2.008721841	0.044566641	0.450725053
CLCC1	317.76307	0.251798457	0.125377873	2.008316544	0.044609665	0.450904271
C1QTNF3	216.3019316	0.482539192	0.240352847	2.00762836	0.044682799	0.451168886
UGCG	741.1685641	0.378344303	0.188463003	2.007525598	0.044693728	0.451168886
RN7SK	47961.35004	-0.575691966	0.286791203	-2.007355733	0.044711799	0.451168886
NIPSNAP3A	319.4895072	-0.380089904	0.189420939	-2.006588636	0.044793482	0.451571157
POLR3G	280.2080947	0.436784372	0.217687842	2.006471139	0.044806005	0.451571157
LANCL2	112.8275021	-0.545134828	0.271715888	-2.006267771	0.044827687	0.451571157
SLC17A5	413.5994903	0.542597126	0.270634273	2.004909136	0.044972762	0.452585408
NOVA1	425.8585753	-0.711271101	0.354775356	-2.004849236	0.044979167	0.452585408
ALG10B	477.6944564	0.241619131	0.12053478	2.004559438	0.045010167	0.452599243
SGF29	102.9985049	-0.467773733	0.233377915	-2.004361607	0.045031339	0.452599243
ELOB	1040.261593	-0.299789757	0.149637993	-2.003433422	0.045130787	0.452770458
OSBPL7	301.208083	0.437667287	0.218468726	2.003340682	0.045140734	0.452770458
UTP15	387.7003758	0.339430614	0.169438894	2.003262684	0.045149101	0.452770458
CEP41	358.9512925	-0.283004978	0.141273874	-2.003236483	0.045151912	0.452770458
DDX1	1995.587212	-0.237158594	0.118401851	-2.002997358	0.045177572	0.452770458
REEP5	804.1832147	-0.278486805	0.139059338	-2.00264728	0.045215161	0.452770458
CCDC200	39.02588201	0.950628408	0.474710315	2.002544242	0.04522623	0.452770458
TMEM91	71.65404578	0.656318596	0.327810381	2.002128775	0.045270883	0.452963023
OVOS	46.67569257	0.710439947	0.355049364	2.000961046	0.045396588	0.453311466
SNX1	1654.057025	-0.205396271	0.102656215	-2.000816712	0.045412146	0.453311466
SPATA6	41.33262119	-0.687583658	0.343677961	-2.000662643	0.045428758	0.453311466
SYT2	18.52884553	1.24435006	0.622000565	2.000560979	0.045439722	0.453311466
WDR24	457.6905762	0.276165531	0.138068021	2.000213577	0.045477206	0.453311466
FOXD1	264.313033	-0.44817002	0.224116821	-1.999716122	0.045530926	0.453311466
MIS18BP1	947.4540016	-0.19072099	0.095400431	-1.999162783	0.045590744	0.453311466
SPATA20	890.4822202	-0.238489291	0.119295285	-1.999151027	0.045592016	0.453311466
STPG1	152.7832597	0.540788135	0.270510528	1.999138955	0.045593321	0.453311466
SLX4	546.1087095	0.232365607	0.116235139	1.9990995	0.045597589	0.453311466
VPS37A	691.5540802	0.264358634	0.132242194	1.999049061	0.045603046	0.453311466
FKBP1A	2511.255174	-0.22255037	0.111346388	-1.998721051	0.045638544	0.453311466
SEC14L1	1578.842373	0.248588464	0.124376328	1.998679871	0.045643002	0.453311466
MEIS1	438.3408514	-0.602121624	0.301285894	-1.99850586	0.045661845	0.453311466
B9D2	68.97016274	0.664386356	0.332626868	1.997392331	0.04578258	0.454257005
GDAP1L1	3.261614697	-2.189703818	1.096846701	-1.996362678	0.04589446	0.454943985
CHPF2	1653.385934	0.283290028	0.141934017	1.995927644	0.045941799	0.454943985

CILP2	109.7571428	0.708979871	0.355263338	1.995646034	0.045972465	0.454943985
NLRP1	132.9472925	0.491840144	0.246459629	1.995621543	0.045975133	0.454943985
BBC3	78.22520723	-0.784547661	0.393142332	-1.995581748	0.045979468	0.454943985
HSD17B7	201.3340519	0.426620622	0.213872534	1.994742441	0.046070978	0.455431931
KCNJ8	58.52027663	0.67266251	0.337373751	1.993819932	0.046171737	0.455431931
FAM98A	1310.29314	0.22585366	0.113283534	1.993702457	0.046184581	0.455431931
CDK10	536.6728293	0.422355258	0.211938778	1.992817271	0.04628146	0.455431931
FAM216B	40.25076912	-0.903513245	0.453412879	-1.992694267	0.046294936	0.455431931
TVP23B	767.1134292	0.361930731	0.181636103	1.992614489	0.046303677	0.455431931
ABHD10	393.2809146	0.26107436	0.131024715	1.99255812	0.046309855	0.455431931
CKM	4.11737745	-2.114462296	1.061317178	-1.992300078	0.046338143	0.455431931
GAN	383.3354581	0.362751488	0.182091872	1.992134432	0.04635631	0.455431931
LOC101927793	5.743824549	2.774637518	1.392869184	1.992030227	0.046367741	0.455431931
AKAP3	8.929427358	-1.43667409	0.721388078	-1.991541214	0.046421419	0.455431931
JOSD2	169.7082568	-0.410519293	0.206132502	-1.991531123	0.046422527	0.455431931
ZNF431	254.8609396	0.495251407	0.248694899	1.991401551	0.046436759	0.455431931
LINC01426	37.82681691	-0.679054364	0.341013413	-1.991283446	0.046449734	0.455431931
HSPA4L	223.48373	0.554222049	0.278332165	1.991225302	0.046456123	0.455431931
LOC653160	37.36846195	0.78001947	0.391733067	1.991201497	0.046458739	0.455431931
CYGB	89.21983663	-1.224007009	0.614720451	-1.991160384	0.046463258	0.455431931
SRRM1	2057.550533	0.302117974	0.15177667	1.990542915	0.046531162	0.455795968
DDOST	4885.286369	-0.279990611	0.140673518	-1.990357643	0.046551552	0.455795968
EHBP1	3598.250835	0.276561057	0.138990446	1.989784658	0.046614662	0.456163251
DHCR7	1115.676299	0.42759246	0.215040402	1.988428486	0.046764321	0.457376619
MED12	867.6238092	-0.264846125	0.133211451	-1.988163358	0.046793626	0.457412186
GLB1L	160.5877372	-0.352753646	0.177511149	-1.987219659	0.04689806	0.457788011
FMN1	3066.84487	-0.39539099	0.19897414	-1.98714763	0.046906039	0.457788011
CUL4A	1652.928418	-0.261433271	0.131563932	-1.987119626	0.046909142	0.457788011
POLR3C	518.3417312	-0.265809248	0.133792328	-1.986730115	0.046952313	0.457958518
SCARNA4	7.921545269	1.796666137	0.904941666	1.985394423	0.047100606	0.458464307
PXK	451.5623774	-0.507639671	0.255714251	-1.985183341	0.047124077	0.458464307
FRMD8	539.0418836	-0.292395802	0.147290469	-1.985164438	0.047126179	0.458464307
PLB1	123.7056033	0.847863133	0.427108888	1.985121728	0.04713093	0.458464307
RIPOR1	727.1867615	0.215350282	0.108483076	1.985104858	0.047132806	0.458464307
HOXB7	170.7060084	-0.451965579	0.227753768	-1.984448304	0.047205886	0.45892465
SMAP1	129.3466519	-0.454618282	0.229140307	-1.984017073	0.047253937	0.459141304
TRIL	14.96997886	-1.199047603	0.604575174	-1.983289513	0.0473351	0.459508479
FRAS1	269.6074543	0.585982845	0.295471003	1.983216086	0.047343298	0.459508479
H2BC4	2856.524818	-0.333750259	0.168330955	-1.982702824	0.047400634	0.459814534
MEX3B	1163.41249	0.423604637	0.213693511	1.982299951	0.047445679	0.460001093
ADAMTS7P1	2.038255888	3.326944633	1.678675186	1.981887062	0.047491882	0.460198663
BAZ1B	2017.837675	-0.245960892	0.124142063	-1.981285685	0.047559245	0.460600947
UHRF2	727.346924	-0.427147499	0.215713721	-1.980159153	0.047685648	0.46071923
EMC3-AS1	70.12072858	0.65870107	0.332675546	1.980010486	0.04770235	0.46071923
VPS37B	652.110094	-0.311481528	0.157316635	-1.979965613	0.047707393	0.46071923
PRTFDC1	111.7334635	-0.430091769	0.217223185	-1.979953335	0.047708773	0.46071923
ELFN1	43.98839451	-1.420313664	0.717377017	-1.979870598	0.047718071	0.46071923
PILRB	20.24473524	1.252848001	0.632817075	1.979794876	0.047726582	0.46071923
RAB6B	16.33979944	-1.048953933	0.530111085	-1.978743631	0.047844878	0.461611112
LINC01011	12.09904806	1.262422144	0.638221223	1.978032223	0.047925072	0.462134619
LBX2-AS1	73.08032405	0.581940304	0.2942825	1.977488653	0.047986422	0.462475953
TNKS	4567.766614	-0.253413791	0.128258154	-1.975810371	0.048176258	0.463819674
CTNNA1	3197.856748	-0.253471426	0.128288266	-1.975795863	0.048177902	0.463819674
CCDC34	277.7692899	0.342101686	0.173245462	1.974664623	0.04830622	0.464803911
BMPR1B	346.7585785	-0.267543394	0.135533919	-1.973995849	0.048382215	0.465283902
UBE2O	1631.870179	0.1704191	0.086355164	1.973467376	0.048442338	0.4654042
SNORD156	24.77500321	1.593654309	0.807556843	1.973426789	0.048446958	0.4654042
COLEC12	915.4704131	0.453439491	0.229862516	1.972655219	0.048534857	0.46599739
RAD52	349.2595448	0.458942884	0.232785102	1.971530312	0.048663249	0.466702367
PRTG	642.4402839	0.242270277	0.122937368	1.970680523	0.04876043	0.466702367
DPY19L1P1	77.00431836	0.512379225	0.260027487	1.970481005	0.04878327	0.466702367
C18orf54	650.215607	0.205020713	0.104065347	1.970115125	0.048825178	0.466702367
LOC100303749	8.340751448	1.551360161	0.787551729	1.969851763	0.048855362	0.466702367
HMGB2	557.5420962	-0.263175424	0.133602821	-1.969834338	0.04885736	0.466702367
JKAMP	614.9399482	0.214748627	0.109018955	1.969828339	0.048858048	0.466702367
FNIP2	309.845318	0.33683973	0.171064096	1.96908491	0.048943341	0.466702367
FBXO45	1132.6851	0.28867228	0.146608072	1.96900673	0.048952318	0.466702367
ZNF8-ERVK3-1	110.568078	0.708333834	0.359756133	1.968927753	0.048961388	0.466702367
TSKU	585.0278953	-0.37674193	0.191381453	-1.968539401	0.049006007	0.466702367
FAM122B	387.5776581	0.361381588	0.183579308	1.968531161	0.049006954	0.466702367
PPIB	2452.524217	-0.253920093	0.129008758	-1.96823919	0.049040523	0.466702367
DDN	291.6319719	0.450618388	0.228991581	1.967838235	0.049086653	0.466702367
RABL2A	72.42197481	-0.465075899	0.236352569	-1.967720938	0.049100155	0.466702367
BUB3	2198.349694	-0.194214763	0.09871148	-1.967499245	0.049125683	0.466702367
LINC00515	4.341020091	2.630930207	1.337239526	1.967433774	0.049133224	0.466702367
CBL	1219.268425	0.285389043	0.145062454	1.967352931	0.049142537	0.466702367
CSNK2A1	2738.970986	-0.268048624	0.136260773	-1.967173803	0.049163177	0.466702367
SLC16A6	10.20545983	-1.501827948	0.763489938	-1.967056635	0.049176682	0.466702367
FAM201B	3.559734791	2.749276579	1.397705267	1.966993074	0.049184009	0.466702367
ERV3-1	36.96056868	0.743652078	0.378078799	1.966923509	0.04919203	0.466702367
POFUT2	1646.063563	0.301599342	0.153348201	1.96676153	0.04921071	0.466702367
VMP1	4787.271716	0.356348935	0.181206501	1.966535049	0.049236838	0.466702367
HINT3	347.8974475	-0.30787272	0.156575985	-1.966283144	0.049265913	0.466729701
HPF1	206.8322952	-0.335352966	0.17058289	-1.96592381	0.049307412	0.466800253
SP3	1640.875804	-0.361496484	0.183896074	-1.965765103	0.049325751	0.466800253
SHISA2	166.8735999	-0.957520106	0.487204543	-1.965334927	0.049375486	0.467022905
CDK14	387.9238499	-0.293348351	0.149279428	-1.965095622	0.049403171	0.467036876
TPCN1	2075.942784	-0.324637282	0.165271473	-1.964266884	0.04949915	0.467470942
HP1BP3	2904.109401	-0.176010307	0.089607052	-1.964246156	0.049501553	0.467470942
DUSP23	98.43208889	-0.549363753	0.279776001	-1.963584262	0.049578324	0.467947952

NACC2	48.36779202	-0.658994201	0.335691423	-1.963095142	0.04963512	0.467976338
CDR2	553.9391974	0.423730747	0.21586301	1.962961353	0.049650665	0.467976338
SGMS1-AS1	27.34809582	-0.864650055	0.44050744	-1.962850059	0.049663599	0.467976338
DOLPP1	419.984227	0.238944407	0.12174555	1.962654128	0.049686377	0.467976338
LOC101928389	4.655342301	3.377561392	1.721585861	1.961889597	0.049775339	0.468491596
PRR22	18.57091286	-1.279838637	0.652402298	-1.961732264	0.049793663	0.468491596
RASAL2	1299.509939	0.2823349	0.143951403	1.961320936	0.049841597	0.468695119
MTARC1	78.57221093	-0.578588076	0.295100433	-1.960648009	0.049920098	0.46918573
ZBTB2	411.1045426	-0.373570226	0.190620352	-1.959760442	0.050023797	0.469766392
H4C1	574.3682688	-0.426862223	0.21782374	-1.959668047	0.050034602	0.469766392
ASB16	44.34562808	0.948156983	0.483966112	1.959139205	0.050096487	0.469803594
TRAPPC2	453.6566851	-0.290318866	0.148216581	-1.958747552	0.050142359	0.469803594
TRPV2	1196.636605	-0.313316534	0.159989403	-1.958358043	0.050188014	0.469803594
ITPA	548.7894657	-0.284414179	0.145233591	-1.958322293	0.050192207	0.469803594
KIZ-AS1	15.91613018	1.24889375	0.637739919	1.958312022	0.050193411	0.469803594
PDE6A	9.423185602	1.541840901	0.78734302	1.95828357	0.050196748	0.469803594
CBLB	1657.665629	-0.241301379	0.123255828	-1.957727938	0.050261945	0.469978251
MYNN	1129.611107	0.191801535	0.09797569	1.957644136	0.050271785	0.469978251
ARL10	2283.554205	-0.162667126	0.083101529	-1.957450467	0.05029453	0.469978251
PTGDR2	5.686485242	2.000833791	1.022294112	1.957199762	0.050323987	0.470007047
DCDC2B	45.07245734	0.737485095	0.376954837	1.956428257	0.050414726	0.470184249
BMS1P14	112.7757614	0.440240486	0.225026975	1.956389832	0.050419249	0.470184249
GS1-124K5.4	24.4605525	0.775686202	0.39651836	1.956242839	0.050436554	0.470184249
SMS	991.2391744	-0.232649615	0.118932924	-1.956141388	0.050448501	0.470184249
SNORA73A	219.2814544	0.686523029	0.351079865	1.955461131	0.050528667	0.470562591
TSPYL5	126.9840308	-0.412290968	0.210854089	-1.955337788	0.050543214	0.470562591
SNORA71C	63.57222529	0.878386547	0.449273848	1.955125032	0.050568314	0.470562591
FOXO4	117.4269618	-0.592777301	0.303269195	-1.954624181	0.050627445	0.470866947
LOC728715	37.94769535	0.703734479	0.360082388	1.954370728	0.05065739	0.470899681
PREX1	109.1879139	-0.993946955	0.50874343	-1.953729317	0.050733237	0.471309801
LINC00824	4.411122359	-1.836074479	0.939865403	-1.953550448	0.050754406	0.471309801
H1-2	4683.058364	-0.34544579	0.176888016	-1.952906691	0.050830653	0.471640335
PLCL1	16.0826381	-1.306365474	0.668969386	-1.952803076	0.050842934	0.471640335
SCARF2	1412.475989	0.218398587	0.111885498	1.951982974	0.050940226	0.472169753
CMKLR1	88.8109056	-1.10262298	0.564904386	-1.951875408	0.050952999	0.472169753
CHL1	1.888004663	3.32937916	1.706065094	1.951495972	0.050998075	0.472341837
SGCD	406.5706216	-0.283100558	0.145112602	-1.950902632	0.051068629	0.472749599
RNF220	950.6831821	-0.229031605	0.117426035	-1.950432932	0.05112454	0.472879412
CDK11A	111.4428096	0.576289769	0.295481844	1.950339015	0.051135725	0.472879412
PCDHGC5	139.6326437	0.837936177	0.429810766	1.949546739	0.051230167	0.473177588
GPNMB	1522.588944	-0.563090595	0.288836109	-1.949515928	0.051233842	0.473177588
HS6ST2	704.1071789	-0.441590121	0.226530768	-1.949360456	0.051252394	0.473177588
LOC100132356	38.53603506	0.920780577	0.472394322	1.94917791	0.051274182	0.473177588
CLTA	698.2457927	-0.268157946	0.137668994	-1.947845619	0.051433438	0.474097066
MIRLET7D	14.45125338	1.308112668	0.671655566	1.947594474	0.051463506	0.474097066
AOC3	24.81625196	1.301512441	0.668308433	1.947472718	0.051478087	0.474097066
UBE2L3	426.0830697	-0.314490566	0.161505843	-1.947239559	0.051506021	0.474097066
RSF1	498.8246576	-0.340980134	0.17511011	-1.94723271	0.051506842	0.474097066
MCCD1	6.672551444	1.6789359	0.862582689	1.946405744	0.05160602	0.474764729
CLASP2	1137.239776	0.219439707	0.112801346	1.945364255	0.051731154	0.475440532
PTPRR	22.28516319	1.148276769	0.590267369	1.945350242	0.051732839	0.475440532
MIR130B	5.01551447	1.751607099	0.900649148	1.944827353	0.051795761	0.475579685
ST6GALNAC3	128.6525734	0.491051966	0.252517822	1.944623003	0.051820369	0.475579685
EHD4	1054.70599	0.258521142	0.132964729	1.9442836	0.051861261	0.475579685
SGO2	795.4835208	0.224804287	0.115623599	1.944276846	0.051862075	0.475579685
MAP1LC3A	69.69838684	-0.498524711	0.256427403	-1.944116366	0.05188142	0.475579685
NEMP1	1453.504393	-0.162008328	0.083379922	-1.943013658	0.052014508	0.476554516
PDGFA	30.04446008	0.761963352	0.392200283	1.942791435	0.052041363	0.476555546
C12orf65	192.0446841	-0.311658267	0.160463325	-1.942239866	0.052108069	0.476921309
H2BC8	313.1164133	-0.482122172	0.248334694	-1.94142093	0.052207242	0.477338512
TBPL1	188.9072274	-0.351426761	0.181046368	-1.94108705	0.05224772	0.477338512
LAMP1	3515.943061	-0.282764577	0.1456781	-1.941023238	0.052255459	0.477338512
HEMK1	496.0921483	0.337531516	0.173897543	1.940979212	0.052260799	0.477338512
CCDC84	137.3421721	0.698380535	0.359888795	1.940545373	0.052313446	0.47757459
TSC22D3	765.1946544	-0.710530367	0.366209499	-1.940229211	0.05235184	0.477680384
FYTTD1	2155.267473	-0.327007429	0.168689104	-1.938521344	0.052559649	0.479316535
CPSF7	982.7119257	-0.218331133	0.112639726	-1.93831379	0.052584951	0.479316535
PHF21A	457.6706178	-0.272930295	0.140886745	-1.937231887	0.052717004	0.480274546
THBS4	23.52933283	-0.83795206	0.432764879	-1.936275564	0.05283396	0.480338964
BMF	544.3018504	1.097299457	0.566727415	1.93620324	0.052842814	0.480338964
COMMD1	188.6117079	-0.322507976	0.166577874	-1.936079317	0.052857987	0.480338964
RPL36AL	183.130331	0.39849188	0.205847734	1.9358575	0.052885156	0.480338964
PSIP1	424.4554419	-0.31859737	0.164615719	-1.935400651	0.05294115	0.480338964
ACOT7	246.1097036	-0.430284851	0.222366722	-1.935023581	0.052987402	0.480338964
DDIAS	269.7746759	0.338547483	0.174962562	1.934971001	0.052993854	0.480338964
PTPN9	1087.214844	-0.232088681	0.119956595	-1.934772173	0.053018259	0.480338964
CD4	93.70666517	0.710131344	0.367050168	1.934698323	0.053027326	0.480338964
SLC38A9	206.7516815	-0.399413943	0.206460092	-1.934581829	0.053041632	0.480338964
GMDS	346.3419825	-0.304307245	0.157301536	-1.93454719	0.053045886	0.480338964
FHL1	2178.420177	-0.268257496	0.138667777	-1.93453376	0.053047535	0.480338964
KHSRP	4681.271708	-0.246369218	0.127399224	-1.93383609	0.053133284	0.480476666
RPL23AP64	38.69455815	0.962632525	0.497826241	1.933671723	0.053153503	0.480476666
CCDC47	2074.373847	-0.283572408	0.146660003	-1.933536084	0.053170192	0.480476666
COQ8A	743.527998	-0.401314197	0.207554893	-1.93353282	0.053170594	0.480476666
TRMT12	334.0544504	0.256528885	0.132713265	1.932955875	0.053241634	0.480874766
MIR130A	2.99576357	-3.175445334	1.643234658	-1.93243571	0.05330575	0.481008228
LIG3	1353.838807	0.241375347	0.124909745	1.932398041	0.053310396	0.481008228
MSTO2P	178.2488664	0.57812298	0.299236002	1.931996738	0.053359908	0.481011498
ZNF33B	232.1045858	-0.498674256	0.258118639	-1.931957566	0.053364744	0.481011498
SCARNA9L	63.17059233	0.629060455	0.325830012	1.930640003	0.053527587	0.482039272

MICAL2	1746.031822	-0.488799675	0.25318572	-1.930597327	0.053532869	0.482039272
ZNF529	557.6754283	0.262419953	0.135977654	1.929875566	0.053622258	0.482600318
NR2E3	3.239978672	2.64872514	1.37310681	1.929001532	0.053730672	0.483151127
PLAC4	50.18986794	1.047358168	0.543023877	1.928751594	0.053761708	0.483151127
CCDC24	33.43858558	-0.663873348	0.344222313	-1.928618	0.053778303	0.483151127
OTUD4	1510.516653	0.211661478	0.109753978	1.928508485	0.05379191	0.483151127
HDAC10	326.2354288	0.401422033	0.208192072	1.928133136	0.053838568	0.483280135
AGAP6	28.94150318	1.036422113	0.537575413	1.927956691	0.053860513	0.483280135
GALNT10	2568.573987	0.557483696	0.289286413	1.927099481	0.053967233	0.483994011
HOXC8	180.4224756	-0.964450315	0.500603907	-1.926573687	0.05403278	0.484338103
CENPP	371.1518393	0.285076775	0.148005184	1.926126958	0.054088523	0.484516366
HERC2P2	2007.745792	0.482827531	0.250727048	1.925709792	0.054140621	0.484516366
CABLES1	422.6720658	-0.268541469	0.139470069	-1.92544158	0.054174138	0.484516366
H3C2	2696.973903	-0.338077253	0.175591928	-1.925357602	0.054184636	0.484516366
CD274	72.75120144	0.982946787	0.510535308	1.925325776	0.054188615	0.484516366
QSOX1	1600.286146	-0.365783805	0.190025184	-1.924922777	0.05423902	0.484723839
SLC25A13	386.7679363	0.491499843	0.255517497	1.923546721	0.054411425	0.485804082
LINC01291	15.08811169	1.094632679	0.569077033	1.923522855	0.054414419	0.485804082
ADAL	62.89207995	-0.504138048	0.262213994	-1.922620678	0.054527705	0.486571711
AASS	612.9439694	0.305589691	0.159001137	1.921934	0.054614063	0.486945741
NRIP2	92.25833637	0.669102184	0.348154737	1.921852878	0.054624273	0.486945741
CCND1	2816.033324	-0.232275917	0.120886026	-1.921445554	0.05467556	0.487159241
LIMD2	182.5908303	-0.394583623	0.205426803	-1.92079912	0.054757037	0.487310758
CRLF3	464.467413	0.228760657	0.11910325	1.92069198	0.054770551	0.487310758
CASP7	385.3112525	0.278229728	0.144861532	1.920659852	0.054774604	0.487310758
LRRFIP2	330.1495119	-0.397505515	0.206991164	-1.920398474	0.054807586	0.487360871
SNORD74	96.30939702	0.536226607	0.279344261	1.919590563	0.054909637	0.488024804
FUCA1	264.9109783	-0.558175135	0.290865772	-1.919012786	0.054982716	0.488430705
MPV17L2	77.73625594	0.492091471	0.256535771	1.918217758	0.055083406	0.488800376
ATP8B5P	2.937314438	-2.457603661	1.281580424	-1.917635144	0.055157291	0.488800376
CREBL2	276.0605556	-0.439908406	0.229420184	-1.917479092	0.055177095	0.488800376
C11orf24	364.5103693	0.256410984	0.133729397	1.917386824	0.055188807	0.488800376
OPLAH	51.31925167	-0.695042302	0.362529028	-1.917204547	0.055211951	0.488800376
CDKL5	298.272546	0.276339604	0.144144865	1.917096415	0.055225684	0.488800376
LNPK	1982.979347	0.243249732	0.126893301	1.916962753	0.055242664	0.488800376
PPP2R1B	687.3708481	0.209325074	0.109216093	1.916613827	0.05528701	0.488800376
RNPC3	181.52024	0.462224788	0.24118941	1.916438988	0.055309242	0.488800376
IL18BP	83.95820025	0.661410087	0.345175322	1.916156938	0.055345123	0.488800376
HELB	217.0754504	0.340603504	0.177761028	1.91607524	0.055355519	0.488800376
ITPR2	767.7711663	0.303947666	0.158664169	1.915666707	0.055407533	0.488800376
SPON2	36.15852746	1.10352466	0.576094988	1.915525534	0.055425516	0.488800376
RASSF8	871.9766893	-0.24830586	0.129662605	-1.91501521	0.055490564	0.488800376
SIX4	1002.44483	0.416557389	0.217522265	1.915010345	0.055491184	0.488800376
CSNK2A3	36.91624215	-1.277200792	0.667002693	-1.914836035	0.055513417	0.488800376
H4C3	1512.733183	-0.53874113	0.281393927	-1.914544269	0.055550648	0.488800376
MICAL3	993.8848677	0.409339725	0.213833317	1.914293487	0.055582666	0.488800376
LOC152048	37.49209483	0.801993532	0.418970883	1.914198728	0.055594768	0.488800376
SLC10A7	168.3208491	0.363419804	0.189863503	1.914110913	0.055605985	0.488800376
PPP2R5D	892.6919311	-0.208420959	0.108889914	-1.914052013	0.05561351	0.488800376
SYNGR1	321.9799475	-0.277734776	0.145111516	-1.91394028	0.055627787	0.488800376
NR1H3	30.60795115	0.833540182	0.435563339	1.913706015	0.05565773	0.488822449
GINS3	258.118135	-0.271745795	0.142114989	-1.912154354	0.055856399	0.490094995
ADAT1	595.6016794	0.204740545	0.107073768	1.912144767	0.055857628	0.490094995
LIN7C	1219.229074	0.188079767	0.098404975	1.911283116	0.055968208	0.490796683
CUL3	1555.1263	-0.329032415	0.172169794	-1.911092572	0.055992686	0.490796683
PQBP1	317.7056018	-0.35823985	0.187492899	-1.910684893	0.056045087	0.490797125
FIBP	734.1776527	-0.258078129	0.135076091	-1.910612951	0.056054339	0.490797125
DYNLT3	284.1996696	-0.280462822	0.146856316	-1.909777043	0.056161926	0.490797125
STAG2	3240.515674	-0.261794607	0.137083675	-1.909743132	0.056166295	0.490797125
HDGF	3682.17812	-0.24127637	0.126340634	-1.909728978	0.056168118	0.490797125
ALPL	1121.894175	1.530465321	0.801483451	1.909540762	0.056192369	0.490797125
SMARCB1	736.3677115	-0.23067444	0.12081149	-1.909375006	0.056213733	0.490797125
NR2F1-AS1	42.94040128	-0.566517577	0.296725279	-1.909232603	0.056232092	0.490797125
EMILIN2	411.644443	0.312811979	0.163847404	1.909166524	0.056240613	0.490797125
SLC36A4	588.4612854	0.235642581	0.123458978	1.908671077	0.056304537	0.490939141
TEX22	48.21681886	0.873502537	0.457663429	1.908613364	0.056311987	0.490939141
UPF3B	125.4317622	-0.36572805	0.191711806	-1.907697062	0.056430382	0.491609103
LRPPRC	6936.38656	-0.211941906	0.111104439	-1.907591705	0.056444008	0.491609103
DENND1B	358.6810567	0.259849276	0.136250763	1.907139978	0.056502464	0.491877821
TBC1D32	516.3224183	0.391672489	0.205498588	1.905961947	0.056655143	0.492966136
FLJ42351	21.85472055	0.934886732	0.490682313	1.90527905	0.056743807	0.493496654
RAN	8562.721972	-0.290068157	0.152273161	-1.90491979	0.056790498	0.493661796
LINC01852	15.24116131	-1.05053064	0.551787764	-1.903867225	0.056927479	0.494611248
CNOT2	1145.969486	-0.343288598	0.180339459	-1.903568974	0.056966343	0.494707713
PBX1	89.66923943	-1.961736989	1.030718532	-1.903271288	0.057005155	0.494803637
CHRAC1	498.0892578	-0.237149331	0.124615984	-1.903041041	0.05703519	0.494823316
POTEI	27.62917723	-1.609138709	0.845778092	-1.902554256	0.057098733	0.495133537
LINC01605	372.9088883	0.441420955	0.232046176	1.902297906	0.057132219	0.495145909
FBXW4	226.8889625	-0.320134416	0.168304184	-1.902117987	0.057155732	0.495145909
PLEKHH3	205.6282175	-0.397966448	0.209260485	-1.901775425	0.05720052	0.495246932
TMEM223	140.8897791	0.377658852	0.198600182	1.901603759	0.057222976	0.495246932
ZIC2	273.819813	0.450687001	0.237036463	1.901340394	0.057257441	0.495304663
PLA2G4B	22.43421273	0.923678511	0.4859246	1.900867978	0.057319307	0.495599251
NAP1L1	5826.466741	-0.267346145	0.140766553	-1.8992164	0.057536029	0.497231834
FERMT1	745.1183864	-0.345549426	0.181964229	-1.89899645	0.057564943	0.497240563
PHLDA1	1755.520976	-1.025452087	0.540183906	-1.898338835	0.057651461	0.497514165
RRP7BP	271.9616663	0.740940489	0.390323784	1.898271434	0.057660335	0.497514165
HPS5	611.7177301	0.209891498	0.110578667	1.898119257	0.057680374	0.497514165
CCNDBP1	252.6271429	-0.317329886	0.167229818	-1.897567607	0.057753065	0.497900153
TMEM159	139.230333	-0.489096517	0.257793727	-1.897239792	0.057796297	0.498031923

ODF3B	66.87485909	0.738488405	0.389355865	1.896692645	0.057868515	0.498158376
LARP4	1551.839152	0.247050123	0.130264532	1.896526395	0.057890473	0.498158376
BARD1	640.6025252	-0.210398997	0.110944857	-1.896428568	0.057903397	0.498158376
PDE4D	177.1426649	-0.409303015	0.215845037	-1.896281796	0.057922792	0.498158376
CGNL1	126.915751	0.47332739	0.249694339	1.89562724	0.058009353	0.498490131
BARX1-DT	13.41888252	1.374950061	0.725350253	1.89556708	0.058017314	0.498490131
RHOA	5230.278536	-0.249866345	0.131857448	-1.894973322	0.058095936	0.49887273
ADAMTS6	287.900993	0.304660956	0.160787238	1.894808074	0.058117833	0.49887273
PCDHGB3	16.93909853	-1.05689101	0.558001216	-1.894065784	0.058216279	0.499323364
SMYD3	407.5926663	-0.266120844	0.140516592	-1.893874875	0.05824162	0.499323364
VPS53	1015.385783	0.197234816	0.104166739	1.893452916	0.058297664	0.499323364
ACAN	78.49401559	1.657468545	0.875443727	1.893289648	0.058319361	0.499323364
RPL10	9445.106844	-0.275607536	0.145574729	-1.8932375	0.058326292	0.499323364
AGGF1	606.773337	-0.235082016	0.124175322	-1.89314602	0.058338454	0.499323364
TEF	150.7302428	-0.500188137	0.26424485	-1.892896448	0.058371642	0.499367573
DGUOK	599.7197815	-0.24689203	0.130446765	-1.892665032	0.058402429	0.499391216
VASH2	599.4694529	0.224918716	0.118868823	1.892159023	0.058469796	0.499727466
NOTCH2	6101.306363	0.146336456	0.07735935	1.89164536	0.058538248	0.500063419
LMAN1	8304.910605	0.292413913	0.154598318	1.891443042	0.058565228	0.500063419
MXD1	192.0748623	-0.385790476	0.204025619	-1.890892325	0.058638719	0.500251207
MIR4292	25.08256293	0.860321734	0.454990247	1.890857527	0.058643365	0.500251207
GNAI2	1553.014751	-0.262369862	0.13882171	-1.889977164	0.058761014	0.501014964
LINC01623	3.536081348	2.368391074	1.253407962	1.889561217	0.058816668	0.501125801
BAZ2A	1853.702497	0.204397963	0.108203828	1.889008615	0.058890674	0.501125801
ATMIN	687.266565	-0.282863987	0.149753027	-1.888869914	0.058909262	0.501125801
NOL4L	127.97023	0.519155925	0.274856237	1.888827157	0.058914993	0.501125801
CTR9	805.8748	-0.201285723	0.106576712	-1.888646389	0.058939227	0.501125801
INPP5K	412.2279479	-0.333682721	0.176680694	-1.888620165	0.058942743	0.501125801
PLAGL2	431.583872	0.241326496	0.127815856	1.888079488	0.05901528	0.50131616
HRAT92	6.268874481	1.919824517	1.016978041	1.887773815	0.059056322	0.50131616
A1BG-AS1	95.29381641	0.517455667	0.274124189	1.887668759	0.059070433	0.50131616
C1orf35	171.8635986	-0.410355213	0.217393496	-1.887614947	0.059077662	0.50131616
ELF2	480.5120103	-0.289430461	0.153377059	-1.887051841	0.059153353	0.501561615
ZC3H7B	1818.376912	-0.215973328	0.114454425	-1.886981012	0.05916288	0.501561615
WASHC5	2054.484219	-0.217077769	0.115067421	-1.88652675	0.059224007	0.501821096
ZFAND4	124.5534479	0.401508526	0.212865696	1.886205875	0.059267217	0.501821096
LINC02864	213.4635091	0.324004397	0.171783002	1.886126063	0.059277969	0.501821096
DOP1B	337.5948728	0.339469508	0.180053105	1.885385468	0.059377815	0.502427662
IQGAP1	5684.066313	-0.187303927	0.099362367	-1.885059027	0.059421869	0.502561797
TXNRD3	252.5304313	-0.296217052	0.157217307	-1.884124953	0.059548076	0.503183559
KCNJ14	111.760002	0.419185938	0.222486391	1.884096979	0.059551859	0.503183559
RAB13	741.7898541	-0.308203685	0.163688031	-1.882872449	0.059717656	0.504273587
FAM91A1	3510.167048	0.160252786	0.085117407	1.882726367	0.05973746	0.504273587
SPON1	29.36953424	0.926300868	0.492445641	1.881021559	0.059968986	0.505557487
KIF3A	454.175978	0.259527769	0.137972807	1.881006662	0.059971013	0.505557487
NKAPD1	548.1816089	-0.244399482	0.129939377	-1.880873127	0.05998918	0.505557487
SGK1	208.3322421	-0.823833562	0.438046528	-1.880698762	0.060012908	0.505557487
BTN3A2	617.0269361	0.327738347	0.174276729	1.880562879	0.060031405	0.505557487
EGR2	11.89663513	-1.109550885	0.590142966	-1.880139133	0.060089118	0.505659359
REXO2	731.7348079	-0.260219873	0.13841392	-1.880012301	0.060106402	0.505659359
LRRC4C	3.538712247	-2.772263967	1.474726767	-1.879849223	0.06012863	0.505659359
MOB1B	966.169706	0.188447113	0.100274964	1.879303717	0.060203035	0.506046262
TRO	446.1660584	0.240721082	0.128107983	1.879048259	0.060237904	0.506100636
ADHFE1	9.619157941	1.296288198	0.690442132	1.877475514	0.06045295	0.507668037
KLHL4	294.2772301	-0.407375172	0.21710468	-1.876399774	0.060600406	0.508551347
USP53	330.978239	0.276598905	0.147417844	1.876291883	0.060615211	0.508551347
EZR	1614.384373	-0.32619679	0.173890264	-1.875877246	0.060672138	0.508758614
BAMBI	86.0390286	0.547606598	0.291948458	1.875696147	0.060697015	0.508758614
PCID2	884.2875544	0.261137415	0.139242738	1.875411385	0.06073615	0.508764029
MBOAT2	3127.578833	0.482325472	0.25720239	1.875276011	0.060754762	0.508764029
FAM111B	183.8780927	-0.5749526	0.306658589	-1.874894819	0.060807195	0.508920967
PARP8	294.9402475	-0.317810019	0.169523576	-1.874724601	0.060830621	0.508920967
PLPP3	280.6404713	-0.376825458	0.201155742	-1.873302021	0.061026691	0.509631108
KCTD16	14.04405916	1.355567809	0.723746641	1.872986667	0.061070226	0.509631108
PTDSS2	743.5711613	-0.242646483	0.129557748	-1.872882837	0.061084566	0.509631108
RAD21	9863.641196	-0.27746619	0.148154761	-1.872813191	0.061094186	0.509631108
CHRNB2	21.876341	1.000878846	0.534459189	1.872694616	0.061110567	0.509631108
PIP5K1A	1797.75613	0.170159526	0.090865438	1.872653998	0.06111618	0.509631108
ATP13A4	10.25964744	1.399736405	0.747527038	1.872489333	0.061138936	0.509631108
WDR70	503.1834026	-0.234100124	0.125023393	-1.872450577	0.061144293	0.509631108
PNMA8A	5.83650463	-1.617321711	0.864082576	-1.87172124	0.06124518	0.510233333
LINC00641	322.6973543	0.541442917	0.289552169	1.869932174	0.061493237	0.511526906
SYCP2	57.68539611	0.524580702	0.280535855	1.869923905	0.061494386	0.511526906
MIR4653	2.19670552	-3.807608225	2.036782505	-1.86942308	0.061563976	0.511526906
GPR183	172.0385821	0.565451084	0.302509021	1.869204037	0.061594433	0.511526906
LINC01088	3.783733468	-2.164880868	1.158190645	-1.869192156	0.061596085	0.511526906
UROD	475.3531406	-0.253510671	0.135634951	-1.869065963	0.061613638	0.511526906
SFXN2	177.3439206	0.642090225	0.34353674	1.869058386	0.061614692	0.511526906
VPS4B	727.4088978	-0.239672109	0.128239068	-1.868947682	0.061630094	0.511526906
GLS	3828.375939	0.208634031	0.111689244	1.867986775	0.061763914	0.512398954
CABCOCO1	25.86827026	-0.796915866	0.426823549	-1.867085047	0.061889712	0.513094946
POLR3A	873.2333981	0.177112337	0.094866054	1.86697274	0.061905395	0.513094946
FRZB	10.92883201	1.110836316	0.595082624	1.866692576	0.061944531	0.513180634
SKIV2L	1678.146643	0.181874589	0.097462955	1.866089417	0.062028856	0.513481539
VPS50	333.4763711	-0.270111216	0.144817048	-1.865189355	0.062154867	0.513481539
VAPA	2033.893063	-0.231581435	0.124165782	-1.865098665	0.062167576	0.513481539
GPRC5B	310.9057417	0.629662328	0.337635901	1.864915213	0.06219329	0.513481539
HIGD1B	7.433105091	-1.536428265	0.82394576	-1.864720154	0.06222064	0.513481539
ATRNL1	31.86606028	-1.505878268	0.807619839	-1.864588009	0.062239175	0.513481539
SMOX	97.89654726	-0.462834907	0.24823115	-1.86453194	0.062247041	0.513481539

FAM160B2	1071.948197	0.363680331	0.1950758	1.864302644	0.062279216	0.513481539
H2AC7	30.32545592	-0.818049013	0.438816247	-1.864217696	0.062291139	0.513481539
AEN	656.3869324	0.366955706	0.19684368	1.864198566	0.062293825	0.513481539
ABHD17B	213.9017086	-0.348758892	0.187085334	-1.864170132	0.062297816	0.513481539
ZNF462	1232.430751	-0.230441449	0.123693572	-1.863002623	0.062461897	0.51437631
CNTNAP2	149.7931755	0.591248652	0.317365968	1.862986937	0.062464104	0.51437631
WTAP	1010.863819	-0.338336895	0.181660227	-1.862470949	0.062536737	0.514521497
PRKAR1A	3582.626489	-0.191371478	0.102752465	-1.862451458	0.062539482	0.514521497
PERP	975.8972255	-0.270016191	0.144999695	-1.862184542	0.062577083	0.514593268
SLC2A6	356.9797321	0.47473884	0.254991434	1.861783481	0.062633616	0.514598455
MRTFA	355.8422645	0.28283399	0.151916691	1.861770343	0.062635468	0.514598455
HNRNPM	2073.474692	-0.540674484	0.290495621	-1.861213888	0.062713978	0.514816225
PDXDC2P-NPIPB14P	294.4108683	0.613948633	0.329872156	1.861171432	0.062719971	0.514816225
GUSBP3	18.05823312	0.904210739	0.485881843	1.860968365	0.062748644	0.514816225
NRL	20.92795355	-0.875198647	0.470574166	-1.859852729	0.062906365	0.515693358
PRKAG2	98.59142659	0.53417216	0.287241222	1.859663999	0.062933078	0.515693358
TMEM245	1716.430962	0.389495295	0.2094513	1.859598363	0.062942371	0.515693358
DCAF6	916.2293165	-0.193117355	0.10386982	-1.859224894	0.062995268	0.515836901
NPTN	1445.211109	-0.32427429	0.174428606	-1.85906599	0.063017785	0.515836901
NFKB1	393.4370204	-0.264575856	0.142342443	-1.858727805	0.06306573	0.515992338
ZNF500	214.5641414	0.320685276	0.172595061	1.858021157	0.06316601	0.516575629
EPB41L4A-AS1	135.7381335	-0.489371226	0.263464484	-1.857446658	0.063247634	0.516936816
SHC4	23.72365822	-0.741297066	0.399125746	-1.857302051	0.063268193	0.516936816
GMPS	2316.600062	-0.221864766	0.11947701	-1.856966167	0.063315968	0.516974494
FICD	115.843928	0.442309052	0.238202468	1.856861747	0.063330826	0.516974494
DHFR	432.4242841	-0.393210558	0.211862101	-1.855974034	0.063457259	0.517429874
UBE2D2	1010.496665	-0.304103248	0.163852066	-1.855962237	0.063458941	0.517429874
H19	20.25090692	-0.806663067	0.434681798	-1.855755341	0.063488439	0.517429874
MCCC2	755.8133249	-0.254141786	0.136955304	-1.85565494	0.063502757	0.517429874
RTF1	1292.464111	-0.225461295	0.121514603	-1.855425505	0.063535488	0.517459962
FAM86B3P	319.925782	0.663250367	0.357661998	1.854405474	0.063681172	0.518409543
TSPAN1	5.829686215	1.843600196	0.994318411	1.854134627	0.063719902	0.518487969
LOC100507551	10.224474	-1.336029141	0.720726631	-1.853725231	0.06377848	0.518627433
SNORD12B	45.07840644	0.74954224	0.404369317	1.853608094	0.063795249	0.518627433
PLPP5	754.2710901	0.33652978	0.18158927	1.853247057	0.063846956	0.518811104
PVR	1798.394435	0.238387215	0.128668729	1.852720676	0.063922405	0.518975311
MIR142	25.94900272	0.859223218	0.463768202	1.85269972	0.06392541	0.518975311
SLC35F6	535.9285657	-0.220324781	0.118968619	-1.851957124	0.06403198	0.519223274
LOC101928731	12.97442082	1.857478826	1.00306491	1.851803216	0.064054086	0.519223274
CCDC78	214.6185739	0.540059751	0.291692187	1.851471435	0.06410176	0.519223274
TMC1	2.390512618	3.182996471	1.719219616	1.851419354	0.064109247	0.519223274
CDK1	1429.784717	0.202501565	0.10937846	1.851384312	0.064114284	0.519223274
RPL19	4873.401638	-0.355216493	0.191902018	-1.851030525	0.064165162	0.519223274
MLLT3	186.0647777	-0.357247338	0.193001496	-1.851008132	0.064168383	0.519223274
SNHG19	38.60203786	0.651335183	0.351908627	1.850864493	0.06418905	0.519223274
ZNF100	119.1197319	0.358790545	0.193900874	1.850381267	0.064258617	0.519550161
PDE3B	473.1823659	0.254469738	0.13756002	1.849881514	0.064330629	0.519694838
DTX3	242.2637215	-0.279466341	0.151074947	-1.849852317	0.064334838	0.519694838
NINL	368.3882552	0.403320503	0.218102432	1.849225149	0.064425309	0.520177087
B3GAT2	18.35730551	0.899734109	0.486613032	1.84897249	0.064461785	0.520177087
LINC01116	289.8903185	-0.310054818	0.16771718	-1.848676554	0.064504531	0.520177087
CTAG2	683.6270731	-0.270868888	0.146524145	-1.848629709	0.0645113	0.520177087
CD276	2189.907383	-0.369004008	0.199824441	-1.846641013	0.064799186	0.522154242
PPEF1	61.20594741	-0.492529188	0.266732116	-1.846531253	0.064815106	0.522154242
PTPRJ	532.5406037	0.491597958	0.266312996	1.845940552	0.064900838	0.522608643
SEC61G	190.5952228	0.326521192	0.176933706	1.845443692	0.064973022	0.522953594
SCAT8	8.787711516	-1.336106665	0.724288071	-1.844717203	0.065078687	0.523567586
LYPD6B	7.473689464	-1.441691609	0.781615037	-1.844503421	0.065109808	0.523581577
RPA2	756.1461183	-0.249073179	0.13508398	-1.843839516	0.065206532	0.523913735
CENPB	1712.51584	-0.239781942	0.130046545	-1.843816318	0.065209914	0.523913735
MTMR8	44.05053805	-0.675715677	0.366548684	-1.843454109	0.065262736	0.524020779
MIR4784	7.385950587	1.797069444	0.974908184	1.843321733	0.06528205	0.524020779
HOMEZ	239.0809781	-0.37113338	0.20136408	-1.843096247	0.065314959	0.524048885
TMED7-TICAM2	19.79471862	0.923088156	0.500954447	1.842658871	0.065378833	0.524325291
TBC1D4	2265.992133	0.21300577	0.115715234	1.840775521	0.065654461	0.526298922
MIR297	7.510295128	2.309757396	1.255272301	1.840044901	0.065761645	0.526921098
NPM3	243.8240521	-0.293728645	0.159669907	-1.839599273	0.065827091	0.527208433
FCN3	8.618414938	1.589882148	0.864495157	1.839087398	0.065902332	0.527573927
CPT1B	3.976439953	-1.924787711	1.048237514	-1.836213344	0.06632611	0.530590113
ANKRD10	517.4259072	0.272995934	0.14868748	1.836038475	0.066351967	0.530590113
FAM20A	63.00855844	-0.926394268	0.504592054	-1.835927184	0.066368427	0.530590113
CNBP	6176.505302	-0.266789972	0.145429595	-1.834495733	0.066580441	0.53196658
SLITRK4	76.85393539	0.490180149	0.267246083	1.834190209	0.066625764	0.53196658
MAP3K20-AS1	16.8275318	0.992893668	0.541414241	1.833889087	0.06667046	0.53196658
PTPRS	1266.067023	-0.261475818	0.142601127	-1.833616774	0.066710901	0.53196658
IMMT	2679.391532	-0.165592939	0.090318833	-1.833426461	0.066739176	0.53196658
MIR3162	2.408708112	2.905882059	1.585080487	1.833270981	0.066762283	0.53196658
TRAK1	456.8067538	0.282091928	0.153886726	1.833114107	0.066785604	0.53196658
RAPGEF2	651.6486246	0.330003084	0.180029265	1.833052442	0.066794773	0.53196658
SURF6	495.2229339	-0.28403676	0.154961125	-1.832954951	0.066809271	0.53196658
PDC-AS1	2.715888368	2.842088614	1.551066555	1.832344721	0.066900078	0.532451716
TRIM17	62.31694145	0.897916503	0.490112607	1.832061631	0.066942239	0.532549419
C9orf64	352.5700469	-0.386938382	0.21126842	-1.831501278	0.067025757	0.532975901
STK10	634.8952482	-0.20774521	0.113451202	-1.831141555	0.067079417	0.533164683
LINC01410	183.9489064	0.495112907	0.270429222	1.830840998	0.067124279	0.533283394
H1-5	1329.942454	-0.379583578	0.207389815	-1.830290358	0.067206532	0.533454991
URB1	1470.816255	0.265885998	0.145275466	1.830219548	0.067217116	0.533454991
LOC100505716	71.93446892	0.794507981	0.434144423	1.830054563	0.06724178	0.533454991
APLN	20.21623702	0.906873374	0.495592525	1.829877022	0.06726833	0.533454991
PRKD2	403.3407581	0.29882951	0.163322033	1.829695015	0.067295557	0.533454991

DOK6	226.207997	0.550623583	0.300988624	1.829383369	0.067342197	0.533587352
MIR22HG	126.3678037	0.693468584	0.379244809	1.828551289	0.067466856	0.534055538
EXTL3	4927.174378	0.295948285	0.161857437	1.828450333	0.067481993	0.534055538
TLE1	641.7049376	-0.260747877	0.142610724	-1.828388983	0.067491194	0.534055538
JMJD7	108.2724443	0.490769776	0.268474019	1.827997281	0.06754996	0.534067539
TMEM126A	316.1656538	0.292671601	0.160116837	1.827862738	0.067570155	0.534067539
PHPT1	289.7701677	-0.305155371	0.166954131	-1.827779703	0.067582621	0.534067539
EPM2AIP1	595.6464459	0.448345308	0.245428935	1.826782595	0.067732464	0.535014408
SLIT1	4.287502846	1.931264412	1.057391911	1.826441448	0.067783793	0.535182631
DTNBP1	163.1337455	-0.3152529	0.172631559	-1.826160301	0.067826119	0.535193065
CXXC4	84.9921074	-0.396763237	0.217281439	-1.826033729	0.067845181	0.535193065
TMEM67	620.0376355	-0.301358028	0.165124277	-1.82503768	0.067995343	0.535962465
RPS16P5	120.8018627	0.875506282	0.479768568	1.824851271	0.068023476	0.535962465
DEGS2	23.73742726	0.7568512	0.414761044	1.82478854	0.068032946	0.535962465
ARMH1	8.780483644	1.187468367	0.650822679	1.824565132	0.068066679	0.535991261
MIR329-1	3.507581449	-2.16864994	1.188940705	-1.824018582	0.068149263	0.536204237
PHF1	393.7595102	0.29797594	0.163365088	1.823987873	0.068153906	0.536204237
POM121	773.3131241	0.222693577	0.122125029	1.823488434	0.068229447	0.536561672
LIF	284.2242409	0.468198214	0.256851962	1.822832931	0.068328699	0.537105164
FMNL2	1689.555975	-0.162690857	0.089282819	-1.822196689	0.068425147	0.537398115
RAP1B	1999.841656	-0.215845681	0.118454722	-1.822178789	0.068427862	0.537398115
NDUFB4	140.5857934	0.336256998	0.184554755	1.82199043	0.068456438	0.537398115
TLL1	6.72146847	-1.521537376	0.835467484	-1.821180842	0.068579372	0.538126113
LOC93622	77.84015293	-0.408541411	0.224454076	-1.8201559	0.068735267	0.539111995
RN7SL1	122549.8135	-1.026058965	0.563808317	-1.819871992	0.068778501	0.53914513
CLDND1	671.1776755	0.255853307	0.140599532	1.819730859	0.068800001	0.53914513
ZGRF1	310.7122823	0.407171583	0.2238332	1.819084847	0.068898487	0.539679573
MGC2889	39.06513936	0.855433181	0.470399074	1.818526497	0.068983701	0.540109643
COP1	977.6699015	-0.303069099	0.166677375	-1.818297766	0.069018634	0.540145832
COX10	381.227648	0.232396795	0.127879657	1.817308551	0.069169881	0.540617531
AHNAK	27690.00461	-0.391070364	0.215208494	-1.817169744	0.069191126	0.540617531
USF3	506.474929	0.233915951	0.128728288	1.817129352	0.069197309	0.540617531
SRFBP1	302.4248715	0.266549818	0.146705566	1.816903235	0.069231931	0.540617531
PUS10	177.4015484	0.285089774	0.15691912	1.816794365	0.069248606	0.540617531
LOC101928053	10.71877758	1.316434044	0.724623754	1.816713896	0.069260933	0.540617531
FUT11	1574.712509	0.224857507	0.123787301	1.816482823	0.069296341	0.540657088
GYS1	1238.823534	0.200108158	0.110253663	1.814979682	0.069527032	0.542219566
FAM43A	63.49257708	-0.69727722	0.384340206	-1.814218781	0.06964405	0.542894562
FAM225B	16.56595683	-1.141629411	0.629382073	-1.813889306	0.069694769	0.542902742
KLRC2	17.77558281	-1.201309865	0.662310702	-1.813816178	0.069706031	0.542902742
TACC1	4343.824383	0.252737065	0.139358909	1.813569491	0.069744031	0.542961394
KDM4D	37.75366377	0.658848452	0.363342036	1.813300932	0.06978542	0.543044857
HSD17B12	1495.37142	-0.238622316	0.131609795	-1.813104537	0.0698157	0.543044857
TXLNG	782.1691954	-0.218846456	0.120724111	-1.81278167	0.069865502	0.54306711
PMM1	314.8355473	-0.308198547	0.170022671	-1.812690887	0.069879511	0.54306711
SFSWAP	688.2612397	0.267919517	0.147833024	1.812311689	0.069938049	0.543152336
PRDM10	264.9130352	0.28403802	0.156734412	1.812225003	0.069951437	0.543152336
LOC101928120	17.09353919	-1.054803158	0.582705755	-1.810181465	0.070267651	0.545370008
LINC00976	7.90775064	1.584026472	0.87542602	1.80943499	0.070383451	0.546030954
SART1	337.1525841	-0.269708518	0.149166902	-1.808098942	0.070591103	0.54723288
MCTP2	876.3717887	-0.368888686	0.204026493	-1.80804307	0.070599797	0.54723288
SNORD131	2.937746675	2.606937302	1.442132322	1.807696327	0.070653777	0.547346276
ACTR3C	5.931675	1.838866518	1.017322835	1.807554549	0.070675858	0.547346276
SKA2	733.3825165	-0.214139919	0.118495128	-1.807162229	0.070736988	0.547394923
REXO1	662.3499027	0.204245005	0.113022382	1.807119973	0.070743575	0.547394923
SNX6	1771.902955	-0.257757	0.14268837	-1.806433141	0.070850709	0.547791964
PCNX3	1222.764665	0.256144329	0.141799485	1.806384051	0.070858371	0.547791964
PRKAR2B	42.14173107	-0.557753623	0.308833105	-1.80600335	0.070917816	0.547791964
HCG27	12.20381227	1.258964098	0.697099616	1.806003144	0.070917849	0.547791964
MIR6753	6.681952587	1.825572155	1.011023498	1.805667383	0.07097031	0.547959675
EVX2	5.166885908	2.022046528	1.12025559	1.804986778	0.071076751	0.548543831
FHAD1	34.41018949	-0.685497841	0.379848857	-1.804659482	0.071127984	0.548678743
WDR7	586.4554815	0.208594407	0.115597957	1.804481784	0.071155812	0.548678743
GLYCTK-AS1	16.20071392	1.007276072	0.558481959	1.80359644	0.071294595	0.549466999
ARHGAP20	10.51051842	-1.054321452	0.584630673	-1.803397429	0.071325821	0.549466999
RNF2	954.0876504	-0.185970024	0.103131047	-1.803239938	0.071350541	0.549466999
GALNT1	2738.703705	-0.197600534	0.109633953	-1.802366218	0.071487807	0.550130439
COG7	292.7966206	0.261271963	0.144965967	1.802298629	0.071498434	0.550130439
THSD7A	73.52060191	0.674284883	0.374259026	1.801652963	0.071600023	0.550674323
CAVIN4	44.29945241	0.550569231	0.305680737	1.801125043	0.071683173	0.551075992
CTSF	332.7534997	-0.494512486	0.274615053	-1.800747922	0.071742621	0.551230254
PDCD7	612.8618531	-0.247942082	0.137710295	-1.800461486	0.0717878	0.551230254
TMED5	1147.783969	0.200336391	0.111272695	1.800409263	0.071796039	0.551230254
BICC1	1204.426591	0.254624297	0.141445307	1.800160801	0.071835251	0.551254826
TFAP4	359.3238447	-0.348954101	0.19386371	-1.799997019	0.071861109	0.551254826
HEG1	1463.915342	0.505983738	0.281157737	1.799643655	0.071916923	0.551445599
RHBDD1	830.8031516	-0.217743482	0.12102969	-1.799091461	0.072004214	0.551877461
NAA25	2546.632146	0.276047793	0.15352553	1.798057905	0.072167832	0.55267951
CNIH4	525.9497135	-0.222064817	0.123513621	-1.797897393	0.072193269	0.55267951
TRIM37	722.511706	0.222710941	0.123880263	1.797791968	0.072209981	0.55267951
A1BG	112.9429567	0.457392174	0.254439329	1.797647305	0.072232917	0.55267951
CLN3	228.0539379	-0.295271169	0.164290222	-1.797253448	0.072295393	0.55292013
LOC100287497	109.1722203	0.727962732	0.405127702	1.796872267	0.0723559	0.553145492
LRP2BP	34.78709239	0.977169132	0.544032801	1.796158485	0.072469315	0.553774955
CCDC183-AS1	83.66546831	0.757401026	0.421797038	1.795652787	0.072549755	0.553813281
MIR4712	31.8975163	0.692909771	0.385905775	1.795541339	0.072567493	0.553813281
VAV2	876.0222209	0.19734353	0.10992039	1.795331433	0.07260091	0.553813281
ALKBH2	326.342423	-0.248490776	0.138411365	-1.795306159	0.072604934	0.553813281
PIGG	639.351227	0.274245437	0.152770163	1.795150519	0.072629722	0.553813281
BUD31	268.3227995	-0.33359639	0.18590543	-1.794441347	0.072742752	0.554117143

ZNF197-AS1	7.431925148	1.267531581	0.706367719	1.794435881	0.072743624	0.554117143
SEMA4C	1684.312973	0.330147548	0.183996396	1.794315302	0.072762857	0.554117143
DSC2	1205.54294	0.333888982	0.186111689	1.794024786	0.072809213	0.554233307
IMPA1P1	3.653794186	2.031116165	1.132518415	1.793450895	0.072900856	0.554607963
ARHGEF7	765.5868298	0.222119435	0.1238724	1.793130957	0.072951987	0.554607963
MINDY4	4.52235784	2.004271791	1.117793636	1.793060657	0.072963226	0.554607963
RBBP7	2751.603213	-0.19932838	0.111179982	-1.792844147	0.072997848	0.554607963
APOBEC2	12.99055061	1.18945191	0.663481594	1.792742891	0.073014045	0.554607963
MRTFA-AS1	24.94455683	1.235882977	0.689635685	1.792080956	0.073119999	0.554714996
ZNF32-AS1	5.214896973	1.699482415	0.948353773	1.792034221	0.073127484	0.554714996
SNHG29	3842.074659	-0.298667784	0.166669608	-1.791975087	0.073136957	0.554714996
VPS11	366.0229368	-0.262909451	0.14673053	-1.791784241	0.073167534	0.554714996
DNAJB6	808.5619426	-0.315047821	0.175839054	-1.791682871	0.07318378	0.554714996
ARPC2	2594.922956	-0.254788004	0.142275079	-1.790812599	0.073323373	0.555511316
UBE2CP5	87.50459619	0.494504358	0.276160777	1.790639366	0.073351186	0.555511316
TP53BP2	669.3749558	0.200878781	0.112213135	1.7901539	0.073429174	0.5558599
ZNF681	53.45258183	0.497298544	0.277847597	1.789824884	0.073482068	0.5558599
DYNC1I1	59.67540673	0.655648817	0.366331197	1.78977063	0.073490793	0.5558599
MAP4	5673.194539	-0.231678875	0.129496052	-1.789080608	0.073601835	0.556334986
RFK	223.1505407	0.341758345	0.191044077	1.788897878	0.073631264	0.556334986
TRMT5	672.0987054	-0.224762399	0.125673407	-1.788464277	0.073701134	0.556334986
C1orf54	5.518757712	-1.843931661	1.031022794	-1.788448976	0.073703601	0.556334986
KIF4A	624.3866275	-0.211576493	0.118317964	-1.788202627	0.073743323	0.556334986
LOC389705	3.562442176	1.990530902	1.113351278	1.78787319	0.073796469	0.556334986
TRAF6	386.3413044	0.244347195	0.136670992	1.787849718	0.073800257	0.556334986
ARHGEF9	291.1775783	-0.265990868	0.148788927	-1.787706073	0.073823441	0.556334986
SEC24C	5589.357954	0.153742749	0.086003335	1.787637065	0.073834582	0.556334986
CLSTN1	3798.605644	-0.302044723	0.169031965	-1.786908899	0.073952214	0.556678704
HMGB1	3619.428237	-0.331834504	0.18571289	-1.78681461	0.073967457	0.556678704
MRM2	548.5994274	-0.261787254	0.14651386	-1.786774667	0.073973915	0.556678704
KMT5A	909.6029853	-0.231309186	0.129515627	-1.785955805	0.074106414	0.557440397
SAP30BP	511.7252164	-0.239855023	0.134331597	-1.785544345	0.074173065	0.557706338
TOPORS	290.2538635	-0.264253425	0.148061175	-1.784758393	0.074300515	0.557845536
BDP1	1165.141469	0.240942774	0.135022605	1.784462487	0.074348545	0.557845536
GPD1L	357.4992557	0.339195087	0.190091053	1.784382175	0.074361585	0.557845536
ZFAS1	401.1266686	0.305798684	0.171377017	1.784362268	0.074364818	0.557845536
MBTPS2	756.3404605	-0.206797954	0.115898886	-1.784296316	0.074375529	0.557845536
SRSF4	762.6930293	0.297778448	0.166890683	1.784272449	0.074379405	0.557845536
BBS5	79.43063599	-0.38388257	0.21522443	-1.783638456	0.074482433	0.558383234
DGAT2	46.62119541	-0.655311143	0.367574673	-1.782797323	0.074619302	0.559174077
HTR7P1	26.45506196	-0.662423435	0.37166671	-1.782304999	0.074699508	0.559522763
FLCN	984.431095	-0.233386694	0.130986019	-1.781767977	0.074787076	0.559522763
HNRNPR	3858.067161	-0.263533225	0.147914551	-1.78165855	0.07480493	0.559522763
ADH5	924.8568194	-0.19551089	0.109751089	-1.781402733	0.074846682	0.559522763
DMXL2	1755.863226	0.186729851	0.10482611	1.781329595	0.074858622	0.559522763
LINC01546	4.671678271	1.80591408	1.013888688	1.781175883	0.074883722	0.559522763
HOMER3	189.032141	-0.30118705	0.169112233	-1.780989138	0.074914225	0.559522763
UBE2I	872.8782284	-0.221397626	0.12431493	-1.780941564	0.074921998	0.559522763
VMO1	3.164713415	2.640969708	1.483088296	1.780723181	0.074957684	0.559522763
ITGAV	10523.19112	-0.238357934	0.133864748	-1.780587777	0.074979818	0.559522763
RBMXL3	3.699445949	3.383768952	1.900584171	1.780383634	0.075013198	0.559537545
MFSD4B	383.7323635	0.370096464	0.207953957	1.779703878	0.075124435	0.56013282
PPAT	1894.324089	0.190691298	0.107166059	1.779400119	0.075174187	0.560269348
TEKT2	5.038471902	2.007857665	1.128528917	1.779181407	0.075210026	0.560302114
MTOR	2445.702214	0.145093147	0.081581977	1.778495117	0.075322573	0.560777529
CAPRIN2	246.9209375	0.411899117	0.231611075	1.778408552	0.075336779	0.560777529
TRAF5	926.1492484	0.17113731	0.09624723	1.778101154	0.075387243	0.560877757
CLEC2D	59.36815685	-0.490376653	0.275811212	-1.777943144	0.075413193	0.560877757
IDH3G	424.508974	-0.248844654	0.139978843	-1.77773047	0.075448133	0.560903516
SIX5	331.1890098	-0.391025917	0.220052547	-1.776966106	0.075573817	0.561219217
FZD4	250.3836966	0.258849987	0.145671898	1.77693838	0.075578379	0.561219217
SNORD45B	29.09419639	0.808008074	0.454729663	1.776897659	0.07558508	0.561219217
TPD52L2	1955.492921	-0.227153926	0.127883746	-1.776253305	0.075691176	0.561772909
SNORD45C	93.70465937	0.579815785	0.326701671	1.774756106	0.075938167	0.563038128
FBXO21	1642.502196	-0.227024896	0.12792196	-1.774714022	0.075945119	0.563038128
RAB40B	260.2303926	-0.290160145	0.163503156	-1.77464553	0.075956434	0.563038128
SNORA28	37.57433753	0.690574732	0.389260441	1.774068615	0.076051801	0.563510645
MRPS35	536.5516256	-0.276625658	0.155987686	-1.773381378	0.076165533	0.564101026
RAB35	877.0690265	-0.198073804	0.111703855	-1.773204718	0.076194791	0.564101026
KBTBD6	562.4479158	0.202958251	0.114473634	1.772969399	0.076233778	0.564155282
WHRN	96.18436495	0.483419766	0.272707891	1.772665122	0.076284214	0.564294184
SNAP47	510.4216172	0.223833138	0.12631342	1.772045577	0.076386992	0.564586816
TSIX	28.97557812	1.193965756	0.673819683	1.771936596	0.076405083	0.564586816
TNFRSF25	11.31058006	1.056737531	0.596418909	1.771804206	0.076427064	0.564586816
FUT8-AS1	12.98546761	1.030252913	0.581517408	1.771663064	0.076450504	0.564586816
RTL8A	107.7903087	0.365009843	0.20608089	1.771196946	0.076527957	0.564771106
PTGS1	33.91539783	-0.728889169	0.411575889	-1.770971501	0.076565441	0.564771106
LRIF1	375.2648677	-0.294204451	0.16614372	-1.770782852	0.076596819	0.564771106
WWP1	1713.314232	0.157518583	0.088962873	1.770610348	0.07662552	0.564771106
UBTD2	713.2349565	-0.23490135	0.132670664	-1.770559844	0.076633925	0.564771106
RMDN1	783.9890689	-0.213224536	0.120459911	-1.77008712	0.076712629	0.565117422
SEC24B	574.1741543	0.238824155	0.134967644	1.769491915	0.076811819	0.565614302
FN1	286811.2716	-0.447523134	0.252978752	-1.769014714	0.076891419	0.565966579
HADHB	2237.127862	-0.215048954	0.121609747	-1.768352944	0.077001918	0.566545904
TUBB3	104.1501465	0.556169873	0.314630461	1.767692396	0.077112342	0.56703674
MRPL20	274.184137	0.30465077	0.172355384	1.767573268	0.07713227	0.56703674
ST3GAL1	2645.173199	0.322944411	0.182790224	1.766748808	0.077270305	0.567799526
COL6A2	42300.61195	-0.41029717	0.232255987	-1.766573061	0.077299756	0.567799526
TARS3	183.6214763	0.312320732	0.176832707	1.766193243	0.077363434	0.567821942
LNCRNA-ATB	43.38410326	1.222628193	0.692246447	1.766174748	0.077366536	0.567821942

TIMM9	263.6376074	-0.308928873	0.174972697	-1.765583307	0.077465783	0.56831628
DPP7	434.0822629	-0.326341198	0.18487297	-1.765218567	0.077527039	0.568402266
BRF2	441.803774	0.236297265	0.133869329	1.765133708	0.077541297	0.568402266
IGBP1	517.6090975	-0.367951623	0.20850123	-1.764745579	0.077606535	0.568646565
RPL36	523.9878513	-0.290830583	0.164852624	-1.764185341	0.07770078	0.569021471
GUSBP9	31.72001399	0.65695816	0.372412199	1.764061868	0.077721563	0.569021471
PPA1	1544.019162	-0.230574014	0.13073457	-1.763680519	0.077785783	0.569257761
PGBD1	244.8167588	0.300829077	0.170632011	1.763028369	0.077895705	0.569632748
TM9SF3	3827.533812	-0.193667476	0.109851264	-1.762997245	0.077900954	0.569632748
GAB1	237.641718	0.378668146	0.214815332	1.762761257	0.077940764	0.569649455
UBE2Q1-AS1	10.52749124	1.20288738	0.682490251	1.762497528	0.077985274	0.569649455
LINC01209	7.865729814	1.644852131	0.933450963	1.762119486	0.078049112	0.569649455
MBTPS1	2526.662828	-0.193892026	0.110038704	-1.762034806	0.078063417	0.569649455
ZNF337	513.0717933	0.367063507	0.20833402	1.761899026	0.078086359	0.569649455
EZR-AS1	5.009518041	1.837051145	1.042684444	1.761847657	0.07809504	0.569649455
GTF2H1	578.3628666	-0.254614484	0.144552759	-1.761394847	0.078171597	0.56993754
ZFYVE16	1115.097674	0.209267268	0.118818427	1.761235801	0.078198501	0.56993754
MIR10526	40.7958588	0.740799817	0.420716326	1.760805965	0.07827125	0.570234536
PLP1	59.10765219	-0.507576547	0.28835184	-1.760268103	0.07836236	0.57047212
STIMATE	343.0296131	0.229513812	0.13039903	1.760088339	0.07839283	0.57047212
TPST2	314.6454835	0.459517366	0.261094669	1.759964568	0.078413814	0.57047212
PAPSS1	974.9952536	-0.252538226	0.143506969	-1.759762804	0.078448032	0.57047212
TLR5	10.93876413	1.313862929	0.746653403	1.759669109	0.078463926	0.57047212
PHF13	740.617338	-0.218333912	0.124095736	-1.759398983	0.078509765	0.570572595
SRGAP2D	154.9929646	0.503485619	0.286246085	1.758925783	0.078590115	0.570769367
SLC14A1	424.0088692	-1.742188779	0.990577961	-1.758759883	0.078618301	0.570769367
ANGPTL3	19.07731542	1.280118309	0.727888427	1.758673805	0.078632929	0.570769367
LRRC43	2.972962216	2.469195743	1.404821975	1.757657402	0.07880582	0.571573178
LDHB	5564.489507	-0.205522533	0.116930583	-1.757645673	0.078807817	0.571573178
RNF150	905.4924195	0.309482554	0.176120661	1.757218901	0.078880505	0.571867614
SMAGP	202.6816553	-0.32492704	0.184937596	-1.75695503	0.078925475	0.571960943
SPOPL	328.9629968	0.257199026	0.146411728	1.756683226	0.078971818	0.572064147
EIF4A3	1448.840264	-0.190391016	0.108420304	-1.756045764	0.079080595	0.572444632
ACTN3	5.360434054	1.419738891	0.808591282	1.755817708	0.07911954	0.572444632
GLYATL1	13.93231398	-0.938915711	0.534754757	-1.755787487	0.079124702	0.572444632
FIZ1	137.2442431	0.342692993	0.195212574	1.755486265	0.079176168	0.572444632
NACC1	1204.381998	0.166001461	0.094564298	1.755434813	0.079184962	0.572444632
DCTD	914.1676756	-0.219093764	0.124825965	-1.755193827	0.079226159	0.572510203
AP1S2	754.2818569	-0.211254875	0.120380744	-1.754889262	0.079278251	0.572654413
TAF1A	276.9644111	0.293916739	0.167521823	1.75449821	0.079345176	0.572905609
SGPL1	856.4398538	-0.239016947	0.136254287	-1.754197627	0.07939665	0.573045079
FAM53B	730.3142341	0.262549594	0.149732471	1.753457963	0.079523429	0.573265108
EIF3H	4917.708877	-0.247056692	0.140908568	-1.753312069	0.079548455	0.573265108
PPP2R1A	3626.954101	-0.24709491	0.140930481	-1.753310621	0.079548703	0.573265108
PABPC4L	201.0466947	0.308326449	0.175869862	1.753151138	0.079576068	0.573265108
PARP4	1365.3567	-0.248190021	0.141578838	-1.753016375	0.079599196	0.573265108
MID2	211.8288648	-0.280835283	0.160225408	-1.752751244	0.079644715	0.573265108
H2AW	328.2472931	-2.242937842	1.279699298	-1.752706941	0.079652324	0.573265108
NR3C2	16.59470296	0.905571881	0.51677285	1.752359631	0.079711988	0.573462911
GALNT3	24.40371296	1.015923936	0.579844773	1.752061902	0.079763164	0.573599511
HERC2P4	19.80265567	1.01349625	0.578520985	1.751874652	0.079795364	0.573599591
RABGGTA	370.194778	0.246299217	0.140630404	1.751393801	0.0798781	0.573962799
CFH	800.9942059	-0.344134996	0.19653277	-1.751031116	0.07994055	0.57418001
COL6A3	100999.1595	-0.432377862	0.247049447	-1.750167297	0.08008945	0.574680109
SDC3	728.8232052	-0.241960394	0.138255696	-1.750093489	0.080102183	0.574680109
CHST11	116.4819598	0.734760045	0.419847047	1.750066009	0.080106924	0.574680109
EBPL	431.7715243	0.269408196	0.153972952	1.749711186	0.080168162	0.57488799
RBP1	16.478242	-0.842550199	0.481602538	-1.749472092	0.080209449	0.574917038
GSTA4	496.7880935	-0.226521528	0.129515649	-1.748989638	0.080292811	0.574917038
CLASP1	1605.133322	0.167747843	0.095912386	1.748969548	0.080296283	0.574917038
FAM161A	300.8525209	-0.301625086	0.172461581	-1.748940742	0.080301263	0.574917038
ATXN7L2	122.0061329	0.406415582	0.232444674	1.74844007	0.080387854	0.57530585
RPH3AL	9.610957153	1.325876265	0.758538025	1.747936452	0.080475032	0.575698542
TAX1BP1	936.5553012	-0.234025481	0.133935469	-1.747300267	0.080585267	0.5762558
SNORA14B	25.78882469	0.860957848	0.492833008	1.746956542	0.080644877	0.576389172
TLR8-AS1	47.76935728	-0.651757039	0.37311064	-1.746819759	0.080668608	0.576389172
SNORD167	27.78988661	0.927335409	0.530943395	1.746580554	0.080710123	0.576454665
PCOLCE-AS1	26.02016446	0.800922425	0.458671777	1.746177691	0.08078008	0.576621991
LOC102724488	18.39701	0.858987255	0.491953802	1.746073007	0.080798267	0.576621991
XRCC6	4749.618358	-0.264415712	0.151489449	-1.745439789	0.080908345	0.577176422
THTPA	515.9874894	0.32424886	0.185821274	1.744950144	0.080993548	0.577407007
C19orf67	5.236644295	1.616902934	0.926714905	1.744768457	0.081025182	0.577407007
FLJ37201	40.64090949	0.642228514	0.368134421	1.744548942	0.081063415	0.577407007
PSMA5	652.7069944	-0.319714789	0.183269153	-1.744509555	0.081070277	0.577407007
MINK1	1689.8834	0.168078117	0.096370471	1.744083179	0.081144587	0.57756638
IGSF8	237.5440109	0.326730214	0.187349005	1.743965567	0.081165094	0.57756638
CDC20	1168.439312	0.195408464	0.112057484	1.743823412	0.081189887	0.57756638
PRAME	625.8212278	-0.192960018	0.110697538	-1.743128363	0.081311195	0.578049122
SNORD97	19.05402118	0.891097464	0.511254886	1.742961267	0.081340381	0.578049122
AFF1	679.5739061	0.297406097	0.170664961	1.742631271	0.081398044	0.578049122
TRPC3	48.9955662	0.559860647	0.321289447	1.742542907	0.08141349	0.578049122
SAMHD1	286.0251297	-0.255288603	0.146506583	-1.742506025	0.081419938	0.578049122
FNDC4	54.18315519	-0.811696152	0.465938931	-1.742065531	0.081496978	0.578365652
RASA1	2077.275874	-0.218456723	0.125464042	-1.741189903	0.081650296	0.5788417
HEXIM2	114.0776377	0.719505108	0.413235369	1.741150833	0.081657143	0.5788417
SDCCAG8	146.0297893	-0.3445317	0.197878679	-1.741125937	0.081661506	0.5788417
KCTD13	151.4392319	0.319804761	0.183730579	1.740618041	0.081750553	0.578954688
LOC440300	159.5776631	0.58006474	0.333265503	1.740548404	0.081762768	0.578954688
GLRX3	694.7741088	-0.236845383	0.136080561	-1.740479179	0.081774913	0.578954688
SLC29A1	1634.326946	0.199965526	0.114907084	1.740236709	0.081817462	0.579025885

POLA1	971.3703141	-0.338520155	0.194608482	-1.73949332	0.081948026	0.579719661
C14orf178	13.47667033	0.941905193	0.541634493	1.739005187	0.082033851	0.579994891
FTH1	1337.793032	-0.386918868	0.222507593	-1.73890186	0.082052027	0.579994891
SLC12A4	997.6821199	0.221879201	0.127631334	1.738438309	0.082133611	0.580117748
EZH1	569.7409532	0.279935129	0.161027259	1.738433171	0.082134516	0.580117748
FAM111A	501.8910506	-0.223073041	0.128341425	-1.738121896	0.082189337	0.580274958
HLA-L	85.17050221	0.664886254	0.382586225	1.737872958	0.082233202	0.580354714
TBC1D7-LOC100130357	7.910521034	1.35095213	0.777821684	1.736840407	0.082415345	0.581409913
SNORA51	20.30418888	0.823490451	0.474214313	1.736536474	0.082469021	0.581558351
DALRD3	244.4196608	-0.272709228	0.157093264	-1.735970214	0.082569101	0.582033776
RBM10	997.9904509	-0.227370699	0.131071455	-1.734707973	0.082792543	0.583207711
PLS3	4197.127697	-0.241476084	0.139206601	-1.73465972	0.082801095	0.583207711
NUDT14	18.15595808	-0.918460813	0.529629367	-1.734157638	0.082890117	0.583604063
VIL1	6.439960628	1.665729297	0.960773172	1.733738353	0.082964518	0.583697454
ARHGEF6	754.3467989	-0.348873007	0.201228729	-1.733713716	0.082968892	0.583697454
CCNB3	3.021697328	-2.28573948	1.319058604	-1.732856655	0.08312115	0.583930872
NPIPB15	12.6063685	1.14089685	0.658438118	1.732732081	0.0831433	0.583930872
SYNJ1	246.1479864	0.276703511	0.159694762	1.732702488	0.083148562	0.583930872
POLG	1840.695885	0.176313144	0.101761255	1.732615657	0.083164005	0.583930872
SNORD168	3.258262004	3.303799656	1.906839577	1.732604932	0.083165912	0.583930872
PRKACA	778.9284965	-0.203597136	0.117555641	-1.731921449	0.083287549	0.584332976
COMMD2	846.5421195	-0.190676535	0.110100107	-1.73184695	0.083300816	0.584332976
BRWD1	1600.091929	0.207979984	0.120111975	1.731550776	0.083353577	0.584332976
TSHR	190.2403839	0.745125979	0.430323987	1.731546467	0.083354345	0.584332976
YBX1	3489.06841	-0.301488669	0.174214718	-1.730557974	0.083530633	0.585338527
SNRPC	442.9169272	-0.280645547	0.162237802	-1.729840666	0.083658747	0.586005843
TANC1	863.3102208	0.19579695	0.113208236	1.729529205	0.083714424	0.586165437
BGN	359.206264	-0.319933427	0.18501714	-1.729209673	0.083771576	0.586191782
ATXN7L1	100.0932465	-0.407520617	0.235678159	-1.729140362	0.083783977	0.586191782
MIR491	3.589246489	2.307533216	1.335059467	1.728412309	0.08391433	0.586873377
ROR1-AS1	7.193777581	2.34380257	1.356753276	1.727508318	0.084076413	0.587776255
RNMT	1171.294555	-0.20614064	0.119354571	-1.727128159	0.084144649	0.588022609
ACER3	316.2889052	-0.264155982	0.15297704	-1.726768811	0.084209192	0.58820272
GDAP2	595.930546	0.211391845	0.122457408	1.726247909	0.084302822	0.58820272
ACP3	22.53716923	-0.804914224	0.46632403	-1.726083522	0.084332388	0.58820272
LMTK2	495.3339158	0.200779537	0.116325052	1.72602146	0.084343552	0.58820272
MRM1	291.3105236	0.383937907	0.222446343	1.72597986	0.084351036	0.58820272
USB1	197.180996	0.342103727	0.19821954	1.72588296	0.084368471	0.58820272
ASH2L	1450.833992	-0.17971144	0.104165163	-1.725254724	0.084481577	0.588548503
LOC340512	15.50037549	1.254330097	0.727046478	1.725240593	0.084484123	0.588548503
RMI2	185.8747861	-0.30186441	0.175022456	-1.724718177	0.084578274	0.588710151
NFYB	381.4425083	-0.28282541	0.163987094	-1.724680909	0.084584993	0.588710151
PRICKLE2-AS3	5.517591136	1.831560024	1.062191827	1.72432133	0.084649851	0.588710151
RIMS4	1758.599788	-0.48199934	0.279537071	-1.724276995	0.08465785	0.588710151
PRR14L	1983.941488	0.196671938	0.114065898	1.724195759	0.084672509	0.588710151
PGM5	3.076437415	-3.193603441	1.852477915	-1.723963031	0.084714517	0.588772502
EIF6	1009.021603	-0.288471498	0.167355006	-1.723710008	0.084760207	0.588860389
EFTUD2	2850.00701	-0.199617002	0.115916188	-1.722080464	0.085054943	0.58973628
FECH	584.8435213	-0.230943643	0.134110081	-1.722045372	0.0850613	0.58973628
STX7	673.56586	0.239393505	0.139031045	1.72187086	0.085092915	0.58973628
SKP1	1730.938676	-0.250807068	0.145666855	-1.72178543	0.085108395	0.58973628
FGFRL1	310.7835048	0.275948086	0.160281646	1.721644947	0.085133856	0.58973628
RAB15	203.2429518	-0.385221223	0.223770046	-1.721504868	0.08515925	0.58973628
EPB41L5	313.6304542	0.281293696	0.163406752	1.721432517	0.085172368	0.58973628
ZNF428	164.8069825	-0.332001912	0.192874011	-1.721340838	0.085188993	0.58973628
TRMT10B	149.2554489	0.304479045	0.176887633	1.721313349	0.085193978	0.58973628
RAPGEF6	813.5560831	0.270468436	0.157147369	1.72111336	0.085230255	0.58973628
PRKG1	297.7775649	-0.315902501	0.183563191	-1.72094688	0.085260464	0.58973628
COL6A1	40947.1146	-0.492409196	0.286153715	-1.720785616	0.085289734	0.58973628
DSCAS	2.412821451	-2.311237828	1.343244553	-1.720638153	0.085316506	0.58973628
RPA3	486.5649564	-0.203320801	0.118193439	-1.720237625	0.085389256	0.589853276
LINC00680	235.9938194	-0.518258066	0.301282473	-1.720173302	0.085400944	0.589853276
FAM169A	106.1521971	0.349391822	0.20313497	1.719998396	0.085432733	0.589853276
RWDD1	264.5383828	-0.249448175	0.14505131	-1.719723692	0.085482679	0.589969536
WIPI2	988.4566154	-0.291689311	0.169665376	-1.71920352	0.08557732	0.590321029
ACTR3-AS1	12.29249598	0.975402498	0.567446901	1.718931757	0.085626799	0.590321029
SNORA40	132.2200756	0.495272292	0.288143304	1.718840191	0.085643475	0.590321029
LOC100129034	702.9342945	0.319267036	0.185772945	1.71858736	0.085689535	0.590321029
NECAB3	449.7997378	0.301789033	0.175626707	1.718355018	0.08573188	0.590321029
PARVB	560.9982516	-0.236209631	0.137462863	-1.718352332	0.085732369	0.590321029
ITM2C	1957.364439	-0.318371332	0.185398174	-1.717230139	0.085937131	0.591502383
WDR27	839.5753835	0.542483711	0.316021978	1.716601214	0.08605206	0.592064756
ZNF91	89.15989233	0.407638004	0.237595536	1.715680396	0.086220555	0.592769257
SSNA1	364.5728197	-0.242567641	0.141382959	-1.715678058	0.086220983	0.592769257
DTWD1	431.9528648	-0.253843598	0.148028828	-1.714825424	0.086377239	0.593614499
ZNF790	65.68703214	0.449164467	0.261962885	1.714611087	0.086416555	0.593655745
ERBIN	2564.692914	0.261515637	0.15254855	1.71431087	0.086471648	0.593805304
PCBD1	133.9746553	-0.358718272	0.20931803	-1.713747601	0.08657509	0.594286638
HERC1	3506.967175	0.192601022	0.112408284	1.713405944	0.086637883	0.594488673
AP1B1	1153.168585	-0.167289326	0.097653591	-1.713089346	0.086696104	0.594632145
C8orf44	74.13661388	0.64132178	0.374426338	1.712811613	0.086747203	0.594632145
MSL1	2086.923251	0.148583659	0.086761912	1.712544776	0.08679632	0.594632145
LRCH4	162.6496148	0.297959022	0.173991343	1.71249338	0.086805784	0.594632145
ANKRD62	6.835363953	1.557222456	0.909387747	1.712385571	0.086825636	0.594632145
IPMK	336.7285764	0.256849991	0.150037631	1.711903802	0.086914398	0.595011361
PLIN1	3.854519581	1.855717793	1.084338267	1.711382739	0.087010481	0.595440391
NLRP11	9.351657161	1.094307596	0.639695217	1.710670281	0.087141997	0.596078525
TAF7	754.7739712	-0.247902767	0.144941471	-1.710364643	0.087198465	0.596078525
PI4KB	1213.358191	-0.149338495	0.087315385	-1.710334257	0.08720408	0.596078525
L3HYPDH	781.7040625	0.449604458	0.262979522	1.709655772	0.087329546	0.596578178

PDZD8	1292.436231	0.262430462	0.153506085	1.709576935	0.087344134	0.596578178
LOC389199	6.454155058	-1.500447606	0.877951772	-1.709031924	0.087445036	0.597038523
MAP3K2	1798.364354	0.221581847	0.129686331	1.708598314	0.087525381	0.597358213
SEC14L2	137.0190266	0.398541928	0.233330431	1.70805808	0.087625566	0.59751841
CSRNP3	188.2087733	0.309732793	0.18134002	1.708022274	0.08763221	0.59751841
ASF1A	310.5363695	-0.332576427	0.194724928	-1.707929386	0.087649446	0.59751841
IL1R1	2534.725936	0.823251253	0.482175446	1.707368676	0.087753549	0.597805549
TVP23C	178.7809369	0.575859922	0.337284624	1.707341162	0.08775866	0.597805549
TCF20	1221.496051	-0.149159075	0.087376319	-1.707088104	0.087805678	0.59789728
MIR4458HG	54.56883534	0.613927511	0.359709757	1.706730217	0.087872208	0.598121752
OTUD7A	13.87138881	2.244976643	1.315550948	1.7064916	0.087916589	0.598195347
RNFT2	31.48477545	0.64773721	0.379626783	1.706247397	0.087962027	0.59827608
EEF2	39016.7044	-0.351423223	0.206173643	-1.704501207	0.088287491	0.60026062
ZBTB5	350.284276	0.303034227	0.177814351	1.704216928	0.088340568	0.600271432
PTCD2	475.4132102	0.2674552	0.15694513	1.704131882	0.088356452	0.600271432
DDX31	680.4979521	0.326351388	0.191544872	1.703785568	0.088421155	0.600482083
H3-3B	4227.245288	-0.288365168	0.169493818	-1.701331473	0.088880761	0.602660668
H3-2	4.265455307	1.885230585	1.10817371	1.701204936	0.088904511	0.602660668
PRKAG1	446.8706049	-0.302857973	0.178030827	-1.701154675	0.088913946	0.602660668
AP1S3	58.09987959	0.551982432	0.324544017	1.700793738	0.088981726	0.602660668
SELENOH	250.0032111	-0.275460554	0.161965776	-1.700733091	0.088993118	0.602660668
XAF1	3.620062004	-2.001517383	1.176876567	-1.700702895	0.088998791	0.602660668
ACAT1	539.3327971	-0.22757335	0.133815101	-1.700655226	0.089007747	0.602660668
KAT2B	247.0023455	-0.281425149	0.165496811	-1.700486838	0.08903939	0.602660668
RBFOX1	4.366280391	-1.789037217	1.05209638	-1.700449932	0.089046327	0.602660668
ARFIP1	431.1432928	-0.197767406	0.116363413	-1.699566905	0.08921242	0.603555552
WASHC2C	317.763551	-0.267441111	0.157387425	-1.699253358	0.089271457	0.603725753
SPTY2D1OS	17.03566336	0.987211102	0.581311365	1.698248411	0.089460887	0.604777316
CCDC85C	650.0152669	0.304736247	0.179530918	1.697402602	0.089620571	0.605078953
CMTM7	152.6706716	0.292149395	0.172117571	1.697382748	0.089624322	0.605078953
AXL	1730.06106	-0.398816399	0.234962788	-1.697359837	0.089628651	0.605078953
GOT1	321.9195146	-0.283791058	0.167202185	-1.697292754	0.089641326	0.605078953
TBL1X	1097.376262	-0.298367355	0.175835032	-1.696859558	0.089723216	0.605219576
LINC01814	112.6645686	0.894668449	0.527260844	1.696823231	0.089730085	0.605219576
FLII	3560.465484	-0.1916609	0.113018465	-1.695837057	0.089916742	0.606210516
SNORD127	5.845928518	1.649491283	0.972781192	1.695644712	0.089953185	0.606210516
STX6	846.7431789	0.18664509	0.110082093	1.69550819	0.089979058	0.606210516
UBALD2	141.8195809	-0.613301324	0.36190636	-1.694640911	0.090143561	0.606875977
PTPN20	28.80315264	0.684917052	0.404169473	1.694628362	0.090145943	0.606875977
SMLR1	3.046481584	2.402495923	1.418003783	1.694280334	0.090212026	0.607091502
BRINP3	196.9975248	-0.313748468	0.185238552	-1.693753621	0.09031211	0.607415008
ATN1	1446.182316	0.184268152	0.108808606	1.693507144	0.090358976	0.607415008
ZNF154	69.18972554	0.846704481	0.499976259	1.693489372	0.090362356	0.607415008
LOC100288123	65.58674845	0.912141598	0.538911678	1.692562314	0.090538809	0.608371638
ZSWIM3	80.29940379	-0.460529381	0.272178813	-1.692010396	0.090643991	0.60852265
TCIRG1	578.5954179	0.324281054	0.191665827	1.691908563	0.090663409	0.60852265
PLSCR1	359.5272185	-0.288726082	0.170651283	-1.69190689	0.090663728	0.60852265
MARCHF9	156.9889529	-0.308545147	0.18243191	-1.691289351	0.090781553	0.609084063
CCBE1	48.37509788	0.925389733	0.547321502	1.690760786	0.090882499	0.609312409
TSN	1392.685987	-0.242080595	0.143179513	-1.690748839	0.090884782	0.609312409
NFX1	662.8199974	-0.255397788	0.151071627	-1.690574153	0.090918165	0.609312409
NUTM2B-AS1	158.7959674	0.490565911	0.290251904	1.690138481	0.091001464	0.60964139
CRYBG1	659.0082135	-0.254410939	0.150585202	-1.689481667	0.091127162	0.610254052
COX17	32.65184407	0.722827598	0.427927032	1.689137503	0.091193082	0.610306046
SNORA71A	82.50694947	0.72063661	0.426643559	1.689083533	0.091203423	0.610306046
CDHR5	117.8800563	0.640472303	0.379295455	1.688584172	0.091299146	0.610417793
ZDHHC21	311.3722706	0.393884452	0.233278823	1.688470675	0.091320914	0.610417793
PGS1	371.8615602	0.237167727	0.140477177	1.688300772	0.091353507	0.610417793
ZNF330	350.9249731	0.224191083	0.132792451	1.688281832	0.091357141	0.610417793
ZNF888	123.6133195	0.341474669	0.202288983	1.688053716	0.091400918	0.610481394
SYCP2L	26.28100728	0.64438033	0.381793679	1.687771081	0.091455181	0.610552752
DEDD	491.5106767	-0.239017157	0.141632097	-1.687591746	0.091489625	0.610552752
RPLP2	2418.659367	-0.266650564	0.158025415	-1.68739037	0.091528314	0.610552752
SPINK5	15.21805286	1.005176376	0.595736135	1.687284549	0.091548651	0.610552752
SNORD9	4.912451155	2.005969902	1.189169793	1.686865841	0.091629152	0.610861011
RASSF7	27.56822878	-0.628156931	0.372473806	-1.686445921	0.091709943	0.611170974
TOP1P1	4.752906114	-1.851812045	1.098445139	-1.685848459	0.091824991	0.611708914
SAMD10	6.529018528	-1.577226455	0.935848754	-1.685343329	0.091922351	0.61197815
FSIP1	61.63713342	-0.429210748	0.254681789	-1.685282443	0.091934091	0.61197815
KIAA1328	213.3614261	0.268621811	0.159427936	1.684910551	0.092005831	0.612039052
CRYBA1	8.184723124	1.202572926	0.713744413	1.684878935	0.092011932	0.612039052
PLXNC1	738.1076939	0.406178536	0.241107037	1.684639904	0.092058068	0.612117449
ST8SIA5	20.11419439	-0.952158737	0.565296935	-1.684351496	0.092113759	0.6122593
RBM5	872.1882188	-0.314710913	0.186888267	-1.683952231	0.092190901	0.612476695
C1orf131	111.3069859	-0.345973353	0.205468529	-1.68382649	0.092215206	0.612476695
ARG1	17.14038973	1.024007834	0.608374619	1.683186316	0.092339029	0.612863384
RFC1	1082.606451	-0.213759054	0.126997908	-1.683169878	0.09234221	0.612863384
SIPA1L1	3123.241507	0.296100999	0.17596279	1.68274781	0.092423922	0.613090553
LYRM1	165.7007503	-0.316647466	0.188185172	-1.682637705	0.092445248	0.613090553
SCP2	932.9720408	-0.271429447	0.16136593	-1.682074069	0.092554478	0.613586606
GYG2	184.531581	0.274340145	0.163136625	1.68165882	0.092635018	0.613892158
PCDH8	3.506920038	2.620083217	1.558669346	1.680974367	0.092767893	0.61435047
ARL14EP	268.5143347	-0.260673313	0.155075232	-1.680947428	0.092773126	0.61435047
IL17RA	289.5981237	-0.272124102	0.161954364	-1.68025174	0.092908346	0.614648862
MGAT3	9.383525207	-1.265755166	0.75331999	-1.680235735	0.092911459	0.614648862
TINF2	900.0706952	0.239116863	0.142338941	1.679911777	0.092974482	0.614648862
KCNS2	5.66904987	1.999654595	1.190644302	1.679472695	0.093059956	0.614648862
MAP2K4	811.8073621	-0.286530208	0.17063187	-1.679230308	0.093107167	0.614648862
NOP14	695.6613952	-0.206158722	0.122774074	-1.679171469	0.093118631	0.614648862
PCNA-AS1	11.33172439	-0.911794904	0.543012275	-1.679142342	0.093124306	0.614648862

CD74	19.65420625	1.22071076	0.726990283	1.679129403	0.093126827	0.614648862
GFPT1	3042.60644	0.298684998	0.177886556	1.679075724	0.093137287	0.614648862
MEOX2	286.3998465	-0.30735712	0.183068695	-1.678916867	0.093168247	0.614648862
MCRS1	793.4690003	-0.216073218	0.128709529	-1.678766296	0.0931976	0.614648862
ZCCHC17	324.9372515	-0.246676533	0.146981002	-1.67828856	0.09329078	0.614736681
NCOA2	1712.760004	-0.16624209	0.099066496	-1.6780859	0.093330331	0.614736681
AP2S1	932.372354	-0.318254206	0.189684061	-1.67781206	0.093383794	0.614736681
PDCD10	949.8680145	-0.221502901	0.132037577	-1.677574716	0.093430152	0.614736681
GPR85	67.55135391	0.422311973	0.251820243	1.677037432	0.093535162	0.614736681
EXOC7	1842.414233	-0.128344175	0.076546478	-1.676682952	0.093604496	0.614736681
SLC2A3	181.3491771	-0.477689237	0.284909111	-1.676637279	0.093613432	0.614736681
ROCK1	1924.2705	-0.1407581	0.083960041	-1.676489182	0.093642413	0.614736681
MACIR	215.2199165	0.321343146	0.191714559	1.67615411	0.09370801	0.614736681
DGKE	225.8235939	0.365119424	0.217833245	1.67614188	0.093710405	0.614736681
WNT11	57.85248947	0.725112545	0.432618473	1.67610167	0.09371828	0.614736681
IPO5	5177.484531	-0.163569877	0.097594977	-1.676007132	0.093736796	0.614736681
PALM3	5.1243835	1.669583527	0.996304665	1.67577608	0.093782061	0.614736681
ADAM9	7303.857083	-0.202771539	0.121011841	-1.675633865	0.093809931	0.614736681
RPL7	13235.03904	-0.284543448	0.169813518	-1.67562307	0.093812047	0.614736681
CSRP1	1111.650899	-0.224398861	0.1339323	-1.675464852	0.093843062	0.614736681
SBDS	388.2753528	-0.240916759	0.143797065	-1.675394135	0.093856927	0.614736681
SNORA71B	29.99046037	0.831322947	0.496207324	1.675354043	0.093864788	0.614736681
NOC3L	830.8167852	0.232104629	0.138584117	1.674828504	0.093967884	0.614736681
PRMT5	2050.97982	-0.234350266	0.139925128	-1.674826162	0.093968344	0.614736681
LOC102723566	72.31818567	0.628291896	0.375195914	1.674570196	0.094018591	0.614736681
GPR135	170.4130099	0.709614519	0.4237912	1.674443732	0.094043424	0.614736681
BOC	182.9690664	-0.303064538	0.181008446	-1.674311583	0.094069379	0.614736681
PSMC3	653.9105081	-0.273692099	0.163476806	-1.674195292	0.094092225	0.614736681
TUBGCP3	788.39818	0.187442133	0.111969351	1.674048578	0.094121053	0.614736681
MDM2	1623.573867	0.169556886	0.10129991	1.673810827	0.094167784	0.614736681
LOC730100	17.8373278	0.73304793	0.437979245	1.67370472	0.094188646	0.614736681
TMC4	77.9456354	0.500252302	0.298921309	1.673525061	0.094223977	0.614736681
LOC102723493	3.801797835	1.828869537	1.092879832	1.67344065	0.094240581	0.614736681
BVES-AS1	33.88040162	0.632409209	0.377915575	1.67341399	0.094245826	0.614736681
CALCOCO1	326.4182124	-0.428501856	0.2561333	-1.672964259	0.094334332	0.614818711
LOC101927960	5.120277747	1.516254885	0.906343109	1.672936958	0.094339707	0.614818711
GOLGA8R	8.81401856	1.211277266	0.724332328	1.6722673	0.094471625	0.614818711
SCYL3	319.9356071	0.303600371	0.181551166	1.672257895	0.094473479	0.614818711
C16orf71	10.31981575	1.155660928	0.691172163	1.672030487	0.09451831	0.614818711
GNE	557.7235942	0.29986151	0.179392461	1.671539084	0.094615245	0.614818711
SLC20A2	674.0178445	0.219936816	0.131590064	1.671378587	0.094646923	0.614818711
SHCBP1	814.7496297	0.208425232	0.124726694	1.671055539	0.094710708	0.614818711
TMEM205	9.897855806	-1.021810987	0.611587646	-1.670751515	0.094770769	0.614818711
CYBC1	667.4683563	0.253536719	0.151762521	1.670614836	0.09479778	0.614818711
TLK2	786.664824	-0.230597084	0.13805388	-1.670341201	0.094851876	0.614818711
CCDC186	269.0170583	0.249023509	0.149090331	1.670286115	0.094862769	0.614818711
NR2C2AP	173.5593067	0.281297764	0.168417044	1.670245227	0.094870855	0.614818711
MT2A	49.93041462	-0.712965508	0.426867224	-1.670227806	0.094874301	0.614818711
MYC	6651.404492	-0.292364399	0.175048355	-1.670192211	0.094881341	0.614818711
ADCYAP1R1	3.032255737	-2.243757576	1.343732316	-1.669795055	0.094959919	0.614818711
SNRPD2	1146.577609	-0.279911769	0.167637	-1.669749333	0.094968969	0.614818711
SEMA3G	167.2581458	-0.348249843	0.208572808	-1.669679988	0.094982695	0.614818711
NDST4	33.65643634	-0.749635112	0.448985137	-1.669621222	0.094994329	0.614818711
GRM7-AS3	5.096089154	2.371650652	1.420575376	1.669500043	0.095018322	0.614818711
P4HB	14535.94984	-0.183510139	0.109920458	-1.66948121	0.095022051	0.614818711
ARHGAP29	285.9601989	0.337516759	0.20217748	1.669408281	0.095036494	0.614818711
CHEK2	196.8657253	0.33852322	0.202799476	1.669250956	0.095067655	0.614818711
KDM1B	721.6318987	0.188909266	0.113186443	1.669009645	0.095115468	0.614818711
PER2	140.1562175	-0.463324446	0.277609009	-1.668982026	0.095120942	0.614818711
SEC24D	2499.539446	0.352136424	0.211056015	1.668450074	0.095226415	0.615028572
GET1	165.2023668	0.28457307	0.170572911	1.668336834	0.09524888	0.615028572
LINC01460	15.31471883	0.99879884	0.598829173	1.667919476	0.095331713	0.615028572
B3GNT4	56.6877002	0.636518839	0.381648501	1.66781433	0.09535259	0.615028572
ZNF276	504.6387307	0.492334042	0.295228412	1.667637741	0.095387661	0.615028572
CCDC170	40.3311918	-0.950052098	0.569707599	-1.667613526	0.095392471	0.615028572
GLIDR	39.93064462	-0.594823238	0.356734495	-1.667411607	0.095432587	0.615028572
KCNV2	12.4534212	1.695815167	1.017188579	1.667159071	0.095482779	0.615028572
NR2F6	666.0529911	0.218207196	0.13090099	1.66696368	0.095521627	0.615028572
AP2B1	5396.472017	-0.239437616	0.143657364	-1.666727068	0.095568688	0.615028572
LOC653513	15.80841716	0.808360254	0.485040775	1.666582062	0.095597538	0.615028572
RERG	33.61640588	-0.658401121	0.395081928	-1.666492629	0.095615335	0.615028572
BRCA1	1101.936543	-0.216115925	0.129719372	-1.666026611	0.095708114	0.615028572
GPATCH2L	1860.685113	0.196684592	0.118057078	1.666012715	0.095710882	0.615028572
C17orf67	51.21681105	0.555267452	0.333314692	1.665895519	0.095734226	0.615028572
EIF4H	3789.729739	-0.195031709	0.117073792	-1.665887001	0.095735923	0.615028572
BROX	1172.081717	-0.243078043	0.145945235	-1.665542849	0.095804502	0.615028572
MACROH2A2	252.3788016	-0.242450861	0.145577812	-1.66543828	0.095825348	0.615028572
SLC16A7	689.3733662	0.253640227	0.152305747	1.665335889	0.095845762	0.615028572
SMIM18	22.19486546	0.869037809	0.521851488	1.665297176	0.095853482	0.615028572
SLC11A2	957.1100629	0.175181042	0.105202891	1.665173273	0.095878192	0.615028572
ZXDC	725.8947687	0.22062628	0.132510266	1.664975002	0.095917743	0.61506088
CCNT1	1449.533441	0.147796377	0.088796445	1.664440241	0.096024484	0.61552385
ATG4A	98.40806295	0.352652603	0.211931465	1.663993603	0.096113708	0.615754242
VPS9D1-AS1	86.60909609	0.474512304	0.285178312	1.663914413	0.096129534	0.615754242
KATNA1	239.9508413	-0.236794302	0.142387757	-1.66302431	0.096307569	0.616672974
CNIH1	2929.308477	-0.216991268	0.13049951	-1.662774578	0.096357567	0.616771498
DNAJC3-DT	35.76520343	0.570117554	0.342933497	1.662472632	0.096418046	0.616795775
CLCN6	755.9650659	0.261751897	0.157453269	1.66241005	0.096430585	0.616795775
CTSK	686.3669579	-0.459319117	0.276362874	-1.662014546	0.096509858	0.61708133
LOC100287467	59.5960706	0.528743427	0.31824221	1.661449711	0.096623161	0.617584192

CREB3L1	1031.523093	0.557687622	0.335740492	1.661067507	0.096699889	0.61781655
IFNLR1	36.63916278	0.703199992	0.42337897	1.660923291	0.096728854	0.61781655
SLC9A5	334.8668842	0.402526384	0.242385803	1.660684658	0.096776796	0.617901293
RBL1	850.8795268	-0.176943457	0.106567728	-1.660385007	0.096837024	0.618064389
LUARIS	12.96913569	1.245975189	0.750619697	1.659928715	0.096928794	0.61842861
OXCT1-AS1	22.32551151	0.877186612	0.528568599	1.659551123	0.097004788	0.618691955
DNAH11	71.72507045	-0.568858976	0.342888149	-1.659021981	0.097111363	0.619150086
WNT10B	317.7077265	0.214281036	0.129218683	1.658282152	0.09726053	0.619542254
MIR3164	10.42704793	1.236056242	0.7454122	1.658218422	0.097273388	0.619542254
TMBIM6	8319.116825	-0.236234797	0.142464628	-1.658199661	0.097277173	0.619542254
GINS2	232.0551607	-0.436023914	0.262986342	-1.657971705	0.097323178	0.619545746
CERS6	963.8164072	0.218953363	0.132070478	1.657852432	0.097347255	0.619545746
ZNF236	521.076312	0.19834628	0.119688054	1.657193627	0.097480335	0.620041626
MAN2C1	2195.485351	0.285871385	0.172522156	1.657012595	0.097516929	0.620041626
SECISBP2L	1059.994349	0.247492345	0.149366199	1.656950143	0.097529555	0.620041626
SLC39A4	154.2215384	0.351774121	0.212424003	1.65599987	0.097721845	0.620911155
STK40	511.1352783	-0.331848002	0.200404342	-1.655892277	0.097743636	0.620911155
THRAP3	3084.548146	-0.266830051	0.161152819	-1.65575789	0.097770859	0.620911155
NFRKB	597.855527	0.340148456	0.20546821	1.655479726	0.097827226	0.621047795
HSPA2	246.9889849	0.330747657	0.199818904	1.655237069	0.097876419	0.62113881
TAP2	1510.046393	0.145884295	0.088172544	1.654531993	0.098019469	0.621617888
TSPAN6	599.9133249	-0.231427402	0.139875762	-1.654521119	0.098021676	0.621617888
HNRNPUL2	449.4353942	-0.236155283	0.142751209	-1.65431372	0.098063787	0.621663706
H2BC5	1879.454468	-0.314248075	0.190013191	-1.653822422	0.098163599	0.622075152
RAB3GAP2	1684.93605	-0.141339756	0.085487891	-1.653330725	0.098263573	0.622292812
POLQ	532.4036616	0.465491802	0.281588533	1.653092179	0.098312105	0.622292812
SNORA63E	5.349990612	1.569526445	0.949504063	1.652996028	0.098331672	0.622292812
WDR25	186.3904297	-0.298673555	0.18068939	-1.652966756	0.09833763	0.622292812
HSF4	313.5572905	0.490979535	0.297060406	1.652793588	0.09837288	0.622294893
PANO1	14.90267189	0.982764408	0.594690696	1.652563953	0.098419639	0.622369756
ZNF468	234.0760093	0.248867621	0.150701159	1.651398191	0.098657294	0.623505285
LOC101929066	27.91250036	-0.614596782	0.37221619	-1.651182291	0.098701358	0.623505285
TSHZ3	14.11452132	-0.844834122	0.511658337	-1.651168489	0.098704176	0.623505285
NDFIP1	1275.085684	-0.20643424	0.12503922	-1.650955911	0.098747578	0.623558412
OLFM1	3.903164183	-1.873540799	1.135107831	-1.650539929	0.098832554	0.62387393
AKTIP	389.1697436	-0.30017465	0.181891166	-1.650298121	0.098881976	0.623949051
LINC01446	150.5932199	0.291350021	0.176560882	1.650139135	0.098914482	0.623949051
NR1D1	163.6942888	-0.368702387	0.223515361	-1.649561734	0.099032607	0.624317498
TRIO	5325.098813	0.18672504	0.113200229	1.649511148	0.099042961	0.624317498
PDCD1LG2	23.62975184	0.846064731	0.513028667	1.649156832	0.099115509	0.62455388
WASHC3	238.8218887	-0.398078143	0.241469121	-1.648567489	0.099236274	0.625093814
FDPSP2	22.87280133	1.05369164	0.639240251	1.648349957	0.099280879	0.625153802
VPS51	783.4540031	-0.261910477	0.158943608	-1.647820134	0.099389586	0.625591871
GRAMD1A	656.1205518	0.237525188	0.144184095	1.647374403	0.099481113	0.625591871
MIR7974	3.457773243	2.304082942	1.398686945	1.647318545	0.099492588	0.625591871
PANK4	341.3698221	0.305394615	0.18539397	1.647273719	0.099501797	0.625591871
FKBP11	495.6048514	0.195452094	0.11866034	1.647156027	0.09952598	0.625591871
TBCA	205.8431278	-0.284757691	0.17291101	-1.646845336	0.09958984	0.62577255
C17orf100	31.34838755	0.683964161	0.415442644	1.646350395	0.09969164	0.626191405
IMPA2	323.9485301	0.378422003	0.2299386	1.6457524	0.099814746	0.626743754
LOC105374988	4.935781051	1.572393832	0.956082925	1.644620764	0.100048042	0.627520783
ACTN1	8030.195668	-0.211061735	0.128346834	-1.644463903	0.100080415	0.627520783
PJA1	198.7498305	-0.30425832	0.185037208	-1.644308857	0.100112421	0.627520783
PRXL2A	15.36036889	1.061343018	0.645482075	1.644264123	0.100121657	0.627520783
ZEB1	1346.693107	-0.161077744	0.097969668	-1.64415934	0.100143293	0.627520783
CRACR2A	363.8824406	0.323067583	0.19652146	1.643930301	0.1001906	0.627520783
MRPL23-AS1	23.62042589	1.294712376	0.787575474	1.643921654	0.100192387	0.627520783
ZNF559	436.4397172	0.211703904	0.128790351	1.643786987	0.10022021	0.627520783
SYF2	110.5567219	-0.396700259	0.241385066	-1.643433312	0.100293313	0.62774156
PICSAR	48.41188475	0.48254078	0.293645679	1.643275602	0.100325924	0.62774156
CBX3	3202.307106	-0.163870468	0.099749706	-1.642816556	0.100420894	0.628115242
TPT1-AS1	56.64207956	0.505008739	0.30748972	1.642359749	0.100515471	0.628338103
ADARB1	338.7048417	0.275925191	0.168011045	1.642303875	0.100527044	0.628338103
SLC36A1	417.7591702	0.264301142	0.161005385	1.641567101	0.10067975	0.628518939
SLC25A15	548.3130377	0.231136946	0.140811861	1.641459357	0.100702097	0.628518939
LINC02475	151.319253	0.514501138	0.313505629	1.641122487	0.100771992	0.628518939
HLA-DOA	15.14726265	-0.94647192	0.576783112	-1.640949432	0.100807913	0.628518939
CXCL16	3.261918033	2.260473188	1.377571303	1.640911932	0.100815698	0.628518939
SDAD1	462.894662	-0.214404446	0.130664629	-1.64087594	0.10082317	0.628518939
SMARCD1	950.5326614	-0.178247915	0.108638142	-1.640748922	0.100849545	0.628518939
NSD3	3880.617554	0.115064548	0.070135286	1.640608525	0.100878704	0.628518939
NAGS	49.48703472	-0.504178879	0.307326277	-1.640532935	0.100894406	0.628518939
MAGEA11	491.9572091	-0.255236736	0.155602936	-1.640307972	0.100941149	0.628518939
BLMH	1072.559107	-0.175303334	0.106875313	-1.640260317	0.100951053	0.628518939
C12orf56	31.68698424	-0.581012517	0.354248968	-1.640124798	0.100979221	0.628518939
ACAP1	134.460482	0.633099238	0.386168279	1.639438745	0.101121918	0.629187353
SCAMP2	674.9988031	0.208912902	0.127448995	1.639188302	0.101174049	0.629291993
ZNF155	82.01548665	-0.430861132	0.262914902	-1.638785515	0.101257937	0.629594012
LOC105375218	2.247943481	2.446785799	1.49322595	1.638590462	0.10129858	0.629617712
LINC01530	4.598787634	1.592927459	0.972229065	1.638428141	0.101332413	0.629617712
SNORA27	14.15378699	0.877360745	0.535632812	1.637989171	0.101423953	0.629789104
NR5A2	225.8261914	1.276629775	0.779403755	1.637956921	0.101430681	0.629789104
SNORD101	61.14265078	0.602640111	0.368062695	1.637330051	0.101561525	0.630381881
GJA3	129.3077809	-0.494421494	0.302155744	-1.636313402	0.101774013	0.631480816
EIF3B	5690.32969	-0.215465617	0.131706434	-1.635953622	0.101849294	0.631530903
WWTR1-AS1	8.066205287	1.096802466	0.67055882	1.635654374	0.101911944	0.631530903
ESF1	218.6579395	0.325275009	0.198866807	1.635642538	0.101914422	0.631530903
TPMT	333.5861899	-0.266709656	0.163065575	-1.635597554	0.101923843	0.631530903
CLCNKB	3.569695577	1.87662098	1.147563357	1.635309257	0.101984234	0.631685454
DEF6	175.2387998	-0.342237202	0.209329576	-1.634920436	0.102065728	0.631795286

SLC35F4	4.570179841	-1.504844877	0.920512856	-1.634789637	0.102093154	0.631795286
LOC101929217	2.566498694	2.666545861	1.631196965	1.634717277	0.102108329	0.631795286
ARPIN	692.1795951	-0.504365513	0.308612557	-1.634300036	0.102195866	0.63209528
GRINA	788.1087343	-0.186332183	0.114024048	-1.63414811	0.102227755	0.63209528
LOC646029	3.428227439	-2.023461831	1.238446081	-1.633871561	0.102285822	0.632234948
TNFSF10	62.83197809	1.102917781	0.675258597	1.633326529	0.10240034	0.632723322
ZNF56	7.985283759	1.28967287	0.789772696	1.632967152	0.102475905	0.632970756
RHNO1	238.5356311	-0.266003398	0.16294118	-1.632511796	0.102571716	0.633302248
TANGO2	209.1886007	0.258125367	0.158137024	1.632289266	0.102618563	0.633302248
SNORD32A	16.11694363	1.081977	0.662892607	1.632205563	0.102636189	0.633302248
CARM1	2091.923711	-0.235207446	0.144137494	-1.631826939	0.102715949	0.63357501
NYNRIN	31.02769887	-0.585282097	0.358786352	-1.631283058	0.102830607	0.633988208
ADAM10	4538.055897	-0.13719782	0.08411417	-1.63109046	0.102871233	0.633988208
PACSIN2	992.385712	0.305597272	0.187367685	1.631003084	0.102889669	0.633988208
LOC105375166	44.37908388	1.050137052	0.643952226	1.630768574	0.102939161	0.634073919
NDN	412.5466335	-0.21169916	0.129835104	-1.63052328	0.102990949	0.634173709
TTC21B	372.5849794	0.247785793	0.151999631	1.630173649	0.103064802	0.634222307
ZNF397	294.4561433	0.263444726	0.161607764	1.630148939	0.103070022	0.634222307
ZNHIT1	396.158589	-0.331834349	0.203626076	-1.629626008	0.103180564	0.634429972
MICALL2	246.1937624	0.391320899	0.240150781	1.629480018	0.103211441	0.634429972
ATXN1	1003.275045	-0.194131	0.119144775	-1.629370653	0.103234578	0.634429972
ZNF672	679.2628377	0.377588764	0.231768012	1.629166855	0.103277701	0.634429972
DACT3	138.5657439	-0.305070335	0.187257643	-1.629147576	0.103281782	0.634429972
ARF6	2582.408953	-0.165540963	0.101622611	-1.628977666	0.103317747	0.634432203
PIK3R3	117.5358925	-0.344461909	0.211587732	-1.627986206	0.103527811	0.63550313
BHLHE41	405.1961334	0.274820004	0.168876307	1.62734494	0.103663858	0.636119131
LPAR4	91.5605364	0.397828958	0.244533812	1.626887317	0.103761032	0.636121619
ADAMTS16	19.53792354	0.872575021	0.536362066	1.626839547	0.10377118	0.636121619
LOC101929599	7.196691697	1.55203144	0.954016779	1.626838723	0.103771355	0.636121619
SERINC1	4601.301373	-0.23790443	0.146303836	-1.626098372	0.10392873	0.636602018
OR3A2	3.853995838	1.643558127	1.010964366	1.625732994	0.104006468	0.636602018
SPRY1	79.47203198	-0.564117958	0.346993247	-1.625731806	0.104006721	0.636602018
C22orf15	20.43642346	1.017404574	0.625853353	1.625627743	0.10402887	0.636602018
ANO8	252.6971899	0.258385568	0.158952186	1.625555294	0.104044292	0.636602018
MIR3605	4.285347317	-1.733232255	1.066301038	-1.62546241	0.104064067	0.636602018
ARHGAP11A	1990.116449	0.207817041	0.127867731	1.625250083	0.104109283	0.636652302
STAB1	58.95790931	-0.706111896	0.434532859	-1.624990796	0.10416452	0.636652302
PDIA6	6593.498837	-0.128843876	0.079293274	-1.624902949	0.10418324	0.636652302
TAF15	1176.157893	-0.348820462	0.214691376	-1.624753022	0.104215195	0.636652302
NFE2	41.64151822	0.734616481	0.452224956	1.624449229	0.104279968	0.636781705
ZNF623	1142.166223	-0.160032589	0.098530084	-1.62420026	0.104333075	0.636781705
ANKRD27	1155.804328	0.152522378	0.093908987	1.624151031	0.104343579	0.636781705
DCP1A	565.6768795	0.209861148	0.129241651	1.623788828	0.104420885	0.636888265
ZNF675	93.43019762	0.443768103	0.273333252	1.623542325	0.104473523	0.636888265
LOC105369187	3.064449263	-1.623454833	0.99994892	-1.623537763	0.104474498	0.636888265
CLIC5	775.2727791	-0.428082083	0.263858227	-1.622394303	0.104718948	0.636888265
ST6GALNAC4	272.4934082	0.318356567	0.196229921	1.622365055	0.104725207	0.636888265
LAMP2	3175.053841	-0.180187438	0.111067128	-1.622329133	0.104732894	0.636888265
UBE2H	1627.7458	-0.24495555	0.150990886	-1.6223201	0.104734827	0.636888265
PARG	348.6828994	-0.210188334	0.129568146	-1.622222286	0.104755761	0.636888265
MT1E	16.37611807	-0.940534186	0.579812141	-1.622136066	0.104774217	0.636888265
FAM161B	134.6069176	-0.3469591	0.213939215	-1.621764853	0.104853707	0.636888265
RAD54B	607.6834775	0.318513514	0.196427119	1.621535336	0.104902878	0.636888265
TAF3	163.1474966	-0.309427122	0.190830697	-1.621474571	0.104915899	0.636888265
ACADM	417.1050117	-0.241081691	0.148694146	-1.621326039	0.104947733	0.636888265
LOC730338	2.54260861	-2.085334104	1.286192566	-1.6213234	0.104948299	0.636888265
ISG20	12.01091918	0.917888198	0.566203637	1.621127344	0.104990331	0.636888265
MYEF2	24.28676991	-1.022717059	0.630972379	-1.620858684	0.105047949	0.636888265
TNK2	1172.151202	0.290337458	0.179125933	1.620856644	0.105048387	0.636888265
MXRA8	8004.674729	-0.340144192	0.209866815	-1.620762159	0.105068657	0.636888265
FKBP8	1717.299221	-0.320806983	0.197944221	-1.620693853	0.105083313	0.636888265
LTBP1	1587.906155	-0.41320811	0.254986603	-1.620509099	0.105122961	0.636888265
ZRANB1	482.0274984	-0.253930057	0.156698485	-1.620501038	0.105124692	0.636888265
FAHD1	217.7488103	-0.303839995	0.18752395	-1.620273011	0.105173644	0.636888265
GRASP	19.6003166	-0.835783773	0.515925171	-1.619970919	0.105238524	0.636888265
EPHA7	566.4463113	-0.368179486	0.227277424	-1.619956261	0.105241673	0.636888265
CHST3	959.5980752	0.290438638	0.179294581	1.619896359	0.105254542	0.636888265
GPX7	77.77197002	-0.548354652	0.338561178	-1.619661935	0.105304917	0.63697679
RPL7L1	5311.913923	-0.165642875	0.102300732	-1.619175849	0.105409433	0.637198397
GALM	209.1468749	-0.318312513	0.196591269	-1.619158948	0.105413068	0.637198397
TEX13C	3.857629668	-1.58646094	0.980025005	-1.61879639	0.105491079	0.637409304
RHBDF2	476.1117121	0.209807914	0.129631902	1.618489816	0.10555708	0.637409304
SLMAP	947.7587072	-0.201718678	0.12463431	-1.618484327	0.105558262	0.637409304
AOC2	40.58055873	0.643311828	0.397515324	1.618332147	0.105591036	0.637409304
SLC4A11	32.1533964	0.662734908	0.409718451	1.617537375	0.105762335	0.637975584
SLC2A1-AS1	2.59255627	2.342839256	1.448505524	1.61741824	0.105788031	0.637975584
LRRC15	3906.507008	0.993716563	0.614391842	1.617398694	0.105792248	0.637975584
SUGCT	1824.44711	-0.359058949	0.222089604	-1.616730103	0.105936553	0.638193042
LINC02239	15.04670875	1.241559288	0.768039499	1.616530517	0.105979661	0.638193042
SEC24A	1875.726266	0.205656877	0.127225393	1.616476649	0.105991298	0.638193042
CAPN2	9061.594601	-0.199886704	0.123658481	-1.61644153	0.105998885	0.638193042
TTC1	287.932328	-0.26626267	0.164725502	-1.616402242	0.106007374	0.638193042
IGF2BP2	2994.958194	-0.198531804	0.12284752	-1.616083119	0.106076344	0.638207977
PKD2	582.0563233	-0.187509676	0.116028955	-1.616059345	0.106081483	0.638207977
BRMS1	191.1459049	-0.266730709	0.165086083	-1.615706806	0.106157719	0.638451079
STAG3L4	81.45301575	0.455716426	0.282098829	1.615449545	0.106213378	0.638474813
SLC6A15	4655.228632	-0.148148064	0.091712252	-1.615357385	0.106233323	0.638474813
LINC02616	62.48801048	-0.459009079	0.284239097	-1.614869606	0.106338936	0.638836499
ZNF48	115.8874382	-0.323958924	0.200625022	-1.614748353	0.106365202	0.638836499
ZNF749	240.0778835	0.269498685	0.166975724	1.613999199	0.1065276	0.639596304

AIM2	137.9932694	-0.434850578	0.269466854	-1.613744218	0.106582919	0.63968026
LINC01787	14.69512734	-1.070802711	0.663661459	-1.613477318	0.106640847	0.63968026
PDE1C	3.940544098	-1.846491004	1.144444613	-1.613438504	0.106649274	0.63968026
ZNF362	337.0338565	-0.240303884	0.148957839	-1.613234226	0.10669363	0.639730987
BASP1-AS1	12.28808979	1.021679096	0.633425402	1.612943043	0.106756883	0.639894937
CWC25	254.1706267	0.288146678	0.178693673	1.612517517	0.106849371	0.640072068
LRP1	16103.40706	-0.21529837	0.133520308	-1.612476582	0.106858272	0.640072068
JAKMIP2	24.37850076	-0.691145017	0.428727608	-1.612084231	0.106943612	0.640190451
MIR4797	2.634027044	-2.194410228	1.36124989	-1.612055393	0.106949886	0.640190451
NAT14	194.9259853	-0.273925985	0.169970459	-1.611609378	0.107046969	0.640556407
PIBF1	281.9514044	-0.220722677	0.136993162	-1.611194841	0.107137262	0.640760699
LYN	663.4642845	0.221337756	0.137381083	1.611122507	0.107153024	0.640760699
LRGUK	11.15411825	1.029808094	0.639548092	1.610212127	0.107351555	0.641725517
MYO9A	1826.75813	0.157858878	0.09806362	1.609759844	0.107450295	0.641725517
RBM26-AS1	22.38950554	-0.743399652	0.461836858	-1.609658559	0.107472417	0.641725517
ZNF264	934.7017965	0.239715532	0.148929503	1.609590624	0.107487256	0.641725517
RAB39B	11.62442811	-1.643464286	1.021065711	-1.609557806	0.107494426	0.641725517
ANKH	1020.627569	-0.27907376	0.173427656	-1.609165266	0.107580209	0.641858368
SYNCRIP	4898.94298	-0.259097914	0.161017756	-1.609126346	0.107588717	0.641858368
LOC105371824	7.735654999	1.190953302	0.740356583	1.60862121	0.107699194	0.642302419
MNAT1	366.9415352	0.252441642	0.156955507	1.608364354	0.107755404	0.642336338
ZBED5-AS1	17.83066957	-0.872784929	0.542765044	-1.608034524	0.107827618	0.642336338
HAX1	830.1298846	-0.269712062	0.167736808	-1.607947978	0.107846573	0.642336338
TPPP	76.93814683	0.825077306	0.5131471	1.607876778	0.107862169	0.642336338
NUP214	1393.545543	0.162790338	0.101252125	1.60777206	0.10788511	0.642336338
PLEKHM1P1	242.2546268	0.413068813	0.256981514	1.607387265	0.107969442	0.642623733
ARSB	404.0378261	-0.222620443	0.138593367	-1.606284968	0.108211312	0.643439436
SMPD4BP	268.061442	0.461033325	0.287025368	1.606245914	0.108219889	0.643439436
CTTN	1754.131957	-0.21638159	0.134715452	-1.606212104	0.108227315	0.643439436
METTL21A	111.1922567	0.344572299	0.214549707	1.606025492	0.108268309	0.643439436
MBNL1-AS1	662.4079107	-0.214420793	0.133517288	-1.605940291	0.10828703	0.643439436
TMEM102	19.49109814	0.757289147	0.471609615	1.605754257	0.108327915	0.643467814
PRPF6	962.0572572	-0.201334833	0.125443561	-1.604983395	0.108497459	0.643895109
ZNF789	109.8334366	0.635794087	0.396168284	1.604858625	0.108524921	0.643895109
RPS14P3	15.19229121	0.919026266	0.572655307	1.604850691	0.108526667	0.643895109
PROCA1	120.3450339	0.441701474	0.275244253	1.604761843	0.108546226	0.643895109
DDAH2	83.81047379	-0.436288503	0.271908506	-1.604541575	0.108594728	0.643895109
SMG1P6	100.4575336	0.695996677	0.433793591	1.604442045	0.10861665	0.643895109
SNAP25	13.88078337	1.236397359	0.770732557	1.604184677	0.108673351	0.644017
POLI	384.3667491	-0.197124275	0.122906239	-1.603858971	0.108745142	0.644228204
WDR88	41.15237523	0.648884294	0.404773829	1.603078676	0.108917285	0.645033574
SEC11C	33.94530888	-0.674037623	0.420740544	-1.60202679	0.109149686	0.646025533
GOLGA7	778.6826196	-0.178881987	0.111663004	-1.601980782	0.109159859	0.646025533
CD40	8.85960262	-1.120070145	0.69924474	-1.60182849	0.109193541	0.646025533
CIR1	101.1410163	-0.486867915	0.304066698	-1.601187889	0.10933531	0.64654337
PBXIP1	713.0752441	-0.421265582	0.263124107	-1.601014773	0.109373647	0.64654337
CACNB2	101.0430115	0.575782152	0.35965225	1.600941333	0.109389914	0.64654337
PPIP5K1	191.970563	-0.380373368	0.237683076	-1.600338463	0.109523519	0.646984874
PDLIM4	1515.977249	0.412929782	0.25804193	1.600242962	0.109544695	0.646984874
AGPAT4	567.1315073	0.264292359	0.165173497	1.60008939	0.109578754	0.646984874
SMARCD2	1545.913706	-0.202251129	0.126414811	-1.599900569	0.109620643	0.646984874
PIANP	21.70272447	-0.771193262	0.482085993	-1.599700621	0.109665014	0.646984874
ASPSCR1	510.4129693	0.203758117	0.127384343	1.599553859	0.109697591	0.646984874
GABBR1	531.6261144	0.441271807	0.275902425	1.59937633	0.109737009	0.646984874
SH2D3A	16.77639169	0.890095736	0.556635856	1.599062882	0.109806631	0.646984874
LXN	15.12051476	1.441441455	0.901584758	1.598786405	0.109868071	0.646984874
SKA1	391.957266	-0.228657423	0.143028231	-1.598687346	0.109890091	0.646984874
LINC00552	2.287297434	-2.537236206	1.587155561	-1.598605876	0.109908203	0.646984874
SNORD44	14.71030425	1.115627889	0.697903764	1.598541156	0.109922594	0.646984874
FBXO25	71.24454987	-0.538362862	0.336815918	-1.598389009	0.109956429	0.646984874
ILF2	3390.120332	-0.178407528	0.111622262	-1.598314932	0.109972906	0.646984874
GPATCH2	460.5684745	0.19860925	0.124286057	1.598001048	0.110042744	0.647019009
SLC4A4	78.60804957	-0.416434365	0.260603342	-1.59796249	0.110051325	0.647019009
DDX41	812.7060917	-0.195389101	0.122287267	-1.597787786	0.110090214	0.647034172
POU2F1	772.4102624	0.205755687	0.128791914	1.597582334	0.110135961	0.64708962
MANSC1	7.8764512	-1.127143852	0.705819311	-1.596929745	0.110281369	0.647730385
GRIK5	44.42964623	-0.718245884	0.449859717	-1.596599688	0.110354969	0.647949107
DCBLD2	1971.505712	-0.273779565	0.171541186	-1.595999015	0.110489013	0.648469742
RBM11	2.035473195	2.957277702	1.853188065	1.595778517	0.110538251	0.648469742
FAM83C	2.206637309	-2.865113614	1.795506468	-1.595713335	0.11055281	0.648469742
AATF	570.9076759	-0.286726141	0.179754175	-1.595101427	0.110689556	0.649058208
MYO18B	1.945441251	2.960234984	1.856397479	1.594612694	0.110798872	0.649485495
RNF170	435.5797206	-0.283610404	0.177908668	-1.594134825	0.11090584	0.649554315
CDV3	2486.831797	-0.155534087	0.097584335	-1.593842775	0.110971254	0.649554315
LOC101926933	7.447798072	1.239120115	0.777728803	1.593664582	0.111011181	0.649554315
DNAJC18	56.53368032	-0.793453337	0.497934757	-1.593488558	0.111050633	0.649554315
GPS2	340.5146961	-0.217373095	0.136414719	-1.593472435	0.111054247	0.649554315
PHKG1	58.55910654	0.667859141	0.41913959	1.593405053	0.111069353	0.649554315
SLFN13	10.9313082	-0.883940511	0.554799987	-1.593259789	0.111101923	0.649554315
NUDC	843.1550377	-0.243756119	0.153006206	-1.593112627	0.111134927	0.649554315
EGOT	4.416786976	1.596191636	1.002103186	1.592841594	0.111195732	0.649554315
DOCK11	467.3241721	-0.246443028	0.1547459	-1.5925658	0.111257631	0.649554315
SLC5A6	954.9414512	0.322752724	0.202671408	1.592492634	0.111274057	0.649554315
PRORSD1P	44.04623223	0.594808541	0.37356882	1.592232835	0.111332398	0.649554315
SAMD4A	714.3573342	-0.175115219	0.109982836	-1.592204981	0.111338654	0.649554315
FAM136A	603.9760748	-0.235211605	0.147739689	-1.59206783	0.111369465	0.649554315
EFNA5	632.8775769	-0.20895412	0.131252874	-1.591996532	0.111385484	0.649554315
ALG11	193.0820765	0.291672354	0.183215949	1.591959404	0.111393826	0.649554315
KANSL3	1977.626563	0.14817375	0.093108648	1.591406956	0.11151802	0.650065788
GPR52	11.21740577	1.648401446	1.036143278	1.590901067	0.111631843	0.650417511

RPS18	6107.612478	-0.299725485	0.188410093	-1.590814379	0.111651356	0.650417511
TMEM106C	509.2387	-0.312373234	0.19638726	-1.590598258	0.111700017	0.650488333
HOXC6	137.1918609	-0.602371899	0.378775311	-1.590314577	0.111763915	0.650647814
CRISP2	2.656579828	2.350306485	1.478101005	1.590085168	0.111815609	0.650736169
CCDC9B	2.785907832	2.339803294	1.47172617	1.589836032	0.11187177	0.650844398
SYNGR2	62.87553294	-0.405490781	0.255077197	-1.589678677	0.111907252	0.650844398
PDE4DIP	468.537273	0.201259134	0.126625724	1.589401645	0.111969743	0.650995376
MASTL	416.8336224	0.222213003	0.139850621	1.58893111	0.112075947	0.651400316
ZNF513	139.9717921	-0.455095276	0.286445989	-1.588764703	0.112113525	0.651406265
LOC108783654	250.4720222	0.237280725	0.149381589	1.588420141	0.112191366	0.65164607
UBR5-AS1	20.83151705	0.798670159	0.503032067	1.58771222	0.112351429	0.652352455
SIRPA	56.40029941	-0.490531177	0.308984621	-1.58755855	0.112386197	0.652352455
ATF1	271.1081298	-0.307381005	0.19363991	-1.587384574	0.112425571	0.652368503
ARHGAP44	45.02986009	0.516874995	0.325694966	1.586991049	0.112514672	0.652672999
USP5	1555.856146	-0.175876779	0.110873175	-1.586287928	0.112674009	0.653236281
PCIF1	736.1608839	0.18922336	0.119305653	1.586038504	0.112730575	0.653236281
WDR54	307.0022466	-0.334526491	0.210930677	-1.585954663	0.112749594	0.653236281
CAMSAP2	1355.238986	0.152333419	0.096058379	1.585842076	0.112775138	0.653236281
NAP1L3	321.9291207	-0.215361724	0.135810275	-1.585754266	0.112795064	0.653236281
MMACHC	416.6259033	0.185413282	0.116957187	1.58530901	0.112896143	0.653609248
H2BC21	266.029984	-0.421690948	0.266033549	-1.585104398	0.112942616	0.653665939
SPOCD1	551.1232456	0.312872382	0.197434421	1.584690144	0.113036752	0.653729073
CASD1	311.0733857	-0.234154567	0.147767113	-1.584618951	0.113052937	0.653729073
LOC100507346	8.958498501	1.216491703	0.767709893	1.584572134	0.11306358	0.653729073
CLP1	152.1968025	-0.285974446	0.180510161	-1.584256772	0.113135299	0.653931567
LOC105377590	2.449215368	2.268247997	1.432537305	1.583377962	0.113335343	0.654690641
GBP4	7.367516803	1.317260577	0.832010122	1.583226624	0.11336982	0.654690641
GAPDH	18307.57556	-0.197263853	0.124598519	-1.583195808	0.113376842	0.654690641
DPP9-AS1	26.00291706	0.806195052	0.509426867	1.58255307	0.113523367	0.655324394
MED12L	96.56712158	0.42338538	0.267594609	1.582189497	0.113606317	0.65548882
LOC644215	10.53963273	1.032253051	0.652479232	1.582047367	0.113638758	0.65548882
ANXA2P2	63.21976559	0.418343217	0.264448704	1.581944667	0.113662203	0.65548882
TP53I3	366.7157334	-0.256423789	0.162167091	-1.581231979	0.113825006	0.656215337
SORD	1371.030465	-0.174524918	0.110420622	-1.580546409	0.113981788	0.656493591
FILIP1	28.25777255	0.622129714	0.393670162	1.580332406	0.114030762	0.656493591
POC1A	180.5774101	-0.260499211	0.16483913	-1.580323864	0.114032717	0.656493591
MARCKSL1	1140.278986	-0.236495177	0.149652745	-1.580292947	0.114039794	0.656493591
SPATA17	17.63125084	-0.776319048	0.491285797	-1.580178082	0.11406609	0.656493591
MPP2	228.5451014	-0.238009606	0.150633765	-1.58005482	0.114094313	0.656493591
NEU3	443.740569	0.178775261	0.113167839	1.579735579	0.114167434	0.656522552
ZNF445	1304.79824	0.168236364	0.106498181	1.579711156	0.11417303	0.656522552
EFL1	617.494277	0.354615981	0.224534422	1.57933905	0.114258309	0.656651287
MGP	95.82892141	1.306324884	0.827184956	1.579241588	0.114280654	0.656651287
LOC729732	32.44348436	0.93875354	0.594474693	1.579131208	0.114305965	0.656651287
WT1-AS	61.4736668	0.450329204	0.285273686	1.578586552	0.114430921	0.657157272
TRUB2	811.3368749	-0.190806113	0.120891242	-1.578328666	0.114490124	0.657249427
IL31RA	94.11129661	-0.444125265	0.281446169	-1.578011409	0.114562989	0.657249427
ZDBF2	326.2009809	-0.223934289	0.14190973	-1.578005177	0.11456442	0.657249427
BTN3A3	582.3735448	0.327816952	0.207795839	1.577591517	0.114659482	0.657249427
SHPK	418.6429158	0.284095194	0.180083464	1.577575128	0.11466325	0.657249427
PITPNB	1720.083475	-0.172711162	0.109493052	-1.577370972	0.114710191	0.657249427
ATG9A	1970.375657	0.321850337	0.204067842	1.577173228	0.114755671	0.657249427
PARPBP	691.1922899	0.311862759	0.197752504	1.5770357	0.114787311	0.657249427
BEX1	67.52296085	0.623750359	0.395550802	1.576915926	0.114814872	0.657249427
STXBP5L	277.759927	0.421683121	0.267444868	1.576710462	0.114862162	0.657249427
MGAT4B	1741.129224	-0.201949906	0.128112998	-1.576342056	0.114946994	0.657249427
CAPS2	74.20832544	0.427809681	0.271414461	1.576222868	0.11497445	0.657249427
COA8	324.8890311	-0.212562498	0.134855643	-1.576222499	0.114974535	0.657249427
AKNAD1	2.593102826	2.34451665	1.487485179	1.576161351	0.114988623	0.657249427
ARHGAP32	751.0208632	0.212688374	0.134963222	1.575898761	0.115049137	0.657249427
PRR11	2116.70066	-0.222924111	0.141463637	-1.575840377	0.115062595	0.657249427
ZNF572	75.31863185	-0.375982946	0.238612229	-1.575706939	0.115093358	0.657249427
DPCD	96.72808635	-0.348653627	0.221278723	-1.575631048	0.115110856	0.657249427
ADPRH	38.83408149	-0.557493228	0.354001625	-1.574832401	0.115295135	0.657905826
EPHA5	231.0992335	0.722843489	0.459002798	1.574812815	0.115299657	0.657905826
ATXN2L	2335.861159	-0.149267179	0.094798888	-1.57456677	0.115356478	0.658019347
PCDHB4	6.082718558	1.508298074	0.958187373	1.574115999	0.115460634	0.658402722
SFPQ	5064.647413	-0.269295431	0.171115114	-1.57376765	0.115541176	0.658651231
TMEM38B	519.7514332	0.23029529	0.146366685	1.573413308	0.115623147	0.658882041
EVA1C	2.01431899	-3.052467692	1.940202143	-1.573273024	0.115655613	0.658882041
GON7	153.6869044	-0.356218089	0.226548184	-1.5723723	0.115864235	0.659656579
ZBTB17	288.0127811	0.227179098	0.144491948	1.5722613	0.115889964	0.659656579
SLC35C1	412.8150665	0.292110835	0.185817433	1.572031383	0.115943273	0.659656579
LOC441666	165.8270686	0.481600545	0.306374674	1.571933277	0.115966026	0.659656579
SCT	12.86347168	0.991171965	0.630660138	1.571642008	0.116033598	0.659656579
STK33	5.742931739	1.376355819	0.87591204	1.571340222	0.116103643	0.659656579
KIAA1217	198.1479524	-0.322509167	0.205258533	-1.571233904	0.116128327	0.659656579
LINC02014	2.47273249	2.024798062	1.288749691	1.571133694	0.116151598	0.659656579
PABPC5	73.52298093	0.395519024	0.251764085	1.570990653	0.11618482	0.659656579
MBTD1	741.7676604	-0.199356285	0.126912596	-1.570815594	0.116225489	0.659656579
LCORL	415.8893827	0.232862857	0.148245958	1.57078723	0.116232079	0.659656579
ZNF503	270.7915057	-0.281883437	0.17945543	-1.570771289	0.116235783	0.659656579
SNHG26	122.051387	0.618914958	0.394073798	1.570555976	0.116285823	0.659730456
FANK1	10.16027502	1.01025055	0.643553218	1.5698011	0.116461392	0.660366868
LOC105371433	3.696889262	1.738926874	1.107926043	1.56953335	0.116523715	0.660366868
HNRNPU	6793.704636	-0.343249168	0.218698424	-1.569509111	0.116529358	0.660366868
SLC30A9	1106.407701	-0.228235523	0.145425126	-1.569436654	0.116546229	0.660366868
SPG7	683.0913468	0.273101187	0.174045678	1.569135124	0.116616457	0.660554756
CFAP300	86.46197333	-0.370314736	0.236032717	-1.568912736	0.116668274	0.660638271
PPP1R3E	284.1562362	0.469924188	0.299554134	1.56874546	0.116707261	0.66064911

TMEM107	324.4313364	-0.287263144	0.183146506	-1.568488255	0.116767229	0.660778666
ABHD2	2166.184765	-0.285692586	0.182200917	-1.568008499	0.116879149	0.661105132
DYNC1I2	1078.623865	-0.181144231	0.115533557	-1.567892795	0.116906154	0.661105132
TIMP1	300.381307	-0.366077856	0.233506577	-1.567741092	0.116941568	0.661105132
DPYSL2	7536.043889	-0.215241224	0.137311159	-1.567543566	0.116987691	0.661105132
RANGAP1	2700.512873	0.165338449	0.10551524	1.566962729	0.117123404	0.661105132
LYG1	22.46289248	0.810861135	0.517532933	1.566781713	0.117165723	0.661105132
SGCB	3987.470647	-0.151875807	0.096943533	-1.566641968	0.117198402	0.661105132
HEATR6	945.1888632	-0.240380106	0.153460196	-1.566400351	0.117254921	0.661105132
TGFBR1	1192.012816	-0.198611846	0.12681527	-1.56615088	0.117313299	0.661105132
IQSEC2	74.3791032	-0.438624526	0.280096214	-1.56597806	0.117353754	0.661105132
EHD1	903.8312837	-0.197437363	0.126097579	-1.565750628	0.117407009	0.661105132
LINC00674	700.5809775	-0.20415157	0.130407693	-1.565487165	0.117468725	0.661105132
TCP11L2	478.8748412	-0.619876025	0.395971604	-1.56545575	0.117476085	0.661105132
H2BC12	1884.543165	-0.269112723	0.171945526	-1.565104538	0.1175584	0.661105132
AGO2	4794.511563	0.13880196	0.088710734	1.564658	0.117663121	0.661105132
MGRN1	884.5493773	-0.15645736	0.100002716	-1.564531106	0.117692894	0.661105132
C9orf85	140.8615897	-0.274133506	0.175234355	-1.564382204	0.117727838	0.661105132
ESS2	159.866663	-0.299419072	0.191414366	-1.564245558	0.117759912	0.661105132
SMC3	1006.376274	-0.183198633	0.117136872	-1.563970671	0.117824456	0.661105132
PPP1R12B	473.975064	0.290937389	0.186052155	1.563741029	0.117878398	0.661105132
EMC3	270.4743958	-0.259972273	0.166263814	-1.563613076	0.117908462	0.661105132
FHOD1	870.5442384	0.235216295	0.150454908	1.563367374	0.117966209	0.661105132
RBBP9	375.3195342	-0.248667433	0.159060927	-1.56334706	0.117970985	0.661105132
SKI	1653.224168	-0.177403708	0.113483265	-1.563258761	0.117991744	0.661105132
PLAC9	43.6156304	-0.659140467	0.421714474	-1.563001767	0.118052179	0.661105132
CUTC	171.4418759	0.36144983	0.231254253	1.562997545	0.118053172	0.661105132
PRC1-AS1	18.71188994	0.77225012	0.494126655	1.562858655	0.118085844	0.661105132
LOC114483834	14.09169103	-0.803311363	0.514009349	-1.562834146	0.11809161	0.661105132
GADD45A	204.1116136	-0.42863061	0.274284796	-1.562720996	0.118118234	0.661105132
TMEM242	161.8333036	-0.336147599	0.215107787	-1.562693772	0.11812464	0.661105132
LONRF3	5.582553808	-1.401611929	0.896990805	-1.562571123	0.118153505	0.661105132
SNORD19	16.33323097	0.83877957	0.536846668	1.562419255	0.118189254	0.661105132
COX7A1	8.044419981	1.438633166	0.920791079	1.562388253	0.118196553	0.661105132
C9orf47	22.63336847	0.845118253	0.540961834	1.562251162	0.118228832	0.661105132
ENO1	29030.14226	-0.198555741	0.127127568	-1.561862184	0.118320459	0.661105132
CGB7	3.968270417	1.6890138	1.081600779	1.561587078	0.118385296	0.661105132
HSPA9	5409.082588	-0.303437263	0.194348871	-1.561301905	0.118452534	0.661105132
RCN1	3803.02515	-0.200149584	0.128195632	-1.561282397	0.118457135	0.661105132
ZNF287	154.4288391	-0.280369379	0.1796022	-1.561057598	0.118510161	0.661105132
RUFY3	324.517497	0.329533217	0.211101272	1.561019568	0.118519134	0.661105132
CEP152	855.0125802	0.262358795	0.168069355	1.561015069	0.118520195	0.661105132
GOLGA8N	119.3188272	0.435586222	0.279057708	1.560918078	0.118543081	0.661105132
IL27RA	208.3186621	-0.281499117	0.180346743	-1.560877187	0.118552731	0.661105132
ZNF70	556.4519227	0.280172403	0.179497268	1.560872798	0.118553767	0.661105132
SEM1	150.6280072	0.307346154	0.196935076	1.560647099	0.118607039	0.661105132
CTCF	775.4111498	-0.262982102	0.168528539	-1.560460346	0.118651134	0.661105132
LINC00410	13.91157215	1.162801527	0.745258678	1.560265665	0.118697114	0.661105132
NRIP3	380.5316581	0.279391614	0.179073228	1.560208731	0.118710563	0.661105132
SRP72	1988.353486	-0.177140633	0.113537884	-1.560189674	0.118715065	0.661105132
BBOF1	65.1419392	-0.504090671	0.323095965	-1.560188693	0.118715297	0.661105132
TIMM8B	139.0060028	-0.347326747	0.222619435	-1.560181606	0.118716971	0.661105132
GPR157	658.9857806	0.397914421	0.255110548	1.559772518	0.118813648	0.661436804
CC2D1A	485.1034249	-0.204926079	0.131510824	-1.558244965	0.11917519	0.663112154
MAFK	145.5505939	0.335286843	0.215177589	1.558186628	0.119189014	0.663112154
NPC1	938.5984876	0.239811672	0.15394977	1.557726734	0.11929804	0.663138884
G0S2	7.246042617	-1.338042458	0.859033186	-1.557614397	0.119324684	0.663138884
RAC3	446.5947661	-0.226767658	0.145617806	-1.55727973	0.119404086	0.663138884
PCDHGB4	4.344158008	2.0015864	1.28573542	1.556763832	0.119526567	0.663138884
STOX2	37.2808907	0.768724149	0.493824928	1.55667344	0.119548038	0.663138884
HTRA2	377.0947584	-0.232116268	0.1491177	-1.556597696	0.119566031	0.663138884
CSNK1G2	1181.204376	-0.199624455	0.128246692	-1.55656612	0.119573533	0.663138884
PDAP1	779.5052067	-0.180520074	0.115980968	-1.556462906	0.119598057	0.663138884
HNRNPA0	2203.162819	-0.165345862	0.106247675	-1.556230411	0.119653312	0.663138884
POLR2A	3513.717936	0.14851821	0.095440045	1.556141453	0.11967446	0.663138884
COQ10A	152.8620456	0.275983838	0.177352478	1.556131843	0.119676744	0.663138884
LOC105371880	4.547838236	2.134632942	1.3718332	1.556044088	0.119697609	0.663138884
FAM219A	158.4502072	0.261628679	0.168147106	1.555951127	0.119719715	0.663138884
ZMYM3	915.3596258	-0.177557342	0.114135918	-1.555665783	0.119787587	0.663138884
TRIM33	1063.009768	-0.222006795	0.142724163	-1.555495517	0.119828101	0.663138884
PPM1G	1653.332503	-0.174769282	0.112356351	-1.555490907	0.119829199	0.663138884
RAP1A	761.1741196	-0.214026798	0.137601689	-1.555408221	0.119848878	0.663138884
QSER1	2359.566361	0.135857009	0.087348408	1.555346133	0.119863656	0.663138884
LOC105374454	12.7891498	0.981846614	0.631449155	1.554910015	0.119967502	0.663507413
RPL13A	15633.51705	-0.356734836	0.229508665	-1.554341473	0.120102986	0.663892739
REC8	67.57071452	0.637256371	0.409994418	1.554304993	0.120111683	0.663892739
PSD3	6562.241223	0.476136308	0.30645915	1.553669744	0.120263214	0.664339443
COMTD1	138.5402305	-0.325989668	0.209857871	-1.553383086	0.120331641	0.664339443
VPS33B-DT	7.264643507	1.263328008	0.813319482	1.553298594	0.120351816	0.664339443
EPN3	25.57042663	0.75058118	0.483274539	1.553115505	0.120395543	0.664339443
RPPH1	98131.91835	-0.515034749	0.331640392	-1.552991615	0.120425138	0.664339443
CCSER2	922.2088017	0.210887803	0.135795508	1.552980694	0.120427747	0.664339443
IRAIN	27.19996442	0.674762263	0.434592714	1.552631331	0.120511236	0.664339443
MLH1	510.5383216	-0.262650613	0.169168198	-1.552600406	0.120518629	0.664339443
ZNF664	2625.971425	-0.178669885	0.115080743	-1.552561097	0.120528026	0.664339443
RPS6KA5	255.8914574	-0.262843711	0.169325255	-1.552300689	0.120590293	0.664477126
LOC100128079	3.664317225	2.120029193	1.366153689	1.55182335	0.120704497	0.664624914
UBE2E4P	2.7199096	-1.93295117	1.245668202	-1.55173839	0.120724833	0.664624914
TXNDC15	387.8665213	-0.224192872	0.14448426	-1.551676788	0.120739579	0.664624914
CLN8	688.1343183	0.169833827	0.109459679	1.551565179	0.1207663	0.664624914

SEMA4B	355.0761319	0.3208268	0.206805863	1.551342867	0.120819539	0.664712624
TAF1	1010.143785	-0.168504895	0.108665575	-1.550674117	0.1209798	0.665188297
NENF	364.6690727	-0.205272379	0.132376525	-1.550670553	0.120980655	0.665188297
GOSR2	1195.707027	0.211360662	0.136326465	1.550400808	0.121045345	0.665338693
GPR149	3.739033045	1.993395345	1.286081324	1.549976124	0.121147247	0.665492252
SLC41A3	293.5067506	-0.282098696	0.182002317	-1.549973101	0.121147972	0.665492252
ATP6V1D	527.2732157	-0.224368234	0.144778247	-1.549737193	0.121204607	0.665598183
SCARNA5	64.27848405	-0.725603279	0.468270688	-1.549538113	0.121252417	0.665655597
LRRC49	159.7748277	-0.255886439	0.165243225	-1.548544204	0.121491328	0.666369252
CHMP7	1509.54897	0.232133813	0.149905347	1.548535914	0.121493322	0.666369252
CACNA1C	670.3890815	0.349007138	0.225390537	1.548455151	0.121512752	0.666369252
TTC14	2342.546801	0.349680012	0.225855388	1.548247379	0.121562748	0.666369252
AFF3	316.5574102	-0.32116507	0.207469009	-1.548014675	0.121618763	0.666369252
FYCO1	842.3969133	-0.35367527	0.228485398	-1.547911915	0.121643506	0.666369252
ADCY9	1056.881434	0.153777235	0.09934513	1.54790914	0.121644174	0.666369252
APCDD1	226.6760803	0.485693686	0.313816143	1.547701405	0.121694204	0.666398025
JPT2	1747.000711	-0.249957916	0.161515677	-1.547576809	0.121724218	0.666398025
SH3RF1	124.480239	0.28475697	0.184039438	1.547260587	0.121800422	0.666508935
POC1B	522.9435502	-0.279500513	0.180651307	-1.547182346	0.121819282	0.666508935
HGSNAT	1756.215401	-0.202208792	0.13073564	-1.546699824	0.121935646	0.666679731
PIMREG	4.085991603	-1.898406536	1.227464328	-1.546608315	0.121957724	0.666679731
HUS1	305.2322439	0.215234845	0.139167579	1.546587549	0.121962734	0.666679731
ABCF2	320.3508647	-0.348017784	0.225098624	-1.546068019	0.122088141	0.666872578
FAM120A	4877.00574	-0.144416862	0.093410038	-1.546052919	0.122091788	0.666872578
SDF4	2257.00245	-0.210111997	0.135935399	-1.545675358	0.122182991	0.666872578
LINC02878	12.99792676	1.020926066	0.660524722	1.545628849	0.12219423	0.666872578
ARHGAP17	841.0898003	0.171930849	0.111250494	1.545438975	0.122240119	0.666872578
LOC100130357	22.50465916	0.789026843	0.510562656	1.545406491	0.122247971	0.666872578
RPP38	101.9451778	0.314403513	0.203469656	1.545210816	0.122295279	0.666872578
PNPO	669.324429	-0.23118818	0.149627666	-1.545089795	0.122324545	0.666872578
KDELR3	916.0148862	0.308281576	0.199528889	1.545047319	0.122334818	0.666872578
FOXK1	5628.432729	-0.232180191	0.15029035	-1.544877567	0.12237588	0.666892413
LINC01637	12.32008168	0.978174108	0.633398249	1.544327144	0.1225091	0.667414295
TEC	63.72155559	0.566601503	0.366954028	1.544066719	0.12257217	0.667553813
INKA1	7.69348226	-1.16693371	0.756009973	-1.543542747	0.122699144	0.66804117
PHF23	373.3948074	-0.248204354	0.160827274	-1.543297654	0.122758573	0.668160588
ABCB8	415.7371887	0.252749484	0.163793011	1.543102982	0.122805791	0.668213496
BRD2	3618.216638	0.145114526	0.094059613	1.54279315	0.122880972	0.668354722
CAST	689.535382	-0.219109522	0.142045713	-1.542528229	0.122945284	0.668354722
KCNC4	111.9977924	0.578794467	0.375227138	1.54251761	0.122947862	0.668354722
MAN2B2	415.781321	-0.312739426	0.202764452	-1.542377979	0.12298177	0.668354722
HDDC3	265.9729863	0.271604033	0.176121718	1.542138219	0.123040009	0.668467367
PYCARD	7.351287093	1.366162451	0.885981906	1.541975567	0.123079531	0.668478282
CREBRF	298.2479431	-0.408206907	0.264806567	-1.541528641	0.123188178	0.668858479
C19orf71	97.87606051	0.410928506	0.266597986	1.541378881	0.123224601	0.668858479
ZUP1	154.6705409	0.311877947	0.202425228	1.540706905	0.123388136	0.669542197
MSN	8358.429061	-0.201272559	0.130688895	-1.540089225	0.123538606	0.670056192
ZNF644	1312.140756	-0.174259758	0.113160713	-1.539931599	0.123577027	0.670056192
FRMD4A	298.652629	-0.211238869	0.137180999	-1.539855162	0.123595662	0.670056192
C2orf72	13.70239129	0.943595368	0.613181354	1.538852023	0.123840426	0.670791234
SPNS3	2.541780632	-1.892492001	1.229816834	-1.53884054	0.12384323	0.670791234
NMNAT1	162.7908913	-0.52423394	0.340668992	-1.538836679	0.123844173	0.670791234
C3orf80	119.0386214	-0.309367962	0.201094067	-1.538424112	0.123944951	0.671133096
IL17D	99.45288282	-0.360428532	0.234328938	-1.538130691	0.124016663	0.671317417
MBD6	254.0349343	-0.34004934	0.22121436	-1.537193786	0.124245862	0.672353857
CEP57	655.2729515	-0.246227383	0.160246528	-1.536553615	0.124402659	0.672878492
DUT	1172.827974	-0.160223578	0.104287858	-1.536358892	0.124450383	0.672878492
ARMC2-AS1	2.382817303	-2.206299711	1.43607926	-1.536335614	0.124456089	0.672878492
RTN4	4115.72795	-0.187477194	0.122059265	-1.535952182	0.124550109	0.673087035
COPS3	1100.993592	-0.157511042	0.102554914	-1.535870256	0.124570204	0.673087035
TUBE1	306.6804967	-0.340545533	0.22177444	-1.53554906	0.124649016	0.673308719
UBXN1	342.9121124	-0.322477652	0.210080994	-1.535015835	0.124779938	0.673480672
SBF2-AS1	28.67204275	-0.772336489	0.50318685	-1.53489005	0.124810838	0.673480672
LOC107986163	50.18019031	0.594166858	0.3871113	1.534873453	0.124814916	0.673480672
HAGLROS	13.65108393	0.997094737	0.649656147	1.534803822	0.124832024	0.673480672
NLE1	704.6338165	-0.220205101	0.143505803	-1.534468261	0.124914496	0.673564297
DOK3	11.70723307	1.078286356	0.702726173	1.534433179	0.12492312	0.673564297
TSC22D2	1593.167261	0.290664975	0.189476797	1.534039949	0.125019825	0.673881815
ONECUT2	282.440385	0.349140858	0.227684454	1.533441796	0.125167037	0.674244319
LINC00977	123.9803323	-0.558614686	0.36431173	-1.533342574	0.12519147	0.674244319
COG3	749.8683204	0.157572394	0.102766467	1.533305555	0.125200587	0.674244319
KATNB1	471.2823921	-0.181785951	0.118625491	-1.532435808	0.125414927	0.675132669
COLCA1	11.75095742	0.952770257	0.621889601	1.532056906	0.125508393	0.675132669
CCDC97	489.3106283	-0.175052372	0.114267714	-1.531949544	0.125534886	0.675132669
PIK3C2A	1615.197964	0.137283604	0.089617027	1.531891967	0.125549096	0.675132669
LOC400627	25.9793688	0.996977446	0.650886514	1.531722388	0.125590955	0.675132669
CDK15	9.792820463	1.040684902	0.679424782	1.531714666	0.125592862	0.675132669
LOC107984813	10.2510335	1.025578769	0.669860336	1.531033731	0.125761056	0.675832939
MAGEA10	632.2952906	-0.382860279	0.250210785	-1.530150984	0.125979361	0.676048354
PKD1	3954.994631	-0.228583773	0.149390125	-1.530113004	0.12598876	0.676048354
ITPRID2	2719.438667	-0.151433709	0.098970356	-1.530091597	0.125994058	0.676048354
FAM135A	636.4636853	0.172929491	0.113020446	1.530072628	0.125998753	0.676048354
TLCD4	90.83089667	0.38937728	0.254489129	1.530035021	0.126008061	0.676048354
LINC01132	2.99158441	2.147387986	1.403566173	1.529951368	0.126028767	0.676048354
SH3RF3-AS1	17.34694658	-0.74775314	0.488840407	-1.529646751	0.126104191	0.676105433
PWARSN	608.4705939	0.493753258	0.322885577	1.529189576	0.126217454	0.676105433
LOC100507065	11.82900677	-1.200963742	0.785416804	-1.529078236	0.12624505	0.676105433
DNM3	269.5280081	-0.429440206	0.280865431	-1.528989184	0.126267125	0.676105433
METRNL	1697.247157	0.463401891	0.303154602	1.528599231	0.126363827	0.676105433
SPDYA	33.20434569	0.663013427	0.433743781	1.52858313	0.126367821	0.676105433

GLYR1	1268.877529	-0.212810715	0.139254008	-1.528219678	0.126458005	0.676105433
IFT52	301.555909	-0.287853048	0.18835948	-1.528211102	0.126460133	0.676105433
CRYBG3	879.5929635	0.203046959	0.132877493	1.528076388	0.126493573	0.676105433
IGSF1	16.1858218	-0.937128839	0.613323282	-1.527952496	0.126524333	0.676105433
RBBP4	3367.793899	-0.171788426	0.112432641	-1.527923071	0.126531639	0.676105433
DTD1	274.7739825	-0.242562522	0.158755378	-1.527901127	0.126537088	0.676105433
C5orf66	63.89006361	0.567984145	0.371766405	1.527798471	0.126562582	0.676105433
ATP6V1G1	273.0831116	-0.212044161	0.13879976	-1.527698326	0.126587456	0.676105433
DNASE1L2	22.62340412	0.676528471	0.442866247	1.527613531	0.12660852	0.676105433
SSBP4	280.9582893	-0.227786292	0.149147659	-1.527253547	0.126697976	0.676380445
LOC107985246	89.82879306	-0.403079964	0.264048797	-1.526535884	0.126876461	0.676946704
HSD17B6	37.51970134	0.580331177	0.380177007	1.526476263	0.126891297	0.676946704
SPATA4	7.037947204	-1.134427204	0.743289338	-1.526225585	0.126953694	0.676946704
LOC284454	878.0281213	0.562454647	0.368597914	1.525930086	0.127027277	0.676946704
ATG9B	39.13804884	0.63207088	0.414242324	1.525848139	0.127047689	0.676946704
BFAR	881.8402759	-0.192428006	0.126130738	-1.525623408	0.127103679	0.676946704
PLEKHO2	242.1862305	-0.212088015	0.139046897	-1.525298437	0.127184678	0.676946704
TFG	1173.659123	-0.162442738	0.106519056	-1.525011053	0.127256341	0.676946704
APEX1	1763.830137	-0.188918233	0.123881328	-1.524993603	0.127260693	0.676946704
ITGB2-AS1	6.793879809	1.337208119	0.876900233	1.524926176	0.127277512	0.676946704
TENT5C	34.39842371	0.743956269	0.48788814	1.524850078	0.127296496	0.676946704
PLEKHB2	949.1471019	0.147980947	0.097046557	1.524844902	0.127297787	0.676946704
ALS2CL	21.33664165	0.890212374	0.583805353	1.524844489	0.12729789	0.676946704
TUBB4B	2504.431105	0.237733863	0.156033913	1.523603802	0.127607713	0.678391838
UBE2Q2P1	97.10291377	0.625704899	0.410760425	1.523284281	0.127687598	0.678614076
IRF1	80.38383403	0.367658216	0.241402291	1.523010469	0.127756086	0.678775628
DBF4	332.2602611	0.232438946	0.152659864	1.522593687	0.12786039	0.679127319
CANX	16143.32877	-0.205491303	0.134975642	-1.522432496	0.127900747	0.679139249
SNORD116-18	93.02725435	0.508205138	0.333869444	1.522167263	0.127967176	0.679289565
NDUFA13	880.7185723	-0.228894037	0.150439277	-1.521504499	0.128133283	0.679674612
DTNA	322.0152394	0.251679243	0.16542297	1.521428636	0.128152307	0.679674612
SYTL1	19.9944787	-0.732806017	0.481658825	-1.521421346	0.128154136	0.679674612
GPER1	66.33842706	0.582994837	0.383240273	1.5212254	0.128203283	0.67973297
DNPH1	356.4578633	-0.182752708	0.120191809	-1.520508844	0.128383138	0.6800448
RAB11A	1709.266957	-0.171578975	0.112856937	-1.520322806	0.128429865	0.6800448
MKRN3	218.5645663	0.267315916	0.175883569	1.51984587	0.128549718	0.6800448
MOGS	1405.394626	0.133906353	0.08811328	1.519706831	0.128584674	0.6800448
NKIRAS1	86.37566519	0.346763818	0.228200297	1.519559013	0.128621846	0.6800448
SP7	865.1094671	0.898434688	0.591256129	1.519535518	0.128627755	0.6800448
PHLDB1	461.7311507	0.206565295	0.135959051	1.519319923	0.128681987	0.6800448
SMAD9	609.4869091	0.519989511	0.342306085	1.519077615	0.128742961	0.6800448
GLIS1	123.2183918	0.344566875	0.226831557	1.51904294	0.128751688	0.6800448
MPP5	763.857661	0.22574726	0.148615189	1.519005297	0.128761163	0.6800448
RNF180	7.344315011	1.106077079	0.72834615	1.518614576	0.12885954	0.6800448
PSMC5	924.1067663	-0.177402289	0.116857105	-1.51811298	0.128985919	0.6800448
C1R	153.0783413	-0.309606894	0.203973612	-1.517877194	0.12904536	0.6800448
MFSD4A	22.00680335	0.92213215	0.607663857	1.517503697	0.129139561	0.6800448
LANCL1	749.6156743	-0.237662113	0.156618025	-1.517463347	0.129149741	0.6800448
LOC101928525	4.966834906	1.464176775	0.964889814	1.517454898	0.129151872	0.6800448
PSMF1	729.3376794	-0.201975838	0.133103569	-1.517433676	0.129157227	0.6800448
DNAJA2	1018.492318	-0.192450803	0.126854656	-1.517096886	0.129242224	0.6800448
GLB1	993.2259339	-0.302959095	0.199706019	-1.517025354	0.129260283	0.6800448
MCM4	7549.735798	-0.137114535	0.090386562	-1.51697921	0.129271933	0.6800448
ESD	1127.512037	-0.217088181	0.143128579	-1.516735388	0.129333506	0.6800448
SEMA5A	908.0868657	-0.883489333	0.582524886	-1.516655088	0.129353789	0.6800448
TXNL4B	239.5106959	-0.316968887	0.208998518	-1.516608298	0.129365609	0.6800448
RACK1	7650.625545	-0.29362701	0.193626827	-1.516458308	0.129403505	0.6800448
SNORA62	47.10603188	0.746832428	0.49249796	1.516417304	0.129413866	0.6800448
SCARNA17	128.2778928	0.479076982	0.315950644	1.516303229	0.129442696	0.6800448
ZNF526	612.9240448	0.21457063	0.141510537	1.516287293	0.129446723	0.6800448
CLDN18	13.77180477	0.917342971	0.60502213	1.516213913	0.129465271	0.6800448
ZNF709	80.6452595	0.38021363	0.250771722	1.516174263	0.129475294	0.6800448
PRELID2	308.1287249	0.257914034	0.170109911	1.516161123	0.129478616	0.6800448
BSG	4198.182727	-0.166620273	0.109898225	-1.516132527	0.129485845	0.6800448
PFKM	1290.972272	0.164480396	0.10849084	1.516076341	0.12950005	0.6800448
TOB2P1	76.56501195	-0.346599599	0.228644446	-1.515888994	0.129547424	0.6800448
KTN1-AS1	63.45990181	0.483957499	0.319270955	1.515820627	0.129564715	0.6800448
TPRG1-AS1	4.826316012	1.665113079	1.098584212	1.515689977	0.129597763	0.6800448
MMP24	60.22358074	0.573032804	0.378267762	1.514886708	0.129801097	0.68091126
TCF7L1-IT1	5.848111917	1.494109489	0.986548215	1.514481976	0.129903642	0.681042578
TMEM65	937.5443401	0.396871378	0.262056493	1.514449705	0.129911821	0.681042578
PARD6A	2.673142494	-2.081306324	1.374446505	-1.514286891	0.129953092	0.681042578
SNORD94	24.04386645	0.764279066	0.504811282	1.513989668	0.13002846	0.681042578
SSBP2	164.1518984	-0.462869548	0.305762154	-1.513822235	0.130070932	0.681042578
LOC100130872	4.784640401	1.912254813	1.263243081	1.513766306	0.130085121	0.681042578
ACTG1P20	90.58403287	0.553505538	0.365708782	1.513514483	0.130149025	0.681042578
FNBP1	521.7212842	-0.242025506	0.159927332	-1.513346736	0.130191607	0.681042578
LOC101928855	21.42977783	1.212624463	0.801308483	1.51330541	0.130202099	0.681042578
CSAD	141.1314276	0.492627316	0.325535926	1.513280953	0.130208309	0.681042578
PHOSPHO2-KLHL23	2.768859335	1.972751148	1.303964411	1.512887263	0.130308298	0.681074551
H2BU1	5.185151568	-1.418166305	0.937400676	-1.512871007	0.130312428	0.681074551
PARP3	91.90536862	-0.301259249	0.199141676	-1.512788563	0.130333375	0.681074551
EFNA4	61.97797596	0.440805715	0.29141192	1.51265506	0.1303673	0.681074551
PTCHD1	5.741699336	-1.232772195	0.815215753	-1.512203598	0.130482076	0.681298175
COPS8	1723.201383	-0.304289635	0.201273106	-1.511824612	0.130578487	0.681298175
PMS2P9	3.134096848	2.050117765	1.356287219	1.511566088	0.130644284	0.681298175
HMGXB4	782.4888977	0.209549341	0.138638853	1.511476301	0.130667142	0.681298175
TRIB1	238.902876	-0.344674446	0.228042091	-1.511450996	0.130673585	0.681298175
LOC100130449	12.34474716	1.410693296	0.933361091	1.511412153	0.130683475	0.681298175
UBE2R2	811.0166076	-0.228833533	0.151415197	-1.511298317	0.130712463	0.681298175

FRMPD4	19.14413498	-0.929378948	0.614959595	-1.511284572	0.130715963	0.681298175
CLASRP	386.3492928	0.32281828	0.213698848	1.510622461	0.130884668	0.681978007
UPF2	489.6793339	-0.247418741	0.163831034	-1.510206796	0.130990665	0.682197724
ZFHX2	17.88575344	0.707700548	0.468699515	1.50992379	0.131062871	0.682197724
GMPPB	482.7677943	0.29683726	0.196628871	1.509632126	0.131137319	0.682197724
PPP6R3	1663.578607	-0.143953351	0.095360402	-1.509571572	0.13115278	0.682197724
SFXN1	1787.997149	0.131394834	0.087041661	1.509562576	0.131155077	0.682197724
LINC00629	5.388955468	1.424741526	0.943820156	1.509547679	0.131158881	0.682197724
TM9SF1	2111.076151	-0.145508451	0.096401073	-1.509406962	0.131194815	0.682197724
ZDHHC13	287.783491	0.23383649	0.154942685	1.509180571	0.131252644	0.682299333
LOC101928042	26.901119	0.693893436	0.459857064	1.508932862	0.131315941	0.682367865
THNSL1	209.0814673	-0.267694866	0.177427347	-1.508757641	0.131360729	0.682367865
ADAM28	2.719085807	-2.283289559	1.51350304	-1.50861247	0.131397846	0.682367865
ERI3	1052.251333	-0.152229149	0.100912261	-1.508529759	0.131418996	0.682367865
RPSAP9	9.22491162	-1.039772441	0.689620743	-1.507745311	0.131619724	0.683193572
PLXDC1	5.550637335	-1.816184493	1.204829353	-1.507420523	0.131702901	0.683193572
CRYZL2P	73.37474481	-0.443401322	0.294165671	-1.507318375	0.13172907	0.683193572
RCC2	4469.548531	-0.168709643	0.111927681	-1.507309375	0.131731376	0.683193572
HDAC3	730.2840658	-0.194204838	0.128881514	-1.506847893	0.13184965	0.683568828
CTRL	36.77494469	0.641481288	0.425785543	1.506583064	0.131917561	0.683568828
ROM1	15.74100414	-0.862612803	0.572564243	-1.506578194	0.13191881	0.683568828
SBDSP1	124.874387	-0.365922635	0.242911371	-1.506403893	0.131963522	0.683601733
TTC21A	55.87210377	0.472084685	0.313444712	1.506117881	0.132036915	0.683606689
DNAJC9	191.8901834	-0.28679205	0.190432875	-1.506000733	0.132066985	0.683606689
PMVK	242.2231864	-0.308310964	0.204743586	-1.505839429	0.132108399	0.683606689
H2BC11	2069.936293	-0.319201863	0.211981252	-1.505802327	0.132117926	0.683606689
KCNAB1	205.9326429	0.367886957	0.244437915	1.5050323	0.132315773	0.684096112
COLGALT1	6896.450232	-0.201387874	0.13381556	-1.504966036	0.132332809	0.684096112
FKRP	317.5996518	-0.258309438	0.17164498	-1.504905294	0.132348427	0.684096112
KRT18	16.63404246	-0.878985633	0.584106997	-1.504836678	0.132366071	0.684096112
SNX17	2136.770689	-0.190702055	0.126753415	-1.504512166	0.132449543	0.684111354
USP12-AS1	8.162949012	1.300890685	0.864664913	1.504502687	0.132451982	0.684111354
PLCE1-AS1	11.47393653	1.173049034	0.779836669	1.504224	0.1325237	0.684111354
TMEM240	10.76452135	1.120391624	0.744895628	1.504092092	0.132557656	0.684111354
S1PR3	154.6210309	0.460575635	0.306235131	1.503993464	0.13258305	0.684111354
PRIMPOL	164.6582966	-0.250047953	0.166264712	-1.503914751	0.132603318	0.684111354
NFYC-AS1	82.16935168	0.566274643	0.376567215	1.503781053	0.132637751	0.684111354
NDUFA11	557.8990602	-0.18049035	0.120042434	-1.50355457	0.132696096	0.684214245
MLH3	57.05295433	0.455476254	0.303035359	1.503046559	0.132827038	0.684691299
NOX5	19.31103058	0.794758055	0.528859974	1.502775959	0.132896827	0.684791769
PMCHL1	3.983923756	1.519953952	1.011563917	1.502578262	0.132947833	0.684791769
CSRP2	560.4048567	0.217263409	0.144598953	1.50252408	0.132961814	0.684791769
MAPT-IT1	4.985285513	1.689980543	1.12545308	1.501600176	0.133200398	0.685821841
RPL35	2450.313902	-0.344729691	0.229650364	-1.501106661	0.133327976	0.685821841
SIAH1	197.3165184	-0.250505647	0.166880716	-1.501106011	0.133328144	0.685821841
DCUN1D5	627.3144797	-0.227744404	0.151736126	-1.50092407	0.133375202	0.685821841
TBCB	581.4430299	-0.206302346	0.137455863	-1.500862472	0.133391136	0.685821841
SLC41A1	917.6339938	0.201012383	0.133937789	1.500789169	0.133410101	0.685821841
CITED1	16.19645913	1.03726633	0.691234393	1.5006	0.133459051	0.685821841
DLGAP3	3.344578318	1.675769704	1.116763744	1.500558836	0.133469705	0.685821841
ZNF805	969.8838136	0.310275554	0.206891428	1.499702318	0.13369153	0.686720912
TGM1	15.90870573	0.842639702	0.561914995	1.49958572	0.133721749	0.686720912
IGF2R	4232.973595	-0.173382173	0.115642907	-1.499289294	0.133798598	0.68691761
DPY19L3	929.9278162	0.150426026	0.100371351	1.498694846	0.133952814	0.687262198
CSNK2A2	618.8308567	-0.171137369	0.114194316	-1.498650504	0.133964323	0.687262198
ZNF514	800.2240321	0.270069927	0.180216665	1.498584646	0.133981418	0.687262198
PPP1R13L	294.6573615	0.372925638	0.248927844	1.49812746	0.134100138	0.687673225
SSBP3-AS1	23.09438474	0.748221585	0.499561501	1.4977567	0.134196475	0.687773636
ARPC1B	897.2330022	-0.194563105	0.129903156	-1.497755025	0.13419691	0.687773636
NMRAL1	322.1867075	-0.28914754	0.193086304	-1.497504137	0.13426213	0.68791005
SMIM30	483.9431871	-0.183155572	0.122340205	-1.497100413	0.134367132	0.688133926
TAS2R50	7.040884913	1.859197103	1.241988444	1.496952014	0.134405744	0.688133926
SNORA3A	41.02114123	0.496636044	0.331826435	1.49667414	0.134478068	0.688133926
SPDYE5	23.21354979	0.878921469	0.587250619	1.496671849	0.134478665	0.688133926
LOC155060	251.5603149	0.436408658	0.291623041	1.496482089	0.134528072	0.688133926
PLPP2	81.90860403	-0.333790133	0.223055276	-1.49644581	0.134537519	0.688133926
MIR181A1HG	40.77591585	0.591743271	0.395476023	1.496281032	0.134580436	0.688155917
LOC105370941	48.0325757	0.830158047	0.554991472	1.495803249	0.134704934	0.688212848
OSGIN2	507.3917278	0.257617786	0.172229383	1.495783012	0.13471021	0.688212848
EGFEM1P	95.91531962	0.635793933	0.42510269	1.495624349	0.134751574	0.688212848
LOC100287049	20.18171959	-0.756869182	0.506139558	-1.495376463	0.13481622	0.688212848
CACNA1A	11.90066098	-0.961223793	0.642802723	-1.495363598	0.134819575	0.688212848
LRP3	950.2764662	-0.185978509	0.124381214	-1.495229893	0.134854455	0.688212848
PBK	905.5411956	0.213098518	0.142521623	1.49520131	0.134861912	0.688212848
LOC101928961	3.680066692	2.631097708	1.760120898	1.494839196	0.134956414	0.688411164
CDK13	2008.310272	-0.145170291	0.097128291	-1.494624171	0.135012554	0.688411164
VPS26B	577.7074121	-0.211799347	0.141721782	-1.494472783	0.135052091	0.688411164
SPARCL1	3.758901155	-1.759632889	1.177517169	-1.494358584	0.13508192	0.688411164
TDP1	709.4896583	0.166307285	0.111299617	1.494230529	0.135115376	0.688411164
B4GALT1-AS1	2.187682985	-2.007397884	1.343491656	-1.494164757	0.135132562	0.688411164
AP4S1	169.0225013	-0.471852241	0.3158842	-1.493750687	0.135240795	0.688765637
RPL8	9666.446502	-0.252267196	0.16891778	-1.493431868	0.135324176	0.688993378
PPIL3	275.5237799	-0.247559297	0.165817961	-1.492958271	0.13544811	0.689131408
TGS1	1101.881911	-0.215515932	0.144357831	-1.492928583	0.135455882	0.689131408
EXT1	2918.166617	-0.260972699	0.17481099	-1.492884962	0.135467302	0.689131408
C6orf201	31.69578954	0.91976245	0.6162831	1.492434971	0.135585153	0.6894621
NPEPPS	1910.173771	-0.190827406	0.127871153	-1.492341322	0.135609689	0.6894621
NOTCH4	12.7217298	0.833451933	0.558636281	1.491940216	0.135714819	0.689799791
ANKRD46	572.5618485	0.207217231	0.138925395	1.49157202	0.135811378	0.689820026
GAS5	1666.982083	-0.275740028	0.184868993	-1.491542864	0.135819026	0.689820026

MAPK11	53.23765462	-0.728511963	0.4884483	-1.491482238	0.135834931	0.689820026
LOC102724050	17.42684043	0.827601343	0.55517746	1.490696946	0.136041078	0.69067009
PSMA6	1379.924688	-0.203266025	0.136392704	-1.490299846	0.136145413	0.69076043
SCAF8	1226.663317	-0.216522887	0.145291121	-1.490269234	0.136153458	0.69076043
SOCS7	726.5453772	0.277857975	0.186471336	1.490084113	0.136202121	0.69076043
GDF11	501.1077785	0.162107891	0.108801177	1.48994612	0.136238403	0.69076043
ADGRA3	1042.154445	0.256382284	0.17212146	1.489542817	0.136344487	0.69076043
FIS1	378.7546218	-0.233667568	0.156873817	-1.489525612	0.136349014	0.69076043
TMEM185B	1020.486312	-0.201083672	0.135003224	-1.489473112	0.136362829	0.69076043
AGAP1	948.7759935	0.294381454	0.197644436	1.489449742	0.136368978	0.69076043
MIR573	6.613165436	1.176850242	0.790259929	1.48919387	0.136436325	0.690905173
STX1A	60.45297814	-0.562306579	0.377726986	-1.488658741	0.136577255	0.691244535
ZNF442	11.46153949	1.094662415	0.735601943	1.488117896	0.136719805	0.691244535
LAMTOR2	193.4692339	-0.283166704	0.190293139	-1.488055245	0.136736325	0.691244535
WDR89	480.3247472	0.175438079	0.117933121	1.487606512	0.136854695	0.691244535
PPP1R3F	92.60768009	0.390540314	0.262543938	1.48752364	0.136876564	0.691244535
LINC00597	6.190872472	2.066836902	1.38945599	1.487515198	0.136878792	0.691244535
NECAP1	434.2511337	0.19132183	0.128628821	1.487394724	0.136910589	0.691244535
SNORA29	5.453352084	1.568263502	1.054494333	1.487218521	0.136957106	0.691244535
CEBPZOS	1125.97966	-0.206851176	0.139097061	-1.48709955	0.13698852	0.691244535
PLCB3	795.5592769	-0.178736934	0.120193247	-1.48707967	0.13699377	0.691244535
SNORA44	9.211821206	1.161287753	0.780932599	1.487052473	0.137000953	0.691244535
FAM53A	24.52061829	-0.546568548	0.367558668	-1.487023966	0.137008481	0.691244535
BCLAF3	116.372647	-0.380499689	0.255887073	-1.486982849	0.137019341	0.691244535
NEB	106.5718524	0.853312722	0.573894665	1.486880387	0.137046405	0.691244535
TOM1L1	92.44609196	-0.436526958	0.293678076	-1.486413162	0.137169871	0.691540368
FAM21EP	4.641121895	-1.559810399	1.049472827	-1.486279929	0.137205093	0.691540368
CMC2	184.3088767	0.252958343	0.170216416	1.486098398	0.137253096	0.691540368
FOXC2	275.4895296	-0.464998356	0.31290449	-1.486071215	0.137260285	0.691540368
TMEM212-AS1	70.39292998	-0.39921143	0.268700456	-1.485711771	0.137355376	0.691701151
B3GNT2	276.3837453	0.213082413	0.143426371	1.48565715	0.13736983	0.691701151
TTC28-AS1	103.2100068	0.352018208	0.237011195	1.485238742	0.137480593	0.69170656
SCPEP1	911.3109386	-0.30165884	0.20311504	-1.485162496	0.137500784	0.69170656
LOC100507477	8.94879594	-1.434562731	0.965934566	-1.485155187	0.13750272	0.69170656
SLC30A5	1063.120015	0.18285994	0.12313248	1.485066649	0.13752617	0.69170656
ATP5F1E	364.2746023	0.190377999	0.128213379	1.48485284	0.137582811	0.691761958
ZNF75A	210.8775428	-0.407827441	0.27468084	-1.484732032	0.137614823	0.691761958
RPS29	913.1965378	-0.276023272	0.185931659	-1.484541543	0.137665311	0.691768141
THEM6	378.8749521	0.231622302	0.156060404	1.48418366	0.137760203	0.691768141
TGFB1	1681.286021	0.280781673	0.189187199	1.484147316	0.137769843	0.691768141
LRRC8C	347.5248303	-0.237064762	0.159735504	-1.484108146	0.137780232	0.691768141
SPHK1	724.6552807	0.312450948	0.210573755	1.483807645	0.137859958	0.691768141
SEPTIN8	1510.249022	-0.150005367	0.101095537	-1.483798111	0.137862488	0.691768141
SCARNA6	53.84571807	-0.650068273	0.438139152	-1.48370277	0.137887791	0.691768141
CYB561D2	96.28324315	0.325842901	0.219650698	1.48345944	0.137952385	0.691897412
CHRNA3	20.36225038	0.750381855	0.505979376	1.483028539	0.138066828	0.692210542
KIAA1109	1963.555778	0.12821502	0.086460492	1.482931892	0.138092507	0.692210542
CAMKMT	158.4140588	-0.264293631	0.178263776	-1.48259864	0.138181079	0.692366376
ZDHHC4	388.5483072	-0.205891262	0.138879006	-1.482522573	0.138201302	0.692366376
FOXD2	20.33706311	0.612509189	0.413303403	1.481984382	0.138344451	0.692888733
NME1-NME2	42.70624495	0.476464221	0.321583626	1.48161841	0.138441858	0.693007693
DBT	1010.056658	-0.237557367	0.160350715	-1.481486168	0.138477068	0.693007693
ZFAT	324.3458281	0.269612596	0.181991526	1.481456867	0.138484871	0.693007693
USP24	2582.019801	0.168870551	0.114012973	1.48115206	0.138566058	0.693141656
MORC4	433.0248073	-0.213857974	0.144398847	-1.481022731	0.138600516	0.693141656
PTTG1	497.6076	0.227363282	0.153538976	1.480818014	0.138655075	0.693141656
HAUS6	1025.621692	0.142254878	0.09607388	1.480682135	0.138691297	0.693141656
CCDC93	571.0646743	0.173932823	0.117484641	1.480472873	0.138747095	0.693141656
PAXIP1-AS2	30.71689633	0.599081928	0.404754874	1.480110474	0.138843767	0.693141656
LOC101928489	61.10637579	-0.505988328	0.341865992	-1.480077984	0.138852436	0.693141656
PLEKHJ1	447.315555	-0.24436326	0.165103767	-1.480058658	0.138857593	0.693141656
TUBGCP6	1008.846416	0.332767029	0.224836021	1.480043222	0.138861712	0.693141656
DERL1	2307.765345	0.175836027	0.118821939	1.479827961	0.138919165	0.693234252
ACOT6	6.018254154	1.230200288	0.831456996	1.479571755	0.138987569	0.693381432
CNN1	5.054353379	1.521340956	1.028707424	1.47888595	0.139170799	0.693571331
CSKMT	77.67712417	0.508563503	0.343925036	1.478704513	0.139219306	0.693571331
PHF5A	229.6771131	-0.222362415	0.150384527	-1.478625623	0.139240401	0.693571331
TRPC6	3.629665463	-1.367250197	0.924723588	-1.478550148	0.139260585	0.693571331
ADAMTS14	114.3210482	-0.485527687	0.328411592	-1.478412149	0.139297496	0.693571331
OTP	3.599215451	1.775747606	1.201221539	1.478284852	0.13933155	0.693571331
LOC105375713	26.47605865	0.938595111	0.634979028	1.478151356	0.139367271	0.693571331
FAM234B	753.7610445	0.181421876	0.122746223	1.478024101	0.139401327	0.693571331
KCNRG	30.12736615	0.974732159	0.659541859	1.47789279	0.139436477	0.693571331
RPL24	2757.401017	-0.303383787	0.205288068	-1.477844233	0.139449476	0.693571331
ZNF302	512.810923	-0.211315416	0.142990514	-1.477828215	0.139453764	0.693571331
PTPDC1	529.2081856	0.196087406	0.132708774	1.4775768	0.139521088	0.693712551
CCDC183	41.46085505	0.608502802	0.41195975	1.477092851	0.13965075	0.693774897
HAUS2	1046.375025	-0.159974221	0.108346622	-1.47650401	0.139808639	0.693774897
FAM230J	1.966435255	2.421031188	1.639742327	1.476470509	0.139817626	0.693774897
DEK	4366.468868	-0.187736866	0.127171351	-1.476251253	0.139876455	0.693774897
CD19	6.412133175	1.375262606	0.931824252	1.475881963	0.139975582	0.693774897
CKLF	144.4661455	-0.298321617	0.202141652	-1.475804783	0.139996306	0.693774897
HIF1A-AS1	3.985126754	-1.516622037	1.027687545	-1.475761815	0.140007845	0.693774897
MBD5	226.9818493	-0.308751017	0.209214852	-1.47576051	0.140008195	0.693774897
ST8SIA4	285.2168292	0.27730566	0.187914542	1.475700906	0.140024203	0.693774897
LOC100129148	14.20463913	1.149636941	0.77918539	1.475434417	0.140095788	0.693774897
SCHLAP1	125.5174542	-0.412290467	0.279453623	-1.47534486	0.140119851	0.693774897
RASGRP1	100.997803	0.635313005	0.43070242	1.475062538	0.140195731	0.693774897
LOC102723313	36.54096335	1.319618048	0.894664271	1.474986865	0.140216075	0.693774897
IDI2-AS1	22.69461763	0.849025535	0.575629063	1.474952517	0.14022531	0.693774897

BCDIN3D-AS1	52.48336793	0.500519717	0.339415564	1.47465164	0.140306224	0.693774897
HPCAL1	547.5181005	0.361851444	0.245397275	1.47455364	0.140332587	0.693774897
PFN1	6686.56632	-0.192387513	0.130474117	-1.474526269	0.14033995	0.693774897
MIR4326	4.645034698	1.700697633	1.153518748	1.4743563	0.140385684	0.693774897
IGFBPL1	271.8061535	0.339545899	0.230312671	1.474282317	0.140405594	0.693774897
AMOT	1344.973569	0.229473622	0.155656862	1.474227467	0.140420357	0.693774897
METAP1D	165.3104325	-0.312506791	0.212012127	-1.47400432	0.140480428	0.693774897
EIF2AK3	572.5989483	0.299732867	0.203352265	1.473958829	0.140492677	0.693774897
ZNF217	1273.64042	-0.198094751	0.134399739	-1.47392214	0.140502556	0.693774897
PCF11	491.8226332	-0.252303865	0.171192098	-1.473805553	0.140533953	0.693774897
CCDC122	56.05204825	-0.399552957	0.271143325	-1.473585815	0.140593143	0.693774897
ZNF621	933.9351269	0.203244041	0.13792698	1.473562608	0.140599395	0.693774897
PITPNM3	18.71760693	-0.740450866	0.50257438	-1.473315982	0.140665853	0.693774897
KLHL13	240.3599835	0.269688896	0.183070941	1.473138747	0.140713627	0.693774897
HMMR	643.9302559	-0.250841949	0.170277393	-1.473137126	0.140714065	0.693774897
YWHAE	8259.853358	-0.216845926	0.14720374	-1.473100654	0.140723897	0.693774897
ELP1	1875.497667	0.128999855	0.08757396	1.473038962	0.14074053	0.693774897
COQ4	201.9894689	-0.30982433	0.210394849	-1.472585144	0.140862933	0.693940949
RABGAP1L	743.3302596	0.201676783	0.136962403	1.472497408	0.140886606	0.693940949
SNORA46	5.216830299	1.458916602	0.990788084	1.472480974	0.140891041	0.693940949
USP8	1313.703161	-0.147591062	0.100257499	-1.472119924	0.140988496	0.694019592
SFXN3	281.4567154	-0.443260093	0.301107603	-1.472098643	0.140994242	0.694019592
AKR7A2	1132.463184	-0.231261239	0.157134544	-1.471740283	0.141091025	0.694019592
RASSF3	570.185623	0.263699505	0.179175933	1.471735074	0.141092432	0.694019592
HDGFL1	7.107259468	1.155374197	0.785162364	1.471509907	0.14115327	0.694019592
STOM	1084.613888	-0.217992868	0.148164833	-1.471286159	0.141213745	0.694019592
PPM1M	250.2351021	-0.441120202	0.299853593	-1.471118612	0.141259042	0.694019592
KIAA0930	1029.410911	0.237706189	0.161589253	1.471051969	0.141277063	0.694019592
BCL2L2	605.9695009	0.163544843	0.111180257	1.470988171	0.141294316	0.694019592
RNF135	374.8336862	0.366297709	0.249042706	1.470822874	0.141339025	0.694019592
CCDC191	45.94599385	-0.463299464	0.315006227	-1.470762877	0.141355255	0.694019592
RIC1	964.3458867	0.148887498	0.101236338	1.470692256	0.141374361	0.694019592
CNTNAP5	3.411527658	1.759578009	1.197059391	1.469917051	0.141584221	0.694255947
TPX2	2327.290593	-0.196730749	0.133852618	-1.469756449	0.141627728	0.694255947
MALAT1	788.1770349	0.762607953	0.518869328	1.469749536	0.141629601	0.694255947
ITSN1	454.0317377	-0.187534071	0.127597439	-1.469732243	0.141634287	0.694255947
PLXNA3	1027.715958	0.36134328	0.245898647	1.469480554	0.141702492	0.694255947
ARHGEF26	252.6462354	0.27456166	0.186844515	1.469465993	0.141706439	0.694255947
LOC100128653	39.72084902	0.544836923	0.37077645	1.469448568	0.141711162	0.694255947
ZNF35	154.0958209	0.323864226	0.220420625	1.469300918	0.141751188	0.694255947
WDR34	491.0359613	-0.274119145	0.186580459	-1.469173925	0.141785621	0.694255947
PDGFC	3322.220225	0.27133215	0.184697517	1.469062251	0.141815905	0.694255947
RSC1A1	4.863594641	1.869989111	1.273125248	1.468817867	0.141882197	0.694255947
MAFG-DT	127.2342321	0.341144565	0.232262468	1.468789031	0.14189002	0.694255947
RPE	605.0989632	0.213321116	0.145291765	1.468225794	0.1420429	0.694577379
DSEL	3517.60772	0.180917012	0.12322444	1.468190992	0.142052351	0.694577379
PSMB6	679.807614	0.152944701	0.104193297	1.467893862	0.142133056	0.694577379
FRK	268.2775716	0.290812279	0.198141755	1.46769811	0.142186244	0.694577379
PERM1	8.012683052	1.070860371	0.729619926	1.467696169	0.142186772	0.694577379
TMEM220	41.61417694	0.446085318	0.303937876	1.467685843	0.142189578	0.694577379
UGT8	307.1612034	0.250804535	0.17090595	1.467500307	0.142240006	0.694633297
C20orf144	14.04402609	0.926944064	0.63176495	1.467229329	0.142313681	0.694794953
SIX1	331.9701942	0.178808287	0.121879414	1.46709179	0.142351087	0.694794953
FPR2	3.114419534	-1.591150786	1.084780774	-1.466794788	0.142431888	0.694813699
HDAC9	10731.81914	-0.318922465	0.217445459	-1.466677974	0.142463678	0.694813699
MAN2A1	2578.777833	-0.163350996	0.11138437	-1.466552227	0.142497904	0.694813699
DNAJB12	342.0733056	-0.187652546	0.127959064	-1.46650452	0.142510891	0.694813699
NSMAF	1942.134606	0.151158455	0.103104083	1.466076328	0.142627494	0.694939584
SHC2	2.277960077	-1.934601922	1.319804233	-1.465824911	0.142695993	0.694939584
PPP3CB-AS1	151.6103391	-0.322538282	0.220041964	-1.465803503	0.142701826	0.694939584
HSD17B7P2	41.96446055	0.652637629	0.445332153	1.465507545	0.142782495	0.694939584
RARG	694.4301043	0.280397552	0.191333056	1.465494556	0.142786037	0.694939584
ITGA7	89.31615104	-0.376173627	0.256750742	-1.465131605	0.142885015	0.694939584
LY6E	1777.868393	-0.16176372	0.110418691	-1.465003057	0.142920084	0.694939584
PHKA2	543.9100441	0.235461634	0.160732593	1.464927738	0.142940634	0.694939584
USP1	1719.783403	-0.191363287	0.130635148	-1.464868298	0.142956854	0.694939584
CNOT10	406.7189589	-0.218399576	0.149107774	-1.464709522	0.143000186	0.694939584
TMEM265	34.01250124	0.524885123	0.358360251	1.464685666	0.143006697	0.694939584
ZNF711	458.3606002	-0.218997969	0.149538477	-1.464492437	0.143059448	0.694939584
SELENOI	1513.890956	0.199055479	0.13593303	1.464364316	0.143094433	0.694939584
RIMBP3	8.848890432	1.047821019	0.715619565	1.464215165	0.143135168	0.694939584
MTRF1	216.9324418	0.243933341	0.166604844	1.464143149	0.14315484	0.694939584
LINC00960	114.2143475	0.308888915	0.210972145	1.464121791	0.143160674	0.694939584
ERAP2	49.32969947	0.472704579	0.322978535	1.463578932	0.143309032	0.695205741
SLC45A1	3.818984353	-1.513077244	1.03386877	-1.463509962	0.14332789	0.695205741
ZNF267	281.4532383	0.209974487	0.143474888	1.463492949	0.143332542	0.695205741
IGSF3	400.0522344	-0.180310462	0.123231703	-1.463182429	0.143417467	0.695365353
JCAD	353.6555582	0.504138434	0.344571669	1.4630873	0.143443492	0.695365353
FZD2	450.1572306	-0.362203162	0.247614403	-1.462770979	0.143530057	0.69559576
TAS2R46	6.329954088	1.594671738	1.090349268	1.462532956	0.14359522	0.695605757
MRPL40	230.6156757	-0.265889566	0.181828806	-1.462307163	0.143657057	0.695605757
ZNF565	79.86596317	0.369166021	0.252477946	1.462171357	0.143694259	0.695605757
UMPS	450.0619005	0.199544384	0.136477091	1.462109014	0.143711339	0.695605757
FKBP1AP1	47.86118661	0.865859687	0.592222718	1.46205078	0.143727295	0.695605757
TBC1D26	5.218761209	1.516209419	1.037250613	1.461758035	0.143807527	0.695649666
TMEM241	410.950223	0.197701943	0.135272687	1.461506736	0.143876428	0.695649666
HAUS5	589.8779433	-0.199995984	0.136844138	-1.461487401	0.143881731	0.695649666
IRS2	9.691668108	-0.928650207	0.635431548	-1.461448067	0.143892518	0.695649666
PPT1	3783.297262	-0.178059597	0.121853748	-1.461256632	0.143945027	0.695663923
CLTCL1	450.6510985	0.183286497	0.125448801	1.461046223	0.144002757	0.695663923

UQCRH	980.6321833	-0.18878069	0.12921362	-1.460996835	0.14401631	0.695663923
LOC220729	354.1710048	0.316782969	0.21684569	1.46086818	0.14405162	0.695663923
PNO1	406.3996782	0.271434203	0.185838068	1.460595272	0.144126545	0.695755898
LOC107985946	23.08028041	0.683590537	0.46804779	1.4605144	0.144148753	0.695755898
LINS1	307.6851178	0.339735451	0.232707728	1.459923369	0.144311136	0.696351053
SLC25A27	233.4545937	0.41247878	0.282639292	1.4593823	0.144459915	0.696624984
GOLM1	1247.998044	-0.199984354	0.137037344	-1.459342019	0.144470996	0.696624984
STK35	1003.181208	0.203974456	0.13978151	1.459237742	0.144499684	0.696624984
CYP3A5	19.26025291	0.988234124	0.677302327	1.459073866	0.144544778	0.696624984
GUCY1A1	119.6104035	-0.314290645	0.215427603	-1.458915392	0.144588396	0.696624984
SLCO4C1	211.2423775	0.368244563	0.252418653	1.458864307	0.144602459	0.696624984
LOC339874	3.818725008	1.800818486	1.234762542	1.458433039	0.144721219	0.697008684
ABCF1	1563.973437	-0.208377085	0.142920415	-1.45799384	0.144842241	0.697307832
TGFA	13.20714584	-1.110898626	0.762007526	-1.457857813	0.144879739	0.697307832
CMIP	558.7882425	0.211898404	0.145358557	1.45776354	0.144905731	0.697307832
CMSS1	380.0097427	-0.228650122	0.156867895	-1.457596681	0.144951745	0.697307832
FAM199X	635.8903991	0.179105336	0.122885483	1.45749792	0.144978985	0.697307832
DLL3	58.57099514	0.390805545	0.268187271	1.457211382	0.14505804	0.697499802
RPA1	2773.248293	-0.142153621	0.097591498	-1.456618908	0.145221605	0.697931468
PKD1P1	229.1849675	-0.389210937	0.267204637	-1.456602476	0.145226144	0.697931468
COL27A1	1052.293816	0.36045609	0.247520783	1.456265957	0.145319113	0.698159223
MAPRE3-AS1	3.442686539	1.849101895	1.269865084	1.456140434	0.145353802	0.698159223
KLHL8	762.7816629	0.300082941	0.206100128	1.456005604	0.14539107	0.698159223
REPIN1	384.6931871	0.192770158	0.132436496	1.455566734	0.14551243	0.698553744
SDHAP3	10.14487288	1.213456934	0.833785427	1.455358772	0.145569964	0.698641734
CYP2T1P	43.69575613	0.47139101	0.323942329	1.45516954	0.145622331	0.698704885
ATP5PB	2019.649924	-0.204805997	0.140767804	-1.454920744	0.145691204	0.698847176
NUDT9P1	2.357302253	-1.878960922	1.29165212	-1.454695807	0.145753494	0.698957821
CLSTN3	205.2510882	0.380588605	0.261674552	1.454434914	0.145825766	0.699108831
XKR8	162.0775454	0.353399433	0.243003296	1.454298928	0.145863447	0.699108831
NECTIN4	34.26597756	1.320204746	0.907915555	1.454105217	0.145917137	0.699178109
MIR6728	4.563822611	-1.464085436	1.007010677	-1.453892664	0.145976067	0.69927245
DNAJC7	539.0869131	-0.228887779	0.157452975	-1.453689769	0.146032336	0.699353998
HELZ	1732.715893	0.149933621	0.103170127	1.453265834	0.14614996	0.699729254
SNAP29	309.5184362	-0.361246419	0.248676013	-1.452678991	0.146312903	0.700321227
RDM1	8.300153042	-1.160348343	0.799057986	-1.452145355	0.146461193	0.700598572
PRMT6	689.1789503	-0.18654505	0.128467356	-1.452081339	0.14647899	0.700598572
SNORA68B	3.92039186	1.778279216	1.224671336	1.452046082	0.146488792	0.700598572
SKIL	2442.135247	0.389700408	0.268409396	1.451888101	0.146532722	0.700620633
RPL7A	15251.69931	-0.300077924	0.206805502	-1.451015189	0.146775631	0.700888675
ENTR1	1516.952035	0.202135801	0.139316785	1.450907735	0.146805554	0.700888675
SLC27A6	18.75125108	-0.656026473	0.452153498	-1.450893281	0.146809579	0.700888675
COX7A2	930.9084169	0.231518377	0.159571224	1.450877997	0.146813836	0.700888675
NXPH2	63.11862891	-0.409669883	0.282360818	-1.450873693	0.146815035	0.700888675
TBC1D8	231.3049018	0.444276549	0.306237115	1.450759976	0.146846708	0.700888675
LPAR6	13.00741266	1.388923812	0.957503969	1.450567159	0.146900426	0.700888675
AZI2	149.4307743	-0.317369834	0.218804815	-1.450470066	0.146927481	0.700888675
L3MBTL2-AS1	35.7106826	0.75976791	0.523827867	1.450415218	0.146942766	0.700888675
NKAIN1	4.827655345	-1.212446825	0.836113399	-1.450098547	0.14703104	0.701109769
PAX9	422.0700893	0.181554431	0.125212829	1.449966695	0.147067807	0.701109769
ZNF83	359.7154882	0.303928706	0.209639117	1.44977097	0.147122397	0.701182434
CACNB4	9.660780271	0.990622796	0.68352701	1.449281129	0.147259089	0.701646247
ZNF438	73.75917476	0.395588343	0.273022933	1.448919835	0.147359971	0.701939238
DPY19L2P4	26.76025263	-0.663184854	0.457763926	-1.448748614	0.147407799	0.701961102
ADAM22	307.2908277	0.212055061	0.146384046	1.448621389	0.147443345	0.701961102
ALDH8A1	4.311859068	-1.388343318	0.958756253	-1.448067028	0.147598306	0.702511167
ARV1	184.9527038	-0.237435829	0.164032196	-1.447495275	0.147758259	0.703029608
LINC00888	141.0831612	-0.30942761	0.213782322	-1.447395683	0.147786134	0.703029608
PARK7	1550.826456	-0.193852915	0.1340146	-1.446505943	0.148035346	0.703517883
CHROMR	383.7690183	-0.301771655	0.20862234	-1.446497315	0.148037765	0.703517883
ATIC	2214.501629	-0.17419255	0.120424699	-1.446485236	0.14804115	0.703517883
MRPL13	621.5335625	0.216437742	0.149632154	1.446465462	0.148046693	0.703517883
SLC38A10	1361.140391	-0.144714171	0.100082005	-1.445955945	0.148189559	0.703801629
RAC1	3193.615474	-0.166019381	0.114825471	-1.445841063	0.148221786	0.703801629
FUT4	303.6249624	0.194843024	0.134762056	1.445830003	0.148224888	0.703801629
LAMC2	31.0268	-0.799151738	0.553051493	-1.444986135	0.14846178	0.704335055
MIR4500HG	21.8193493	0.735198091	0.508840411	1.444850045	0.148500011	0.704335055
H1-0	106.8402576	-0.488186179	0.337891154	-1.444803079	0.148513206	0.704335055
ZBTB41	988.8615384	0.256739602	0.177723311	1.444602851	0.148569472	0.704335055
LONP1	1575.664423	-0.262553865	0.181758816	-1.444517909	0.148593346	0.704335055
AMOTL2	557.0811947	0.499483098	0.345793215	1.444456042	0.148610736	0.704335055
LDLR	3609.171949	0.337030733	0.233328917	1.444444766	0.148613906	0.704335055
ANKRD52	1329.644545	0.223916189	0.155047275	1.444180102	0.148688321	0.704379239
QRICH1	682.8784709	-0.242804534	0.168148496	-1.443988741	0.148742143	0.704379239
CACNB3	329.9487282	-0.539544338	0.373682582	-1.443857338	0.148779109	0.704379239
ARHGAP27P1	73.01989254	0.444175525	0.30763286	1.443849414	0.148781339	0.704379239
TLE3	1464.874582	-0.218539529	0.15140903	-1.443371829	0.148915756	0.704706669
LDHAL6A	10.62501815	1.015230008	0.703397803	1.443322689	0.148929591	0.704706669
RUBCN	1310.6743	0.219427202	0.152051609	1.443110033	0.148989477	0.704726896
A4GNT	2.37775299	2.187495315	1.516020148	1.442919685	0.149043097	0.704726896
SOX2	245.7539414	-0.217203916	0.150534327	-1.442886287	0.149052507	0.704726896
ZNF71	207.4344066	-0.255631845	0.177186008	-1.442731551	0.149096108	0.704746059
SYN2	6.80742118	-1.111960455	0.770972175	-1.442283511	0.14922241	0.705071424
HSP90AB4P	16.93047196	1.02104225	0.708022921	1.442103385	0.14927321	0.705071424
TCF19	1446.065949	-0.206791403	0.143399362	-1.442066406	0.149283641	0.705071424
FAM102B	727.8288336	0.306869576	0.212877181	1.441533441	0.149434037	0.705594736
EEFSEC	120.0832026	-0.34911339	0.242325965	-1.440676776	0.149676019	0.706550108
LURAP1L-AS1	2.516727341	-2.162014331	1.500922011	-1.440457476	0.149738013	0.706655558
MYSM1	1744.144043	0.246566595	0.171191013	1.440301042	0.149782247	0.706677161
LOC101928491	5.352689821	1.981558398	1.375948136	1.440140326	0.149827702	0.706704513

JARID2	1243.289979	0.300927244	0.209060141	1.43942907	0.150028993	0.707209625
WDR31	184.3564432	-0.502154501	0.348867787	-1.439383401	0.150041925	0.707209625
LOC100128966	9.160575496	0.910487284	0.632611943	1.439250862	0.150079459	0.707209625
NTMT1	261.1946353	0.197396092	0.137164611	1.439118225	0.150117029	0.707209625
IL11	57.46481512	0.558843991	0.388339317	1.439061066	0.150133222	0.707209625
MED29	599.4829418	-0.311009487	0.21617574	-1.438688204	0.150238883	0.707367201
DAP3	1323.21905	-0.166966996	0.116057069	-1.438662871	0.150246064	0.707367201
TRPC4AP	1716.884338	-0.131926845	0.091716153	-1.43842541	0.150313387	0.707497242
AGAP2	96.95719973	-0.49590081	0.344843076	-1.43804775	0.150420506	0.707814477
M1AP	11.96427112	0.863455965	0.600652768	1.43752932	0.150567648	0.708216424
CARD14	13.73169929	0.789218064	0.549033958	1.43746676	0.150585411	0.708216424
ZNF628	115.955658	0.343504979	0.239007757	1.437212682	0.150657571	0.708368841
CFB	53.27768426	0.468381709	0.325953818	1.436957271	0.150730135	0.708487285
HSPE1	698.8716676	0.315219663	0.219383334	1.436844167	0.150762277	0.708487285
VDR	85.91124614	0.379026632	0.263869583	1.436416535	0.150883851	0.70882774
OAZ1	4953.422693	-0.207055294	0.144157848	-1.436309547	0.150914279	0.70882774
SCARNA7	147.2258708	0.530678636	0.369549126	1.436016485	0.150997651	0.708831571
USP48	1367.458379	0.164793494	0.11476009	1.435982619	0.151007287	0.708831571
FOXG1	255.6955797	0.212255806	0.147822056	1.435887253	0.151034426	0.708831571
COX7A2L	764.3618829	-0.295359373	0.20576817	-1.435398745	0.151173503	0.70929748
COX4I1	1089.216337	-0.200383956	0.139676332	-1.434630715	0.151392357	0.709758596
ZNF197	2.15279271	2.418289254	1.685949766	1.434378	0.151464422	0.709758596
RAPGEF4-AS1	3.309067807	1.901259081	1.325531064	1.434337628	0.151475937	0.709758596
ATP6V1B1-AS1	54.47314924	0.623828499	0.434928232	1.434325146	0.151479498	0.709758596
TYSND1	499.9925031	0.209461784	0.146039169	1.434284967	0.151490959	0.709758596
OGDH	1391.541254	0.236566294	0.16495893	1.434092072	0.15154599	0.709758596
SAV1	657.2157153	-0.270283204	0.188488384	-1.433951517	0.151586099	0.709758596
PTGER4	20.09421108	-1.018015983	0.709959289	-1.433907548	0.151598648	0.709758596
KIF2A	1393.93252	-0.157667741	0.109971399	-1.433715884	0.151653357	0.709758596
TENM3-AS1	585.342674	0.227466219	0.158675587	1.433530027	0.151706424	0.709758596
NIT1	377.1704745	-0.203124405	0.141696461	-1.43351784	0.151709904	0.709758596
HLA-DMA	134.059438	0.362728594	0.253121912	1.433019338	0.151852311	0.710018723
PAK3	262.5427197	-0.447066644	0.3120256	-1.432788346	0.151918333	0.710018723
RHOF	39.25280775	-0.512954142	0.358037602	-1.432682321	0.151948644	0.710018723
SERGEF	153.4373031	-0.268070284	0.187115337	-1.432647312	0.151958654	0.710018723
ZNF518A	614.7263637	-0.215795678	0.150635472	-1.432568808	0.151981101	0.710018723
IL7R	41.69871629	0.839258793	0.585959309	1.432281697	0.152063219	0.710018723
SPTBN5	32.61227823	0.896099726	0.62575584	1.432027747	0.152135881	0.710018723
ABCD1	170.3081129	0.461324304	0.322161966	1.431963897	0.152154154	0.710018723
MTMR14	257.6165276	-0.252762423	0.176530506	-1.43183424	0.152191266	0.710018723
CTDSPL	320.9092454	-0.209043997	0.146011796	-1.431692526	0.152231836	0.710018723
FAM218A	39.34919934	0.591044471	0.41283192	1.431683071	0.152234543	0.710018723
RPLP0P2	3.673206578	-1.50046355	1.048064933	-1.431651325	0.152243633	0.710018723
EHMT2	1383.097537	-0.161906767	0.113114174	-1.431357015	0.152327921	0.710225943
PXDC1	695.3154207	0.321147514	0.224416119	1.431035863	0.152419937	0.710469076
KCTD21	77.94184554	-0.359397731	0.251171835	-1.430883885	0.152463496	0.710486272
NDUFS6	64.20115363	0.409325065	0.286106092	1.430675808	0.15252315	0.710578439
SOX2-OT	19.61191151	-0.684544106	0.478739854	-1.429887443	0.152749327	0.711446157
ITPR1	705.8987566	0.3927484	0.274805092	1.429188949	0.152949933	0.712194357
PGP	441.8995689	0.264649078	0.185244721	1.428645719	0.153106087	0.712659122
TANGO6	460.7754091	-0.314206747	0.219946491	-1.428559944	0.153130754	0.712659122
LGR4-AS1	6.716998011	1.310128235	0.917184119	1.428424465	0.153169721	0.712659122
NCKAP5L	534.659962	-0.185042153	0.129572557	-1.428096788	0.153264002	0.712911646
UBE2Z	1934.771956	-0.169826222	0.118961166	-1.427576975	0.153413655	0.712973607
NUDT18	180.3696814	0.317291728	0.222301832	1.427301452	0.153493022	0.712973607
HSD17B10	282.117433	-0.267542766	0.187470422	-1.427119881	0.153545343	0.712973607
RDH10-AS1	18.89520049	0.756618454	0.53017967	1.427098202	0.153551591	0.712973607
RPS3	10812.60494	-0.293491307	0.205667878	-1.42701578	0.153575346	0.712973607
LOC100287042	48.25579227	0.559231529	0.391902007	1.426967759	0.153589188	0.712973607
PFKFB3	550.2524051	0.177525272	0.124408651	1.426952794	0.153593502	0.712973607
ERICH6-AS1	7.753570455	1.141201087	0.799890944	1.426695845	0.153667584	0.712973607
CYB561	310.7260943	-0.234918177	0.164670782	-1.426592956	0.153697256	0.712973607
ELAC2	1640.309661	0.185017239	0.129698654	1.426516265	0.153719375	0.712973607
LINC01775	5.39245835	1.143943231	0.801980142	1.426398449	0.153753361	0.712973607
RBM42	664.0015639	-0.179920227	0.126137273	-1.426384312	0.15375744	0.712973607
SNORA58	9.830776436	1.5323042	1.074531977	1.426020103	0.153862539	0.71320638
ZNF207	2512.179276	-0.208560173	0.146262253	-1.425932995	0.153887684	0.71320638
PLA2G4A	654.294119	0.286494499	0.200981814	1.425474743	0.154020016	0.713301121
SUGT1P1	14.70814319	0.693585393	0.4865823	1.425422569	0.154035088	0.713301121
LRRCC1	210.4202388	0.262600953	0.184230626	1.425392505	0.154043773	0.713301121
LRRC75A	270.9734253	0.848674939	0.595432736	1.425307826	0.154068239	0.713301121
NUDT2	143.4091196	-0.340991169	0.239275757	-1.425097023	0.154129157	0.713397813
RNF139	1261.916719	-0.167338721	0.117449296	-1.424774146	0.154222498	0.713644486
TOP2A	7620.948472	-0.173498637	0.121797148	-1.424488502	0.154305111	0.713830206
KRBOX4	146.1300025	-0.311950029	0.219016192	-1.424324048	0.154352689	0.713830206
LOC100996437	6.65031824	-1.022413481	0.717975115	-1.424023562	0.154439651	0.713830206
RPL4	20372.03025	-0.284430246	0.199782318	-1.423700802	0.154533101	0.713830206
CPB2-AS1	5.783988856	-1.274726053	0.895442894	-1.423570461	0.154570852	0.713830206
ZNF341	38.63708962	0.486072663	0.341453202	1.423541088	0.15457936	0.713830206
XRCC5	4893.088675	-0.150050838	0.105411365	-1.423478757	0.154597416	0.713830206
HAT1	492.7762973	-0.241261486	0.169490283	-1.423453204	0.154604819	0.713830206
RALB	588.4689359	-0.273949466	0.19251797	-1.422981273	0.154741584	0.713830206
SLC52A2	988.959496	0.180116107	0.126601623	1.42269982	0.154823193	0.713830206
VPS33B	339.1136006	0.176102615	0.123780738	1.422698056	0.154823705	0.713830206
CST3	166.5314683	-0.415418602	0.291999221	-1.422670241	0.154831771	0.713830206
RPGRIP1L	987.7499274	0.179883909	0.12645368	1.422528065	0.15487301	0.713830206
RPS23	3077.873721	-0.226269296	0.159067164	-1.422476459	0.154887981	0.713830206
CRBN	225.967489	-0.208466083	0.146562774	-1.422367204	0.154919679	0.713830206
CDIP1	289.9392784	-0.189731283	0.1334015	-1.422257493	0.154951514	0.713830206
EVA1B	206.3644311	-0.284407429	0.199982915	-1.422158631	0.154980205	0.713830206

MRPS9	313.0616013	-0.212317885	0.149293943	-1.42214668	0.154983674	0.713830206
SNORA21	2.36234096	1.948525339	1.370286159	1.421984252	0.155030823	0.713862858
C8orf31	13.28892284	-0.820783276	0.57747941	-1.421320418	0.15522363	0.714566027
LOC100132249	20.14414955	0.683692137	0.481150479	1.420952835	0.155330471	0.714714914
SMAD6	383.4874242	0.233817658	0.164598132	1.420536521	0.155451544	0.714714914
NHS	183.6876719	0.275060939	0.193634408	1.420516852	0.155457266	0.714714914
CHD6	999.4798683	-0.164275633	0.115667423	-1.420241138	0.155537491	0.714714914
MIR6081	13.19705275	0.98128041	0.690970955	1.420147117	0.155564856	0.714714914
CC2D1B	708.3378481	0.182204816	0.128302206	1.420122247	0.155572095	0.714714914
MIR3122	6.57302054	1.56981783	1.105428348	1.420099126	0.155578825	0.714714914
CHST5	4.471945873	1.616017536	1.137967264	1.42009141	0.155581071	0.714714914
SETDB2	87.9372464	-0.346989188	0.244383393	-1.419855837	0.155649655	0.714714914
FZD7	1166.601962	0.495614582	0.349123978	1.419594795	0.155725682	0.714714914
CLN6	950.0857144	-0.30165643	0.212523115	-1.419405272	0.155780896	0.714714914
HNRNPD	1626.672297	-0.321853953	0.226759576	-1.419362125	0.155793468	0.714714914
ATXN7L3B	1952.949043	-0.130002747	0.091594299	-1.419332305	0.155802158	0.714714914
HDAC2	2014.17625	-0.165235708	0.116448832	-1.41895548	0.155911996	0.714714914
RNF13	595.1581237	-0.178788193	0.12601735	-1.418758552	0.15596942	0.714714914
CARD9	37.62138016	0.748614818	0.527671024	1.418715039	0.155982111	0.714714914
RAB28	235.7149691	0.26494222	0.186752098	1.418684034	0.155991154	0.714714914
C3orf52	114.0974881	0.354165924	0.249686032	1.418445086	0.156060861	0.714714914
POGLUT1	352.8077721	-0.238262259	0.168005351	-1.418182564	0.156137471	0.714714914
LRCH2	509.9259149	0.213955041	0.150871735	1.418125413	0.156154153	0.714714914
WLS	2273.108149	-0.251185583	0.177139855	-1.41800716	0.156188674	0.714714914
CREBZF	1727.486242	0.316425334	0.223148226	1.418005155	0.15618926	0.714714914
LDB1	688.4965997	-0.320791072	0.226232918	-1.417968152	0.156200063	0.714714914
VWA7	8.191633193	-1.02318861	0.721620088	-1.417904832	0.156218552	0.714714914
CYCS	2284.772296	0.250931089	0.177037392	1.417390342	0.156368835	0.715218361
RND3	539.2934784	-0.469311679	0.331141693	-1.417253367	0.156408864	0.715218361
TUFM	2900.705239	-0.173800011	0.122653747	-1.416997164	0.156483757	0.715247042
DLC1	1776.835586	-0.214959809	0.151705213	-1.416957305	0.156495411	0.715247042
EXOSC10	1445.42601	-0.127952908	0.090331558	-1.416480688	0.156634814	0.715357007
LGALS3BP	1531.998294	-0.202328849	0.142844629	-1.416426018	0.156650811	0.715357007
IL23A	3.965175393	-1.408194468	0.994231712	-1.416364466	0.156668822	0.715357007
TYK2	1990.610296	0.160561416	0.113364722	1.416326116	0.156680045	0.715357007
IL20RA	11.99330349	0.836305259	0.590579078	1.41607668	0.156753055	0.715507029
PBX3	213.0079899	-0.314264759	0.221988159	-1.415682532	0.156868475	0.715850506
TCEANC2	297.5182134	-0.293840154	0.207608965	-1.415353878	0.156964765	0.715965733
FAM32A	377.6328373	-0.264242365	0.186701223	-1.415322091	0.156974081	0.715965733
ROBO2	248.2908001	0.44733426	0.316104325	1.415147547	0.15702524	0.715984501
MN1	14.85934055	0.973545271	0.688019151	1.414997344	0.157069275	0.715984501
EHBP1L1	404.5856485	-0.231817151	0.163840315	-1.414896884	0.157098732	0.715984501
MTMR7	112.7284448	0.532079519	0.376167384	1.414475423	0.157222359	0.716364722
TMEM14C	852.4215511	-0.292336999	0.206714754	-1.414204811	0.157301776	0.716400906
SNORA81	68.9075624	0.44222684	0.312749889	1.413995193	0.157363314	0.716400906
CRTC3	640.1025505	0.189675021	0.13414163	1.413990725	0.157364626	0.716400906
SNORD35B	6.624101915	1.135507418	0.803102756	1.413900537	0.157391108	0.716400906
ATP23	93.92326495	0.33277641	0.235398459	1.413672851	0.15745798	0.716407303
APOL4	33.87368051	-0.624726372	0.441986556	-1.413451075	0.157523137	0.716407303
RAB30-DT	96.89469268	0.401600445	0.284135703	1.413410709	0.157534999	0.716407303
ADPRHL1	8.638258427	-0.950847112	0.672853687	-1.413155834	0.157609909	0.716407303
IQCC	118.3461663	0.310528075	0.219764741	1.413002261	0.157655059	0.716407303
LINC01599	6.855243962	1.07889631	0.763563139	1.412975896	0.157662812	0.716407303
IQCE	399.0953447	-0.187679883	0.132878654	-1.412415592	0.157827628	0.716407303
USP44	60.23100026	0.393625104	0.278702797	1.412347158	0.157847767	0.716407303
ARVCF	34.32366139	0.630235965	0.446235448	1.412339534	0.157850011	0.716407303
RWDD4	206.6178314	-0.268872654	0.190380883	-1.412288088	0.157865153	0.716407303
ENO2	256.7179102	0.272425033	0.192940541	1.411963663	0.157960661	0.716407303
KPNB1	10397.59918	-0.17543285	0.124276369	-1.411634821	0.158057514	0.716407303
ZNF219	94.12600884	-0.297955409	0.211099056	-1.411448325	0.158112462	0.716407303
ACSL4	1248.794633	0.214289377	0.15183434	1.411336706	0.158145356	0.716407303
CYCSP52	2.463135506	2.156966068	1.5284224	1.411236885	0.158174777	0.716407303
AURKAP1	32.08568893	0.685577012	0.485799012	1.411235913	0.158175064	0.716407303
SNORA3B	23.9732468	0.766132707	0.542912232	1.411153889	0.158199243	0.716407303
AGTR1	393.9007223	-0.380013391	0.269344291	-1.410883404	0.158278996	0.716407303
ANKRD39	85.20551083	0.315138459	0.223372378	1.410821077	0.158297378	0.716407303
ITFG1-AS1	3.159130732	1.56856894	1.111827495	1.410802437	0.158302876	0.716407303
TMPRSS15	1785.782858	-0.279934621	0.198446718	-1.410628628	0.158354146	0.716407303
C11orf98	8.669463864	1.056888452	0.749314353	1.410474052	0.158399753	0.716407303
GSTO1	497.0095184	-0.256233673	0.181678147	-1.410371458	0.158430029	0.716407303
CDC5L	1483.461437	-0.146508526	0.103884122	-1.410307213	0.15844899	0.716407303
EPHA2	20.96256991	0.792584228	0.562080744	1.410089631	0.158513219	0.716407303
CINP	190.5655891	0.277886279	0.197088242	1.40995869	0.158551881	0.716407303
SGTA	661.4715947	-0.181620779	0.128815573	-1.409928742	0.158560725	0.716407303
LACTB2	328.1591705	-0.211289228	0.149880735	-1.409715721	0.158623641	0.716407303
SNORD126	6.672091409	1.475218816	1.046471437	1.409707674	0.158626018	0.716407303
RPL39	3255.768654	-0.266560103	0.189093959	-1.409670119	0.158637112	0.716407303
DNM1	3144.659293	-0.157589025	0.111791871	-1.409664442	0.158638789	0.716407303
DCLK1	460.2489107	-0.349307981	0.247830734	-1.409461913	0.158698628	0.716495958
LRIG2	1164.306191	0.146601499	0.104069189	1.408692621	0.158926078	0.717341111
SNORD116-27	35.39549768	0.514936451	0.365620175	1.408391784	0.159015091	0.717372188
ZNF425	10.90360934	-1.001205682	0.710945385	-1.408273691	0.159050043	0.717372188
SIGIRR	6.461727045	-1.141431853	0.810557172	-1.40820647	0.159069941	0.717372188
ACTBL2	3.79246154	-1.401652896	0.995403575	-1.408125237	0.159093989	0.717372188
LOC100506691	29.68813923	0.592553342	0.420924252	1.407743412	0.159207062	0.717700441
ZKSCAN1	1695.016523	-0.204604501	0.145366931	-1.407503758	0.159278064	0.717728956
SUDS3	1305.700712	0.142002287	0.100893296	1.40745018	0.15929394	0.717728956
TMEM202-AS1	7.083027915	1.125742828	0.800277321	1.406690903	0.159519064	0.718317305
MEN1	448.7241	-0.165265844	0.117486798	-1.406675873	0.159523523	0.718317305
LOXL3	174.1981226	-0.251422697	0.178744736	-1.40660197	0.159545448	0.718317305

SPATA21	13.59750038	1.054974963	0.750167555	1.406319103	0.15962939	0.718350049
D2HGDH	401.790702	0.369423649	0.262694921	1.406283938	0.159639828	0.718350049
TYRO3P	10.17198421	1.546662638	1.100017327	1.406034797	0.159713792	0.718350049
ZNF282	457.0864824	-0.184242046	0.131036674	-1.406034208	0.159713967	0.718350049
RBM12B-AS1	80.02534372	0.664936315	0.473172619	1.405272173	0.15994036	0.718858638
TGIF1	278.5062939	-0.215311655	0.153218302	-1.405260676	0.159943777	0.718858638
GHRLOS	54.4204189	0.496376652	0.353244473	1.405192974	0.159963903	0.718858638
WASHC2A	343.5144259	-0.243855103	0.173548694	-1.405110562	0.159988404	0.718858638
LRFN1	280.3440654	0.229387331	0.163281768	1.404855756	0.160064175	0.718983013
WDR61	560.7611953	-0.249154273	0.177379269	-1.404641445	0.160127926	0.718983013
TRIM44	2086.718216	-0.211687849	0.150709286	-1.404610523	0.160137126	0.718983013
CLGN	58.14862461	-0.567222452	0.403939557	-1.404226059	0.160251544	0.71907906
EXPH5	90.41700522	0.59928567	0.426773846	1.404223046	0.160252441	0.71907906
LOC101929679	10.42734632	1.307372377	0.931118159	1.404088583	0.160292473	0.71907906
PI16	2.453661473	-1.90829979	1.359191388	-1.403996381	0.160319927	0.71907906
SRSF8	405.812099	-0.224615189	0.160137743	-1.402637414	0.160724989	0.720692785
ADAM3A	66.35562406	0.358621179	0.25569808	1.402518078	0.160760596	0.720692785
MIR548L	4.803869687	1.756308078	1.252593474	1.402137337	0.160874239	0.721020862
HACD1	250.7116579	0.239737563	0.171007479	1.401912741	0.160941305	0.72114007
IFI27L2	62.51554579	-0.50130378	0.357713276	-1.401412289	0.16109082	0.721628559
LUZP1	1102.474112	0.138011485	0.098521155	1.400830967	0.161264626	0.72216498
TFPI2	29.80245922	0.575453357	0.410820749	1.400740733	0.161291617	0.72216498
TRIM14	445.6487893	-0.229722546	0.164033355	-1.400462394	0.161374897	0.722209363
PLD4	14.18115045	-0.801847629	0.572569705	-1.400436701	0.161382586	0.722209363
TMEM101	509.0222454	-0.196170087	0.140115795	-1.40005691	0.161496277	0.722354063
DLGAP1	21.53089436	0.647918409	0.462898835	1.399697644	0.16160388	0.722354063
SNHG4	232.3194671	0.522022988	0.372970152	1.399637439	0.161621917	0.722354063
B4GALT5	1408.226596	0.236888153	0.169255854	1.399586171	0.161637278	0.722354063
ZNF337-AS1	82.09428873	0.446923223	0.31934072	1.399518427	0.161657576	0.722354063
FAF2	1533.950605	-0.136536643	0.097574585	-1.399305384	0.161721426	0.722354063
LOC102724849	7.038750646	-1.025299075	0.732777901	-1.399194863	0.161754556	0.722354063
C17orf97	76.8928277	0.331336598	0.236817626	1.399121357	0.161776594	0.722354063
GPI	3760.451777	-0.144097486	0.102992187	-1.399110847	0.161779746	0.722354063
CEMIP	106.8090607	0.44740278	0.319808464	1.398971043	0.161821667	0.722360246
GHITM	1345.089205	-0.19248533	0.137609705	-1.398777288	0.161879779	0.722438684
DIS3L	784.500188	-0.180341916	0.128972689	-1.398295383	0.162024384	0.722902984
RNF19B	135.3649518	-0.393879269	0.281720764	-1.398119411	0.162077212	0.722957676
C11orf54	375.1813703	0.184795847	0.132189729	1.397959195	0.162125321	0.722991296
IQCB1	262.7461077	-0.200935422	0.143766634	-1.397649904	0.162218225	0.723224609
DCAF8	1480.992613	-0.163771864	0.117207559	-1.397280729	0.162329168	0.723538215
SUMO2	1667.295272	-0.196673247	0.140779985	-1.397025632	0.162405863	0.723699046
TFAP2A-AS2	21.32689123	-0.835346205	0.598038361	-1.396810404	0.162470593	0.723806491
MIPEP	466.4855461	-0.238098928	0.170537658	-1.396166277	0.162664429	0.724344969
TAFA5	10.76203364	-1.474260391	1.056029854	-1.396040448	0.162702315	0.724344969
MAP3K7	945.6976349	0.140565714	0.100691511	1.396003614	0.162713407	0.724344969
UNC13C	13.95451225	1.413231259	1.012520648	1.395755495	0.162788136	0.724496651
SIRT7	153.3257718	0.428772958	0.307282221	1.39537184	0.162903739	0.724658119
PRAP1	31.7002825	0.489534243	0.35086078	1.39523786	0.162944124	0.724658119
MYADML2	5.380284105	-1.283979871	0.920263775	-1.395230265	0.162946413	0.724658119
RALGPS1	77.72001763	0.515573867	0.36964975	1.394763196	0.163087261	0.724889798
RAB3IL1	75.50910077	0.404357432	0.289931556	1.394665132	0.163116844	0.724889798
PDE7A	562.8022625	0.185322502	0.132880738	1.39465287	0.163120544	0.724889798
AP3B1	811.2096369	-0.174586395	0.125212655	-1.394319089	0.163221269	0.725156573
MYO1C	2937.100705	-0.149102381	0.106947097	-1.394169501	0.163266426	0.725176397
ALDH7A1	772.2171297	-0.166315282	0.119334626	-1.393688383	0.163411726	0.725255341
SUCLG2	985.4250779	-0.220573137	0.158273149	-1.393623235	0.163431408	0.725255341
MAGEA4	11.84464949	-0.778349383	0.558579499	-1.393444235	0.163485497	0.725255341
TTBK2	922.8349527	0.306776416	0.220161015	1.393418435	0.163493294	0.725255341
NIPSNAP2	740.8840003	-0.224527716	0.161154123	-1.393248348	0.163544704	0.725255341
F8	47.01825472	-0.702103757	0.503956125	-1.393184294	0.163564068	0.725255341
CEP55	549.2309519	0.165748509	0.118973852	1.393150734	0.163574214	0.725255341
ROCK1P1	40.9018123	0.473323898	0.339780479	1.393028523	0.163611166	0.725255341
APLP1	18.20055907	-0.685209788	0.492005319	-1.392687763	0.163714231	0.725255341
MOCS3	236.6181624	-0.238727321	0.171416056	-1.392677709	0.163717272	0.725255341
CAMTA1-DT	7.804835344	-1.096780931	0.787705437	-1.392374457	0.163809035	0.725255341
POPDC3	145.100802	0.272430022	0.195660895	1.392358047	0.163814002	0.725255341
SESTD1	1359.767461	0.225207952	0.161759329	1.392240893	0.163849463	0.725255341
MIR100HG	1137.814922	0.238635779	0.171438175	1.391964069	0.163933279	0.725255341
GNB4	9504.720816	0.126525246	0.090921035	1.391594868	0.164045114	0.725255341
GUSBP15	19.30103761	0.759475396	0.545884672	1.391274449	0.164142219	0.725255341
CAPZA1	3188.059468	-0.156310755	0.112359639	-1.391164629	0.16417551	0.725255341
OTULIN	1144.185344	0.167753851	0.120593396	1.391069966	0.164204211	0.725255341
FOXS1	4.836285046	-1.653595049	1.188847262	-1.390923041	0.164248765	0.725255341
IPPK	181.4569691	0.403426867	0.290048793	1.390893105	0.164257844	0.725255341
C20orf204	13.51872806	0.829098274	0.596125098	1.390812561	0.164282273	0.725255341
LOC102724814	435.2126172	0.406670371	0.292421656	1.390698545	0.164316859	0.725255341
SPNS1	592.6369011	-0.191918276	0.138024428	-1.390466012	0.164387413	0.725255341
KANSL1	1080.275472	-0.137901794	0.099180816	-1.390407945	0.164405035	0.725255341
L3MBTL1	65.67267245	0.60040679	0.431852376	1.390305632	0.164436088	0.725255341
CDC123	961.9404845	-0.216085034	0.155424034	-1.39029356	0.164439753	0.725255341
AUTS2	253.6423951	-0.207443861	0.149220594	-1.390182518	0.164473461	0.725255341
MMUT	1349.881703	-0.235724945	0.169576412	-1.390080977	0.164504289	0.725255341
KCNIP3	179.1391539	-0.309942476	0.222995916	-1.38990203	0.164558629	0.725255341
MIR4305	2.261926494	2.146871266	1.544945002	1.389610156	0.16464729	0.725255341
SYNJ2-IT1	2.926178662	1.664416696	1.197882633	1.389465588	0.164691218	0.725255341
MIR637	10.96841334	1.141359691	0.821522041	1.389323273	0.16473447	0.725255341
ZSCAN16	56.63169514	0.662824188	0.477104213	1.389265006	0.164752181	0.725255341
SELENOO	442.6798294	0.282544019	0.203388402	1.38918452	0.164776648	0.725255341
EMBP1	17.40875526	-0.684763819	0.492939205	-1.389144569	0.164788793	0.725255341
OXSR1	1082.269252	0.224125035	0.161340654	1.389141726	0.164789658	0.725255341

TATDN1	669.9653643	-0.194334267	0.139914135	-1.388953782	0.164846805	0.725255341
REC114	3.977043092	1.638277382	1.179642456	1.388791471	0.164896169	0.725255341
BTG3	310.0403266	0.210318725	0.151440408	1.388788684	0.164897017	0.725255341
CENPF	3191.683142	-0.166219723	0.119691125	-1.388738912	0.164912157	0.725255341
SH3BGRL	404.2657687	-0.284472286	0.204876602	-1.38850549	0.164983174	0.725388642
LOC100506730	58.63800962	0.382028293	0.275196443	1.388202149	0.165075498	0.725615535
FCF1	1229.691206	-0.175906789	0.126742376	-1.387908252	0.165164985	0.725829847
PCDHGB7	1094.986103	0.390828693	0.2816433	1.387672607	0.165236761	0.725870424
SEPTIN7P2	133.4352182	0.425694231	0.306792881	1.387562286	0.165270372	0.725870424
AFAP1L1	45.81232391	0.518544476	0.373731996	1.387476806	0.165296419	0.725870424
NDUFB11	386.5919161	-0.28924288	0.208499034	-1.387262449	0.165361749	0.72597841
NPIPB11	8.658672297	1.187835495	0.856424114	1.3869711	0.165450575	0.726070191
HCG18	701.2120634	-0.189753609	0.136815898	-1.386926606	0.165464144	0.726070191
TUBG1	425.4842536	0.274508198	0.198012563	1.386317082	0.165650103	0.726707247
LOC102723665	5.608675868	1.34448448	0.970029401	1.386024464	0.165739434	0.726920184
OBSCN	200.9035075	0.43354151	0.312830248	1.385868256	0.165787136	0.726950481
CHCHD7	478.3239134	0.151167295	0.1091781	1.384593564	0.166176781	0.728247472
BCL6	342.7030697	-0.448273709	0.323809244	-1.384375884	0.166243389	0.728247472
DERL2	592.0565968	-0.198153624	0.143141466	-1.38432021	0.166260428	0.728247472
CFAP299	2.709675565	1.632169316	1.179166345	1.384172236	0.166305723	0.728247472
PSMA7	1751.840013	-0.214975655	0.155317292	-1.384106384	0.166325882	0.728247472
HSPA12B	28.65567497	0.541276563	0.391067793	1.384099055	0.166328126	0.728247472
ADAM19	2501.013112	-0.212989107	0.153899909	-1.383945638	0.166375101	0.728274207
SPA17	12.70771109	-0.855299646	0.618094597	-1.383768198	0.166429444	0.728333174
HEXD	254.5449461	0.267028182	0.1930603	1.383133573	0.166623913	0.728384698
PTGER1	9.279651756	-1.191425668	0.861408369	-1.383113643	0.166630023	0.728384698
ASPM	3847.981697	0.126307564	0.091334504	1.38291181	0.166691909	0.728384698
MPPED2	23.93846036	-0.716978224	0.518461871	-1.382894798	0.166697126	0.728384698
PSMD5	1321.014927	0.158011946	0.114275282	1.382730741	0.166747442	0.728384698
B3GALT1	8.372877575	0.989803585	0.715900217	1.382599924	0.166787572	0.728384698
DOC2A	11.68273657	0.950574691	0.687527325	1.382599144	0.166787812	0.728384698
GOLGA8B	622.333139	0.430308896	0.311237088	1.382575895	0.166794945	0.728384698
LOC105369306	3.121721667	1.769311207	1.280026106	1.382246189	0.166896122	0.728384698
MAPK8IP2	23.11700777	0.581314018	0.420568507	1.382210054	0.166907214	0.728384698
ZNF788P	174.0455361	-0.334891364	0.242355898	-1.381816438	0.16702807	0.728384698
ENGASE	694.9613257	0.29976364	0.216944098	1.381755219	0.167046873	0.728384698
REST	1278.584674	-0.149094001	0.107921457	-1.381504702	0.167123833	0.728384698
TAF9	465.0920203	-0.232675613	0.168428314	-1.381451895	0.167140059	0.728384698
ZCRB1	203.9137465	-0.286885624	0.207672334	-1.381434	0.167145558	0.728384698
SNORD117	32.5260288	0.59743998	0.432511517	1.381327334	0.167178337	0.728384698
HASPIN	378.9260409	0.177343182	0.128404905	1.381124675	0.16724063	0.728384698
NDUFAF5	139.4721981	0.395246848	0.286189894	1.381065005	0.167258974	0.728384698
LINC00221	145.3305085	-0.271469062	0.196579861	-1.380960696	0.167291046	0.728384698
STPG3	25.6114694	0.769254606	0.55704553	1.380954634	0.16729291	0.728384698
EIF1AX	2895.200711	-0.192874611	0.139674106	-1.380890246	0.16731271	0.728384698
TFIP11	319.691669	-0.195626687	0.141689067	-1.380675952	0.167378619	0.728384698
SNORA21B	37.26554166	0.573354673	0.415273645	1.380667131	0.167381332	0.728384698
CNTN6	8.056236325	-1.111727951	0.805517992	-1.380140433	0.16754341	0.728574647
GRIN3B	32.28482175	-0.582876802	0.422367376	-1.380023258	0.167579484	0.728574647
PITRM1-AS1	224.4650586	0.413174673	0.299421718	1.379908829	0.167614718	0.728574647
HAUS7	209.1754849	0.283140616	0.205190334	1.379892563	0.167619727	0.728574647
NARF	501.3025815	-0.190019938	0.137715598	-1.379799682	0.167648331	0.728574647
SPRED3	11.99724288	0.888168905	0.643727361	1.379728373	0.167670293	0.728574647
KMT2A	1858.17988	-0.164092714	0.118949297	-1.379518147	0.167735055	0.728608099
NAT10	1698.831252	-0.251760407	0.18251194	-1.379418833	0.167765656	0.728608099
GIPC1	746.4532181	-0.177273096	0.128523468	-1.379305267	0.167800653	0.728608099
NATD1	635.7708448	-0.465454519	0.33749939	-1.379126994	0.167855602	0.728669143
NUP153	3636.232077	0.120616132	0.087476865	1.37883465	0.167945741	0.728847569
PLK2	1889.547214	-0.475574767	0.344994319	-1.378500283	0.16804888	0.728847569
RNASEH2B	567.1928924	-0.195690822	0.141961084	-1.378482165	0.16805447	0.728847569
SH3GL3	93.31001921	-0.322451382	0.233956704	-1.378252372	0.168125383	0.728847569
MRPS9-AS1	7.775808819	1.006905735	0.730628384	1.378136625	0.16816111	0.728847569
ZNF697	701.7926868	-0.24868047	0.180452437	-1.378094272	0.168174184	0.728847569
CLIC2	16.42844426	-0.728598265	0.528710838	-1.378065688	0.168183008	0.728847569
UBLCP1	476.5554471	-0.177848911	0.129103215	-1.37757151	0.168335622	0.729068886
LUM	56818.33179	0.149370593	0.108437826	1.37747683	0.168364873	0.729068886
ZNF667	58.30192113	-0.451850156	0.328079144	-1.377259613	0.168431997	0.729068886
KMT2C	1989.53191	0.225784967	0.163938125	1.37725722	0.168432737	0.729068886
ST13	2584.093326	-0.221715588	0.160985661	-1.377238112	0.168438642	0.729068886
PPM1K	262.6269535	0.306789871	0.222888028	1.37643046	0.168688403	0.729567085
RNF38	393.0912386	-0.172755337	0.125533255	-1.376171888	0.168768423	0.729567085
NCEH1	262.3750735	0.262660966	0.190877083	1.376073864	0.168798766	0.729567085
NMU	5.91839344	-1.37733417	1.001003735	-1.375953079	0.168836161	0.729567085
PABPC4	4252.769517	-0.168718916	0.122631363	-1.375821913	0.168876775	0.729567085
C9orf152	5.16849194	1.153260814	0.838284203	1.375739647	0.168902253	0.729567085
SNORD52	29.02890104	0.719388679	0.522951578	1.375631528	0.16893574	0.729567085
RRAS2	701.3318356	0.160077516	0.116372102	1.375566085	0.168956013	0.729567085
ATE1	546.1211998	0.177845208	0.129289464	1.37555839	0.168958397	0.729567085
LOC100507507	44.71652461	0.429708484	0.312391886	1.375543041	0.168963152	0.729567085
KIF9-AS1	27.81751385	0.502870183	0.365796236	1.374727607	0.169215909	0.730481468
GASK1B	445.8661358	-0.18122058	0.131861684	-1.374323264	0.169341347	0.730845921
CCDC162P	5.540374365	1.69302005	1.23203997	1.374160004	0.169392014	0.730887578
CIAO2B	260.2619138	0.258277031	0.188003676	1.373787131	0.169507778	0.731210021
GAB2	224.2213967	0.397692629	0.289524917	1.373604156	0.169564606	0.731278142
CUX1	1632.220827	0.426562416	0.310617332	1.373273064	0.169667473	0.73154473
ZNF441	91.82150017	0.314157491	0.228797427	1.3730814	0.169727043	0.731624552
TAF10	360.345989	-0.307633087	0.224106129	-1.372711618	0.169842016	0.731751198
ZFC3H1	1740.139626	0.208338008	0.151776895	1.372659576	0.169858202	0.731751198
NPW	69.15201152	-0.382721391	0.278831396	-1.372590737	0.169879613	0.731751198
HOXD9	457.1950891	-0.261552399	0.190597686	-1.372274785	0.169977912	0.731857493

ZNF823	77.60889825	0.358151073	0.261018553	1.372128799	0.170023346	0.731857493
ZNF10	239.6223816	0.253368537	0.184657206	1.372102078	0.170031663	0.731857493
PIGH	397.6932481	-0.195389635	0.142413981	-1.371983521	0.170068568	0.731857493
ALMS1	1437.169663	0.203870345	0.148614907	1.371802799	0.170124836	0.731922881
TRIM22	4.973784997	1.440027719	1.049997276	1.371458528	0.170232063	0.732207426
PSMB7	1273.762259	-0.176581065	0.128777888	-1.371206404	0.170310622	0.732368554
EIF3L	3653.63344	-0.282674372	0.206187775	-1.370956026	0.170388665	0.732430812
GNPDA2	253.7331152	0.19846861	0.144783556	1.370795242	0.170438795	0.732430812
FAM227A	120.1251704	0.311936925	0.227564212	1.370764422	0.170448405	0.732430812
ITPRIP	464.597797	-0.212247906	0.154909861	-1.370138126	0.170643788	0.732997173
DLX6	122.7278597	0.497050208	0.362819227	1.369966558	0.170697341	0.732997173
ITGA11	20535.72145	-0.52113969	0.380448655	-1.36980295	0.170748421	0.732997173
ZNF721	284.9012431	0.215089567	0.157048053	1.369578055	0.170818654	0.732997173
KIAA1257	59.55868896	0.581723268	0.424873015	1.369169723	0.170946228	0.732997173
NHSL1	463.0630502	-0.235580211	0.172062906	-1.369151648	0.170951877	0.732997173
DYNC2LI1	217.4507702	-0.224728384	0.164139972	-1.369126491	0.170959739	0.732997173
ANO7	67.66498786	0.413829733	0.302260682	1.369115329	0.170963227	0.732997173
MFSD2B	19.82545838	0.781911083	0.571246955	1.368779433	0.171068232	0.732997173
MGME1	614.833093	-0.154500809	0.112875653	-1.368770011	0.171071178	0.732997173
ATP5ME	36.25277094	0.484586937	0.354049573	1.368697983	0.171093701	0.732997173
KLHL9	856.0322991	-0.146714156	0.107195347	-1.368661604	0.171105078	0.732997173
LVRN	5.668301404	1.115101717	0.814757577	1.368630067	0.17111494	0.732997173
IFI16	1453.553381	-0.31954469	0.23350999	-1.368441196	0.171174017	0.733055638
COQ10B	545.5273305	0.242109131	0.176938527	1.368323423	0.171210862	0.733055638
ZIC1	60.80924754	-0.417719719	0.305383959	-1.367850886	0.171358756	0.733512618
ZNF12	814.3373188	-0.127631698	0.093360764	-1.367080692	0.171600014	0.733731782
CEP135	223.5422231	0.261966941	0.191631985	1.367031398	0.171615464	0.733731782
ANKRD26	363.3116934	0.211041945	0.154398229	1.366867655	0.171666792	0.733731782
SLC26A7	248.3053872	1.441413961	1.05463135	1.36674674	0.171704702	0.733731782
FAM66D	14.08090534	-0.814894216	0.596280334	-1.366629369	0.171741507	0.733731782
PCNX2	458.887068	-0.202643086	0.148282424	-1.3666022	0.171750027	0.733731782
SNORD58C	9.246693963	1.042532812	0.762934646	1.366477218	0.171789227	0.733731782
HMG20A	1143.365464	-0.219769513	0.160830526	-1.366466416	0.171792615	0.733731782
SEC23A	8425.515727	0.262424357	0.192046812	1.366460367	0.171794513	0.733731782
NABP2	429.4803144	-0.294711781	0.215766147	-1.365885175	0.171975007	0.733731782
TCHP	328.3770031	0.290384189	0.212602405	1.365855616	0.171984286	0.733731782
GMPR2	1124.495654	-0.228633946	0.167393006	-1.36585125	0.171985657	0.733731782
MAGEL2	9.931374451	1.031027514	0.754888459	1.365801135	0.17200139	0.733731782
RASA2	406.2746595	0.221747736	0.162363207	1.365751147	0.172017085	0.733731782
GSTK1	268.3622845	-0.192342612	0.140836285	-1.365717737	0.172027575	0.733731782
DNAJC30	101.9503063	0.339394928	0.248557379	1.365459072	0.172108808	0.733784152
WDR26	2409.513852	-0.124649204	0.091290249	-1.365416412	0.172122209	0.733784152
ACAA1	209.7982455	-0.238552592	0.174750671	-1.365102584	0.17222081	0.733793286
LOC100506472	41.83300894	-0.525828144	0.385200515	-1.36507643	0.172229029	0.733793286
CA12	3999.259105	0.187616091	0.137483445	1.364644965	0.172364665	0.733793286
DAGLB	375.4408079	0.224979644	0.164865395	1.364626244	0.172370552	0.733793286
ZNF815P	45.11214864	0.540544489	0.39617996	1.364391298	0.172444445	0.733793286
PPT2	186.4318137	0.25834332	0.189349017	1.364376346	0.172449149	0.733793286
RECQL5	667.8291165	-0.148853557	0.109111408	-1.364234583	0.172493747	0.733793286
PPP1R37	307.5247436	-0.191250495	0.140189127	-1.364232015	0.172494555	0.733793286
HRC	5.222586764	1.303149617	0.955226686	1.364230748	0.172494954	0.733793286
ADGRB2	519.7051976	0.329467915	0.241583525	1.3637847	0.172635337	0.734086798
PFDN4	97.89917611	0.391787961	0.287309837	1.3636427	0.172680046	0.734086798
DNAJC13	1312.90287	-0.180704933	0.132518646	-1.36361892	0.172687534	0.734086798
ESRP2	8.166085481	1.049856587	0.7700007	1.363448873	0.172741087	0.734139321
GCC1	366.176316	-0.228292621	0.167462216	-1.363248541	0.172804194	0.734186454
SLMO2-ATP5E	5.872477546	1.149388855	0.843235485	1.363069837	0.172860502	0.734186454
GPATCH8	870.3457448	-0.134011109	0.098319967	-1.363010108	0.172879325	0.734186454
OPA1-AS1	52.55728623	0.761904901	0.559049878	1.362856754	0.172927661	0.734186454
SNRNP200	10708.19669	-0.131047707	0.096174275	-1.362606662	0.173006508	0.734186454
HAO1	9.571816189	-1.122080845	0.823489892	-1.362592129	0.173011091	0.734186454
F3	376.5207594	-0.391361339	0.287246657	-1.36245742	0.173053573	0.734186454
SIM2	141.9851645	-0.507001939	0.372147651	-1.362367698	0.173081873	0.734186454
ALOX12B	19.47824352	-0.655624254	0.481339375	-1.362083154	0.173171644	0.734186454
ZNF639	3226.413563	0.13123904	0.096356508	1.362015323	0.17319305	0.734186454
CDR1	2495.233937	0.432004891	0.317189738	1.361976255	0.173205379	0.734186454
ENSA	1176.11643	-0.14012229	0.102946819	-1.361113354	0.17347787	0.735166623
SNAPC2	131.5699618	-0.307533462	0.226029225	-1.360591588	0.173642792	0.73556354
MELTF-AS1	74.3094718	0.459164974	0.337497148	1.360500308	0.173671656	0.73556354
RICTOR	1419.28711	0.175222416	0.128810821	1.360308199	0.173732415	0.73556354
NUP205	4517.681041	0.137723969	0.101254151	1.360180969	0.173772663	0.73556354
KLHL28	582.3119713	0.230891235	0.169752452	1.36016436	0.173777918	0.73556354
TMEM86B	66.91275438	0.337162948	0.247992621	1.359568465	0.173966523	0.735766714
SDHAP2	310.4110602	0.412163028	0.303168141	1.359519595	0.173981997	0.735766714
IL15RA	30.01142521	0.707163144	0.520326395	1.359076055	0.17412249	0.735766714
SNAPIN	273.2058068	-0.222941217	0.164059817	-1.358902025	0.174177637	0.735766714
KCNJ12	30.45613499	0.564274619	0.415311981	1.358676476	0.17424913	0.735766714
RPL18	7312.486891	-0.250311166	0.184238805	-1.358623477	0.174265932	0.735766714
SLC31A1	1827.341559	-0.240257293	0.17684692	-1.358560798	0.174285805	0.735766714
LINC00894	42.19776103	0.505694298	0.372246663	1.358492497	0.174307462	0.735766714
MIR193BHG	12.03046582	0.82527669	0.6075049	1.35846919	0.174314852	0.735766714
LOC284009	13.36335094	0.868018562	0.638971421	1.358462261	0.17431705	0.735766714
CACNG7	9.554970422	-1.86140376	1.370254831	-1.358436196	0.174325315	0.735766714
MYO1D	334.017005	-0.316744266	0.233185239	-1.358337547	0.174356601	0.735766714
TMEM87B	697.4921422	0.196196797	0.144440947	1.358318404	0.174362673	0.735766714
IFITM3	180.7964979	-0.316427779	0.233048108	-1.357778794	0.174533886	0.736174936
PYCR1	2997.34042	-0.239879591	0.176773865	-1.356985612	0.174785782	0.736174936
LOC101930421	16.90434311	1.223112465	0.901381429	1.356931068	0.174803114	0.736174936
PRR29	3.0365592	-1.716460997	1.264989154	-1.356897797	0.174813687	0.736174936
EMCN	182.0951912	-0.348335216	0.256746389	-1.356728783	0.174867403	0.736174936

STARD8	996.8820039	0.308332941	0.227322599	1.356367308	0.17498233	0.736174936
SEMA3B	51.87688246	0.660209079	0.486846157	1.35609385	0.175069309	0.736174936
TBCC	266.76748	-0.231795471	0.170929293	-1.356089796	0.175070599	0.736174936
PLEC	21591.80385	-0.256272269	0.188991276	-1.356000521	0.175099002	0.736174936
IRGM	29.46222217	-0.537395955	0.396342717	-1.355887042	0.175135111	0.736174936
MANF	387.3830746	0.191798603	0.141486201	1.355599355	0.175226677	0.736174936
PLAGL1	8282.27042	0.175450075	0.129427483	1.355585926	0.175230952	0.736174936
NPTN-IT1	197.5288528	0.492657143	0.363429381	1.355578741	0.17523324	0.736174936
SPCS2	605.865648	-0.190620368	0.140624158	-1.355530734	0.175248523	0.736174936
LOC101929479	121.2513459	0.295272219	0.217850869	1.355386921	0.175294314	0.736174936
LRRN4	6.63541117	1.416501059	1.045183216	1.355265791	0.17533289	0.736174936
EP300	1435.604562	-0.140999882	0.104049211	-1.355126864	0.175377141	0.736174936
BTF3	2060.667029	-0.266196675	0.19647049	-1.354893932	0.175451353	0.736174936
ALKBH4	90.40490169	-0.360022544	0.265747541	-1.354754001	0.175495946	0.736174936
ARAP1-AS2	29.34185464	0.860188717	0.634944422	1.354746475	0.175498345	0.736174936
ZC3H10	315.2762442	0.183401676	0.135379925	1.354718404	0.175507292	0.736174936
SRBD1	417.502758	-0.260805087	0.192516703	-1.354714078	0.175508671	0.736174936
HTR2A	2.732928541	-1.601905473	1.182468092	-1.354713488	0.175508859	0.736174936
PCDHGB8P	17.57732466	1.131049674	0.834981657	1.354580265	0.175551324	0.736174936
CASC2	14.85591568	-0.700964549	0.517491751	-1.354542459	0.175563377	0.736174936
ZDHHC8	857.7658345	0.204706577	0.151145678	1.354366059	0.17561962	0.736174936
LIG1	815.5021624	-0.242145858	0.178795383	-1.354318293	0.175634853	0.736174936
S100A16	214.5256801	-0.349284061	0.257923836	-1.354213968	0.175668124	0.736174936
LCMT1-AS2	19.88533628	0.80217818	0.592427511	1.354052884	0.175719506	0.736174936
PDCD2L	104.8656499	0.294102744	0.217213995	1.353976958	0.175743729	0.736174936
WBP1	108.038158	-0.335420233	0.247729746	-1.353976413	0.175743903	0.736174936
KCNS3	249.9092859	0.34464318	0.254563583	1.353858928	0.175781389	0.736174936
ZMIZ2	292.3408569	0.273022809	0.20171124	1.353532949	0.175885431	0.736289934
UNC45A	1187.869484	-0.146338696	0.108117609	-1.353513988	0.175891484	0.736289934
URI1	1391.204445	-0.135392991	0.100086717	-1.352756837	0.176133326	0.7368953
AAMDC	45.39813926	0.384346634	0.284140824	1.352662486	0.17616348	0.7368953
CELSR3	659.2393037	0.176152915	0.130236157	1.35256537	0.176194522	0.7368953
EXOSC6	668.9218603	-0.179418496	0.13265266	-1.352543516	0.176201508	0.7368953
CD82	28.05338527	-0.863323009	0.638386138	-1.352352374	0.176262617	0.736977908
RRS1	411.990577	-0.255926691	0.189276792	-1.352129275	0.176333964	0.737080105
LINC00630	51.7758984	-0.375315672	0.277596799	-1.352017288	0.176369785	0.737080105
RPS19BP1	273.248353	-0.245353511	0.181515913	-1.351691473	0.176474034	0.737342857
AFAP1-AS1	23.42936431	0.776894942	0.574838339	1.351501611	0.176534804	0.737407736
PISD	371.8982511	0.281477807	0.20830618	1.351269591	0.176609089	0.737407736
METAP1	1579.361542	0.135642829	0.100382838	1.351255178	0.176613705	0.737407736
LOC100996664	10.56437129	1.027650848	0.760648387	1.351019559	0.176689167	0.737496006
NT5C3A	416.5954844	-0.21997886	0.162844148	-1.350855171	0.176741831	0.737496006
CCDC33	2.526141609	2.370699446	1.755034217	1.350799559	0.176759649	0.737496006
LTB4R2	178.1874501	0.532827045	0.394516737	1.350581597	0.176829499	0.737496006
SCAMP4	389.9797662	-0.174145409	0.128944707	-1.350543297	0.176841775	0.737496006
TSPAN12	90.14928875	0.332919698	0.246538407	1.350376611	0.176895209	0.737546241
GRN	2761.152178	-0.291809119	0.21612933	-1.350159737	0.17696475	0.73766359
RPL15	9137.02072	-0.245511881	0.181906409	-1.349660425	0.177124932	0.738049272
ZNF740	1174.074488	-0.166273332	0.123217488	-1.349429657	0.177199	0.738049272
TOMM70	1920.907785	-0.164159896	0.121654248	-1.349397155	0.177209434	0.738049272
TTC26	414.1022202	-0.228692033	0.169482471	-1.349355077	0.177222943	0.738049272
UQCC2	480.5726167	-0.24013196	0.178008616	-1.348990655	0.177339968	0.738364071
UBQLN2	481.8768509	-0.185303243	0.137464296	-1.348009976	0.177655174	0.739503668
MAFB	202.503024	-0.914370345	0.678479378	-1.347675957	0.177762628	0.73967147
CACNA1C-AS4	16.15004489	1.054853733	0.782749252	1.347626627	0.177778502	0.73967147
RAB4A	532.9000617	-0.213460398	0.158436437	-1.347293601	0.177885692	0.73985366
MXI1	365.9926678	-0.240183201	0.178320833	-1.346916101	0.178007255	0.73985366
SLC9C1	5.444864638	1.385954259	1.029087186	1.346780212	0.178051029	0.73985366
PTPN22	10.07846078	-0.871782454	0.647345011	-1.346704523	0.178075415	0.73985366
OR1E1	6.703467904	-1.187512174	0.881876149	-1.346574772	0.178117224	0.73985366
SLC1A3	400.5683445	0.341562434	0.253662645	1.346522398	0.178134102	0.73985366
TJAP1	539.569711	-0.220912333	0.164067239	-1.346474375	0.178149579	0.73985366
MTHFD1	3814.020099	-0.181881159	0.135104312	-1.346227642	0.178229113	0.73985366
EEF1A1	89609.06931	-0.287696074	0.21373266	-1.346055746	0.178284539	0.73985366
GREB1	14.9738552	-0.69853108	0.518977186	-1.345976468	0.178310106	0.73985366
TMEM168	767.921771	0.145605485	0.108190075	1.345830337	0.178357239	0.73985366
METTL6	239.773265	-0.199317405	0.148107727	-1.34575966	0.178380039	0.73985366
PRUNE1	354.2587318	0.262163853	0.194812624	1.345723127	0.178391825	0.73985366
SPOCK1	1207.699852	-0.291476425	0.216600494	-1.3456868	0.178403545	0.73985366
JAM3	951.4343692	-0.131107306	0.097450344	-1.3453755	0.178504003	0.739940733
ZFP69B	109.9782578	-0.294478963	0.21890473	-1.345238008	0.178548386	0.739940733
LAMA2	3359.415226	-0.177423429	0.131890212	-1.345235762	0.178549111	0.739940733
DCXR	578.5353155	0.1798483	0.133717494	1.344987067	0.178629411	0.740070641
RGS7	34.20293396	0.456016773	0.339103363	1.344772192	0.178698813	0.740070641
ZBTB49	34.22271142	0.593229409	0.441143807	1.344752889	0.178705049	0.740070641
SAMD15	7.355877333	-1.022238503	0.760517011	-1.344136277	0.178904323	0.740456972
MED30	194.8204803	0.241223012	0.179466173	1.34411409	0.178911496	0.740456972
NRP1	1069.087553	-0.176339525	0.131197337	-1.344078536	0.178922992	0.740456972
TGFBRAP1	405.4019655	0.171962679	0.127956008	1.343920323	0.178974154	0.74049673
ANKRD36B	488.6293112	0.482412785	0.359039614	1.343619941	0.179071318	0.740726761
PXN-AS1	71.28975914	0.431113793	0.320924605	1.343349143	0.179158946	0.740857041
RPAP2	501.8594149	-0.187662481	0.139706153	-1.343265676	0.179185962	0.740857041
BDH2	221.6634143	-0.239605777	0.178443233	-1.342756307	0.179350896	0.74129917
TOB1	406.1415854	-0.177475767	0.132189273	-1.342588274	0.179405329	0.74129917
KIAA0825	89.98239624	-0.32003693	0.238379877	-1.342550109	0.179417695	0.74129917
TMEM234	148.3041993	0.403993469	0.301031231	1.34203175	0.179585701	0.741721521
STRIP2	257.6819844	0.294412872	0.219387273	1.341977895	0.179603163	0.741721521
LRPAP1	712.5762995	-0.17584339	0.131047651	-1.341827871	0.179651813	0.741750535
COCH	1969.952463	-0.160602138	0.119710951	-1.34158267	0.179731348	0.741907025
SBNO1	3575.557178	0.111848715	0.083391077	1.341255193	0.179837612	0.742099709

CCDC22	189.4741089	-0.200350897	0.149414945	-1.340902664	0.179952057	0.742099709
STARD3	611.0431396	-0.185650869	0.138455264	-1.340872599	0.17996182	0.742099709
NT5E	364.26878	-0.558141458	0.416253075	-1.340870474	0.17996251	0.742099709
MIR6739	6.853421647	1.2932297	0.964522845	1.340797376	0.179986248	0.742099709
NUMA1	1367.88088	-0.159077738	0.11867439	-1.340455497	0.180097303	0.742385831
SRSF6	3325.127186	-0.323789578	0.241644003	-1.339944605	0.180263355	0.742843602
ZNF121	3407.256621	0.161828412	0.120789177	1.3397592	0.180323644	0.742843602
PRKAA2	108.173212	0.330493319	0.246687612	1.339724019	0.180335086	0.742843602
COL3A1	204252.6627	-0.241275126	0.180115388	-1.339558655	0.180388873	0.742843602
SPECC1	389.2998206	0.331498852	0.247486865	1.339460391	0.180420841	0.742843602
CENPX	414.8212239	0.245857149	0.183565248	1.339344737	0.180458471	0.742843602
TTLL12	1780.185715	-0.151919959	0.113442457	-1.339180796	0.180511823	0.742891612
DCAF11	761.4366474	-0.244054001	0.18225915	-1.339049378	0.180554599	0.742896086
KCTD10	1745.646772	-0.134617411	0.100552364	-1.338779172	0.180642573	0.742943645
SMC1B	6.102742395	-1.115561133	0.833280787	-1.338757776	0.180649541	0.742943645
ACAD10	1330.898252	-0.233417823	0.174395367	-1.338440507	0.180752882	0.743175704
CCNH	387.9242538	0.295505122	0.220801636	1.338328498	0.180789376	0.743175704
UCHL1	2917.225898	-0.155947848	0.116545241	-1.338088512	0.180867585	0.743215226
LOC401261	147.5776799	0.411462038	0.307510314	1.33804305	0.180882404	0.743215226
GNB3	100.1685528	0.496092869	0.370881398	1.337605153	0.181025184	0.743429183
DOK7	4.4926426	1.518963172	1.135587812	1.337600806	0.181026602	0.743429183
SH3BGR	3.523929056	1.518555064	1.135423446	1.337435007	0.181080684	0.743429183
C9orf131	14.23825637	0.851302081	0.636597382	1.337269214	0.181134776	0.743429183
RASL10B	335.1690661	-0.194234727	0.145251625	-1.337229291	0.181147804	0.743429183
B4GALNT1	1270.374165	0.164374368	0.122932022	1.337115957	0.181184789	0.743429183
LOC400684	27.40082738	0.570962165	0.427133862	1.336728871	0.181311154	0.74377642
LOXL2	5048.400152	-0.182638047	0.136643846	-1.336599137	0.181353521	0.743778997
ACAD8	225.4773679	-0.23252729	0.173990923	-1.336433453	0.181407638	0.743829754
SS18L1	377.5623294	0.285957088	0.213995092	1.336278719	0.18145819	0.74386587
SNRPB	2433.030802	-0.200193957	0.149861825	-1.33585692	0.181596044	0.744259776
POLR2H	1202.408087	0.140207489	0.104984756	1.335503311	0.181711672	0.744441103
C9orf163	8.162910214	-1.008844213	0.755424792	-1.335466115	0.181723838	0.744441103
PDHX	466.7192871	0.16025753	0.120020066	1.335256144	0.181792527	0.744460134
MIR933	3.674211743	1.484441292	1.111777387	1.335196515	0.181812037	0.744460134
EDA2R	2.656836062	-1.565294759	1.172478318	-1.335030879	0.18186624	0.744511004
PSMC4	1033.316599	-0.22422685	0.168027709	-1.334463529	0.182051992	0.7450647
ATP2A2	9699.167123	0.119024012	0.089199165	1.334362401	0.182085116	0.7450647
ZNF713	17.48327721	-0.719558643	0.539425007	-1.333936385	0.182224706	0.74523074
SACS	4762.598766	0.180872352	0.135601482	1.333852328	0.182252257	0.74523074
TRPM2	3.578705687	1.891513037	1.418138639	1.333799803	0.182269476	0.74523074
ZMYM1	769.3849133	0.181501367	0.136091391	1.333672654	0.182311161	0.74523074
ARHGAP11B	272.3152342	0.538383204	0.403710472	1.333587412	0.182339111	0.74523074
USP30-AS1	7.168304882	1.200122134	0.900053698	1.333389482	0.182404022	0.74523074
WDR74	402.0051575	-0.20605058	0.15453652	-1.333345546	0.182418434	0.74523074
RPS5	6259.312748	-0.232169943	0.174146709	-1.333185935	0.182470794	0.74527379
EGR1	209.5387732	-1.216312465	0.912552945	-1.332867832	0.182575181	0.745386213
SNORD116-13	44.47104433	0.617098543	0.463007638	1.332804239	0.182596054	0.745386213
H3-5	7.820395097	-1.418188508	1.064181102	-1.332657105	0.182644356	0.745386213
DBNDD1	95.44698311	0.564345138	0.423494221	1.332592299	0.182665634	0.745386213
MIR1207	16.4920025	1.202797758	0.902836736	1.332242819	0.18278041	0.745517329
HAS3	23.96487376	0.870640172	0.653593259	1.332082545	0.182833065	0.745517329
IQGAP2	102.323897	0.46807841	0.351427595	1.331934134	0.182881833	0.745517329
RRBP1	4805.72602	0.311702699	0.234043794	1.331813561	0.182921461	0.745517329
CAMK1	62.08927076	-0.671582849	0.504282056	-1.331760354	0.18293895	0.745517329
DEPTOR	184.386989	-0.416803898	0.312979176	-1.331730445	0.182948781	0.745517329
MCEE	144.8139034	0.321863817	0.241726546	1.331520359	0.183017851	0.745573584
PSME1	471.5713666	-0.279911585	0.210237079	-1.33140922	0.183054397	0.745573584
CENPA	410.1764407	0.211467167	0.158915075	1.330692927	0.183290071	0.745573584
NRIP1	1023.330523	0.149669344	0.112491193	1.330498321	0.183354139	0.745573584
ASAH2B	74.16417546	0.305643305	0.22973963	1.330389995	0.183389809	0.745573584
LINC01278	85.8592086	-0.295296055	0.221973828	-1.330319241	0.18341311	0.745573584
SCARA5	197.2158039	1.58650074	1.192658496	1.330222143	0.18344509	0.745573584
TPM3	2902.912386	-0.192008383	0.144344062	-1.330213245	0.183448021	0.745573584
GPR162	223.8273547	0.536022356	0.40298736	1.330122007	0.183478076	0.745573584
PSPN	17.99100993	0.717140853	0.539171434	1.330079465	0.183492091	0.745573584
INSIG1	1603.366531	0.632976701	0.475933063	1.329970011	0.183528152	0.745573584
MIR3177	3.340325419	1.718769579	1.292471404	1.329831805	0.183573694	0.745573584
SNORD10	21.56336464	0.631128146	0.474612689	1.329775122	0.183592375	0.745573584
LOC153684	79.84271516	0.377004241	0.283514734	1.329751844	0.183600047	0.745573584
MTMR9	1036.791509	0.14766712	0.111050108	1.329734139	0.183605882	0.745573584
TOX2	9.448404453	-1.162728666	0.874534953	-1.329539387	0.18367008	0.745573584
LPP	5717.257569	0.11349012	0.085368839	1.329409192	0.183713007	0.745573584
OSCP1	59.00383484	-0.403382538	0.303431787	-1.329401053	0.183715691	0.745573584
FAM86B1	49.19270641	-0.360288978	0.271046649	-1.32925081	0.183765237	0.745604855
CSF1	2019.087152	0.317139161	0.2386673	1.328791844	0.183916654	0.74570735
CELF2	177.9312285	-1.63715748	1.232102088	-1.328751486	0.183929973	0.74570735
HLA-F-AS1	58.16533363	0.415543495	0.312751669	1.328669151	0.183957147	0.74570735
TMEM59	1500.522355	-0.219633217	0.165324467	-1.328497957	0.184013658	0.74570735
SHF	58.20790245	-0.35616316	0.268157283	-1.328187533	0.184116162	0.74570735
ABCF3	1622.492981	-0.140693216	0.105945967	-1.327971418	0.184187549	0.74570735
CLPX	1202.592637	-0.150540598	0.113367283	-1.327901612	0.184210612	0.74570735
ACER2	10.63059557	-0.799102954	0.601935432	-1.327555932	0.184324851	0.74570735
KANK2	943.2549856	-0.239507378	0.180412682	-1.327552895	0.184325855	0.74570735
LTA4H	3558.156416	-0.246506964	0.185701677	-1.327435316	0.184364724	0.74570735
TMCO4	142.7156326	-0.288777183	0.217579465	-1.327226275	0.184433843	0.74570735
SNORD24	23.17096063	0.582964552	0.439255192	1.32716599	0.18445378	0.74570735
RBM20	79.55342935	-0.324365378	0.244414374	-1.32711253	0.184471461	0.74570735
RPS20	10077.57917	-0.219815023	0.165645728	-1.327018969	0.184502408	0.74570735
ZNF226	243.0806403	0.236750184	0.178422166	1.32691015	0.184538406	0.74570735
DYNLRB1	119.3888149	0.331049468	0.249511158	1.326792238	0.184577419	0.74570735

VCPIP1	1900.364873	-0.143846469	0.108426684	-1.326670368	0.184617747	0.74570735
MIR503HG	22.94417593	-0.731734801	0.551560941	-1.326661745	0.184620601	0.74570735
LOC100287846	3.592710034	-1.483447937	1.118450038	-1.326342604	0.184726241	0.74570735
LINC01239	125.6712892	-0.339213433	0.255752185	-1.326336402	0.184728294	0.74570735
PTPMT1	219.4734002	0.198545859	0.149695534	1.326331209	0.184730014	0.74570735
BTN3A1	628.3669674	0.255728065	0.192872549	1.325891457	0.184875653	0.74570735
PDCD6IP	2087.925763	-0.128676596	0.097058538	-1.32576277	0.184918289	0.74570735
SNX19	1087.002119	0.125877304	0.094950762	1.325711354	0.184935325	0.74570735
LOC105371763	69.65730356	0.478578583	0.36104033	1.325554358	0.184987353	0.74570735
IER5	366.2964678	0.30294617	0.228544572	1.325545242	0.184990374	0.74570735
ITGBL1	23.91493679	-0.635253726	0.479249301	-1.325518316	0.184999299	0.74570735
TAB3-AS1	4.383360614	1.25907421	0.949906722	1.325471418	0.185014843	0.74570735
SNORA30	4.641028939	1.724507768	1.30105482	1.325468951	0.185015661	0.74570735
POLR2E	934.387706	-0.190374246	0.143637728	-1.325377735	0.185045898	0.74570735
TMEM255A	33.13644997	-0.599959806	0.452873265	-1.324785216	0.1852424	0.746306515
SENP6	1979.880961	-0.131544624	0.099303177	-1.324676893	0.18527834	0.746306515
USP32P3	23.85256454	0.631817852	0.477202387	1.324003966	0.185501728	0.746785593
CAVIN3	234.6270423	-0.21837868	0.16494707	-1.323931854	0.185525678	0.746785593
RFX7	1453.337026	0.138515337	0.104648801	1.32362087	0.18562899	0.746785593
SLC25A25-AS1	80.41459792	0.377810373	0.285450388	1.323558801	0.185649615	0.746785593
SUN1	2916.571738	-0.109686401	0.082874439	-1.323525111	0.185660811	0.746785593
ZRSR2	57.28032524	0.426057825	0.321917057	1.323501864	0.185668537	0.746785593
ANK2	511.9366742	-0.230773291	0.174382584	-1.323373501	0.1857112	0.746785593
LIFR-AS1	20.70070217	-0.603995361	0.456427951	-1.32330932	0.185732534	0.746785593
ACVR2B	357.7630615	0.181544724	0.137220039	1.323019035	0.185829048	0.746865493
IL4R	141.3399089	0.448946514	0.339351871	1.322952819	0.185851068	0.746865493
PDGFRA	2152.814889	-0.211577822	0.159938312	-1.322871422	0.18587814	0.746865493
MCFD2	2039.562401	-0.172205844	0.130194264	-1.322683799	0.185940553	0.74694785
GPN3	416.8499677	0.218699263	0.165426804	1.322030393	0.18615803	0.747376442
ZBTB4	1219.410179	0.291844603	0.220787145	1.321836933	0.186222457	0.747376442
MARCHF1	108.1006159	0.610523026	0.461953166	1.321612387	0.186297257	0.747376442
ZZZ3	1418.640129	-0.155369682	0.117563287	-1.32158335	0.186306931	0.747376442
LARP7	230.6281285	-0.24363162	0.184352478	-1.32155327	0.186316953	0.747376442
QRFPR	96.71909625	0.29356317	0.222136642	1.32154321	0.186320305	0.747376442
LOC100129917	74.29003242	0.63258475	0.478693566	1.321481621	0.186340827	0.747376442
PVRIG	19.26481697	0.844227224	0.638979678	1.321211382	0.186430892	0.747569419
DTX2P1-UPK3BP1-PMS2P11	20.60183497	0.742468959	0.562205725	1.320635716	0.186622858	0.747834984
ATF6	1427.577925	-0.213866095	0.161971786	-1.320391044	0.186704492	0.747834984
BCAS3	484.8157542	-0.296012451	0.224197064	-1.320322602	0.186727332	0.747834984
SLITRK6	64.58620024	0.40641982	0.307860346	1.320143451	0.186787128	0.747834984
DKK2	3.922494351	-1.515602911	1.148219765	-1.319958911	0.186848737	0.747834984
SAYSD1	508.5504996	0.212522963	0.161018845	1.319863917	0.186880457	0.747834984
LOC102031319	6.99173936	1.131994343	0.857678491	1.319835293	0.186890015	0.747834984
DDX27	396.6496727	-0.193314383	0.146484892	-1.319688196	0.186939143	0.747834984
DPM2	312.948226	-0.210325293	0.159397769	-1.319499601	0.187002143	0.747834984
RASGRP3	17.71118883	0.749031652	0.567756477	1.319283323	0.187074411	0.747834984
ALDH4A1	254.3952108	-0.198113604	0.150169066	-1.319270401	0.187078729	0.747834984
ARL15	165.7944163	0.2334922	0.176989705	1.319241709	0.187088318	0.747834984
RFTN1	467.9671695	-0.506311354	0.383809104	-1.319174947	0.187110632	0.747834984
PAM	3269.077296	-0.227228206	0.172252525	-1.319157474	0.187116472	0.747834984
GABPB1-AS1	303.9246199	0.302739489	0.229529188	1.318958566	0.187182965	0.747834984
RASL11A	8.937925622	0.981258504	0.744006828	1.318883735	0.187207985	0.747834984
S100G	2.839171563	1.975945439	1.49836662	1.318732954	0.187258406	0.747834984
SNORD83B	10.99458668	0.891402357	0.67598201	1.318677633	0.187276908	0.747834984
MYPOP	68.45368914	0.342962588	0.260114724	1.318505089	0.187334623	0.747834984
MIR181A2HG	12.59080073	0.949980009	0.720561909	1.318387771	0.187373872	0.747834984
RTCB	1022.723074	-0.171667562	0.130227765	-1.318210148	0.187433309	0.747834984
MRPL47	818.6740023	-0.195122014	0.148022899	-1.318188029	0.187440712	0.747834984
CTSZ	402.0578465	-0.230607128	0.17495109	-1.318123411	0.187462339	0.747834984
SLC27A5	18.78881453	0.620785997	0.471013421	1.317979422	0.187510537	0.747859839
DACT1	182.0674841	0.281399004	0.213565626	1.317623109	0.187629846	0.748095176
LOC100128882	29.63199401	0.818307831	0.621238084	1.31722097	0.187764567	0.748095176
ANKRD54	279.537895	-0.204125715	0.15499534	-1.316979689	0.187845433	0.748095176
KRBA1	133.4447183	0.398744218	0.302774534	1.316967492	0.187849521	0.748095176
ST7-OT4	57.79624483	0.544572914	0.413523278	1.316909937	0.187868815	0.748095176
EBNA1BP2	619.3957368	-0.303014422	0.230109578	-1.316826638	0.187896742	0.748095176
PRXL2B	117.40515	-0.338225747	0.256852673	-1.316808358	0.18790287	0.748095176
MED1	2305.341187	0.120964123	0.091862124	1.316800851	0.187905388	0.748095176
CAP1	1976.437924	-0.188402065	0.143117516	-1.316415138	0.188034744	0.748440049
ABTB1	160.3132399	-0.456310262	0.346676936	-1.316240613	0.188093296	0.748440049
TMEM86A	17.37693798	-0.720332807	0.547332203	-1.316079711	0.18814729	0.748440049
TBC1D20	543.6806214	-0.217626209	0.165367176	-1.316018173	0.188167943	0.748440049
CCDC167	108.4519977	0.303350344	0.230533577	1.315861871	0.188220408	0.748440049
AGO3	1412.598519	0.247843022	0.188381216	1.315646156	0.188292834	0.748440049
SNORA16B	2.76448758	-2.233845489	1.698124532	-1.315478015	0.188349301	0.748440049
ZAR1	26.22143511	0.61450609	0.467146097	1.315447337	0.188359605	0.748440049
RBAK	459.289259	0.178071724	0.1353997	1.315155973	0.188457488	0.748440049
FARP2	270.1821827	0.184335565	0.140216143	1.314652945	0.188626568	0.748440049
POLE3	1326.821086	-0.172607385	0.131359464	-1.314007988	0.188843516	0.748440049
CCNB1IP1	1008.633124	-0.16837694	0.128140814	-1.3139993	0.18884644	0.748440049
ARL4A	416.1899815	0.343411245	0.261355679	1.313961291	0.188859231	0.748440049
KDM2B	1200.695749	0.124239863	0.094574517	1.313671665	0.18895672	0.748440049
YWHAZ	22432.27565	-0.163012538	0.124092284	-1.313639595	0.188967517	0.748440049
RUSC1-AS1	190.647485	0.50600162	0.385208982	1.313576899	0.188988627	0.748440049
FAIM	31.40189388	-0.459905643	0.350123715	-1.313551819	0.188997072	0.748440049
ELP4	274.3451856	-0.200248661	0.152448391	-1.313550498	0.188997516	0.748440049
ZC3H18	545.8555096	-0.17002414	0.129440196	-1.313534322	0.189002963	0.748440049
GATA2	171.794283	0.300775659	0.229000567	1.313427574	0.189038911	0.748440049
CCDC178	9.353304083	-1.132095309	0.862164263	-1.313085403	0.189154172	0.748440049
MELK	750.459362	-0.176188232	0.134198782	-1.312889946	0.189220035	0.748440049

TNPO1	5286.847239	0.125710836	0.095751959	1.312880042	0.189223373	0.748440049
DPF2	444.5957081	-0.235618181	0.179504977	-1.312599712	0.189317867	0.748440049
CDR2L	512.7836127	0.257338842	0.196053245	1.312596699	0.189318883	0.748440049
GEMIN7	320.1355974	0.195777668	0.14917409	1.31241068	0.189381605	0.748440049
ZNF567	147.9568112	0.27752167	0.211460672	1.312403238	0.189384115	0.748440049
PLAT	32.08674449	-0.606302616	0.462069893	-1.312144819	0.189471276	0.748440049
RN7SL3	1577.114735	-0.737704932	0.562282384	-1.311983007	0.189525868	0.748440049
CDCA7L	1039.279117	0.227588842	0.173470663	1.311973088	0.189529215	0.748440049
FBXO48	76.65106659	-0.317976202	0.242446252	-1.311532761	0.189677833	0.748440049
NAPSA	18.06779417	0.62082619	0.473372931	1.311494909	0.189690612	0.748440049
INTS10	2949.233677	0.117316353	0.089454792	1.311459689	0.189702504	0.748440049
DDX3Y	3.612796276	-1.667851189	1.271927175	-1.311278839	0.189763575	0.748440049
XRN2	1614.879767	-0.18344278	0.139912916	-1.311121125	0.189816845	0.748440049
RANBP3L	31.74524764	0.67402685	0.514136744	1.310987512	0.189861983	0.748440049
MORN2	75.99056738	0.329405839	0.251282512	1.310898387	0.189892096	0.748440049
CRIP2	45.84286689	-0.48165271	0.36763935	-1.310122841	0.190154283	0.748440049
KCNQ1OT1	2536.218925	0.747199687	0.570411679	1.309930554	0.19021933	0.748440049
GSN-AS1	97.31230299	0.551592787	0.421091975	1.309910469	0.190226125	0.748440049
ANGPT2	114.5480641	1.022855719	0.78087333	1.309886867	0.190234111	0.748440049
LINC01963	51.09715799	-0.538338216	0.410988959	-1.309860531	0.190243021	0.748440049
SNORA79B	31.54288689	0.717029655	0.54752328	1.309587521	0.190335412	0.748440049
ENAH	7591.578713	0.334716844	0.255590071	1.309584694	0.190336369	0.748440049
LOC221946	88.06514949	0.690313384	0.52718911	1.309422692	0.190391209	0.748440049
NCL	7337.31049	-0.190157713	0.145241149	-1.309255083	0.190447959	0.748440049
HSBP1L1	8.136878056	1.151849412	0.879866067	1.30911903	0.190494034	0.748440049
RYBP	589.6444078	-0.235244152	0.179703849	-1.309065735	0.190512085	0.748440049
ZC3H11B	23.1996331	-0.558831046	0.426913723	-1.309002301	0.190533571	0.748440049
SLC22A4	27.96984888	0.493329093	0.376877207	1.308991587	0.190537201	0.748440049
LINC00533	2.393363725	1.886430133	1.441143769	1.308981224	0.190540711	0.748440049
SPECC1L	432.6682292	-0.171268931	0.130844763	-1.308947545	0.19055212	0.748440049
LOC100129083	1.765267438	2.342691161	1.789946371	1.308805224	0.190600337	0.748440049
H3P4	9.875917602	-0.935692652	0.71498921	-1.308680802	0.190642497	0.748440049
TP53INP1	303.4907656	-0.293843296	0.224542295	-1.308632283	0.19065894	0.748440049
FRMD5	157.9499888	0.383969555	0.293438626	1.308517424	0.190697869	0.748440049
AHCTF1	3565.150587	0.181059154	0.138370985	1.308505204	0.190702011	0.748440049
PPIE	511.3686129	-0.136070153	0.10399041	-1.308487505	0.19070801	0.748440049
PEG13	9.062111028	1.153618858	0.881651315	1.308475173	0.19071219	0.748440049
KIFC1	1070.169805	-0.140705045	0.107584734	-1.307853254	0.190923087	0.748440049
ID1	1346.516495	0.440924421	0.33716765	1.307730506	0.190964733	0.748440049
VASH1-AS1	13.125407	-0.747209765	0.571385352	-1.307715996	0.190969656	0.748440049
ARSL	903.8151893	0.17069168	0.13053424	1.307639134	0.190995737	0.748440049
LOC257396	30.16941694	-0.518010837	0.396165835	-1.307560601	0.191022388	0.748440049
LOC171391	64.44489461	0.598150255	0.457461869	1.30754123	0.191028962	0.748440049
YY1AP1	756.207971	-0.153074572	0.117070669	-1.307539907	0.191029411	0.748440049
SNHG17	428.9855124	0.4612186	0.352740207	1.307530561	0.191032583	0.748440049
ZBTB18	137.2555624	-0.434841624	0.332578196	-1.307486866	0.191047413	0.748440049
TCAP	4.375819433	1.437517869	1.099585948	1.307326519	0.191101841	0.748440049
LINC02257	82.85238803	-0.327732905	0.250706789	-1.307235862	0.191132619	0.748440049
C1orf216	327.7416471	-0.297201326	0.227353563	-1.307220881	0.191137706	0.748440049
ROR1	269.1299885	0.221658749	0.169575303	1.307140513	0.191164994	0.748440049
UGGT2	936.3187219	0.169569488	0.129725962	1.307136097	0.191166494	0.748440049
OSBPL10	39.07936105	0.544375397	0.416469917	1.307118171	0.191172581	0.748440049
SCIN	15.15605851	0.675030045	0.516465795	1.307017911	0.191206628	0.748440049
EFS	7.506212719	1.231390913	0.942191596	1.306943214	0.191231998	0.748440049
RHOBTB2	1000.733356	0.204990848	0.156874347	1.306720004	0.191307822	0.748440049
TMEM51	11.58892613	-0.769844796	0.589164624	-1.306671795	0.191324202	0.748440049
NBPF25P	228.0071837	0.547474965	0.41908151	1.306368696	0.191427206	0.748440049
SH3BGRL2	93.55150675	0.302698336	0.231719975	1.30631093	0.191446842	0.748440049
LINC00869	25.28717508	0.532356706	0.407548918	1.306240018	0.191470948	0.748440049
LRRC66	61.88318936	-0.470799777	0.360430897	-1.306213704	0.191479894	0.748440049
CDCA4	892.5980252	-0.196536811	0.15046887	-1.30616261	0.191497265	0.748440049
LOC100288846	8.879858702	-1.258586603	0.963695093	-1.306000842	0.191552271	0.748440049
RBBP6	674.6714681	0.151650199	0.116120542	1.305972197	0.191562013	0.748440049
LINC00705	27.58569748	0.625247508	0.478827255	1.305789305	0.191624218	0.748518976
FAM135A-AS1	3.361899336	1.554805616	1.190928364	1.30554084	0.19170875	0.748685059
ITGAE	202.8402373	0.251309744	0.192517555	1.305386121	0.191761402	0.748726595
BCL11A	292.543966	-0.246284597	0.188724646	-1.30499435	0.191894772	0.748912474
HES4	82.04179515	0.320180711	0.245377972	1.304847003	0.19194495	0.748912474
CR1	7.868305612	-0.993092784	0.761113122	-1.304789991	0.191964368	0.748912474
RAB11FIP1	813.7423726	0.190890245	0.146303789	1.30475257	0.191977114	0.748912474
RNF217	1103.372741	0.160937265	0.123369542	1.304513756	0.192058472	0.749065874
IGDCC3	94.95240896	-0.487509716	0.373834541	-1.304078844	0.192206701	0.74947996
SORT1	622.6668651	-0.296000872	0.227097591	-1.303408243	0.192435424	0.750088355
BMP4	270.7726093	0.345576461	0.26513979	1.303374578	0.192446911	0.750088355
NUTM2A	17.72982035	0.684139575	0.525005813	1.303108571	0.192537698	0.750122252
CPQ	512.7791974	-0.225598239	0.173168749	-1.302765305	0.192654899	0.750122252
NEK9	4915.74698	-0.231243754	0.177517205	-1.302655448	0.192692419	0.750122252
LEFTY1	1.887325505	1.858566432	1.426849272	1.302566759	0.192722712	0.750122252
TRMT61B	330.7237085	0.192365095	0.147689229	1.30249915	0.192745808	0.750122252
LOC101928595	4.745777217	1.203886225	0.924322024	1.30245325	0.192761489	0.750122252
ZNF837	28.15130017	0.613062356	0.47079368	1.302189011	0.192851781	0.750122252
NELFA	345.1237177	-0.181226076	0.139172619	-1.302167606	0.192859096	0.750122252
UBXN6	468.576315	-0.24290606	0.186553291	-1.302073303	0.192891328	0.750122252
FAM238C	11.10506194	0.839981674	0.645184367	1.301925026	0.192942016	0.750122252
LENG8	2829.110624	0.374264912	0.287475387	1.301902454	0.192949734	0.750122252
TACO1	244.3576906	0.201677844	0.154913934	1.301870259	0.192960741	0.750122252
STX4	136.9184352	-0.231442365	0.177794706	-1.301739347	0.193005504	0.750132624
PPARGC1B	624.6866168	0.271200604	0.20842827	1.301169961	0.193200285	0.750521609
LIN28B	376.002402	0.219574513	0.168761578	1.301093027	0.193226614	0.750521609
AKIP1	144.3833582	-0.238340669	0.183187146	-1.301077471	0.193231938	0.750521609

HNRNPF	2950.849777	-0.166900428	0.128301219	-1.300848349	0.193310369	0.750662621
ADGRL3	390.0480347	0.224866252	0.172909189	1.30048758	0.19343391	0.750843261
DMWD	921.9639663	0.146548984	0.11269549	1.300397953	0.193464611	0.750843261
NMB	48.75350642	-0.418501081	0.321915382	-1.300034431	0.193589169	0.750843261
ACMSD	4.17578367	-1.78963252	1.376622066	-1.300017313	0.193595035	0.750843261
LOC441081	1.963762955	2.103186909	1.617842229	1.299995062	0.193602662	0.750843261
C6orf89	2361.77127	-0.1630595	0.125440629	-1.299893829	0.193637361	0.750843261
DLEC1	22.61629458	0.559418059	0.430370707	1.299851615	0.193651831	0.750843261
LINC01301	16.51206	0.885489556	0.681292578	1.299719952	0.19369697	0.750854905
MYO18A	1292.671059	0.212176776	0.163278147	1.299480545	0.193779066	0.750896768
SYTL5	3.043692655	-1.551174564	1.193722939	-1.299442703	0.193792045	0.750896768
JRK	1768.915474	0.350482481	0.269776568	1.299158348	0.193889592	0.751019184
TCTE3	36.47750068	0.680008436	0.523492331	1.298984524	0.19394924	0.751019184
PPP3CA	1895.831964	-0.137664214	0.105982414	-1.298934501	0.193966408	0.751019184
CSTF3	841.015558	-0.162931171	0.125448218	-1.29879223	0.194015242	0.751019184
PPP1R18	1461.219879	-0.241189829	0.185745088	-1.298499099	0.194115886	0.751019184
MICB	69.49513827	0.379156893	0.292037963	1.298313714	0.194179556	0.751019184
CCDC126	127.3703638	0.23886947	0.18398533	1.298307154	0.194181809	0.751019184
MRPS30	426.0368212	-0.164847588	0.126978393	-1.298233375	0.194207153	0.751019184
LOC102724301	4.940500186	-1.394261248	1.073989484	-1.298207542	0.194216027	0.751019184
COL4A5	701.0213439	-0.227592837	0.17532456	-1.298122961	0.194245085	0.751019184
C12orf10	262.7914257	-0.216230954	0.166624064	-1.297717439	0.194384449	0.751181232
ABTB2	159.2021649	-0.25524537	0.196692785	-1.297685473	0.194395437	0.751181232
SRSF10	2190.608021	-0.286789023	0.22105951	-1.297338544	0.194514728	0.751181232
TRPM6	15.67162405	-0.62611781	0.482689442	-1.2971442	0.194581576	0.751181232
HDHD3	149.2720229	-0.274993338	0.212015099	-1.297046011	0.194615356	0.751181232
BNC2-AS1	18.62268032	0.949957906	0.732419021	1.297014248	0.194626284	0.751181232
DCST1-AS1	17.21997846	0.605103425	0.466559481	1.296948083	0.194649051	0.751181232
CEP85L	454.4978435	0.172903385	0.133318841	1.296916351	0.19465997	0.751181232
LOC100506302	24.05318342	0.706006939	0.54469109	1.296160251	0.194920281	0.751181232
IRF3	636.7262228	0.146353042	0.11292028	1.296074025	0.194949983	0.751181232
GGCX	1719.360334	0.15719172	0.121290165	1.295997251	0.194976432	0.751181232
SLC35G6	21.2863624	0.819660818	0.632462119	1.295984048	0.194980981	0.751181232
SERPINE3	6.015804458	-1.07155947	0.826897973	-1.2958787	0.195017279	0.751181232
TMEM11	891.5851945	0.145444066	0.112238546	1.295847745	0.195027945	0.751181232
PC	207.7605312	-0.221183257	0.17071821	-1.295604357	0.195111827	0.751181232
TMEM115	398.1733429	-0.18731196	0.144579563	-1.29556319	0.195126018	0.751181232
SCARNA18B	6.001462235	1.354708316	1.04572245	1.295475981	0.195156082	0.751181232
FZD9	29.97944786	0.533355907	0.411746169	1.295351233	0.195199093	0.751181232
PTCSC1	6.764748122	1.22764653	0.947838659	1.295206224	0.195249098	0.751181232
TTPA	25.17119435	0.491690293	0.379626225	1.295195803	0.195252692	0.751181232
CHI3L1	165.9151417	0.968593398	0.747837356	1.295192584	0.195253802	0.751181232
LOC645967	4.655516756	-1.07941668	0.833403524	-1.295190924	0.195254375	0.751181232
KLHL22	255.4917771	0.289892479	0.22382328	1.295184659	0.195256536	0.751181232
LINC02595	44.94070725	0.857655863	0.662330586	1.29490602	0.195352651	0.751193931
C3orf14	72.904665	-0.280696434	0.216782705	-1.294828542	0.195379383	0.751193931
PLEKHA2	4555.732016	0.280485776	0.216625591	1.294795206	0.195390886	0.751193931
SRCAP	2238.234813	0.158746412	0.122614046	1.294683736	0.195429353	0.751193931
ZFAND1	734.0646262	-0.14707331	0.113608356	-1.294564199	0.195470609	0.751193931
LINC01424	31.68705008	0.566748229	0.437888994	1.294273747	0.195570881	0.751261371
BLOC1S2	222.6228212	-0.219265659	0.169412723	-1.294269132	0.195572475	0.751261371
ZNF826P	2.769771421	-1.551641205	1.199135172	-1.293966887	0.195676859	0.75150035
ZADH2	1026.82459	0.162750019	0.125797229	1.293748834	0.195752191	0.751555392
UQCRC1	1590.998258	-0.158886473	0.122817326	-1.293681262	0.19577554	0.751555392
MAP3K12	177.8315669	-0.268807526	0.207827767	-1.293414878	0.195867607	0.751746879
ABCB7	490.7052008	-0.22268884	0.172220918	-1.293041767	0.195996614	0.751852933
MIR27B	11.89436117	0.939383898	0.726496471	1.293032982	0.195999652	0.751852933
SNX25	240.4372521	0.211301975	0.163423865	1.292968904	0.196021814	0.751852933
SMIM32	12.66211047	1.026559118	0.794143645	1.292661755	0.196128071	0.752063999
CSDC2	5.696000611	1.212287023	0.93807922	1.292307726	0.196250597	0.752063999
BRI3	208.682521	-0.291177802	0.225344994	-1.292142315	0.196307864	0.752063999
MARCHF5	436.8352437	-0.149374997	0.115604399	-1.29212209	0.196314867	0.752063999
IFT81	500.862008	-0.162359822	0.125655983	-1.292097816	0.196323272	0.752063999
TYW1B	30.42685486	0.594869961	0.460397799	1.292078204	0.196330063	0.752063999
RPL29	4439.782045	-0.21745666	0.168338592	-1.291781391	0.196432862	0.752296066
RHBDF1	136.2778036	-0.325896286	0.252337277	-1.291510671	0.196526657	0.752395008
RPS25	2130.920682	-0.233388224	0.180727696	-1.291380512	0.196571765	0.752395008
VPS39	1916.676122	-0.153886214	0.119181091	-1.291196548	0.196635532	0.752395008
DLEU2	293.9950479	0.477475251	0.369796546	1.29118364	0.196640007	0.752395008
SAMM50	595.669458	-0.174052408	0.134819491	-1.291003301	0.196702533	0.752395008
GLOD4	750.394218	-0.154008915	0.119305754	-1.290875836	0.196746736	0.752395008
GSR	3971.221525	0.198332024	0.15364402	1.290854169	0.19675425	0.752395008
HPS3	1065.038526	0.174343452	0.135075585	1.290710321	0.196804144	0.752424341
SERINC3	1821.316449	-0.267157672	0.207046924	-1.290324275	0.196938092	0.752628341
FAHD2A	676.1174707	0.157938873	0.122403526	1.290313093	0.196941973	0.752628341
BST1	2.574622009	1.684196893	1.305571049	1.290007844	0.197047935	0.752871826
RAB10	2708.691143	-0.160542821	0.124464805	-1.289865199	0.197097466	0.752899644
PTMA	3493.40844	-0.197371325	0.15304186	-1.289655817	0.197170187	0.752942659
GINM1	718.5786674	-0.165018319	0.127965206	-1.289556161	0.197204805	0.752942659
CDKN2A-DT	5.880458684	-1.452706593	1.126809431	-1.289221187	0.197321201	0.752942659
ANKRD53	9.832666416	0.926515085	0.718664528	1.289217776	0.197322387	0.752942659
C10orf143	4.543224882	-1.299640082	1.008402213	-1.288811215	0.197463726	0.752942659
SHBG	3.416427705	1.684955616	1.30748172	1.288703001	0.197501359	0.752942659
SPATA6L	4.772550865	1.080089487	0.838130572	1.288688806	0.197506296	0.752942659
CTDSPL2	1865.885927	-0.141645776	0.109921543	-1.288607975	0.19753441	0.752942659
CHMP4B	441.9190283	-0.228348038	0.177220161	-1.288499215	0.197572242	0.752942659
MMP14	23997.95854	-0.426255237	0.330875022	-1.288266591	0.197653178	0.752942659
PIGL	176.7347346	0.238531587	0.185162302	1.288229757	0.197665996	0.752942659
VPS28	827.7218197	-0.129952511	0.100881162	-1.288174203	0.19768533	0.752942659
MTTP	3.118834147	-1.708704334	1.326464693	-1.288164202	0.19768881	0.752942659

EP400P1	229.5507858	0.410650077	0.318795203	1.288131293	0.197700264	0.752942659
PHF8	645.686331	0.155379027	0.120660833	1.287733751	0.197838661	0.753308746
LLGL1	1013.214051	-0.117976317	0.091637058	-1.287430208	0.197944381	0.753351179
ASAH1	1804.602755	-0.134559695	0.104524681	-1.287348538	0.197972833	0.753351179
ABCA2	149.2904216	-0.292624641	0.227322334	-1.287267448	0.198001085	0.753351179
LOC100287175	2.910934874	1.598486712	1.24181671	1.287216301	0.198018907	0.753351179
LYRM4-AS1	84.1971734	-0.337088282	0.261911247	-1.287032483	0.198082967	0.753394886
TTLL4	620.9818522	0.203003311	0.157740996	1.286940719	0.198114951	0.753394886
EIF3I	2281.85129	-0.184261628	0.143211902	-1.286636276	0.198221094	0.753637698
MSH2	1551.360765	-0.173324417	0.134730359	-1.286454049	0.198284646	0.753718512
BAG5	978.4684674	-0.149168007	0.115998526	-1.285947439	0.198461406	0.753719395
COPG2	637.9714261	-0.222795815	0.173261951	-1.285890026	0.198481445	0.753719395
KLF2	42.23776123	-0.473163586	0.367967684	-1.285883534	0.198483711	0.753719395
ZNF519	158.0459442	0.432062878	0.336193172	1.28516256	0.198735484	0.753719395
MARK1	84.13584982	-0.389729976	0.30326489	-1.285114065	0.198752427	0.753719395
CTBP1-DT	289.3888617	-0.268513244	0.208953224	-1.285039971	0.198778317	0.753719395
RPN2	5593.001878	-0.14467008	0.112601811	-1.284793542	0.19886444	0.753719395
MARS2	356.0276316	0.296647031	0.230912829	1.28467107	0.198907252	0.753719395
ZMYND8	646.4690294	-0.234901436	0.182866155	-1.284553918	0.198948211	0.753719395
KCNK1	52.83423238	0.577466689	0.449705123	1.284100755	0.199106704	0.753719395
UTP25	579.7382981	-0.159772007	0.124436664	-1.283962468	0.199155088	0.753719395
MRO	16.42871043	0.636057597	0.495389088	1.283955607	0.199157489	0.753719395
MSH3	497.5352092	-0.147457883	0.114858753	-1.283819292	0.199205192	0.753719395
FANCM	614.9664801	0.207987526	0.162041819	1.283542282	0.199302156	0.753719395
CENPJ	313.8420198	0.271707828	0.211694604	1.283489623	0.199320593	0.753719395
ZNF354A	147.5936101	0.241687764	0.188327772	1.283335759	0.19937447	0.753719395
LAMB2P1	9.755106196	1.168108746	0.910286272	1.283232299	0.199410703	0.753719395
INPP4A	1196.841473	0.169924862	0.132421657	1.283210511	0.199418335	0.753719395
MAN1A1	1370.978928	-0.138648425	0.108049106	-1.283198264	0.199422624	0.753719395
ST7-AS2	14.06701931	1.159438208	0.903562957	1.283184751	0.199427357	0.753719395
ARMC9	191.6512102	-0.250784513	0.195463809	-1.283022743	0.199484108	0.753719395
LINC02541	20.71007259	0.701870436	0.547053947	1.283000405	0.199491934	0.753719395
MKRN1	1047.156501	-0.274667382	0.214091844	-1.282941833	0.199512456	0.753719395
TIMM17A	748.0293317	0.179220152	0.139695083	1.282938152	0.199513745	0.753719395
GRK5	122.5047381	0.518064913	0.403930388	1.282559889	0.19964631	0.753719395
NFE2L2	642.16591	-0.163375373	0.127383123	-1.282551164	0.199649369	0.753719395
CTNNBIP1	187.9601714	-0.434109861	0.338478922	-1.282531445	0.199656282	0.753719395
PMCH	10.47984687	1.202917102	0.938127717	1.282253024	0.199753901	0.753719395
LYRM7	668.2966321	-0.208941659	0.162958359	-1.282178222	0.199780134	0.753719395
PCDH20	112.058691	0.322204781	0.251296843	1.282168042	0.199783704	0.753719395
CAPN14	3.309607172	1.802463902	1.405936849	1.282037599	0.199829457	0.753719395
WIF1	14.54278256	-0.801780398	0.62544047	-1.281945182	0.199861877	0.753719395
OPN1SW	7.84622193	1.081524054	0.843721199	1.281850042	0.199895256	0.753719395
RABEP1	1809.281239	-0.127274562	0.099304057	-1.28166528	0.199960089	0.753719395
HMGN2	3108.116291	-0.25452913	0.198597417	-1.28163364	0.199971194	0.753719395
LOC101928100	11.20512131	-0.854985436	0.667233932	-1.281387824	0.200057479	0.753719395
KCTD18	330.5634878	-0.277983842	0.216950017	-1.281326669	0.200078949	0.753719395
B4GALNT3	303.5218945	0.207309041	0.161797655	1.281285814	0.200093294	0.753719395
TBX20	253.4141919	0.212380021	0.165762845	1.28122814	0.200113545	0.753719395
FTCD	19.33800099	0.758982208	0.59252092	1.280937401	0.200215654	0.753719395
ZSCAN16-AS1	38.25484293	0.423392928	0.33057104	1.280792559	0.200266539	0.753719395
HERPUD2	672.2390416	-0.29199371	0.227998396	-1.280683176	0.200304971	0.753719395
INCA1	231.280243	0.289455789	0.226023325	1.280645655	0.200318156	0.753719395
DLGAP1-AS1	14.09089179	-0.833137149	0.650581024	-1.28060475	0.200332531	0.753719395
CHEK1	600.5101192	-0.142507991	0.111293651	-1.280468289	0.20038049	0.753719395
SERF2	3106.931416	-0.279393843	0.218293802	-1.279898194	0.200580943	0.753719395
GLIPR1L2	2.508548041	1.907628313	1.490649571	1.279729555	0.200640266	0.753719395
HAPLN1	2352.319266	-0.327991809	0.256300824	-1.279714216	0.200645663	0.753719395
METTL25	183.2305778	0.239581391	0.187217855	1.279693069	0.200653103	0.753719395
DMRTA1	121.4391021	0.44700877	0.349326598	1.279629929	0.200675319	0.753719395
INAFM2	381.5168676	-0.171648032	0.134140646	-1.27961239	0.20068149	0.753719395
TMEM171	14.84769892	0.64831223	0.506690943	1.279502306	0.200720228	0.753719395
LOC105372480	12.92135235	-0.737668796	0.576583548	-1.279378849	0.200763678	0.753719395
RPS4X	10609.6364	-0.224311188	0.175357805	-1.279162839	0.200839719	0.753719395
CRY1	538.1401097	0.248478029	0.194267961	1.279047908	0.200880186	0.753719395
CYP2J2	504.6384271	-0.309789184	0.242205322	-1.279035413	0.200884586	0.753719395
LOC100130691	102.6664067	-0.322704606	0.252324549	-1.278926711	0.200922865	0.753719395
ZNF385A	99.00063987	-0.32581096	0.254844101	-1.278471656	0.201083173	0.753719395
ELOVL1	686.2371431	0.150138994	0.117475392	1.278046327	0.201233092	0.753719395
MIRLET7I	7.800615584	0.942864885	0.737793657	1.277952008	0.201266349	0.753719395
EIF4EBP3	3.486621546	-1.305490836	1.021664084	-1.277808289	0.201317031	0.753719395
PGGHG	735.0926872	0.263604661	0.20629446	1.277807758	0.201317218	0.753719395
LBH	284.8201236	0.521322428	0.407997846	1.277757794	0.20133484	0.753719395
SZRD1	2735.735205	-0.234495047	0.183530694	-1.277688448	0.2013593	0.753719395
VMA21	882.2310455	-0.228191601	0.178604244	-1.277638179	0.201377032	0.753719395
YTHDF1	809.7695518	-0.131862384	0.103215607	-1.277543082	0.201410581	0.753719395
SEC31B	191.1269524	0.329802927	0.258163187	1.277497892	0.201426524	0.753719395
CDC42EP2	101.3735345	0.31854423	0.249364303	1.277425141	0.201452193	0.753719395
HTT	2248.390496	0.138777264	0.108659619	1.277174224	0.201540745	0.753719395
CYB5D2	176.5263581	-0.434190004	0.339988496	-1.277072633	0.201576606	0.753719395
KCCAT198	11.38783369	0.736997554	0.577104357	1.277061151	0.201580659	0.753719395
HNRNPC	8284.240021	-0.177424823	0.138939667	-1.276991855	0.201605123	0.753719395
CBX2	283.1773539	-0.33390574	0.261494076	-1.27691512	0.201632215	0.753719395
MALT1	495.9355359	0.295709525	0.231627692	1.27665877	0.201722743	0.753719395
WAKMAR2	2.117699625	1.885043887	1.476807478	1.276431705	0.201802955	0.753719395
TPTE2P5	33.56503464	0.614261857	0.48124486	1.276401906	0.201813483	0.753719395
ACSS1	208.6048387	-0.272632399	0.213599862	-1.276369735	0.20182485	0.753719395
TST	98.47612241	0.267221498	0.20936253	1.276357802	0.201829066	0.753719395
BMS1P4-AGAP5	96.81560503	0.480332288	0.376357813	1.276264958	0.201861873	0.753719395
CEBPB-AS1	16.43583049	-0.720792558	0.56478945	-1.276214627	0.201879659	0.753719395

RER1	1222.18111	-0.130359514	0.102154009	-1.276107665	0.201917462	0.753719395
ZRANB2-AS2	5.628549755	-1.080951694	0.847102976	-1.276057013	0.201935366	0.753719395
SH3RF3	47.55314667	-0.542647126	0.425275136	-1.275990717	0.201958801	0.753719395
LINC00909	235.3738079	-0.26000459	0.203798526	-1.275792298	0.202028951	0.753719395
BRK1	525.6117176	-0.234076672	0.183476616	-1.275784765	0.202031615	0.753719395
LRRC46	24.2932275	0.59330219	0.465081124	1.275696128	0.202062958	0.753719395
TMEM182	111.0222925	0.286319475	0.224450167	1.275648304	0.202079871	0.753719395
SNORD53	7.17268714	1.517984429	1.190006955	1.275609712	0.202093519	0.753719395
PELI1	246.0983439	0.17992921	0.141061007	1.275541794	0.202117541	0.753719395
INE1	88.08069787	0.670144727	0.525401452	1.275490816	0.202135573	0.753719395
GPR182	10.13060238	0.834906912	0.654590003	1.275465417	0.202144557	0.753719395
MPZL1	2570.765584	0.156260332	0.122520977	1.275376156	0.202176134	0.753719395
FBXO10	235.8156821	0.195185709	0.153092661	1.274951441	0.20232643	0.754041676
SLC12A8	52.85930286	0.384257212	0.301412168	1.274856335	0.202360097	0.754041676
LRTOMT	110.2129313	-0.291816756	0.228916605	-1.274773212	0.202389525	0.754041676
LINC00260	14.86301973	0.844268457	0.662493786	1.274379435	0.202528977	0.754403506
CEP83-DT	38.52851485	0.597750389	0.469375419	1.273501691	0.202840073	0.75540441
SPHKAP	22.62926905	0.647509598	0.508521845	1.273317173	0.202905515	0.755490239
LOC101929165	3.735304143	-1.511441345	1.187253541	-1.273056928	0.202997842	0.75567611
ELL2	820.8766704	0.160865133	0.126393681	1.272730814	0.203113579	0.755854473
PPM1B	431.8160429	-0.156682865	0.123119361	-1.272609473	0.203156655	0.755854473
ARHGAP39	151.7803685	-0.354700223	0.278740291	-1.272511489	0.203191445	0.755854473
NSMCE1-DT	3.90556415	1.420369321	1.11634012	1.272344598	0.203250709	0.755854473
DOCK7	1212.734798	-0.215346682	0.169299234	-1.27198852	0.203377198	0.755854473
CITED2	683.2561382	-0.442883949	0.348292265	-1.271587095	0.203519864	0.755854473
GORASP1	347.1237982	0.177176493	0.139342893	1.271514379	0.203545715	0.755854473
KIAA1324L	406.1842347	-0.168555124	0.132562683	-1.271512622	0.20354634	0.755854473
ZBBX	7.722109131	-0.921117629	0.72468799	-1.271054083	0.203709409	0.755854473
CDKN2A	2047.708201	-0.203965332	0.16047099	-1.271041771	0.203713789	0.755854473
CISD2	362.4864361	-0.212782481	0.167416566	-1.270976261	0.203737094	0.755854473
ZMAT2	436.7041385	-0.218291822	0.171758121	-1.270925765	0.203755059	0.755854473
CSF1R	54.66159617	0.455229715	0.358198708	1.270885979	0.203769215	0.755854473
HMGN1	949.6135879	-0.133324838	0.104907138	-1.270884333	0.203769801	0.755854473
ACTR2	10466.35501	-0.148179294	0.116598864	-1.270846815	0.20378315	0.755854473
SNORA65	41.79121562	0.594316296	0.467719047	1.270669433	0.203846274	0.755854473
FAM83D	356.5798196	0.164972044	0.129837641	1.270602591	0.203870065	0.755854473
LIMD1	474.5005587	-0.219869602	0.173073741	-1.270381057	0.203948928	0.755854473
ARL16	307.9644391	0.194799447	0.153347301	1.270315462	0.203972283	0.755854473
HERC2	4353.318908	0.178807847	0.140764501	1.270262361	0.203991191	0.755854473
ZNF17	159.3354069	-0.24559303	0.193362021	-1.270120312	0.204041777	0.755854473
SIAH2	679.5750799	-0.207211955	0.1631562	-1.270021948	0.204076812	0.755854473
MTREX	1303.853386	-0.144675773	0.113940635	-1.269746947	0.204174784	0.755854473
MRPS26	378.5969721	-0.193279982	0.152232798	-1.269634298	0.204214926	0.755854473
CD247	4.837921394	1.204201667	0.94851322	1.269567615	0.204238691	0.755854473
PDCL	444.2684366	0.161324182	0.127092455	1.269345075	0.204318017	0.755854473
MRPL19	1496.018763	0.125207112	0.098641017	1.269320975	0.204326608	0.755854473
NLGN4X	130.9429873	-0.290393404	0.228791886	-1.269246953	0.204353	0.755854473
PWRN3	28.49893563	0.574870179	0.45294006	1.269197031	0.2043708	0.755854473
ZNF841	411.4742031	0.286640549	0.225848349	1.269172654	0.204379492	0.755854473
ZNF814	236.1242187	0.400221772	0.31541039	1.268892161	0.204479528	0.755854473
LINC00963	265.789218	-0.214201506	0.168813176	-1.268867227	0.204488423	0.755854473
BRMS1L	278.3118264	-0.21862333	0.172309985	-1.268779228	0.204519816	0.755854473
MAN1B1	3177.90905	-0.170854644	0.134664613	-1.268741955	0.204533113	0.755854473
C15orf62	110.00196	0.51329995	0.404597625	1.268667727	0.204559598	0.755854473
LOC102724784	3.33104266	1.821464504	1.435771814	1.268630911	0.204572734	0.755854473
GAS7	11.322928	-2.790992138	2.200272033	-1.268475941	0.204628037	0.755902078
LINC00562	8.81610306	-0.949164036	0.748480399	-1.268121432	0.204754589	0.756059166
KREMEN1	406.2733124	-0.350003982	0.276002723	-1.268117859	0.204755865	0.756059166
SOX6	559.6461349	0.203634223	0.160601382	1.267948138	0.204816472	0.756059166
CA14	20.41214415	0.571817443	0.451002277	1.267881498	0.204840272	0.756059166
CAMK4	105.8002199	-0.276239082	0.217979522	-1.267270796	0.205058479	0.756386248
ITM2A	104.5473368	-0.325531321	0.256882413	-1.267238645	0.205069972	0.756386248
LOC100288866	3.003580038	1.892698862	1.493647359	1.267165808	0.205096009	0.756386248
CCNJ	221.7754984	0.316782031	0.250001646	1.267119782	0.205112463	0.756386248
RHEBL1	38.15748972	-0.381928319	0.3014336	-1.267039633	0.205141119	0.756386248
FMNL1	56.23310747	0.52373687	0.413610207	1.266257121	0.205421043	0.757015143
PNN	778.9249635	0.302015755	0.238524441	1.266183684	0.205447327	0.757015143
CCDC8	540.8524953	-0.200423833	0.158303684	-1.266071822	0.20548737	0.757015143
BCAP31	1304.440209	-0.191169969	0.151004292	-1.265990299	0.205516555	0.757015143
SYNPO2	14.88386141	-0.849349775	0.671002229	-1.265792777	0.205587282	0.757015143
MOSMO	215.8675173	-0.220993234	0.174592783	-1.265763853	0.20559764	0.757015143
ABHD11-AS1	2.617169547	1.653753944	1.306677147	1.265617867	0.205649926	0.757015143
RNF181	366.0625027	-0.239032843	0.188873154	-1.265573419	0.205665848	0.757015143
DDB1	4144.028995	-0.176352503	0.139354704	-1.265493717	0.205694399	0.757015143
OBSL1	1358.29142	-0.101693447	0.080370376	-1.26531008	0.205760194	0.757015143
BMP5	77.4786944	0.550595136	0.435185523	1.265196353	0.205800949	0.757015143
EPN2-AS1	18.56383771	0.945810254	0.747593865	1.265139132	0.205821457	0.757015143
ARSK	217.8295239	0.297701113	0.235373264	1.264804286	0.205941495	0.757072886
CMYA5	12.66431304	0.790895595	0.62532162	1.264782105	0.205949448	0.757072886
NUCB2	343.2555048	-0.223220597	0.176535676	-1.26445035	0.206068431	0.757072886
YLPM1	1867.983555	-0.129775626	0.102634477	-1.264444752	0.206070439	0.757072886
TSSC4	242.3248666	-0.207683104	0.164260566	-1.264351565	0.206103869	0.757072886
SCUBE2	8.086437488	-0.94660751	0.748846235	-1.26408796	0.206198457	0.757072886
WDR5B	163.3262237	0.253556284	0.200597028	1.264008182	0.20622709	0.757072886
FBXO5	464.7964652	-0.19839243	0.156963955	-1.263936228	0.206252917	0.757072886
TUFT1	58.67411653	-0.351813674	0.278350991	-1.263921037	0.20625837	0.757072886
BRCC3	397.8401026	-0.2131831	0.168675181	-1.263867621	0.206277545	0.757072886
CENPS-CORT	5.4989284	1.158281093	0.916512023	1.263792579	0.206304486	0.757072886
WWOX	114.142757	-0.265738309	0.21037992	-1.263135327	0.20654055	0.757783118
KIF12	3.276303928	1.653945847	1.309674447	1.262867921	0.20663665	0.757979643

RPS6	4849.423332	-0.267302698	0.211720075	-1.262528825	0.206758561	0.758270746
IFT88	202.1888654	-0.346189033	0.274247232	-1.26232462	0.206832001	0.758384004
SNORD116-20	22.43566015	0.671393234	0.532036707	1.261930287	0.206973873	0.758572498
CLIC3	16.22645166	0.80161789	0.635248388	1.261896771	0.206985934	0.758572498
LOC105372990	2.015730145	2.059587335	1.632455499	1.261649911	0.207074788	0.758572498
MZF1-AS1	92.6902806	0.329274615	0.260991956	1.261627447	0.207082875	0.758572498
ANP32AP1	2.191158732	2.253623405	1.786631172	1.261381442	0.207171451	0.758572498
ANKIB1	1111.242838	0.181758875	0.144101903	1.261321824	0.207192921	0.758572498
NPEPPSP1	170.8629592	0.428546919	0.33976229	1.261313958	0.207195754	0.758572498
PFDN6	122.8407874	-0.262953911	0.208492339	-1.261216174	0.207230973	0.758572498
RNF165	268.1914231	0.31304146	0.248238893	1.261049212	0.207291117	0.758572498
SLC12A2	821.3108708	-0.146562396	0.116242223	-1.260836141	0.20736789	0.758572498
BARHL1	2.70186288	-1.981353076	1.57168535	-1.260655051	0.207433155	0.758572498
CATSPERE	10.81299351	-0.798310768	0.633264494	-1.260627709	0.207443011	0.758572498
BHLHE22	6.09103071	-1.401311128	1.111683842	-1.260530265	0.207478137	0.758572498
PEG10	7370.945966	-0.195044956	0.154732885	-1.260526848	0.207479369	0.758572498
TMEM214	1816.577318	0.162596365	0.129020476	1.260236897	0.207583916	0.758658154
ZNF527	142.7408503	0.24536901	0.194702428	1.260225729	0.207587944	0.758658154
CXXC5-AS1	8.676793365	0.917038115	0.727901748	1.25983777	0.207727892	0.758827622
TRIP4	301.647458	-0.210846921	0.167383255	-1.259665555	0.207790036	0.758827622
MED4-AS1	7.951817565	-0.884987461	0.702587643	-1.25961148	0.207809552	0.758827622
NORAD	6580.232058	-0.225040558	0.178669551	-1.259535033	0.207837145	0.758827622
DLST	1993.521139	-0.158626557	0.125959082	-1.259349897	0.207903978	0.758827622
R3HCC1	343.9997624	-0.196636651	0.15614172	-1.25934728	0.207904923	0.758827622
ACOX1	1123.141424	0.144163993	0.114492732	1.259154108	0.207974674	0.758827622
NFS1	442.1614868	0.184730024	0.146714474	1.259112477	0.207989708	0.758827622
SLC39A8	670.7927321	0.204306932	0.162279688	1.258980311	0.208037444	0.758827622
WDR81	835.1805037	-0.193006348	0.153311359	-1.258917468	0.208060144	0.758827622
CARNMT1-AS1	21.90954661	0.901053255	0.715920208	1.258594527	0.208176825	0.758894956
ZCCHC8	446.403915	-0.19336549	0.153640684	-1.258556557	0.208190547	0.758894956
ZNF350-AS1	4.465828843	1.259308696	1.000632421	1.258512787	0.208206366	0.758894956
LINC01909	4.728949484	-1.201510364	0.954890965	-1.258269696	0.208294237	0.75905998
GPAT3	88.09356344	-0.349127211	0.277644512	-1.257461236	0.208586667	0.759403723
EID1	1606.821718	-0.190231816	0.151294113	-1.257364296	0.208621752	0.759403723
ITGB3	137.9859295	0.308322061	0.245231643	1.257268668	0.208656365	0.759403723
BECN1	466.54796	-0.155150246	0.123418301	-1.257108919	0.208714198	0.759403723
STMP1	646.1742846	-0.162465796	0.129239073	-1.257095029	0.208719227	0.759403723
NBPF10	208.7633113	0.387749888	0.308452623	1.257080859	0.208724358	0.759403723
SNAP23	980.0937578	-0.186340124	0.148276804	-1.256704482	0.208860664	0.759403723
HOXC-AS2	35.72068522	0.705035832	0.561027505	1.25668675	0.208867088	0.759403723
ATP6V1C1	1634.642885	-0.175531143	0.139689461	-1.256581147	0.208905345	0.759403723
TAS2R20	27.83238815	0.993067915	0.790362671	1.256471176	0.20894519	0.759403723
NMRK1	116.803818	0.301281564	0.239797721	1.25639878	0.208971424	0.759403723
PIR	48.60104668	0.403539776	0.321201628	1.256344119	0.208991233	0.759403723
TLCD1	130.822583	0.257235708	0.204754009	1.256315854	0.209001476	0.759403723
INIP	698.0590997	-0.165492375	0.131744677	-1.256159861	0.209058017	0.759403723
ARHGAP42	256.5385712	0.191366292	0.152355289	1.256052831	0.209096816	0.759403723
TTC30B	131.1128995	-0.235476293	0.187484869	-1.255974917	0.209125064	0.759403723
TMCC1-AS1	104.2131378	0.315097423	0.250941654	1.255660106	0.209239229	0.759403723
GAS2L3	614.2018716	0.313548657	0.249710507	1.255648637	0.209243389	0.759403723
MYH9	21743.93033	-0.208898051	0.166385195	-1.255508647	0.209294171	0.759403723
PIP4P2	205.1620732	-0.247828313	0.197400138	-1.255461704	0.209311202	0.759403723
PDE4B-AS1	8.15665423	1.588029651	1.264963799	1.255395334	0.209335282	0.759403723
BTN1A1	13.62170216	-0.677195943	0.539452699	-1.255338875	0.209355769	0.759403723
SUOX	187.3962025	0.196098622	0.156228886	1.255200797	0.209405876	0.759403723
TXNRD1	2771.85365	0.223421854	0.178000898	1.255172619	0.209416103	0.759403723
C4orf3	370.4279993	-0.199323204	0.158829387	-1.254951668	0.209496306	0.759403723
EXOC5	2533.937956	0.140164354	0.111697595	1.254855609	0.209531181	0.759403723
TSPAN2	10.95123208	-0.757500226	0.603665882	-1.254833592	0.209539175	0.759403723
LANCL1-AS1	8.792424056	1.007187041	0.802810161	1.254576847	0.209632413	0.759472612
AMT	107.253288	0.387771729	0.309093369	1.254545609	0.209643759	0.759472612
LOC339260	44.04366688	0.510903248	0.407279457	1.25442921	0.209686041	0.759472612
C21orf91-OT1	2.780013327	1.655077203	1.319641598	1.254186899	0.209774081	0.759637089
UBE2F	206.4513268	-0.244761319	0.195185369	-1.253994192	0.209844116	0.759736317
TCTN2	629.6771903	-0.159454761	0.127172786	-1.253843421	0.209898923	0.759780381
RHBDD2	298.4710567	-0.209635258	0.167273699	-1.253246983	0.210115836	0.760411089
ATP6V0B	660.145075	-0.178684406	0.142591819	-1.253118216	0.210162688	0.760419814
CEACAM1	4.317839843	-1.283984591	1.024723572	-1.253005812	0.210203592	0.760419814
RABGAP1	1112.926635	-0.13065035	0.104288033	-1.252783722	0.210284427	0.760557844
MIR4803	2.560045707	1.662078402	1.327004823	1.252503663	0.210386395	0.76077223
GUCA1B	17.29715754	0.746524436	0.59612265	1.25230007	0.210460543	0.760832487
TOR1B	904.761975	0.1798494	0.143639174	1.252091577	0.210536497	0.760832487
ZNF345	46.49208688	0.492696478	0.393551165	1.251924836	0.210597254	0.760832487
ZNF143	242.4279024	0.192395478	0.153696987	1.251784317	0.210648466	0.760832487
SLC35D3	10.55477718	0.914040087	0.730236179	1.25170474	0.210677472	0.760832487
IFI35	162.9138786	-0.318632218	0.254559852	-1.251698626	0.210679701	0.760832487
PHF11	15.95317306	0.667164383	0.533177983	1.251297698	0.210825885	0.760832487
CHMP4A	248.2939002	-0.255599546	0.204269342	-1.251286872	0.210829833	0.760832487
TRIM47	148.838455	-0.271779931	0.217209848	-1.251232082	0.210849816	0.760832487
CADPS2	361.7275403	0.323059192	0.258217098	1.251114641	0.210892655	0.760832487
C7orf61	2.68618212	1.652178816	1.320590709	1.251090747	0.210901371	0.760832487
LCOR	930.5823353	0.181595922	0.145154543	1.251052282	0.210915403	0.760832487
PPP2CA	3403.026549	-0.166693732	0.133274368	-1.250756118	0.211023469	0.760951986
GSS	564.2389298	-0.152137259	0.121649682	-1.250617808	0.21107395	0.760951986
ARMC12	3.1902144	-1.303604918	1.04246895	-1.250497598	0.211117832	0.760951986
RRAGA	587.6024291	-0.158066709	0.126417958	-1.250350119	0.211171678	0.760951986
DNAJB7	18.51419913	0.698997914	0.559055554	1.250319239	0.211182953	0.760951986
FOXN2	988.7620912	0.144763628	0.115786859	1.250259568	0.211204743	0.760951986
MNT	487.435587	-0.202609835	0.16208236	-1.250042477	0.211284031	0.761083774
DHDDS-AS1	27.40283138	0.710942752	0.568907688	1.249662761	0.211422766	0.761282519

NAA60	366.6883366	-0.148726913	0.119014129	-1.249657618	0.211424646	0.761282519
ABCA3	5.892952382	1.225154191	0.980554584	1.249450271	0.211500431	0.761288396
SUMF2	927.2854766	-0.132484495	0.106036849	-1.24941939	0.21151172	0.761288396
PAXBP1-AS1	40.66867979	0.567132956	0.454086281	1.248954173	0.211681836	0.761746833
C12orf66	274.6913505	0.26428487	0.211635943	1.248771195	0.211748772	0.761833863
MAGIX	14.2854788	0.646735235	0.517963636	1.248611274	0.211807287	0.761859128
MIR1180	3.115157915	1.433959377	1.148532004	1.24851495	0.211842537	0.761859128
FNBP4	905.1620717	0.161689955	0.129524499	1.248334921	0.211908431	0.761859128
RPS7	9613.174351	-0.21591311	0.172981803	-1.248183945	0.211963702	0.761859128
DNAAF3	3.308910881	-1.589100032	1.273146003	-1.248167946	0.21196956	0.761859128
EBAG9	583.4749893	-0.172752749	0.138432372	-1.247921616	0.212059764	0.761872146
COA7	962.1622596	-0.174761323	0.140076942	-1.247609497	0.212174099	0.761872146
ZFP90	549.3596944	-0.157478104	0.12623286	-1.247520687	0.21220664	0.761872146
CHMP2A	689.2692832	-0.223033378	0.17880045	-1.247387116	0.212255588	0.761872146
CBWD3	57.11814956	0.466155939	0.373753979	1.247226695	0.212314387	0.761872146
ARRDC1-AS1	205.2207501	0.230007763	0.184419252	1.247200387	0.212324031	0.761872146
IL17RB	43.30429843	-0.417484832	0.334744087	-1.247176122	0.212332926	0.761872146
ACOT11	16.8662518	-0.548387159	0.439704099	-1.247173182	0.212334004	0.761872146
CASZ1	6.122309687	-1.114448518	0.893626437	-1.247107821	0.212357966	0.761872146
TASOR2	3052.267773	0.138566301	0.111151209	1.246646822	0.212527025	0.761907641
RPL14	2720.32002	-0.218695576	0.175437641	-1.246571576	0.212554629	0.761907641
TRIM13	485.3184523	0.22956536	0.18416242	1.24653749	0.212567134	0.761907641
LOC100270746	30.00392231	0.501241553	0.402152177	1.246397711	0.212618421	0.761907641
GCLM	440.0299153	0.2539901	0.203786736	1.246352463	0.212635025	0.761907641
CEP170P1	6.325813747	0.990748247	0.79494397	1.246312047	0.212649857	0.761907641
LOC101928323	28.29920013	0.743321214	0.596461717	1.246217808	0.212684443	0.761907641
MIR485	2.083434841	2.002559554	1.607025794	1.246127823	0.212717472	0.761907641
EID3	19.39436778	0.957457724	0.768427629	1.245995965	0.212765877	0.761907641
HOXA2	20.75985808	0.647811042	0.520010239	1.245765937	0.212850338	0.761907641
MCM8	755.027998	0.219364049	0.17612559	1.245497879	0.212948794	0.761907641
HOXB2	111.4098285	0.30313327	0.243398345	1.245420423	0.21297725	0.761907641
GDI2	3166.52003	-0.221535805	0.177880429	-1.24541978	0.212977486	0.761907641
IVD	1434.946155	-0.198310031	0.159238283	-1.245366556	0.212997041	0.761907641
ZNF878	12.76815603	0.681509519	0.547281587	1.245263016	0.213035085	0.761907641
LOC390705	2.061816622	2.167065641	1.740400963	1.245153092	0.213075481	0.761907641
NSUN6	167.2843228	0.273284793	0.219529609	1.244865303	0.213181267	0.761907641
LPIN2	800.4199353	0.186085294	0.149488066	1.244817056	0.213199005	0.761907641
DNAL1	449.7168335	0.212131114	0.170412915	1.244806559	0.213202864	0.761907641
LINC00294	204.6175424	0.341457715	0.274317898	1.244751865	0.213222975	0.761907641
GCM1	4.780109169	1.276783415	1.025914762	1.244531673	0.21330395	0.761920961
CCDC150	127.3208921	0.548052233	0.4403762	1.244509201	0.213312215	0.761920961
MIR544A	4.474888105	1.264712532	1.016428887	1.244270552	0.213400005	0.761963351
ECHDC1	510.4201122	-0.162816356	0.130855599	-1.244244472	0.213409601	0.761963351
LOC729218	116.8510579	0.439099828	0.352990364	1.24394282	0.213520608	0.762203661
LOC101927151	138.5160809	0.217564901	0.174927527	1.243743079	0.213594136	0.762203661
LOC101927919	14.81014847	0.636918783	0.512154984	1.243605554	0.213644771	0.762203661
LINC02609	68.48080353	0.404079615	0.324928157	1.243596796	0.213647996	0.762203661
TMPRSS5	8.044410993	-0.880890699	0.708513262	-1.24329458	0.213759301	0.762396546
DZIP1L	157.2158127	0.492441517	0.396102407	1.243217682	0.213787628	0.762396546
METTL2A	362.7408697	0.171326496	0.137834254	1.242989252	0.213871794	0.762544093
NUBP2	392.3575532	-0.157458847	0.126694805	-1.242820076	0.213934143	0.762613808
SDCBP	3492.863006	-0.172223233	0.138610116	-1.242501186	0.214051703	0.76288027
CHCHD6	54.50575314	-0.389729218	0.313700431	-1.242361116	0.214103355	0.762911776
CHKA	269.8891522	0.197369263	0.158937646	1.24180311	0.214309215	0.763492645
SNORA71E	4.415549804	1.59343397	1.283388462	1.241583524	0.214390264	0.763628723
KIF2C	929.1324338	-0.155689906	0.125435169	-1.241198195	0.214532541	0.763842532
EPHX2	10.36805466	0.765789177	0.616987286	1.24117497	0.214541119	0.763842532
THNSL2	292.63028	-0.339869777	0.273851621	-1.241072726	0.214578884	0.763842532
EIF2S1	3054.332955	-0.141868218	0.114325463	-1.240915314	0.214637035	0.763859599
LOC100128108	33.88744846	0.460793537	0.37136293	1.240817272	0.214673259	0.763859599
RC3H1	1393.103103	-0.118026186	0.095131677	-1.240661259	0.214730911	0.763859599
ECT2	2347.399737	0.171320481	0.138095337	1.240595704	0.21475514	0.763859599
MAPK8IP3	1056.453552	0.240821221	0.194150972	1.240381231	0.21483442	0.763870753
TRAPPC1	789.7544861	-0.200177969	0.161387603	-1.240355298	0.214844007	0.763870753
NFATC1	249.0285931	-0.222245357	0.179287692	-1.239601859	0.215122693	0.764709033
LAMA4	1432.034259	-0.196563192	0.158613224	-1.239261063	0.215248833	0.765004828
MGST3	686.8086834	-0.228862314	0.184751812	-1.23875545	0.215436077	0.765245895
CSTF2	422.9567118	-0.205926419	0.166238115	-1.238743707	0.215440427	0.765245895
LOC285819	9.850023408	0.792133449	0.63953234	1.238613592	0.215488632	0.765245895
METTL4	285.5812812	0.205318186	0.16579053	1.23841926	0.215560643	0.765245895
FGF12	10.71538735	-0.823320359	0.664954435	-1.238160565	0.215656531	0.765245895
SNORD35A	14.06794405	0.705834988	0.570087082	1.238117843	0.215672369	0.765245895
SP100	460.4757643	-0.17541878	0.141693442	-1.238016224	0.215710045	0.765245895
ANXA7	1278.395668	-0.114176838	0.092229033	-1.237970664	0.215726938	0.765245895
LOC100422556	9.554491836	0.869109262	0.702136941	1.237805919	0.215788033	0.765245895
IKZF4	88.61589882	0.321050585	0.259372446	1.237797577	0.215791127	0.765245895
TSACC	5.770042841	1.026950874	0.829683283	1.237762524	0.215804128	0.765245895
LOC101927278	7.667661257	-0.885443252	0.715427579	-1.237642045	0.215848817	0.765245895
PURPL	2.303219401	1.563887168	1.263674015	1.237571676	0.215874922	0.765245895
CUEDC1	631.4241975	-0.148643375	0.120135764	-1.237294963	0.215977597	0.765457594
SSR1	6442.186216	0.140087542	0.113247054	1.237008263	0.216084015	0.765682471
TPRG1L	588.4899916	-0.28347872	0.229224789	-1.236684398	0.216204273	0.765956292
PAOX	20.08824874	0.579608738	0.46875872	1.23647564	0.216281815	0.766078702
CARD8	453.2624878	0.17497582	0.141527669	1.236336481	0.216333516	0.766109551
TAS2R42	2.224602092	1.896539805	1.534427876	1.235991495	0.216461725	0.766411274
API5	1550.731839	-0.132847928	0.107500902	-1.235784308	0.21653875	0.766462395
PANX2	33.80267312	0.491289748	0.397607481	1.235614951	0.216601725	0.766462395
CSNK1A1L	41.68552016	-2.019504695	1.63460825	-1.235467088	0.216656718	0.766462395
GOLGA8K	18.54910603	0.675720068	0.547049263	1.235208808	0.216752802	0.766462395
KIF11	1426.388425	-0.107102217	0.086721419	-1.235014584	0.216825077	0.766462395

SZT2	1532.148468	0.326398147	0.264301966	1.234944076	0.216851318	0.766462395
GHRL	23.28598557	0.774703526	0.627332476	1.234916979	0.216861404	0.766462395
C16orf87	235.3137699	0.189895697	0.153779347	1.23485826	0.21688326	0.766462395
TRIP12	5165.080386	-0.15920991	0.128932277	-1.234833617	0.216892433	0.766462395
OSBPL3	533.6883774	-0.192314846	0.155767153	-1.234630295	0.216968129	0.766462395
CD24	1289.221484	0.738383277	0.598062786	1.234625018	0.216970094	0.766462395
MIR133A1HG	19.43575145	0.957063902	0.775223343	1.234565381	0.2169923	0.766462395
DIAPH3-AS2	7.464963264	1.226727478	0.993949998	1.234194356	0.21713049	0.76679852
TAS2R14	8.905842898	0.935308125	0.757992692	1.233927629	0.217229873	0.766997491
EPAS1	79.96882628	0.321074867	0.260263872	1.233651312	0.217332864	0.767188167
SLFNL1-AS1	134.8804026	0.541117699	0.438666394	1.233551753	0.217369981	0.767188167
ODF3	7.520538514	1.282407606	1.040049144	1.233025972	0.217566074	0.767365396
IQCD	29.57903659	-0.553720028	0.449092892	-1.232974375	0.217585325	0.767365396
LINC00632	84.93115746	0.382411137	0.310169645	1.232909613	0.217609489	0.767365396
RAB6A	1482.997556	-0.209716188	0.170136764	-1.232632989	0.217712723	0.767365396
H2AX	543.9388031	-0.207576843	0.16841633	-1.232522062	0.217754131	0.767365396
UFC1	250.6154234	-0.271482835	0.220270254	-1.232498848	0.217762797	0.767365396
ADAM8	4.194502943	-1.199764348	0.973765753	-1.232087229	0.217916502	0.767365396
TRAPPC6A	86.34498997	-0.486042806	0.394519947	-1.231985379	0.217954546	0.767365396
THYN1	176.4813289	-0.276524201	0.224454475	-1.231983462	0.217955262	0.767365396
WEE1	1013.660191	-0.143747841	0.116684963	-1.231931151	0.217974804	0.767365396
TBC1D10C	10.46563145	0.867454725	0.704168221	1.231885648	0.217991803	0.767365396
NME1	634.5992373	-0.250474727	0.203358013	-1.231693423	0.218063628	0.767365396
PRKRA	668.5393654	0.173939073	0.141221478	1.231675766	0.218070226	0.767365396
BPNT1	354.588124	0.162275809	0.131762997	1.231573446	0.218108465	0.767365396
ANKRD23	94.09171548	0.469867774	0.381542621	1.231494852	0.218137841	0.767365396
CISD1	291.347913	0.16538356	0.134310584	1.23135166	0.218191368	0.767365396
WDPCP	189.1424702	0.22176521	0.180128975	1.231146796	0.218267966	0.767365396
TSNAX	502.0109016	-0.19181293	0.155812087	-1.231052955	0.218303059	0.767365396
MAPKAPK5	737.0796531	-0.194497039	0.157993552	-1.231044159	0.218306349	0.767365396
TBC1D8B	852.7012517	0.279827817	0.227357026	1.230785879	0.218402958	0.767365396
CLDN12	383.176922	0.205233545	0.166793385	1.230465736	0.21852275	0.767365396
CHI3L2	1.718132094	2.496647344	2.02910749	1.230416504	0.218541176	0.767365396
SNORD42B	22.57297293	0.545086982	0.443167149	1.22998057	0.218704381	0.767365396
MIR3136	6.303448575	1.223326675	0.994602541	1.229965363	0.218710076	0.767365396
NDUFA5	505.8573363	-0.191860815	0.156001492	-1.229865258	0.218747566	0.767365396
PTPN23	748.5407604	0.130122645	0.105814738	1.229721383	0.218801457	0.767365396
KAT14	281.3615019	-0.213554642	0.173665432	-1.229689988	0.218813217	0.767365396
ASAP1-IT2	32.42635575	0.693059342	0.563644249	1.229604212	0.218845352	0.767365396
BMS1P4	47.0684428	0.636725746	0.517836823	1.229587618	0.218851569	0.767365396
WNT5B	540.3821169	-0.248450002	0.202077175	-1.229480776	0.218891601	0.767365396
SLAIN2	1055.641735	0.188148795	0.153050115	1.229328025	0.218948844	0.767365396
NCBP3	1344.161396	0.181341782	0.14757923	1.228775771	0.219155888	0.767365396
JTB	1181.037825	0.193014758	0.157085585	1.228723555	0.219175471	0.767365396
FBXO44	24.07800571	-0.590875353	0.480891092	-1.228709291	0.219180821	0.767365396
YY2	24.5515533	-0.553483442	0.450463396	-1.228697931	0.219185082	0.767365396
SEPTIN11	2100.378829	-0.165188316	0.134443172	-1.228685053	0.219189913	0.767365396
MEF2C-AS1	17.60381348	0.600042483	0.48837639	1.228647606	0.219203958	0.767365396
RAD51	312.765898	-0.235731303	0.191863558	-1.228640316	0.219206693	0.767365396
NUDT19	31.74709358	-0.464179131	0.377802738	-1.228628286	0.219211205	0.767365396
CYB561D1	226.8539915	-0.222367512	0.181000197	-1.22854845	0.219241153	0.767365396
UROS	442.5981034	-0.18477803	0.150406078	-1.228527681	0.219248945	0.767365396
FAM156A	4.554985409	-1.348982417	1.098246868	-1.228305271	0.219332393	0.767365396
TENM3	4037.163234	0.125859666	0.102471255	1.228243623	0.219355527	0.767365396
EML4-AS1	7.272784015	0.952298803	0.775402478	1.228134847	0.219396352	0.767365396
LPIN3	245.4787768	0.326756543	0.266067495	1.228096437	0.219410768	0.767365396
NTRK2	62.62794163	-0.416657833	0.339276384	-1.228077911	0.219417722	0.767365396
MBNL2	937.897572	-0.214174129	0.174434557	-1.227819376	0.219514781	0.767365396
POLDIP2	2155.932924	-0.164686007	0.134135365	-1.227759782	0.219537157	0.767365396
GBA	854.0339117	-0.25650043	0.208926769	-1.227704956	0.219557745	0.767365396
AOAH	53.13332219	-0.468381528	0.381522715	-1.227663544	0.219573297	0.767365396
CCPG1	272.7462754	-0.371942256	0.303032178	-1.227401853	0.21967159	0.76755838
BCYRN1	2.605616381	1.866645433	1.521701825	1.226682786	0.21994184	0.768054578
PIGF	249.6338003	0.173291656	0.141275815	1.226619405	0.219965672	0.768054578
LOC101928166	6.217461946	1.155350555	0.941953084	1.226547877	0.21999257	0.768054578
LRRC45	399.7518338	0.26725522	0.217904963	1.226476056	0.22001958	0.768054578
WDR77	750.9709131	0.290944905	0.237242664	1.226359966	0.220063244	0.768054578
COPA	9082.6785	-0.199334297	0.162544582	-1.226336147	0.220072204	0.768054578
NEK6	782.8102872	0.286279291	0.23347792	1.226151453	0.220141686	0.768146633
SNORA22	4.920311555	1.125571397	0.918391405	1.225590082	0.220352972	0.768627047
NOL4	65.07965783	0.557806197	0.455145249	1.225556454	0.220365633	0.768627047
PRSS23	805.2969602	-0.386092362	0.315102016	-1.225293216	0.220464764	0.768736213
MSL2	982.1276062	-0.179594185	0.146592079	-1.225128853	0.220526675	0.768736213
PAIP1	778.1174044	-0.194846706	0.159058893	-1.224997251	0.220576256	0.768736213
CNTROB	635.9844563	0.170402272	0.139109087	1.224954284	0.220592446	0.768736213
PMS1	735.626122	0.167845766	0.137028057	1.224900729	0.220612626	0.768736213
MMD	345.8496163	0.221664477	0.18101431	1.224568801	0.220737729	0.768895314
MIR3171	3.940326131	1.491287836	1.217824567	1.224550626	0.220744581	0.768895314
TULP3	439.598736	-0.198769283	0.16235288	-1.224304018	0.220837562	0.769068859
MTA3	383.2953006	-0.164809638	0.134650473	-1.223981129	0.220959347	0.769342625
ICAM1	16.33805538	0.788480491	0.644282684	1.223811396	0.221023384	0.769415258
LINC02762	2.667445452	1.696274762	1.386355931	1.223549251	0.221122314	0.769530661
MAFA	7.132667057	-0.838237196	0.685117141	-1.223494707	0.221142902	0.769530661
SPRYD7	156.3066732	0.218142544	0.178323545	1.223296364	0.22121778	0.76964093
TTLL11	110.9330784	-0.237236516	0.193955611	-1.223148508	0.22127361	0.769684898
OR7E12P	2.104423551	1.999467471	1.635328899	1.22266993	0.221454389	0.770102309
PTPRD	2405.623965	0.180434571	0.147582422	1.222602043	0.221480041	0.770102309
ATP6V1B2	1943.293186	-0.125644876	0.10278124	-1.222449503	0.221537689	0.770152481
ZFP64	439.7814576	0.187193499	0.153146688	1.222315034	0.221588516	0.770178927
LRFN5	379.6648336	0.185478448	0.151771567	1.222089565	0.221673759	0.770320522

TSPAN9	401.506333	-0.310960835	0.254493585	-1.221880839	0.221752693	0.770320522
NAPA	569.4555988	-0.164516175	0.134644752	-1.221853598	0.221762996	0.770320522
SLC30A1	1017.301942	0.164840969	0.134923792	1.221733894	0.221808274	0.770320522
USP7	1802.780889	-0.187975092	0.153871633	-1.221635775	0.221845394	0.770320522
CBS	38.54041435	-0.948181101	0.776443021	-1.221185683	0.222015724	0.770761776
LOC101927391	2.795606368	1.600418903	1.310930003	1.220827122	0.222151482	0.770968721
LOC285638	16.22663803	0.814008392	0.666782915	1.220799714	0.222161862	0.770968721
SMIM7	375.9357738	-0.175806765	0.144032815	-1.220602161	0.222236688	0.771045097
EIF4E3	134.4403634	-0.335097218	0.274554369	-1.22051315	0.222270408	0.771045097
TCIM	3.950372311	-1.324021251	1.085121512	-1.220159435	0.222404441	0.771359895
ZFAND5	2389.435225	0.152015751	0.124598757	1.220042277	0.222448849	0.771363783
NEIL2	410.79297	0.177523017	0.145535714	1.219790059	0.222544471	0.771378824
NACA	2292.080001	-0.249229857	0.204425403	-1.219172629	0.222778678	0.771378824
FAAP24	90.1895996	-0.306217417	0.251195362	-1.219040886	0.222828675	0.771378824
SNRNP27	363.2581207	0.174297377	0.142979833	1.219034698	0.222831023	0.771378824
HOXA1	70.63119268	0.322775546	0.264810653	1.218891849	0.222885244	0.771378824
LOC100506551	71.89941321	0.599728287	0.492031948	1.218880785	0.222889444	0.771378824
TAF1L	3.572196413	-1.431128689	1.174292271	-1.218715924	0.222952032	0.771378824
LINC01234	34.07341477	-0.414840053	0.340393066	-1.218708884	0.222954705	0.771378824
ATP8A1	45.13478773	0.462592456	0.379576732	1.218706041	0.222955784	0.771378824
CSGALNACT1	40.71033637	0.50665211	0.415782085	1.218552045	0.22301426	0.771378824
ZNF280C	230.7141845	0.209465042	0.171896907	1.218550383	0.223014891	0.771378824
SPATA2	162.6421402	0.194216158	0.159387121	1.218518516	0.223026993	0.771378824
NCAPG	983.4980617	-0.193694308	0.158973834	-1.2184037	0.2230706	0.771378824
TDRD15	3.087042161	-1.274638947	1.04628723	-1.21824955	0.223129155	0.771378824
PPY2P	1.783982444	1.944237113	1.595991206	1.218200392	0.223147831	0.771378824
C6orf52	3.849238402	-1.298675003	1.066066193	-1.21819359	0.223150415	0.771378824
CETN2	170.7756665	-0.213493316	0.175268651	-1.218091853	0.223189069	0.771378824
DMTN	6.345234205	-1.031987354	0.847477188	-1.21771697	0.223331546	0.771721572
PRPF8	9431.095208	-0.112246178	0.092216694	-1.217200194	0.223528056	0.77210651
GUSBP14	53.26296781	0.507875583	0.417317469	1.217000534	0.223604013	0.77210651
SUSD6	429.9731759	0.18168342	0.149307445	1.216840999	0.223664717	0.77210651
GPX4	577.945447	-0.175093325	0.14389417	-1.216820144	0.223672654	0.77210651
MFSD14B	1635.041443	-0.144611732	0.118847818	-1.216780709	0.223687662	0.77210651
NEK10	24.11954333	0.668352627	0.549301851	1.216731066	0.223706555	0.77210651
TMCC1	375.9090885	0.229677926	0.188782563	1.2166268	0.223746241	0.77210651
STX2	1037.879471	0.153904498	0.126531617	1.216332345	0.223858345	0.77227363
LOC101928557	3.696947467	1.526771315	1.255287785	1.216271945	0.223881345	0.77227363
DNAJB9	267.4393624	0.269365649	0.221591681	1.215594594	0.224139394	0.772870091
ZNF137P	33.13423781	0.453579065	0.373134793	1.21559038	0.224141001	0.772870091
SLC7A6	799.6219787	0.220690739	0.181644575	1.214959157	0.224381669	0.77343651
IGSF10	1323.19452	0.363105302	0.298926785	1.214696444	0.224481889	0.77343651
SETBP1	352.2424457	-0.255398267	0.210306845	-1.214407773	0.224592048	0.77343651
TRA2A	646.3461137	-0.377300519	0.310778883	-1.21404812	0.224729349	0.77343651
PPP5C	849.0563405	-0.137291679	0.113090706	-1.213996129	0.224749202	0.77343651
NDUFAF7	424.670833	0.24390887	0.200917686	1.213974112	0.22475761	0.77343651
LOC100507002	7.810776105	-1.053692785	0.868054941	-1.213854948	0.224803119	0.77343651
ZBTB14	233.3440759	-0.18144983	0.149492133	-1.21377511	0.224833613	0.77343651
AHRR	421.125166	0.840018864	0.692077009	1.213765018	0.224837468	0.77343651
QRICH2	10.64179208	0.714622225	0.588845997	1.213597831	0.224901336	0.77343651
MORC3	592.9145014	0.163031426	0.134339988	1.213573327	0.224910698	0.77343651
MT1M	1.914046891	2.508741387	2.06735227	1.213504551	0.224936976	0.77343651
ALG9	463.41121	-0.149552522	0.123247669	-1.213430832	0.224965144	0.77343651
NAA16	424.3795966	0.272457568	0.224540873	1.213398541	0.224977484	0.77343651
CEBPA	60.16220526	-1.177874578	0.970797964	-1.213305571	0.225013014	0.77343651
LINC00308	11.59699032	-0.97992488	0.807651796	-1.213301183	0.225014691	0.77343651
IGF2BP2-AS1	43.11270583	0.968515144	0.798400362	1.21306952	0.225103242	0.77343651
MME	12177.15848	-0.690368857	0.569119521	-1.213047229	0.225111764	0.77343651
TMEM138	201.388403	0.270250078	0.222794836	1.21299974	0.22512992	0.77343651
PHLDB2	338.7856076	-0.1934499	0.159542271	-1.21253069	0.225309302	0.773542902
KANK1	448.9023713	-0.208615358	0.172075518	-1.212347695	0.225379314	0.773542902
AURKAIP1	542.6233031	-0.207212644	0.170921975	-1.212323013	0.225388758	0.773542902
CCDC15	82.2170077	0.27547317	0.227231951	1.212299453	0.225397773	0.773542902
HS3ST5	3.088116884	-1.480518327	1.221306216	-1.212241703	0.225419872	0.773542902
SPRN	26.20846515	-0.552202695	0.455543105	-1.212185386	0.225441424	0.773542902
RPL23AP87	9.922812415	0.80598273	0.665033508	1.211943038	0.225534186	0.773542902
COX19	403.8983544	-0.185788802	0.153301898	-1.21191456	0.225545088	0.773542902
CENPN	448.8809941	-0.150934298	0.124543768	-1.211897637	0.225551567	0.773542902
NFYC	263.6296345	-0.174798828	0.144318975	-1.211197821	0.225819598	0.773605294
IWS1	501.7355522	0.152420244	0.125843569	1.211188189	0.225823288	0.773605294
TMPRSS11F	32.28797408	-0.434674055	0.358914392	-1.211080036	0.225864732	0.773605294
TLR2	85.88668945	-0.278604951	0.230056795	-1.211026831	0.225885122	0.773605294
GUK1	930.795625	-0.108234935	0.089393709	-1.2107668	0.225984793	0.773605294
HCG15	67.98274431	0.356059526	0.294079947	1.210757583	0.225988326	0.773605294
CTTNBP2	98.84863641	-0.3531165	0.291680329	-1.210628435	0.226037841	0.773605294
GYPE	72.08262199	-0.377398719	0.311746205	-1.210596033	0.226050265	0.773605294
MRPL27	351.7437262	0.152550979	0.126039261	1.210344911	0.226146571	0.773605294
CRPPA	74.53034088	-0.425580322	0.351709969	-1.210032015	0.226266608	0.773605294
TRAF3IP2-AS1	125.4815758	0.251225968	0.207627665	1.209983114	0.226285372	0.773605294
PCYOX1L	333.8639228	-0.325303229	0.268861132	-1.209930296	0.226305641	0.773605294
RNF208	2.251102672	2.16909838	1.793147316	1.209659887	0.226409428	0.773605294
TMEM263	3142.171933	0.192035463	0.158762813	1.209574576	0.226442179	0.773605294
WDR97	52.8812567	-0.516186442	0.426790964	-1.209459633	0.22648631	0.773605294
PEX11B	283.4171142	-0.20283068	0.167706113	-1.209441185	0.226493394	0.773605294
GANAB	7537.121001	-0.15084764	0.124726213	-1.209430127	0.22649764	0.773605294
WDR13	404.1879155	-0.189147863	0.156400135	-1.209384264	0.226515251	0.773605294
BAG6	4519.286642	-0.155806048	0.128835978	-1.209336473	0.226533604	0.773605294
TAF6	560.8811087	-0.161446988	0.133509495	-1.20925473	0.226564997	0.773605294
ADPRM	72.59683522	-0.290076073	0.239913934	-1.209083891	0.226630617	0.773605294
ADA	256.1319497	0.338704692	0.280189096	1.20884323	0.22672308	0.773605294

NUP43	1363.118503	-0.12602746	0.104269282	-1.208672941	0.226788521	0.773605294
ZNF107	175.8174028	0.329335611	0.27250396	1.208553484	0.226834436	0.773605294
MRPS5	838.5690092	0.119211381	0.098639728	1.208553419	0.226834461	0.773605294
ATP7A	537.3532538	0.190499252	0.157626403	1.208549131	0.22683611	0.773605294
ERVK13-1	384.1509421	0.392359671	0.324671166	1.208483266	0.226861429	0.773605294
BORCS6	145.4788433	-0.260240305	0.215352674	-1.208437768	0.22687892	0.773605294
STAC3	40.68627348	-0.538534752	0.445722108	-1.208229841	0.226958866	0.773605294
GPRC5D-AS1	12.8583221	0.722996746	0.598401489	1.208213481	0.226965158	0.773605294
NDUFA12	385.1551924	-0.211104659	0.174727214	-1.208195644	0.226972017	0.773605294
TNFRSF10D	2855.002558	0.241689202	0.200054239	1.208118372	0.227001734	0.773605294
DPF1	73.30807465	-0.372083622	0.30798648	-1.20811674	0.227002362	0.773605294
SP1	2343.308634	-0.160067166	0.132524259	-1.20783295	0.227111525	0.773829325
RPL38	596.4716717	-0.184012557	0.152373408	-1.207642193	0.227184922	0.773931431
CLDN15	102.6633235	0.307359459	0.254623141	1.207115182	0.227387789	0.774210895
TATDN3	230.8147752	-0.185837184	0.15398286	-1.206869286	0.227482488	0.774210895
KCNG2	8.763066567	-0.80296797	0.665403422	-1.206738565	0.227532842	0.774210895
TMEM225B	9.705715661	0.833198536	0.690479429	1.206695669	0.227549368	0.774210895
SPAG5-AS1	46.8834012	0.411695684	0.34120276	1.206601271	0.227585737	0.774210895
OSGEPL1	347.6825546	0.146001283	0.121008342	1.206538993	0.227609734	0.774210895
RASD1	43.54404911	-0.684259911	0.567141653	-1.206506184	0.227622376	0.774210895
YEATS4	216.8386082	-0.188276206	0.156055988	-1.206465756	0.227637955	0.774210895
NDUFAF2	46.5750598	-0.391419009	0.324448382	-1.206413811	0.227657974	0.774210895
SHTN1	294.1379913	0.369570446	0.306386825	1.206221729	0.227732008	0.774287198
RNLS	57.52895995	-0.336493797	0.279033916	-1.205924362	0.227846656	0.774287198
LOC100506071	83.95773419	0.565629359	0.469052996	1.205896485	0.227857406	0.774287198
PAQR9	22.17933104	0.599010854	0.496758141	1.205840034	0.227879176	0.774287198
RUVBL2	1684.743748	-0.159167615	0.132021326	-1.205620477	0.22796386	0.774287198
MRFAP1L1	235.2656822	-0.21136061	0.17532006	-1.205570033	0.22798332	0.774287198
RPS21	2679.059401	-0.26329119	0.218418715	-1.205442446	0.228032544	0.774287198
LOC389765	10.08557683	-0.782068104	0.648812636	-1.20538359	0.228055254	0.774287198
ITGA2B	68.38422241	0.315109578	0.261427618	1.205341578	0.228071465	0.774287198
ZNF761	34.95680553	0.530039167	0.439812975	1.205146727	0.228146664	0.774394961
FAU	797.6106631	-0.201699085	0.167495784	-1.204203949	0.22851076	0.775220948
CTSD	1754.317906	-0.235590677	0.195643426	-1.204183967	0.228518481	0.775220948
LOC100422781	20.0698459	0.604121728	0.501687773	1.204178696	0.228520518	0.775220948
KMT2E-AS1	129.026132	0.458151647	0.380528561	1.203987544	0.228594393	0.775323959
DYNLL2	714.4489233	-0.168158789	0.139693952	-1.203765707	0.228680148	0.775467218
HOXA4	55.16846664	-0.414606515	0.344480704	-1.203569632	0.228755963	0.775576723
LOC100652768	36.84771836	0.434074044	0.360744827	1.203271708	0.228871194	0.775694186
REREP3	2.329461027	-1.488013348	1.236656719	-1.203254974	0.228877667	0.775694186
NMNAT2	3.457200072	-1.403560572	1.166815462	-1.202898503	0.2290156	0.775795289
BHLHE40	608.3533127	0.737297755	0.613004383	1.202760985	0.229068827	0.775795289
RAB3IP	55.56805393	0.347286027	0.288758355	1.202687371	0.229097323	0.775795289
PPP1R26	67.98505155	-0.314036314	0.261153549	-1.202496825	0.229171096	0.775795289
NUP54	634.7730832	-0.140680624	0.117011274	-1.202282647	0.229254038	0.775795289
HOXB5	29.73241548	0.623981227	0.51909844	1.202047973	0.229344942	0.775795289
MTMR11	123.2633771	0.273291307	0.227371058	1.201961714	0.229378362	0.775795289
NDUFA4L2	51.45948639	-0.434243155	0.361283801	-1.201944714	0.229384949	0.775795289
IL7	18.19105411	0.613237115	0.5103245	1.201661128	0.229494848	0.775795289
PANK1	219.9024917	0.224657701	0.18695621	1.201659475	0.229495488	0.775795289
YPEL2	159.7481415	-0.298134372	0.24812496	-1.201549297	0.229538196	0.775795289
PRDX5	432.5307096	-0.227349442	0.189241458	-1.201372279	0.229606824	0.775795289
CALML4	129.4986467	0.401885417	0.33452466	1.201362605	0.229610575	0.775795289
KCNE2	2.110417578	-2.182334925	1.816776099	-1.201212921	0.229668618	0.775795289
ZC3HC1	388.2604382	0.201306479	0.167590647	1.20117968	0.22968151	0.775795289
SNORD114-15	3.103693425	1.7455508	1.453450603	1.200970157	0.229762778	0.775795289
LOC646652	29.1943531	0.857431069	0.713977075	1.200922409	0.229781301	0.775795289
MIR4730	4.984058837	1.126841415	0.938355511	1.20086833	0.229802281	0.775795289
HMGA1	1782.693447	-0.253047363	0.210732798	-1.200797245	0.229829861	0.775795289
RAB2B	601.0948617	0.205226815	0.170910297	1.200786718	0.229833945	0.775795289
USP12	815.9074377	0.158997457	0.132423403	1.200674901	0.229877334	0.775795289
MAD2L1	1072.685759	0.136265016	0.113494793	1.200627911	0.229895569	0.775795289
SLC16A1-AS1	105.8090149	0.358134407	0.298370587	1.200300639	0.230022602	0.775795289
MCUB	46.69742395	-0.33320536	0.277620671	-1.200218123	0.230054639	0.775795289
PLXNB3	95.93124809	0.383236733	0.319321774	1.200158476	0.230077799	0.775795289
ARL8B	942.981558	-0.189373915	0.157819286	-1.199941524	0.230162052	0.775795289
POLR2M	125.5260056	-0.242524012	0.202117035	-1.199918709	0.230170913	0.775795289
DBR1	265.5955146	-0.180307793	0.150278913	-1.199820987	0.230208872	0.775795289
MIPEPP3	26.65371314	-0.483883153	0.403314409	-1.19976659	0.230230003	0.775795289
CALM2	4899.454258	-0.188758231	0.157337206	-1.199704995	0.230253932	0.775795289
CDHR2	13.66971703	0.747054112	0.62270239	1.199696877	0.230257087	0.775795289
MRPS18B	1911.327252	-0.203618496	0.169743247	-1.199567578	0.230307325	0.775817869
RDH10	362.3921004	0.176386566	0.147103961	1.199060617	0.230504376	0.776031063
CDK18	50.54790483	0.388380436	0.323943536	1.198913986	0.230561393	0.776031063
YTHDC1	745.8209111	-0.156617751	0.130641382	-1.198837215	0.230591249	0.776031063
CYREN	561.0761752	-0.138821287	0.115808336	-1.198715835	0.230638459	0.776031063
ACD	157.2833667	0.217273642	0.181262318	1.198669668	0.230656417	0.776031063
ZNF581	203.3456004	-0.246604865	0.205738566	-1.19863218	0.230671	0.776031063
LIN52	309.6647612	0.176310168	0.147143149	1.198222064	0.230830578	0.776031063
ATP11A	915.1518003	0.119096924	0.099395461	1.198212909	0.230834141	0.776031063
CCT8	2627.584688	-0.137005853	0.114346087	-1.198168266	0.230851517	0.776031063
GPR158	118.4338871	0.240677403	0.200892191	1.198042596	0.230900434	0.776031063
FAM229A	24.11671266	0.611165913	0.510137294	1.198042018	0.23090066	0.776031063
SNORA52	11.27733355	0.794338667	0.663073344	1.197965013	0.230930638	0.776031063
CFL1	2560.329387	-0.162124337	0.135334886	-1.197949336	0.230936741	0.776031063
ORC5	437.5551928	-0.206758506	0.172621637	-1.197755448	0.231012236	0.776079633
UBE2G1	1287.659335	-0.167589709	0.139928063	-1.197684765	0.231039763	0.776079633
LOC100128317	163.2732301	-0.548563119	0.458115149	-1.197435011	0.231137044	0.776079633
SDR42E1	4.123397047	1.254392161	1.047717832	1.197261441	0.231204669	0.776079633
FLYWCH2	215.758478	-0.247843623	0.207009451	-1.197257527	0.231206194	0.776079633

NAA10	485.0947638	-0.190586806	0.159188294	-1.19724134	0.231212501	0.776079633
TTI2	355.5635846	0.145739161	0.121761059	1.196927507	0.231334812	0.776343945
SGK3	4.729591967	-1.219954331	1.01948941	-1.196632667	0.231449762	0.776583461
LOC101928000	47.48007302	0.35984614	0.30086857	1.196024366	0.231687051	0.776993288
IRX6	56.98340368	0.425884066	0.356090395	1.19599987	0.23169661	0.776993288
MTAP	1978.438376	-0.155510556	0.130027262	-1.195984232	0.231702712	0.776993288
SHOC1	32.76678326	0.759818358	0.635618502	1.195400001	0.231930785	0.777270351
OXTR	6.530197799	0.997303618	0.834533949	1.195042597	0.232070388	0.777270351
LINC01914	2.928213314	2.118212117	1.772527172	1.195023778	0.23207774	0.777270351
CD207	2.011109293	-1.590160228	1.330764657	-1.194922197	0.23211743	0.777270351
LOC102724034	2.588527371	1.628605599	1.362942455	1.194918827	0.232118746	0.777270351
RB1-DT	6.764505535	0.902322312	0.755205803	1.194803203	0.232163929	0.777270351
PMF1-BGLAP	4.152013646	-1.162214668	0.972779764	-1.194735654	0.232190327	0.777270351
LOC100507516	6.309813584	1.025138632	0.858298174	1.194385194	0.232327326	0.777270351
VAMP2	185.2707534	-0.219963392	0.184189451	-1.194223618	0.232390507	0.777270351
MIR493	2.582729767	1.426981035	1.194954438	1.19417192	0.232410725	0.777270351
LINC00537	342.8810171	0.303853067	0.254458786	1.194115055	0.232432965	0.777270351
ENG	1236.229186	0.20948874	0.175436524	1.194099925	0.232438883	0.777270351
LOC105371038	9.585770541	0.731067691	0.612322631	1.193925644	0.232507055	0.777270351
HS6ST1	465.1870042	-0.250781126	0.210050813	-1.193906951	0.232514368	0.777270351
PRKCZ	6.0611888	-1.099112272	0.920680291	-1.193804498	0.232554452	0.777270351
LINC01347	27.5965155	-0.491061087	0.411345432	-1.19379249	0.23255915	0.777270351
FAR2	219.8257454	-0.224772453	0.188286827	-1.193776837	0.232565275	0.777270351
SLC9A6	520.9464511	-0.14758679	0.123631505	-1.193763597	0.232570455	0.777270351
TMEM145	13.25124019	-0.686843312	0.575492354	-1.193488162	0.232678246	0.777484782
WDR76	682.4623982	-0.129365602	0.108417054	-1.193221892	0.232782483	0.777623146
LSM14B	747.0471562	-0.151377772	0.126871367	-1.193159457	0.232806929	0.777623146
MRPL16	301.295851	-0.158983446	0.133268461	-1.192956271	0.232886499	0.777743145
METTL18	105.8099546	-0.258615925	0.21683598	-1.192679949	0.232994741	0.777878217
PRKCH	82.804958	-0.293882348	0.246415325	-1.192630158	0.233014249	0.777878217
LINC02256	58.46554629	0.337579874	0.283101953	1.192432165	0.233091835	0.777991477
CXorf38	643.2505528	0.199717071	0.16751868	1.192207766	0.233179789	0.778054573
ARHGAP31	294.6996793	0.290517828	0.243690064	1.192161152	0.233198063	0.778054573
SCAANT1	5.05838441	1.239790606	1.040102583	1.191988777	0.233265646	0.778134371
CSTF3-DT	12.94761084	0.675385605	0.566844533	1.191482965	0.23346404	0.77817893
PCF11-AS1	4.185243555	-1.165564183	0.978275314	-1.191448017	0.233477752	0.77817893
MFSD1	949.4647652	-0.166321984	0.139597616	-1.191438574	0.233481457	0.77817893
TIMM10B	410.6247086	0.236820981	0.198788092	1.191323778	0.233526503	0.77817893
MIR3936HG	4.933283748	1.323861215	1.111317562	1.191253751	0.233553984	0.77817893
XK	3.940025399	1.430247455	1.200635728	1.191241791	0.233558678	0.77817893
LOC100129434	16.36586131	-0.870552613	0.730834872	-1.191175525	0.233584686	0.77817893
PCNP	1694.81762	-0.138419739	0.116230457	-1.190907638	0.233689846	0.778383748
CORO7	46.42381445	-0.430416009	0.361452873	-1.190794265	0.233734361	0.778386528
MYCBP2-AS1	3.567233613	-1.2238162	1.028144627	-1.190315222	0.23392252	0.778615231
TSPEAR-AS2	3.148468622	1.648124874	1.384727525	1.190216014	0.233961501	0.778615231
NLGN1	453.153256	0.238691404	0.200560057	1.190124334	0.233997527	0.778615231
LZTS3	42.910102	0.380663219	0.319901447	1.189939037	0.234070354	0.778615231
LINC01776	11.22318594	-0.655072167	0.550551359	-1.189847516	0.23410633	0.778615231
GUSB	526.2125387	-0.203204632	0.170783285	-1.189839115	0.234109632	0.778615231
DUSP3	593.0649537	-0.174929575	0.147045245	-1.189630951	0.234191475	0.778615231
PODXL	37.55939022	0.545537085	0.458578935	1.189625259	0.234193713	0.778615231
INSYN2A	35.5282116	0.857111064	0.720492233	1.189618742	0.234196276	0.778615231
TBC1D8-AS1	15.72403452	-0.655735248	0.551308967	-1.189415168	0.234276335	0.778736111
PI4KAP2	129.1086541	0.316710099	0.266318868	1.189213898	0.234355507	0.778853997
GNPTG	307.9312719	-0.234666239	0.197351325	-1.189078605	0.234408737	0.77888564
LOC102724467	2.42489868	-1.632395577	1.373508038	-1.188486366	0.234641848	0.779450736
ATG4D	133.7593006	0.26851219	0.225967639	1.188277189	0.234724222	0.779450736
SERPING1	2.88320033	-1.278633166	1.076061151	-1.188253256	0.234733648	0.779450736
KHDC1	114.4835455	0.270333973	0.227528825	1.188130662	0.234781936	0.779450736
CPM	52.83200036	0.565441495	0.475924344	1.188091137	0.234797506	0.779450736
DDX12P	438.9846696	0.454961005	0.383104692	1.187563126	0.235005572	0.779856472
ZNF446	168.067136	-0.242173484	0.203925457	-1.187558862	0.235007253	0.779856472
SLC9A1	375.8107784	0.192089747	0.161816686	1.187082445	0.235195102	0.780334522
FAM86B2	85.77013039	0.390324677	0.328987289	1.186443033	0.235447386	0.780924598
SLC35F1	7.404687531	0.890640847	0.75073136	1.18636425	0.235478484	0.780924598
LINC02511	33.14688518	-0.413119922	0.348242796	-1.186298544	0.235504422	0.780924598
THUMPD1	957.3252285	-0.112182413	0.09457947	-1.18611801	0.235575699	0.78101562
TTN-AS1	484.0427327	0.278716212	0.235015522	1.185948101	0.235642796	0.781037506
GNL1	992.1534731	-0.163667837	0.138020279	-1.18582456	0.23569159	0.781037506
PHKG2	321.9148156	0.230023512	0.193997756	1.18570192	0.235740036	0.781037506
TMEM216	115.8296085	0.234970789	0.19817764	1.185657417	0.235757617	0.781037506
SNORD116-26	67.80684021	0.402419941	0.339549207	1.185159422	0.23595442	0.781544196
MTX2	528.2053731	-0.146163911	0.123396021	-1.184510729	0.236210952	0.782169146
SCNM1	31.31564575	-0.488360372	0.412330291	-1.18439121	0.236258238	0.782169146
SALRNA1	6.366183554	0.947175604	0.799812906	1.184246462	0.236315515	0.782169146
ZNF785	136.6872202	0.305437237	0.257918687	1.184238492	0.236318669	0.782169146
RUNX3	375.8743581	0.331725058	0.280144302	1.184122098	0.236364734	0.782176333
RILPL2	129.743131	-0.27384252	0.23141548	-1.183337087	0.23667558	0.782872265
PRPS2	929.3328476	-0.154441494	0.130521243	-1.183267109	0.236703304	0.782872265
EFCAB13	29.25894869	0.501540887	0.423864282	1.183258201	0.236706833	0.782872265
SV2A	695.8814063	-0.149949334	0.126751559	-1.183017673	0.236802143	0.783042159
C1orf52	319.4470158	0.190063624	0.160696927	1.182745855	0.236909885	0.78325309
SCRN3	188.0664417	-0.222606949	0.188285255	-1.182285618	0.23709239	0.783711072
RNASEH2A	817.5153961	-0.158994712	0.134499697	-1.182119482	0.237158295	0.783733284
LINC02603	168.1781874	0.59505943	0.503474902	1.181904852	0.237243456	0.783733284
FANCL	480.9092738	0.21929616	0.185567704	1.18175822	0.23730165	0.783733284
TANK	156.0704544	-0.204180027	0.172780574	-1.181730223	0.237312762	0.783733284
SPTB	2.07832395	1.600639363	1.354555378	1.181671409	0.237336107	0.783733284
NEDD1	1100.82882	0.151074082	0.127863233	1.181528724	0.23739275	0.783733284
CYP4V2	37.29936513	-0.592232931	0.501480768	-1.180968383	0.237615284	0.783733284

HNRNPH3	1025.058335	0.218867854	0.185337142	1.180917392	0.237635541	0.783733284
SNORA12	12.41629534	-0.948190505	0.803024917	-1.180773454	0.237692732	0.783733284
GPT	204.2271038	0.445037559	0.376918363	1.180726657	0.237711328	0.783733284
LOC101929532	12.97565077	1.263766837	1.070391039	1.180659022	0.237738207	0.783733284
EPM2A	114.3728414	-0.239501458	0.202888282	-1.180459787	0.237817395	0.783733284
LINC01894	3.355906909	-1.422375933	1.205171499	-1.180226991	0.237909947	0.783733284
MEMO1	685.6810036	-0.226105836	0.191586405	-1.180176828	0.237929893	0.783733284
ZNF518B	89.27067742	-0.461411674	0.390988472	-1.180115801	0.237954161	0.783733284
RPTOR	1193.695336	0.178886387	0.151628587	1.179766894	0.23809294	0.783733284
CCDC102B	21.5528924	-0.54782717	0.464375622	-1.179706997	0.23811677	0.783733284
CD2AP	1290.117283	0.131513179	0.111479727	1.179704888	0.238117609	0.783733284
AMN	16.92612032	0.586130253	0.49690387	1.179564676	0.238173399	0.783733284
AFDN	308.3511432	0.310425932	0.263188564	1.179481082	0.238206666	0.783733284
CNTN5	5.823519296	1.020015493	0.864802152	1.17947844	0.238207717	0.783733284
RPL34	2314.256704	-0.182247665	0.154517589	-1.17946226	0.238214156	0.783733284
TMEM167A	1145.105807	0.120744245	0.102376142	1.179417815	0.238231845	0.783733284
ATOH8	1781.567969	0.751358733	0.637081418	1.179376312	0.238248363	0.783733284
PARP1	2867.657574	-0.111678824	0.094723898	-1.178993122	0.238400915	0.783733284
CYP7B1	385.1124059	-0.244126258	0.207084966	-1.178870019	0.238449938	0.783733284
LOC102724064	30.49484913	0.56571973	0.479890522	1.178851642	0.238457256	0.783733284
CCNO	66.48759771	-0.308029251	0.261314409	-1.178768718	0.238490284	0.783733284
LUC7L2	309.6181288	-0.250813706	0.212800512	-1.178633004	0.238544344	0.783733284
ZNF629	748.1557841	-0.154475558	0.131068211	-1.17858904	0.238561858	0.783733284
PPP3R1	2133.688463	-0.14962204	0.126952207	-1.178569823	0.238569514	0.783733284
UBA1	8058.144038	-0.135873785	0.115310783	-1.178326792	0.238666351	0.783733284
CXCL1	7.645041226	0.931583014	0.790731088	1.178128731	0.23874529	0.783733284
SLC35A4	489.5604706	-0.165135618	0.140198261	-1.177872085	0.238847605	0.783733284
TIPARP	522.1434306	0.294713323	0.250281342	1.177528139	0.238984773	0.783733284
CDK5	97.66244953	-0.274796691	0.233382323	-1.177452892	0.239014789	0.783733284
ADORA2A-AS1	7.435207559	0.965070699	0.819641737	1.177429913	0.239023956	0.783733284
SLC2A4	25.04734719	0.604635845	0.51352345	1.177425967	0.23902553	0.783733284
CASKIN1	10.4642231	0.709411797	0.602542069	1.177364757	0.23904995	0.783733284
ZDHHC2	737.6086351	-0.160347535	0.136207286	-1.177231706	0.239103037	0.783733284
ZBTB43	463.7068941	0.154538724	0.131285791	1.177116911	0.239148846	0.783733284
DYRK4	78.63098195	-0.268894203	0.228435195	-1.177113724	0.239150118	0.783733284
ACSL5	8.965206757	0.831308674	0.706318648	1.176959827	0.239211541	0.783733284
DHX29	389.8773451	-0.153657392	0.130578286	-1.176745361	0.239297157	0.783733284
HEATR5B	961.8787288	0.129402516	0.10997122	1.17669437	0.239317516	0.783733284
NOL3	132.8352566	0.289486411	0.246080105	1.176390961	0.239438682	0.783733284
SLC44A3	66.52720868	0.427863308	0.363735144	1.176304557	0.239473195	0.783733284
PAPSS2	602.6116467	0.192820916	0.16392163	1.176299406	0.239475253	0.783733284
CEP131	269.9788197	-0.297869927	0.253235905	-1.176254716	0.239493106	0.783733284
FZD8	454.3478826	-0.277266482	0.235721578	-1.176245654	0.239496726	0.783733284
RFLNA	36.83638384	0.589537972	0.501249794	1.176136088	0.239540499	0.783733284
POLE4	160.4736348	-0.273193325	0.232289735	-1.176088668	0.239559446	0.783733284
LOC105369632	10.87781725	0.721206139	0.613227801	1.176081936	0.239562136	0.783733284
PRH1-TAS2R14	14.65666303	0.958628351	0.815187039	1.175961227	0.23961037	0.783733284
CRTAM	6.401182887	-1.588294868	1.350637182	-1.17595968	0.239610988	0.783733284
KANTR	127.1355436	0.508644165	0.432544638	1.175934507	0.239621048	0.783733284
CPB2	11.22553337	0.907410985	0.771663365	1.175915594	0.239628607	0.783733284
CCDC85B	392.0183381	0.216913747	0.184493752	1.175724085	0.239705151	0.783733284
PLEKHA4	101.8823394	-0.324243032	0.275816995	-1.175573073	0.239765521	0.783733284
MYO5B	38.60613905	0.599457702	0.509935184	1.175556659	0.239772084	0.783733284
GLIPR2	106.3761768	-0.254003539	0.216095577	-1.175422202	0.239825845	0.783733284
PCBD2	43.42399103	-0.533355448	0.453792016	-1.175330173	0.239862648	0.783733284
ARL6IP5	1263.130256	-0.189902871	0.16157655	-1.175312079	0.239869884	0.783733284
DHRS4L1	11.52433016	0.703829229	0.599200027	1.174614815	0.240148853	0.784293572
NT5C	278.1046708	-0.19140565	0.162954108	-1.174598493	0.240155386	0.784293572
PCED1A	620.456137	0.15686001	0.133548631	1.174553485	0.240173402	0.784293572
TMEM222	389.8749447	-0.173064923	0.147405584	-1.174073042	0.24036577	0.784719958
ACADL	26.83619211	-0.453665661	0.386436403	-1.173972373	0.240406092	0.784719958
TUBA4A	177.7674706	0.389948603	0.332182836	1.173897506	0.240436081	0.784719958
CLINT1	1405.350834	0.124934904	0.106450028	1.173648385	0.240535892	0.784901959
NID2	3554.267575	0.303735578	0.258920125	1.173086016	0.240761314	0.785006759
PIWIL4	44.95890761	0.35690653	0.304287253	1.172926326	0.240825351	0.785006759
FZD6	1426.036263	-0.204660526	0.174497616	-1.172855716	0.240853671	0.785006759
VENTXP1	5.406595083	1.0550651	0.899760655	1.172606397	0.240953683	0.785006759
AIG1	303.1494747	-0.180373732	0.153832995	-1.172529551	0.240984515	0.785006759
FRG1-DT	12.63593788	0.923876891	0.787963056	1.172487572	0.241001359	0.785006759
TSFM	336.0695745	-0.190482432	0.162483827	-1.172316256	0.241070107	0.785006759
MIR548AQ	8.497408784	1.164470467	0.993449332	1.172148825	0.24113731	0.785006759
PMS2CL	94.38549849	0.250889459	0.21405329	1.17208878	0.241161414	0.785006759
CABIN1	1375.012696	0.112513272	0.095998054	1.172037013	0.241182196	0.785006759
DOCK4-AS1	19.41901036	0.812821021	0.693562856	1.171950045	0.241217113	0.785006759
FUZ	247.1494052	-0.216178452	0.184478593	-1.171834891	0.241263352	0.785006759
ATRIP	20.23934131	0.614348995	0.524308722	1.171731403	0.241304911	0.785006759
MAPT	80.02733544	-0.375421963	0.320403335	-1.171716775	0.241310786	0.785006759
MIR378I	4.584448385	-1.327622993	1.133165852	-1.17160519	0.241355604	0.785006759
EIF3J-DT	99.34827256	0.315739049	0.26950277	1.171561425	0.241373183	0.785006759
ACBD3-AS1	25.01569662	0.724549072	0.618449779	1.171556846	0.241375022	0.785006759
ADGRG6	40.80473592	0.491395366	0.419463883	1.171484332	0.241404152	0.785006759
CFAP157	128.3387269	0.296307989	0.252934259	1.171482229	0.241404997	0.785006759
SREBF2-AS1	171.6992658	0.238948281	0.203993452	1.1713527	0.241457036	0.785032729
CYP51A1	1408.080562	-0.208608913	0.178126925	-1.1711251	0.241548496	0.785132679
FOCAD	1599.852996	0.101413706	0.086602277	1.171028168	0.241587455	0.785132679
LOC646736	2.30099628	-1.494207846	1.276097248	-1.170920044	0.241630917	0.785132679
UNC13D	16.51199281	-0.629995029	0.538072033	-1.170837715	0.241664015	0.785132679
MAFF	44.63253936	-0.455539576	0.389174673	-1.170527291	0.241788838	0.785395023
RNF26	1052.980683	0.184631991	0.157763333	1.170309902	0.241876278	0.785452947
SYDE1	660.6766841	-0.147011824	0.12562281	-1.170263771	0.241894836	0.785452947

GOLGA8A	2036.599132	0.394662654	0.337274043	1.170154246	0.241938902	0.785452947
NRG4	37.43734744	0.488556036	0.417651423	1.169769833	0.242093607	0.785812034
RPL23	3917.725526	-0.223774318	0.191324603	-1.169605557	0.24215974	0.785883549
METTL3	859.6747099	0.126715592	0.108366455	1.169324882	0.242272762	0.786107177
MARCHF3	47.73035659	-0.58783694	0.502772632	-1.169190409	0.242326924	0.786139776
CCDC188	8.084356361	-0.749054192	0.640781533	-1.168969693	0.242415842	0.786285094
CUL9	695.030734	0.155658807	0.133179146	1.168792647	0.242487183	0.786373359
MIR1245B	43.66067561	0.54000721	0.462160225	1.16844155	0.242628703	0.786672018
BIRC2	837.1345264	0.21108366	0.180668923	1.168345152	0.242667569	0.786672018
GABRE	23.47002722	0.585319624	0.501137633	1.16798178	0.242814114	0.787003914
STUM	4.173635123	1.255386897	1.075208602	1.167575198	0.242978159	0.787390033
TPM2	697.7139409	-0.204866137	0.175514729	-1.167230455	0.243117315	0.787390033
ZSWIM7	123.2546163	0.226876799	0.194391969	1.167109939	0.243165974	0.787390033
ASIC4	12.77446154	0.739390975	0.633582782	1.166999793	0.243210453	0.787390033
LINC00607	609.7182131	-0.364960187	0.312774013	-1.166849455	0.243271171	0.787390033
TRIM23	286.1782252	-0.168821042	0.144712733	-1.166594247	0.243374268	0.787390033
MPP3	236.8234336	0.466708253	0.40007027	1.166565697	0.243385803	0.787390033
RRN3P3	62.31895556	0.560452802	0.480500633	1.166393474	0.243455396	0.787390033
CEPT1	690.9411815	-0.175151216	0.150194047	-1.166166163	0.24354727	0.787390033
POLR2G	455.2604072	-0.172663074	0.14806515	-1.166129059	0.243562269	0.787390033
SHC1	1690.49152	-0.117168164	0.100477495	-1.166113508	0.243568556	0.787390033
RAB27B	574.2984727	-0.284573975	0.244040308	-1.166094148	0.243576382	0.787390033
TESK2	60.51258662	0.401615848	0.344412839	1.166088491	0.243578669	0.787390033
LOC100130992	15.66086335	-0.603570414	0.517630219	-1.166026232	0.24360384	0.787390033
EBF4	13.80919678	0.687611839	0.58977908	1.165880349	0.243662825	0.787390033
TMA16	285.1252981	0.182364566	0.156433585	1.16576352	0.24371007	0.787390033
CDC37	1355.213981	-0.224564363	0.192642694	-1.165704023	0.243734133	0.787390033
HLA-DMB	42.64808751	0.452909669	0.388547143	1.165649207	0.243756304	0.787390033
PAPLN	52.39428911	0.378564923	0.324778796	1.165608492	0.243772773	0.787390033
TECPR1	456.2439468	0.1916197	0.164426222	1.165384077	0.24386356	0.787390752
PIK3R4	696.1052388	0.170053863	0.145929637	1.165314099	0.243891874	0.787390752
PLBD1	560.6645738	0.205321619	0.176209587	1.16521253	0.243932975	0.787390752
PES1	1349.910869	-0.231821366	0.198959075	-1.165171107	0.243949739	0.787390752
BEND3P3	124.4286859	0.356922483	0.306411746	1.164845953	0.244081354	0.787543509
GRIK2	163.1245202	0.268959132	0.230909183	1.164783174	0.244106771	0.787543509
IL18R1	85.22556015	0.321198145	0.275836409	1.164451588	0.244241052	0.787543509
TM4SF19-TCTEX1D2	10.27280298	0.914690856	0.785700283	1.164172747	0.244354012	0.787543509
BMS1P1	20.74244572	0.72329165	0.621336509	1.164090053	0.244387519	0.787543509
FN3KRP	459.6128479	-0.168737763	0.144953095	-1.164085273	0.244389456	0.787543509
RNF216-IT1	2.556648346	1.523978658	1.309267305	1.163993519	0.244426638	0.787543509
C12orf4	250.5081807	-0.333550844	0.286576887	-1.163913976	0.244458875	0.787543509
SLC16A3	233.6395091	-0.339913073	0.292081366	-1.163761586	0.244520644	0.787543509
TONSL-AS1	28.83335161	0.740559553	0.636431074	1.163613129	0.244580828	0.787543509
GORAB-AS1	26.35542296	0.890720767	0.765536333	1.163525137	0.244616506	0.787543509
CTBP1-AS	71.52139091	0.501226603	0.430787629	1.163512061	0.244621808	0.787543509
METTL16	1171.588832	0.136279707	0.117128186	1.163509075	0.244623018	0.787543509
SYCE1L	5.57441806	0.961304192	0.82627412	1.163420431	0.244658964	0.787543509
TENT4A	814.5855829	0.152576044	0.131149879	1.1633716	0.244678767	0.787543509
FAT1	3215.728388	0.24330748	0.2091578	1.163272322	0.244719032	0.787543509
ZXDB	140.4751403	0.217500422	0.186990476	1.163163101	0.244763334	0.787543509
FAM20B	2609.056804	0.226928482	0.195116621	1.163040243	0.244813175	0.787543509
LOC100294145	271.8627692	-0.199587646	0.171617124	-1.162982115	0.244836759	0.787543509
TTC6	13.31850795	0.735310124	0.632358446	1.162805887	0.244908268	0.787631355
ZDHHC5	1287.220756	-0.162331252	0.139622084	-1.16264739	0.244972595	0.787647069
IMPDH2	3036.682222	-0.185853327	0.159868253	-1.162540556	0.245015961	0.787647069
LINC02241	90.48165882	0.349135994	0.300340521	1.162467165	0.245045755	0.787647069
UXS1	449.6690922	0.183298204	0.157710084	1.162247838	0.245134808	0.787647835
LOC102723582	14.89957052	0.72578277	0.624634308	1.161932286	0.245262971	0.787647835
PMS2P5	37.97474595	0.410344255	0.353189361	1.161825074	0.245306527	0.787647835
UBTF	1767.63072	-0.143224918	0.123277015	-1.161813643	0.245311171	0.787647835
LINC01192	32.8415294	0.430810705	0.370877571	1.161598163	0.245398728	0.787647835
TBC1D14	319.4858203	-0.198464826	0.170860993	-1.161557255	0.245415353	0.787647835
CDC42BPA	3432.49063	-0.173592379	0.149448328	-1.161554502	0.245416472	0.787647835
LOC100128361	3.61540057	1.506539437	1.297112497	1.161456265	0.245456399	0.787647835
NR2C2	1518.04226	0.142851015	0.122993704	1.161449819	0.245459019	0.787647835
NKD1	14.96374305	0.913910579	0.786981336	1.161286218	0.245525522	0.787647835
CD47	972.7423705	-0.111640741	0.096136781	-1.161269803	0.245532196	0.787647835
RPRD1A	1236.388148	0.107284632	0.092409189	1.160973631	0.245652623	0.787768562
HACE1	466.1273202	0.153799273	0.13251059	1.16065647	0.245781631	0.787768562
CD14	6.540073383	-1.00336801	0.864523112	-1.160602876	0.245803435	0.787768562
ETAA1	388.6099507	0.185877528	0.160163121	1.160551366	0.245824393	0.787768562
ARHGAP15	5.808582792	-1.315069544	1.133267897	-1.160422481	0.245876838	0.787768562
DGAT1	692.2061945	0.154206792	0.132888904	1.160418872	0.245878307	0.787768562
LOC653653	6.92080027	-0.806633966	0.695173905	-1.160334069	0.245912819	0.787768562
MIEF2	121.6278177	-0.257174814	0.221654159	-1.160252596	0.245945978	0.787768562
ERBB2	1224.339078	0.115829462	0.099835836	1.16019925	0.245967692	0.787768562
NDUFAF4	111.6244995	0.253754595	0.218754959	1.159994709	0.24605096	0.787893641
MAP11	100.1076684	0.399010744	0.344062399	1.159704591	0.2461691	0.788130321
AGFG2	112.3032199	0.25531513	0.220194693	1.159497202	0.246253576	0.788135972
ZNF550	271.1265082	0.196181031	0.169212559	1.159376302	0.246302832	0.788135972
SLC38A4	105.2118453	0.435859696	0.375964097	1.159312018	0.246329025	0.788135972
MAGEF1	525.7110183	-0.237438176	0.204817683	-1.159266001	0.246347776	0.788135972
LOC729291	14.74547525	0.707229895	0.610174331	1.159062024	0.246430904	0.788260404
VPS35L	1075.941679	-0.134368957	0.115967091	-1.158681786	0.246585918	0.78861469
BTN2A1	379.7542499	0.243533259	0.210218039	1.15847936	0.24666847	0.788707163
GCC2	547.807599	-0.138084706	0.11920831	-1.158347991	0.246722054	0.788707163
FBXO9	977.6931368	-0.331070244	0.285827881	-1.158285341	0.246747611	0.788707163
SYNPO	53.37028368	-0.41626104	0.359493618	-1.1579094	0.246901011	0.788940286
ESCO1	368.926054	-0.157434554	0.135966809	-1.157889606	0.24690909	0.788940286
PRRC1	2498.961637	0.185116964	0.159900313	1.157702326	0.246985535	0.789043067

SLC25A5-AS1	26.9660051	0.453434558	0.391770426	1.157398639	0.24710953	0.789297691
SNORA60	7.823847713	0.917577776	0.792914442	1.157221672	0.247181806	0.789387057
LINC02154	901.5282632	-0.977194631	0.844608696	-1.156979127	0.247280888	0.789561983
METTL9	1508.138432	-0.201157399	0.17391082	-1.156669836	0.247407278	0.789643129
ATXN2	1119.780585	0.115984778	0.100281534	1.156591584	0.247439262	0.789643129
SLC3A1	14.9369683	0.566373859	0.489744752	1.156467437	0.247490011	0.789643129
GJD3	11.12235656	-0.684481407	0.591899429	-1.156415049	0.247511429	0.789643129
PRPS1	1086.171906	0.158311655	0.136903405	1.156374853	0.247527863	0.789643129
PPP4R1-AS1	5.57451554	-1.11101286	0.960910728	-1.156208196	0.247596008	0.789670368
PEX2	860.5352972	0.13453405	0.116365122	1.156137232	0.247625029	0.789670368
GNAI3	1729.784552	-0.117810339	0.101928569	-1.155812738	0.247757762	0.789952283
OASL	4.204246719	-1.671619565	1.446644245	-1.155515304	0.247879469	0.790058419
IPO9	3527.526559	-0.109765913	0.095009643	-1.155313385	0.247962117	0.790058419
MFSD9	190.3810312	0.202207783	0.175044116	1.155181835	0.248015972	0.790058419
FAH	366.1785833	0.151981945	0.131569983	1.155141481	0.248032494	0.790058419
MB21D2	243.4011389	0.195347006	0.169125819	1.155039529	0.248074239	0.790058419
SPNS2	72.81421073	0.501605004	0.434313949	1.154936436	0.248116457	0.790058419
LRRK2	20.73137841	-0.581772719	0.503756718	-1.154868408	0.248144318	0.790058419
ISL1	106.1604804	0.259788115	0.224972031	1.154757392	0.24818979	0.790058419
CDRT15	15.77716166	0.781090244	0.676411089	1.154756711	0.248190069	0.790058419
NRDE2	502.4376439	0.23022736	0.19942047	1.154482089	0.248302577	0.790086999
DENND3	53.85734354	0.348711431	0.302070828	1.15440287	0.248335039	0.790086999
NDUFS2	1328.962612	-0.119982007	0.10394289	-1.15430701	0.248374324	0.790086999
GLCCI1	263.239281	0.314107205	0.27211875	1.154301954	0.248376396	0.790086999
GAS6	453.0118877	-0.298700151	0.258917969	-1.153647823	0.248644583	0.790616074
GATA1	2.230345263	1.888008793	1.636597333	1.153618397	0.248656652	0.790616074
BCL9L	393.8011142	0.181287227	0.157152981	1.153571669	0.248675819	0.790616074
LDLR-AS1	10.79562729	0.697982142	0.605164286	1.153376295	0.248755967	0.790658336
TCF4-AS1	2.408930612	1.543792303	1.338593366	1.153294452	0.248789546	0.790658336
UBE2M	488.1721464	-0.168357187	0.14598944	-1.153214827	0.248822219	0.790658336
FUBP3	1117.33879	-0.112356063	0.09746428	-1.152792214	0.248995681	0.790693252
DYSF	88.37209467	0.417318761	0.36203848	1.15269173	0.249036937	0.790693252
ETFRF1	69.34051928	-0.340502617	0.295419218	-1.152608211	0.249071231	0.790693252
TUBA1B	8385.88406	0.228836248	0.198540163	1.15259424	0.249076968	0.790693252
LOC101928163	5.034499047	-1.158162114	1.004852038	-1.152569801	0.249087004	0.790693252
SNORA71D	34.73130982	0.541270428	0.469632852	1.152539533	0.249099434	0.790693252
EXTL3-AS1	7.360475185	1.105246642	0.959093572	1.152386664	0.249162218	0.790751689
SH2D4A	260.7545427	-0.163674189	0.1420455	-1.152265916	0.249211818	0.79076827
TTC3	5368.818835	0.182077986	0.158099866	1.151664387	0.249459012	0.791411713
TMPRSS9	87.28603998	0.552057917	0.47948675	1.151351766	0.249587549	0.791678555
STARD3NL	750.6907429	0.152298051	0.132290172	1.151242372	0.249632538	0.79168034
RPLP1	4821.029674	-0.250634255	0.217738567	-1.151078831	0.249699806	0.791752765
B3GLCT	293.1421164	-0.175305796	0.152345355	-1.150713105	0.249850284	0.792055316
EIF4B	12275.71648	-0.261453195	0.227247771	-1.150520396	0.249929599	0.792055316
LARGE1	10.6524521	0.765899935	0.665736491	1.15045509	0.249956481	0.792055316
GPRACR	3.533095139	-1.431843977	1.244632643	-1.150414931	0.249973013	0.792055316
CAT	493.017896	-0.166754146	0.145028394	-1.149803436	0.25022484	0.792712292
ATPAF1	485.950765	-0.15464786	0.134527197	-1.149565762	0.250322767	0.792779904
LRRC37A3	40.10061179	0.539516692	0.469334442	1.149535692	0.250335158	0.792779904
ADCK2	369.9045391	-0.215942622	0.187882921	-1.149346736	0.250413034	0.79286031
EBLN3P	960.6182613	0.143449694	0.12482435	1.14921243	0.250468396	0.79286031
SMYD5	654.4586109	0.166520729	0.144907706	1.149150262	0.250494026	0.79286031
WDR38	17.38015983	0.56875892	0.495042181	1.148910017	0.250593087	0.792873216
CAPRIN1	5256.431061	-0.138102137	0.120206465	-1.148874452	0.250607754	0.792873216
MIR148B	5.472398633	-0.93242837	0.81165328	-1.148801333	0.25063791	0.792873216
C9orf78	259.6563069	-0.208108906	0.181167679	-1.148708797	0.250676077	0.792873216
MYO19	1463.080799	0.199211868	0.17346464	1.148429262	0.2507914	0.793097203
NUP35	461.902398	0.20269806	0.176551727	1.148094465	0.250929569	0.793267366
CHMP1B	457.416104	-0.271610829	0.236609202	-1.147930119	0.250997413	0.793267366
ZNF768	482.9613104	-0.173670476	0.151293958	-1.147900937	0.251009461	0.793267366
NUF2	472.8608694	0.189936683	0.165469172	1.147867491	0.25102327	0.793267366
KANSL1L-AS1	11.8306689	0.745694331	0.649822705	1.14753505	0.251160556	0.793521435
SRR	104.8588742	0.256741088	0.22375722	1.147409183	0.251212548	0.793521435
ILRUN	2282.589438	0.182009947	0.158635157	1.147349369	0.251237258	0.793521435
BRSK1	70.78972277	0.293062256	0.255486992	1.147073099	0.251351411	0.793608621
MIR199A1	13.21224508	0.655283624	0.571358123	1.146887735	0.251428022	0.793608621
SP4	388.4786675	0.142952838	0.124646911	1.146862258	0.251438554	0.793608621
PCDHB10	27.78096852	0.438574485	0.382434443	1.14679651	0.251465732	0.793608621
USPL1	491.7387118	0.219552429	0.191472042	1.146655291	0.251524115	0.793608621
RNF112	19.03190939	0.651141951	0.567871515	1.146636051	0.25153207	0.793608621
ORC2	812.4703561	0.140325736	0.122419859	1.146266114	0.251685059	0.793950745
LYSMD3	462.7257654	0.238818558	0.208393852	1.145996178	0.251796734	0.794131023
FAM172A	570.3806323	-0.194686514	0.169896484	-1.145912554	0.251831336	0.794131023
SCARNA2	830.7125362	0.229756051	0.200525077	1.14577216	0.251889437	0.794173704
USF2	553.6789138	-0.19498254	0.17025711	-1.145224067	0.252116351	0.794363957
GPR3	16.01682938	0.651410739	0.568861649	1.145112771	0.252162446	0.794363957
PSMD6-AS2	50.25031851	0.651454372	0.568929085	1.145053733	0.252186899	0.794363957
LINC01145	80.37141031	-0.371081045	0.324082393	-1.145020688	0.252200588	0.794363957
EFR3B	16.51123407	0.635331935	0.554878991	1.144991874	0.252212524	0.794363957
GALNT4	46.62077587	0.33945803	0.296474949	1.144980481	0.252217243	0.794363957
ARHGEF10	2102.803704	0.12612216	0.110195438	1.144531597	0.252403242	0.794744246
TAS2R4	7.512281573	1.17485338	1.026572489	1.14444269	0.252440093	0.794744246
ZBTB21	803.0825107	0.26594128	0.232404058	1.144305664	0.252496895	0.794744246
FBL	1949.445449	-0.170792084	0.149260031	-1.144258661	0.252516382	0.794744246
MIR3619	11.71388662	0.926004726	0.809461413	1.143976366	0.252633439	0.794826594
MARVELD3	2.367286144	1.525334572	1.333472583	1.143881465	0.252672799	0.794826594
C12orf60	22.19236561	-0.469799708	0.410719417	-1.143845868	0.252687564	0.794826594
LINC01465	209.0292954	0.423851718	0.370622299	1.143621739	0.252780542	0.794826594
E2F4	758.7872886	0.16838359	0.147253807	1.14349227	0.252834262	0.794826594
MAF1	1356.687417	-0.19938411	0.174370348	-1.143451923	0.252851005	0.794826594

SCRIB	3259.858724	0.187588319	0.16406144	1.143402854	0.252871368	0.794826594
EIF5A	10493.15152	-0.242472041	0.212108257	-1.143152295	0.252975365	0.794826594
TNKS2	1400.646264	0.127707349	0.111717954	1.143122876	0.252987578	0.794826594
AAR2	371.1746541	-0.143113906	0.125196811	-1.143111427	0.25299233	0.794826594
ZNF234	278.7441611	0.226659533	0.198314503	1.142929688	0.253067785	0.794826594
SYNE1	1142.704387	-0.15896178	0.139085659	-1.14290561	0.253077783	0.794826594
BLCAP	422.8386924	-0.198124971	0.173380505	-1.142717696	0.25315582	0.79493158
WAC	1810.825502	-0.172071127	0.150624068	-1.142387995	0.253292779	0.795201114
IFT46	317.2487206	-0.153946169	0.134769041	-1.142296237	0.253330905	0.795201114
ARMC2	46.70740272	0.403653685	0.353422001	1.142129477	0.253400204	0.795278556
ARL8A	204.8659314	-0.187287095	0.164003113	-1.141972806	0.253465323	0.795304732
LINC02302	13.23278341	0.942019498	0.824961814	1.141894669	0.253497805	0.795304732
SEC11A	2182.230839	-0.144961016	0.126965982	-1.141731143	0.253565792	0.795377998
TTC19	548.7588333	-0.142704627	0.125011459	-1.14153237	0.253648449	0.795497249
RNPS1	2051.380901	0.123970611	0.108669731	1.14080168	0.253952461	0.796229842
TTLL1	97.42424521	-0.382146833	0.335015795	-1.140683033	0.25400185	0.796229842
C16orf46	13.42047424	0.694749521	0.609151721	1.140519672	0.254069862	0.796229842
SEPTIN4	7.868562158	0.770676053	0.675798595	1.140393098	0.254122567	0.796229842
CCDC85A	89.63391068	0.530346401	0.465134617	1.14019981	0.254203067	0.796229842
MEAK7	207.1653823	-0.205473902	0.180250515	-1.139935171	0.254313311	0.796229842
APBA1	86.40643102	-0.271958175	0.238573626	-1.139933945	0.254313822	0.796229842
MIR7110	2.913686672	1.511584267	1.326250107	1.139742994	0.25439339	0.796229842
LOC644554	4.744776468	1.351265218	1.18572049	1.139615305	0.254446606	0.796229842
CTC1	423.4039317	0.312219961	0.273979235	1.139575271	0.254463293	0.796229842
LOC101927809	4.918524381	-1.112738324	0.976483981	-1.139535667	0.254479801	0.796229842
MAB21L2	8.755005008	0.957732391	0.840585189	1.139363866	0.254551421	0.796229842
LINC01012	29.45661244	0.480957196	0.422215225	1.139128028	0.254649759	0.796229842
AARD	34.79980003	-0.370388816	0.325154743	-1.139115525	0.254654973	0.796229842
LINC00461	21.65644898	-0.586359686	0.514849182	-1.138896024	0.254746524	0.796229842
EXTL1	23.06270029	0.717568583	0.630059245	1.138890649	0.254748766	0.796229842
NGEF	14.28359757	-0.650368302	0.571067386	-1.138864375	0.254759726	0.796229842
BNIP3L	3028.248782	-0.174020624	0.152804988	-1.138841252	0.254769373	0.796229842
MMADHC-DT	4.486430398	1.227400567	1.077776395	1.138826729	0.254775431	0.796229842
GNG11	40.82374212	-0.332922532	0.29233833	-1.13882614	0.254775677	0.796229842
KIAA1671	3.721307021	-1.129092279	0.99154663	-1.138718286	0.254820673	0.796230825
MAP3K7CL	4.571175351	-1.009924581	0.887215612	-1.138307947	0.254991917	0.796505442
BAALC-AS2	2.277220358	2.077287771	1.824971945	1.138257373	0.255013028	0.796505442
MAT2B	854.0996365	-0.179634785	0.157825452	-1.138186411	0.255042652	0.796505442
BCAM	21.76190313	0.544564807	0.478593547	1.137844023	0.255185619	0.796564644
MAP3K4	1018.054203	0.198299783	0.17430652	1.137649834	0.255266729	0.796564644
HPS4	628.2722107	0.127537256	0.112111716	1.137590786	0.255291396	0.796564644
SNORD14A	12.71866278	0.623093377	0.547776835	1.137494938	0.255331439	0.796564644
RBP5	81.01631133	0.390873475	0.343664181	1.137370423	0.255383466	0.796564644
HOXC5	30.92409288	-0.51543426	0.453200591	-1.137320359	0.255404387	0.796564644
ZNF554	55.04584641	-0.381303565	0.335305486	-1.137182603	0.255461958	0.796564644
PLEKHG6	46.39096632	0.561640469	0.493921688	1.137104289	0.255494691	0.796564644
DLGAP4-AS1	12.92971411	0.769704164	0.676917305	1.137072665	0.25550791	0.796564644
RNASET2	56.39603989	-0.341296886	0.300154424	-1.13707098	0.255508614	0.796564644
TLN1	6802.145831	-0.146636966	0.129057231	-1.136216588	0.25586593	0.797512878
ZNF800	716.9281788	0.176584723	0.155431338	1.136094721	0.255916924	0.797512878
USP47	1082.787363	-0.128407636	0.113032625	-1.136022771	0.255947034	0.797512878
ARID4A	382.4529912	-0.169451535	0.149191518	-1.135798718	0.256040814	0.797665612
KMT2B	1419.076047	0.120165223	0.105825376	1.13550481	0.256163868	0.797782699
GUSBP16	7.634512408	0.91818182	0.80861804	1.135495097	0.256167935	0.797782699
FZR1	695.5970515	-0.110348706	0.097204016	-1.135227843	0.256279866	0.797923861
LINC02237	4.182753453	1.144152942	1.008031638	1.135036737	0.256359926	0.797923861
BTBD11	6.75241708	-1.164148743	1.025660664	-1.135023292	0.256365559	0.797923861
SLC14A2	7.176299033	1.034500119	0.91155286	1.13487672	0.256426975	0.797923861
SLC43A3	748.9028504	-0.267870744	0.23605093	-1.134800631	0.256458862	0.797923861
REX1BD	218.0349969	0.182704387	0.161009689	1.134741569	0.256483615	0.797923861
LINC02043	16.83999441	0.77646114	0.684324525	1.134638774	0.2565267	0.797923861
PDLIM1	367.5976355	-0.182254369	0.16067278	-1.134320133	0.256660287	0.798118559
ZNF469	11.55327079	0.827840777	0.729938575	1.13412389	0.256742584	0.798118559
MRPS31P5	106.3562567	0.292637713	0.258031937	1.134114313	0.256746601	0.798118559
PFN2	4719.275537	0.209521763	0.184761211	1.1340138	0.256788759	0.798118559
BCL2L14	3.014196893	1.44838761	1.277442002	1.133818684	0.256870612	0.798118559
SNORD28	21.63883083	0.811692881	0.715946477	1.133734025	0.256906133	0.798118559
SLIRP	279.3449948	0.223297458	0.196974234	1.133637903	0.256946467	0.798118559
POLH	1027.364431	0.174713405	0.154117836	1.133635211	0.256947597	0.798118559
PPIA	7226.281953	-0.129730128	0.114448242	-1.133526619	0.256993169	0.798120996
KDM6A	1214.747766	-0.238686739	0.210590679	-1.133415494	0.257039811	0.798126751
IGIP	87.38845101	-0.276606552	0.244107598	-1.133133725	0.257158101	0.79835494
ARL2-SNX15	4.636250471	0.941755508	0.831467074	1.132643177	0.257364129	0.798719146
ABHD18	225.7146121	0.18562948	0.163893948	1.13261949	0.25737408	0.798719146
SYT12	2.40145766	-1.718692435	1.517563274	-1.132534283	0.25740988	0.798719146
NBPF6	52.50495006	0.339308484	0.299674656	1.132256188	0.257526744	0.798942649
EIF3F	1746.696123	-0.220008306	0.194342994	-1.13206194	0.257608395	0.798976999
SNORA80A	4.61803204	1.096952341	0.969025027	1.132016523	0.257627488	0.798976999
PPDPF	123.899305	-0.213412817	0.188555858	-1.131828095	0.257706714	0.798985271
MIR4691	9.004077082	0.872625458	0.771008873	1.131796908	0.257719828	0.798985271
FGF7P3	28.15703721	0.589127121	0.520638074	1.131548287	0.257824392	0.799170406
DERL3	6.05491756	-0.981110835	0.867232203	-1.131312735	0.257923486	0.799338525
COPS4	341.9108669	-0.175894	0.155560535	-1.130710946	0.258176773	0.79954061
GPRASP2	2.37260503	1.493935321	1.321272299	1.130679363	0.25819007	0.79954061
MRPL38	613.3155082	-0.128630139	0.113764921	-1.130666095	0.258195657	0.79954061
LLPH	236.3389746	-0.187497645	0.165867497	-1.130406189	0.258305107	0.79954061
YIPF3	844.7194635	-0.216330113	0.191381877	-1.130358404	0.258325234	0.79954061
H2BS1	9.000244972	-0.653219594	0.578063727	-1.130013117	0.258470697	0.79954061
LINC02015	40.39182107	-0.399671502	0.353700465	-1.129971662	0.258488166	0.79954061
RTL8B	160.4010449	-0.207260156	0.18343146	-1.129905175	0.258516183	0.79954061

VTI1B	660.4348909	-0.149293214	0.132130549	-1.129891723	0.258521852	0.79954061
FAM174A	54.46544983	0.388028235	0.343428737	1.129865365	0.25853296	0.79954061
MIR3193	4.211911479	1.026447212	0.908519788	1.129801712	0.258559787	0.79954061
PRB2	3.066801216	1.601613856	1.417618327	1.129792007	0.258563877	0.79954061
IGLON5	3.668647652	1.336172056	1.182751471	1.129714982	0.258596342	0.79954061
MBD2	339.0855058	-0.161811554	0.143243645	-1.129624661	0.258634415	0.79954061
LMBR1	630.6125092	-0.167451147	0.148245491	-1.129553057	0.258664601	0.79954061
PGM2L1	462.2457269	0.329468995	0.29170656	1.129453501	0.258706574	0.79954061
RIPPLY2	17.60189945	0.555275436	0.491800705	1.129065961	0.258870009	0.799906982
SHANK3	17.51138806	-0.55416917	0.490910071	-1.128860871	0.25895653	0.79994599
PCDH19	121.5630718	-0.382475115	0.338826405	-1.128823223	0.258972414	0.79994599
TCTA	213.6854804	-0.269804842	0.239051602	-1.128646867	0.259046832	0.799951641
AKAP10	588.3126271	-0.130264827	0.11542247	-1.12859158	0.259070164	0.799951641
C1orf56	125.6855125	0.299422802	0.265334503	1.12847292	0.259120247	0.799951641
APBB3	267.6716237	0.181696183	0.161022016	1.128393419	0.259153806	0.799951641
ARSI	21.6382585	0.870933211	0.771924115	1.128262733	0.259208977	0.79998337
IDNK	27.64783889	0.503267901	0.446180122	1.127947832	0.259341952	0.800166687
ZFPM2	657.7899628	-0.32714758	0.290080805	-1.12778086	0.259412479	0.800166687
PTPN12	998.2779636	-0.186903326	0.165737962	-1.127703778	0.259445042	0.800166687
GCNA	23.54452699	-0.540483669	0.479281014	-1.127696808	0.259447986	0.800166687
PLD2	296.9068712	-0.17424141	0.154605584	-1.12700593	0.259739974	0.800833439
KMT2D	2266.32622	0.250871682	0.222606723	1.126972624	0.259754056	0.800833439
TMEM128	169.0432653	-0.251577449	0.223331548	-1.126475194	0.259964435	0.801296016
GPR75-ASB3	3.590246762	-1.392712449	1.236422196	-1.126405247	0.259994027	0.801296016
IFT172	565.1080566	-0.168709567	0.14986495	-1.12574399	0.260273898	0.80178018
EFR3A	3668.706434	-0.098669078	0.087689704	-1.125207099	0.260501286	0.80178018
LY6E-DT	3.544244644	1.26630707	1.125597904	1.125008376	0.260585485	0.80178018
LOC101930071	3.201878338	1.237792614	1.100353763	1.124904241	0.260629615	0.80178018
MC4R	11.71986586	-0.688774007	0.612373803	-1.124760733	0.260690438	0.80178018
ZNF571	65.2217083	-0.284573286	0.253021286	-1.124700968	0.260715771	0.80178018
SLC25A45	30.08841984	-0.446738047	0.397251076	-1.124573537	0.260769793	0.80178018
ARMCX5-GPRASP2	61.02953492	0.327773123	0.291532139	1.12431214	0.26088063	0.80178018
MIR570	22.4032462	0.592368157	0.526903217	1.124244714	0.260909225	0.80178018
UNC5D	50.5679707	0.419404689	0.373122914	1.124038953	0.260996501	0.80178018
CFHR4	96.34234796	0.432546258	0.38486582	1.123888471	0.261060343	0.80178018
DPP8	1399.002955	0.106560847	0.094816325	1.123866035	0.261069862	0.80178018
ARFGAP3	1021.901784	0.281369342	0.250371214	1.123808677	0.2610942	0.80178018
RAD51B	541.6427307	-0.239275687	0.212961783	-1.123561627	0.261199043	0.80178018
ITGB1BP2	12.18833719	0.801168679	0.713087532	1.123520806	0.261216369	0.80178018
GSTO2	19.09992409	-0.516361595	0.459612177	-1.123472398	0.261236917	0.80178018
DBNL	1182.563723	-0.143646072	0.127863545	-1.123432581	0.261253819	0.80178018
TENT5A	1751.916476	0.116800833	0.103976095	1.12334314	0.261291789	0.80178018
MUC20	8.769630199	-0.775349384	0.69025297	-1.123282938	0.261317348	0.80178018
CAPN5	38.40219895	-0.348701598	0.31047056	-1.123139012	0.26137846	0.80178018
GOPC	1590.012201	0.123278693	0.109767845	1.123085658	0.261401116	0.80178018
GNAQ	1517.257691	0.123855409	0.110298433	1.122911773	0.261474967	0.80178018
PUM1	2606.740055	-0.10588675	0.094300696	-1.122862873	0.261495738	0.80178018
PPIP5K2	1292.151803	0.110991187	0.098855943	1.122756848	0.261540777	0.80178018
LOC100310756	36.22191122	-0.604500014	0.538433256	-1.122701852	0.261564141	0.80178018
LOC646471	79.7869688	0.367094349	0.326981896	1.122674846	0.261575615	0.80178018
PPP1CB	16403.21139	0.273501242	0.243638648	1.122569198	0.261620503	0.80178018
ELK3	1982.866089	-0.25822277	0.230029309	-1.122564645	0.261622438	0.80178018
RPL26	5517.064835	-0.223144366	0.198783725	-1.122548472	0.26162931	0.80178018
U2AF1L5	23.71664139	0.695538836	0.619609865	1.122543191	0.261631554	0.80178018
SHOC2	482.9610353	-0.150361953	0.133955239	-1.122479072	0.261658801	0.80178018
FMNL3	1677.275836	-0.172411954	0.153632793	-1.122234062	0.261762934	0.80178018
MTA2	1218.682366	-0.16238613	0.144709002	-1.122156383	0.261795954	0.80178018
GOLGA6L10	3.526233293	1.281491935	1.142196365	1.121954136	0.261881941	0.80178018
PLK4	545.0024894	0.175387632	0.156334293	1.121875619	0.261915328	0.80178018
VSIG10L	42.24859149	0.435542086	0.388228079	1.121871676	0.261917005	0.80178018
GOLGA8H	115.607773	0.365292355	0.325625346	1.121817941	0.261939856	0.80178018
KCNH6	2.763201402	1.531482561	1.36518989	1.121809187	0.261943579	0.80178018
ZNF213	200.9607269	0.199117959	0.177505743	1.121755024	0.261966614	0.80178018
SUCO	1291.710446	0.099636604	0.088833405	1.121611897	0.262027491	0.80178018
PRR7-AS1	102.1014985	0.557369272	0.497016785	1.121429473	0.262105096	0.80178018
LARGE2	106.5225457	-0.236096023	0.210533229	-1.121419284	0.262109431	0.80178018
C2orf16	22.33801966	0.476637616	0.425036892	1.121402931	0.262116389	0.80178018
ACVRL1	6.463586681	-1.091103667	0.973097071	-1.121269089	0.262173339	0.80178018
VASP	706.1420397	-0.163335616	0.145694944	-1.121079505	0.262254022	0.80178018
CCDC103	17.7529929	-0.55683355	0.496718891	-1.121023502	0.262277859	0.80178018
TAS2R3	10.59017031	1.188243345	1.060254743	1.120714953	0.262409215	0.80178018
PAQR6	57.7271312	0.435274743	0.388412013	1.120652113	0.262435973	0.80178018
FLNC	4965.906649	0.181861927	0.16232796	1.120336427	0.262570424	0.80178018
GOLGA2	1193.919955	0.243823488	0.21764595	1.12027579	0.262596256	0.80178018
HEBP1	377.9767063	-0.298029675	0.266053537	-1.120186853	0.262634145	0.80178018
CAND1	5247.718474	0.102244392	0.091276059	1.120166598	0.262642775	0.80178018
CLMN	31.79220332	-0.499409239	0.445875134	-1.120065241	0.262685961	0.80178018
LINGO1	502.8512801	0.133702742	0.119395593	1.119829793	0.262786301	0.80178018
BAG2	1254.018931	0.253226911	0.226156287	1.119698746	0.262842159	0.80178018
IRX5	310.7247743	0.313814579	0.280267318	1.119697373	0.262842745	0.80178018
H4C4	1025.433091	-0.280079354	0.250141012	-1.119685859	0.262847653	0.80178018
TOMM20	4840.964818	-0.160029458	0.142938674	-1.119567251	0.262898218	0.80178018
RHOXF1-AS1	6.632035946	1.029636516	0.919724377	1.119505519	0.262924538	0.80178018
PPP2R5A	834.406572	-0.14536232	0.129848734	-1.119474301	0.262937849	0.80178018
LOC100130283	30.15247533	0.521885899	0.466204232	1.119436213	0.262954089	0.80178018
JMJD7-PLA2G4B	2.884315424	1.25241248	1.118806896	1.119417913	0.262961893	0.80178018
ST6GAL1	96.71655174	-0.521308666	0.465719424	-1.119362086	0.262985699	0.80178018
DEPDC5	381.0841416	0.163602597	0.146175472	1.11922058	0.263046048	0.801826988
MAP1S	275.7347513	-0.177298464	0.158483733	-1.118717111	0.263260843	0.802344488
OSBPL6	264.6077726	-0.232049535	0.207454071	-1.118558597	0.263328495	0.802347577

H2AC12	894.0697203	-0.221966847	0.198449802	-1.118503748	0.263351907	0.802347577
ZNF474	50.41745074	1.154457659	1.032427232	1.118197606	0.263482607	0.802482713
CABYR	46.12636822	0.424644639	0.379761096	1.118188892	0.263486327	0.802482713
KHDC4	918.0877336	0.211293878	0.189093944	1.117401609	0.263822648	0.803325486
LOC107984282	6.819523497	0.972878189	0.870716875	1.117330118	0.263853202	0.803325486
AP1G1	2145.847228	0.123119917	0.110210254	1.117136671	0.263935893	0.803394214
JUNB	645.3636164	-0.339290438	0.303753048	-1.116994349	0.263996741	0.803394214
SCARNA18	5.608212007	1.026400554	0.919092609	1.116754225	0.264099426	0.803394214
LOC101929057	52.29563938	0.688389493	0.616419904	1.11675416	0.264099453	0.803394214
SUGP1	199.0901312	-0.218410593	0.195576965	-1.116750087	0.264101196	0.803394214
B3GNT9	247.6248294	0.386635541	0.346401421	1.11614883	0.264358433	0.803748478
ADAM17	1292.554532	-0.141174599	0.126521299	-1.115816859	0.264500536	0.803748478
FGD6	1065.149937	0.177839273	0.159392757	1.115729948	0.264537747	0.803748478
CTIF	1082.918283	0.230179781	0.206329438	1.115593505	0.264596173	0.803748478
YTHDF3	3598.071783	-0.124390885	0.111510916	-1.11550411	0.264634458	0.803748478
IGFBP2	47.45899821	-0.315771014	0.283083057	-1.115471256	0.264648529	0.803748478
HS1BP3	393.7577297	-0.256579482	0.230039942	-1.115369268	0.264692213	0.803748478
UBE2V1	127.6531394	0.302462556	0.271187266	1.115327284	0.264710198	0.803748478
CTPS2	524.5368442	-0.130809237	0.117283698	-1.115323265	0.264711919	0.803748478
SIK2	872.4984384	-0.192917146	0.17297383	-1.115296726	0.264723288	0.803748478
CABP4	3.481677204	-1.24917204	1.120078884	-1.115253629	0.264741751	0.803748478
MIR601	3.285284599	1.618704134	1.45181494	1.114952112	0.264870944	0.803748478
SMCHD1	1787.68243	0.114605847	0.102795144	1.114895528	0.264895193	0.803748478
TNFSF11	147.2083267	-1.007901924	0.904052972	-1.114870429	0.26490595	0.803748478
LINC02615	16.65603034	0.533577352	0.478704978	1.114626704	0.265010422	0.803748478
COL25A1	55.28054843	-0.388185559	0.348272476	-1.114603036	0.265020569	0.803748478
PPM1H	313.789944	0.288894811	0.259198407	1.114570166	0.265034661	0.803748478
RASA4	3.354149219	-1.28777129	1.155473072	-1.114497015	0.265066024	0.803748478
PHF12	830.7719713	-0.162793918	0.146082772	-1.114395048	0.265109747	0.803748478
C1orf53	22.19713765	0.519134532	0.465924593	1.114202899	0.265192152	0.803748478
ITGA2	191.5715547	-0.180826999	0.16231944	-1.114019364	0.265270879	0.803748478
QSOX2	1490.128279	0.129471828	0.116221873	1.114005686	0.265276747	0.803748478
TMEM160	278.77129	-0.154961398	0.139108768	-1.113958521	0.265296982	0.803748478
RINT1	705.584927	0.118746489	0.106612642	1.113812459	0.265359651	0.803748478
SLC30A4	617.197277	0.214013655	0.192169628	1.113670546	0.26542055	0.803748478
CYP2R1	150.3095482	-0.185084205	0.166196537	-1.113646577	0.265430836	0.803748478
LOC728673	12.97635208	0.581572663	0.522289835	1.113505614	0.265491339	0.803748478
MIR548XHG	175.4963599	0.289202151	0.259728486	1.113478753	0.265502869	0.803748478
ECE1	745.0726903	-0.19510926	0.175247432	-1.113335914	0.265564188	0.803748478
KIF17	34.45751587	0.433108519	0.389024079	1.113320593	0.265570765	0.803748478
GSK3B	1044.560911	-0.133168559	0.119636099	-1.113113516	0.26565968	0.803750626
ERCC4	447.3964385	0.186162227	0.167245303	1.113108851	0.265661683	0.803750626
TMEM14B	659.5099584	-0.164466732	0.147794181	-1.112809255	0.26579036	0.804003431
ZNF674-AS1	64.64835926	0.354272699	0.318406753	1.112641916	0.265862251	0.804084404
PACC1	242.8539186	0.182136002	0.163722835	1.112465481	0.265938064	0.80417721
BAHCC1	990.2057879	0.170708429	0.15351525	1.111996555	0.266139631	0.804650192
ALOX12	84.94061752	0.524776435	0.472090099	1.111602289	0.266309188	0.804907292
SNX13	1622.745237	0.106281775	0.095612495	1.111588765	0.266315005	0.804907292
NUMB	753.3209218	-0.151216067	0.136058897	-1.111401538	0.266395551	0.804956054
FSIP2	124.6329198	0.454149705	0.40868878	1.11123605	0.266466758	0.804956054
ARHGEF4	8.710300026	0.742214305	0.66798905	1.111117472	0.266517788	0.804956054
WDFY2	326.2393694	-0.19009171	0.171082139	-1.111113709	0.266519408	0.804956054
ADSL	1068.208002	-0.17871891	0.160863071	-1.111000238	0.266568247	0.804956054
ECM1	13.73362445	-0.847272949	0.762675849	-1.110921436	0.266602168	0.804956054
RPS3A	12427.3094	-0.218975258	0.197130862	-1.110811654	0.266649429	0.804962363
NCAM1	23.22883552	-1.008594874	0.908132668	-1.110625032	0.266729783	0.805066008
TPT1	8647.30141	-0.216529046	0.194979505	-1.110522082	0.266774118	0.805066008
SRD5A1	464.2914551	-0.137093405	0.123470358	-1.110334553	0.266854889	0.805173403
PLEKHG4	1060.275974	-0.187091661	0.168533717	-1.110114132	0.266949848	0.805323565
GBP7	33.41476387	0.417191482	0.375869001	1.109938518	0.267025521	0.805415501
TNFRSF12A	224.3525794	0.468366164	0.422143264	1.109495767	0.26721637	0.805782625
MPHOSPH9	1383.035729	0.141173831	0.127247099	1.109446361	0.267237672	0.805782625
ATPSCKMT	268.9325579	-0.326366489	0.294237314	-1.109194766	0.26734617	0.805860397
HMGCR	2192.384195	0.214989362	0.193827853	1.109176822	0.267353909	0.805860397
LOC100507373	61.52500335	0.346373894	0.312331122	1.108995773	0.267432005	0.805959468
SSC5D	5.394558397	0.881058946	0.794579585	1.108836626	0.267500667	0.806030078
AACSP1	2.565663912	-1.356925121	1.223983165	-1.108614203	0.267596648	0.806182971
TP53BP1	2380.577632	0.094029406	0.084828117	1.108469802	0.267658974	0.806234434
CARD19	158.2274975	-0.286176059	0.258252465	-1.108125179	0.267807758	0.806329432
DHX9	4578.224682	-0.254050331	0.229356855	-1.107663996	0.268006954	0.806329432
BRAT1	802.7899287	-0.134054785	0.121025429	-1.107658	0.268009544	0.806329432
SLC66A3	340.5699127	-0.139430881	0.125884384	-1.107610617	0.268030016	0.806329432
MIR4734	2.064074925	1.519760774	1.372166255	1.107563146	0.268050527	0.806329432
AURKA	797.7315756	0.138280796	0.124851505	1.1075621	0.268050979	0.806329432
AATK	7.066227143	1.074311623	0.970160605	1.107354408	0.268140729	0.806329432
KAZN	18.39078889	-0.520448719	0.470024552	-1.107279857	0.26817295	0.806329432
UBQLNL	2.667181824	1.27888121	1.155011142	1.107245777	0.268187681	0.806329432
LIPE-AS1	2.836299038	1.459714493	1.318356666	1.10722275	0.268197634	0.806329432
ADAMTS20	54.44440192	0.397672072	0.3591858	1.107148646	0.268229666	0.806329432
ASB4	4.132680512	1.542956104	1.393641632	1.10713979	0.268233495	0.806329432
MDGA1	35.1659863	-0.573051065	0.51775249	-1.10680504	0.268378229	0.806510606
ASH1L-AS1	41.81147931	0.504264235	0.455623624	1.106756123	0.268399383	0.806510606
HRCT1	5.061668386	1.247449197	1.127201917	1.106677675	0.268433311	0.806510606
SPATA18	7.541349274	0.974373003	0.880550924	1.106549294	0.268488841	0.806510606
MAGT1	1303.77384	-0.142770291	0.129031607	-1.106475338	0.268520833	0.806510606
LOC728752	30.04141321	0.469401278	0.42429215	1.106316197	0.268589683	0.806510606
MIR100	8.284165627	0.822328274	0.74340365	1.106166582	0.268654424	0.806510606
ZNHIT3	227.6425879	0.291248082	0.263321589	1.106054703	0.268702843	0.806510606
ENKD1	83.06036487	-0.252087013	0.227929187	-1.105988297	0.268731585	0.806510606
DGLUCY	586.6457963	-0.171975757	0.155502496	-1.105935667	0.268754365	0.806510606

INTS7	879.9559455	-0.138803086	0.125517143	-1.105849629	0.26879161	0.806510606
GCHFR	10.19880274	-0.671793003	0.607636217	-1.105584204	0.26890653	0.80671959
GBF1	1673.173338	0.123765887	0.111987497	1.105175943	0.269083359	0.806841227
NXT1	258.2972798	-0.191294936	0.17309308	-1.105156464	0.269091798	0.806841227
PRRX2-AS1	12.27058215	0.635111651	0.574695979	1.105126317	0.269104858	0.806841227
URB2	1116.109969	0.188424398	0.170508633	1.105072479	0.269128185	0.806841227
LAMTOR5-AS1	5.072864877	-0.902468445	0.816891585	-1.10475914	0.26926397	0.806934929
CDH7	694.9450049	-0.257978821	0.233524889	-1.104716597	0.26928241	0.806934929
ZNF423	8.710758222	-0.724154445	0.655627416	-1.104521299	0.26936707	0.806934929
DSTNP2	33.44956592	0.371839261	0.336661872	1.104488782	0.269381167	0.806934929
FAM86HP	153.3146988	0.217295558	0.196753676	1.104404058	0.269417901	0.806934929
TMEM132A	522.5262725	-0.15175598	0.137413912	-1.104371296	0.269432107	0.806934929
TIGAR	170.4329886	0.240660928	0.21794139	1.104246092	0.269486401	0.806934929
LOC100506282	64.46308185	0.437886911	0.396589183	1.104132263	0.269535768	0.806934929
LOC105447645	3.40631212	1.434363524	1.299277344	1.103970242	0.269606046	0.806934929
SNORD114-13	21.61371314	-0.661257298	0.599049957	-1.103843327	0.269661106	0.806934929
EIF4E2	803.6002311	-0.138607093	0.125572334	-1.103802794	0.269678693	0.806934929
NTN1	94.44425876	-0.363615295	0.329437128	-1.103747164	0.26970283	0.806934929
PFKL	1192.251628	-0.100515809	0.091098766	-1.103371794	0.269865741	0.807219546
DARS-AS1	12.00532886	-0.74130514	0.671886354	-1.103319237	0.269888555	0.807219546
PLPBP	1464.773929	-0.153898187	0.139533706	-1.102946317	0.270050478	0.807568304
ULBP3	226.858913	0.162455751	0.147314409	1.102782489	0.270121634	0.807645558
DPP9	872.9279316	-0.14548549	0.131947386	-1.102602284	0.270199918	0.807664319
TRMT10C	411.8269613	-0.134161187	0.121681597	-1.102559385	0.270218556	0.807664319
TRIM62	42.84270662	0.35139348	0.318742778	1.102435897	0.270272213	0.807681161
CCT5	4235.450149	-0.125721715	0.11405008	-1.102337805	0.27031484	0.807681161
ZC3H6	561.0349935	0.426685349	0.387147719	1.102125437	0.270407142	0.807821504
TAF1A-AS1	12.41252694	-0.58800567	0.533665104	-1.10182522	0.270537664	0.808075959
DNM1P46	21.1287472	-0.578490577	0.52508835	-1.101701414	0.270591502	0.808101319
TC2N	69.82875341	0.460626092	0.418211901	1.101417943	0.2707148	0.80833407
CHST15	411.4965538	0.160229716	0.14553294	1.100985903	0.270902792	0.808387328
NAGLU	810.2596791	0.126397279	0.114822023	1.100810424	0.270979174	0.808387328
CENPBD1	89.9554096	-0.321508899	0.292075718	-1.100772434	0.270995712	0.808387328
WWP2	551.2111274	-0.140916767	0.128020045	-1.100739863	0.271009891	0.808387328
CRKL	1987.631151	0.105253208	0.095627239	1.100661365	0.271044067	0.808387328
ATP5F1A	8790.901284	-0.130065982	0.11817285	-1.100641835	0.27105257	0.808387328
RING1	505.2649098	-0.126905804	0.115314869	-1.100515532	0.271107566	0.808387328
CPE	1192.57258	-0.273200616	0.248267682	-1.100427625	0.271145848	0.808387328
PNPT1	1078.570143	0.195409227	0.177588232	1.100350091	0.271179615	0.808387328
CPEB1	117.7964534	0.216399147	0.19670408	1.100125359	0.271277506	0.808387328
APOL1	11.44746272	1.256752453	1.142393429	1.100104763	0.271286479	0.808387328
FNDC5	11.83805485	0.70668642	0.642442476	1.099999528	0.271332327	0.808387328
GDAP1	402.5679355	-0.387888493	0.352627433	-1.099995226	0.271334202	0.808387328
PGGT1B	392.183822	0.156251533	0.142063214	1.099873283	0.271387337	0.808387328
ZMYM5	297.257407	0.168325769	0.153064931	1.099701726	0.271462103	0.808387328
IDI2	2.218161668	1.359871477	1.236747642	1.099554534	0.271526261	0.808387328
PRR3	400.8696949	0.152400382	0.138615384	1.099447822	0.271572781	0.808387328
NIPBL-DT	260.5546879	0.282740313	0.257205203	1.099279134	0.271646331	0.808387328
ZNF251	351.5221145	-0.158098268	0.143822676	-1.099258286	0.271655422	0.808387328
ELP3	625.2919877	-0.21033896	0.191359311	-1.099183304	0.27168812	0.808387328
SLC25A22	336.3836608	0.211810292	0.192710069	1.099113782	0.271718439	0.808387328
SLC26A4	8.125333398	1.096410537	0.997645102	1.098998567	0.271768691	0.808387328
RSKR	204.8496457	0.446396928	0.406216749	1.098913153	0.271805949	0.808387328
ARPP19	5080.970506	-0.110508955	0.100565282	-1.098877795	0.271821373	0.808387328
ATP1A1	9424.884438	-0.194420473	0.176997998	-1.098433173	0.272015384	0.808521912
DCHS2	48.26853886	0.408350257	0.371909603	1.097982558	0.272212106	0.808521912
CASP3	332.0112328	-0.176098295	0.160405245	-1.097833767	0.272277084	0.808521912
PUM3	582.9149785	0.222387557	0.202572763	1.097815688	0.272284979	0.808521912
SYMPK	1981.167809	-0.103280099	0.094079678	-1.097793928	0.272294484	0.808521912
GIN1	65.47470613	-0.326128459	0.297081853	-1.097773076	0.272303591	0.808521912
MSANTD2	126.7618225	0.312073267	0.28429512	1.097708842	0.272331647	0.808521912
RASAL2-AS1	18.13752166	0.494012904	0.450201295	1.097315601	0.272503453	0.808521912
REEP2	48.57258669	-0.352950813	0.321667966	-1.097251981	0.272531256	0.808521912
CPT2	194.2149613	-0.254621461	0.232057184	-1.097235847	0.272538307	0.808521912
GLB1L2	8.401217163	-0.768676829	0.700630065	-1.097122243	0.272587958	0.808521912
RNF138	696.7506515	-0.129271871	0.117843863	-1.096975835	0.272651956	0.808521912
RPS2	14505.38316	-0.210423587	0.191831155	-1.09692081	0.272676011	0.808521912
IQCJ	3.921867439	-1.041664681	0.949654112	-1.096888506	0.272690134	0.808521912
LRRC9	5.049910702	-1.31828381	1.201932997	-1.096803077	0.272727485	0.808521912
PRB3	10.68488639	1.10651099	1.008907946	1.096741278	0.272754507	0.808521912
CHST10	622.7935993	-0.111001641	0.101221332	-1.096623002	0.272806228	0.808521912
PPP2R2A	1726.024625	0.132107586	0.120502319	1.096307413	0.272944266	0.808521912
NECTIN3-AS1	2.074364805	1.638777047	1.494928101	1.096224658	0.272980471	0.808521912
ELMOD2	403.2515332	-0.141972307	0.129510302	-1.096224045	0.272980739	0.808521912
LTB	4.94457099	1.343318942	1.225446179	1.09618763	0.272996672	0.808521912
LOC100996419	18.60887896	-0.639632842	0.583519311	-1.096163965	0.273007026	0.808521912
ANKDD1A	57.6214984	0.337360136	0.307841747	1.095888195	0.273127706	0.808521912
MRPS14	176.1646175	0.224391851	0.204758024	1.095887948	0.273127814	0.808521912
PYGO1	1121.580197	0.136074315	0.124184204	1.095745761	0.273190051	0.808521912
TAS2R13	9.202764645	0.921879644	0.841327711	1.095743824	0.273190898	0.808521912
CYSRT1	5.148958194	1.018563352	0.929674312	1.095613097	0.273248128	0.808521912
MUC6	7.12027842	-1.134937191	1.03590433	-1.095600394	0.273253689	0.808521912
PGF	104.6851611	0.362106217	0.330520954	1.095562059	0.273270473	0.808521912
ATXN3L	9.848798085	0.831774701	0.75923089	1.095549078	0.273276157	0.808521912
TTN	2921.774589	0.518116186	0.472952769	1.095492448	0.273300952	0.808521912
PPFIA3	257.3648335	0.211374641	0.192968511	1.095384113	0.273348391	0.808521912
TMED10P1	78.95165444	0.324682204	0.296419028	1.095348722	0.27336389	0.808521912
SUGT1P3	3.844736686	1.15695091	1.056359753	1.095224337	0.273418366	0.808548836
CCDC159	5.159991776	-0.98788231	0.90226661	-1.094889581	0.273565014	0.808743096
RELT	220.4505965	0.292006247	0.26670472	1.09486719	0.273574825	0.808743096

ERF	933.5047587	-0.119968681	0.109619639	-1.094408642	0.273775795	0.809202964
ZNF491	23.43599526	0.456461072	0.417212258	1.094073969	0.273922538	0.809502425
LRRK1	1174.206	0.233768813	0.213735364	1.093730155	0.274073345	0.809750032
DYNC1LI2	1613.0317	0.128024286	0.11705872	1.093675769	0.274097205	0.809750032
LOC642846	301.5436962	0.39176157	0.358287387	1.093428306	0.274205791	0.809936549
LIFR	974.5220558	0.14088779	0.128879689	1.093172953	0.27431787	0.810133318
ITGA9-AS1	4.22224779	1.010690297	0.924912826	1.092741142	0.274507471	0.810504646
DMAC1	241.0210314	0.190661012	0.17448943	1.092679439	0.27453457	0.810504646
RPF2	508.7095053	-0.18278867	0.16730171	-1.092569048	0.274583059	0.810513518
DCTN4	1430.267761	-0.140950361	0.129027059	-1.092409316	0.27465323	0.810531792
MREG	202.6834371	-0.189950313	0.173891774	-1.092347889	0.274680219	0.810531792
MIR6079	3.821080269	1.193591884	1.093317056	1.091716146	0.274957886	0.810895193
CCDC17	19.54300643	0.585082481	0.536025496	1.091519872	0.275044193	0.810895193
MSC-AS1	310.9197839	0.211791712	0.194045339	1.091454777	0.275072821	0.810895193
CNNM4	320.1395108	0.147433095	0.135083658	1.091420657	0.275087827	0.810895193
PRKAR2A	1961.899943	0.141890858	0.130015955	1.091334192	0.275125858	0.810895193
PAK2	3682.83566	0.138863021	0.127247971	1.091278863	0.275150195	0.810895193
EML2-AS1	26.80636401	0.441483557	0.404662032	1.090993278	0.27527584	0.810895193
UTP14A	195.2090738	-0.239844874	0.219842335	-1.09098584	0.275279113	0.810895193
RPRM	17.60970615	0.557057291	0.510639022	1.090902315	0.275315869	0.810895193
WNK1	4430.976315	0.180754473	0.165702687	1.090836105	0.275345006	0.810895193
KLB	41.35763567	0.50940309	0.467170509	1.090400784	0.275536637	0.810895193
KIAA0355	540.4731342	-0.187431851	0.171895031	-1.090385512	0.275543361	0.810895193
MARCHF6	4095.118662	0.105725431	0.096965421	1.090341592	0.2755627	0.810895193
SNORD6	9.657326679	-0.663556035	0.608637477	-1.090231969	0.275610974	0.810895193
EMD	268.6543463	0.149589586	0.137219767	1.090146044	0.275648816	0.810895193
CHD1	1043.855068	0.166260513	0.152515814	1.090119831	0.275660362	0.810895193
GRK6P1	4.09597545	1.10435646	1.013123764	1.090050889	0.275690728	0.810895193
CIRBP-AS1	58.17240281	0.335676564	0.307976944	1.089940567	0.275739325	0.810895193
HOXA5	126.8036798	-0.217011996	0.199112848	-1.089894491	0.275759624	0.810895193
EPS15	1806.078774	-0.17597026	0.161477896	-1.089748284	0.275824041	0.810895193
LOC100506804	27.55506171	0.601059632	0.551584346	1.089696682	0.275846778	0.810895193
ADGRL1	819.1454579	-0.107210789	0.098393682	-1.089610501	0.275884755	0.810895193
RPS15AP10	13.26264903	0.844655557	0.775220897	1.08956758	0.275903671	0.810895193
TGFB1I1	560.2578828	-0.140843978	0.129271691	-1.089519116	0.27592503	0.810895193
BMP2K	293.8589323	0.217939825	0.200046706	1.089444707	0.275957826	0.810895193
EIF4G2	14118.32277	-0.228527147	0.20978968	-1.089315482	0.276014788	0.810895193
HSD17B1	9.200003332	-0.70698497	0.649072453	-1.089223502	0.276055338	0.810895193
CD34	2.20435896	-1.435390942	1.317903408	-1.089147303	0.276088933	0.810895193
TSPAN15	593.9474945	0.571129626	0.524419566	1.089070019	0.27612301	0.810895193
STARD5	36.61742964	0.402570296	0.369695279	1.088924633	0.276187122	0.810939119
TRMT2A	588.3652067	0.147372919	0.135349831	1.088829723	0.276228982	0.810939119
CLNS1A	1055.579341	-0.171666581	0.157715216	-1.088459218	0.276392431	0.811004953
RAB18	495.4018796	-0.164738426	0.151353089	-1.088437817	0.276401874	0.811004953
GAS2	7.494756946	-0.743616819	0.683212652	-1.088411955	0.276413286	0.811004953
MGC12916	80.76450442	0.441914897	0.406044718	1.088340466	0.276444833	0.811004953
CNOT3	495.3895872	-0.1529368	0.140532924	-1.088263133	0.276478961	0.811004953
ENDOU	11.41482494	0.696792812	0.640497908	1.08789241	0.276642607	0.811351426
FAM86FP	47.97679299	0.399338533	0.367145688	1.087684114	0.276734583	0.811434056
KLRC4	3.21370492	-1.287550704	1.183869816	-1.087577947	0.276781471	0.811434056
NDUFA9	723.1127846	-0.131213909	0.12065433	-1.087519271	0.276807386	0.811434056
PRRG4	13.13247818	0.718927134	0.661190348	1.087322487	0.276894314	0.811500351
HDAC2-AS2	14.14807665	0.599216451	0.551153452	1.087204387	0.276946492	0.811500351
NIF3L1	465.5620366	0.12560055	0.115533603	1.087134362	0.276977434	0.811500351
CEP72	285.0176329	0.37514198	0.34512792	1.086965031	0.277052264	0.811500351
PDHA1	849.0675049	-0.118107297	0.108667385	-1.086869782	0.277094363	0.811500351
PIAS4	278.0237701	-0.170764121	0.157118431	-1.086849711	0.277103234	0.811500351
ALDH18A1	2255.579862	0.140194402	0.12903972	1.086443785	0.277282699	0.81187337
CPTP	289.8037468	0.14825126	0.136470041	1.086328249	0.277333793	0.81187337
CXorf56	290.5051641	-0.164345028	0.151328525	-1.086014871	0.277472413	0.81187337
DENND5B	509.7603527	0.277489353	0.255533181	1.085922977	0.27751307	0.81187337
NUS1	1764.167136	0.142509672	0.131236979	1.085895708	0.277525136	0.81187337
ZSCAN1	1.838416585	1.650015077	1.519529924	1.085872052	0.277535603	0.81187337
LPIN1	1576.222978	0.247988757	0.228385398	1.085834556	0.277552195	0.81187337
LRRC27	42.96707833	0.355389016	0.32732495	1.085737632	0.277595086	0.81187337
OTUD6B-AS1	300.2296904	0.181109304	0.166824259	1.085629307	0.277643028	0.811873948
ZNF658B	3.031639246	1.182643865	1.089528979	1.085463433	0.277716451	0.811873948
PGLS	334.2790402	-0.281228648	0.259112609	-1.085353001	0.27776534	0.811873948
STON1	990.7403055	0.355465569	0.327541548	1.085253369	0.277809453	0.811873948
CCDC149	143.8110437	-0.3622059	0.333823561	-1.085021979	0.277911921	0.811873948
NIM1K	92.21912144	0.391555575	0.360926787	1.084861498	0.277983003	0.811873948
ARID2	855.2676596	-0.138438269	0.127623845	-1.084736706	0.278038286	0.811873948
TPTE2P6	5.301294552	-1.330501469	1.226747336	-1.084576611	0.278109219	0.811873948
FADS2	2683.276103	0.338922098	0.312495582	1.084566046	0.2781139	0.811873948
ZNF582-AS1	6.74459841	0.877432187	0.809170484	1.0843601	0.278205167	0.811873948
PRB1	5.603210448	1.183210838	1.091394459	1.084127584	0.278308234	0.811873948
FSD1	239.9705421	-0.281233871	0.259429545	-1.084047198	0.278343873	0.811873948
RNF113A	77.94850594	-0.241565418	0.222907911	-1.083700515	0.278497607	0.811873948
BARX1	448.0809558	0.178415055	0.164654098	1.083574947	0.278553304	0.811873948
HDAC1	2035.049002	-0.117778161	0.108696955	-1.083546095	0.278566102	0.811873948
ANAPC16	612.2744677	-0.208481144	0.192408705	-1.083532806	0.278571998	0.811873948
SNORD7	7.122491719	1.029905202	0.950537078	1.083498189	0.278587354	0.811873948
TCF12	3601.611506	-0.140062167	0.129275623	-1.083438352	0.2786139	0.811873948
RNF149	955.3531404	-0.135580588	0.125147724	-1.083364392	0.278646714	0.811873948
TUBA8	6.479547246	0.942832545	0.87032965	1.083305096	0.278673024	0.811873948
ITPRIPL2	2710.345422	-0.135435175	0.125027391	-1.083244035	0.278700119	0.811873948
LMCD1-AS1	5.024636999	-0.870634407	0.803810424	-1.083134009	0.278748946	0.811873948
LINC01351	8.760428827	0.761745306	0.703305356	1.083093282	0.278767021	0.811873948
RSBN1	644.6079806	-0.133252946	0.12303062	-1.083087656	0.278769517	0.811873948
POU2AF1	2.859144852	-1.641475344	1.515573596	-1.083072012	0.278776461	0.811873948

ZNF470	149.9915367	-0.22368261	0.206527524	-1.083064406	0.278779837	0.811873948
LINC01194	20.70504868	0.516346745	0.476899322	1.082716459	0.278934296	0.812070386
CERK	1364.212736	-0.169374827	0.156436417	-1.082707147	0.278938431	0.812070386
LNCOC1	127.8913216	0.245546189	0.226830672	1.082508759	0.279026525	0.812194165
C22orf24	11.39300909	0.809498643	0.748008365	1.082205334	0.279161299	0.812211064
STK38	1241.297241	-0.138217293	0.127719277	-1.082196014	0.279165439	0.812211064
ASAP2	624.5860733	0.207112622	0.191415746	1.082004099	0.279250707	0.812211064
KIAA0754	704.473144	-0.150256774	0.138870367	-1.081993067	0.279255609	0.812211064
EN1	76.27797104	0.301669117	0.27881159	1.081981986	0.279260533	0.812211064
SUPT4H1	402.6731287	-0.172311157	0.159286478	-1.081768894	0.279355232	0.812211064
C9orf147	15.48127977	0.747134037	0.690683286	1.081731745	0.279371743	0.812211064
RBSN	523.9919673	0.182322851	0.168559568	1.081652338	0.279407039	0.812211064
FADS1	4128.782174	0.207013457	0.191435556	1.081374123	0.279530727	0.812211064
LOC100134868	10.13025051	0.651236145	0.602355147	1.081149797	0.279630486	0.812211064
PIGU	309.3004632	-0.14957507	0.138348721	-1.081145299	0.279632486	0.812211064
CD86	2.991076032	-1.13570094	1.050653501	-1.08094718	0.27972061	0.812211064
GLIPR1	810.0945937	-0.319006637	0.295128039	-1.080909282	0.27973747	0.812211064
AP3M2	640.0172852	-0.122908114	0.113719073	-1.08080475	0.279783975	0.812211064
TEAD2	364.8734945	0.159377741	0.147501815	1.080513758	0.279913464	0.812211064
TACR1	12.69430625	1.250742828	1.1575508	1.080507938	0.279916054	0.812211064
LOC101926935	51.738152	0.515082788	0.476739549	1.080428064	0.279951604	0.812211064
MIR7851	2.94591302	1.561498675	1.445259362	1.080427995	0.279951635	0.812211064
GTF3C6	244.275712	-0.254858405	0.235893728	-1.080395004	0.27996632	0.812211064
HOTAIRM1	83.81905028	0.337854409	0.31272853	1.080344059	0.279988997	0.812211064
GVINP1	11.84937706	-0.656458765	0.607639225	-1.08034297	0.279989482	0.812211064
TMEM14A	303.1546436	-0.167684274	0.155252112	-1.080077255	0.28010778	0.812337765
B4GALT7	451.2400154	0.181167949	0.167741875	1.080040085	0.28012433	0.812337765
VAMP1	126.8730071	0.335664123	0.310849391	1.079828792	0.280218427	0.812343858
NAPEPLD	171.0395595	-0.235440857	0.218052864	-1.079742099	0.280257041	0.812343858
RIOK1	479.8857369	0.152484764	0.141252089	1.079522185	0.280355009	0.812343858
LTBP4	548.4018118	-0.167456666	0.155122538	-1.079512158	0.280359477	0.812343858
CHD9	2128.686982	0.112902669	0.104594396	1.079433255	0.280394633	0.812343858
ENOSF1	145.2635764	0.357529819	0.331271496	1.079265265	0.280469493	0.812343858
C1orf220	25.408626	0.624158853	0.57840405	1.079105261	0.280540806	0.812343858
RNF114	638.1092136	-0.138757433	0.12859328	-1.079041091	0.28056941	0.812343858
WEE2-AS1	4.72918468	1.040712498	0.964500771	1.079016761	0.280580256	0.812343858
CNOT11	1147.081311	-0.112514183	0.104284891	-1.078911645	0.280627117	0.812343858
MYCBP	9.133684792	-0.681771783	0.631907965	-1.078909938	0.280627878	0.812343858
HSPBP1	606.4785805	-0.192271274	0.178232255	-1.078768115	0.280691112	0.812394936
EDRF1	474.1229886	0.133875531	0.12412901	1.078519287	0.280802079	0.81245179
GPR161	962.1784289	0.286321085	0.265493861	1.078447102	0.280834276	0.81245179
NHP2	295.9234312	-0.170179789	0.15782002	-1.078315593	0.280892941	0.81245179
ADAMTS5	2065.224383	-0.177201063	0.164331418	-1.078315181	0.280893125	0.81245179
SLC6A1	2.354652981	-1.308373659	1.213563867	-1.078125095	0.280977934	0.812565204
RTL5	82.02386451	-0.346604339	0.321650606	-1.077580246	0.281221122	0.8126105
SNHG6	242.8191446	0.193343864	0.179451906	1.077413265	0.281295681	0.8126105
AK5	119.6681568	-0.455963104	0.423239095	-1.07731802	0.281338214	0.8126105
STARD13	507.7013939	-0.159156864	0.14773463	-1.07731589	0.281339166	0.8126105
TTLL9	4.882976345	1.000259491	0.928526868	1.077254224	0.281366707	0.8126105
MIR151B	6.584779914	1.187859946	1.102677724	1.077250334	0.281368444	0.8126105
BRPF1	340.0465235	-0.18374515	0.170585522	-1.077143874	0.281415995	0.8126105
ZNRF3	451.961529	0.148131648	0.137594117	1.076584169	0.28166608	0.8126105
RAD54L2	1049.642728	0.102084307	0.094833614	1.076456997	0.281722923	0.8126105
NCS1	2083.182469	0.124573445	0.115726753	1.076444657	0.281728439	0.8126105
AATBC	43.09515814	0.405934519	0.377120219	1.076406139	0.281745658	0.8126105
BRI3BP	564.8549369	0.180101735	0.167328707	1.076334942	0.281777487	0.8126105
DANCR	227.3333592	0.287282593	0.266908688	1.076332866	0.281778415	0.8126105
CTSB	9622.593181	-0.287160056	0.266810199	-1.076270914	0.281806113	0.8126105
TACR3	3.179189244	1.555845661	1.445592235	1.07626869	0.281807107	0.8126105
EPYC	1131.845939	0.300507545	0.279280492	1.076006211	0.281924479	0.8126105
IMMP2L	116.2332545	0.23652766	0.21983255	1.075944667	0.281952004	0.8126105
RPL22	1617.085314	-0.152947139	0.142152112	-1.075939969	0.281954105	0.8126105
PTCHD4	5.602878432	1.224026711	1.137670776	1.075905909	0.281969339	0.8126105
ZNF211	108.8949431	0.271598078	0.252440745	1.075888436	0.281977154	0.8126105
SORD2P	32.84940227	-0.428665731	0.398430839	-1.075884919	0.281978728	0.8126105
NIPAL4	23.16326747	0.477337377	0.44368623	1.07584447	0.28199682	0.8126105
CD63	2447.051763	-0.143511167	0.133420112	-1.075633687	0.282091115	0.812750796
ERFL	11.95690188	0.619287972	0.575846401	1.075439511	0.282178	0.8128697
GBP1	750.9619702	0.217255193	0.202122438	1.074869247	0.282433272	0.813307726
NAV3	552.9756972	0.169412509	0.157617864	1.074830636	0.282450562	0.813307726
PAICS	3955.115876	-0.158675112	0.147642404	-1.074725875	0.282497476	0.813307726
COG6	734.9914084	0.250312797	0.2329416	1.074573182	0.282565864	0.813307726
EIF3K	1486.561895	-0.148583024	0.13828304	-1.074484796	0.282605455	0.813307726
ATG16L2	215.3455808	0.292408616	0.272154146	1.074422786	0.282633234	0.813307726
MIR2278	5.48267865	1.220296614	1.135807954	1.074386396	0.282649537	0.813307726
BIN1	1160.628197	-0.189221548	0.176169889	-1.074085636	0.282784302	0.813333927
GDF7	27.23158152	0.509439825	0.474341156	1.073994568	0.282825116	0.813333927
IGDCC4	1393.748724	-0.165708012	0.154292421	-1.073986723	0.282828632	0.813333927
LRRC69	50.22526407	0.616672994	0.574205522	1.073958662	0.282841209	0.813333927
LINC00997	121.6344565	-0.202675826	0.188823432	-1.073361625	0.283108895	0.813421005
C2orf68	807.5561288	-0.168753216	0.157224153	-1.073328833	0.283123603	0.813421005
C8orf48	101.0569373	-0.267773063	0.249539096	-1.07307058	0.28323945	0.813421005
ACSS3	405.018585	-0.302483979	0.281897831	-1.073026984	0.283259009	0.813421005
EIF3CL	14.53016161	-0.556754082	0.518953144	-1.072840754	0.283342572	0.813421005
AJUBA	778.8187793	0.247011014	0.230248578	1.072801474	0.283360199	0.813421005
PINK1-AS	90.51072714	-0.229683768	0.214121496	-1.07267963	0.283414883	0.813421005
RAVER1	1283.074598	0.101927229	0.095022374	1.072665574	0.283421192	0.813421005
FOXRED2	90.88139777	-0.310017915	0.289027197	-1.072625406	0.283439221	0.813421005
CYB5R1	336.2913753	-0.150684767	0.14048932	-1.072570973	0.283463654	0.813421005
THAP12	1046.796631	-0.136747822	0.127499822	-1.072533429	0.283480507	0.813421005

ATAD3B	912.8140917	0.367083757	0.342294838	1.072419784	0.283531526	0.813421005
GADD45B	224.6877705	0.331627216	0.309235078	1.072411378	0.2835353	0.813421005
MAP4K1	9.515358005	0.798224671	0.744431131	1.072261271	0.283602697	0.813421005
SH2D5	125.5707085	0.300524232	0.280273347	1.072254055	0.283605937	0.813421005
PIKFYVE	868.2735434	0.123995333	0.115649936	1.07216084	0.283647796	0.813421005
PCDH12	21.89273053	0.583418721	0.544197048	1.072072557	0.283687444	0.813421005
LOC105373021	7.151607441	-0.955161583	0.890959154	-1.072059901	0.283693128	0.813421005
RNF115	662.2084396	-0.125903135	0.117477461	-1.071721621	0.283845087	0.813507694
SIGLEC15	184.8410613	-1.231511322	1.149184585	-1.071639263	0.283882091	0.813507694
PPIL6	47.16011387	0.380108713	0.35470974	1.071604947	0.28389751	0.813507694
ITGB4	90.43133326	-1.236283156	1.153906287	-1.071389566	0.283994303	0.813507694
NEIL1	297.3997478	0.320298626	0.298992506	1.071259711	0.28405267	0.813507694
CFAP70	11.46747216	-0.774975729	0.723467417	-1.071196451	0.284081107	0.813507694
ID3	1959.918132	0.296771016	0.277085084	1.071046522	0.284148512	0.813507694
TAS2R43	3.771537751	1.451267599	1.3550754	1.070986603	0.284175454	0.813507694
UBR1	1441.146506	0.115372756	0.107730961	1.070934073	0.284199074	0.813507694
AASDHPPT	622.9949015	-0.151035182	0.141038586	-1.070878444	0.28422409	0.813507694
MINDY1	193.6815534	0.188720695	0.176230327	1.070875246	0.284225528	0.813507694
FAM133CP	20.84288334	0.899280607	0.839895086	1.070705879	0.284301698	0.813595032
PIGA	155.6770712	0.243674844	0.227631025	1.070481688	0.284402547	0.813727796
PDIA5	597.7763856	-0.177715493	0.166035665	-1.070345294	0.284463914	0.813727796
BOLA1	42.33459649	0.357866125	0.33438744	1.070214015	0.284522987	0.813727796
WAS	2.017418952	-1.731811629	1.618346712	-1.070111625	0.284569067	0.813727796
CTTNBP2NL	521.8073848	0.170745828	0.159561328	1.07009531	0.28457641	0.813727796
ZNF512B	727.5205204	-0.303093793	0.283275675	-1.069960535	0.284637073	0.813770679
CTU2	233.7961021	0.161489571	0.150953268	1.069798444	0.284710043	0.813848727
CBLL1	446.7883717	0.143707198	0.134350296	1.069645563	0.284778878	0.81389305
PRDM1	31.74029453	-0.387425974	0.362228917	-1.069561141	0.284816894	0.81389305
SPATA1	17.15387253	0.735728878	0.688053063	1.069290897	0.284938611	0.814110317
LOC101929162	58.9078895	0.410162087	0.383654016	1.06909369	0.285027455	0.814209842
C3orf33	62.18648928	0.289378559	0.270722193	1.068913325	0.285108727	0.814209842
DNAJC6	135.9861332	0.287278838	0.268767391	1.068875347	0.285125842	0.814209842
LRCH1	547.656842	0.134195214	0.125556725	1.068801485	0.28515913	0.814209842
DNALI1	11.03126004	0.920536487	0.861431185	1.068612912	0.285244128	0.814209842
BPTF	1817.272588	-0.091557403	0.085679351	-1.068605233	0.28524759	0.814209842
LINC01398	2.694654059	-1.599561161	1.497491307	-1.068160565	0.28544809	0.814614392
MED18	207.9522656	0.179984354	0.16851788	1.068043074	0.285501082	0.814614392
KRTCAP2	377.2486399	-0.165974622	0.155409884	-1.067979832	0.285529609	0.814614392
TCAF2	24.09241075	0.480360258	0.449823756	1.067885482	0.285572172	0.814614392
RAB41	11.06600129	0.577356666	0.540786248	1.067624536	0.285689911	0.814819818
OGFOD1	1022.219449	-0.112841491	0.105715494	-1.067407315	0.285787947	0.814929551
PICK1	25.72927998	-0.487356729	0.456610137	-1.067336641	0.285819848	0.814929551
MYO1E	1222.981825	-0.16077664	0.150655393	-1.067181445	0.28588991	0.81499891
TAS1R1	3.06722337	1.233914225	1.156512472	1.066926864	0.286004863	0.815102753
BFSP1	13.38117018	-0.580057695	0.543724348	-1.066823101	0.286051725	0.815102753
ZBTB7C	9.599347345	-0.874318003	0.819651942	-1.066694237	0.28610993	0.815102753
NNT-AS1	236.8866339	-0.256330881	0.240317931	-1.066632354	0.286137885	0.815102753
LOC101929592	10.11222171	0.713589573	0.669035566	1.066594377	0.286155041	0.815102753
SPRTN	384.872118	0.124465644	0.116737932	1.066197098	0.286334554	0.815483738
LOC107985939	22.75851776	0.484424913	0.454436131	1.065991192	0.286427624	0.815618449
RNF166	193.8989129	0.169165809	0.158727269	1.065764	0.286530339	0.815723826
ADGRF3	16.80911548	0.646859207	0.607001684	1.065662952	0.286576031	0.815723826
SNORD114-17	8.201391261	-0.87806837	0.824008763	-1.065605622	0.286601957	0.815723826
HEXB	965.5112575	-0.226491231	0.212645408	-1.065112262	0.286825131	0.815876001
ADGRF5	2.71538608	1.532454388	1.438816408	1.065079867	0.286839789	0.815876001
LINC02301	17.36678879	0.530238677	0.497934947	1.064875401	0.286932318	0.815876001
TADA3	536.5275044	-0.185978105	0.174649333	-1.06486582	0.286936654	0.815876001
PDIA2	6.2918171	-0.844541596	0.793101195	-1.06485982	0.28693937	0.815876001
RAET1K	5.647627675	1.062757591	0.998037082	1.0648478	0.28694481	0.815876001
HSD11B2	6.364602626	0.865907235	0.813364914	1.064598706	0.287057566	0.815876001
HSPB1	58.16269986	-0.399546533	0.375394068	-1.064338963	0.287175173	0.815876001
NBPF26	200.6405946	0.27150207	0.255111873	1.064247095	0.287216777	0.815876001
BAALC-AS1	25.71313388	-0.426723836	0.401001111	-1.064146269	0.287262443	0.815876001
PTPRD-AS1	19.09842421	0.516641077	0.48550845	1.06412376	0.287272639	0.815876001
TRIM36	52.31829215	-0.352563843	0.331323336	-1.064108092	0.287279736	0.815876001
CD164	4356.01013	-0.111495498	0.104788951	-1.064000519	0.287328464	0.815876001
PNISR	1420.086431	0.287483037	0.270234147	1.063829427	0.287405978	0.815876001
LIVAR	7.135031325	0.81342328	0.764682586	1.063739772	0.287446602	0.815876001
CROCC	275.4281577	-0.164298356	0.154462774	-1.063676069	0.287475469	0.815876001
EYA4	554.3975563	-0.133140043	0.125192782	-1.063480193	0.287564243	0.815876001
MYOM3	2.239232411	1.461284382	1.374251978	1.063330747	0.287631986	0.815876001
ZDHHC23	75.29691802	0.269870381	0.253819905	1.063235686	0.287675083	0.815876001
SGSM3	699.374526	0.151515899	0.142512368	1.063177192	0.287701604	0.815876001
ZDHHC9	448.8777992	-0.210186261	0.197711771	-1.063094324	0.287739178	0.815876001
WDFY3-AS2	64.04940965	-0.296373506	0.278795825	-1.063048578	0.287759922	0.815876001
PDP2	499.3256508	0.198519053	0.186748218	1.063030509	0.287768116	0.815876001
FRS2	666.593624	0.118934355	0.111892984	1.06292951	0.287813919	0.815876001
ZFX-AS1	3.007252042	1.545759843	1.454435155	1.062790484	0.287876976	0.815876001
BACH1-IT2	6.405265781	1.133130868	1.066291004	1.062684449	0.287925076	0.815876001
NRM	428.7991251	-0.14661533	0.137984731	-1.062547493	0.287987209	0.815876001
RBFOX2	5225.461246	-0.203341131	0.19138472	-1.062473172	0.288020931	0.815876001
NAA20	447.6815558	-0.142374374	0.134003289	-1.062469249	0.288022711	0.815876001
CHCHD4	120.022296	0.207773225	0.19558977	1.062290857	0.288103664	0.815876001
PIN4	43.53411795	-0.388051117	0.365297878	-1.062286809	0.288105501	0.815876001
OSER1	183.1388195	-0.238840866	0.224843522	-1.062253715	0.28812052	0.815876001
HNRNPH2	73.78365487	-0.274224587	0.25828168	-1.061726822	0.288359719	0.815969417
PKD2L2	16.22594538	0.740348375	0.697321442	1.061703155	0.288370467	0.815969417
GRIA4	41.09040451	0.48396785	0.455952959	1.061442503	0.28848885	0.815969417
SURF2	79.27505436	-0.279984507	0.263786397	-1.06140616	0.288505359	0.815969417
MTRNR2L3	12.83603547	0.732516222	0.690183225	1.061335883	0.288537284	0.815969417

ZNF395	1105.483665	-0.147469047	0.138966639	-1.061183083	0.288606706	0.815969417
ACTL10	93.08226336	0.446040707	0.420324757	1.061181144	0.288607587	0.815969417
CREBBP	1351.315402	-0.139576455	0.131537492	-1.06111537	0.288637474	0.815969417
POLD2	1185.96407	-0.122260094	0.115219566	-1.061105318	0.288642041	0.815969417
ARHGEF40	1431.219976	-0.11720204	0.110455722	-1.061077129	0.288654851	0.815969417
INO80B-WBP1	21.58924068	-0.467695471	0.440776388	-1.061071972	0.288657194	0.815969417
MED15	721.1389238	0.134052107	0.126388887	1.060632072	0.288857141	0.816375615
TJP1	3133.454816	0.138290318	0.130394382	1.060554267	0.288892516	0.816375615
IGHMBP2	375.4623002	0.159401117	0.150326162	1.060368438	0.288977015	0.816484923
USP10	1049.337778	-0.131523282	0.124070513	-1.060068812	0.289113295	0.816498877
PIP5KL1	45.78891085	0.358452366	0.338141475	1.060066253	0.289114459	0.816498877
SH3BP5-AS1	70.99441583	0.395613918	0.373201184	1.060055367	0.289119412	0.816498877
MAPK1	2470.119962	-0.115466908	0.108977448	-1.05954865	0.289349985	0.816665637
TMOD1	2.790942255	1.365425293	1.289068998	1.059233676	0.289493372	0.816665637
CNP	1069.064847	-0.119507229	0.112826155	-1.059215649	0.28950158	0.816665637
LOC731157	7.285649535	-0.877092448	0.828068736	-1.059202466	0.289507582	0.816665637
UTRN	15987.54168	0.161337386	0.152335929	1.059089521	0.289559012	0.816665637
MEI1	9.809498898	0.704458045	0.665183761	1.059042759	0.289580307	0.816665637
UXT	419.0697233	-0.203780834	0.192449614	-1.058878892	0.289654939	0.816665637
STRADA	379.8967611	0.146596035	0.138450898	1.058830511	0.289676976	0.816665637
PLEKHF1	149.9630918	-0.420695871	0.397403596	-1.058611132	0.289776915	0.816665637
LOC642648	38.84479101	0.564301031	0.533060503	1.05860597	0.289779267	0.816665637
LIMA1	1084.754139	-0.180724064	0.170728656	-1.058545581	0.289806782	0.816665637
CDC27	2503.936079	-0.088595245	0.083699831	-1.058487737	0.289833138	0.816665637
CTPS1	1363.248949	0.188425952	0.178023699	1.058431843	0.289858608	0.816665637
BCL2L12	292.6811901	0.161798619	0.152883804	1.058311052	0.289913656	0.816665637
MZF1	208.3984964	0.237643368	0.224585387	1.058142613	0.28999043	0.816665637
ZNF429	13.40565261	0.702828029	0.664236249	1.058099479	0.290010092	0.816665637
PSEN1	1299.962048	-0.147465017	0.139374171	-1.058051253	0.290032077	0.816665637
OXNAD1	197.0513982	0.231934235	0.21921397	1.058026707	0.290043267	0.816665637
C22orf34	10.91821549	0.684237458	0.646718911	1.058013684	0.290049204	0.816665637
COL18A1	556.9623098	0.202869656	0.191771494	1.057871804	0.290113891	0.816718728
LOC105378330	4.825115623	1.520777617	1.437781561	1.05772508	0.290180798	0.816778047
POTEKP	110.4143615	-1.360529868	1.286784896	-1.057309479	0.290370369	0.817064977
NKTR	1050.705142	0.267211738	0.252730159	1.057300558	0.290374439	0.817064977
AP1M1	793.6906973	-0.156155766	0.14774093	-1.056956702	0.290531349	0.817306683
MC1R	61.42258657	0.276950891	0.262037968	1.056911308	0.290552067	0.817306683
SNORD91A	6.192969695	0.951008691	0.900071338	1.056592573	0.290697572	0.81758692
MCOLN3	116.6308882	-0.287041771	0.27175433	-1.056254639	0.290851894	0.817692249
RAB11FIP4	401.2464812	-0.264307532	0.25023351	-1.056243554	0.290856957	0.817692249
WIZ	666.649961	0.104088953	0.098561751	1.056078569	0.290932321	0.817692249
OR1E2	3.895122836	1.16597924	1.104192232	1.05595675	0.290987975	0.817692249
MPG	175.6160913	-0.232784737	0.220452747	-1.055939379	0.290995912	0.817692249
C16orf70	303.3612573	0.144031604	0.136429619	1.055720932	0.291095731	0.817692249
YPEL4	50.03625309	0.437446078	0.414413092	1.055579775	0.291160245	0.817692249
MAPK12	825.6957169	0.207800205	0.196878143	1.055476257	0.291207563	0.817692249
LOC727896	19.50686477	0.704058887	0.667208635	1.055230477	0.291319928	0.817692249
GCNT4	15.75589243	0.640400614	0.606965975	1.055084865	0.291386513	0.817692249
POGZ	2008.220768	0.102539051	0.097193081	1.055003611	0.291423673	0.817692249
HSD17B11	743.7104461	-0.205209288	0.194514023	-1.054984545	0.291432393	0.817692249
UCHL3	237.1877287	0.22651006	0.214717877	1.054919426	0.291462177	0.817692249
DGUOK-AS1	10.88252284	0.766542709	0.726837023	1.054628046	0.291595472	0.817692249
AMIGO1	22.94545107	0.496140788	0.470491051	1.05451695	0.291646304	0.817692249
LINC00612	5.128110616	0.890158788	0.844185502	1.054458749	0.291672937	0.817692249
ALPK2	7.229086092	-0.747041258	0.708461503	-1.054455682	0.291674341	0.817692249
FBLN2	453.4167971	0.209128191	0.198344983	1.054365922	0.291715418	0.817692249
JMJD4	552.9379635	0.237730181	0.225475585	1.054349994	0.291722708	0.817692249
GSDME	332.6283486	-0.242871527	0.23035462	-1.054337559	0.291728399	0.817692249
PI4K2B	570.1625926	-0.14925996	0.141571997	-1.054304266	0.291743636	0.817692249
TRAF4	508.3569034	0.233515303	0.221487987	1.054302338	0.291744518	0.817692249
ATP13A3	7307.411391	0.130665756	0.123986463	1.053871146	0.291941915	0.81811683
LIMS1	763.9322062	-0.140155365	0.133033665	-1.053533065	0.292096748	0.81812305
SLC25A17	344.0964728	0.178991626	0.169924797	1.053357896	0.292176993	0.81812305
BLM	549.4076749	0.218257119	0.207201301	1.053357863	0.292177008	0.81812305
TMEM170A	472.5677415	0.121220844	0.115114082	1.053049649	0.292318238	0.81812305
ELOVL7	15.69869618	0.683918059	0.649523729	1.052953153	0.292362463	0.81812305
LRRC71	7.716323554	0.810526335	0.769822184	1.052874745	0.292398402	0.81812305
BCLAF1	2722.179938	-0.224736118	0.213459509	-1.052827862	0.292419893	0.81812305
LOC613206	26.79248416	0.465196701	0.441893176	1.052735651	0.292462164	0.81812305
GNPTAB	1523.67227	0.106211361	0.100901564	1.05262354	0.292513564	0.81812305
BGLAP	3.608656669	1.214648756	1.153994372	1.052560381	0.292542523	0.81812305
RPP38-DT	2.782579044	1.443251723	1.371450655	1.052354102	0.292637118	0.81812305
DECR2	230.5189431	-0.148519592	0.141148584	-1.052221628	0.292697879	0.81812305
MMS22L	1196.468421	0.137406718	0.130601679	1.052105298	0.292751242	0.81812305
OARD1	289.9961199	0.16588757	0.15767323	1.05209724	0.292754938	0.81812305
PGM5P2	14.97774763	0.611839238	0.581577537	1.052033819	0.292784034	0.81812305
GLRB	273.2956287	0.256715051	0.244057113	1.051864654	0.292861651	0.81812305
HNRNPA1P10	30.94112627	-0.399289531	0.379633833	-1.051775412	0.292902603	0.81812305
RNU4-2	13.90190413	-0.647306451	0.615572124	-1.051552573	0.293004877	0.81812305
TRIM35	772.2404189	-0.130813004	0.124460086	-1.051043816	0.293238466	0.81812305
CTSS	54.80444861	0.585448376	0.557027493	1.051022405	0.2932483	0.81812305
MIR4434	4.225563175	0.934542904	0.889182802	1.051013247	0.293252506	0.81812305
RPSAP58	71.06049182	-0.243639879	0.231831149	-1.050936772	0.29328763	0.81812305
RTL6	1347.957658	-0.106950421	0.101769841	-1.050904865	0.293302286	0.81812305
TBP	190.4543315	0.174157461	0.165725191	1.050881037	0.293313231	0.81812305
RFX4	3.382764539	1.20533651	1.146996828	1.050862985	0.293321523	0.81812305
EEF1G	9834.005895	-0.223960044	0.213123916	-1.050844263	0.293330123	0.81812305
SENP7	388.0784555	0.168000531	0.159872738	1.050839147	0.293332473	0.81812305
DUSP10	437.179963	-0.351738071	0.334735341	-1.050794549	0.29335296	0.81812305
MDN1	5071.722921	0.234852193	0.223510443	1.050743715	0.293376313	0.81812305

TERT	324.496239	0.696425349	0.662846304	1.05065887	0.293415293	0.81812305
C3orf35	13.29033294	0.665388665	0.6333237	1.050629662	0.293428712	0.81812305
LINC00871	4.488580922	1.240186626	1.18051977	1.05054287	0.293468592	0.81812305
VPS16	430.4516627	-0.124998897	0.119003068	-1.050383819	0.293541682	0.81812305
PSME3	2146.992193	-0.178541352	0.169983552	-1.050344869	0.293559583	0.81812305
ANKRD13A	1563.110342	0.133215863	0.126831934	1.050333764	0.293564687	0.81812305
XG	2.136499068	-1.925528339	1.833994896	-1.049909323	0.293759805	0.81812305
AGAP2-AS1	147.741175	-0.199443508	0.189968904	-1.049874499	0.293775818	0.81812305
MIR1276	2.473819911	2.143122883	2.041398887	1.049830534	0.293796034	0.81812305
MFSD3	758.4765572	0.117229746	0.11167161	1.049772148	0.293822884	0.81812305
LEAP2	11.59372206	0.812506953	0.773988219	1.049766564	0.293825451	0.81812305
ABCC2	20.08024344	0.48093082	0.458208907	1.049588545	0.293907326	0.81812305
RPP14	140.5925264	0.209778863	0.199897986	1.049429597	0.293980442	0.81812305
ITGA6	157.1653842	-0.443847336	0.423078942	-1.049088698	0.294137297	0.81812305
CAV2	555.9883219	0.239530314	0.228327001	1.049066963	0.2941473	0.81812305
PSPC1	655.3693452	-0.268372867	0.255822952	-1.049057034	0.29415187	0.81812305
ZNF93	45.31644803	0.383733706	0.365816422	1.048978897	0.294187831	0.81812305
PGM3	1341.374465	0.174364628	0.166226331	1.048959132	0.294196928	0.81812305
NUP50-DT	125.4656286	0.246687031	0.235184247	1.048909669	0.294219695	0.81812305
CCDC102A	29.61696241	-0.39236371	0.374098912	-1.048823446	0.294259385	0.81812305
SNORD42A	16.66753132	-0.511572531	0.487765111	-1.048809191	0.294265947	0.81812305
LOC100287944	9.904041223	0.916947072	0.874348075	1.048720868	0.294306607	0.81812305
ALKBH3	204.8904825	-0.159602196	0.152195328	-1.048666854	0.294331475	0.81812305
SCN2B	2.218238042	-1.306002047	1.245847719	-1.048283853	0.294507847	0.818188723
PLA2G12A	374.4536599	0.177967419	0.169773077	1.04826644	0.294515867	0.818188723
RNF123	593.4481569	0.161846131	0.154402704	1.048207881	0.29454284	0.818188723
ADAM23	293.0998467	-0.135969689	0.129723835	-1.048147307	0.294570744	0.818188723
ANKRD31	6.911542084	-0.807259602	0.770199841	-1.048117073	0.294584671	0.818188723
LINC01585	3.142981941	1.207993325	1.153157558	1.047552711	0.294844735	0.818671396
GPC6-AS2	3.017947289	1.399237834	1.336056324	1.047289556	0.294966052	0.818671396
PHACTR1	20.36371565	0.59201358	0.565312855	1.047231768	0.294992697	0.818671396
MIR3975	3.923476649	1.423708221	1.359801648	1.046996982	0.29510097	0.818671396
MIRLET7A2	15.95336528	0.651156988	0.622013485	1.046853491	0.295167155	0.818671396
SMIM10L2A	12.95638151	-0.595092835	0.568532906	-1.046716609	0.295230301	0.818671396
LOC101928438	4.373717507	1.151010379	1.099658157	1.04669835	0.295238725	0.818671396
FOXA3	13.81391079	-0.658610193	0.629265195	-1.046633753	0.295268528	0.818671396
GLRX2	110.8232411	-0.246896636	0.235908874	-1.046576296	0.295295039	0.818671396
TDP2	679.3336264	-0.136095658	0.130041797	-1.04655319	0.295305701	0.818671396
IRF7	84.47840809	0.528026223	0.504541181	1.046547325	0.295308407	0.818671396
C6orf141	123.4980292	0.387558968	0.370322522	1.046544416	0.295309749	0.818671396
LOC101927429	3.368012874	1.087401915	1.039152495	1.04643151	0.295361851	0.818688472
P2RX4	134.7550722	-0.285495415	0.2728663	-1.046283162	0.295430317	0.81869927
NOB1	801.351957	-0.144595357	0.138206884	-1.046223984	0.295457632	0.81869927
OAS3	615.2133701	-0.248110617	0.237280232	-1.045643859	0.295725492	0.819075711
CHTF18	674.2610343	0.360802431	0.345054928	1.045637668	0.295728351	0.819075711
LOC101927124	17.5216382	0.616442537	0.589571748	1.045576792	0.295756469	0.819075711
ROCK2	2769.528625	-0.087324774	0.083531133	-1.045415895	0.295830793	0.819075711
ASTE1	159.695078	0.205656926	0.196731009	1.045371175	0.295851454	0.819075711
MSTN	3.537959739	1.178816366	1.127695	1.045332617	0.295869268	0.819075711
CDYL	664.8640947	0.155614691	0.148915728	1.044984922	0.296029938	0.819323247
DPY19L2P3	4.394764122	1.146682774	1.097484819	1.044827914	0.296102511	0.819323247
SLC35G2	134.5625464	0.231571605	0.221655853	1.044734895	0.296145512	0.819323247
ZSCAN12	541.472921	0.253179357	0.242342363	1.044717704	0.29615346	0.819323247
SAMD1	753.8544052	0.152429247	0.145925399	1.044569674	0.296221902	0.819323247
CBR4	191.0367231	-0.168449801	0.161266612	-1.04454232	0.29623455	0.819323247
LOC101928504	22.10950178	-0.558343721	0.534759414	-1.044102649	0.2964379	0.81975844
AMIGO3	116.8478414	0.289013682	0.276874371	1.043844114	0.296557518	0.819891985
GRIA3	15.14001615	-0.57297251	0.548929721	-1.043799394	0.296578212	0.819891985
GRAMD4	603.7709049	0.191886358	0.183855386	1.043680918	0.296633041	0.819916363
LINC01232	14.24101333	-0.740471831	0.709880437	-1.043093727	0.296904886	0.820540487
TFAP2E	35.34111432	0.456290755	0.437532943	1.042871772	0.297007685	0.820573139
UQCR10	235.2476074	0.190702094	0.182862877	1.042869373	0.297008796	0.820573139
FLT1	14.04797246	-0.970367627	0.930835735	-1.042469246	0.297194177	0.820958026
BDNF-AS	30.09763954	0.456889764	0.438369213	1.042248751	0.297296366	0.821113026
LOC440084	3.773261573	1.188100538	1.140446998	1.041784967	0.297511386	0.821493071
PHKA1	503.3920316	0.201530472	0.193453203	1.041753094	0.297526166	0.821493071
EMG1	744.096472	0.163048494	0.15653508	1.041609932	0.297592562	0.821549102
CEND1	13.84662626	0.564252987	0.541790093	1.04146051	0.297661872	0.821613159
GAA	669.441692	-0.231141919	0.22200126	-1.041173908	0.297794843	0.821735544
CFL1P1	8.158162903	0.710074774	0.682024624	1.041127767	0.297816254	0.821735544
ANGPTL7	13.20607867	0.774690293	0.744131211	1.041066793	0.29784455	0.821735544
LYSMD4	252.3952769	0.226068659	0.217225576	1.040709215	0.298010525	0.821903916
SLC35E1	1115.112064	0.199108348	0.191335987	1.040621533	0.298051233	0.821903916
TRPC4	22.68353341	0.696297781	0.669199382	1.04049376	0.298110562	0.821903916
NRXN3	2.367193906	1.574491338	1.513291665	1.040441426	0.298134864	0.821903916
NCKIPSD	256.0077311	0.184576961	0.17740895	1.040403889	0.298152296	0.821903916
NEXN	63.03830328	0.600307356	0.577119968	1.04017776	0.298257322	0.821903916
MRPS36	81.96772435	-0.227245054	0.218489351	-1.040073816	0.298305607	0.821903916
MIR4779	2.066338233	1.589389526	1.52836136	1.039930456	0.298372212	0.821903916
PYM1	69.6512252	-0.263219992	0.253125557	-1.039879159	0.298396046	0.821903916
ARHGEF11	1120.378077	0.124122339	0.119363156	1.039871462	0.298399623	0.821903916
MIR3606	6.273464508	1.034681191	0.99503853	1.039840327	0.29841409	0.821903916
C1QTNF3-AMACR	3.263102571	1.274734542	1.226008574	1.039743579	0.298459048	0.821903916
ISL2	139.5409076	-0.334176728	0.321506371	-1.039409349	0.298614398	0.822030148
ZBED5	753.363015	0.147283576	0.141700143	1.039403158	0.298617276	0.822030148
MIR589	9.169992126	0.816033982	0.785140863	1.039347231	0.298643276	0.822030148
ZNF730	5.783077456	-0.813172585	0.782591285	-1.039076974	0.298768939	0.822249033
KCNMA1-AS1	2.433892665	1.605120355	1.545104633	1.038842497	0.298877994	0.82242215
DNAJA3	1044.166875	-0.13582898	0.130790914	-1.038519996	0.299028031	0.822707968
TMEM165	637.1779189	-0.133778338	0.128838974	-1.038337498	0.299112957	0.822814587

F2RL1	417.2187126	0.154059095	0.148395955	1.038162361	0.299194472	0.822911792
ARHGEF10L	20.25898917	0.455053058	0.438375841	1.038043193	0.299249946	0.822937353
CHRNA1	3.967758427	-0.987301829	0.951306301	-1.037838	0.299345482	0.822968563
MAB21L1	44.71378041	0.320863991	0.309232294	1.037614754	0.299449446	0.822968563
SGMS1	228.2722726	-0.177785761	0.171354328	-1.037532948	0.299487549	0.822968563
STX3	178.8402738	0.237811255	0.229208741	1.037531357	0.29948829	0.822968563
SPHK2	293.9249622	0.169360639	0.163248035	1.037443665	0.299529138	0.822968563
TMEM139	7.301650185	0.744071707	0.717230223	1.037423804	0.299538389	0.822968563
MIR4517	4.641496815	0.980819408	0.945638367	1.037203484	0.299641035	0.823123669
PTGER4P2-CDK2AP2P2	4.068791365	1.209319801	1.166320324	1.036867639	0.299797547	0.823426679
APPL2	705.4262814	0.161329285	0.155651423	1.036478063	0.299979169	0.823684383
TP53I13	428.8860581	0.118825126	0.114644269	1.03646809	0.299983819	0.823684383
ENPP2	843.5026604	-0.531168657	0.512541154	-1.03634343	0.300041952	0.823686527
PGD	1626.991548	-0.138787086	0.133929699	-1.036268184	0.300077045	0.823686527
CXCL12	16.85865775	-0.520878441	0.50272629	-1.036107423	0.300152029	0.823755593
SLC2A4RG	398.0070387	-0.141106005	0.136200598	-1.036016041	0.300194659	0.823755593
APOL6	161.5150005	-0.424224323	0.409565664	-1.035790741	0.300299778	0.823808139
PEX11A	113.8374883	0.236538398	0.22836811	1.035776836	0.300306267	0.823808139
SNORD124	5.483634306	0.895038878	0.864707027	1.035077604	0.300632671	0.824297806
ARL6	144.434991	0.243963475	0.235720146	1.03497083	0.300682534	0.824297806
TRAK2	1443.530356	0.12644562	0.122197926	1.03476077	0.300780648	0.824297806
AHCYL2	278.6019481	0.249917263	0.241546804	1.034653572	0.300830725	0.824297806
OCRL	679.9464728	0.152964249	0.147843044	1.034639474	0.300837312	0.824297806
GPBAR1	4.719824512	0.871496621	0.842371183	1.03457554	0.300867182	0.824297806
COX5B	941.7835379	-0.143091767	0.138313566	-1.034546151	0.300880913	0.824297806
GYS2	2.741701612	-1.350140424	1.305084144	-1.034523659	0.300891422	0.824297806
MFHAS1	1709.995173	0.185462529	0.179276938	1.034502992	0.300901079	0.824297806
RPL22L1	685.5425319	0.207508256	0.200674523	1.034053812	0.301111007	0.824746103
FARP1	875.7416849	-0.121971312	0.11802565	-1.033430548	0.301402457	0.825020315
TMEM43	2261.022192	-0.128102094	0.123959903	-1.033415571	0.301409463	0.825020315
C3AR1	30.90334776	-0.44769033	0.433308841	-1.033189926	0.301515026	0.825020315
KCNT2	349.2128796	-0.178352098	0.172625918	-1.033171033	0.301523866	0.825020315
UQCR11	280.2561676	0.13786529	0.133455663	1.03304189	0.301584296	0.825020315
MIR210HG	45.32840805	0.420729195	0.407277825	1.033027503	0.301591028	0.825020315
MAN1C1	2.333524996	2.046486502	1.981312208	1.03289451	0.301653268	0.825020315
WDR18	409.8359464	-0.189691073	0.183652851	-1.032878453	0.301660783	0.825020315
HTRA1	1316.867914	0.35535876	0.344055636	1.032852605	0.301672881	0.825020315
PLAAT1	84.67837062	0.267584044	0.259129292	1.032627539	0.301778235	0.825020315
KIF1A	5.44512462	-0.868254848	0.840858235	-1.032581726	0.301799683	0.825020315
SLC6A13	2.293406664	1.743472023	1.688515894	1.032547001	0.301815941	0.825020315
DDX53	9.72630391	-0.656017193	0.635484622	-1.0323101	0.301926871	0.825020315
FEZF1-AS1	134.7908986	0.268506078	0.260102381	1.03230919	0.301927297	0.825020315
RPL23AP82	85.24129773	-0.218561933	0.211724836	-1.032292372	0.301935174	0.825020315
PIGN	328.7613831	0.156211884	0.15134436	1.032161914	0.301996273	0.825020315
MANEA	404.9841355	0.135901854	0.131681716	1.032048017	0.302049624	0.825020315
C1QL4	62.45795942	-0.297294457	0.288132377	-1.031798164	0.30216668	0.825020315
RN7SL2	31295.54803	-0.475682995	0.461047523	-1.031743955	0.302192081	0.825020315
AGXT2	4.328885239	-1.087326345	1.053899134	-1.031717656	0.302204404	0.825020315
TRPT1	60.41963593	0.287648133	0.278819871	1.031662956	0.302230037	0.825020315
GNA14	15.15213705	0.490204538	0.475169058	1.03164238	0.302239679	0.825020315
ZC3H12A	85.77366511	0.236878306	0.229630042	1.031564962	0.302275962	0.825020315
KYNU	95.53070864	-0.239530167	0.232289193	-1.031172239	0.302460057	0.825058794
CCND2-AS1	6.930607185	0.913436964	0.885877195	1.031110146	0.302489171	0.825058794
ZNF275	263.0075133	0.216175381	0.209662488	1.031063701	0.302510948	0.825058794
CLK4	256.01669	0.284124438	0.275581947	1.030998009	0.302541754	0.825058794
CBX5	7491.387698	-0.161203256	0.156370937	-1.030902925	0.302586345	0.825058794
ZNF529-AS1	49.76651808	0.379731797	0.368361204	1.030868052	0.3026027	0.825058794
PTPRA	953.7880777	-0.149825461	0.145349953	-1.030791258	0.302638718	0.825058794
GOLGA3	6475.742114	0.187522617	0.181934852	1.030712999	0.302675427	0.825058794
NSD2	2419.846226	0.080524834	0.078141882	1.030495202	0.302777603	0.825058794
COL4A6	42.51546582	-0.381635131	0.370361935	-1.03043832	0.302804292	0.825058794
SNORA5C	81.09553613	-0.33103547	0.321260599	-1.03042661	0.302809787	0.825058794
TIAM2	148.4297768	0.246971208	0.239696382	1.030350172	0.302845655	0.825058794
NRTN	10.47689264	0.72980407	0.708378612	1.030245772	0.302894648	0.825065939
MIR6768	1.755592486	2.007115777	1.948511094	1.030076649	0.302974026	0.825065939
SPATA25	33.13394092	0.485038802	0.470889225	1.03004863	0.302987178	0.825065939
FHIT	68.6479445	0.2943607	0.285813662	1.02990423	0.303054965	0.825124443
DCLRE1A	296.9738562	-0.16171138	0.157033583	-1.029788514	0.303109294	0.825133188
FGF10	12.33978339	-0.622249456	0.604307586	-1.029689963	0.303155569	0.825133188
ANXA2P1	9.182623127	0.736530945	0.71535532	1.029601547	0.303197089	0.825133188
FBXO28	803.0465329	0.132355776	0.128577017	1.02938907	0.303296883	0.825212681
ZNF593	157.806279	-0.219914567	0.213653545	-1.029304555	0.303336583	0.825212681
GPR108	510.9977124	-0.172929966	0.168016558	-1.029243591	0.303365223	0.825212681
PA2G4	1500.420031	-0.127668998	0.124082873	-1.028901049	0.303526175	0.825524488
C20orf96	77.96849031	0.318999191	0.310143742	1.028552723	0.303689904	0.825843749
CDC42EP5	24.70884215	-0.466200739	0.453420046	-1.028187315	0.303861724	0.82593491
N4BP2L2-IT2	75.02396222	0.585738974	0.569720489	1.028116393	0.30389508	0.82593491
MPEG1	3.751352142	1.098089816	1.068200273	1.027981216	0.303958664	0.82593491
CDC25C	149.530487	0.179207022	0.174352541	1.027842903	0.304023731	0.82593491
BRPF3	607.2227818	0.143566062	0.139679473	1.027825058	0.304032127	0.82593491
EHD4-AS1	8.651310935	0.812217239	0.790245033	1.027804296	0.304041895	0.82593491
ELF1	779.7059386	-0.146575647	0.142612225	-1.027791601	0.304047868	0.82593491
BDKRB2	67.63823987	1.094461065	1.065020342	1.027643343	0.304117628	0.825998495
TNFRSF11A	46.15910123	-0.297003607	0.289128664	-1.027236812	0.304308968	0.826392229
DOCK4	1160.933322	0.182002814	0.177215696	1.027012947	0.304414368	0.826512937
OR2B6	3.232645952	-1.25649339	1.22364649	-1.026843456	0.304494183	0.826512937
INTS6L	141.4164157	-0.239905436	0.233668313	-1.026692207	0.30456542	0.826512937
ADH4	4.090085568	1.421533708	1.38496378	1.026404971	0.304700736	0.826512937
FEZF1	41.13908974	0.49937251	0.486530469	1.026395143	0.304705367	0.826512937
DEXI	272.1506764	0.157334729	0.153301097	1.026311828	0.304744624	0.826512937

N4BP3	12.45534368	0.53100533	0.517404997	1.026285663	0.304756953	0.826512937
CYB5B	1013.184473	-0.118730141	0.11570202	-1.026171719	0.304810649	0.826512937
SRP14	577.5086168	-0.13179166	0.128440678	-1.026089727	0.304849292	0.826512937
DCPS	364.0699284	0.145157472	0.141467415	1.026084147	0.304851922	0.826512937
PLEKHB1	3.557906576	-1.008939409	0.983449824	-1.025918541	0.304929982	0.826512937
GREB1L	76.45720487	0.376691009	0.367195634	1.025859172	0.30495797	0.826512937
RASL11B	34.59254672	-0.446683664	0.435456616	-1.025782241	0.304994239	0.826512937
CSAG4	7.499668235	0.853369879	0.832014585	1.025666971	0.305048588	0.826512937
NFE4	46.98190984	-0.293486126	0.286167986	-1.025572883	0.305092955	0.826512937
ACTA2-AS1	23.40345639	0.624628381	0.6090932	1.025505426	0.305124767	0.826512937
MRS2P2	16.29253617	0.80617135	0.786186347	1.025420185	0.305164968	0.826512937
GPR153	22.04706953	-0.551428965	0.537784951	-1.025370762	0.305188279	0.826512937
COMMD7	137.0025811	-0.292395841	0.285218853	-1.025163094	0.305286239	0.826652602
SERPINB7	2.923992634	1.774952521	1.731882656	1.024868813	0.305425092	0.826698203
SLC51A	22.35237359	-0.406868214	0.397028226	-1.024784101	0.30546507	0.826698203
C12orf73	113.3457367	0.228404649	0.222892099	1.02473192	0.305489698	0.826698203
CEP170	1824.639064	0.099472388	0.097073972	1.024707096	0.305501414	0.826698203
COL18A1-AS1	9.679890432	0.850485694	0.830102588	1.024554923	0.305573243	0.826698203
CHD3	4294.653924	-0.076819855	0.07498542	-1.024463891	0.305616218	0.826698203
SEPHS2	869.3149338	-0.132615511	0.129452049	-1.024437334	0.305628756	0.826698203
CTSO	55.72255858	-0.357260471	0.348799339	-1.024257878	0.305713488	0.826698203
STK32B	21.17144065	-0.452906008	0.442211573	-1.024183979	0.305748385	0.826698203
RBM44	19.43948182	0.488848979	0.477324191	1.02414457	0.305766995	0.826698203
ARL5B	842.3582749	0.133774744	0.130719623	1.023371557	0.306132202	0.827557015
BCHE	937.9771224	-0.144756015	0.141467076	-1.023248788	0.306190231	0.827557015
TMEM121	33.0869251	-0.356806417	0.348723891	-1.023177436	0.306223959	0.827557015
SNORA32	78.19037846	0.439695594	0.429920754	1.022736376	0.306432507	0.827737675
ZNF718	234.1374774	0.209431046	0.204776684	1.022728961	0.306436014	0.827737675
CCDC51	170.6771821	-0.169829122	0.166071771	-1.022624859	0.306485251	0.827737675
RPS6KB1	1119.857782	-0.103518018	0.101235105	-1.022550606	0.306520374	0.827737675
TLE5	734.742737	-0.216910226	0.212148586	-1.022444838	0.306570408	0.827737675
TMEM183B	13.73488551	-0.533467228	0.521781183	-1.022396449	0.306593301	0.827737675
APOA1-AS	5.223623972	1.1991454	1.172932061	1.022348556	0.306615959	0.827737675
LINC01609	4.255190453	1.242068321	1.215313722	1.022014562	0.306774008	0.827999026
MYH4	13.51591153	0.735959148	0.720257408	1.021800179	0.306875485	0.827999026
CDK19	465.3017797	-0.251393023	0.24604164	-1.02174991	0.306899282	0.827999026
ZNF140	232.7144194	0.166040129	0.162516185	1.02168365	0.306930652	0.827999026
FBXO30	953.5661649	-0.117219931	0.114741616	-1.021599094	0.306970687	0.827999026
ZNF277	451.2780607	-0.293627499	0.2874319	-1.021555015	0.306991558	0.827999026
KIF22	912.9207708	-0.134386181	0.131593239	-1.021224059	0.307148296	0.828077313
STON1-GTF2A1L	6.309672797	0.797289286	0.780796708	1.021122755	0.307196284	0.828077313
IRF2BP1	393.0779166	-0.153238753	0.150070432	-1.021112225	0.307201272	0.828077313
ACOT13	305.7651253	-0.14467281	0.141683116	-1.021101273	0.30720646	0.828077313
FBXO3	490.5297828	0.129553947	0.126934703	1.020634578	0.307427601	0.828197135
CYLD	443.9658715	-0.157940087	0.154759883	-1.020549274	0.307468033	0.828197135
LAMB3	20.48286792	-0.589026307	0.577201675	-1.020486136	0.307497961	0.828197135
NPHP3	54.82650639	0.274186584	0.268690952	1.020453356	0.3075135	0.828197135
WDR41	890.1541299	-0.099532525	0.097545124	-1.020374173	0.307551038	0.828197135
LOC101927539	6.996529824	0.933553322	0.914964414	1.020316536	0.307578363	0.828197135
TSPAN10	61.32516518	-0.255022391	0.249963295	-1.020239353	0.307614958	0.828197135
SENP2	2448.256857	-0.131755894	0.129146652	-1.020203715	0.307631856	0.828197135
GALNT11	331.0139331	-0.132428069	0.129815534	-1.020124974	0.307669194	0.828197135
SOX21	58.52437438	-0.297575989	0.291789705	-1.019830322	0.30780894	0.828448166
SNX33	707.6571088	0.124097762	0.121731233	1.019440613	0.307993833	0.828820615
EARS2	1104.43636	0.101252497	0.099354888	1.019099303	0.308155825	0.82913133
TBX19	30.97326888	0.426353119	0.418490296	1.018788545	0.308303365	0.829403075
MYDGF	728.3659243	0.165637688	0.162643943	1.01840674	0.3084847	0.829765623
EDRF1-AS1	10.6081008	-0.709160475	0.696410086	-1.018308736	0.308531258	0.829765623
RPAP1	1065.610755	0.098994332	0.097229223	1.018154095	0.308604731	0.829837982
DICER1	4041.906256	0.112083275	0.110114088	1.017883161	0.308733485	0.829851434
SNORA6	15.41806227	0.688190562	0.676107561	1.017871417	0.308739067	0.829851434
RNF103	521.8940271	-0.126717444	0.124506902	-1.017754367	0.308794703	0.829851434
SAXO2	36.53129371	0.369408247	0.362965029	1.017751623	0.308796008	0.829851434
RPL19P12	3.129900088	-1.153611176	1.133943702	-1.017344312	0.308989664	0.830246653
MIR539	8.245268722	-0.891385782	0.87649189	-1.016992619	0.309156941	0.830519814
ZBTB9	333.7656332	0.152360369	0.149836708	1.016842736	0.309228248	0.830519814
KLC2	383.3054747	0.149012829	0.146545494	1.016836652	0.309231143	0.830519814
SPPL2A	2159.368197	-0.159536222	0.156917836	-1.016686354	0.309302659	0.830586707
MSANTD3	218.2878281	-0.230942485	0.227205329	-1.016448362	0.309415925	0.830765676
TUBB6	1918.394484	0.197038497	0.193944765	1.015951609	0.309652431	0.831015295
TMEM161B	290.9366013	0.221267607	0.217804138	1.015901759	0.309676171	0.831015295
LOC105372069	17.47076889	0.846601446	0.833585476	1.01561444	0.309813026	0.831015295
LTK	6.954452677	0.719599287	0.708606351	1.015513461	0.309861133	0.831015295
RAP2B	1829.554928	-0.115632659	0.113872642	-1.015456009	0.309888506	0.831015295
PCNX4	1663.294645	0.121751663	0.119922067	1.015256547	0.309983552	0.831015295
RRN3P1	75.94284628	0.320750259	0.315935821	1.015238659	0.309992077	0.831015295
GLE1	752.3449068	-0.129573166	0.127640906	-1.015138253	0.31003993	0.831015295
MYLK-AS2	3.033732918	1.42080476	1.399635183	1.015125068	0.310046214	0.831015295
SENCR	4.146171986	1.292980156	1.273800811	1.015056785	0.31007876	0.831015295
CLIP3	75.39866458	0.34233306	0.337319833	1.01486194	0.310171643	0.831015295
TRAF1	14.27529918	0.531587282	0.523852475	1.014765239	0.310217747	0.831015295
CCDC120	15.77274969	-0.539858237	0.532023798	-1.01472573	0.310236585	0.831015295
GPR89B	47.73096112	0.375916236	0.370526495	1.014546171	0.31032221	0.831015295
MAPK15	9.503649278	0.744064974	0.733420043	1.014514098	0.310337506	0.831015295
SETDB1	1125.573074	-0.096316401	0.0949513	-1.014376857	0.310402963	0.831015295
ANKMY1	195.9472837	0.348901463	0.343958064	1.014372099	0.310405233	0.831015295
LGSN	13.44494017	0.751235497	0.740652008	1.014289422	0.31044467	0.831015295
CCZ1B	84.4175042	0.291403911	0.287306534	1.014261345	0.310458064	0.831015295
MYRF-AS1	6.210012795	0.870031694	0.857934584	1.014100271	0.310534909	0.831015295
LINC00471	30.51633527	0.424468257	0.4186268	1.013953852	0.310604773	0.831015295

TYW5	258.5518992	0.232687187	0.229485146	1.01395315	0.310605108	0.831015295
FOXG1-AS1	5.039230211	1.121229054	1.105985457	1.013782819	0.310686395	0.831015295
CDCA3	144.4356352	0.22279248	0.219764417	1.013778677	0.310688372	0.831015295
PACS2	804.6140733	-0.125669409	0.123967698	-1.013727049	0.310713013	0.831015295
DAAM1	420.8279733	-0.126999355	0.1252818	-1.013709529	0.310721376	0.831015295
MICOS10	577.0564834	-0.126460128	0.12479495	-1.01334331	0.310896207	0.831260136
PTGFRN	3513.242237	0.137895942	0.136088208	1.013283547	0.310924744	0.831260136
ERMAP	206.8188033	-0.147779158	0.145850337	-1.013224657	0.310952866	0.831260136
NSMCE2	439.1423959	-0.171433156	0.169286099	-1.012683009	0.311211596	0.831352536
ATG16L1	440.1193798	0.208225634	0.205685854	1.01234786	0.311371759	0.831352536
KIAA0513	92.09988036	0.371506099	0.366995913	1.012289473	0.311399667	0.831352536
PSMB10	104.9250363	0.31631404	0.312479592	1.012271037	0.311408479	0.831352536
TAS2R30	14.0024092	0.925492112	0.914412843	1.012116266	0.311482466	0.831352536
PDP1	995.3356766	0.158919122	0.157026127	1.01205529	0.311511618	0.831352536
PPAN	235.3984075	-0.170275407	0.168252974	-1.012020196	0.311528397	0.831352536
NAALAD2	151.3108341	0.321941975	0.318125863	1.011995605	0.311540155	0.831352536
MGMT	64.75785929	-0.238628346	0.235819807	-1.01190968	0.31158124	0.831352536
NUTM1	4.307670713	1.043725145	1.031481369	1.011870089	0.311600172	0.831352536
ZBTB20-AS5	2.718366307	1.457347506	1.440258138	1.01186549	0.311602372	0.831352536
SSB	485.7546909	-0.182355786	0.180241458	-1.011730526	0.311666916	0.831352536
TK2	261.3077833	-0.312448686	0.308832291	-1.011709898	0.311676781	0.831352536
LMNB1-DT	2.373244407	-1.720983238	1.701065679	-1.011708872	0.311677272	0.831352536
PDGFRB	1385.672706	-0.314548266	0.310914279	-1.011688069	0.311687222	0.831352536
SORBS3	791.833377	-0.141998755	0.140442587	-1.011080461	0.311977921	0.83157047
CHD8	2734.94473	0.104234198	0.103100615	1.01099492	0.312018861	0.83157047
GALNT12	44.70484364	-0.440659826	0.435884665	-1.010955103	0.312037919	0.83157047
SARS2	333.5800843	0.161533685	0.159798442	1.010858949	0.312083944	0.83157047
VDAC3	1724.17904	-0.121763515	0.120483845	-1.010621098	0.312197814	0.83157047
RIPK1	335.6450702	-0.157701183	0.156061708	-1.010505299	0.312253262	0.83157047
LSM10	290.8496661	-0.178728698	0.176892339	-1.010381224	0.31231268	0.83157047
SUGT1P4-STRA6LP-CCDC180	8.819410451	0.982123776	0.972070863	1.010341749	0.312331585	0.83157047
LAMC1	6861.546604	-0.111511367	0.11038179	-1.010233365	0.312383498	0.83157047
CSNK1A1P1	25.17029369	0.746518626	0.7390357	1.010125256	0.312435283	0.83157047
DOK1	246.3646217	-0.151643678	0.150129626	-1.010084967	0.312454584	0.83157047
RPL21	4840.962185	-0.172593646	0.170874925	-1.010058356	0.312467332	0.83157047
BCL2	103.1967404	-0.242084279	0.239688439	-1.009995639	0.312497379	0.83157047
IGFBP4	757.3766885	-0.237321201	0.234975115	-1.009984402	0.312502763	0.83157047
NT5C2	366.2767722	-0.133065462	0.131750906	-1.009977584	0.31250603	0.83157047
TRIM45	209.7365597	0.18475356	0.182941716	1.009903941	0.312541314	0.83157047
LOC105371795	2.895300847	-1.177250101	1.165881557	-1.009751028	0.312614588	0.83157047
LOC102724708	16.62663403	0.562041987	0.556707322	1.009582532	0.312695342	0.83157047
MED16	773.0502642	-0.125104973	0.12392219	-1.009544566	0.312713539	0.83157047
PTPN1	983.8133112	0.161661476	0.160139166	1.009506168	0.312731945	0.83157047
RN7SL832P	6.619819442	0.841962169	0.834111089	1.009412511	0.312776841	0.83157047
BUB1B-PAK6	4.388476347	1.312460579	1.300291851	1.009358459	0.312802753	0.83157047
PRKCA	1428.203369	0.2222611	0.220218326	1.009276131	0.312842224	0.83157047
SPATA13	187.887938	0.219688474	0.217715305	1.009063071	0.312944387	0.831717969
POC1B-GALNT4	7.142752773	0.693691586	0.68762272	1.008825865	0.313058154	0.831896258
GAP43	9.914485177	-0.597836891	0.592677872	-1.008704592	0.313116329	0.83192679
MYO7B	5.982082249	1.029307381	1.02069368	1.008439067	0.313243726	0.831939102
MRPS12	353.443208	0.177249386	0.175773314	1.008397588	0.313263631	0.831939102
NPY1R	5.4649932	0.892671586	0.885357032	1.0082617	0.313328845	0.831939102
C6orf223	22.76909744	-0.634063179	0.62890369	-1.008203942	0.313356566	0.831939102
ACVR2A	306.8658464	-0.662201321	0.656917508	-1.008043343	0.313433655	0.831939102
TXLNA	2540.081614	-0.111819924	0.110932117	-1.008003159	0.313452946	0.831939102
HAND2-AS1	59.75305792	-0.36852064	0.365659366	-1.007824969	0.313538497	0.831939102
TRIM3	177.2281963	0.222528583	0.220832742	1.007679302	0.313608445	0.831939102
PTK2B	102.0975771	-0.299640012	0.297369066	-1.007636794	0.313628859	0.831939102
TGFBR2	671.071149	-0.173539454	0.172241141	-1.007537766	0.313676419	0.831939102
STAG3	9.860509378	-0.63416378	0.629535449	-1.007351979	0.313765659	0.831939102
KCNMB3	76.14064659	0.355062057	0.352476424	1.007335621	0.313773517	0.831939102
CNGA1	4.600587036	0.881602728	0.875386648	1.007100954	0.313886263	0.831939102
ZNF2	216.2639894	0.175520449	0.174284078	1.007094001	0.313889604	0.831939102
CENPC	263.9593265	0.191437263	0.190181767	1.006601559	0.314126285	0.831939102
ZNF296	34.33051326	0.367413709	0.36500477	1.006599747	0.314127156	0.831939102
RNF34	557.4367326	-0.121641512	0.120851494	-1.006537092	0.314157278	0.831939102
MIR6837	2.2023728	-2.021124444	2.008671345	-1.00619967	0.314319531	0.831939102
LINC00702	29.99448737	-0.387408137	0.385021364	-1.006199066	0.314319821	0.831939102
SLK	923.5213341	-0.158093061	0.157128417	-1.006139207	0.314348611	0.831939102
SNHG21	62.66292404	0.4337848	0.431240201	1.005900654	0.314463362	0.831939102
PDXDC1	1383.356366	0.18139288	0.180348622	1.00579022	0.314516493	0.831939102
DAZAP2	1634.172908	-0.134734163	0.133965915	-1.005734648	0.314543231	0.831939102
SIDT1	1.938592182	1.456808255	1.448515112	1.005725272	0.314547743	0.831939102
EXOC8	595.9309535	0.14018481	0.139391617	1.005690392	0.314564526	0.831939102
TRIM6	25.37990125	-0.382705155	0.380590764	-1.005555549	0.314629415	0.831939102
NPAS3	123.2691389	-0.272345133	0.270863512	-1.00546999	0.314670593	0.831939102
C3orf38	399.4823617	0.153424928	0.152593757	1.005446954	0.31468168	0.831939102
COX5A	387.831039	-0.131862387	0.131165802	-1.00531072	0.314747255	0.831939102
ACSM3	9.178723683	0.653095347	0.649793771	1.005080959	0.314857868	0.831939102
LOC101559451	3.368551491	1.338256234	1.331758335	1.004879187	0.314955027	0.831939102
MYOF	3841.662654	0.126157736	0.125563597	1.004731778	0.315026022	0.831939102
MAP7D3	359.5242327	-0.187360793	0.186496017	-1.004636969	0.315071689	0.831939102
SCGB1B2P	27.80451237	0.399515433	0.397693357	1.004581611	0.315098356	0.831939102
LRR1	339.2587636	0.138338887	0.137712059	1.004551733	0.315112749	0.831939102
SULF2	9.816295546	0.701461567	0.698308861	1.004514773	0.315130554	0.831939102
DNLZ	82.27419127	0.224157524	0.223159437	1.004472526	0.315150907	0.831939102
FAM174B	1.784531834	1.582620983	1.575664815	1.004414751	0.315178743	0.831939102
RPL13	6441.604084	-0.208092052	0.207187398	-1.004366358	0.315202059	0.831939102
PCTP	232.4531018	0.146029855	0.145408446	1.004273544	0.315246782	0.831939102
NUDT22	157.9324462	0.206424784	0.205562543	1.004194545	0.315284851	0.831939102

FBXL5	888.9289691	-0.196499867	0.195691997	-1.004128272	0.31531679	0.831939102
MAP4K2	175.4345847	0.173021305	0.172310738	1.004123755	0.315318967	0.831939102
SRP19	235.384168	0.158038717	0.157390644	1.004117614	0.315321926	0.831939102
POLR3H	878.3708207	-0.215024392	0.214148485	-1.004090183	0.315335147	0.831939102
ATXN10	2056.263476	-0.119478401	0.118993015	-1.004079114	0.315340482	0.831939102
SP2	144.7868072	0.207129274	0.206289618	1.004070276	0.315344741	0.831939102
GNPDA1	909.2032987	-0.151624379	0.151015074	-1.004034725	0.315361876	0.831939102
CAMK2N2	13.29738363	0.560961213	0.558808965	1.00385149	0.315450201	0.832048933
RYR1	7.293618835	0.850049475	0.846955121	1.003653504	0.315545656	0.832177533
FNTA	1247.184979	-0.146853583	0.146384257	-1.00320612	0.315761421	0.832623338
SMARCD3	244.6866779	0.217371235	0.216728147	1.002967256	0.31587666	0.832803978
DNAJB5	73.0996457	0.300781107	0.300051367	1.00243205	0.316134969	0.833316921
BTG2	55.34981319	0.311341004	0.310604753	1.00237038	0.316164742	0.833316921
ENC1	151.4115592	0.353919467	0.353209192	1.00201092	0.316338319	0.833556151
KPNA4	3631.068517	-0.127977334	0.127723333	-1.00198868	0.31634906	0.833556151
TMEM200A	233.9250371	-0.214607742	0.214223866	-1.001791942	0.31644409	0.833614817
BAP1	1100.223354	-0.117248363	0.117069491	-1.001527908	0.316571655	0.833614817
OSCAR	12.45595259	-0.703920278	0.702918402	-1.001425309	0.316621233	0.833614817
CDH18	789.1063856	-0.110133082	0.109983656	-1.001358622	0.316653461	0.833614817
TBXAS1	65.19656199	0.312289775	0.311887873	1.001288612	0.316687297	0.833614817
GNAL	150.9701998	0.303743722	0.303362807	1.001255642	0.316703232	0.833614817
SMIM5	55.58420101	0.511604244	0.511123167	1.000941216	0.316855229	0.833614817
SGIP1	7.694466889	0.80342219	0.802716782	1.000878776	0.316885419	0.833614817
AP1AR	660.2722631	-0.161553311	0.161418494	-1.000835204	0.316906487	0.833614817
TMEM267	297.6094582	-0.145308557	0.145202127	-1.000732978	0.316955919	0.833614817
ZNF204P	189.9962568	0.183226659	0.183100983	1.000686376	0.316978456	0.833614817
ZSCAN5A	57.647909	0.340053819	0.339865741	1.00055339	0.317042774	0.833614817
C2CD4D-AS1	8.453607054	-0.603321867	0.603076797	-1.000406365	0.317113891	0.833614817
SNORD37	9.151076033	0.657777824	0.657631189	1.000222974	0.317202614	0.833614817
ZNF738	133.3319385	0.259735209	0.259678948	1.000216656	0.31720567	0.833614817
LOC728613	3.035336378	-1.179572103	1.179321513	-1.000212487	0.317207688	0.833614817
WDR43	2069.399379	-0.13757999	0.137552642	-1.000198823	0.317214299	0.833614817
HOXA10	286.2876199	0.15363005	0.153640463	0.999932224	0.317343309	0.833614817
SSR4	609.9867849	0.118029947	0.118061628	0.999731654	0.317440389	0.833614817
SNORD19C	9.381447202	0.678824626	0.6790202	0.999711976	0.317449915	0.833614817
MIR617	4.77477178	1.362619867	1.363057915	0.999678628	0.317466058	0.833614817
SRCIN1	16.14726481	0.499996384	0.50017062	0.999651647	0.31747912	0.833614817
MCAM	926.4902597	0.155023396	0.155090198	0.999569266	0.317519003	0.833614817
PLEKHF2	380.5890579	-0.122673231	0.122734996	-0.999496766	0.317554105	0.833614817
ATG2B	1602.314975	0.089963926	0.090014727	0.999435637	0.317583704	0.833614817
YIPF6	631.0891534	0.209030505	0.209150224	0.999427595	0.317587598	0.833614817
MIR6071	6.377205218	0.741967878	0.742609264	0.999136308	0.317728665	0.833853577
THBS3	486.3161952	0.223980817	0.2242101	0.998977372	0.317805653	0.833853577
LOC101928119	43.07283326	0.420282096	0.42073322	0.998927766	0.317829684	0.833853577
LOC440896	2.746575672	1.297018523	1.298516422	0.998846454	0.317869079	0.833853577
MIG7	5.650224511	0.871338725	0.872423335	0.998756784	0.317912525	0.833853577
ACTRT3	3.116449719	1.120040899	1.12161188	0.998599354	0.317988813	0.833930928
SNORD116-1	18.9168351	0.495506787	0.496381107	0.998238612	0.318163667	0.83416146
FAM27E3	17.59292862	0.648908964	0.650062923	0.99822485	0.318170339	0.83416146
SNORA63D	2.421691524	1.331213096	1.334238778	0.997732278	0.318409197	0.83447056
ARPC1A	610.1103135	-0.190461943	0.19090832	-0.997661827	0.31844337	0.83447056
UBA5	487.1231417	-0.119805276	0.12008811	-0.997644779	0.318451639	0.83447056
SNORA8	29.81777602	0.566227374	0.567645288	0.997502113	0.318520849	0.83447056
ST3GAL5	84.22081037	-0.402056194	0.403064272	-0.997498964	0.318522377	0.83447056
SNORD125	3.591474055	1.127161481	1.130246443	0.997270541	0.318633209	0.834571988
NOL9	1781.999427	-0.148607483	0.149022453	-0.997215387	0.318659974	0.834571988
ZBTB11-AS1	67.32080786	-0.28179257	0.282647697	-0.996974582	0.318776848	0.834571988
RPL32	3819.082346	-0.200955361	0.201572398	-0.99693888	0.318794178	0.834571988
SLC31A2	22.89123956	-0.397853847	0.399090926	-0.996900259	0.318812926	0.834571988
LINC01238	3.580217612	1.01326968	1.01657192	0.996751592	0.318885101	0.834571988
PKP3	6.817409065	0.690082283	0.692410302	0.996637805	0.318940349	0.834571988
FAM230G	3.156945937	1.219301115	1.223499485	0.996568556	0.318973975	0.834571988
CPEB2	223.1363308	-0.198763109	0.199464891	-0.996481672	0.319016168	0.834571988
HS1BP3-IT1	3.141724355	-1.062021042	1.065870751	-0.996388202	0.319061563	0.834571988
NUTM2G	66.2585723	0.531376257	0.533318634	0.996357943	0.31907626	0.834571988
GPATCH4	232.9605888	0.277868524	0.278956117	0.996101205	0.319200975	0.834681036
UBE2T	134.949515	0.200231607	0.201019751	0.996079271	0.319211631	0.834681036
ARHGAP30	37.88227033	0.4472716	0.449139581	0.995840978	0.319327417	0.834790191
NFATC2	73.49820542	0.399321073	0.401038185	0.995718333	0.31938702	0.834790191
FAM92A	593.4141492	0.142223786	0.142861224	0.995538065	0.31947464	0.834790191
PCMT1	924.8060182	-0.131091522	0.13169224	-0.995438467	0.319523057	0.834790191
UBA52	930.2366344	-0.176224444	0.177039927	-0.995393791	0.319544777	0.834790191
TMED6	14.78006819	0.620623216	0.623538694	0.995324303	0.319578561	0.834790191
DDX23	1011.040785	-0.099274117	0.099741037	-0.99531868	0.319581295	0.834790191
PRH2	23.83933801	0.742923287	0.746560394	0.995128182	0.319673926	0.834909769
KIAA0100	6065.293559	0.09850326	0.099011078	0.994871102	0.319798959	0.834940399
ITPKB	613.2191677	0.181096569	0.182068508	0.994661685	0.319900835	0.834940399
LACC1	389.8024459	-0.593608428	0.596855746	-0.994559293	0.319950654	0.834940399
MIR10523	6.538175745	1.129448927	1.135630715	0.994556516	0.319952005	0.834940399
MLLT6	3112.23212	-0.135259874	0.136013299	-0.994460652	0.319998653	0.834940399
ESR2	26.03788851	0.518363839	0.521315416	0.994338213	0.320058238	0.834940399
ACY1	18.28293042	-0.488858692	0.491643901	-0.994334905	0.320059848	0.834940399
MAP3K3	797.2325053	-0.104235559	0.104881756	-0.993838803	0.320301352	0.834940399
CCDC71	265.0474916	-0.19162993	0.192819575	-0.993830267	0.320305508	0.834940399
RNU5B-1	20.27220866	-0.723109495	0.727688905	-0.993706912	0.320365577	0.834940399
DDX19B	178.2220407	-0.188014481	0.189221897	-0.993619048	0.320408367	0.834940399
EDARADD	51.34890836	-0.323318353	0.325441599	-0.993475799	0.320478139	0.834940399
RXRA	2237.671856	0.29114291	0.293061073	0.993454734	0.320488399	0.834940399
ANKRD17	2774.921922	-0.141313538	0.142274501	-0.993245715	0.320590225	0.834940399
CREB1	793.3912432	-0.145932533	0.146930344	-0.993208954	0.320608136	0.834940399

THAP9-AS1	259.9849376	0.25890403	0.260680662	0.99318464	0.320619982	0.834940399
LOC145845	6.032506736	1.05867718	1.065962387	0.993165606	0.320629256	0.834940399
HOXA11	48.90516391	-0.282471274	0.284439522	-0.993080258	0.320670844	0.834940399
DENND10	222.7185481	-0.214528683	0.216030606	-0.993047641	0.320686738	0.834940399
BAG3	352.8297043	0.170846337	0.172044802	0.993033992	0.320693389	0.834940399
ZC3H15	1160.754773	-0.11736966	0.118198385	-0.992988691	0.320715466	0.834940399
SDAD1P1	94.87257802	-0.318795943	0.321047551	-0.992986684	0.320716444	0.834940399
EXOC3-AS1	32.86445155	0.341556401	0.344058364	0.992728084	0.320842485	0.835146521
TXNDC2	2.596783704	1.165498905	1.174430629	0.992394848	0.321004951	0.835394931
ZNF771	74.25288988	0.45116486	0.454687313	0.992253019	0.321074115	0.835394931
MARCHF2	54.56746683	0.410571363	0.413821642	0.992145701	0.321126456	0.835394931
OPA3	473.7453471	0.171301761	0.172661869	0.992122707	0.321137671	0.835394931
TRIM9	345.8973516	-0.13835197	0.139473551	-0.991958462	0.321217789	0.835394931
PFN1P2	70.07555597	0.477626104	0.481499489	0.991955578	0.321219196	0.835394931
ZNF175	171.0948423	-0.172947793	0.174389033	-0.991735493	0.321326572	0.835491327
STK31	4.933762461	-0.768036034	0.774473912	-0.991687417	0.321350031	0.835491327
DKC1	1754.079386	-0.157847982	0.159230519	-0.99131739	0.321530624	0.835603338
ARID1A	1852.924214	-0.126956773	0.128077782	-0.991247434	0.321564774	0.835603338
APBA3	241.2477442	0.162607493	0.164049023	0.991212809	0.321581677	0.835603338
HEIH	48.95992604	-0.417001315	0.420704082	-0.991198645	0.321588592	0.835603338
FIGNL2	20.74766787	0.534416098	0.53920488	0.991118808	0.32162757	0.835603338
LEKR1	22.8921157	-0.391458286	0.395146824	-0.990665398	0.321848993	0.83596001
PLK3	67.39054446	-0.348791183	0.352149559	-0.99046321	0.321947763	0.83596001
ZNF774	41.86247859	0.31374803	0.316825569	0.990286328	0.322034187	0.83596001
MIR4296	5.448973237	1.190494073	1.202326649	0.990158602	0.322096604	0.83596001
ZNF503-AS2	33.28113768	0.382200767	0.3860272	0.990087659	0.322131275	0.83596001
GPAM	628.2503456	0.124954613	0.126212514	0.990033473	0.322157758	0.83596001
DIS3L2	460.5461633	-0.140329825	0.141746986	-0.990002181	0.322173053	0.83596001
ATL2	1457.230746	0.095625163	0.096591888	0.989991655	0.322178198	0.83596001
ERBB4	36.75259992	0.323109275	0.326383446	0.989968329	0.322189599	0.83596001
FLJ46875	47.50666214	0.406357605	0.410564833	0.989752586	0.322295065	0.83596001
SAMD8	663.9905	0.172504058	0.174299964	0.989696467	0.322322503	0.83596001
PKD1P6-NPIPP1	858.1647049	-0.192506255	0.194512673	-0.989684903	0.322328157	0.83596001
FN3K	9.809625314	0.734990845	0.742722805	0.989589709	0.322374702	0.83596001
ITPK1	420.3160135	0.150521721	0.152134859	0.989396655	0.322469111	0.836083159
DLG5	958.6227229	-0.128781722	0.130200204	-0.989105374	0.322611589	0.836330886
EIF4A2	8603.860936	0.239881035	0.242636371	0.988644177	0.322837264	0.836740244
HSF1	1336.361786	0.124137377	0.125570035	0.988590763	0.322863407	0.836740244
INSRR	101.4339334	-0.957452852	0.968693616	-0.988395955	0.322958768	0.836743266
SCNN1D	71.91563923	0.382695799	0.387278426	0.9881671	0.323070818	0.836743266
PDGFRL	148.7871356	-0.286331848	0.289779797	-0.988101485	0.323102949	0.836743266
CLUAP1	222.0238827	-0.165297806	0.167292142	-0.988078719	0.323114097	0.836743266
CRTC1	105.507827	-0.216069056	0.21868727	-0.988027586	0.323139138	0.836743266
PPM1J	31.38277248	0.385784756	0.390465282	0.988012952	0.323146305	0.836743266
SRSF2	3255.310532	-0.285140765	0.288673342	-0.98776272	0.323268869	0.836903795
BID	120.8560341	0.211822467	0.214461497	0.987694622	0.323302229	0.836903795
GPAT4	3798.894867	-0.104358463	0.105692827	-0.987375078	0.323458797	0.837187476
TMEM50B	292.0731337	0.143848414	0.145718193	0.98716853	0.323560027	0.837327864
LCMT2	110.3754318	-0.184143834	0.186577857	-0.986954382	0.323665003	0.837404916
DMAP1	211.952568	0.159374452	0.161503042	0.986820124	0.323730828	0.837404916
ZHX1-C8orf76	34.98932995	0.378781082	0.383874437	0.986731715	0.323774178	0.837404916
ZDHHC14	161.0143469	0.159037979	0.16119177	0.986638333	0.323819971	0.837404916
TUSC2	95.58352402	0.217311588	0.220277182	0.986536989	0.323869673	0.837404916
CDAN1	695.1938356	0.176786958	0.179200294	0.986532744	0.323871756	0.837404916
DCLK2	56.11819756	0.276632812	0.280469702	0.98631977	0.323976221	0.83753762
CLPSL2	2.021975577	1.36471363	1.383759018	0.986236485	0.324017079	0.83753762
MIR924HG	93.0387326	0.236584367	0.239962569	0.985921962	0.324171409	0.837815012
AKAP9	681.823603	0.136423035	0.138390792	0.985781155	0.324240515	0.837872097
LAGE3	224.9533364	-0.15619851	0.158505182	-0.985447335	0.324404389	0.838055853
CHRNB4	6.392470432	-0.716339055	0.72691959	-0.985444698	0.324405683	0.838055853
FSD1L	260.8846605	0.352342051	0.357633782	0.985203493	0.324524125	0.838123103
CALR	16939.93973	-0.131008688	0.132976727	-0.985200122	0.324525781	0.838123103
FOXD4	2.909548198	-1.317605828	1.337653188	-0.985013036	0.324617668	0.838179104
ZNF398	343.3292457	0.133290003	0.135334631	0.984892055	0.324677096	0.838179104
RPARP-AS1	29.22869484	0.419677981	0.426126101	0.984868049	0.32468889	0.838179104
PRKCI	2479.864887	0.118327189	0.120156822	0.984772955	0.324735608	0.838179104
MECR	435.682831	-0.14004355	0.142236883	-0.984579717	0.324830557	0.838290467
QARS1	2078.496945	-0.156910409	0.159381835	-0.98449368	0.324872837	0.838290467
IGSF6	21.83210411	0.719086312	0.730598357	0.984242991	0.324996052	0.838486992
BAHD1	1105.127666	0.101915297	0.1035862	0.983869444	0.325179709	0.838707206
MFSD11	411.2152263	-0.155100882	0.157649608	-0.98383297	0.325197645	0.838707206
RCC1L	448.9400462	0.159072617	0.16169495	0.983782219	0.325222604	0.838707206
ZNF582	22.68319738	-0.383751685	0.390167156	-0.983557122	0.325333316	0.83887132
LINC00887	4.529245544	0.952930218	0.96896656	0.983450057	0.325385984	0.838885741
LINC00174	20.56435975	0.475446968	0.483525042	0.98329337	0.325463072	0.838924009
PIF1	263.985204	0.368070669	0.374435179	0.983002371	0.325606272	0.838924009
BCAR3-AS1	20.27098031	0.818644467	0.832811845	0.982988502	0.325613098	0.838924009
SALL1	199.1544538	0.20313873	0.20666398	0.982942114	0.325635929	0.838924009
LOC105370757	11.24228927	0.69350785	0.705664475	0.982772797	0.325719273	0.838924009
PPP1R3C	127.2099084	-0.201932339	0.205502556	-0.982626896	0.325791102	0.838924009
TNRC17	37.67069523	0.846996746	0.862002405	0.982592091	0.325808239	0.838924009
SMPD2	99.89629339	0.318059582	0.323726578	0.9824945	0.325856291	0.838924009
ELK1	296.0949529	-0.143699016	0.146276533	-0.98237915	0.325913094	0.838924009
TBL2	715.3021666	0.14715929	0.149805351	0.982336673	0.325934013	0.838924009
GDF6	19.26354577	0.825522917	0.840421141	0.982272907	0.325965418	0.838924009
MRPS16	1176.467882	-0.117982956	0.120112388	-0.982271339	0.325966191	0.838924009
PTPN4	408.4502471	-0.142276902	0.144858773	-0.982176632	0.326012838	0.838924009
FNDC8	4.429291886	0.928203288	0.945235592	0.98198089	0.326109263	0.839030167
KDM5B	1345.242178	-0.136935031	0.139458996	-0.98190174	0.326148258	0.839030167
PEX16	146.0778073	-0.167383274	0.170505914	-0.981686028	0.326254551	0.839155824

CTDSP2	4155.218183	-0.212719548	0.21670442	-0.981611489	0.326291285	0.839155824
MFF-DT	6.76470163	0.752894942	0.767097763	0.981484993	0.326353631	0.839195052
PHOSPHO1	4.612433066	1.039595714	1.059323226	0.98137725	0.326406741	0.839210521
OGFOD3	525.885931	-0.120092602	0.122383539	-0.981280678	0.326454348	0.839211842
THAP6	158.0493018	0.182354046	0.185868978	0.981089194	0.326548759	0.839332897
CEP83	721.6322077	0.226655643	0.23104763	0.980990989	0.326597185	0.839332897
BCL2L2-PABPN1	2.178635005	1.548198333	1.578937786	0.980531561	0.326823798	0.839332897
TAOK1	3131.972607	0.091246344	0.093060595	0.98050462	0.32683709	0.839332897
APOBEC3C	196.5722036	-0.220828321	0.225222796	-0.980488322	0.326845131	0.839332897
SERPINB5	181.6963128	0.219174391	0.22357035	0.980337469	0.326919565	0.839332897
CCDC73	11.68690119	0.696195834	0.710267761	0.980187856	0.326993398	0.839332897
ENO4	4.116559028	-1.027649074	1.048501217	-0.980112428	0.327030625	0.839332897
SLC25A46	735.7121428	-0.119298149	0.121724388	-0.980067767	0.327052669	0.839332897
CEP295NL	25.52668537	0.631286635	0.644162635	0.980011259	0.327080561	0.839332897
ADAP1	4.260693561	1.06807867	1.090160362	0.979744547	0.327212231	0.839332897
HSPA5	10877.36027	-0.228434554	0.233170005	-0.979690993	0.327238673	0.839332897
CETN4P	13.42452021	0.612761632	0.625479572	0.979666898	0.327250571	0.839332897
ALPK3	11.5549735	0.61435959	0.627154498	0.979598474	0.327284358	0.839332897
SNORD110	32.59093317	0.489821032	0.500038055	0.979567508	0.32729965	0.839332897
CAMK2A	2.592349957	1.39997437	1.429470759	0.979365518	0.327399409	0.839332897
MAP1B	9418.915857	0.191620789	0.195677189	0.979269939	0.327446619	0.839332897
LOC100996643	2.74591115	-1.734341857	1.771186804	-0.979197594	0.327482357	0.839332897
MDGA2	585.4929054	-0.108333295	0.110634989	-0.979195611	0.327483337	0.839332897
BRAP	730.4664634	-0.113261276	0.115670385	-0.979172643	0.327494683	0.839332897
TMEM184A	5.632946516	0.971243281	0.991933493	0.979141532	0.327510053	0.839332897
MCRIP2	177.3561668	0.179795101	0.183637695	0.979075135	0.327542856	0.839332897
ATF7IP	1882.016416	0.152852007	0.156132293	0.978990339	0.327584753	0.839332897
PHKA1-AS1	2.495697251	1.285151406	1.312941923	0.9788334	0.327662304	0.839410904
ESRRG	57.85242783	0.306789626	0.313554083	0.978426505	0.327863425	0.839805408
SFR1	108.859167	-0.293185082	0.299706961	-0.97823915	0.327956058	0.839837619
LECT2	12.54911667	0.653663767	0.66822408	0.978210433	0.327970258	0.839837619
RPAIN	372.3480296	0.152580439	0.155995866	0.978105662	0.328022068	0.839849604
PDE5A	421.081521	0.196379871	0.200846914	0.977758963	0.328193552	0.840167949
MSTO1	346.6474983	0.222509362	0.227632041	0.977495793	0.328323759	0.84038055
SYT1	258.8538809	0.235124373	0.240574464	0.977345515	0.328398127	0.840450183
DOK4	117.1502062	-0.203222678	0.207967657	-0.977184053	0.328478041	0.840533988
RBM45	148.2609488	-0.259032096	0.265117391	-0.977046789	0.328545989	0.840587153
SHARPIN	603.9462218	-0.115287103	0.118026898	-0.976786691	0.328674766	0.840795914
CAMSAP3	3.27283931	-1.026357292	1.050858899	-0.976684208	0.328725516	0.840805037
MROH6	64.44886188	0.457425405	0.468427835	0.976512007	0.328810801	0.840892909
TMC7	59.33997691	0.286733914	0.293679238	0.976350647	0.32889073	0.840892909
SNORA69	16.99342991	0.528396691	0.541309162	0.976145849	0.328992193	0.840892909
LOC728975	15.35259553	-0.540152111	0.553375282	-0.976104515	0.329012674	0.840892909
NPRL3	526.2351526	0.115607048	0.11843803	0.976097356	0.329016222	0.840892909
CDK9	594.6365765	0.144592667	0.148141649	0.976043315	0.329043	0.840892909
AP3S2	80.30110932	-0.253533069	0.259784608	-0.97593568	0.329096339	0.840908627
SNORA74B	8.606819313	1.035442219	1.061208495	0.975719874	0.329203301	0.841061336
NME2	659.1439057	-0.232781674	0.238652813	-0.975398828	0.329362465	0.841241951
ATP6V1H	579.0907816	-0.126013807	0.129205764	-0.975295557	0.329413674	0.841241951
LOC100507156	61.79219218	0.522721491	0.535990967	0.975243097	0.329439689	0.841241951
SCARNA1	6.877123283	0.831293624	0.852522502	0.975098747	0.32951128	0.841241951
PSKH1	614.5838757	-0.141846678	0.145515362	-0.974788339	0.329665263	0.841241951
NCBP1	1657.758104	-0.15040743	0.154303892	-0.974748133	0.329685211	0.841241951
ANXA9	25.43043272	-0.492143821	0.504893794	-0.974747217	0.329685665	0.841241951
SPTLC2	1871.899045	0.144935954	0.148696314	0.974711141	0.329703565	0.841241951
RGL1	490.379827	0.180843173	0.18554558	0.974656325	0.329730764	0.841241951
RPS18P9	26.22089271	0.381026108	0.390969872	0.97456642	0.329775377	0.841241951
ITPKA	15.58426977	-0.547652412	0.56201573	-0.974443211	0.329836523	0.841241951
ME1	1528.716289	0.140119633	0.143804343	0.974376925	0.329869422	0.841241951
NLRX1	361.4308868	-0.184981576	0.189870723	-0.974250131	0.329932359	0.841241951
MACROH2A1	1254.681514	0.140717536	0.144437524	0.974245003	0.329934904	0.841241951
HLA-B	1520.121083	-0.236877641	0.243207867	-0.973971954	0.330070465	0.841347204
LOC100506321	30.09353035	0.577334197	0.592762831	0.973971658	0.330070612	0.841347204
LOC651337	49.75272264	0.505724198	0.519306936	0.973844488	0.33013376	0.841387815
MIR221	58.03131371	0.507992632	0.521773389	0.973588616	0.330260841	0.841426636
MGST1	373.2479047	0.131179606	0.134778463	0.973297982	0.330405225	0.841426636
MEIS3	38.25205853	-0.538460735	0.553252103	-0.973264688	0.330421768	0.841426636
H2AJ	66.38517176	-0.268247948	0.275664338	-0.973096301	0.330505442	0.841426636
CCDC144NL	44.92114739	0.385760617	0.396427538	0.973092382	0.33050739	0.841426636
CHAF1A	599.7479591	-0.179261769	0.1842191	-0.973090027	0.33050856	0.841426636
SNX3	1100.696335	-0.142102832	0.146041147	-0.973032839	0.330536981	0.841426636
SLC9A9	163.4657405	-0.319248129	0.328107904	-0.972997374	0.330554607	0.841426636
C17orf75	341.8327215	-0.125345823	0.128829575	-0.972958446	0.330573955	0.841426636
HLA-A	3809.512224	-0.177203352	0.182200872	-0.972571371	0.330766377	0.841796178
MAPK7	268.9030473	-0.140117891	0.144093234	-0.972411317	0.330845964	0.841826261
LOC93463	9.778680212	0.809915452	0.832939911	0.972357599	0.330872678	0.841826261
TMEM158	317.6022261	-0.87021515	0.895423026	-0.971848082	0.331126131	0.842288113
RHBDL3	2.360371766	-1.172158958	1.206169752	-0.971802647	0.331148739	0.842288113
TCEA2	111.7912011	-0.375711472	0.386685802	-0.971619516	0.33123987	0.842293468
RSPH9	33.12599881	0.429660017	0.442215192	0.971608449	0.331245377	0.842293468
SF3B2	1601.15183	-0.17546605	0.180692932	-0.971073128	0.331511864	0.842527045
PGAP1	1426.084831	0.135005339	0.13904198	0.970968188	0.33156412	0.842527045
LOC643201	7.283127993	0.820440806	0.845008675	0.970925898	0.331585181	0.842527045
M6PR	1074.768426	0.113717953	0.117140287	0.970784318	0.331655693	0.842527045
FBXL13	141.8440377	-0.370999714	0.382199223	-0.970697195	0.331699089	0.842527045
CREB3L4	212.595722	-0.262523332	0.270513755	-0.970462045	0.331816235	0.842527045
ADAT2	846.486948	0.272520994	0.280821749	0.970441197	0.331826622	0.842527045
C4orf19	3.004522468	-1.059835976	1.092436839	-0.970157668	0.331967908	0.842527045
FCHSD1	15.4960835	0.48070262	0.495516536	0.970104094	0.331994609	0.842527045
ANXA11	1086.951511	-0.121662396	0.125424962	-0.970001453	0.332045768	0.842527045

KIRREL3	18.60337944	0.576364564	0.594317601	0.969792185	0.332150089	0.842527045
NPL	103.5314031	-0.27264444	0.281140379	-0.969780438	0.332155946	0.842527045
WWC2	1076.959663	0.113293283	0.116838288	0.969658873	0.332216557	0.842527045
ALX3	116.1186016	-0.20557458	0.212009357	-0.969648622	0.332221668	0.842527045
GGA1	537.8869268	0.122506347	0.126369553	0.969429297	0.332331041	0.842527045
SETDB2-PHF11	3.393061333	1.00432104	1.036003445	0.969418629	0.332336361	0.842527045
RPS14	6653.412086	-0.202965592	0.209370104	-0.969410571	0.33234038	0.842527045
APTR	59.36777008	0.274878183	0.283553453	0.969405167	0.332343075	0.842527045
LINC01617	3.204612649	-1.140466618	1.176524063	-0.969352565	0.332369311	0.842527045
KRTCAP3	12.16356733	0.65592899	0.67666798	0.969351307	0.332369938	0.842527045
SMIM15	669.9983059	-0.108774932	0.112214973	-0.969344185	0.332373491	0.842527045
EFNB1	368.9921102	-0.152661816	0.157491065	-0.969336363	0.332377392	0.842527045
SRSF12	10.61286732	-0.624305041	0.644167628	-0.969165499	0.332462622	0.842553256
NXPH1	3.450380058	1.302641452	1.344140356	0.969126063	0.332482295	0.842553256
ALDH1A3	38.48321096	0.481546946	0.496940593	0.969023164	0.332533632	0.842563532
MAPRE2	575.0664111	0.120886469	0.12477131	0.968864307	0.332612896	0.842571301
NT5M	92.34627616	-0.3576949	0.369248734	-0.968709888	0.332689958	0.842571301
PLOD1	4960.338244	-0.103205705	0.106546528	-0.968644473	0.332722606	0.842571301
MTIF2	709.9702239	-0.100933715	0.104207617	-0.968582891	0.332753343	0.842571301
KAT2A	1341.068264	0.174461208	0.180127424	0.96854329	0.33277311	0.842571301
OTUD3	719.5818229	0.123084477	0.127130791	0.96817204	0.332958456	0.842740766
ZYG11B	1108.576014	0.197690327	0.20419668	0.968136835	0.332976036	0.842740766
CTXND1	1.933657998	1.614434732	1.667589032	0.96812506	0.332981916	0.842740766
FBF1	365.6666457	0.144982536	0.149776664	0.967991484	0.333048623	0.842789897
CA5BP1	213.6592292	-0.188158585	0.194442527	-0.967682266	0.333203078	0.843061032
EP300-AS1	6.220075874	-0.910209755	0.940963916	-0.967316323	0.333385926	0.843403919
MAP2K1	1187.719922	0.140212865	0.14500331	0.966963201	0.33356243	0.84373066
VSTM2A	14.95386765	-0.595671521	0.616102887	-0.966837736	0.333625157	0.843769556
BAZ1A	1634.716106	0.148629557	0.153783366	0.966486561	0.333800768	0.843859841
AK3	670.1745344	0.173026903	0.17904698	0.966377109	0.333855514	0.843859841
JAG2	6.130641365	0.981322975	1.015627035	0.966223762	0.333932225	0.843859841
PPARA	391.0958393	0.358309125	0.370834561	0.966223655	0.333932279	0.843859841
KLRC3	5.080075952	-1.198429046	1.240629048	-0.965984995	0.334051689	0.843859841
NUP50	2083.56609	-0.137017515	0.141845394	-0.965963799	0.334062296	0.843859841
ANTXR1	2866.491286	-0.214252757	0.221806745	-0.965943381	0.334072513	0.843859841
DPYD	382.1834617	-0.192704815	0.199511811	-0.965881737	0.334103362	0.843859841
AHNAK2	257.1006372	-0.400613663	0.414783913	-0.965837032	0.334125735	0.843859841
CARHSP1	345.0152251	0.145613989	0.150767257	0.965819714	0.334134402	0.843859841
UPK3BL2	9.943365651	0.739828655	0.766704643	0.964946099	0.33457181	0.844844786
ZSCAN9	177.0158729	0.165786359	0.171847383	0.964730194	0.334679968	0.844922958
PPTC7	1123.456914	0.117460567	0.121765316	0.964647163	0.334721568	0.844922958
TTC17	1118.054573	-0.106353256	0.110264535	-0.964528221	0.334781166	0.844922958
SPC25	82.70280552	-0.256508083	0.265947696	-0.964505755	0.334792425	0.844922958
STT3A	3301.976273	-0.130041583	0.134848641	-0.964352193	0.334869382	0.844990202
DRAIC	2.186342881	-1.354654303	1.404867099	-0.964257974	0.334916605	0.844990202
NKAIN3	111.3137633	0.394226267	0.408876831	0.96416876	0.334961324	0.844990202
MIR1285-1	2.90879507	1.715872684	1.780269704	0.963827379	0.335132477	0.8453023
BBS4	671.2337286	-0.140792273	0.146098449	-0.963680821	0.335205973	0.845368021
INPP5J	4.889704638	0.876917983	0.910151964	0.963485239	0.335304068	0.845490645
VWCE	7.71999923	0.758327494	0.787141033	0.963394694	0.335349488	0.845490645
ATP5F1B	7331.939309	-0.122367185	0.127042149	-0.963201474	0.335446426	0.845615406
ST7-OT3	3.37342269	1.042434271	1.082528338	0.96296257	0.335566307	0.845686687
SCCPDH	797.6314008	-0.106143913	0.110235325	-0.962884746	0.335605366	0.845686687
SDHAP1	417.4971059	0.318288297	0.330615674	0.962713876	0.335691131	0.845686687
PLGLB2	3.098262061	1.715186391	1.781811436	0.962608252	0.335744155	0.845686687
SLC25A29	256.9455885	0.199172661	0.206912802	0.962592254	0.335752186	0.845686687
BAALC	543.4807742	-0.271961189	0.282574986	-0.962439007	0.335829128	0.845686687
HSP90B1	14532.1055	-0.116450565	0.120995691	-0.962435631	0.335830823	0.845686687
DDRGK1	223.4180682	0.145256841	0.150952767	0.962266834	0.335915585	0.845686687
SNORD12C	21.62964299	0.45839604	0.47638514	0.962238328	0.3359299	0.845686687
ISCA2	207.9707627	0.162856102	0.169299127	0.961942956	0.336078259	0.845686687
MIR5585	1.941506348	1.551530435	1.613358573	0.961677374	0.336211691	0.845686687
TFDP1	2210.044031	-0.136016507	0.1414489	-0.961594662	0.336253254	0.845686687
CLPTM1	824.9831394	-0.100740512	0.104775674	-0.961487607	0.336307054	0.845686687
TMUB1	231.4486317	-0.1484639	0.154413225	-0.9614714	0.336315199	0.845686687
WDYHV1	485.6877593	0.127720124	0.132846975	0.961407839	0.336347144	0.845686687
HENMT1	189.9404047	-0.186202274	0.193682147	-0.961380679	0.336360795	0.845686687
MCM3AP	1874.372465	0.135296963	0.140734215	0.9613651	0.336368626	0.845686687
RAD50	807.289512	-0.132521864	0.13784844	-0.961359182	0.3363716	0.845686687
ZNF304	263.0369123	-0.140264866	0.145916422	-0.961268539	0.336417162	0.845686687
LBX2	53.30380001	0.392589199	0.408413063	0.961255244	0.336423845	0.845686687
PANX1	539.2341227	0.151239643	0.157357256	0.961122781	0.336490436	0.845734778
HSFX4	2.574191696	1.457627845	1.516976132	0.960877244	0.336613894	0.845925764
ARPC5	2257.893664	0.124886097	0.130023257	0.960490455	0.336808433	0.845971963
LINC02587	3.869679162	-1.156583024	1.204210827	-0.96044895	0.336829312	0.845971963
YES1	1280.073957	0.149307265	0.155457916	0.960435269	0.336836195	0.845971963
IER5L	468.1116705	-0.259418966	0.270117357	-0.960393544	0.336857186	0.845971963
TSTD2	416.2545581	-0.118973735	0.123883384	-0.960368782	0.336869644	0.845971963
RRP8	358.0130243	0.201941947	0.210344597	0.960052928	0.337028578	0.846143013
SLC23A2	839.0925975	0.103136112	0.107430432	0.960026963	0.337041645	0.846143013
VANGL2	18.19848989	-0.427707233	0.445551412	-0.959950348	0.337080205	0.846143013
SFI1	372.8509876	0.287155047	0.299250677	0.959580274	0.337266501	0.846311766
GTSE1	413.5685142	0.138206713	0.144029302	0.959573579	0.337269872	0.846311766
CNTLN	366.5504649	-0.18076393	0.18841094	-0.959413132	0.337350662	0.846311766
MAGEB2	15.29941146	-0.538192068	0.561018303	-0.959312851	0.337401163	0.846311766
C5orf24	835.3877153	-0.15106951	0.157483154	-0.959274095	0.337420682	0.846311766
GPR63	43.17922819	0.299326282	0.312054393	0.959211884	0.337452015	0.846311766
RAB20	32.62069919	0.381032552	0.397284093	0.959093401	0.337511695	0.846311766
MYLK3	9.170985467	-0.628528058	0.655356872	-0.959062284	0.337527369	0.846311766
DNTTIP2	526.5692894	-0.120700376	0.125875633	-0.958885951	0.337616203	0.846406221

SH3YL1	556.906261	0.213852122	0.223045337	0.958783202	0.337667973	0.846406221
NTN4	429.1995621	0.364497038	0.380197406	0.958704695	0.337707533	0.846406221
TRMO	79.33870204	-0.232466164	0.242526052	-0.958520385	0.337800417	0.84651996
NAPG	602.9058115	0.158270877	0.16513727	0.958420087	0.33785097	0.8465276
CLYBL	97.86043346	0.237618018	0.24797621	0.958229089	0.337947252	0.846591335
TRANK1	154.8203907	-0.223258461	0.233002352	-0.958181146	0.337971423	0.846591335
SERTAD4-AS1	21.94619547	-0.461659015	0.481936308	-0.957925367	0.338100394	0.846669003
NCAPD3	1616.378802	0.11766915	0.122843632	0.957877492	0.338124538	0.846669003
TFAP2A-AS1	2.198483711	-1.14472696	1.195197939	-0.957771866	0.338177809	0.846669003
SUPT20H	692.5077951	0.122772147	0.12818906	0.957742781	0.338192478	0.846669003
LOC105372582	9.548336201	-0.613007589	0.640135521	-0.957621581	0.338253613	0.846703102
ANP32A-IT1	66.1980332	0.570638522	0.595994926	0.957455336	0.33833748	0.846794085
LOC728024	26.93355275	0.398283317	0.416212596	0.956922786	0.338606229	0.847347705
FOXK2	2101.677067	0.136603176	0.142793938	0.956645489	0.33874622	0.847579001
CAMTA1	233.154333	0.13630305	0.142547566	0.956193459	0.338974504	0.847968397
MAPK10	30.04612028	-0.38063567	0.398092467	-0.956148889	0.338997018	0.847968397
NT5DC4	7.111520862	0.766502011	0.801759077	0.956025361	0.339059422	0.847973061
ADH6	6.104389941	0.867199729	0.907153666	0.955956814	0.339094053	0.847973061
NFIC	1370.520811	0.115767094	0.121136605	0.955673922	0.339237002	0.848152093
CEP97	814.0890726	0.171436885	0.179410757	0.955555219	0.339296995	0.848152093
NCOR1	2582.120441	-0.082113437	0.085941632	-0.955455873	0.33934721	0.848152093
BCKDHA	429.2998915	-0.26307593	0.27534577	-0.955438429	0.339356028	0.848152093
PAXIP1	263.0267311	0.19902547	0.208365711	0.955173809	0.339489809	0.848367464
TSSK4	67.86310417	0.452264266	0.473558932	0.955032701	0.33956116	0.848393103
TOB2	967.2857285	-0.122009575	0.127765501	-0.954949293	0.33960334	0.848393103
NSUN5P1	288.384649	0.252850668	0.264800843	0.954871082	0.339642896	0.848393103
MAP1A	149.6522395	0.380677146	0.398763789	0.954643215	0.339758155	0.848562064
LMNTD2-AS1	3.776693785	-0.891190449	0.934011607	-0.954153506	0.340005945	0.848632823
SCARNA27	9.359948916	0.821347078	0.860874085	0.954085031	0.340040603	0.848632823
TET3	1009.877715	-0.115667997	0.121239814	-0.954043014	0.34006187	0.848632823
SCARA3	747.1521807	-0.178603566	0.187216428	-0.953995156	0.340086095	0.848632823
STARD6	3.032294796	1.386485058	1.454070442	0.953519869	0.340326734	0.848632823
ACLY	9226.737388	0.12307022	0.129128233	0.953085294	0.340546857	0.848632823
PRDM13	169.3179408	-0.18426251	0.193353053	-0.952984749	0.340597798	0.848632823
NUDCD1	1538.081274	0.145598773	0.152810958	0.952803221	0.340689782	0.848632823
TSSK3	46.03878364	0.374112843	0.392799772	0.952426325	0.340880814	0.848632823
CRPPA-AS1	4.759967484	0.917227573	0.963100606	0.952369428	0.340909659	0.848632823
DCTN6	212.6938834	0.156205703	0.164031481	0.952290995	0.340949423	0.848632823
EIPR1	482.7211276	-0.123798013	0.130004315	-0.952260798	0.340964734	0.848632823
OSBPL2	437.3696685	0.178628629	0.187599887	0.952178769	0.341006326	0.848632823
FRMD6	2559.50798	-0.206279332	0.216642207	-0.952165947	0.341012828	0.848632823
AXIN2	221.2903013	-0.189174172	0.198693045	-0.952092573	0.341050035	0.848632823
HINFP	163.3231562	-0.17413559	0.18296366	-0.951749602	0.341223987	0.848632823
MX2	11.32078424	0.916752842	0.963272921	0.951706232	0.341245988	0.848632823
CCDC57	547.4023918	0.334889302	0.351903288	0.951651528	0.34127374	0.848632823
HOXA3	78.75659094	0.31185642	0.32779858	0.951365987	0.34141862	0.848632823
CCDC28A	191.7671349	-0.223296345	0.234713066	-0.95135882	0.341422257	0.848632823
USP9X	6876.723663	-0.110772889	0.116456087	-0.95119879	0.341503473	0.848632823
ZC3HAV1L	520.9414017	0.195328351	0.20540093	0.950961378	0.341623982	0.848632823
HSCB	110.4638033	-0.200359273	0.210741936	-0.950732813	0.341740027	0.848632823
SIRT4	26.44854419	0.425754878	0.447997855	0.950350259	0.341934309	0.848632823
GULP1	634.1181276	0.135866642	0.142967475	0.950332524	0.341943318	0.848632823
KPNA5	277.3678321	0.181069096	0.190549655	0.950246255	0.341987141	0.848632823
PHYH	183.1335038	-0.249533829	0.262619383	-0.950172933	0.34202439	0.848632823
GOLGA1	252.0042834	0.185580827	0.195325339	0.950111378	0.342055662	0.848632823
PDZD2	21.97240659	0.628643125	0.661733844	0.949993915	0.342115344	0.848632823
USP17L7	4.270666886	1.017346218	1.070907907	0.949984785	0.342119984	0.848632823
MIR4656	2.613805916	1.39023377	1.463548636	0.949906096	0.342159969	0.848632823
DMPK	602.6413185	0.240126024	0.252797376	0.949875461	0.342175537	0.848632823
ARHGAP1	949.1291953	-0.166862435	0.175697177	-0.949716083	0.342256535	0.848632823
LOC105377621	6.320745131	-0.68853161	0.725028744	-0.949661121	0.342284471	0.848632823
IDUA	156.8603878	0.269866643	0.284208011	0.949539186	0.342346452	0.848632823
TNK2-AS1	21.41457272	0.574943406	0.605525043	0.949495669	0.342368574	0.848632823
AP4B1-AS1	25.19139639	0.659801566	0.69489745	0.949494872	0.342368979	0.848632823
CPNE8	223.6760032	0.15298633	0.161143641	0.949378634	0.342428073	0.848632823
TMEM246-AS1	16.2278765	0.562286439	0.592281713	0.949356407	0.342439374	0.848632823
SPDL1	488.917627	-0.232754799	0.245182189	-0.949313653	0.342461112	0.848632823
BOLL	4.50734748	1.185457083	1.248792142	0.949282945	0.342476725	0.848632823
SPIRE1	684.2274573	0.148616907	0.156564027	0.949240445	0.342498336	0.848632823
IMPG2	10.33668861	0.664185087	0.699765301	0.949154076	0.342542255	0.848632823
RPS8	9195.324719	-0.171954787	0.181172994	-0.949119312	0.342559934	0.848632823
TSR3	281.219389	-0.16037654	0.168977169	-0.949101827	0.342568825	0.848632823
MIR378A	2.731418352	-1.134665519	1.195596314	-0.949037318	0.342601632	0.848632823
LYSMD2	74.02781602	-0.345473797	0.364033995	-0.949015207	0.342612878	0.848632823
LINC00649	43.8632505	-0.32998583	0.347733492	-0.948961886	0.342639997	0.848632823
FLJ42627	173.0538079	-0.207040833	0.218185814	-0.94891977	0.342661419	0.848632823
LYPLA2	490.2991238	-0.144128903	0.151914437	-0.94875053	0.342747508	0.848632823
TMEM14EP	98.13412859	1.022636822	1.078058192	0.948591486	0.342828423	0.848632823
LINC01686	4.950334741	-0.921205198	0.97120233	-0.948520374	0.342864606	0.848632823
NBPF15	1037.674248	0.154019318	0.162405049	0.948365333	0.342943502	0.848632823
TTC36	5.989896281	0.714326796	0.753286365	0.948280533	0.342986659	0.848632823
TMPO	6553.215679	-0.10938135	0.115362051	-0.948157121	0.343049473	0.848632823
GPR22	17.43475101	0.892874563	0.941716907	0.948134791	0.343060839	0.848632823
MIR1908	6.086204645	0.893700024	0.94268986	0.948031863	0.343113234	0.848632823
BNIP3	441.1324119	0.16377421	0.172760681	0.947983126	0.343138044	0.848632823
SELENOT	641.2636842	-0.184209061	0.194336458	-0.947887307	0.343186828	0.848632823
RBMXL1	604.9963333	-0.126345175	0.133326032	-0.947640707	0.343312395	0.848632823
SMAD5-AS1	34.14733051	0.537220079	0.566908689	0.947630702	0.34331749	0.848632823
CD8A	17.50309792	-0.496007797	0.523431039	-0.947608683	0.343328704	0.848632823
NINJ1	20.43921397	-0.467870695	0.493783377	-0.947522166	0.343372767	0.848632823

GPR68	29.82776708	-0.522563477	0.551510309	-0.947513525	0.343377168	0.848632823
XPC	321.9511664	-0.211586814	0.22332168	-0.947453084	0.343407952	0.848632823
TRPM3	3.297190998	1.246342706	1.315709326	0.947278157	0.343497058	0.848632823
KAT5	187.8111052	-0.156635107	0.165370455	-0.947177096	0.343548544	0.848632823
CHORDC1	667.7887787	0.245958434	0.259684262	0.947144166	0.343565322	0.848632823
FER1L6	4.743489124	-0.910688536	0.961509971	-0.947144142	0.343565334	0.848632823
SNORA38	10.9715054	0.633178876	0.668658339	0.946939325	0.343669698	0.848632823
CCDC89	15.58324477	-0.531347627	0.561125987	-0.946931062	0.343673908	0.848632823
TRA2B	2999.561086	-0.236812763	0.250090025	-0.946910067	0.343684608	0.848632823
DNAJA1	1340.360252	-0.179635324	0.189743061	-0.946729349	0.343776711	0.848632823
MIGA2	415.8039603	0.186600619	0.197103486	0.946713945	0.343784563	0.848632823
ZNF404	71.30717286	0.359052982	0.379277126	0.946677131	0.343803327	0.848632823
XRN1	2075.263371	0.143239997	0.151317968	0.94661592	0.343834528	0.848632823
PDZRN4	79.6568511	0.313633053	0.331361044	0.946499471	0.343893892	0.848632823
KIAA1656	43.86196666	0.331989258	0.350768842	0.946461653	0.343913172	0.848632823
ZFP28	99.84789744	0.238226027	0.251705631	0.946446948	0.343920669	0.848632823
MADD	594.2790456	0.106184911	0.11220805	0.946321687	0.343984535	0.848632823
JAZF1	651.3941881	0.13898639	0.146894054	0.946167564	0.344063127	0.848632823
TBC1D9	575.3079808	0.158517135	0.167538308	0.946154564	0.344069757	0.848632823
TPTE	25.67275873	0.464796385	0.491257597	0.946135769	0.344079342	0.848632823
MROH2A	3.240851206	1.143128382	1.208256194	0.94609768	0.344098767	0.848632823
SRGN	3.691616991	0.952972573	1.007278224	0.946086742	0.344104346	0.848632823
STXBP3	446.1427592	-0.126572242	0.133793428	-0.946027349	0.344134637	0.848632823
CILK1	1078.29495	0.106595028	0.112676732	0.946025198	0.344135734	0.848632823
HSPA13	1152.355004	-0.112772299	0.119206816	-0.94602224	0.344137243	0.848632823
LINC01389	18.64753622	0.429434123	0.453977905	0.945936176	0.344181141	0.848632823
ICAM5	12.50262129	0.69758518	0.737514083	0.945860149	0.344219922	0.848632823
AQP1	3.826535628	-0.988840401	1.045536105	-0.945773557	0.344264096	0.848632823
SMIM10L1	186.6405127	-0.182384906	0.192842375	-0.945771935	0.344264923	0.848632823
UBA6-AS1	102.0980288	-0.216012697	0.228408398	-0.945730099	0.344286267	0.848632823
KCNK6	34.08415208	0.53338019	0.564008793	0.945694814	0.344304268	0.848632823
LINCR-0003	8.792077503	-0.604100107	0.638841535	-0.945618083	0.344343418	0.848632823
ADNP-AS1	20.38865592	0.42082321	0.445076585	0.945507412	0.344399889	0.848632823
ITM2B	1243.348286	-0.182047283	0.192541632	-0.945495693	0.344405869	0.848632823
MIR5587	23.01617283	0.674756021	0.714138136	0.944853645	0.3447336	0.848948533
ZDHHC6	540.5146712	-0.174833108	0.185057444	-0.944750474	0.344786282	0.848948533
CIAO1	2256.53026	-0.100481126	0.106370603	-0.944632468	0.344846545	0.848948533
PHB	1992.150903	-0.132875779	0.140664343	-0.944630142	0.344847733	0.848948533
CCAR1	672.703141	-0.23501614	0.248830252	-0.944483793	0.34492248	0.848948533
PRAG1	47.84388269	0.312473721	0.330851761	0.944452342	0.344938545	0.848948533
SLC25A33	239.1593731	0.168231408	0.178149322	0.944328086	0.345002018	0.848948533
CLCF1	29.09467703	-0.398826324	0.422377841	-0.944240642	0.345046691	0.848948533
DEFB109B	2.373959893	1.215894674	1.287745329	0.944204297	0.34506526	0.848948533
CHTOP	841.0606514	-0.175066764	0.185426295	-0.944131271	0.345102571	0.848948533
HECW2	221.0105333	0.210004734	0.222434614	0.944118948	0.345108868	0.848948533
MBOAT7	552.0752347	0.118656657	0.125707816	0.943908343	0.345216488	0.848948533
TYMP	234.0219063	0.391250976	0.414511395	0.94388473	0.345228556	0.848948533
EVL	966.9745874	0.198550402	0.210357589	0.943870879	0.345235635	0.848948533
LOC643072	8.598567185	0.607042633	0.643276338	0.943673188	0.345336679	0.848948533
LIPI	7.447699416	1.052522173	1.115386447	0.94363902	0.345354145	0.848948533
VCPKMT	302.6445714	0.16550957	0.175397218	0.94362711	0.345360233	0.848948533
TMEM99	124.0324092	-0.222876937	0.236207068	-0.943565912	0.345391519	0.848948533
HDAC5	1803.450245	0.251674105	0.266765444	0.943428433	0.345461805	0.849004189
DANT2	64.44188145	0.884112303	0.937327499	0.943226678	0.345564971	0.84914062
SLC19A1	802.3896311	0.21798266	0.231150003	0.943035509	0.34566274	0.849263757
TSPYL1	1804.81642	-0.147103154	0.156043731	-0.942704673	0.345831983	0.84935119
TAP1	427.6308682	0.246321472	0.261341736	0.942526345	0.345923229	0.84935119
RASD2	32.31235717	0.61726723	0.654930945	0.942492081	0.345940764	0.84935119
NAT9	561.7927337	0.118637663	0.125882725	0.942445938	0.345964377	0.84935119
KIF1C-AS1	13.25315483	0.794088591	0.842787405	0.942216965	0.34608157	0.84935119
MIR1322	5.67388196	1.024687827	1.087652304	0.942109738	0.346136459	0.84935119
CHST1	9.793387926	-0.778530483	0.826455764	-0.942011076	0.346186969	0.84935119
PDCD4-AS1	30.81082248	-0.333608933	0.3541596	-0.94197343	0.346206243	0.84935119
CDC42SE2	429.5415769	-0.123098197	0.130683982	-0.941953215	0.346216593	0.84935119
BCOR	1133.108309	-0.110150124	0.116939553	-0.941940696	0.346223003	0.84935119
DCDC1	8.157537148	0.774688018	0.822460979	0.941914617	0.346236356	0.84935119
DEPP1	9.232971021	0.932989263	0.990735695	0.941713585	0.346339298	0.84935119
FAM156B	43.3716822	0.433708632	0.460556822	0.941704934	0.346343728	0.84935119
ATXN3	589.3750992	-0.128745554	0.13673559	-0.941565796	0.346414988	0.84935119
CLVS1	5.065813204	0.914186467	0.970999964	0.941489703	0.346453964	0.84935119
TRMU	311.8558289	0.253220346	0.268962854	0.941469582	0.34646427	0.84935119
SOD2-OT1	10.2839307	-0.546114939	0.580119789	-0.941383054	0.346508594	0.84935119
SNORA31	4.345883235	-0.95484608	1.014840441	-0.940882962	0.34676484	0.849862391
SNORA11	3.224398861	1.197891734	1.273538983	0.940600759	0.346909493	0.849884925
DSCC1	465.6634675	0.132791384	0.141187367	0.940533041	0.34694421	0.849884925
TBX3	1347.892278	0.286117833	0.304229308	0.940467685	0.346977718	0.849884925
P3H3	1775.227571	-0.087082248	0.092596413	-0.940449477	0.346987054	0.849884925
HSPBAP1	102.2064431	-0.208753011	0.221984818	-0.940393191	0.347015914	0.849884925
C5	171.5689248	-0.369522581	0.392980849	-0.940306843	0.347060191	0.849884925
SEL1L3	205.0643954	-0.337995618	0.359527053	-0.940111783	0.347160226	0.849977069
ZBTB47	440.6107427	0.155126286	0.165041456	0.939923154	0.34725698	0.849977069
DYRK1A	1283.814999	-0.100001757	0.106405506	-0.939817499	0.347311181	0.849977069
LOC105379152	34.8642534	-0.376164807	0.400270819	-0.939775744	0.347332603	0.849977069
DUOXA1	1.978294794	1.698000285	1.806828219	0.939768522	0.347336308	0.849977069
SUMF1	473.2041349	-0.138699801	0.147631104	-0.939502567	0.347472775	0.850162492
RPL21P28	12.96019263	0.588328738	0.626370413	0.939266489	0.34759394	0.850162492
TARDBP	1954.605879	-0.180261328	0.191921713	-0.939244057	0.347605454	0.850162492
UTP23	864.7774545	-0.135908286	0.14471613	-0.939137095	0.347660361	0.850162492
METTL21EP	4.361385081	-0.992201654	1.05652444	-0.939118506	0.347669904	0.850162492
ALDH16A1	349.1190847	-0.127474457	0.135754041	-0.939010404	0.347725403	0.850162492

PCMTD2	589.8555568	-0.149686078	0.159415151	-0.938970212	0.347746039	0.850162492
RFXANK	211.9586389	-0.203023934	0.216262805	-0.938783412	0.347841957	0.85028034
LSG1	1357.444032	0.161858653	0.172483427	0.938401186	0.348038277	0.850484027
LRP5	208.5202519	-0.159772292	0.170269939	-0.938347032	0.348066097	0.850484027
UBA3	562.698844	-0.101382847	0.108044973	-0.938339331	0.348070054	0.850484027
MACROD1	278.803271	0.189065603	0.201508871	0.938249531	0.348116189	0.850484027
UBE2D3-AS1	16.66466176	0.453647623	0.483744853	0.937782841	0.348356021	0.850953296
MKLN1	2397.096039	-0.075326985	0.080343968	-0.93755619	0.348472535	0.85112124
COG1	648.0250756	0.126236971	0.134684737	0.937277481	0.348615843	0.85119553
UCKL1-AS1	71.32421628	0.519183204	0.553933532	0.937266248	0.34862162	0.85119553
LINC01678	5.793306212	-0.939505734	1.002515313	-0.937148512	0.34868217	0.85119553
CALCRL	344.9649753	-0.28812824	0.307585656	-0.936741473	0.348891557	0.85119553
RESF1	253.8617782	0.162006132	0.172948703	0.936729385	0.348897777	0.85119553
TNFAIP8L3	442.7243779	0.258093629	0.275549339	0.936651234	0.348937988	0.85119553
EIF2B5	2092.751233	0.092133417	0.098366447	0.936634598	0.348946549	0.85119553
FGF11	126.9499617	0.229307236	0.244831367	0.936592554	0.348968183	0.85119553
MIR631	6.950659518	0.867093001	0.925835671	0.936551731	0.348989191	0.85119553
APPBP2	1058.396487	-0.145568847	0.155435677	-0.93652146	0.349004769	0.85119553
TRIM26	558.8012634	-0.133591022	0.142652969	-0.936475579	0.34902838	0.85119553
ACTG1P4	7.296507935	0.772709194	0.825240813	0.936343891	0.349096157	0.851244323
DNAL4	109.3213133	-0.323028802	0.345110165	-0.936016483	0.349264701	0.851503373
EPB41	992.032067	0.163195793	0.174363443	0.935951886	0.349297961	0.851503373
ESYT3	11.6276987	0.649133015	0.693656441	0.935813432	0.349369255	0.851560678
SH2D2A	3.250690753	-0.932551351	0.996800155	-0.93554495	0.34950753	0.851713952
PDE4B	244.1352229	-0.24246822	0.259184108	-0.935505736	0.349527729	0.851713952
SMDT1	254.856889	-0.179169155	0.191560024	-0.935315998	0.349625473	0.851752794
ADAM21	7.649189346	0.79617261	0.851258182	0.93528923	0.349639264	0.851752794
GOLGA6L5P	30.73823672	0.376249665	0.402489102	0.934807088	0.349887726	0.852207097
NLGN2	812.2755495	-0.114370408	0.122355085	-0.934741763	0.349921399	0.852207097
LOC102723729	11.36956074	0.73705786	0.788803	0.934400426	0.350097378	0.852426634
DDX46	1071.360571	-0.193722672	0.207333364	-0.934353586	0.350121531	0.852426634
BIRC3	4.07545735	0.918583661	0.983190494	0.93428859	0.350155048	0.852426634
SHLD3	33.82179798	-0.425666708	0.455800472	-0.933888257	0.350361537	0.852593742
PRICKLE2	221.3496371	0.334528117	0.358211095	0.933885413	0.350363004	0.852593742
PPIP5K1P1-CATSPER2	20.23125259	0.587834901	0.629456295	0.933877229	0.350367226	0.852593742
TDRD3	271.0858067	-0.190009472	0.203499165	-0.933711308	0.35045283	0.852614805
HTR2B	29.60107653	0.75247402	0.805987649	0.933604902	0.350507736	0.852614805
CFAP298	44.49694468	0.321038338	0.343877934	0.933582259	0.35051942	0.852614805
ISX	2.301550984	-1.340799385	1.436625227	-0.933297954	0.350666151	0.852855304
C1orf226	16.68871249	-0.503730899	0.539885259	-0.933033251	0.350802801	0.852868113
SNHG5	176.3967953	0.164139453	0.175933915	0.932960841	0.350840187	0.852868113
ITGB5	4157.672458	-0.187730401	0.201253503	-0.932805633	0.350920332	0.852868113
FAM87B	18.05903087	-0.465652965	0.499221462	-0.932758307	0.350944773	0.852868113
LOC102724719	3.152849446	1.261058452	1.352013007	0.932726568	0.350961164	0.852868113
PLPPR4	6.17737186	0.778075742	0.83427133	0.932641113	0.351005298	0.852868113
TMEM140	399.0841257	-0.110969843	0.118996454	-0.932547476	0.351053663	0.852868113
SRRM5	18.61717059	0.488374409	0.523735917	0.932482179	0.351087392	0.852868113
NPHP3-AS1	6.567953533	0.671390963	0.720026142	0.932453593	0.351102159	0.852868113
SLC44A1	2212.868504	0.19299218	0.207007878	0.932293893	0.351184663	0.852891241
MGC4859	3.497481555	0.941658921	1.010092831	0.93224988	0.351207403	0.852891241
STYXL1	192.1670909	-0.202189969	0.216937471	-0.932019574	0.35132641	0.852960809
ZNF205-AS1	11.19581957	0.637807605	0.684436685	0.931872324	0.351402512	0.852960809
GRIA1	8.163139312	-0.630591214	0.67670796	-0.931851331	0.351413362	0.852960809
GOT2	1481.543588	-0.111568111	0.119746885	-0.931699484	0.351491853	0.852960809
MAPRE3	108.1761873	0.242809225	0.260630006	0.931624217	0.351530763	0.852960809
CACNA1I	2.212533384	-1.234473784	1.325455692	-0.931358016	0.3516684	0.852960809
CACNA1C-AS1	4.754753096	0.922677429	0.990689112	0.931349116	0.351673003	0.852960809
H1FX-AS1	63.04845786	-0.281384581	0.302162035	-0.931237378	0.351730787	0.852960809
PTBP1	4501.925809	0.100433786	0.107854133	0.93120016	0.351750035	0.852960809
CLCN7	1371.916831	0.224982438	0.241609839	0.931180779	0.351760059	0.852960809
ZFYVE28	14.06780667	-0.808704247	0.868540436	-0.931107192	0.351798119	0.852960809
PROSER2	7.617324865	-0.620113968	0.666141482	-0.930904297	0.351903072	0.852960809
BZW1	3488.368228	-0.147229702	0.158158385	-0.930900392	0.351905092	0.852960809
DNM2	2924.337964	-0.0908375	0.097580475	-0.930898317	0.351906165	0.852960809
RIPOR2	287.2081805	0.601593742	0.646507169	0.930529111	0.352097199	0.853199715
SNORD114-10	108.791958	-0.224188285	0.240927246	-0.930522754	0.352100488	0.853199715
EVI5L	227.538933	-0.193695721	0.208301941	-0.929879575	0.352433439	0.853431892
LIPE	44.83618427	0.40230135	0.432640768	0.92987388	0.352436388	0.853431892
CIDEB	8.171473761	0.827759554	0.890198799	0.929859212	0.352443984	0.853431892
OXSM	82.12797036	0.213255859	0.229360711	0.929783737	0.352483068	0.853431892
CIRBP	1510.282653	-0.203540565	0.218944721	-0.929643631	0.352555629	0.853431892
INTS5	553.7364069	0.142086627	0.152841723	0.929632465	0.352561412	0.853431892
TEX2	644.080337	0.117803895	0.126727206	0.92958646	0.352585241	0.853431892
KIFBP	407.6441447	-0.115503906	0.124260351	-0.929531463	0.352613728	0.853431892
SPAAR	2.278324572	1.15635711	1.24453276	0.929149595	0.352811565	0.853431892
RPS26	529.6863131	0.150222674	0.161681074	0.929129612	0.35282192	0.853431892
NUCB1	2071.627757	-0.206069869	0.221868776	-0.92879166	0.352997067	0.853431892
MYL6B	386.6469594	-0.184311536	0.198444921	-0.928779309	0.35300347	0.853431892
KAT8	253.0770226	0.181325416	0.19523555	0.928752047	0.353017601	0.853431892
FBP2	38.89090313	0.335198305	0.360936971	0.928689304	0.353050126	0.853431892
EYA1	433.7709261	-0.29795852	0.320922749	-0.928443124	0.353177758	0.853431892
LINC01588	22.79626263	-0.452306451	0.48718164	-0.928414401	0.353192651	0.853431892
RACGAP1	1782.434544	-0.106975509	0.115251571	-0.928191335	0.353308328	0.853431892
LOC107984974	41.621841	0.264289114	0.284738112	0.928183136	0.353312581	0.853431892
ANKRD20A9P	3.433301046	1.182066625	1.273580002	0.928144775	0.353332476	0.853431892
PCBP1-AS1	76.40872826	0.247929489	0.267160441	0.928017215	0.35339864	0.853431892
LINC00640	2.241577433	1.540752574	1.660412142	0.927933815	0.353441902	0.853431892
NANOS1	34.25973985	0.456450455	0.491916917	0.92790152	0.353458656	0.853431892
TFPT	82.14561587	-0.226152654	0.243736289	-0.927857951	0.353481259	0.853431892
GTF2IRD2	8.11209204	-0.733999088	0.7910837	-0.927839984	0.35349058	0.853431892

NPAS4	2.630005789	1.368886077	1.475382277	0.927817894	0.35350204	0.853431892
SLC16A14	24.86570247	0.480368017	0.51776579	0.927770869	0.353526438	0.853431892
SFTA3	2.209484615	1.55795108	1.679294761	0.927741285	0.353541787	0.853431892
RBM7	472.1871634	0.209735301	0.226081404	0.927698151	0.353564168	0.853431892
PSMD14	900.6319728	-0.177646785	0.191502063	-0.927649456	0.353589435	0.853431892
TM7SF2	27.77134525	-0.410484205	0.442539372	-0.927565389	0.353633058	0.853431892
DHCR24	4406.295353	0.130917396	0.141161547	0.92742959	0.353703533	0.853486386
HAGH	65.33252181	-0.28231369	0.304441217	-0.927317573	0.353761673	0.853511104
LOC105370802	48.68194024	-0.48293108	0.520951651	-0.927017084	0.353917664	0.853771867
MIR302CHG	4.567622444	0.875551053	0.944603633	0.926897825	0.353979587	0.853805662
PIAS2	1207.525559	0.166881588	0.180072295	0.926747714	0.354057538	0.85386821
SDHC	1871.37048	-0.116298869	0.125517226	-0.926557041	0.354156568	0.85386821
PHF3	5628.988394	-0.100749401	0.108742786	-0.926492737	0.35418997	0.85386821
EXOC4	1621.301662	-0.148001317	0.159765894	-0.926363652	0.354257027	0.85386821
GARS-DT	158.4793818	0.238297371	0.257281932	0.92621106	0.354336306	0.85386821
MCPH1	1040.268765	0.102271923	0.110421656	0.926194433	0.354344945	0.85386821
GTF3C1	2132.147367	0.09462635	0.102170804	0.926158416	0.354363659	0.85386821
REEP6	6.836000197	-0.682838787	0.737319359	-0.92610994	0.354388849	0.85386821
PGAP6	876.5313746	-0.132407715	0.143022914	-0.925779736	0.35456046	0.854013197
DPP4	1316.140069	0.126774574	0.136951556	0.925689179	0.354607533	0.854013197
RPS15	2836.526657	-0.145158359	0.156832581	-0.925562519	0.354673379	0.854013197
ERVH48-1	4.87694037	1.039149319	1.122764308	0.925527568	0.354691551	0.854013197
PLEKHA7	3.601855172	0.956572531	1.033591086	0.925484501	0.354713942	0.854013197
TIE1	2.716473215	-1.150099105	1.242779071	-0.925425228	0.354744761	0.854013197
ENPP3	18.43910187	-0.432668659	0.467573581	-0.925348814	0.354784495	0.854013197
TREH	3.496521536	0.96264239	1.040499965	0.92517292	0.354875967	0.854084186
LOC100288637	296.9339168	0.200029965	0.216223415	0.925107787	0.354909843	0.854084186
TPI1P2	12.06528544	-0.579794671	0.626853571	-0.924928401	0.355003152	0.854100744
ANKRD20A12P	11.60675543	0.550733828	0.595468756	0.924874433	0.355031227	0.854100744
EFNA1	16.62673751	0.461907106	0.499457228	0.924818144	0.355060511	0.854100744
LOC105371215	19.27186705	0.499437546	0.540338527	0.924304896	0.355327595	0.854627849
CEL	15.39670253	0.49803595	0.538970685	0.924050164	0.355460199	0.854702433
SLC25A10	466.3947699	0.193670904	0.209611895	0.923949967	0.355512366	0.854702433
ZBTB6	608.3553426	0.18445168	0.199644931	0.923898638	0.355539093	0.854702433
GPR61	3.051816187	1.14693309	1.241470595	0.923850387	0.355564217	0.854702433
KLHL11	943.5537357	0.124422212	0.134704674	0.923666623	0.355659915	0.854702433
ITGAD	5.524584541	0.916875022	0.992648101	0.923665719	0.355660386	0.854702433
ABRAXAS1	267.6464594	0.130230426	0.141002981	0.923600514	0.355694346	0.854702433
KCTD11	184.2258031	0.207758388	0.224976535	0.923466919	0.355763932	0.854754384
FSTL1	10585.71672	0.130663664	0.141519905	0.923288238	0.355857016	0.854841682
LINC01492	21.2906689	0.709054048	0.768046554	0.923191498	0.355907419	0.854841682
ERC1	824.9674669	-0.125146154	0.135568534	-0.923120947	0.35594418	0.854841682
TCN2	2.501578446	-1.089555006	1.180618852	-0.922867702	0.356076154	0.854998756
HDHD5-AS1	3.160591262	1.263770755	1.369653411	0.922693833	0.356166781	0.854998756
LINC02055	20.62406607	0.486526826	0.527302243	0.922671641	0.356178349	0.854998756
ENTPD1	561.3231465	0.119471843	0.129490914	0.922627227	0.356201502	0.854998756
SNORA7B	7.918873232	0.629116496	0.682063533	0.922372281	0.356334423	0.855202616
ATG10	98.755224	-0.194903243	0.211336793	-0.922239993	0.356403407	0.85525299
FANCA	623.5792227	0.349671256	0.379306036	0.921871057	0.356595838	0.855520121
ABHD1	34.77405464	0.391914024	0.425152041	0.921820871	0.356622019	0.855520121
ARHGAP22-IT1	4.462844435	0.969568399	1.052071918	0.921579963	0.356747714	0.855520121
NPAT	664.6032754	-0.104941701	0.113882334	-0.921492367	0.356793424	0.855520121
DEPDC1B	233.8722622	0.206216311	0.223827381	0.921318516	0.356884157	0.855520121
SLC35F2	1306.613017	-0.132375215	0.143700256	-0.921189834	0.356951325	0.855520121
ANKRD40	1837.822941	0.128698738	0.139719546	0.92112193	0.356986772	0.855520121
CHRNA5	274.8033458	0.179891897	0.19530039	0.921103625	0.356996328	0.855520121
DDO	8.092151223	0.578999337	0.628595693	0.921099752	0.35699835	0.855520121
RNF41	571.5642616	-0.130585685	0.14178032	-0.921042391	0.357028295	0.855520121
LIAS	163.3258232	-0.163449615	0.177466918	-0.921014561	0.357042825	0.855520121
ATP1A2	3.520315853	1.170552113	1.271281594	0.920765406	0.35717292	0.855716783
SYN3	6.162837853	-0.857671926	0.931809807	-0.920436681	0.357344608	0.856013027
ZNF569	201.9563505	0.194495605	0.211338096	0.920305466	0.357413154	0.856026224
PXDNL	15.58198038	-0.539326032	0.586173776	-0.920078745	0.357531611	0.856026224
SPX	19.47732668	-0.414184154	0.450175869	-0.920049658	0.35754681	0.856026224
TNFAIP8L2	10.3221254	0.753891148	0.819451195	0.919995178	0.357575279	0.856026224
LINC00319	3.308235111	1.141413347	1.240711958	0.919966427	0.357590304	0.856026224
LOC100505501	66.36560446	-0.295442736	0.321288362	-0.919556295	0.357804675	0.856375824
GABRQ	24.98111219	0.442217948	0.48093141	0.919503153	0.357832457	0.856375824
ACSBG1	15.44727443	0.623762172	0.678513135	0.919307438	0.357934788	0.856505697
LAG3	7.492515663	0.70013575	0.76215746	0.918623495	0.358292539	0.857246648
AGK	397.4342984	-0.128586061	0.140052279	-0.918129011	0.358551328	0.857410682
BSN	185.9600125	0.286948814	0.312560617	0.918058125	0.358588436	0.857410682
CBWD6	52.22798404	0.41297378	0.449884506	0.917955109	0.358642368	0.857410682
PSMB2	1297.577449	0.100864073	0.109892203	0.917845578	0.358699717	0.857410682
CYP2A6	1.990519727	-1.859632677	2.026327297	-0.91773559	0.35875731	0.857410682
PIP5K1C	722.2475642	-0.103393752	0.112673185	-0.91764294	0.358805829	0.857410682
LOC101927178	8.153824983	0.777224797	0.846993089	0.917628263	0.358813516	0.857410682
PARGP1	17.24920775	0.449017326	0.489344118	0.917590116	0.358833495	0.857410682
CLDN1	4.849155279	-1.17398993	1.279576911	-0.917482896	0.358889652	0.857410682
S1PR1	64.93017065	-0.310425583	0.338352102	-0.917463143	0.358899998	0.857410682
SMG8	505.64656	0.126271146	0.137636072	0.917427711	0.358918558	0.857410682
C9orf116	11.26318773	-0.602301724	0.656668641	-0.917207989	0.359033661	0.857410682
IL6ST	2895.570341	-0.133039238	0.145069397	-0.91707308	0.359104347	0.857410682
LINC02588	31.290827	-0.308250364	0.336131756	-0.917052194	0.359115291	0.857410682
SMURF2	613.3681652	-0.101579434	0.110776766	-0.916974178	0.359156172	0.857410682
ZNF530	124.2402665	0.220724788	0.240733448	0.916884586	0.359203122	0.857410682
CTNNBL1	402.7202598	-0.169575762	0.184967517	-0.916786712	0.359254417	0.857410682
RPS16	3779.844557	-0.185742447	0.202620245	-0.916702309	0.359298656	0.857410682
PLAA	596.8642462	0.098897108	0.107899146	0.916569883	0.359368072	0.857410682
TTC23L	13.22090869	0.534117305	0.58279523	0.91647508	0.359417772	0.857410682

KIAA0895	161.1516705	0.176830292	0.192954201	0.916436597	0.359437948	0.857410682
ANP32E	2933.3686	-0.137648782	0.150202692	-0.916420205	0.359446542	0.857410682
ZBED6	1907.035021	0.119530345	0.130437565	0.916379766	0.359467744	0.857410682
LOC101927296	4.977113166	1.22897339	1.341589118	0.916057959	0.359636496	0.857510407
LOC105371855	1.923520812	1.437586296	1.569383103	0.916019992	0.35965641	0.857510407
LINC01305	1.836382634	1.261479921	1.377193143	0.915978944	0.359677939	0.857510407
PRPF40B	111.2226174	-0.22277643	0.243243672	-0.915857039	0.359741882	0.857510407
ILF3	7489.716834	-0.127858796	0.139609529	-0.915831445	0.359755308	0.857510407
GPC1	2913.804132	0.240128574	0.262238628	0.915687273	0.359830942	0.857510407
G2E3-AS1	22.31251836	0.585104124	0.638998669	0.915657813	0.359846399	0.857510407
DVL2	1040.88739	-0.08392456	0.091668658	-0.915520764	0.359918307	0.857567085
ITFG2	225.2412939	0.206505816	0.225618288	0.915288464	0.360040214	0.857742862
SNX16	292.7123916	0.189408954	0.206960325	0.915194514	0.360089524	0.857745665
GLIS3	305.938711	0.188231443	0.205708475	0.915039807	0.360170733	0.85782444
ZC3H3	441.5382583	0.138821243	0.151744083	0.914837932	0.360276718	0.857854358
FAM214A	540.0572431	-0.132196738	0.144513048	-0.914773719	0.360310434	0.857854358
DIP2A	1002.656692	0.170248273	0.186122081	0.914712927	0.360342356	0.857854358
TIMP2	6460.921565	-0.282509671	0.308872178	-0.914649136	0.360375854	0.857854358
ARHGAP9	3.621442613	-0.914882253	1.000553649	-0.91437601	0.360519303	0.858081205
ZNF750	28.45300266	0.830206326	0.908239419	0.914083125	0.360673169	0.858308703
TTC32	58.02954397	0.293076402	0.320679484	0.913923142	0.360757232	0.858308703
CDC34	363.5526561	-0.134599564	0.147287298	-0.913857242	0.360791864	0.858308703
LINC02505	2.336624163	-1.386989559	1.517950147	-0.91372537	0.36086117	0.858308703
FEN1	702.2801162	-0.159654387	0.174739399	-0.913671374	0.36088955	0.858308703
SLC9A3-AS1	105.1850367	0.278580311	0.304911186	0.913644114	0.360903878	0.858308703
BRD1	744.6653	-0.136319242	0.149230469	-0.9134813	0.360989464	0.858397685
TMEM120A	98.75606853	-0.209873733	0.229821773	-0.913202135	0.361136241	0.858582708
HADH	552.3323253	-0.171985974	0.188348827	-0.913124743	0.361176939	0.858582708
GJB7	6.323904382	0.789022077	0.864221286	0.912986164	0.361249818	0.858582708
SIVA1	472.8972342	-0.141288139	0.154766436	-0.91291201	0.36128882	0.858582708
CLDN20	22.90532601	0.740916177	0.811629202	0.912875208	0.361308178	0.858582708
MRS2	771.6541195	0.103883128	0.113830072	0.912615857	0.361444611	0.85864878
SCN9A	7.008368824	-0.736581693	0.807117715	-0.912607516	0.361449	0.85864878
STRIP1	759.5949043	-0.088766454	0.097302853	-0.912269798	0.361626709	0.85864878
STEAP2	163.3700677	0.248065739	0.271922306	0.912266975	0.361628195	0.85864878
NPIPB3	31.46558732	0.561738853	0.615799489	0.912210651	0.361657838	0.85864878
SPRY4-IT1	12.26705249	-0.608728665	0.66733862	-0.912173591	0.361677344	0.85864878
TULP4	1735.637898	0.135224003	0.148259448	0.912076797	0.361728292	0.85864878
PPP1R3B	482.8972146	-0.113590792	0.124587395	-0.911735836	0.361907795	0.85864878
RPP25L	110.1803537	-0.204370988	0.224165318	-0.911697624	0.361927915	0.85864878
MOCS1	219.9503535	-0.126096111	0.138314915	-0.911659533	0.361947973	0.85864878
FAM89A	162.7597549	0.204882529	0.22475989	0.911561796	0.361999441	0.85864878
CORO2A	18.88539286	0.467287526	0.512644768	0.911523058	0.362019842	0.85864878
XPO6	3089.169034	0.127619777	0.140013812	0.911479909	0.362042567	0.85864878
ITGA5	6641.738159	0.15598222	0.171156133	0.911344617	0.362113825	0.85864878
SNRPN	29.21145493	-0.466652961	0.512216958	-0.911045513	0.362271394	0.85864878
CHPT1	1081.931441	0.104088641	0.114262343	0.9109619	0.362315449	0.85864878
ZNF490	374.1036421	-0.159809785	0.175438619	-0.910915658	0.362339815	0.85864878
LOC143666	14.66564985	0.506506717	0.556064965	0.910876872	0.362360253	0.85864878
TNFAIP2	2.868351157	1.020402348	1.120252271	0.91086836	0.362364739	0.85864878
RAB12	389.5185107	-0.120811734	0.1326595	-0.910690402	0.362458523	0.85864878
MIR487A	5.746919361	-0.904857253	0.993598381	-0.910687125	0.36246025	0.85864878
TTC27	681.8006074	0.140790114	0.154607132	0.910631431	0.362489604	0.85864878
MAD2L1BP	276.9021442	-0.12810909	0.140686743	-0.910598165	0.362507138	0.85864878
THAP4	445.1553865	0.124512807	0.136764125	0.910420088	0.362601008	0.85864878
NLN	1179.9846	0.110307198	0.121170233	0.910348979	0.362638496	0.85864878
WDR62	570.6924034	0.247101955	0.271448144	0.910309981	0.362659056	0.85864878
KLRB1	5.090356708	0.963345005	1.058322933	0.910256194	0.362687415	0.85864878
CCDC142	472.7088219	0.235102797	0.2583459	0.910031073	0.362806123	0.85864878
AGPS	2262.130188	-0.102966905	0.113155752	-0.909957317	0.36284502	0.85864878
USP33	1397.687853	-0.128086374	0.140766231	-0.909922597	0.362863332	0.85864878
CSNK1E	36.87837396	0.390610593	0.429320182	0.909835152	0.362909453	0.85864878
BTBD1	2009.211554	-0.111625529	0.122698615	-0.909753775	0.362952377	0.85864878
RNU5A-1	10.36217274	-0.578046472	0.635412235	-0.909718825	0.362970814	0.85864878
ACBD7	12.50524115	-0.540865705	0.594555571	-0.90969748	0.362982074	0.85864878
SYS1	301.540612	-0.124116965	0.136483513	-0.909391637	0.363143435	0.85864878
TPCN2	583.4579241	0.176079891	0.193642022	0.9093062	0.363188519	0.85864878
ADAM32	144.8636686	-0.197112043	0.21678121	-0.909267195	0.363209102	0.85864878
ATG2A	364.9118759	0.137331181	0.151041842	0.909226075	0.363230803	0.85864878
C7orf50	405.7426348	0.203787425	0.224145105	0.909176337	0.363257053	0.85864878
CYSTM1	74.50491817	-0.321263381	0.353443537	-0.908952484	0.363375208	0.85864878
COX11	733.382852	0.095648942	0.105236265	0.908897154	0.363404416	0.85864878
MIR4531	2.201491908	1.536544917	1.690608431	0.908870966	0.363418241	0.85864878
DHX30	1133.600095	0.110906941	0.12203193	0.908835427	0.363437003	0.85864878
PDIK1L	226.5596882	-0.232667192	0.256046773	-0.908690194	0.363513682	0.85864878
PRPF38A	554.4248458	-0.106734589	0.117472114	-0.908595113	0.363563887	0.85864878
MON1A	113.5311016	0.20831678	0.229275359	0.908587742	0.36356778	0.85864878
PXYLP1	68.19379684	-0.292443025	0.321905427	-0.908474976	0.363627329	0.85864878
NOCT	208.7145678	0.170162438	0.18733084	0.908352503	0.363692011	0.85864878
LOC101929536	2.704288306	-0.955427572	1.051835662	-0.90834301	0.363697025	0.85864878
SLC2A12	94.29087447	0.424267381	0.467167646	0.908169443	0.363788706	0.858751456
NSUN5P2	28.53155837	0.460634183	0.507276306	0.908053811	0.363849792	0.858781893
BMP8B	27.99525597	-0.377779109	0.416140596	-0.907816043	0.363975421	0.858964641
POM121L2	6.770306246	0.749782695	0.826008059	0.907718377	0.364027032	0.858972685
RAB22A	910.7471647	-0.137390883	0.151374122	-0.907624642	0.36407657	0.858975835
ASAP1-IT1	31.65798304	1.152432717	1.269921426	0.907483482	0.364151181	0.859038131
SNORD123	5.444731689	0.920257387	1.014191659	0.907380157	0.364205799	0.859053254
TSPAN17	560.5283058	-0.100598108	0.110924998	-0.906902054	0.364458595	0.859128293
SLC16A1	3458.62523	0.128638176	0.141866561	0.906754735	0.364536511	0.859128293
DCP1B	190.8236264	-0.159340855	0.175738876	-0.906690988	0.364570231	0.859128293

SEPTIN5	2.704157061	-1.063347636	1.172952531	-0.90655641	0.364641421	0.859128293
ZFP30	308.3098546	0.131122067	0.144654424	0.906450448	0.364697481	0.859128293
CHKB	24.4233406	0.412258843	0.454811912	0.906438096	0.364704016	0.859128293
ZNF687-AS1	11.53785811	-0.630991587	0.696129311	-0.9064287	0.364708987	0.859128293
LOC100996740	60.82307696	0.254970129	0.281312421	0.906359303	0.364745706	0.859128293
PKNOX2	4.395913888	-1.080775647	1.19292872	-0.905985101	0.364943738	0.859128293
LINC02220	2.246215479	1.499959179	1.655754608	0.905906691	0.364985242	0.859128293
TMEM41A	1168.204417	0.102184962	0.112800086	0.905894365	0.364991767	0.859128293
MIRLET7BHG	342.1666268	0.526102876	0.580764666	0.905879622	0.364999571	0.859128293
DPY19L2P1	359.6775113	0.212411908	0.234499775	0.90580858	0.365037179	0.859128293
KLHL31	91.22448254	0.4016684	0.44345004	0.905780502	0.365052043	0.859128293
COPZ1	2281.18987	-0.168037757	0.18551824	-0.905774856	0.365055032	0.859128293
TOR3A	872.9457771	0.121060519	0.133660782	0.905729543	0.365079022	0.859128293
LPAL2	4.737108616	0.950747979	1.04971791	0.905717593	0.365085348	0.859128293
SCML2	232.0729398	0.158139575	0.174616714	0.905638251	0.365127356	0.859128293
FAHD2B	162.0291773	-0.268035232	0.295982139	-0.905579076	0.365158688	0.859128293
ZNF331	95.45885072	0.336166639	0.371250731	0.905497579	0.365201842	0.859128293
GOLGA6L3	3.978468009	0.974103499	1.075931027	0.905358685	0.365275396	0.859187903
SEPTIN1	33.23679408	0.406585114	0.44938471	0.904759563	0.365592778	0.859383286
SHISA4	57.12298235	-0.252144205	0.278688684	-0.904752217	0.365596671	0.859383286
LOC105371046	15.33312214	0.498465742	0.550984122	0.904682588	0.365633568	0.859383286
DDR1	175.2917824	0.183788069	0.203164713	0.904625935	0.365663591	0.859383286
RAB3B	191.2109294	0.252955091	0.279624534	0.904624096	0.365664566	0.859383286
TET2	474.2035012	-0.134392416	0.148576724	-0.904532099	0.365713322	0.859383286
MACC1	3.105338875	-0.970975667	1.073515811	-0.904481943	0.365739905	0.859383286
CBY3	12.46587689	0.723372487	0.799771709	0.904473712	0.365744267	0.859383286
SLC5A10	4.392117733	0.88420677	0.977834218	0.904250183	0.365862756	0.85954836
MIF4GD	146.3786193	-0.269882212	0.298497016	-0.904137052	0.365922734	0.859559329
NBPF19	53.24860902	-0.306758034	0.339318318	-0.904042068	0.365973095	0.859559329
MAGEA12	713.4029432	-0.117120234	0.129566432	-0.903939639	0.366027409	0.859559329
ZNF614	240.7665009	0.140804933	0.155783793	0.903848404	0.366075791	0.859559329
ARID5A	148.6264405	-0.167811246	0.18568204	-0.903755936	0.366124831	0.859559329
ZNF665	3.745049282	1.000250662	1.106885741	0.903662072	0.366174616	0.859559329
NKG7	2.2164681	1.406981432	1.557351096	0.903445238	0.36628964	0.859559329
EPN1	1534.131521	-0.08394358	0.092916209	-0.90343311	0.366296074	0.859559329
CCDC184	1.849908214	-1.374996773	1.521985916	-0.903422796	0.366301546	0.859559329
LOC100506606	11.15868602	-0.863239461	0.956084291	-0.90289054	0.366583992	0.859992256
ATP5F1C	1128.860829	-0.105000063	0.116312417	-0.902741646	0.366663028	0.859992256
FDX2	209.8166765	-0.157731589	0.17475911	-0.90256576	0.366756406	0.859992256
ST6GALNAC2	25.13513894	0.477527282	0.529110267	0.902509952	0.366786037	0.859992256
LINC00999	9.333265888	0.56037495	0.620952392	0.902444305	0.366820894	0.859992256
ZBTB8B	27.61907918	0.34796107	0.385578745	0.902438412	0.366824024	0.859992256
ST7-AS1	33.75397839	-0.377557946	0.418376422	-0.902436005	0.366825302	0.859992256
IL13RA1	1178.409429	0.22155862	0.245544872	0.90231418	0.366889995	0.859992256
FOXRED1	348.3897937	-0.115267142	0.127772221	-0.902129909	0.366987862	0.859992256
SLC26A1	198.4618457	0.255511913	0.283282403	0.901968885	0.367073396	0.859992256
ACOXL	8.6839016	-0.582323711	0.645687645	-0.901865965	0.367128073	0.859992256
ABCC11	7.738815721	-0.693506991	0.768977684	-0.901855809	0.367133469	0.859992256
RRAGB	165.0281717	-0.20221505	0.224255671	-0.90171655	0.367207459	0.859992256
ATG13	988.4405215	-0.159753854	0.177196803	-0.90156172	0.367289734	0.859992256
OMA1	314.5291835	0.155519841	0.172518787	0.901466115	0.367340542	0.859992256
PER3	293.9504112	0.171213248	0.189928004	0.901463948	0.367341694	0.859992256
MRPS23	467.1057174	-0.127204622	0.141163791	-0.901113671	0.367527885	0.859992256
FRMD1	2.153543628	1.644770794	1.825454315	0.901019971	0.367577701	0.859992256
NRSN2	557.7654978	-0.115486767	0.128188032	-0.900916923	0.367632493	0.859992256
PTTG1IP	1932.435915	-0.145029873	0.160981635	-0.90090943	0.367636477	0.859992256
VPS25	708.4795189	-0.136719122	0.151762817	-0.90087364	0.367655508	0.859992256
LOC105373878	4.533815757	-0.830555788	0.922020863	-0.900799343	0.367695017	0.859992256
VPS9D1	187.4963973	0.15944031	0.177018802	0.900697037	0.367749425	0.859992256
FEM1A	778.3403823	0.143000721	0.158774357	0.900653759	0.367772442	0.859992256
OR3A3	2.688638051	-1.348522738	1.497334735	-0.900615412	0.367792837	0.859992256
TMEM208	266.7436597	0.140330446	0.155816931	0.900611029	0.367795169	0.859992256
GPR146	20.51004463	0.731798437	0.812607391	0.90055597	0.367824454	0.859992256
DIP2C	1194.765941	0.216224474	0.240107542	0.900531788	0.367837316	0.859992256
GPHN	681.4853054	-0.098428617	0.10934424	-0.900171947	0.368028753	0.860245483
ASPA	35.23197836	0.647922283	0.719846266	0.900084245	0.36807542	0.860245483
SNORD83A	22.63249097	0.489003546	0.543303467	0.900056001	0.368090449	0.860245483
ZEB2-AS1	6.216783555	-0.688250971	0.764862868	-0.899835773	0.368207654	0.860406555
ZMIZ1-AS1	18.22906368	0.536744794	0.596676652	0.899557225	0.36835593	0.860640181
C2orf76	43.95454438	0.269089707	0.299166671	0.899464187	0.368405463	0.860643072
CDC7	617.6478744	0.117525993	0.130703125	0.89918273	0.368555338	0.860774432
TBILA	10.06471489	0.647785952	0.720534171	0.899035712	0.368633639	0.860774432
CCZ1P-OR7E38P	85.10346169	-0.244784876	0.27228946	-0.8989877	0.368659212	0.860774432
C8orf58	107.6481688	0.218300444	0.242845178	0.898928467	0.368690764	0.860774432
MUTYH	227.3610151	-0.139171573	0.154823432	-0.898905099	0.368703212	0.860774432
QRFP	6.957121499	-0.683717215	0.760711427	-0.898786572	0.368766354	0.860809068
PMEPA1	1689.26304	-0.448501296	0.49909506	-0.898629002	0.368850305	0.860892265
C12orf57	282.0151624	-0.245079431	0.27278616	-0.898430594	0.368956032	0.861026256
LOC101928123	2.88940122	1.023849101	1.139739175	0.898318776	0.369015625	0.861052566
FANCB	254.2375292	0.275196068	0.306405192	0.898144271	0.36910864	0.861111458
MTMR12	341.1045843	0.125642114	0.139899229	0.898090112	0.36913751	0.861111458
NBPF8	370.6160666	0.205315257	0.228655527	0.897923875	0.369226135	0.86117856
EMC6	84.43902789	0.221890615	0.247134167	0.897854869	0.369262928	0.86117856
NECTIN1	148.9274739	0.19741737	0.219941066	0.897592133	0.369403035	0.861371389
MAGEA10-MAGEA5	3.552438115	-1.125448701	1.254124049	-0.89739823	0.369506457	0.861371389
SIPA1L3	860.3505677	0.164436966	0.183241849	0.897376701	0.36951794	0.861371389
ANKHD1	142.2815176	-0.190689344	0.212505759	-0.897337297	0.36953896	0.861371389
TTYH2	78.08807449	-0.246955996	0.275265221	-0.897156551	0.369635386	0.861483466
RCAN1	299.1639396	-0.146507739	0.163343624	-0.896929651	0.369756457	0.861652945
SNORD23	10.76973576	0.662733692	0.739105971	0.896669378	0.369895365	0.86186394

ENDOG	216.9499	-0.164350207	0.183335774	-0.896443741	0.370015815	0.861893073
MBL1P	15.3138322	0.518986029	0.579052582	0.896267533	0.370109895	0.861893073
LOC100291105	7.492639582	-0.67465748	0.752758422	-0.896247004	0.370120856	0.861893073
EXOC2	871.0826943	0.139212285	0.15533568	0.896202888	0.370144413	0.861893073
ANKS1A	722.1645307	0.152016257	0.169624467	0.896192985	0.370149702	0.861893073
MIR98	4.674767224	0.944241559	1.053768406	0.896061747	0.370219786	0.861929156
FRG1DP	9.207680878	-0.640082967	0.714392004	-0.895982826	0.370261936	0.861929156
PATE2	4.486250874	1.255874238	1.402012294	0.895765496	0.370378022	0.862086774
MVP	507.7715532	-0.286146312	0.319492836	-0.895626693	0.370452175	0.862146761
POLR1E	751.8169105	-0.101825709	0.113720151	-0.895406031	0.370570078	0.862243673
GABRA2	35.90339582	0.501395675	0.559992325	0.895361691	0.370593772	0.862243673
H3-4	2.257072814	1.270426337	1.419378409	0.895058237	0.370755957	0.862243673
SDC2	5422.52578	-0.115310017	0.128836182	-0.895012689	0.370780305	0.862243673
TMEM97	1566.33167	-0.076735541	0.085737447	-0.895006137	0.370783807	0.862243673
P4HA2-AS1	4.206766983	0.950402787	1.062037861	0.894885975	0.370848045	0.862243673
C19orf12	530.7963748	0.112990813	0.126273686	0.894808859	0.370889274	0.862243673
REEP3	1024.109464	-0.104722578	0.117035907	-0.894790166	0.370899268	0.862243673
TSPYL2	139.430321	-0.255755281	0.285850496	-0.894716937	0.370938423	0.862243673
TMEM42	163.1371504	-0.245106295	0.273974478	-0.894631854	0.370983918	0.862243673
TMEM54	26.2922533	0.518576955	0.579711617	0.894542975	0.371031447	0.862243673
ADGRL4	100.2385788	-0.264211673	0.295464178	-0.894225739	0.371201123	0.862243673
NFE2L3	121.2191209	0.362268212	0.405156804	0.894143228	0.371245263	0.862243673
TRRAP	2194.400946	0.104455583	0.116838617	0.894015915	0.371313376	0.862243673
LINC01715	67.96094182	-0.295240797	0.330258343	-0.893969233	0.371338353	0.862243673
FLJ42393	41.70384828	0.73966106	0.827452595	0.893901433	0.371374631	0.862243673
CCDC114	7.243769802	0.708967471	0.793198968	0.893807859	0.371424703	0.862243673
BACH1	1058.276244	-0.142680535	0.159636637	-0.893783142	0.371437931	0.862243673
TAF6L	154.2335394	0.178366634	0.199604072	0.893602178	0.371534781	0.862243673
PRMT2	508.0894242	-0.145022282	0.162296364	-0.893564577	0.371554906	0.862243673
LRRC32	94.5378994	-0.339594228	0.380046732	-0.893559133	0.37155782	0.862243673
TMEM60	90.92361783	-0.223072828	0.249645547	-0.89355821	0.371558314	0.862243673
BAK1	497.7595065	0.112114813	0.125525191	0.893165846	0.371768365	0.862300616
INO80C	94.34772353	0.190337819	0.213119661	0.893103049	0.37180199	0.862300616
GUSBP4	85.2644017	0.266912122	0.298912683	0.892943449	0.371887458	0.862300616
ZNF568	136.4339632	0.179200319	0.200691175	0.892915787	0.371902272	0.862300616
TRIM67	2.949447777	-0.938499977	1.051052853	-0.892914162	0.371903143	0.862300616
PIK3R2	770.6210756	-0.127349275	0.142635211	-0.892831958	0.371947169	0.862300616
ARPC5L	379.7159683	-0.167656607	0.187811202	-0.89268694	0.372024846	0.862300616
ANKRD36	996.767024	0.49113911	0.550181445	0.892685704	0.372025508	0.862300616
TMEM98	528.2534824	-0.114700498	0.128492617	-0.892662167	0.372038117	0.862300616
UAP1L1	339.0214023	-0.137825586	0.154407617	-0.892608721	0.372066747	0.862300616
EPB41L1	185.3757185	-0.177204214	0.19862388	-0.892159665	0.372307358	0.86266398
DNAJC14	799.9135764	0.096209386	0.1078434	0.892121228	0.372327958	0.86266398
CLDN7	5.924664167	0.805997703	0.90353919	0.892045095	0.372368762	0.86266398
DHX15	3353.959966	-0.108063248	0.121199402	-0.891615358	0.372599135	0.862747526
FAAP100	742.4964189	0.215833467	0.242078583	0.891584312	0.372615782	0.862747526
ISLR2	27.51521152	0.616983341	0.69203078	0.891554767	0.372631624	0.862747526
CPT1A	390.4753134	-0.192152153	0.215528787	-0.891538226	0.372640494	0.862747526
ANGPTL5	2.779441908	1.377720241	1.545350109	0.891526284	0.372646897	0.862747526
FRAT1	54.25202037	-0.320466862	0.359546751	-0.891307907	0.372764008	0.862821828
GINS1	895.3582753	0.112893579	0.126677888	0.891186148	0.372829314	0.862821828
FAM114A1	977.9334094	0.235176122	0.263902227	0.891148683	0.372849411	0.862821828
THOC6	398.6979048	0.192620082	0.216158601	0.891105331	0.372872665	0.862821828
LARS2-AS1	9.22106697	0.84580735	0.949721825	0.890584304	0.373152223	0.863056942
PSMB8-AS1	10.25867566	0.730977264	0.820841605	0.890521703	0.37318582	0.863056942
HLA-H	25.12794436	0.328002678	0.368333124	0.890505513	0.37319451	0.863056942
RAB5C	779.8275879	-0.141080014	0.158427319	-0.890503073	0.373195819	0.863056942
UBTD1	56.80110672	-0.255501557	0.286930594	-0.890464671	0.373216431	0.863056942
JUN	5801.761585	-0.182097813	0.204536786	-0.890293706	0.3733082	0.863157146
FBXW5	869.7680155	-0.11215153	0.126025411	-0.889912035	0.373513121	0.863445545
SLF1	346.4878592	0.17794128	0.19996078	0.889880906	0.373529837	0.863445545
TRIM27	1596.617004	-0.078830798	0.088641575	-0.889320814	0.373830688	0.864028906
NBPF12	127.9165972	0.251408504	0.282739161	0.889188829	0.373901605	0.864080742
ZNF141	700.9812441	0.215887399	0.242884474	0.888848083	0.37408473	0.864391843
SPATS2	439.1805619	0.216213781	0.243300768	0.888668716	0.374181149	0.864395546
METTL15	418.2051333	0.121626609	0.136864465	0.888664627	0.374183347	0.864395546
THBD	5.808686675	0.937335876	1.055019176	0.888453875	0.374296656	0.864412117
DDA1	305.4113828	-0.157927139	0.177788179	-0.888288182	0.374385755	0.864412117
LOC646762	258.3776967	-0.161997978	0.182379903	-0.888244675	0.374409152	0.864412117
POLR2D	1065.848629	-0.113912477	0.128248164	-0.888219166	0.374422871	0.864412117
ZNF816	94.62994837	0.215473885	0.242623875	0.888098444	0.3744878	0.864412117
ABCC10	920.4435519	0.209635683	0.236067248	0.888033747	0.374522599	0.864412117
C15orf54	6.67281363	-0.762813567	0.859044128	-0.88797949	0.374551784	0.864412117
ASAH2	12.37771305	0.479342665	0.539864618	0.887894203	0.374597663	0.864412117
TWIST2	25.55594963	-1.629337209	1.835170893	-0.8878395	0.374627092	0.864412117
ADO	718.4784669	-0.088407087	0.099595032	-0.887665641	0.374720633	0.864516013
CTSA	1118.787514	-0.10169482	0.114599405	-0.887393963	0.374866832	0.864591755
SHISAL1	685.098296	-0.276542874	0.311645801	-0.887362747	0.374883633	0.864591755
ISM2	15.70848828	0.5230673	0.589481755	0.887334164	0.374899017	0.864591755
LINC01798	5.241206421	0.790252183	0.89127006	0.88665851	0.375262782	0.865087718
ST13P4	3.810573437	0.868300966	0.979487769	0.886484745	0.375356371	0.865087718
COMMD8	173.5261793	-0.164926954	0.186053336	-0.886449862	0.37537516	0.865087718
PPP1R3D	188.1213372	0.14868921	0.167745815	0.886395947	0.375404202	0.865087718
LOC101929234	3.799050694	0.986284347	1.112707774	0.886382184	0.375411616	0.865087718
ITGB8	216.872515	0.222157614	0.250694417	0.886168971	0.375526481	0.865087718
PDZK1	17.40420296	0.628593401	0.709408919	0.886080487	0.375574157	0.865087718
ADAMTS10	6.180034266	-0.846076193	0.955014981	-0.885929761	0.375655378	0.865087718
MIR5087	7.437705159	0.660835152	0.745927374	0.885924253	0.375658346	0.865087718
TM2D1	252.9856251	-0.169104585	0.190883426	-0.885905019	0.375668711	0.865087718
CDC42EP1	718.2419374	0.156971403	0.177233728	0.885674559	0.375792921	0.865087718

TOLLIP	337.1320815	-0.146436965	0.165360099	-0.885564087	0.375852471	0.865087718
PEAK3	10.71752555	0.664593111	0.750571408	0.885449544	0.375914221	0.865087718
CYTH1	265.5628686	-0.130212722	0.147070207	-0.885377974	0.375952808	0.865087718
CEP295	396.7095001	0.216495947	0.244525236	0.885372615	0.375955697	0.865087718
UCK1	389.4395964	-0.128113692	0.144720461	-0.88524934	0.376022167	0.865087718
LOC102723345	8.468509767	0.605535656	0.684082886	0.885178782	0.376060214	0.865087718
LOC285847	6.510074779	0.690728536	0.780345258	0.885157601	0.376071637	0.865087718
CRYGS	47.71555653	0.409647456	0.462800192	0.88514971	0.376075892	0.865087718
IL1RL2	3.926376436	-0.96547978	1.090813748	-0.885100487	0.376102437	0.865087718
CENPO	911.0021499	0.174052355	0.196659813	0.885042819	0.376133538	0.865087718
GABRA5	497.7043394	0.131878902	0.149029561	0.884917738	0.376201001	0.865131221
TSC22D1	2252.862306	-0.095283016	0.107713485	-0.884596905	0.376374076	0.865143092
ESYT1	2013.830794	-0.107838997	0.121925326	-0.884467574	0.376443859	0.865143092
ZNF19	35.42571982	-0.291923374	0.33010396	-0.884337693	0.376513947	0.865143092
KC6	105.7210072	0.250519599	0.283316208	0.884240268	0.376566525	0.865143092
HBQ1	12.99508123	-0.620775105	0.702119385	-0.88414466	0.376618128	0.865143092
NEURL4	1109.98076	0.207558519	0.234759602	0.884132181	0.376624863	0.865143092
INTS4	431.1033356	0.118081442	0.133569233	0.884046718	0.376670994	0.865143092
SYNE1-AS1	7.524817519	0.69325671	0.784296987	0.883921171	0.376738768	0.865143092
FAM85B	3.934529852	0.840413141	0.95079583	0.883904951	0.376747524	0.865143092
CNPY3	529.9013315	-0.113344017	0.128241021	-0.883835891	0.376784809	0.865143092
SNX11	318.6760395	0.147300608	0.166672124	0.883774712	0.37681784	0.865143092
TNFSF9	89.66816069	0.213703529	0.24185831	0.883589774	0.376917701	0.865143092
GSAP	7.561881908	-0.715595256	0.80991271	-0.883546149	0.37694126	0.865143092
ZNF350	59.72071491	-0.218856959	0.247705238	-0.88353787	0.376945731	0.865143092
ATP5MC3	1878.938758	-0.137398384	0.1555237	-0.883456244	0.376989814	0.865143092
PRKCD	202.0887779	0.182517975	0.206614622	0.883373951	0.37703426	0.865143092
LGALS8	316.1833405	-0.132688636	0.150213631	-0.883332861	0.377056454	0.865143092
ACAT2	612.3194336	-0.158868985	0.179878166	-0.883203271	0.377126455	0.865143092
SEPT5-GP1BB	51.41614043	0.275719372	0.312205837	0.883133302	0.377164253	0.865143092
KLHL24	1978.479932	-0.41892682	0.474377013	-0.883109443	0.377177142	0.865143092
MAGEC2	126.389718	-0.323400145	0.366341821	-0.882782488	0.377353804	0.865276487
SPDYE16	3.470356184	0.983888977	1.114659612	0.882681105	0.377408594	0.865276487
FBXL14	147.7100655	-0.25451587	0.288351295	-0.882659017	0.377420531	0.865276487
MIR611	1.994544215	1.252322256	1.418973529	0.882555052	0.377476723	0.865276487
TTC33	396.2042237	0.181358866	0.205493564	0.882552538	0.377478082	0.865276487
ANKRD19P	19.55771698	-0.43256946	0.490263277	-0.882320745	0.377603381	0.86545238
TRIM21	115.7983173	-0.193096341	0.2189118	-0.882073697	0.377736955	0.865478768
MYL9	458.7688323	0.17561265	0.199092087	0.882067453	0.377740331	0.865478768
UBXN7	4062.158497	-0.150351411	0.170460659	-0.882029975	0.377760598	0.865478768
LOC101926948	3.637479578	0.892258492	1.011700818	0.881939084	0.37780975	0.865480106
ZKSCAN3	350.508725	0.103514046	0.117390754	0.881790454	0.377890135	0.86549093
SOCS2	21.58274444	-0.634760599	0.719905546	-0.881727612	0.377924125	0.86549093
DNAJB11	1101.454876	0.109672716	0.1243933	0.881660957	0.37796018	0.86549093
CCDC181	9.853493149	0.563236111	0.639085373	0.881315916	0.378146854	0.865782627
CRB2	5.02153487	0.801486485	0.909623871	0.88111857	0.378253647	0.865782627
CP	14.35281259	0.652110921	0.740170492	0.88102799	0.37830267	0.865782627
PARS2	105.754321	-0.195276634	0.221654145	-0.880996985	0.378319452	0.865782627
ULK3	425.6782886	0.168683646	0.191474961	0.880969736	0.378334201	0.865782627
PCDH9-AS4	4.424567924	1.508303916	1.712300095	0.880864237	0.378391306	0.865782627
IMPDH1	1548.637948	0.172971013	0.196381178	0.880792216	0.378430293	0.865782627
FMOD	20.34538899	-0.527407161	0.598844971	-0.880707339	0.378476244	0.865782627
IQCK	34.85179496	-0.299365427	0.340027551	-0.880415206	0.378634422	0.865888622
ZNF254	197.0226212	0.189563456	0.215315573	0.880398261	0.378643598	0.865888622
FHOD3	101.5598056	0.173862417	0.197491808	0.880352552	0.378668352	0.865888622
FST	83.56786288	-0.890982906	1.01232817	-0.880132483	0.378787544	0.866050043
AMOTL1	1240.566766	0.157136191	0.178577907	0.879930746	0.378896828	0.866054003
ARLNC1	3.147908048	1.102199586	1.252696088	0.879861921	0.378934115	0.866054003
WDR35	706.4640012	-0.223991835	0.254576635	-0.879860148	0.378935076	0.866054003
HPRT1	965.4512801	-0.156912728	0.178383938	-0.879634849	0.379057154	0.866141661
ZNF625-ZNF20	13.81310119	-0.518382714	0.58934702	-0.879588249	0.379082407	0.866141661
MIR10525	4.911519492	-0.847945137	0.964333198	-0.879307213	0.379234726	0.866141661
APH1B	327.2430066	-0.127201413	0.144703587	-0.879048093	0.3793752	0.866141661
CDNF	12.79138838	-0.585658478	0.666404756	-0.878832981	0.379491841	0.866141661
NFKBIE	604.2463198	0.322052984	0.366485858	0.878759649	0.379531609	0.866141661
SYNE2	974.2476356	-0.137138266	0.156094858	-0.87855723	0.379641394	0.866141661
NPPA	4.031262155	0.948976337	1.080170256	0.878543296	0.379648952	0.866141661
GAMT	105.033563	-0.181939426	0.207114509	-0.878448483	0.379700383	0.866141661
TRAF3	1207.27163	0.093407853	0.106337115	0.878412517	0.379719894	0.866141661
KIAA0319	4.699680179	0.935337193	1.064830349	0.87839081	0.37973167	0.866141661
DEPDC1	1305.667971	0.137187827	0.15619195	0.87832841	0.379765522	0.866141661
N4BP1	662.5532322	0.109266535	0.124417278	0.878226371	0.379820884	0.866141661
PMM2	783.7361368	0.113646753	0.129409164	0.878197106	0.379836762	0.866141661
NSDHL	192.259452	0.146530813	0.166860248	0.878164896	0.379854239	0.866141661
APPL1	1105.762218	-0.126832753	0.14443599	-0.878124297	0.379876269	0.866141661
HMOX1	121.2387211	-0.198402945	0.22594855	-0.878089039	0.379895401	0.866141661
PLEKHG7	5.093478933	1.011899077	1.152412194	0.878070436	0.379905496	0.866141661
WDR44	227.6265453	0.16592017	0.18900142	0.8778779	0.380009984	0.866141661
SNORD113-9	7.722867832	0.59785788	0.681050736	0.877846316	0.380027126	0.866141661
AKR1A1	604.1215184	-0.130060819	0.14816113	-0.877833604	0.380034025	0.866141661
TTPAL	957.1057545	-0.093937403	0.107023286	-0.877728633	0.380091002	0.866141661
MIR6075	5.67141363	0.776627481	0.884815663	0.877727999	0.380091346	0.866141661
ADSS1	137.4285909	0.25321685	0.288525769	0.877622997	0.380148345	0.866160786
LINC02021	67.30342605	0.274853017	0.313266589	0.877377374	0.380281699	0.866172304
CLCN2	303.7588047	0.2488732	0.283675238	0.87731732	0.380314307	0.866172304
LOC102725021	46.64518525	0.367630461	0.419081124	0.877229825	0.38036182	0.866172304
LOC107985770	20.49650719	0.4752454	0.541788593	0.877178675	0.380389597	0.866172304
CELSR1	317.7489246	0.135203681	0.154140325	0.877146725	0.380406948	0.866172304
MEGF11	36.11402729	0.5408513	0.616673495	0.87704645	0.380461409	0.866172304
ZNF182	123.6961243	0.165661817	0.188898808	0.876987096	0.380493647	0.866172304

MRPL36	88.39901451	0.210612255	0.240272032	0.876557514	0.380727024	0.866460715
LAPTM4A	3500.020573	-0.135769027	0.154917394	-0.876396276	0.380814642	0.866460715
SCARNA10	589.7530737	-0.529263937	0.603957228	-0.876326853	0.380852371	0.866460715
VPS36	704.625406	-0.084848518	0.096827632	-0.876284143	0.380875583	0.866460715
RNGTT	615.8116279	0.116709895	0.133193999	0.876239893	0.380899633	0.866460715
LRATD1	240.8551426	0.242218645	0.276458223	0.876149176	0.380948942	0.866460715
MROH9	26.12181007	0.394606641	0.450398626	0.876127542	0.380960701	0.866460715
UBN2	959.7456379	0.083634535	0.095485737	0.875885103	0.381092499	0.86662085
CD46	2999.781864	0.08670225	0.098999565	0.875784157	0.381147384	0.86662085
NCBP2-AS1	36.2612346	0.495576086	0.565968673	0.875624588	0.381234154	0.86662085
MICA-AS1	26.96575768	0.381975132	0.436251474	0.875584738	0.381255825	0.86662085
TBC1D31	673.615769	0.11105943	0.126845212	0.875550825	0.381274268	0.86662085
ADAMTSL4	46.87121434	0.282998936	0.323290733	0.875369775	0.38137274	0.866716302
KCNA7	5.467260771	0.79943444	0.913331674	0.875294773	0.381413538	0.866716302
ZNF781	29.2246125	-0.368438992	0.420997139	-0.875157946	0.381487972	0.866754452
PCDHA2	2.311878981	-1.029726886	1.176716286	-0.875085097	0.381527605	0.866754452
SETMAR	136.7947816	0.240053408	0.274386554	0.874873076	0.38164297	0.866843514
EPB41L4B	66.88753915	0.315037449	0.360148331	0.874743604	0.381713429	0.866843514
SLC44A2	304.1451142	-0.308480409	0.352692688	-0.874643621	0.381767845	0.866843514
SNORD159	23.34514927	0.525555185	0.601024093	0.874432808	0.381882597	0.866843514
SULT4A1	3.564959139	0.970285689	1.109640684	0.874414306	0.381892669	0.866843514
AHCY	2054.541536	-0.110717589	0.12662281	-0.874388976	0.381906459	0.866843514
MTHFD2L	104.5938235	0.275690462	0.315295558	0.874387396	0.381907319	0.866843514
ANK1	50.04423113	-0.367274709	0.42010205	-0.87425117	0.381981485	0.866901434
TONSL	760.9364097	0.307774268	0.352148138	0.873990902	0.382123208	0.866961278
MIR378J	6.381740549	0.845623935	0.967608572	0.873931835	0.382155376	0.866961278
NBPF14	25.61299805	0.375804401	0.430017113	0.873928944	0.382156951	0.866961278
TBX18	440.1536169	-0.11313147	0.129472262	-0.873789246	0.382233038	0.866961278
TMF1	676.6053822	0.121758594	0.139350784	0.87375607	0.382251109	0.866961278
LOC101927543	20.42662155	0.776101407	0.888632164	0.873366324	0.38246344	0.867332464
MIR3186	10.93354506	0.711232114	0.814458247	0.873257919	0.382522512	0.867356045
ADGRV1	76.24098613	-0.216783027	0.248344488	-0.872912578	0.38271073	0.867672418
EIF3E	16183.20926	-0.15864703	0.181768385	-0.872797708	0.382773349	0.867703992
UBR3	1259.708733	0.099473251	0.114006239	0.872524633	0.382922236	0.867931092
HP09053	30.2720942	0.550985975	0.631624958	0.872330911	0.383027879	0.86806013
COMMD3-BMI1	5.866115495	0.683808916	0.784088995	0.872106253	0.383150415	0.868166368
PLA2G7	2.977277951	-1.03998995	1.19261069	-0.87202803	0.383193086	0.868166368
LOC283922	158.3767629	0.303893747	0.348511186	0.871977024	0.383220912	0.868166368
AMIGO2	271.6481437	-0.264808176	0.303757545	-0.871774807	0.383331241	0.868305924
SOX10	2.941898783	1.10888928	1.272179517	0.871645287	0.383401917	0.868355638
ARRDC3	431.371634	-0.23437029	0.268966743	-0.871372747	0.383550661	0.868416324
C6orf226	10.3800423	0.575150349	0.660061048	0.871359326	0.383557987	0.868416324
NCOR2	4395.363916	0.171280344	0.196573828	0.871328324	0.38357491	0.868416324
FIRRE	379.3445975	0.239641516	0.275094877	0.871123149	0.383686916	0.868454286
CAB39L	70.84336172	0.223587228	0.256666669	0.871119063	0.383689147	0.868454286
SNORD113-7	2.796969782	-1.068973246	1.227451571	-0.870888328	0.38381513	0.868457484
USP17L2	1.986175491	1.302259656	1.495427798	0.870827503	0.383848346	0.868457484
SYPL1	1582.49015	-0.092585331	0.106321469	-0.870805596	0.383860309	0.868457484
MCM8-AS1	16.32017268	0.503516777	0.57825013	0.87075947	0.383885499	0.868457484
LOC101927359	2.36311059	-1.085249725	1.246847132	-0.870395173	0.384084484	0.868592068
CACNG8	9.042654167	-0.896985968	1.030701838	-0.870267167	0.384154418	0.868592068
GPR75	33.34269077	0.450940295	0.518184185	0.870231682	0.384173805	0.868592068
DUSP28	69.3690117	0.287019775	0.329820671	0.870229794	0.384174837	0.868592068
DTL	1223.692181	-0.169555649	0.194845769	-0.870204417	0.384188703	0.868592068
CHAF1B	331.8204377	-0.117257396	0.134780842	-0.869985627	0.384308259	0.868615663
HOXC12	351.2757376	-0.168395754	0.193562954	-0.869979253	0.384311743	0.868615663
MIR4762	3.022003174	1.513210573	1.739558289	0.869882075	0.384364852	0.868615663
PYGL	3002.369863	-0.135968467	0.156392105	-0.869407482	0.384624292	0.868615663
DNMBP-AS1	5.233238699	1.043066386	1.199863771	0.869320677	0.384671756	0.868615663
ANAPC11	555.8869851	0.126389408	0.145404439	0.869226617	0.384723192	0.868615663
SNORA11F	9.504379213	0.704885525	0.810990026	0.8691667	0.384755958	0.868615663
SCIMP	7.223038714	0.760011388	0.874499976	0.869081085	0.384802782	0.868615663
PTGER3	3.013322076	1.018066559	1.171527368	0.869007918	0.3848428	0.868615663
RAD51D	649.3140674	0.124852033	0.143681101	0.868952371	0.384873183	0.868615663
CSNK1G3	993.4595718	-0.116169094	0.133690959	-0.868937547	0.384881291	0.868615663
SNORD157	18.62365457	0.423961123	0.487924116	0.868907909	0.384897504	0.868615663
RORA-AS1	15.61049027	0.597170454	0.687325307	0.868832338	0.384938843	0.868615663
MYBPH	51.82365511	0.900066796	1.035951935	0.868830653	0.384939764	0.868615663
DIAPH1	3819.262458	-0.099208895	0.114196362	-0.868757052	0.384980028	0.868615663
TMEM53	25.16305396	0.340423328	0.39195664	0.868522927	0.385108127	0.868615663
C4orf47	2.219501258	-1.329764223	1.531071951	-0.86851844	0.385110582	0.868615663
ZNF497	71.61404625	0.220309015	0.253675518	0.868467786	0.3851383	0.868615663
ODC1-DT	28.88896985	-0.420076624	0.483743802	-0.868386576	0.385182741	0.868615663
MIRLET7F1	9.94317689	0.73728338	0.849157774	0.868252523	0.385256107	0.868615663
THBS1	22988.22715	-0.1929118	0.222202433	-0.868180414	0.385295575	0.868615663
TAL1	53.15189679	-0.271673643	0.312949925	-0.868105793	0.38533642	0.868615663
RORB	24.94135857	-0.428517654	0.493636328	-0.868083708	0.385348509	0.868615663
MIR26A2	3.850989427	0.890577013	1.025955669	0.868046291	0.385368992	0.868615663
OTUB2	175.4109402	0.139828315	0.16110683	0.867922951	0.385436514	0.868632992
ZNF845	414.4318181	0.131995893	0.152096743	0.86784168	0.385481009	0.868632992
ACTR3	3235.33962	-0.118407392	0.136450966	-0.867765146	0.385522914	0.868632992
PDK2	71.33026337	-0.237882131	0.274171102	-0.867641151	0.385590811	0.86865425
RXRB	1184.923517	-0.103414162	0.119209449	-0.86749971	0.385668271	0.86865425
MRPL4	627.5378161	-0.135180827	0.155841451	-0.867425364	0.38570899	0.86865425
TOMM34	1315.734934	-0.169002515	0.194853041	-0.867333221	0.385759459	0.86865425
DHX36	1996.73988	-0.135869533	0.15666164	-0.867280161	0.385788524	0.86865425
PRR34-AS1	27.53159276	0.491796962	0.567108873	0.86720026	0.385832294	0.86865425
SLC25A40	335.0345409	0.132261004	0.15252824	0.867124699	0.385873689	0.86865425
TFAM	1061.394199	-0.13394273	0.154486779	-0.867017425	0.385932462	0.86865425
SCRN1	4814.503484	-0.103016652	0.118836814	-0.866874904	0.386010556	0.86865425

ZNF613	57.61711087	0.258760578	0.298503991	0.866858016	0.386019809	0.86865425
ADCY10P1	41.90062452	0.323926369	0.373785116	0.866611202	0.386155074	0.86883228
HERC2P3	87.89755472	0.341354553	0.393930149	0.86653574	0.386196436	0.86883228
COL4A2-AS1	2.923073493	1.050774596	1.213084136	0.866200921	0.386379989	0.868925576
SCAND1	200.2443128	-0.164155627	0.189512711	-0.866198505	0.386381313	0.868925576
EDEM2	33.9117225	0.374032004	0.43198526	0.865844366	0.386575515	0.868925576
COPS5	881.7633817	-0.104804292	0.121048786	-0.865802094	0.3865987	0.868925576
CH17-340M24.3	5.859919099	-0.925716782	1.069253	-0.865760285	0.386621633	0.868925576
CD27-AS1	5.835621809	0.801767436	0.926162474	0.865687672	0.386661462	0.868925576
LOC101929657	3.883568849	1.028685184	1.188305121	0.865674283	0.386668807	0.868925576
EEF1AKMT4	125.9264334	0.190145218	0.219663811	0.865619226	0.386699009	0.868925576
HCN2	44.03203079	0.300706738	0.347393003	0.865609658	0.386704257	0.868925576
CALM1	1677.823325	-0.162289182	0.187503878	-0.865524399	0.38675103	0.868925576
ACAD11	46.73336544	-0.285726068	0.330147177	-0.865450587	0.386791526	0.868925576
TRIM61	65.28755046	0.252962977	0.292309892	0.865393147	0.386823041	0.868925576
FAN1	1299.718043	0.092133781	0.106488016	0.865203289	0.38692722	0.869046727
RPS6KB2	405.57579	-0.143850184	0.166304176	-0.86498239	0.387048454	0.869046727
POLR3F	190.0771165	0.205352912	0.237414403	0.864955575	0.387063172	0.869046727
KLF13	2795.816099	-0.234040498	0.27060852	-0.86486744	0.38711155	0.869046727
RPS15A	2219.349233	-0.172151531	0.1990535	-0.864850563	0.387120815	0.869046727
PUDP	557.8855408	-0.115657989	0.133747078	-0.864751518	0.387175186	0.869059305
EFHC1	569.4106222	-0.121008988	0.140008415	-0.864297963	0.387424229	0.86936958
PTDSS1	4231.372275	-0.104847651	0.121327753	-0.864168739	0.387495202	0.86936958
PRMT3	626.1977766	0.103737078	0.120043879	0.864159666	0.387500186	0.86936958
RUNDC1	223.7116697	0.154502129	0.178792024	0.864144417	0.387508562	0.86936958
SEBOX	11.60796405	0.61649333	0.713595056	0.863926012	0.387628537	0.86952927
LINC02604	8.95617743	-0.738387705	0.854871494	-0.863741171	0.387730092	0.869542473
PRKDC	42189.76954	-0.094586626	0.10950851	-0.863737672	0.387732014	0.869542473
PNRC2	1659.315524	-0.085551896	0.099063593	-0.863605824	0.387804464	0.869573546
PGAP2	350.2392481	-0.107206488	0.124161671	-0.863442692	0.387894115	0.869573546
MAP10	74.49491208	-0.223685312	0.259069157	-0.863419306	0.387906969	0.869573546
ACYP2	53.26582365	0.263972225	0.305762191	0.863325266	0.387958656	0.869573546
ZNF346	282.5222481	-0.114489132	0.132640111	-0.863156186	0.3880516	0.869573546
PHTF1	400.3200178	0.116972131	0.135519833	0.863136624	0.388062354	0.869573546
LRRTM4	4.344229453	-1.007344316	1.167357733	-0.862926837	0.388177694	0.869573546
ARHGAP35	5349.438004	0.189022229	0.219050429	0.862916494	0.388183381	0.869573546
METTL11B	4.99335077	-0.874382471	1.01339456	-0.862825305	0.388233524	0.869573546
LOC101929089	3.946402298	-0.907922834	1.052354827	-0.862753522	0.388272998	0.869573546
BORCS8	68.31475148	0.267706296	0.310301157	0.862730577	0.388285617	0.869573546
PBX2	994.9770029	-0.102910808	0.119297916	-0.862637092	0.38833703	0.869573546
MAB21L3	2.539105075	-1.079096824	1.251069636	-0.862539376	0.388390774	0.869573546
TYMS	1092.733063	-0.109077847	0.126487343	-0.862361752	0.388488481	0.869573546
NOP53	981.2158548	-0.258583752	0.299879035	-0.862293532	0.388526011	0.869573546
LINC00622	31.65742976	0.478375354	0.554784654	0.862272146	0.388537777	0.869573546
EDN1	6.343802557	0.704084543	0.816652826	0.86215895	0.388600055	0.869573546
RGS9BP	2.46158616	1.033167535	1.198410318	0.86211502	0.388624227	0.869573546
UQCRFS1	969.4249679	0.113511034	0.131688092	0.861968856	0.388704656	0.869644316
RAB2A	2291.026898	-0.104063374	0.120748635	-0.861818221	0.388787556	0.869648511
DHX34	819.7075928	0.231287907	0.268390773	0.86175804	0.38882068	0.869648511
LOC101926944	14.00774718	0.629941102	0.731045046	0.861699434	0.388852937	0.869648511
DGKQ	305.859249	0.195762449	0.227228378	0.861522888	0.388950121	0.869756702
PBRM1	1515.200307	-0.127181496	0.147656815	-0.861331705	0.389055379	0.869780108
ZNF324B	246.9422083	-0.131669841	0.15288676	-0.861224615	0.389114346	0.869780108
LOC100420587	4.292953781	0.878162068	1.019943983	0.860990488	0.389243282	0.869780108
LHFPL6	162.754117	-0.196353405	0.228132576	-0.860698669	0.389404027	0.869780108
PICALM	2551.888426	-0.134226803	0.155960151	-0.860648072	0.389431901	0.869780108
CR1L	4.697711793	0.921634454	1.070953272	0.860573919	0.389472756	0.869780108
MAST1	18.70132186	-0.455255686	0.529098281	-0.860436904	0.389548251	0.869780108
GPR34	3.237633964	1.293469006	1.503296232	0.860421904	0.389556516	0.869780108
MIR3654	27.94384197	-0.436035048	0.506800824	-0.860367679	0.389586397	0.869780108
SNORD80	168.9333803	0.312196048	0.362895344	0.860292239	0.38962797	0.869780108
SND1-IT1	53.34481162	0.657472179	0.764290073	0.860239066	0.389657274	0.869780108
ECSIT	517.53069	0.159820032	0.18578695	0.86023282	0.389660717	0.869780108
RPL6	9583.084336	-0.141542156	0.164543804	-0.860209573	0.389673529	0.869780108
ZMAT1	2.471129683	-1.372986068	1.596321118	-0.860093907	0.389737279	0.869780108
ISY1	207.3043474	-0.177436894	0.206311933	-0.860041838	0.38976598	0.869780108
ACBD5	430.5948742	0.177829434	0.206771324	0.860029477	0.389772794	0.869780108
DPYSL3	2991.06075	-0.19493238	0.226688678	-0.859912288	0.389837394	0.869780108
NDUFA6-DT	36.25937358	0.33819655	0.393298192	0.859898562	0.389844961	0.869780108
PPT2-EGFL8	24.54789906	0.366495913	0.426334002	0.859645045	0.389984736	0.869780108
LOC729737	5.620356101	-0.797323313	0.927638827	-0.859519126	0.390054173	0.869780108
NOVA2	2.134617748	1.167900046	1.358902248	0.859443752	0.39009574	0.869780108
LATS1	1093.732814	0.110934089	0.129082158	0.859406839	0.390116098	0.869780108
CCDC62	5.820961341	0.678148145	0.789187294	0.859299371	0.390175371	0.869780108
ARMCX5	205.4331213	-0.159607568	0.185756507	-0.85923002	0.390213624	0.869780108
NSF	977.1360583	-0.132572031	0.154297584	-0.859197059	0.390231806	0.869780108
ZNF410	846.9825708	-0.109565422	0.127531401	-0.859125055	0.390271526	0.869780108
ZYX	376.4604448	0.119882185	0.139580703	0.858873628	0.390410241	0.869780108
ILVBL	597.7284715	-0.119506892	0.139150697	-0.858830716	0.390433918	0.869780108
ZNF766	322.9310841	-0.134002828	0.156030819	-0.858822821	0.390438275	0.869780108
LINC00941	18.88322898	-0.412375384	0.480165638	-0.858819024	0.39044037	0.869780108
TRG-AS1	138.6038376	-0.215116432	0.250496955	-0.85875867	0.390473674	0.869780108
UBAP2	605.88792	0.090389816	0.105275706	0.858600894	0.390560745	0.869865326
PCNA	1583.113019	-0.108055249	0.125904218	-0.858233747	0.390763405	0.87012902
MIR3150BHG	5.125492306	1.085358027	1.264677265	0.858209487	0.390776798	0.87012902
LOH12CR2	4.724462323	0.817907353	0.953284988	0.857988286	0.390898932	0.870292224
EPS8L1	46.01131152	0.263293287	0.306911881	0.857879096	0.390959228	0.870317732
MIR3685	11.32687005	0.621879155	0.725267123	0.857448429	0.391197105	0.870462096
FMR1-AS1	3.321067038	-1.09377947	1.275697655	-0.8573971	0.391225461	0.870462096
ZBED8	206.5614573	0.193750381	0.225978221	0.857385194	0.391232039	0.870462096

VPS13B	3449.388094	0.111933682	0.130558566	0.857344603	0.391254465	0.870462096
GRIN2C	12.24085115	0.479312157	0.559082277	0.857319534	0.391268316	0.870462096
FABP6	2.727689704	1.070500098	1.248966282	0.857108885	0.391384711	0.870562753
RRM1	1772.699026	-0.095129823	0.11101805	-0.856886085	0.391507844	0.870562753
GTF2H2C	5.935153202	0.832500483	0.971620884	0.856816169	0.391546488	0.870562753
C7orf25	69.57993819	0.219839103	0.256605294	0.85672084	0.391599183	0.870562753
TMEM41B	1169.614169	0.15494754	0.180916163	0.856460463	0.391743133	0.870562753
PCDHA6	3.367364991	1.142965098	1.334543439	0.85644653	0.391750837	0.870562753
AKAP13	3002.385805	-0.106173132	0.123975184	-0.856406328	0.391773065	0.870562753
CNTNAP3	107.0347554	0.181568418	0.212015779	0.85639106	0.391781508	0.870562753
BTBD6	737.0040485	-0.130238583	0.152125785	-0.85612431	0.391929023	0.870562753
PCGF3	352.2963361	0.184165313	0.215163626	0.855931442	0.392035702	0.870562753
XRCC2	281.0848503	0.218835222	0.255672056	0.855921551	0.392041174	0.870562753
LOC105371414	87.13289601	-0.455591258	0.532288871	-0.855909793	0.392047678	0.870562753
ATXN7L3	1468.144082	-0.100477974	0.117402489	-0.85584194	0.392085214	0.870562753
BOP1	2598.316933	-0.171250438	0.200105166	-0.855802183	0.392107208	0.870562753
DSP	3379.027337	0.245736078	0.287194272	0.855644079	0.392194681	0.870562753
EDC3	1105.505537	-0.098568021	0.115200574	-0.855620917	0.392207496	0.870562753
LOC101927045	38.66345528	-0.331553357	0.38750246	-0.855616134	0.392210143	0.870562753
ELAVL2	47.69007774	-0.343982249	0.402039978	-0.855592149	0.392223414	0.870562753
CGN	3.243545734	1.167795601	1.365008824	0.855522382	0.392262019	0.870562753
ARMC6	760.6535952	-0.118173612	0.138138703	-0.855470691	0.392290623	0.870562753
CEP19	65.04307438	-0.293982512	0.34369282	-0.855364135	0.392349592	0.870585199
SNORD2	32.37618146	0.402247113	0.470401933	0.85511365	0.392488234	0.870711448
ZC3H12D	3.232108674	-0.88767822	1.038143071	-0.855063473	0.39251601	0.870711448
TROAP	322.7327826	0.168072271	0.196576555	0.854996521	0.392553074	0.870711448
TADA2B	313.1138208	-0.13143379	0.153762767	-0.85478294	0.392671324	0.870787459
GTF2A1	1173.514923	-0.109012281	0.127540055	-0.854729765	0.392700768	0.870787459
ART4	5.589187314	0.932378227	1.090922085	0.854669861	0.39273394	0.870787459
MANEA-DT	46.77919233	0.398333615	0.466127966	0.8545585	0.392795611	0.870815847
SEPTIN3	5.673531322	0.92947777	1.088398322	0.853986772	0.393112319	0.871118507
PSMB4	2170.060845	-0.098456365	0.115306655	-0.853865412	0.393179566	0.871118507
KCNJ3	3.78290502	-0.775553796	0.908294787	-0.853856928	0.393184267	0.871118507
PABPC4-AS1	43.46577893	0.473659874	0.554751307	0.853823809	0.39320262	0.871118507
CUEDC2	299.5926687	-0.233041132	0.272950964	-0.853783876	0.39322475	0.871118507
CAB39	2270.641556	0.110488105	0.129418343	0.853728317	0.39325554	0.871118507
MIR656	4.9727641	-0.991218727	1.161093116	-0.853694431	0.393274321	0.871118507
EPC1	325.3416953	-0.214833847	0.251681111	-0.853595436	0.393329188	0.871131571
AP5S1	222.9039792	-0.145287423	0.170238004	-0.85343707	0.393416971	0.871131571
ZW10	453.6075553	-0.118450622	0.138795355	-0.853419208	0.393426873	0.871131571
TMEM134	69.78419751	0.239534611	0.280784108	0.853091768	0.393608416	0.871253784
USP19	1021.650875	-0.10400439	0.121919397	-0.8530586	0.393626808	0.871253784
LIN7B	36.61394181	0.366789503	0.42999229	0.853014139	0.393651464	0.871253784
CHRM3	18.26105665	-0.412622189	0.483781041	-0.852911035	0.393708642	0.871253784
WWTR1	1087.255779	-0.146680367	0.171982665	-0.852878785	0.393726528	0.871253784
LSM8	314.8714804	-0.14972934	0.175609726	-0.852625557	0.393866985	0.871414616
SPTSSA	629.9446347	0.136436234	0.160029095	0.852571432	0.393897011	0.871414616
SULF1	4821.322913	-0.173488916	0.203525253	-0.852419605	0.393981242	0.871492768
NECAP2	765.8122628	-0.098355721	0.11542203	-0.852139933	0.39413643	0.871679935
SLC8A1	544.0520276	0.154812742	0.181738706	0.851842439	0.394301548	0.871679935
CNPY2	282.6647072	-0.126714479	0.148760483	-0.851802014	0.394323988	0.871679935
TMEM25	95.88340592	-0.289197138	0.33951508	-0.851794677	0.394328062	0.871679935
IFRD2	855.2719086	-0.192225297	0.225675936	-0.851775782	0.39433855	0.871679935
YY1	3104.083962	-0.096225107	0.112976854	-0.851724079	0.394367253	0.871679935
LOC101928977	5.912925414	-0.792538891	0.930640041	-0.851606267	0.39443266	0.871679935
LOC101927974	15.99092609	-0.420291155	0.493553148	-0.8515621	0.394457183	0.871679935
RAD1	583.7582546	0.110332736	0.129590494	0.851395289	0.394549808	0.871776512
DYNLL1	1334.889729	-0.130072812	0.152798917	-0.851267892	0.394620556	0.871824735
TNFSF13	26.08576659	0.463762577	0.545233888	0.850575482	0.395005214	0.872197271
SLC25A43	383.6459925	0.133382852	0.156816996	0.850563752	0.395011732	0.872197271
ADK	690.0778024	-0.106240106	0.12490685	-0.850554684	0.395016771	0.872197271
DNAJC1	279.870605	-0.186391058	0.219147823	-0.85052662	0.395032367	0.872197271
NOP9	2017.003998	0.121011108	0.142297307	0.850410389	0.395096962	0.872197271
ZNF8	755.0589281	0.112003603	0.13175061	0.850118286	0.395259326	0.872197271
TGFB3	1341.884091	0.656616773	0.772491978	0.849998177	0.3953261	0.872197271
DUSP18	68.4002913	0.253179763	0.297865497	0.849980162	0.395336116	0.872197271
POLR2L	132.7364558	-0.187521211	0.220642373	-0.849887574	0.395387595	0.872197271
KLHL21	924.3577806	0.098437032	0.115825711	0.849872029	0.395396238	0.872197271
ZNF669	116.3957763	-0.185585525	0.218372467	-0.849857712	0.395404199	0.872197271
OPRL1	71.56931195	0.337875096	0.397572429	0.84984539	0.39541105	0.872197271
HLCS	587.3853554	-0.14311411	0.168433611	-0.849676669	0.395504873	0.872197271
PDCD11	2018.887604	-0.130252014	0.153296859	-0.849671773	0.395507596	0.872197271
NKD2	60.30921703	1.007568486	1.186016494	0.849540028	0.395580867	0.872197271
RECQL	724.1969549	-0.158283298	0.186326487	-0.849494349	0.395606273	0.872197271
LOC644285	54.50608808	0.909156417	1.070319943	0.849424906	0.395644899	0.872197271
RPL17	2987.223581	-0.15353219	0.180758071	-0.849379445	0.395670187	0.872197271
PSMC6	1060.973284	-0.138062502	0.162670585	-0.848724443	0.396034642	0.872451849
ALKBH7	271.0074924	-0.197561164	0.232780288	-0.848702293	0.39604697	0.872451849
UPF3A	398.6963768	0.1070217	0.126100786	0.848699711	0.396048407	0.872451849
LRRC14	1137.320077	0.215037879	0.253374015	0.848697445	0.396049668	0.872451849
CHN2	76.31709549	0.264508544	0.311689542	0.848628228	0.396088194	0.872451849
LOC102723701	4.394567837	0.859846918	1.013325601	0.848539618	0.396137518	0.872451849
SLC44A3-AS1	46.2658783	0.429003688	0.505754418	0.848245063	0.396301506	0.872451849
FAM151B	18.52208178	0.373559478	0.440452947	0.848125731	0.396367953	0.872451849
SLC35G1	312.4935326	0.188158237	0.221854723	0.848114635	0.396374132	0.872451849
PROX2	48.72289315	-0.367032937	0.432891967	-0.847862666	0.396514459	0.872451849
OSMR	820.658702	-0.149691522	0.17658135	-0.847719885	0.396593989	0.872451849
KIF24	189.9147478	-0.193934089	0.228814146	-0.847561622	0.396682155	0.872451849
FGFR1OP	266.7740066	0.226536748	0.267285534	0.847545861	0.396690936	0.872451849
ZGPAT	191.9988772	0.207187864	0.244456512	0.847544874	0.396691486	0.872451849

ZNF880	32.33083931	-0.34135187	0.40281009	-0.847426313	0.396757543	0.872451849
ARMCX4	379.7590258	0.215010401	0.253762901	0.847288554	0.396834305	0.872451849
CHDH	18.13502304	-0.381988686	0.450935709	-0.847102321	0.396938092	0.872451849
CLDN10	56.97409216	-0.229455492	0.270894735	-0.847028247	0.396979377	0.872451849
FAM83H-AS1	2.872149275	1.152701602	1.360891924	0.84701921	0.396984414	0.872451849
LNCSRLR	9.381554458	-0.749288702	0.88466168	-0.846977685	0.39700756	0.872451849
SDHB	666.7501301	0.131027853	0.154709647	0.84692749	0.397035539	0.872451849
DPP4-DT	17.31265034	0.405718125	0.479069401	0.846887997	0.397057553	0.872451849
GRIK1	14.53435393	0.455043532	0.537338664	0.846846807	0.397080515	0.872451849
KCNMB4	10.55577633	-0.577432302	0.68189406	-0.84680647	0.397103001	0.872451849
LRRC58	1927.252679	0.074685019	0.0881994	0.846774681	0.397120723	0.872451849
NIFK	394.403009	-0.145075964	0.171342588	-0.846701136	0.397161726	0.872451849
ODF2L	511.7722353	0.230718422	0.272591554	0.84638874	0.397335918	0.872451849
SNORD114-6	2.33168307	-1.058373135	1.250470314	-0.846380056	0.397340761	0.872451849
TERF2IP	483.5834486	-0.152662152	0.180370938	-0.846378879	0.397341417	0.872451849
MPL	4.650164762	0.888867904	1.050351594	0.846257491	0.397409116	0.872451849
TMEM26-AS1	12.89428832	0.45193118	0.534046142	0.846239949	0.3974189	0.872451849
DENND4C	612.1340709	0.119276578	0.140960144	0.846172363	0.397456597	0.872451849
SEZ6	28.63856101	0.443753931	0.524578664	0.845924475	0.397594878	0.872451849
KDELR2	5132.98486	0.144576519	0.170915356	0.845895433	0.39761108	0.872451849
FGF5	332.2512165	-0.135969929	0.160778963	-0.845694778	0.397723037	0.872451849
RHPN1	11.92988543	0.555080924	0.656566631	0.845429691	0.397870973	0.872451849
LINC02714	6.019297371	0.732052881	0.865913463	0.845411132	0.397881331	0.872451849
URGCP	282.5598582	-0.13944362	0.164969576	-0.845268706	0.397960829	0.872451849
H2BC6	10.59848868	-0.669370614	0.792073242	-0.845086766	0.398062397	0.872451849
SMIM27	28.7711553	-0.416142311	0.492438689	-0.84506421	0.39807499	0.872451849
ZSCAN32	201.1177867	-0.120894114	0.143107706	-0.844777106	0.398235299	0.872451849
RALY	844.6038755	-0.093173709	0.110306308	-0.844681607	0.398288632	0.872451849
SYNJ2	1414.633331	0.614189309	0.727180839	0.844617013	0.398324707	0.872451849
LOC105378305	19.15494375	0.427992492	0.506751034	0.844581389	0.398344604	0.872451849
WFS1	323.6676573	0.114123554	0.135137852	0.844497321	0.39839156	0.872451849
ATP6AP2	1476.728721	-0.111771554	0.132353258	-0.844494164	0.398393323	0.872451849
ORMDL1	643.9401242	0.09141349	0.108253254	0.844441036	0.398423	0.872451849
MIR3149	3.519209829	1.272748228	1.507329418	0.844372977	0.398461018	0.872451849
NOL10	961.1295051	-0.138030512	0.163478593	-0.844333862	0.398482869	0.872451849
SDE2	723.5949967	0.099408129	0.117771659	0.844075136	0.39862742	0.872451849
LOC101927620	2.176685491	-1.177790317	1.395587007	-0.84393901	0.398703488	0.872451849
ZC3HAV1	1312.164311	-0.135518133	0.160603833	-0.84380385	0.398779023	0.872451849
ECT2L	14.41197323	0.579221075	0.686445406	0.843797729	0.398782445	0.872451849
SNORD3A	264.8572655	-0.354257111	0.419852379	-0.843765877	0.398800247	0.872451849
SERTAD3	145.4291404	0.164615036	0.195106102	0.843720594	0.398825557	0.872451849
KDM7A-DT	42.52448125	-0.363850537	0.431271282	-0.843669755	0.398853973	0.872451849
SNRPG	346.5299911	0.160953024	0.19078567	0.843632671	0.398874702	0.872451849
CDCA7	647.1467651	-0.240078737	0.284611319	-0.843531935	0.398931013	0.872451849
DUSP11	389.0848517	0.107963841	0.128001919	0.843454867	0.398974097	0.872451849
NPNT	125.3538261	0.234504665	0.278036053	0.843432578	0.398986558	0.872451849
LRRC37A2	45.14949441	0.361433385	0.428591683	0.84330471	0.399058049	0.872451849
RAB7A	1405.743484	-0.132946869	0.157721154	-0.842923512	0.399271223	0.872451849
NDUFA4	804.8368232	0.148452104	0.176116686	0.842919018	0.399273737	0.872451849
MIRLET7C	2.999181108	1.268289268	1.504687913	0.84289191	0.399288899	0.872451849
NAA35	453.2935835	-0.140236943	0.166396056	-0.842790065	0.399345866	0.872451849
SNX5	1443.983297	-0.116395726	0.138126221	-0.842676545	0.399409368	0.872451849
SLITRK2	21.95909002	0.352549891	0.418371371	0.842672122	0.399411843	0.872451849
PIGV	185.3577436	-0.217434366	0.258035868	-0.84265171	0.399423262	0.872451849
MIR99A	3.754610819	0.886273048	1.051967434	0.842490955	0.3995132	0.872451849
HSPB11	74.5666241	0.254372734	0.301946356	0.842443465	0.399539773	0.872451849
CCDC74A	55.65378569	0.297932676	0.353745066	0.84222426	0.399662437	0.872451849
AKIRIN2	579.7229554	-0.112627201	0.13372805	-0.842210745	0.399670001	0.872451849
SEMA4G	110.1481698	0.219880111	0.26107921	0.842196937	0.399677728	0.872451849
ZNF283	240.1919074	-0.150349246	0.178531562	-0.842143791	0.399707472	0.872451849
BEX3	597.4597183	-0.141078258	0.167525229	-0.842131413	0.3997144	0.872451849
CCT6P3	28.27708726	-0.412280935	0.489571234	-0.842126552	0.399717121	0.872451849
CAP2	202.0488106	-0.156509884	0.185861738	-0.842076946	0.399744885	0.872451849
LINC01473	21.12421735	0.382156904	0.453834359	0.842062518	0.39975296	0.872451849
LMOD1	23.67164687	0.338977557	0.402604041	0.84196263	0.399808872	0.872451849
ZNF747	305.6505815	0.116517508	0.138389499	0.841953388	0.399814045	0.872451849
MYOM2	4.934275152	-0.779427845	0.926018462	-0.841697954	0.399957044	0.872451849
KCNIP2-AS1	11.30802646	0.577278346	0.685862063	0.841682865	0.399965492	0.872451849
SNAI3	63.91511541	0.432601411	0.514013545	0.841614808	0.400003598	0.872451849
GRTP1	7.903322652	0.727793679	0.86478612	0.841588066	0.400018572	0.872451849
SNORA16A	47.38863335	0.251404628	0.29873122	0.84157467	0.400026073	0.872451849
PGK1	5632.027083	-0.15705556	0.186625194	-0.841556042	0.400036503	0.872451849
RPS27L	335.615879	0.157539098	0.187203194	0.841540652	0.400045121	0.872451849
FMO3	7.860480129	0.802056787	0.953361971	0.841293036	0.400183791	0.872647474
LOC105373338	2.345374987	1.452555303	1.727049153	0.841061936	0.400313238	0.872715888
NTPCR	278.122196	-0.171954778	0.204459052	-0.841023062	0.400335015	0.872715888
AADAC	45.33883334	1.01246239	1.203915342	0.840974738	0.400362087	0.872715888
SCRN2	326.4326626	-0.144640709	0.172038927	-0.840744077	0.400491322	0.872792491
PTOV1-AS2	136.6339032	0.328418127	0.390631144	0.840737183	0.400495185	0.872792491
UBIAD1	471.7802985	-0.106157022	0.126330782	-0.840310016	0.400734587	0.873107195
PLPP6	89.82937609	-0.212273588	0.252615029	-0.84030467	0.400737584	0.873107195
OSGEP	407.21098	-0.168062004	0.200148862	-0.839685033	0.401085008	0.873735145
NKX2-1	177.0400967	0.167555607	0.199562248	0.839615756	0.401123862	0.873735145
MCM9	316.3963209	0.165713473	0.197440791	0.839307175	0.401296958	0.873938869
FKBP15	774.7086664	0.083276276	0.099224162	0.839274169	0.401315475	0.873938869
LINC00374	4.957022896	0.92491881	1.102883066	0.83863724	0.401672906	0.874589769
ADAMTS12	1080.731665	0.113354839	0.135176898	0.838566656	0.401712528	0.874589769
MAMSTR	18.30107264	0.415854476	0.495966902	0.838472233	0.401765536	0.874598321
POLRMT	1591.073082	0.117697002	0.140386044	0.838381074	0.401816716	0.874602891
MIR618	12.20621888	0.841795471	1.004286013	0.838202922	0.401916746	0.874635093

TIMM21	209.0073803	0.142742349	0.170308013	0.8381423	0.401950789	0.874635093
RFX3	196.2248375	-0.182624472	0.217927991	-0.838003742	0.402028602	0.874635093
TOR4A	19.18220488	-0.460534526	0.549575294	-0.837982587	0.402040483	0.874635093
HDAC4	364.6075081	-0.146981369	0.175412652	-0.837917716	0.402076918	0.874635093
STXBP5-AS1	3.544522455	-0.892585396	1.065539651	-0.837683887	0.402208265	0.874729312
STYX	1218.221599	0.103975848	0.124129009	0.837643425	0.402230996	0.874729312
VTA1	681.8941125	0.100076766	0.119493521	0.837507879	0.40230715	0.874729312
ADGRD1-AS1	2.253288502	-1.124345334	1.342516163	-0.837491097	0.402316579	0.874729312
MED13	3716.322073	0.072352778	0.086417622	0.837245648	0.402454503	0.874841362
GNRHR2	15.34467624	0.451616125	0.53942048	0.837224655	0.402466301	0.874841362
MRPL50	392.5556397	-0.101249244	0.12097793	-0.836923264	0.402635703	0.874955183
TUT7	453.4331015	0.103611456	0.123802469	0.836909443	0.402643472	0.874955183
TMEM19	590.381134	-0.10338953	0.123543202	-0.836869434	0.402665963	0.874955183
C12orf45	749.366269	0.247489846	0.295836048	0.836577719	0.402829974	0.875127446
ACCS	123.489823	-0.234355658	0.280187855	-0.836423328	0.402916794	0.875127446
HILPDA	318.2320633	0.146513004	0.175207138	0.836227369	0.403027004	0.875127446
IRAK2	30.86972995	-0.417438076	0.499193645	-0.836224739	0.403028483	0.875127446
IKBKB	815.8561646	-0.101518066	0.121421274	-0.836081379	0.403109123	0.875127446
SNORD175	4.46931667	0.879600195	1.052052456	0.836080169	0.403109804	0.875127446
PATE4	5.883199067	1.384018161	1.655627726	0.835947683	0.403184336	0.875127446
MAD2L2	325.0539536	-0.169688627	0.202999444	-0.835906856	0.403207305	0.875127446
CNOT6L	1028.413533	-0.098613102	0.11797349	-0.83589205	0.403215635	0.875127446
RASGRF2	167.3139681	0.16335317	0.195456441	0.835752301	0.403294265	0.875127446
USP2-AS1	31.8015922	-0.282731906	0.338319353	-0.835695338	0.403326319	0.875127446
FAM239B	100.4633966	-0.221421957	0.264959996	-0.835680707	0.403334552	0.875127446
RIPOR3	45.74097837	0.303523864	0.363364026	0.835316217	0.403539689	0.875465943
FBXL2	186.0508485	0.271785637	0.325404818	0.835223149	0.403592078	0.875473017
TLCD4-RWDD3	20.68043447	0.382389943	0.45788262	0.835126572	0.403646447	0.875484383
JMJD6	462.0208993	0.119262692	0.142825806	0.835022015	0.403705313	0.875505499
HIF1A-AS2	18.19576745	0.529933392	0.634716104	0.834914048	0.403766104	0.875530784
FLT4	4.855937152	0.784920002	0.940551978	0.834531232	0.403981695	0.875761005
MICU3	325.0397046	0.217702516	0.260900132	0.834428539	0.404039541	0.875761005
C1QTNF6	52.67650485	0.369099891	0.442346682	0.834413157	0.404048206	0.875761005
CDK5R1	138.4859551	0.239066199	0.286520771	0.834376502	0.404068854	0.875761005
SNORD58A	17.29015732	0.451226934	0.540944558	0.83414636	0.404198514	0.875935488
ZNF76	305.8813067	-0.153854942	0.184608305	-0.833412897	0.404611903	0.87663971
MRPL45	585.8443745	-0.166345256	0.199599481	-0.83339523	0.404621864	0.87663971
CDC16	1018.117293	-0.083658863	0.10041855	-0.833101681	0.404787385	0.876804299
GOLT1B	786.6529503	0.193282316	0.232023289	0.833029809	0.404827918	0.876804299
LSM3	152.010038	-0.176156454	0.211472655	-0.832998736	0.404845442	0.876804299
NEGR1	53.44667766	-0.343436938	0.412343868	-0.832889646	0.40490697	0.876830988
NECTIN2	431.2306036	0.152697005	0.183356862	0.832785881	0.404965499	0.876851178
NDUFS3	480.9354426	-0.126425018	0.151842953	-0.832603791	0.40506822	0.876967038
LOXL4	21.26852726	0.386879867	0.464891737	0.832193469	0.40529975	0.877265808
NXT2	276.7204023	-0.126776368	0.152341618	-0.832184733	0.40530468	0.877265808
UBE2V2	1432.14412	0.075668457	0.090947718	0.83199951	0.405409221	0.877279382
LCMT1	193.0922049	-0.178719649	0.214837497	-0.83188294	0.405475022	0.877279382
USP43	3.530202036	0.947090752	1.138584984	0.831813844	0.405514028	0.877279382
KDM4A-AS1	33.01091773	0.405017846	0.486970443	0.831709299	0.40557305	0.877279382
CARMIL3	6.100603208	0.824334757	0.991325037	0.831548409	0.405663893	0.877279382
TAB2	1516.436807	0.152497207	0.183399308	0.831503716	0.40568913	0.877279382
SLC45A3	156.7049607	0.166069679	0.199728586	0.831476771	0.405704346	0.877279382
RHOD	5.915276698	-0.717265569	0.862890924	-0.831235501	0.405840603	0.877279382
H1-10	662.2003915	-0.238195367	0.286587269	-0.831144274	0.405892131	0.877279382
OTUD6B	473.4624905	0.224356439	0.269998172	0.830955399	0.405998826	0.877279382
MAU2	530.2319921	-0.130343071	0.15689583	-0.830761856	0.406108175	0.877279382
AMZ2P1	106.6675358	-0.185845331	0.223737463	-0.830640198	0.406176919	0.877279382
LIMK1	469.2385316	0.194061321	0.233647452	0.83057324	0.406214757	0.877279382
CXXC5	355.0539128	-0.199253007	0.239914402	-0.830517073	0.406246499	0.877279382
EXOC1L	9.879273403	0.503219042	0.605981277	0.830420115	0.406301297	0.877279382
DUSP13	2.182287535	-1.258921877	1.516176547	-0.830326705	0.406354093	0.877279382
BMS1	838.0451525	-0.10591588	0.127571888	-0.830244666	0.406400466	0.877279382
MRPL44	333.8763712	-0.140500745	0.169238693	-0.830192806	0.406429782	0.877279382
DOCK1	1690.314181	-0.107820492	0.129875705	-0.830182151	0.406435805	0.877279382
MIR17HG	672.6692185	0.551454388	0.664261935	0.830176108	0.406439221	0.877279382
SLC25A36	1728.100731	-0.076886956	0.09262494	-0.830089135	0.40648839	0.877279382
SLC18B1	387.945837	0.101630161	0.122474979	0.829803462	0.406649913	0.877279382
LOC104054148	4.583857565	1.065929293	1.284611173	0.829768038	0.406669945	0.877279382
MIR29B2CHG	6.913459372	0.776046247	0.935350474	0.829684988	0.406716911	0.877279382
RABEP2	155.6631175	0.151122741	0.182163444	0.829599712	0.406765139	0.877279382
ABCA8	214.9739912	-0.217083299	0.261700437	-0.829510646	0.406815515	0.877279382
LDLRAP1	99.78593878	-0.217037479	0.26165383	-0.829483285	0.406830991	0.877279382
LOC101928861	9.696501984	0.817773046	0.986081035	0.829316271	0.406925466	0.877279382
PTPN3	320.3165165	0.12182942	0.14691195	0.829268283	0.406952614	0.877279382
LINC02338	25.15961113	0.450048149	0.542728571	0.829232463	0.406972879	0.877279382
PRDX1	2806.592916	-0.093166928	0.112366528	-0.829134168	0.407028492	0.877279382
TSTA3	472.0387121	-0.195662702	0.236000985	-0.829075788	0.407061524	0.877279382
LOC101928837	5.795044544	0.702339911	0.847242462	0.828971567	0.407120496	0.877279382
LOC101928659	3.083132507	-0.937134257	1.130565943	-0.828907206	0.407156917	0.877279382
MOXD1	20.98892884	-0.494369284	0.596423379	-0.828889848	0.407166741	0.877279382
CDC25B	1072.808208	-0.107627519	0.129875449	-0.828697952	0.407275345	0.877279382
PPIH	180.7057402	0.146750463	0.177087003	0.828691322	0.407279098	0.877279382
HEY2	450.8530272	0.296457582	0.357815256	0.82852136	0.407375304	0.877279382
PIK3AP1	4.20532416	-0.815188948	0.984117005	-0.828345556	0.40747483	0.877279382
TP73	8.757209446	0.537663475	0.649173861	0.828227239	0.40754182	0.877279382
SPIN2A	2.985615497	1.04128203	1.257361976	0.828148178	0.407586588	0.877279382
CDK7	227.529	0.131988329	0.159380249	0.828134793	0.407594167	0.877279382
SMURF2P1-LRRC37BP1	243.6896401	-0.213350047	0.257663018	-0.828019667	0.407659362	0.877279382
LINC01970	9.721936759	0.48052925	0.580348708	0.828000895	0.407669993	0.877279382
NAGPA	201.4821084	-0.232661833	0.28100638	-0.827959253	0.407693576	0.877279382

LINC01206	3.228824345	-0.801260808	0.968041961	-0.827712889	0.407833118	0.877279382
DSTYK	740.483181	0.139068357	0.168071262	0.82743686	0.407989496	0.877279382
ARID3A	239.1364865	-0.150725497	0.182181898	-0.827335198	0.408047099	0.877279382
ZNF213-AS1	51.70829311	0.312285518	0.377508256	0.827228315	0.408107666	0.877279382
TVP23A	15.84836132	0.56234827	0.67995383	0.827038903	0.408215012	0.877279382
TAF5	215.6041544	-0.146378944	0.176994355	-0.82702606	0.408222291	0.877279382
DDX58	78.11039751	-0.231639621	0.280151543	-0.826836856	0.408329537	0.877279382
PWAR5	854.9051889	0.260069626	0.314536086	0.826835575	0.408330263	0.877279382
GGT1	11.52584658	0.527632913	0.638185667	0.826770233	0.408367305	0.877279382
SH3BP5L	670.1193395	0.117598958	0.142258709	0.826655593	0.408432298	0.877279382
ALKBH1	246.1513259	0.121896728	0.14756012	0.82608179	0.408757697	0.877279382
TMBIM4	412.4173968	-0.122382398	0.148175407	-0.825929216	0.408844246	0.877279382
FTO-IT1	46.65851245	0.550211904	0.666194559	0.825902729	0.408859273	0.877279382
NPPA-AS1	9.415290697	-0.770981268	0.933520416	-0.825885813	0.408868869	0.877279382
ANKDD1B	3.750187845	-0.761732139	0.922406939	-0.825809203	0.408912333	0.877279382
HMGN4	1589.882714	-0.106111655	0.128499384	-0.82577559	0.408931403	0.877279382
OPTN	107.9686744	-0.322597213	0.390673044	-0.825747306	0.408947451	0.877279382
CHRNA7	8.235066323	0.619708467	0.750503305	0.825723834	0.408960769	0.877279382
TMEM192	438.5611825	0.10951446	0.132641691	0.825641313	0.409007592	0.877279382
PALLD	2159.667811	0.073021421	0.088454531	0.825524937	0.409073631	0.877279382
MYRF	186.3549244	0.21207074	0.256898206	0.825504947	0.409084975	0.877279382
LSR	89.56388024	0.332477958	0.402808869	0.825398803	0.409145214	0.877279382
MIR3146	18.19606824	0.438291821	0.531009152	0.825394099	0.409147884	0.877279382
CTF1	5.039904894	-0.684430337	0.829229286	-0.82538129	0.409155154	0.877279382
DOC2GP	2.828754509	-1.203312703	1.457934862	-0.825354229	0.409170513	0.877279382
ZNF671	19.39565226	0.505444378	0.612433497	0.825304919	0.4091985	0.877279382
ZNF836	115.411501	-0.180018429	0.218126514	-0.825293661	0.40920489	0.877279382
SMC5	700.9603995	-0.101730539	0.123269161	-0.825271615	0.409217403	0.877279382
MIR3190	7.298379419	-0.626692508	0.759384931	-0.825263293	0.409222127	0.877279382
LAMTOR1	302.8783043	-0.146028018	0.176957111	-0.825217005	0.409248401	0.877279382
ZNF460-AS1	12.82437499	0.580566313	0.703648961	0.825079471	0.409326474	0.877279382
FBXL7	451.7555371	0.241261225	0.292426047	0.825033292	0.40935269	0.877279382
FRG1HP	149.9213377	0.225414664	0.273229804	0.825000276	0.409371434	0.877279382
MYL6	1087.509387	-0.138290385	0.167633945	-0.824954547	0.409397396	0.877279382
IFIT1	91.32116565	0.188087515	0.228002202	0.824937275	0.409407203	0.877279382
LINC01910	3.496962504	0.831779717	1.008334776	0.824904323	0.409425912	0.877279382
WASF1	417.8378221	-0.108489155	0.131517653	-0.824901846	0.409427318	0.877279382
ABCC3	2.590315112	-1.144717413	1.387876933	-0.824797491	0.409486571	0.877279382
LMNB2	4402.087094	-0.109154568	0.132353318	-0.824721055	0.409529975	0.877279382
SNORA5B	13.5739062	0.481767674	0.584174224	0.824698616	0.409542717	0.877279382
SNORD96A	94.59223828	0.302662965	0.36699985	0.824695064	0.409544735	0.877279382
TMEM229B	6.117145296	1.028362861	1.247246435	0.824506555	0.409651793	0.87733038
SNORD89	12.23635136	0.572065307	0.693850027	0.824479766	0.409667008	0.87733038
NOM1	618.3547872	0.155157711	0.188219364	0.824345103	0.409743498	0.877388744
MIR1914	2.153428133	1.24844975	1.515127356	0.823989974	0.409945255	0.877547641
GNB5	430.9287568	-0.202574782	0.24587539	-0.823892059	0.410000893	0.877547641
RPSA	11593.93503	-0.128073584	0.155474891	-0.823757351	0.410077445	0.877547641
TNFRSF14	2.961161512	-1.186099328	1.439897726	-0.823738594	0.410088105	0.877547641
NUDT9	140.4728385	0.143821868	0.174636393	0.823550378	0.410195081	0.877547641
IPO5P1	30.71214542	0.349165837	0.423989249	0.823525214	0.410209385	0.877547641
HAUS4	809.346178	-0.140492854	0.170602548	-0.823509704	0.410218201	0.877547641
ELMOD3	256.0657734	0.182392702	0.221496079	0.823457925	0.410247635	0.877547641
PLD1	666.8555522	-0.161537351	0.196175947	-0.823430971	0.410262957	0.877547641
ERI1	975.6274654	-0.09676952	0.117531744	-0.823347949	0.410310154	0.877547641
ZNF273	83.52309162	0.194884302	0.236773348	0.823083777	0.410460353	0.87776353
DENND4A	1047.38264	-0.090361542	0.109841953	-0.822650537	0.410706751	0.877937542
MRNIP	115.1060097	0.233474652	0.283938079	0.822273126	0.410921468	0.877937542
COL5A1	54579.98895	0.128736495	0.156564406	0.822259022	0.410929493	0.877937542
AOPEP	847.7851231	0.175932347	0.21398531	0.822170208	0.410980032	0.877937542
BCL10	452.7780774	-0.152513005	0.185549784	-0.82195194	0.41110425	0.877937542
ZSCAN30	230.9279668	-0.19033458	0.231572584	-0.821921909	0.411121343	0.877937542
SCAF11	1644.05248	-0.111631541	0.135843635	-0.821764977	0.41121067	0.877937542
HCN3	117.4507075	0.270814499	0.329557693	0.821751411	0.411218393	0.877937542
NADK	1441.052233	-0.098920801	0.120407114	-0.821552794	0.411331465	0.877937542
PRSS36	2.395683669	1.092333192	1.329673624	0.821504746	0.411358822	0.877937542
INF2	2014.215613	-0.079103334	0.096302227	-0.821407113	0.411414413	0.877937542
PPP6C	833.0451122	-0.101393457	0.123458896	-0.82127299	0.411490789	0.877937542
CEP76	170.6889638	0.164899716	0.200789156	0.821258075	0.411499283	0.877937542
SNRPD1	872.8727394	-0.151914975	0.184984218	-0.821232087	0.411514083	0.877937542
TAF4B	694.818543	-0.119142849	0.145084445	-0.821196573	0.411534308	0.877937542
TSTD3	61.37703862	0.343543692	0.418381711	0.821125023	0.411575058	0.877937542
PA2G4P4	23.85073512	0.457088083	0.556686526	0.821087023	0.411596701	0.877937542
C6orf62	2841.921102	-0.126507334	0.154077998	-0.821060348	0.411611895	0.877937542
SMAD7	491.2905721	-0.287049115	0.34966174	-0.820933726	0.411684019	0.877937542
TCERG1	1459.915138	0.128028806	0.15597473	0.820830439	0.411742858	0.877937542
ZNF784	81.23621672	0.183811893	0.223973542	0.820685747	0.411825291	0.877937542
NSFP1	2.370151523	-1.202646639	1.465590525	-0.820588437	0.411880736	0.877937542
PCCA-AS1	3.762109628	1.117460017	1.361811623	0.820568717	0.411891973	0.877937542
FRYL	1716.629454	0.129840795	0.158237586	0.820543326	0.411906441	0.877937542
RHOU	143.6785475	-0.290719479	0.354307422	-0.820528901	0.411914661	0.877937542
GNAT2	21.88457096	-0.565937711	0.689745659	-0.820502027	0.411929974	0.877937542
GEMIN7-AS1	26.93142119	0.562737359	0.685866981	0.820475944	0.411944837	0.877937542
RTKN	199.6487686	0.127703703	0.155656295	0.820421067	0.41197611	0.877937542
TRNAU1AP	274.0698365	-0.114823753	0.139971721	-0.820335365	0.412024951	0.877937542
SERTM1	2.178090812	-1.124548617	1.371070367	-0.820197595	0.412103473	0.877937542
ATP11C	1047.26359	0.086035614	0.104899878	0.820168865	0.412119848	0.877937542
NRARP	9.145081844	0.717060885	0.874410394	0.820050734	0.412187186	0.877937542
PLN	19.41669569	0.653183208	0.796532461	0.820033382	0.412197077	0.877937542
PRKCA-AS1	11.56147679	0.850704101	1.037445568	0.819998781	0.412216802	0.877937542
FABP3	19.40330504	-0.406259532	0.495493797	-0.819908411	0.412268322	0.877942339

STK24	586.1570087	-0.213977378	0.261083677	-0.819573937	0.412459036	0.8779565
C3orf18	110.9343925	0.247766382	0.302313636	0.81956734	0.412462799	0.8779565
ITIH4	9.075646118	-0.635145088	0.775029641	-0.819510706	0.412495096	0.8779565
RNF40	1503.115461	-0.083920561	0.102417698	-0.819395109	0.412561025	0.8779565
BMI1	134.8014253	-0.167472212	0.204400748	-0.819332676	0.412596634	0.8779565
HMG20B	514.926666	-0.116672777	0.142414171	-0.819249773	0.412643923	0.8779565
NCSTN	1966.371131	-0.0916549	0.111881812	-0.819211791	0.412665589	0.8779565
UBP1	1591.396524	-0.122763626	0.149896697	-0.818988196	0.412793148	0.8779565
HERC2P9	203.1566398	0.280099112	0.342026376	0.818940093	0.412820594	0.8779565
TAOK2	954.9973208	-0.142866996	0.174463002	-0.818895664	0.412845944	0.8779565
BRAF	1038.114254	0.078143779	0.095427095	0.818884606	0.412852254	0.8779565
COX20	544.7238224	0.28451932	0.347462227	0.81884964	0.412872205	0.8779565
IDO1	29.52112449	-0.725369417	0.885921579	-0.818773845	0.412915456	0.8779565
TMA7	142.9765623	-0.203583013	0.248690094	-0.818621318	0.4130025	0.878036812
LOC100131626	5.752677012	1.090810122	1.33305559	0.818278045	0.41319844	0.878348586
RARS1	1297.762115	0.096489907	0.117933663	0.818171034	0.413259532	0.878373672
LINC00648	233.8493642	-0.190104251	0.232409987	-0.817969373	0.413374676	0.878513622
OXA1L	1643.418356	-0.139093229	0.170141412	-0.81751543	0.413633935	0.878885343
SPATA22	191.2555109	0.192446125	0.235410864	0.817490414	0.413648225	0.878885343
FAM50B	137.4394382	-0.169525927	0.207529054	-0.816878042	0.413998127	0.879165863
TOMM7	163.1686484	0.163499042	0.200190898	0.816715661	0.41409094	0.879165863
TEX264	336.1989974	-0.107838315	0.132039525	-0.816712386	0.414092811	0.879165863
RPL23A	2813.857175	-0.178831265	0.218967361	-0.816702836	0.41409827	0.879165863
GLUD1P3	85.08956445	0.34104648	0.417626276	0.816630801	0.414139448	0.879165863
RILPL1	293.793071	0.174619377	0.213847861	0.816558912	0.414180544	0.879165863
SYTL2	219.7258907	0.301938079	0.369784179	0.816525141	0.41419985	0.879165863
FAT2	7.012268846	0.839037771	1.027625576	0.816481985	0.414224523	0.879165863
PLEKHG2	655.5884568	0.120322939	0.147374545	0.816443155	0.414246723	0.879165863
RPRD1B	606.0604806	-0.094230571	0.115433628	-0.816318194	0.414318171	0.879165863
AP4M1	128.5117061	0.149954936	0.183698553	0.816309842	0.414322947	0.879165863
SNORA80B	11.90984643	0.672846743	0.824352927	0.81621199	0.414378901	0.879179903
PRR5	91.23522268	0.207120382	0.253831049	0.815977332	0.4145131	0.879359934
DDX43	5.784293483	0.657924059	0.806517813	0.815758869	0.414638062	0.879520327
NTHL1	163.2853654	-0.157624602	0.193268972	-0.815571173	0.414745443	0.879524682
TYMSOS	37.75244783	-0.280987674	0.344551666	-0.815516805	0.414776549	0.879524682
SLC16A4	93.68976046	0.388887699	0.476872326	0.815496472	0.414788183	0.879524682
LINC00355	81.82450622	0.258249884	0.316770856	0.815257716	0.414924807	0.879587823
HIVEP3	37.80657237	-0.314412745	0.385703182	-0.81516762	0.41497637	0.879587823
NIPAL2	448.4703464	-0.134225487	0.164691688	-0.815010697	0.415066187	0.879587823
MFAP1	359.2531469	-0.122787402	0.1506921	-0.814823087	0.415173584	0.879587823
CCDC26	17.27146898	0.403305244	0.494964332	0.814816781	0.415177194	0.879587823
AUH	211.2825101	-0.137796111	0.169158363	-0.814598274	0.415302299	0.879587823
NAGPA-AS1	3.706574528	0.789824831	0.969634927	0.814558974	0.415324802	0.879587823
LINC01490	4.096951646	-1.034469953	1.270051748	-0.814510082	0.415352799	0.879587823
MAPKAPK5-AS1	318.9863629	0.180343012	0.221426562	0.814459703	0.415381648	0.879587823
LOC100128386	2.687366814	0.90045913	1.10569437	0.814383391	0.415425351	0.879587823
MARF1	1274.821807	0.140731057	0.17282808	0.81428352	0.415482549	0.879587823
TIMM23	221.8934547	0.120678425	0.148202094	0.814282863	0.415482925	0.879587823
LITAF	845.2182652	-0.254076446	0.312026749	-0.814277772	0.415485841	0.879587823
NCOA4	1750.222592	-0.167038222	0.20517965	-0.814107156	0.415583567	0.879587823
MKRN2OS	6.06698406	0.626195407	0.769184834	0.814102644	0.415586151	0.879587823
UBE2G2	850.3180519	0.207425929	0.254823009	0.814000001	0.415644951	0.879587823
DLX4	17.10235594	-0.509348324	0.625766754	-0.81395875	0.415668583	0.879587823
GAB3	6.317743933	-0.63820673	0.784189568	-0.813842413	0.415735234	0.879587823
CUL4B	1028.089716	-0.118311823	0.145380773	-0.813806531	0.415755793	0.879587823
FRMD3	38.97422799	-0.254317963	0.312640178	-0.813452589	0.415958618	0.879812633
PLD3	1095.346756	-0.249842062	0.307139265	-0.813448784	0.415960799	0.879812633
PPIL1	550.2988961	-0.104571579	0.128605934	-0.813116286	0.41615139	0.880111295
PLXNA4	17.93751014	0.614933429	0.756369092	0.813007083	0.416213997	0.880123431
ZACN	11.34148755	-0.508903992	0.62605227	-0.8128778	0.416288124	0.880123431
TEKT4P2	355.8457571	-0.130934479	0.161090425	-0.812801132	0.416332086	0.880123431
CCDC27	9.6386587	0.565459679	0.695726269	0.81276172	0.416354687	0.880123431
ARMC5	318.9799299	0.12503955	0.153937285	0.81227592	0.416633324	0.880579338
LAS1L	688.2753515	0.150503843	0.185304046	0.812199444	0.416677199	0.880579338
EIF2B1	1110.368582	-0.080316966	0.098905904	-0.812054313	0.416760467	0.880579338
TBC1D15	709.4726042	-0.12903233	0.158900687	-0.812031293	0.416773676	0.880579338
LOC105373159	66.58870009	-0.214863912	0.264625385	-0.811955029	0.416817437	0.880579338
OPN3	225.6251295	0.147069724	0.181191279	0.811682135	0.416974048	0.880805777
ZNF142	794.7254607	0.108681749	0.133911751	0.811592325	0.417025597	0.880810256
PODN	4.306212704	-0.903365107	1.113420592	-0.811342195	0.417169185	0.881009111
LPL	437.9924094	0.138231438	0.170423601	0.811105018	0.417305364	0.881192273
EEF1AKMT1	26.24578716	-0.428398621	0.528259147	-0.810962996	0.417386921	0.881216939
MID1IP1-AS1	17.53942598	0.446736613	0.550906094	0.810912455	0.417415947	0.881216939
CDKL2	6.069210163	0.829144678	1.022629203	0.810796988	0.417482264	0.881252541
ZSCAN21	56.02050279	-0.227420456	0.280598401	-0.810483791	0.417662178	0.881525785
NEK5	17.80779308	0.438847399	0.541519878	0.810399427	0.417710647	0.881525785
NAPA-AS1	25.84860875	-0.306630307	0.378435174	-0.810258476	0.417791635	0.881592296
SERPINF2	2.459586006	1.135099097	1.401198998	0.810091285	0.417887713	0.881606743
GALR3	3.781533436	0.807273015	0.996541842	0.810074381	0.417897427	0.881606743
DNAH14	666.0442633	0.160431931	0.198095875	0.809870124	0.418014824	0.88175002
PPP1R1C	14.46647669	-0.408732523	0.504788566	-0.809710343	0.418106672	0.881839377
ZNF69	8.125261188	-0.647556608	0.799876248	-0.809570992	0.418186786	0.881903967
PPP6R1	1311.301064	0.089615324	0.110721383	0.809376846	0.418298416	0.881932
CEP57L1	366.0764384	0.10691381	0.132094164	0.809375725	0.418299061	0.881932
LOC100499489	23.75359472	-0.32937728	0.40703395	-0.809213285	0.418392475	0.882024595
DEGS1	2046.984004	-0.096533311	0.119327133	-0.80898039	0.418526426	0.882118364
PSTK	41.21943331	0.272076238	0.336359444	0.808885385	0.418581076	0.882118364
SHROOM1	268.9471644	0.196413646	0.242822407	0.808877766	0.418585459	0.882118364
MKI67	2037.752147	-0.106110184	0.131202945	-0.808748494	0.418659828	0.882170763
TBX6	14.00683451	0.440770494	0.5451418	0.808542831	0.41877816	0.882252493

LOC145474	11.28751435	0.889076859	1.099736004	0.808445714	0.418834045	0.882252493
CRNDE	589.9289845	0.102352651	0.126635899	0.808243569	0.418950381	0.882252493
MYBL2	639.9327923	-0.182166033	0.225398607	-0.808195023	0.418978323	0.882252493
AFAP1	765.1051313	0.151433875	0.187399277	0.808081427	0.419043709	0.882252493
CCDC18-AS1	331.1128349	0.236744039	0.292975256	0.808068375	0.419051222	0.882252493
CISH	9.135857112	0.539661501	0.668068309	0.807793895	0.41920924	0.882252493
INTS12	133.5034397	-0.17145761	0.212255616	-0.807788333	0.419212443	0.882252493
ZNF703	1013.730819	0.152301619	0.188550848	0.807748259	0.419235516	0.882252493
C9orf50	2.545209387	1.035170312	1.281557527	0.807743929	0.41923801	0.882252493
SPATC1L	173.3642419	0.155210379	0.192162156	0.807705232	0.419260291	0.882252493
ABRA	9.233031472	0.858223531	1.062834603	0.8074855	0.419386825	0.882252493
NUCKS1	15254.4674	0.095942168	0.118831894	0.807377251	0.419449169	0.882252493
PNMA3	85.1011105	0.238281911	0.295134061	0.807368387	0.419454275	0.882252493
ZNF79	119.676264	0.208505224	0.258256366	0.80735754	0.419460522	0.882252493
UFD1	276.3710727	-0.148154799	0.183517743	-0.807305039	0.419490762	0.882252493
GDF9	8.925095023	-0.586996159	0.727304265	-0.80708472	0.419617675	0.882415266
MRM3	289.6122357	0.107399517	0.133117086	0.806804901	0.419778895	0.882469069
PLCXD2	6.480303134	0.638989457	0.792011624	0.806793029	0.419785736	0.882469069
MDM1	241.178531	0.218410466	0.270717914	0.806782465	0.419791824	0.882469069
TAF12	235.516734	-0.180449945	0.223710447	-0.806622792	0.419883839	0.882497178
ACOT1	17.86533905	-0.421240334	0.52240167	-0.806353345	0.42003914	0.882497178
ABHD3	156.2627909	0.189133707	0.234591157	0.806226923	0.420112018	0.882497178
BAZ2B	276.563417	-0.130803221	0.162271689	-0.806075428	0.420199359	0.882497178
SAP25	9.027330128	-0.505860163	0.627600803	-0.806022173	0.420230064	0.882497178
BMT2	301.9949773	0.148765521	0.184593646	0.805908136	0.420295819	0.882497178
RPUSD4	396.6090168	-0.101151701	0.12552675	-0.805817888	0.420347862	0.882497178
SERPINH1	4815.982408	-0.127419084	0.15812694	-0.8058025	0.420356736	0.882497178
FANCD2	872.7390934	0.1536875	0.190741592	0.805736694	0.420394687	0.882497178
SS18	993.880392	0.083443239	0.103581734	0.805578697	0.420485813	0.882497178
RSPH6A	4.127878271	0.910828364	1.130685609	0.805554043	0.420500033	0.882497178
P2RY1	130.3140068	0.176179025	0.218710131	0.805536646	0.420510068	0.882497178
UBXN4	1175.200422	-0.09003458	0.111777653	-0.805479249	0.420543176	0.882497178
LOC101927189	3.90948102	0.940196164	1.167478398	0.805322108	0.420633827	0.882497178
DCAF8L2	17.67549168	-0.387773115	0.481526688	-0.805299321	0.420646973	0.882497178
LOC105369911	5.146980068	0.702080787	0.872030773	0.805110104	0.420756146	0.882497178
FER1L5	21.49677557	0.541242526	0.6723061	0.80505372	0.420788681	0.882497178
SNUPN	310.2214034	-0.124157794	0.154255861	-0.804882185	0.42088767	0.882497178
ZMYM2	1863.821001	0.11956349	0.148551449	0.804862492	0.420899035	0.882497178
RNF139-AS1	94.92991377	0.426786548	0.530269197	0.80484884	0.420906914	0.882497178
LINC01273	4.158929577	0.787456081	0.978525132	0.804737717	0.42097105	0.882497178
FLJ44635	7.148363593	0.69215058	0.860141169	0.804694165	0.420996188	0.882497178
PPP1R14D	3.390573371	0.974079517	1.210681738	0.804571083	0.421067235	0.882497178
LINC00662	126.4131839	-0.166739125	0.20726171	-0.804485909	0.421116404	0.882497178
PRICKLE2-AS1	2.753259233	0.950436678	1.181444127	0.804470272	0.421125431	0.882497178
MRFAP1	1525.571222	-0.101003498	0.125560015	-0.804424069	0.421152105	0.882497178
RRAGD	316.120731	0.317003858	0.394101785	0.80437052	0.421183021	0.882497178
LMO2	4.115819601	-1.17541785	1.461316803	-0.804355255	0.421191835	0.882497178
LRRC14B	5.776168112	0.913780606	1.136348266	0.80413781	0.421317391	0.882656466
USH2A	32.60487512	0.456840307	0.568368981	0.803774172	0.421527409	0.882904413
ETV3	705.8978297	-0.094360408	0.117409027	-0.803689549	0.421576291	0.882904413
PRDM4	1186.815345	0.089571212	0.111451956	0.803675547	0.42158438	0.882904413
TIAL1	1413.298342	-0.128883873	0.160392639	-0.803552295	0.421655583	0.882949764
MIR645	3.010735743	0.919926276	1.145169288	0.803310293	0.42179541	0.883138787
LOC100130172	3.964774954	0.787999334	0.981105582	0.803174855	0.421873677	0.883198886
LINC02201	6.112086367	-0.668880836	0.833496033	-0.802500324	0.422263601	0.883672374
CCDC12	153.7461578	-0.174174463	0.217041598	-0.802493462	0.422267569	0.883672374
CCN2	1133.178645	0.281232246	0.350481799	0.802416123	0.42231229	0.883672374
LINC00216	54.08641292	0.35653246	0.444341684	0.802383555	0.422331123	0.883672374
ZNF818P	17.77969836	0.657513026	0.81961628	0.802220554	0.422425389	0.883672374
POU4F1	12.59845772	-0.48274	0.601831258	-0.802118523	0.422484401	0.883672374
CD68	668.8834853	0.30447147	0.379622746	0.802036952	0.422531584	0.883672374
TRIAP1	401.71627	-0.114931	0.143305789	-0.801998305	0.422553939	0.883672374
FADS3	161.8754699	-0.159277351	0.198610023	-0.801960285	0.422575932	0.883672374
POLR3D	1999.739365	0.160066061	0.199635024	0.801793478	0.422672432	0.883672374
ARC	16.66498208	-0.366118232	0.456682881	-0.801690291	0.422732134	0.883672374
MOSPD2	323.8431218	-0.147480423	0.183987117	-0.801580164	0.422795856	0.883672374
KRT10	132.9399427	0.158149937	0.197305859	0.801547088	0.422814995	0.883672374
ZDHHC24	145.6961354	0.188074386	0.234652308	0.801502394	0.422840858	0.883672374
FERMT3	16.09028921	0.428978929	0.535245741	0.80146164	0.422864443	0.883672374
HEXIM1	347.6588799	0.123169219	0.153716114	0.801277207	0.422971183	0.883672374
CCDC6	2098.569281	-0.11045937	0.137854128	-0.801277202	0.422971186	0.883672374
ARPIN-AP3S2	4.919777117	-0.845750501	1.055551395	-0.801240475	0.422992444	0.883672374
FBXL19-AS1	32.08739883	0.298154223	0.372156716	0.801152338	0.42304346	0.883675355
FANCC	733.155438	-0.105944336	0.132270212	-0.800968973	0.423149608	0.883793486
LSM12	807.9148826	-0.133908177	0.167218818	-0.800796097	0.423249699	0.883822347
NBEAL2	298.8327774	0.207020895	0.258526066	0.80077378	0.423262621	0.883822347
COQ6	181.681862	0.166401565	0.207848774	0.800589592	0.423369278	0.883899411
TMEM123	2745.963573	0.104280705	0.130276541	0.800456508	0.423446353	0.883899411
MKKS	319.3195868	-0.133422998	0.16671053	-0.80032736	0.423521155	0.883899411
CCND3	623.0188217	0.082483159	0.103070152	0.800262323	0.423558827	0.883899411
CCDC30	11.30901565	0.583379361	0.729191729	0.800035625	0.423690157	0.883899411
SUMO4	10.37258053	0.52727825	0.659139628	0.799949248	0.423740202	0.883899411
C19orf38	4.303874096	0.830449037	1.038133357	0.799944469	0.423742972	0.883899411
C2CD3	384.9621841	0.108493751	0.135645712	0.79983178	0.423808268	0.883899411
LOC100507283	7.849554237	-0.599387618	0.749535217	-0.799679061	0.423896768	0.883899411
LSM5	386.1385507	-0.144434682	0.18061764	-0.799670959	0.423901463	0.883899411
PFKFB4	213.3689009	0.145770905	0.18229121	0.799659536	0.423908083	0.883899411
B4GALT4	265.1639996	0.13530989	0.169236007	0.799533692	0.423981019	0.883899411
MLX	548.797962	-0.085403756	0.106830444	-0.799432752	0.424039525	0.883899411
UCN2	4.10378962	-0.79349584	0.992582049	-0.799425942	0.424043473	0.883899411

LOC100996447	2.909649247	1.004230079	1.256295701	0.799358048	0.424082829	0.883899411
XIAP	1480.345071	0.149286805	0.186762529	0.79934024	0.424093152	0.883899411
CAPN7	433.3976934	0.10815589	0.135376028	0.798929405	0.424331348	0.883981703
UQCC1	420.6675776	0.098567724	0.123399301	0.798770523	0.424423485	0.883981703
KCNK12	44.92673101	0.380039776	0.475814533	0.7987141	0.424456209	0.883981703
LDLRAD3	290.103086	0.106540694	0.133445706	0.798382329	0.424648655	0.883981703
UBE2D1	297.6546507	0.110544709	0.138481779	0.798261759	0.424718605	0.883981703
ARHGAP23	396.7107692	-0.139645806	0.17494602	-0.798222252	0.424741526	0.883981703
CCNJL	62.32782091	0.229104031	0.287102646	0.797986486	0.424878333	0.883981703
DNAJC25	141.5857866	0.169707424	0.212670901	0.797981401	0.424881284	0.883981703
GPX8	832.7808867	-0.170864073	0.214133669	-0.797931845	0.424910042	0.883981703
NAV1	2481.478582	0.130832338	0.163974369	0.797882852	0.424938475	0.883981703
LINC00578	228.4538966	-0.217148674	0.272237782	-0.797643415	0.425077449	0.883981703
EHD2	1839.896286	-0.100962026	0.126667913	-0.797060787	0.425415729	0.883981703
MIR4435-1	4.921135569	1.448785086	1.817717051	0.797035537	0.425430393	0.883981703
STARD9	722.5636622	0.191701304	0.240544349	0.796947857	0.425481315	0.883981703
ZNF440	185.9406686	-0.152203516	0.190992426	-0.796908646	0.425504089	0.883981703
SNORA50A	8.743020211	-0.879966165	1.104343168	-0.796823117	0.425553767	0.883981703
FAM174C	192.3571514	0.166488428	0.208950679	0.796783382	0.425576848	0.883981703
LOC108783645	7.324993439	-0.690920851	0.867223277	-0.796704688	0.42562256	0.883981703
DUXAP10	16.90562005	-0.435429632	0.546567676	-0.79666188	0.425647429	0.883981703
ING5	539.1554464	0.160000874	0.200846372	0.796633128	0.425664132	0.883981703
LINC01139	72.71871704	-0.191827214	0.240813179	-0.796581047	0.425694388	0.883981703
SRRD	435.109974	0.105227164	0.132118828	0.796458504	0.425765585	0.883981703
SNORD11	10.17838946	0.650235774	0.816661519	0.796212089	0.425908772	0.883981703
CRY2	207.1691509	-0.124355664	0.156218131	-0.796038613	0.426009593	0.883981703
RB1CC1	1917.57236	-0.142345334	0.178822068	-0.796016598	0.426022388	0.883981703
DDT	190.9240967	-0.120029177	0.150813761	-0.795876825	0.426103632	0.883981703
SNORD43	74.70420584	0.256340122	0.322158551	0.795695541	0.426209019	0.883981703
PDK4	4.252681413	-0.688108477	0.864871115	-0.795619676	0.426253127	0.883981703
PKLR	24.9203689	0.300701498	0.377975641	0.795557874	0.42628906	0.883981703
ASCL5	8.748630276	0.608762591	0.765299525	0.795456643	0.426347922	0.883981703
ZNF510	518.5409504	-0.091038153	0.114462285	-0.795355021	0.426407017	0.883981703
BBS12	183.0564818	-0.121755615	0.153084049	-0.795351416	0.426409113	0.883981703
ACAD9	886.064776	-0.109551212	0.13774298	-0.795330637	0.426421196	0.883981703
RFX8	350.9500844	-0.224766706	0.282645359	-0.795225178	0.426482528	0.883981703
LOC100505715	39.11393377	0.265084822	0.33335761	0.795196553	0.426499177	0.883981703
MDH1	1955.924764	-0.09310744	0.117094926	-0.795144952	0.426529189	0.883981703
NKAP	116.8167339	0.185578208	0.233407708	0.795081745	0.426565953	0.883981703
NRG1	866.9099988	-0.274060715	0.344711063	-0.795044731	0.426587483	0.883981703
PGRMC1	2545.797291	0.098525078	0.123938969	0.794948344	0.426643551	0.883981703
MED6	455.2008146	-0.107375246	0.135077782	-0.794914187	0.426663421	0.883981703
DNM1L	1093.484754	-0.095307946	0.119903443	-0.794872469	0.42668769	0.883981703
ADM	3.553045785	-0.891767577	1.122042176	-0.794771886	0.426746207	0.883981703
MYH7B	115.8975762	0.373692708	0.470270506	0.794633523	0.426826712	0.883981703
SINHCAF	1119.944139	0.117204331	0.147536053	0.794411462	0.426955934	0.883981703
MIR4492	3.465368585	1.108517438	1.395560324	0.794317106	0.427010848	0.883981703
PDLIM5	2023.923641	0.085891874	0.108133981	0.794309734	0.427015139	0.883981703
RNF126	288.5953896	0.169703103	0.213663027	0.794255823	0.427046516	0.883981703
SVIL	338.3256319	-0.109306778	0.137635656	-0.794174861	0.427093641	0.883981703
A2M-AS1	10.15171692	0.560773609	0.706114104	0.794168542	0.42709732	0.883981703
CYP2U1	266.4138567	-0.15757991	0.198510059	-0.793813223	0.427304174	0.883981703
SOX11	21.80898367	-0.326826939	0.411741308	-0.79376767	0.427330697	0.883981703
CRIPT	418.204451	0.109021522	0.137350141	0.793748888	0.427341633	0.883981703
POLA2	582.6949463	0.13918834	0.175367738	0.793694109	0.427373531	0.883981703
VCX	3.028711864	1.051945246	1.325394371	0.793684709	0.427379004	0.883981703
ID2	2343.88888	0.436105633	0.549491337	0.793653337	0.427397272	0.883981703
SNORA68	19.59575756	0.402729318	0.507503028	0.793550572	0.427457117	0.883981703
ABCB4	27.19491688	-0.287428244	0.362260578	-0.79342954	0.427527606	0.883981703
DUSP16	496.0301763	0.13384195	0.168700276	0.793371258	0.427561552	0.883981703
AP1G2	489.8711087	0.186005918	0.234451309	0.793366942	0.427564065	0.883981703
LOC100505938	21.11296713	-0.370261195	0.466734478	-0.793301571	0.427602142	0.883981703
ZBTB32	21.67267777	0.367393861	0.463147625	0.793254335	0.427629656	0.883981703
PRH1-PRR4	3.450786309	-0.951520417	1.199524543	-0.793247976	0.42763336	0.883981703
LOC729870	4.269839066	0.878413655	1.107369615	0.793243414	0.427636018	0.883981703
ILKAP	184.0684526	-0.235910926	0.297419901	-0.793191461	0.427666281	0.883981703
ATAD1	1753.975139	-0.099344603	0.125249737	-0.793172149	0.427677532	0.883981703
LOC101927100	14.17033204	0.546013249	0.688405718	0.793156179	0.427686835	0.883981703
GTF2IRD1	657.398732	0.167518309	0.211205401	0.793153529	0.427688379	0.883981703
PPP4R1	1020.847105	-0.142264327	0.1793852	-0.793066135	0.427739292	0.883981703
FANCG	254.0029129	0.20657421	0.260517728	0.792937246	0.427814386	0.883981703
PRKACB	707.9515931	-0.153921522	0.19415204	-0.792788594	0.427901003	0.883981703
MESP1	11.90172135	0.489988004	0.618088623	0.792747165	0.427925145	0.883981703
LOC105371049	27.21543711	-0.471212297	0.594437287	-0.792703128	0.427950807	0.883981703
TH2LCRR	9.907820523	0.686422687	0.86619971	0.792453147	0.4280965	0.883981703
WARS2	329.0982878	-0.11871845	0.149818374	-0.792415825	0.428118255	0.883981703
TTLL7	269.4400255	-0.159851006	0.2017397	-0.792362665	0.428149242	0.883981703
SLC2A1	610.2451348	0.255322884	0.322244539	0.792326488	0.428170331	0.883981703
MFAP2	366.1890146	-0.240373024	0.303376308	-0.792326286	0.428170449	0.883981703
CCDC137	472.2912293	-0.140570443	0.177425018	-0.792280843	0.428196939	0.883981703
GAS1RR	5.142046559	0.826589623	1.043310187	0.792276002	0.428199762	0.883981703
STAG3L5P	16.40965198	0.539396733	0.680889402	0.792194344	0.428247366	0.883981703
BOLA3-AS1	170.9313248	0.1508966	0.190483084	0.792178478	0.428256616	0.883981703
RNF157	548.4924035	-0.232403844	0.293394949	-0.792119442	0.428291035	0.883981703
SNORD15B	10.47921369	-0.477551801	0.602971873	-0.791996813	0.428362535	0.883981703
MYB	14.93554513	0.514727357	0.649936689	0.79196538	0.428380863	0.883981703
CDK5RAP3	1450.866342	0.106829879	0.134896435	0.791939973	0.428395678	0.883981703
HIF1A-AS3	32.62942828	0.438514481	0.553725569	0.791934679	0.428398765	0.883981703
LINC01461	7.811315815	0.568425141	0.71796072	0.791721782	0.428522918	0.88400134
SMC2	1549.180513	-0.088410592	0.111694076	-0.791542356	0.42862757	0.88400134

H4C11	93.24563494	-0.353483819	0.446596443	-0.791506123	0.428648704	0.88400134
RPS28	1415.635335	-0.139346983	0.176081371	-0.791378342	0.428723244	0.88400134
TARID	23.82192824	0.344945867	0.435896598	0.791347923	0.428740991	0.88400134
DPH2	473.8397882	-0.127372555	0.160961077	-0.791325187	0.428754254	0.88400134
IGFBP5	10831.70159	0.609359386	0.77023705	0.79113227	0.42886681	0.88400134
AP3M1	554.2705521	-0.100051303	0.12647896	-0.791050962	0.428914253	0.88400134
MTSS1	92.22884837	0.281478857	0.355868684	0.790962707	0.428965754	0.88400134
HCG25	29.34569377	0.468348599	0.592148033	0.790931613	0.428983899	0.88400134
PHIP	2299.202484	0.135941849	0.171894335	0.790845428	0.429034197	0.88400134
E2F5	346.8956681	0.130780716	0.165374472	0.79081562	0.429051594	0.88400134
HIBCH	387.3444604	0.127808548	0.161616668	0.790812909	0.429053176	0.88400134
PURG	34.95173258	-0.388022031	0.490764306	-0.790648437	0.429149173	0.884096909
ADGRE1	15.53719602	-0.386963898	0.489557702	-0.790435727	0.429273344	0.88425049
TCF25	1071.442315	-0.073510946	0.093032464	-0.79016446	0.429431728	0.884416451
MFN1	2964.076713	0.066301288	0.083918104	0.790071337	0.429486108	0.884416451
FXYD5	196.8966199	0.131637181	0.166620327	0.790042748	0.429502804	0.884416451
HNRNPA3P1	6.195063782	-0.77328056	0.979190152	-0.789714397	0.429694581	0.884709121
LOC105371998	22.70142964	0.467771144	0.592466166	0.789532248	0.42980099	0.884825975
TJP3	3.294940885	0.858221289	1.087179827	0.78940141	0.429877432	0.884833802
NELFE	762.3489084	-0.14106019	0.178714336	-0.789305399	0.429933532	0.884833802
CDS1	14.71102382	0.421066159	0.533496305	0.789257872	0.429961304	0.884833802
NME9	13.19151843	0.446570888	0.565862786	0.789185821	0.430003408	0.884833802
CD320	1161.343151	-0.100440954	0.127340222	-0.788760637	0.43025192	0.88485114
SRSF1	5628.827069	-0.193548503	0.245410062	-0.788673869	0.430302644	0.88485114
CDK6	4021.503924	0.068989998	0.087478023	0.78865521	0.430313553	0.88485114
LOC101927969	17.29602766	0.793790348	1.006518852	0.788649261	0.430317031	0.88485114
UBE3D	171.9456519	0.17638228	0.223671768	0.788576412	0.430359622	0.88485114
PHF7	55.85009294	0.207559681	0.263212461	0.788563278	0.430367301	0.88485114
ENPEP	2445.052158	-0.112915077	0.14320377	-0.788492348	0.430408773	0.88485114
CLEC2B	103.0346299	-0.22643203	0.287171052	-0.788491834	0.430409073	0.88485114
AK4	1146.34968	-0.112894317	0.143236261	-0.788168555	0.430598119	0.885107602
LOC652276	83.31421393	0.220110361	0.279333469	0.787984203	0.430705946	0.885107602
MIR365A	3.372471447	0.836229008	1.061331494	0.787905582	0.430751936	0.885107602
LHX4	85.89182063	0.383523445	0.486852559	0.787760972	0.430836534	0.885107602
POLD1	1813.060338	-0.103840826	0.131821334	-0.787739151	0.4308493	0.885107602
TRPV1	97.23794067	-0.250221826	0.317671633	-0.787674441	0.430887159	0.885107602
VPS13D	2419.671653	0.073552609	0.093388094	0.787601564	0.4309298	0.885107602
KCNT1	4.693153058	-0.812821078	1.032023732	-0.787599211	0.430931176	0.885107602
LZIC	1018.677148	-0.119288244	0.151502216	-0.787369628	0.431065521	0.8852815
SNORA74A	39.28769487	0.357257977	0.453955841	0.78698839	0.431288662	0.88552298
ATP8A2	3.732913057	-0.797865738	1.013968288	-0.78687445	0.431355366	0.88552298
SNORA1	19.23706584	0.669071904	0.85033211	0.786835986	0.431377885	0.88552298
SOCS5	692.1942646	-0.106385238	0.135207541	-0.786829172	0.431381874	0.88552298
LONRF2	2.14624734	1.154011542	1.46750342	0.786377413	0.431646412	0.885963956
ZFYVE9	730.1513427	-0.105646889	0.13437652	-0.786200515	0.431750024	0.886074563
ACVR1B	258.9928745	-0.115437763	0.146885663	-0.785902181	0.431924797	0.886234491
GALK1	323.0127596	-0.129334398	0.164570854	-0.785888843	0.431932611	0.886234491
CAPN10	187.560358	0.164805212	0.209727471	0.78580651	0.431980852	0.886234491
C2orf42	169.2192831	-0.152520066	0.19411307	-0.785727957	0.432026881	0.886234491
PIGT	866.4885167	-0.168585989	0.214637075	-0.78544673	0.432191692	0.886251919
GNL3	1164.931448	0.078228388	0.099608469	0.785358811	0.432243223	0.886251919
PRPF4	1326.319905	-0.080478052	0.102482478	-0.785285963	0.432285924	0.886251919
CDS2	954.0505023	-0.078266479	0.099667209	-0.78527812	0.432290521	0.886251919
TRAPPC6B	422.8379354	-0.116352184	0.148168467	-0.785269542	0.43229555	0.886251919
GPC6	465.6783386	0.250521502	0.319058318	0.785190317	0.432341992	0.886251919
HOXA10-AS	5.503335449	0.741213705	0.944077572	0.785119493	0.432383512	0.886251919
MSX2	88.04121247	0.244632566	0.311679094	0.784886026	0.432520396	0.886319273
KLHDC4	151.6140743	0.26058461	0.332035559	0.784809346	0.43256536	0.886319273
KLRA1P	74.32041464	0.282554749	0.360029973	0.784808962	0.432565585	0.886319273
TRAPPC11	709.8707802	-0.087950665	0.11208692	-0.784664843	0.432650101	0.886390248
TRHDE-AS1	122.829277	0.235029754	0.299595597	0.784490015	0.432752639	0.886390248
C12orf29	528.2619874	-0.08914119	0.113635267	-0.784450043	0.432776085	0.886390248
MIR4484	3.413040763	1.156847127	1.474797827	0.784410654	0.432799189	0.886390248
GMCL1	592.2788107	-0.084914968	0.108296817	-0.784094772	0.432984503	0.886620515
EMC1-AS1	21.65042395	0.452288852	0.576971141	0.783902037	0.433097594	0.886620515
MIR597	3.334439528	1.126554628	1.437220922	0.783842352	0.433132619	0.886620515
RRP1	381.3185375	0.190608316	0.243173458	0.783836848	0.43313585	0.886620515
NUAK1	591.7565828	0.235181003	0.300099069	0.783677884	0.433229143	0.886620515
CENPM	178.0626398	0.210681671	0.268850693	0.783638193	0.433252438	0.886620515
FAM149A	8.750709677	0.594855649	0.759169313	0.78356124	0.433297606	0.886620515
CCT6P1	85.07276103	0.278386892	0.355293462	0.783540713	0.433309656	0.886620515
EML2	143.1189398	0.157562637	0.201147958	0.783317108	0.433440919	0.886696462
KDM4C	365.5332735	-0.124930519	0.15950191	-0.783254064	0.433477933	0.886696462
ATF7-NPFF	2.231844442	1.227134357	1.566774763	0.783223209	0.433496048	0.886696462
CBARP	106.8544585	0.243342043	0.310761106	0.783051799	0.433596695	0.886793887
SNORD114-31	4.217836242	0.966157081	1.23396028	0.782972594	0.433643206	0.886793887
PGAM1P5	6.166965792	0.686948709	0.877926497	0.782467224	0.43394004	0.887299084
MORF4L2-AS1	17.74050316	0.574553785	0.734444984	0.782296561	0.434040307	0.887364735
LINC01144	9.688350189	0.532436536	0.68065357	0.782243066	0.434071739	0.887364735
ZFP14	161.9172654	0.131790666	0.168506594	0.782109844	0.434150022	0.887422964
ST6GALNAC5	19.77415027	0.365247112	0.467122515	0.7819086	0.43426829	0.887562901
PLSCR4	164.6175604	0.261386849	0.33433463	0.78181207	0.434325027	0.887577061
ZNF418	9.294465085	-0.554956835	0.71002535	-0.781601439	0.434448841	0.887728282
GPM6A	83.34825006	0.290110788	0.371235273	0.7814742	0.434523645	0.887743452
CCDC163	31.7036208	0.300422167	0.384457041	0.78141934	0.4345559	0.887743452
PPIL4	404.3993639	-0.092187003	0.118048683	-0.780923603	0.434847429	0.88821431
ZNF764	98.08470773	0.167131601	0.214038187	0.78084945	0.434891046	0.88821431
DHX35	371.8441949	0.165452494	0.21190853	0.780773167	0.434935919	0.88821431
AMPH	257.2174529	-0.168002859	0.215317393	-0.780256793	0.435239739	0.888732916
RGS7BP	4.26842728	0.667157076	0.855379017	0.779954924	0.435417408	0.888824215

EPB41L4A	64.69610691	0.20986394	0.269091654	0.779897617	0.435451141	0.888824215
KCNQ5	533.1179864	-0.116293943	0.149125176	-0.779841109	0.435484406	0.888824215
BRD4	1415.994856	-0.072013852	0.092348156	-0.779808226	0.435503764	0.888824215
DLGAP4	275.9950216	-0.129406595	0.165959242	-0.779749255	0.435538481	0.888824215
S100A3	10.03193278	-0.495287224	0.635299383	-0.779612317	0.435619104	0.888824215
ESPNL	24.69716924	-0.348567348	0.447117556	-0.779587702	0.435633597	0.888824215
TRAPPC8	1339.699633	0.08049546	0.103266999	0.779488717	0.435691882	0.888841366
B3GALT6	1203.62466	-0.08700008	0.111661256	-0.779142945	0.435895515	0.889033229
MIR8055	8.680385066	0.675264279	0.86672833	0.779095658	0.435923368	0.889033229
DOLK	241.2781137	0.11448965	0.14698075	0.778943159	0.436013199	0.889033229
LOC283335	15.88242128	0.36694143	0.471076442	0.778942433	0.436013627	0.889033229
LINC00448	4.553436565	0.720126258	0.924536093	0.778905511	0.436035377	0.889033229
DNAJC24	219.9760096	0.147784774	0.189780698	0.778713405	0.436148558	0.889162259
PSMG3-AS1	38.45045617	-0.28747357	0.369222041	-0.778592658	0.436219705	0.889202497
ZBTB8OS	153.2224212	-0.142379373	0.182886891	-0.778510543	0.436268094	0.889202497
VASH1	335.7660234	-0.148521913	0.190881322	-0.778085102	0.436518845	0.889542265
COL5A2	35286.14345	-0.092447912	0.11884892	-0.777860767	0.436651099	0.889542265
NIPSNAP3B	107.5642615	0.233968374	0.300797834	0.777825991	0.436671603	0.889542265
MORF4L1	4703.626694	-0.099855129	0.12839311	-0.777729655	0.436728406	0.889542265
PWWP3B	99.94982651	0.156330272	0.20103943	0.777610004	0.436798962	0.889542265
ANKRD36C	899.7056568	0.39453623	0.507659812	0.777166561	0.437060508	0.889542265
OSBPL11	275.3817768	0.108548052	0.139683076	0.777102386	0.437098367	0.889542265
ADNP	2756.706449	-0.089965683	0.115790127	-0.776971964	0.437175311	0.889542265
RPL39L	108.7179434	0.159964887	0.205895051	0.77692439	0.437203381	0.889542265
ATP10A	929.7316315	0.276574781	0.355988139	0.776921337	0.437205182	0.889542265
RAB9B	16.06795882	-0.365691802	0.470714887	-0.776885994	0.437226035	0.889542265
SF3B6	762.2315375	-0.151267984	0.19471177	-0.776881564	0.437228649	0.889542265
LMBRD1	394.1798617	-0.155426932	0.200068179	-0.776869827	0.437235575	0.889542265
NECAB1	340.2757534	0.100938595	0.129941198	0.776802093	0.437275542	0.889542265
UBALD1	153.2354153	-0.162064662	0.208670508	-0.776653414	0.437363279	0.889542265
LINC01003	20.19007778	0.386669286	0.497900153	0.776600054	0.43739477	0.889542265
C1orf43	1986.826948	-0.112049031	0.144290574	-0.77655129	0.43742355	0.889542265
SNORA57	4.910303006	0.734779931	0.946410568	0.776386016	0.437521099	0.889542265
MIR758	3.165087715	0.994844799	1.281410965	0.776366698	0.437532502	0.889542265
RPLP0	29356.33146	-0.155362132	0.200154756	-0.776210048	0.437624974	0.889542265
TRNP1	61.03258808	0.221956128	0.285950014	0.77620604	0.43762734	0.889542265
SLC10A4	189.7838458	0.162522925	0.209390205	0.776172529	0.437647124	0.889542265
PCDHGA8	3.772190951	0.919969315	1.185423188	0.776068264	0.437708681	0.889542265
FAM83G	480.3945932	-0.159010818	0.204912086	-0.775995313	0.437751754	0.889542265
COPRS	621.8506812	-0.153551058	0.197889004	-0.775945378	0.437781238	0.889542265
TAF2	2228.96502	0.083249958	0.107288932	0.77594172	0.437783398	0.889542265
PLXDC2	758.5692182	-0.131251882	0.16916487	-0.775881432	0.437818997	0.889542265
ZNF670	124.2883665	-0.153803336	0.198303131	-0.775597115	0.437986904	0.889542265
DDX18	1915.810884	-0.117756342	0.151832484	-0.775567511	0.43800439	0.889542265
CCL28	114.5276941	-0.174388493	0.224892615	-0.775430056	0.438085581	0.889542265
PCCA-DT	41.89762029	-0.247248229	0.318859756	-0.775413719	0.438095231	0.889542265
DNAH3	3.166756728	0.85612666	1.104447404	0.775162907	0.438243403	0.889542265
ZNF419	133.7144311	-0.203656427	0.262733023	-0.775145903	0.43825345	0.889542265
MMP3	2.636898689	0.987997236	1.274823278	0.775007213	0.438335397	0.889542265
FAM157B	55.4346862	0.393692048	0.507990093	0.774999462	0.438339978	0.889542265
LOC101928068	36.38475159	0.452641999	0.584070864	0.774977878	0.438352732	0.889542265
URAHP	17.55870958	0.641211309	0.827486607	0.774890256	0.438404511	0.889542265
LAMTOR5	368.1140261	0.151375826	0.195357016	0.774867622	0.438417886	0.889542265
DPEP2	3.813558264	-0.839898072	1.083947145	-0.774851501	0.438427413	0.889542265
TMEM218	224.96772	0.122917527	0.158635081	0.774844549	0.438431522	0.889542265
REEP4	758.713612	-0.111367657	0.143805293	-0.774433647	0.438674393	0.889811113
NBPF3	101.8319679	-0.23713494	0.306221597	-0.774389991	0.438700201	0.889811113
SLC6A16	14.52703566	0.391539499	0.50562677	0.774364653	0.43871518	0.889811113
LOC101928445	25.18517063	0.487797749	0.629999725	0.774282479	0.438763763	0.889811113
CLTB	131.4552707	-0.187433137	0.242127202	-0.774110201	0.438865625	0.889819772
C4orf46	316.5059888	-0.139985565	0.180835054	-0.774106355	0.4388679	0.889819772
F2RL2	184.4567684	-0.187137972	0.241849824	-0.773777582	0.439062332	0.890073713
MIR548AC	3.209554238	-1.223263267	1.581236189	-0.773611985	0.439160282	0.890073713
ZNF124	405.3439136	0.111621962	0.144324497	0.773409674	0.439279967	0.890073713
ZNF454	7.641800702	0.510977598	0.66076588	0.773311114	0.43933828	0.890073713
LINC02282	17.31265302	0.394243552	0.509827995	0.773287375	0.439352326	0.890073713
ASPN	135.1862406	0.25014257	0.323482222	0.773280735	0.439356255	0.890073713
SYNC	106.4686069	0.304665303	0.394013296	0.773236096	0.439382667	0.890073713
HGF	4179.496374	-0.196605396	0.254271583	-0.773210257	0.439397957	0.890073713
ATG12	607.2560274	-0.098466731	0.127360386	-0.773134681	0.439442678	0.890073713
RRP12	839.7881905	-0.165309265	0.213886319	-0.772883768	0.439591172	0.890239952
MT1X	83.69166326	0.284145236	0.367669829	0.772827177	0.439624668	0.890239952
CCNQ	93.44624533	0.18544619	0.240019255	0.77263047	0.439741106	0.890374561
NPRL2	158.3115353	-0.151831048	0.196549494	-0.772482518	0.439828697	0.890450737
ABRACL	117.7693855	0.156293757	0.202353234	0.772380821	0.43988891	0.890471473
APLF	36.09798828	-0.270590269	0.350473177	-0.772071264	0.440072221	0.890741364
ATAD2B	510.0789044	0.161581355	0.209323757	0.771920769	0.440161355	0.890820597
OAT	666.8402483	-0.116645289	0.151170149	-0.771615886	0.440341962	0.890986843
KCTD21-AS1	15.69056875	-0.394648586	0.511550006	-0.771476066	0.440424803	0.890986843
CRYM-AS1	31.51110975	0.331445002	0.429657155	0.771417393	0.440459569	0.890986843
FAM171A2	269.2675381	-0.189730784	0.245964131	-0.771375825	0.4404842	0.890986843
ZNF136	182.4589536	-0.142093424	0.184221606	-0.771317911	0.440518519	0.890986843
PLEKHA5	587.5438454	-0.108349625	0.14048621	-0.771247406	0.4405603	0.890986843
SRP54-AS1	24.24320788	0.366256647	0.474935145	0.771171918	0.440605037	0.890986843
SOX12	991.4767235	-0.111084191	0.144066689	-0.7710609	0.440670835	0.890986843
RNF224	18.48800007	0.490990915	0.636804769	0.771022673	0.440693492	0.890986843
YEATS2-AS1	74.66816337	0.34596034	0.448766828	0.770913354	0.440758291	0.891016761
ARMCX6	195.9303997	0.122893801	0.159451761	0.770727148	0.440868677	0.891138818
IMMP1L	62.38519609	-0.263530816	0.342065785	-0.77040975	0.441056871	0.89127988
RNF157-AS1	15.97659777	0.51301132	0.665934618	0.770362895	0.441084658	0.89127988

APC2	207.5184538	-0.194041973	0.251886495	-0.770354808	0.441089453	0.89127988
ASIC1	607.5211577	0.140486734	0.182385862	0.77027206	0.441138527	0.89127988
PRPH2	4.668524526	0.791293651	1.027430521	0.770167554	0.441200508	0.891304053
FLJ20021	18.21707458	-0.378180021	0.491178433	-0.769944273	0.44133295	0.891470547
LOC101927018	18.09990204	0.545985963	0.709765788	0.769248072	0.441746058	0.891984762
LOC399975	38.44493563	0.423328042	0.55036132	0.769182039	0.441785252	0.891984762
LINC01297	3.161205698	0.918246939	1.193833134	0.76915853	0.441799205	0.891984762
LOC644656	24.03630819	-0.349631779	0.454580128	-0.769131243	0.441815402	0.891984762
ZNF114	98.63828081	-0.191237376	0.248652953	-0.769093521	0.441837794	0.891984762
TRIM16L	161.5860939	-0.172095923	0.22381864	-0.768907908	0.441947982	0.892026391
UBA7	98.24238018	0.403984226	0.525412155	0.768890142	0.44195853	0.892026391
MIR4523	22.02077227	0.396194046	0.515388855	0.768728392	0.442054567	0.892113214
RETREG2	1093.751682	-0.120299089	0.156528094	-0.76854631	0.442162689	0.892113214
USP3-AS1	60.76958339	0.433788446	0.564458612	0.768503547	0.442188084	0.892113214
PPP1R14B-AS1	26.68502557	0.297723526	0.387418474	0.768480457	0.442201797	0.892113214
CPD	3434.852845	-0.10658056	0.138712293	-0.768356994	0.442275123	0.892160142
MYO6	406.9453794	0.174046447	0.226604725	0.768061863	0.442450433	0.892352088
ITGA1	410.394668	0.113722248	0.148073854	0.768010316	0.442481056	0.892352088
IFITM2	9.06007897	-0.502511744	0.654398557	-0.767898612	0.442547422	0.892352088
PEX3	375.5755253	0.140942909	0.183562033	0.767821681	0.442593132	0.892352088
ORAI1	322.0091866	0.111904086	0.145772506	0.767662499	0.442687721	0.892352088
PEX14	314.1627776	-0.104582009	0.136253378	-0.767555345	0.442751401	0.892352088
FUT2	11.7431801	0.541255802	0.705178674	0.7675442	0.442758024	0.892352088
MIR1204	44.04611548	0.413379544	0.53865183	0.767433657	0.442823724	0.892352088
NHLH2	32.31935171	-0.29332409	0.382235006	-0.76739201	0.442848478	0.892352088
CWF19L2	172.2367494	0.140517609	0.183148859	0.767231691	0.442943774	0.892352088
SLC16A11	12.05698687	0.4269406	0.556654695	0.766975657	0.443095989	0.892352088
CCT4	6356.42444	-0.094521939	0.123252161	-0.766898836	0.443141666	0.892352088
INAFM1	84.39962251	-0.166637753	0.217340919	-0.766711367	0.443253144	0.892352088
TBC1D2	74.6688959	0.234701655	0.306154055	0.766612922	0.443311691	0.892352088
LEF1-AS1	4.887697956	0.824471922	1.075572652	0.766542288	0.4433537	0.892352088
SOX5	97.56616932	0.265964098	0.347092958	0.766261869	0.443520502	0.892352088
LTV1	1725.035153	0.12856937	0.167802951	0.766192546	0.443561743	0.892352088
OTOF	3.405408426	-0.823583025	1.075028864	-0.766103174	0.443614915	0.892352088
GPR19	2.822746325	-0.805811566	1.051834239	-0.766101289	0.443616036	0.892352088
TPBGL	10.860818	-0.543367292	0.709337917	-0.766020367	0.443664183	0.892352088
DGKZ	777.5073085	0.087588298	0.114346233	0.765991985	0.443681071	0.892352088
IKZF2	19.93081481	-1.381386622	1.803475694	-0.765957993	0.443701297	0.892352088
NMUR1	18.92230403	-0.413538159	0.539936916	-0.765900879	0.443735283	0.892352088
GPN1	725.8786198	0.104609163	0.136586623	0.765881465	0.443746836	0.892352088
PHYHD1	5.263149197	-0.716449551	0.935644096	-0.765728715	0.443837737	0.892352088
LOC101929718	9.283020874	0.609016353	0.795476733	0.765599204	0.443914818	0.892352088
SAMD4B	1176.600607	-0.116001251	0.151538113	-0.76549225	0.44397848	0.892352088
SWSAP1	10.96353929	-0.489530238	0.639515773	-0.765470155	0.443991631	0.892352088
CLUHP3	58.46043853	0.396455253	0.518016296	0.765333554	0.444072948	0.892352088
CCNE2	232.7373505	-0.181039149	0.23655052	-0.765329745	0.444075216	0.892352088
NUB1	389.7377943	-0.142839739	0.186670148	-0.765198615	0.444153284	0.892352088
RIPK2	342.9035401	-0.147147691	0.192312198	-0.765150064	0.444182191	0.892352088
NUDT4	111.5477711	0.17469977	0.228332954	0.765109754	0.444206192	0.892352088
LOC100996654	1.864292776	1.129733153	1.476583681	0.765099308	0.444212412	0.892352088
E4F1	517.1183159	0.150389382	0.196612885	0.764900945	0.444330531	0.892352088
GABRA3	113.0901069	-0.272031703	0.355678207	-0.764825332	0.444375561	0.892352088
ADAMTSL1	452.5760223	-0.208247131	0.272308425	-0.764747296	0.444422037	0.892352088
UTP4	1042.473769	-0.15699153	0.205304555	-0.764676313	0.444464315	0.892352088
TP53TG5	21.61630196	-0.330631652	0.432402266	-0.764639036	0.444486518	0.892352088
LOC112694756	204.3951458	-0.146652049	0.191799702	-0.764610412	0.444503568	0.892352088
CADM2	283.4696714	0.283189046	0.370391017	0.764567801	0.444528949	0.892352088
KPTN	167.167591	0.150249302	0.196589199	0.764280554	0.444700071	0.892352088
RNF25	152.927204	-0.160794896	0.210389951	-0.764270799	0.444705883	0.892352088
DDX24	1766.659567	-0.116820741	0.152862206	-0.764222524	0.444734646	0.892352088
GTPBP3	473.0767683	0.135373655	0.177163648	0.764116436	0.444797859	0.892352088
KCNK15	23.86787074	-0.337658401	0.441900926	-0.764104309	0.444805085	0.892352088
PON2	261.8439937	0.161593762	0.211483023	0.764098035	0.444808823	0.892352088
LOC100507071	38.33177469	0.313772776	0.410698395	0.763998057	0.4448684	0.892352088
LSM14A	1716.62863	-0.125057368	0.163706098	-0.763913928	0.444918537	0.892352088
TKFC	362.6502156	-0.119414642	0.156380621	-0.763615349	0.4450965	0.892352088
CCDC58	36.13710542	0.334024973	0.437444227	0.763582995	0.445115786	0.892352088
GVQW3	161.9283869	0.217009701	0.284212707	0.763546794	0.445137366	0.892352088
CREB3	170.0954163	-0.124365584	0.162893973	-0.763475663	0.445179771	0.892352088
MRAP2	27.62199999	-0.322530552	0.422475318	-0.76343052	0.445206684	0.892352088
MIDN	448.927077	0.092952004	0.12176014	0.763402572	0.445223347	0.892352088
MAP3K6	498.1132361	0.11145945	0.146044556	0.763187984	0.445351294	0.892352088
SURF4	6173.417104	0.113820118	0.149150198	0.763124148	0.44538936	0.892352088
CCNL2	3292.358697	0.155877194	0.204279565	0.763058184	0.445428697	0.892352088
LOC101059948	8.072370255	0.53126579	0.696235278	0.76305497	0.445430613	0.892352088
LINC02809	68.34138749	0.284371409	0.372709482	0.7629841	0.445472879	0.892352088
MYH11	64.50444359	-0.313220372	0.410532962	-0.762960349	0.445487043	0.892352088
MCUR1	472.510972	-0.094048179	0.123276222	-0.762906078	0.445519411	0.892352088
UBC	4166.28532	-0.133513723	0.175008934	-0.762896611	0.445525058	0.892352088
ILDR2	8.981356878	-0.503843745	0.660711899	-0.762577071	0.445715663	0.892633526
WASHC4	2767.842631	0.057166342	0.074984001	0.762380524	0.445832927	0.892756713
BBS2	469.5615452	-0.183743167	0.241035959	-0.762306039	0.445877371	0.892756713
ORMDL2	300.1580721	-0.131757762	0.172912636	-0.76199036	0.446065758	0.892956614
STKLD1	68.26092563	0.23078088	0.302949427	0.761780216	0.44619119	0.892956614
BCL7A	488.6033845	0.094966741	0.124670826	0.76173989	0.446215262	0.892956614
AZIN1	8033.26519	-0.093965984	0.123361263	-0.761713862	0.4462308	0.892956614
ELOVL3	3.977875769	-0.815940044	1.07129275	-0.761640591	0.446274542	0.892956614
KAT7	1364.765611	-0.067734932	0.088933584	-0.761635019	0.446277868	0.892956614
THUMPD3-AS1	187.4842396	0.206714203	0.271645479	0.760970525	0.446674672	0.893290566
ALG13	551.0574708	0.194149545	0.255146179	0.760934558	0.446696156	0.893290566

PDLIM3	58.74356444	0.240363345	0.315904207	0.76087415	0.44673224	0.893290566
TRHR	20.0839403	0.449907895	0.591421983	0.760722307	0.446822948	0.893290566
TMEM201	668.9850432	0.177213519	0.232965946	0.760684219	0.446845703	0.893290566
RTN3	1217.641313	-0.116443297	0.153079577	-0.760671664	0.446853204	0.893290566
PCDH15	6.590802485	0.591601591	0.77777978	0.760628659	0.446878897	0.893290566
ZNF252P-AS1	19.62388001	0.524789511	0.689978466	0.760588246	0.446903042	0.893290566
PPP1R3G	32.46421138	-0.293027065	0.385348986	-0.760419971	0.447003589	0.893290566
TRPA1	207.4660611	0.313495283	0.412330558	0.760300873	0.44707476	0.893290566
ZMAT3	3224.78051	0.109666239	0.144252805	0.760236439	0.447113267	0.893290566
PCCA	395.3948818	-0.129037135	0.169741483	-0.760197995	0.447136243	0.893290566
AOX1	21.35780278	0.308989548	0.406492101	0.760136661	0.447172901	0.893290566
DSC3	220.206892	-0.116673565	0.153507881	-0.760049349	0.447225087	0.893290566
PRRT1	2.142531144	1.119418707	1.473075743	0.759919313	0.447302816	0.893290566
CNOT2-DT	23.32225776	-0.385281203	0.507005518	-0.759915206	0.447305271	0.893290566
LOC115308161	9.472069894	-0.627071861	0.825209134	-0.759894475	0.447317665	0.893290566
DHRS11	149.1287882	0.156515368	0.205994967	0.759801906	0.447373004	0.893290566
PTGR2	253.378053	-0.119425639	0.157211904	-0.759647559	0.447465283	0.893290566
SGTB	421.7252368	0.165843858	0.218323901	0.759623008	0.447479962	0.893290566
MIR186	33.10341328	0.352184088	0.463685718	0.759531886	0.447534447	0.893290566
CIP2A	552.4384471	0.169236612	0.222823445	0.759509895	0.447547597	0.893290566
AMMECR1L	826.66757	-0.103805888	0.136708958	-0.759320307	0.447660973	0.893402091
ATF7	413.1059702	0.125053015	0.164734395	0.759119032	0.447781355	0.893402091
EGLN3	443.2794788	-0.126292174	0.166383734	-0.759041593	0.447827676	0.893402091
CDC73	1267.578951	-0.071958668	0.094810269	-0.758975464	0.447867234	0.893402091
ING1	131.4373253	0.166822333	0.219899385	0.758630285	0.448073751	0.893402091
SLC35B1	506.3760078	0.139931469	0.184535303	0.758291054	0.448276762	0.893402091
USP16	606.8458023	-0.08819203	0.116313855	-0.758224637	0.448316514	0.893402091
ZNF705E	6.499846801	0.580073865	0.765073361	0.758193781	0.448334984	0.893402091
LOC728392	29.05985293	0.335391532	0.442381163	0.758150572	0.448360847	0.893402091
ALDH3B1	140.3258445	0.169977874	0.224311225	0.757776943	0.448584528	0.893402091
GZF1	398.1371071	0.092900379	0.122606087	0.757714245	0.448622069	0.893402091
BDH1	59.43652008	0.215507187	0.284421243	0.75770426	0.448628048	0.893402091
RPL12	4167.848772	-0.142349625	0.187920927	-0.757497463	0.448751885	0.893402091
HSD52	6.86343874	0.725607905	0.958373379	0.757124437	0.448975315	0.893402091
ACKR4	11.24571985	0.601419784	0.794491488	0.756987071	0.449057609	0.893402091
LOC105371925	15.56248818	-0.469942346	0.620839407	-0.75694671	0.44908179	0.893402091
STAM-AS1	13.62352371	0.679984146	0.898327386	0.756944692	0.449082999	0.893402091
BEX4	11.02493658	-0.496383825	0.655802048	-0.756911064	0.449103147	0.893402091
HDAC11	16.72981901	-0.395082055	0.521969762	-0.756906019	0.449106169	0.893402091
JAK3	5.159712844	0.636781993	0.841318821	0.756885472	0.44911848	0.893402091
PDK1	446.8244567	-0.220325629	0.291177114	-0.756672205	0.449246271	0.893402091
THPO	3.539036099	0.738999429	0.976691495	0.75663547	0.449268285	0.893402091
PTPRQ	390.2154352	0.248624914	0.32859864	0.756621858	0.449276442	0.893402091
ERVK3-1	13.09126615	-0.403836205	0.533791183	-0.756543416	0.449323452	0.893402091
SNORD65C	3.254071074	1.093670653	1.445639475	0.756530706	0.449331069	0.893402091
CA11	25.58924926	-0.395385479	0.522672745	-0.756468523	0.449368338	0.893402091
ABL1	2068.156982	-0.115092715	0.152178566	-0.756300429	0.449469091	0.893402091
ABCG8	4.120123496	-0.682749352	0.90290983	-0.756165598	0.449549916	0.893402091
CFAP206	5.653498589	-0.621367784	0.82199897	-0.755922825	0.449695468	0.893402091
ZNF821	94.7402465	-0.15581251	0.206162621	-0.755774785	0.449784237	0.893402091
STAU1	707.4982083	-0.108303196	0.143319365	-0.755677337	0.449842675	0.893402091
PSMD13	951.4930493	0.089354208	0.118246707	0.755659171	0.449853569	0.893402091
SCAT2	42.08013587	0.403885505	0.534494842	0.755639667	0.449865267	0.893402091
ADRA1D	25.22978558	0.308490734	0.408272375	0.755600311	0.449888869	0.893402091
C14orf39	208.6034749	0.169302972	0.22406467	0.755598694	0.449889839	0.893402091
PRPSAP1	489.4119546	-0.086468872	0.114443334	-0.755560575	0.449912701	0.893402091
GPR37L1	6.41263568	0.642999529	0.851143708	0.755453542	0.449976898	0.893402091
ADRB2	18.95452278	0.441467251	0.584379565	0.755446078	0.449981375	0.893402091
ABCE1	2842.316944	-0.103908076	0.137553318	-0.755402177	0.450007707	0.893402091
DCK	374.5997864	-0.113208801	0.149874001	-0.755359833	0.450033107	0.893402091
SMKR1	2.243456115	-1.015091251	1.343865229	-0.755351972	0.450037823	0.893402091
LINC00893	31.53398458	0.382548755	0.50648519	0.755300968	0.450068418	0.893402091
PI15	547.8519119	-0.274191638	0.363070249	-0.755202715	0.45012736	0.893402091
PDCD6IPP2	6.769174738	-0.594226064	0.78701414	-0.755038613	0.450225814	0.893402091
SNX4	274.8113614	0.115062879	0.152411455	0.75494902	0.450279572	0.893402091
DNAH5	10.86328839	0.5261702	0.696978733	0.754930064	0.450290946	0.893402091
CDC14B	732.4681974	-0.167170263	0.221458278	-0.754861208	0.450332264	0.893402091
VMAC	85.0652577	0.254701937	0.337475539	0.754727105	0.45041274	0.893402091
MIR647	7.837554538	0.697706043	0.924587131	0.754613621	0.450480849	0.893402091
ERVFRD-1	38.69664587	0.476757686	0.631835525	0.754559798	0.450513153	0.893402091
ERFE	58.85997368	-0.2474992	0.328046982	-0.754462663	0.450571457	0.893402091
CFAP47	404.7302218	0.249044896	0.330115213	0.754418113	0.450598198	0.893402091
PCDH9-AS2	11.16762748	0.866607805	1.148711112	0.754417535	0.450598546	0.893402091
PPM1L	109.7619191	-0.152581985	0.202288129	-0.754280468	0.450680828	0.893402091
JAZF1-AS1	7.999502535	-0.484899327	0.642915767	-0.754219062	0.450717693	0.893402091
HROB	144.447412	0.148202898	0.196506879	0.754186816	0.450737053	0.893402091
DOK5	3.499083054	-0.848266453	1.124835828	-0.754124675	0.450774362	0.893402091
NNT	2160.24111	-0.143174905	0.189867775	-0.754076907	0.450803043	0.893402091
C17orf80	494.2085412	-0.083283286	0.110452853	-0.754016608	0.450839249	0.893402091
ICA1L	113.7631195	0.193339054	0.256414409	0.754010098	0.450843158	0.893402091
SMARCAL1	483.5474714	0.101508596	0.134640601	0.75392263	0.450895681	0.893402091
DIDO1	1696.526308	-0.128885879	0.170956343	-0.753911066	0.450902625	0.893402091
SNORD55	42.43555109	-0.250082768	0.331728223	-0.753878477	0.450922196	0.893402091
MAML3	66.83704066	-0.265866178	0.352671747	-0.75386299	0.450931496	0.893402091
LOC101928063	4.926889015	0.719564931	0.954527852	0.753843829	0.450943003	0.893402091
CDC25A	287.8014147	0.205627915	0.272799929	0.753768212	0.450988415	0.893402091
RBPMS2	19.5125076	0.421726284	0.559534388	0.753709321	0.451023784	0.893402091
MAP2K6	436.7761519	0.110217186	0.146240156	0.753672514	0.45104589	0.893402091
HCG11	715.0569178	-0.157086836	0.20843628	-0.753644405	0.451062773	0.893402091
AP4E1	1340.814854	-0.1087527	0.144438618	-0.752933681	0.451489768	0.893837147

FKBP3	464.6629655	-0.104226617	0.138429764	-0.752920574	0.451497644	0.893837147
PCDHGB5	4.914635446	-0.637863247	0.847326013	-0.752795543	0.451572785	0.893837147
STPG3-AS1	39.24333856	0.301258306	0.400206411	0.752757322	0.451595757	0.893837147
PDZRN3-AS1	22.04183643	0.348152533	0.462538422	0.752699704	0.451630388	0.893837147
TTC5	731.7617138	0.089969486	0.119542964	0.752612137	0.451683022	0.893837147
TLR8	18.52179919	-0.318606718	0.423449355	-0.752408085	0.451805686	0.893837147
C11orf65	26.54899226	0.417872137	0.555379993	0.752407616	0.451805968	0.893837147
TARS2	580.2919529	-0.126222115	0.167760448	-0.752394958	0.451813578	0.893837147
PLCG1	1812.468494	0.070429107	0.093616197	0.75231754	0.451860123	0.893837147
APOC1	10.07250148	-0.467153316	0.621016787	-0.752239433	0.451907083	0.893837147
CFHR1	3.427022448	0.862823219	1.147056726	0.752206233	0.451927046	0.893837147
COQ7	169.3452489	-0.131301031	0.174564967	-0.752161409	0.451953998	0.893837147
LOC102723672	10.63518034	0.457839507	0.608836408	0.751991012	0.452056464	0.893837147
LINC01703	16.13719572	0.38627567	0.513695511	0.751954537	0.4520784	0.893837147
STAB2	26.01223505	0.393354202	0.523116624	0.751943607	0.452084972	0.893837147
UBXN11	209.5643418	-0.158449133	0.210770682	-0.751760783	0.45219493	0.893887063
LOC101928008	10.72717004	-0.575389738	0.765415834	-0.751734826	0.452210543	0.893887063
SNX29	439.9709038	-0.137829664	0.18339691	-0.75153755	0.452329211	0.89396177
ZNF746	200.365807	-0.113228473	0.150675016	-0.751474767	0.452366981	0.89396177
C9orf72	22.6662084	0.347740044	0.46286565	0.751276411	0.452486323	0.89396177
KCTD4	7.601361678	0.756775769	1.0073308	0.751268371	0.45249116	0.89396177
NBR1	1931.134819	-0.208226623	0.277171673	-0.751255064	0.452499167	0.89396177
SLC12A7	18.89294024	0.725815365	0.966329062	0.751105802	0.452588984	0.894040095
ADAMTS1	6115.508754	-0.14432854	0.19221684	-0.750863141	0.452735025	0.894229455
FBXL18	865.2618138	0.075714101	0.100902533	0.750368686	0.453032685	0.894554849
UBBP4	11.05654298	-0.459197306	0.611999896	-0.750322524	0.45306048	0.894554849
GRIPAP1	275.0469043	0.126595678	0.168727008	0.750298841	0.453074741	0.894554849
CHP1	2339.168724	-0.19471994	0.25955803	-0.750198098	0.453135404	0.894554849
MTUS2	6.350563673	0.656160856	0.874722186	0.750136291	0.453172624	0.894554849
ZNF28	323.5877937	0.121424065	0.161897438	0.750006095	0.453251034	0.894554849
ASPHD1	36.56795609	0.297965535	0.397284265	0.75000588	0.453251163	0.894554849
PASK	349.9067521	0.205618486	0.274219177	0.749832626	0.453355516	0.894661717
SNORA119	4.262263288	0.773166879	1.031291869	0.749707142	0.453431105	0.894667399
BET1L	783.9211478	0.111242925	0.148435439	0.749436429	0.453594201	0.894667399
AKAP8	398.9857329	0.115623453	0.154292655	0.749377561	0.453629672	0.894667399
ADCY10	3.939164645	0.721608693	0.96297196	0.749355872	0.453642741	0.894667399
DAPK2	103.1927592	0.329856834	0.44020442	0.749326493	0.453660444	0.894667399
DUSP14	284.0951089	0.110280405	0.147182282	0.749277719	0.453689834	0.894667399
PCDHGA9	13.5122764	-0.469511894	0.626675337	-0.749210741	0.453730196	0.894667399
LINC01358	47.88726855	-0.31817549	0.424735189	-0.749114975	0.45378791	0.894667399
RAB21	955.038167	0.092026722	0.122855509	0.749064671	0.453818228	0.894667399
TMEM44	843.2084225	0.084230335	0.112457866	0.74899461	0.453860454	0.894667399
IGF1R	2162.859995	0.162865909	0.21749519	0.748825338	0.453962486	0.894713523
S100A1	7.158721417	0.649822772	0.867877827	0.748749135	0.454008423	0.894713523
ALMS1-IT1	33.2220664	0.377150073	0.503735933	0.748705915	0.454034477	0.894713523
LYSMD1	244.7655771	0.167662595	0.22401671	0.748437893	0.454196073	0.894721582
ANGPTL2	1290.373963	-0.53881823	0.719983964	-0.748375321	0.454233804	0.894721582
SAMD13	36.11562627	-0.249785291	0.333857382	-0.748179624	0.45435182	0.894721582
FAM72D	2.659439716	-0.841525243	1.124853962	-0.748119553	0.454388049	0.894721582
ANG	17.0932874	0.484117915	0.647465879	0.747711857	0.454633978	0.894721582
USP18	26.90214729	0.297701703	0.398158387	0.747696677	0.454643136	0.894721582
KHNYN	1547.370196	0.155900532	0.208536358	0.747594011	0.454705078	0.894721582
PGLYRP1	5.917623964	0.662662578	0.886437779	0.747556787	0.454727538	0.894721582
LOC101593348	7.528521239	-0.740582515	0.990705219	-0.747530648	0.45474331	0.894721582
DZANK1	42.73652594	-0.320061968	0.428184123	-0.747486772	0.454769785	0.894721582
COX6A1	1218.682257	-0.105962734	0.141760333	-0.747478027	0.454775061	0.894721582
MIR125B1	9.905731042	0.543208146	0.7267671	0.747430843	0.454803534	0.894721582
PLCH2	7.027363445	0.590952372	0.790658416	0.747418051	0.454811253	0.894721582
FAM8A1	694.5204078	-0.136147702	0.182168247	-0.747373401	0.454838197	0.894721582
YWHAG	2677.780104	0.093706946	0.125386223	0.74734643	0.454854473	0.894721582
RNF128	229.0023261	0.171818119	0.229985883	0.747081155	0.455014575	0.894721582
RPL18A	4416.202761	-0.169843216	0.22734343	-0.747077742	0.455016635	0.894721582
SFTPB	4.932169007	0.702873563	0.940845658	0.747065745	0.455023876	0.894721582
UBXN7-AS1	14.17487838	0.541064162	0.72445224	0.746859671	0.455148272	0.894721582
GBX2	64.05177289	0.327774499	0.438903048	0.74680388	0.455181953	0.894721582
KCND2	9.851644693	0.526506134	0.705110192	0.746700501	0.455244367	0.894721582
CCT7	4338.466368	-0.08712505	0.11668948	-0.746640138	0.455280813	0.894721582
CCDC138	224.8964082	0.200849064	0.269025226	0.746580782	0.455316652	0.894721582
RBM39	2616.330044	-0.165036866	0.221065688	-0.746551253	0.455334483	0.894721582
HEBP2	428.7705516	-0.089221996	0.11951527	-0.74653219	0.455345994	0.894721582
TMED4	935.6151999	0.116642593	0.156261485	0.746457727	0.455390959	0.894721582
LNCAROD	5.744234316	0.677758537	0.908128178	0.746324752	0.455471263	0.894721582
MHENCR	40.89946343	0.302163896	0.404905338	0.74625812	0.455511505	0.894721582
CCDC121	28.42197528	-0.303456763	0.406642712	-0.746249113	0.455516945	0.894721582
ALDH1A1	29.20316899	-0.330367467	0.442731393	-0.74620294	0.455544832	0.894721582
TSR2	352.6429487	-0.088938133	0.119230115	-0.745936825	0.455705579	0.894892861
ZNF320	135.8870395	0.214062059	0.287005368	0.745846882	0.455759916	0.894892861
TMEM95	2.227417724	1.050201167	1.408204724	0.745773075	0.455804507	0.894892861
NKX2-5	12.21507876	-0.433296346	0.581161612	-0.745569454	0.455927541	0.894892861
USF1	373.5548638	0.110811742	0.148634822	0.745530154	0.455951289	0.894892861
NR4A2	28.03500398	-0.328737392	0.440968913	-0.745488814	0.455976271	0.894892861
FREM1	21.12366275	-0.345307157	0.463223142	-0.745444529	0.456003033	0.894892861
SLC17A7	3.162007538	1.334798712	1.790729939	0.745393642	0.456033786	0.894892861
FGF14-AS2	2.532209773	0.842296279	1.130172178	0.745281379	0.456101636	0.894927455
MCMDC2	66.30776898	0.24209598	0.324922234	0.745088994	0.456217922	0.895057071
ZNF382	179.9646548	-0.178931243	0.240175328	-0.745002596	0.45627015	0.895060995
DDX19A	621.3204032	-0.084832309	0.113919119	-0.744671393	0.456470397	0.895180982
LINC01629	2.684331454	-0.877039791	1.177764779	-0.744664645	0.456474478	0.895180982
CR2	17.88716239	0.428025083	0.574798676	0.744652172	0.456482019	0.895180982
SPPL3	734.614415	-0.089695633	0.120474167	-0.744521712	0.456560911	0.895237173

ELAVL1	1828.276055	0.080463085	0.108105638	0.744300542	0.456694674	0.895400933
ZNF416	102.5526985	-0.171514721	0.230545963	-0.743950225	0.456906589	0.895633947
SPC24	852.3057656	-0.113740265	0.152890244	-0.743934093	0.456916349	0.895633947
PCDHGC3	412.8097199	0.212946392	0.286274125	0.743854836	0.456964301	0.895633947
REV1	1011.804721	0.110285791	0.148299971	0.743666981	0.45707797	0.895758213
LAMA1	5.153683049	0.612084166	0.823213195	0.743530558	0.457160528	0.895821487
ATP1B3	2075.252275	0.099142426	0.133360871	0.743414658	0.457230672	0.895852583
RNF227	48.69019768	0.272386625	0.366437	0.743338214	0.457276941	0.895852583
DUSP12	261.5956392	-0.168254031	0.226451176	-0.743003565	0.457479522	0.89589821
NUFIP1	260.4392122	-0.132391073	0.178185871	-0.742994228	0.457485175	0.89589821
SNRPB2	286.5225125	-0.108413893	0.145916675	-0.742984948	0.457490793	0.89589821
CFAP44-AS1	3.667601278	0.981750376	1.321474236	0.742920557	0.457529779	0.89589821
MIR655	7.70950608	0.740566977	0.996880361	0.742884508	0.457551605	0.89589821
TMEM254-AS1	20.81089658	-0.405092153	0.545398657	-0.742745051	0.457636049	0.895965105
RBMS3-AS3	2.153035674	-1.148964401	1.54721939	-0.742599536	0.457724169	0.896039185
ARMCX3	776.4634699	-0.094605263	0.127414887	-0.74249772	0.457785832	0.89606146
RIC3	12.14370724	0.609656038	0.821352481	0.742258716	0.4579306	0.896092522
C14orf132	1207.934281	-0.125726356	0.169412406	-0.742131931	0.458007405	0.896092522
SARM1	90.06595703	0.182673209	0.246168983	0.74206428	0.458048391	0.896092522
TSPAN4	577.9271046	-0.138831952	0.187112495	-0.741970503	0.458105208	0.896092522
PLBD1-AS1	16.99920221	0.352873349	0.475602087	0.7419508	0.458117145	0.896092522
IFNE	12.36137773	0.634524167	0.855468374	0.741727206	0.458252633	0.896092522
GLO1	5285.784078	-0.074063338	0.099870289	-0.741595305	0.458332569	0.896092522
BAD	124.0674734	-0.18279553	0.246493387	-0.741583909	0.458339475	0.896092522
LOXL1-AS1	128.0180076	0.157844184	0.212886126	0.741448899	0.458421305	0.896092522
ZNF148	914.5126062	-0.09870759	0.133133605	-0.741417538	0.458440314	0.896092522
SCART1	101.3348795	0.303835802	0.409805101	0.741415374	0.458441626	0.896092522
SNAPC4	526.5064134	0.268152287	0.361697408	0.74137188	0.458467989	0.896092522
TBC1D13	720.0553346	-0.081810002	0.110353902	-0.741342179	0.458485994	0.896092522
LINC01881	16.06877288	-0.376620801	0.508047876	-0.741309666	0.458505702	0.896092522
SNAI3-AS1	136.5722015	0.194959415	0.263027752	0.741212337	0.458564704	0.896109554
RPS12	2538.678041	-0.12796161	0.172695841	-0.740965211	0.458714534	0.896246863
BCL11B	4.131906072	-0.70927416	0.957274835	-0.740930539	0.458735558	0.896246863
MBLAC1	18.08382031	0.41681817	0.562749876	0.740681052	0.458886851	0.896444165
GCSH	361.4645035	-0.169979138	0.229589054	-0.740362552	0.459080035	0.896723252
MARK4	457.3324416	0.170068707	0.229758365	0.740206814	0.459174514	0.896809495
OGG1	123.5377471	-0.133427851	0.180314624	-0.739972431	0.459316723	0.896918333
MAGI1-AS1	5.271604884	1.085145735	1.466659951	0.73987548	0.459375554	0.896918333
SAMD9L	155.8494281	-0.166507346	0.225072016	-0.739795862	0.45942387	0.896918333
ERAP1	407.8167326	-0.120775262	0.163257649	-0.739783177	0.459431568	0.896918333
FAM225A	35.72799114	-0.402148563	0.543837287	-0.73946486	0.45962477	0.897058637
GLRX	54.98102972	-0.254848085	0.344653551	-0.739432639	0.459644329	0.897058637
ACTR3B	142.5199313	-0.203391921	0.275071046	-0.739415956	0.459654456	0.897058637
SNHG1	829.6253221	0.142945445	0.193397855	0.739126316	0.459830298	0.897201564
MEGF8	2030.521187	-0.094038434	0.127240617	-0.73905987	0.459870643	0.897201564
ZNF184	443.8002336	0.124093625	0.167910433	0.739046541	0.459878737	0.897201564
LINC02246	103.7973221	0.299941597	0.405914113	0.738928722	0.45995028	0.897225495
KBTBD2	818.1323613	-0.102278374	0.138427176	-0.738860514	0.459991702	0.897225495
EIF1B	265.3643031	-0.149452838	0.202314684	-0.738714737	0.460080235	0.897299966
PRSS21	912.8783461	-0.109093175	0.147709464	-0.738565908	0.460170632	0.897378055
CCDC116	2.681459563	0.995193815	1.347627274	0.738478535	0.460223706	0.897383351
OBSCN-AS1	13.89373624	0.467061933	0.632873711	0.738001793	0.46051336	0.897796822
STBD1	14.38163584	0.458179364	0.620887002	0.737943237	0.460548943	0.897796822
PDE10A	6.607927073	0.96437138	1.306947418	0.73788078	0.460586899	0.897796822
UACA	374.6923952	-0.093342874	0.126517312	-0.737787362	0.460643675	0.897809284
GOLGB1	1182.77692	-0.115248613	0.156264477	-0.737522791	0.460804488	0.897842126
ZNF778	213.06717	0.135807849	0.184161392	0.737439307	0.460855239	0.897842126
RABL2B	182.1171394	-0.12542818	0.170111451	-0.737329437	0.460922035	0.897842126
C17orf113	64.95770071	0.358259156	0.485983169	0.737184288	0.461010287	0.897842126
HIRA	855.2683006	-0.126897204	0.172137917	-0.737183335	0.461010866	0.897842126
DNMT1	4619.365188	-0.092440247	0.125418244	-0.737055824	0.461088402	0.897842126
SEC16A	3067.056744	0.093340566	0.126645189	0.737024176	0.461107647	0.897842126
LOC101060553	5.608936583	-0.701631141	0.952048806	-0.736969719	0.461140764	0.897842126
PITX2	2491.789329	-0.107046157	0.145257324	-0.736941549	0.461157896	0.897842126
MFAP3L	48.47824979	0.257722741	0.349744482	0.736888656	0.461190063	0.897842126
TXNDC5	730.207172	0.146741739	0.199152529	0.736830906	0.461225186	0.897842126
RBM6	493.6576197	0.177724052	0.241291477	0.736553377	0.461393997	0.897842126
EGLN2	18.76007748	-0.35642157	0.483910506	-0.736544392	0.461399462	0.897842126
LOC101927699	3.049556786	0.900374465	1.222562764	0.736464819	0.46144787	0.897842126
ATP6V1E1	558.5665159	-0.13534045	0.183774217	-0.736449609	0.461457123	0.897842126
CENPW	109.8514838	-0.164260717	0.223051541	-0.73642494	0.461472132	0.897842126
ANKRD29	2.269115471	-1.069122618	1.451919621	-0.736351106	0.461517052	0.897842126
MRPS6	146.6869417	0.144349436	0.196063524	0.736238101	0.461585809	0.897877864
ANKEF1	47.53884487	-0.198555707	0.269813153	-0.73590077	0.461791089	0.898179132
PRPF18	154.5354091	-0.17016522	0.231274051	-0.735773077	0.461868809	0.898215432
LINC01920	3.143677117	-0.823580613	1.119550888	-0.735634817	0.461952968	0.898215432
ZIC4	3.339130041	0.787824704	1.070964505	0.735621677	0.461960967	0.898215432
LGALS8-AS1	4.147613733	-0.787052222	1.070338469	-0.73533022	0.462138408	0.898462407
ETFDH	170.9983658	0.127424185	0.173342074	0.735102459	0.462277097	0.898479284
RP2	290.5268894	0.108367629	0.147458878	0.73490068	0.462399984	0.898479284
POLR3GL	138.8796067	-0.196250645	0.267045992	-0.734894552	0.462403717	0.898479284
SPSB1	74.39405774	0.283511761	0.385887404	0.734700738	0.462521771	0.898479284
P3H4	1699.761976	0.123984772	0.168809955	0.734463627	0.462666221	0.898479284
CDPF1	114.3614429	-0.157181132	0.214016348	-0.734435164	0.462683562	0.898479284
DENND10P1	6.769145276	0.543895129	0.740649933	0.734348449	0.462736397	0.898479284
NARS2	346.0592864	0.120089643	0.163545587	0.734288495	0.462772928	0.898479284
HOPX	34.4928808	0.400766999	0.545877851	0.734169739	0.462845294	0.898479284
PKD1P5-LOC105376752	197.6068442	-0.166012836	0.226131793	-0.73414195	0.462862229	0.898479284
ESR1	10.72441362	0.5384455	0.733541554	0.734035444	0.462927136	0.898479284
FAM71F2	15.78754026	-0.397433316	0.541450807	-0.734015557	0.462939256	0.898479284

RDH5	7.512994216	0.627863373	0.855411771	0.733989634	0.462955056	0.898479284
MED19	139.8995592	0.151863417	0.206920624	0.733921128	0.46299681	0.898479284
MMP2	4511.087454	-0.200187492	0.272774369	-0.733894072	0.4630133	0.898479284
PLEKHG5	85.60002081	0.195119054	0.265898788	0.73380949	0.463064856	0.898479284
SPART	1231.75513	-0.090677711	0.123579786	-0.733758439	0.463095975	0.898479284
RELL1	191.3967628	0.172080773	0.234560234	0.733631487	0.463173365	0.898479284
EEF2K	1977.921842	-0.079452873	0.108306315	-0.733594092	0.463196163	0.898479284
TMEM231	278.3263498	0.120526409	0.164307332	0.733542487	0.463227625	0.898479284
KDM1A	2536.986016	-0.06465684	0.08814701	-0.733511445	0.46324655	0.898479284
PKDREJ	69.99408215	0.190987815	0.26040544	0.733424829	0.46329936	0.898479284
SPG11	2158.86406	0.055425082	0.075578388	0.733345646	0.463347642	0.898479284
TBC1D19	202.8951948	-0.132840629	0.181147127	-0.733330033	0.463357162	0.898479284
LOC101929555	5.707472471	0.582890295	0.795197901	0.733012869	0.463550581	0.898756539
B3GALNT2	877.4524887	-0.125476586	0.171213097	-0.732867915	0.463638994	0.898830165
TGFBR3L	20.23157333	-0.338068002	0.461442563	-0.732632897	0.463782361	0.898851151
OIP5-AS1	5200.905092	0.128105271	0.174879998	0.732532437	0.463843652	0.898851151
CAHM	24.1050248	0.287486538	0.392509268	0.732432484	0.463904638	0.898851151
MTCH1	3449.395638	-0.114740436	0.156662699	-0.732404308	0.46392183	0.898851151
CHUK	623.2569078	0.095453693	0.130336296	0.732364631	0.46394604	0.898851151
CDCP1	46.9367648	-0.824275713	1.125515246	-0.732354107	0.463952463	0.898851151
POU5F1P4	7.738549464	0.720984148	0.984737974	0.732158368	0.464071912	0.898915443
CLUH	3038.916063	0.141230684	0.192918349	0.732074919	0.464122841	0.898915443
ERGIC3	2340.118129	0.179026893	0.244554969	0.732051753	0.464136981	0.898915443
ZFAND2B	148.75166	0.132067509	0.180473315	0.731784134	0.464300334	0.899134097
TAS2R31	31.99063323	0.572504885	0.782610618	0.731532223	0.46445413	0.899151477
LOC100130027	110.4122717	0.191128446	0.261289631	0.731481177	0.464485298	0.899151477
CEP44	438.0938097	0.102588276	0.14025645	0.731433572	0.464514365	0.899151477
SLBP	886.7167317	-0.117128657	0.160147751	-0.731378721	0.464547859	0.899151477
MIR497HG	8.924696659	-0.584592484	0.79941001	-0.731279915	0.464608196	0.899151477
TBK1	464.6220684	-0.103533847	0.14158018	-0.731273598	0.464612054	0.899151477
HAGLR	801.770777	-0.112762533	0.154271957	-0.730933441	0.464819808	0.899423086
LYRM9	2.987345171	-0.805521818	1.102128159	-0.730878539	0.464853346	0.899423086
SCRT1	5.609368543	0.588320475	0.805096755	0.730745059	0.464934888	0.89943408
SMIM19	480.2770216	-0.199008053	0.272351122	-0.730703995	0.464959975	0.89943408
CCDC196	11.36818173	-0.615983178	0.843105525	-0.730612195	0.465016061	0.899444938
TNRC6A	1623.295056	-0.156280996	0.213969211	-0.730390112	0.46515176	0.899609765
MIR5047	354.4267443	-0.445443387	0.610030702	-0.730198309	0.465268976	0.899689438
TAGAP	3.497112543	-0.994100246	1.361572876	-0.730111669	0.465321929	0.899689438
STXBP1	437.1043039	0.249124221	0.341332406	0.729858098	0.465476927	0.899689438
DPH5	424.3230121	-0.116436205	0.159563757	-0.729715864	0.465563882	0.899689438
GTF2IP12	16.41662525	0.38251425	0.524347343	0.729505459	0.46569253	0.899689438
RPL34-AS1	35.6770811	0.386336274	0.529768446	0.729254974	0.465845709	0.899689438
NDUFA10	1053.258207	0.088110792	0.120823672	0.729251068	0.465848098	0.899689438
JUND	263.0350089	-0.132407709	0.181572955	-0.729225942	0.465863465	0.899689438
ANTKMT	104.5321515	-0.201931896	0.276919817	-0.729207099	0.465874989	0.899689438
PLA2G6	141.7503313	-0.175164015	0.240229516	-0.729152764	0.465908222	0.899689438
ZNF704	131.3922012	0.334905032	0.459308969	0.729149777	0.465910049	0.899689438
PKIA	248.0555685	-0.114810521	0.157466821	-0.729109288	0.465934813	0.899689438
USP45	883.9443751	0.166309241	0.228107255	0.729083521	0.465950574	0.899689438
EEF1AKMT3	274.6664215	0.122318276	0.167770223	0.729082155	0.46595141	0.899689438
IFITM10	10.03928371	0.69542482	0.953899969	0.729033277	0.465981307	0.899689438
NUP88	717.8695352	-0.143884968	0.197397607	-0.728909382	0.466057095	0.899689438
ZNF608	340.732931	-0.128324804	0.176061099	-0.728865176	0.466084138	0.899689438
TRIM66	148.5095244	-0.226183106	0.310347711	-0.728805458	0.466120672	0.899689438
LINC02562	2.072998792	-0.90762312	1.245445316	-0.728753891	0.466152221	0.899689438
CERS1	14.30039444	0.406051966	0.557454461	0.728403832	0.466366418	0.89993249
TRIM71	1.792870454	1.112022798	1.526763799	0.728352872	0.466397605	0.89993249
MAST4	185.3491098	-0.199519111	0.273951632	-0.7283005	0.466429657	0.89993249
MIR4426	38.34286456	-0.239852912	0.329386997	-0.72817966	0.466503615	0.899977744
ACYP1	82.58885783	0.283843731	0.389948839	0.727899925	0.466674849	0.900210631
LOC101928510	9.318009409	0.723724494	0.994446312	0.72776628	0.466756669	0.900244848
CUL7	2821.270541	0.140281536	0.192807024	0.727574819	0.466873899	0.900244848
MXD3	147.1039739	-0.165096651	0.226922068	-0.7275478	0.466890445	0.900244848
VPS37C	145.6677553	0.140413009	0.192996719	0.727540912	0.466894662	0.900244848
BAIAP2	434.4667195	0.223697865	0.307618307	0.727192953	0.467107763	0.9003426
PALM	11.31627811	-0.436579757	0.600363483	-0.727192392	0.467108106	0.9003426
CD81-AS1	6.166417229	0.605942833	0.833277975	0.727179706	0.467115876	0.9003426
MFAP4	789.1789461	-0.263829899	0.362915306	-0.726973744	0.46724204	0.9003426
ACRV1	17.26376563	0.427475092	0.588026625	0.726965538	0.467247067	0.9003426
ABCD2	61.61955459	-0.245404859	0.337575357	-0.726963191	0.467248505	0.9003426
KCNG3	68.35062468	0.261847193	0.360314911	0.726717615	0.46739896	0.900395977
SNHG11	69.51881548	-0.214485793	0.295184007	-0.726617253	0.467460456	0.900395977
CAPN10-DT	60.60446091	0.40433846	0.556518174	0.726550325	0.467501468	0.900395977
KDM6B	640.238646	0.08546689	0.117656579	0.726409784	0.467587595	0.900395977
PTPN7	3.148649523	-0.73302729	1.009231031	-0.726322584	0.467641038	0.900395977
ISOC1	225.3734192	0.145974969	0.201076366	0.725967813	0.467858503	0.900395977
WDFY3	1197.922284	0.072280855	0.099567901	0.725945354	0.467872271	0.900395977
MAMDC4	214.3945489	0.234342268	0.322830359	0.725899104	0.467900626	0.900395977
MND1	52.09863202	0.212766512	0.293111467	0.725889418	0.467906564	0.900395977
HMGN2P46	2.543991734	1.023279733	1.409742398	0.72586292	0.46792281	0.900395977
TMEM35B	182.843574	0.14926301	0.205700231	0.725633654	0.468063384	0.900395977
MYH3	42.5655287	0.274649386	0.378616359	0.725402851	0.468204924	0.900395977
FARSA	1323.063201	0.096908818	0.13363366	0.725182699	0.468339955	0.900395977
MOB3A	324.8212942	-0.118716918	0.163707755	-0.725175897	0.468344128	0.900395977
PTRH1	14.67758583	-0.349020188	0.481291778	-0.725173799	0.468345414	0.900395977
TRPM5	2.145049645	-0.989185164	1.364205085	-0.725100042	0.468390659	0.900395977
NDUFB9	547.1050565	-0.142097394	0.195986972	-0.725034899	0.468430621	0.900395977
GATD1	695.2487586	0.200260866	0.276218976	0.725007633	0.468447348	0.900395977
DCAF1	664.4321228	-0.114640213	0.158131314	-0.724968447	0.468471388	0.900395977
TMCO6	331.7206803	0.117638146	0.162274903	0.724931234	0.468494218	0.900395977

ELOVL4	304.0935994	0.116945882	0.161344373	0.72482157	0.468561501	0.900395977
HNRNPAB	2821.471167	-0.14726115	0.203215181	-0.724656246	0.468662944	0.900395977
MARCKS	11975.20759	-0.112409384	0.155160023	-0.724473878	0.468774858	0.900395977
ZNF85	89.93132074	0.181766047	0.250919026	0.724401214	0.468819455	0.900395977
XPNPEP1	628.9683805	-0.112422483	0.155211065	-0.724320027	0.468869284	0.900395977
CARNMT1	763.331199	-0.099882579	0.137927347	-0.724168056	0.468962567	0.900395977
ANKRD34A	111.9598465	0.183545378	0.253511347	0.724012477	0.469058075	0.900395977
INKA2-AS1	2.929982201	0.788997396	1.089948025	0.723885339	0.469136131	0.900395977
TIMMDC1	747.4324284	-0.09133174	0.126173195	-0.723860092	0.469151633	0.900395977
CARMIL2	12.57020264	0.40461807	0.559017966	0.72380155	0.469187578	0.900395977
C22orf23	112.8128128	0.289120332	0.39960631	0.723512929	0.469364814	0.900395977
DTD2	308.4987047	-0.105710975	0.146118678	-0.723459702	0.469397504	0.900395977
LOC101927267	19.26900062	0.433754203	0.599558762	0.723455699	0.469399962	0.900395977
CLHC1	138.678611	0.184987829	0.255735	0.723357496	0.469460277	0.900395977
GDPD5	95.47407623	-0.18896994	0.261254924	-0.723316281	0.469485592	0.900395977
TUBB4A	2.242758199	1.037777584	1.434872241	0.723254346	0.469523636	0.900395977
LINC01681	4.437428902	0.649418808	0.898040143	0.723151202	0.469586995	0.900395977
LOC100132077	10.18621547	0.785795328	1.086661263	0.723128131	0.469601168	0.900395977
FGGY	1104.08185	-0.126551466	0.17502105	-0.72306426	0.469640406	0.900395977
CYC1	1979.649094	0.090637677	0.125374339	0.72293643	0.469718941	0.900395977
COL24A1	585.506601	0.087993224	0.121719215	0.722919747	0.469729191	0.900395977
GFPT2	404.8131391	0.203895567	0.282054386	0.722894508	0.469744698	0.900395977
STARD13-AS	24.50432358	0.501859792	0.694237981	0.722893021	0.469745612	0.900395977
DESI2	1249.446946	0.103648899	0.143415599	0.72271705	0.469853738	0.900395977
SEC22B	295.9500112	-0.12698011	0.175698454	-0.722716147	0.469854293	0.900395977
GRHL1	8.71368987	0.493886434	0.683412783	0.722676612	0.469878588	0.900395977
SELENOW	509.3576141	-0.245535055	0.339794321	-0.722599056	0.469926249	0.900395977
ARFGEF3	4.45876333	-0.675050138	0.93426976	-0.722543067	0.469960657	0.900395977
C1QTNF9B	2.336177338	0.996402326	1.379113546	0.722494771	0.469990339	0.900395977
WWC2-AS1	2.747309562	-0.83448088	1.155232733	-0.722348715	0.470080109	0.900395977
RRS1-AS1	4.086400237	0.878710514	1.216666465	0.722227939	0.470154349	0.900395977
EPX	11.2092892	-0.435373814	0.602863311	-0.722176662	0.47018587	0.900395977
SFRP4	33.13058372	0.380837089	0.527346513	0.722176177	0.470186168	0.900395977
PIGK	1205.24367	-0.090847978	0.125804076	-0.722138588	0.470209276	0.900395977
BMS1P22	101.0016671	0.371009355	0.513768199	0.722133748	0.470212251	0.900395977
LOC339803	385.8089325	-0.161784788	0.224039834	-0.722125101	0.470217567	0.900395977
ALDH5A1	301.8470782	-0.104781998	0.145107469	-0.722099269	0.470233447	0.900395977
MCHR2	4.352579547	-0.641113503	0.88820823	-0.721805407	0.470414125	0.900395977
FOSL1	69.60401297	0.27439102	0.380173487	0.72175212	0.470446892	0.900395977
TBX4	16.55862524	0.369439959	0.511877785	0.721734698	0.470457605	0.900395977
MRPS25	774.3598685	0.189277749	0.262266005	0.721701422	0.470478067	0.900395977
PTPRU	416.6678004	0.25826787	0.357867705	0.721685322	0.470487969	0.900395977
CASC4	1665.64246	-0.135911164	0.188334174	-0.721648976	0.47051032	0.900395977
TCAF2P1	1.983560461	1.053565141	1.460113519	0.721563856	0.470562668	0.900395977
DLGAP5	1270.738684	-0.075217785	0.10425503	-0.721478717	0.470615031	0.900395977
MIR421	4.582455615	0.787171833	1.091094188	0.72145177	0.470631605	0.900395977
NUP93	1325.334313	0.097741892	0.135511263	0.721282422	0.47073577	0.900395977
LRRC42	314.9172041	-0.122617358	0.170030064	-0.721151044	0.470816589	0.900395977
ABL2	2335.760676	-0.097416729	0.135086337	-0.721144205	0.470820796	0.900395977
WDR36	1607.357133	0.086805551	0.120388002	0.721048192	0.470879865	0.900395977
DCHS1	1116.569248	0.205290081	0.284718125	0.721029199	0.47089155	0.900395977
RAB30	229.4049479	0.129916037	0.180197148	0.720966112	0.470930365	0.900395977
CDK20	103.6368756	0.177138661	0.245715254	0.720910313	0.470964697	0.900395977
TMOD2	244.4820993	0.135611515	0.188136102	0.720816015	0.471022721	0.900410307
JRKL	312.6552026	0.128343605	0.178107395	0.720596721	0.471157672	0.900571674
TM6SF2	3.477348557	0.749565208	1.040374331	0.720476454	0.471231692	0.900616555
NHEJ1	196.7692454	0.135646147	0.188295951	0.720388017	0.471286126	0.900623996
SLC26A2	999.5046546	0.135275849	0.18782544	0.720221121	0.471388862	0.900723731
SNORD149	2.637317049	1.008565649	1.400830315	0.719977029	0.471539139	0.900817741
CCDC127	423.724212	-0.109350047	0.151895246	-0.719904338	0.471583897	0.900817741
MKRN9P	70.54523136	-0.176576839	0.245306086	-0.719822497	0.471634291	0.900817741
KCP	12.00749358	0.495886246	0.688947879	0.719773238	0.471664625	0.900817741
TMEM116	130.9668879	-0.208040869	0.289053763	-0.719730707	0.471690816	0.900817741
ZNF471	29.41908016	0.307271758	0.427199362	0.719270169	0.471974472	0.901057648
RHBDL1	43.08865597	0.23761924	0.330390152	0.719207998	0.472012772	0.901057648
EML6	78.04335961	0.296222615	0.411984172	0.719014553	0.472131953	0.901057648
INO80	1519.673189	-0.103398661	0.14381145	-0.718987682	0.47214851	0.901057648
IFRD1	469.5000518	-0.094792598	0.131853071	-0.718925982	0.472186527	0.901057648
CCNYL1	219.3425062	0.136879124	0.190396196	0.718917328	0.472191859	0.901057648
LINC00526	18.69056324	0.472811428	0.657694842	0.718891799	0.47220759	0.901057648
C16orf58	1159.774288	-0.144597802	0.201145937	-0.718870113	0.472220953	0.901057648
RBM19	1006.375009	0.168350798	0.234250644	0.718678057	0.472339306	0.901126402
RPP25	200.2835251	-0.123624359	0.172023634	-0.718647528	0.472358122	0.901126402
FBP1	18.54232342	0.783741344	1.090799338	0.718501852	0.472447906	0.901201209
NUTM2F	4.143021507	0.700136708	0.974754774	0.718269586	0.472591079	0.901377826
HOXD8	398.5321301	-0.158594442	0.220826828	-0.718184669	0.472643429	0.901381198
PIEZO2	1343.638432	-0.076521145	0.106565256	-0.718068424	0.472715098	0.901383112
RAF1	1384.173438	0.063539617	0.088499946	0.717962213	0.472780586	0.901383112
TESK1	131.4596029	0.175304517	0.244178161	0.717936922	0.472796181	0.901383112
CNKSR3	928.6519224	0.076867723	0.107089559	0.717789145	0.472887307	0.9014604
SMU1	1160.111061	-0.093941345	0.130932403	-0.717479729	0.47307814	0.90172772
CHM	734.9046451	-0.11823944	0.164850245	-0.71725365	0.473217601	0.901812082
DPY19L1	2701.017974	0.081496733	0.113624852	0.717243905	0.473223613	0.901812082
GGN	22.654149	0.394187377	0.549780574	0.71699037	0.473380039	0.902009266
JPT1	860.2278126	0.091505636	0.127638567	0.716912125	0.47342832	0.902009266
LOC148696	5.189866004	1.082028424	1.50970189	0.716716612	0.473548973	0.902108382
DISP1	213.2954719	0.145118589	0.202491874	0.716663765	0.473581589	0.902108382
TMED2	5338.971808	-0.091636667	0.127905863	-0.716438361	0.473720715	0.90227695
ATR	1685.014241	0.153454507	0.214246854	0.716250923	0.473836424	0.902336405
GCDH	388.5385678	-0.109384897	0.152740727	-0.716147544	0.473900249	0.902336405

SLC39A11	738.7243298	0.215012019	0.300259237	0.716087939	0.47393705	0.902336405
FAM72A	6.542492813	0.573262134	0.800578666	0.716059718	0.473954475	0.902336405
ARMC1	1315.344519	0.107500821	0.150171516	0.715853605	0.474081749	0.902482295
DSN1	641.8697366	0.079033933	0.110436839	0.71564827	0.47420856	0.902592935
RGMB	363.6594684	-0.096654979	0.135069299	-0.715595472	0.47424117	0.902592935
NOG	9.033748873	0.601735894	0.841337849	0.715213151	0.474477344	0.902896634
TECTA	6.317384936	-0.556621562	0.778303245	-0.715173123	0.474502075	0.902896634
WDR19	495.9551432	-0.099182681	0.138733758	-0.714913823	0.474662295	0.902933674
SPARC	22003.20162	0.113386468	0.158605015	0.714898375	0.474671841	0.902933674
IFIT3	26.0375994	-0.343430198	0.480491449	-0.714747784	0.474764905	0.902933674
EEF1D	4184.600724	-0.070333215	0.098403278	-0.71474463	0.474766855	0.902933674
RYR3	31.26943532	-0.360366648	0.504277754	-0.714619363	0.474844277	0.902933674
GLMP	191.4583075	-0.134603289	0.188382349	-0.714521765	0.474904603	0.902933674
EPHX1	651.5956631	0.187120923	0.261899504	0.71447605	0.474932861	0.902933674
ABRAXAS2	297.892256	0.091540205	0.128133584	0.714412272	0.474972286	0.902933674
RMDN3	601.6226817	-0.083243303	0.116548898	-0.71423501	0.475081871	0.902933674
BAG4	2074.214839	0.064581979	0.09042212	0.714227662	0.475086415	0.902933674
PRDX3	1451.556047	0.086037101	0.120491739	0.714049791	0.475196391	0.902933674
MRI1	577.4663712	0.128409747	0.179861554	0.7139366	0.475266384	0.902933674
TUSC3	2949.33259	-0.058664143	0.082174635	-0.713895999	0.475291491	0.902933674
TBCK	304.3919503	0.10370126	0.145291722	0.713745136	0.475384791	0.902933674
SCN2A	280.27842	-0.108215669	0.151629229	-0.71368607	0.475421322	0.902933674
EPHA5-AS1	8.249246999	0.487567013	0.683223924	0.713626962	0.475457881	0.902933674
APOL2	297.262512	0.124657185	0.174694313	0.713573227	0.475491117	0.902933674
P2RX7	44.74541027	0.274192305	0.384293766	0.713496625	0.475538501	0.902933674
FAM219B	375.1849161	-0.130825595	0.183371826	-0.713444362	0.47557083	0.902933674
MRTO4	1164.478336	-0.115942048	0.162552879	-0.713257427	0.475686476	0.902933674
MPPE1	205.3537078	0.181283229	0.254215698	0.713107925	0.475778976	0.902933674
ASAP1	4241.575129	0.074061862	0.103860042	0.713092936	0.475788251	0.902933674
CNKSR1	17.01774254	0.357625863	0.501604981	0.71296314	0.475868566	0.902933674
LRRC73	14.41188282	-0.468251106	0.656876124	-0.712845373	0.475941446	0.902933674
SEC61A1	5124.328111	0.076569728	0.107419257	0.71281193	0.475962142	0.902933674
BUD23	444.4304968	-0.085419379	0.119839482	-0.712781613	0.475980905	0.902933674
LOC100505549	50.40333746	0.24353971	0.341711075	0.712706516	0.476027384	0.902933674
FAM155B	10.93902177	0.42314231	0.593795024	0.712606696	0.476089167	0.902933674
PGM2	490.7708482	0.121820303	0.1709532	0.712594458	0.476096742	0.902933674
YIF1B	448.5639819	-0.131003136	0.183847925	-0.712562491	0.476116529	0.902933674
GMEB1	368.932099	-0.172270889	0.24177097	-0.712537527	0.476131982	0.902933674
TMC3	31.39506309	0.351286086	0.493213873	0.712238858	0.476316878	0.902933674
SCARNA26A	2.667265813	-0.890472156	1.250321176	-0.712194733	0.476344198	0.902933674
RTKN2	140.2867577	-0.229389738	0.322138245	-0.71208477	0.476412285	0.902933674
UBL4A	695.8299343	-0.098547277	0.138404844	-0.71202188	0.476451227	0.902933674
SNORD36C	6.287822418	-0.553802616	0.777807983	-0.712004283	0.476462124	0.902933674
PCAT1	4.817688649	0.74678445	1.048875449	0.711985823	0.476473555	0.902933674
SDHAF4	99.50145607	0.245563156	0.344952289	0.711875711	0.476541744	0.902933674
GPC3	6.411790496	-0.664855357	0.934079971	-0.711775627	0.476603728	0.902933674
SNRPE	518.3481219	0.097953824	0.137627666	0.711730621	0.476631602	0.902933674
IPCEF1	41.15102311	0.312204798	0.438683489	0.711685772	0.47665938	0.902933674
VSIG2	2.959000208	0.914596788	1.285226181	0.711623215	0.476698127	0.902933674
LINC00863	68.95795253	-0.19498254	0.274017954	-0.711568485	0.476732028	0.902933674
LRRC19	11.32376709	0.486748279	0.684079253	0.711537847	0.476751007	0.902933674
BVES	469.731127	-0.080803006	0.113581224	-0.711411649	0.476829182	0.902985763
UBAC2	902.7977409	-0.10456845	0.147031872	-0.711195803	0.476962909	0.903094773
LACTB	277.387682	-0.113891969	0.160171117	-0.711064334	0.47704437	0.903094773
SUCLG1	712.496024	-0.087989877	0.123748825	-0.71103606	0.477061891	0.903094773
TMEM161A	580.0631404	0.078954694	0.111054432	0.710954911	0.477112177	0.903094773
EOMES	24.31209054	0.355921016	0.500735762	0.710796079	0.477210611	0.903094773
SLC6A17	10.22047519	-0.510323997	0.717977517	-0.710779913	0.47722063	0.903094773
HIP1	370.1694781	0.302897247	0.426294054	0.710535942	0.477371851	0.903094773
DNAJC21	438.7118979	-0.090803098	0.12779658	-0.71052839	0.477376532	0.903094773
SCARNA12	338.6303185	-0.220282795	0.310028647	-0.710524003	0.477379251	0.903094773
SH3BP5	44.90066401	0.260980919	0.367324545	0.710491369	0.477399481	0.903094773
LOC286437	29.56519779	0.274347706	0.386177204	0.710419214	0.477444212	0.903094773
UBQLN1	2078.813258	0.088214522	0.124207167	0.710220862	0.477567186	0.903170411
LINC01193	7.07935064	0.775850855	1.092453462	0.710191218	0.477585566	0.903170411
LGI4	2.73135863	1.072322076	1.510853579	0.709745862	0.477861746	0.903435181
LOC105369332	10.07186854	0.536321731	0.755655661	0.709743549	0.477863181	0.903435181
PM20D2	675.103057	-0.106372377	0.149897585	-0.709633693	0.47793132	0.903435181
BMS1P21	16.46511984	0.379057923	0.534176239	0.7096121	0.477944714	0.903435181
C11orf71	33.88202503	0.252628401	0.356041785	0.709547057	0.477985061	0.903435181
DIAPH3	592.1671581	-0.090977418	0.128232028	-0.709475	0.47802976	0.903435181
LOC100506124	15.62610697	0.409410306	0.577270469	0.709217478	0.478189529	0.903549332
PBX4	26.44500678	0.330453619	0.466065281	0.709028611	0.478306722	0.903549332
LINC01276	2.384187985	-0.952999907	1.344227071	-0.708957532	0.478350831	0.903549332
LINC02608	3.171937376	-0.860547678	1.214424447	-0.708605365	0.478569406	0.903549332
LINC02226	2.821463374	-0.918469099	1.296223913	-0.708572871	0.478589577	0.903549332
SMIM6	17.5979546	0.436712226	0.61636435	0.70852934	0.478616599	0.903549332
POC5	113.2105213	0.195848153	0.276431771	0.708486411	0.478643248	0.903549332
HSPA8	14231.49277	0.133008951	0.187772945	0.708349919	0.478727985	0.903549332
ZNF77	52.02637567	-0.199535755	0.281695879	-0.708337502	0.478735694	0.903549332
LINC00857	18.920762	0.38233565	0.539778937	0.708318951	0.478747211	0.903549332
LOC100419583	303.0294157	0.182328992	0.257431816	0.708261297	0.478783007	0.903549332
CXCL2	6.277467738	0.815595035	1.151662701	0.708189155	0.4788278	0.903549332
TOM1L2	559.4821556	-0.104240842	0.147211398	-0.708103064	0.478881257	0.903549332
GHDC	271.5899181	-0.143421356	0.202547928	-0.708086018	0.478891842	0.903549332
ZNF432	186.1118147	0.145596043	0.205664142	0.707931105	0.478988043	0.903549332
SATB2-AS1	1.858407512	1.105114358	1.561198842	0.707862656	0.479030552	0.903549332
ZNF624	167.9975217	0.133591684	0.188760302	0.707731882	0.479111775	0.903549332
CD72	4.99840294	0.759081858	1.072850527	0.707537386	0.479232588	0.903549332
ELOF1	297.2860107	-0.109379097	0.154594544	-0.707522363	0.479241921	0.903549332

MSH5	30.56892343	0.347470182	0.491108809	0.707521786	0.479242279	0.903549332
MIR34AHG	8.166040325	0.489460403	0.691946281	0.707367633	0.479338045	0.903549332
ABCA7	127.0175931	0.156775735	0.221713763	0.707108722	0.479498916	0.903549332
FBXW9	282.5498929	-0.109515973	0.154894183	-0.70703735	0.479543267	0.903549332
NOTCH2NLB	616.1220455	0.254121445	0.359430268	0.707011811	0.479559138	0.903549332
ZNRD1	261.5119249	-0.105158041	0.148737325	-0.707005056	0.479563336	0.903549332
KCTD6	53.23404042	-0.220789393	0.312431078	-0.706681915	0.479764171	0.903549332
FAM120B	571.1655919	-0.081012746	0.11463872	-0.706678736	0.479766146	0.903549332
MYL12B	1610.345661	-0.103080162	0.145873028	-0.70664305	0.479788329	0.903549332
HLA-E	1254.695553	-0.170889083	0.241838806	-0.706623911	0.479800226	0.903549332
TICRR	969.4315424	0.193583186	0.273955194	0.706623531	0.479800462	0.903549332
RIN2	665.5388256	-0.192761355	0.272800832	-0.706601052	0.479814435	0.903549332
PHKB	929.9049362	-0.108956202	0.154216221	-0.706515833	0.47986741	0.903549332
ZNF486	47.58317937	0.262931359	0.372221664	0.706383815	0.479949483	0.903549332
LOC105369146	2.145263448	1.144119408	1.619933736	0.706275438	0.480016865	0.903549332
SMPD1	231.8069196	0.217449984	0.307895847	0.706245264	0.480035626	0.903549332
OAF	377.2627319	0.183901224	0.260413731	0.706188659	0.480070822	0.903549332
HIF1A	4702.707875	-0.111089658	0.157315388	-0.706158876	0.480089341	0.903549332
TET1	552.5107746	0.143676227	0.203464862	0.706147615	0.480096344	0.903549332
SLC35E3	462.801574	-0.130727331	0.185136288	-0.706114032	0.480117226	0.903549332
TTYH3	4127.638872	0.069760944	0.098795826	0.706112258	0.480118329	0.903549332
THSD1	9.687252393	0.43844572	0.621049792	0.70597515	0.480203591	0.903614361
BLACAT1	21.4634315	-0.346843958	0.491668617	-0.70544254	0.480534878	0.904142279
CAPS	45.30624795	0.300391858	0.426014297	0.705121541	0.480734602	0.904234521
SF3B5	1538.887733	-0.10302213	0.146106493	-0.705116711	0.480737608	0.904234521
SSH2	668.9238611	0.078137153	0.110819908	0.705082278	0.480759035	0.904234521
GABRB1	11.44408191	0.438093478	0.6213761	0.705037542	0.480786873	0.904234521
ZNF205	102.427728	-0.172617543	0.244922952	-0.704783041	0.480945264	0.904436957
SF1	2085.111582	-0.088933456	0.126219691	-0.704592567	0.481063826	0.904457975
ANKRD30B	15.13319361	0.412172306	0.584994246	0.704574975	0.481074776	0.904457975
SYDE2	259.0269516	-0.108751199	0.154362015	-0.704520471	0.481108706	0.904457975
CYP1B1-AS1	10.68395665	0.555045905	0.788090578	0.704292021	0.481250935	0.904629921
NUP188	2737.15753	0.067063216	0.095232497	0.704205159	0.481305019	0.90463616
SNORD112	12.87961367	-0.569692112	0.80929158	-0.703939256	0.481470603	0.904736983
MMP24OS	123.8064515	-0.161355306	0.229246721	-0.703850008	0.481526187	0.904736983
PCDHGA11	45.61585248	0.366185302	0.520276556	0.703828181	0.481539782	0.904736983
PRRX2	851.7174763	0.491945315	0.699046659	0.703737452	0.481596292	0.904736983
VPS52	1086.62394	-0.071866883	0.10213675	-0.703633931	0.481660775	0.904736983
ADORA1	15.45083444	0.49469491	0.703077365	0.703613762	0.481673338	0.904736983
FURIN	976.6973448	0.159872036	0.227238175	0.703543919	0.481716847	0.904736983
GRK2	751.7765178	0.079274414	0.112701601	0.703400952	0.481805914	0.904736983
CCDC82	436.431166	0.122657756	0.174406326	0.703287312	0.481876716	0.904736983
CARD10	99.53847914	-0.140616473	0.199953216	-0.703246867	0.481901917	0.904736983
DLK1	440.4946216	-0.653143903	0.928787069	-0.703222434	0.481917141	0.904736983
SEPHS1	500.0426945	-0.087191611	0.124024277	-0.703020515	0.482042965	0.904877872
CCDC173	4.788481496	0.767568075	1.092005424	0.702897676	0.48211952	0.904926252
MIR199B	4.207504761	0.746331929	1.062058227	0.702722233	0.48222887	0.905036172
ASCC2	497.3879173	-0.091764036	0.130604082	-0.702612312	0.482297388	0.905069445
LINC02381	64.08116315	-0.192049214	0.273380769	-0.702497162	0.482369171	0.905108838
NETO1	126.3533738	0.275278134	0.391968363	0.702296818	0.482494077	0.905120717
OSBPL5	361.3519155	0.191616342	0.272844795	0.702290625	0.482497939	0.905120717
SIGMAR1	1025.194822	0.087997225	0.125316256	0.702201198	0.482553699	0.905120717
MIR324	4.584369428	0.773634077	1.101813585	0.702146068	0.482588075	0.905120717
LINC01279	309.3514006	-0.131810986	0.187764571	-0.702001369	0.48267831	0.905120717
DST-AS1	4.072981434	1.016033079	1.447464391	0.701939948	0.482716615	0.905120717
LINC00189	72.56216218	-0.186993738	0.266404418	-0.701916803	0.482731049	0.905120717
CPPED1	3.019329811	-0.840647455	1.198052122	-0.701678532	0.482879664	0.905204098
C2orf88	212.5514259	-0.174878955	0.249266341	-0.701574688	0.482944442	0.905204098
MRPL48	158.420234	-0.201212711	0.28686032	-0.701430967	0.483034102	0.905204098
PRORP	1016.431145	0.084479977	0.120472475	0.701238827	0.483153983	0.905204098
NXF3	2.038343114	1.027019256	1.464915662	0.701077395	0.483254717	0.905204098
PCDHB3	21.53558317	-0.378277713	0.539568395	-0.701074631	0.483256441	0.905204098
LAP3	549.255507	-0.080026385	0.114159914	-0.701002493	0.48330146	0.905204098
C22orf39	213.3652109	0.134523947	0.191905993	0.700988772	0.483310023	0.905204098
ARRB1	31.85218142	-0.305458867	0.435773896	-0.700957239	0.483329702	0.905204098
NFIL3	666.0613425	0.243145474	0.346883378	0.700942996	0.483338591	0.905204098
MIR544B	3.740224322	0.703144624	1.003227237	0.70088271	0.483376216	0.905204098
ARSA	344.7802419	0.092486696	0.131990103	0.700709325	0.483484435	0.905204098
CDH24	344.7748647	0.119919127	0.171143774	0.700692313	0.483495055	0.905204098
MIR574	5.383539371	0.598242761	0.853851411	0.700640362	0.483527483	0.905204098
CLDN24	2.320334059	1.202160085	1.715970755	0.700571429	0.483570514	0.905204098
JADE3	364.8450039	0.128286151	0.183135349	0.700499122	0.483615653	0.905204098
DNAAF5	1114.827826	-0.105409503	0.150485767	-0.700461614	0.48363907	0.905204098
PATL2	7.363503839	-0.658657689	0.940644119	-0.700219855	0.483790014	0.905286155
CAGE1	6.598404633	-0.69677128	0.99523272	-0.700108895	0.483859301	0.905286155
PLIN2	315.9523718	-0.121629414	0.173740396	-0.700064099	0.483887275	0.905286155
STK16	295.3885075	-0.166087121	0.237248818	-0.700054576	0.483893222	0.905286155
LAPTM5	2.929636121	-0.846223809	1.209039199	-0.699914287	0.483980835	0.905286155
ACO2	1081.542307	0.069166902	0.098832707	0.699838179	0.484028368	0.905286155
ZHX1	1698.004393	-0.082994756	0.118594108	-0.699821921	0.484038523	0.905286155
VPS4A	1134.895668	-0.092111699	0.131690757	-0.699454548	0.484268008	0.905620308
CTBP1	1618.040027	-0.102911904	0.147171412	-0.699265587	0.484386069	0.905644865
BCDIN3D	98.27845875	-0.17421491	0.249188042	-0.699130298	0.484470605	0.905644865
GTF2IP20	102.4011669	-0.231761042	0.331568471	-0.698983957	0.484562057	0.905644865
RUBCNL	68.58726983	0.237152343	0.339410563	0.698718217	0.484728147	0.905644865
LINC02035	26.91072178	-0.376004374	0.538168855	-0.698673605	0.484756033	0.905644865
BRD9	879.3477332	0.138298403	0.197972525	0.698573717	0.484818474	0.905644865
IGSF23	3.397671121	1.060714429	1.518435958	0.698557238	0.484828775	0.905644865
TMEM150A	125.1728622	-0.154226373	0.220786304	-0.698532337	0.484844342	0.905644865
LINC00665	117.7720208	-0.195515273	0.279918502	-0.698472134	0.484881979	0.905644865

C2CD5	801.379416	-0.09125458	0.130660575	-0.698409446	0.484921171	0.905644865
LOC101928673	22.75147522	0.360836338	0.516833997	0.6981668	0.485072886	0.905644865
MIR944	2.74879703	-0.884654309	1.267192067	-0.698121722	0.485101074	0.905644865
CCDC174	108.0124379	-0.156903339	0.224754646	-0.698109439	0.485108756	0.905644865
ORMDL3	358.3596247	0.123524348	0.176952945	0.698063252	0.485137638	0.905644865
LOC102724159	5.724110404	0.641391307	0.918966382	0.697948607	0.485209335	0.905644865
ZMIZ1	1416.597128	0.244317997	0.350184495	0.697683652	0.485375054	0.905644865
LOC105373064	16.49302147	0.360169665	0.516247255	0.69766892	0.485384269	0.905644865
ZNF843	9.083132844	0.53299043	0.764193775	0.697454555	0.48551837	0.905644865
LOC642366	21.83637483	0.35988424	0.516118785	0.697289559	0.485621601	0.905644865
GDNF	58.24425453	0.244426471	0.350546358	0.697272886	0.485632033	0.905644865
LOC107984036	24.93790479	0.359330741	0.515487986	0.69706909	0.485759557	0.905644865
COL7A1	515.828824	-0.146210538	0.209776185	-0.696983491	0.485813126	0.905644865
COL8A2	2946.954296	-0.20557883	0.294959593	-0.696972857	0.48581978	0.905644865
ZNF793	7.955974279	-0.476166414	0.683224765	-0.696939629	0.485840576	0.905644865
DCUN1D1	2034.771756	-0.060061783	0.086181192	-0.696924484	0.485850054	0.905644865
C19orf48	1219.215259	-0.139269568	0.199848887	-0.696874375	0.485881415	0.905644865
GEMIN5	1317.437002	0.113301207	0.162613555	0.696751308	0.485958443	0.905644865
INPP1	327.3015841	0.1200167	0.172299387	0.696559068	0.486078779	0.905644865
RLF	1269.865148	0.104606648	0.150177909	0.696551499	0.486083518	0.905644865
EXT2	2169.437563	-0.090114796	0.129379774	-0.696513785	0.486107127	0.905644865
UBE2D4	178.5571048	0.160643984	0.230646914	0.696493102	0.486120076	0.905644865
GFAP	6.465862329	-0.542994034	0.779666837	-0.696443671	0.486151022	0.905644865
AURKC	7.76580814	0.48762536	0.700277935	0.696331178	0.486221452	0.905644865
KLF3-AS1	44.00752566	0.293684585	0.421802918	0.696260203	0.486265892	0.905644865
CCDC136	257.7471374	0.18785965	0.269926155	0.695966827	0.486449605	0.905644865
TCAIM	195.2587387	-0.19155504	0.275255127	-0.695918154	0.486480087	0.905644865
TERF2	427.7826846	-0.113550356	0.163176271	-0.695875421	0.486506851	0.905644865
LAMTOR4	199.1890975	-0.119764143	0.172193017	-0.695522645	0.486727825	0.905644865
CBX1	1491.756998	-0.081048256	0.116536186	-0.69547716	0.48675632	0.905644865
PLOD2	2638.872948	-0.109201878	0.157040837	-0.695372494	0.486821893	0.905644865
LOC101927787	2.527232727	1.04473935	1.502432131	0.69536542	0.486826326	0.905644865
DNAJB14	971.2113463	0.096514553	0.138807575	0.695311856	0.486859886	0.905644865
GMEB2	312.9801941	-0.127283686	0.183068283	-0.695279838	0.486879947	0.905644865
EXOC1	532.6087148	-0.084755389	0.121913798	-0.695207517	0.486925262	0.905644865
SNORD114-26	7.90625094	0.499112436	0.718028752	0.695114833	0.48698334	0.905644865
LOC100287808	23.96463946	-0.323196981	0.465135513	-0.694844776	0.487152584	0.905644865
B3GNT8	11.99307377	0.406869816	0.585740747	0.694624402	0.487290715	0.905644865
CREB3L2-AS1	8.540659955	0.520337367	0.749195018	0.6945286	0.487350772	0.905644865
PPCDC	134.5978789	-0.165328565	0.238045188	-0.694525969	0.487352421	0.905644865
RHPN1-AS1	6.735655657	0.604700008	0.870878901	0.694356021	0.487458967	0.905644865
ROS1	4.752054461	0.652185092	0.939302402	0.694329207	0.487475779	0.905644865
MRPL21	225.8854768	-0.123347443	0.177679661	-0.694212509	0.487548949	0.905644865
HECTD3	502.8692772	-0.093193189	0.134255698	-0.694146997	0.487590028	0.905644865
ZNF417	436.6592904	0.114330384	0.164722173	0.694080111	0.487631971	0.905644865
INTS11	1193.803893	-0.091011719	0.131129529	-0.694059678	0.487644784	0.905644865
ALYREF	402.6346501	-0.118994176	0.171468885	-0.693969498	0.487701338	0.905644865
IMPACT	732.1616649	-0.126054668	0.181653906	-0.693927651	0.487727582	0.905644865
SOSTDC1	46.43644874	0.212656003	0.306488721	0.693846096	0.487778731	0.905644865
C16orf74	14.35820565	0.349385389	0.503570388	0.69381639	0.487797362	0.905644865
LINC01036	25.38188897	0.446455458	0.643511108	0.6937805	0.487819873	0.905644865
RFC3	863.2836974	-0.126489816	0.182348871	-0.693669312	0.487889615	0.905644865
ATP5MD	499.9161053	0.111912494	0.161336906	0.693657123	0.487897261	0.905644865
LOC102724562	4.087946737	-0.645769819	0.931031721	-0.693606678	0.487928905	0.905644865
ANAPC1	2170.735315	0.094796016	0.136693731	0.693492055	0.48800081	0.905644865
RANGRF	75.39780872	-0.170182655	0.245478103	-0.693270206	0.488139997	0.905644865
CRMP1	88.11830158	0.236115852	0.340630495	0.693172969	0.48820101	0.905644865
HRAT5	5.402678738	0.587221122	0.847211748	0.693122025	0.488232977	0.905644865
SRPK3	30.01851745	0.45879451	0.661954242	0.693090973	0.488252462	0.905644865
SLC35B2	1256.875159	0.100233634	0.144621223	0.693076936	0.488261271	0.905644865
NCAPH2	796.2783277	-0.122847641	0.177253452	-0.693062049	0.488270613	0.905644865
NHLRC3	424.6611378	0.154064206	0.222316029	0.692996389	0.488311818	0.905644865
RNF111	897.7340709	0.065320875	0.094263802	0.692958205	0.488335781	0.905644865
DAAM2	759.6882361	0.162229618	0.234130223	0.69290336	0.488370201	0.905644865
GUSBP11	247.8350295	0.239013347	0.345037006	0.692718007	0.488486536	0.905644865
IFITM4P	2.231270058	1.355112217	1.956254483	0.692707533	0.488493111	0.905644865
CSGALNACT2	380.1160853	-0.160138664	0.231179959	-0.692701323	0.488497009	0.905644865
LRRFIP1	1029.333157	-0.085082292	0.122872109	-0.692445927	0.488657332	0.905644865
RGS17	118.1666223	0.153134501	0.221156099	0.692427215	0.48866908	0.905644865
TMEM232	3.709495597	-0.658716233	0.951467453	-0.692316097	0.488738844	0.905644865
SUSD1	142.6645915	-0.147640689	0.213269938	-0.692271451	0.488766875	0.905644865
KCTD1	312.3265833	-0.119045125	0.171969511	-0.692245528	0.488783152	0.905644865
PCDHGA2	5.562944583	0.614004786	0.887122949	0.692130427	0.488855425	0.905644865
ZNF674	48.77860005	-0.207908958	0.300413693	-0.692075505	0.488889914	0.905644865
ANKRD12	485.9005121	-0.084192862	0.121652809	-0.69207495	0.488890262	0.905644865
NOSIP	685.8650597	-0.127878318	0.184792122	-0.692011737	0.488929958	0.905644865
FOXD3	17.84212701	-0.302823716	0.437662656	-0.69191125	0.488993065	0.905644865
TCF15	4.50611026	-0.705801195	1.020101198	-0.691893311	0.489004332	0.905644865
NPAS1	101.8444824	0.140119704	0.202521412	0.691875999	0.489015205	0.905644865
TEX41	2.815780329	0.875353619	1.265301713	0.691814142	0.489054055	0.905644865
SAMD5	267.270979	0.142706413	0.206286272	0.691788219	0.489070336	0.905644865
ZSWIM1	156.1220047	0.117688197	0.170149505	0.691675224	0.489141309	0.905644865
LINC01203	5.531414376	0.787630614	1.138859797	0.691595766	0.489191221	0.905644865
EGFL7	13.50708939	-0.347542625	0.502672537	-0.69138972	0.489320663	0.905644865
SH3TC2	3.038154955	0.838257505	1.212454472	0.691372356	0.489331572	0.905644865
MIR4442	3.334653403	-0.706851236	1.022389935	-0.691371474	0.489332126	0.905644865
SNORD113-1	17.58150353	0.47377572	0.685285374	0.691355365	0.489342246	0.905644865
SNIP1	301.970231	-0.11637244	0.16834333	-0.69128037	0.489389365	0.905644865
MIR381HG	25.69918458	0.5576604	0.806744867	0.691247535	0.489409996	0.905644865
CRACD	50.77101941	-0.308601834	0.446447612	-0.691238625	0.489415594	0.905644865

FLVCR1-DT	15.2950444	0.382277789	0.553100898	0.691153802	0.489468893	0.905644865
ZNF436-AS1	64.91074435	0.2354874	0.340800385	0.690983376	0.489575988	0.905644865
AK6	118.1311076	0.148415935	0.214819152	0.690887815	0.489636044	0.905644865
LINC01730	7.449015073	0.5467508	0.7916277	0.690666585	0.489775093	0.905644865
SLC16A10	130.5325866	0.123028935	0.178166153	0.690529222	0.48986144	0.905644865
LOC100287036	8.163888089	-0.852577189	1.234840909	-0.690434843	0.489920771	0.905644865
ECE1-AS1	6.697770407	-0.47755771	0.691752165	-0.690359545	0.489968111	0.905644865
CCDC9	214.8865348	-0.12209318	0.176871927	-0.690291456	0.49001092	0.905644865
DNAJC5G	3.231320487	0.703216438	1.018859025	0.69019994	0.490068461	0.905644865
SRA1	278.1729125	-0.104409732	0.151311755	-0.690030541	0.490174982	0.905644865
COMMD3	185.4441545	-0.130752845	0.189513374	-0.689939936	0.49023196	0.905644865
GASAL1	21.28476103	-0.359074832	0.520481874	-0.689889217	0.490263858	0.905644865
WDR83OS	560.9602979	-0.08122888	0.117749295	-0.689845998	0.490291039	0.905644865
SLC50A1	400.2109991	0.148052003	0.214637763	0.689776119	0.490334989	0.905644865
HFM1	37.46287052	-0.267262798	0.387473998	-0.689756729	0.490347185	0.905644865
LHX4-AS1	47.6958589	0.358542363	0.519824073	0.689737897	0.490359029	0.905644865
SPOCK2	4.926426186	-0.566237568	0.821019111	-0.689676478	0.490397661	0.905644865
MAPK9	901.7506235	0.072268398	0.104791999	0.689636606	0.490422741	0.905644865
TBC1D30	54.46836038	0.211245012	0.306345049	0.689565616	0.490467396	0.905644865
SUV39H2	380.3050092	0.135270453	0.196176242	0.689535346	0.490486438	0.905644865
SRSF3	4183.257757	-0.113988497	0.165386278	-0.689225842	0.490681157	0.905644865
ARL5C	4.845113932	0.93401542	1.355248513	0.689183874	0.490707564	0.905644865
WBP1L	417.2814395	0.1321447	0.191742333	0.689178534	0.490710924	0.905644865
RRM2	3019.174547	-0.134751374	0.195538979	-0.68912794	0.490742759	0.905644865
DPY19L1P2	8.465631448	-0.519150157	0.753360197	-0.689112804	0.490752283	0.905644865
SMG1P2	147.9587422	0.323281614	0.469168599	0.689052113	0.490790474	0.905644865
IGFBP3	3515.532895	-0.664635678	0.964589522	-0.689034727	0.490801414	0.905644865
LNPEP	2680.134396	0.083044619	0.12053188	0.688984678	0.490832909	0.905644865
SYNRG	1114.314398	0.062344921	0.090494927	0.688932773	0.490865574	0.905644865
LOC100505502	44.44307574	0.533401538	0.77426855	0.688910247	0.49087975	0.905644865
PIGBOS1	108.0749541	-0.146192301	0.212212078	-0.688897175	0.490887977	0.905644865
GTPBP4	954.5064507	0.092601924	0.134461554	0.688686996	0.491020261	0.90579514
POMT2	708.1147388	0.073641756	0.106998921	0.688247651	0.491296841	0.906136883
TAL2	2.851578945	0.789541268	1.147352245	0.688142	0.491363364	0.906136883
DGCR11	28.64855576	0.387439352	0.563110205	0.688034685	0.491430939	0.906136883
SOWAHA	5.539513247	-0.678014089	0.985449869	-0.688024942	0.491437075	0.906136883
XPO4	1837.566233	0.086505376	0.125745547	0.687939878	0.491490643	0.906136883
MIR590	17.28186759	0.444064787	0.645568811	0.687865924	0.491537217	0.906136883
IFITM5	2.998351687	0.783609462	1.139643558	0.687591709	0.491709931	0.906136883
ARFGAP2	721.236464	-0.105656533	0.153684793	-0.687488529	0.491774927	0.906136883
MIR23B	2.843835649	0.812003383	1.181137325	0.687475847	0.491782916	0.906136883
CZ1P-ASNS	18.64587601	-0.394357804	0.573647112	-0.687457142	0.4917947	0.906136883
TNIP3	4.537680381	-0.710584284	1.033782095	-0.687363698	0.491853568	0.906136883
KBTBD8	48.68036269	0.257460766	0.374637588	0.687226201	0.491940196	0.906136883
RETREG1	37.58462619	-0.331090232	0.481874583	-0.687087975	0.492027291	0.906136883
VDAC2	1561.855543	0.100375584	0.14609987	0.68703404	0.492061278	0.906136883
SLC39A6	2293.488755	0.089364997	0.130140766	0.686679506	0.492284714	0.906136883
NOL12	173.1852904	-0.216708869	0.315625089	-0.686602163	0.492333465	0.906136883
CDRT15P1	18.13817194	0.352726879	0.513792884	0.686515694	0.492387971	0.906136883
PPP2R5B	72.69366211	-0.178763197	0.260396718	-0.686503267	0.492395805	0.906136883
ZNF622	265.751034	-0.101972641	0.148569977	-0.68636102	0.492485479	0.906136883
COX16	105.3613826	0.173478774	0.252770388	0.686309718	0.492517822	0.906136883
HMGCL	514.2414278	-0.227989878	0.332213565	-0.686275043	0.492539684	0.906136883
LOC100133286	27.20787543	-0.2419499	0.35259245	-0.686202724	0.49258528	0.906136883
MGC15885	4.607946171	0.757330137	1.103827168	0.686094852	0.492653297	0.906136883
FAM124A	10.40114118	-0.444336564	0.647650323	-0.686074797	0.492665943	0.906136883
KCMF1	835.7635343	-0.083774869	0.122111261	-0.68605359	0.492679316	0.906136883
APC	1001.81477	0.071509885	0.104235943	0.686038646	0.492688739	0.906136883
C18orf32	64.98814865	0.214431186	0.312597587	0.685965582	0.492734813	0.906136883
TMEM131	2336.430072	0.061929052	0.090283117	0.685942783	0.49274919	0.906136883
TET2-AS1	2.892401404	0.812445163	1.184502674	0.685895593	0.49277895	0.906136883
KPNA2	3266.254816	0.068368696	0.099718259	0.685618631	0.49295363	0.906136883
MRPS2	308.1564353	-0.137213396	0.20014639	-0.685565178	0.492987346	0.906136883
CCDC144A	2.71553739	0.938046675	1.368291631	0.685560486	0.492990306	0.906136883
ANKMY2	362.3749486	0.087333372	0.127391555	0.685550718	0.492996468	0.906136883
PSCA	45.28612542	0.282619187	0.412277452	0.685507261	0.49302388	0.906136883
MAP2K7	751.9279428	-0.089214175	0.130175275	-0.685338865	0.493130112	0.906136883
GUSBP1	148.3254612	-0.171756263	0.250617272	-0.685332906	0.493133872	0.906136883
CUL2	706.4817184	-0.078306935	0.114272245	-0.685266441	0.493175804	0.906136883
LINC01450	6.525769445	0.853618843	1.245679661	0.685263531	0.49317764	0.906136883
ZBTB3	100.4810384	-0.164673119	0.240339265	-0.685169434	0.493237009	0.906136883
POLM	261.3130114	0.140170212	0.20457862	0.685165498	0.493239493	0.906136883
ARRDC1	128.2042919	-0.14053215	0.205220023	-0.68478771	0.493477892	0.90633317
POLR2J	45.14286223	0.258564373	0.377587215	0.684780529	0.493482424	0.90633317
SLC22A14	2.722926482	-0.780820868	1.140438928	-0.684666972	0.493554095	0.90633317
KCNN1	2.177065509	0.990556772	1.446906309	0.684603257	0.493594311	0.90633317
PUF60	2219.679868	-0.102124484	0.149175423	-0.68459323	0.493600641	0.90633317
TPP2	1312.741098	-0.143011307	0.20893351	-0.684482385	0.493670609	0.906368251
LOC101929054	30.29272678	-0.273519828	0.399703856	-0.684306204	0.49378183	0.906479057
CAD	8278.07706	0.081271977	0.118803903	0.684085076	0.493921445	0.906585202
ATP11A-AS1	6.760440986	0.972062668	1.421178112	0.683983703	0.493985457	0.906585202
WDR86	3.701650321	0.906949667	1.326119984	0.683912224	0.494030595	0.906585202
RASSF1-AS1	11.47390428	0.455861707	0.666588426	0.68387282	0.494055479	0.906585202
ELOVL5	5729.69753	0.08904739	0.130222075	0.683811787	0.494094022	0.906585202
AK2	2717.630743	-0.091954085	0.134513553	-0.683604612	0.494224872	0.906731928
ZNF106	3463.919355	-0.053867542	0.078850277	-0.683162366	0.49450425	0.906833834
TRNT1	523.809516	0.155895939	0.228200693	0.683152786	0.494510303	0.906833834
ERLIN2	4203.366172	-0.10545223	0.154364733	-0.6831368	0.494520403	0.906833834
VKORC1	734.4787153	-0.128241634	0.187740987	-0.683077446	0.494557906	0.906833834
SLC39A1	2150.713957	0.08766525	0.128367927	0.682921758	0.494656285	0.906833834

MAGOH2P	4.483761372	0.720242698	1.054728973	0.682869928	0.494689038	0.906833834
LRRC70	13.77266439	0.556143936	0.814427155	0.682865168	0.494692046	0.906833834
FLAD1	435.0243655	0.106864793	0.156496697	0.682856541	0.494697498	0.906833834
NFKBIB	255.6822559	-0.121704916	0.178246013	-0.682791797	0.494738414	0.906833834
ZNF563	22.92762348	0.331712302	0.485909319	0.682662977	0.494819829	0.906851262
PCYT1B	147.4535628	-0.154571088	0.226439393	-0.682615715	0.494849701	0.906851262
ZNF160	355.3129886	0.092053512	0.13490062	0.6823802	0.494998573	0.906933532
GGA2	1181.003886	0.08297092	0.121605679	0.682294778	0.495052575	0.906933532
NRF1	278.946225	-0.111964138	0.164114228	-0.682232975	0.495091647	0.906933532
PCDHA13	15.205315	0.34636216	0.507762908	0.682133638	0.495154452	0.906933532
SNORD111	9.766450801	0.54719653	0.802347227	0.681994667	0.495242323	0.906933532
OVGP1	14.84304011	0.408349792	0.598837272	0.681904435	0.495299381	0.906933532
LOC101927635	2.34663086	-1.242807939	1.822636432	-0.68187375	0.495318785	0.906933532
PARD6B	67.74089491	0.228510436	0.335156652	0.681801883	0.495364233	0.906933532
SMC2-AS1	5.222860709	0.67895519	0.996123941	0.6815971	0.495493749	0.906933532
DESI1	486.9151996	0.095318769	0.139863478	0.681512933	0.495546986	0.906933532
MTF1	480.5380854	0.101263944	0.148610465	0.681405207	0.495615129	0.906933532
NADSYN1	213.5233893	0.119020352	0.174675164	0.68138108	0.495630392	0.906933532
LOC102723769	19.82917593	0.497187752	0.729697187	0.681361749	0.49564262	0.906933532
MALRD1	37.06427851	-0.307147135	0.450797935	-0.681341044	0.495655718	0.906933532
AURKB	657.4662666	0.090262135	0.132498394	0.681231914	0.495724757	0.906933532
IRF1-AS1	9.324573248	-0.442139131	0.649092589	-0.681164965	0.495767114	0.906933532
PLEKHH2	179.8405386	0.188401992	0.276618264	0.68109021	0.495814411	0.906933532
RABAC1	898.3504929	-0.187486865	0.275299071	-0.681029777	0.495852649	0.906933532
KARS1	2059.15917	-0.087168124	0.127997238	-0.681015658	0.495861583	0.906933532
ALX1	53.58725016	0.187537636	0.275438831	0.680868545	0.495954673	0.907001025
C2orf92	12.38445776	0.374873548	0.550678136	0.680748924	0.496030374	0.907001025
LOC105372631	4.580790136	0.610739857	0.897501258	0.680489138	0.496194797	0.907001025
ZSCAN22	257.1610563	0.121008466	0.177845037	0.680415199	0.4962416	0.907001025
FLJ13224	27.15822391	0.293789448	0.431789794	0.680399241	0.496251701	0.907001025
NDUFA3	70.44083939	0.218275153	0.320877001	0.680245552	0.496348994	0.907001025
ZNF266	485.7596566	0.082867655	0.121823259	0.68022852	0.496359777	0.907001025
RAB11FIP5	1467.445614	0.166125862	0.244266671	0.680100406	0.496440888	0.907001025
HMGCS1	3009.120293	0.178191251	0.262009314	0.680095101	0.496444246	0.907001025
NUP107	1509.476635	-0.095006556	0.139697112	-0.680089624	0.496447714	0.907001025
ZNF638	2121.299736	-0.093971804	0.138200621	-0.679966577	0.496525624	0.907001025
SNORD13D	3.253454221	0.813271679	1.196112757	0.67992894	0.496549456	0.907001025
IKZF5	294.16378	-0.087237641	0.128307246	-0.679912041	0.496560157	0.907001025
RPP40	195.195059	-0.190577417	0.280371958	-0.679730664	0.496675016	0.907117842
C7orf26	258.4914967	0.109964923	0.161804399	0.679616402	0.496747382	0.907157034
ZBTB38	461.5807699	-0.105718955	0.155583521	-0.6794997	0.496821298	0.907171563
ZNF557	490.6281231	0.080623991	0.118661874	0.679443095	0.496857152	0.907171563
KCNN4	179.1487003	-0.247361064	0.36418767	-0.679213177	0.497002799	0.907268617
CACNB1	165.9292151	0.243135516	0.357976548	0.679193979	0.497014962	0.907268617
UMAD1	211.7065277	0.142673691	0.210086721	0.67911808	0.497063047	0.907268617
LOC100289580	40.23508383	0.477467446	0.703243243	0.678950635	0.497169141	0.907369325
TAF5L	617.595574	0.098192568	0.144650102	0.678828196	0.497246726	0.907417989
SLC35C2	843.1897644	0.067568577	0.09955423	0.678711258	0.497320831	0.907460292
AKR1C1	707.5729733	0.554482021	0.817180558	0.678530608	0.497435323	0.907576274
DONSON	499.5796305	0.079413505	0.117092357	0.678212546	0.497636939	0.907807216
GSEC	47.94125473	-0.2065236	0.304583375	-0.678052765	0.497738238	0.907807216
TNFRSF17	4.885302347	-0.666041888	0.982345541	-0.678011819	0.497764199	0.907807216
FAM86EP	89.89216576	-0.18160013	0.26784305	-0.678009494	0.497765674	0.907807216
JAM2	64.75531609	0.203354433	0.299978531	0.677896622	0.497837242	0.907843727
HIGD1A	457.5709281	-0.126723028	0.187009685	-0.677628156	0.498007489	0.907843727
TEX14	5.031583274	-0.664283752	0.980364386	-0.67758862	0.498032563	0.907843727
CEP170B	1152.298113	0.142080295	0.209702904	0.677531367	0.498068875	0.907843727
KATNAL1	538.424403	0.100016362	0.147639308	0.677437219	0.498128591	0.907843727
PROB1	28.43692309	0.423693241	0.625526802	0.677338269	0.498191356	0.907843727
MTMR10	412.9416224	0.096834226	0.142975159	0.67728007	0.498228273	0.907843727
ZNF506	64.46564197	0.207183057	0.305943058	0.677194829	0.498282348	0.907843727
NRBP2	722.6257674	-0.143411759	0.211792006	-0.677134901	0.498320367	0.907843727
IL1R2	16.09591848	0.426484579	0.629913177	0.677052957	0.498372355	0.907843727
IL12RB2	233.8517516	0.52971016	0.782381536	0.677048391	0.498375252	0.907843727
MIR1304	28.57800426	0.346768784	0.512316819	0.676863946	0.498492281	0.907843727
UBFD1	1285.371215	-0.077241757	0.114151658	-0.676659096	0.498622275	0.907843727
SHOX2	548.4993812	-0.090751911	0.13411793	-0.676657554	0.498623254	0.907843727
TNS2	80.73137996	0.181822305	0.268715107	0.676635961	0.498636957	0.907843727
KNTC1	3816.154574	0.108455775	0.160292787	0.67661045	0.498653147	0.907843727
LOC102724596	8.876789256	0.512545406	0.75761331	0.676526401	0.49870649	0.907843727
MIR152	3.807997987	-0.622638585	0.920505228	-0.676409613	0.498780615	0.907843727
TPTE2P3	3.114643258	-0.807995524	1.194598231	-0.676374285	0.49880304	0.907843727
HIKESHI	371.246905	-0.103309384	0.152825061	-0.675997659	0.499042131	0.907843727
CABP7	7.541410152	0.50613953	0.748741484	0.675987028	0.499048881	0.907843727
TMEM9B	319.595444	-0.107073268	0.158406973	-0.67593785	0.499080106	0.907843727
DUSP5	32.01306288	-0.305473513	0.451998936	-0.675827946	0.499149889	0.907843727
VILL	30.87220354	0.25479427	0.377014509	0.675820861	0.499154389	0.907843727
NSUN4	435.1319021	0.096989069	0.143532594	0.675728535	0.499213015	0.907843727
SNORD113-5	6.554861431	-0.539460611	0.798381898	-0.675692437	0.499235938	0.907843727
BMP1	1244.531995	-0.086748037	0.128384013	-0.67569189	0.499236286	0.907843727
KIF20B	755.0489471	0.086296232	0.127731309	0.675607512	0.499289871	0.907843727
DKK1	223.9673093	-0.223074679	0.330189272	-0.675596385	0.499296937	0.907843727
WDR93	3.490324611	0.763619963	1.130335268	0.675569439	0.49931405	0.907843727
C10orf90	2.415380454	1.297955529	1.921531733	0.675479622	0.499371094	0.907854815
USP42	390.6903837	0.077988385	0.115510102	0.675165059	0.499570903	0.907992217
MRPL28	328.0559882	-0.098048992	0.145224697	-0.675153703	0.499578117	0.907992217
TOMM6	749.3871943	0.105586271	0.156397819	0.675113449	0.49960369	0.907992217
IL21R	45.86291308	-0.34933095	0.517564522	-0.674951499	0.499706579	0.907992217
TIRAP	69.6631188	0.183844711	0.272401614	0.674903165	0.49973729	0.907992217
MKLN1-AS	53.34427845	-0.234035838	0.346781722	-0.674879393	0.499752394	0.907992217

TNFSF12	13.28616595	0.359164695	0.532289741	0.674754118	0.499831995	0.908044261
CCHCR1	482.270985	0.098525158	0.146066675	0.674521808	0.499979626	0.908135937
GNB1	5448.543852	-0.100052561	0.148341869	-0.674472837	0.500010749	0.908135937
IL17RC	132.3547979	-0.138373517	0.205169798	-0.674434141	0.500035343	0.908135937
GTPBP1	810.6015631	-0.107855549	0.159956828	-0.674279123	0.500133874	0.908139047
EFCAB14-AS1	7.054979369	0.516397033	0.765859666	0.674271092	0.500138979	0.908139047
PHACTR2-AS1	10.23467383	0.656452393	0.973733964	0.674159901	0.50020966	0.908174848
CEP112	45.11183727	-0.215375331	0.319668573	-0.673745713	0.500472994	0.908533967
EIF5	3363.686589	-0.189016904	0.280570213	-0.673688421	0.500509425	0.908533967
RWDD3	111.3670629	-0.14840086	0.220433636	-0.673222394	0.500805817	0.90897939
CCNYL2	78.87410714	0.198408217	0.294750179	0.673140276	0.500858053	0.908981617
SLC35B3	456.1152713	0.098834368	0.146889696	0.672847522	0.501044301	0.908992073
LIX1L-AS1	3.279121098	0.709414038	1.054606012	0.67268158	0.501149889	0.908992073
MIR1307	5.477380916	0.863647236	1.284003754	0.672620491	0.501188762	0.908992073
ZBTB20-AS1	9.881551664	-0.509274028	0.757209085	-0.672567244	0.501222646	0.908992073
CBFA2T2	469.0433029	0.133424044	0.198397061	0.672510182	0.50125896	0.908992073
NEU1	664.364093	-0.120803782	0.17964277	-0.672466705	0.501286629	0.908992073
MPP1	122.0209671	-0.140549007	0.209032052	-0.672380174	0.501341701	0.908992073
LOC389247	12.30312574	0.391583176	0.58239542	0.67236651	0.501350398	0.908992073
PCDHGA7	11.6801476	0.418771469	0.62287183	0.672323662	0.501377669	0.908992073
CACNA1C-IT3	5.519162663	0.777586115	1.156702897	0.672243596	0.501428631	0.908992073
ALX4	54.47567472	0.225045791	0.334794633	0.672190559	0.50146239	0.908992073
ZNF57	47.26384916	0.189738116	0.282277276	0.672169289	0.50147593	0.908992073
LRRC37A	13.87721661	0.4058285	0.604295599	0.671572822	0.501855685	0.9094114
ALG1L	6.749262962	-0.601104726	0.895115294	-0.671538885	0.501877297	0.9094114
PLOD3	1461.430598	0.064520962	0.096080328	0.671531444	0.501882035	0.9094114
LINC01271	2.670007634	1.012307153	1.507564052	0.671485335	0.501911398	0.9094114
RPL13AP5	77.61233435	-0.234936722	0.349962218	-0.671320246	0.50201654	0.909441384
ESRRA	433.1297972	0.103903853	0.154781776	0.671292547	0.502034181	0.909441384
TMEM183A	903.256019	-0.076962402	0.114672146	-0.671151663	0.502123918	0.909441384
SLC10A3	259.8141293	0.106285502	0.1583906	0.671034155	0.502198771	0.909441384
UBE2Q2L	59.05332992	0.276012135	0.411365747	0.670965284	0.502242645	0.909441384
UFM1	796.9718565	-0.078525038	0.117045436	-0.67089364	0.502288288	0.909441384
VEPH1	178.0236891	-0.128956359	0.192217398	-0.670888067	0.502291839	0.909441384
CYB5D1	366.6758627	-0.136966951	0.204209583	-0.670717552	0.502400479	0.909441384
HTATSF1	565.4832223	-0.082849612	0.123546805	-0.670592914	0.502479898	0.909441384
ZNF221	95.09819481	0.160241704	0.238960089	0.670579361	0.502488534	0.909441384
BRWD1-AS2	8.731088924	0.46081883	0.687252762	0.670523067	0.502524407	0.909441384
HEATR3	397.0401313	0.148171212	0.220986798	0.670498025	0.502540365	0.909441384
MPP6	1465.747329	0.098774975	0.147349173	0.67034631	0.502637052	0.909523991
C2CD2L	99.82571926	0.134201271	0.200282619	0.670059498	0.502819862	0.909661455
IL15	12.39467788	0.436241194	0.651054994	0.670052758	0.502824159	0.909661455
LRRC4	48.52470701	0.516035443	0.770284386	0.669928474	0.502903388	0.909661455
ZNF562	598.6048081	-0.0959027	0.143165067	-0.669875002	0.502937477	0.909661455
KANSL1L	130.8676127	0.163658742	0.244335843	0.669810616	0.502978526	0.909661455
SSX2IP	451.9074233	0.125574207	0.187495086	0.669746658	0.503019303	0.909661455
RBM48	169.6931834	-0.132580168	0.197989653	-0.669631798	0.503092539	0.909665811
QRSL1	376.3684941	0.097345954	0.145383005	0.669582761	0.503123807	0.909665811
FUS	2075.626476	-0.18615908	0.278121295	-0.669344937	0.503275468	0.909745691
LENEP	8.245689785	0.675661122	1.009465495	0.669325624	0.503287784	0.909745691
SNF8	580.5397804	-0.08574888	0.128123237	-0.669268758	0.503324052	0.909745691
C18orf21	108.7836511	-0.149045986	0.22272487	-0.669193276	0.503372195	0.909745691
TMEM39A	596.1419627	0.076696117	0.114669715	0.6688437	0.503595187	0.909953743
FXR1	5037.094707	-0.079727053	0.119205448	-0.668820547	0.503609958	0.909953743
GATAD2A	1655.109973	-0.090524323	0.13536421	-0.668746363	0.503657287	0.909953743
TSPAN5	324.6052572	-0.15810017	0.23643175	-0.668692635	0.503691567	0.909953743
PHAX	359.6142905	-0.089725199	0.134217378	-0.66850657	0.503810289	0.909972908
SBF2	1581.638051	-0.118311988	0.177000885	-0.66842597	0.503861722	0.909972908
AGRN	757.8098737	-0.151804927	0.227177988	-0.668220227	0.503993026	0.909972908
LMOD3	5.087975599	-0.606683481	0.907932831	-0.668203044	0.504003992	0.909972908
NUBP1	123.8044068	0.151258851	0.226382895	0.668154945	0.504034692	0.909972908
FKBP10	7742.754827	-0.101002588	0.151170613	-0.668136397	0.504046531	0.909972908
JMY	624.9984069	-0.19798711	0.296378949	-0.668020151	0.50412073	0.909972908
RTN2	58.25387016	0.272422117	0.407931931	0.667812684	0.504253169	0.909972908
RRM2B	992.3202377	0.088878827	0.133098004	0.667769799	0.504280548	0.909972908
PPP1R27	9.827150795	0.568638548	0.85176609	0.667599421	0.504389327	0.909972908
AKR1E2	2.73880448	0.789273628	1.182359632	0.667541082	0.504426578	0.909972908
POLR2F	106.6060263	-0.128014488	0.191771641	-0.66753607	0.504429778	0.909972908
LATS2	533.8989923	0.154003488	0.230768371	0.667350935	0.504547999	0.909972908
SLC48A1	87.31523124	-0.188334558	0.282213458	-0.667347899	0.504549938	0.909972908
FLRT3	2.877989266	-1.015784963	1.522156448	-0.667332826	0.504559563	0.909972908
ZDHHC12	217.3364326	-0.115631972	0.173289877	-0.667274826	0.504596604	0.909972908
ZNF891	161.0025603	0.173443961	0.260007768	0.667072227	0.504725999	0.909972908
C8orf82	385.3966997	-0.133066775	0.199496112	-0.667014381	0.504762948	0.909972908
LRRC7	7.7197042	-0.571175814	0.856402392	-0.666947943	0.504805386	0.909972908
ULK4P3	56.55124683	0.245778405	0.368618516	0.666755451	0.504928353	0.909972908
NAF1	273.8598864	0.125968387	0.188935769	0.666725988	0.504947176	0.909972908
SEMA3F-AS1	29.48737502	-0.332642481	0.498955895	-0.666677122	0.504978395	0.909972908
DLG5-AS1	11.92393541	0.414740389	0.622141773	0.666633246	0.505006428	0.909972908
DIAPH1-AS1	18.49372208	0.586125002	0.879273172	0.666601712	0.505026575	0.909972908
EXOSC7	306.5071938	-0.087571616	0.131385312	-0.66652516	0.505075488	0.909972908
TMEM177	216.0919295	0.161798806	0.242800384	0.666386121	0.505164331	0.909972908
STMN2	5.437291257	-0.880479618	1.321390428	-0.666328134	0.505201387	0.909972908
LINC00858	11.44255941	-0.428703666	0.643425013	-0.666283805	0.505229715	0.909972908
QDPR	304.810148	-0.110253642	0.165502253	-0.666176077	0.505298562	0.909972908
HDAC8	205.0390511	-0.100738387	0.151219013	-0.666175404	0.505298992	0.909972908
PDPK1	1165.960765	0.061387727	0.09215882	0.666107999	0.505342072	0.909972908
ERC2	6.386987349	0.605863725	0.909596806	0.666079434	0.505360329	0.909972908
CALHM5	208.2054129	-0.099977112	0.150107395	-0.66603722	0.505387311	0.909972908
SPATS2L	1120.406821	-0.082815861	0.124418662	-0.665622501	0.50565242	0.910218628

IGSF22	4.767529544	0.741213824	1.113627943	0.665584793	0.505676529	0.910218628
BZW2	2308.380804	-0.094180945	0.141501209	-0.665584032	0.505677015	0.910218628
FBH1	831.4754206	0.075263654	0.113127354	0.665300219	0.50585849	0.910453318
CASTOR1	37.75318059	0.254314942	0.382322751	0.66518391	0.505932871	0.910495229
C9orf92	3.568102128	0.833954889	1.254247068	0.664904795	0.506111389	0.910537412
ZNF235	35.12226021	0.363722785	0.547158598	0.664748368	0.506211452	0.910537412
SDHAF3	95.97033638	-0.132691708	0.1996192	-0.664724177	0.506226927	0.910537412
GS1-124K5.11	148.9633839	-0.182631	0.274768078	-0.664673283	0.506259486	0.910537412
ALOXE3	1.936172476	1.019041994	1.533452302	0.664541044	0.506344089	0.910537412
LTB4R	464.1321789	0.205399333	0.309114499	0.66447654	0.506385359	0.910537412
LOC102606465	120.0374843	-0.140261884	0.211102356	-0.664425954	0.506417726	0.910537412
METTL5	228.0417413	0.108349519	0.16307725	0.664406097	0.506430432	0.910537412
IKBKE	171.5398474	-0.173327661	0.260881211	-0.664393043	0.506438784	0.910537412
ZNF777	181.3657022	-0.113450672	0.17083058	-0.664112201	0.506618502	0.910537412
LOC101927516	5.547958339	0.798363314	1.202234284	0.664066334	0.506647856	0.910537412
C19orf54	264.3725469	-0.154678446	0.232945217	-0.664012114	0.506682558	0.910537412
N6AMT1	217.4695462	0.158102828	0.238107489	0.66399771	0.506691777	0.910537412
ZNF169	85.22935845	0.203586779	0.306609124	0.663994524	0.506693816	0.910537412
MZT2B	341.856217	-0.11772593	0.177311624	-0.663949308	0.506722756	0.910537412
FANCE	287.5846384	-0.097762512	0.147297432	-0.663708194	0.506877094	0.910548081
SGCZ	92.72305731	-0.137358945	0.206996193	-0.663581985	0.506957891	0.910548081
EDEM3	2457.987313	0.081694644	0.123112596	0.663576649	0.506961307	0.910548081
SETD1B	1246.479095	-0.104096203	0.156887705	-0.663507719	0.507005437	0.910548081
ELOA	953.5796763	-0.096051035	0.144764419	-0.663498918	0.507011072	0.910548081
GPC2	49.32780774	0.20373706	0.307102923	0.663416217	0.507064022	0.910548081
ACP5	9.351911831	-0.432322822	0.651710049	-0.663366818	0.507095652	0.910548081
TRAPPC2L	165.5859336	-0.115433277	0.174028364	-0.663301511	0.507137469	0.910548081
SUSD4	4.017358565	0.864086711	1.303065878	0.663118209	0.50725485	0.910573743
PEPD	505.8057191	-0.099907121	0.150667967	-0.663094636	0.507269946	0.910573743
QKI	5725.898198	-0.066104744	0.09969951	-0.663039811	0.507305057	0.910573743
YAP1	2429.299595	-0.113971683	0.171933638	-0.662881819	0.507406246	0.910576131
SUZ12P1	231.466838	0.171575758	0.258834529	0.662878168	0.507408585	0.910576131
TMOD3	2754.455881	-0.10312134	0.155644065	-0.662545918	0.507621417	0.910799399
C16orf96	4.101236446	-0.783064437	1.182154617	-0.662404415	0.507712075	0.910799399
MSMO1	2300.991732	0.210589522	0.317934201	0.662368255	0.507735243	0.910799399
PRKCQ-AS1	66.07593582	-0.166756513	0.251805593	-0.662243085	0.507815446	0.910799399
IDH3B	824.9673385	-0.095460571	0.14418652	-0.662063077	0.507930798	0.910799399
ZNF224	172.9885294	0.161494238	0.243976474	0.661925455	0.508018997	0.910799399
SACS-AS1	5.723006737	0.575143914	0.869170834	0.661715616	0.508153495	0.910799399
RLIM	2844.699006	0.067126094	0.101457356	0.661618797	0.508215558	0.910799399
IPP	287.2285297	-0.143241491	0.216502738	-0.661615148	0.508217897	0.910799399
RNPEP	1051.898006	-0.079160113	0.119651341	-0.661589847	0.508234116	0.910799399
TRIM5	348.8359057	0.093236088	0.140933358	0.661561533	0.508252267	0.910799399
PSMB5	1405.719965	0.080931285	0.122350179	0.661472554	0.50830931	0.910799399
SERHL2	117.9413077	0.28689505	0.433755302	0.661421424	0.50834209	0.910799399
GPX2	26.23921267	0.289486998	0.437711767	0.66136444	0.508378625	0.910799399
TNFSF4	28.27954591	-0.242376087	0.366531676	-0.661269143	0.508439726	0.910799399
NOTCH1	66.94768317	-0.181233112	0.274089888	-0.661217797	0.50847265	0.910799399
FCSK	213.4849537	0.151956528	0.229848616	0.661115697	0.50853812	0.910799399
KIAA1522	393.8621127	0.242156678	0.366302501	0.661083878	0.508558524	0.910799399
QTRT2	474.4071394	0.082655799	0.12503154	0.661079586	0.508561276	0.910799399
GNRH2	3.225064494	0.897424743	1.357880734	0.660901006	0.508675801	0.910799399
ZC3H18-AS1	5.216180759	0.712369435	1.077897468	0.660887938	0.508684182	0.910799399
POF1B	11.13653579	-0.384235418	0.581471382	-0.660798502	0.508741544	0.910799399
C2orf69	635.8566498	0.085153771	0.128882319	0.660709486	0.508798639	0.910799399
TMEM233	44.01793804	0.264775767	0.400820246	0.660584813	0.508878611	0.910799399
PCDHGB1	34.13464736	0.258441484	0.391238541	0.660572662	0.508886406	0.910799399
NQO1	227.4696103	0.176232565	0.266819702	0.660493073	0.508937462	0.910799399
MAK	35.86027199	0.394679882	0.597634283	0.660403684	0.508994809	0.910799399
GPALPP1	203.8092648	0.122941736	0.18617817	0.660344526	0.509032763	0.910799399
RAPGEFL1	64.62350704	0.345415344	0.523111313	0.66030945	0.509055267	0.910799399
CD9	283.3160045	0.415743233	0.629635226	0.660292206	0.509066331	0.910799399
FAM184B	17.36091365	0.308409496	0.467145959	0.660199431	0.509125857	0.910814454
FAM13A-AS1	19.29366077	0.401391818	0.608138572	0.660033479	0.509232345	0.910852676
FBXL12	464.4099571	0.082336996	0.124751734	0.660006823	0.509249451	0.910852676
TRDMT1	404.6548173	-0.141107547	0.213857422	-0.659820671	0.509368917	0.910874675
PPL	27.58330887	0.281064483	0.426024454	0.65973791	0.509422034	0.910874675
EMC4	655.632774	-0.078860308	0.11954412	-0.659675342	0.509462194	0.910874675
SLC12A9-AS1	2.68279122	0.890660322	1.350571919	0.659469007	0.509594642	0.910874675
ZNF292	1566.586667	0.088219223	0.133773307	0.659468056	0.509595252	0.910874675
ELP6	261.090916	-0.110467439	0.167535457	-0.659367522	0.509659793	0.910874675
ASXL2	1317.771301	-0.061871399	0.093878565	-0.65905779	0.509858659	0.910874675
TSC1	860.1955078	-0.065520957	0.099417113	-0.659051087	0.509862963	0.910874675
COL14A1	90.39743938	-0.171728186	0.260591762	-0.658993147	0.509900169	0.910874675
SNORD144	6.873952606	0.600941025	0.912161285	0.658810054	0.51001775	0.910874675
DFFB	188.8372411	0.120198083	0.182461006	0.658760385	0.51004965	0.910874675
HSDL1	253.2276971	0.122439057	0.185902193	0.658620833	0.510139282	0.910874675
LINC02258	12.42671361	0.384114732	0.583434111	0.658368656	0.510301271	0.910874675
CFAP69	33.64699498	-0.271871173	0.412986587	-0.65830509	0.510342108	0.910874675
FOXM1	1053.932717	0.071934845	0.109276914	0.658280352	0.510358001	0.910874675
SRRT	991.2366208	-0.136203063	0.206942265	-0.658169385	0.510429295	0.910874675
LOC101928386	4.930488625	-0.597812957	0.908312413	-0.658157864	0.510436697	0.910874675
C11orf95	460.5894146	-0.121703081	0.184959333	-0.657999132	0.510538689	0.910874675
ALG8	610.0070097	0.09885439	0.150262037	0.65788001	0.510615237	0.910874675
GABRG1	12.89726194	-0.346134538	0.5261649	-0.657844218	0.510638237	0.910874675
SAMD12	25.97102084	0.355843045	0.540928718	0.657837222	0.510642734	0.910874675
SNORD114-24	10.72386813	-0.392003872	0.595962383	-0.657766134	0.510688419	0.910874675
NOTCH2NLC	620.4297488	0.358071105	0.544494834	0.657620758	0.510781853	0.910874675
BIRC6-AS2	8.582175525	0.522186275	0.794284357	0.657429888	0.510904539	0.910874675
SH2D6	6.895626325	-0.553091218	0.841305919	-0.657419858	0.510910986	0.910874675

LOC102723885	13.01126854	0.40511697	0.616266706	0.6573728	0.510941236	0.910874675
PTH1R	29.70805164	-0.366589786	0.557691225	-0.657334686	0.510965738	0.910874675
SDS	10.07993389	0.462167534	0.703310078	0.657131966	0.511096066	0.910874675
RPS10	1828.267277	-0.150591755	0.2291948	-0.657046997	0.511150698	0.910874675
SNORD1C	18.32342427	0.36801675	0.560162625	0.656981979	0.511192504	0.910874675
LIN9	225.3313137	0.108675394	0.165419382	0.656968929	0.511200895	0.910874675
LOC101927855	3.265522206	-0.806405683	1.227482037	-0.656959254	0.511207116	0.910874675
SLC26A11	65.30037169	-0.163371591	0.2487029	-0.656894595	0.511248694	0.910874675
MAT2A	5161.971902	-0.166563893	0.253564412	-0.656889867	0.511251734	0.910874675
SPACA6P-AS	25.87692154	0.352070566	0.536005555	0.656841264	0.511282989	0.910874675
PPP1R21	483.8237181	-0.081355068	0.123865223	-0.656803143	0.511307503	0.910874675
NIP7	692.4376429	-0.102708993	0.156408509	-0.656671392	0.511392233	0.910874675
SCAT1	3.980376504	0.650295292	0.990394315	0.656602408	0.5114366	0.910874675
DIPK1C	112.8096913	0.159098397	0.242317017	0.656571293	0.511456612	0.910874675
MFSD12	509.0383171	0.102869308	0.156701532	0.656466513	0.511524007	0.910874675
HCFC2	597.4291236	0.086295056	0.131466686	0.656402463	0.511565206	0.910874675
TRAF2	488.6282264	0.121321085	0.184831704	0.656386766	0.511575303	0.910874675
GSE1	922.5244921	0.124731157	0.190044095	0.656327455	0.511613456	0.910874675
LINC02867	7.617482526	0.596613626	0.909076366	0.656285488	0.511640453	0.910874675
HOXD12	17.06742568	-0.320861467	0.488969279	-0.656199645	0.511695677	0.910874675
PALM2AKAP2	3477.769447	0.09992071	0.152281332	0.656158633	0.511722062	0.910874675
LRIG1	267.006712	-0.134570417	0.205118476	-0.656061902	0.511784296	0.910874675
WDR33	1134.912738	-0.098115493	0.149567445	-0.655994977	0.511827356	0.910874675
OBI1	278.9201084	-0.112290222	0.17118702	-0.655950562	0.511855934	0.910874675
ZDHHC1	103.9549153	-0.15103758	0.230267818	-0.655921357	0.511874726	0.910874675
POU3F2	11.84648905	0.382880319	0.583784465	0.655859041	0.511914824	0.910874675
KIF27	107.1747008	0.161469303	0.246211935	0.65581428	0.511943627	0.910874675
FGFR1OP2	289.2880976	0.114434151	0.174520009	0.65570791	0.512012078	0.910874675
FGFBP3	19.23553135	0.323876271	0.493998397	0.65562211	0.512067296	0.910874675
HIP1R	345.5705829	0.109066631	0.166358223	0.655613104	0.512073092	0.910874675
LAMA3	5.978618402	0.518741227	0.791388832	0.655482116	0.512157397	0.910933707
DDX39B-AS1	6.002126015	-0.56081036	0.855892939	-0.65523424	0.512316952	0.910999371
ZNF280A	14.59252788	0.350229712	0.534544852	0.655192375	0.512343903	0.910999371
GABRG2	4.599362731	-0.763957297	1.166030926	-0.65517756	0.512353441	0.910999371
FLVCR2	20.88826955	-0.268838956	0.410448277	-0.654988634	0.512475072	0.910999371
NEK7	986.8729915	-0.073542541	0.112297483	-0.654890381	0.512538334	0.910999371
MED26	139.3679922	0.132980121	0.203106009	0.65473258	0.512639945	0.910999371
RBBP8	714.6999013	-0.106585906	0.162836317	-0.654558565	0.512752009	0.910999371
AMZ1	15.14631671	0.413089945	0.63123322	0.654417308	0.512842986	0.910999371
CRIM1	1040.492543	0.114955283	0.175668407	0.654387918	0.512861916	0.910999371
PER1	192.6468816	0.124613249	0.19049797	0.654144759	0.513018547	0.910999371
ECPAS	5123.656597	-0.056114446	0.085797597	-0.65403284	0.513090649	0.910999371
FAM220A	458.4989819	-0.12857348	0.196612671	-0.653943002	0.513148528	0.910999371
ZNF528	57.41885727	0.233766773	0.357514592	0.653866382	0.513197895	0.910999371
THAP7	266.1382912	0.104115355	0.159241233	0.653821579	0.513226763	0.910999371
MIR4263	13.26074348	0.338894619	0.518370497	0.653769111	0.51326057	0.910999371
FSCN2	39.37827692	0.266786687	0.408099431	0.653729622	0.513286016	0.910999371
SLC16A12	2.891716551	0.873699376	1.336527642	0.653708422	0.513299676	0.910999371
BAIAP3	21.76820615	0.315343792	0.482393029	0.65370719	0.51330047	0.910999371
POMGNT1	1007.626471	0.070210297	0.107417476	0.653620802	0.513356139	0.910999371
JAK2	314.8439222	-0.09755287	0.149252014	-0.653611753	0.513361971	0.910999371
MRPS18A	102.8363291	-0.134923301	0.206461825	-0.653502416	0.513432433	0.910999371
TAF1B	848.9847169	-0.064951493	0.099402444	-0.653419483	0.513485882	0.910999371
COA5	250.0869646	-0.112851427	0.172719903	-0.653378241	0.513512463	0.910999371
ADGRB1	16.49919528	-0.362179128	0.554328901	-0.653365047	0.513520967	0.910999371
MLST8	387.7885361	-0.115906786	0.177423249	-0.653278453	0.513576781	0.910999371
MYADM	693.4082173	0.126383598	0.193481174	0.653208761	0.513621703	0.910999371
RNF5	461.3264245	-0.140816613	0.215592007	-0.653162494	0.513651527	0.910999371
WIPF3	2.525039178	1.159191297	1.774830172	0.653128009	0.513673756	0.910999371
KLHL18	524.7207904	0.121738609	0.186401294	0.653099593	0.513692075	0.910999371
ANAPC13	425.4144521	0.153469449	0.235006332	0.653043889	0.513727984	0.910999371
MAML1	1028.538561	0.06970717	0.106770249	0.652870725	0.513839624	0.911106676
PHOSPHO2	43.92218027	0.322086012	0.493517106	0.652633937	0.513992302	0.911239994
ZFAT-AS1	10.33564093	0.447677105	0.686057593	0.65253575	0.514055618	0.911239994
MATK	2.517720423	-0.760308351	1.165220637	-0.652501618	0.514077629	0.911239994
SLC27A4	1073.490051	0.124094549	0.190201605	0.652436919	0.514119354	0.911239994
CCDC25	1216.924137	-0.123228764	0.188929473	-0.652247435	0.514241564	0.911365954
GAS6-DT	2.670714949	0.789242496	1.210208332	0.652154241	0.514301676	0.911371833
UNC119	193.2481105	0.109684825	0.168206662	0.652083717	0.514347168	0.911371833
NT5DC1	454.8378985	-0.097728869	0.149915375	-0.651893573	0.514469832	0.911498548
CCDC115	265.1736126	-0.089370061	0.137119724	-0.651766635	0.514551729	0.911553017
HSPA14	549.6447898	0.093934003	0.144173518	0.651534379	0.514701592	0.91172787
CYBRD1	1353.887653	-0.179589047	0.275738369	-0.651302348	0.514851333	0.911756783
LOC729558	6.167411069	-0.797303553	1.22430737	-0.651228256	0.514899153	0.911756783
ZKSCAN8	1473.271764	0.093666732	0.14385288	0.651128658	0.514963438	0.911756783
OLFM2	315.12672	-0.127334036	0.195576765	-0.651069343	0.515001725	0.911756783
GIT2	889.9676036	-0.06368998	0.097848263	-0.650905576	0.515107442	0.911756783
PNPLA2	218.4926315	-0.118716325	0.182390393	-0.650891324	0.515116642	0.911756783
ZNF846	96.5845134	-0.153588854	0.236080478	-0.650578378	0.515318691	0.911756783
ACTR1B	1383.875096	-0.066301779	0.101913272	-0.650570606	0.51532371	0.911756783
SPAG8	18.38177245	0.312349835	0.480201836	0.650455313	0.515398158	0.911756783
ABHD5	174.3402393	0.109087074	0.167724649	0.650393814	0.515437872	0.911756783
TOR1A	476.0895463	0.073183108	0.11252395	0.650378055	0.515448049	0.911756783
CNTN2	7.44729088	-0.51975141	0.799227975	-0.650316838	0.515487583	0.911756783
KCNK5	6.244485754	0.531318633	0.81711	0.650241256	0.515536396	0.911756783
PSMG4	419.2439265	0.208532448	0.320719547	0.650201866	0.515561836	0.911756783
KLF15	3.228594288	0.754413353	1.160276809	0.650201182	0.515562278	0.911756783
CDYL2	11.14121946	0.528811035	0.813304084	0.650200885	0.515562469	0.911756783
CC2D2B	27.58575901	0.409225945	0.629644609	0.649931627	0.515736388	0.911756783
COX6B1	273.4193937	0.177860244	0.273679288	0.649885656	0.515766084	0.911756783

MIGA1	452.8009798	0.091235428	0.140394576	0.649850091	0.515789059	0.911756783
ZNF92	106.1691837	0.14860893	0.22869893	0.649801599	0.515820386	0.911756783
STIM2	217.1845836	0.105666648	0.162725658	0.649354555	0.516109231	0.911756783
HDLBP	8183.605422	-0.111889033	0.172331241	-0.649267261	0.516165643	0.911756783
PARP12	267.3861604	-0.096398218	0.148472799	-0.64926518	0.516166988	0.911756783
ZNF473	353.2560433	-0.111374151	0.171555038	-0.649203619	0.516206773	0.911756783
ZNF334	37.03253534	-0.255191992	0.393122216	-0.649141619	0.516246843	0.911756783
MIR3934	17.35933809	0.445528961	0.686411181	0.649070081	0.516293079	0.911756783
CCDC91	234.5765758	-0.098274556	0.151410862	-0.649058823	0.516300356	0.911756783
ZBTB10	1279.563442	0.057552178	0.088672608	0.649041224	0.516311731	0.911756783
HDDC2	432.8397876	0.087574076	0.134932966	0.64901913	0.516326011	0.911756783
ZMYM6	388.4290465	0.085960761	0.132450546	0.649002692	0.516336636	0.911756783
FAM50A	381.7203186	-0.084387826	0.130032766	-0.648973551	0.516355472	0.911756783
RBM12B	1292.110605	0.121910995	0.187910176	0.648772718	0.516485294	0.911756783
LOC100128494	29.5697508	0.367475373	0.566424959	0.648762677	0.516491785	0.911756783
VSIG10	350.691137	0.135030177	0.208187082	0.648600171	0.516596845	0.911756783
UBR4	9836.078521	0.106749013	0.164591241	0.648570434	0.516616071	0.911756783
NBPF11	53.81942345	0.191056215	0.294613879	0.648496995	0.516663554	0.911756783
ALK	6.370965968	0.845883978	1.304428036	0.648471172	0.51668025	0.911756783
TSPYL4	270.6629356	0.142386845	0.219614251	0.648349751	0.516758762	0.911756783
LRRC34	146.9818196	0.170429257	0.262895916	0.648276548	0.5168061	0.911756783
LINC01142	34.84074853	0.221476856	0.341724127	0.648115946	0.516909961	0.911756783
ZNF192P1	23.15753084	0.314143507	0.484781416	0.648010623	0.51697808	0.911756783
YWHAH	1174.685223	-0.10877732	0.167864753	-0.648005717	0.516981252	0.911756783
EFCAB11	82.72599381	0.176098729	0.271757011	0.648000683	0.516984509	0.911756783
ACTL6A	2175.810979	-0.089944219	0.138811046	-0.647961536	0.517009828	0.911756783
CLTC	15060.71502	-0.081452656	0.125711159	-0.647934971	0.517027011	0.911756783
SMCO3	11.57138967	0.567626662	0.876147982	0.647866197	0.517071495	0.911756783
MIR1271	6.067199814	0.670859972	1.0357531	0.647702596	0.517177325	0.911853164
TMTC4	371.7041241	0.086113058	0.133040295	0.647270497	0.517456892	0.912092313
ZNF426-DT	4.137025371	0.648469336	1.002042845	0.647147314	0.517536606	0.912092313
GLI3	1335.560828	0.175696269	0.271518347	0.647088019	0.517574979	0.912092313
SKIDA1	120.6612185	0.129357908	0.199939905	0.646983946	0.517642334	0.912092313
FOSL2	17429.52798	0.143948977	0.222531226	0.646870908	0.517715495	0.912092313
RARRES1	22.56905877	0.587217364	0.907930086	0.646764958	0.517784075	0.912092313
NDUFA2	144.0655424	0.137222281	0.212169326	0.646758341	0.517788358	0.912092313
PTPN6	7.803258737	-0.452900371	0.700507514	-0.646531781	0.517935022	0.912092313
ADAMTS19	24.51960992	0.437882552	0.67734107	0.646472762	0.517973231	0.912092313
BLID	39.67324598	0.465551422	0.720166529	0.64644968	0.517988175	0.912092313
SLC45A4	24.11508277	-0.38788275	0.600061483	-0.646405012	0.518017096	0.912092313
LSM1	655.4367509	0.08938549	0.138303919	0.646297592	0.518086647	0.912092313
MAGI1-IT1	16.83528827	0.68733377	1.063500939	0.646293524	0.518089281	0.912092313
SPOP	1454.758482	-0.080956717	0.125271388	-0.646250654	0.51811704	0.912092313
GANC	316.8622589	-0.110652096	0.171225095	-0.64623761	0.518125486	0.912092313
NOA1	559.0287429	-0.158980416	0.246013001	-0.646227702	0.518131902	0.912092313
ATP6V0A1	354.8013554	0.121256638	0.187672815	0.646106563	0.518210345	0.912140295
ADAM7	3.104208935	-0.968164657	1.498807783	-0.645956518	0.518307516	0.912221227
JAK1	1935.176653	-0.104410964	0.161690495	-0.645745841	0.518443968	0.912288309
CASC11	5.021243425	-0.632409438	0.979356775	-0.645739585	0.51844802	0.912288309
CLBA1	162.1972633	-0.112532545	0.174314772	-0.645570906	0.518557285	0.912390483
ARGFXP2	11.86707106	0.443001926	0.686359139	0.645437499	0.518643709	0.912452453
NFAT5	1540.104049	-0.082560195	0.128025998	-0.644870549	0.519011079	0.912736489
FAM118B	154.1527025	0.117158817	0.181688626	0.644832974	0.519035431	0.912736489
FAM207A	117.0273092	-0.181549881	0.281546073	-0.644831871	0.519036146	0.912736489
TLR4	239.7377547	0.412408803	0.639623008	0.644768556	0.519077182	0.912736489
VPS72	451.9437701	-0.111529208	0.172987272	-0.644724937	0.519105453	0.912736489
RAB31	1357.53504	-0.150726143	0.23378757	-0.644714103	0.519112476	0.912736489
TCFL5	436.9721381	-0.094495586	0.146605875	-0.644555243	0.519215447	0.912826076
ZNF300	145.4228875	0.167360345	0.259683788	0.644477447	0.519265878	0.912826076
MYO1H	19.26227844	0.40513925	0.628792345	0.644313267	0.519372315	0.912858053
LOC100130207	5.778906545	0.57888254	0.898575975	0.644222143	0.519431394	0.912858053
RTN4RL1	2.874271144	-0.772615102	1.199317443	-0.644212344	0.519437747	0.912858053
NGRN	1302.42742	-0.07659585	0.118928502	-0.644049559	0.519543298	0.912863953
GOLGA2P7	114.7520738	0.148435053	0.230503113	0.643961165	0.519600617	0.912863953
HYPK	23.90903352	0.271942596	0.422381952	0.643831004	0.519685027	0.912863953
CRYL1	117.6810362	-0.224903343	0.349343845	-0.643787907	0.519712976	0.912863953
STAMBPL1	48.41697125	0.189156269	0.293841322	0.643736109	0.519746571	0.912863953
C1QTNF7-AS1	11.10142845	0.477178817	0.741351663	0.643660547	0.519795579	0.912863953
TMEM155	3.989097912	0.68026613	1.05717335	0.643476427	0.519915006	0.912863953
UBE2Q2P2	5.112148102	0.587931528	0.913732777	0.643439245	0.519939125	0.912863953
UBE2J1	1118.205242	-0.107077626	0.166462252	-0.643254702	0.520058843	0.912863953
HSD17B14	35.57395889	-0.251037804	0.39026621	-0.643247604	0.520063449	0.912863953
ZNF583	112.3031236	0.132136851	0.205460807	0.643124365	0.520143406	0.912863953
TRPC1	804.4240334	0.131687629	0.204771918	0.643094184	0.520162988	0.912863953
MRPL9	310.9988152	-0.097007231	0.150877538	-0.642953436	0.520254315	0.912863953
CCDC36	237.403251	0.136419073	0.212239039	0.642761453	0.520378899	0.912863953
KPNA6	2596.337173	0.094493277	0.147013805	0.642751045	0.520385653	0.912863953
ZNF496	1200.600165	0.084338355	0.131221015	0.642719875	0.520405882	0.912863953
AVEN	102.785829	-0.177436038	0.276084602	-0.642687192	0.520427093	0.912863953
MGAT2	1244.652122	0.068818363	0.107085458	0.642649003	0.520451879	0.912863953
PWP1	1692.087395	-0.088220977	0.137285878	-0.642607805	0.520478617	0.912863953
STARD7	5265.440142	0.071290883	0.110945489	0.642575772	0.520499408	0.912863953
MYOZ1	3.20566028	-0.764980009	1.190539812	-0.642548868	0.52051687	0.912863953
NPC2	618.3516382	-0.152500299	0.237381917	-0.64242593	0.520596668	0.912892695
SLC12A6	719.8046292	0.142809944	0.222347861	0.642281618	0.520690347	0.912892695
BACE1	1214.852236	-0.137331394	0.213863289	-0.642145713	0.520778577	0.912892695
GNG3	15.28188083	-0.457881007	0.713128428	-0.642073698	0.520825333	0.912892695
LRRN4CL	6.879424064	-0.655010152	1.020270641	-0.641996472	0.520875474	0.912892695
MCOLN2	84.53379454	0.183208405	0.285374037	0.641993948	0.520877113	0.912892695
PDCL3	161.6149625	-0.131169087	0.204322392	-0.64197118	0.520891896	0.912892695

ZBTB42	92.63395093	0.145156049	0.226153111	0.641848564	0.520971514	0.912892695
ZNF618	1018.043198	0.082312646	0.128250111	0.641813449	0.520994316	0.912892695
SMG1	4256.394227	0.114813136	0.178944043	0.641614742	0.521123359	0.912963708
TNFSF13B	8.832015963	-0.38429097	0.598963537	-0.641593262	0.521137309	0.912963708
DDX20	394.3221097	-0.092662475	0.144456184	-0.641457312	0.521225607	0.912965756
GBE1	843.6994672	-0.06857074	0.106940607	-0.641203954	0.521390181	0.912965756
FRS3	82.39502301	0.170385505	0.265729599	0.641198819	0.521393517	0.912965756
PPIAP30	3.392445889	0.869573058	1.356179467	0.641193204	0.521397164	0.912965756
RHOT2	1205.848627	0.147874507	0.230643727	0.641138212	0.52143289	0.912965756
PSMD8	1136.128968	-0.092717951	0.144648382	-0.640988512	0.521530147	0.912965756
TP73-AS1	632.2914538	-0.091522287	0.142783367	-0.640987036	0.521531106	0.912965756
ST7L	155.8078894	-0.120772004	0.188444808	-0.640887937	0.521595494	0.912965756
ARFGEF1	2802.492857	-0.058808384	0.091762483	-0.640876117	0.521603174	0.912965756
GCFC2	439.3605335	0.095612027	0.149246568	0.640631326	0.521762242	0.912965756
RAB4B-EGLN2	2.660762425	0.723318348	1.129154905	0.640583807	0.521793123	0.912965756
LINC01635	12.35008975	0.543551134	0.848809578	0.640368756	0.52193289	0.912965756
GPATCH1	213.0528731	0.110600291	0.172724354	0.640328292	0.521959191	0.912965756
TSEN2	250.8828583	0.120959923	0.188922966	0.640260557	0.522003219	0.912965756
THUMPD2	327.0869827	0.172553113	0.269506633	0.640255533	0.522006485	0.912965756
LMTK3	25.77180734	-0.316732602	0.494798174	-0.640124841	0.522091441	0.912965756
SLC12A5	3.133097866	-0.759332704	1.186308657	-0.640080218	0.522120449	0.912965756
TPK1	31.46237184	0.237850744	0.371606011	0.640061616	0.522132542	0.912965756
KIRREL1-IT1	17.72212663	0.510071239	0.797016036	0.639976131	0.522188117	0.912965756
MIR663A	91837.64475	0.178705793	0.279241543	0.639968507	0.522193074	0.912965756
COIL	366.2362821	0.091814111	0.143473949	0.639935762	0.522214363	0.912965756
SNCAIP	24.38534533	0.387613113	0.605846988	0.639787142	0.522310993	0.912990537
LINC01622	10.73268617	0.580561464	0.907472746	0.639756364	0.522331006	0.912990537
SVIL2P	9.869688404	-0.423645652	0.662577805	-0.639390044	0.522569224	0.913146123
MSH4	7.528762423	0.583091112	0.911977685	0.639369933	0.522582304	0.913146123
DLG4	357.1127667	-0.130742118	0.204496384	-0.639337065	0.522603681	0.913146123
NLGN3	12.77556264	-0.370616809	0.579718933	-0.639304304	0.522624989	0.913146123
MKNK1	247.0256015	-0.12074939	0.18891906	-0.639159382	0.522719253	0.913221283
PROS1	227.8003212	-0.180238394	0.282080078	-0.638961798	0.522847784	0.913315393
GRAMD1C	77.04447455	-0.199763942	0.312676021	-0.638884751	0.522897909	0.913315393
POLB	266.5753795	-0.097398186	0.152478424	-0.638767002	0.522974518	0.913315393
ZIK1	126.6082836	-0.166008529	0.259891277	-0.638761451	0.52297813	0.913315393
SOGA3	80.49751298	0.164601691	0.25778993	0.638510941	0.523141134	0.913421406
SLC41A2	270.7078124	0.173908853	0.272366428	0.638510608	0.523141351	0.913421406
BNIP2	1335.661275	-0.077062586	0.120738217	-0.638261754	0.523303304	0.913476871
MIR4505	5.645499199	0.640510733	1.003626731	0.638196167	0.523345992	0.913476871
EXOSC1	176.8805265	-0.106582391	0.167007994	-0.638187364	0.523351722	0.913476871
SLC10A5	8.288019361	-0.456911922	0.715998207	-0.638146741	0.523378163	0.913476871
MMS19	1291.780318	-0.060023545	0.094076646	-0.638028109	0.523455382	0.913522173
AMDHD1	21.22060541	-0.324190467	0.508207835	-0.637909226	0.523532772	0.913567762
DUS4L	43.04388999	-0.190052929	0.298032928	-0.637691044	0.523674816	0.913567828
FAM155A-IT1	13.15222903	0.531616462	0.833722767	0.637641771	0.523706898	0.913567828
TIPIN	216.1862881	-0.148085488	0.23225149	-0.637608345	0.523728662	0.913567828
RTF2	785.603448	-0.101424507	0.159152081	-0.637280435	0.523942193	0.913567828
NDUFAF3	498.9701905	0.109846237	0.172380732	0.637230366	0.523974801	0.913567828
SERINC5	1177.960554	-0.152629862	0.239530809	-0.637203468	0.523992319	0.913567828
RMI1	348.1480123	-0.081406416	0.127766464	-0.637150106	0.524027073	0.913567828
CALML6	2.616791147	-0.773285189	1.214301638	-0.636814745	0.52424552	0.913567828
LINC00240	3.833341183	0.678240932	1.06505514	0.636812975	0.524246673	0.913567828
MFSD10	736.9880341	-0.099416915	0.156117937	-0.636806485	0.524250902	0.913567828
ATP6V1G2	8.192670452	-0.418425601	0.65725312	-0.63662779	0.524367319	0.913567828
COPZ2	183.182207	0.123595671	0.194191141	0.636464005	0.524474035	0.913567828
PDC	3.889878307	0.681320729	1.070601787	0.636390427	0.524521979	0.913567828
PPP1R14B	497.8028198	-0.099140218	0.155811174	-0.636284394	0.524591075	0.913567828
DGCR6L	379.2883683	-0.091736823	0.144181127	-0.636260961	0.524606346	0.913567828
LOC101928988	2.397991625	0.749221015	1.177724254	0.636159961	0.524672167	0.913567828
CDK8	436.8737224	-0.092612953	0.145585858	-0.636139761	0.524685332	0.913567828
CD36	2.951004758	0.742647599	1.167578669	0.636057868	0.524738705	0.913567828
NDUFS5	97.9958653	0.142536489	0.224113715	0.636000742	0.524775938	0.913567828
HSBP1	534.1058594	-0.079708757	0.125346335	-0.63590816	0.524836284	0.913567828
POLR3K	169.9501168	0.150425405	0.236652907	0.635637257	0.52501288	0.913567828
RAB14	1978.85618	-0.083225912	0.13093967	-0.635605024	0.525033894	0.913567828
ADIPOR2	1675.160323	-0.054960034	0.08647621	-0.635550913	0.525069172	0.913567828
UBE2E2	441.1428965	0.100321981	0.157864152	0.635495645	0.525105206	0.913567828
ATAD5	618.5917355	0.150723392	0.237193718	0.635444282	0.525138695	0.913567828
CA1	7.173732093	0.653005442	1.027641304	0.635441023	0.52514082	0.913567828
ELL3	37.10104832	0.215237821	0.338847983	0.635204669	0.525294939	0.913567828
PANCR	2.706768885	0.918016409	1.445300954	0.635173184	0.52531547	0.913567828
EMSY	303.8167023	0.116634397	0.183642747	0.635115725	0.525352942	0.913567828
LOC100506679	6.391524708	0.523279655	0.823954679	0.635083056	0.525374247	0.913567828
DDX47	634.1891973	0.066606029	0.104884955	0.635038925	0.525403028	0.913567828
C4orf48	34.49884014	-0.224212278	0.353070846	-0.63503481	0.525405712	0.913567828
DNAJC27-AS1	2.785077048	-1.023474656	1.611696064	-0.635029569	0.525409131	0.913567828
TIGD1	200.8403231	0.159143566	0.250653903	0.634913577	0.525484782	0.913567828
FAM66C	40.27846257	-0.195305837	0.307620569	-0.634891996	0.525498857	0.913567828
PHF2	1103.684211	0.106124894	0.167154793	0.634889921	0.525500211	0.913567828
PJVK	41.63435125	0.218141621	0.343607149	0.634857633	0.525521271	0.913567828
NME3	90.9454776	-0.149401171	0.235343715	-0.634821164	0.525545059	0.913567828
KNSTRN	412.9859159	0.09788823	0.154200785	0.634810194	0.525552214	0.913567828
TBC1D22A-AS1	17.42269946	0.45175587	0.711760583	0.634702006	0.525622785	0.913567828
CFAP53	17.40935205	-0.273420033	0.43079732	-0.634683691	0.525634733	0.913567828
IBA57-DT	6.297003014	0.674739649	1.063393241	0.634515645	0.52574436	0.91366925
S1PR5	11.61724082	-0.339498055	0.535287812	-0.634234606	0.525927726	0.913701176
ERCC8	191.4630508	-0.118901588	0.18747592	-0.634223254	0.525935133	0.913701176
LINC01362	3.573595835	1.029611909	1.623868201	0.634048939	0.526048884	0.913701176
OTX1	54.30772314	-0.190095405	0.299821379	-0.634028853	0.526061993	0.913701176

MYBL1	388.8657299	0.126233561	0.199104486	0.634006613	0.526076506	0.913701176
MIR151A	4.082549469	0.687760203	1.084973457	0.633895879	0.526148775	0.913701176
LRP6	1584.311755	-0.072538196	0.114436554	-0.633872595	0.526163972	0.913701176
TPI1	4847.974422	-0.087460632	0.138029414	-0.633637639	0.526317331	0.913701176
RNF214	239.1154493	-0.097629969	0.154083072	-0.633619048	0.526329467	0.913701176
HSPH1	2053.723701	0.130832368	0.206484286	0.633619007	0.526329494	0.913701176
UTP3	194.4996686	0.110019863	0.173640071	0.63360872	0.526336209	0.913701176
CMBL	526.6646844	-0.166216957	0.262360279	-0.633544673	0.526378018	0.913701176
CDC23	1003.601086	-0.071017334	0.112151461	-0.633227004	0.526585414	0.913744062
FBXL16	12.3724567	0.397940157	0.628522525	0.633135872	0.526644919	0.913744062
LINC01032	8.881990436	0.581716328	0.919033226	0.632965503	0.526756171	0.913744062
YAE1	175.6071813	-0.100994274	0.159564678	-0.632936282	0.526775253	0.913744062
DENND2B	313.2515391	-0.126722507	0.200213867	-0.632935715	0.526775624	0.913744062
SRP54	1230.17862	-0.074322922	0.117468526	-0.632704983	0.526926315	0.913744062
LINC02294	4.455901149	-0.668528724	1.056636699	-0.632694969	0.526932856	0.913744062
ALKBH5	2539.2262	0.107588563	0.170050889	0.632684509	0.526939688	0.913744062
SNORD133	107.9314942	-0.223007697	0.352496772	-0.632651743	0.52696109	0.913744062
NOS3	129.0134065	-0.126179461	0.199454034	-0.632624259	0.526979041	0.913744062
NEDD4L	514.1387397	0.100614951	0.159050055	0.632599283	0.526995356	0.913744062
TIMM29	210.6219586	0.098351093	0.15547993	0.632564555	0.52701804	0.913744062
SNORD111B	12.66850176	0.407899238	0.644947035	0.632453855	0.527090352	0.913780531
CDC6	677.6556366	-0.136433247	0.215790452	-0.632248769	0.527224334	0.913923894
BCAT2	407.1073274	-0.088311682	0.139728612	-0.632022897	0.527371916	0.914017652
ZNF528-AS1	11.6421843	-0.46583355	0.737074177	-0.632003623	0.52738451	0.914017652
ATP1A1-AS1	46.50932586	-0.225172542	0.356351566	-0.631883128	0.527463249	0.914017652
DLG1-AS1	6.388166425	0.502265944	0.794952522	0.631818795	0.527505291	0.914017652
ZNF396	16.3116502	-0.37033529	0.586189417	-0.631767273	0.527538962	0.914017652
ZNF586	169.6007447	0.142746873	0.225974346	0.631695038	0.527586171	0.914017652
USP35	203.5663095	0.118915148	0.188298255	0.631525492	0.527696987	0.914101103
AGBL5	608.5421938	-0.077731042	0.123110589	-0.631392003	0.527784243	0.914101103
JMJD8	826.9775287	0.066265567	0.10495256	0.631385905	0.52778823	0.914101103
RHEB	576.601865	0.112457258	0.178148235	0.631256649	0.527872727	0.914158601
MICU1	508.3782163	0.109526096	0.173567941	0.631027225	0.528022724	0.914329505
ACACB	61.97310806	-0.164991377	0.261581677	-0.630745159	0.528207166	0.914354914
PPM1F	530.2937635	0.076280798	0.120948706	0.630687172	0.528245088	0.914354914
TTC39C	361.6149376	0.094848836	0.150399383	0.630646444	0.528271724	0.914354914
ZNF7	638.0856198	0.118091621	0.187312708	0.630451732	0.528399073	0.914354914
PRR15L	10.76655772	0.433369118	0.687455381	0.630395993	0.528435532	0.914354914
LOC100505736	54.93410025	0.420315491	0.66695188	0.630203623	0.528561369	0.914354914
LINC01619	2.488944679	0.819299195	1.30017464	0.630145497	0.528599395	0.914354914
DNAJC5	1065.268956	-0.0985687	0.156445283	-0.630052231	0.528660412	0.914354914
WDR55	459.5682133	-0.092294077	0.146488964	-0.630041166	0.528667651	0.914354914
MALSU1	279.8904084	-0.093354074	0.148203175	-0.629906032	0.528756066	0.914354914
THRB-AS1	5.433113653	0.519021014	0.824298801	0.629651545	0.528922591	0.914354914
PHLDA2	28.56039392	0.231567371	0.367786575	0.629624316	0.528940411	0.914354914
BCAP29	40.81868588	-0.196259597	0.311714287	-0.629613737	0.528947334	0.914354914
SLC25A5	2446.80256	-0.07859469	0.124848292	-0.629521549	0.529007666	0.914354914
FGD5-AS1	1781.24702	-0.082570975	0.131168525	-0.629502961	0.529019831	0.914354914
MIR668	3.92116434	-0.922753253	1.465979612	-0.629444806	0.529057892	0.914354914
ACTR5	182.8330197	0.142537471	0.226540467	0.629192093	0.529223305	0.914354914
ALCAM	1193.784277	-0.157223273	0.249894653	-0.629158212	0.529245484	0.914354914
MMAA	107.4051462	-0.137823062	0.219111167	-0.629009758	0.529342668	0.914354914
SLC25A38	343.6254071	-0.093939853	0.149349302	-0.628994257	0.529352816	0.914354914
POGLUT3	1202.252758	-0.126915394	0.201800816	-0.62891418	0.529405242	0.914354914
VPS29	668.5760073	-0.095639858	0.152085755	-0.628854807	0.529444115	0.914354914
ANKRD20A4-ANKRD20A20P	6.710764776	-0.480485758	0.764124533	-0.628805563	0.529476357	0.914354914
DIRC3	74.99909071	-0.266763153	0.424275971	-0.628749142	0.5295133	0.914354914
TFCP2	498.3087201	-0.079250101	0.126045498	-0.628742021	0.529517963	0.914354914
PARD6G	203.163487	-0.096198418	0.153008798	-0.628711677	0.529537832	0.914354914
RPS11	1959.110377	-0.176814359	0.281271065	-0.628626192	0.529593809	0.914354914
BCKDK	539.7759773	-0.063562042	0.101115251	-0.628609841	0.529604516	0.914354914
RBM4	575.5652798	-0.082951014	0.131992312	-0.628453372	0.529706983	0.914354914
AQR	2000.834169	0.067309229	0.10710309	0.628452727	0.529707405	0.914354914
TMEM75	162.3310161	0.618532297	0.984424453	0.628318705	0.529795181	0.914354914
PLCB1-IT1	3.946185454	1.084379125	1.72587951	0.628305231	0.529804005	0.914354914
PCDHB5	175.574981	-0.128465517	0.20458359	-0.627936566	0.530045496	0.914354914
DNAH2	250.9351695	-0.140109488	0.223128772	-0.627931069	0.530049097	0.914354914
CSMD3	275.062589	0.142096154	0.226294498	0.627925801	0.530052548	0.914354914
ARHGAP10	319.9222338	0.111652181	0.177825454	0.627875136	0.530085741	0.914354914
ZNF559-ZNF177	6.472910505	0.550225232	0.876352955	0.62785802	0.530096954	0.914354914
CADM3	3.918491339	-0.697833276	1.111676153	-0.627730724	0.530180355	0.914354914
DCLRE1C	369.7172631	0.140645347	0.224102646	0.627593423	0.530270318	0.914354914
F12	142.6294304	0.141738633	0.22585469	0.627565596	0.530288552	0.914354914
HEXA-AS1	7.930736169	-0.470229004	0.749295958	-0.627561111	0.530291491	0.914354914
CNTNAP3B	1.950474157	1.195436447	1.905113259	0.627488388	0.530339146	0.914354914
ZNF551	289.3987319	0.188369742	0.300312607	0.627245536	0.530498299	0.914354914
KDM2A	1437.551992	-0.067605565	0.107794356	-0.627171653	0.530546722	0.914354914
DCUN1D4	3081.864408	-0.14315168	0.228287518	-0.627067486	0.530614999	0.914354914
LINC01352	3.528288426	-0.79423294	1.266596492	-0.627060745	0.530619418	0.914354914
KLHDC10	1187.269097	0.112605896	0.179585171	0.627033375	0.530637358	0.914354914
SMOC1	2.467197342	0.758170739	1.209193095	0.627005515	0.53065562	0.914354914
LINC01089	126.2154071	0.184356773	0.29403031	0.626999214	0.53065975	0.914354914
RNF144A	4389.567025	0.323771126	0.516442949	0.626925253	0.530708233	0.914354914
LINC00623	12.41656608	0.381712996	0.608883347	0.626906612	0.530720453	0.914354914
CDO1	31.57261633	-0.261943659	0.417885378	-0.626831358	0.530769786	0.914354914
CREG2	7.730248494	0.485703018	0.774858553	0.626827975	0.530772004	0.914354914
ZNF253	63.79531973	0.190277647	0.303592757	0.62675292	0.530821209	0.914354914
ANAPC5	4031.793346	0.060729455	0.09690526	0.626688947	0.53086315	0.914354914
TULP2	2.805130336	0.731198406	1.166899782	0.626616285	0.530910791	0.914354914
KLHL32	8.728454021	0.508826245	0.812329812	0.626378889	0.531066453	0.914534615

NAAA	121.0841347	-0.143524423	0.229204759	-0.626184305	0.53119406	0.914665973
FBXO33	370.5328578	0.088847373	0.141915273	0.626059275	0.531276063	0.914718787
NAIP	73.75240502	0.200339012	0.320061667	0.625938787	0.531355092	0.914766299
MYH1	13.22719583	0.388361406	0.620596324	0.625787474	0.531454349	0.914766299
FAM162B	37.88579847	0.228033256	0.364397027	0.625782428	0.531457659	0.914766299
GADD45GIP1	397.1369038	0.094274163	0.150707939	0.625542118	0.531615315	0.914949286
CEP78	830.9271232	0.10681413	0.170777168	0.625459076	0.531669799	0.914954691
PSAP	4959.153462	-0.171109276	0.273613138	-0.625369369	0.531728661	0.914967627
SLC4A9	5.465093182	0.623686431	0.997565272	0.625208644	0.531834129	0.91506075
LRRC29	20.52878778	-0.348938904	0.558297888	-0.62500488	0.531967856	0.915116246
CNN3	2866.757018	0.09621943	0.153963	0.624951643	0.532002796	0.915116246
MIR10527	6.171283078	0.569576073	0.911431444	0.624924756	0.532020444	0.915116246
BICRA	151.0501574	-0.141705829	0.226836899	-0.624703608	0.532165605	0.915124803
GNRH1	71.35613375	0.328940592	0.526737975	0.624486191	0.532308336	0.915124803
STOML2	1139.51372	-0.091850069	0.147082159	-0.624481373	0.532311499	0.915124803
LINC02449	39.26363504	0.293449835	0.469931463	0.62445241	0.532330515	0.915124803
ZC3H12B	61.57191496	-0.175700586	0.281406326	-0.624366158	0.532387145	0.915124803
LURAP1	43.81144495	0.303185667	0.485625859	0.624319444	0.532417817	0.915124803
CHIC1	265.2355064	0.108553383	0.173890509	0.624262839	0.532454985	0.915124803
ATM	2908.513739	0.060344301	0.096680827	0.624159962	0.532522538	0.915124803
IFT43	142.0376171	-0.128332645	0.205620255	-0.62412453	0.532545805	0.915124803
RB1	268.6996411	0.108457151	0.173776897	0.624117207	0.532550614	0.915124803
CHRNB1	69.83600215	0.184619871	0.29595709	0.623806211	0.53275486	0.915124803
KLHL7	340.3298588	-0.099301296	0.159190759	-0.623788069	0.532766776	0.915124803
C8orf76	188.0061945	0.110380503	0.176966334	0.623737296	0.532800125	0.915124803
PTAFR	2.430140722	-0.880666656	1.41225917	-0.62358714	0.532898759	0.915124803
RRAS	219.4605245	0.193810007	0.310846808	0.623490422	0.532962294	0.915124803
XRCC3	900.5983997	0.14295251	0.229299498	0.623431412	0.533001061	0.915124803
TEDC1	275.8537665	0.188941152	0.303117905	0.623325607	0.533070574	0.915124803
GLRX5	489.9812965	0.095837119	0.153785889	0.623185388	0.533162703	0.915124803
SLC39A14	9425.83214	-0.098639117	0.158304262	-0.623098299	0.533219927	0.915124803
FASTK	592.3911373	0.091840526	0.147466598	0.622788666	0.533423407	0.915124803
LINC01128	210.6016232	-0.101844724	0.163563196	-0.62266284	0.533506107	0.915124803
METTL8	670.2444091	-0.096688605	0.155303991	-0.622576439	0.533562898	0.915124803
SNORD49A	158.5124598	0.198984414	0.319629752	0.622546596	0.533582515	0.915124803
RPL13AP20	3.285161295	0.720498028	1.157384509	0.622522612	0.53359828	0.915124803
UBOX5-AS1	11.30945826	0.428375924	0.688195416	0.622462624	0.533637713	0.915124803
NFATC2IP	616.4817705	0.122750032	0.197217849	0.622408332	0.533673403	0.915124803
SMG7-AS1	16.57121351	0.379735261	0.610164472	0.622349021	0.533712394	0.915124803
GRAMD2A	11.7766048	0.347118249	0.557807378	0.622290531	0.533750847	0.915124803
CASP4	64.37147383	-0.260939327	0.419350949	-0.622245707	0.533780316	0.915124803
BHLHA15	3.176454631	-0.656151161	1.054548692	-0.622210398	0.53380353	0.915124803
SATB1	136.0102098	0.253666747	0.4076952	0.622197041	0.533812311	0.915124803
AKIRIN1	1050.201901	0.056506497	0.090818784	0.622189531	0.533817249	0.915124803
CORO1B	414.2113863	-0.105101818	0.168929185	-0.622164954	0.533833408	0.915124803
ASPH	4379.533611	-0.06676714	0.107329337	-0.622077263	0.533891065	0.915124803
NOP16	340.5366269	0.119326293	0.191821202	0.622070405	0.533895574	0.915124803
PKD1P3-NPIPA1	981.7894095	-0.167652154	0.269531815	-0.622012486	0.533933658	0.915124803
TACC3	892.4815682	0.067317087	0.10824476	0.621896959	0.534009625	0.915124803
B4GAT1	438.2121378	0.123861888	0.199222412	0.621726678	0.534121606	0.915124803
FOCAD-AS1	11.42265547	0.423668327	0.681519671	0.621652383	0.534170469	0.915124803
AGBL4	2.34614194	-0.888879266	1.430124372	-0.621539835	0.534244493	0.915124803
FOXO3B	264.644076	0.118791728	0.1911354	0.621505633	0.534266989	0.915124803
GLCE	768.9850913	-0.088372102	0.142198907	-0.621468221	0.534291597	0.915124803
SEZ6L2	67.54379513	-0.187427788	0.301652373	-0.621337024	0.534377898	0.915124803
CSK	1799.443392	0.074587778	0.120094972	0.62107328	0.534551409	0.915124803
CD59	1641.383005	0.108630211	0.174907765	0.621071406	0.534552642	0.915124803
SNORD73B	15.13125721	0.358549967	0.577349217	0.621027892	0.534581272	0.915124803
CEP192	1575.921175	0.068381948	0.110120869	0.620971739	0.534618218	0.915124803
STK39	288.6472628	-0.079932366	0.128746556	-0.620850517	0.534697982	0.915124803
LBHD1	108.278932	0.154456758	0.248841953	0.620702242	0.534795554	0.915124803
ATP6AP1	975.2940555	-0.092764859	0.149460971	-0.620662757	0.534821539	0.915124803
LINC00115	24.97254443	0.285392327	0.459831576	0.620645344	0.534832998	0.915124803
HTR3E	1.962152916	0.880809388	1.419328529	0.620581754	0.534874848	0.915124803
NUDT6	34.50816119	0.222110929	0.35792261	0.620555739	0.534891969	0.915124803
FBXL22	67.72959419	0.230444788	0.37136127	0.620540714	0.534901858	0.915124803
ENTPD3-AS1	17.46421523	-0.342112263	0.55133543	-0.620515651	0.534918353	0.915124803
BRICD5	54.02788904	0.259662475	0.418483296	0.620484683	0.534938735	0.915124803
ILF3-DT	153.7353429	0.133446508	0.215075468	0.62046364	0.534952585	0.915124803
MIR5194	7.000820234	0.636038895	1.025300025	0.620344171	0.53503122	0.915171466
GGACT	217.9582618	-0.120689195	0.194628221	-0.620101207	0.535191158	0.915274874
PFDN5	515.9658565	-0.123981289	0.19993877	-0.620096284	0.535194399	0.915274874
ZNF540	22.56793682	0.284157174	0.458308934	0.620012295	0.535249692	0.915281597
SNORD114-20	406.2852043	-0.137251322	0.221515628	-0.619601078	0.535520457	0.915656739
TRABD	652.5105097	0.103296134	0.166778494	0.619361233	0.535678414	0.915773861
ENO3	83.92115861	-0.172360668	0.278296877	-0.619341007	0.535691736	0.915773861
LOC101928495	2.958998445	-0.671146917	1.083944847	-0.619170725	0.535803895	0.915778122
CWC22	648.5892612	-0.0747533	0.12073269	-0.619163708	0.535808517	0.915778122
MKNK1-AS1	12.84004579	0.434666231	0.702178128	0.61902559	0.535899501	0.915778122
ZNF23	190.5633104	0.098635651	0.159340297	0.619025151	0.53589979	0.915778122
DIPK1B	66.43197078	0.249907866	0.403794754	0.618898249	0.535983392	0.915833162
C8orf37-AS1	2.299661977	-0.857802663	1.386354585	-0.618746944	0.536083079	0.915915673
MGLL	236.6099756	-0.390736139	0.63161758	-0.618627712	0.536161641	0.915962079
RIDA	281.5082238	-0.096194133	0.155526078	-0.618508063	0.536240484	0.916008956
KLF12	250.380574	-0.083374621	0.134839176	-0.61832639	0.536360209	0.916125652
SNHG8	388.5395053	0.0819728	0.132630793	0.618052554	0.536540697	0.916138631
GEMIN4	1267.594981	0.065774888	0.106429702	0.618012512	0.536567091	0.916138631
IRAK1BP1	87.02128997	-0.17848032	0.28882479	-0.617953606	0.536605921	0.916138631
GSC-DT	2.368470612	0.923743058	1.494877918	0.617938794	0.536615685	0.916138631
RRN3	1095.605587	0.078380479	0.126844674	0.617924874	0.536624862	0.916138631

JAG1	542.8943048	-0.121347962	0.196426576	-0.617777718	0.536721873	0.916216474
SNORD113-2	18.19250229	0.410592031	0.664854224	0.617567004	0.536860801	0.916365849
CATSPER3	10.3985278	-0.400194905	0.648136078	-0.617455067	0.53693461	0.916404056
PDE6C	11.22193818	0.407925389	0.660907678	0.617219928	0.537089673	0.916547627
EIF4ENIF1	296.9319746	-0.099600692	0.161382514	-0.617171522	0.537121598	0.916547627
STX8	86.79379751	0.198020842	0.320947418	0.616988424	0.537242361	0.916610045
PSME3IP1	781.7958657	-0.061654468	0.099946749	-0.61687317	0.537318385	0.916610045
WDR47	373.7239189	0.099195968	0.160864892	0.616641502	0.537471214	0.916610045
CAMK1D	277.780096	-0.24552357	0.398206925	-0.616572829	0.537516521	0.916610045
NUBPL	509.5463429	-0.090954327	0.147525267	-0.616533891	0.537542211	0.916610045
SELENOP	14.4488454	-0.359469288	0.583151441	-0.616425276	0.537613875	0.916610045
KIF1B	2882.876084	0.063489154	0.102998039	0.616411283	0.537623109	0.916610045
STAR	16.59079508	0.28920821	0.469197449	0.616389135	0.537637722	0.916610045
C8orf34	220.1284274	0.195144195	0.3166125	0.616350254	0.537663378	0.916610045
IFT27	58.99621359	-0.228203885	0.370258678	-0.616336358	0.537672547	0.916610045
SNRPA	695.2134809	-0.083020049	0.134736567	-0.616165687	0.537785172	0.916714346
LOC101928728	20.91144612	-0.26356208	0.427888408	-0.615959851	0.537921018	0.91674723
TXLNB	4.457307538	-0.557091755	0.904494776	-0.61591484	0.537950727	0.91674723
RAD51C	120.605184	-0.142780328	0.231822898	-0.615902611	0.537958798	0.91674723
ANKRD63	13.10660166	0.369600466	0.600318907	0.615673539	0.538110005	0.916810898
ITCH	2028.620611	0.06977592	0.113369717	0.615472295	0.53824286	0.916810898
ZNF648	2.508331671	0.795513008	1.292571719	0.615449802	0.538257711	0.916810898
GLG1	4934.787937	-0.100254036	0.162906487	-0.615408493	0.538284984	0.916810898
HOXB4	19.28991466	-0.373061354	0.606467848	-0.615137892	0.53846366	0.916810898
BACE2	368.3555417	-0.094851471	0.154212911	-0.615068285	0.538509626	0.916810898
KCNMB2	106.6850069	0.148754116	0.241895738	0.614951371	0.538586836	0.916810898
KIF23	1310.974318	0.076172773	0.123904924	0.614767929	0.538707992	0.916810898
ATAD3C	2.416672849	-0.808455356	1.31527643	-0.614665737	0.538775492	0.916810898
NEDD8-MDP1	32.22586389	0.312106885	0.508054354	0.614317902	0.539005275	0.916810898
CCDC90B	229.6228997	-0.114824806	0.186946151	-0.614213266	0.539074408	0.916810898
OPRD1	8.620299517	0.406575115	0.661944836	0.614212987	0.539074592	0.916810898
OR52I1	2.128160077	0.825609939	1.344408043	0.614106665	0.539144844	0.916810898
SMAP2	862.6165781	0.075036841	0.122191261	0.614093346	0.539153645	0.916810898
ABCC5-AS1	17.86746701	0.532416554	0.86701845	0.614077536	0.539164092	0.916810898
SNU13	1041.328814	-0.093147996	0.151689205	-0.614071361	0.539168172	0.916810898
MLIP	448.4788005	-0.146894346	0.239230114	-0.614029494	0.539195838	0.916810898
C14orf28	62.44074406	-0.178786241	0.291220581	-0.613920349	0.539267963	0.916810898
PSD4	7.98414636	-0.422270872	0.687834876	-0.613913146	0.539272723	0.916810898
SLC13A4	42.6294849	0.331352417	0.53974325	0.613907477	0.539276469	0.916810898
LIN7A	1676.603349	0.116778323	0.190250698	0.613812849	0.539339005	0.916810898
SUGT1	760.4251916	0.091898126	0.149732251	0.613749712	0.539380733	0.916810898
ADGRB3	225.1914984	0.118276723	0.192730402	0.613690012	0.53942019	0.916810898
ZNF295-AS1	9.574678965	0.485768993	0.791591926	0.613660875	0.539439448	0.916810898
CCDC39	274.8880215	0.231276643	0.376953813	0.613541064	0.539518639	0.916810898
HIF1AN	1006.657001	0.055760653	0.090900127	0.61342767	0.539593595	0.916810898
LOC102723704	5.289958469	0.602247874	0.981801286	0.613411169	0.539604503	0.916810898
PHF6	1138.737241	-0.079819975	0.13012665	-0.613402208	0.539610427	0.916810898
SNORD116-28	8.877831956	-0.723903131	1.180151709	-0.613398366	0.539612966	0.916810898
LOC105374727	2.448853662	0.782811925	1.276458344	0.613268681	0.539698698	0.916810898
TERC	201.9480098	-0.186263916	0.303761194	-0.61319194	0.539749434	0.916810898
RNF4	677.5198864	-0.074921946	0.122183767	-0.613190669	0.539750274	0.916810898
ZNF585A	148.3433555	0.185444593	0.302460344	0.613120354	0.539796763	0.916810898
ITGB7	3.984207666	0.648368308	1.057502851	0.613112587	0.539801898	0.916810898
LOC401127	2.818956695	-0.733125842	1.195830613	-0.613068301	0.539831179	0.916810898
C1RL-AS1	221.7616536	0.204518771	0.333625243	0.613019474	0.539863463	0.916810898
ETFBKMT	39.6276495	-0.222342234	0.362753814	-0.612928727	0.539923467	0.916810898
MAST3	298.8361394	0.123014513	0.200713272	0.612886789	0.539951199	0.916810898
SLC33A1	735.7054505	0.07105916	0.115975353	0.612709151	0.54006867	0.91692299
DNAJB2	310.6054869	0.134634137	0.219800424	0.612529015	0.540187807	0.91703789
MAIP1	243.8608112	0.09481428	0.154815912	0.612432396	0.540251713	0.917059014
ABAT	17.65965251	0.354699921	0.579278647	0.612313129	0.540330605	0.91707832
RCOR3	737.5661955	-0.10858301	0.17734799	-0.612259602	0.540366013	0.91707832
LYPLAL1-AS1	10.82448311	-0.442237717	0.722537132	-0.61206227	0.540496559	0.917143088
SNORD116-30	2.247632066	1.037292367	1.69479389	0.612046322	0.54050711	0.917143088
ZBTB26	280.0876427	-0.13401662	0.219060166	-0.611779963	0.540683349	0.917223309
GOLGA6L9	35.50614478	0.225420663	0.368484308	0.61175105	0.540702481	0.917223309
RNPEPL1	611.5735574	-0.105603388	0.172627473	-0.611741496	0.540708803	0.917223309
EXOSC8	316.3789969	-0.101396785	0.165784605	-0.611617613	0.540790782	0.917275056
ETNK1	908.8723144	0.084620434	0.138439556	0.611244624	0.541037645	0.917606437
DDX11L5	2.798104744	-0.872439537	1.427805324	-0.611035358	0.541176173	0.917711886
TMEM164	544.4239433	0.072494243	0.11865124	0.610985966	0.541208871	0.917711886
PRR26	55.80598972	0.592727338	0.970288683	0.610877308	0.541280809	0.917711886
KLC4	355.3359686	0.13098575	0.214446457	0.610808644	0.54132627	0.917711886
DDX3X	8402.22286	-0.073450251	0.120274472	-0.610688616	0.541405744	0.917711886
PPFIA2	27.7275717	0.233278772	0.381995892	0.610683981	0.541408813	0.917711886
ZNF544	725.2815752	0.163258901	0.267401929	0.610537481	0.541505822	0.917767154
USP49	355.7781423	0.105530862	0.17286801	0.610470739	0.541550021	0.917767154
ZNF26	424.0341209	0.169255589	0.277305213	0.610358481	0.541624365	0.917767154
NPTXR	60.75208728	0.219525201	0.359695011	0.610309274	0.541656955	0.917767154
TMEM237	444.2046907	0.071121625	0.116553061	0.610208127	0.541723947	0.917767154
UBE3A	3411.551142	-0.059197224	0.097025439	-0.610120657	0.541781884	0.917767154
DNAH12	1.998951718	0.857928052	1.406386822	0.610022818	0.541846693	0.917767154
LINC00412	4.132173638	0.598361807	0.981277756	0.609778224	0.542008728	0.917767154
LOC389906	200.1150925	0.098831142	0.162107685	0.609663525	0.542084722	0.917767154
PPCS	350.2320463	-0.127376592	0.208947242	-0.609611259	0.542119351	0.917767154
OR51M1	2.455824052	0.942429906	1.546359294	0.609450798	0.542225676	0.917767154
SLC16A13	86.37525783	0.174542931	0.286428384	0.609377214	0.542274437	0.917767154
MIR3677	8.693541664	-0.494896821	0.812267373	-0.609278223	0.542340039	0.917767154
LOC100130744	87.22397004	-0.201529223	0.330793813	-0.609229119	0.542372582	0.917767154
ECH1	704.4503599	-0.109842534	0.180304922	-0.609204302	0.542389029	0.917767154

WBP4	98.29197177	0.125872453	0.206653731	0.609098381	0.542459231	0.917767154
CASC1	2.621583221	0.742114443	1.218557179	0.609010767	0.542517302	0.917767154
ESYT2	1512.259193	-0.083599052	0.137271968	-0.609003083	0.542522395	0.917767154
PRRT3	133.833704	0.253911549	0.416973579	0.60893918	0.542564753	0.917767154
C17orf107	21.4600936	0.26844898	0.440854174	0.608929202	0.542571367	0.917767154
CIC	3317.428043	0.089780412	0.147442371	0.608918667	0.54257835	0.917767154
DDX17	9330.374347	0.061555433	0.10110234	0.608842813	0.542628633	0.917767154
CXXC1	642.7553043	-0.086559083	0.142170277	-0.608840925	0.542629884	0.917767154
FAM76B	219.187546	0.114007461	0.187275361	0.608769143	0.542677469	0.917767154
FRMD7	57.11025909	-0.226258292	0.371769583	-0.608598183	0.542790809	0.917871723
RNF5P1	2.750790356	-0.737407358	1.21210045	-0.608371491	0.542941115	0.918038777
MAGEA1	2.962682137	-0.626592416	1.030216049	-0.608214574	0.54304517	0.918127602
ZNHIT6	762.8717589	-0.065802736	0.108237308	-0.607948744	0.543221469	0.91826709
PRPF40A	1299.778375	-0.099098166	0.163026221	-0.607866422	0.543276071	0.91826709
LOC105370687	76.97932107	0.144408413	0.237616337	0.607737732	0.543361434	0.91826709
TUBA1A	1957.723861	-0.183284322	0.301597447	-0.607711782	0.543378647	0.91826709
ZNF271P	299.2592516	-0.09343573	0.153752618	-0.607701718	0.543385323	0.91826709
LINC01355	158.6998024	0.419956423	0.691174926	0.607597884	0.543454204	0.918296408
UQCRBP1	13.737128	-0.448911803	0.739016039	-0.607445278	0.543555448	0.9183804
MIB2	810.2383673	0.088900882	0.146432121	0.607113262	0.54377575	0.918628202
L3MBTL3	278.5396303	0.097144313	0.160021901	0.607068862	0.543805213	0.918628202
LRRC39	20.55621051	0.313153082	0.51600907	0.60687515	0.543933771	0.918758275
SGPP1	554.1584153	0.067845078	0.111815329	0.606760075	0.544010148	0.918758648
TXNDC11	473.9796902	0.080169838	0.132136587	0.606719457	0.544037107	0.918758648
CCDC117	1139.875815	0.100947834	0.166417949	0.606592225	0.544121561	0.918814197
GK3P	3.501158893	0.855962709	1.411608669	0.606373939	0.54426647	0.918947733
CNTNAP4	9.291042958	0.429796083	0.708949953	0.606243192	0.544353275	0.918947733
FLVCR1	470.0776478	0.161504223	0.26640308	0.606240073	0.544355346	0.918947733
TAF1C	450.4098927	0.143343221	0.236525901	0.606036043	0.54449082	0.918980915
ANKRD33B	226.2090095	-0.110390149	0.182152133	-0.606032704	0.544493037	0.918980915
DDX54	1947.777644	-0.069253529	0.114296684	-0.605910219	0.544574374	0.918980915
LINC01137	6.237934693	0.436913227	0.721098137	0.605899814	0.544581283	0.918980915
CDKN2B	448.0364264	0.106041036	0.175096249	0.605615691	0.54476998	0.91909111
NLRP3	2.180800308	1.0363032	1.711242589	0.605585208	0.544790227	0.91909111
TIMM8A	207.4843366	0.1523154	0.251557012	0.605490575	0.544853085	0.91909111
NDUFAF4P1	9.644963563	0.531308801	0.877833823	0.605249863	0.545012989	0.91909111
ZNF606	209.7594118	-0.096538072	0.159502879	-0.605243447	0.545017252	0.91909111
LOC101928069	18.1234879	0.34792366	0.574880255	0.605210663	0.545039032	0.91909111
DHX33	1546.459757	0.057007928	0.094203967	0.605154216	0.545076534	0.91909111
MIR8063	10.76054886	0.719715126	1.189327632	0.605144542	0.54508296	0.91909111
KIF13A	1076.775417	-0.103654697	0.171319578	-0.605037081	0.545154358	0.91909111
LHFPL1	8.612404034	-0.400012501	0.661150305	-0.605025057	0.545162348	0.91909111
LOX	1468.175074	0.314107311	0.519248214	0.60492709	0.545227443	0.919113899
IQCJ-SCHIP1-AS1	19.53370994	0.380520235	0.629324404	0.604648784	0.545412385	0.919338697
RAB5IF	96.25029477	-0.166796791	0.276053589	-0.604218882	0.545698129	0.919662039
NDC1	2084.434873	0.108972517	0.180356893	0.604204891	0.54570743	0.919662039
RNVU1-19	13.49418246	-0.493819535	0.81756019	-0.604016122	0.545832924	0.919786544
LIN54	402.8586601	-0.084192002	0.139413515	-0.603901295	0.545909268	0.919802335
GSTM1	4.096771685	-0.773643591	1.281227957	-0.603829776	0.545956821	0.919802335
DDX10	477.9055832	-0.07282303	0.120648223	-0.603598034	0.546110921	0.919802335
MIR561	4.200810002	0.711098915	1.178129148	0.603583161	0.546120811	0.919802335
HPCAL4	2.409279545	-0.726510397	1.203823833	-0.603502254	0.546174616	0.919802335
ADAR	4482.549574	-0.056000809	0.092794176	-0.603494863	0.546179532	0.919802335
GNS	3985.839495	-0.09826198	0.162837052	-0.603437477	0.546217697	0.919802335
CRIM1-DT	5.828214161	0.500692611	0.829854718	0.603349719	0.546276064	0.919802335
NDUFV2-AS1	19.94818879	0.326690388	0.541597254	0.603198013	0.546376969	0.919802335
TMC5	3.952479782	-0.779421209	1.292204293	-0.603171815	0.546394396	0.919802335
TRIM59	23.30615845	0.42372867	0.702541965	0.603136455	0.546417916	0.919802335
MIR548U	3.826974687	1.052574887	1.745359167	0.60307065	0.546461691	0.919802335
CENPI	369.417927	0.140672589	0.233298345	0.602972937	0.546526693	0.919824865
DENND6A	496.8289789	0.088943294	0.147529538	0.60288465	0.546585428	0.919828235
RUSC1	502.9162162	0.078413157	0.130102042	0.602705047	0.546704924	0.919828235
WASF3	122.8528286	0.206162523	0.342072869	0.602685983	0.546717608	0.919828235
COL4A3	7.815995232	-0.492984146	0.818014267	-0.602659594	0.546735167	0.919828235
ZBTB45	190.2862547	0.094888308	0.157567098	0.602208896	0.547035095	0.920133538
PKNOX1	443.7602955	-0.076514816	0.127064874	-0.602171267	0.54706014	0.920133538
SECISBP2	450.805951	-0.1100116	0.18270712	-0.602119939	0.547094303	0.920133538
TAF11	477.8903841	0.083567335	0.138798518	0.602076563	0.547123174	0.920133538
PPFIA2-AS1	2.347024043	0.98289998	1.632826735	0.601962204	0.547199297	0.920174716
TRMT11	556.8756914	0.178502678	0.296653112	0.601721912	0.547359262	0.920352187
KANK3	7.021814589	0.489262012	0.813422216	0.601485923	0.547516385	0.920352187
MRPL10	590.0702382	-0.113046178	0.18795821	-0.601443152	0.547544865	0.920352187
BIN3-IT1	27.57552452	0.344085798	0.572107455	0.601435613	0.547549884	0.920352187
TTC7B	481.479544	0.090762004	0.150913897	0.601415813	0.547563069	0.920352187
NDUFAB1	392.5734062	0.102920274	0.171160595	0.601308229	0.54763471	0.92038579
RXYLT1	291.6162756	0.097378168	0.16199878	0.601104326	0.547770502	0.920527193
ZNF300P1	2.362329632	-0.828221587	1.378017403	-0.60102404	0.547823974	0.920530243
CFAP99	2.585762967	-0.71058521	1.182535164	-0.600899856	0.547906689	0.920582425
SNORD114-2	6.330007898	0.479739518	0.798600208	0.600725511	0.548022825	0.920690745
CWF19L1	368.5595583	-0.083513533	0.139041482	-0.600637538	0.54808143	0.920702402
MOV10	1011.393246	-0.090020099	0.149951554	-0.600327883	0.54828774	0.920962157
TIPARP-AS1	18.12179763	-0.294173946	0.490180021	-0.600134508	0.548416597	0.921014254
GSDMD	323.1493768	-0.100163324	0.166922866	-0.600057539	0.548467889	0.921014254
RHOC	964.3809379	-0.102139912	0.170219382	-0.600048657	0.548473808	0.921014254
RAB3A	18.69373196	0.290666471	0.484502531	0.599927664	0.548554445	0.921062867
FSIP2-AS1	4.252428072	0.529643989	0.883414188	0.599542091	0.54881145	0.921304606
RIC8A	1159.328894	-0.067282813	0.112225302	-0.599533368	0.548817266	0.921304606
DEPDC1-AS1	8.206709439	0.431908796	0.72047362	0.599478988	0.548853518	0.921304606
MIR4288	2.304562721	0.819962716	1.368039856	0.599370488	0.548925852	0.921316367
PSMD11	2571.207194	0.092891543	0.154996612	0.59931338	0.548963927	0.921316367

ADAM15	1281.946176	-0.056312938	0.093997752	-0.599088132	0.549114115	0.921408328
HRNR	139.1238437	0.313836582	0.523942718	0.598990255	0.549179383	0.921408328
TRIM4	403.1860647	-0.131982166	0.220414243	-0.59879146	0.549311958	0.921408328
CHGA	3.648846362	0.560922313	0.936912408	0.598692373	0.549378044	0.921408328
PNPLA7	34.88261273	-0.220694042	0.368664716	-0.598630767	0.549419134	0.921408328
GEN1	667.5809854	0.165756384	0.276901897	0.598610501	0.549432652	0.921408328
SEC31A	3338.130745	-0.096292146	0.160882049	-0.598526352	0.549488781	0.921408328
GIPC3	7.554935434	-0.427207823	0.71397794	-0.598348771	0.549607241	0.921408328
OR3A1	4.688250326	-0.717722982	1.200199461	-0.598003086	0.549837874	0.921408328
EID2	126.5463024	-0.121974808	0.203999753	-0.59791645	0.549895683	0.921408328
PCSK5	214.2738603	-0.155705385	0.260474315	-0.597776351	0.549989172	0.921408328
PSD2	2.245354165	0.813279914	1.360819798	0.597639684	0.550080379	0.921408328
CSNK1D	1639.187054	-0.052981261	0.08865122	-0.597637133	0.550082081	0.921408328
LOC100507377	104.7383431	0.246162202	0.411997869	0.597484165	0.550184176	0.921408328
PDGFD	160.4121659	-0.211347429	0.353735683	-0.597472745	0.550191798	0.921408328
FGD1	459.6860228	0.072833408	0.121918422	0.597394614	0.550243949	0.921408328
PPP1R35	101.8711892	-0.126732748	0.212217077	-0.597184499	0.550384207	0.921408328
LINC02175	17.09637394	0.275124135	0.460856383	0.596984537	0.550517704	0.921408328
SLC10A1	36.61207357	0.448634401	0.75152244	0.596967405	0.550529143	0.921408328
USP13	2871.765753	0.04483984	0.075114736	0.59695131	0.550539889	0.921408328
IFI44L	98.19703722	0.253046461	0.423923202	0.596915809	0.550563592	0.921408328
CBX8	81.17516512	-0.178217647	0.298592339	-0.59685941	0.550601249	0.921408328
MIR380	2.884002439	-0.7960565	1.333805856	-0.596830863	0.55062031	0.921408328
AVPI1	58.47007788	-0.18626837	0.312129075	-0.596767124	0.550662871	0.921408328
ZNF408	84.14733278	0.170160225	0.285194458	0.596646325	0.550743535	0.921408328
CBFB	1342.320464	-0.067300095	0.112799348	-0.596635495	0.550750768	0.921408328
CHMP3	37.92074428	-0.204061117	0.342027455	-0.596622036	0.550759755	0.921408328
LOC100130987	14.54511777	-0.312564102	0.523925297	-0.596581429	0.550786873	0.921408328
LRP5L	146.2327369	0.218892255	0.366914621	0.596575451	0.550790865	0.921408328
CCN1	1794.372783	0.40874954	0.68535324	0.596407103	0.550903297	0.921408328
EGLN1	602.247679	-0.089039214	0.149299489	-0.596379902	0.550921464	0.921408328
CAMLG	526.307702	-0.169228506	0.283766287	-0.596365789	0.55093089	0.921408328
CDC40	592.187528	-0.069899949	0.117235985	-0.596232884	0.551019661	0.921408328
TAX1BP3	75.08664601	-0.17578721	0.294867718	-0.596156172	0.551070902	0.921408328
RBM15	1003.945876	0.112583046	0.188856117	0.596131318	0.551087503	0.921408328
CCNI	4220.584833	-0.130931503	0.219659389	-0.596066043	0.551131108	0.921408328
NEO1	3593.911891	-0.101667029	0.170570276	-0.596041884	0.551147247	0.921408328
LOC100133091	92.00131824	-0.232103597	0.389465699	-0.595953888	0.551206032	0.921408328
SELPLG	3.159753007	-0.758365495	1.27255821	-0.595937764	0.551216804	0.921408328
YTHDC2	690.5627862	0.115450493	0.193730926	0.595932179	0.551220535	0.921408328
LRRN3	80.75621014	0.175973984	0.295304889	0.595906098	0.551237959	0.921408328
FBXO6	25.25522858	0.243282841	0.408257612	0.595905218	0.551238547	0.921408328
BEAN1	1.955482424	1.099308351	1.844786926	0.595899903	0.551242098	0.921408328
PRKX-AS1	3.085343908	-0.899852054	1.510593775	-0.595694268	0.551379488	0.921551536
LRRC75B	41.94040767	-0.214945767	0.361002951	-0.595412768	0.551567593	0.921565579
NCOA6	858.059512	0.076456495	0.128418796	0.595368416	0.551597233	0.921565579
SYNM	418.9642451	0.076834104	0.129122021	0.595050355	0.551809812	0.921565579
FAM83H	37.9851231	-0.22855807	0.38412375	-0.595011556	0.551835746	0.921565579
ZNF573	114.8089195	0.16475685	0.276970118	0.594854242	0.551940906	0.921565579
PCDHB2	306.6063201	0.09568567	0.160861743	0.594831741	0.551955948	0.921565579
NBL1	80.4096643	0.221264065	0.372109018	0.594621616	0.552096428	0.921565579
STRCP1	2.944462908	0.715605488	1.203541805	0.594582993	0.552122251	0.921565579
NOP58	1096.44956	-0.139221734	0.234164603	-0.59454645	0.552146684	0.921565579
CSAG1	165.5823986	-0.134819393	0.22676158	-0.594542485	0.552149336	0.921565579
EDNRB	13.4200998	0.428114202	0.720135927	0.594490826	0.552183876	0.921565579
KLHDC1	23.25009721	-0.274056355	0.461024919	-0.5944502	0.552211041	0.921565579
MAGEC1	61.57221758	-0.247610225	0.416542455	-0.594441748	0.552216692	0.921565579
PRG4	14.12212865	-0.470473423	0.791533598	-0.594382127	0.55225656	0.921565579
RPL41	1711.977728	-0.152864307	0.257256881	-0.594208818	0.552372456	0.921565579
PLEKHG4B	302.039291	0.15597758	0.262518362	0.594158744	0.552405943	0.921565579
MAP2K5	379.8922438	-0.108097169	0.181967527	-0.594046478	0.552481027	0.921565579
RPL28	3102.822358	-0.140625217	0.236787319	-0.593888294	0.552586829	0.921565579
MED20	509.1626186	-0.084327924	0.142002779	-0.593846999	0.552614451	0.921565579
THRSP	2.11372355	1.035027036	1.743404845	0.593681404	0.552725223	0.921565579
MST1	114.9997113	0.16339364	0.275227094	0.593668443	0.552733894	0.921565579
LHX2	24.20139174	-0.226744754	0.381991912	-0.593585221	0.552789568	0.921565579
FTX	105.601842	0.271320029	0.457121124	0.593540782	0.552819299	0.921565579
TMEM150C	20.5115709	0.256738553	0.432715192	0.593319943	0.552967054	0.921565579
SNORA77B	11.8136089	-0.407837885	0.687508573	-0.593211344	0.553039722	0.921565579
OSTF1	162.2348016	0.138993426	0.234318384	0.593181907	0.55305942	0.921565579
BLOC1S5	7.639234975	-0.403639324	0.680544484	-0.59311233	0.55310598	0.921565579
TEX10	846.216313	-0.124707459	0.210292414	-0.593019295	0.55316824	0.921565579
VXN	4.444760884	0.625481587	1.05521841	0.592750829	0.553347919	0.921565579
FBXO43	149.9266123	0.179691927	0.303149828	0.592749559	0.55334877	0.921565579
CDH12	870.0310012	-0.123561575	0.208467342	-0.592714299	0.55337237	0.921565579
UBE3C	2305.272938	0.060402402	0.101918166	0.592655892	0.553411466	0.921565579
LILRB3	9.936832167	0.553458741	0.933909917	0.592625403	0.553431874	0.921565579
LINC01138	36.61079245	0.188512957	0.318124777	0.592575526	0.553465262	0.921565579
CD70	74.28969657	0.216593999	0.365576446	0.59247252	0.553534217	0.921565579
ARHGAP31-AS1	2.545323438	-0.834677125	1.408833604	-0.592459693	0.553542804	0.921565579
RPGRIP1	6.378344168	-0.477083586	0.80529522	-0.592433152	0.553560572	0.921565579
PTBP3	2972.674725	0.080454921	0.135817977	0.592373133	0.553600754	0.921565579
COMMD6	368.1044909	0.119854411	0.202333852	0.592359657	0.553609775	0.921565579
KCNK2	414.8470378	-0.184805181	0.311990403	-0.592342518	0.55362125	0.921565579
PIWIL2	17.35166975	0.49540941	0.836364973	0.592336391	0.553625352	0.921565579
MAP1LC3C	3.072118759	0.865151779	1.460578909	0.592334843	0.553626388	0.921565579
RIT1	362.5376057	0.09894333	0.167040746	0.592330509	0.55362929	0.921565579
LINC02610	2.112072984	-0.761470376	1.28576073	-0.592233344	0.553694344	0.921565579
GLTPD2	22.40240955	0.274106443	0.462859408	0.59220238	0.553715076	0.921565579
RNF213	3664.650016	0.119820569	0.202389863	0.592028509	0.553831498	0.921627788

HYDIN	5.789443196	-0.457132554	0.772193698	-0.591992081	0.553855891	0.921627788
SCARNA8	2.346075456	0.937974712	1.585149039	0.591726512	0.55403374	0.921714162
RPL37A	1870.386365	-0.078336177	0.132389691	-0.591709044	0.554045439	0.921714162
TRABD2A	827.7417019	0.126658283	0.214098066	0.591590036	0.554125148	0.921714162
ZNF420	158.0954894	0.121789596	0.205894796	0.591513716	0.554176268	0.921714162
DPH6-DT	10.46873195	0.416783138	0.704727828	0.591410075	0.554245692	0.921714162
LOC101929882	6.036566326	0.457046764	0.772916707	0.591327319	0.554301128	0.921714162
SPCS3	2395.345379	-0.069954466	0.118305378	-0.59130419	0.554316623	0.921714162
ATF7IP2	35.94246824	0.249770336	0.422440112	0.591256201	0.554348771	0.921714162
UBE3B	1552.290412	0.075579496	0.127856132	0.591129223	0.554433841	0.921714162
HDAC6	676.1317167	0.095187168	0.161034802	0.591096873	0.554455515	0.921714162
PPP1R8	657.4549463	-0.069966102	0.118375715	-0.59105114	0.554486156	0.921714162
IDH3A	372.9336788	-0.091890779	0.155486717	-0.590987971	0.554528481	0.921714162
CHMP2B	696.4922072	-0.120639002	0.204166166	-0.590886356	0.554596569	0.921741359
YBX3P1	2.495442095	-0.794793079	1.345850284	-0.590550887	0.55482138	0.922029002
LOC100130964	6.211073467	-0.515050181	0.872285007	-0.59046089	0.554881699	0.922043256
SELENOF	1890.744139	-0.091818504	0.155550021	-0.590282812	0.555001061	0.922071598
TTLL3	83.33780366	0.156668261	0.265438207	0.590224984	0.555039824	0.922071598
TMEM50A	957.2547481	-0.087316518	0.14794298	-0.590203858	0.555053986	0.922071598
KLHL12	1044.805434	-0.082046975	0.139059779	-0.590012265	0.555182427	0.922198998
SMC4	3200.955038	-0.093432982	0.158453645	-0.589654987	0.555421979	0.92251092
PDF	81.2378021	-0.157605737	0.267409268	-0.589380235	0.555606232	0.922546509
TINAG	11.79131118	-0.380485459	0.645686744	-0.589272526	0.555678472	0.922546509
ZBTB20	663.4730529	-0.083308459	0.141381283	-0.589246729	0.555695774	0.922546509
WNK4	22.02079174	0.278411945	0.47249007	0.589244013	0.555697596	0.922546509
PTCD1	15.85620946	0.329048946	0.558432193	0.589237064	0.555702257	0.922546509
FAM110B	232.6992084	-0.111079877	0.188569499	-0.58906598	0.555817014	0.922575918
TMEM69	350.8753049	-0.08525732	0.144749183	-0.589000352	0.555861038	0.922575918
MTFMT	250.3894865	-0.085430887	0.145054132	-0.588958658	0.555889007	0.922575918
SEMA3F	3.546144575	-0.674583657	1.14571835	-0.58878664	0.556004409	0.922575918
NUP160	2607.438886	-0.050609018	0.085955887	-0.588778964	0.556009559	0.922575918
FAM200A	88.87748783	-0.128293139	0.217908557	-0.588747597	0.556030604	0.922575918
LMNTD2	10.87270691	0.399511236	0.678778953	0.588573399	0.556147483	0.922683934
ZNF18	176.0585249	0.098625804	0.167623335	0.588377528	0.556278918	0.922816078
GATD3A	52.28772595	0.254894191	0.433359177	0.588182285	0.556409947	0.922947524
RCAN3	544.0163201	-0.072100532	0.122610792	-0.588043934	0.556502804	0.922977915
ZSWIM8	2066.017139	-0.078210199	0.133016281	-0.587974634	0.556549319	0.922977915
MIR2682	3.962972999	-0.662884308	1.127912682	-0.587708888	0.556727709	0.922977915
GTF2F2	380.9034981	-0.097040086	0.165127608	-0.587667242	0.556755667	0.922977915
FDXACB1	98.71584934	0.138659795	0.235953272	0.587657861	0.556761965	0.922977915
NLRP14	6.0135056	0.537950671	0.915415709	0.587657242	0.556762381	0.922977915
DNAH10	35.16396785	0.285076388	0.485188882	0.587557545	0.556829315	0.922977915
C2CD6	44.68953017	0.203446519	0.346283523	0.587514294	0.556858353	0.922977915
DENND6B	48.44471856	-0.192174487	0.32712746	-0.587460579	0.556894419	0.922977915
TMEM126B	458.8474451	0.085174325	0.145013198	0.587355675	0.556964856	0.92300881
HTRA4	9.270199694	0.424061515	0.722577555	0.586873356	0.557288765	0.923422114
HAVCR1P1	4.533847682	0.598877923	1.020530533	0.586829991	0.557317892	0.923422114
DENND2D	22.10270115	-0.238501703	0.406540145	-0.58666212	0.557430653	0.923496281
RDH14	84.25094767	0.139543134	0.237895995	0.586572021	0.557491178	0.923496281
TRAPPC4	173.1568572	0.10289055	0.175421913	0.586531909	0.557518125	0.923496281
SNORD84	68.86380049	0.202959629	0.346142914	0.586346334	0.5576428	0.923533127
LINC01311	40.33533881	-0.224449933	0.382813457	-0.586316726	0.557662693	0.923533127
OAZ3	9.661458992	-0.411484912	0.701872429	-0.586267383	0.557695846	0.923533127
COL1A2-AS1	6.690021834	0.470970548	0.803475295	0.586166806	0.557763426	0.923538847
ZSWIM9	234.765828	0.117170471	0.199912771	0.586107983	0.557802952	0.923538847
CUTA	920.9940808	-0.123219321	0.210345412	-0.585795143	0.558013188	0.923754306
LOC105370489	3.375596214	0.752626982	1.284872465	0.585760067	0.558036762	0.923754306
CARS-AS1	31.49828536	-0.298088079	0.509441884	-0.585126761	0.558462486	0.92434377
IKBKG	181.5538052	0.10375852	0.177388956	0.584920966	0.55860086	0.92434377
MINDY3	240.6256102	-0.117153661	0.200299773	-0.584891629	0.558620587	0.92434377
LOC101928371	5.748362972	0.505595875	0.864693805	0.584710879	0.558742138	0.92434377
ATP8B1	76.91040811	0.233280009	0.398990778	0.58467519	0.55876614	0.92434377
DPH3	285.1307294	-0.096532463	0.165111367	-0.58465062	0.558782664	0.92434377
CKAP2L	432.7669303	-0.081779283	0.139894586	-0.584577898	0.558831573	0.92434377
SNORA116	3.04703155	0.92315939	1.579288256	0.584541414	0.558856111	0.92434377
LINC00898	41.97313632	0.205390799	0.351436782	0.584431708	0.558929899	0.92434377
STX12	641.0733349	0.11534719	0.197368634	0.584425133	0.558934322	0.92434377
ELOA2	12.56754131	-0.480835686	0.822810787	-0.584381845	0.558963438	0.92434377
C9orf40	255.1644358	-0.106687932	0.182630644	-0.584173223	0.559103775	0.924490049
GALT	201.2122889	0.128515933	0.220028241	0.58408835	0.559160872	0.924498677
SLC9B1	4.228221401	0.635781335	1.088989343	0.583826958	0.559336739	0.924596485
MAGEA6	396.0559901	0.103852564	0.177950195	0.583604666	0.55948632	0.924596485
FAM86C1	113.0286178	-0.123407031	0.211473109	-0.583558978	0.559517067	0.924596485
CLCA4-AS1	4.842004656	-0.471244726	0.807594676	-0.583516385	0.55954573	0.924596485
LHX9	86.09194026	0.138400222	0.237220055	0.583425472	0.559606915	0.924596485
CAMKK2	2058.716652	-0.063050165	0.108109856	-0.583204597	0.559755577	0.924596485
STIL	545.2218189	-0.078373234	0.134397768	-0.583143864	0.559796457	0.924596485
SERAC1	95.94593086	0.153102309	0.262580346	0.583068427	0.559847237	0.924596485
WRNIP1	1089.611748	-0.07117686	0.122079473	-0.583037086	0.559868335	0.924596485
DHX38	1140.662407	-0.062326441	0.106902276	-0.583022586	0.559878096	0.924596485
PSMC3IP	73.02405054	0.231302376	0.396778754	0.582950507	0.559926618	0.924596485
DCAF13P3	23.83720995	0.33685697	0.577999825	0.582797702	0.560029492	0.924596485
TRMT2B	162.9429991	-0.110973743	0.19043776	-0.582729721	0.560075262	0.924596485
NFYA	963.7279608	0.091996199	0.157908563	0.582591578	0.560168277	0.924596485
MVB12A	276.6294745	-0.126742091	0.217570309	-0.582533949	0.560207081	0.924596485
SPIN4	219.7425392	0.089308962	0.153313517	0.582525033	0.560213085	0.924596485
ZNF202	259.9305525	0.117302026	0.201370763	0.582517662	0.560218048	0.924596485
VPS45	580.7882849	-0.066008571	0.113316524	-0.58251497	0.560219861	0.924596485
DGKI	75.99381176	-0.161685581	0.277671897	-0.582290045	0.56037133	0.924596485
SRPRA	2698.267013	0.077133377	0.132481016	0.582222108	0.560417084	0.924596485

SETD6	234.9599943	0.163262088	0.280464772	0.582112636	0.560490815	0.924596485
SLC26A10	4.103643609	0.581908086	0.999654395	0.582109267	0.560493084	0.924596485
TAF13	185.0834343	0.097233704	0.167043226	0.58208708	0.560508027	0.924596485
ECHDC3	4.136670625	-0.567731488	0.975450903	-0.582019543	0.560553517	0.924596485
TOR1AIP1	1112.849782	0.090039986	0.154709804	0.581992757	0.560571559	0.924596485
ZNF319	104.2424578	0.127903861	0.219800624	0.581908544	0.560628285	0.924596485
ZFP37	162.1241101	0.132696895	0.228056717	0.58185918	0.560661538	0.924596485
ZNF322	416.6122263	0.081661666	0.140350138	0.581842434	0.560672818	0.924596485
COL17A1	16.5186348	0.371962679	0.640004824	0.581187305	0.561114223	0.924795215
PUM2	3755.030857	-0.057687176	0.09929483	-0.580968576	0.561261633	0.924795215
SNX27	1554.337399	0.055332876	0.09524473	0.580954722	0.56127097	0.924795215
NUDCD3	989.9770849	-0.076338625	0.131406275	-0.580935914	0.561283647	0.924795215
MIR635	9.876031571	-0.385653277	0.663883785	-0.5809048	0.561304617	0.924795215
KRT86	5.005499627	-0.966232692	1.663424712	-0.580869507	0.561328405	0.924795215
OLMALINC	47.93946173	0.276818115	0.476581851	0.580840655	0.561347852	0.924795215
RBM27	1360.498979	0.053031421	0.091303624	0.580824924	0.561358456	0.924795215
F5	5.463162865	0.50180055	0.86411967	0.580707241	0.561437781	0.924795215
ACOX3	228.5937539	0.086196092	0.14845373	0.580625978	0.56149256	0.924795215
HMX2	44.04377775	-0.168741537	0.290625306	-0.58061543	0.561499671	0.924795215
UEVLD	371.4850402	-0.106722552	0.183818492	-0.580586594	0.56151911	0.924795215
ABCG2	15.65028764	-0.349383801	0.601821134	-0.580544253	0.561547653	0.924795215
MTM1	90.08770872	0.149047712	0.256779853	0.580449403	0.561611598	0.924795215
GAK	934.1996293	0.066012411	0.113732958	0.580415842	0.561634225	0.924795215
FKBP2	4.728824506	0.700041356	1.20625237	0.580344025	0.561682644	0.924795215
SLC46A2	4.072357123	-0.827762265	1.426688488	-0.580198321	0.561780886	0.924795215
NKPD1	8.872867579	0.39169903	0.675121253	0.580190637	0.561786067	0.924795215
PP2D1	16.44698999	0.299726745	0.516691186	0.580088752	0.561854769	0.924795215
FAM168A	1199.895909	-0.106399951	0.183428903	-0.580060992	0.561873488	0.924795215
FAM180A	53.86838335	-0.434060542	0.748320045	-0.580046659	0.561883153	0.924795215
ANKZF1	599.4327372	0.075684011	0.130528801	0.57982614	0.562031868	0.92494637
ALG12	427.3613403	0.090337882	0.155820372	0.579756552	0.562078801	0.92494637
TPRKB	206.7347551	-0.091850496	0.158455405	-0.579661491	0.562142917	0.924966462
LINC02476	160.9347472	0.216546898	0.373676805	0.579503184	0.562249699	0.925056748
PTGR1	436.379119	-0.100928772	0.174218237	-0.579323807	0.562370705	0.925105876
ADAMTS13	134.3620155	-0.162207128	0.280009791	-0.579290914	0.562392895	0.925105876
LOC730183	14.95947724	0.308519879	0.532639735	0.579228058	0.562435301	0.925105876
SNORD36A	3.684431867	0.62082952	1.072052526	0.57910364	0.562519244	0.925119747
PPP2R3C	513.9945084	-0.069139557	0.119400568	-0.57905551	0.562551718	0.925119747
LIPT1	97.59005657	-0.138356131	0.238981367	-0.578941081	0.56262893	0.925119747
ABI1	529.8149189	-0.123435445	0.213221254	-0.578907791	0.562651393	0.925119747
LOC392196	2.473976073	-0.787542376	1.361342825	-0.578504078	0.562923845	0.925482324
RTEL1-TNFRSF6B	2.919377531	-0.624760916	1.080113624	-0.578421476	0.562979598	0.925488601
FKBP5	340.0754706	-0.152046764	0.262951342	-0.578231559	0.563107795	0.925596631
CDKN1A	318.5670295	-0.343203418	0.593632714	-0.578141013	0.563168919	0.925596631
MCPH1-AS1	55.76160695	0.167043198	0.288956851	0.578090458	0.563203049	0.925596631
LOC101929452	14.64720494	0.353874109	0.612480707	0.577771846	0.563418165	0.925596631
ALKBH6	101.3205067	0.138369006	0.239535658	0.577655148	0.563496966	0.925596631
MAMDC2-AS1	2.272690934	0.82470773	1.427775903	0.577617068	0.563522681	0.925596631
ANKS3	312.3120852	0.100043386	0.173250377	0.577449742	0.56363568	0.925596631
ZNF90	6.541006422	-0.469917275	0.814308203	-0.577075453	0.563888486	0.925596631
TALAM1	16.66490753	0.392209938	0.679699166	0.577034602	0.563916081	0.925596631
TUBA3C	17.01826649	0.271799026	0.471046844	0.577010609	0.563932289	0.925596631
TFR2	8.125632806	0.435413492	0.754702425	0.576934003	0.563984039	0.925596631
GIPR	48.18055634	0.191749971	0.33236152	0.576931924	0.563985444	0.925596631
GDPD1	50.04089414	-0.185450192	0.321442103	-0.576931867	0.563985482	0.925596631
HAL	10.31059697	0.380160081	0.658976068	0.576895124	0.564010304	0.925596631
LOC284950	10.40387786	0.404279586	0.700873765	0.576822256	0.564059533	0.925596631
NPIPB13	3.767208519	-0.892519175	1.547329826	-0.576812494	0.564066129	0.925596631
KDM3B	1947.285034	-0.06327898	0.109720789	-0.576727348	0.564123655	0.925596631
IZUMO4	11.93684087	-0.335325657	0.581429905	-0.576725851	0.564124667	0.925596631
CD151	2593.870911	-0.087657959	0.151996959	-0.576708637	0.564136297	0.925596631
COL10A1	62.98596298	-0.251085001	0.435493479	-0.576552839	0.564241566	0.925596631
LINC02228	6.825893536	0.477947403	0.829258057	0.576355453	0.564374948	0.925596631
SNORD116-8	4.310700776	0.682581043	1.184320757	0.576348121	0.564379903	0.925596631
PPIL2	574.8096817	0.112150593	0.194588645	0.576347056	0.564380623	0.925596631
SFTPD	6.669334844	-0.618558012	1.073365091	-0.576279233	0.564426457	0.925596631
MFSD13A	39.53970647	-0.191385004	0.332131466	-0.576232676	0.564457922	0.925596631
MED13L	6772.678572	0.076348449	0.132498573	0.576220917	0.564465869	0.925596631
GUCY1B1	260.9744932	0.116435774	0.202137653	0.576022194	0.56460018	0.925596631
UNC5B-AS1	6.466299148	-0.532883701	0.92528169	-0.575915104	0.564672566	0.925596631
USP22	25033.43912	-0.062518266	0.108564979	-0.57586034	0.564709585	0.925596631
THCAT158	2.679595602	0.716999059	1.245284523	0.575771276	0.564769791	0.925596631
U2AF2	2598.471403	-0.092942987	0.161465519	-0.575621266	0.564871203	0.925596631
BMERB1	165.8614856	-0.250374848	0.435089068	-0.575456537	0.564982577	0.925596631
CSTF2T	270.5985418	-0.078158076	0.135844832	-0.575348171	0.565055849	0.925596631
SPEG	1144.435494	0.127875818	0.22233226	0.57515638	0.56518554	0.925596631
TMEM186	131.7324935	0.108942038	0.189414365	0.575151936	0.565188546	0.925596631
MTG2	313.1832985	-0.11058575	0.192285149	-0.575113321	0.565214659	0.925596631
FAM78A	22.43448124	-0.240384239	0.418038943	-0.57502834	0.56527213	0.925596631
DNAJC19	384.8209647	-0.0939337	0.163381536	-0.57493461	0.565335522	0.925596631
HSF2	745.1331869	0.063007661	0.109591613	0.574931412	0.565337685	0.925596631
FDX1	255.5766512	-0.082724241	0.143887842	-0.574921685	0.565344263	0.925596631
TTC23	421.8026133	-0.077434219	0.134689429	-0.574909401	0.565352572	0.925596631
SKAP2	594.7871444	-0.097967073	0.170410771	-0.5748878	0.565367181	0.925596631
NSD1	2339.22205	0.070371787	0.122447483	0.574709954	0.565487474	0.925596631
SNORD69	3.984210714	0.646961785	1.125807918	0.574664448	0.565518256	0.925596631
KDM5A	2714.136974	-0.044793743	0.077975272	-0.574460872	0.565655971	0.925596631
ZNF542P	246.425831	-0.096607272	0.168220003	-0.574291226	0.565770746	0.925596631
AMY2B	67.06323208	0.209970371	0.365639676	0.574254887	0.565795332	0.925596631
SETD4	184.4105853	0.192012165	0.334387439	0.574220629	0.565818511	0.925596631

TRIM52	267.4709611	0.13951119	0.242965897	0.574200707	0.565831991	0.925596631
NREP	994.0589429	-0.070210059	0.122312794	-0.574020563	0.565953887	0.925596631
SNORD121B	2.109658789	0.85250825	1.48557959	0.573855656	0.566065483	0.925596631
UHRF1	1520.069933	-0.1396405	0.24336063	-0.573800701	0.566102674	0.925596631
MIR3074	12.82805465	0.323636076	0.56404956	0.573772411	0.566121821	0.925596631
ZNF575	13.18227502	-0.44118534	0.768966413	-0.573738115	0.566145032	0.925596631
NPIPA5	2.620049357	-0.764824687	1.333662056	-0.573477129	0.56632168	0.925596631
SHLD2	514.8188465	0.080739601	0.140806752	0.573407167	0.566369039	0.925596631
SEC14L1P1	33.71027045	-0.263584925	0.459696386	-0.57338916	0.566381228	0.925596631
SLC38A1	4.530978147	-0.637972323	1.112710552	-0.573349755	0.566407903	0.925596631
S100PBP	855.2110331	0.081462116	0.142152019	0.573063375	0.566601785	0.925596631
PCGF1	175.4272635	-0.10494275	0.183142091	-0.573012733	0.566636073	0.925596631
DARS2	2582.623255	-0.068051539	0.118766249	-0.572987186	0.56665337	0.925596631
HSPA4	2600.543839	0.086475306	0.150931108	0.572945547	0.566681564	0.925596631
ARMH4	105.514612	0.234466388	0.409331404	0.572803323	0.566777869	0.925596631
PET117	61.24654901	-0.144910404	0.253003512	-0.572760444	0.566806905	0.925596631
GNPAT	1301.193425	-0.063870001	0.11151689	-0.572738356	0.566821863	0.925596631
GNL2	856.1178056	-0.066753741	0.116560679	-0.572695195	0.566851091	0.925596631
COL4A2-AS2	9.273186616	0.470250513	0.821151008	0.572672393	0.566866533	0.925596631
FER	622.1931556	0.061506178	0.107407551	0.572642959	0.566886467	0.925596631
BRD3	970.1359042	-0.074547009	0.130217357	-0.572481356	0.566995913	0.925596631
OPA1	6314.118645	0.05431308	0.094877449	0.572455099	0.567013697	0.925596631
HOXC4	44.78272963	-0.205419901	0.358856798	-0.572428619	0.567031631	0.925596631
PACRG	19.0378283	0.397431386	0.694342842	0.572384941	0.567061216	0.925596631
LOC100287015	145.3357311	0.13432775	0.234714717	0.572302206	0.567117256	0.925596631
ARHGAP21	1034.813519	-0.063568752	0.111112618	-0.572111013	0.567246769	0.925596631
APCDD1L	647.1121873	0.365460569	0.639111218	0.571826247	0.567439694	0.925596631
MIR379	4.225684429	0.673646541	1.178224739	0.571747069	0.567493342	0.925596631
H1-1	25.69459068	0.240278035	0.420268776	0.571724689	0.567508506	0.925596631
PCOLCE	1994.76093	-0.059278492	0.103701272	-0.571627437	0.567574403	0.925596631
RBFOX3	14.09906686	-0.29044286	0.508108539	-0.571615782	0.567582301	0.925596631
MRGBP	238.4067962	-0.087641555	0.153346272	-0.571527134	0.567642373	0.925596631
KIN	107.3082749	-0.171666243	0.300369659	-0.571516588	0.567649519	0.925596631
MAGI1	312.9312904	-0.127740071	0.223537864	-0.57144713	0.567696589	0.925596631
PCDHB16	59.55730403	-0.200530622	0.350918139	-0.571445588	0.567697634	0.925596631
BTN2A2	295.2389313	0.100815685	0.176429336	0.571422458	0.567713309	0.925596631
TMPRSS6	9.412261587	0.404955596	0.708838015	0.571294975	0.567799707	0.925596631
DNAJC10	3300.921344	-0.075139957	0.13153455	-0.571256427	0.567825833	0.925596631
MIR4642	4.839031644	-0.515547881	0.902706123	-0.571113753	0.567922537	0.925596631
SLC40A1	108.8115965	0.157993945	0.276645656	0.571105823	0.567927911	0.925596631
ACSL1	233.8467632	0.115470022	0.202191622	0.571092023	0.567937266	0.925596631
MYO7A	18.69439136	-0.269242857	0.471454349	-0.571089984	0.567938648	0.925596631
PSMD7	1422.935206	-0.078895407	0.138149597	-0.571086769	0.567940827	0.925596631
PRRG2	8.426642144	0.396421492	0.694208279	0.571041147	0.567971751	0.925596631
ENO1-AS1	7.212718468	0.470110317	0.823482211	0.570880962	0.568080337	0.925596631
GABARAPL1	201.2739611	-0.167777899	0.293904771	-0.57085803	0.568095883	0.925596631
BEX5	3.482542435	0.760980678	1.33307509	0.570846072	0.56810399	0.925596631
CDH2	1032.687434	-0.099299387	0.173955284	-0.570832826	0.568112969	0.925596631
LDOC1	45.84844289	0.181750949	0.318416717	0.570795876	0.568138019	0.925596631
AIMP2	356.7961193	0.095917166	0.16804247	0.570791218	0.568141177	0.925596631
ANKRD42	139.3974846	-0.112957947	0.197929482	-0.570697934	0.568204419	0.925596631
RNU5F-1	9.400607034	-0.403122488	0.706504211	-0.570587523	0.568279278	0.925596631
LOC101929563	3.239725764	-0.759851606	1.331742079	-0.570569646	0.568291399	0.925596631
CASS4	4.394796748	0.610877911	1.07107017	0.570343501	0.568444742	0.925610406
RIOX2	525.9706408	-0.079421336	0.139267401	-0.570279443	0.568488181	0.925610406
C1orf115	32.61850473	-0.208288072	0.365269453	-0.57023129	0.568520836	0.925610406
MIR6719	3.844172985	-0.798330228	1.400498342	-0.570032969	0.568655337	0.925610406
TUBGCP2	751.6148061	-0.060537625	0.106206267	-0.570000494	0.568677363	0.925610406
CCDC7	83.91806135	0.20505485	0.359827321	0.569870153	0.56876577	0.925610406
NME7	138.3398149	0.102562152	0.179989577	0.569822728	0.568797939	0.925610406
DNA2	391.6100543	0.12296698	0.215812662	0.569785753	0.56882302	0.925610406
RCOR2	139.7200176	0.12985245	0.228072156	0.569348104	0.569119928	0.925610406
TMEM248	1743.416079	-0.061489629	0.108014769	-0.569270567	0.569172538	0.925610406
GATM	6.508318007	-0.420363523	0.738428545	-0.569267704	0.56917448	0.925610406
LINC00513	33.80689219	0.465984978	0.818617144	0.569234326	0.569197129	0.925610406
FBN1	21830.55446	-0.145515167	0.255646424	-0.569204784	0.569217175	0.925610406
VTN	16.12196295	-0.32902298	0.578094186	-0.569151166	0.569253558	0.925610406
MINCR	51.12601036	0.173106714	0.30416689	0.569117547	0.569276371	0.925610406
SLC25A51	305.8672951	0.075589734	0.132829969	0.569071382	0.569307698	0.925610406
RDH12	10.46059743	-0.3570551	0.627723759	-0.568809281	0.569485576	0.925610406
TSG1	12.52587631	-0.32722076	0.575299482	-0.568783338	0.569503183	0.925610406
ZNF615	168.116523	-0.121109802	0.212965797	-0.568681938	0.569572007	0.925610406
ST3GAL4	699.9630344	-0.094368467	0.165999012	-0.568488125	0.569703567	0.925610406
TLR9	4.346137476	0.634149227	1.115602213	0.568436688	0.569738485	0.925610406
DCST2	27.17095391	0.243798721	0.428919904	0.568401509	0.569762366	0.925610406
VAT1	2580.166995	-0.158502861	0.278858303	-0.56839929	0.569763873	0.925610406
TPPP3	20.05073858	-0.267065941	0.469953357	-0.568281803	0.569843633	0.925610406
LOC105371267	1.982009938	-0.777401124	1.36800255	-0.568274616	0.569848512	0.925610406
SCO1	948.9620109	0.062884146	0.110667676	0.56822505	0.569882164	0.925610406
CPS1	32.75012774	-0.331669929	0.583725728	-0.56819481	0.569902695	0.925610406
C1QBP	2723.372341	-0.106492884	0.187427958	-0.568180357	0.569912508	0.925610406
EDAR	3.94615832	0.778547639	1.370303154	0.568157226	0.569928213	0.925610406
SNORD103C	5.258183571	0.500392558	0.880745449	0.568146629	0.569935408	0.925610406
CDK2AP2	83.87264551	0.155358083	0.273492145	0.568053183	0.569998856	0.925610406
CRABP1	2.638788791	-0.756917234	1.332567449	-0.568014201	0.570025326	0.925610406
HLA-C	2169.321537	-0.124047214	0.218387564	-0.56801409	0.5700254	0.925610406
POLR1C	521.739584	-0.074997016	0.132079627	-0.56781669	0.570159445	0.925610406
CAMKV	4.481883904	0.644544253	1.135238796	0.567760946	0.570197302	0.925610406
FMC1-LUC7L2	3.90972532	-0.573558555	1.010282414	-0.567721013	0.570224421	0.925610406
MOSPD3	56.58989888	-0.202149879	0.356099176	-0.567678592	0.570253231	0.925610406

NICN1	187.6881967	0.141335739	0.248984603	0.567648509	0.570273661	0.925610406
SNORD51	4.184014313	0.621296653	1.094991208	0.56739876	0.570443292	0.925798441
SLC8B1	573.5700211	0.066299115	0.116863727	0.567319876	0.570496876	0.925798441
LOC100505942	2.42016983	-0.693277333	1.22225181	-0.567213177	0.570569356	0.925798441
FCHO2	393.6773503	-0.101554525	0.179054189	-0.567172011	0.570597322	0.925798441
NNAT	31.75034637	0.385169555	0.679233397	0.567065102	0.570669952	0.925798979
NIPSNAP1	359.8776294	-0.160065305	0.282292876	-0.567018579	0.570701559	0.925798979
CPA4	35.54587689	-0.261575457	0.461434865	-0.566874064	0.570799746	0.925873974
CLMP	2644.688038	0.092707701	0.163632664	0.566559873	0.571013244	0.926075601
ZNF584	320.6613523	-0.118260151	0.208749614	-0.566516742	0.571042555	0.926075601
LINC01133	6.937139468	-0.5522272	0.974956537	-0.566412121	0.571113657	0.926075601
LOC574538	12.53875153	-0.381986724	0.674429156	-0.566385246	0.571131922	0.926075601
CYP27A1	44.73537733	-0.183189307	0.323581532	-0.566130292	0.571305213	0.926207803
DHRS4L2	114.8263255	-0.111073591	0.19621871	-0.566070338	0.571345967	0.926207803
SLC38A5	14.80287538	-0.408441719	0.721582721	-0.566035892	0.571369382	0.926207803
MAP3K1	185.5501705	-0.090896531	0.16064015	-0.565839433	0.571502937	0.926298392
BBS10	342.2924055	-0.095693744	0.169176057	-0.565645903	0.571634516	0.926298392
TWF1	1036.485236	0.07902926	0.139725606	0.565603273	0.571663503	0.926298392
LINC01869	2.34513393	-0.772960888	1.366763137	-0.565541217	0.571705698	0.926298392
RPUSD3	351.8217028	0.085312651	0.150853027	0.565534895	0.571709996	0.926298392
ZNF311	13.14765506	0.385219094	0.681206942	0.565494962	0.57173715	0.926298392
LOC107984507	87.57827044	-0.221644136	0.392034554	-0.565368877	0.571822889	0.926299135
PEX1	270.2552072	-0.103057415	0.182292352	-0.565341407	0.57184157	0.926299135
TCTN1	478.6479495	-0.09074269	0.160557259	-0.565173389	0.571955835	0.926392651
PPP1R32	5.52346864	-0.499946034	0.884697956	-0.565103638	0.572003274	0.926392651
ZNF407	740.1910692	0.121630881	0.215266795	0.565023887	0.572057517	0.926396306
LOC101928222	6.80883471	0.467003972	0.826865131	0.564788567	0.572217584	0.926427085
COX18	270.1382581	0.115402746	0.204338721	0.564762004	0.572235654	0.926427085
ZNF689	222.0993648	-0.096248912	0.170432011	-0.564734944	0.572254062	0.926427085
SUPT3H	509.1634781	0.167673169	0.296929462	0.564690238	0.572284475	0.926427085
MIR5685	6.758931963	0.55222429	0.978174123	0.564546002	0.572382602	0.926501769
LRRIQ1	32.99019218	-0.279116077	0.49469252	-0.564221342	0.572603505	0.926761488
SLC4A1AP	499.5839778	-0.072349183	0.12824292	-0.564157325	0.572647068	0.926761488
ZFYVE21	441.5632284	0.066779663	0.118430967	0.563869948	0.572842643	0.926891106
ATXN1-AS1	48.68835447	-0.18866235	0.334641212	-0.563775003	0.572907266	0.926891106
DENND11	221.4155588	0.118947102	0.210991806	0.563752235	0.572922763	0.926891106
AP2M1	11620.13687	-0.08430895	0.149571731	-0.563669013	0.572979409	0.926891106
COL16A1	5791.064395	0.115048432	0.204112307	0.5636526	0.572990582	0.926891106
MAP3K13	798.1274811	-0.078137313	0.138650333	-0.563556622	0.573055915	0.926891106
C11orf96	14.7370155	-0.329713553	0.585112333	-0.563504705	0.573091257	0.926891106
SELENOM	134.2476823	0.118956775	0.211132226	0.563423106	0.573146807	0.926896824
MED14OS	9.54526749	0.350296696	0.622046632	0.563135748	0.57334245	0.92692357
NEK11	51.99072945	-0.146369589	0.259967454	-0.563030436	0.573414159	0.92692357
SLC9A3R2	73.31686718	0.141801693	0.251907138	0.562912565	0.573494423	0.92692357
TBCEL	379.1684068	-0.110487838	0.196317958	-0.562800467	0.573570762	0.92692357
YARS2	251.028055	0.09238307	0.164165062	0.562745011	0.57360853	0.92692357
LOC101927354	2.772239347	-0.897743303	1.595512066	-0.562667824	0.573661098	0.92692357
C14orf119	741.6216787	0.138150461	0.245574695	0.562559838	0.573734646	0.92692357
PREPL	1430.711444	-0.065885859	0.11711981	-0.562550938	0.573740709	0.92692357
FUCA2	2728.336596	0.069439107	0.123439535	0.562535391	0.573751298	0.92692357
POFUT1	2320.683561	-0.081014861	0.144078253	-0.562297636	0.573913249	0.92692357
RPL11	2081.139841	0.098642061	0.17543218	0.562280314	0.573925049	0.92692357
ETV2	5.99529618	0.485246035	0.863128789	0.562194242	0.573983684	0.92692357
LRRC6	108.2477591	-0.13971525	0.248521341	-0.562186127	0.573989212	0.92692357
MUSTN1	3.282415306	0.631717272	1.123713294	0.562169439	0.574000581	0.92692357
DBN1	1699.242885	-0.093718088	0.166717166	-0.562138204	0.574021861	0.92692357
TREX2	7.311566104	0.421568462	0.749994876	0.562095123	0.574051211	0.92692357
MYO1G	2.860925741	0.686708711	1.221734674	0.562076796	0.574063697	0.92692357
ZNF451-AS1	22.49446741	0.318130782	0.566049957	0.562018915	0.574103132	0.92692357
PINLYP	9.51130913	0.475761547	0.846629572	0.561947708	0.574151648	0.92692357
KIF5A	34.91910583	0.200154368	0.356550839	0.561362774	0.574550256	0.927437311
MRPL11	248.9639214	-0.084469255	0.150481395	-0.561326904	0.574574704	0.927437311
PTENP1-AS	2.133693655	-0.826039781	1.471781492	-0.561251643	0.574626002	0.927437311
YME1L1	2414.17608	-0.07165781	0.12769907	-0.5611459	0.57469808	0.927469641
TMEM151B	256.211883	0.445237525	0.793579415	0.56104974	0.574763629	0.927491431
SEC61A2	90.59190368	0.214392567	0.382267301	0.560844639	0.574903453	0.927633061
LOC100130548	42.87189819	0.350392294	0.625020307	0.560609455	0.575063804	0.927807786
SYVN1	419.9472729	-0.086130078	0.153670212	-0.560486494	0.575147649	0.927859054
BTBD7	1764.93594	0.052425802	0.093588952	0.560170849	0.575362909	0.928116522
SRGAP2	1241.838278	0.084379837	0.15065145	0.560099736	0.57541141	0.928116522
GS1-24F4.2	21.1290368	0.297630945	0.531559573	0.559920204	0.575533867	0.92819656
MCIDAS	65.44085687	0.215016206	0.384081223	0.559819623	0.575602477	0.92819656
PYDC2	3.121260554	-0.75253418	1.344296176	-0.559797903	0.575617294	0.92819656
CD248	14.77067037	-0.336608551	0.601714803	-0.559415439	0.575878227	0.928445808
RAVER2	525.1052877	0.077581974	0.138695426	0.559369379	0.575909655	0.928445808
DNAI1	4.615439283	0.515308145	0.921359408	0.55929113	0.575963048	0.928445808
MIR4312	8.586827288	0.556808218	0.995715903	0.559203902	0.576022571	0.928445808
GTF2B	227.0003994	-0.108465141	0.193992499	-0.559120283	0.576079633	0.928445808
KIRREL1	4159.238182	-0.053539816	0.095770505	-0.559042842	0.576132482	0.928445808
SHISA7	3.2679548	-0.813051624	1.454379313	-0.559036846	0.576136574	0.928445808
LRIG2-DT	28.22901663	0.26997069	0.483006198	0.558938355	0.576203792	0.928470167
ANKRD36BP2	9.135575002	0.40408557	0.723090885	0.558830955	0.576277095	0.928504324
PRPF3	537.8075951	0.121563444	0.21759188	0.558676382	0.576382601	0.928590357
MLANA	9.701668889	-0.377525207	0.675986515	-0.558480381	0.576516397	0.928630127
HNF4G	128.1520213	-0.107034728	0.191692593	-0.558366531	0.576594122	0.928630127
C11orf53	1.971680656	-0.703621533	1.260241814	-0.558322637	0.57662409	0.928630127
HLA-DPB1	20.97857121	0.31969854	0.572694848	0.558235403	0.576683649	0.928630127
FAM155A	120.198718	-0.213079895	0.381752373	-0.558162593	0.576733362	0.928630127
MIR3916	25.88148203	0.246002827	0.440805727	0.558075387	0.576792907	0.928630127
LINC02249	29.12737272	0.356884309	0.639702244	0.557891289	0.57691862	0.928630127

TMEM144	133.3150191	0.108841813	0.195125143	0.557805166	0.576977435	0.928630127
KLF11	173.9606595	0.119369877	0.214018792	0.557754184	0.577012252	0.928630127
SLC15A3	4.797668874	-0.605323782	1.085463308	-0.557663974	0.577073863	0.928630127
CDH19	74.98239205	-0.231787581	0.415670962	-0.557622741	0.577102025	0.928630127
ARF4-AS1	12.52418149	0.344009736	0.616959802	0.557588574	0.57712536	0.928630127
POU5F1P5	4.147331296	0.717508237	1.28687796	0.557557329	0.577146702	0.928630127
ZNF724	37.75475163	0.176753563	0.317082976	0.557436306	0.577229366	0.928630127
LOC101928882	69.3632931	-0.193568669	0.347252788	-0.557428697	0.577234563	0.928630127
LINC01547	89.75742898	-0.131980479	0.236770614	-0.557419167	0.577241073	0.928630127
RERE	871.3319976	-0.07473632	0.134117414	-0.557245458	0.577359735	0.928635303
DLEU7-AS1	98.78820301	0.148854076	0.267125672	0.557243618	0.577360992	0.928635303
SGO1-AS1	2.439683734	0.689045398	1.236653273	0.557185602	0.577400626	0.928635303
DTX4	537.8808264	0.200213468	0.359450333	0.556998977	0.577528128	0.928675571
NKX2-1-AS1	31.33304598	-0.245731881	0.441173198	-0.556996395	0.577529892	0.928675571
CEP63	26.78255256	-0.250521379	0.449975189	-0.556744873	0.577701753	0.928868108
FAM71E2	2.206262073	0.860899437	1.546608979	0.556636777	0.577775621	0.928903065
HM13-AS1	12.1999521	0.316918531	0.569499017	0.556486528	0.577878301	0.928984333
LOC254896	87.57745709	-0.122414671	0.220058269	-0.556282986	0.578017416	0.929124153
MIR593	10.64102955	-0.413024868	0.742600964	-0.556186819	0.578083149	0.929146001
TFB1M	233.4142888	-0.147651646	0.265607183	-0.55590231	0.578277639	0.929214614
SNORA63C	3.865893283	0.683890143	1.230280929	0.555881284	0.578292014	0.929214614
SPTAN1	2354.860447	-0.060325201	0.108525978	-0.555859548	0.578306874	0.929214614
ARHGEF5	3.628609218	-0.590645977	1.062928526	-0.555677981	0.578431012	0.929214614
SPEN	1960.03143	0.06288283	0.113199288	0.555505527	0.578548931	0.929214614
MON1B	551.7643769	-0.069460795	0.125049707	-0.555465475	0.578576319	0.929214614
STK4	1316.367889	-0.07615433	0.137100385	-0.555464014	0.578577318	0.929214614
RP9P	109.3733296	-0.151271485	0.272380696	-0.555367863	0.578643069	0.929214614
CYP51A1-AS1	6.448818174	0.496304704	0.894024512	0.555135455	0.578802013	0.929214614
CENPL	357.4980718	-0.093180636	0.167852333	-0.555134593	0.578802603	0.929214614
IGF2	3.044706193	-0.886476396	1.596919003	-0.555116693	0.578814846	0.929214614
MCTS1	403.4335865	-0.116825933	0.210496521	-0.555001731	0.578893477	0.929214614
MTMR9LP	41.75551387	0.208030101	0.374889233	0.554910844	0.578955644	0.929214614
LOC441087	3.198381429	0.624251038	1.125155829	0.554812962	0.5790226	0.929214614
WRAP73	239.4287388	0.091772801	0.165438658	0.554724044	0.579083427	0.929214614
MIR942	2.257878899	0.783089717	1.411830598	0.554662662	0.57912542	0.929214614
RHOBTB1	836.9192308	-0.115613784	0.208475967	-0.554566481	0.579191221	0.929214614
P2RX5-TAX1BP3	23.59505281	0.242222906	0.43678006	0.554564936	0.579192278	0.929214614
IARS2	2511.224528	-0.067207143	0.121194858	-0.554537912	0.579210767	0.929214614
UBE2C	445.7382338	0.087789559	0.158327573	0.554480544	0.579250017	0.929214614
CCDC169-SOHLH2	5.475234233	-0.574216019	1.035633554	-0.554458685	0.579264973	0.929214614
CTSV	10.15735667	-0.386862357	0.697808895	-0.554395853	0.579307964	0.929214614
PAXBP1	580.142799	0.128984525	0.232693966	0.55430971	0.579366906	0.929214614
RNF44	402.3903272	0.073162452	0.131999221	0.554264276	0.579397995	0.929214614
RAD51-AS1	83.25043399	0.218005049	0.393687962	0.553750864	0.57974936	0.929214614
PDPR	1470.809137	0.075117569	0.135659031	0.553723324	0.579768211	0.929214614
TLDC2	6.866652694	-0.440790432	0.796288573	-0.553556143	0.579882648	0.929214614
ASTN2	119.5472059	0.117630917	0.212535664	0.553464368	0.579945473	0.929214614
TPTEP2-CSNK1E	5.254244216	0.546089223	0.986743903	0.553425485	0.579972092	0.929214614
LOC106660606	12.02663743	-0.334350417	0.604207645	-0.553370054	0.58001004	0.929214614
ZNRF3-AS1	6.730552241	0.572603509	1.035032786	0.553222581	0.580111006	0.929214614
DPT	2.953498107	0.724506814	1.309963104	0.553074214	0.580212593	0.929214614
WDR91	432.0670597	0.077058863	0.139357556	0.552957908	0.580292233	0.929214614
TGDS	183.8376257	-0.105213789	0.190339666	-0.55276859	0.580421879	0.929214614
OSBPL1A	836.4208354	-0.092314018	0.167011276	-0.552741227	0.580440619	0.929214614
UBA6	1854.396305	0.066410296	0.12014944	0.552730799	0.580447761	0.929214614
PITRM1	2321.841761	-0.069958574	0.126590159	-0.552638329	0.580511091	0.929214614
TERF1	411.4759162	-0.091813746	0.166146024	-0.552608744	0.580531353	0.929214614
NRDC	2777.450109	-0.063152888	0.114289892	-0.552567565	0.580559557	0.929214614
ZNF844	38.92634179	0.25317539	0.458240753	0.552494268	0.58060976	0.929214614
LINC00339	165.4872151	-0.171755854	0.310895679	-0.55245494	0.580636699	0.929214614
SNORD114-28	13.81882961	-0.390640991	0.707125766	-0.552434956	0.580650387	0.929214614
FAM160A2	408.8091881	0.100292225	0.181577037	0.552339804	0.580715564	0.929214614
SOD1	896.1560843	-0.065602473	0.118775943	-0.552321217	0.580728296	0.929214614
LRRC8C-DT	7.664002864	0.476924015	0.863496223	0.55231743	0.58073089	0.929214614
RAB32	312.6571346	0.09053823	0.163931295	0.552293755	0.580747109	0.929214614
FAM177A1	211.282119	0.094812671	0.171693936	0.552219101	0.580798249	0.929214614
PRKD3	2569.9816	0.117644108	0.213105012	0.552047591	0.580915747	0.929214614
NCF2	10.99197754	0.499085908	0.904178423	0.551977238	0.580963949	0.929214614
LOC101930370	37.49400478	0.208548352	0.377866738	0.55190979	0.581010161	0.929214614
NRBP1	1627.743099	-0.090772902	0.164506577	-0.551788895	0.581092997	0.929214614
SRP9	2996.971623	-0.072020052	0.13055241	-0.551656243	0.581183895	0.929214614
COL13A1	54.96390182	-0.171255381	0.310439575	-0.551654475	0.581185106	0.929214614
LINC01000	1028.672675	0.126289901	0.228939451	0.551630139	0.581201783	0.929214614
GTPBP10	242.1766485	0.081955173	0.148577366	0.55159931	0.581222909	0.929214614
ADAMTS9-AS2	14.64371078	0.285324696	0.517318654	0.551545347	0.58125989	0.929214614
TMEM255B	3.552584441	-0.582291083	1.055745886	-0.551544733	0.58126031	0.929214614
ZNF329	311.6761983	0.107059999	0.194157363	0.551408393	0.581353749	0.929214614
TRIP6	808.8879583	-0.08390415	0.152174752	-0.55136709	0.581382056	0.929214614
DANT1	16.23983865	0.514911196	0.93403938	0.551273541	0.581446174	0.929214614
DSG2-AS1	17.76525799	-0.355369755	0.644641391	-0.551267356	0.581450412	0.929214614
LINC01037	26.61741894	0.234319734	0.425119754	0.551185241	0.581506696	0.929214614
DENND2C	145.6922193	-0.115196384	0.209005811	-0.551163549	0.581521565	0.929214614
CHMP1B2P	6.992647051	0.481757395	0.874090321	0.551152877	0.58152888	0.929214614
LOC105371814	5.947625985	-0.479070437	0.869305167	-0.551095812	0.581567996	0.929214614
LZTS2	740.5549988	-0.061795809	0.112157424	-0.550973861	0.581651593	0.929214614
FAM27B	3.529240234	-0.802577464	1.45668108	-0.550963059	0.581658998	0.929214614
NOP53-AS1	5.275155669	0.467824069	0.849170234	0.550919063	0.581689159	0.929214614
RPGR	121.9703416	-0.112609072	0.204436873	-0.550825642	0.581753204	0.929214614
MRPS10	1274.408464	-0.072006578	0.130732686	-0.550792464	0.581775951	0.929214614
PCDHGB2	26.24424827	0.205853727	0.37381393	0.550685008	0.581849624	0.929248996

TTC8	277.3503245	-0.079191103	0.143841611	-0.550543773	0.581946462	0.929320365
IDE	730.4090966	-0.086457754	0.157070722	-0.550438383	0.582018728	0.929352486
EVI5	529.4236572	-0.078900956	0.14340103	-0.550211916	0.582174032	0.929431838
SPEF2	59.92145496	-0.143518696	0.26085549	-0.550184685	0.582192707	0.929431838
MYO15B	161.509976	0.192443525	0.349809705	0.550137753	0.582224894	0.929431838
KMT5C	160.1553682	-0.132939026	0.241698623	-0.550019792	0.582305799	0.929458796
LOC285626	2.362950982	-0.776530651	1.411973936	-0.549961038	0.582346098	0.929458796
RERE-AS1	3.681655197	-0.609613022	1.109730759	-0.549334167	0.582776144	0.929672063
DLAT	873.0648944	0.065372263	0.119005	0.54932367	0.582783346	0.929672063
TEX21P	3.483552514	-0.775027205	1.411405364	-0.549117372	0.582924904	0.929672063
CSNK1G2-AS1	17.05911591	0.438911248	0.799335129	0.549095407	0.582939977	0.929672063
NUDT7	9.853625067	0.408953101	0.744803294	0.549075311	0.582953767	0.929672063
LOC101928279	2.975622685	0.590921	1.076227906	0.549066788	0.582959616	0.929672063
SLC66A1	174.4194816	-0.11492868	0.209391344	-0.548870252	0.583094494	0.929672063
DUS1L	1112.793513	0.101241874	0.184462479	0.548848063	0.583109723	0.929672063
IER3IP1	711.5155901	-0.091954714	0.167561143	-0.548783039	0.583154351	0.929672063
GTF2IRD2B	2.706433965	0.839535098	1.530093161	0.548682341	0.583223467	0.929672063
SNORD136	4.47178146	-0.55215699	1.006372848	-0.548660461	0.583238485	0.929672063
PAPOLG	636.1266972	0.08914861	0.162525672	0.548520172	0.583334782	0.929672063
CMAS	326.6731835	0.07112727	0.129679292	0.548485955	0.583358271	0.929672063
CCDC81	5.155970669	-0.516909087	0.942492462	-0.548449041	0.583383611	0.929672063
TM2D3	519.2725982	0.070931137	0.129335304	0.548428271	0.583397869	0.929672063
GEM	95.68316743	0.17610527	0.32113231	0.548388515	0.583425161	0.929672063
FLJ12825	6.958684196	-0.395621671	0.721725918	-0.548160544	0.583581672	0.929672063
ZFP41	545.8174859	0.140985899	0.257213335	0.548128266	0.583603833	0.929672063
KLF6	5693.843353	-0.085077273	0.155251466	-0.547996581	0.583694251	0.929672063
HAS1	621.9595098	0.157505373	0.287442581	0.547954212	0.583723343	0.929672063
COMMD10	228.9598544	-0.092714465	0.169210116	-0.547925069	0.583743354	0.929672063
HCRTR2	43.74964516	0.160592166	0.29310574	0.547898398	0.583761669	0.929672063
KLF14	3.825853965	0.67506264	1.232255991	0.547826624	0.583810955	0.929672063
CISD3	186.8341907	0.089144501	0.162775159	0.547654211	0.583929358	0.929672063
FAM98B	1103.449641	-0.063996272	0.116864477	-0.547610995	0.583959038	0.929672063
LINC01186	15.7609806	0.39707438	0.725134017	0.547587578	0.583975121	0.929672063
FBXO22	648.3596968	0.100967915	0.184388516	0.547582448	0.583978644	0.929672063
PLAUR	557.4190739	-0.136137584	0.248618259	-0.547576773	0.583982542	0.929672063
SLC49A3	10.87586631	0.336380644	0.614324246	0.547562051	0.583992653	0.929672063
TDRD6	28.99810876	0.25282873	0.461822712	0.547458415	0.584063833	0.929702322
LOC648987	92.30471846	-0.12639271	0.230994391	-0.547167876	0.584263404	0.92983193
RPL13P5	25.39771539	-0.214285528	0.391766795	-0.546972155	0.584397862	0.92983193
SFMBT1	149.0896149	-0.117939699	0.215663714	-0.546868532	0.584469056	0.92983193
PMS2P1	150.6438014	0.096485319	0.176446983	0.546823285	0.584500144	0.92983193
TTC37	2737.947568	-0.047893768	0.087589731	-0.546796616	0.584518468	0.92983193
PCSK1	21.68690817	-0.226385496	0.414031338	-0.546783481	0.584527493	0.92983193
SH3D21	138.8686444	0.177746309	0.325103466	0.546737663	0.584558975	0.92983193
FSTL5	2.946968622	-0.607918127	1.111911942	-0.546732259	0.584562689	0.92983193
S1PR2	413.5440857	0.094451678	0.172845744	0.546450704	0.584756165	0.929857805
TSHZ2	11.15459963	-0.603197661	1.103848363	-0.546449749	0.584756821	0.929857805
ZNF317	763.6285835	-0.066996115	0.12263259	-0.546315752	0.584848911	0.929857805
TPD52	23.27048681	-0.28077673	0.514037434	-0.54621845	0.584915785	0.929857805
UPP1	16.04576226	0.299760899	0.548825734	0.546185939	0.584938131	0.929857805
WAC-AS1	136.2824867	-0.150221361	0.275056788	-0.546146714	0.584965091	0.929857805
ALOX12-AS1	16.46322892	-0.27712533	0.507442861	-0.54612125	0.584982594	0.929857805
LOC284930	87.53895608	0.257945	0.472339225	0.546101164	0.5849964	0.929857805
GSKIP	352.8577241	-0.075835371	0.138916939	-0.545904424	0.585131638	0.929881532
NDNF	488.5039811	0.120424799	0.220631094	0.545819704	0.585189878	0.929881532
NSUN5	274.1308181	-0.094151336	0.17250148	-0.545800166	0.585203309	0.929881532
IGF2BP1	2131.594426	-0.042819842	0.078470619	-0.545679924	0.585285974	0.929881532
CTSL	474.3970888	-0.084204569	0.15434192	-0.545571607	0.585360446	0.929881532
KIF15	515.7780151	-0.077232348	0.141585406	-0.545482404	0.585421779	0.929881532
PCOTH	8.869990866	-0.397623716	0.728982193	-0.54545052	0.585443703	0.929881532
SNORD14B	23.66317176	0.248022714	0.454725826	0.545433532	0.585455383	0.929881532
MTR	2527.632512	-0.054029649	0.09906664	-0.545386917	0.585487437	0.929881532
ZBED9	276.9491154	-0.106529283	0.19535178	-0.54532026	0.585533273	0.929881532
ARHGAP26	110.9127363	0.121200411	0.222286327	0.54524456	0.585585328	0.929881532
PAFAH1B3	483.4711093	-0.077848492	0.142823628	-0.54506732	0.585707218	0.929933845
ZNF654	605.1162271	0.078442174	0.143931053	0.544998265	0.585754711	0.929933845
AP1M2	4.480969547	0.484326654	0.888723282	0.544969018	0.585774826	0.929933845
RDH13	235.3463719	0.108703906	0.199526488	0.544809398	0.585884615	0.929988994
MSANTD4	405.3138717	-0.078388076	0.143901114	-0.544735715	0.585935297	0.929988994
B3GNT7	16.37065778	0.989771756	1.817125581	0.544690893	0.585966129	0.929988994
ASB18	10.29294906	-0.533036643	0.978937433	-0.544505323	0.586093787	0.930108761
SYPL2	5.237543403	-0.451524384	0.829363609	-0.544422711	0.586150621	0.930110256
ZNF189	478.8753209	-0.071541608	0.131437802	-0.544300097	0.58623498	0.930110256
CCNE1	156.9372704	-0.183254163	0.336708244	-0.544252084	0.586268015	0.930110256
ELP2	2120.937564	0.053659303	0.098602082	0.5442005	0.586303508	0.930110256
ARGLU1	853.7464775	0.122818593	0.225739037	0.544073346	0.586391001	0.930166249
ZNF558	713.6289206	0.070494216	0.129602435	0.543926628	0.586491964	0.930195545
PCDHB6	193.6068761	0.12169625	0.223749612	0.543894798	0.586513869	0.930195545
NPR2	435.2954199	-0.061311225	0.112742454	-0.543816658	0.586567645	0.930198045
PTK2	2761.281651	0.047519728	0.087432199	0.543503752	0.586783008	0.930456772
DHX8	1522.96272	-0.066643868	0.122637856	-0.543420035	0.586840634	0.930465353
NSMCE4A	255.3975977	-0.091439801	0.168321636	-0.543244488	0.58696148	0.930574161
RNF113B	3.326844875	0.896179101	1.650210622	0.543069526	0.587081933	0.930682328
NMI	29.14532317	-0.19171868	0.353139681	-0.542897584	0.58720032	0.930728611
SMG1P3	331.6144997	0.341652899	0.629382451	0.542838298	0.587241142	0.930728611
HELLS	1335.817951	-0.131116424	0.241624587	-0.542645209	0.587374105	0.930728611
INHBA	1289.934093	-0.092274117	0.170061519	-0.542592573	0.587410354	0.930728611
AP1S1	365.4679667	-0.113175675	0.208667222	-0.542373997	0.587560889	0.930728611
ZNF318	1348.896384	0.081411791	0.150107267	0.542357429	0.5875723	0.930728611
SALL4	48.10301051	0.278263799	0.513120701	0.542296965	0.587613946	0.930728611

SMPDL3A	79.2112374	-0.124013168	0.2287036	-0.542244056	0.58765039	0.930728611
ZGLP1	39.85271867	0.178876322	0.329948061	0.542134788	0.587725655	0.930728611
GTF3C3	1272.464234	0.065518539	0.12085657	0.542118144	0.587737121	0.930728611
RPL26L1	136.2403902	-0.108262994	0.199708765	-0.542104367	0.58774661	0.930728611
ZNF876P	27.02149878	-0.214787534	0.396264441	-0.542030805	0.587797285	0.930728611
ZNF146	2156.154723	-0.052434144	0.096745638	-0.541979412	0.587832689	0.930728611
RTCA	339.4965469	-0.075517746	0.139341095	-0.5419632	0.587843858	0.930728611
TBC1D12	328.5736669	-0.087265596	0.161090442	-0.541718024	0.588012771	0.930728611
MRPL37	1191.09046	-0.08555087	0.157964666	-0.541582322	0.588106273	0.930728611
LOC101929950	43.15792	0.179837205	0.332071003	0.541562507	0.588119927	0.930728611
TSNAX-DISC1	7.769390493	0.368740604	0.680908287	0.541542246	0.588133887	0.930728611
MIR7111	2.090473891	0.744877602	1.375494888	0.541534257	0.588139392	0.930728611
RTL9	3.044494617	-0.561016767	1.03602183	-0.541510565	0.588155718	0.930728611
FANCF	288.71817	0.088931828	0.164321894	0.541204985	0.588366302	0.930979178
SLC30A6	884.5813958	0.052941541	0.097846908	0.541065034	0.588462758	0.931049129
SULT2B1	2.395948725	0.74749225	1.381946123	0.540898258	0.588577712	0.93106478
DNASE1	71.00173877	0.226604739	0.418957963	0.540877031	0.588592344	0.93106478
CFAP43	8.128006344	0.368377579	0.681149801	0.540817274	0.588633535	0.93106478
B3GALT2	27.53526349	0.373820268	0.69130283	0.540747486	0.588681643	0.93106478
ARMH3	501.3312238	-0.07908843	0.146281906	-0.54065764	0.58874358	0.931080103
ATAD2	2789.01021	-0.077412149	0.143237093	-0.540447638	0.588888362	0.931181828
FOXP2	370.9697453	0.092634258	0.1714139	0.540412755	0.588912413	0.931181828
AMPD2	1705.759605	0.086332372	0.159776741	0.540331289	0.588968584	0.93118802
ADGRG2	12.6269557	0.329509441	0.609992873	0.540185723	0.589068957	0.93126409
USP25	926.1642593	-0.054840235	0.101546825	-0.540048747	0.589163415	0.931330795
SLC16A9	189.1606569	0.097341992	0.180322895	0.539820481	0.589320841	0.931497018
LOC101928140	5.228012993	-0.506607395	0.938657481	-0.539714865	0.589393687	0.931529535
CENPE	614.8072125	0.105567955	0.195637611	0.539609713	0.589466217	0.931561545
SNORD14E	8.719321939	0.494122249	0.915842076	0.539527788	0.589522728	0.931568238
NEFM	32.30023033	-0.182675821	0.338680835	-0.539374543	0.589628443	0.931652674
MIR3671	3.603229151	0.851136017	1.578234827	0.539296182	0.589682503	0.931655484
ARID3B	132.3573522	0.136648488	0.253532128	0.538978981	0.589901358	0.931918634
LSMEM2	16.76404153	0.320769868	0.595368018	0.538775779	0.590041578	0.931982125
RAB29	732.6536164	0.072413605	0.134405623	0.538769159	0.590046147	0.931982125
RRP36	396.3516948	-0.087771735	0.162958763	-0.538613164	0.590153802	0.932069552
TMEM141	385.1030073	-0.067454941	0.125269337	-0.53847927	0.590246213	0.932132888
GABPA	644.4387969	-0.071800279	0.13341466	-0.538173828	0.590457047	0.932247255
DPH3P1	41.57848586	0.167248259	0.310779247	0.538157745	0.590468149	0.932247255
COX14	99.22029788	0.129217308	0.240115269	0.538146987	0.590475576	0.932247255
NUSAP1	1593.764354	-0.069748896	0.129630501	-0.538059293	0.590536115	0.932260238
MTERF1	213.7280831	-0.108291984	0.201328388	-0.537887304	0.590654854	0.932344309
ANKRD44	371.536475	0.068092886	0.126615846	0.53779119	0.590721215	0.932344309
MRPL42	2132.223645	0.054352688	0.101073364	0.537754816	0.59074633	0.932344309
DIO1	7.640899633	0.505137973	0.939725445	0.537537827	0.590896163	0.932422479
DCAF12	712.4454384	-0.093731207	0.174386201	-0.537492108	0.590927735	0.932422479
BOLA2-SMG1P6	76.63110361	-0.137787223	0.256421871	-0.537345828	0.591028755	0.932422479
WDR4	643.7821441	0.1606278	0.298942513	0.537320031	0.591046571	0.932422479
GATA6-AS1	11.71389931	0.350192951	0.651759152	0.537304233	0.591057482	0.932422479
SUFU	299.6972387	-0.106022897	0.19738965	-0.537124904	0.591181339	0.932535316
CGB5	2.181722852	1.247860724	2.324737512	0.536774891	0.591423118	0.932767725
UGGT1	3213.492248	-0.062114051	0.11572033	-0.536760061	0.591433363	0.932767725
RHD	10.77165152	0.33970914	0.633015855	0.536651867	0.59150811	0.932803054
LOC107984132	3.127140469	-0.552148661	1.029425693	-0.536365728	0.591705812	0.933027113
SVIP	64.23391093	-0.185818559	0.346485926	-0.53629468	0.591754906	0.933027113
TP53I11	165.5372337	0.107306455	0.200172367	0.536070273	0.591909984	0.933103988
DHDH	5.034029807	0.507727477	0.94713009	0.536069419	0.591910574	0.933103988
CIZ1	1361.196427	-0.08597324	0.160421755	-0.535920083	0.592013784	0.933103988
ITFG1	787.8031139	-0.09437351	0.176117346	-0.535855849	0.59205818	0.933103988
IFT122	530.1943495	-0.078351205	0.146223495	-0.535831848	0.592074769	0.933103988
PRXL2C	648.2412743	-0.076503601	0.142791996	-0.535769535	0.592117839	0.933103988
NEURL2	22.62462486	0.287223205	0.536319346	0.535545114	0.592272971	0.933175462
FCGRT	344.4308294	-0.180403548	0.336899682	-0.535481503	0.592316945	0.933175462
SPDYE3	41.45751868	0.178415112	0.333289916	0.535315061	0.592432014	0.933175462
PIGP	113.6639692	0.131048517	0.24484176	0.535237604	0.592485567	0.933175462
THOC7-AS1	19.15573461	0.363198341	0.67863711	0.535187859	0.592519961	0.933175462
C1orf21	341.3279004	0.133897092	0.250195438	0.535169999	0.59253231	0.933175462
SNX24	168.5530071	-0.096644472	0.180601074	-0.535126786	0.592562189	0.933175462
LOC339059	21.94604358	0.400093094	0.74770067	0.535097948	0.592582129	0.933175462
VWDE	77.80728269	0.227150914	0.424594381	0.534983326	0.592661387	0.933217807
CLPTM1L	1861.509279	0.070248812	0.131339676	0.5348636	0.59274418	0.933265708
LIMCH1	6.006414185	0.463914596	0.867672544	0.534665525	0.592881165	0.933398918
LFNG	50.60950578	0.341954249	0.63974197	0.534519017	0.592982496	0.933475978
DTX3L	234.4295227	0.201410949	0.376974594	0.534282554	0.593146061	0.933650985
GSTT2	10.15178124	-0.327563954	0.613313381	-0.534089038	0.593279933	0.933760634
OSBP2	6.739514082	0.483600785	0.905567938	0.534030374	0.59332052	0.933760634
HACD4	162.5795465	-0.100450426	0.188137067	-0.533921506	0.593395842	0.93378847
SNORA47	2.277919561	0.69735362	1.306471512	0.533768715	0.593501561	0.93378847
RAB23	1151.555867	0.09670876	0.181198075	0.533718472	0.593536327	0.93378847
CHCHD2	2022.666824	-0.080717401	0.151262765	-0.533623729	0.593601887	0.93378847
GFM2	483.4158275	0.067905982	0.127258341	0.533607312	0.593613248	0.93378847
THAP8	30.61684581	0.214211946	0.401507341	0.533519375	0.593674102	0.93378847
PITPNM1	324.7519922	0.097333393	0.182451748	0.533474707	0.593705015	0.93378847
ATAT1	111.1580989	0.140940111	0.264262889	0.533332967	0.593803111	0.933860333
SRPK1	3444.462301	-0.052487231	0.09843547	-0.533214613	0.593885027	0.933875904
GAL3ST4	14.42167227	0.378781981	0.710471218	0.533141909	0.59393535	0.933875904
AP3D1	3342.762899	-0.049626424	0.093093024	-0.533084242	0.593975267	0.933875904
TMED3	2518.566891	0.079015304	0.148241951	0.533015811	0.594022636	0.933875904
CCDC125	148.6624428	0.120250201	0.22564377	0.532920545	0.594088582	0.933897189
TPI1P3	2.234548451	-0.808351556	1.5170655	-0.532838929	0.594145084	0.933903624
SNRPF	610.5590226	0.104877885	0.197026236	0.53230416	0.594515351	0.934284909

XYLT2	811.5032688	0.07490988	0.140733871	0.532280395	0.594531809	0.934284909
NSMCE1	427.7439799	-0.136037567	0.255584116	-0.532261429	0.594544942	0.934284909
SLC9B2	359.3355008	-0.073137603	0.137482462	-0.531977695	0.594741444	0.934420871
POMGNT2	156.4715021	0.11163107	0.209850919	0.531954163	0.594757743	0.934420871
NUDCD2	425.4238686	0.07781208	0.146305748	0.531845678	0.594832883	0.934420871
SH2D3C	4.206607586	-0.514575245	0.967549231	-0.531833656	0.59484121	0.934420871
STK36	534.684829	0.141570594	0.266389478	0.531442141	0.595112426	0.934671641
LINC01184	199.4132643	-0.123584342	0.232582758	-0.531356421	0.595171814	0.934671641
LRRC63	12.53041222	0.340032805	0.639943994	0.531347755	0.595177819	0.934671641
TEX15	2401.834212	-0.118254199	0.222575037	-0.531300369	0.59521065	0.934671641
WBP11	637.5013862	-0.072814124	0.137094574	-0.53112331	0.595333332	0.9346832
GHR	212.9863851	0.084999006	0.160056867	0.531055044	0.595380636	0.9346832
PIK3IP1-AS1	14.75106111	0.318693811	0.600323634	0.530870006	0.595508864	0.9346832
PCDHB11	33.16043018	-0.197533265	0.372131272	-0.530816087	0.595546231	0.9346832
GPR173	15.68044901	-0.282544696	0.532303138	-0.530796601	0.595559736	0.9346832
EDDM13	19.46485468	0.403082508	0.759414912	0.530780344	0.595571003	0.9346832
TM7SF3	849.6739549	-0.06820769	0.12852008	-0.530716215	0.595615448	0.9346832
ZC3H12C	282.8456031	-0.077459968	0.145962448	-0.530684221	0.595637622	0.9346832
ZNF833P	22.11824891	0.339744416	0.640441189	0.530484957	0.595775736	0.934808921
TMED9	2350.478475	0.07489607	0.141202171	0.530417269	0.595822656	0.934808921
DMAC2	548.5367716	0.079850847	0.150593365	0.53024147	0.595944522	0.934917808
MMADHC	856.0365543	-0.107546794	0.202876352	-0.530110054	0.596035629	0.934966086
PPHLN1	540.1376653	-0.072544242	0.136880488	-0.529982346	0.596124171	0.934966086
EMILIN1	10506.07155	-0.063130506	0.119120894	-0.529970048	0.596132698	0.934966086
PSMB8	144.1753671	-0.194236183	0.366556798	-0.529893823	0.596185549	0.934966689
IRF2	182.6719597	-0.104326557	0.196963448	-0.529674712	0.596337484	0.934971306
LOC100996842	39.92296229	0.226188723	0.427059918	0.529641658	0.596360405	0.934971306
CYB5RL	245.7474705	-0.093777728	0.177070872	-0.529605616	0.5963854	0.934971306
LINC01541	47.01943714	-0.399481535	0.754326662	-0.529586922	0.596398363	0.934971306
LOC100630923	6.408253866	0.39983853	0.755257354	0.529407	0.596523144	0.935005322
SEMA6D	52.35031375	0.314471502	0.594029021	0.529387438	0.596536711	0.935005322
MIB1	3105.157952	0.076049273	0.143673672	0.529319481	0.596583843	0.935005322
PTP4A3	46.13464908	0.320722966	0.606064084	0.529189857	0.596673752	0.935005322
MYLK2	3.279792843	-0.57266642	1.082561385	-0.5289921	0.59681093	0.935005322
HESX1	20.69311526	0.287657708	0.543786793	0.528989875	0.596812473	0.935005322
LINC00211	2.926116792	0.622398203	1.176622524	0.528970158	0.596826151	0.935005322
C2orf81	33.22740689	-0.261483309	0.494343666	-0.528950459	0.596839817	0.935005322
COL4A2	2443.979299	-0.089756721	0.169758141	-0.52873294	0.596990723	0.935159519
FAM157A	17.58515693	-0.265319803	0.501953808	-0.528574142	0.597100902	0.935191836
LINC02478	2.363529236	0.699262524	1.323188303	0.528467885	0.597174631	0.935191836
NUDT17	65.88557239	0.164475791	0.311240042	0.528453183	0.597184833	0.935191836
LOC103611081	60.8252209	0.158689338	0.300354904	0.528339428	0.597263771	0.935191836
LOC100506281	6.582601298	-0.452686527	0.856833372	-0.528325042	0.597273753	0.935191836
FITM2	229.821187	-0.119703869	0.226690134	-0.528050633	0.597464194	0.93525312
LINC01204	181.5893536	-0.445172173	0.843127321	-0.528001124	0.597498556	0.93525312
DHDDS	667.8279505	0.072177744	0.136700355	0.527999681	0.597499557	0.93525312
MED17	364.5728511	-0.079827593	0.151198826	-0.527964368	0.597524067	0.93525312
SUGT1P4-STRA6LP	33.50406194	0.219244932	0.415322713	0.527890541	0.59757531	0.93525312
LOC105373759	29.53712306	-0.206322706	0.39097957	-0.527707129	0.597702625	0.935364793
FAM24B-CUZD1	3.172868243	0.696884185	1.321132391	0.527490045	0.597853328	0.935364793
TMEM260	439.5432901	-0.084976609	0.161120751	-0.527409461	0.597909275	0.935364793
MRPL32	167.1983911	0.106161566	0.201302386	0.52737361	0.597934166	0.935364793
PCDHA10	4.96636557	0.473630722	0.898154829	0.5273375	0.597959237	0.935364793
GIHCG	59.90540617	0.19548164	0.370697895	0.527334097	0.5979616	0.935364793
SUCNR1	127.7736596	0.612753095	1.162489577	0.527104163	0.598121256	0.935507377
APTX	243.7187596	0.089152072	0.169178561	0.526970269	0.598214235	0.935507377
UBL7-AS1	83.09001246	-0.117364905	0.222739518	-0.526915504	0.598252267	0.935507377
SH2B2	26.90175075	-0.233965593	0.444073929	-0.526861808	0.598289557	0.935507377
MAPKAPK2	1580.842374	0.079314599	0.150555677	0.526812409	0.598323865	0.935507377
PEX11G	2.890415138	-0.630876543	1.197679078	-0.526749239	0.598367738	0.935507377
CD3EAP	130.6121386	-0.134792474	0.255960358	-0.52661465	0.598461216	0.935569142
PLCD3	276.2665416	0.095701722	0.181768582	0.526503101	0.598538698	0.935569142
RCHY1	294.7565136	0.085369942	0.162233627	0.52621607	0.59873809	0.935569142
LOC100133315	71.3023719	-0.148039804	0.281352571	-0.526171856	0.598768806	0.935569142
RAB24	307.5610374	0.086475397	0.164365811	0.526115474	0.598807978	0.935569142
LARP1	7856.696979	-0.066162251	0.12576026	-0.52609824	0.598819952	0.935569142
CETP	4.477203729	-0.598722903	1.138098101	-0.526073194	0.598837353	0.935569142
TMC3-AS1	24.31684627	0.22976582	0.436815626	0.526001834	0.598886933	0.935569142
ABCA11P	76.29245096	-0.157593505	0.299612923	-0.525990347	0.598894914	0.935569142
PTK6	2.650913412	0.746328323	1.419393103	0.525808052	0.599021579	0.935569142
ZNF775	105.7882182	-0.128804934	0.244966184	-0.525806998	0.599022312	0.935569142
CYHR1	607.6196074	-0.087403528	0.166257311	-0.525712388	0.599088055	0.935569142
ANKRD13C	620.21619	0.090254263	0.171683967	0.525700006	0.59909666	0.935569142
MYCL	24.66411053	-0.287841406	0.547607638	-0.525634388	0.599142259	0.935569142
LOC100379224	14.35848405	-0.294052772	0.559783645	-0.525297183	0.599376616	0.935787649
RBPMS	418.7127136	0.113185972	0.215476615	0.525281929	0.599387218	0.935787649
RFTN2	223.9223585	0.120479697	0.229428042	0.52513065	0.599492371	0.935798795
SLC20A1	1490.534311	-0.109760227	0.209019101	-0.525120559	0.599499385	0.935798795
TMEM175	172.0717005	-0.091375037	0.174089024	-0.524875345	0.59966985	0.935982896
PACERR	11.67210125	-0.305431265	0.582232802	-0.524586152	0.599870916	0.936175752
MTBP	684.0653529	0.097209627	0.185335062	0.524507486	0.599925614	0.936175752
DLGAP1-AS2	4.486166717	-0.520884979	0.993162676	-0.524470957	0.599951015	0.936175752
PDK3	395.8153378	-0.080121088	0.152831009	-0.524246279	0.600107256	0.936297588
SOX21-AS1	66.61348658	-0.185831611	0.354500055	-0.524207567	0.600134178	0.936297588
PEX6	945.9163958	0.066721759	0.12739147	0.523753745	0.600449829	0.93649683
SH3GL1	910.9971662	-0.066151916	0.126306398	-0.523741603	0.600458275	0.93649683
COL12A1	37436.25012	-0.14427119	0.275466337	-0.523734375	0.600463303	0.93649683
ARMC8	462.217183	-0.081421342	0.1554668	-0.523721733	0.600472098	0.93649683
SCAMP1-AS1	47.80196471	0.147776949	0.282352672	0.523377193	0.600711792	0.936624479
AKR7A2P1	24.73642652	0.340805155	0.651285958	0.523280366	0.600779162	0.936624479

PRADC1	176.5611498	0.107886578	0.206196361	0.523222513	0.600819416	0.936624479
CPLANE2	90.08681579	-0.156788247	0.299691228	-0.523165952	0.600858773	0.936624479
HMCES	363.5306447	-0.084645112	0.161796619	-0.523157482	0.600864666	0.936624479
EPHA1	6.024726845	0.457310718	0.874297903	0.523060523	0.600932136	0.936624479
SCYL1	858.12743	-0.074043682	0.141563368	-0.523042667	0.600944562	0.936624479
GOLGA2P10	5.726882873	-0.59354753	1.134892288	-0.522998999	0.60097495	0.936624479
SHKBP1	893.0141503	-0.078484225	0.150115063	-0.522827109	0.601094573	0.936624479
SEMA4F	680.361333	0.070617888	0.135100442	0.522706564	0.60117847	0.936624479
ACIN1	2228.037964	-0.072265697	0.138275432	-0.522621378	0.601237761	0.936624479
DDTL	38.35734496	0.254105386	0.486311633	0.522515541	0.601311429	0.936624479
ALDH3A2	445.0300156	0.074208525	0.142042795	0.522437799	0.601365544	0.936624479
RREB1	1148.922945	0.056298844	0.107780515	0.52234714	0.601428653	0.936624479
SNORD50B	5.624703486	-0.512270763	0.980927107	-0.522231223	0.601509349	0.936624479
TFB2M	329.5708783	-0.101057303	0.193544603	-0.522139605	0.601573133	0.936624479
ZNF524	66.27283096	-0.165082491	0.316188256	-0.522101905	0.60159938	0.936624479
SLC25A26	136.3428513	-0.110423755	0.21154408	-0.521989343	0.601677751	0.936624479
SNORA94	55.0703441	0.161580737	0.309556243	0.521975379	0.601687473	0.936624479
DNAJC27	105.9974877	0.139927293	0.268101075	0.521919924	0.601726086	0.936624479
GNB2	1381.728021	-0.071112979	0.136256846	-0.521903894	0.601737247	0.936624479
MIRLET7G	2.345832156	0.784673704	1.503640815	0.521849165	0.601775355	0.936624479
MRPL39	257.1227714	0.093285097	0.178788554	0.521762133	0.601835958	0.936624479
C5orf15	906.5701512	0.060676578	0.116301664	0.521717198	0.601867249	0.936624479
SMIM2-AS1	3.695548613	-0.649297104	1.244568038	-0.521704788	0.601875891	0.936624479
SLC35A5	340.1285826	0.117807044	0.225848447	0.521619899	0.601935005	0.936624479
CHRM5	4.106997671	-0.630497609	1.208934144	-0.521531808	0.601996354	0.936624479
SNORD88B	3.599663625	0.755926914	1.450548113	0.521131914	0.60227488	0.936624479
DRG2	668.8271013	0.050073559	0.096120277	0.520946881	0.602403776	0.936624479
LOC100506422	1161.020714	-0.156545959	0.30050287	-0.520946635	0.602403947	0.936624479
GMPPA	622.3138036	0.05897429	0.113221216	0.520876668	0.60245269	0.936624479
RANBP2	3757.066615	-0.066967247	0.128569088	-0.520865849	0.602460227	0.936624479
COQ3	172.5729542	-0.123686919	0.237517323	-0.520749043	0.602541605	0.936624479
GM2A	597.3474285	-0.099027349	0.190207047	-0.52062923	0.602625083	0.936624479
LINC01719	21.08058622	0.233704253	0.448888106	0.520629194	0.602625108	0.936624479
FAM24B	24.07785663	0.250196384	0.480581589	0.520611671	0.602637317	0.936624479
RITA1	520.2613918	-0.077434981	0.14875472	-0.520554781	0.602676957	0.936624479
MOB1A	3750.111765	-0.054719535	0.105124561	-0.52052094	0.602700537	0.936624479
WDHD1	1618.685012	0.04994684	0.095962347	0.520483728	0.602726466	0.936624479
ZNF732	28.53749555	0.220485998	0.423629925	0.52046842	0.602737133	0.936624479
CCDC32	312.7064777	-0.079918442	0.153571234	-0.520399819	0.602784936	0.936624479
ASB9P1	5.224705903	0.474779686	0.912398495	0.52036439	0.602809625	0.936624479
PCSK1N	24.03059816	-0.214234119	0.411721936	-0.520336909	0.602828775	0.936624479
CHCHD1	250.2409977	0.086771588	0.166796778	0.520223405	0.602907874	0.936624479
FBXL19	606.2802643	0.084095758	0.161658192	0.520207213	0.602919158	0.936624479
CCDC96	4.972898592	-0.584416395	1.123665107	-0.520098374	0.602995012	0.936660662
ITPKC	298.1958986	-0.102979407	0.198107169	-0.519816661	0.603191366	0.93685662
MRPL49	584.1954836	-0.084512389	0.162627267	-0.519669247	0.603294125	0.93685662
NAE1	1097.407773	-0.071912603	0.138393049	-0.519625826	0.603324394	0.93685662
DNAJC22	54.0533259	-0.2075444	0.399419036	-0.519615694	0.603331458	0.93685662
NFASC	5.149063121	-0.514517904	0.990466086	-0.519470491	0.603432686	0.936932165
EFCAB1	35.11462613	0.238265395	0.458888926	0.519222369	0.603605682	0.937038265
UTP6	1133.576297	-0.072033847	0.138746292	-0.519176739	0.603637498	0.937038265
LRRC37A4P	112.8285116	-0.158964048	0.306320728	-0.518946429	0.6037981	0.937038265
MTX1	478.2464538	0.067502005	0.13008443	0.518909183	0.603824074	0.937038265
NDUFB2-AS1	10.78510646	0.332528897	0.640850962	0.518886476	0.603839909	0.937038265
CASTOR3	54.88016235	-0.207471015	0.399923345	-0.518776955	0.60391629	0.937038265
GPR176	1226.485984	-0.088132293	0.169920428	-0.518668028	0.603992261	0.937038265
MOAP1	162.1241241	-0.142635897	0.275007533	-0.518661783	0.603996617	0.937038265
MALINC1	18.28228368	-0.256547812	0.494669771	-0.518624397	0.604022692	0.937038265
CDKL1	296.4700833	0.098079475	0.189173854	0.518462107	0.604135892	0.937038265
RNASEK	80.77904797	0.133781021	0.258131696	0.518266539	0.604272315	0.937038265
TUBA3FP	7.298531907	0.600054103	1.157821645	0.518261259	0.604275999	0.937038265
MGAT5	1370.705447	0.094326355	0.182009412	0.518249878	0.604283938	0.937038265
SRSF9	1135.737192	-0.071278426	0.137551577	-0.518194174	0.604322799	0.937038265
JPH1	184.3717954	0.119003044	0.229659837	0.518170897	0.604339039	0.937038265
ZNF433	23.93168716	-0.20269539	0.391228046	-0.518100356	0.604388252	0.937038265
PHRF1	940.6271685	0.102175301	0.19721997	0.518077865	0.604403943	0.937038265
OVOS2	33.2532733	0.198125982	0.382471219	0.518015403	0.604447523	0.937038265
ANGPTL1	63.24384794	-0.19139679	0.369557851	-0.517907519	0.604522796	0.937045109
TRMT6	237.6156969	0.110037458	0.212490658	0.517846096	0.604565654	0.937045109
GALNTL6	40.82850168	0.18957139	0.366121318	0.517782988	0.604609689	0.937045109
MIR1305	5.243988055	0.604732638	1.168172689	0.517674009	0.604685736	0.937081469
CCDC71L	301.9848641	-0.110834777	0.214216773	-0.517395421	0.604880155	0.937142858
C12orf40	4.768379926	-0.537845298	1.039757279	-0.517279666	0.604960946	0.937142858
DNM1P41	15.32834582	-0.294238778	0.568906636	-0.517200468	0.605016226	0.937142858
C5orf34	208.4557891	0.099891454	0.193156349	0.517153357	0.605049109	0.937142858
PRKCE	365.8943781	0.065412738	0.12649709	0.517108639	0.605080324	0.937142858
PACRGL	296.1836044	0.117783705	0.227773842	0.517108128	0.60508068	0.937142858
NEK3	194.930041	0.084113757	0.1626676	0.517089798	0.605093475	0.937142858
FMO6P	4.66358644	0.873671215	1.690231741	0.516894337	0.605229921	0.937193997
SOAT1	1680.866628	-0.063254109	0.12237398	-0.516891819	0.605231679	0.937193997
TSG101	305.4040825	-0.102639487	0.198732293	-0.516471105	0.605525415	0.937395479
MTFR2	134.4900436	0.113455515	0.219702462	0.516405297	0.605571367	0.937395479
MIR335	2.678919279	0.679646159	1.316311461	0.516326249	0.605626566	0.937395479
SNORD170	5.360050649	0.534433358	1.035303034	0.516209593	0.605708031	0.937395479
LOC100131635	3.470016611	0.767449424	1.486774797	0.516184042	0.605725875	0.937395479
RET	2.198200757	0.846864876	1.64085427	0.516112181	0.605776061	0.937395479
LOC100505912	3.901577719	-0.571311803	1.107007294	-0.516086756	0.605793817	0.937395479
LOC646214	4.358760814	-0.498657645	0.966261691	-0.516068939	0.605806261	0.937395479
MAP2K2	1250.121331	-0.077676199	0.150527258	-0.516027466	0.605835226	0.937395479
PSTPIP1	4.947614923	0.584107857	1.132379513	0.515823405	0.605977754	0.937534604

NR2F2-AS1	3.151717423	0.564635019	1.094958525	0.515667952	0.606086342	0.937621201
TBL3	817.1952115	0.087091492	0.168954462	0.51547317	0.606222413	0.937707761
MFSD14A	1002.391392	0.06348114	0.123163112	0.515423315	0.606257243	0.937707761
LIMS2	9.210525344	0.517197609	1.003562001	0.51536189	0.606300158	0.937707761
TRUB1	327.4588622	-0.09573979	0.185846883	-0.515154134	0.606445317	0.937724227
FIG4	186.9430266	-0.116093683	0.225425304	-0.514998455	0.606554099	0.937724227
SPTBN2	650.0765171	-0.070878839	0.137649435	-0.514922852	0.606606932	0.937724227
LOC105376731	3.236152163	0.559750454	1.087128474	0.514888964	0.606630613	0.937724227
MTCP1	39.28777793	0.18697649	0.363151737	0.514871527	0.606642798	0.937724227
SND1	7448.251838	-0.074052591	0.143869786	-0.514719543	0.606749015	0.937724227
KRR1	755.8448023	0.061686711	0.119878209	0.514578184	0.606847813	0.937724227
TASOR	2804.407057	0.071648718	0.139241986	0.5145626	0.606858706	0.937724227
TM2D2	772.3251216	0.067684866	0.131541154	0.514552775	0.606865573	0.937724227
BABAM2-AS1	38.67088022	0.241942146	0.470242691	0.514504852	0.606899069	0.937724227
USP51	32.21108224	-0.194447153	0.377967962	-0.51445406	0.606934572	0.937724227
DSTN	2173.330218	0.086360788	0.167931375	0.514262379	0.607068561	0.937724227
SPDYE6	25.98660872	0.349458726	0.679615723	0.514200473	0.607111837	0.937724227
POLD4	75.49253663	-0.178496774	0.347255497	-0.514021449	0.607236995	0.937724227
ALDH6A1	435.0414627	-0.169882612	0.330505111	-0.514009031	0.607245677	0.937724227
HAS2-AS1	111.7466691	-0.155942573	0.303518506	-0.513782751	0.607403889	0.937724227
CEP128	427.1220565	0.148187125	0.288454793	0.513727378	0.607442607	0.937724227
KRCC1	251.9774808	-0.150940085	0.293898962	-0.513578149	0.60754696	0.937724227
PDE8B	34.47566675	-0.245010687	0.477070463	-0.513573372	0.6075503	0.937724227
MAP3K14	163.9770983	0.148567668	0.289302755	0.513536997	0.607575738	0.937724227
SNAPC1	420.2010553	-0.086435862	0.168315636	-0.513534357	0.607577584	0.937724227
JARID2-AS1	14.77430458	0.32662008	0.636051654	0.513511879	0.607593304	0.937724227
FAAH	13.91416338	-0.311021143	0.605685377	-0.51350281	0.607599646	0.937724227
LOC101927357	8.905595532	-0.38403934	0.748266659	-0.51323861	0.607784421	0.937724227
OGT	3634.719608	-0.115615471	0.225270157	-0.513230305	0.607790229	0.937724227
ZNF707	283.5763669	-0.127036265	0.247564005	-0.513145133	0.607849802	0.937724227
CIITA	76.90517224	0.27424747	0.534502714	0.513089014	0.607889056	0.937724227
MATN4	6.137858485	0.492977268	0.960875833	0.513049919	0.607916403	0.937724227
ASTL	2.447173164	0.668744453	1.303545143	0.513019788	0.607937479	0.937724227
LOC102725191	15.01160684	0.416752828	0.812370513	0.513008314	0.607945505	0.937724227
STX10	562.6459488	-0.073503326	0.143301778	-0.512926828	0.608002506	0.937724227
KCNMB2-AS1	20.97373744	0.387594409	0.755666676	0.512917166	0.608009265	0.937724227
C6orf132	9.41390172	0.330144612	0.643895017	0.512730496	0.608139854	0.937724227
STK3	530.0864374	0.064230553	0.125295089	0.512634244	0.608207195	0.937724227
ZNF32-AS2	30.95308125	0.196653584	0.383756715	0.51244337	0.608340744	0.937724227
MAN2B1	1314.778611	0.076632918	0.149553732	0.512410601	0.608363673	0.937724227
SLC35E4	74.9063383	0.223230177	0.435701367	0.512346744	0.608408356	0.937724227
MIR4720	15.3183958	0.261819605	0.51120532	0.512161347	0.608538092	0.937724227
ADIPOR1	833.4655591	0.08521103	0.166376692	0.512157253	0.608540958	0.937724227
TEX9	78.34594686	0.184090383	0.35947816	0.512104499	0.608577876	0.937724227
LOC101928335	2.37886116	0.7669539	1.498083734	0.51195663	0.608681363	0.937724227
ABCC6P1	2.016139956	0.731123982	1.428540157	0.51179799	0.608792397	0.937724227
PRRC2C	4650.405051	0.049466232	0.09666769	0.511714223	0.60885103	0.937724227
CBLN3	6.005557981	0.457261322	0.89379446	0.51159561	0.608934058	0.937724227
MARCHF10	7.015781175	-0.481167537	0.940616345	-0.511544945	0.608969525	0.937724227
LOC105369140	9.419873345	-0.356282368	0.696498245	-0.511533763	0.608977353	0.937724227
DMAC2L	179.9608118	-0.090550803	0.177023393	-0.511518851	0.608987792	0.937724227
CCDC153	3.35998902	0.682481685	1.334323763	0.511481324	0.609014062	0.937724227
STAG3L5P-PVRIG2P-PILRB	243.6553861	0.177897041	0.347813871	0.511471955	0.609020621	0.937724227
STXBP4	521.208659	0.093742766	0.183289857	0.511445464	0.609039166	0.937724227
TNPO3	2448.027111	-0.058424815	0.114238382	-0.511428945	0.609050731	0.937724227
COX7C	791.5849782	0.089623978	0.175279446	0.51132052	0.609126638	0.937724227
ACE	5.116902021	-0.5264475	1.029806307	-0.511210211	0.609203869	0.937724227
SNORA92	3.544250883	-0.515554132	1.008592489	-0.511161978	0.60923764	0.937724227
EXOC6B	1109.941142	0.070198011	0.13736935	0.511016551	0.609339467	0.937724227
EXO1	589.2034626	-0.133613241	0.261545342	-0.510860716	0.60944859	0.937724227
BANF1	868.2080357	-0.07868064	0.154022487	-0.510838657	0.609464038	0.937724227
SNTB2	2527.759918	-0.076219594	0.149249133	-0.510687015	0.609570235	0.937724227
KIFC2	444.4327249	0.099365511	0.194584262	0.510655432	0.609592353	0.937724227
CCDC160	1.911450436	0.767497865	1.503292997	0.510544429	0.609670097	0.937724227
GPRIN1	185.2704622	-0.149189101	0.292235318	-0.510510167	0.609694094	0.937724227
MORC2	932.8113988	0.054189755	0.106164672	0.510431146	0.609749441	0.937724227
PECAM1	1.978549144	0.764334815	1.497496693	0.510408349	0.609765409	0.937724227
EFNA3	25.02023216	0.287556048	0.563412794	0.510382531	0.609783493	0.937724227
MRPS22	521.6415593	0.062548014	0.122551541	0.510381292	0.609784361	0.937724227
PARP14	625.6260807	-0.121340715	0.237754658	-0.510361041	0.609798546	0.937724227
METRN	453.2547549	0.074683771	0.146350702	0.510306886	0.609836479	0.937724227
SNORD139	15.25787874	0.29609201	0.580328315	0.510214653	0.609901087	0.937742656
ZSCAN2	223.3847892	-0.105469675	0.206825788	-0.509944509	0.610090338	0.937952707
NBAT1	27.29539222	0.192189151	0.376957052	0.509843629	0.610161017	0.937960564
TMEM258	221.1317302	-0.137941831	0.270631916	-0.509702749	0.610259727	0.937960564
BMS1P20	319.6359493	0.090914696	0.178393331	0.509630576	0.610310299	0.937960564
IGSF9	27.54589089	-0.182844088	0.358850627	-0.509527014	0.610382868	0.937960564
ZBTB7B	515.319718	0.069980054	0.137360068	0.509464319	0.610426802	0.937960564
FLJ31104	6.3616458	0.423506185	0.831496778	0.509329917	0.610520991	0.937960564
ZNNT1	80.83125763	0.194692888	0.382256646	0.509325056	0.610524398	0.937960564
LRRC31	3.662767441	-0.518410496	1.017963064	-0.50926258	0.610568184	0.937960564
PPARGC1A	11.96444198	0.376740973	0.739782453	0.509259136	0.610570597	0.937960564
MIR623	4.378397383	-0.687530117	1.350327239	-0.509158149	0.610641376	0.937960564
ACP6	179.4621023	0.113380654	0.222797601	0.508895309	0.610825608	0.937960564
TTI1	711.2354222	-0.080862389	0.158953254	-0.508718047	0.61094987	0.937960564
HSPA12A	5.670715734	-0.381092653	0.749139378	-0.508707277	0.610957421	0.937960564
LYAR	139.8202118	0.115769455	0.227589031	0.508677655	0.610978187	0.937960564
FAM49B	1879.126016	-0.0599168	0.11779635	-0.508647338	0.610999441	0.937960564
NET1	934.3117227	-0.130982083	0.257563311	-0.508543248	0.611072417	0.937960564
GYG1	441.9864116	0.082924293	0.163125468	0.508346698	0.611210227	0.937960564

CTNND1	125.0837882	-0.107952129	0.212365668	-0.50833136	0.611220981	0.937960564
SOHLH2	2.450010835	0.628229318	1.235866512	0.508331047	0.6112212	0.937960564
ARID1B	1216.146409	-0.057169639	0.112473389	-0.508294798	0.611246617	0.937960564
MIR137HG	4.571687284	-0.525575103	1.034101865	-0.508243067	0.611282892	0.937960564
BCORL1	325.7868721	-0.072144232	0.141952698	-0.508227268	0.61129397	0.937960564
MIR154	3.596202155	-0.613301442	1.206787371	-0.508210027	0.61130606	0.937960564
BIRC6	5802.305782	0.0727214	0.143168223	0.507943724	0.61149281	0.937996936
SGO1	191.9983784	0.096652309	0.190303544	0.507884969	0.611534016	0.937996936
CPSF4	479.4452501	0.069267905	0.136443132	0.507668681	0.611685716	0.937996936
ROMO1	183.6303093	-0.12580266	0.247828381	-0.507620067	0.611719815	0.937996936
INTS6-AS1	30.24102112	0.221369645	0.436111609	0.507598608	0.611734867	0.937996936
CPSF1	2587.4367	-0.047127551	0.092845106	-0.507593272	0.61173861	0.937996936
VAMP3	1251.902082	0.074477526	0.146730453	0.507580561	0.611747526	0.937996936
PDE4C	4.102634529	-0.501426235	0.98793502	-0.507549814	0.611769094	0.937996936
NDRG2	41.68197453	-0.168608766	0.332264042	-0.507454146	0.611836202	0.937996936
RTRAF	1793.884251	-0.06774888	0.133541573	-0.507324262	0.611927318	0.937996936
NELL2	7.612176844	-0.363016627	0.715555832	-0.507321177	0.611929482	0.937996936
CERCAM	2156.768866	-0.09567733	0.188611379	-0.507272311	0.611963764	0.937996936
PAN3-AS1	29.52662647	0.294342694	0.580366018	0.507167348	0.612037403	0.937996936
HGD	5.078368149	0.460021755	0.907178232	0.507090822	0.612091095	0.937996936
ACSS2	628.0201159	0.170453521	0.336166697	0.507050587	0.612119325	0.937996936
TCOF1	1908.558301	0.062099675	0.122536561	0.506784874	0.612305771	0.938201964
RNASEL	215.7331008	0.105865869	0.20896371	0.506623225	0.612419209	0.9382951
PHC2-AS1	16.82922309	0.341044716	0.673332853	0.506502415	0.612503995	0.938344325
NRG2	5.232597741	-0.482740447	0.953406308	-0.50633234	0.612623364	0.938446518
CFLAR	356.6160412	0.10320982	0.203903228	0.506170605	0.612736889	0.938539742
MED10	229.3488057	-0.082233806	0.162580312	-0.505804207	0.612994105	0.938635999
FAF1	1384.641468	-0.059394921	0.117435297	-0.505767197	0.613020089	0.938635999
LINC00899	4.541344129	0.514408252	1.017298558	0.505661045	0.613094619	0.938635999
MIR4516	7.162203139	0.409719266	0.810308453	0.505633706	0.613113815	0.938635999
OR51B4	2.000696838	0.68493043	1.35460094	0.505632626	0.613114573	0.938635999
SLC1A2	3.477441597	0.493288889	0.975590828	0.505630921	0.613115771	0.938635999
AKT2	1670.350986	-0.049209798	0.097343994	-0.505524744	0.613190324	0.938669493
TOP1MT	464.5675755	-0.065667028	0.129953462	-0.50531188	0.6133398	0.93875239
HPS1	297.9643153	-0.09918277	0.196285857	-0.505297589	0.613349836	0.93875239
CCDC69	14.17832789	-0.265990101	0.526653738	-0.505056895	0.613518876	0.93892464
GTF2H2B	16.56755288	-0.299460345	0.59307659	-0.504926935	0.613610155	0.93892464
PUS7L	1223.298503	0.055528641	0.109976808	0.504912283	0.613620446	0.93892464
ZMPSTE24	1010.171617	-0.060333086	0.119550364	-0.504666684	0.613792964	0.939058485
MIR378H	3.126496541	-0.649515912	1.287600038	-0.504439183	0.613952789	0.939058485
LLGL2	4.450313581	-0.503293032	0.997961336	-0.504321173	0.614035701	0.939058485
LINC01578	429.7427576	-0.106076708	0.210347807	-0.504291958	0.614056228	0.939058485
LOC101241902	8.49690335	0.338853528	0.672002355	0.504244554	0.614089535	0.939058485
MDM4	2247.898602	0.106058425	0.210335555	0.504234413	0.614096661	0.939058485
STIM1	945.2902398	0.072770102	0.144329424	0.504194502	0.614124704	0.939058485
ERVW-1	5.725335132	0.46399392	0.920280154	0.504187684	0.614129494	0.939058485
ZNF347	81.074239	-0.182841245	0.36276019	-0.504027868	0.614241794	0.939139156
ARAP1	1408.674243	0.083008451	0.164713903	0.50395534	0.614292761	0.939139156
BTBD9	209.2653075	-0.09057683	0.179756017	-0.503887613	0.614340356	0.939139156
ZSCAN23	138.7633411	0.152230917	0.302192261	0.50375518	0.614433427	0.939150174
SNRNP25	131.0168083	-0.157158319	0.311990824	-0.503727377	0.614452967	0.939150174
RAPGEF1	1096.436528	0.106098831	0.210676545	0.503610075	0.614535411	0.939175896
USP30	298.4450706	-0.106410914	0.211322633	-0.503547172	0.614579624	0.939175896
TENT4B	374.002079	0.085142003	0.169120083	0.503441114	0.614654172	0.939175896
DHRS7	822.1845063	-0.097452323	0.193597819	-0.503375108	0.614700569	0.939175896
LINC02693	3226.873131	-0.083863558	0.166617931	-0.503328526	0.614733314	0.939175896
GIGYF2	1287.64583	0.041999341	0.083458647	0.503235343	0.614798819	0.939195452
WDR46	995.6100765	-0.055583953	0.11049264	-0.503055707	0.614925107	0.939307852
MTIF3	92.82760931	0.106427904	0.211606039	0.502953053	0.61499728	0.939337578
MAGEA2	16.24789617	-0.277666232	0.552275843	-0.502767295	0.61512789	0.939456548
GAS2L1	203.1574966	-0.098046632	0.195061259	-0.502645336	0.615213649	0.939507004
IFIT2	36.00052024	-0.184113643	0.366464741	-0.502404796	0.615382806	0.939571363
SNORD114-7	17.98464689	0.305204505	0.607662042	0.502260276	0.615484448	0.939571363
OXER1	15.31344404	0.250011186	0.497804618	0.502227536	0.615507476	0.939571363
UCN	11.22021439	-0.356732871	0.710333252	-0.502204943	0.615523366	0.939571363
SACM1L	546.7513272	0.050825886	0.1012243	0.502111509	0.615589085	0.939571363
AKR7A3	11.92218645	-0.312723108	0.6228781	-0.502061491	0.615624267	0.939571363
MIR641	12.3760214	-0.294268823	0.586248662	-0.501952229	0.615701125	0.939571363
SLC46A1	132.9738522	-0.123673208	0.246468784	-0.50178041	0.615821995	0.939571363
CMTM4	908.4030904	0.066487868	0.132506132	0.501772012	0.615827902	0.939571363
PIP4K2B	2158.851415	0.062224252	0.124013483	0.501753926	0.615840626	0.939571363
TSLP	25.3176779	-0.26430274	0.526865576	-0.501651185	0.615912908	0.939571363
VPREB3	2.354110622	0.729393437	1.454293246	0.501544952	0.61598765	0.939571363
UBL3	507.6404283	0.090590684	0.180678765	0.501390875	0.616096061	0.939571363
PSRC1	238.8490567	-0.074830757	0.149249154	-0.50138145	0.616102692	0.939571363
LOC100128076	7.31410633	-0.319851899	0.638065328	-0.501283937	0.616171309	0.939571363
FAM234A	966.556463	-0.118760082	0.236918445	-0.501269888	0.616181194	0.939571363
NFE2L1	5133.359799	-0.065966771	0.131612499	-0.501219654	0.616216544	0.939571363
MANCR	308.8741642	0.113788784	0.22703063	0.501204544	0.616227177	0.939571363
WNT3	183.1306173	0.086280618	0.172192196	0.501071596	0.616320736	0.939571363
TAPT1-AS1	39.68527628	-0.19474628	0.388747964	-0.500957685	0.616400903	0.939571363
ZNF335	424.3660172	0.135245574	0.270007596	0.500895443	0.61644471	0.939571363
NINJ2	12.26700904	-0.274233467	0.547591482	-0.500799365	0.616512332	0.939571363
SCARB1	3559.273407	0.064495683	0.128817236	0.500675881	0.616599249	0.939571363
ATP13A2	476.9050305	0.055232873	0.110328223	0.500623247	0.616636299	0.939571363
GEMIN6	97.80557826	0.111574977	0.222874084	0.500618894	0.616639363	0.939571363
GPC5	16.82427229	0.25110158	0.501864233	0.500337668	0.616837335	0.939571363
DDX42	1705.46567	-0.087750536	0.175442577	-0.500166708	0.616957698	0.939571363
SMIM26	93.49710292	-0.13516799	0.270357615	-0.49995999	0.61710325	0.939571363
MOK	108.3190435	-0.188413723	0.376879911	-0.499930395	0.61712409	0.939571363

OST4	864.7107677	-0.103956269	0.207945754	-0.499920133	0.617131315	0.939571363
DNAJC4	75.25917794	0.162136108	0.324342266	0.499892011	0.617151118	0.939571363
TMEM189	314.478317	-0.073526256	0.147130184	-0.499736041	0.617260951	0.939571363
PKP4	687.9695648	0.058787224	0.117641039	0.499716971	0.617274381	0.939571363
RPA4	6.07685823	0.602954464	1.206609785	0.499709576	0.617279589	0.939571363
STAP2	29.42909764	0.196769579	0.393815273	0.499649437	0.617321941	0.939571363
ADAM11	198.1224645	0.106305942	0.212806795	0.499542046	0.617397574	0.939571363
FCGR2A	3.199769904	0.625635132	1.252431119	0.499536559	0.617401438	0.939571363
TEX38	2.798812928	-0.58842527	1.17797411	-0.499523092	0.617410923	0.939571363
BNIPL	9.818341548	0.32024502	0.641146735	0.499487875	0.617435726	0.939571363
RAMP2	2.27883845	0.769970166	1.541589425	0.499465132	0.617451745	0.939571363
LINC01208	19.02398344	0.239772855	0.480059902	0.49946445	0.617452225	0.939571363
DYRK1B	55.55927351	-0.322039404	0.644908884	-0.499356439	0.617528301	0.939571363
MCF2L2	9.977213201	-0.339043952	0.679090989	-0.499261451	0.617595209	0.939571363
C2orf49	357.6582711	-0.084966847	0.170204366	-0.499204862	0.61763507	0.939571363
GALNT5	1310.612227	0.06882942	0.137887501	0.49917084	0.617659035	0.939571363
EFCAB5	3.37230326	-0.573145462	1.148429376	-0.499068967	0.617730799	0.939571363
EIF2AK3-DT	46.91976318	0.157648452	0.315946319	0.498972272	0.617798918	0.939571363
LOC101927972	4.945840474	0.722149474	1.447396362	0.498930005	0.617828695	0.939571363
LINC00167	4.805504263	0.424732717	0.851312987	0.49891488	0.617839351	0.939571363
SF3A2	578.1342037	-0.05883942	0.117986553	-0.498695983	0.617993575	0.939584177
CERNA1	2.515403784	0.706895325	1.417641748	0.498641724	0.618031806	0.939584177
FAM131B	10.41886734	-0.350325043	0.702624746	-0.498594798	0.618064871	0.939584177
ZNF257	15.34215176	-0.272481309	0.54654606	-0.49855141	0.618095443	0.939584177
FH	980.7938906	-0.06753906	0.135497282	-0.498453243	0.618164617	0.939584177
MIR499A	2.947155238	-0.615179353	1.234362966	-0.498378005	0.618217636	0.939584177
CBX7	8.247326041	-0.406197993	0.815041437	-0.498377107	0.61821827	0.939584177
B3GALT5	9.171233441	-0.489623533	0.982579415	-0.498304285	0.618269588	0.939584177
FAM210A	542.199473	-0.069178227	0.138912853	-0.497997307	0.618485941	0.939752264
NAXD	539.5765688	-0.070956474	0.142505935	-0.497919433	0.61854083	0.939752264
MRPL45P2	17.32054085	0.25194016	0.506112215	0.497795059	0.618628499	0.939752264
FGD4	27.73112305	0.224792295	0.451587935	0.497781888	0.618637783	0.939752264
LOC646588	15.29034004	-0.260010572	0.522384265	-0.497738139	0.618668623	0.939752264
OSBPL9	1554.074333	0.05991962	0.120393426	0.497698439	0.618696609	0.939752264
C1orf74	99.27394766	-0.104957703	0.210975058	-0.497488679	0.618844484	0.939839724
APOBEC4	6.556449212	-0.485728177	0.976402537	-0.497467139	0.61885967	0.939839724
AEBP1	1173.066864	-0.096239054	0.193512649	-0.497326944	0.618958514	0.939904415
MIPOL1	220.0054506	0.125895095	0.253179082	0.497257097	0.619007762	0.939904415
CARMIL1	290.0855253	0.112230483	0.225750564	0.497143755	0.619087681	0.939904415
ZNF391	340.8891044	0.095147119	0.191401495	0.497107502	0.619113245	0.939904415
CHST12	304.7800672	0.082565968	0.166153571	0.496925631	0.619241496	0.93996394
KIF6	6.612015106	0.42213265	0.849669156	0.496820023	0.619315974	0.93996394
AP4B1	374.9754574	0.08315265	0.167402243	0.49672363	0.619383957	0.93996394
LEPROT	831.4785871	-0.088379129	0.177960191	-0.496623029	0.61945491	0.93996394
BPGM	254.7028102	-0.099909198	0.201253511	-0.49643456	0.619587847	0.93996394
LDLRAD4	5.877768356	-0.612807991	1.234522644	-0.49639267	0.619617396	0.93996394
ONECUT1	4.588381721	0.487829707	0.98282727	0.496353451	0.619645061	0.93996394
FAM198B-AS1	8.304238884	-0.332621952	0.670132187	-0.496352747	0.619645557	0.93996394
MRPL42P5	4.656343315	0.464074674	0.935082314	0.496292857	0.619687805	0.93996394
KIAA0753	368.5506172	-0.123294474	0.248437086	-0.49628047	0.619696543	0.93996394
ASIP	15.77975376	-0.259324486	0.522590088	-0.496229247	0.619732679	0.93996394
LOC100129931	9.565596525	-0.332305406	0.669817186	-0.496113587	0.619814274	0.939966823
MIR145	10.06689348	0.461830634	0.930965595	0.496077016	0.619840075	0.939966823
FOXQ1	382.9596899	-0.44059392	0.888406068	-0.49593754	0.619938479	0.93999727
SNORD67	5.033688902	-0.464892203	0.937543875	-0.49586181	0.619991912	0.93999727
TRIM69	10.94814844	0.374065301	0.754456022	0.495807959	0.620029909	0.93999727
SLC7A10	2.935297103	-0.716203615	1.44492412	-0.495668669	0.620128196	0.93999727
TMEM256-PLSCR3	6.004077263	0.43002845	0.867744124	0.49557057	0.620197421	0.93999727
THOC7	273.2622842	-0.07675361	0.15488809	-0.495542364	0.620217325	0.93999727
MIR103A2	2.413148835	0.696425265	1.405428397	0.495525255	0.620229399	0.93999727
SNORD12	25.18306787	0.268251623	0.541619764	0.495276652	0.620404849	0.940172708
MTCH2	921.8780004	0.078999491	0.159526697	0.495211723	0.620450676	0.940172708
UBAC2-AS1	31.02756012	-0.185420086	0.374525514	-0.495079986	0.62054366	0.940233656
KLK14	2.92896292	-0.666377091	1.346341187	-0.4949541	0.620632521	0.940288345
GPLD1	35.12628448	0.244534544	0.494603356	0.494405348	0.621019938	0.940717778
MIR545	4.156569225	-0.61378814	1.241664702	-0.494326801	0.6210754	0.940717778
SETX	2644.337627	-0.067692197	0.136955387	-0.494264584	0.621119334	0.940717778
UC.134	6.596316197	0.559531063	1.132072929	0.494253549	0.621127126	0.940717778
EFCAB14	1242.492945	-0.073011405	0.14775674	-0.494132488	0.621212615	0.940722444
PTX4	2.447675448	-0.634918324	1.285096797	-0.494062646	0.621261938	0.940722444
CNST	645.3497675	0.053976545	0.109258748	0.494024925	0.621288577	0.940722444
LPP-AS1	15.91953503	0.40823592	0.826649866	0.493843811	0.62141649	0.94083618
GPR82	9.647815623	0.375525163	0.760862847	0.493551714	0.621622809	0.941068597
APEX2	344.2148061	0.073667582	0.149340748	0.493285209	0.621811078	0.941150592
TTL	2094.35572	-0.062817697	0.127350056	-0.493267919	0.621823293	0.941150592
SMIM12	444.8252554	0.080243413	0.16269433	0.493215793	0.62186012	0.941150592
NAMPT	1155.159463	-0.070221769	0.14238683	-0.493176009	0.621888228	0.941150592
PDIA4	3422.984734	-0.058623455	0.118907643	-0.493016711	0.62200078	0.94120769
SSC4D	18.23767657	-0.236825935	0.48040336	-0.492973104	0.622031592	0.94120769
NFIA-AS1	3.752630694	-0.716838945	1.454765578	-0.492752204	0.622187686	0.941363947
MIR12122	7.407306213	0.385801867	0.783184786	0.492606437	0.622290699	0.941377101
PUS3	113.5987796	0.101560718	0.206188533	0.492562397	0.622321823	0.941377101
FAM86C2P	38.88620333	-0.153285829	0.311230373	-0.49251565	0.622354861	0.941377101
SH3PXD2A-AS1	6.555308312	0.428133089	0.869466113	0.492409172	0.622430117	0.941411023
PPP1R12C	655.2554682	-0.094586719	0.192160997	-0.492226416	0.622559292	0.941450065
LINC02029	2.590735185	0.566770368	1.151450016	0.492223163	0.622561592	0.941450065
TCEAL3	36.17845536	-0.190454586	0.387171344	-0.49191292	0.622780905	0.941622817
ZNF43	46.37659382	0.159246028	0.323809762	0.491788842	0.622868625	0.941622817
HBE1	10.30475187	-0.311733134	0.634028909	-0.491670221	0.622952493	0.941622817
LINC01684	75.16140532	0.16139722	0.328273664	0.491654487	0.622963618	0.941622817

NFKB2	230.8907127	-0.113092243	0.230075596	-0.491543843	0.623041851	0.941622817
TSNARE1	83.48980584	-0.121965731	0.248148077	-0.491503831	0.623070143	0.941622817
ICE2	1792.052695	0.067735369	0.137823469	0.49146469	0.62309782	0.941622817
STRN4	1725.88425	-0.056758879	0.115527614	-0.491301407	0.623213285	0.941622817
SLC25A21-AS1	57.98236349	0.119762065	0.243781409	0.491268244	0.623236737	0.941622817
FKBP1B	79.06744709	0.157923885	0.321534056	0.49115757	0.623315006	0.941622817
PARP9	66.2815422	-0.177641447	0.361795052	-0.491000212	0.623426297	0.941622817
MIR570HG	69.9887734	0.177511054	0.361538608	0.490987823	0.62343506	0.941622817
SLC25A4	113.4048883	-0.16637686	0.338902437	-0.490928485	0.623477029	0.941622817
MAML2	578.6796845	0.107380266	0.218735356	0.490914077	0.62348722	0.941622817
LOC105369201	7.665746589	0.323965554	0.659925948	0.490911981	0.623488703	0.941622817
COPS9	75.13985409	-0.127032016	0.258791678	-0.490865921	0.623521282	0.941622817
LOC643339	2.410559368	-0.745703666	1.519430801	-0.4907783	0.623583259	0.941636614
CLIP1	612.1831133	0.065978488	0.134488351	0.490588872	0.623717258	0.941759155
MTHFR	166.2927495	-0.100169212	0.204276866	-0.490360038	0.623879149	0.941856962
NBAS	2217.892557	0.072326503	0.147513388	0.490304672	0.623918322	0.941856962
MAPK14	1839.120771	-0.053331678	0.10880139	-0.490174602	0.624010351	0.941856962
CFAP44	156.2094014	0.182990563	0.373340371	0.490144052	0.624031967	0.941856962
SCN3A	17.8158082	-0.267046374	0.544854953	-0.49012379	0.624046305	0.941856962
DAND5	4.422824851	0.484922243	0.990195349	0.489723814	0.624329348	0.942086137
MIDEAS	1354.974473	-0.045122886	0.092151264	-0.48966107	0.624373754	0.942086137
PGBD2	82.16254301	0.139531544	0.285089472	0.489430716	0.624536795	0.942086137
BRD8	887.1457918	-0.050390801	0.10296245	-0.489409498	0.624551813	0.942086137
NEPRO	582.9161715	-0.068438614	0.139844787	-0.489389814	0.624565746	0.942086137
STAT5A	50.92246848	-0.298158051	0.609283802	-0.489358244	0.624588093	0.942086137
LHX1-DT	2.99656886	0.626441415	1.280209856	0.48932713	0.624610117	0.942086137
ARHGAP33	179.7294733	0.175805695	0.359317973	0.489276097	0.624646241	0.942086137
SLC16A5	20.95712892	0.26645926	0.544642545	0.489236954	0.62467395	0.942086137
ASB16-AS1	86.00083015	0.202489678	0.414294096	0.4887583	0.625012823	0.942517434
LINC00645	154.8806106	0.407529307	0.834083459	0.488595358	0.6251282	0.942539975
ZC3H8	114.7002117	-0.14253413	0.291790691	-0.488480731	0.625209371	0.942539975
SNORD114-11	8.288967802	0.41687871	0.853600769	0.488376681	0.625283056	0.942539975
CYBA	59.37157953	-0.135791059	0.278052793	-0.488364305	0.625291821	0.942539975
ZBTB22	364.6751524	0.110519721	0.226306164	0.488363723	0.625292233	0.942539975
DOCK10	374.8708651	-0.076720464	0.157121619	-0.488287124	0.62534648	0.942542018
SERTAD1	69.33749935	0.155425718	0.318462062	0.488050968	0.62551374	0.942653161
PAQR8	430.4324067	0.166423311	0.341007872	0.488033634	0.625526018	0.942653161
ATG4C	267.5061912	-0.08124803	0.166541795	-0.487853696	0.625653474	0.942765509
LACTB2-AS1	52.21617364	0.315128563	0.64650998	0.487430315	0.625953415	0.942827845
LOC114841035	7.791214485	-0.340686616	0.699052684	-0.487354707	0.626006985	0.942827845
ZNF791	605.9594026	0.064031577	0.131401678	0.487296495	0.626048231	0.942827845
RBM14-RBM4	2.990811139	0.5810596	1.192416277	0.487295931	0.626048631	0.942827845
RAB40AL	3.732848592	-0.476616362	0.978371374	-0.487152808	0.626150046	0.942827845
STX1B	11.57428751	-0.338429191	0.694714078	-0.487148888	0.626152823	0.942827845
TCAF1	474.673919	0.100999176	0.207395448	0.486988394	0.626266556	0.942827845
GPR21	10.51482025	0.55004335	1.129555421	0.48695561	0.626289789	0.942827845
CREB3L2	3088.375589	0.117803286	0.241929389	0.486932517	0.626306155	0.942827845
SLC25A3	7911.369756	-0.084427575	0.173395123	-0.486908587	0.626323113	0.942827845
DHX32	400.9916476	-0.0724818	0.148889491	-0.486816092	0.626388666	0.942827845
MSRA	15.44383115	-0.286190973	0.587907922	-0.486795572	0.626403209	0.942827845
LINC02211	3.000485281	0.560995257	1.152547397	0.486743763	0.626439928	0.942827845
RNF11	991.7845324	-0.080463848	0.165325095	-0.486700754	0.626470411	0.942827845
ADCY4	4.74721556	-0.488820125	1.004407147	-0.486675276	0.626488469	0.942827845
TPTE2	2.030929454	0.647375738	1.330586115	0.486534265	0.626588417	0.942895711
C1orf112	374.9068233	0.12350927	0.253927453	0.486395893	0.626686502	0.942895711
ALG6	241.6444955	-0.093564581	0.192366251	-0.486387712	0.626692301	0.942895711
SYP	5.230832118	0.415849783	0.855114207	0.486309056	0.62674806	0.942899993
FASTKD5	268.1793542	-0.089121794	0.183350127	-0.486074351	0.626914452	0.942979933
SNORA50C	21.68877116	0.243954533	0.502231507	0.485741196	0.627150672	0.942979933
MCRIP1	708.2996969	-0.101766955	0.209528017	-0.485696169	0.6271826	0.942979933
PRRC2A	7599.966412	0.046498319	0.09575385	0.485602604	0.62724895	0.942979933
WDR11-AS1	3.702927426	0.546698279	1.126604315	0.485262014	0.627490497	0.942979933
PKP2	3.340486587	0.606627696	1.250134415	0.485249977	0.627499035	0.942979933
MYORG	263.4675895	0.081679782	0.168340282	0.485206399	0.627529943	0.942979933
CASP8	208.911981	-0.094422327	0.194615589	-0.4851735	0.627553278	0.942979933
PID1	60.78936386	-0.147876845	0.304805998	-0.485150706	0.627569446	0.942979933
H3-3A	15.5190539	0.341540604	0.704085321	0.485084114	0.62761668	0.942979933
TCTE1	12.74286698	-0.381325637	0.786246592	-0.484994963	0.627679919	0.942979933
DM1-AS	31.49679445	0.255999646	0.527933914	0.484908507	0.627741247	0.942979933
LOC100506023	36.08525074	0.190183899	0.392234994	0.48487234	0.627766904	0.942979933
SLC39A7	3467.356399	0.083357803	0.171939234	0.484809666	0.627811366	0.942979933
MIR3682	2.67099045	-0.744173785	1.53502525	-0.484795794	0.627821206	0.942979933
ATXN2-AS	9.592643807	0.349163895	0.720262555	0.484773077	0.627837322	0.942979933
RSRC1	602.4294315	-0.063293663	0.130613564	-0.484587215	0.627969185	0.942979933
WDR63	12.22532505	-0.288051032	0.594474299	-0.484547494	0.627997367	0.942979933
ALDOA	455.0079314	-0.064723806	0.133605812	-0.484438554	0.628074662	0.942979933
TMEM238	6.783446706	-0.349133003	0.720761745	-0.484394469	0.628105943	0.942979933
USP2	52.32109349	0.151598957	0.313050392	0.48426375	0.628198699	0.942979933
MIOS	659.1283703	-0.067925559	0.140273631	-0.484236123	0.628218303	0.942979933
PCDH10	38.09101422	0.187270549	0.386740222	0.484228271	0.628223875	0.942979933
IGF1	13.21336494	-0.349540553	0.721893202	-0.484199813	0.62824407	0.942979933
POLR3B	795.4897658	-0.13143913	0.271474447	-0.484167595	0.628266932	0.942979933
SEC22A	207.4706463	-0.077609139	0.160314097	-0.484106765	0.6283101	0.942979933
LINC02474	20.96283949	0.230796381	0.476776065	0.484077113	0.628331143	0.942979933
SDF2L1	203.4324287	0.117883834	0.243560878	0.484001515	0.628384794	0.942979933
NADK2	817.0499764	-0.071537978	0.147833115	-0.483910373	0.628449478	0.942979933
LETMD1	854.5921603	0.074438716	0.153927293	0.483596606	0.628672184	0.942979933
SAAL1	200.4732177	0.099867567	0.206530538	0.48354867	0.628706211	0.942979933
SF3B1	7662.656633	-0.048360946	0.100043066	-0.483401278	0.628810841	0.942979933
PAN3	591.101887	-0.080837878	0.16724814	-0.483340966	0.628853657	0.942979933

WNT8B	2.314878997	0.703723221	1.456003759	0.48332514	0.628864892	0.942979933
EZH2	334.9573399	-0.197450153	0.40854351	-0.483302629	0.628880874	0.942979933
SAT2	104.9952592	0.130816544	0.270688002	0.483274261	0.628901013	0.942979933
TRIQK	683.5324007	0.109003364	0.22556711	0.483241391	0.628924349	0.942979933
TTC39C-AS1	3.257753301	0.584400026	1.209470203	0.483186791	0.628963114	0.942979933
OSTN	4.674713977	-0.442395467	0.915586784	-0.483182451	0.628966194	0.942979933
POTEE	5.249255279	-0.604295406	1.250915504	-0.483082513	0.62903715	0.942979933
TNNT1	21.6800992	-0.261931601	0.542401381	-0.482911014	0.629158921	0.942979933
SNORD114-22	2.347312381	0.648098528	1.342094133	0.482900947	0.629166069	0.942979933
LINC01763	3.606162874	0.542066989	1.123174281	0.482620549	0.629365186	0.942979933
TBX1	11.50747571	-0.29435939	0.610030595	-0.482532176	0.629427947	0.942979933
MIR10226	5.554840894	0.453124886	0.939137065	0.482490685	0.629457414	0.942979933
G6PD	605.2875862	0.062145382	0.12882475	0.482402507	0.62952004	0.942979933
BEND3	342.9147093	0.110535958	0.229154475	0.482364386	0.629547116	0.942979933
GTF2H4	400.3115137	-0.072012212	0.149314104	-0.482286735	0.629602269	0.942979933
NDEL1	767.3126499	0.063244597	0.131141877	0.482260883	0.629620632	0.942979933
CHFR	712.6806502	0.078664863	0.163120921	0.482248766	0.629629238	0.942979933
ZKSCAN5	239.4123693	-0.091888277	0.190597409	-0.482106646	0.629730189	0.942979933
TOGARAM2	3.602548718	0.543070056	1.126651854	0.48202118	0.629790901	0.942979933
MEF2D	637.5683696	-0.086144596	0.178738741	-0.481958166	0.629835665	0.942979933
OR1F1	11.60910476	-0.272229558	0.564848392	-0.48195155	0.629840365	0.942979933
HES6	16.71752157	-0.261796863	0.543211132	-0.481943111	0.62984636	0.942979933
IMPG1	2.236707043	0.732291532	1.519562635	0.481909409	0.629870302	0.942979933
CENPV	509.8093572	0.121162953	0.251437405	0.481881179	0.629890357	0.942979933
DR1	1684.418326	0.053915872	0.111888445	0.481871669	0.629897113	0.942979933
DEPDC4	82.9856046	0.124921875	0.259309107	0.481748892	0.62998434	0.942979933
CTBP2	891.7510119	-0.063646655	0.132154311	-0.481608619	0.630084002	0.942979933
PCDHA3	2.116004261	0.788483145	1.637213243	0.481600762	0.630089585	0.942979933
PTPN18	214.5829654	0.110804191	0.230099986	0.481548012	0.630127065	0.942979933
SCG5	310.1426062	-0.13603512	0.282524489	-0.481498509	0.630162239	0.942979933
RNF32	19.02862785	0.222970501	0.463110909	0.481462424	0.63018788	0.942979933
SHPRH	670.4402845	0.103039138	0.214087894	0.481293621	0.63030783	0.943015522
DCAF12L2	81.84933413	-0.134816892	0.280133568	-0.481259325	0.630332202	0.943015522
GPR18	23.10455326	0.364916803	0.758518124	0.481091739	0.630451299	0.943015522
ECD	319.6017763	0.072723059	0.151198932	0.480976011	0.630533548	0.943015522
ZNF701	131.5368174	0.11005166	0.228823239	0.480946167	0.63055476	0.943015522
FBXW7	296.9603428	0.079647166	0.165610189	0.480931558	0.630565143	0.943015522
RELL2	47.99918443	0.227581561	0.473233343	0.480907705	0.630582096	0.943015522
GEMIN8	214.6252006	-0.110951825	0.230777144	-0.480774754	0.630676595	0.943077698
S100A5	8.122368437	-0.398421601	0.828920384	-0.480651229	0.6307644	0.943129854
LOC285074	248.2408413	0.06562491	0.136601826	0.4804102	0.630935743	0.943228587
SNORD72	7.522675886	0.335184485	0.697932999	0.480253099	0.631047434	0.943228587
LINC01534	12.86849031	0.255290314	0.531597301	0.480232525	0.631062062	0.943228587
RNASEH2C	393.2336187	-0.072315016	0.150600351	-0.480178274	0.631100634	0.943228587
ADCY5	2.459762121	0.783940244	1.632689477	0.480152689	0.631118825	0.943228587
GBP6	62.75376259	-0.118215159	0.246280171	-0.480002747	0.63122544	0.943228587
MIR10B	7.27222782	0.366840053	0.764402516	0.479904298	0.631295445	0.943228587
SERPINF1	45.60931454	-0.163946217	0.341658895	-0.479853502	0.631331566	0.943228587
MIR3153	24.07967414	-0.22640932	0.471843779	-0.479839577	0.631341468	0.943228587
NAV2	54.82780016	-0.182480766	0.380359188	-0.479759058	0.631398729	0.943228587
LEPROTL1	1129.109784	0.055766476	0.116252068	0.479703085	0.631438534	0.943228587
GUF1	792.0559411	0.076300672	0.159070744	0.479665023	0.631465603	0.943228587
SNORD98	5.42666529	0.54802849	1.142796672	0.479550303	0.631547192	0.943235594
EPHB4	1224.220962	0.053509361	0.111605233	0.479452078	0.631617053	0.943235594
CTNNA3	36.03525845	-0.260760374	0.544013815	-0.47932675	0.631706195	0.943235594
LHX6	147.9503908	0.11144202	0.232502599	0.479315157	0.631714441	0.943235594
KIAA0040	40.54190032	-0.204998773	0.427960001	-0.479013862	0.631928767	0.943235594
LRRC20	117.5687861	0.153549144	0.320554011	0.479011771	0.631930255	0.943235594
TNFRSF10C	19.19728203	-0.264072062	0.55136162	-0.47894531	0.631977536	0.943235594
LOC100287329	17.48867971	0.320168232	0.668776752	0.478737084	0.632125681	0.943235594
DUSP2	222.1966592	0.161794601	0.337981033	0.478709114	0.632145581	0.943235594
LINC00476	136.0393983	-0.10749994	0.224595644	-0.478637691	0.6321964	0.943235594
DEFB131B	3.541263523	0.486641321	1.01675097	0.478623906	0.632206208	0.943235594
DDX60	124.694347	-0.176735506	0.369296477	-0.478573495	0.632242078	0.943235594
BIK	4.023415725	0.534966522	1.117963542	0.478518755	0.632281029	0.943235594
ZNF767P	143.6528661	0.171406578	0.35855799	0.478044229	0.632618726	0.943235594
LINC00663	12.11670392	0.424718937	0.888568714	0.477980971	0.632663749	0.943235594
HERC2P11	4.729924032	0.472167177	0.988191798	0.477809245	0.632785981	0.943235594
PCDHB7	4.103567739	0.503173254	1.053092844	0.477805217	0.632788848	0.943235594
RCOR1	1407.604447	-0.059884758	0.125339269	-0.477781291	0.632805878	0.943235594
NDUFA1	133.5022799	0.114701682	0.24012178	0.477681291	0.632877063	0.943235594
SEC24B-AS1	9.039911793	0.318220828	0.666178904	0.477680734	0.632877459	0.943235594
MRPS31	182.9535148	0.083287074	0.174363881	0.477662424	0.632890493	0.943235594
SGCG	5.697997509	0.569973167	1.193524018	0.477554837	0.632967082	0.943235594
CMTM8	17.57638227	0.234553623	0.491183111	0.477527866	0.632986283	0.943235594
AK1	360.309796	0.127861273	0.267766518	0.47751031	0.632998781	0.943235594
INTS4P1	25.14742126	0.320516077	0.671285963	0.477465781	0.633030482	0.943235594
TMEM200C	1.821025246	0.929918005	1.947629099	0.477461548	0.633033496	0.943235594
PAK1	436.2080419	-0.069056959	0.144658494	-0.477379218	0.63309211	0.943235594
LIX1	2.52303624	0.614487953	1.287263249	0.477359975	0.63310581	0.943235594
TAFA3	2.664214219	0.62558347	1.310536309	0.477349208	0.633113476	0.943235594
CLU	70.87106147	0.210952255	0.44193041	0.477342699	0.63311811	0.943235594
ZNF30	95.60381905	0.119597855	0.250553869	0.4773339	0.633124375	0.943235594
SLC30A7	1215.005628	-0.04430959	0.09284747	-0.477229915	0.633198411	0.943235594
B4GALT2	1705.234951	0.06574537	0.13777211	0.477203771	0.633217026	0.943235594
KIAA2026	376.4812765	0.081513735	0.170969677	0.476773055	0.633523735	0.943296685
LINC01947	2.039868218	-0.590314637	1.238286355	-0.476719003	0.633562229	0.943296685
DPH1	458.2324771	-0.057071737	0.119726108	-0.476685808	0.63358587	0.943296685
TXNDC12	778.855688	-0.071111143	0.149195666	-0.476630084	0.633625557	0.943296685
MIR644A	4.049708478	0.465266691	0.97642284	0.476501237	0.633717326	0.943296685

LOC105371083	3.651907793	0.449879417	0.944171417	0.476480658	0.633731984	0.943296685
DPY19L2P2	564.5625437	0.131990927	0.277155639	0.476233958	0.633907709	0.943296685
SNN	252.6218988	0.101148904	0.212396962	0.476225758	0.633913551	0.943296685
SNORA79	2.695572576	-0.622448564	1.307118731	-0.476198948	0.633932649	0.943296685
SMYD4	623.2796656	0.058092145	0.122017393	0.476097248	0.634005097	0.943296685
SNORA23	9.463389013	-0.396501144	0.832867528	-0.476067478	0.634026305	0.943296685
RP1L1	6.770717834	0.384180566	0.807024079	0.476045977	0.634041623	0.943296685
CHMP6	383.9424051	0.079672474	0.167407226	0.475920164	0.634131256	0.943296685
TBC1D17	320.9617574	-0.119874091	0.251886065	-0.475906003	0.634141345	0.943296685
VWA1	45.26407956	-0.141359412	0.297123434	-0.47575989	0.634245448	0.943296685
LINC01449	5.029201594	0.408333526	0.858386725	0.47569879	0.634288983	0.943296685
RBX1	525.9490195	-0.066903855	0.140688714	-0.475545286	0.634398363	0.943296685
SREK1IP1	350.2023208	-0.088235579	0.18556124	-0.475506519	0.634425988	0.943296685
STK32A	5.345580801	0.39504672	0.830888515	0.475450934	0.634465598	0.943296685
EGFR	835.4209618	-0.099459467	0.209194674	-0.475439764	0.634473558	0.943296685
PARD3	697.4491504	-0.073317821	0.154259234	-0.475289676	0.634580517	0.943296685
NSMCE3	258.694973	-0.124546734	0.262093498	-0.475199634	0.634644688	0.943296685
RASSF5	33.53545768	0.160410188	0.337584879	0.475169944	0.634665848	0.943296685
LINC01431	2.458443612	-0.625075948	1.315535292	-0.475149509	0.634680412	0.943296685
CDK5RAP2	1138.518839	0.044235412	0.093117166	0.475051099	0.634750552	0.943296685
MCM3AP-AS1	244.7915962	0.16487345	0.347065674	0.475049715	0.634751538	0.943296685
IER3	346.3179888	0.322880642	0.679733794	0.47501043	0.634779539	0.943296685
DCLRE1B	330.2921479	-0.078881938	0.16608639	-0.474945227	0.634826014	0.943296685
SETD3	957.9635374	0.065589604	0.13810408	0.474928794	0.634837727	0.943296685
DIAPH2-AS1	3.235465607	0.640587538	1.348881692	0.474902686	0.634856337	0.943296685
FGF13	17.65750215	-0.324630681	0.683784393	-0.47475591	0.634960962	0.943296685
NOP2	788.4637667	0.077008942	0.162210395	0.474747267	0.634967123	0.943296685
ORC4	275.1775947	-0.078755602	0.165908501	-0.474692989	0.635005816	0.943296685
ABHD17C	209.8467018	0.077316279	0.162942583	0.474500141	0.635143298	0.943296685
ZSWIM2	5.957968344	0.395172333	0.83290915	0.474448303	0.635180255	0.943296685
SNX10	149.6222961	-0.103349699	0.218028253	-0.474019757	0.635485819	0.943296685
TIMM10	104.9636558	-0.12547646	0.264727635	-0.473983232	0.635511865	0.943296685
DCAF4L1	5.465122674	-0.391249763	0.8254568	-0.473979696	0.635514386	0.943296685
NPAP1	4.312146263	0.515648513	1.0879604	0.473958899	0.635529217	0.943296685
SNORA9B	6.06214296	0.423655895	0.894012076	0.473881625	0.635584323	0.943296685
C12orf49	1256.273357	0.063113514	0.133205032	0.473807281	0.635637342	0.943296685
EIF1AX-AS1	34.04676282	0.169442717	0.357671433	0.473738469	0.635686417	0.943296685
STN1	195.863165	-0.145419347	0.306975102	-0.473717074	0.635701676	0.943296685
LOC101929705	10.31873699	0.313865208	0.662659958	0.473644445	0.635753476	0.943296685
AKT1	2393.944808	-0.047218627	0.099703255	-0.473591631	0.635791144	0.943296685
RHBDD3	356.4756511	-0.113574977	0.23991045	-0.47340571	0.635923757	0.943296685
NUDT16L1	156.8668506	-0.07746209	0.163631799	-0.473392646	0.635933076	0.943296685
HELZ2	443.3800248	-0.109313419	0.23092719	-0.473367466	0.635951037	0.943296685
RNVU1-8	5.071201849	-0.533142846	1.126669031	-0.473202717	0.63606856	0.943296685
SLC7A11-AS1	15.24042445	-0.35223894	0.744470134	-0.473140457	0.636112975	0.943296685
THAP3	152.4568858	-0.092383773	0.195279523	-0.473084794	0.636152685	0.943296685
PRPF4B	1782.188719	0.116508074	0.246286303	0.473059492	0.636170736	0.943296685
LOC100130452	3.824371868	0.602676524	1.27437691	0.472918584	0.636271266	0.943296685
PHLDB3	84.29541944	0.125866012	0.266156806	0.472901722	0.636283296	0.943296685
VBP1	776.7901282	-0.067356359	0.142436156	-0.472888069	0.636293038	0.943296685
PAQR5	21.50123928	0.2129159	0.450415544	0.472709929	0.636420142	0.943296685
ATP2C2-AS1	5.160956029	0.44067059	0.932306688	0.472666984	0.636450785	0.943296685
CFAP20	203.9645712	-0.093196194	0.197189791	-0.472621801	0.636483026	0.943296685
ZNF555	57.63769534	0.158115554	0.33460292	0.472546845	0.636536513	0.943296685
NEMF	599.8353196	0.082469138	0.174528427	0.472525533	0.636551721	0.943296685
HECTD1	4748.190612	0.051015685	0.107966311	0.472514848	0.636559346	0.943296685
COA3	209.1861862	-0.100536783	0.212861598	-0.472310573	0.636705125	0.943296685
GAPVD1	2217.176983	-0.042702888	0.090413724	-0.472305376	0.636708834	0.943296685
ITPR1-DT	4.905635446	-0.472024132	0.999542638	-0.472240117	0.636755408	0.943296685
MIR378D2	2.104549688	0.815039495	1.7260304	0.4722046	0.636780757	0.943296685
PRSS16	78.81251176	0.161145882	0.341282079	0.472177979	0.636799756	0.943296685
CCN5	187.328558	0.310663382	0.657946622	0.472171103	0.636804664	0.943296685
TEK	5.175298862	-0.400745765	0.848897631	-0.472077846	0.636871225	0.943316867
TMEM45B	5.257905013	0.428399605	0.907712545	0.471955144	0.636958806	0.943368178
MSC	194.5200897	0.116151436	0.246243025	0.471694318	0.637144993	0.943535364
ETFB	452.4851518	-0.103876994	0.220242316	-0.471648664	0.637177585	0.943535364
ZBTB44	1269.635627	0.066384351	0.140835328	0.471361495	0.637382608	0.943540196
QTRT1	648.1223705	-0.09771621	0.20734465	-0.471274327	0.637444847	0.943540196
DKFZP434A062	5.905514056	-0.471033769	0.999587627	-0.471228091	0.637477861	0.943540196
LOC101927817	14.73225254	0.405154271	0.86002542	0.471095693	0.637572401	0.943540196
PHETA2	8.775318699	0.513584728	1.090251258	0.471070062	0.637590704	0.943540196
WNT7B	67.58967286	-0.225776346	0.479347518	-0.471007647	0.637635274	0.943540196
ZNF564	163.3769613	0.079943608	0.169838328	0.470704164	0.637852011	0.943540196
CCDC59	206.8528942	-0.083409122	0.177241203	-0.470596682	0.637928778	0.943540196
CCDC168	5.679024984	0.402736704	0.855818875	0.470586377	0.637936138	0.943540196
APCDD1L-DT	36.18348187	0.318424098	0.676824031	0.470468073	0.638020639	0.943540196
CCDC88A	2249.695426	0.045338761	0.096370033	0.470465344	0.638022588	0.943540196
UBAP1L	21.51803753	0.270985659	0.576049892	0.470420466	0.638054645	0.943540196
P3H1	2380.196136	-0.066274719	0.140885642	-0.470415002	0.638058548	0.943540196
LOC105371592	21.93743075	0.240555625	0.511637129	0.470168428	0.638234689	0.943540196
MRPL22	273.8365676	-0.075605878	0.160819792	-0.470127943	0.638263611	0.943540196
CDKN2B-AS1	121.4154199	0.142325961	0.302852979	0.46995067	0.638390262	0.943540196
LRRC17	18.15672002	-0.277156968	0.589942503	-0.469803356	0.638495517	0.943540196
ABCB9	198.4632949	-0.07975886	0.169777409	-0.469784885	0.638508714	0.943540196
EOLA1	182.4513333	0.094341812	0.200826075	0.46976874	0.63852025	0.943540196
ELOC	439.1849094	-0.05682994	0.120994016	-0.469692156	0.638574973	0.943540196
CCDC3	10.94692297	-0.287579493	0.612349254	-0.469633123	0.638617155	0.943540196
TMED10	6705.735769	-0.057595941	0.122663334	-0.469544882	0.638680212	0.943540196
MIR320A	4.87973343	-0.444794943	0.947394196	-0.469493	0.638717287	0.943540196
LOC389641	171.2478938	-0.093241089	0.198610602	-0.469466828	0.63873599	0.943540196

ERCC6L	234.239861	0.117229339	0.249720264	0.469442635	0.63875328	0.943540196
ABCB5	14.05777456	0.247721748	0.527787332	0.469359026	0.638813031	0.943540196
KDM8	61.92495722	0.156391385	0.333213635	0.469342695	0.638824702	0.943540196
REPS1	499.5062274	0.067129262	0.143033973	0.469323902	0.638838133	0.943540196
UTP20	2790.381503	0.106070912	0.226018138	0.469302656	0.638853317	0.943540196
LRRC59	4297.308155	0.055090234	0.1174091	0.469216049	0.638915215	0.943540196
USP40	433.71888	-0.068368606	0.145708351	-0.469215428	0.638915659	0.943540196
MOSPD1	189.7799872	0.168888574	0.360063638	0.469052012	0.639032459	0.943540196
MTDH	6482.843183	-0.058745672	0.125257693	-0.468998512	0.6390707	0.943540196
DIAPH2	982.0184139	0.058808572	0.125393201	0.468993303	0.639074423	0.943540196
MASP2	45.51910653	-0.159004215	0.339072308	-0.468938957	0.639113269	0.943540196
LOC554206	9.527034246	0.343123601	0.731776201	0.468891446	0.639147231	0.943540196
CHMP1A	688.5466497	-0.061253007	0.130634059	-0.46889002	0.63914825	0.943540196
CFLAR-AS1	7.005591875	0.478287225	1.020177245	0.468827576	0.639192887	0.943540196
ZNF233	10.68369228	0.37373031	0.797570593	0.468585869	0.63936568	0.943685193
SASH1	945.7996521	-0.077118303	0.164592073	-0.468542023	0.639397027	0.943685193
LAMA5	13.73893217	-0.258954369	0.553234802	-0.468073172	0.639732263	0.943838801
CHCHD10	165.753607	-0.099437281	0.212464184	-0.468019029	0.639770982	0.943838801
TNIK	785.7918419	-0.052962358	0.113190747	-0.4679036	0.639853529	0.943838801
LOC653712	11.0662345	-0.329070758	0.703377661	-0.46784363	0.639896417	0.943838801
PLXNB1	262.111352	0.090423497	0.193281724	0.467832628	0.639904285	0.943838801
TMEM269	5.433188377	0.418328837	0.894204363	0.467822407	0.639911595	0.943838801
CCDC124	249.126247	0.09386662	0.200648837	0.46781542	0.639916592	0.943838801
SLC12A1	4.095528654	-0.483104136	1.032971188	-0.467684037	0.640010558	0.943838801
TIA1	1665.904113	-0.06931742	0.148216464	-0.467676924	0.640015645	0.943838801
MBD1	913.056478	0.055460925	0.118656279	0.467408262	0.640207813	0.943838801
SLC35F5	1714.166407	0.040889156	0.08748834	0.467366916	0.640237388	0.943838801
FLJ37453	59.79154061	0.120972561	0.258862983	0.46732275	0.640268983	0.943838801
COL19A1	20.60770798	0.325665583	0.696893892	0.467310141	0.640278002	0.943838801
GTSE1-DT	8.413963372	0.423486987	0.906402067	0.467217588	0.640344211	0.943838801
VWA3B	2.682711085	0.589844118	1.26252407	0.467194355	0.640360832	0.943838801
CDK5RAP1	336.942336	0.08637196	0.184884754	0.467166484	0.640380771	0.943838801
C2	19.58788618	-0.237726574	0.508900652	-0.467137492	0.640401512	0.943838801
DRAXIN	6.953058724	-0.592850907	1.269514092	-0.46699041	0.640506739	0.943838833
SNORD63B	22.58311148	-0.2125201	0.455085493	-0.466989397	0.640507464	0.943838833
MIR1205	10.92406105	-0.387539788	0.830083279	-0.466868564	0.640593918	0.943888177
IL17REL	2.186464046	-0.705708125	1.511983863	-0.466743159	0.640683647	0.943942339
R3HCC1L	262.20392	-0.110863651	0.237687009	-0.466427051	0.640909852	0.944137236
PTMAP11	15.32837209	0.325340788	0.697653063	0.466336071	0.640974963	0.944137236
C17orf58	128.8502608	0.085615995	0.183625732	0.466252709	0.641034625	0.944137236
LYPLA1	1942.101207	-0.059571076	0.127767632	-0.466245438	0.641039829	0.944137236
GNA12	1690.132505	-0.04219722	0.090515431	-0.466188138	0.641080839	0.944137236
TBRG4	1087.430929	0.066192941	0.142022258	0.466074417	0.641162235	0.944165026
PDCD2	715.1808819	0.063343538	0.135926335	0.466013725	0.641205676	0.944165026
XKR6	6.349616461	-0.467229608	1.002998367	-0.46583287	0.641335135	0.944277626
ASCC3	2232.468589	0.065771867	0.141368581	0.465250952	0.641751755	0.944403576
FSIP2-AS2	3.414288144	-0.539711266	1.160136409	-0.465213626	0.641778481	0.944403576
SOX7	57.26216274	-0.120146668	0.258289962	-0.465161972	0.641815469	0.944403576
FKBP7	624.4017667	-0.105614001	0.227080752	-0.465094464	0.64186381	0.944403576
ADSS2	793.107086	-0.077778262	0.167260501	-0.465012731	0.64192234	0.944403576
EGFLAM	106.1327356	-0.138871342	0.298698969	-0.464920726	0.641988227	0.944403576
HOXA-AS2	16.38173794	0.225148154	0.484315923	0.464878694	0.642018329	0.944403576
LSP1	18.60576445	0.204230922	0.439704149	0.464473493	0.642308546	0.944403576
RAB11B-AS1	27.71878936	-0.17577848	0.378542101	-0.464356487	0.64239236	0.944403576
MEIS2	2532.509812	0.080324662	0.173053663	0.464160427	0.642532811	0.944403576
IDI1	712.0751548	0.110585455	0.238253465	0.464150459	0.642539952	0.944403576
BTG3-AS1	43.00168773	0.155808461	0.335728651	0.464090451	0.642582943	0.944403576
LOC100507291	12.78244363	0.370045849	0.79748313	0.464017149	0.642635459	0.944403576
AARSD1	6.023170832	0.359371081	0.774516365	0.463994174	0.64265192	0.944403576
FBXL20	822.678692	-0.139854477	0.301470115	-0.463908261	0.642713474	0.944403576
LINC00342	146.1180877	0.196714793	0.424051202	0.463893965	0.642723717	0.944403576
RARA-AS1	7.409769189	0.488052013	1.052084114	0.463890678	0.642726072	0.944403576
ETFA	1209.382451	-0.051554726	0.111145015	-0.463850997	0.642754504	0.944403576
SCD5	283.7836125	-0.121665518	0.262349012	-0.463754435	0.642823692	0.944403576
LOC102724874	30.39655213	-0.243813264	0.525771699	-0.463724588	0.642845079	0.944403576
MPV17	382.8678147	0.100116103	0.215917427	0.463677733	0.642878653	0.944403576
GDE1	858.5927478	-0.066270299	0.142942818	-0.463614055	0.642924282	0.944403576
GPR179	7.676044178	-0.324969922	0.701000553	-0.463580123	0.642948598	0.944403576
GJC2	10.57456453	-0.320958756	0.692413343	-0.463536353	0.642979963	0.944403576
VPS18	618.6073131	-0.087524153	0.188822002	-0.463527302	0.64298645	0.944403576
BCAN	5.513808686	0.36085014	0.778599098	0.463460774	0.643034126	0.944403576
CLEC4A	6.934070601	0.338182091	0.729761797	0.463414353	0.643067392	0.944403576
LINC01297-DUXAP10-NBEAP6	46.72472418	0.21462986	0.463250488	0.463312756	0.643140204	0.944403576
ASTN2-AS1	7.04914068	0.568931727	1.228239917	0.463208954	0.643214599	0.944403576
SLC27A3	110.1419341	0.099132787	0.21401906	0.46319607	0.643223833	0.944403576
LINC00327	7.194104774	0.376803888	0.813603562	0.463129594	0.643271478	0.944403576
PRIM2	1029.613339	-0.062081661	0.134060068	-0.463088389	0.643301012	0.944403576
GPR62	2.756463167	0.568142926	1.227006995	0.46303153	0.643341767	0.944403576
VSIG10L2	1.964082377	-0.716851178	1.548180127	-0.463028278	0.643344098	0.944403576
LOC112268124	88.10842619	0.139110768	0.300465489	0.462984178	0.643375708	0.944403576
USP27X	43.94376134	-0.146825939	0.317170254	-0.462924681	0.643418356	0.944403576
GLT8D1	940.2193368	-0.072896403	0.157471675	-0.462917552	0.643423466	0.944403576
DNAAF4	7.013339296	-0.355164578	0.767256392	-0.462902078	0.643434558	0.944403576
ACTG1	29023.50098	0.062042702	0.134078795	0.462733138	0.643555662	0.944460843
SSBP1	257.9969936	-0.118045975	0.255199277	-0.46256391	0.643676982	0.944460843
ZNF696	315.6808025	0.096577929	0.208793874	0.462551543	0.643685849	0.944460843
SNX21	228.9945029	-0.081661224	0.176592272	-0.462428069	0.643774374	0.944460843
TRIP13	790.4040497	0.074895438	0.161972996	0.462394594	0.643798375	0.944460843
PCDHGA10	98.81302396	0.13937859	0.301465782	0.462336353	0.643840134	0.944460843
B4GALT1	2236.553924	0.091461623	0.197850515	0.462276396	0.643883125	0.944460843

ATP8B3	64.52780219	0.195512799	0.422953291	0.462256243	0.643897575	0.944460843
MUSK	7.381573919	0.406577202	0.880005865	0.462016469	0.644069511	0.944635282
TRIM54	3.2115932	0.524262804	1.135456669	0.46171978	0.644282285	0.944678085
COX15	795.9945621	-0.067229694	0.14561371	-0.461698933	0.644297236	0.944678085
SAFB2	402.7274254	0.108894992	0.235860092	0.461693161	0.644301376	0.944678085
ZNF260	456.7901571	-0.065767676	0.142483131	-0.461582195	0.644380965	0.944678085
PDZD7	19.03960527	0.237538811	0.514639087	0.461563874	0.644394106	0.944678085
MRPL23	334.4464994	-0.075825057	0.164298636	-0.461507526	0.644434523	0.944678085
HEY1	70.27888073	0.128851266	0.279246147	0.461425402	0.64449343	0.944678085
FERMT2	1590.591379	-0.068969263	0.149483279	-0.461384469	0.644522792	0.944678085
LOC101928737	1.930582538	0.891229609	1.932371388	0.461210311	0.644647725	0.94478349
SLC12A9	331.8245025	0.073086254	0.158516966	0.461062658	0.644753651	0.944839323
PCDHB12	105.1877349	0.099581294	0.216014722	0.460993091	0.644803562	0.944839323
ATP5PO	733.6076239	-0.077951646	0.169127503	-0.460904612	0.644867043	0.944839323
CKAP5	3899.90742	0.070398265	0.152753595	0.460861596	0.644897906	0.944839323
ARL13B	228.2571449	0.080674595	0.175121774	0.460677124	0.64503027	0.944872961
MICU2	511.1785399	-0.057929918	0.125769169	-0.460605074	0.64508197	0.944872961
ANKRD13B	406.7941808	-0.080835777	0.175509881	-0.460576788	0.645102268	0.944872961
EPN2	448.3465406	-0.068528618	0.148802509	-0.460534021	0.645132958	0.944872961
PMFBP1	23.3641	0.228702131	0.496731918	0.4604136	0.645219375	0.94488603
LOC100131496	9.414541011	0.341658595	0.742132993	0.460373811	0.645247929	0.94488603
LOC105379331	6.346079649	0.392311215	0.852502677	0.460187663	0.645381525	0.945004008
XRRA1	120.8177499	0.113453955	0.246610082	0.460054001	0.64547746	0.945066826
TCF7L1	339.2384097	0.103449115	0.22494966	0.459876733	0.645604701	0.945113131
TAPBPL	60.23446514	0.137272633	0.298518446	0.459846402	0.645626474	0.945113131
ZNF517	96.590219	-0.122355531	0.266116405	-0.459781995	0.645672708	0.945113131
STT3A-AS1	5.768535834	0.402540096	0.875734307	0.459660074	0.645760232	0.945113131
GMPR	36.81915702	-0.21887664	0.476465384	-0.459375745	0.645964363	0.945113131
GSTM2	12.13606003	0.274027504	0.5965287	0.459370194	0.645968349	0.945113131
FOXA1	11.79114529	0.281946871	0.613769156	0.459369567	0.645968799	0.945113131
LRBA	1350.847028	-0.051588346	0.11230539	-0.459357704	0.645977316	0.945113131
CCNG1	1900.176606	0.094155264	0.205016105	0.459257891	0.646048983	0.945113131
LTN1	1230.780149	0.046559644	0.101389181	0.459217081	0.646078286	0.945113131
DYNC1H1	11514.90008	-0.04752857	0.103508555	-0.459175279	0.646108301	0.945113131
KBTBD3	35.30168303	-0.151185691	0.329340862	-0.459055369	0.646194405	0.945113131
ENTPD6	1147.485426	-0.080322543	0.174975746	-0.459049579	0.646198563	0.945113131
TMEM68	735.883276	0.072349622	0.157695134	0.458794261	0.646381917	0.945171381
TTC3P1	9.33846648	-0.303913681	0.662500697	-0.458737148	0.646422934	0.945171381
LCA5L	10.26734522	-0.317426909	0.69203449	-0.458686544	0.646459279	0.945171381
SNORA19	6.819219153	0.349594552	0.7621829	0.458675408	0.646467277	0.945171381
DKFZP586I1420	23.28851178	0.186123233	0.405828933	0.458624849	0.646503589	0.945171381
RAB34	1437.808402	-0.061551926	0.134232123	-0.458548406	0.646558494	0.945174107
PAM16	28.43814592	-0.212340743	0.46357028	-0.458055126	0.646912837	0.945351098
NCK1	325.1035859	-0.064263018	0.140297201	-0.458049181	0.646917108	0.945351098
ITGB1BP1	1065.397171	-0.043638305	0.095273434	-0.458032245	0.646929275	0.945351098
MECOM	829.9536433	0.051284089	0.111978611	0.457981114	0.64696601	0.945351098
RO60	3494.846194	-0.041951632	0.091622241	-0.457876078	0.647041475	0.945351098
UNC5B	1087.113843	-0.164532138	0.359397948	-0.457799325	0.647096621	0.945351098
C5orf17	112.4778606	0.135063627	0.295123713	0.45765088	0.647203283	0.945351098
SLC2A10	734.5187934	0.148039009	0.323491389	0.4576289	0.647219077	0.945351098
MYHAS	4.50204827	0.419103977	0.915851563	0.457611248	0.647231761	0.945351098
ANKRD1	7.14889069	-0.424986855	0.928760386	-0.457585037	0.647250596	0.945351098
SCAPER	286.3185554	0.086334846	0.188682162	0.457567612	0.647263117	0.945351098
ZFYVE1	300.5849164	-0.074370911	0.162571845	-0.457464889	0.647336934	0.945381426
NTS	11.98288696	0.28161321	0.615735933	0.457360363	0.647412049	0.945413645
LINC02657	2.278867231	-0.668073222	1.461482043	-0.457120377	0.647584525	0.945588023
LOC100132813	34.1637234	0.374190486	0.818870484	0.456959303	0.647700297	0.945616457
SIN3B	701.7133813	0.056340452	0.123297934	0.456945627	0.647710128	0.945616457
PSMA3	974.4501222	0.08847369	0.19365767	0.456856113	0.647774471	0.945632921
WDR92	280.1480216	-0.067088605	0.146961682	-0.456504063	0.64802755	0.945827537
LINC02101	3.291966025	-0.540812178	1.1848611	-0.456435086	0.64807714	0.945827537
VSTM5	2.631134636	-0.586766456	1.286016727	-0.456266582	0.648198292	0.945827537
TCF3	1688.995542	-0.046424377	0.101759835	-0.456215138	0.64823528	0.945827537
GALK2	606.6235525	0.080764334	0.177035055	0.456205321	0.64824234	0.945827537
ARMC3	30.4361366	-0.184271546	0.403974808	-0.456146132	0.648284898	0.945827537
LINC02328	29.49748463	0.203306658	0.445813664	0.456035053	0.648364772	0.945827537
ANGPT1	170.0982834	-0.136243482	0.298771186	-0.456012791	0.64838078	0.945827537
IKZF3	4.45454942	-0.421016731	0.923311544	-0.45598556	0.648400362	0.945827537
P3H2	197.9513328	0.097540092	0.213982586	0.455831912	0.648510854	0.945827537
ZNF546	73.11634728	0.12746023	0.27974869	0.455624047	0.648660348	0.945827537
PRKD1	577.7513823	-0.053295474	0.116985352	-0.455573904	0.648696412	0.945827537
TUG1	2732.166773	-0.061214633	0.134423781	-0.455385442	0.648831967	0.945827537
SLC25A28	144.6376732	0.088767274	0.194955444	0.455320825	0.648878446	0.945827537
GTF2I	1999.128548	-0.047175824	0.103627558	-0.455243996	0.648933712	0.945827537
NOP14-AS1	77.07183437	0.157579116	0.346171403	0.455205469	0.648961426	0.945827537
ZMYND19	491.9433003	0.068379221	0.150217996	0.45519993	0.648965411	0.945827537
LYG2	6.364518954	0.475381975	1.044350005	0.455194113	0.648969595	0.945827537
MYH16	3.212170561	-0.514705221	1.130740518	-0.455193046	0.648970363	0.945827537
IQSEC3	159.2376888	-0.178855523	0.392925991	-0.455188831	0.648973395	0.945827537
NBN	1954.299953	-0.058114976	0.127694103	-0.455110885	0.649029468	0.945827537
C14orf93	213.6153318	0.101897682	0.223927851	0.455046934	0.649075474	0.945827537
ZNF783	272.6556293	0.11312023	0.248707999	0.45483149	0.649230473	0.945905008
ZNF571-AS1	29.06412608	-0.181722909	0.399544263	-0.454825475	0.649234801	0.945905008
MRPL46	118.0511182	0.093365262	0.205334566	0.454698219	0.649326362	0.945961068
HOXB6	126.6203476	0.092676318	0.203856392	0.454615708	0.649385732	0.945964034
SLC39A9	2686.66743	0.078446313	0.17261236	0.454465211	0.649494026	0.945964034
SEPTIN7P9	2.524598938	0.595498428	1.310554501	0.454386618	0.649550582	0.945964034
PRMT9	183.9962873	0.0929148	0.204493273	0.454366046	0.649565386	0.945964034
STK11IP	413.1674823	0.080659232	0.177598542	0.454166068	0.649709303	0.945964034
LTC4S	6.999096591	0.353251721	0.778049954	0.454021903	0.649813062	0.945964034

FAM27C	2.973417486	0.580160064	1.278007912	0.453956551	0.6498601	0.945964034
ELP5	484.7930636	-0.070289099	0.154847247	-0.453925402	0.64988252	0.945964034
RAD51AP2	10.21345378	-0.341009292	0.751332779	-0.453872506	0.649920593	0.945964034
CLSTN2	2.769406094	-0.600003998	1.322576537	-0.453662969	0.650071424	0.945964034
SMIM21	3.562795745	0.496069118	1.093836298	0.453513125	0.650179294	0.945964034
CACTIN-AS1	14.4353577	0.31993369	0.705731213	0.453336461	0.650306482	0.945964034
ANGPTL8	5.335618424	0.488517203	1.077655026	0.453315013	0.650321924	0.945964034
RHOB	1138.605131	-0.111698646	0.246408925	-0.45330601	0.650328406	0.945964034
INTS13	513.8248719	0.061189523	0.134989267	0.45329176	0.650338665	0.945964034
LY6G5C	5.594488768	0.488794879	1.078463106	0.453232824	0.650381099	0.945964034
SPATA24	21.33922284	-0.224135143	0.49454489	-0.453214961	0.650393961	0.945964034
MYH15	21.98094043	0.21148339	0.466653905	0.453191086	0.65041115	0.945964034
U2AF1L4	75.23896342	0.141741599	0.312776901	0.45317157	0.650425203	0.945964034
PRICKLE4	53.47539809	-0.145264615	0.320559349	-0.453159814	0.650433667	0.945964034
SLC22A13	5.158223392	-0.445892097	0.983990795	-0.453146614	0.650443171	0.945964034
ADAP2	2.565460595	-0.695409142	1.534875702	-0.45307196	0.650496926	0.945965009
EIF2B2	408.8322512	0.076935772	0.169902683	0.452822582	0.650676501	0.946073169
DTNB	182.2392942	0.084754439	0.187169761	0.452821218	0.650677484	0.946073169
SMARCA5-AS1	35.41436893	0.143860751	0.317792945	0.452687049	0.650774106	0.946111109
PARL	904.2323497	-0.083317339	0.184075674	-0.452625475	0.650818452	0.946111109
APOM	22.40801716	0.244004589	0.539197956	0.452532481	0.650885427	0.946111109
FNDC3B	7135.375091	-0.051830118	0.114544201	-0.452490105	0.650915948	0.946111109
TM6SF1	7.228762532	-0.354274945	0.783106891	-0.452396664	0.65098325	0.946131761
ITGA9	116.4612765	-0.203063966	0.448999218	-0.452259063	0.651082364	0.946178646
ULK4	131.6103916	0.130526054	0.288667592	0.452167329	0.651148442	0.946178646
SERPINB1	3.425949423	-0.57437259	1.270443796	-0.452103896	0.651194137	0.946178646
ALDH1L1	45.44203084	0.190362804	0.421103509	0.452057035	0.651227895	0.946178646
PPP1R9A	92.85542855	0.138114071	0.305802118	0.451645241	0.651524572	0.946246773
USP20	567.4998381	0.092865171	0.2056177	0.451639964	0.651528375	0.946246773
GTF2H2	72.85420122	0.10868038	0.240672045	0.451570436	0.651578472	0.946246773
LOC100507564	5.327922717	0.363626633	0.805424903	0.451471802	0.651649543	0.946246773
CCDC180	4.604809599	-0.452129832	1.001515985	-0.451445448	0.651668534	0.946246773
PSMD12	1237.34565	0.055826481	0.123671087	0.451410939	0.651693401	0.946246773
MTERF3	480.4265582	0.064055864	0.141921313	0.451347742	0.651738941	0.946246773
SGSM2	1143.81792	0.07861998	0.174191329	0.451342674	0.651742593	0.946246773
CPEB1-AS1	3.882403039	0.420105354	0.930819124	0.451328666	0.651752687	0.946246773
EIF1B-AS1	3.589970915	0.481341889	1.066891006	0.451163133	0.651871978	0.946247789
KCNAB3	57.69874713	0.178853892	0.396457303	0.451130275	0.651895659	0.946247789
FCGBP	18.19767152	0.228547763	0.506644114	0.451101191	0.65191662	0.946247789
CWC27	159.3044465	0.077443257	0.171721981	0.450980454	0.652003636	0.946247789
SRPRB	1374.154884	-0.06299994	0.139702055	-0.450959292	0.652018889	0.946247789
SNORD93	5.596030612	0.517084268	1.146846895	0.450874716	0.652079847	0.946259192
C1orf229	14.25055357	0.267198546	0.593082806	0.450524857	0.652332035	0.946389103
SNORD1B	6.839478708	0.399791197	0.887765592	0.450334188	0.652469491	0.946389103
UNC50	674.7184714	0.069997691	0.15551878	0.450091565	0.652644419	0.946389103
KIF14	1077.718442	0.061194615	0.136030838	0.449858399	0.652812546	0.946389103
HNRNPH1	6175.0606	-0.103081815	0.229155069	-0.449834322	0.652829908	0.946389103
PFKP	1717.445813	0.054489163	0.121136642	0.449815702	0.652843335	0.946389103
EIF1AD	340.4004728	0.067386496	0.149817592	0.449790276	0.65286167	0.946389103
NTNG1	216.4909495	0.092827647	0.206395112	0.449757003	0.652885664	0.946389103
TRIM46	88.33978118	0.137052341	0.304760538	0.44970501	0.652923159	0.946389103
FAM120AOS	570.3804269	-0.047990832	0.106737214	-0.449616683	0.652986856	0.946389103
ORAI2	754.8255295	-0.071361437	0.158743184	-0.449540166	0.65304204	0.946389103
IFT22	135.590194	0.093823586	0.208736405	0.44948358	0.653082851	0.946389103
MED23	1129.114196	0.05089978	0.11325098	0.449442295	0.653112626	0.946389103
UQCC3	123.2637636	0.090593245	0.201605533	0.449358925	0.653172757	0.946389103
LYRM4	397.082486	0.066551088	0.148105152	0.449350257	0.653179009	0.946389103
PCDHA1	5.628163741	-0.430532438	0.958242209	-0.449293961	0.653219614	0.946389103
SEPTIN9	2793.7032	-0.059808787	0.133160967	-0.449146535	0.653325953	0.946389103
ZNF20	3.242646518	0.578388182	1.287849698	0.449111556	0.653351185	0.946389103
GPCPD1	300.7847234	0.148100651	0.329765548	0.449108925	0.653353082	0.946389103
FAM104A	426.1046396	-0.053717629	0.119618198	-0.449075728	0.653377029	0.946389103
MIEF1	1315.810218	0.048611295	0.108255952	0.449040392	0.653402519	0.946389103
NPIPB2	3.001859746	-0.539979485	1.202851658	-0.448916108	0.653492176	0.946389103
MIR196A1	3.826461313	-0.572244173	1.274984541	-0.448824401	0.653558335	0.946389103
CHAC2	72.42103768	-0.18147382	0.404348549	-0.44880542	0.653572029	0.946389103
ATP8B2	3545.825707	0.043316428	0.096521828	0.448773392	0.653595135	0.946389103
ACTG2	147.4188561	0.31048942	0.691899488	0.448749314	0.653612507	0.946389103
BLVRA	200.9031567	-0.092862353	0.206961668	-0.448693487	0.653652783	0.946389103
AEBP2	609.2892099	-0.062145625	0.138505063	-0.448688472	0.653656402	0.946389103
ARAF	573.5028203	0.053795979	0.119925568	0.44857806	0.653736064	0.946427546
DHX37	1354.017879	0.064900582	0.144788719	0.448243362	0.653977572	0.946621223
LOC100128398	10.13535724	0.346191972	0.772356041	0.448228477	0.653988313	0.946621223
MAP3K21	174.6886259	0.089902399	0.200625786	0.448109892	0.654073889	0.946621223
TAS1R3	96.82575659	0.158175963	0.352993973	0.448098196	0.65408233	0.946621223
C1orf116	1.892007489	0.661589314	1.477153735	0.44788115	0.654238973	0.946709478
AHCYL1	3566.236234	-0.081678612	0.182476476	-0.447611737	0.654433431	0.946709478
ME2	731.3876132	0.062805463	0.140364185	0.447446497	0.65455271	0.946709478
G6PC3	705.142509	0.080670097	0.180294261	0.447435744	0.654560472	0.946709478
NTF4	3.033668347	0.647193643	1.446870871	0.447305738	0.654654324	0.946709478
PIGQ	590.8542467	0.061508829	0.137511677	0.447298955	0.654659222	0.946709478
SLC6A7	2.440843904	0.532105706	1.189609653	0.447294375	0.654662528	0.946709478
HS2ST1	1423.180908	0.059641013	0.133341019	0.447281813	0.654671597	0.946709478
TIMM22	487.2205986	0.063485514	0.141945758	0.44725193	0.65469317	0.946709478
MMP25-AS1	33.9926673	0.178002117	0.398095043	0.447134724	0.654777789	0.946709478
ACP4	50.86753198	0.169810797	0.379870863	0.447022432	0.654858864	0.946709478
NDUFS8	282.19492	-0.097684403	0.218525878	-0.447015262	0.654864041	0.946709478
PITPNM2-AS1	13.73149016	0.373654357	0.835924924	0.446995114	0.654878587	0.946709478
MIR215	4.765977903	0.481540545	1.07731203	0.446983355	0.654887078	0.946709478
ARFGEF2	1496.803585	-0.048802337	0.10921079	-0.446863696	0.654973477	0.946757574

SMIM4	62.45505982	0.218476535	0.489085179	0.446704468	0.655088455	0.946846968
OCIAD1-AS1	19.46602667	0.214571481	0.480488151	0.446569765	0.65518573	0.946883796
IFI27	7.43699842	0.344064572	0.770733537	0.446411835	0.655299786	0.946883796
PEX5	672.4514103	-0.070299009	0.157483085	-0.446390858	0.655314936	0.946883796
FAM27E5	3.811883937	-0.46258811	1.036322017	-0.446374875	0.655326479	0.946883796
GPC6-AS1	2.382488749	-0.595003901	1.333249701	-0.446280918	0.655394338	0.946905068
ARHGEF3	173.1409484	-0.095228756	0.213690044	-0.44563965	0.655857565	0.947497511
LOC105372383	3.510252835	0.443206458	0.99481953	0.445514432	0.655948032	0.94751089
MIR323B	5.455799847	-0.483479043	1.085299968	-0.445479643	0.655973168	0.94751089
SNORD16	4.9818493	0.410789911	0.922316267	0.445389425	0.656038353	0.947528242
MRE11	668.2039611	-0.046403531	0.104210159	-0.445287973	0.656111659	0.947557319
RCE1	152.2428818	0.075257342	0.169110138	0.445019695	0.656305521	0.947683966
RPL35A	1719.947242	-0.080974544	0.182006638	-0.444898847	0.656392856	0.947683966
MTERF4	263.1390856	0.10163817	0.228476961	0.444850849	0.656427544	0.947683966
MCU	497.9166774	-0.073199946	0.164552911	-0.444841393	0.656434379	0.947683966
SLC5A2	15.23579581	0.222190961	0.499690424	0.444657233	0.65656748	0.947683966
IL1A	5.199562779	0.634615606	1.427520285	0.44455803	0.656639184	0.947683966
DDB2	211.6101868	0.089729097	0.201839287	0.444557142	0.656639825	0.947683966
IST1	1422.600112	-0.043180316	0.097138563	-0.4445229	0.656664576	0.947683966
FGD3	2.111024448	-0.685840765	1.543106022	-0.444454727	0.656713854	0.947683966
CELF1	2961.676005	-0.05405906	0.121642266	-0.44441017	0.656746062	0.947683966
ZNF133	148.1516002	-0.094950802	0.213705192	-0.444307416	0.65682034	0.947683966
PYCR2	836.8516254	-0.057696634	0.129874276	-0.444249902	0.656861917	0.947683966
RTN4RL2	16.02436417	-0.276458897	0.622360698	-0.444210082	0.656890704	0.947683966
MEG8	1883.96364	-0.117201235	0.263909492	-0.444096321	0.656972946	0.947725039
XAB2	1059.632148	-0.058767313	0.132351787	-0.444023574	0.65702554	0.947725039
ODF3L1	4.035447495	0.430670721	0.970599748	0.443716086	0.657247864	0.947899638
BCAR3	607.0273568	0.075597074	0.17039762	0.443650998	0.657294929	0.947899638
FRAT2	171.1164273	-0.102784288	0.231724146	-0.443563131	0.657358467	0.947899638
ACVR2B-AS1	10.95025227	-0.302129303	0.681143493	-0.443561901	0.657359356	0.947899638
IFITM1	12.69240232	0.371061392	0.837040696	0.443301496	0.657547674	0.948020058
ZNF507	1219.283471	0.048473998	0.109366824	0.443223968	0.657603744	0.948020058
TRAPPC5	214.3134889	-0.075633116	0.170669508	-0.443155412	0.657653327	0.948020058
EIF5B	1759.661605	-0.059074189	0.133319624	-0.443101982	0.657691972	0.948020058
KLHL2	172.2687581	0.096554043	0.217916273	0.443078626	0.657708865	0.948020058
GXYLT1	747.6489204	0.09274809	0.20936707	0.442992731	0.657770992	0.948032925
MYO15A	10.25050522	0.287444407	0.64922012	0.442753387	0.657944122	0.948055566
SRD5A3	185.9313388	0.077497963	0.175072807	0.442661339	0.65801071	0.948055566
SNHG30	84.17213019	0.164315045	0.371350904	0.442479184	0.65814249	0.948055566
SRRM3	7.221641299	-0.35807359	0.809278682	-0.442460178	0.65815624	0.948055566
UBXN2A	575.913553	-0.053013135	0.119822713	-0.442429764	0.658178245	0.948055566
ZC3H7A	964.4973843	-0.055866762	0.126302025	-0.44232673	0.658252791	0.948055566
LOC101927752	168.5511841	-0.094853349	0.214450882	-0.442308037	0.658266316	0.948055566
TIMELESS	1348.083537	-0.038858297	0.087868827	-0.442230753	0.658322234	0.948055566
ZNF430	143.031028	0.078219487	0.176892545	0.442186453	0.658354288	0.948055566
TAMM41	123.198055	0.118216651	0.267346747	0.442184735	0.658355531	0.948055566
RNF169	683.6558615	0.069965614	0.158235219	0.442162085	0.658371921	0.948055566
SNORC	18.23442802	0.258048301	0.583709042	0.442083782	0.65842858	0.948060544
SNORA54	7.994000881	-0.388129023	0.878330229	-0.441894188	0.658565777	0.948149009
CCDC154	3.504577339	-0.41690091	0.943531033	-0.441851826	0.658596433	0.948149009
PEX5L	48.75356778	0.17438234	0.394895171	0.441591472	0.658784856	0.948343658
SLFNL1	34.65092726	0.276117343	0.625713776	0.441283785	0.659007563	0.948359764
PPARG	317.622039	0.065615265	0.148723098	0.44119082	0.659074858	0.948359764
IRX3	1096.721373	-0.09540103	0.216235643	-0.441190126	0.659075361	0.948359764
AGTRAP	276.0567083	-0.073656578	0.166956329	-0.44117272	0.659087961	0.948359764
IQCH	68.7774684	-0.17320643	0.392629983	-0.441144176	0.659108624	0.948359764
CATSPER1	3.165772572	-0.463249809	1.050336636	-0.441048891	0.659177603	0.948359764
MSRB2	124.4041943	-0.117459457	0.266340986	-0.441011572	0.659204619	0.948359764
C19orf73	3.951273618	0.439977474	0.997824876	0.440936565	0.659258921	0.948359764
RPL9	1487.279395	-0.089060413	0.202099408	-0.440676268	0.65944738	0.948359764
PMS2P4	21.97276475	-0.251311292	0.570363353	-0.440616128	0.659490925	0.948359764
BTBD2	1910.195449	-0.080515065	0.182759562	-0.440551859	0.659537461	0.948359764
NEIL3	156.9363017	0.102062755	0.231689286	0.440515641	0.659563686	0.948359764
SETD5	3167.206846	0.049064656	0.111389668	0.440477622	0.659591216	0.948359764
MRPL53	199.835186	0.085994466	0.195240782	0.440453401	0.659608755	0.948359764
WDR60	136.7968127	0.122326822	0.277800257	0.440340925	0.659690204	0.948359764
MX1	15.74606219	-0.245544347	0.557711481	-0.440271278	0.65974064	0.948359764
REXO5	125.5293598	-0.111024312	0.252208884	-0.440207776	0.659786628	0.948359764
SNORD116-23	1.993014539	-0.606689821	1.379024026	-0.439941444	0.659979518	0.948359764
PCCB	1082.077549	0.081665019	0.185752463	0.439644343	0.660194719	0.948359764
BRWD1-AS1	3.159422214	0.525816306	1.196126526	0.439599235	0.660227395	0.948359764
NDUFAF1	283.4056627	0.080893911	0.184058136	0.439501958	0.660297863	0.948359764
ARL9	6.488693826	0.361584116	0.822748509	0.439483161	0.660311481	0.948359764
AKR1C3	157.1171572	0.145326145	0.330718854	0.439425039	0.660353587	0.948359764
AFF1-AS1	14.46255359	-0.207685065	0.472644222	-0.439410989	0.660363765	0.948359764
LMX1B	3.10219333	-0.567164904	1.290930956	-0.439345653	0.660411099	0.948359764
NSUN7	8.054651881	0.38897846	0.885370037	0.439339986	0.660415205	0.948359764
LNP1	97.26131426	-0.109087243	0.2483975	-0.439164012	0.6605427	0.948359764
ZNF443	53.70110692	0.11256037	0.256319394	0.439141059	0.66055933	0.948359764
TMEM251	120.9330738	0.083564454	0.190292253	0.439137449	0.660561946	0.948359764
RNH1	1609.654221	-0.066045796	0.150399009	-0.439137173	0.660562146	0.948359764
CDKN2AIP	181.4677587	0.06826243	0.155468903	0.439074492	0.660607561	0.948359764
LOC105369747	2.157714541	-0.643809522	1.466542908	-0.438998081	0.660662927	0.948359764
C3orf49	11.97760074	0.321224575	0.731763474	0.438973229	0.660680934	0.948359764
NMT2	236.1733982	-0.070793759	0.16127864	-0.438953099	0.66069552	0.948359764
CCDC88B	15.83124152	-0.221012609	0.503571387	-0.438890323	0.660741009	0.948359764
SPAG7	210.6031527	-0.081213988	0.185088021	-0.43878576	0.66081678	0.948359764
MAPK8IP1	66.86284411	-0.111511379	0.254142848	-0.438774415	0.660825	0.948359764
JADE1	454.5108134	0.076124545	0.173495919	0.438768504	0.660829284	0.948359764
MYOM1	10.89675007	-0.356971405	0.813709374	-0.438696439	0.660881508	0.948359764

CLDND2	6.184170995	0.408846205	0.932083893	0.438636702	0.660924799	0.948359764
PKN2-AS1	2.337194349	0.725385613	1.654146828	0.438525529	0.661005368	0.948399006
INTS1	3799.644375	0.044919438	0.10246224	0.438399924	0.661096402	0.948453255
HYAL1	19.57285921	0.236004768	0.538563833	0.43821132	0.661233104	0.94846917
ZNF223	69.78130736	-0.115675011	0.263978865	-0.438198003	0.661242756	0.94846917
ATP11B	2915.583214	-0.046522496	0.106205368	-0.438042799	0.661355259	0.94846917
CFAP58	4.249514072	0.410819182	0.937894639	0.438022742	0.661369798	0.94846917
KCTD5	520.9625777	0.066546013	0.151931982	0.437998717	0.661387214	0.94846917
SIL1	520.1895636	-0.082172714	0.187632905	-0.437944046	0.661426845	0.94846917
ACBD6	325.471597	-0.067184911	0.153475599	-0.437756305	0.661562949	0.948537436
MKRN2	382.7352982	-0.071241191	0.162765938	-0.437691032	0.661610271	0.948537436
RANBP1	908.3712451	-0.067063015	0.153231512	-0.437658113	0.661634138	0.948537436
SNORA80C	4.375572687	-0.467595731	1.068583496	-0.437584647	0.661687403	0.948537489
TAC4	17.68200121	0.355992546	0.813844213	0.437420996	0.66180606	0.9485753
ACADVL	2077.947536	-0.06228187	0.142391458	-0.43739892	0.661822068	0.9485753
PRMT7	320.777871	0.105345986	0.240904684	0.437293223	0.66189871	0.9485753
ZNF101	152.984091	-0.092323992	0.211144693	-0.437254618	0.661926703	0.9485753
SAXO1	3.066232469	0.540303226	1.236646207	0.436910106	0.662176542	0.948848823
WDTC1	551.3240498	-0.137242981	0.314277286	-0.436693924	0.662333336	0.948848823
DDX55	910.7096014	0.081907737	0.187575931	0.436664429	0.66235473	0.948848823
ALG10	354.5511641	-0.053714843	0.123022287	-0.436626928	0.662381931	0.948848823
LOC105372483	17.41762514	0.213638633	0.489296198	0.436624347	0.662383803	0.948848823
ZBTB46	7.842858029	-0.30405194	0.69656262	-0.436503383	0.662471545	0.948861168
LOC414300	8.403963376	-0.309516813	0.70918045	-0.436442958	0.662515377	0.948861168
ZNRF2P2	7.50658584	-0.396134725	0.907749221	-0.43639225	0.662552161	0.948861168
MTRNR2L4	3.12805915	0.625866652	1.434899194	0.436174649	0.66271002	0.949010973
SPDYE1	41.02455141	0.213330757	0.489288088	0.436002351	0.662835024	0.949041664
ARF3	3094.011427	-0.047189468	0.108233148	-0.435998297	0.662837965	0.949041664
LINC02202	7.018293648	-0.380893158	0.873765683	-0.435921399	0.662893759	0.949045295
CAMK2D	966.8919227	-0.086160581	0.197702407	-0.43580947	0.662974972	0.94907141
RPS6KA4	464.2344895	0.09312164	0.2137567	0.435643141	0.663095665	0.94907141
MLF1	630.835884	0.070940544	0.162853569	0.435609393	0.663120155	0.94907141
OGA	1644.284832	-0.047145826	0.108231262	-0.435602668	0.663125035	0.94907141
MMP25	51.4005244	0.196522892	0.451283434	0.435475529	0.663217297	0.949127228
TMEM9	1040.766259	-0.07149389	0.164350878	-0.435007653	0.663556872	0.949536936
PEX19	973.74453	0.067150128	0.154392801	0.434930436	0.663612921	0.949540891
CCDC80	2271.493827	0.079771333	0.183600622	0.434482913	0.6639378	0.949823977
ASH1L	1739.554193	-0.048836937	0.112413903	-0.434438586	0.663969983	0.949823977
LOC105374972	9.295522974	0.313880039	0.722497334	0.434437644	0.663970667	0.949823977
MAN1B1-DT	53.03233639	0.128329839	0.295549385	0.434207769	0.664137572	0.949871665
UBE2W	781.7754276	-0.049528751	0.114075107	-0.43417668	0.664160146	0.949871665
ZBED3-AS1	14.94364843	-0.240454167	0.553838154	-0.434159628	0.664172528	0.949871665
CDCA5	612.8533604	0.071116557	0.163843399	0.434052009	0.664250674	0.949871665
CHRD	6.653817564	-0.392825055	0.905075394	-0.434024677	0.664270521	0.949871665
ZNF24	2108.434213	0.044408213	0.102356132	0.433859822	0.664390237	0.949896954
RIMS3	16.63477526	-0.225761648	0.520556681	-0.433692729	0.664511587	0.949896954
CCDC50	1726.787854	-0.086222211	0.198830507	-0.433646791	0.664544951	0.949896954
ZFP3	42.68673636	-0.152621134	0.351973961	-0.433614843	0.664568154	0.949896954
PGAP4	1125.234588	0.052890781	0.121987803	0.433574344	0.664597568	0.949896954
MIR4307HG	30.19499608	0.375886801	0.867038601	0.433529488	0.664630148	0.949896954
CLN5	716.0592976	0.05532922	0.127645673	0.433459426	0.664681036	0.949896954
MIR382	7.81494817	-0.44959643	1.037716723	-0.433255454	0.664829197	0.949896954
CACNA2D1-AS1	12.50074771	0.266160685	0.614354393	0.433236399	0.664843038	0.949896954
MIOX	4.865122598	0.4179056	0.964919463	0.433098944	0.66494289	0.949896954
DAD1	1115.377942	-0.075874982	0.175220117	-0.433026662	0.664995401	0.949896954
PPID	312.1207437	0.064616064	0.149224918	0.433011223	0.665006617	0.949896954
ABHD15	555.102556	-0.063406135	0.146450502	-0.432952667	0.665049157	0.949896954
ZNF852	61.64115264	0.134363774	0.310413278	0.432854466	0.665120502	0.949896954
PPP2R2D	277.734151	-0.073244915	0.169216154	-0.432848242	0.665125023	0.949896954
ITPR3	4083.353935	0.066244642	0.153051393	0.43282613	0.665141088	0.949896954
MCAT	149.7030917	-0.089695561	0.20733377	-0.432614336	0.665294973	0.950002534
RMRP	16505.60813	-0.13997397	0.323581153	-0.432577635	0.66532164	0.950002534
PRMT1	2308.731642	-0.063802801	0.147540993	-0.43244118	0.665420794	0.950067987
ZNF670-ZNF695	5.245980899	0.42798874	0.989879836	0.432364338	0.665476633	0.950071591
RHOBTB3	3604.121202	0.065232904	0.150923374	0.432225326	0.665577654	0.950139694
STX16-NPEPL1	8.556242815	0.310512883	0.718667711	0.432067392	0.665692433	0.950210186
CIAO3	298.1248616	0.08212047	0.190089	0.43201064	0.66573368	0.950210186
TMEM38A	20.07061824	0.277961456	0.643581589	0.431897775	0.665815712	0.950251161
SLC35A1	394.2537128	-0.069724983	0.161482692	-0.431779918	0.665901376	0.950252435
INTS3	1852.196953	-0.058145333	0.134685522	-0.431711829	0.665950869	0.950252435
TGFB2-AS1	6.744166158	0.351601932	0.814503377	0.431676457	0.665976581	0.950252435
SOWAHD	3.305119012	0.45537861	1.055495077	0.431436034	0.666151354	0.950296868
TPM1	4891.014324	0.061836627	0.143349429	0.43136989	0.66619944	0.950296868
GFER	208.8030078	-0.073205857	0.169715298	-0.431345068	0.666217485	0.950296868
BRF1	631.1323004	-0.076362733	0.177049974	-0.431306097	0.666245818	0.950296868
CHPF	1352.872099	-0.079131215	0.183506384	-0.431217779	0.666310027	0.950296868
VGLL4	874.0448337	-0.122343301	0.28373179	-0.431193491	0.666327686	0.950296868
AFG3L2	1461.906964	-0.047534006	0.110273861	-0.431054159	0.666428991	0.950365287
HUWE1	10293.9064	-0.055681675	0.129201387	-0.430968091	0.666491572	0.950374184
JPH4	99.27596286	-0.139937537	0.324780665	-0.430867818	0.666564484	0.950374184
RPL23AP53	431.4114249	-0.088328528	0.205021566	-0.430825542	0.666595226	0.950374184
C11orf42	5.950820422	0.388053545	0.901019761	0.430682613	0.666699163	0.950422491
STX11	57.777234	-0.109409549	0.25414862	-0.430494365	0.666836065	0.950422491
SCARF1	6.483035672	0.428668995	0.995882891	0.430441168	0.666874754	0.950422491
CHRM4	2.234195515	0.575411114	1.337175955	0.430318173	0.666964209	0.950422491
SCLY	12.08524602	-0.237194668	0.551288154	-0.430255333	0.667009915	0.950422491
POMZP3	42.31278755	0.172526895	0.400998061	0.430243713	0.667018367	0.950422491
DIPK2B	2.430527905	-0.517519231	1.202891502	-0.430229351	0.667028813	0.950422491
SNORD113-4	37.67331456	0.191751418	0.445734225	0.430192269	0.667055785	0.950422491
PLGRKT	119.0527656	-0.094937282	0.220731475	-0.430103057	0.667120676	0.950438955

ZBTB34	1004.601185	0.051343831	0.119418084	0.429950217	0.667231855	0.95045881
OXR1	2320.590523	0.090167042	0.209755052	0.429868274	0.667291465	0.95045881
SCARNA21	20.23538022	0.190232815	0.442568194	0.429838424	0.66731318	0.95045881
RGS14	78.12306155	-0.1018171	0.236899309	-0.429790617	0.667347959	0.95045881
CCT6B	9.317241021	-0.306471143	0.71319669	-0.429714759	0.667403146	0.950461445
CCDC88C	106.8307503	0.119059342	0.277177933	0.4295412	0.667529418	0.950484398
COL9A3	6.077188979	0.417095401	0.971236967	0.429447617	0.667597507	0.950484398
DDX59	227.3001694	0.094784763	0.220743459	0.429388773	0.667640323	0.950484398
SATL1	4.202215329	0.396669472	0.924042329	0.4292763	0.667722162	0.950484398
CROCCP2	232.6722933	0.120932906	0.281726739	0.429256045	0.667736901	0.950484398
TRHDE	530.5205659	0.072192843	0.168211219	0.429179714	0.667792445	0.950484398
SLC5A11	2.650805477	0.613952677	1.430664524	0.429138115	0.667822716	0.950484398
ULK2	353.4756077	-0.0784158	0.182769082	-0.429043029	0.667891911	0.950484398
MPC1	161.1435883	0.07841505	0.182771661	0.429032867	0.667899306	0.950484398
TEN1-CDK3	15.24998857	0.201690376	0.470245856	0.4289041	0.667993016	0.950497329
PEA15	1460.187838	-0.053765832	0.125365159	-0.428873797	0.66801507	0.950497329
KLF16	538.155709	0.052768157	0.123158326	0.428457891	0.668317785	0.950755788
PHF14	932.1065368	-0.075154861	0.175412978	-0.428445271	0.668326971	0.950755788
TMIE	2.032787229	-0.61358599	1.432259093	-0.428404325	0.668356777	0.950755788
GAL3ST1	4.682062427	0.447987638	1.046070386	0.428257643	0.668463553	0.950762451
LOC105374194	5.774668692	0.456453168	1.065955936	0.428210166	0.668498116	0.950762451
LOC105377623	8.770085474	0.325641399	0.760528071	0.428178014	0.668521522	0.950762451
FAM104B	58.34483728	0.113454967	0.265161255	0.427871588	0.668744614	0.950880159
LAT	14.1490724	0.291095887	0.680494485	0.427771119	0.668817766	0.950880159
NID1	6010.902092	-0.089125943	0.208356964	-0.427756006	0.66882877	0.950880159
ANKRD37	19.13770949	-0.216826386	0.50689729	-0.427752111	0.668831606	0.950880159
LSP1P3	2.425077071	-0.642463666	1.502314398	-0.427649277	0.668906484	0.950880159
GALNT18	278.4863749	0.128923288	0.301487067	0.427624606	0.668924448	0.950880159
ATRX	2123.02416	-0.042916495	0.100384415	-0.427521494	0.668999533	0.950884272
EFEMP2	612.8100909	0.0703148	0.164489039	0.427474076	0.669034063	0.950884272
RSRC2	617.0891239	0.081058908	0.189734126	0.427223661	0.669216429	0.950896354
INPP5F	223.9823702	0.06324939	0.148055115	0.427201654	0.669232457	0.950896354
LPO	4.520025406	-0.399727995	0.935993899	-0.427062608	0.669333727	0.950896354
LINC00667	581.9459868	0.051268079	0.120071349	0.426980116	0.669393811	0.950896354
FAM126A	1734.342138	-0.041952294	0.09825496	-0.426973803	0.669398409	0.950896354
POLN	20.26436629	-0.192237399	0.450296897	-0.426912554	0.669443021	0.950896354
PRRT3-AS1	24.35501574	0.207023996	0.485042587	0.426816122	0.669513263	0.950896354
HCG17	3.501400294	-0.463869331	1.086881601	-0.426789202	0.669532872	0.950896354
AHCTF1P1	17.75856476	0.190449092	0.44625076	0.42677595	0.669542526	0.950896354
LDAH	374.0072204	-0.056843272	0.133230355	-0.426654064	0.669631313	0.950896354
CTNS	417.3038914	-0.072005066	0.168774628	-0.426634423	0.669645621	0.950896354
ARTN	6.387809535	0.331097439	0.776161365	0.426583252	0.669682898	0.950896354
MPZ	2.267277739	0.562047164	1.318107484	0.426404652	0.669813012	0.950968366
MAGEA8	2.583845927	0.591509505	1.387324334	0.426367137	0.669840344	0.950968366
SNORA84	6.361829923	-0.346024995	0.811936624	-0.426172419	0.669982214	0.951005236
BTBD10	338.2482778	0.056792071	0.133320682	0.425980949	0.670121728	0.951005236
ZKSCAN2	532.5321125	0.062579152	0.146923468	0.425930265	0.670158661	0.951005236
IPO11	444.0782473	-0.092355377	0.216838008	-0.425918765	0.670167041	0.951005236
ARPC3	1308.590959	-0.071578764	0.168082604	-0.425854679	0.670213741	0.951005236
MIS12	396.6534924	0.053447276	0.125506458	0.425852793	0.670215115	0.951005236
AGAP1-IT1	13.46544569	0.343575015	0.807130702	0.425674571	0.670344994	0.951005236
MBD4	557.1394278	0.057801813	0.135799987	0.425639311	0.670370691	0.951005236
XKR5	340.4544627	-0.120443168	0.283106282	-0.425434457	0.670519992	0.951005236
SELL	10.89736956	0.258673843	0.608106872	0.425375628	0.67056287	0.951005236
LOC102724580	5.736751409	0.350085531	0.823099397	0.425325947	0.670599081	0.951005236
ACTA2	1444.063659	0.108649457	0.255451358	0.425323464	0.670600891	0.951005236
MIRLET7A1	4.659264163	-0.370311502	0.870791567	-0.425258485	0.670648254	0.951005236
LINC02861	2.947534854	0.558137818	1.312494607	0.425249609	0.670654724	0.951005236
CARD6	70.62121584	0.171813051	0.404044483	0.425233008	0.670666824	0.951005236
VWA8-AS1	4.250075875	0.442281854	1.040352361	0.425126976	0.670744114	0.95101878
SETD9	75.05462744	-0.107164791	0.252155235	-0.424995307	0.670840096	0.95101878
SLC39A13	723.3189052	0.063668317	0.149814757	0.424980278	0.670851052	0.95101878
VENTXP7	8.176122141	-0.317240064	0.746666379	-0.42487525	0.670927618	0.95101878
STX18	207.5826279	0.092924887	0.218722005	0.424853854	0.670943216	0.95101878
PITX1	790.5403621	-0.072796506	0.171463435	-0.424559944	0.671157499	0.951246848
CCSER1	42.99162362	0.169460227	0.399405088	0.424281593	0.671360461	0.951448773
NASP	1278.240137	-0.071722459	0.169097658	-0.424148153	0.67145777	0.951448773
OMG	18.37535602	0.40278612	0.949867156	0.424044686	0.671533224	0.951448773
NPHP3-ACAD11	2.630863634	-0.526785661	1.24232847	-0.424030902	0.671543277	0.951448773
CCDC40	54.33733992	0.142843687	0.336896703	0.423998471	0.671566929	0.951448773
NFKBID	51.21037564	0.172137149	0.406107986	0.423870387	0.671660341	0.951487213
N4BP2L2	927.7781464	-0.072970441	0.172184461	-0.423792256	0.671717326	0.951487213
RUNDC3A	4.867047567	0.393859928	0.929507976	0.423729477	0.671763115	0.951487213
TRAPPC9	589.255038	-0.085782832	0.202530636	-0.423554843	0.671890494	0.951487213
TLR7	12.77434439	-0.230097944	0.543254946	-0.423554255	0.671890923	0.951487213
STT3B	2851.216452	-0.045623351	0.107723677	-0.423522033	0.671914427	0.951487213
WDR59	920.9313761	-0.047248889	0.11164106	-0.423221429	0.672133713	0.95171816
SLC23A1	11.98561837	0.308754971	0.72973511	0.423105545	0.672218257	0.95171816
KLHL25	133.7923164	0.105030664	0.248336862	0.422936262	0.672341765	0.95171816
PIK3R1	498.0523487	-0.056153421	0.132782622	-0.422897368	0.672370144	0.95171816
LOC541473	2.908088933	-0.598545132	1.415760618	-0.422772836	0.672461008	0.95171816
TSPAN11	171.1149143	0.191627602	0.453326798	0.422714039	0.672503911	0.95171816
CACFD1	73.05830539	-0.126106247	0.298327123	-0.422711303	0.672505908	0.95171816
ATXN1L	876.0851316	0.048763821	0.115378904	0.422640704	0.672557424	0.95171816
CHRNE	18.32701272	0.218710286	0.517486416	0.422639667	0.672558181	0.95171816
ZNF619	203.2838241	0.087492572	0.207059852	0.422547255	0.672625617	0.951738009
ICMT	2685.190926	-0.051184257	0.121158239	-0.422457915	0.672690813	0.951740345
MIR4740	7.062029675	0.309407106	0.732500274	0.422398621	0.672734084	0.951740345
KCTD17	132.683292	-0.085872077	0.203389711	-0.422204628	0.672875664	0.951865073
SSPO	4.724836006	0.455600621	1.079875421	0.421901093	0.673097212	0.951955531

STRADB	434.5489899	0.060205004	0.142714929	0.421854981	0.673130871	0.951955531
FRRS1	708.9248942	-0.056330862	0.133554029	-0.421783324	0.673183179	0.951955531
LYPD6	87.55910468	-0.148313546	0.351699304	-0.421705542	0.673239959	0.951955531
OR51J1	3.957400244	0.571611173	1.355553459	0.421681026	0.673257856	0.951955531
MFSD8	226.2801243	0.081480272	0.193228696	0.421677907	0.673260132	0.951955531
AASDH	537.9861987	-0.047456005	0.112647061	-0.421280454	0.673550302	0.952224194
ATP6V1FNB	16.15354602	0.247570548	0.587684458	0.421264413	0.673562014	0.952224194
MFAP3	1485.511361	0.041151063	0.097702406	0.421187821	0.673617937	0.952224194
VWF	8.028289157	0.374717806	0.890320601	0.420879631	0.67384298	0.952224194
LASP1	3799.237664	-0.057829601	0.137422808	-0.420815161	0.67389006	0.952224194
ETHE1	85.08671303	-0.123180562	0.292725871	-0.420805176	0.673897352	0.952224194
CCDC106	130.449201	-0.078958804	0.187657636	-0.420759879	0.673930431	0.952224194
LINC02012	2.49595045	0.515392765	1.225209448	0.420656865	0.674005663	0.952224194
ZNF284	269.1769877	0.076109284	0.181058416	0.420357615	0.674224227	0.952224194
PNPLA6	1534.371693	0.066371268	0.157932084	0.420251961	0.6743014	0.952224194
B2M	6960.270915	-0.093405157	0.222279083	-0.420215683	0.674327899	0.952224194
BBS9	584.7841913	0.069324765	0.165009059	0.420127025	0.674392661	0.952224194
FOXP3	7.611191955	0.304978462	0.7263558	0.419874753	0.674576952	0.952224194
ENPP5	117.7725494	0.145464237	0.346549193	0.419750616	0.674667644	0.952224194
INSYN2B	15.98664373	0.304076036	0.724457442	0.41972933	0.674683197	0.952224194
ERCC3	731.7134806	-0.051168266	0.121918235	-0.419693296	0.674709523	0.952224194
OR6C70	5.143275206	0.384327718	0.91594698	0.419596032	0.674780588	0.952224194
FAM167B	4.379906748	0.428685443	1.02210966	0.419412378	0.674914779	0.952224194
FASTKD2	460.5813813	0.050710727	0.120925218	0.419356091	0.674955909	0.952224194
LOC100132057	5.612513147	-0.366611714	0.87441047	-0.419267297	0.675020794	0.952224194
KIF18B	853.4430506	0.05944004	0.14179593	0.41919426	0.675074166	0.952224194
RBM12	1983.700114	0.079657051	0.190096405	0.41903502	0.675190538	0.952224194
RASEF	13.4333684	-0.242839823	0.579569964	-0.419000014	0.675216122	0.952224194
MYLK4	21.91262347	0.189518635	0.452368446	0.418947512	0.675254493	0.952224194
SAMD14	5.823284103	0.331013109	0.790155509	0.418921472	0.675273524	0.952224194
RUVBL1	1083.303139	-0.054950231	0.131176911	-0.418901698	0.675287976	0.952224194
SON	7138.603975	0.050698951	0.121042145	0.418853704	0.675323053	0.952224194
LOC105378047	4.807296198	0.468775386	1.119265277	0.418824201	0.675344616	0.952224194
SFXN4	338.4668525	0.08024257	0.191635268	0.418725485	0.675416768	0.952224194
RAC2	8.662337484	-0.286923636	0.685249546	-0.418714085	0.675425101	0.952224194
ZNF525	238.8497117	0.068454182	0.163519946	0.418628944	0.675487333	0.952224194
PTH2R	268.5887381	0.081870465	0.195571051	0.418622617	0.675491958	0.952224194
SLC25A20	82.03259725	0.11886932	0.283995455	0.41856064	0.67553726	0.952224194
TMEM256	197.0529049	-0.121136166	0.289416488	-0.418553091	0.675542778	0.952224194
SHISA3	2.146346161	0.549101694	1.312206392	0.418456805	0.675613162	0.952224194
MRPL18	422.2152566	-0.07001062	0.167321494	-0.418419766	0.675640237	0.952224194
CFAP97	1001.99595	0.057079811	0.13644485	0.418336133	0.675701375	0.952224194
HOXD11	25.89731706	0.182695143	0.436755085	0.418301124	0.675726968	0.952224194
WNT5A	227.2482288	-0.177259166	0.423776895	-0.418284168	0.675739364	0.952224194
SNX22	170.9699069	0.081818678	0.195647314	0.418194742	0.67580474	0.952224194
POLG2	255.2017457	0.087993396	0.210443866	0.418132385	0.675850328	0.952224194
ZC2HC1B	10.98423373	-0.405420873	0.96961389	-0.418126098	0.675854924	0.952224194
GPR137	160.7208833	-0.110755772	0.264914268	-0.418081567	0.675887482	0.952224194
CNFN	2.318539801	-0.585665717	1.400901461	-0.418063464	0.675900717	0.952224194
ZNF30-AS1	2.431793083	0.773392684	1.850687396	0.417894824	0.676024017	0.952224194
SLC52A1	5.080496287	0.411516111	0.984742354	0.417892162	0.676025964	0.952224194
CAMSAP1	1336.27287	-0.039914469	0.095527491	-0.41783228	0.676069748	0.952224194
HGH1	152.4789919	0.10580039	0.253235433	0.417794573	0.676097319	0.952224194
LOC105372795	5.256985185	-0.337398868	0.807629736	-0.417764296	0.676119458	0.952224194
PIH1D2	11.13412669	-0.280670712	0.671863285	-0.417749739	0.676130102	0.952224194
CDK12	1691.367738	0.067178839	0.160835036	0.417687842	0.676175362	0.952224194
RBFA	374.0995749	0.077048066	0.184550423	0.417490597	0.6763196	0.952251422
MECP2	782.6943038	-0.055669827	0.133351676	-0.417466271	0.67633739	0.952251422
ATP5MC2	983.7589071	-0.066353713	0.158953063	-0.41744218	0.676355008	0.952251422
SNORA20	3.002294907	0.446420213	1.069794021	0.417295483	0.676462292	0.952327228
KIF16B	14.51956054	-0.226289055	0.542453204	-0.417158667	0.676562356	0.952392857
EAF1	466.289531	0.067304383	0.161396512	0.417012626	0.676669172	0.952467982
DLK2	181.0127685	-0.083298624	0.19986616	-0.416772026	0.676845165	0.952638683
SOX30	2.418595542	0.563451014	1.35220812	0.416689565	0.676905488	0.952638683
CIB1	637.0509683	-0.058074727	0.139392417	-0.416627595	0.676950822	0.952638683
DMC1	3.572692518	-0.458014392	1.099703115	-0.416489129	0.677052121	0.95266869
TMC6	18.04861616	0.21822668	0.524013629	0.416452298	0.677079066	0.95266869
PCDHGA12	12.71721302	0.284956411	0.684521954	0.416285277	0.677201265	0.952723703
CCS	119.5230235	-0.10677619	0.256517705	-0.416252711	0.677225092	0.952723703
NHLRC1	38.81158257	-0.131467556	0.316031226	-0.415995461	0.677413325	0.95277717
NKRF	420.7512001	-0.049333967	0.118598429	-0.415974882	0.677428383	0.95277717
ATP5IF1	131.0355065	-0.097495759	0.234440181	-0.415866252	0.677507875	0.95277717
TMEM266	11.54440311	-0.248126884	0.596763325	-0.415787756	0.677565319	0.95277717
ELMO1	267.6462007	-0.067351519	0.161988976	-0.41577841	0.677572159	0.95277717
B3GNT5	933.8283103	0.045601732	0.109682202	0.415762368	0.677583899	0.95277717
PARP10	200.2135801	-0.140818641	0.338869075	-0.415554713	0.677735871	0.952860204
GLRA2	6.021425901	0.361071663	0.869059146	0.415474212	0.67779479	0.952860204
PFDN1	393.0244519	-0.064515593	0.155289432	-0.415453853	0.67780969	0.952860204
ELF3-AS1	5.101381351	0.396257874	0.953998237	0.415365415	0.67787442	0.952860204
PSMA1	1633.635558	-0.044346023	0.10679482	-0.415245073	0.677962506	0.952860204
MPHOSPH6	219.6682227	0.109239419	0.263081338	0.415230588	0.677973109	0.952860204
WRAP53	238.9185759	0.070767808	0.170463377	0.415149632	0.678032368	0.952860204
LOC101926889	5.877897128	-0.335892991	0.8091911	-0.415097239	0.678070721	0.952860204
ATRN	1785.008176	-0.041987071	0.101186908	-0.414945686	0.678181664	0.952915955
TRIM68	179.5043513	-0.080759341	0.194705739	-0.414776378	0.678305614	0.952915955
DBF4B	262.5359868	0.093198772	0.224703067	0.414764128	0.678314582	0.952915955
ZNF597	199.2012265	0.073425456	0.177105448	0.414586095	0.678444929	0.952915955
GABPB1-IT1	192.8751065	0.119871567	0.289136583	0.414584573	0.678446043	0.952915955
SEMA4A	47.90129722	0.217755158	0.525258165	0.41456787	0.678458273	0.952915955
MIR194-1	2.154217325	0.502845638	1.213044913	0.414531756	0.678484716	0.952915955

CDKN1B	390.9668953	-0.094215108	0.227508144	-0.414117517	0.678788045	0.953112859
AGAP3	715.404467	0.055602681	0.134269968	0.414111076	0.678792761	0.953112859
ZNF414	139.2951716	0.072941242	0.176140775	0.414107649	0.678795272	0.953112859
GNG12	2000.916243	-0.044255078	0.106888705	-0.414029512	0.678852494	0.953112859
PRKN	20.67621614	0.217863793	0.526378199	0.413892129	0.678953109	0.953112859
TBC1D22A	288.8599028	-0.07013075	0.169476318	-0.413808552	0.679014321	0.953112859
SEMA5A-AS1	7.590932574	-0.380410429	0.919453513	-0.413735359	0.679067929	0.953112859
PNKP	657.4978228	0.077339473	0.186963844	0.413660049	0.679123089	0.953112859
LOC729683	61.43008086	-0.154622065	0.373794209	-0.413655592	0.679126354	0.953112859
SSTR2	80.80695649	-0.165734298	0.400822049	-0.413485981	0.679250592	0.953112859
CELSR2	98.05707014	0.137756834	0.333205539	0.413429003	0.679292329	0.953112859
MROH8	4.145563244	-0.386943961	0.93595743	-0.413420471	0.679298579	0.953112859
PLEKHH1	42.82761553	-0.15192546	0.367529433	-0.413369507	0.679335912	0.953112859
DACH1	108.6827909	0.115739572	0.280025544	0.413317908	0.679373711	0.953112859
ANAPC15	49.84273464	0.127010309	0.307482425	0.413065264	0.679558798	0.953297471
SCARNA20	4.990520527	-0.430042568	1.041583778	-0.412873719	0.679699137	0.953419287
ZWINT	704.9613738	-0.083829901	0.203122331	-0.412706473	0.679821682	0.953506882
PTGES2-AS1	6.300853143	0.309261918	0.749467057	0.412642444	0.679868599	0.953506882
MRPL17	249.4131006	0.070481466	0.170884261	0.41245148	0.680008537	0.953628089
SCARNA14	2.684360569	-0.469752743	1.139674018	-0.412181672	0.680206269	0.953772415
C5orf38	33.44428162	-0.144672123	0.351005368	-0.412164988	0.680218497	0.953772415
C3orf70	267.915683	0.109892021	0.266820132	0.411858059	0.680443464	0.954012787
FAM86DP	212.6304474	0.111765537	0.271425776	0.411772008	0.68050654	0.954026162
FAM90A25P	2.0163919	0.574600893	1.395979944	0.411611138	0.680624467	0.954063244
DUSP19	164.6335291	-0.117876462	0.286471261	-0.411477442	0.680722479	0.954063244
ANKRD44-IT1	14.89328402	-0.360575244	0.876378966	-0.411437583	0.6807517	0.954063244
RASA3	1128.43415	-0.091183367	0.221685036	-0.411319451	0.680838309	0.954063244
ZNF595	84.69886845	0.095339794	0.231834488	0.411240772	0.680895995	0.954063244
CBWD1	135.622043	-0.086137934	0.209475389	-0.411207898	0.680920097	0.954063244
LINC01303	11.86575942	0.262121899	0.637562265	0.41113145	0.68097615	0.954063244
EAF2	10.11694346	0.299676129	0.728935698	0.41111463	0.680988483	0.954063244
HIC1	314.1762428	0.103032114	0.250638426	0.411078682	0.681014841	0.954063244
SNCA	93.83277289	-0.118388569	0.288091596	-0.410940724	0.681116	0.954129952
C9orf16	92.85993476	-0.133121059	0.324021169	-0.410840623	0.681189404	0.954157772
CAPN12	7.42196276	0.288986014	0.703972755	0.410507384	0.681433788	0.954294172
DPEP1	2.009807646	0.567409516	1.382285226	0.410486566	0.681449056	0.954294172
LIPT2	10.68634437	0.267330147	0.651381233	0.410405049	0.681508843	0.954294172
TRADD	105.6876261	-0.083717503	0.204019492	-0.410340708	0.681556034	0.954294172
FOXO1	94.97774129	-0.122053548	0.297569772	-0.41016783	0.681682838	0.954294172
SHISA8	2.766741008	-0.599727927	1.462209214	-0.410151927	0.681694503	0.954294172
DICER1-AS1	36.03624707	0.198884449	0.484931519	0.410128938	0.681711366	0.954294172
CEP104	704.0824364	0.057082204	0.139182891	0.410123715	0.681715197	0.954294172
IKBIP	919.3515395	0.055614191	0.135724444	0.409758107	0.6819834	0.954375967
LRRC3	13.49792166	-0.224026229	0.547036526	-0.409527003	0.682152955	0.954375967
VARS2	729.4261664	-0.047214936	0.115294989	-0.40951421	0.68216234	0.954375967
NAA50	3302.720353	-0.04691047	0.114584639	-0.409395798	0.682249223	0.954375967
SGK2	1.840222133	0.706219393	1.725141819	0.409368891	0.682268966	0.954375967
ROPN1L-AS1	2.651907578	0.568432847	1.388674066	0.409334963	0.682293861	0.954375967
RFC4	1332.941219	-0.04473582	0.109318528	-0.409224505	0.682374913	0.954375967
TIMM23B-AGAP6	24.28411641	0.192950706	0.471525168	0.409205529	0.682388837	0.954375967
HLA-DOB	12.88896011	0.212935177	0.520519145	0.409082316	0.682479253	0.954375967
CLDN5	77.81914623	0.489506615	1.19662609	0.409072323	0.682486586	0.954375967
ZNF625	83.72015675	-0.098075006	0.239756816	-0.409060343	0.682495378	0.954375967
TM9SF2	2961.225797	0.049298282	0.12052943	0.409014484	0.682529032	0.954375967
ZNF263	454.0123491	-0.044454467	0.108699508	-0.408966591	0.682564179	0.954375967
SCRG1	14.30322207	-0.304365519	0.744298916	-0.408929144	0.682591661	0.954375967
HBS1L	982.0160231	-0.047423525	0.115986685	-0.408870424	0.682634755	0.954375967
ZNF383	215.0931814	-0.071569126	0.17505922	-0.408828089	0.682665825	0.954375967
CHKB-DT	17.33235772	0.241055093	0.589660479	0.408803205	0.682684088	0.954375967
ABITRAM	284.928971	-0.055800825	0.136603061	-0.408488834	0.682914827	0.954456852
COG5	1133.350579	-0.049032763	0.120039649	-0.408471398	0.682927625	0.954456852
AQP11	28.63482942	0.183632913	0.449676147	0.408367031	0.683004234	0.954456852
LINC01260	3.432730242	0.587009951	1.437761077	0.408280597	0.683067683	0.954456852
INHA	4.135394626	0.381335649	0.934051488	0.408259774	0.683082968	0.954456852
YWHAQ	8134.896553	-0.056073745	0.137352301	-0.408247586	0.683091915	0.954456852
PIN1	472.1875476	-0.059041423	0.144658858	-0.408142463	0.683169087	0.954456852
ACTR8	350.8790012	0.055519842	0.136031159	0.408140627	0.683170435	0.954456852
RC3H2	2081.224071	0.043193829	0.105895574	0.40789079	0.683353855	0.954638264
CCZ1	314.2299832	-0.070564836	0.17309271	-0.407670758	0.683515409	0.954674845
AGBL2	21.27425244	-0.181199982	0.444738387	-0.407430497	0.683691832	0.954674845
BTK	2.335392481	-0.67161089	1.648507773	-0.407405353	0.683710296	0.954674845
SERBP1	4898.30069	0.042371004	0.104008434	0.407380467	0.683728571	0.954674845
IL1RAPL1	3.323216279	-0.404335797	0.99275866	-0.407285086	0.683798615	0.954674845
CNTD1	16.90425631	0.220319434	0.541071989	0.407190612	0.683867996	0.954674845
RNF6	504.4578283	-0.05778266	0.141924875	-0.407135539	0.683908443	0.954674845
LINC01969	3.162627619	0.482908842	1.186292842	0.40707389	0.683953719	0.954674845
PIGO	649.6855486	0.058402017	0.143484657	0.407026213	0.683988736	0.954674845
CIAO2A	718.7174048	0.044025094	0.10817178	0.406992421	0.684013555	0.954674845
COPS7B	359.0569892	0.083881148	0.206127717	0.406937743	0.684053714	0.954674845
DLSTP1	51.18856646	0.167717228	0.412219195	0.406864187	0.684107741	0.954674845
HIPK1	2162.712062	0.074684575	0.183601804	0.406774734	0.684173445	0.954674845
HIPK4	4.170096088	0.426575764	1.048706124	0.406763873	0.684181423	0.954674845
RAB11B	595.9236077	-0.069722526	0.17146942	-0.406617844	0.684288689	0.954674845
STAM2	366.6073317	0.068838395	0.169304573	0.406595012	0.684305462	0.954674845
ERGIC2	693.914564	-0.04932895	0.121333341	-0.406557254	0.684333198	0.954674845
TBC1D10A	242.0626093	0.061270823	0.150712136	0.406542061	0.684344358	0.954674845
ZC3H11A	4481.974469	0.061391428	0.151043121	0.406449676	0.684412226	0.954694784
GRM3	6.103950128	0.426325324	1.049968656	0.406036239	0.684715974	0.954895124
LOC100130673	2.15340169	0.481353874	1.185580263	0.40600699	0.684737465	0.954895124
LARS2	494.6596406	0.068056151	0.167659651	0.405918484	0.684802496	0.954895124

MIR25	4.13710662	-0.525503905	1.294677581	-0.405895578	0.684819327	0.954895124
PPP4R3A	2004.478777	0.044345291	0.109262918	0.405858567	0.684846523	0.954895124
SCX	3.187932427	0.587717962	1.448408337	0.405768143	0.684912968	0.954895124
DPH7	505.890238	0.120191453	0.296416676	0.405481413	0.685123678	0.954895124
CAPN3	33.61377771	0.169372769	0.417964071	0.405232843	0.685306366	0.954895124
TEPSIN	241.5969062	-0.101771161	0.251249528	-0.405060107	0.685433329	0.954895124
TRMT61A	415.2253085	-0.071311196	0.176060974	-0.405036926	0.685450368	0.954895124
MORC1	5.378175211	-0.466936098	1.152829058	-0.405034983	0.685451797	0.954895124
FBXO16	49.94001581	0.11512682	0.284304791	0.404941541	0.685520483	0.954895124
HHAT	204.162612	0.076438403	0.188785359	0.404895822	0.685554089	0.954895124
FAM193A	512.8820319	-0.058155434	0.143633392	-0.404887978	0.685559855	0.954895124
SMIM1	14.6089566	0.229851145	0.567744589	0.404849557	0.685588099	0.954895124
TRIM24	595.2128611	-0.066971594	0.165454235	-0.404774131	0.685643546	0.954895124
CETN3	108.3490079	0.086256143	0.213124927	0.404721043	0.685682573	0.954895124
PGAM5	1708.624135	0.054420607	0.134466589	0.404714713	0.685687226	0.954895124
LINC01733	3.716288874	-0.441018244	1.089789184	-0.404682162	0.685711156	0.954895124
ZMYM4-AS1	8.983094095	-0.338243576	0.835961427	-0.404616248	0.685759614	0.954895124
FAM111A-DT	22.73305998	-0.203068728	0.502368809	-0.404222406	0.686049179	0.954895124
TBL1XR1	49127.78935	-0.037079575	0.091763441	-0.404077864	0.686155462	0.954895124
CPNE1	1917.114156	0.075330409	0.186439407	0.404047674	0.686177662	0.954895124
TUT1	440.2205756	-0.071404839	0.176744031	-0.404001418	0.686211677	0.954895124
HCRTR1	4.260584831	0.393885153	0.975081893	0.403950843	0.686248867	0.954895124
CAAP1	182.6877873	-0.072253871	0.178903404	-0.403870853	0.686307691	0.954895124
PARD6G-AS1	17.04083669	-0.263730995	0.653191376	-0.403757619	0.686390965	0.954895124
DERA	236.2567729	-0.061910546	0.153400215	-0.403588392	0.686515423	0.954895124
LOC100289495	7.586327802	0.421641054	1.044800886	0.403561157	0.686535454	0.954895124
AANAT	4.586997878	-0.373189897	0.92484102	-0.40351789	0.686567277	0.954895124
HIC2	210.9715623	0.111559314	0.276483801	0.403493131	0.686585487	0.954895124
SCARNA16	2.429682602	-0.629013668	1.55931203	-0.403391788	0.686660027	0.954895124
PELI2	799.3510658	-0.132608616	0.32892942	-0.40315219	0.686836269	0.954895124
FOXL1	96.95754078	-0.11821528	0.293371201	-0.402954617	0.686981611	0.954895124
TSL	13.92517069	0.383403366	0.951904536	0.402775017	0.687113741	0.954895124
RFWD3	1325.381163	-0.060979524	0.151404752	-0.402758321	0.687126025	0.954895124
UBOX5	138.9577708	0.083782483	0.20803942	0.402724074	0.687151222	0.954895124
TACC2	114.9040522	-0.112441296	0.279304372	-0.402576212	0.687260013	0.954895124
DUSP22	165.4049397	-0.06808283	0.169127098	-0.402554241	0.687276178	0.954895124
KIF20A	884.9974089	0.062331401	0.154847639	0.402533752	0.687291254	0.954895124
FLNA	42015.65424	-0.066306224	0.164737703	-0.402495739	0.687319224	0.954895124
RIMS2	178.074866	-0.086939335	0.216014203	-0.402470455	0.687337828	0.954895124
YAF2	234.5089002	0.127346017	0.316431613	0.402444043	0.687357262	0.954895124
GLRA1	5.141007949	0.523472629	1.300879224	0.4023991	0.687390332	0.954895124
ARL4D	450.7343367	0.105674359	0.26262096	0.402383568	0.687401761	0.954895124
DDX6	3212.360557	-0.049125205	0.122160505	-0.402136556	0.687583531	0.954895124
MAPKAP1	1530.805396	-0.051238943	0.127420045	-0.402126237	0.687591125	0.954895124
CLPP	468.8642818	-0.069944369	0.173967678	-0.402053815	0.687644422	0.954895124
TMEM70	341.2338449	-0.073049239	0.181726889	-0.401972648	0.687704156	0.954895124
SENP3-EIF4A1	25.53404273	0.19401144	0.482662401	0.401960956	0.687712761	0.954895124
PKHD1L1	17.54218074	-0.208904545	0.519821798	-0.401877233	0.687774379	0.954895124
HOXA-AS3	7.938038292	-0.257196913	0.640011651	-0.40186286	0.687784957	0.954895124
LRRC8B	393.6329167	-0.076349893	0.190010668	-0.40181898	0.687817253	0.954895124
GOLGA8T	20.89175161	0.218299812	0.54349249	0.401661138	0.687933428	0.954895124
NBDY	167.0092639	-0.092510883	0.230363999	-0.40158568	0.68798897	0.954895124
UBR2	1395.820016	-0.044925354	0.11188457	-0.401533059	0.688027703	0.954895124
MIR4453HG	52.80807137	0.178245435	0.443916054	0.4015296	0.68803025	0.954895124
STAT1	1883.581603	0.065885466	0.16413448	0.401411491	0.688117191	0.954895124
OTUB1	482.6952603	-0.071709771	0.178662715	-0.401369537	0.688148074	0.954895124
LINC02728	4.00409877	-0.407234771	1.014831398	-0.401283181	0.688211645	0.954895124
PLD6	100.5695087	0.141484286	0.352630425	0.401225408	0.688254176	0.954895124
SELENON	953.2046505	0.085453869	0.212991341	0.401208182	0.688266857	0.954895124
PPP2R3A	292.2151276	0.07019049	0.174966769	0.401164692	0.688298874	0.954895124
LINC00618	4.471793689	0.405687674	1.011289732	0.4011587	0.688303286	0.954895124
EXOG	280.3815907	0.109389014	0.272726643	0.401093975	0.688350936	0.954895124
RASGRP4	5.231617905	-0.384874619	0.959688594	-0.401041151	0.688389827	0.954895124
NOC2L	2437.617571	0.0584116	0.145745373	0.400778416	0.688583271	0.954895124
NDUFB6	329.829041	0.072357854	0.180629697	0.4005867	0.688724439	0.954895124
ZCCHC10	201.2965302	0.067862351	0.169430765	0.400531458	0.688765118	0.954895124
SMC6	1076.440526	-0.036251724	0.090521537	-0.400476236	0.688805783	0.954895124
HOXD-AS2	38.94550782	0.207568121	0.51839353	0.400406465	0.688857163	0.954895124
KLHL42	507.0643223	-0.06712967	0.167666613	-0.400375894	0.688879677	0.954895124
INCENP	771.0440613	0.049200834	0.122918954	0.400270527	0.688957274	0.954895124
POU6F1	238.0978713	0.085143515	0.21281881	0.40007514	0.689101174	0.954895124
SPEF1	4.036179846	-0.41694237	1.042254761	-0.400038825	0.689127921	0.954895124
RPS13	2767.499894	-0.066676642	0.166698889	-0.399982519	0.689169392	0.954895124
MED22	749.8962244	-0.107682959	0.269239466	-0.399952355	0.689191609	0.954895124
LINC00668	42.05564904	0.139710852	0.349375276	0.399887633	0.689239282	0.954895124
PELP1	1203.337819	-0.050622177	0.126615595	-0.399809965	0.689296491	0.954895124
SNORA5A	7.449741185	0.296071097	0.740678405	0.399729619	0.689355674	0.954895124
LINC02421	4.742597107	0.45091883	1.128160224	0.399693962	0.68938194	0.954895124
RASA4B	5.654182556	-0.447672184	1.120415493	-0.39955908	0.689481301	0.954895124
CHST14	858.2913443	0.064187035	0.160647415	0.399552247	0.689486335	0.954895124
ZNF239	295.8729825	0.120859882	0.302492643	0.399546519	0.689490554	0.954895124
SLIT2-IT1	10.25331131	-0.463950374	1.16122021	-0.399536944	0.689497608	0.954895124
MAP4K5	1356.555914	0.035151641	0.087991351	0.399489727	0.689532392	0.954895124
LUC7L3	2012.295904	0.100116989	0.250618247	0.399480046	0.689539524	0.954895124
SUZ12	1756.425763	-0.048425058	0.121248065	-0.399388296	0.689607117	0.954895124
EEPD1	82.92215927	-0.118997616	0.297961033	-0.399373082	0.689618325	0.954895124
C10orf88	137.9721513	0.08990775	0.225264311	0.399121145	0.689803943	0.954895124
FADD	322.0685935	0.052385382	0.131288396	0.399009995	0.68988584	0.954895124
ALG1	402.5440347	-0.102281397	0.256349292	-0.398992313	0.689898868	0.954895124
PAXX	225.726713	-0.101704775	0.255001677	-0.398839633	0.690011372	0.954895124

FAXC	52.47388109	-0.122023314	0.305950038	-0.398834119	0.690015435	0.954895124
CASP8AP2	837.5279041	-0.0492552	0.123512965	-0.398785662	0.690051142	0.954895124
COMP	1.991774534	0.630675116	1.581577146	0.398763422	0.690067531	0.954895124
ADIRF	9.846156079	0.281327859	0.705514198	0.398755773	0.690073168	0.954895124
AJM1	126.9372133	-0.102761155	0.257740252	-0.398700453	0.690113934	0.954895124
C8orf88	295.4062343	0.058634719	0.147076354	0.398668563	0.690137435	0.954895124
TMC2	3.240072618	-0.444700812	1.115587336	-0.39862483	0.690169663	0.954895124
ABCA12	14.69280691	0.265044016	0.665059545	0.398526746	0.690241947	0.954895124
EDEM1	1426.024825	0.038755164	0.097256061	0.398485847	0.690272089	0.954895124
WDR12	1359.347527	-0.040438341	0.101513714	-0.398353477	0.690369646	0.954895124
FBXW2	2366.298924	-0.049151992	0.123401752	-0.398308703	0.690402646	0.954895124
ZDHHC7	856.3361865	-0.055852044	0.140231539	-0.398284471	0.690420506	0.954895124
ANKRD9	760.7675266	0.04389663	0.110221158	0.398259565	0.690438863	0.954895124
NOL8	887.7456835	-0.076828975	0.192948602	-0.398183629	0.690494833	0.954895124
CKMT2-AS1	75.81516425	-0.098709988	0.247901421	-0.39818242	0.690495724	0.954895124
ADM5	29.91127953	0.197742499	0.496685748	0.398123964	0.690538811	0.954895124
SCGB2B2	7.185737072	0.316392533	0.795047177	0.397954413	0.690663789	0.954895124
USP11	1107.596664	-0.06249867	0.157068515	-0.397907055	0.690698699	0.954895124
YBEY	139.2323136	0.080428749	0.202227208	0.397714778	0.690840443	0.954895124
TLR3	13.38653546	-0.238955449	0.601014422	-0.397586881	0.690934733	0.954895124
ANAPC10	86.87492344	-0.098545451	0.247943217	-0.397451689	0.691034405	0.954895124
PIK3C2B	235.2261575	-0.092535323	0.232829777	-0.397437667	0.691044744	0.954895124
HECTD2	253.4373389	-0.102814381	0.258771818	-0.397316764	0.691133887	0.954895124
WHAMMP1	84.76834909	0.091391976	0.230032435	0.39730039	0.69114596	0.954895124
PCSK6	2.660650602	0.51226653	1.289547249	0.397245259	0.691186611	0.954895124
ADCY3	1688.136571	0.042076835	0.105936168	0.397190459	0.691227017	0.954895124
ZHX2	179.4453085	-0.085454809	0.215167063	-0.39715562	0.691252706	0.954895124
FLG2	36.65288341	-0.298148571	0.750832324	-0.397090751	0.69130054	0.954895124
FBXW10	3.167499147	0.416157051	1.0480242	0.397087253	0.69130312	0.954895124
RAD9B	38.29882415	0.128480232	0.323570177	0.397070684	0.691315337	0.954895124
WASH2P	164.0218743	0.066440594	0.167345399	0.397026714	0.691347762	0.954895124
LOC100506639	64.53863016	0.124908991	0.314633105	0.396998881	0.691368285	0.954895124
C20orf194	498.2844882	0.071055813	0.178996388	0.396967861	0.69139116	0.954895124
C11orf16	6.524419112	0.340707388	0.858395885	0.396911721	0.691432561	0.954895124
LINC00639	2.824459709	-0.423971363	1.068304614	-0.396863738	0.691467946	0.954895124
SEPSECS-AS1	4.205903674	-0.355367232	0.895551943	-0.396813646	0.691504887	0.954895124
PSMG1	502.7392352	-0.06608358	0.166653316	-0.396533241	0.69171169	0.954895124
LEMD2	1816.923906	0.068196029	0.172006145	0.396474378	0.691755106	0.954895124
FAM133A	54.5991782	-0.150190188	0.378895003	-0.396389993	0.691817348	0.954895124
UPF1	1635.387834	0.042079574	0.106190341	0.396265548	0.69190914	0.954895124
GLTP	627.8648496	-0.076019195	0.191850995	-0.39624082	0.691927381	0.954895124
C15orf40	370.7601925	0.063656425	0.160687571	0.396150275	0.691994172	0.954895124
FAM49A	19.58455074	0.180313922	0.45522385	0.396099461	0.692031656	0.954895124
AP3B2	4.414697241	-0.36515753	0.921929801	-0.396079539	0.692046352	0.954895124
ABHD4	343.0291027	-0.136600964	0.344897013	-0.396063053	0.692058514	0.954895124
C1orf159	196.9580602	-0.09042006	0.228329922	-0.396006182	0.692100468	0.954895124
FLI1	46.63533213	-0.257057367	0.649301071	-0.395898573	0.692179854	0.954895124
LOC101927759	5.876708494	-0.346334863	0.874869946	-0.395870112	0.692200851	0.954895124
SLC7A5P2	324.952415	0.239255959	0.604434188	0.395834589	0.692227058	0.954895124
HMGA2-AS1	121.4932857	0.464526218	1.174254303	0.395592519	0.692405657	0.954895124
GRASP-AS1	3.379725235	0.450130307	1.137943593	0.395564692	0.692426188	0.954895124
JMJD1C-AS1	21.75719031	0.246442815	0.623107738	0.395505945	0.692469535	0.954895124
RABIF	76.51006562	-0.09029165	0.228331729	-0.395440662	0.692517705	0.954895124
MCOLN1	170.9862001	-0.075479924	0.190886029	-0.395418796	0.69253384	0.954895124
ZNF736	247.9452277	-0.056751555	0.143522745	-0.395418544	0.692534026	0.954895124
TTYH1	42.05448546	0.216888824	0.548694398	0.395281644	0.692635045	0.954895124
CNPY4	228.2103782	-0.063432524	0.160478959	-0.395270036	0.692643611	0.954895124
TMEM212	17.61897703	0.330750808	0.836865824	0.395225613	0.692676392	0.954895124
AP5M1	1047.722237	-0.04945224	0.125139865	-0.395175746	0.692713191	0.954895124
IL12A	17.45335261	0.23562456	0.596336938	0.395119847	0.692754443	0.954895124
RBP4	4.59925086	0.376862012	0.954240727	0.3949339	0.692891671	0.955010408
WTAPP1	5.37734765	0.305399731	0.77373418	0.394708853	0.693057768	0.955116683
SMC5-AS1	17.0254391	-0.269499614	0.682823435	-0.394684189	0.693075972	0.955116683
THBS2	132.551832	-0.084449338	0.214057251	-0.394517531	0.693198987	0.955147309
TRIM39	219.493953	0.064146066	0.162600227	0.394501698	0.693210674	0.955147309
RNF216	1202.857222	0.035892817	0.091011375	0.394377268	0.693302524	0.955147309
RAP2C-AS1	35.70025877	-0.163277791	0.414028527	-0.394363625	0.693312595	0.955147309
CHD1L	1266.398343	-0.069251032	0.175734353	-0.39406656	0.693531899	0.955374573
EMC1	4588.096326	-0.045192371	0.114725129	-0.393918673	0.693641083	0.955374573
PODNL1	19.31220086	-0.214171936	0.543969032	-0.393720826	0.693787164	0.955374573
C19orf44	150.3324855	0.090616635	0.230170183	0.393694066	0.693806923	0.955374573
PCDHGB6	2.750330167	0.675992702	1.717941537	0.393489934	0.693957657	0.955374573
LOC100128288	24.52857799	0.188069435	0.478227318	0.39326368	0.694124741	0.955374573
PTPN21	416.1412687	-0.0542741	0.138019128	-0.393236072	0.69414513	0.955374573
ELMO3	69.27156283	0.166355304	0.423170403	0.393116585	0.694233375	0.955374573
CLEC14A	2.296611121	0.473221181	1.204482612	0.392883365	0.694405629	0.955374573
DLEU1	345.4945221	0.063962887	0.162830921	0.392817816	0.694454045	0.955374573
IRX2	120.2369274	0.097765354	0.248895869	0.392796211	0.694470003	0.955374573
TAFA2	12.33237074	0.245495078	0.625078411	0.392742851	0.694509418	0.955374573
TMEM94	795.4809906	0.049377162	0.125744584	0.392678239	0.694557145	0.955374573
LOC100129534	5.850602619	0.339582447	0.864803355	0.392670132	0.694563134	0.955374573
POT1	659.5578811	0.061143962	0.155737856	0.392608218	0.694608869	0.955374573
TLCD3A	406.5266228	0.057007942	0.145217837	0.392568456	0.694638241	0.955374573
RNF144B	62.4313764	0.141985863	0.36174982	0.392497399	0.694690733	0.955374573
PRRC2B	8935.215557	0.06127271	0.156236492	0.392179247	0.694925777	0.955374573
ZC2HC1C	11.89024325	0.216914656	0.553129595	0.392158831	0.69494086	0.955374573
FCRLB	7.73397799	-0.239554097	0.61093195	-0.392112571	0.69497504	0.955374573
TOP3A	1167.780833	-0.056193041	0.143332236	-0.392047474	0.695023137	0.955374573
JOSD1	1192.19853	-0.058683062	0.149689254	-0.392032561	0.695034156	0.955374573
LOC642929	2.042673071	-0.576943001	1.471728085	-0.392017389	0.695045366	0.955374573

COA1	646.1948083	-0.063869757	0.162955578	-0.391945816	0.69509825	0.955374573
TIMM50	1077.012588	-0.051087378	0.130415345	-0.391728273	0.695258997	0.955374573
SNORA70	40.03363023	-0.147178032	0.375741836	-0.391699879	0.69527998	0.955374573
ANKRD18A	11.88088013	0.253090582	0.646448359	0.391509358	0.695420773	0.955374573
TBC1D23	947.89716	-0.080049551	0.204467887	-0.391501824	0.695426341	0.955374573
MAGOHB	309.333766	-0.106847219	0.272933031	-0.391477789	0.695444103	0.955374573
FLT3LG	37.69793239	-0.160821592	0.410844475	-0.391441535	0.695470896	0.955374573
LOC102723335	3.624748345	0.490721825	1.254303585	0.391230505	0.695626862	0.955374573
WT1	86.3391243	-0.090110981	0.230453637	-0.391015658	0.695785662	0.955374573
DOHH	177.2991218	-0.079992819	0.204606076	-0.390960137	0.695826701	0.955374573
KCNQ5-IT1	33.50631038	0.310105333	0.793414291	0.39084919	0.695908713	0.955374573
ARNT	1144.875591	0.066472111	0.170071808	0.390847322	0.695910094	0.955374573
MIR548X	2.71595702	0.608399008	1.556655224	0.390837353	0.695917463	0.955374573
SMO	995.648426	-0.043206363	0.110558476	-0.390801005	0.695944332	0.955374573
KTN1	2412.875927	0.034437113	0.088131303	0.390747807	0.695983658	0.955374573
TAF8	724.8078249	0.083695431	0.214197236	0.390740013	0.695989419	0.955374573
PRRT4	14.14507746	0.200937295	0.514366465	0.390650069	0.696055911	0.955374573
MTOR-AS1	6.674369051	-0.324874975	0.831880385	-0.390530876	0.696144029	0.955374573
ISOC2	240.4727276	-0.059615569	0.152657425	-0.390518636	0.696153078	0.955374573
SYT6	18.65054826	0.21076648	0.539736487	0.390498855	0.696167702	0.955374573
PHLPP1	643.0622599	0.049234904	0.126126487	0.390361335	0.696269374	0.955374573
TRAF7	1300.495464	0.051258066	0.131320088	0.390329213	0.696293124	0.955374573
SRSF7	1941.814043	-0.071688894	0.183679607	-0.390293162	0.696319779	0.955374573
HIGD2A	190.0067631	-0.108044242	0.276854068	-0.390257016	0.696346505	0.955374573
HOOK1	45.32704135	0.200074542	0.512813599	0.390150617	0.696425175	0.955374573
LINC01832	2.685689178	0.526860217	1.35040759	0.390149035	0.696426346	0.955374573
CYTOR	498.1481218	0.100918747	0.258672062	0.390141658	0.6964318	0.955374573
PDE12	601.2606765	-0.073425675	0.18822088	-0.39010377	0.696459815	0.955374573
LINC01237	3.087698666	-0.486808523	1.24804158	-0.390057936	0.696493706	0.955374573
SSH1	2243.756654	-0.099051378	0.253980224	-0.389996417	0.696539196	0.955374573
PYROXD2	5.536811271	0.302364197	0.775729012	0.389780699	0.696698717	0.955374573
LOC101928764	5.678089152	-0.389096845	0.998501602	-0.389680742	0.696772639	0.955374573
CCNT2-AS1	16.73697848	-0.214712951	0.550997288	-0.389680595	0.696772747	0.955374573
ATF2	1555.283857	-0.039805813	0.102179715	-0.389566682	0.696856993	0.955374573
MGAT4C	6.881061517	-0.28281672	0.726072878	-0.38951561	0.696894766	0.955374573
UBA2	2637.273316	-0.055502395	0.142520652	-0.389434053	0.696955085	0.955374573
LOC399815	24.68742754	0.19332348	0.496513312	0.389362129	0.697008283	0.955374573
MIR4766	2.55622888	0.483073396	1.241127661	0.38922136	0.697112404	0.955374573
ABALON	29.73823252	-0.165416564	0.425142513	-0.389084976	0.697213287	0.955374573
FOS	164.1837519	-0.467042805	1.200379046	-0.389079438	0.697217383	0.955374573
C2CD4A	5.480340019	-0.327215967	0.841227986	-0.388974181	0.697295246	0.955374573
RNFT1	237.1633762	0.06591349	0.169464979	0.388950511	0.697312756	0.955374573
PREP	1104.330604	-0.036348888	0.093460836	-0.388921066	0.697334538	0.955374573
STX18-AS1	33.51889662	0.144664642	0.372186283	0.388688805	0.697506365	0.955374573
VASN	1749.261404	0.125202048	0.322194808	0.388591141	0.697578621	0.955374573
USP6	17.86381311	0.215431362	0.554550079	0.388479544	0.69766119	0.955374573
UBL7	615.9560354	-0.064833747	0.166897592	-0.388464242	0.697672511	0.955374573
SWT1	155.2581348	-0.076705526	0.197478326	-0.388425036	0.69770152	0.955374573
GMNN	620.5503246	0.063965736	0.164680386	0.388423524	0.697702639	0.955374573
PDE1A	36.3106357	-0.13025151	0.335356484	-0.388397171	0.697722138	0.955374573
FAM178B	4.996110236	0.44835592	1.154462161	0.388367791	0.697743876	0.955374573
SCARNA28	4.040272102	0.367911309	0.947381648	0.388345404	0.697760441	0.955374573
MERTK	101.9814512	-0.093344497	0.240427067	-0.388244546	0.697835071	0.955374573
DVL3	2273.418786	0.047936756	0.123471066	0.388242831	0.69783634	0.955374573
SNORD116-14	64.75421007	-0.154001857	0.39678593	-0.38812328	0.697924805	0.955374573
APIP	188.7454274	0.068606245	0.176770394	0.388109365	0.697935102	0.955374573
MAP6D1	139.5916058	-0.151045394	0.389208561	-0.388083433	0.697954291	0.955374573
SPAG9	3263.989111	0.072549013	0.187035885	0.3878882	0.698098771	0.955374573
CASP6	95.85288736	-0.081364092	0.209780121	-0.387854156	0.698123966	0.955374573
CRLF1	11.72645221	-0.302730353	0.780582547	-0.387826187	0.698144665	0.955374573
TCF7	589.916915	0.093925622	0.242319198	0.387611147	0.698303819	0.955374573
RGS10	57.87662588	-0.112263548	0.289691024	-0.387528569	0.698364939	0.955374573
FBXO24	24.43719426	0.173100699	0.446845699	0.387383607	0.698472238	0.955374573
ZNF232	137.8829151	-0.076909299	0.198554698	-0.387345652	0.698500333	0.955374573
LOC100506274	6.470927022	0.441347916	1.139423007	0.387343342	0.698502043	0.955374573
PVT1	876.7067175	0.13852151	0.357625324	0.387336972	0.698506758	0.955374573
TMEM259	2592.200011	0.066378415	0.171426377	0.387212377	0.698598988	0.955374573
SMCO4	74.9615186	-0.098850996	0.255308274	-0.387182895	0.698620813	0.955374573
DMTF1	800.8106206	0.097757903	0.252488822	0.387177153	0.698625063	0.955374573
PCDHGA1	11.37904471	0.227750985	0.588286052	0.387143268	0.698650147	0.955374573
COA6	148.2085318	-0.085068001	0.219774868	-0.38706883	0.698705253	0.955374573
PLCG2	2.029956429	0.599962036	1.550062741	0.387056614	0.698714296	0.955374573
SLC25A18	8.397224765	0.263384425	0.680560696	0.387010925	0.69874812	0.955374573
ADORA2B	348.6711361	-0.055814424	0.144296556	-0.386803575	0.69890163	0.955374573
MMGT1	932.4389765	-0.049140753	0.127046244	-0.386794221	0.698908556	0.955374573
EIF2A	1501.839574	-0.059179275	0.153069479	-0.386617078	0.699039713	0.955374573
SNORA66	15.88592065	0.229737511	0.594291777	0.386573599	0.699071906	0.955374573
TUBD1	339.2252363	0.069759014	0.180484174	0.386510419	0.699118688	0.955374573
SPAG1	32.42319083	0.137702063	0.356318841	0.386457429	0.699157925	0.955374573
SMG7	1442.459919	0.050598949	0.130969834	0.386340482	0.699244523	0.955374573
LBR	2144.063375	0.072123309	0.186694592	0.386317075	0.699261856	0.955374573
DNAAF1	1.849332662	0.799115975	2.070050361	0.386036973	0.699469287	0.955374573
TLE4	366.0413407	-0.06290983	0.162965013	-0.386032734	0.699472426	0.955374573
ZNF354B	151.2477296	0.103434388	0.268107159	0.38579495	0.699648536	0.955374573
MIR663AHG	76239.1443	0.118165889	0.30633341	0.38574274	0.699687206	0.955374573
NIPAL3	858.2437098	0.105749734	0.274157614	0.385726052	0.699699566	0.955374573
C7orf31	45.16146032	-0.10904539	0.282740508	-0.385673036	0.699738835	0.955374573
RGL2	906.3869542	0.070211239	0.18205242	0.385664962	0.699744816	0.955374573
ERCC6	261.3569757	-0.067124523	0.174049911	-0.385662498	0.69974664	0.955374573
TRIM28	6070.383488	-0.042963321	0.111446535	-0.385506115	0.699862476	0.955374573

CIB2	125.9389628	-0.105185286	0.272917819	-0.385410106	0.699933597	0.955374573
MBIP	334.811352	0.049417886	0.128267144	0.385273147	0.700035055	0.955374573
LMBRD2	489.9748328	0.056633334	0.147004382	0.385249295	0.700052724	0.955374573
MIR4435-2HG	602.6234838	-0.059508593	0.154502589	-0.385162429	0.700117077	0.955374573
MIR1262	5.996726561	0.379058255	0.984537746	0.385011399	0.70022897	0.955374573
COASY	1042.458515	0.047456379	0.123289344	0.384918744	0.700297618	0.955374573
A2M	84.00467557	0.356435926	0.926006214	0.384917423	0.700298597	0.955374573
FAM110A	52.48590853	-0.113265419	0.294262719	-0.384912568	0.700302194	0.955374573
LINC00311	7.13541227	0.532403964	1.383533684	0.384814602	0.70037478	0.955374573
IFI30	89.56529791	0.095418486	0.24804889	0.384676127	0.700477385	0.955374573
DNMBP	298.7485534	0.06064212	0.15764897	0.384665501	0.700485258	0.955374573
STX17	689.8437045	0.044804559	0.116527517	0.384497671	0.700609622	0.955374573
PKP4-AS1	18.15428465	0.326041989	0.848062452	0.384455164	0.700641121	0.955374573
NALCN	6.044717762	-0.404326918	1.051696682	-0.384452024	0.700643448	0.955374573
GJC1	1613.861036	-0.036856273	0.095878101	-0.384407628	0.700676348	0.955374573
TNS1	147.7915937	-0.098678837	0.256723552	-0.384377812	0.700698443	0.955374573
RNF7	826.7791685	-0.058823964	0.153047369	-0.384351355	0.70071805	0.955374573
CYS1	57.41860036	-0.205322479	0.534262814	-0.384309881	0.700748785	0.955374573
TCL6	3.144061187	0.384175233	0.999801565	0.384251481	0.700792065	0.955374573
TMEM39B	244.2528961	-0.0643931	0.167618321	-0.384165048	0.700856122	0.955374573
HSD17B13	6.17058826	0.287878208	0.749447141	0.384120763	0.700888943	0.955374573
DCAKD	299.654336	0.11786855	0.306858581	0.384113587	0.700894261	0.955374573
PLCB2	11.49094907	0.216777793	0.564386259	0.384094739	0.700908231	0.955374573
RRP15	554.0649694	0.065453167	0.170415289	0.384080371	0.70091888	0.955374573
IFNAR1	834.3896157	-0.047685941	0.124172317	-0.384030371	0.700955937	0.955374573
MMP28	42.92541509	-0.20337603	0.529629244	-0.383996981	0.700980685	0.955374573
CYFIP1	2138.700722	-0.05967313	0.15540143	-0.383993443	0.700983307	0.955374573
BRD7	359.3482587	-0.060552783	0.157724176	-0.38391567	0.701040952	0.955380068
PRDM11	32.26728598	0.143461219	0.373802412	0.383788908	0.70113491	0.955435046
BDNF	110.5935572	-0.118459749	0.308810373	-0.383600291	0.701274724	0.955520423
RPS10-NUDT3	61.68594851	-0.129872964	0.338599076	-0.383559713	0.701304804	0.955520423
ARL13A	4.697897602	-0.335206149	0.874238821	-0.383426292	0.701403712	0.955582122
ZNF667-AS1	26.31488544	-0.164805905	0.430215569	-0.383077501	0.701662301	0.955861342
LINC02518	8.943932319	0.414971423	1.083648839	0.382939018	0.70176498	0.955928141
SNORD116-2	19.02696496	-0.184373292	0.481784917	-0.38268797	0.701951135	0.956108631
ZNF165	21.99775871	-0.200906078	0.52518737	-0.382541717	0.702059592	0.956183272
NKX2-8	30.5693003	0.136279557	0.356337789	0.382444863	0.702131419	0.956208015
MIXL1	2.436241139	0.435149266	1.138029071	0.382370957	0.702186229	0.956209583
POLD3	438.7863658	-0.052301329	0.136868923	-0.382127135	0.702367066	0.956298175
DPM3	72.1891169	0.116499083	0.304877537	0.382117632	0.702374114	0.956298175
C2orf66	3.090690817	0.457644947	1.197815914	0.382066177	0.702412279	0.956298175
DBIL5P	16.20633111	-0.2149955	0.562971747	-0.381893944	0.702540033	0.956304095
POLL	270.4626668	0.060326843	0.157968335	0.381891996	0.702541477	0.956304095
RGS4	29.28904144	-0.133034582	0.348401012	-0.38184327	0.702577621	0.956304095
LOC286059	17.2515559	0.241725585	0.633629808	0.381493392	0.702837173	0.956584314
LOC101927066	6.596130882	-0.343731687	0.90164338	-0.381227983	0.703034087	0.956585809
FNTB	36.77358344	-0.118456267	0.310729024	-0.381220476	0.703039656	0.956585809
FAM122A	127.5289339	0.06942364	0.182136512	0.381162673	0.703082545	0.956585809
LINC02470	2.282662841	0.552772768	1.45070118	0.381038339	0.7031748	0.956585809
ZNF37BP	430.9329336	-0.07416481	0.194650547	-0.381015166	0.703191995	0.956585809
LENG9	185.2912209	-0.099129112	0.260234333	-0.380922496	0.70326076	0.956585809
NEBL	26.36137787	0.184879737	0.48548005	0.380818403	0.703338003	0.956585809
FAM221A	10.11160972	-0.221251238	0.581001705	-0.380809964	0.703344265	0.956585809
HLX-AS1	2.045348623	-0.506728893	1.331040983	-0.380701195	0.703424982	0.956585809
IL11RA	159.0717247	-0.083148649	0.218508209	-0.380528721	0.703552981	0.956585809
PLAC8	16.83116519	-0.195992079	0.515067588	-0.380517204	0.703561528	0.956585809
LINC01772	38.84511122	-0.130229893	0.342287675	-0.380469126	0.70359721	0.956585809
SRGAP2-AS1	10.28192086	0.260346081	0.684328832	0.380440029	0.703618806	0.956585809
RNF8	409.4831847	-0.051659236	0.135793413	-0.380425202	0.70362981	0.956585809
TBCE	475.8696135	-0.053192419	0.139830364	-0.380406786	0.703643478	0.956585809
WDR82	2355.556074	-0.039072009	0.102765153	-0.380206789	0.70379192	0.956628195
NLRC3	21.20977621	0.180671518	0.475301433	0.380119869	0.703856438	0.956628195
NDUFB10	199.0076699	-0.07692053	0.202476925	-0.37989776	0.70402131	0.956628195
SIAE	86.7028275	-0.105683567	0.278189659	-0.379897539	0.704021474	0.956628195
PAQR7	296.1330287	0.074603128	0.196397828	0.379857192	0.704051425	0.956628195
ACTR1A	1488.766246	0.053654453	0.141349931	0.379585983	0.704252767	0.956628195
MIR4697HG	1.837292177	0.588546706	1.550848085	0.379499908	0.704316672	0.956628195
GJA1	31936.12739	-0.072351434	0.190673961	-0.379451045	0.704352951	0.956628195
PI4KA	1248.446033	-0.036748915	0.096871252	-0.379358311	0.704421804	0.956628195
KIF26B-AS1	12.18315117	-0.3621394	0.954718307	-0.379315446	0.704453631	0.956628195
EEF2KMT	166.863993	0.099808591	0.263139012	0.379299864	0.7044652	0.956628195
CCN6	4.718020623	0.404648608	1.066853428	0.379291661	0.704471292	0.956628195
ADAM18	3.866033789	-0.544446367	1.435538459	-0.379262822	0.704492705	0.956628195
DCT	7.38200722	-0.254836808	0.672032012	-0.379203376	0.704536845	0.956628195
FOXCUT	34.29793459	-0.166327512	0.438766088	-0.379080143	0.704628351	0.956628195
TNFRSF13C	20.43491079	0.214323775	0.565457238	0.37902738	0.704667532	0.956628195
FLG-AS1	242.6242988	-0.098212838	0.259148321	-0.378983116	0.704700402	0.956628195
LOC101929709	88.0657234	0.09810147	0.25886035	0.378974495	0.704706804	0.956628195
KDELR1	1967.292096	-0.075805586	0.200108536	-0.378822351	0.704819789	0.956628195
GPR150	15.71361011	0.212044285	0.559837198	0.378760621	0.704865632	0.956628195
C1orf109	383.9829839	0.046862307	0.123730499	0.378744989	0.704877241	0.956628195
NFXL1	518.5786678	-0.044394415	0.117214914	-0.378743738	0.704878171	0.956628195
IGFALS	7.576938444	0.337879408	0.892204535	0.378701739	0.704909362	0.956628195
LOC400464	43.44867705	-0.139999038	0.369944109	-0.378432944	0.705108999	0.956736448
DCTPP1	458.3658346	0.073947253	0.195404353	0.378431965	0.705109726	0.956736448
RPE65	12.44114657	-0.230568006	0.609580422	-0.378240505	0.705251938	0.956736448
NOD1	278.86706	-0.062168651	0.164383564	-0.378192623	0.705287505	0.956736448
MIR1296	2.652203674	0.67711724	1.790536256	0.378164495	0.705308399	0.956736448
SLC25A25	202.2343408	-0.057982417	0.153477807	-0.377790234	0.705586429	0.956736448
ZNF451	3210.75661	0.035271392	0.093365818	0.377776286	0.705596792	0.956736448

STARD7-AS1	142.8835765	-0.070805382	0.187536653	-0.377554897	0.705761276	0.956736448
RALGAPB	1828.820153	0.051437663	0.136246897	0.37753273	0.705777746	0.956736448
HARBI1	70.39051013	-0.100616908	0.266534884	-0.37749996	0.705802094	0.956736448
GSTM4	140.5699925	0.114120248	0.302364549	0.377426019	0.705857034	0.956736448
TRIM32	776.4466079	0.052771031	0.139819075	0.377423684	0.705858769	0.956736448
MRPS7	503.8880387	-0.064707252	0.171486955	-0.377330461	0.705928038	0.956736448
CSTF1	522.0207822	0.049300857	0.130664691	0.377308183	0.705944591	0.956736448
MINDY2	817.2084428	-0.052278566	0.138577624	-0.377251135	0.705986983	0.956736448
MIR4785	5.949410747	0.332049817	0.88023829	0.37722719	0.706004775	0.956736448
NFKBIA	208.1739999	-0.109021702	0.289012199	-0.377221799	0.706008782	0.956736448
MFAP5	5.405708753	0.391818558	1.038699912	0.377220171	0.706009991	0.956736448
TNS3	1155.416746	0.048595507	0.128867364	0.377097082	0.70610146	0.956736448
HOXD10	27.85442798	0.185489437	0.492024046	0.376992626	0.706179085	0.956736448
CCDC66	152.3986749	0.130719736	0.346760122	0.376974537	0.706192528	0.956736448
SNED1	54.01765857	0.167584516	0.444585374	0.376945634	0.706214008	0.956736448
ZP1	2.827103996	0.543554732	1.442117765	0.376914248	0.706237333	0.956736448
RPL13AP6	4.695555679	0.437149443	1.160097421	0.376821321	0.706306395	0.956736448
FLJ36000	43.07161715	-0.152084954	0.403635624	-0.376787739	0.706331353	0.956736448
LEMD3	1021.960422	0.045096078	0.119734745	0.376633184	0.706446224	0.956761223
LINC02542	3.289183354	0.421372259	1.119047322	0.376545523	0.706511379	0.956761223
ASPHD2	15.57012552	-0.201337821	0.534775289	-0.37649051	0.70655227	0.956761223
CPSF6	1738.100404	-0.087477165	0.232359	-0.376474183	0.706564405	0.956761223
KLHL10	7.45651952	0.311086211	0.826646288	0.376323243	0.706676602	0.956840441
ACSBG2	27.56382736	0.219304127	0.582964515	0.37618778	0.7067773	0.956904078
TUSC1	321.9873664	0.051218837	0.136333838	0.375686902	0.707149677	0.957058833
MIR4258	9.389505855	-0.28220127	0.751310391	-0.375612095	0.707205297	0.957058833
CMTR2	413.2883128	0.051150369	0.136182778	0.375600859	0.707213652	0.957058833
AKT1S1	379.2372327	-0.051664424	0.137551995	-0.375599236	0.707214859	0.957058833
CMC4	47.49830697	0.147856697	0.393805514	0.375456136	0.707321263	0.957058833
SRPX2	253.5182791	-0.101182536	0.269572937	-0.375343821	0.70740478	0.957058833
ST3GAL6	99.4105504	0.099873325	0.266110662	0.375307493	0.707431795	0.957058833
MRPS28	193.2389433	0.063595663	0.169474481	0.375252149	0.707472949	0.957058833
MED9	312.3368464	0.057714289	0.153824322	0.375196123	0.707514613	0.957058833
NR1D2	884.6002433	0.125746491	0.335293899	0.375033638	0.70763545	0.957058833
PITHD1	760.3036147	0.052364277	0.139625595	0.375033513	0.707635543	0.957058833
ASB8	216.8254853	0.063795421	0.170136538	0.374966025	0.707685734	0.957058833
KCTD3	1547.888528	-0.032840114	0.087585894	-0.374947521	0.707699496	0.957058833
NF2	1620.27314	0.043405398	0.11577189	0.374921738	0.707718672	0.957058833
ZFP69	130.0674495	0.078309167	0.20888977	0.374882727	0.707747685	0.957058833
HOTAIR	59.52077052	-0.105754698	0.282104017	-0.374878386	0.707750915	0.957058833
MAGEA3	1106.362027	-0.045429725	0.121220739	-0.374768583	0.707832582	0.957096639
PKIB	13.34153327	0.202409513	0.540264222	0.374649116	0.70792144	0.957144163
CYP27B1	8.826447087	-0.253566773	0.677524579	-0.374254723	0.708214814	0.957468173
SRSF5	1753.052355	-0.064479024	0.172419343	-0.373966302	0.708429387	0.957569213
GSTP1	888.3254298	-0.058384279	0.156122413	-0.373964748	0.708430544	0.957569213
LDHA	8027.667937	-0.063762322	0.170540285	-0.373884224	0.708490454	0.957569213
AP5Z1	442.317464	-0.066457311	0.177757341	-0.373865355	0.708504493	0.957569213
GABRA4	2.273795629	0.483577516	1.294030997	0.373698557	0.708628599	0.957664313
RSAD1	1045.265091	-0.038221503	0.102301942	-0.373614644	0.708691038	0.957676067
SPATA41	3.531948672	-0.347189569	0.930129794	-0.373270023	0.708947485	0.957741331
MIR541	11.62516375	-0.291563975	0.781152222	-0.373248602	0.708963426	0.957741331
DGKA	139.6782192	0.085085072	0.228008236	0.373166661	0.709024408	0.957741331
SF3B3	5924.960928	0.036838198	0.098727067	0.373131699	0.709050427	0.957741331
NIPA2	862.8609241	-0.067729935	0.181543684	-0.37307789	0.709090474	0.957741331
ZKSCAN4	183.4859705	-0.065809414	0.176423051	-0.373020495	0.70913319	0.957741331
EHHADH	1133.792186	-0.044469392	0.119217898	-0.373009367	0.709141473	0.957741331
NXPH4	32.63412958	0.154151989	0.413369403	0.372915818	0.709211099	0.957741331
MTUS1	268.3966477	0.078869757	0.211524791	0.372862949	0.709250449	0.957741331
THAP7-AS1	28.27112654	-0.150469094	0.403983393	-0.372463565	0.709547735	0.957741331
PITPNA-AS1	7.638454912	-0.250661917	0.673341144	-0.372265856	0.709694918	0.957741331
ALKBH3-AS1	14.45805526	0.285656531	0.76748157	0.372199857	0.709744053	0.957741331
CFAP45	8.149353665	-0.240274935	0.64567018	-0.372132619	0.709794111	0.957741331
TMEM217	5.675648998	-0.310591933	0.83541454	-0.371781814	0.710055305	0.957741331
KLHL29	329.9205354	-0.072466484	0.19496077	-0.371697775	0.710117882	0.957741331
LOC100507547	8.777421245	0.257616354	0.693241164	0.37161145	0.710182163	0.957741331
GPX1	582.6034415	-0.071266704	0.191777883	-0.371610648	0.71018276	0.957741331
ARFIP2	575.1799646	-0.045291096	0.121892997	-0.371564383	0.710217211	0.957741331
GGT7	569.0778487	-0.047355136	0.127461447	-0.37152517	0.710246412	0.957741331
MITF	117.0420678	0.088897317	0.239287439	0.371508498	0.710258827	0.957741331
ANKLE2	2067.802474	0.054109036	0.145662924	0.371467459	0.710289389	0.957741331
SNORD173	19.98791315	-0.243389529	0.655393428	-0.371364006	0.710366431	0.957741331
LOC101928674	1.916561458	-0.611559438	1.647288257	-0.37125223	0.710449675	0.957741331
SPAST	750.6261284	-0.041260557	0.111160442	-0.371180213	0.71050331	0.957741331
PRKRIP1	37.86790009	-0.138334515	0.372689812	-0.371178687	0.710504447	0.957741331
ANGPTL4	8.372389036	-0.280670638	0.756270094	-0.371124866	0.710544531	0.957741331
LOC100289361	10.36618163	-0.2352459	0.63388789	-0.37111594	0.710551179	0.957741331
PFKFB1	5.895648426	-0.301717871	0.813084861	-0.37107796	0.710579467	0.957741331
ASIC5	21.86950603	0.249829512	0.673399956	0.370997221	0.710639602	0.957741331
PRICKLE1	175.2950418	-0.124476554	0.33553484	-0.370979521	0.710652785	0.957741331
LINC02880	2.582840675	0.435780913	1.174759369	0.370953341	0.710672284	0.957741331
USP32	1406.254446	0.065724637	0.17718087	0.370946577	0.710677323	0.957741331
HOXB-AS3	66.4872436	0.168672492	0.455027978	0.37068598	0.710871434	0.957741331
LOC148709	2.531694251	0.45122583	1.217510932	0.37061337	0.710925522	0.957741331
LINC01711	5.337931098	-0.350227584	0.945027614	-0.37060037	0.710935206	0.957741331
ERN2	5.775763901	-0.427024598	1.152308721	-0.370581764	0.710949067	0.957741331
SEMA3D	29960.53097	-0.030756949	0.083007383	-0.370532689	0.710985624	0.957741331
MET	2937.213889	-0.081181577	0.219240213	-0.370285977	0.711169421	0.957741331
COG8	474.0933323	-0.054133631	0.146195027	-0.370283673	0.711171137	0.957741331
LOC101927418	9.92822024	-0.232824962	0.628816787	-0.370258821	0.711189653	0.957741331
CNRIP1	488.4978299	-0.079277595	0.214122181	-0.370244664	0.7112002	0.957741331

LANCL3	11.13413351	-0.272690752	0.736527852	-0.370238208	0.71120501	0.957741331
PPP1R12A	2708.45107	0.043081276	0.116396521	0.370125119	0.711289267	0.957741331
ZNF646	454.2904744	-0.054055847	0.146054408	-0.370107606	0.711302315	0.957741331
VPS37D	12.92416004	-0.217469909	0.587951155	-0.369877509	0.71147376	0.957741331
FOXD3-AS1	9.912742263	-0.23639722	0.63912969	-0.369873633	0.711476648	0.957741331
MIR3174	3.374991485	0.463378928	1.253082372	0.369791275	0.711538017	0.957741331
LINC01414	4.478747472	-0.437048735	1.181946323	-0.369770375	0.711553591	0.957741331
SNORA77	2.658037209	0.518779081	1.403178724	0.369717038	0.711593336	0.957741331
LINC01123	5.381471291	-0.335377335	0.907143928	-0.369706862	0.711600919	0.957741331
CNOT9	1513.621069	0.037582651	0.101669129	0.369656467	0.711638472	0.957741331
GID4	554.0095103	-0.102922085	0.278436113	-0.369643448	0.711648174	0.957741331
CABP1	16.57834066	-0.206854662	0.559635155	-0.369624137	0.711662564	0.957741331
CTC-338M12.4	81.8020755	0.108004725	0.292320148	0.369474103	0.711774372	0.957741331
TM9SF4	1250.666548	0.055806902	0.151065439	0.369422034	0.711813177	0.957741331
ZFX	1857.59873	-0.040311049	0.109132297	-0.369377811	0.711846135	0.957741331
HECA	779.9954446	0.114258054	0.309458336	0.369219506	0.711964118	0.957741331
ANKRD49	121.1831623	-0.087100061	0.235906503	-0.369214328	0.711967977	0.957741331
NUTM2D	16.33194913	-0.195413125	0.529383951	-0.369133073	0.712028538	0.957741331
ZNF883	140.7692275	0.080604714	0.218402524	0.369064937	0.712079323	0.957741331
LAT2	14.22628796	0.19778291	0.535907735	0.369061496	0.712081888	0.957741331
ITGB2	4.637032493	0.325314806	0.881511112	0.369042206	0.712096266	0.957741331
FNDC1	70.18953975	0.146175374	0.396356039	0.368798151	0.712278183	0.957741331
UVRAG	319.2829006	0.060479003	0.164033775	0.368698476	0.712352485	0.957741331
SLC30A3	40.08114204	0.132574248	0.359612777	0.368658337	0.712382407	0.957741331
LINC02082	6.042880249	0.295208889	0.80077867	0.368652288	0.712386916	0.957741331
RAMP2-AS1	3.980221564	0.47095516	1.27779299	0.368569216	0.712448845	0.957741331
FAM83B	4.217733184	-0.334431714	0.907402152	-0.368559534	0.712456063	0.957741331
RASL12	56.6731894	-0.228664021	0.620554216	-0.368483551	0.712512708	0.957741331
SMARCAD1	903.9498615	-0.043745949	0.118769723	-0.368325763	0.712630344	0.957741331
HGS	2100.31829	-0.040056765	0.108757764	-0.368311775	0.712640774	0.957741331
ERO1A	3958.080905	-0.046659728	0.126698743	-0.368273016	0.712669671	0.957741331
DHRS7B	866.4938056	-0.046309464	0.125748686	-0.368269959	0.71267195	0.957741331
RGMB-AS1	9.712441858	-0.222258667	0.603814088	-0.368091225	0.712805214	0.957741331
CA4	2.340484996	-0.547511421	1.487678268	-0.368030798	0.71285027	0.957741331
SNHG28	13.47203241	0.260650536	0.708315559	0.367986462	0.712883329	0.957741331
STRIT1	3.154756957	-0.526042797	1.429674782	-0.367945776	0.712913667	0.957741331
LINC00565	7.546489296	0.289235813	0.786403959	0.367795469	0.713025748	0.957741331
FLJ30679	3.04779299	-0.41471621	1.127808772	-0.367718553	0.713083105	0.957741331
MRPL54	75.63218228	-0.120447681	0.327615975	-0.367648985	0.713134984	0.957741331
RARS2	576.1582901	-0.04382918	0.119218602	-0.367637089	0.713143856	0.957741331
KCNV1	55.04003581	0.206170857	0.560820495	0.367623613	0.713153906	0.957741331
PLPPR3	24.46972798	-0.185401961	0.504410662	-0.367561544	0.713200194	0.957741331
KRBA2	142.2663771	0.095499277	0.259918187	0.367420524	0.713305364	0.957810383
SNORD114-14	23.54508512	-0.24682032	0.672048092	-0.367265859	0.713420718	0.957893097
VRK2	420.523762	-0.053584688	0.145943793	-0.36715976	0.713499853	0.957927174
REEP1	11.12718463	0.259941278	0.7081227	0.36708508	0.713555556	0.957929788
UBE2K	1189.500935	0.037838764	0.10313192	0.366896724	0.713696055	0.95804623
AKAP7	98.47452031	0.084767123	0.231183329	0.366666246	0.713867987	0.958135391
GPR27	3.186622768	0.521192973	1.421447527	0.366663534	0.713870011	0.958135391
MTRF1L	318.2248857	-0.05895176	0.160893986	-0.366401268	0.714065674	0.958268502
MAP3K5-AS1	3.5735622	0.437110127	1.193030304	0.366386441	0.714076736	0.958268502
LOC101927583	11.55243866	0.233208136	0.636734576	0.366256435	0.714173734	0.958326502
TENM1	176.5064561	0.096996601	0.265052844	0.365951936	0.714400941	0.958559203
WDR90	773.018688	0.216626764	0.592244789	0.365772343	0.714534959	0.958582912
NECTIN3	401.0019812	0.067852909	0.185640968	0.365506114	0.714733644	0.958582912
LOC100133331	19.83304188	0.191245424	0.523536745	0.365295131	0.714891113	0.958582912
MYH2	6.257224968	0.284420993	0.778636472	0.365280851	0.714901771	0.958582912
FOSB	240.5950045	-0.59177584	1.620121701	-0.365266288	0.714912641	0.958582912
SNORD114-18	3.362732877	0.413920089	1.133241118	0.365253328	0.714922314	0.958582912
MIR6840	3.655607483	0.376922996	1.032268143	0.365140587	0.715006465	0.958582912
CCNI2	7.509246749	0.294185806	0.805883271	0.365047664	0.715075827	0.958582912
BCL7B	231.0308227	-0.065013033	0.178120189	-0.364995309	0.715114908	0.958582912
CDSN	6.166761947	-0.304162217	0.833336461	-0.36499329	0.715116415	0.958582912
PLEKHM2	1252.807174	0.079765001	0.218621534	0.364854272	0.715220191	0.958582912
TOPBP1	1517.515998	0.040948122	0.112250364	0.364792778	0.715266096	0.958582912
PAIP2B	107.1190488	-0.164797577	0.451851028	-0.364716615	0.715322955	0.958582912
ACTB	41802.77136	0.052743071	0.144622283	0.364695328	0.715338846	0.958582912
PDZD9	5.302569564	-0.452727827	1.241741644	-0.364590999	0.715416735	0.958582912
MIR3657	5.616016023	0.306981761	0.842148133	0.364522284	0.715468037	0.958582912
HSP90AA1	21819.6861	-0.06251926	0.171512149	-0.364517967	0.715471259	0.958582912
C1orf194	10.86826182	-0.267598566	0.734165888	-0.364493325	0.715489657	0.958582912
RRNAD1	336.925337	-0.103206443	0.283170586	-0.364467386	0.715509024	0.958582912
BLOC1S3	214.2075023	0.06889744	0.189048685	0.364442841	0.715527349	0.958582912
C16orf86	71.27593927	0.118028074	0.323919241	0.364375	0.715578001	0.958582912
PPM1D	293.7055888	0.050700718	0.139182759	0.364274417	0.715653102	0.958582912
ARHGEF28	20.57254562	0.180333081	0.495140265	0.364206051	0.715704148	0.958582912
SUPV3L1	358.3594403	-0.048237123	0.132470168	-0.364135742	0.715756648	0.958582912
ATP5MC1	560.5984253	-0.050328061	0.138215807	-0.364126667	0.715763424	0.958582912
PFAS	2603.65916	-0.076433744	0.210065439	-0.363856826	0.715964926	0.958600532
PSMD3	2042.846213	0.039904553	0.10968676	0.363804649	0.716003891	0.958600532
ZNF333	284.336352	0.068373884	0.187956649	0.363774758	0.716026214	0.958600532
PELI3	133.9154159	0.08455447	0.232609302	0.363504253	0.716228237	0.958600532
PDSS1	150.717359	0.122029555	0.335800588	0.363398873	0.716306943	0.958600532
CALD1	7739.299237	0.048058779	0.132250321	0.363392531	0.716311681	0.958600532
RAP1GAP	16.20657228	-0.205327923	0.565050935	-0.363379494	0.716321418	0.958600532
CENPH	383.3893417	-0.063367893	0.174495976	-0.363148163	0.716494208	0.958600532
AMZ2	541.6518212	-0.064783792	0.178397535	-0.363142867	0.716498164	0.958600532
EXOC6	63.95345307	0.099994806	0.275377673	0.363118784	0.716516153	0.958600532
DVL1	2276.985982	0.050579074	0.13929166	0.363116314	0.716517998	0.958600532
EDIL3	6134.137202	-0.048199919	0.132769935	-0.363033386	0.716579945	0.958600532

PEMT	389.0360518	-0.058030079	0.159855993	-0.363014726	0.716593884	0.958600532
SNORD78	31.94949386	0.216804482	0.597268696	0.362993212	0.716609955	0.958600532
TRIM65	1250.929487	0.041947395	0.115561148	0.362988736	0.716613299	0.958600532
LINC02694	10.73072925	-0.259518693	0.715012943	-0.362956637	0.716637277	0.958600532
HVCN1	6.155057112	0.292739111	0.807155354	0.362680008	0.716843935	0.95870431
ZC2HC1A	246.3832932	-0.055893224	0.154136035	-0.362622695	0.716886755	0.95870431
LOC100132215	26.48901222	0.233570771	0.644324173	0.362505057	0.716974645	0.95870431
MIR548S	3.917530312	0.427851409	1.180283795	0.362498757	0.716979352	0.95870431
LOC107985184	2.391883401	-0.454691268	1.254345906	-0.362492727	0.716983858	0.95870431
RFT1	609.9887111	0.049407037	0.136344966	0.362367887	0.717077133	0.958757092
RABGEF1	450.0241138	-0.043334504	0.119658989	-0.362150007	0.717239936	0.958902818
COA6-AS1	4.652847039	0.344494955	0.951549336	0.362035831	0.717325255	0.958904536
KHK	29.8562713	-0.148767884	0.410956161	-0.362004266	0.717348842	0.958904536
MIR377	4.198352173	-0.447399867	1.236163678	-0.361926074	0.717407275	0.958910714
MAP3K14-AS1	20.3538084	-0.167881838	0.464020419	-0.361798384	0.7175027	0.958957492
SNORA40B	3.230608825	0.513027337	1.418239908	0.361735228	0.717549899	0.958957492
UBE2N	1785.997747	-0.047381218	0.13101599	-0.361644542	0.717617675	0.95897615
HACL1	162.5388084	0.060058418	0.166132786	0.361508524	0.717719334	0.958981625
SLURP1	2.10302746	0.431796795	1.194717746	0.361421596	0.717784306	0.958981625
EIF2AK2	1903.274247	0.073914343	0.204517078	0.361409147	0.717793611	0.958981625
HMCN2	2.144170179	0.488189865	1.351019375	0.36134927	0.717838366	0.958981625
RAB8B	1204.573122	0.050949123	0.141032808	0.361257238	0.717907158	0.958981625
NDUFC2	150.6471802	-0.080934162	0.224065832	-0.361207065	0.717944661	0.958981625
GTF3C4	2683.737326	-0.032785452	0.090791644	-0.361106493	0.718019839	0.959010159
RAB11FIP2	428.9550726	0.05344047	0.148050864	0.360960204	0.718129197	0.959084336
FASTKD3	227.0970134	0.088657786	0.245698074	0.360840377	0.718218777	0.959132089
XRCC4	101.7623007	-0.070688892	0.195946487	-0.360756109	0.718281776	0.959144343
PRSS35	45.58550776	0.142861242	0.396321848	0.360467743	0.718497375	0.959261735
CHSY1	2375.078912	-0.05617173	0.155849788	-0.360422243	0.718531395	0.959261735
EPHA3	12165.38594	-0.114042429	0.31643907	-0.360393008	0.718553254	0.959261735
LINC02875	26.50031847	0.16610489	0.460953636	0.360350537	0.718585011	0.959261735
BCL9	496.2332904	-0.045357699	0.125929636	-0.360182881	0.718710376	0.959263823
SPATA33	54.51262286	-0.162850923	0.452204136	-0.36012701	0.718752155	0.959263823
EPHX3	2.336606449	-0.465061397	1.291617315	-0.36006129	0.7188013	0.959263823
GATAD2B	1539.641515	-0.063336822	0.17590606	-0.36006049	0.718801899	0.959263823
METTL2B	595.8997048	0.043860784	0.122037993	0.359402702	0.719293854	0.959419909
ARF4	1968.183182	0.051509268	0.143366667	0.359283429	0.719383069	0.959419909
FAM222B	187.0981773	0.059008214	0.164364167	0.359008995	0.71958836	0.959419909
SMYD2	541.7712167	-0.056349942	0.156960962	-0.359006096	0.719590529	0.959419909
CYB5A	203.6999953	0.058878089	0.164005835	0.358999966	0.719595114	0.959419909
RSPRY1	714.3642716	-0.049482099	0.137834693	-0.35899597	0.719598104	0.959419909
NF1	5310.744297	-0.029191845	0.081318347	-0.358982273	0.719608351	0.959419909
NINJ2-AS1	33.41844087	-0.191183727	0.532644473	-0.358933091	0.719645144	0.959419909
LINC00265	127.1801796	0.083205598	0.231864846	0.358853875	0.719704407	0.959419909
SMG1P1	249.0000617	0.24952822	0.695392269	0.358830879	0.719721611	0.959419909
MPLKIP	600.5464095	-0.043214753	0.120432915	-0.358828427	0.719723445	0.959419909
XKR9	8.510072086	-0.276490473	0.770693296	-0.358755519	0.719777991	0.959419909
ZCCHC24	734.2834251	-0.078645432	0.219227376	-0.358739101	0.719790274	0.959419909
MSANTD3-TMEFF1	3.202497527	0.418747176	1.167285087	0.358735994	0.719792599	0.959419909
FRG1	142.9279964	-0.071839779	0.200269624	-0.358715305	0.719808078	0.959419909
LOC101929243	17.66239745	-0.158691428	0.442398808	-0.358706726	0.719814496	0.959419909
ATL3	2581.816138	-0.098638472	0.275003733	-0.358680485	0.719834129	0.959419909
DDX51	648.1747686	-0.08856671	0.24705927	-0.358483654	0.719981399	0.959544426
ZXDA	76.04676162	-0.092740691	0.258820387	-0.358320656	0.720103362	0.959635201
NUDT5	567.5751896	-0.041055703	0.11461069	-0.358218792	0.720179585	0.959644348
MOB2	113.0061672	-0.102837677	0.287125272	-0.358163098	0.720221261	0.959644348
EDC4	1362.755256	-0.050006454	0.139645548	-0.358095587	0.720271782	0.959644348
NRK	559.5108318	0.058486627	0.163475416	0.35777017	0.720515316	0.959879841
FHDC1	198.6914123	0.12329267	0.344666879	0.357715457	0.720556265	0.959879841
SNHG15	303.0373714	0.088999087	0.248897733	0.357572912	0.720662953	0.959950203
AACS	832.7024755	-0.059797978	0.167312932	-0.357402009	0.720790873	0.959981936
GTF2A2	348.9975714	0.051660871	0.144547523	0.35739714	0.720794518	0.959981936
HLF	3.277773249	-0.51113084	1.4307476	-0.357247386	0.720906614	0.960059478
LOC101927248	2.923000277	0.407637602	1.141477676	0.35711395	0.721006501	0.960120748
DHTKD1	365.5740993	-0.075037991	0.210298698	-0.356816241	0.721229377	0.960165733
PRPF38B	649.5247858	-0.07320791	0.205177824	-0.356802255	0.721239848	0.960165733
GEMIN2	348.0678087	-0.056426609	0.158208283	-0.356660267	0.721346154	0.960165733
MIS18A	206.05925	-0.059354853	0.166450362	-0.356591916	0.72139733	0.960165733
SNHG18	58.19512486	0.140966154	0.395320883	0.356586661	0.721401265	0.960165733
HPDL	10.17760299	0.237674181	0.666619453	0.356536521	0.721438807	0.960165733
ZNF576	141.8871424	0.078242339	0.219490154	0.356473114	0.721486283	0.960165733
TMED1	568.8375214	0.045461096	0.1275602	0.35638934	0.721549011	0.960165733
TSPEAR	3.608667836	0.403797885	1.133038286	0.356385031	0.721552238	0.960165733
CRYZL1	203.8398968	0.069352423	0.194619296	0.356349162	0.721579096	0.960165733
MIR4466	2.10006998	-0.460345626	1.293306446	-0.355944739	0.72188195	0.960371154
CLNK	18.49670597	-0.330465008	0.928599082	-0.355874795	0.721934332	0.960371154
CACNA2D1	496.6514282	-0.073418328	0.206309039	-0.355865785	0.72194108	0.960371154
INSR	4.120532696	-0.405387984	1.139193807	-0.35585515	0.721949045	0.960371154
CCDC130	244.8375091	0.055026031	0.154681259	0.355738186	0.722036645	0.96041599
SLC43A2	85.66203669	0.13363274	0.375765017	0.355628475	0.722118816	0.960453597
ZNF776	388.1886869	0.048591873	0.136684083	0.355504989	0.722211308	0.960504927
LYST	1310.409598	0.061302353	0.172538615	0.355296424	0.722367535	0.96051178
STX18-IT1	2.637128116	0.651147497	1.83357422	0.355124701	0.722496172	0.96051178
RASSF9	56.06670822	-0.094030569	0.264855774	-0.355025558	0.722570445	0.96051178
DNAAF2	391.4310604	0.059435848	0.167434063	0.354980625	0.722604107	0.96051178
PAPOLA	5176.691162	0.060338415	0.170025364	0.354878907	0.722680311	0.96051178
CKMT2	26.92140185	0.219933201	0.619748859	0.354874716	0.722683451	0.96051178
MESP2	8.097011955	0.27293546	0.7691096	0.354871997	0.722685488	0.96051178
IFI6	242.1219595	-0.1194991	0.336793093	-0.354814579	0.722728505	0.96051178
NKILA	108.682324	0.165201794	0.46568073	0.35475334	0.722774387	0.96051178

XYLB	291.4860455	0.102903205	0.290076757	0.354744745	0.722780827	0.96051178
TTC4	26.51953079	0.13961736	0.393626652	0.354694886	0.722818183	0.96051178
ZNF32	110.5578661	0.087707291	0.247317278	0.354634708	0.722863271	0.96051178
PPP1R11	557.8137684	-0.053201773	0.150136509	-0.354356	0.723072105	0.960606659
MED7	147.8995529	0.078391936	0.221259432	0.354298729	0.72311502	0.960606659
DDX21	3763.831417	-0.037906148	0.107011488	-0.354225037	0.723170242	0.960606659
MIR661	15.25126293	0.235626981	0.665251853	0.354192145	0.72319489	0.960606659
LINC01399	22.18078223	0.186734275	0.527230293	0.354179715	0.723204205	0.960606659
SNORA55	14.97693144	-0.206536574	0.583868687	-0.353738056	0.7235352	0.9608666
KREMEN2	13.79619517	-0.249049664	0.704110469	-0.353708224	0.723557559	0.9608666
INVS	741.7609055	-0.03799079	0.107408809	-0.353702743	0.723561667	0.9608666
CCDC112	44.79106997	-0.109426798	0.309857993	-0.353151446	0.723974906	0.961343728
RUNX2	10231.62835	0.087913501	0.249144415	0.352861616	0.724192188	0.961530885
SPOUT1	642.0291654	0.067344192	0.190874352	0.352819495	0.724223768	0.961530885
PPIEL	16.31080395	-0.228807826	0.648904633	-0.352606244	0.724383656	0.961671515
KLC1	1429.846218	0.054196997	0.153782807	0.352425592	0.724519112	0.961779691
FBXO46	397.7667753	0.070215576	0.199358995	0.352206712	0.724683244	0.961925915
ACBD3	1500.928485	0.074876768	0.212712161	0.352009812	0.724830904	0.961959581
TEX52	14.77591336	0.204296682	0.580387835	0.352000283	0.72483805	0.961959581
MYLK-AS1	10.63063448	0.267020609	0.758963997	0.351822498	0.724971385	0.961959581
SMCR5	16.35972002	0.321426943	0.913617047	0.351818023	0.724974742	0.961959581
TPP1	1142.101375	0.074672781	0.212411041	0.351548493	0.725176899	0.961959581
UNC93B1	226.428415	-0.058659485	0.166935877	-0.351389324	0.725296291	0.961959581
FTCDNL1	39.56240057	-0.114555572	0.326007966	-0.351388874	0.725296628	0.961959581
LINC00852	34.45881041	0.179316143	0.510330849	0.351372337	0.725309033	0.961959581
PARP6	623.7417526	-0.077336441	0.220191519	-0.351223524	0.725420664	0.961959581
TXK	5.732824688	0.279254492	0.795153808	0.351195567	0.725441636	0.961959581
TMSB10	2292.950225	-0.079475643	0.226313922	-0.351174343	0.725457557	0.961959581
VRK1	679.1830188	0.046066814	0.131188555	0.351149639	0.725476089	0.961959581
STEAP3-AS1	8.040039428	0.321078678	0.914447063	0.351117841	0.725499944	0.961959581
SRF	1157.583204	0.041423794	0.117979654	0.351109637	0.725506099	0.961959581
ACOT9	531.7845727	-0.04937173	0.140622812	-0.351093323	0.725518337	0.961959581
CDK16	1879.605415	-0.031912169	0.09094498	-0.350895336	0.72566687	0.961960403
AAAS	721.6904448	0.052289337	0.149038908	0.350843534	0.725705735	0.961960403
GRPEL1	409.3940573	0.050737202	0.144635149	0.350794409	0.725742592	0.961960403
MBP	4.349069675	0.358433321	1.021872643	0.350761245	0.725767474	0.961960403
TPH1	7.136989207	0.439707223	1.253798381	0.350700104	0.725813347	0.961960403
AMDHD2	203.5552142	-0.109942554	0.313785879	-0.350374448	0.7260577	0.961960403
MAP2	19.92601471	-0.180939171	0.516428355	-0.350366453	0.7260637	0.961960403
POMT1	515.1792775	-0.041602022	0.118741423	-0.350358123	0.72606995	0.961960403
REP15	9.406739301	-0.234357267	0.668935262	-0.350343718	0.726080759	0.961960403
COLQ	8.64378895	0.232813469	0.664543213	0.350336087	0.726086486	0.961960403
SNHG31	9.538472272	-0.226644025	0.647176553	-0.35020432	0.726185365	0.961960403
WBP2	387.654157	0.086957321	0.248317203	0.350186455	0.726198772	0.961960403
KCTD7	129.3781042	-0.105004196	0.299888668	-0.350143929	0.726230685	0.961960403
TIGD4	9.314141228	-0.213607557	0.610240606	-0.350038254	0.726309989	0.961960403
FAM20C	717.6689186	-0.093698673	0.267700292	-0.350013338	0.726328688	0.961960403
USP39	871.664307	0.047341257	0.135290591	0.349922761	0.726396665	0.961977656
ACHE	2.781472716	0.453587851	1.296513079	0.349852121	0.726449681	0.961977656
CZIB	377.5477946	-0.05628216	0.160928179	-0.349734646	0.72653785	0.962022922
WWC3	930.9969065	-0.050324645	0.143953661	-0.349589199	0.726647018	0.962083351
GAPDHS	122.599348	0.125204794	0.358251328	0.349488709	0.726722446	0.962083351
BTAF1	1772.21836	-0.057474055	0.164466246	-0.349458058	0.726745454	0.962083351
LCLAT1	752.2346752	0.044223129	0.126585492	0.349353851	0.726823675	0.962115428
ID4	229.2137403	0.088558113	0.253641973	0.34914613	0.726979606	0.962214245
CENPT	604.8222128	0.088856763	0.254565226	0.349053029	0.727049499	0.962214245
CCT6A	2904.432917	-0.043106221	0.12355111	-0.348893836	0.727169013	0.962214245
BMPR1A	584.5097861	0.042060945	0.12057612	0.348833128	0.727214591	0.962214245
LRRIQ3	15.19902981	-0.182239057	0.522517801	-0.348771002	0.727261235	0.962214245
TMEM184B	761.4447132	0.066698118	0.191253569	0.348741824	0.727283142	0.962214245
IMP3	459.0498058	0.048707494	0.139701908	0.348653032	0.727349809	0.962214245
LYPLAL1-DT	19.54923179	-0.207540448	0.595472431	-0.348530742	0.72744163	0.962214245
RIN3	297.0216059	-0.050421837	0.144688591	-0.348485233	0.727475802	0.962214245
TYRP1	11.33283617	0.22655226	0.650129443	0.348472542	0.727485331	0.962214245
CPAMD8	2.029221384	-0.456158663	1.309058048	-0.348463281	0.727492285	0.962214245
PRR19	5.760922348	0.262274536	0.753368143	0.348135952	0.727738085	0.962445227
SAT1	314.3451926	0.134964257	0.387731531	0.348086876	0.72777494	0.962445227
GAREM2	332.5704907	-0.0543045	0.156056394	-0.347979975	0.727855223	0.962479969
NPIPB4	60.30900136	0.180965178	0.520383865	0.347753246	0.728025505	0.962504987
SRP14-AS1	107.8291896	0.113179511	0.325487724	0.347722824	0.728048354	0.962504987
PWWP2A	482.9006275	0.060979535	0.175386911	0.347685778	0.728076178	0.962504987
LRP11	1489.038457	0.084305918	0.242490339	0.34766712	0.728090193	0.962504987
ZER1	717.9477351	-0.104588301	0.30100516	-0.34746348	0.72824315	0.962592138
SIRT6	177.4387223	-0.063138337	0.18174299	-0.347404522	0.728287436	0.962592138
DHPS	706.4078355	-0.052653624	0.151580716	-0.347363607	0.72831817	0.962592138
TMCO3	1099.650684	-0.066641492	0.191992346	-0.347104944	0.728512479	0.962626594
GALE	464.6569232	0.044232944	0.127434839	0.347102445	0.728514357	0.962626594
SPTBN4	5.296288883	-0.268077322	0.772491054	-0.347029678	0.728569023	0.962626594
TBXA2R	14.89849251	0.198294277	0.571448409	0.347002937	0.728589112	0.962626594
KIFC3	219.7590152	0.059014666	0.170152888	0.346833172	0.728716655	0.962626594
PGR-AS1	4.1649645	-0.359288395	1.036041691	-0.346789514	0.728749455	0.962626594
ATL1	49.48847555	0.132260433	0.381418672	0.346759198	0.728772232	0.962626594
NUTF2	485.5636949	-0.043237192	0.124722148	-0.346668115	0.728840667	0.962626594
ZFP36	28.96934661	0.16761377	0.483535936	0.346641806	0.728860434	0.962626594
SNORD88A	10.59210267	-0.236955685	0.683775137	-0.346540364	0.728936655	0.962626594
COPS7A	620.7691054	-0.037534047	0.108312905	-0.346533467	0.728941837	0.962626594
RNU5E-1	35.87383494	-0.201879554	0.582682022	-0.346466077	0.728992474	0.962626594
FAM230D	5.541747533	0.30226584	0.872975917	0.346247627	0.729156624	0.962693594
CTBS	435.9500257	0.080185681	0.231586864	0.346244512	0.729158965	0.962693594
CLK1	908.4899529	0.12726624	0.367734724	0.34608165	0.729281353	0.962693594

EFNB2	840.8161676	0.09032666	0.261048026	0.346015489	0.729331074	0.962693594
PSMD10	460.8894974	0.054488712	0.157481261	0.346001245	0.729341779	0.962693594
LINC00672	9.60759803	0.263543763	0.761856223	0.345923227	0.729400412	0.962693594
FHL3	470.8677969	0.059563581	0.172224994	0.345847486	0.729457336	0.962693594
TTC31	652.0443153	0.050766541	0.146799012	0.345823453	0.729475399	0.962693594
NIT2	532.2554346	0.057727803	0.166993593	0.345688728	0.729576656	0.962755925
ACBD4	97.21836074	-0.090619814	0.262354156	-0.345410247	0.729785975	0.962878149
LOC101927789	56.77520156	0.112323929	0.325258713	0.345337187	0.729840893	0.962878149
FUBP1	1651.30805	0.086631007	0.250962563	0.34519494	0.729947823	0.962878149
LIPH	4.022383939	0.37111121	1.075125003	0.345179592	0.72995936	0.962878149
CCDC107	42.57303392	0.102749291	0.297674888	0.345172855	0.729964424	0.962878149
PSORS1C1	2.100400669	0.476476058	1.380552983	0.345134206	0.729993479	0.962878149
ZSWIM6	475.3350972	0.047132383	0.136639666	0.34493924	0.73014005	0.963000199
FRMPD3	25.13667958	-0.139249357	0.40384006	-0.344813134	0.730234859	0.96301737
RFC5	1005.054939	0.041940657	0.121645341	0.344778162	0.730261152	0.96301737
PPFIA1	488.2717413	0.039116136	0.113482062	0.344690035	0.730327411	0.963033481
ANGPTL6	4.264712531	-0.329193967	0.955823791	-0.344408635	0.730538996	0.963153272
FSD2	114.104197	0.262827483	0.763381267	0.34429386	0.730625301	0.963153272
XPNPEP3	897.1192276	0.074836174	0.217428728	0.344187148	0.730705547	0.963153272
COG2	569.3540832	0.042056692	0.122218042	0.344111973	0.730762079	0.963153272
INTS2	964.5239438	0.048734152	0.141631121	0.344092117	0.730777011	0.963153272
ZNF516	955.2571442	0.034504456	0.100284099	0.344067072	0.730795845	0.963153272
MVB12B	19.85218127	-0.165807221	0.481953803	-0.344031357	0.730822704	0.963153272
TMCO1-AS1	31.36102791	0.184254499	0.535871559	0.343840788	0.730966024	0.963153272
KCNJ15	86.8931284	-0.338548694	0.984796303	-0.343775351	0.731015239	0.963153272
MIAT	14.43573068	0.2335869	0.679513881	0.343755892	0.731029874	0.963153272
C1orf174	322.8969529	0.049478222	0.143954995	0.343706182	0.731067261	0.963153272
RNF43	52.64761856	0.188508228	0.548501824	0.343678398	0.731088159	0.963153272
LINC01590	6.795483921	-0.281967813	0.820621971	-0.343602564	0.731145197	0.963153272
LPXN	37.15209419	-0.165785081	0.482546354	-0.343563017	0.731174942	0.963153272
ZNF561-AS1	95.64003429	-0.071489336	0.208138142	-0.343470615	0.731244443	0.963173625
PIPOX	3.476395116	-0.397817437	1.15897168	-0.343250351	0.731410128	0.963320656
MEGF6	9.316771203	0.423083206	1.233184236	0.343081912	0.731536838	0.963374576
RAG2	2.180656116	0.435454196	1.269381694	0.343044332	0.731565109	0.963374576
TRAF3IP2	319.6952815	0.073228448	0.2135296	0.342942844	0.731641459	0.963374576
LOC100506444	24.39289236	0.236841584	0.690743541	0.342879187	0.73168935	0.963374576
G3BP1	7230.235347	0.032107603	0.093663515	0.342797337	0.731750929	0.963374576
ZNF41	234.3591253	0.061139102	0.178370439	0.342764765	0.731775435	0.963374576
PDXK	1237.108393	-0.046427533	0.135515795	-0.342598684	0.731900392	0.96345158
TECR	384.6151073	0.077293732	0.225646597	0.342543307	0.731942059	0.96345158
ZNF449	158.0690453	-0.063610474	0.185900107	-0.342175565	0.732218773	0.963669161
MVK	474.0039679	0.065468441	0.191386061	0.342075284	0.732294237	0.963669161
DRC3	58.17483873	-0.118762036	0.347198962	-0.342057578	0.732307562	0.963669161
PPIC	777.0812218	0.066530089	0.194631016	0.341826758	0.732481271	0.963669161
NAP1L2	48.54502239	0.128732184	0.376630009	0.341800126	0.732501315	0.963669161
RAPGEF4	24.33638563	0.2231367	0.652962157	0.341729911	0.73255416	0.963669161
MLLT10	888.0506215	0.048885063	0.143060934	0.341707984	0.732570663	0.963669161
MIR425	1.986407734	0.473160505	1.384736055	0.341697253	0.73257874	0.963669161
CPNE3	2035.028535	0.048501933	0.141952626	0.341676899	0.732594059	0.963669161
PAAF1	366.8700777	0.052506804	0.153825853	0.341339268	0.732848189	0.963932295
ASXL3	122.220534	0.101239628	0.29698304	0.340893636	0.733183656	0.964253898
MGC16275	17.94342025	0.158097238	0.463867291	0.340824286	0.733235866	0.964253898
PCDHAC1	5.202467546	-0.300360538	0.881342617	-0.340798836	0.733255026	0.964253898
TBCCD1	854.1672802	0.034234666	0.100558722	0.340444526	0.733521792	0.964289803
ARL2BP	810.2942359	0.06884117	0.202228907	0.340412113	0.733546198	0.964289803
TM4SF20	2.018753465	0.621582564	1.826128981	0.340382619	0.733568407	0.964289803
PPP4R1L	10.75849057	0.234337049	0.688583259	0.340317668	0.733617313	0.964289803
GOLGA6B	2.123140684	0.473029314	1.389984449	0.340312666	0.733621079	0.964289803
CA13	161.5734399	-0.128789202	0.378532943	-0.340232481	0.73368146	0.964289803
LINC02344	11.15626785	-0.313500318	0.921710427	-0.340128862	0.733759488	0.964289803
CRHR2	3.901498172	-0.346942259	1.020045164	-0.34012441	0.73376284	0.964289803
FOXN3-AS1	16.93423322	0.14598692	0.429411111	0.339970057	0.733879077	0.964289803
INO80D	455.8334098	-0.049303854	0.145078253	-0.339843175	0.733974632	0.964289803
SLC25A41	3.176872434	-0.359486405	1.057909402	-0.339808309	0.73400089	0.964289803
PARD3B	23.09994121	-0.17703025	0.521005532	-0.339785739	0.734017888	0.964289803
ATP6V1G2-DDX39B	23.53071579	0.19704362	0.580046232	0.339703302	0.734079975	0.964289803
ACOT8	170.0710126	-0.07754888	0.228310447	-0.339664178	0.734109442	0.964289803
HSD17B8	31.53265779	0.146818053	0.432250743	0.339659458	0.734112996	0.964289803
SLC35D2	388.8240937	-0.078141511	0.230090001	-0.339612804	0.734148134	0.964289803
KCNJ2-AS1	4.39934082	0.350802319	1.033271791	0.339506336	0.734228325	0.964324053
TEX30	164.1845327	-0.071123053	0.209583391	-0.339354432	0.734342742	0.964403247
TCEAL9	218.8215078	-0.064778663	0.19094199	-0.339258343	0.734415121	0.964427226
GFM1	1136.28335	0.064331726	0.189806887	0.33893252	0.734660566	0.964514856
ASB14	22.88628773	0.195519218	0.576989626	0.338860889	0.734714529	0.964514856
FIGN	372.1933577	-0.105889268	0.312598902	-0.33873845	0.734806773	0.964514856
DCTN5	1832.676558	-0.03438498	0.101528727	-0.338672426	0.734856515	0.964514856
UQCRQ	181.1504123	-0.076856515	0.227064691	-0.338478494	0.735002631	0.964514856
SPTY2D1	988.9486844	-0.052158259	0.154112982	-0.338441695	0.735030358	0.964514856
THORLNC	3.539437716	-0.489216393	1.445894243	-0.338348669	0.735100451	0.964514856
ZNF501	31.79315996	-0.145084644	0.428849141	-0.338311612	0.735128374	0.964514856
FTO	3875.709146	0.044740807	0.132269873	0.338253953	0.735171821	0.964514856
ZNF236-DT	11.79393327	-0.232670418	0.688093612	-0.338137739	0.735259392	0.964514856
MBNL1	7449.119082	-0.059668975	0.176508545	-0.338051478	0.735324395	0.964514856
ENOPH1	434.9472186	0.050054138	0.148132341	0.337901482	0.735437431	0.964514856
HAPLN3	70.9444399	-0.129860728	0.384316941	-0.337900088	0.735438481	0.964514856
DRAP1	83.87807327	-0.104714127	0.309906812	-0.337889077	0.735446779	0.964514856
SCAMP3	532.5006379	0.047307976	0.140021079	0.337863245	0.735466247	0.964514856
DHX16	769.304891	-0.033743002	0.099924038	-0.337686529	0.735599428	0.964514856
COL1A2	76762.45969	0.045734968	0.135442118	0.337671685	0.735610615	0.964514856
TTC13	369.7626073	-0.049332813	0.146109655	-0.337642391	0.735632693	0.964514856

TRIT1	427.2703752	-0.072013394	0.213332167	-0.33756463	0.7356913	0.964514856
AKAP1	819.5031971	0.054800243	0.162341875	0.337560736	0.735694235	0.964514856
SNORD114-4	7.218764694	-0.246986918	0.731811959	-0.337500522	0.735739619	0.964514856
LINC01014	10.80216368	0.25097187	0.743624817	0.337497976	0.735741538	0.964514856
RAB1B	964.4683385	-0.067329925	0.199498476	-0.337495938	0.735743074	0.964514856
KCNG1	340.5745872	-0.053323352	0.158052201	-0.337378107	0.735831886	0.964514856
PDZD11	130.5661491	-0.062751638	0.186102854	-0.337187942	0.735975226	0.964514856
DNM1P35	22.38014363	0.178849108	0.530545633	0.3371041	0.736038427	0.964514856
TIGD5	925.2636407	-0.041776687	0.123935294	-0.337084662	0.736053079	0.964514856
HERC4	961.5356044	0.044556092	0.132207919	0.337015301	0.736105366	0.964514856
MIR1302-3	2.592506752	0.381320798	1.131468264	0.337014135	0.736106245	0.964514856
MIR329-2	3.793813489	0.460299226	1.3660353	0.336959979	0.73614707	0.964514856
NRAV	112.139305	-0.102195248	0.303413354	-0.336818558	0.736253683	0.964514856
CHAMP1	516.9144207	-0.046453702	0.138018399	-0.336576152	0.736436436	0.964514856
HHEX	61.64099369	0.095920085	0.285118012	0.336422396	0.736552363	0.964514856
ZNF25	139.2065246	0.097784513	0.2906701	0.336410635	0.736561231	0.964514856
TEX12	2.078874135	-0.532684829	1.583665799	-0.336361895	0.736597981	0.964514856
LINC02210	231.6673059	0.070384098	0.209270631	0.336330511	0.736621644	0.964514856
ZNF655	552.3273204	-0.046997008	0.139745962	-0.336303158	0.736642269	0.964514856
LRRC4B	4.707041281	0.288308477	0.857384693	0.336265016	0.736671029	0.964514856
IQCH-AS1	68.63668128	0.086716639	0.257927769	0.336205128	0.736716186	0.964514856
ZNF502	44.28424132	0.114933075	0.341862583	0.336196708	0.736722535	0.964514856
PATE1	3.232844818	0.678883095	2.019497852	0.336164306	0.736746968	0.964514856
FARS2	296.8715402	-0.073874294	0.219763575	-0.336153496	0.736755119	0.964514856
ASNSD1	556.5576994	-0.039597893	0.117836125	-0.336042051	0.736839156	0.964554012
E2F3	1312.741742	0.044015587	0.131062903	0.335835588	0.736994852	0.964563948
LRFN4	540.649225	0.057948823	0.172601026	0.33573858	0.73706801	0.964563948
THAP9	93.74885689	0.067352121	0.200609331	0.335737727	0.737068653	0.964563948
TMEM17	59.95143224	-0.092040598	0.274315608	-0.335528112	0.737226742	0.964563948
NAA40	396.1543535	0.076251531	0.227457505	0.335234186	0.737448436	0.964563948
LOC100289561	12.77674964	0.235865036	0.703776875	0.335141782	0.737518136	0.964563948
CENPS	15.79010634	-0.168304726	0.502195698	-0.33513773	0.737521192	0.964563948
RNU4ATAC	2.528814913	-0.375470767	1.120365778	-0.335132306	0.737525284	0.964563948
FGL2	3.569352145	-0.385257517	1.150055188	-0.33499046	0.737632283	0.964563948
PLPP7	203.2610462	-0.078514665	0.234513835	-0.334797581	0.737777785	0.964563948
HM13	1516.433639	-0.036823982	0.110017265	-0.334710935	0.737843152	0.964563948
LINC00944	4.399790401	-0.325712036	0.973197953	-0.334682204	0.737864827	0.964563948
ANKHD1-EIF4EBP3	26.02259245	0.146091308	0.437196615	0.33415471	0.738262818	0.964563948
HSP90AB1	36170.68054	-0.036497433	0.109235382	-0.334117316	0.738291034	0.964563948
POU3F4	66.28380314	0.135517509	0.405638503	0.334084433	0.738315847	0.964563948
NXN	1848.703361	-0.057586749	0.172373167	-0.334081861	0.738317787	0.964563948
WDR78	26.52161755	-0.135254258	0.404919743	-0.334027322	0.738358941	0.964563948
CRCP	452.1243734	0.05644333	0.169014737	0.33395508	0.738413456	0.964563948
PMP22	387.9372393	-0.060520568	0.181284497	-0.33384304	0.738498004	0.964563948
ZNF484	323.0033977	0.059060236	0.176950809	0.333766411	0.738555832	0.964563948
MAK16	2120.526192	-0.057104182	0.171149939	-0.333650028	0.738643663	0.964563948
LOC100130370	2.971177394	0.459784658	1.378222139	0.333607076	0.738676078	0.964563948
LRRC28	215.2326414	-0.066179113	0.198395048	-0.333572403	0.738702246	0.964563948
TPR	3160.649417	0.05892314	0.176700149	0.33346401	0.738784053	0.964563948
CHML	884.5660117	-0.085039179	0.255134413	-0.333311284	0.738899323	0.964563948
CXADRP3	7.379841561	0.309742042	0.92977431	0.333136804	0.739031019	0.964563948
SOX9	593.8008253	0.061350571	0.184189129	0.333084647	0.739070388	0.964563948
ZNF132	222.9484773	0.071459989	0.214548712	0.333071162	0.739080567	0.964563948
LOC100652758	57.92934316	-0.087004701	0.261243702	-0.333040379	0.739103803	0.964563948
NETO2	892.6097446	0.047235615	0.141877633	0.332932078	0.739185555	0.964563948
MST1L	6.098199597	-0.291344395	0.875678006	-0.33270722	0.739355299	0.964563948
MRPL55	215.4917101	0.056050449	0.168468619	0.332705577	0.739356539	0.964563948
OVOL3	18.06794071	0.18231992	0.548016108	0.332690805	0.739367691	0.964563948
PMS2P3	68.4536431	-0.096599081	0.290404135	-0.332636726	0.739408517	0.964563948
HPS6	298.5461484	-0.046990879	0.141294884	-0.332573107	0.739456546	0.964563948
WASHC1	82.77861175	-0.080273723	0.241392782	-0.332544006	0.739478517	0.964563948
TIFA	295.2633943	-0.067023593	0.201664386	-0.332352153	0.739623363	0.964563948
TTC21B-AS1	10.04670221	-0.285090474	0.857948934	-0.332293057	0.739667982	0.964563948
SOCS6	863.0262089	-0.040148348	0.12086657	-0.332170827	0.739760271	0.964563948
FAM201A	3.655377703	0.393104831	1.183487173	0.332158083	0.739769893	0.964563948
LOC101926934	2.29883855	0.574418965	1.729380485	0.332153028	0.73977371	0.964563948
TXN2	565.2430945	-0.05289779	0.159258004	-0.332151533	0.739774839	0.964563948
PRSS48	6.602257226	0.469881715	1.414716319	0.332138471	0.739784702	0.964563948
LOC100507053	26.18727019	-0.129996301	0.391405745	-0.332126707	0.739793584	0.964563948
MIR503	2.790232931	-0.362671367	1.092057732	-0.332099079	0.739814446	0.964563948
TPRN	296.0484772	0.057978087	0.174598237	0.332065706	0.739839645	0.964563948
PEX26	1271.487067	0.03546073	0.106832977	0.331926816	0.739944521	0.964563948
ZNF436	329.0643404	0.069068834	0.208092187	0.331914597	0.739953748	0.964563948
SMCO2	3.468088191	-0.357123206	1.076189639	-0.331840405	0.740009772	0.964563948
SSR4P1	32.57881822	0.13027848	0.392632936	0.331807314	0.740034761	0.964563948
TM4SF19-AS1	36.03476985	0.121506083	0.366227061	0.331778002	0.740056896	0.964563948
TLR1	38.22544315	0.152703399	0.460305675	0.331743465	0.740082977	0.964563948
NEK8	103.280606	0.091283657	0.275177946	0.331725918	0.740096228	0.964563948
GALNT6	163.6026379	-0.123524519	0.372444524	-0.331658841	0.740146883	0.964563948
DELE1	605.4239004	0.043981222	0.132618017	0.331638366	0.740162345	0.964563948
LYNX1	10.54747475	-0.191670016	0.577989963	-0.331614783	0.740180155	0.964563948
DDX11-AS1	28.89769893	0.121178701	0.365488199	0.33155298	0.740226829	0.964563948
LOC112268068	2.626856522	-0.412804829	1.245232287	-0.331508293	0.740260578	0.964563948
ARMCX2	814.987263	0.033893047	0.102267859	0.331414459	0.740331444	0.964563948
LOC100507250	4.084807636	-0.32092405	0.96867773	-0.331301154	0.740417019	0.964563948
SWI5	192.1398125	0.080993674	0.244482675	0.331285944	0.740428508	0.964563948
SCNN1A	43.4240437	0.195232088	0.589340471	0.331272156	0.740438922	0.964563948
OCA2	3.841495525	-0.429074124	1.295232155	-0.331271982	0.740439052	0.964563948
ARHGEF26-AS1	4.327310789	0.309154147	0.933365106	0.33122531	0.740474303	0.964563948
SPR	347.0404191	0.054388713	0.164333134	0.330966202	0.740670015	0.964563948

TMEM262	4.059492037	-0.420162773	1.269558944	-0.330951765	0.74068092	0.964563948
C1QTNF2	3.593151659	0.352599103	1.06572193	0.330854694	0.740754246	0.964563948
CPLANE1	967.7459046	0.049353878	0.149172284	0.330851528	0.740756637	0.964563948
LOC728095	26.26723319	0.612382501	1.850978959	0.330842497	0.740763459	0.964563948
VIRMA	3508.585481	-0.029275491	0.088493577	-0.330820525	0.740780056	0.964563948
MIR496	4.59590143	0.487913591	1.475158031	0.330753438	0.740830734	0.964563948
CYLC2	6.273073053	0.386669678	1.169187835	0.330716474	0.740858657	0.964563948
PWAR6	180.7680289	0.157608153	0.47664447	0.330661872	0.740899905	0.964563948
TTK	1083.074163	0.036742156	0.111147306	0.330571717	0.740968013	0.964563948
STAC	10.91362407	0.247404843	0.748560443	0.330507503	0.741016524	0.964563948
ELMO2	384.4436591	-0.054410709	0.164703469	-0.330355576	0.741131304	0.964563948
BCAR1	811.8045186	-0.047130419	0.142754813	-0.330149421	0.741287062	0.964563948
DGCR8	930.8559717	0.095431955	0.289100214	0.33009991	0.741324471	0.964563948
LINC02268	5.951048632	0.266053791	0.80601107	0.330087019	0.741334212	0.964563948
LMO7-AS1	6.126564769	0.363019091	1.1000428	0.330004515	0.74139655	0.964563948
COMMD9	219.9303263	-0.069933636	0.211945102	-0.329961087	0.741429365	0.964563948
CCDC18	239.520456	0.100851453	0.305755514	0.329843449	0.741518255	0.964563948
SNORA58B	7.140234995	-0.257646838	0.78112874	-0.329839148	0.741521505	0.964563948
PCDHGA3	4.837605695	-0.298256676	0.904544436	-0.32973137	0.741602948	0.964563948
NGDN	268.6959765	0.052579338	0.159486465	0.329678997	0.741642525	0.964563948
RHOG	217.5300189	-0.052481495	0.159202317	-0.329652836	0.741662295	0.964563948
IFI27L1	118.7646677	-0.090163256	0.273597938	-0.329546548	0.741742617	0.964563948
LLPH-DT	16.74184925	-0.164154951	0.498295921	-0.329432661	0.741828684	0.964563948
MEIG1	2.280877178	0.409049976	1.241831198	0.329392575	0.741858979	0.964563948
MRTFB	749.5532366	-0.036771169	0.111726246	-0.329118451	0.742066158	0.964563948
ZNF626	26.84737004	0.188497665	0.572869083	0.329041435	0.742124369	0.964563948
ERI2	275.4298796	-0.060805873	0.184825476	-0.328990758	0.742162674	0.964563948
PCLO	129.8478582	0.069023084	0.209813061	0.328974202	0.742175188	0.964563948
LRMDA	24.90391883	-0.160062738	0.486565289	-0.328964563	0.742182473	0.964563948
CFAP36	144.3054838	-0.075218299	0.228673474	-0.328933207	0.742206174	0.964563948
FAR1	1745.627627	-0.061574774	0.187226425	-0.328878648	0.742247414	0.964563948
CDC37L1	196.2506283	-0.059922953	0.182228628	-0.328833916	0.742281226	0.964563948
SKA3	259.0861163	-0.068204986	0.207426203	-0.328815669	0.742295019	0.964563948
CCNYL6	12.96787695	0.255314105	0.776736823	0.328700916	0.742381762	0.964563948
LOC100134368	19.9761248	-0.166896056	0.507918339	-0.328588364	0.742466844	0.964563948
ABCB1	13.13742934	-0.177144982	0.539331795	-0.328452696	0.742569405	0.964563948
SHQ1	375.457592	0.048490357	0.147699201	0.328304802	0.742681213	0.964563948
NLRC4	14.54651354	-0.194507705	0.592513471	-0.328275583	0.742703304	0.964563948
MIR181B2	2.297160176	0.450323682	1.372618956	0.328076252	0.742854009	0.964563948
ZNF248	229.3240041	0.083332323	0.254011811	0.328064757	0.7428627	0.964563948
RAB38	3.937605452	-0.303255851	0.924406532	-0.328054639	0.742870351	0.964563948
LOC101926915	3.20472432	0.44731839	1.364080374	0.327926711	0.742967077	0.964563948
UNC13B	388.2190234	0.054102289	0.164997475	0.32789768	0.742989028	0.964563948
FCF1P2	45.01167109	0.106090866	0.323590164	0.327855657	0.743020803	0.964563948
POLR1B	1235.055202	-0.045481683	0.138733481	-0.327834947	0.743036463	0.964563948
HLX	103.4422643	0.081844784	0.249775936	0.327672814	0.743159061	0.964563948
LOC100288162	32.85274495	0.182466421	0.557125751	0.327513889	0.74327924	0.964563948
UTP11	618.1447137	-0.045765373	0.139748664	-0.327483437	0.743302269	0.964563948
FAM81A	112.2036735	-0.084705912	0.258742127	-0.327375805	0.743383664	0.964563948
CATIP-AS2	4.126500925	0.452496105	1.382664101	0.327263942	0.743468262	0.964563948
PPP1R12A-AS1	21.49646259	-0.188200121	0.575129575	-0.327230818	0.743493313	0.964563948
LOC100996583	4.312180728	0.354023975	1.081908417	0.327221759	0.743500165	0.964563948
SIRT2	228.4375376	0.088977157	0.271946275	0.327186525	0.743526812	0.964563948
FNDC3A	1468.066326	-0.038809939	0.118621724	-0.327173956	0.743536318	0.964563948
MRPS18C	60.0717618	0.116662585	0.35658689	0.327164538	0.743543441	0.964563948
BOD1L1	723.4214684	-0.051861551	0.158539693	-0.327120294	0.743576903	0.964563948
PHACTR4	568.5569505	0.040177508	0.122831543	0.327094388	0.743596496	0.964563948
CSTB	62.71633878	0.079808396	0.244138753	0.326897695	0.743745265	0.964563948
AMACR	58.60295029	0.114126845	0.349160892	0.326860332	0.743773525	0.964563948
KRI1	537.5071414	-0.071539463	0.218913475	-0.326793326	0.743824208	0.964563948
SLC27A1	83.49719182	0.116712536	0.357265835	0.326682611	0.743907954	0.964563948
NFATC4	913.5442646	0.072279642	0.221257057	0.326677225	0.743912027	0.964563948
TBC1D9B	2550.070982	0.026425174	0.080914224	0.326582551	0.743983643	0.964563948
MIR12115	1.99939484	0.466712896	1.42911813	0.32657405	0.743990073	0.964563948
CORT	3.831792512	-0.328996103	1.00761106	-0.326511008	0.744037762	0.964563948
LINC00298	3.827203822	-0.379929804	1.163618332	-0.326507234	0.744040617	0.964563948
ZNF599	70.87687835	-0.089926361	0.275544904	-0.326358279	0.744153299	0.964563948
SRRM2-AS1	15.72136477	0.177024685	0.54262601	0.326237007	0.744245044	0.964563948
SCARNA9	49.63976778	0.124632617	0.382063215	0.326209413	0.74426592	0.964563948
LOC103344931	69.6076457	-0.073618383	0.2256785	-0.326209114	0.744266146	0.964563948
KLHL36	454.7158732	0.071423795	0.218986102	0.326156749	0.744305763	0.964563948
PPP3CB	3744.853663	-0.043514822	0.133433028	-0.326117324	0.74433559	0.964563948
NR3C1	1128.326742	-0.052418171	0.160757412	-0.326070008	0.744371388	0.964563948
BEGAIN	137.4218089	-0.082534281	0.253168264	-0.326005636	0.744420091	0.964563948
PEX7	107.9464539	-0.067538507	0.207173615	-0.325999557	0.74442469	0.964563948
YPEL1	5.222241318	-0.264250614	0.810795006	-0.325915444	0.744488331	0.964576273
NAALADL2-AS2	42.50200081	-0.121860571	0.374505465	-0.325390634	0.744885443	0.964954675
AAGAB	868.0564623	0.041793545	0.128455128	0.325355211	0.744912249	0.964954675
NQO2	308.2202754	-0.043233519	0.132908924	-0.325286801	0.744964019	0.964954675
SERPINB6	823.5520436	-0.04351882	0.133816887	-0.325211718	0.745020841	0.964954675
NHLH1	3.005809241	0.415691617	1.278427699	0.325158487	0.745061125	0.964954675
RBIS	331.928228	0.046242733	0.142241511	0.325100126	0.745105293	0.964954675
LOC100288798	7.342161509	-0.242424763	0.74611373	-0.324916636	0.745244166	0.964990672
IFT140	542.6873696	-0.064807968	0.199503924	-0.32484558	0.745297946	0.964990672
RTBDN	2.304557067	0.396546328	1.221139617	0.324734635	0.745381919	0.964990672
ELOVL6	1377.008527	-0.03935331	0.121199818	-0.324697766	0.745409825	0.964990672
DCAF17	465.3303176	0.038843807	0.119699734	0.324510384	0.745551662	0.964990672
FAM114A2	520.2218801	-0.039388295	0.121378551	-0.324507868	0.745553566	0.964990672
RBPMS-AS1	15.19345644	-0.216100737	0.665948072	-0.324500883	0.745558854	0.964990672
LAMP5	3.00734412	0.401727592	1.238207281	0.324442925	0.745602726	0.964990672

STK38L	428.6569976	0.060540289	0.186611128	0.3244195	0.745620458	0.964990672
RPL23AP7	43.73147063	-0.094638528	0.291951339	-0.324158568	0.745817988	0.965176219
TIGD7	132.7755785	0.066495086	0.205192752	0.324061571	0.74589142	0.965201155
NT5C1B	2.838352264	0.3397178	1.049044126	0.323835567	0.746062527	0.965246096
MIR196A2	4.098759075	-0.421407012	1.301489247	-0.323788317	0.746098302	0.965246096
PPP2CB	2152.508137	0.039805654	0.122959199	0.32373059	0.746142009	0.965246096
SAP30	96.26740246	-0.075796169	0.234134249	-0.323729525	0.746142816	0.965246096
TAF4	320.5097918	-0.049536057	0.153124495	-0.323501848	0.746315208	0.9653207
SPINDOC	224.1919299	0.054080421	0.167208552	0.32343095	0.746368894	0.9653207
ZNRF2	90.78427728	0.122953079	0.380215301	0.323377514	0.746409357	0.9653207
PPP1R13B	11.67035447	0.187786979	0.58079851	0.323325518	0.746448731	0.9653207
MSL3	446.4796577	0.042437513	0.131285819	0.323245214	0.746509542	0.9653207
METTL7B	17.5511937	-0.23393032	0.723863111	-0.323169279	0.746567046	0.9653207
CRTAP	4453.423912	-0.061804481	0.191271345	-0.323124622	0.746600864	0.9653207
ARHGAP12	476.089955	-0.048818174	0.151157833	-0.322961587	0.746724334	0.9653207
PSMA2	598.8160235	-0.048376659	0.149791836	-0.322959249	0.746726104	0.9653207
EED	296.2209275	-0.076779123	0.237752851	-0.322936708	0.746743176	0.9653207
POLR2I	102.2603106	0.067710938	0.209753567	0.322811854	0.746837735	0.9653207
ERICH1	260.7181325	0.065134817	0.201798537	0.322771504	0.746868295	0.9653207
SPIDR	913.1827541	0.033334157	0.103312277	0.322654365	0.746957016	0.9653207
RNF146	361.6087708	0.050759503	0.157353353	0.322582915	0.747011134	0.9653207
MEA1	287.3227618	-0.053530459	0.165944554	-0.322580394	0.747013044	0.9653207
SLC35A2	363.3084321	0.052943465	0.164266621	0.322302029	0.747223897	0.965383731
C15orf61	102.9128602	0.070320365	0.21819273	0.322285556	0.747236375	0.965383731
LOC283387	9.483823721	0.204540558	0.634663415	0.322281942	0.747239112	0.965383731
SKOR1	5.949401847	-0.262988274	0.816512476	-0.322087269	0.747386582	0.965383731
LOC100128164	12.00900494	0.195519288	0.607172432	0.322016083	0.74744051	0.965383731
ATP6V1B1	24.85016911	0.144009812	0.447235584	0.321999897	0.747452772	0.965383731
LEO1	261.0858775	0.059806891	0.185782424	0.321918995	0.747514063	0.965383731
OR51B5	6.842923686	-0.274932433	0.854055306	-0.321914086	0.747517782	0.965383731
BASP1	1161.139644	0.072161013	0.224259833	0.32177413	0.747623814	0.965383731
TSC22D4	184.6186595	-0.060294656	0.187459479	-0.321641007	0.747724674	0.965383731
LOC101929340	3.959066813	-0.338852591	1.054378666	-0.321376562	0.747925042	0.965383731
NCAPG2	1135.64092	0.050508975	0.157164653	0.321376173	0.747925337	0.965383731
MBD3	1633.638485	-0.031085959	0.09672962	-0.3213696	0.747930318	0.965383731
DCAF4	557.3438725	-0.045801268	0.142538511	-0.321325567	0.747963683	0.965383731
NDUFA7	75.77793724	0.096143359	0.299224041	0.321308938	0.747976284	0.965383731
PAXIP1-AS1	28.4573767	-0.130017372	0.404972843	-0.321052075	0.748170928	0.965383731
SUSD5	680.903154	0.057466592	0.179005855	0.321031914	0.748186206	0.965383731
BABAM2	1402.029351	-0.085342566	0.265891196	-0.320968004	0.748234639	0.965383731
DOK2	3.807583139	0.331753871	1.033625645	0.320961339	0.748239689	0.965383731
EPS15L1	433.7292527	-0.03965612	0.123574161	-0.320909478	0.748278991	0.965383731
NAGA	565.5049097	-0.052137586	0.162480511	-0.320885166	0.748297416	0.965383731
GGH	1885.173999	-0.036003394	0.112257803	-0.320720636	0.748422107	0.965383731
DNAH9	2.908284611	0.398505155	1.243391445	0.32049855	0.748590429	0.965383731
MPI	1097.767065	0.041406257	0.129204704	0.320470196	0.74861192	0.965383731
TICAM1	139.5918621	0.061687608	0.192560161	0.320354988	0.748699243	0.965383731
HDHD2	563.3079114	-0.040353403	0.125968007	-0.320346445	0.748705719	0.965383731
IGFLR1	23.84311734	-0.134762468	0.420772695	-0.320273795	0.748760787	0.965383731
NDST2	1084.191953	-0.039760367	0.12415812	-0.32023976	0.748786585	0.965383731
CASTOR2	70.49608888	0.115036604	0.359349051	0.320124969	0.748873598	0.965383731
KRT80	2.100159946	-0.528102565	1.649818598	-0.320097352	0.748894533	0.965383731
AIFM1	467.0458594	-0.050341904	0.157288993	-0.320059929	0.748922901	0.965383731
MIF-AS1	90.39876499	-0.081862852	0.255809602	-0.320014775	0.74895713	0.965383731
LOC729609	4.291801566	0.316965699	0.990489277	0.320009218	0.748961343	0.965383731
GATA3-AS1	8.614823495	0.273614137	0.855061044	0.319993688	0.748973115	0.965383731
TLR6	321.4826271	0.075912395	0.237311165	0.319885476	0.749055148	0.965383731
TMEM117	126.8238854	-0.067869769	0.212201589	-0.31983629	0.749092436	0.965383731
NGLY1	316.7352175	-0.042554314	0.133056887	-0.319820452	0.749104443	0.965383731
CXCL8	4.004072671	-0.325078419	1.016836295	-0.319695924	0.74919885	0.965383731
SHISA5	1019.319498	0.07934345	0.248262306	0.319595235	0.749275187	0.965383731
TNFAIP6	48.64223514	-0.087443698	0.273647427	-0.319548766	0.749310418	0.965383731
DCUN1D2	235.1707206	0.062123259	0.194478593	0.31943495	0.749396712	0.965383731
LINC00933	4.554908846	0.301613654	0.944762195	0.319248225	0.74953829	0.965383731
FOXP4-AS1	21.83457119	-0.190336909	0.596249371	-0.319223664	0.749556913	0.965383731
GATAD1	748.1561902	0.050915239	0.159520123	0.319177528	0.749591896	0.965383731
C10orf95	14.95428374	0.217496056	0.681429044	0.319176381	0.749592766	0.965383731
SEC22C	703.8939472	0.037613007	0.117855992	0.31914378	0.749617487	0.965383731
HEPH	100.2463987	-0.111098045	0.348180639	-0.319081628	0.749664614	0.965383731
LRP1-AS	28.33810057	0.203735071	0.638757896	0.318955072	0.749760582	0.965383731
RAB11FIP3	569.8359512	0.041270082	0.129404315	0.318923537	0.749784495	0.965383731
AIF1L	6.852821169	-0.247103243	0.774860161	-0.318900436	0.749802014	0.965383731
MFN2	2180.556525	0.038938763	0.12213994	0.3188045	0.749874766	0.965383731
NPC1L1	3.231370679	0.320363938	1.00490861	0.318799078	0.749878878	0.965383731
MIR3064	14.004532	-0.239585348	0.752558275	-0.31836119	0.750210974	0.965741498
PIGY	8.21040908	-0.223553849	0.702500723	-0.318225792	0.75031367	0.965803929
TCEAL1	71.64272471	-0.080336742	0.252556623	-0.318093982	0.750413648	0.965821128
ZNF222	38.53564272	0.100586251	0.316296612	0.318012422	0.750475515	0.965821128
RANBP3	541.5136502	-0.038587172	0.121370261	-0.317929386	0.750538501	0.965821128
C1S	44.35350591	0.101750018	0.320087555	0.317881832	0.750574575	0.965821128
BCO2	22.00204096	0.156888161	0.49368449	0.317790338	0.75064398	0.965821128
ADPGK-AS1	38.26837195	0.144206898	0.453884397	0.31771724	0.750699433	0.965821128
HAAO	43.70246891	-0.097727209	0.307707702	-0.317597538	0.750790242	0.965821128
AIDA	1458.258354	0.047336369	0.149065551	0.317554049	0.750823235	0.965821128
ZNF384	418.8309894	-0.03986953	0.125612486	-0.317401009	0.750939342	0.965821128
PILRA	1.98036704	0.481787271	1.518149061	0.317351757	0.750976709	0.965821128
ROBO3	16.6085823	-0.169274691	0.533501341	-0.317290095	0.751023493	0.965821128
LOC101928796	15.71717674	-0.142495378	0.449120307	-0.317276632	0.751033707	0.965821128
UCKL1	300.9020428	-0.082819133	0.261062505	-0.317238714	0.751062476	0.965821128
LOC100506142	19.32014557	0.220616625	0.695495245	0.317207956	0.751085813	0.965821128

ABHD8	147.16127	0.075430284	0.237901645	0.317064994	0.751194286	0.965825305
ACTN4	5204.324117	0.053939601	0.170123835	0.317060812	0.751197459	0.965825305
DEF8	468.1273756	0.044374581	0.140005561	0.316948706	0.751282524	0.965864987
UNC5CL	12.56367832	0.188290367	0.5942583	0.316849368	0.751357902	0.96589221
CDCA2	1080.795196	0.044161969	0.139434098	0.316722883	0.751453884	0.965945914
NANS	538.7724112	-0.043974481	0.138953619	-0.316468773	0.751646723	0.966124106
TSR1	2159.61306	-0.040650062	0.128524136	-0.316283491	0.751787341	0.966162936
LINC02582	198.8314399	-0.114362119	0.361806704	-0.316086235	0.751937055	0.966162936
DLEU7	32.21139854	-0.159753129	0.505424082	-0.316077398	0.751943762	0.966162936
TCEA3	32.75063291	0.190803413	0.603943915	0.315929026	0.75205638	0.966162936
RANBP17	166.4702541	0.079086972	0.250430832	0.315803654	0.752151544	0.966162936
RTL3	46.91679508	0.131897241	0.417668788	0.315793865	0.752158975	0.966162936
ZNF780B	553.4233142	-0.080145968	0.253812558	-0.315768331	0.752178357	0.966162936
C19orf57	26.80467267	0.135181336	0.428129529	0.315748685	0.75219327	0.966162936
TELO2	744.3495428	0.101707003	0.322213524	0.315650945	0.752267465	0.966162936
CACYBP	987.4784586	-0.058799558	0.186300502	-0.315616745	0.752293426	0.966162936
TOMM5	393.8212299	-0.05026099	0.159279425	-0.31555231	0.75234234	0.966162936
SERPINC1	2.462695835	0.42871318	1.35890152	0.315485098	0.752393364	0.966162936
MORC2-AS1	5.701766826	-0.308110562	0.976997641	-0.315364694	0.75248477	0.966162936
SYT16	35.84488083	0.148979793	0.472772943	0.315119118	0.752671213	0.966162936
LINC01376	2.493184764	-0.353811579	1.123051154	-0.315044936	0.752727535	0.966162936
C8orf37	181.0512356	-0.061420423	0.194974593	-0.315017573	0.752748311	0.966162936
VTI1A	388.5429298	-0.041985244	0.133296399	-0.314976584	0.752779433	0.966162936
GCAT	228.4726405	0.046889892	0.148875418	0.314960605	0.752791565	0.966162936
LINC01695	2.681894231	0.598854366	1.902062542	0.314844729	0.752879549	0.966162936
NUDT10	18.39669255	-0.162384416	0.515834719	-0.314799315	0.752914032	0.966162936
HBEGF	28.21996207	0.125067085	0.39732937	0.314769294	0.752936828	0.966162936
MPND	200.123588	0.069849775	0.221926572	0.314742732	0.752956997	0.966162936
DPH6	181.4250284	-0.062246874	0.197889556	-0.314553609	0.753100607	0.966162936
NGFR	37.03459892	-0.15847911	0.503925698	-0.314489043	0.753149637	0.966162936
NPHP4	521.9483716	0.048050755	0.152829983	0.314406597	0.753212246	0.966162936
AKT3	1493.328258	0.096886278	0.308165476	0.314396926	0.753219591	0.966162936
FBRSL1	347.8582399	0.057884595	0.184129291	0.314369294	0.753240574	0.966162936
KMO	6.077412561	0.304036568	0.967276234	0.31432238	0.753276203	0.966162936
PIM1	1983.064767	0.172984942	0.550403938	0.314287253	0.753302879	0.966162936
NUP42	341.580193	-0.049861617	0.158650217	-0.314286475	0.753303469	0.966162936
HLA-F	116.1648658	-0.060507681	0.192599176	-0.314163758	0.753396667	0.966212926
TTBK1	6.079724702	0.220213444	0.701823463	0.313773271	0.753693246	0.966479686
FAM43B	4.283917796	-0.42446784	1.352906747	-0.313745083	0.753714657	0.966479686
CEBPA-DT	12.32596396	0.185651007	0.591967903	0.313616678	0.753812191	0.966479686
NRROS	2.937637238	0.359901012	1.148779819	0.313289811	0.754060491	0.966479686
SPEGNB	20.40388099	0.157815639	0.503764908	0.313272395	0.754073721	0.966479686
MIR1185-2	5.936217002	0.290873255	0.928576612	0.313246372	0.75409349	0.966479686
TMEM9B-AS1	40.56196416	-0.115019769	0.367191042	-0.313242306	0.754096579	0.966479686
SNORA11B	4.297410947	0.263586139	0.841495056	0.313235517	0.754101736	0.966479686
SFRP1	768.5405845	-0.314964107	1.006108334	-0.313051881	0.754241246	0.966479686
SDR39U1	150.1286396	-0.057744328	0.184460962	-0.313043623	0.75424752	0.966479686
AGAP4	50.20243444	0.109550951	0.34997761	0.31302274	0.754263385	0.966479686
ORAI3	156.6578791	0.119790058	0.38289577	0.312852916	0.754392411	0.966479686
DMXL1	792.7404922	0.036787126	0.117673774	0.312619581	0.7545697	0.966479686
LOC730101	297.8499711	-0.079439436	0.254170666	-0.312543682	0.754627371	0.966479686
GIT1	565.4998757	-0.040067891	0.128202238	-0.312536598	0.754632754	0.966479686
ACTR6	489.798033	0.035745629	0.114393982	0.312478228	0.754677107	0.966479686
ZFYVE27	249.0990453	0.06637902	0.212576006	0.312260171	0.754842807	0.966479686
MDK	84.30231757	-0.105094046	0.336580995	-0.312239989	0.754858144	0.966479686
HOXD4	124.8923423	-0.133895452	0.429073831	-0.312056905	0.754997277	0.966479686
PLA2G4E-AS1	2.916911755	0.385622483	1.235814934	0.312039022	0.755010868	0.966479686
HOXA11-AS	26.34887606	0.156185637	0.500980326	0.311760022	0.755222909	0.966479686
SLC25A53	15.93728332	-0.166207918	0.533253712	-0.311686377	0.755278882	0.966479686
ST20	29.64522998	0.105758885	0.339420424	0.311586686	0.755354654	0.966479686
LIMD1-AS1	2.252800565	-0.472045871	1.515121644	-0.311556417	0.755377661	0.966479686
LINC00862	8.26222717	0.226975229	0.72863142	0.311508977	0.755413719	0.966479686
KIAA1191	806.4991826	0.044251559	0.142068131	0.31148125	0.755434794	0.966479686
STAMBP	797.4932797	0.041557874	0.13343347	0.311450146	0.755458437	0.966479686
MIR3144	2.466676561	-0.526841341	1.691677049	-0.311431394	0.755472691	0.966479686
FES	5.50524183	0.26350645	0.846215405	0.31139406	0.755501069	0.966479686
EIF4G1	20840.25963	-0.031879759	0.102380304	-0.311385662	0.755507452	0.966479686
PHF20	754.3208292	-0.053494333	0.171829201	-0.311322714	0.755555301	0.966479686
ODR4	422.0899051	0.052629678	0.169192148	0.311064543	0.755751556	0.966479686
SNORD114-1	28.45641292	-0.136415405	0.438576934	-0.311040994	0.755769458	0.966479686
ANKRD18B	172.5739948	-0.058502027	0.188139597	-0.310950102	0.755838555	0.966479686
HCP5	17.8865987	-0.216127864	0.695173828	-0.310897585	0.75587848	0.966479686
BICDL1	11.64798331	-0.219486417	0.706104276	-0.310841365	0.755921221	0.966479686
SPATA2L	68.63438861	-0.102539489	0.329887879	-0.310831334	0.755928848	0.966479686
ATOX1	135.6709433	-0.059246388	0.190682224	-0.310707452	0.756023032	0.966479686
HOTS	3.414324466	0.426769636	1.373630048	0.310687464	0.756038228	0.966479686
INPP5E	405.8033199	0.069033506	0.22221208	0.310664958	0.75605534	0.966479686
P2RX5	9.20115374	0.193475287	0.622787891	0.310660001	0.756059108	0.966479686
BICDL2	2.904363572	0.385006334	1.239343476	0.310653456	0.756064084	0.966479686
ZBED4	1372.214277	-0.031680757	0.102041579	-0.310469102	0.756204253	0.966479686
TGIF2	491.0445967	0.04449002	0.143326697	0.310409864	0.756249294	0.966479686
BIN3	303.9288875	0.046294496	0.149149562	0.310389754	0.756264585	0.966479686
LINC00938	75.40048272	0.077461825	0.249599248	0.310344786	0.756298777	0.966479686
SATB2	1032.932585	0.049097204	0.158215091	0.310319348	0.756318119	0.966479686
OR51Q1	5.754511021	0.358610074	1.155661397	0.310307219	0.756327342	0.966479686
LOC105379183	3.219745398	0.405701761	1.307499441	0.310288286	0.756341738	0.966479686
BLVRB	151.2424312	-0.112410563	0.362321956	-0.310250487	0.75637048	0.966479686
MAL	1.79174909	0.739527729	2.383649405	0.31025021	0.756370691	0.966479686
IFNGR1	491.8335829	0.046969209	0.151448323	0.310133569	0.756459386	0.966523715
ATG14	607.6388722	-0.038602601	0.124584913	-0.309849725	0.756675236	0.966648906

SARNP	262.4476781	-0.052975564	0.170995721	-0.309806375	0.756708204	0.966648906
HOXC10	162.9140714	0.062107709	0.200482788	0.309790727	0.756720103	0.966648906
ZNF135	2.454201099	-0.357533769	1.154421416	-0.309708192	0.756782873	0.966659795
TMEM109	1368.228233	0.03324257	0.107411178	0.309488921	0.756949638	0.966783181
PLBD2	2586.519088	-0.050849606	0.164329645	-0.309436596	0.756989436	0.966783181
DRAM1	199.413704	-0.058536705	0.18921433	-0.309367187	0.757042228	0.966783181
MVD	360.6001266	0.061862545	0.200168696	0.309052045	0.757281937	0.967020001
RAB40A	4.77588857	0.293041941	0.948606483	0.308918341	0.757383645	0.96705825
ACOXL-AS1	2.22533894	-0.446135271	1.444411197	-0.308869989	0.757420426	0.96705825
MIR4307	5.27642597	-0.354933102	1.149583124	-0.308749402	0.757512161	0.967075996
ZCCHC2	268.6522242	0.053762149	0.17415152	0.308709044	0.757542864	0.967075996
PXT1	2.266424109	0.387003177	1.253933341	0.30863138	0.757601948	0.967082143
GRPEL2-AS1	5.161145607	-0.278751856	0.90390438	-0.308386442	0.757788297	0.967250731
KLHL7-DT	53.56885046	0.083342954	0.270406158	0.308213966	0.757919526	0.967348944
AXDND1	3.615328819	-0.317178959	1.02946388	-0.308101105	0.7580054	0.967387673
ISG20L2	705.4089745	-0.033358459	0.108329276	-0.30793577	0.758131207	0.967387673
SAMD9	475.3056507	-0.061037069	0.198227724	-0.307913888	0.758147858	0.967387673
FREM2	5.199933057	-0.294096131	0.955202714	-0.30788871	0.758167017	0.967387673
ZCCHC18	13.02264663	0.172211865	0.560085329	0.307474336	0.758482354	0.967679887
ADAMTS17	34.19483837	-0.134008508	0.436022711	-0.307342955	0.758582343	0.967679887
GPANK1	320.1382452	-0.044942115	0.14622878	-0.307341104	0.758583752	0.967679887
LINC01094	2.765179084	0.463663177	1.508817531	0.307302353	0.758613245	0.967679887
ZCCHC14	1801.197501	-0.077204633	0.251337946	-0.307174598	0.758710479	0.967734646
ST6GALNAC6	671.1005229	-0.050879699	0.165694431	-0.307069459	0.758790503	0.967740435
LOC102724908	4.982354286	0.332343445	1.082719746	0.306952419	0.758879589	0.967740435
LIME1	52.21465412	0.106080766	0.3457556	0.306808528	0.758989117	0.967740435
CT75	4.326883922	-0.405513721	1.32187953	-0.306770558	0.75901802	0.967740435
MICALL1	626.8560661	-0.052150216	0.170033106	-0.306706246	0.759066975	0.967740435
PCDHB15	58.96665171	-0.083463911	0.272147891	-0.306685865	0.759082489	0.967740435
LOC202181	8.360271187	0.248005673	0.809052425	0.306538445	0.759194712	0.967740435
MPDU1	462.3066628	-0.047903124	0.156297123	-0.30648756	0.75923345	0.967740435
PWWP3A	957.0718558	0.070873109	0.231268454	0.306453853	0.759259111	0.967740435
ZNF316	1057.711517	-0.04543509	0.148320154	-0.306331196	0.759352489	0.967740435
POP4	231.8524408	-0.050429178	0.164629417	-0.30631936	0.7593615	0.967740435
SEL1L	3644.259726	-0.032679517	0.10668684	-0.306312537	0.759366695	0.967740435
LRP1B	286.6659043	0.061227754	0.200024644	0.30610105	0.759527708	0.967771825
TOLLIP-AS1	7.422918696	-0.270155122	0.882939809	-0.305972297	0.759625738	0.967771825
KRT14	6.579002939	-0.38679025	1.264241694	-0.305946443	0.759645424	0.967771825
GSTM3	459.7564395	-0.053031764	0.173453282	-0.305740909	0.759801923	0.967771825
CYB5R4	220.9506839	0.061038379	0.199719459	0.305620588	0.759893543	0.967771825
PTGER2	151.2002505	0.099763292	0.326450898	0.305599686	0.75990946	0.967771825
TMSB15B-AS1	7.620916794	0.299440332	0.980120319	0.30551385	0.759974823	0.967771825
NPM2	3.518570488	0.38156454	1.248937848	0.305511231	0.759976817	0.967771825
FAM186B	24.66388536	0.183067197	0.599377718	0.305428766	0.760039615	0.967771825
AGFG1	1231.614636	0.033922607	0.11114228	0.305217842	0.760200244	0.967771825
TMEM196	15.60039348	0.181916999	0.596087713	0.305184951	0.760225293	0.967771825
ZNF286B	90.34707138	0.064129013	0.210134438	0.305180884	0.760228391	0.967771825
KATNAL2	172.2440305	0.066922506	0.219360996	0.305079333	0.760305731	0.967771825
LGALSL	417.431053	0.054022347	0.177115316	0.305012285	0.760356796	0.967771825
FOXN3-AS2	5.72384768	-0.349426277	1.145800839	-0.30496249	0.760394721	0.967771825
SEMA3B-AS1	10.60627109	0.227012983	0.7446634	0.304853149	0.760478	0.967771825
ITSN2	694.1353544	0.03139836	0.102999885	0.304838789	0.760488937	0.967771825
GUSBP5	5.041294734	-0.321235975	1.054921609	-0.304511703	0.760738079	0.967771825
SPSB4	12.47830655	-0.219425888	0.720605188	-0.304502231	0.760745293	0.967771825
SLC2A8	234.0756583	0.050888541	0.167129192	0.304486252	0.760757465	0.967771825
LONRF1	819.3391319	0.044947046	0.14765151	0.304413049	0.760813227	0.967771825
ZNF181	216.3222714	-0.053169789	0.174709657	-0.304332281	0.760874754	0.967771825
MIR26B	2.986116722	-0.350118641	1.150864109	-0.3042224	0.760958461	0.967771825
SNORA112	6.319044581	0.333036417	1.09486785	0.304179557	0.760991099	0.967771825
GOLGA2P5	89.55198902	0.125998529	0.414256694	0.304155687	0.761009283	0.967771825
ATP13A1	1237.781044	-0.046433478	0.152668459	-0.304145851	0.761016776	0.967771825
RTL8C	283.1807867	0.057614893	0.189516863	0.30400932	0.761120791	0.967771825
PKD1L3	6.453617972	0.264149664	0.86914202	0.303920025	0.761188821	0.967771825
TBC1D24	429.1715878	0.052527144	0.172842243	0.303902235	0.761202375	0.967771825
SPATA12	7.728668921	0.251347694	0.827131997	0.303878576	0.7612204	0.967771825
SLC35D1	632.409318	0.048429235	0.159376266	0.303867298	0.761228993	0.967771825
ZNF552	88.13760971	-0.089384548	0.294211814	-0.303810195	0.761272499	0.967771825
MIR3613	2.644215862	0.430734131	1.417932343	0.303776223	0.761298383	0.967771825
COPB2	4534.504546	0.048926967	0.161067135	0.303767536	0.761305001	0.967771825
FBXL6	420.2309063	0.113157666	0.37256102	0.303729214	0.7613342	0.967771825
CA3	5.794060724	0.22807844	0.750966568	0.303713174	0.761346421	0.967771825
COL4A4	25.83184584	0.151733491	0.499719968	0.303637037	0.761404431	0.967776531
NKX6-1	184.6701491	-0.057246511	0.188648204	-0.303456434	0.761542044	0.967882407
PHYKPL	831.9860736	0.053985185	0.178006222	0.303276957	0.761678806	0.967919741
KCNH3	16.3408021	-0.157601109	0.519792218	-0.303200208	0.761737291	0.967919741
STAT3	1933.689143	0.066617573	0.219796735	0.303087184	0.761823421	0.967919741
NOX4	24.44301016	-0.1226645	0.404845205	-0.302991116	0.761896632	0.967919741
LINC01356	18.0613146	-0.170017975	0.561324295	-0.302887255	0.761975785	0.967919741
PCDHB19P	11.65981233	0.169459378	0.559620812	0.302811073	0.762033845	0.967919741
SNORD114-8	3.569105147	0.351055802	1.159469803	0.3027727	0.76206309	0.967919741
DUBR	108.5303452	-0.100765658	0.33282451	-0.302759129	0.762073433	0.967919741
HOXB-AS1	35.73481189	0.115758221	0.382416108	0.302702263	0.762116773	0.967919741
PCDHA12	5.97792457	0.245416259	0.811040982	0.302594153	0.762199171	0.967919741
AXIN1	571.8657263	-0.038945675	0.128731843	-0.302533343	0.762245519	0.967919741
POMP	732.1540481	0.045633037	0.150855211	0.302495596	0.76227429	0.967919741
RUNDC3B	48.30003539	0.103121891	0.340942743	0.302461023	0.762300642	0.967919741
PENK	618.7166424	-0.312553297	1.033507095	-0.302420079	0.76233185	0.967919741
CABLES2	79.2343638	-0.073285113	0.242510448	-0.302193631	0.762504459	0.968058557
MAPKAPK3	462.7353453	-0.050376764	0.166741884	-0.302124233	0.76255736	0.968058557
MAGEH1	114.4337562	-0.059066298	0.195543074	-0.302062847	0.762604154	0.968058557

TTC28	774.8146326	-0.038034546	0.126003752	-0.301852487	0.762764517	0.968143993
RGL4	189.1049286	0.096539066	0.319875284	0.301802205	0.76280285	0.968143993
OSBP	1531.84376	-0.051898227	0.172097436	-0.301563045	0.762985183	0.968143993
FKBP4	2093.660639	0.046471352	0.154157119	0.301454466	0.763067967	0.968143993
PARM1	4.602284907	0.261747021	0.868843695	0.301411685	0.763100585	0.968143993
KIF1C	2538.900239	-0.041999265	0.139370186	-0.301350429	0.76314729	0.968143993
ALDH1A2	4.883195443	-0.260143172	0.863332821	-0.301324316	0.763167201	0.968143993
SMAD3	2655.011925	0.068681932	0.228062653	0.301153789	0.763297226	0.968143993
VARS1	5341.246115	0.040757663	0.135359881	0.30110593	0.763333719	0.968143993
AGPAT5	4552.589552	-0.039114498	0.129911521	-0.301085678	0.763349162	0.968143993
PRCD	7.414808974	0.240393248	0.79849135	0.301059301	0.763369275	0.968143993
STAG3L2	172.068683	0.09300302	0.309000821	0.300979846	0.763429863	0.968143993
SPATA32	2.59007917	-0.517377261	1.719097919	-0.300958576	0.763446083	0.968143993
ANKRD18CP	20.65032112	0.131060534	0.435492925	0.300947561	0.763454482	0.968143993
RPS6KL1	33.02739765	0.155350513	0.516276384	0.300905713	0.763486394	0.968143993
AFMID	377.1706358	-0.041473138	0.137911467	-0.300722914	0.763625795	0.968242625
PEF1	742.3822489	-0.042916996	0.142759056	-0.300625383	0.763700173	0.968242625
MRPL57	231.4579289	0.050057707	0.166633148	0.300406659	0.763866985	0.968242625
LINC00517	2.620927583	0.421338534	1.403082515	0.300294907	0.763952217	0.968242625
HNRNPDL	2261.257035	-0.063937072	0.212974818	-0.300209538	0.76401733	0.968242625
THOP1	1019.290052	-0.066357441	0.221065589	-0.300170827	0.764046856	0.968242625
CDH18-AS1	23.19151447	0.180242398	0.600582554	0.30011261	0.764091261	0.968242625
KIF3C	391.3996959	0.091733215	0.305665972	0.300109348	0.764093749	0.968242625
KRIT1	557.3846418	-0.034016848	0.113356706	-0.300086771	0.764110969	0.968242625
TRAF3IP1	146.313815	-0.055782602	0.185914264	-0.300044765	0.76414301	0.968242625
RPS10P7	11.61763522	-0.191275708	0.63754307	-0.300020057	0.764161856	0.968242625
LOC101928429	4.171693964	0.361406267	1.205928756	0.299691226	0.764412692	0.968478066
SRD5A3-AS1	2.660832391	-0.385753886	1.287417055	-0.299633972	0.764456368	0.968478066
RPP21	60.20607304	-0.09160126	0.305797397	-0.299548854	0.764521302	0.968485379
BMPER	13.05737521	-0.173866028	0.580847118	-0.299331825	0.764686874	0.968485379
EMC9	76.75820397	-0.082159957	0.274509883	-0.299296899	0.76471352	0.968485379
FAM228B	42.30443738	-0.12675822	0.423558364	-0.299269785	0.764734207	0.968485379
CCDC169	45.45815757	-0.089092396	0.297724086	-0.299244838	0.76475324	0.968485379
POTEG	2.169185658	0.393971221	1.316827426	0.29918212	0.764801091	0.968485379
NKAIN3-IT1	138.3977319	0.28466626	0.951654489	0.299127744	0.764842578	0.968485379
ABT1	560.8408064	0.041347737	0.13830061	0.298970026	0.764962916	0.968509985
DTYMK	502.8180982	-0.056629165	0.189420659	-0.298959812	0.764970709	0.968509985
SNORA67	5.158953349	0.241177439	0.80699873	0.298857273	0.765048949	0.96854023
KCTD19	19.21557872	0.154187321	0.516202657	0.298695327	0.765172523	0.968584096
NEURL1	15.02957001	-0.1410006	0.472138415	-0.298642507	0.765212828	0.968584096
ZNF605	661.1443549	0.058359252	0.195444103	0.298598172	0.765246661	0.968584096
LINC00654	40.06763324	-0.107121043	0.358880257	-0.298486864	0.7653316	0.968593608
ZMAT4	27.84167466	-0.161041105	0.539648257	-0.298418651	0.765383656	0.968593608
TCP11L1	299.521085	0.052283719	0.175278957	0.298288626	0.765482885	0.968593608
TUBB2A	231.4205562	0.106214775	0.356236139	0.298158337	0.765582319	0.968593608
ZNF485	92.75565076	-0.080421814	0.269748107	-0.298136712	0.765598823	0.968593608
BIVM	425.6921643	0.043340107	0.145512344	0.297844887	0.765821554	0.968593608
STAU2	350.949255	-0.044045342	0.147922052	-0.297760483	0.765885977	0.968593608
GBP3	252.0755515	0.072242428	0.242710283	0.297648814	0.765971214	0.968593608
OGFR-AS1	10.30633243	-0.276774391	0.930186092	-0.297547333	0.766048677	0.968593608
CSRNP1	33.44046431	-0.139343212	0.46850323	-0.297422095	0.766144277	0.968593608
DLEU2L	15.23812628	0.194261969	0.653178513	0.297410226	0.766153338	0.968593608
ASB13	478.6567602	-0.052287906	0.175816985	-0.29739963	0.766161426	0.968593608
ATP6V0E2	7.479013238	-0.323371144	1.087604253	-0.297324273	0.766218952	0.968593608
GABRR2	16.23921325	0.170418806	0.573205137	0.29730858	0.766230931	0.968593608
MRC2	7623.711542	-0.054589173	0.183614992	-0.297302375	0.766235668	0.968593608
SH3PXD2B	3601.27915	-0.073358029	0.246801962	-0.297234382	0.766287574	0.968593608
ADAMTSL3	43.06600923	-0.149127802	0.501951222	-0.297096203	0.766393063	0.968593608
KCNAB2	224.4913264	0.187157852	0.630000938	0.297075513	0.766408859	0.968593608
C17orf78	13.52002536	0.212321352	0.714883516	0.297001325	0.766465497	0.968593608
PIM3	325.6040976	0.049668795	0.167253726	0.296966746	0.766491897	0.968593608
MYO5C	37.64736814	0.111380532	0.375274531	0.296797471	0.766621136	0.968593608
LRRIQ4	5.474997853	0.28127598	0.947761223	0.296779371	0.766634956	0.968593608
RDH11	2236.947233	0.033451956	0.112744406	0.296706129	0.766690878	0.968593608
NCAM2	333.1289896	0.036019406	0.121473629	0.296520371	0.766832712	0.968593608
GTF3C2-AS1	9.677181377	-0.245541723	0.828443132	-0.296389352	0.766932756	0.968593608
ME3	39.35224301	-0.134955338	0.455459676	-0.296305789	0.766996565	0.968593608
ATP13A5	4.961422986	-0.276425457	0.932983103	-0.296281311	0.767015257	0.968593608
DCAF8L1	4.21129727	-0.330453006	1.115592027	-0.29621313	0.767067322	0.968593608
LINC00693	4.336106559	0.27806486	0.938807282	0.296189501	0.767085366	0.968593608
LOC101927839	3.386862211	0.440494522	1.487571302	0.296116577	0.767141055	0.968593608
SNORA73B	200.3907807	0.092275205	0.311667757	0.29606914	0.76717728	0.968593608
TNFAIP8	137.35545	-0.068802952	0.232437123	-0.296006727	0.767224944	0.968593608
IL22RA1	20.77567335	0.170770118	0.577350424	0.295782442	0.767396233	0.968593608
ZSCAN20	172.435219	0.061056861	0.206533545	0.295626849	0.767515067	0.968593608
CYTH3	934.7076504	-0.054617439	0.18476927	-0.295598066	0.767537051	0.968593608
LRCH3	1346.012488	-0.044215485	0.149601905	-0.295554294	0.767570483	0.968593608
ATG7	335.9348208	-0.037135142	0.125662999	-0.295513732	0.767601464	0.968593608
LRRC8A	925.5803279	-0.037773558	0.127825859	-0.295507951	0.76760588	0.968593608
MLLT10P1	26.81632601	-0.147235384	0.498310278	-0.295469289	0.76763541	0.968593608
VPS8	1880.979702	0.026739544	0.090507329	0.295440652	0.767657282	0.968593608
PSMC1	460.5397924	-0.065404374	0.221434197	-0.295367086	0.767713473	0.968593608
LINC01592	28.44832884	-0.18770081	0.635551748	-0.295335213	0.76773782	0.968593608
PACSIN3	430.8104568	-0.03831654	0.129741865	-0.295329037	0.767742537	0.968593608
SLC25A16	370.9519903	0.055379504	0.187542561	0.295290326	0.767772106	0.968593608
NUCB1-AS1	4.972989342	0.307239801	1.040481987	0.295286036	0.767775383	0.968593608
MIR5699	4.766111325	-0.47698027	1.61571581	-0.295212975	0.767831191	0.968593608
PDS5B	1638.344064	0.031841039	0.107881318	0.29514878	0.767880227	0.968593608
LOC407835	3.491484149	0.304187339	1.031015053	0.295036758	0.7679658	0.968593608
DHRS1	169.3964503	0.054378587	0.184434002	0.294840355	0.768115837	0.968593608

RNF222	7.373779935	0.217077066	0.736325361	0.29481134	0.768138003	0.968593608
GATB	227.91719	-0.056206061	0.190703185	-0.294730586	0.768199697	0.968593608
CLDN2	3.304737805	-0.326184151	1.106724653	-0.294729271	0.7682007	0.968593608
RBM28	1356.533189	0.063266293	0.214669221	0.294715248	0.768211414	0.968593608
MDP1	86.97835035	0.078948099	0.267931331	0.294657957	0.768255184	0.968593608
SH3GLB1	1405.651096	-0.061370603	0.208307439	-0.294615515	0.768287609	0.968593608
PRR5L	286.31667	-0.066831002	0.226901396	-0.294537643	0.768347103	0.968593608
DDX11	699.5237506	0.054396664	0.184689252	0.294530749	0.76835237	0.968593608
APOO	193.0111254	0.054535779	0.185327608	0.294266892	0.76855397	0.968607306
HLTF	1625.708317	-0.037752349	0.128300461	-0.294249523	0.768567241	0.968607306
DIRAS3	3.274756945	-0.32809691	1.115644912	-0.29408722	0.768691257	0.968607306
SVIL-AS1	393.4796906	-0.044316618	0.150750966	-0.293972368	0.768779018	0.968607306
HMOX2	407.4710297	-0.042723999	0.145349882	-0.293938998	0.768804518	0.968607306
SCOC-AS1	9.718302322	-0.206379939	0.702391497	-0.293824654	0.768891895	0.968607306
SIKE1	1159.604743	0.045145203	0.153653844	0.293811089	0.768902261	0.968607306
SAPCD2	236.517595	-0.06134219	0.208859145	-0.293701242	0.768986205	0.968607306
EXOSC10-AS1	3.593863865	0.34961642	1.19062385	0.293641371	0.769031959	0.968607306
LRRC10B	3.352630841	0.327926631	1.116798016	0.293631101	0.769039808	0.968607306
TMEM74	272.1977343	0.042554898	0.144946765	0.293589842	0.769071339	0.968607306
PDE11A	3.984211504	-0.276283226	0.941341097	-0.29349959	0.769140313	0.968607306
AGER	76.22439301	0.085653987	0.291848997	0.293487344	0.769149672	0.968607306
LINC00383	3.148917156	-0.40137573	1.36845081	-0.293306655	0.769287767	0.968607306
LRRTM2	52.12055086	0.117603712	0.401065632	0.293228097	0.769347809	0.968607306
FBLIM1	274.1872427	-0.061782118	0.210737362	-0.293171167	0.769391322	0.968607306
DAB2IP	884.4869507	0.171146171	0.583869373	0.293124077	0.769427314	0.968607306
CFAP73	17.89977992	0.18000201	0.614318844	0.293010725	0.769513954	0.968607306
MIR3192	3.633188452	-0.292090422	0.997404629	-0.292850477	0.769636443	0.968607306
ZBTB12	268.8438505	0.04820769	0.164647684	0.292793007	0.769680374	0.968607306
RAP2A	1009.03265	0.030457037	0.104023061	0.292791204	0.769681752	0.968607306
RAB33B	108.9592452	-0.074569089	0.254707783	-0.292763292	0.769703089	0.968607306
PSMD2	9770.861937	-0.036493703	0.12469029	-0.292674777	0.769770751	0.968607306
PTPN2	283.6363873	-0.04823402	0.164845283	-0.292601758	0.76982657	0.968607306
SLC29A4	67.89766256	0.11659529	0.398618605	0.292498364	0.769905611	0.968607306
SEC13	1239.947581	0.052939437	0.181062577	0.29238199	0.769994577	0.968607306
RHCE	9.377112404	-0.196836616	0.673276844	-0.292356135	0.770014343	0.968607306
PIP4K2A	1051.15283	0.050731478	0.173618681	0.292200571	0.770133275	0.968607306
NRADDP	2.494892972	0.480631247	1.645299767	0.292123816	0.770191958	0.968607306
TRAM2	2100.603134	0.039422178	0.134971298	0.292078235	0.770226808	0.968607306
LOC101927550	35.62790183	0.148339454	0.507943226	0.292039438	0.77025647	0.968607306
HNRNPL	4818.978044	-0.04054613	0.138852492	-0.292008661	0.770280002	0.968607306
PSMD1	2330.494059	-0.058728768	0.201150517	-0.291964291	0.770313926	0.968607306
RPN1	4728.717033	-0.030285839	0.103841982	-0.29165313	0.770551849	0.968607306
DAPK3	763.0454364	0.041661807	0.142889143	0.291567338	0.770617451	0.968607306
TRAPPC13	186.0194893	0.055863498	0.19161999	0.291532727	0.770643918	0.968607306
COL11A1	4153.79139	-0.087005339	0.298578651	-0.291398391	0.770746645	0.968607306
NDRG1	4407.883201	0.233414316	0.801202952	0.291329825	0.770799079	0.968607306
C3orf20	3.470647568	-0.297320154	1.020639596	-0.291307681	0.770816014	0.968607306
SNX7	574.0937937	-0.052724169	0.181018849	-0.291263419	0.770849862	0.968607306
TSPAN32	30.29965122	-0.11127026	0.382424442	-0.290960115	0.771081824	0.968607306
NOMO1	2421.357054	-0.032481005	0.111679763	-0.290840566	0.771173259	0.968607306
TENM4	229.7094507	-0.192060018	0.660429668	-0.290810706	0.771196097	0.968607306
CCN3	523.8543948	-0.043503033	0.149645479	-0.290707296	0.771275191	0.968607306
MIR376B	3.241266358	-0.319390651	1.098738212	-0.29068858	0.771289507	0.968607306
EOGT	492.0539182	0.03724889	0.128169839	0.290621334	0.771340942	0.968607306
NAA30	745.0602429	-0.033762018	0.116172742	-0.290619098	0.771342652	0.968607306
SERPINE2	748.9644579	0.048214592	0.16590743	0.290611408	0.771348534	0.968607306
PART1	3.235753302	0.330935966	1.139211407	0.290495657	0.771437073	0.968607306
MRPL35	607.9298419	-0.05147613	0.177218868	-0.290466419	0.771459437	0.968607306
HSPB6	1198.607727	-0.044695695	0.154006411	-0.290219707	0.77164816	0.968607306
IRAK4	306.457368	-0.051047731	0.175986428	-0.290066296	0.77176552	0.968607306
MRPL34	91.61666649	0.082241163	0.283542928	0.290048366	0.771779236	0.968607306
COPE	1044.825952	-0.052202077	0.179978184	-0.290046692	0.771780517	0.968607306
STOX1	3.277757918	0.340518537	1.174049149	0.290037719	0.771787382	0.968607306
THAP5	681.6888136	0.045936662	0.158391874	0.290019054	0.771801661	0.968607306
AADACL2-AS1	264.1101516	0.084159252	0.290206218	0.2899981	0.771817691	0.968607306
LINC01126	39.4564573	0.133456405	0.460431561	0.289850688	0.771930468	0.968607306
MRPL15	910.8891915	-0.053303336	0.183918597	-0.289820261	0.771953747	0.968607306
LINC01001	29.9797383	0.12458996	0.429923309	0.289795778	0.771972478	0.968607306
PRMT5-AS1	35.97429001	0.147636559	0.50946845	0.28978548	0.771980357	0.968607306
MIER3	324.2682663	-0.043625691	0.1505977	-0.289683643	0.772058271	0.968607306
CLCN5	593.3759758	-0.037980334	0.131111924	-0.289678722	0.772062036	0.968607306
KCTD8	24.06731118	-0.125063381	0.431862504	-0.289590738	0.772129353	0.968607306
LINC00351	9.30199657	0.215085114	0.743303649	0.289363726	0.77230305	0.968607306
LRFN3	190.445064	0.071045819	0.245547069	0.289336863	0.772323605	0.968607306
ITPK1-AS1	12.13775475	0.286352337	0.990227531	0.289178323	0.772444919	0.968607306
PHLDA3	154.3613779	0.068713024	0.237666758	0.289114996	0.772493378	0.968607306
GLYCTK	82.66496966	0.128039792	0.442920642	0.289080661	0.772519652	0.968607306
TRIM38	646.2480602	-0.048758325	0.168742154	-0.288951657	0.772618372	0.968607306
HERC5	12.62778636	0.223787963	0.774632939	0.288895491	0.772661355	0.968607306
BYSL	557.5279585	0.044437659	0.153942396	0.288664203	0.772838359	0.968607306
GUCY2C	38.11218964	0.090768976	0.314458552	0.288651638	0.772847975	0.968607306
POLE2	334.8939535	0.084975264	0.294567222	0.288474947	0.772983205	0.968607306
RNF39	20.12539134	-0.16504266	0.572124671	-0.288473244	0.772984509	0.968607306
AHDC1	528.6505684	0.042008502	0.14565285	0.288415242	0.773028902	0.968607306
XPNPEP2	5.142046331	-0.259827713	0.900901058	-0.288408711	0.773033901	0.968607306
RYK	814.7977855	-0.03286794	0.113969394	-0.288392689	0.773046163	0.968607306
CCN4	16363.55466	-0.163701946	0.567919298	-0.288248605	0.773156445	0.968607306
ABCB10	803.6957384	-0.047216951	0.163880041	-0.288118985	0.77325566	0.968607306
PTRHD1	128.7404447	-0.09502196	0.329825209	-0.28809793	0.773271777	0.968607306
MIR300	6.329315018	0.303286345	1.052870051	0.288056769	0.773303284	0.968607306

CCL27	3.893476912	0.315799031	1.096418982	0.288027694	0.77332554	0.968607306
MEGF9	716.1994081	-0.072345106	0.251255592	-0.287934313	0.77339702	0.968607306
HDAC7	389.3288784	-0.041976563	0.145800892	-0.287903335	0.773420734	0.968607306
ALG1L9P	16.16170098	0.186713156	0.64926965	0.287574132	0.773672748	0.968607306
STXBP6	444.323786	-0.035531189	0.123584317	-0.287505649	0.773725177	0.968607306
GOLGA8O	8.908199168	0.241699716	0.840812663	0.287459652	0.773760392	0.968607306
ZRANB2-AS1	86.48376272	-0.07970482	0.277407439	-0.287320411	0.773866995	0.968607306
MMP19	6.029334165	0.245177256	0.853466293	0.287272337	0.773903802	0.968607306
FAM227B	63.37944253	0.075185904	0.261731537	0.287263449	0.773910607	0.968607306
SSR2	2425.908691	-0.047716267	0.1661176	-0.287243899	0.773925576	0.968607306
HFE	255.7193838	0.058089304	0.202263755	0.287195817	0.773962389	0.968607306
TSPAN19	2.683526468	-0.419977712	1.462357378	-0.287192254	0.773965117	0.968607306
EGFR-AS1	9.821092953	0.223717887	0.779111751	0.287144799	0.774001451	0.968607306
SNORD121A	3.043809819	0.334363665	1.164646275	0.287094607	0.774039881	0.968607306
C21orf62	6.406128242	0.23303389	0.81175172	0.287075326	0.774054644	0.968607306
KIAA1614-AS1	4.520959584	-0.272081452	0.947816038	-0.287061456	0.774065264	0.968607306
COPG1	3694.483726	0.055547286	0.193564803	0.286969971	0.774135313	0.968607306
TMEM30A	3111.604486	0.030326346	0.105688805	0.286939999	0.774158263	0.968607306
ZNF22-AS1	4.120099815	-0.287757677	1.003024061	-0.286890104	0.774196468	0.968607306
ENDOV	97.19323143	0.09524465	0.332048593	0.286839494	0.774235221	0.968607306
AUNIP	128.5593579	0.075501224	0.263220813	0.286836073	0.774237841	0.968607306
ORC6	381.6975548	-0.075011287	0.261558129	-0.286786295	0.774275957	0.968607306
MIA3	2561.411472	0.03648608	0.127270403	0.286681579	0.774356143	0.968607306
SERP1	3246.192239	0.050576676	0.176425126	0.28667502	0.774361166	0.968607306
POU3F1	1.834485545	-0.418930289	1.461471672	-0.286649613	0.774380622	0.968607306
PRH1	14.58440352	0.200292128	0.69876602	0.286636903	0.774390355	0.968607306
NKIRAS2	442.0251674	-0.044486708	0.155208833	-0.28662485	0.774399585	0.968607306
SNRNP35	93.03425256	0.080179907	0.279840213	0.286520318	0.774479634	0.968607306
DOP1A	406.7737465	0.066187589	0.231010356	0.286513516	0.774484843	0.968607306
DXO	262.2892144	-0.040104831	0.13998818	-0.286487269	0.774504943	0.968607306
FAM171B	1354.812544	-0.035600113	0.124301105	-0.286402227	0.774570069	0.968607306
SEPTIN7-AS1	11.31127222	-0.154928007	0.541142674	-0.286297892	0.774649973	0.968607306
ZNF225	115.899735	-0.05974751	0.208718096	-0.286259369	0.774679476	0.968607306
BDKRB1	9.262217746	0.231387437	0.808517708	0.286187222	0.77473473	0.968607306
HABP4	433.7591044	-0.070965495	0.248015424	-0.286133394	0.774775955	0.968607306
LOC100506188	7.352558362	0.309666851	1.08226675	0.286128028	0.774780065	0.968607306
NSFL1C	515.8733327	0.039545775	0.138242511	0.286060885	0.77483149	0.968607306
TMEM106B	1276.350735	-0.054373298	0.190183357	-0.285899352	0.77495521	0.968623111
CCNA2	1062.662861	0.028118936	0.098356984	0.285886517	0.774965041	0.968623111
USP14	1999.186329	-0.038757978	0.135712396	-0.285589078	0.775192869	0.968623111
LINC02509	2.075755666	-0.477524436	1.672172769	-0.285571231	0.77520654	0.968623111
WHAMMP3	8.896865601	-0.248643647	0.870696383	-0.285568715	0.775208468	0.968623111
ZC3H13	724.5563835	0.033578818	0.117605874	0.285519905	0.775245857	0.968623111
ZNF343	249.1511527	-0.047759969	0.167322597	-0.285436456	0.775309781	0.968623111
TCF21	4.428356951	0.321416577	1.126599227	0.285298063	0.775415797	0.968623111
MLKL	155.1900474	-0.055388262	0.194178165	-0.285244544	0.775456796	0.968623111
ZNF786	68.88987043	-0.071049421	0.249168387	-0.285146211	0.775532128	0.968623111
TSHZ1	852.749506	0.080649955	0.282876329	0.285106765	0.775562347	0.968623111
LOC645513	98.08069482	0.073500568	0.257812459	0.285093157	0.775572772	0.968623111
GORASP2	920.0513621	0.044172322	0.154948219	0.285077956	0.775584418	0.968623111
GBP1P1	2.549855514	0.340107257	1.193145541	0.285050939	0.775605116	0.968623111
SLC4A2	1301.566488	-0.071504594	0.251169436	-0.284686685	0.775884193	0.968650729
ITGB1	7185.649313	-0.040811803	0.143398035	-0.284605036	0.775946752	0.968650729
GOLGA8Q	2.244030474	-0.414858008	1.457786062	-0.284580858	0.775965279	0.968650729
LAMB2	1838.295241	0.033259009	0.116872989	0.28457396	0.775970563	0.968650729
TMEM253	25.50329602	0.131561488	0.462902041	0.284210213	0.776249289	0.968650729
ANKRD65	2.120406363	-0.403227539	1.418891242	-0.284184952	0.776268647	0.968650729
GPR137C	55.21843338	0.084901327	0.298799476	0.284141484	0.776301957	0.968650729
TIGD6	110.8525731	-0.071791897	0.252708368	-0.284089908	0.776341481	0.968650729
OVCA2	49.63926012	0.078609909	0.27686125	0.283932507	0.776462104	0.968650729
MATN1	28.73093336	0.109776352	0.386684201	0.283891485	0.776493542	0.968650729
APOOL	549.0244867	0.049989144	0.176094269	0.283877177	0.776504508	0.968650729
LOC101927770	47.88237968	-0.127879337	0.450555243	-0.283826099	0.776543652	0.968650729
TMEM18	373.1542902	-0.036585524	0.128913938	-0.283798049	0.77656515	0.968650729
MAP3K11	240.6865091	-0.065543048	0.230956731	-0.283789297	0.776571857	0.968650729
ZBTB8A	463.4024008	-0.05982782	0.210882726	-0.283701853	0.776638875	0.968650729
BIRC5	1714.80291	-0.039888418	0.140606227	-0.283688843	0.776648846	0.968650729
ESCO2	644.0003208	0.0335814	0.118381277	0.283671552	0.776662097	0.968650729
IMP4	616.0420102	0.040397146	0.142443034	0.283602118	0.776715314	0.968650729
SPDYE18	6.148045164	-0.210408635	0.741965628	-0.283582725	0.776730177	0.968650729
MEF2C	1042.39062	0.048334035	0.170510565	0.283466511	0.776819249	0.968650729
PROX1	4.245477409	0.316390783	1.11727716	0.283180212	0.777038696	0.968650729
SNORD38D	10.49906977	0.191001722	0.674900839	0.283007089	0.777171403	0.968650729
FPGT	391.2105309	-0.057572411	0.203495282	-0.282917668	0.777239951	0.968650729
GOLGA6L4	13.9023819	-0.181669265	0.642309018	-0.282837794	0.777301182	0.968650729
LY6G5B	59.08462478	0.147290582	0.520761882	0.282836718	0.777302006	0.968650729
FCHO1	4.306997058	0.252836238	0.894007192	0.282812309	0.777320718	0.968650729
WASF2	3735.670098	-0.050473569	0.178512894	-0.282744667	0.777372573	0.968650729
LOC101927851	3.169053271	0.426520014	1.508640149	0.282718191	0.777392871	0.968650729
PPP4R2	1261.776666	-0.029710722	0.105105693	-0.282674719	0.777426198	0.968650729
SLC25A24	1655.21199	-0.035554473	0.125794688	-0.282638901	0.777453657	0.968650729
COMMD4P1	2.291393636	-0.383541383	1.357088283	-0.282620805	0.77746753	0.968650729
CLDN14	2.487116899	-0.423937877	1.500239513	-0.28258013	0.777498713	0.968650729
CNEP1R1	289.9347151	-0.043275877	0.153167314	-0.2825399	0.777529557	0.968650729
TNK1	76.08152011	-0.075846276	0.268562976	-0.282415236	0.777625134	0.968650729
SCARB2	1317.079922	0.048973638	0.173460689	0.282332779	0.777688353	0.968650729
PTPRO	2.809057249	-0.325963149	1.154634172	-0.282308594	0.777706897	0.968650729
HNRNPA1L2	126.2040085	-0.061496158	0.21787837	-0.282249943	0.777751865	0.968650729
SQOR	46.76510526	-0.091498475	0.324201953	-0.282226785	0.777769621	0.968650729
HIGD1C	3.844934239	-0.295000309	1.045283616	-0.282220351	0.777774555	0.968650729

FTH1P3	15.24649713	0.248313397	0.879966158	0.282185167	0.777801531	0.968650729
PPM1F-AS1	29.12587048	0.103174286	0.365861429	0.282003725	0.777940654	0.968661856
TSPAN33	61.0781899	0.077417241	0.274571712	0.281956361	0.777976972	0.968661856
TPRA1	425.0448895	0.045127531	0.160063065	0.281935944	0.777992627	0.968661856
CXCR6	22.25779692	-0.223106928	0.791468195	-0.281889947	0.778027898	0.968661856
ZNF321P	4.458729644	0.312232368	1.108337226	0.281712425	0.778164026	0.968763654
APRT	796.6881831	-0.03622712	0.128662617	-0.281566791	0.778275707	0.968835005
CTSC	926.8637499	0.055027739	0.195567221	0.281375062	0.778422744	0.968932578
EIF2B4	468.5632051	0.046762254	0.166293951	0.281202377	0.778555181	0.968932578
CNNM3	933.0689164	0.039517733	0.140548509	0.281167931	0.7785816	0.968932578
TMEM44-AS1	139.1127443	0.072420201	0.257594797	0.281139999	0.778603023	0.968932578
SNORA38B	4.696478285	-0.314102113	1.117607946	-0.281048569	0.778673148	0.968932578
FSCN1	8354.806138	0.029380801	0.104568181	0.280972667	0.778731364	0.968932578
HNRNPA2B1	8026.648023	-0.0663999	0.236325225	-0.280968316	0.778734702	0.968932578
SNORD63	11.44740322	0.21122079	0.752066556	0.280853853	0.778822497	0.96897416
TMEM178A	12.80971732	0.164204095	0.584875088	0.280750708	0.778901613	0.969004939
NPTX2	3.121682401	0.377570508	1.345822999	0.2805499	0.779055647	0.969008797
ADAM5	10.9420657	-0.168133493	0.599317843	-0.280541445	0.779062133	0.969008797
FOXO3	1691.388313	-0.058664739	0.209118112	-0.280533996	0.779067847	0.969008797
CHRDL1	11.28262926	0.346001156	1.234292863	0.280323387	0.779229409	0.969107982
MYH8	18.74447763	0.165451432	0.590290266	0.280288261	0.779256356	0.969107982
NOMO2	696.3195861	0.033336277	0.119002	0.280132073	0.779376179	0.969189358
CASKIN2	313.9341349	0.043020011	0.153610665	0.28005875	0.779432431	0.969190207
DNAJC11	678.3499098	0.045158165	0.161285265	0.279989401	0.779485636	0.969190207
HK1	1205.063445	-0.046184735	0.165059093	-0.279807273	0.779625372	0.969194573
TRPC5	6.664948298	0.27101031	0.968637729	0.279785003	0.779642458	0.969194573
SOCS1	119.788751	0.080614441	0.288326325	0.279594453	0.779788663	0.969194573
ZFP82	257.3346136	0.042226568	0.151124846	0.279415124	0.779926266	0.969194573
TMEFF1	35.92932528	0.094194168	0.337120431	0.279408068	0.779931681	0.969194573
CHCHD5	56.61328385	-0.07405208	0.265050595	-0.279388468	0.77994672	0.969194573
HMGN5	59.02075342	-0.073604785	0.263472738	-0.279363949	0.779965534	0.969194573
SLC39A5	7.530450316	0.274364519	0.98246866	0.279260327	0.78004505	0.969194573
SNORD3D	7.398823743	0.263365753	0.943471052	0.279145557	0.780133122	0.969194573
LRRC40	592.2353437	-0.032111712	0.115041866	-0.27913066	0.780144554	0.969194573
GNPNAT1	1446.433028	0.029492725	0.10565934	0.27913032	0.780144815	0.969194573
GLIS2	269.5258849	-0.067955667	0.243541275	-0.279031418	0.780220714	0.969194573
AGPAT3	106.2886053	-0.088339807	0.316625399	-0.279004172	0.780241623	0.969194573
NAPRT	247.8557328	0.076691893	0.274888727	0.2789925	0.78025058	0.969194573
STARD10	65.36163575	0.097671871	0.350296676	0.278826142	0.780378252	0.969285596
SMCR8	1272.376263	-0.031058625	0.111458821	-0.2786556	0.78050914	0.969380601
ARHGEF12	2824.693608	-0.03794987	0.136278239	-0.278473443	0.780648948	0.969420045
SNORD18B	21.00330509	-0.13056619	0.46890198	-0.278450924	0.780666233	0.969420045
TMSB15A	38.36409407	0.107418985	0.385841852	0.278401589	0.7807041	0.969420045
GKAP1	8.664979541	-0.194632953	0.699287765	-0.278330271	0.780758841	0.969420467
QPCT	158.9388949	0.239257818	0.860533134	0.278034405	0.780985948	0.969481096
RUFY2	217.4297714	-0.055487655	0.199671235	-0.277895088	0.781092895	0.969481096
WDCP	598.8533479	-0.033459913	0.120413721	-0.27787459	0.781108631	0.969481096
SAP130	333.4132936	-0.034890846	0.12557693	-0.27784439	0.781131814	0.969481096
CAPN15	1462.44895	0.049965876	0.17988258	0.277769399	0.781189383	0.969481096
NAP1L4	1657.589996	-0.036246698	0.130519813	-0.277710312	0.781234744	0.969481096
TNFRSF10A	976.7754094	0.03260594	0.117411118	0.277707428	0.781236958	0.969481096
CUL1	1062.129915	-0.031354855	0.112909221	-0.277699684	0.781242903	0.969481096
ZNF229	80.04396486	-0.069972382	0.252191605	-0.277457223	0.781429048	0.969509265
GOLPH3L	452.8996331	-0.041633506	0.150106086	-0.277360548	0.781503272	0.969509265
LPCAT2	31.52320655	-0.158435203	0.571591172	-0.277182733	0.781639798	0.969509265
PDE6D	143.2245402	-0.056568917	0.204129125	-0.277123205	0.781685504	0.969509265
CELF6	19.44852156	0.137329307	0.495732985	0.277022735	0.781762649	0.969509265
ESPL1	751.7405961	0.099839698	0.360601277	0.276870062	0.781879881	0.969509265
TMEM92	64.80832952	-0.088102996	0.318292674	-0.276798694	0.781934684	0.969509265
PTGS2	86.48483348	-0.098650319	0.35656132	-0.2766714	0.782032435	0.969509265
SNORA37	6.277617946	-0.29750725	1.07539366	-0.276649622	0.782049158	0.969509265
TP53INP2	106.9072135	-0.08516869	0.307984414	-0.276535715	0.782136632	0.969509265
LOC101927814	2.798719264	0.397159107	1.436271748	0.276520866	0.782148036	0.969509265
LOC100505555	2.172671561	0.484997306	1.754569349	0.276419571	0.782225827	0.969509265
STK11	850.3348504	0.030849626	0.111652222	0.276301048	0.782316851	0.969509265
SDR16C5	37.09236267	-0.338146756	1.224344191	-0.276186025	0.78240519	0.969509265
ITPRIPL1	414.0050944	-0.045750423	0.165758959	-0.276005732	0.782543664	0.969509265
LINC01429	5.557149731	0.247358208	0.896570588	0.275893735	0.782629686	0.969509265
ATP6AP1L	47.20618001	-0.083482884	0.30259427	-0.275890498	0.782632172	0.969509265
PPP1R1B	7.16387109	0.204940121	0.743037407	0.275814002	0.782690928	0.969509265
LOC102724532	4.356148529	-0.253349596	0.919076959	-0.275656563	0.782811861	0.969509265
SMIM8	87.92613266	0.085123488	0.308869965	0.275596523	0.782857981	0.969509265
MRPL1	84.05096558	-0.096611069	0.350553065	-0.275596132	0.782858281	0.969509265
ATP5MF	286.5211363	-0.043343708	0.157276598	-0.27558905	0.782863721	0.969509265
UGDH-AS1	27.06224382	-0.124870378	0.453135191	-0.275569808	0.782878502	0.969509265
FAM229B	171.3983109	0.062089414	0.225318168	0.275563284	0.782883513	0.969509265
BCKDHB	363.4716138	-0.056550067	0.205259665	-0.275505017	0.782928272	0.969509265
MAGI2-AS3	357.8614095	0.041306584	0.149936464	0.275493918	0.782936798	0.969509265
TEDDM1	4.146211716	-0.271144734	0.984501177	-0.275413316	0.782998715	0.969509265
NCOA7-AS1	4.349142157	0.333549535	1.211530799	0.275312468	0.783076186	0.969509265
ANKRD20A4	4.537426129	0.330478694	1.200873652	0.275198555	0.783163697	0.969509265
GLUL	3196.760534	-0.062776848	0.228216367	-0.275076013	0.78325784	0.969509265
ADAMTS4	609.462959	0.075130737	0.27312805	0.275075141	0.783258509	0.969509265
KLHL41	4.283148188	0.261621797	0.951257244	0.27502739	0.783295195	0.969509265
RNVU1-15	15.56998696	0.15334051	0.557729761	0.274936934	0.783364691	0.969509265
ALOX15P1	12.61839939	-0.196573536	0.71499641	-0.274929403	0.783370477	0.969509265
FAHD2CP	12.68108839	-0.279823195	1.018452858	-0.274753213	0.783505845	0.969509265
ZNF585B	402.2807887	0.039524834	0.143856199	0.274752386	0.783506481	0.969509265
LOC284412	3.754375484	0.332955463	1.212350449	0.274636317	0.783595662	0.969509265
FAM185BP	31.3792287	0.166482425	0.606227093	0.274620563	0.783607767	0.969509265

MIR548K	2.87865224	-0.445123647	1.621244619	-0.274556746	0.783656801	0.969509265
SELENBP1	37.06942116	-0.086173433	0.313893994	-0.274530368	0.78367707	0.969509265
FOLH1	14.82044202	0.147312829	0.536633721	0.274512807	0.783690563	0.969509265
LINC02293	13.65931695	0.180642569	0.658138713	0.274474917	0.783719677	0.969509265
FRA10AC1	144.5725313	0.082433019	0.30035302	0.274453771	0.783735926	0.969509265
DDHD2	2368.117477	0.04666597	0.170043021	0.274436255	0.783749385	0.969509265
TARBP2	278.6317646	0.039797077	0.145020574	0.274423662	0.783759061	0.969509265
PDE1B	3.477329582	-0.289033259	1.053363352	-0.274390844	0.783784279	0.969509265
FNBP1L	654.1761641	-0.036867501	0.134505908	-0.274095775	0.784011021	0.969509265
TAGLN2	3661.576006	-0.049126414	0.179275076	-0.27402813	0.784063004	0.969509265
SNORD114-25	4.138071492	0.32265334	1.177839412	0.273936613	0.784133334	0.969509265
CERKL	13.63181549	-0.167322401	0.610811404	-0.273934638	0.784134852	0.969509265
ABCD3	1179.369419	0.028579087	0.10433448	0.273917949	0.784147678	0.969509265
SLC4A3	65.74830859	0.111639679	0.40758685	0.273904026	0.784158378	0.969509265
R3HDM1	515.7784291	0.045514965	0.166220435	0.273822918	0.78422071	0.969509265
RBM17	454.4042522	-0.048453681	0.176973917	-0.273789955	0.784246043	0.969509265
ITPRIP-AS1	11.86833491	-0.190419345	0.695533513	-0.273774507	0.784257916	0.969509265
GNG12-AS1	44.04322993	0.086161373	0.314860304	0.273649525	0.78435397	0.969560749
SOX9-AS1	7.841039835	0.217829975	0.796406867	0.273515942	0.784456639	0.969594928
MIR3173	5.183065342	-0.318520585	1.164728485	-0.273471963	0.784490442	0.969594928
CASP10	33.60952011	-0.084249372	0.308207529	-0.273352738	0.784582079	0.969620939
EIF4EBP2	1689.058238	0.027900778	0.102087346	0.273302998	0.784620311	0.969620939
TCEAL7	3.837027354	-0.278687648	1.020053178	-0.27320894	0.784692608	0.969640598
PRELID1	678.7069336	-0.050702391	0.185669085	-0.273079338	0.78479223	0.969640598
PHKA2-AS1	4.83211062	0.278968509	1.021600969	0.273069934	0.784799458	0.969640598
MIR1206	16.27501239	0.215547503	0.789650348	0.272965755	0.78487954	0.96967231
RCBTB2	42.77673115	-0.091610798	0.335954295	-0.272688278	0.785092846	0.969678316
BSN-DT	3.902975828	0.273253147	1.002610537	0.272541667	0.785205558	0.969678316
NLGN1-AS1	16.37512777	0.245635224	0.901406949	0.27250203	0.78523603	0.969678316
LINC02405	29.24474211	0.116553499	0.427817483	0.272437439	0.785285689	0.969678316
CATSPER2	9.594143011	0.259697898	0.953840514	0.272265535	0.785417855	0.969678316
PTPRK	611.2957171	-0.043973482	0.161582559	-0.272142506	0.785512448	0.969678316
LOC101928978	9.422647084	-0.16788229	0.617194002	-0.272008946	0.785615141	0.969678316
MTO1	442.7397265	-0.038853569	0.142846505	-0.271995236	0.785625683	0.969678316
ADCY6	798.3369623	-0.066405676	0.244258821	-0.271866031	0.785725031	0.969678316
ZFHX4-AS1	59.23709667	0.158806739	0.584195334	0.271838423	0.78574626	0.969678316
STOML1	293.7771599	-0.055471093	0.204158189	-0.271706432	0.785847755	0.969678316
LOC100335030	12.99108411	0.179363571	0.660156219	0.271698676	0.785853719	0.969678316
GSDMC	2.643448332	0.296194081	1.090179707	0.271692896	0.785858164	0.969678316
SNX30	820.3283368	0.082646614	0.304196085	0.271688617	0.785861454	0.969678316
FLOT1	2285.376309	0.066595413	0.245125448	0.271678904	0.785868924	0.969678316
TXNDC16	444.9065595	0.052868555	0.194601494	0.271675999	0.785871158	0.969678316
CCDC157	42.08908709	-0.086298301	0.317689642	-0.271643421	0.78589621	0.969678316
GK	59.68851862	0.09213338	0.339239111	0.271588318	0.785938583	0.969678316
POLE	2739.960491	0.066285253	0.244151109	0.271492738	0.786012085	0.969678316
C11orf87	5.171344836	-0.402842745	1.483923452	-0.271471379	0.78602851	0.969678316
PRAF2	61.26449788	0.126068655	0.464540737	0.271383423	0.786096151	0.969678316
POC1B-AS1	25.38331306	-0.125254261	0.461591829	-0.271352857	0.786119657	0.969678316
FAM204A	344.9465422	-0.034485093	0.127095718	-0.27133167	0.78613595	0.969678316
NUDT16	306.7337488	-0.045928483	0.169473246	-0.271007276	0.786385436	0.969793669
CSMD2	2.617678299	-0.324695664	1.198237024	-0.270977826	0.786408087	0.969793669
LOC647070	18.18056734	-0.1614973	0.596145999	-0.270902262	0.786466205	0.969793669
TLCD3B	3.087915612	0.335711105	1.239701628	0.270799922	0.78654492	0.969793669
RCC1	1121.460457	0.034350516	0.126853415	0.270789057	0.786553277	0.969793669
UBE2J2	568.2320711	0.03280091	0.121158853	0.270726478	0.78660141	0.969793669
ZSCAN12P1	9.891149331	0.187293533	0.69184824	0.270714764	0.786610421	0.969793669
USP54	954.8652139	-0.051911356	0.19184595	-0.270588749	0.78670735	0.969846073
CKS2	241.0202855	0.054263353	0.200685913	0.270389448	0.786860657	0.969967965
TOMM40	1343.700229	-0.052900504	0.195839335	-0.270121957	0.78706643	0.970014668
MR1	703.1382357	0.077575687	0.287368423	0.269952024	0.787197163	0.970014668
GLIPR1L1	9.074048066	-0.245127901	0.90813011	-0.26992597	0.787217207	0.970014668
IRAK3	231.4141393	0.053708221	0.198974843	0.269924682	0.787218198	0.970014668
LOC100505915	19.99161378	0.180167435	0.66769779	0.269833805	0.787288114	0.970014668
NUDT1	175.3897604	0.046140298	0.17099629	0.269832158	0.787289382	0.970014668
TAS2R19	20.00248249	0.245582374	0.910151259	0.26982589	0.787294204	0.970014668
ARMC7	132.2284598	-0.052569888	0.194956654	-0.269649107	0.787430217	0.970014668
CDK11B	233.1569029	-0.04785683	0.177484066	-0.269640146	0.787437112	0.970014668
ITGB3BP	300.1683457	-0.035910811	0.133203214	-0.269594181	0.787472477	0.970014668
GATA2-AS1	56.72028209	-0.110232084	0.408996018	-0.269518722	0.787530537	0.970014668
ATAD3A	762.6262585	-0.05801197	0.215319132	-0.269423201	0.787604034	0.970014668
SRSF11	2278.457041	0.052792901	0.195978612	0.269380934	0.787636556	0.970014668
LOC103908605	8.17274257	0.188151264	0.69853908	0.269349661	0.78766062	0.970014668
PFKFB2	432.2134503	0.059912527	0.222724482	0.268998389	0.787930923	0.970280495
ZNF662	117.1205246	-0.0715813	0.266341323	-0.268757772	0.788116091	0.970441456
LINC01220	5.761653799	0.24868115	0.925995316	0.268555516	0.788271748	0.970560586
LINCR-0002	48.91726873	-0.097848357	0.364541974	-0.268414515	0.788380268	0.970560586
MIR1843	2.294706135	-0.379807498	1.4155263	-0.268315395	0.788456557	0.970560586
GBP2	356.4564391	-0.094855581	0.353524852	-0.268313756	0.788457818	0.970560586
LDB3	4.952726764	-0.238909831	0.890928369	-0.268158294	0.788577476	0.970560586
C1orf198	604.1685114	0.036668648	0.136749924	0.268143826	0.788588612	0.970560586
ANAPC4	296.0080245	-0.075062487	0.279941109	-0.268136706	0.788594092	0.970560586
LEF1	252.7971892	-0.063370016	0.236537347	-0.26790702	0.78877089	0.970598325
WASH5P	3.395520364	0.334323878	1.248357539	0.267810998	0.788844805	0.970598325
HYLS1	102.9524691	0.064076329	0.239261755	0.267808488	0.788846737	0.970598325
LINC00271	4.028736436	-0.254347995	0.950088044	-0.26770992	0.788922613	0.970598325
FAM193B	541.9808488	0.074543601	0.278496471	0.267664436	0.788957627	0.970598325
FDFT1	4491.827211	0.068345709	0.255438295	0.267562501	0.789036099	0.970598325
ERN1	168.079204	0.061162416	0.22872218	0.267409205	0.789154113	0.970598325
RSPH4A	10.59422702	0.23686882	0.885860858	0.26738829	0.789170215	0.970598325
EID2B	59.45469848	-0.091480102	0.342262693	-0.267280379	0.789253292	0.970598325

LINC00638	56.05008451	-0.081486975	0.304878795	-0.267276623	0.789256185	0.970598325
DND1	205.6973306	-0.045447292	0.170044008	-0.267267825	0.789262958	0.970598325
NCBP2	2757.222221	-0.030618554	0.11456989	-0.267247823	0.789278357	0.970598325
FPGT-TNNI3K	9.061009477	0.191610599	0.71720777	0.267161912	0.789344501	0.970612683
CDH4	8.143045022	0.199954223	0.748840536	0.267018428	0.789454972	0.97064335
SCAMP1	1034.826169	0.04465092	0.167239411	0.266988028	0.789478379	0.97064335
TMPO-AS1	126.7287653	-0.058461107	0.219096699	-0.266827875	0.789601691	0.970661265
DNM3OS	2890.820196	0.092194271	0.345519976	0.266827615	0.789601891	0.970661265
ERICH6	5.63375113	0.261579649	0.981008474	0.266643618	0.789743569	0.970720985
GNRHR	15.32898146	-0.202671426	0.760142224	-0.266623034	0.789759419	0.970720985
MIR301B	3.216077589	0.366967181	1.376807631	0.266534825	0.789827343	0.970737516
LINC00921	15.12104673	0.194623561	0.731370414	0.266108059	0.790155989	0.971052568
LOC729970	32.63511146	0.129194946	0.485653145	0.266023082	0.790221432	0.971052568
LOC112268140	9.628866259	0.203274348	0.764218929	0.26598968	0.790247157	0.971052568
NUDT8	21.96211359	-0.12427654	0.467718913	-0.26570775	0.790464294	0.971102478
CNDP2	968.4780694	0.040477663	0.152341156	0.265704056	0.79046714	0.971102478
LIPA	2519.468213	0.051693754	0.19459662	0.265645695	0.79051209	0.971102478
HARS2	522.1800275	0.030177411	0.113673482	0.265474502	0.79064395	0.971102478
MAP4K3-DT	72.13308661	0.101033985	0.380586801	0.265468968	0.790648212	0.971102478
MIR760	3.189544025	-0.38425431	1.44785336	-0.265395875	0.790704513	0.971102478
CLUL1	2.378234851	-0.356046432	1.342130016	-0.265284606	0.790790223	0.971102478
SAR1A	1725.977484	-0.038510143	0.145167519	-0.26528071	0.790793223	0.971102478
TMEM187	69.51441686	-0.123152303	0.464281791	-0.265253355	0.790814295	0.971102478
KCTD14	2.485561415	-0.278436433	1.050130967	-0.265144484	0.79089816	0.971102478
MIR2116	2.870700341	0.361523608	1.363569703	0.265130273	0.790909107	0.971102478
LOC102546294	6.474428814	0.204250501	0.770500908	0.265087943	0.790941715	0.971102478
HOOK2	162.6860779	-0.05466009	0.20627508	-0.264986396	0.791019942	0.971113562
LUZP2	6.173202746	-0.238089165	0.898696464	-0.26492723	0.791065521	0.971113562
LMO3	38.36913119	-0.119720355	0.45209451	-0.264812672	0.791153774	0.971113562
DET1	211.5732724	-0.040326517	0.15238053	-0.264643499	0.791284107	0.971113562
ZNF691	119.5906441	0.056388902	0.213080794	0.264636249	0.791289693	0.971113562
ALG14	77.70032186	0.059177866	0.223692369	0.26455022	0.791355972	0.971113562
ARAP3	107.7048624	-0.057559623	0.21758325	-0.264540692	0.791363314	0.971113562
PLA2G15	386.257152	-0.04624554	0.174834535	-0.264510326	0.791386709	0.971113562
LINC02323	30.98398308	-0.135503271	0.512465091	-0.264414638	0.791460433	0.971120732
ZNF134	327.2275196	-0.046771545	0.176922826	-0.264361281	0.791501544	0.971120732
ARHGEF39	265.6411901	0.053484467	0.202555325	0.264048683	0.791742406	0.971290629
PMPCA	1152.310809	-0.036635985	0.138793077	-0.263961184	0.791809829	0.971290629
ELL	144.0708999	-0.053939889	0.204408921	-0.263882265	0.791870642	0.971290629
DPF3	38.7054109	0.116909892	0.443293848	0.263730012	0.791987968	0.971290629
NOTCH2NLR	10.66833428	0.156857726	0.594782947	0.263722635	0.791993652	0.971290629
RBM14	883.4299431	0.075104142	0.284823211	0.263686873	0.792021211	0.971290629
PLCL2	174.0798822	-0.042127936	0.159841587	-0.263560545	0.792118564	0.971290629
INSYN1	7.599000006	0.182287985	0.691659735	0.263551536	0.792125507	0.971290629
KCTD9	963.2237519	0.029595119	0.11232386	0.263480249	0.792180445	0.971290629
HJURP	535.4987324	0.040578286	0.154039127	0.263428436	0.792220375	0.971290629
MIR320B2	3.970596283	0.277571745	1.054169567	0.263308441	0.792312854	0.971290629
MRPL3	1014.283032	0.031413146	0.119319218	0.263269797	0.792342637	0.971290629
AFF2	347.6023965	-0.054897302	0.208535774	-0.263251243	0.792356937	0.971290629
NCBP2AS2	241.3129566	0.053700124	0.204034529	0.263191353	0.792403096	0.971290629
CRAMP1	577.1947049	0.048765617	0.185359293	0.263086985	0.792483536	0.971322416
CYP39A1	116.8248419	-0.072043883	0.273921591	-0.263009143	0.792543532	0.971329143
EOLA2	149.5774154	-0.059288462	0.225543108	-0.262869757	0.792650967	0.971394005
SEMA7A	570.8288404	-0.067378625	0.256435988	-0.262750269	0.792743068	0.971440069
IRF2BPL	1573.091997	-0.057488171	0.218961263	-0.262549504	0.792897825	0.971460835
MAP6	3.041749825	0.296847412	1.131657367	0.262312092	0.79308084	0.971460835
KAT6A	2673.625727	0.030344521	0.115685556	0.262301722	0.793088835	0.971460835
DKK3	453.0153974	0.097602979	0.372378728	0.262106753	0.793239141	0.971460835
CRYM	3.967167222	0.3364951	1.28407161	0.26205322	0.793280412	0.971460835
LINC00449	15.33952607	0.134130613	0.511958448	0.261995115	0.793325208	0.971460835
ATP9B	319.8843643	-0.061697025	0.235611873	-0.261858727	0.793430361	0.971460835
RRP9	316.8649467	-0.058090281	0.221886402	-0.261801899	0.793474175	0.971460835
GLA	309.3630483	0.040304335	0.153997803	0.261720194	0.79353717	0.971460835
EFHD2	837.0533984	-0.054357387	0.207702961	-0.261707328	0.793547091	0.971460835
SNORA24	5.29832945	-0.248375343	0.949112421	-0.261692227	0.793558733	0.971460835
KL	53.87752719	-0.114235738	0.436568752	-0.261667235	0.793578003	0.971460835
MIR5009	3.062644539	0.310778873	1.189014907	0.261375086	0.793803268	0.971460835
LINC00161	3.400883298	0.33244661	1.272523241	0.261249932	0.793899775	0.971460835
LSAMP	14.20236119	0.200908133	0.769053911	0.261240636	0.793906943	0.971460835
USP28	548.3843296	0.043450426	0.166370694	0.261166348	0.793964228	0.971460835
RRH	2.705580564	0.358643953	1.373420868	0.261131865	0.793990819	0.971460835
CXADR	55.35294898	0.07987855	0.305912243	0.261115899	0.794003132	0.971460835
IFT74	86.8954375	-0.062701382	0.240196084	-0.261042481	0.794059748	0.971460835
PKIG	130.8175085	0.218474612	0.837293683	0.260929488	0.794146884	0.971460835
PTGES3L	3.961623068	0.268784722	1.030471552	0.260836625	0.794218499	0.971460835
DPYD-AS2	4.970961449	0.327986058	1.257707279	0.260780917	0.794261461	0.971460835
UBL5	359.4287631	0.034777742	0.133417804	0.260667924	0.794348604	0.971460835
SNTB1	967.9449431	0.040534051	0.155522052	0.260632178	0.794376172	0.971460835
GSTZ1	266.5667786	-0.066277851	0.254383617	-0.260542923	0.794445011	0.971460835
FTCD-AS1	4.588799145	0.267680786	1.02747185	0.260523718	0.794459823	0.971460835
PPP4C	909.9751824	-0.031620883	0.121386659	-0.260497184	0.794480287	0.971460835
USP21	495.5980867	0.03821383	0.14669619	0.260496402	0.79448089	0.971460835
NDUFB3	309.7354583	-0.042708078	0.163958983	-0.260480259	0.794493341	0.971460835
RTL1	152.0295641	0.205109963	0.787604069	0.26042268	0.79453775	0.971460835
ZMYND10	11.95324331	0.14087587	0.541129908	0.260336506	0.794604215	0.971460835
SLC6A4	4.300520275	0.218121062	0.83800598	0.260285807	0.79464332	0.971460835
TRIM34	3.395470281	0.368680725	1.416570511	0.260262883	0.794661001	0.971460835
LIMS1-AS1	3.417687188	-0.302697228	1.163680516	-0.26012056	0.794770779	0.971460835
UTP18	798.7033346	-0.026276328	0.101026257	-0.260094043	0.794791233	0.971460835
KCNE4	15.82828693	0.139524867	0.536457967	0.260085367	0.794797925	0.971460835

CD38	3.722098967	-0.271669949	1.044588287	-0.260073708	0.794806918	0.971460835
ALKBH8	180.9186719	0.060499582	0.232679956	0.260012005	0.794854513	0.971460835
MIR1249	3.467103115	0.336630785	1.295215179	0.259903366	0.794938315	0.971460835
LOC101927322	2.376961567	-0.332062549	1.277653213	-0.259900375	0.794940622	0.971460835
SNORD74B	3.973374625	0.388911689	1.496812334	0.25982662	0.794997516	0.971463742
MIR619	4.642917292	0.334359841	1.287700655	0.259656497	0.795128752	0.971557485
CASC3	1680.256178	-0.033239742	0.128083147	-0.259516903	0.795236442	0.971562829
FCHSD2	398.3437526	0.037262128	0.143614187	0.259459938	0.795280388	0.971562829
TCEAL8	255.8514419	0.058839621	0.226921893	0.259294598	0.795407947	0.971562829
ZNF274	429.4101073	0.036049603	0.13906332	0.259231571	0.795456573	0.971562829
C19orf25	190.0102008	0.091503858	0.352982895	0.25923029	0.795457562	0.971562829
MICOS10P1	8.394606155	-0.273353859	1.054496893	-0.259226804	0.795460251	0.971562829
HMGB3	856.8231057	-0.026901012	0.10384953	-0.259038364	0.79560564	0.971607859
LINC00242	6.866740591	-0.222204024	0.857805792	-0.259037683	0.795606166	0.971607859
LINC00673	40.88785378	0.080879625	0.312475307	0.258835252	0.795762357	0.971650109
DCAF15	346.3849305	0.049740995	0.192236837	0.258748509	0.795829289	0.971650109
EMILIN3	16.10142004	0.119063449	0.460189187	0.258727177	0.795845749	0.971650109
PAK1IP1	412.2795306	-0.045718878	0.176797791	-0.258594173	0.79594838	0.971650109
NUTM2A-AS1	95.09093706	0.056677323	0.219199542	0.258564971	0.795970913	0.971650109
FAM221B	3.443976465	0.386776733	1.496071499	0.258528241	0.795999256	0.971650109
POU3F3	9.662074252	-0.182022201	0.704309193	-0.258440757	0.796066765	0.971650109
COL5A3	241.6293114	-0.172357967	0.666948928	-0.258427534	0.796076969	0.971650109
MMP15	7.640082339	0.208244441	0.807897176	0.25776107	0.796591311	0.972198276
PTCH2	16.19937353	0.129613078	0.502952897	0.257704209	0.796635198	0.972198276
LOC90246	20.86264067	0.181386348	0.704114241	0.257609259	0.796708483	0.97222113
TSPAN13	492.4482724	0.057286804	0.222475108	0.257497588	0.796794677	0.972259733
NPAS2-AS1	5.301148188	-0.283139604	1.10065007	-0.257247614	0.79698763	0.972382204
APMAP	1906.263376	-0.028694254	0.11156114	-0.257206532	0.797019342	0.972382204
SMIM13	861.0547902	-0.032825154	0.127675701	-0.257097894	0.797103203	0.972382204
C12orf76	136.220355	0.064235085	0.249901651	0.257041461	0.797146767	0.972382204
MIER1	793.1398039	0.031393344	0.12215922	0.256987102	0.79718873	0.972382204
CCNK	232.7068719	0.048472176	0.188668293	0.256917444	0.797242504	0.972382204
ZNHIT2	69.41068573	-0.063619591	0.247725487	-0.256814879	0.797321683	0.972382204
LINC00535	5.052140477	0.233237257	0.908237489	0.256802059	0.79733158	0.972382204
FAM85A	11.96180327	0.162741859	0.63409767	0.256651091	0.797448131	0.972411146
FAM66B	43.82096087	0.085722944	0.334033274	0.256629954	0.797464449	0.972411146
TECPR2	939.2444777	-0.042186196	0.1645867	-0.256315949	0.797706883	0.972496413
TAB3	356.1874948	0.044432117	0.173367696	0.256288328	0.797728209	0.972496413
FAM30C	3.528254728	-0.25117671	0.980232483	-0.256241978	0.797763997	0.972496413
ZNRD2-AS1	8.221315407	0.244750544	0.955233335	0.25622069	0.797780433	0.972496413
LOC100126584	3.026761749	0.360889837	1.408874494	0.25615471	0.797831379	0.972496413
NR1I3	30.14656581	0.12688086	0.495405253	0.25611529	0.797861816	0.972496413
HDHD5	526.9281346	-0.041417957	0.161790639	-0.255997238	0.79795297	0.972540998
C3orf62	87.56543463	-0.079002752	0.308730079	-0.25589587	0.798031244	0.972546949
PASD1	2.222390426	-0.388452272	1.519561926	-0.255634381	0.798233168	0.972546949
LOC145783	92.61493391	-0.06261008	0.244958545	-0.255594592	0.798263894	0.972546949
HSD11B1L	74.16442466	-0.085707446	0.335333011	-0.255589051	0.798268174	0.972546949
CBWD2	202.6815698	0.05462131	0.2137853	0.2554961	0.798339955	0.972546949
TMEM219	460.2775699	0.051586303	0.201948321	0.25544309	0.798380893	0.972546949
NAP1L5	107.0732482	0.057996936	0.227056403	0.255429643	0.798391278	0.972546949
SPATS1	2.745566152	0.308158026	1.206812233	0.255348776	0.798453731	0.972546949
PIGM	354.7783235	-0.042025635	0.164590464	-0.255334565	0.798464706	0.972546949
LINC00923	3.891441199	-0.283880658	1.112527809	-0.255167247	0.798593927	0.972546949
AIFM2	122.0010264	0.064158868	0.251476	0.255129189	0.79862332	0.972546949
SORBS1	7.387208731	0.168669479	0.661236336	0.255081988	0.798659776	0.972546949
ETNK2	59.71574006	0.072761208	0.285391126	0.254952593	0.798759714	0.972546949
ANO10	267.5108339	-0.050488999	0.198169525	-0.254776807	0.79889549	0.972546949
RHPN2	35.15064702	-0.11270318	0.442370503	-0.25477101	0.798899967	0.972546949
CCDC61	295.9337571	-0.036093605	0.14169134	-0.254734024	0.798928535	0.972546949
SNAI2	3987.847694	0.054759072	0.214999752	0.254693652	0.79895972	0.972546949
MUC1	14.14039682	-0.146571899	0.575883457	-0.254516599	0.799096482	0.972546949
CDADC1	85.75071249	0.061375848	0.24114918	0.254514024	0.799098471	0.972546949
LOC101928126	12.5830042	0.175055572	0.687986486	0.254446236	0.799150835	0.972546949
GPRASP1	51.63619793	-0.131450476	0.516702656	-0.254402555	0.799184577	0.972546949
MIR370	2.770197425	-0.28173114	1.107512609	-0.25438188	0.799200548	0.972546949
BAX	333.8310458	-0.036577489	0.14379887	-0.254365626	0.799213105	0.972546949
SRC	365.1716803	-0.037473807	0.147390147	-0.254249064	0.799303148	0.972590106
ADI1	398.1142195	0.03376367	0.132888009	0.254076123	0.799436749	0.972647204
RFX2	187.2974872	-0.041029715	0.16154034	-0.253990522	0.79950288	0.972647204
LINC00634	8.673416027	-0.176748056	0.696412202	-0.253798046	0.799651583	0.972647204
GPR132	2.595360553	0.291137441	1.148023416	0.25359887	0.79980547	0.972647204
NISCH	1164.93774	-0.036499526	0.143971264	-0.253519522	0.799866778	0.972647204
GSG1	7.322547549	0.219587226	0.866250858	0.253491495	0.799888432	0.972647204
RARA	296.1422199	-0.04933572	0.194645197	-0.253464873	0.799909002	0.972647204
LEMD1	3.145678416	-0.387642229	1.529424479	-0.253456274	0.799915647	0.972647204
SNRK	175.8458944	-0.04617946	0.182250968	-0.253383894	0.799971572	0.972647204
MRPS27	1751.129057	-0.039997418	0.157854161	-0.253382096	0.799972962	0.972647204
SLC38A7	260.4291642	-0.041767485	0.164926777	-0.253248662	0.800076065	0.972647204
CCDC146	18.43227867	0.126133265	0.498184149	0.253186026	0.800124465	0.972647204
TMEM74B	5.280256131	-0.248368568	0.981572827	-0.253031218	0.80024409	0.972647204
NBPF1	502.9887958	0.073913767	0.292122943	0.253022805	0.800250591	0.972647204
HOXC-AS1	13.03772998	0.166453235	0.658069049	0.252941899	0.800313111	0.972647204
POU2F2	13.64663969	-0.158721867	0.627628514	-0.252891421	0.800352119	0.972647204
PARP2	483.1874141	-0.032289628	0.127682756	-0.252889495	0.800353608	0.972647204
MAP3K15	11.54809473	-0.168457881	0.666191514	-0.252867047	0.800370955	0.972647204
SNORD8	6.386206814	0.246478308	0.974815466	0.25284612	0.800387127	0.972647204
CYP2W1	4.383835841	-0.243680079	0.964507581	-0.252647138	0.800540901	0.972659016
ACTN1-AS1	3.761903361	0.263977891	1.045268731	0.252545478	0.800619467	0.972659016
EI24	1215.60158	0.028387084	0.112408084	0.252535969	0.800626815	0.972659016
DNAJC16	898.7745053	0.033734538	0.133623747	0.252459152	0.800686183	0.972659016

GALNT15	118.2944766	0.278582433	1.103527592	0.252447184	0.800695433	0.972659016
NBEA	295.0614083	-0.046707901	0.185047905	-0.252409781	0.800724341	0.972659016
ZNF587B	267.149966	0.090931357	0.360362602	0.252332945	0.800783725	0.972664495
EXOSC9	290.6273736	0.056060431	0.222263909	0.252224626	0.800867444	0.972664495
LOC101927572	20.21725124	0.113109411	0.448692138	0.252086901	0.800973894	0.972664495
HES1	238.7322917	-0.074677328	0.296285576	-0.252045101	0.801006203	0.972664495
SP140L	250.2372714	-0.046305778	0.184047367	-0.251597066	0.801352526	0.972664495
LDHAL6B	11.83975208	-0.205393326	0.816473283	-0.2515616	0.801379943	0.972664495
FAM13B	378.2277468	-0.084753917	0.336920928	-0.251554327	0.801385565	0.972664495
CENPK	204.5512807	0.055069584	0.218929727	0.251540001	0.80139664	0.972664495
ZSCAN18	79.8751798	-0.1226231	0.487562114	-0.251502519	0.801425615	0.972664495
ZNF81	262.1735943	-0.040854267	0.162446402	-0.251493823	0.801432337	0.972664495
MAP9	335.4792766	0.038946051	0.154909845	0.251411076	0.801496305	0.972664495
NCK1-DT	33.92847121	0.099443735	0.395609857	0.251368193	0.801529457	0.972664495
TGIF2-RAB5IF	3.560943467	-0.269678764	1.072853896	-0.251365787	0.801531317	0.972664495
LOC730202	12.97623358	-0.156288504	0.6218644	-0.251322481	0.801564795	0.972664495
MIR1245A	4.542429103	0.274928771	1.094600591	0.251168118	0.801684132	0.972664495
ZNF720	60.8863852	0.061299412	0.244111585	0.251112261	0.801727317	0.972664495
LOC101927989	38.90315454	0.092273947	0.367539691	0.251058454	0.801768916	0.972664495
TMEM198	48.8932238	0.091659181	0.365095187	0.251055573	0.801771144	0.972664495
IDH1-AS1	3.384457939	0.27966091	1.114540102	0.250920455	0.801875609	0.972664495
SDCBP2-AS1	101.8024935	-0.101026404	0.402674976	-0.250888211	0.801900539	0.972664495
SNORA63	10.37024503	-0.158666356	0.632491234	-0.250859375	0.801922834	0.972664495
GALNT2	2681.09584	-0.029175787	0.116320787	-0.250821782	0.8019519	0.972664495
RAE1	458.0183555	-0.043676179	0.174179864	-0.250753317	0.802004836	0.972664495
LINC00847	32.04479161	0.093486299	0.372929074	0.250681177	0.802060615	0.972664495
ALOX12P2	32.76987937	0.149923888	0.598405719	0.250538863	0.802170655	0.972664495
SNORD100	21.90960349	-0.126502191	0.50499746	-0.250500648	0.802200204	0.972664495
NKX2-4	185.5639998	0.041763698	0.16677133	0.250424928	0.802258754	0.972664495
MIR4451	3.823561672	0.287248394	1.147315079	0.250365744	0.802304518	0.972664495
ATP9A	1646.713991	0.059957783	0.239489725	0.25035639	0.802311752	0.972664495
CORO1C	6754.908995	0.028814087	0.115130859	0.250272492	0.802376628	0.972676973
POR	418.2190049	-0.030932476	0.123637445	-0.250186957	0.802442772	0.972690986
LCP1	1100.532042	-0.060083968	0.240280642	-0.250057465	0.802542909	0.972732997
WDR5	1671.195948	-0.033918615	0.135673938	-0.250000965	0.802586602	0.972732997
SNORD46	91.36676757	0.108681852	0.434860494	0.24992349	0.802646517	0.972739454
AGMO	61.95306297	-0.086375913	0.345792171	-0.249791406	0.802748666	0.972797091
LOC101929710	4.561798878	0.266749992	1.068564078	0.249634063	0.802870354	0.972878397
IGFBP6	62.72219128	0.075381957	0.302259997	0.249394421	0.8030557	0.97299151
IFIT1B	2.547088233	0.318676971	1.279318029	0.249099101	0.803284125	0.97299151
PRR25	9.034074436	0.234205191	0.940376142	0.249054799	0.803318393	0.97299151
HIBADH	610.8454392	-0.043898269	0.176349993	-0.248926969	0.803417273	0.97299151
B3GAT3	513.6273513	0.036717402	0.147505186	0.24892279	0.803420507	0.97299151
CARS2	513.4328906	0.055288388	0.222124355	0.248907366	0.803432437	0.97299151
MIR4513	6.796291317	0.255530925	1.02696246	0.24882207	0.803498418	0.97299151
ENTPD1-AS1	28.55122298	0.096172613	0.386590929	0.248771004	0.803537922	0.97299151
SLC4A10	4.800806605	0.251135487	1.009721046	0.248717691	0.803579163	0.97299151
LINC01117	34.70455904	-0.093625045	0.376439415	-0.248712121	0.803583472	0.97299151
RBM41	197.1097245	-0.048820857	0.196317075	-0.248683703	0.803605456	0.97299151
EDNRA	99.02785448	0.057654628	0.231855407	0.248666306	0.803618914	0.97299151
ZNF44	78.56136284	0.064217738	0.25843325	0.248488684	0.803756324	0.973079253
ARIH2	1386.527586	-0.030041604	0.120925121	-0.24843146	0.803800595	0.973079253
PLAG1	1236.999224	0.031817323	0.12829963	0.247992321	0.804140349	0.97339701
LOC101928896	4.191118864	0.426094446	1.718462328	0.247950996	0.804172322	0.97339701
ZNF215	166.8411983	0.08660186	0.349634513	0.247692538	0.804372306	0.973520236
PRR7	466.2602071	-0.040477467	0.163427644	-0.247678218	0.804383387	0.973520236
AMFR	1831.082436	-0.030344239	0.12266463	-0.247375625	0.804617537	0.973671813
GPR137B	96.76858642	0.092533068	0.374109561	0.247342164	0.80464343	0.973671813
PI4KAP1	55.62770573	0.092752338	0.375153823	0.247238152	0.804723921	0.973671813
MORN3	7.930715759	-0.228865631	0.925704774	-0.247233932	0.804727186	0.973671813
C4orf36	18.83892391	0.120497034	0.487599118	0.247123158	0.804812912	0.973709423
RETREG3	1110.16212	-0.049153262	0.199251834	-0.246689134	0.805148817	0.973753011
RXYLT1-AS1	3.030576438	0.26459058	1.072750569	0.246646879	0.805181521	0.973753011
SNORD18C	4.364314989	0.259082159	1.050594661	0.24660525	0.805213741	0.973753011
CERS2	2257.205005	-0.032117498	0.130249886	-0.246583695	0.805230424	0.973753011
ZNF547	23.23785777	0.130781061	0.530725472	0.246419416	0.805357578	0.973753011
C11orf45	23.88978131	0.102862523	0.417439341	0.246413101	0.805362466	0.973753011
LARP4B	1478.337159	-0.034081096	0.138312055	-0.246407274	0.805366976	0.973753011
DNAJC17	214.7972774	-0.071284318	0.289423465	-0.246297645	0.805451833	0.973753011
CAPZB	2395.935816	-0.043893069	0.178255632	-0.246236646	0.805499049	0.973753011
FAM117A	139.0671605	0.060773453	0.246854933	0.246190959	0.805534414	0.973753011
NIPA1	64.43147933	0.086092712	0.349813941	0.246110007	0.805597077	0.973753011
ZFAND2A	155.7911663	-0.053819599	0.218763748	-0.246016991	0.80566908	0.973753011
VEZF1	1137.800313	-0.03289946	0.133783198	-0.245916229	0.80574708	0.973753011
LINC00926	17.57299607	0.166200742	0.675905891	0.245893317	0.805764817	0.973753011
ZFP1	368.1507428	0.037500715	0.152517635	0.245877895	0.805776756	0.973753011
PAGE5	16.43443292	0.161598801	0.657267651	0.245864528	0.805787103	0.973753011
TXNDC12-AS1	7.046411059	0.175708333	0.714772159	0.245824254	0.805818281	0.973753011
DDX52	1077.409957	0.030874872	0.125642077	0.245736719	0.805886046	0.973753011
NUP37	659.6695085	0.029234002	0.119000408	0.245663039	0.805943086	0.973753011
SNORA49	4.503143328	-0.252087315	1.026361535	-0.24561259	0.805982142	0.973753011
GTF2IP23	31.23595979	0.135809957	0.55299375	0.245590402	0.80599932	0.973753011
DLX3	2.765538114	0.34092133	1.388548593	0.245523514	0.806051103	0.973753011
FOXJ3	1484.593594	-0.026513354	0.108022822	-0.245442154	0.806114093	0.973763092
SNORD114-9	2.742428529	0.308066412	1.256368913	0.245203784	0.806298646	0.973820399
OR6C2	2.453781283	-0.28277708	1.153546561	-0.245137119	0.806350263	0.973820399
FAM217B	394.7590063	0.046452489	0.189523359	0.245101653	0.806377723	0.973820399
CNNM3-DT	8.443454997	0.186590507	0.761287676	0.245098552	0.806380124	0.973820399
ANKRD2	9.759427622	-0.184798482	0.754919549	-0.244792286	0.806617267	0.974040774
PPP1R2	894.3169526	0.045325779	0.185271337	0.244645392	0.806731014	0.974064937

LINC00882	3.299806926	-0.283545007	1.159099436	-0.244625265	0.8067466	0.974064937
GINS4	945.2788773	-0.054706436	0.223756588	-0.244490837	0.806850698	0.974124623
RBKS	84.89415124	-0.066098901	0.270579245	-0.244286663	0.807008812	0.974163083
OFD1	468.4522772	-0.036882815	0.151048273	-0.244178989	0.807092199	0.974163083
ANKRA2	133.9665134	-0.056072426	0.229698664	-0.244112982	0.807143318	0.974163083
SCLT1	232.0139976	-0.044657579	0.182982447	-0.244053896	0.807189079	0.974163083
ZNF677	9.347432619	0.216984256	0.889101921	0.244048799	0.807193026	0.974163083
SNORA70G	8.971051778	-0.217985405	0.89328701	-0.244026167	0.807210554	0.974163083
MECOM-AS1	7.36635993	0.237516706	0.974248488	0.24379479	0.807389756	0.974313364
DSG2	5069.109368	-0.026099697	0.107145751	-0.243590597	0.807547911	0.97443823
RFESD	41.91317493	0.075058827	0.308461407	0.243332959	0.807747474	0.974569581
CBR3	33.89844737	-0.090746449	0.372968134	-0.243308855	0.807766145	0.974569581
NXF1	390.4169291	-0.049632047	0.204151001	-0.243114396	0.807916779	0.974685329
MMP24-AS1-EDEM2	11.63152862	0.150188086	0.618171197	0.24295549	0.808039878	0.974767847
SNHG20	55.87891711	-0.104057219	0.428510579	-0.242834656	0.808133487	0.97481478
EEF1E1	228.172244	-0.036159528	0.149023679	-0.242642835	0.808282095	0.974882383
RPL27A	1803.245113	-0.0506718	0.208895043	-0.242570619	0.808338044	0.974882383
TYW1	310.6686361	0.036540555	0.150724802	0.242432262	0.808445238	0.974882383
LOC441242	48.56370937	-0.088884951	0.36674498	-0.242361738	0.808499879	0.974882383
COMMD5	218.7018169	0.054849998	0.226344415	0.242329805	0.808524621	0.974882383
SMAD5	2388.686123	-0.02601774	0.107385528	-0.242283484	0.80856051	0.974882383
LOC101929140	10.16446102	-0.146364672	0.604143558	-0.242268033	0.808572482	0.974882383
DEDD2	190.9945825	-0.046843866	0.193428233	-0.242176985	0.808643028	0.974901479
MPRIP	6965.566549	0.04220725	0.174507939	0.241864356	0.80888527	0.974905293
TTC3-AS1	8.859270578	-0.196926462	0.814242607	-0.241852319	0.808894598	0.974905293
TRAPPC3	570.2528667	-0.041732126	0.17258856	-0.241801231	0.808934185	0.974905293
GTF2H5	144.8007029	-0.056734576	0.234760706	-0.241669813	0.809036022	0.974905293
ZNF879	40.35906526	0.075450095	0.312233064	0.241646717	0.80905392	0.974905293
ARHGEF37	2.081828751	-0.348738816	1.443216658	-0.241639961	0.809059155	0.974905293
MST1P2	32.06806177	-0.102180338	0.423281411	-0.241400484	0.809244737	0.974905293
GPR107	2586.757157	0.036765877	0.152323176	0.241367585	0.809270233	0.974905293
PRSS8	3.77462391	0.290858148	1.205053499	0.241365341	0.809271973	0.974905293
GSTCD-AS1	3.226863534	-0.305015784	1.264500585	-0.241214427	0.80938893	0.974905293
SNHG7	629.5629776	-0.051791007	0.214748748	-0.24117024	0.809423175	0.974905293
FKTN	1406.402726	0.031446833	0.130444822	0.241073833	0.809497893	0.974905293
ZFYVE26	1358.071794	0.029511376	0.12241779	0.241070977	0.809500106	0.974905293
OXGR1	13.12660358	0.145379848	0.603094719	0.24105641	0.809511397	0.974905293
CHID1	1039.546203	0.026196913	0.108680571	0.241045041	0.809520208	0.974905293
ASPDH	8.896113397	0.21579372	0.895248636	0.241043338	0.809521528	0.974905293
SHISAL2B	4.967196815	-0.196513635	0.816197795	-0.240767172	0.809735574	0.975038938
GBAP1	84.71003329	0.079806002	0.331476758	0.240758968	0.809741933	0.975038938
CCP110	647.8334358	-0.032211421	0.133883106	-0.24059362	0.809870096	0.975068072
EPN2-IT1	11.3098919	0.217924563	0.905805202	0.240586566	0.809875563	0.975068072
ZNF790-AS1	8.489105931	-0.207858581	0.864554913	-0.240422648	0.810002623	0.975155164
TBC1D3P1-DHX40P1	13.39194941	0.177771606	0.73993572	0.24025277	0.810134307	0.97521756
ENTPD5	413.6454476	-0.036557138	0.152185336	-0.24021459	0.810163903	0.97521756
LINC01287	4.885145819	0.242057438	1.008883086	0.239926153	0.810387506	0.975420827
MIR155HG	55.12198389	-0.096925354	0.404297346	-0.239737794	0.810533534	0.975476642
PSMB9	22.17998042	-0.146515419	0.611198027	-0.23971841	0.810548562	0.975476642
TMEM63C	19.19295059	0.136097184	0.567889085	0.239654516	0.810598099	0.975476642
SCAMP5	25.78423392	-0.128449956	0.536489019	-0.239427	0.810774496	0.975558463
STRAP	1951.837143	-0.030675015	0.12814383	-0.239379569	0.810811271	0.975558463
RPS17	134.4090393	-0.052079397	0.217582244	-0.239354994	0.810830325	0.975558463
LINC01600	7.042060317	-0.184687303	0.77201913	-0.23922633	0.810930087	0.975612621
ZNF611	283.7015155	-0.039659097	0.165889738	-0.239069018	0.811052066	0.975685382
RSPH3	190.3663206	-0.070469689	0.294922256	-0.238943271	0.811149571	0.975685382
MORF4L2	4419.756064	0.02577824	0.10788741	0.2389365	0.811154822	0.975685382
LINC00839	176.5234237	-0.046314422	0.193978191	-0.238760977	0.811290931	0.975748973
MAP4K3	849.1415802	-0.042511731	0.178076688	-0.2387271	0.811317201	0.975748973
GPR89A	143.2692639	0.049353345	0.207010695	0.238409638	0.811563393	0.975874596
LTO1	147.8574706	0.061307817	0.257257524	0.238313018	0.811638325	0.975874596
RPS6KA1	426.8201771	0.030765764	0.129108537	0.2382938	0.81165323	0.975874596
CACUL1	1024.77871	0.023399663	0.098239545	0.238189852	0.811733848	0.975874596
RCL1	330.4336016	0.052642078	0.221009123	0.238189614	0.811734032	0.975874596
DOCK6	490.6546217	0.034428345	0.144605263	0.238085007	0.811815163	0.975874596
ZSCAN31	42.54002198	0.100379729	0.421699926	0.238035919	0.811853236	0.975874596
FOXJ2	497.2456824	0.045116902	0.189644595	0.237902389	0.811956802	0.975874596
NME4	766.0439963	-0.035227336	0.148105241	-0.237853405	0.811994796	0.975874596
BAIAP2L2	7.565879497	0.23685326	0.996447932	0.237697578	0.812115663	0.975874596
CIAPIN1	480.3668284	0.030836022	0.129739736	0.237676005	0.812132396	0.975874596
G3BP2	1922.136862	-0.030847917	0.1298169	-0.237626356	0.812170907	0.975874596
CCDC28A-AS1	18.33034715	-0.109403146	0.46042221	-0.237614833	0.812179845	0.975874596
SETD7	1098.984246	0.029622666	0.124675851	0.237597462	0.812193319	0.975874596
UNC5C	110.5589171	0.063410284	0.267023206	0.237471062	0.812291366	0.975874596
SREK1	977.8478213	0.042032202	0.177034964	0.237423167	0.812328519	0.975874596
UQCRC2	2331.649836	-0.033373322	0.140587733	-0.237384313	0.812358658	0.975874596
NAT8L	18.23311946	-0.118658463	0.499994941	-0.237319328	0.812409069	0.975874596
AIP	347.198673	0.051007313	0.214993126	0.237250901	0.81246215	0.975874596
PREX2	100.3957377	0.056118675	0.236832749	0.236954877	0.812691796	0.976008839
ADAM12	146.5361912	0.126051817	0.532167684	0.236864847	0.812761642	0.976008839
ARHGAP27	14.46897364	-0.127557108	0.53854616	-0.236854549	0.812769632	0.976008839
MTPAP	660.3133301	0.029342283	0.123907633	0.236807713	0.812805967	0.976008839
LINC02606	20.77226228	-0.110399618	0.466397075	-0.236707354	0.812883829	0.976008839
ZFHX4	3613.319584	-0.056803292	0.240103844	-0.236578021	0.812984173	0.976008839
PCGF6	241.2837956	-0.042567916	0.179986555	-0.236506087	0.813039985	0.976008839
ZNF578	2.192524932	-0.352362501	1.49021762	-0.236450366	0.813083218	0.976008839
ZNRF2P1	76.52100297	-0.070814446	0.299854264	-0.236162878	0.813306284	0.976008839
PCAT7	257.9068009	0.069250685	0.293265048	0.236136852	0.813326479	0.976008839
SNORD15A	21.84507704	-0.092117428	0.390102808	-0.236136286	0.813326917	0.976008839
LSS	2938.102484	0.02765388	0.117145153	0.236065077	0.813382172	0.976008839

OCIAD2	14.07123418	-0.174781751	0.74055567	-0.236014331	0.81342155	0.976008839
ZNF804A	262.72769	-0.04797958	0.203322685	-0.235977508	0.813450123	0.976008839
KLF7	316.9953615	0.046810937	0.198397216	0.235945535	0.813474934	0.976008839
RUNX1	168.5937294	-0.050012784	0.212014294	-0.235893453	0.813515348	0.976008839
TJP2	236.5100235	0.054462161	0.230913198	0.235855555	0.813544757	0.976008839
YIPF1	169.3658106	-0.046682496	0.197944578	-0.235836195	0.813559781	0.976008839
H2BC14	9.279267183	-0.190435723	0.807787462	-0.235749788	0.813626833	0.976023573
LINC01551	2.862411913	0.290775261	1.235036771	0.235438545	0.813868372	0.976247603
PZP	5.026485091	0.241599917	1.026884346	0.23527471	0.813995523	0.976334402
NAB1	879.2535975	0.033826765	0.143847104	0.235157776	0.814086277	0.976377538
METTL17	709.2642746	-0.044569569	0.189681928	-0.234970036	0.81423199	0.976486578
RAB40C	294.6874957	0.038763252	0.165062528	0.234839804	0.814333072	0.976518236
ZEB1-AS1	97.39244693	0.071683562	0.30530299	0.234794823	0.814367986	0.976518236
PCGF5	659.0955907	0.038348999	0.163412453	0.234676112	0.81446013	0.976529902
RAB43	22.55075466	0.109388321	0.466194225	0.23464109	0.814487314	0.976529902
STK17A	292.252397	-0.036774562	0.156826511	-0.234492	0.814603043	0.976541453
DIRC1	20.85770241	-0.157235166	0.670686789	-0.234439038	0.814644155	0.976541453
TWSG1	696.9551966	0.034175961	0.14584542	0.234330027	0.814728776	0.976541453
AFG3L1P	165.4710732	0.088965019	0.379783778	0.234251763	0.814789532	0.976541453
CPNE2	193.4047894	-0.04782815	0.204186733	-0.234237303	0.814800757	0.976541453
CBX3P2	18.78125995	-0.123679382	0.528081488	-0.234205107	0.81482575	0.976541453
RASSF8-AS1	33.99379501	0.098627506	0.421269689	0.234119635	0.814892103	0.976555298
CES3	22.86182898	0.09910906	0.423503234	0.234021967	0.814967925	0.976580487
STEAP4	2.298948632	-0.319947179	1.368485203	-0.233796594	0.815142894	0.976724473
MYH13	112.7187808	0.127043092	0.543626173	0.233695688	0.815221236	0.976750241
VWA5B2	33.07350411	0.089908508	0.384836682	0.233627698	0.815274023	0.976750241
SNORD22	2.928608085	0.297885632	1.275770974	0.233494599	0.815377363	0.976808377
FLYWCH1	627.9345163	-0.027000651	0.115815215	-0.233135612	0.815656102	0.977076616
PRELID3A	100.4797182	0.078559542	0.337319541	0.232893539	0.815844075	0.977236098
SLAMF8	2.896159848	-0.330314185	1.419006045	-0.232778561	0.815933362	0.977277356
ITGAL	3.100784311	-0.273148911	1.174834029	-0.232499999	0.816149688	0.977408791
PLCD4	3.151396243	-0.283886809	1.221198421	-0.232465752	0.816176285	0.977408791
GPN2	392.1471001	-0.029030409	0.124956827	-0.232323509	0.816286755	0.977408791
RPF1	265.2115306	-0.044108836	0.189955398	-0.232206277	0.816377803	0.977408791
NR5A1	1.980920064	0.374774928	1.614357198	0.23215118	0.816420595	0.977408791
DUS3L	289.6472775	-0.058310629	0.251228962	-0.232101539	0.81645915	0.977408791
TGM2	33.00241341	0.139104266	0.599473184	0.232044184	0.816503696	0.977408791
MEIS1-AS3	14.60609766	0.122711634	0.528915698	0.232006035	0.816533327	0.977408791
HOXD1	112.4933755	0.060828028	0.26218792	0.232001643	0.816536738	0.977408791
LSMEM1	9.45676218	0.196025987	0.845469094	0.231854704	0.816650867	0.977453081
ARL5A	658.3258161	0.033209774	0.143292277	0.231762486	0.816722496	0.977453081
MEIS1-AS2	11.1158769	0.140367229	0.605882704	0.231673932	0.81679128	0.977453081
SNX32	47.10103807	-0.089827568	0.387736756	-0.231671532	0.816793144	0.977453081
MIR410	2.574724434	-0.356611928	1.542745656	-0.231154064	0.817195115	0.977823099
UBE2L6	60.70728196	0.097153892	0.420339035	0.231132216	0.817212088	0.977823099
ZBTB7A	614.6313635	-0.02907367	0.12591746	-0.230894664	0.817396636	0.977905982
FGF14	5.012566989	0.196078277	0.849431291	0.230834771	0.817443168	0.977905982
BEX2	2.641133022	0.302268141	1.309477314	0.23083114	0.817445988	0.977905982
METTL27	32.85982612	0.091945679	0.398555414	0.230697353	0.81754993	0.977954225
ASAP3	1128.22006	0.055618768	0.241333588	0.230464266	0.817731028	0.977954225
PTHLH	23.41896886	0.098826428	0.428919034	0.23040812	0.817774652	0.977954225
TXNRD2	363.5787125	0.038850754	0.168684133	0.230316588	0.817845772	0.977954225
NAIF1	132.2938884	0.052756698	0.229286355	0.230090875	0.818021156	0.977954225
HAUS3	281.1736095	-0.040611123	0.176618821	-0.229936553	0.818141072	0.977954225
TTF1	192.6020162	0.035590712	0.154809895	0.229899464	0.818169893	0.977954225
DYNLT1	305.6536152	0.039770673	0.173064198	0.229803005	0.81824485	0.977954225
NUP58	1046.109541	-0.030074748	0.130874059	-0.229799153	0.818247843	0.977954225
KLHDC3	1562.964657	-0.027494675	0.119667634	-0.229758658	0.818279311	0.977954225
FBXL15	164.2537054	0.044925202	0.195543476	0.229745337	0.818289663	0.977954225
LOC101928272	71.72037013	-0.053641479	0.233505796	-0.229722261	0.818307596	0.977954225
LOC101927811	10.17919648	-0.173856381	0.757052886	-0.229648924	0.818364587	0.977954225
ACOT4	15.17129371	0.109734944	0.477963709	0.229588443	0.818411588	0.977954225
LINC00539	8.786412008	-0.195079535	0.849814569	-0.229555414	0.818437256	0.977954225
FOXH1	3.716982366	0.267613187	1.16579166	0.229554899	0.818437656	0.977954225
VAMP5	46.72414813	0.101761203	0.443411258	0.229496209	0.818483267	0.977954225
USO1	1600.689082	0.032159175	0.140194843	0.229389143	0.818566474	0.977954225
MEX3A	490.2151139	-0.032070312	0.139855153	-0.229310908	0.818627276	0.977954225
ABCD4	604.0472362	0.059536612	0.259760068	0.229198475	0.818714658	0.977954225
PRX	61.89007974	-0.063427452	0.276785545	-0.229157387	0.818746591	0.977954225
RWDD2A	68.40645128	-0.074272307	0.324112058	-0.229156261	0.818747467	0.977954225
MBL2	7.245022774	0.163840582	0.715159038	0.229096708	0.818793752	0.977954225
NUP210L	27.7521042	0.126460255	0.552165808	0.229025871	0.818848809	0.977954225
SELENOK	182.3582676	0.037834758	0.165207433	0.229013653	0.818858305	0.977954225
SNORD114-3	7.480187513	-0.18015538	0.786969868	-0.228922843	0.818928886	0.977961356
HMX3	2.955543256	-0.300264705	1.312058563	-0.228850078	0.818985443	0.977961356
ZNF461	82.12391599	0.054153131	0.236814601	0.228673109	0.819122997	0.977961356
SYT7	29.00131646	0.090867891	0.397413347	0.228648311	0.819142272	0.977961356
CALU	12357.27578	0.036727188	0.160754165	0.228468037	0.819282402	0.977961356
LOC100132781	3.803836351	0.282267036	1.236506616	0.228277821	0.819430266	0.977961356
SOCS3	253.7471321	-0.090987662	0.398812998	-0.228146181	0.819532599	0.977961356
NCKAP1	7083.097012	-0.022756467	0.099806737	-0.228005318	0.819642106	0.977961356
PINX1	218.9924382	0.054711701	0.23998167	0.227982835	0.819659585	0.977961356
MPDZ	776.2510343	0.025805428	0.11319342	0.227976394	0.819664592	0.977961356
SEC23IP	964.5604268	-0.025155955	0.110348458	-0.227968343	0.819670851	0.977961356
STK24-AS1	7.517785021	0.152707788	0.669937895	0.227943201	0.819690397	0.977961356
SOS1	1445.810466	0.027443324	0.120409315	0.227916955	0.819710801	0.977961356
MIR3939	9.046253787	0.218069704	0.956821035	0.22791065	0.819715703	0.977961356
TRAP1	1887.057025	-0.027119569	0.119037324	-0.227824079	0.819783006	0.977961356
EML3	702.6239888	0.032621214	0.143203566	0.227796099	0.81980476	0.977961356
ZBTB48	261.2122599	-0.052051947	0.228613508	-0.22768535	0.819890862	0.977961356

HDAC4-AS1	2.919203732	0.283887937	1.247390383	0.227585478	0.819968511	0.977961356
CCDC189	10.16533911	0.137385152	0.603897965	0.227497292	0.820037075	0.977961356
NDUFV3	383.3443695	-0.03444902	0.151467027	-0.227435767	0.820084911	0.977961356
MACF1	3365.270331	-0.024214114	0.106478737	-0.227407972	0.820106522	0.977961356
EFCAB8	2.232087967	-0.314761893	1.38417805	-0.227399859	0.82011283	0.977961356
PGBD4	171.1344527	0.052893079	0.232670902	0.227330013	0.820167137	0.977961356
ZNF326	618.9339408	-0.076896688	0.338525607	-0.227151763	0.820305735	0.977961356
PSD	12.75178301	-0.146291846	0.644426996	-0.227010735	0.820415394	0.977961356
SNORA80D	8.534803914	0.150020125	0.661139689	0.226911388	0.820492646	0.977961356
CFD	7.286080109	-0.185269252	0.816543152	-0.226894624	0.820505682	0.977961356
WFIKKN2	2.911696938	-0.260392102	1.14791091	-0.226839992	0.820548165	0.977961356
SMAD1	366.0843234	-0.044613738	0.19671009	-0.226799438	0.820579701	0.977961356
NDST1-AS1	11.19812896	0.130787694	0.57674567	0.226768402	0.820603835	0.977961356
SCYL2	1689.125534	0.02374328	0.104706562	0.226760187	0.820610223	0.977961356
CMTR1	1344.232323	-0.022818175	0.100641983	-0.226726207	0.820636647	0.977961356
CKAP4	11485.16367	0.031983691	0.141098408	0.226676483	0.820675315	0.977961356
KBTBD7	242.4690354	-0.054278596	0.239687889	-0.226455314	0.820847311	0.978009615
HNRNPA3	5086.644375	-0.052378572	0.231309957	-0.226443221	0.820856715	0.978009615
ARHGAP5-AS1	30.67516686	-0.081767698	0.361218418	-0.226366359	0.820916491	0.978009615
RAI1	1266.964954	-0.020902486	0.092349077	-0.226342117	0.820935343	0.978009615
RANBP6	504.1460102	0.02830636	0.125151832	0.226176151	0.821064419	0.978097998
C21orf58	333.9468059	0.074988381	0.331937107	0.225911414	0.82127032	0.978197135
TRIM59-IFT80	27.81895919	-0.118360851	0.524139421	-0.225819402	0.821341886	0.978197135
AGPAT1	878.0661088	-0.03643752	0.161425381	-0.225723612	0.821416392	0.978197135
MPHOSPH10	336.0621745	0.033150345	0.146890709	0.225680342	0.821450049	0.978197135
IRF5	5.991112339	0.206256075	0.914114976	0.225634718	0.821485536	0.978197135
CSMD1	1033.162987	0.028106281	0.124566731	0.22563232	0.821487401	0.978197135
LRIG3	677.1689052	0.077673751	0.344414246	0.225524211	0.821571493	0.978197135
CATSPER2P1	23.4839637	-0.117293187	0.520455403	-0.225366451	0.821694208	0.978197135
TIMM23B	83.0616768	-0.061865298	0.274557894	-0.225326967	0.821724922	0.978197135
HS3ST1	225.2647825	-0.173268332	0.769081903	-0.225292431	0.821751787	0.978197135
GAS1	491.7511552	0.078270603	0.347741641	0.225082629	0.821914994	0.978197135
MRAP	4.780699051	-0.203600081	0.90481742	-0.225017862	0.821965379	0.978197135
PSEN2	217.9687051	-0.078028407	0.346781149	-0.225007636	0.821973334	0.978197135
EYA3	1110.435451	-0.033548859	0.149123328	-0.224973915	0.821999567	0.978197135
GPAT2	5.128233342	-0.197230154	0.877274436	-0.2248215	0.82211814	0.978197135
ASPRV1	7.102368463	-0.176948035	0.787848753	-0.224596452	0.822293225	0.978197135
DDX11L2	33.86851503	-0.077129814	0.343500159	-0.224540838	0.822336494	0.978197135
GRIP1	8.920267234	-0.18498558	0.823997096	-0.224497854	0.822369937	0.978197135
GSK3A	559.9718286	-0.027959925	0.1245509	-0.224485936	0.822379209	0.978197135
EIF4G3	1586.870084	0.030126302	0.134201408	0.224485738	0.822379363	0.978197135
IPO13	578.5808604	-0.039081118	0.174128238	-0.224438716	0.822415948	0.978197135
SHROOM3	154.001407	-0.04950771	0.220615404	-0.224407312	0.822440381	0.978197135
BOK	65.46296539	-0.094890416	0.423050349	-0.224300527	0.822523466	0.978197135
SETSIP	2.815467046	0.241568297	1.077007078	0.224295923	0.822527048	0.978197135
NDOR1	612.3030957	0.045019732	0.200918597	0.224069511	0.822703216	0.978197135
ST7	303.3394429	-0.038032678	0.169749606	-0.224051645	0.822717118	0.978197135
SDHA	1599.976347	0.03721488	0.16612948	0.224011298	0.822748512	0.978197135
BET1	222.0929227	0.051830528	0.231458976	0.223929651	0.822812043	0.978197135
RAB3D	4.866522622	-0.202461966	0.904306383	-0.223886472	0.822845642	0.978197135
SS18L2	175.6445045	0.040964455	0.182991698	0.223859637	0.822866523	0.978197135
KCNMA1	380.3797136	0.07960246	0.355622215	0.223839954	0.822881839	0.978197135
SKP2	1320.448311	0.030010242	0.134087317	0.223811189	0.822904223	0.978197135
MTSS2	672.0703761	-0.032799821	0.1466921	-0.223596369	0.823071389	0.978276414
C11orf49	216.0663668	0.049133757	0.219754862	0.223584389	0.823080712	0.978276414
AGAP11	4.110083378	0.235908121	1.058687142	0.222830817	0.823667182	0.978868261
LINC01694	16.20780454	0.104099288	0.467266676	0.222783463	0.823704039	0.978868261
LOC101926943	11.26545841	0.163476248	0.733999018	0.222719981	0.823753449	0.978868261
TRAPPC2B	37.66918057	0.087554903	0.393218456	0.222662242	0.82379839	0.978868261
YIPF2	1331.28736	0.063816903	0.286923483	0.222417847	0.82398862	0.979029018
ARRDC3-AS1	32.11177496	0.10590874	0.476422284	0.222300139	0.824080244	0.979072601
TIMM13	496.4074622	-0.041193131	0.185583636	-0.22196532	0.824340881	0.979258176
GNAZ	7.653909126	-0.176405155	0.795044834	-0.221880764	0.824406706	0.979258176
GABRB2	46.35790941	0.097469083	0.439358087	0.221844291	0.824435099	0.979258176
ERP44	849.0425088	0.030057668	0.135626605	0.221620734	0.82460914	0.979258176
ENHO	5.902661834	-0.170504664	0.769445551	-0.221594191	0.824629805	0.979258176
VPS26C	565.652792	0.0358831	0.161932156	0.22159342	0.824630406	0.979258176
FALEC	4.145900799	0.198960554	0.897935346	0.22157559	0.824644286	0.979258176
STC2	8.496133005	0.171456701	0.77394938	0.221534775	0.824676063	0.979258176
KYAT3	338.5281342	0.049306695	0.222772158	0.221332395	0.824833627	0.979286417
CDON	665.7753146	-0.075720268	0.342239571	-0.221249307	0.824898319	0.979286417
RIMKLB	495.376402	0.054989112	0.248735859	0.221074323	0.825034563	0.979286417
KCNH1	12.1912302	0.139399281	0.630739248	0.221009365	0.825085141	0.979286417
AGMAT	45.46732801	-0.061693399	0.279146485	-0.221007256	0.825086783	0.979286417
MIR29A	2.609743571	0.398862108	1.80585462	0.22087166	0.825192365	0.979286417
MTA1	2192.590828	-0.01956722	0.088617807	-0.220804605	0.825244578	0.979286417
HECW2-AS1	2.342633745	-0.276934756	1.254681818	-0.220721104	0.825309598	0.979286417
INSIG2	278.4630639	0.03770936	0.170870663	0.220689491	0.825334214	0.979286417
GDPGP1	65.27392055	0.063438356	0.287471266	0.220677206	0.825343781	0.979286417
HCG20	2.257375626	0.300377185	1.361199672	0.220670921	0.825348675	0.979286417
H2BC20P	18.81815004	-0.114748357	0.520029785	-0.220657278	0.825359298	0.979286417
LINC01322	2.224718716	0.318947536	1.446736159	0.220460057	0.825512877	0.97931251
LOC400710	1.880422817	0.315243795	1.430465888	0.220378408	0.82557646	0.97931251
VAMP4	315.6925274	0.039097078	0.17745866	0.220316538	0.825624641	0.97931251
OSMR-AS1	2.387123515	-0.251661099	1.143152154	-0.220146634	0.825756957	0.97931251
FPR1	10.10980819	0.146773188	0.666840157	0.220102504	0.825791324	0.97931251
PLIN3	1307.836532	-0.037207996	0.169059531	-0.220088129	0.82580252	0.97931251
CCDC144NL-AS1	491.2029541	-0.0390455	0.177562353	-0.219897401	0.82595106	0.97931251
GCNT7	17.76738488	0.159793514	0.72732599	0.219699992	0.826104811	0.97931251
L2HGDH	421.4795145	0.032879973	0.149662289	0.219694438	0.826109137	0.97931251

ADAMTSL4-AS1	22.90730818	0.129311693	0.588883733	0.219587816	0.826192181	0.97931251
NIFK-AS1	68.31271971	-0.083616094	0.380867143	-0.219541369	0.826228358	0.97931251
RNF151	2.957455831	-0.286085885	1.303992523	-0.219392274	0.826344488	0.97931251
SNORD1A	17.25708925	-0.157551104	0.718406718	-0.219306278	0.826411472	0.97931251
XXYLT1	955.8807152	-0.036649282	0.167208024	-0.21918375	0.826506914	0.97931251
FOXL2NB	249.8741744	-0.059765713	0.272726745	-0.219141372	0.826539923	0.97931251
FAM168B	2715.391764	-0.025829333	0.11792299	-0.219035604	0.826622313	0.97931251
AVIL	28.89456042	-0.083353312	0.38066081	-0.218970037	0.826673388	0.97931251
PER3P1	4.353790364	0.270922833	1.237325613	0.218958397	0.826682456	0.97931251
MED31	98.83703759	-0.052642157	0.240467017	-0.218916331	0.826715225	0.97931251
SLC11A1	5.110745414	-0.220282313	1.006281689	-0.218907206	0.826722333	0.97931251
TWNK	442.6788325	-0.026600495	0.121571975	-0.218804499	0.826802342	0.97931251
SYNJ2BP	768.8424414	-0.029500033	0.134924457	-0.218641109	0.826929627	0.97931251
DNAH7	90.89080207	0.063891688	0.29242572	0.218488607	0.827048434	0.97931251
SEC61B	358.2846937	-0.033247393	0.152234584	-0.218395793	0.827120743	0.97931251
POP7	121.0814263	0.050986518	0.233484892	0.21837181	0.827139428	0.97931251
CTNNB1	4973.138198	0.026373058	0.120784508	0.218348017	0.827157964	0.97931251
MIR600HG	40.05108487	-0.085872714	0.393390504	-0.218288732	0.827204153	0.97931251
CIT	1878.613015	-0.021333792	0.097800911	-0.2181349	0.827324006	0.97931251
SDHAF2	253.8994596	-0.036823597	0.16882955	-0.218111087	0.82734256	0.97931251
HMMR-AS1	8.848651762	0.177336907	0.813228932	0.218065172	0.827378333	0.97931251
PPP1R9B	2192.150945	-0.031125201	0.142803341	-0.217958496	0.82746145	0.97931251
TRAPPC10	611.8344117	0.023912644	0.109737087	0.2179085	0.827500404	0.97931251
MAGEB1	24.75451571	-0.098390592	0.451726544	-0.217810074	0.827577095	0.97931251
PHF20L1	1869.843213	-0.022976026	0.105514706	-0.217751881	0.827622439	0.97931251
KIAA0556	416.8363368	0.028807324	0.132441978	0.217509017	0.82781168	0.97931251
RXFP4	7.060229482	-0.197931754	0.910315441	-0.217432051	0.827871655	0.97931251
ATP6V0E1	670.7714911	0.03139033	0.144371558	0.217427385	0.827875291	0.97931251
SLC38A3	11.18342436	-0.444513953	2.044605305	-0.217408197	0.827890244	0.97931251
TUBA3E	3.467671396	-0.209178907	0.962578104	-0.217311101	0.827965907	0.97931251
EPPK1	3.646058255	-0.204689387	0.942560969	-0.21716302	0.828081302	0.97931251
LINC00653	16.1476723	-0.114298878	0.526363362	-0.217148242	0.828092819	0.97931251
SLC23A3	30.77932556	0.094660096	0.436000385	0.21711012	0.828122528	0.97931251
CHERP	677.0436315	0.024255322	0.111768257	0.217014406	0.828197118	0.97931251
AMD1	2137.000822	-0.040994148	0.188991354	-0.216910175	0.828278348	0.97931251
TPM4	10881.99725	0.039918081	0.184130651	0.216792155	0.828370326	0.97931251
ATRAID	1054.492438	-0.04580329	0.211293307	-0.216775869	0.828383018	0.97931251
SNORA87	3.69178547	-0.288916657	1.333284779	-0.216695384	0.828445746	0.97931251
SCOC	451.0265241	0.029916699	0.138070134	0.216677558	0.828459638	0.97931251
RALGDS	588.9765625	0.02585492	0.119336283	0.216655985	0.828476452	0.97931251
ARF1	3954.47173	0.038523627	0.177851986	0.216604986	0.828516199	0.97931251
LHX1	1.971257051	-0.309366929	1.428286453	-0.216600058	0.82852004	0.97931251
PCDHB18P	20.14040995	-0.09579612	0.442283598	-0.216594331	0.828524504	0.97931251
ST8SIA1	227.1898647	-0.04239465	0.195758357	-0.216566235	0.828546401	0.97931251
UNK	726.9656708	0.032553652	0.150388071	0.216464324	0.828625831	0.97931251
ANAPC7	1693.400398	-0.032231484	0.148932947	-0.216416075	0.828663437	0.97931251
EXD3	92.49873374	0.066337915	0.306576078	0.216383209	0.828689054	0.97931251
ZCCHC7	242.6845927	-0.03855984	0.178291872	-0.216273684	0.828774421	0.97931251
RAG1	66.41277036	0.104035831	0.48104655	0.21626978	0.828777464	0.97931251
B4GALNT4	22.63412381	-0.131484716	0.607994234	-0.216259808	0.828785237	0.97931251
SCFD1	1167.263352	-0.024375436	0.112731025	-0.216226511	0.82881119	0.97931251
TBC1D5	1945.925836	0.043095776	0.199320788	0.216213155	0.8288216	0.97931251
PCDHGA5	5.341944092	0.183678486	0.849548461	0.21620719	0.828826249	0.97931251
GRK4	9.900713895	0.121797921	0.563396592	0.216185051	0.828843506	0.97931251
MGAT4FP	2.917729736	0.302066526	1.397824387	0.216097622	0.828911653	0.979328095
ADPGK	1920.729059	0.034648766	0.1604537	0.215942458	0.829032601	0.979406056
BSCL2	28.40359501	-0.090663483	0.420204926	-0.215760163	0.829174703	0.979442095
CPED1	610.9577633	-0.025676706	0.11904638	-0.215686577	0.829232065	0.979442095
ANXA2	11633.35573	-0.040127865	0.186165593	-0.215549308	0.829339073	0.979442095
MTFR1L	460.9622325	-0.032178713	0.149313054	-0.215511717	0.829368378	0.979442095
SRM	1152.42372	0.025136797	0.116664281	0.215462662	0.829406619	0.979442095
HERC2P10	1.977337375	0.272735128	1.265892063	0.215448959	0.829417302	0.979442095
TEAD3	385.7774272	0.027285162	0.12668702	0.21537457	0.829475295	0.979442095
GGPS1	511.0860032	-0.035118147	0.163082869	-0.215339275	0.829502811	0.979442095
DYNC1LI1	354.3120271	-0.030503959	0.141727643	-0.215229427	0.829588449	0.979450706
MIR889	10.43117963	0.158639786	0.737211693	0.215188918	0.829620031	0.979450706
SDF2	626.4499996	-0.038801964	0.180565195	-0.214891713	0.829851746	0.979481527
FTSJ1	585.3967497	-0.02468669	0.114906211	-0.214842081	0.829890443	0.979481527
TMEM179B	224.039833	-0.040877963	0.190281643	-0.214828727	0.829900855	0.979481527
RSBN1L	496.6047258	-0.026117795	0.121604156	-0.214777161	0.82994106	0.979481527
ACP2	268.2445246	-0.030403819	0.141588123	-0.214734246	0.829974521	0.979481527
SGPP2	10.83150527	0.154931191	0.721508082	0.21473244	0.829975929	0.979481527
DPP3	487.6351742	-0.025811121	0.120273256	-0.214603994	0.83007608	0.979534848
BSDC1	660.2212013	-0.03105501	0.144829988	-0.214423892	0.830216512	0.979568412
SLC25A42	113.3109272	0.067429313	0.314630419	0.214312758	0.830303317	0.979568412
SF3B4	283.9409739	-0.03017501	0.140812032	-0.214292838	0.830318703	0.979568412
ATPAF2	193.8294817	0.038137071	0.178024949	0.214223184	0.830373017	0.979568412
CCDC86	401.6712752	-0.042730586	0.199543694	-0.214141503	0.830436712	0.979568412
CCNT2	643.0846474	-0.047548859	0.222121465	-0.214066926	0.830494867	0.979568412
ADAMTS9	235.5194737	-0.048109451	0.224779508	-0.214029524	0.830524034	0.979568412
RUVBL1-AS1	14.84840402	0.161336453	0.754607098	0.213801929	0.83070152	0.979568412
SBF1	2499.558409	0.018332634	0.085747819	0.21379709	0.830705294	0.979568412
TBX10	2.412486686	-0.249192095	1.165926862	-0.213728754	0.830758587	0.979568412
CHRND	7.238947451	0.188696867	0.883310736	0.213624559	0.830839846	0.979568412
POLR3E	694.7608969	0.028834556	0.134984715	0.213613489	0.83084848	0.979568412
FAAH2	3.087483453	-0.236183199	1.105761963	-0.213593167	0.830864328	0.979568412
DGKG	29.42634605	0.104212297	0.487929532	0.213580631	0.830874105	0.979568412
CHAD	31.56349893	0.09743941	0.456561047	0.213420331	0.830999124	0.979604745
TMEM249	22.55099708	0.144218926	0.676077783	0.213317062	0.831079666	0.979604745
SNORD113-3	37.54973811	0.10716688	0.502720198	0.213174009	0.83119124	0.979604745

GPR35	2.721137044	-0.284708146	1.335949517	-0.213112952	0.831238862	0.979604745
GRHL3	8.121076691	-0.202799514	0.951694883	-0.213092996	0.831254428	0.979604745
COL23A1	18.56318242	0.179540319	0.842731438	0.213045711	0.831291308	0.979604745
STAT5B	1083.380403	-0.025900082	0.121577357	-0.213033768	0.831300624	0.979604745
SPOCK3	62.06917167	-0.105163719	0.493779099	-0.212977259	0.8313447	0.979604745
MIR3651	5.771302969	0.176580067	0.829672838	0.212830961	0.831458814	0.979674429
CECR7	145.3161476	-0.070687746	0.332395801	-0.212661369	0.8315911	0.979693155
AK9	25.62953039	0.103220754	0.485480927	0.212615468	0.831626906	0.979693155
IFNGR2	316.0635219	-0.041330031	0.194403561	-0.212599145	0.831639639	0.979693155
ALKAL2	13.00911403	0.144836837	0.68188486	0.212406589	0.831789846	0.979805331
IDS	708.5411805	-0.032013233	0.15086532	-0.212197431	0.831953011	0.979932756
WASIR2	2.510901854	0.265860916	1.25483393	0.211869403	0.832208921	0.980018066
GTPBP8	156.5573609	0.03718445	0.175528711	0.211842553	0.832229869	0.980018066
MSX1	145.1207713	-0.040917214	0.193163366	-0.21182699	0.832242011	0.980018066
SLC39A3	303.7319116	0.037220899	0.175717299	0.211822621	0.83224542	0.980018066
RPL27	267.7588925	-0.041235834	0.194879806	-0.211596238	0.832422044	0.980110712
BRIP1	993.0931143	0.052392123	0.247622304	0.211580789	0.832434098	0.980110712
MSL3P1	139.5619872	0.043283427	0.204651166	0.211498561	0.832498255	0.980121492
ENOX1-AS2	5.837286725	-0.209971504	0.994060953	-0.211225985	0.832710935	0.980210816
PAPOLB	3.85116573	-0.253159012	1.198555143	-0.211220162	0.832715479	0.980210816
CCNB2	801.4944704	-0.033303379	0.157694046	-0.211189833	0.832739144	0.980210816
UST-AS1	6.522542823	-0.208917963	0.989772172	-0.21107682	0.832827328	0.980249867
NAA80	59.51506383	-0.058439816	0.277032982	-0.210948946	0.83292711	0.980269178
MICOS13	262.6635586	0.036034231	0.171003362	0.210722357	0.833103928	0.980269178
SP2-AS1	66.41424478	0.070774722	0.335916899	0.210691162	0.833128272	0.980269178
SC5D	529.1205211	0.046201152	0.219420632	0.210559745	0.833230827	0.980269178
TRIM16	335.2496517	-0.038765143	0.184139095	-0.210520979	0.833261079	0.980269178
MDFIC	1539.861855	-0.041551943	0.197446212	-0.210446898	0.833318893	0.980269178
TUT4	918.0561902	0.0189737	0.09018219	0.210392985	0.833360967	0.980269178
SNORD54	4.462641484	0.195906079	0.931200674	0.210380087	0.833371033	0.980269178
RNVU1-20	7.065932974	-0.164016661	0.779880202	-0.210310071	0.833425676	0.980269178
BANP	240.4319098	-0.03976949	0.189140068	-0.210264755	0.833461042	0.980269178
SLC7A8	299.2343146	-0.198578691	0.945100692	-0.210113793	0.83357886	0.980269178
AADAT	226.6521799	0.048454928	0.230634927	0.210093627	0.833594599	0.980269178
GON4L	1048.118464	0.026482647	0.126141671	0.209943685	0.833711626	0.980269178
MOB3C	169.9730751	-0.044527066	0.212161267	-0.20987368	0.833766265	0.980269178
PTOV1	1199.150825	-0.023178311	0.110447709	-0.209857778	0.833778677	0.980269178
TRPV4	23.54047145	0.095561902	0.455381027	0.209850424	0.833784416	0.980269178
ZNF532	1214.722011	0.019967235	0.095183228	0.209776826	0.833841861	0.980269178
PTPRB	12.56020106	0.115393464	0.55014488	0.209751046	0.833861983	0.980269178
THEGL	12.35077714	-0.122166936	0.582533623	-0.209716541	0.833888915	0.980269178
MIR494	5.603629931	-0.21859381	1.042786677	-0.209624667	0.833960626	0.980288809
DTD1-AS1	6.054996628	0.246405487	1.177424231	0.209275026	0.834233546	0.980392583
LINC02639	7.703216573	0.177934168	0.850479544	0.209216281	0.834279403	0.980392583
STIP1	2007.683724	-0.035401816	0.169308155	-0.209096933	0.834372569	0.980392583
MOB3B	1.78119499	-0.344859648	1.649348751	-0.209088373	0.834379251	0.980392583
MMAB	603.14517	-0.049184749	0.235252941	-0.209071771	0.834392211	0.980392583
CLEC16A	446.5818117	0.027826812	0.133206174	0.208900316	0.834526058	0.980392583
ZKSCAN7	75.93210078	-0.068372776	0.327398917	-0.208836291	0.83457604	0.980392583
TSSK6	29.88059605	0.072613155	0.347830986	0.208759881	0.834635692	0.980392583
CELF4	8.789229645	-0.147855303	0.708358564	-0.208729464	0.834659438	0.980392583
PHACTR2	4722.745066	0.023410653	0.112216737	0.208619981	0.834744911	0.980392583
XYLT1	36.05191376	-0.10900837	0.522538252	-0.208613187	0.834750216	0.980392583
EIF2D	805.0736112	0.026175886	0.125496922	0.208577913	0.834777754	0.980392583
FAM90A1	6.603247544	-0.161522575	0.774427652	-0.208570258	0.834783731	0.980392583
ZNF807P	2.10416566	0.290493308	1.393427081	0.208473994	0.834858887	0.980392583
STUB1	387.5836817	-0.037721241	0.180956767	-0.208454437	0.834874155	0.980392583
ALDH2	569.4901079	0.16158904	0.775734875	0.208304467	0.834991244	0.980465469
P2RX2	9.553601241	0.177078697	0.850506589	0.208203792	0.835069848	0.980486805
FUT8	1735.241109	0.036442239	0.175139752	0.208075201	0.83517025	0.980486805
SETD1A	571.0303007	0.031621893	0.151991637	0.208050219	0.835189756	0.980486805
CS	2813.460898	-0.023140146	0.111255616	-0.207990813	0.835236141	0.980486805
OTOGL	695.2311621	0.140369735	0.675234491	0.207882946	0.835320366	0.980486805
PCNT	1180.797121	0.043200753	0.207837434	0.207858384	0.835339544	0.980486805
CPXM1	21.39103047	-0.137885154	0.664161838	-0.207607763	0.835535242	0.980587757
C15orf41	1442.037845	0.040023339	0.192790333	0.207600341	0.835541038	0.980587757
PFN4	30.88828025	0.081094905	0.390749467	0.207536829	0.835590633	0.980587757
SHB	119.3740346	-0.048773342	0.235369931	-0.207219936	0.835838099	0.98075424
TFEB	26.96333194	-0.107338909	0.518299171	-0.207098362	0.835933041	0.98075424
ZCWPW1	25.06342069	0.107720892	0.520395352	0.206998182	0.836011278	0.98075424
L1CAM	216.7435385	-0.069528837	0.336017251	-0.20692044	0.836071993	0.98075424
BEND6	148.8230261	0.057631825	0.278602391	0.206860482	0.83611882	0.98075424
THAP2	88.47124557	0.060803852	0.293966412	0.206839454	0.836135242	0.98075424
LOC101928617	15.52262668	-0.109647034	0.530234936	-0.206789531	0.836174232	0.98075424
PIGZ	54.9339433	-0.059097856	0.285848091	-0.206745672	0.836208487	0.98075424
AKAP12	195.4030054	-0.052552162	0.254317135	-0.206640272	0.836290807	0.98075424
FAM98C	132.7432173	0.042769616	0.207027302	0.206589255	0.836330652	0.98075424
RSAD2	4.223252934	-0.229313825	1.110602498	-0.206476958	0.836418362	0.98075424
RABL6	1991.289821	0.019107752	0.092588095	0.206373742	0.83649898	0.98075424
FBXO36	77.59822333	0.082306757	0.398903214	0.206332649	0.836531077	0.98075424
PPP1R15B	2824.680161	0.02341497	0.113521858	0.206259571	0.836588157	0.98075424
COPB1	3942.430304	0.030680078	0.148803785	0.206178078	0.836651811	0.98075424
PNRC1	837.3292641	-0.038070651	0.184783911	-0.206027955	0.836769075	0.98075424
LOC105377763	18.64183082	0.121874793	0.591754236	0.205955084	0.836825997	0.98075424
COL18A1-AS2	10.3626	0.188496827	0.915554931	0.205882597	0.83688262	0.98075424
ARHGAP6	130.1366654	0.048724827	0.236677288	0.205870311	0.836892217	0.98075424
DUS2	370.3664241	0.037813475	0.183692327	0.205852231	0.836906341	0.98075424
CYB5R3	944.0861247	-0.038096133	0.185082363	-0.205833406	0.836921046	0.98075424
SLIT3	1883.959271	-0.11437371	0.555738496	-0.205804909	0.836943307	0.98075424
LINC00987	4.07392473	0.202026359	0.98321852	0.205474526	0.837201399	0.980961161

FXN	148.7990467	0.050649433	0.246547725	0.205434598	0.837232592	0.980961161
CCDC43	398.0325518	0.027889363	0.135961639	0.205126702	0.837473135	0.980961161
PNPLA8	320.2767107	-0.044592856	0.217392324	-0.205126176	0.837473546	0.980961161
RAD9A	129.6397475	-0.059604857	0.290606128	-0.2051053	0.837489856	0.980961161
DRAM2	457.6955278	0.049691492	0.242280423	0.205099081	0.837494714	0.980961161
ERMN	3.074577512	-0.238952457	1.165309772	-0.205054882	0.837529247	0.980961161
IRGQ	592.4994627	0.043471756	0.212152375	0.204908173	0.83764387	0.980961161
NDUFS4	314.9863556	0.040426784	0.197324924	0.20487419	0.837670421	0.980961161
GOSR1	947.39711	0.030028676	0.146589091	0.204849321	0.837689852	0.980961161
RBM23	1610.13638	0.024725562	0.120750191	0.204766237	0.837754767	0.980961161
DENND5B-AS1	5.759143875	0.169254322	0.826856051	0.204696237	0.837809461	0.980961161
NR1H2	491.9538773	-0.036936494	0.180535128	-0.204594499	0.837888954	0.980961161
CDH26	3.254125627	0.254897665	1.245880182	0.204592439	0.837890563	0.980961161
NVL	344.0452867	-0.04501495	0.220124338	-0.204497832	0.837964486	0.980983257
PHC1	464.0136157	-0.026378184	0.12915579	-0.204235395	0.838169553	0.98106435
RPS6KA6	249.1118316	0.038247332	0.187464742	0.204024133	0.83833464	0.98106435
SLC25A11	875.4217238	-0.029022926	0.142262267	-0.204010009	0.838345678	0.98106435
SERHL	4.643078411	0.183842336	0.901558852	0.203916068	0.838419088	0.98106435
ARL6IP6	332.4454559	-0.029427275	0.144323314	-0.203898275	0.838432993	0.98106435
FEM1C	429.9806258	-0.036110846	0.17711282	-0.203886122	0.83844249	0.98106435
CGGBP1	2482.255798	-0.029304407	0.143750162	-0.203856518	0.838465625	0.98106435
LINC00333	7.67386718	0.16137288	0.792286648	0.203679919	0.838603636	0.98106435
GS1-279B7.1	4.598963383	0.202758945	0.996095679	0.203553684	0.83870229	0.98106435
NOLC1	1955.964208	-0.021691825	0.106634278	-0.203422633	0.838804711	0.98106435
GS1-600G8.3	24.69324858	0.135554503	0.666385589	0.203417518	0.838808709	0.98106435
ZNF594	232.3053732	-0.070126863	0.344783609	-0.203393842	0.838827213	0.98106435
OSR2	94.05705974	-0.060338199	0.296674381	-0.203381899	0.838836546	0.98106435
ZNF185	31.21312887	0.08536029	0.41987711	0.20329827	0.838901908	0.98106435
C19orf53	58.64109728	0.055138159	0.271260515	0.203266437	0.838926787	0.98106435
TMEM80	113.0538519	-0.046448361	0.228615406	-0.20317249	0.839000215	0.98106435
FAM117B	626.9625823	0.042588254	0.209710364	0.203081301	0.839071487	0.98106435
KLHDC9	14.68172921	-0.099808076	0.49151609	-0.203061665	0.839086834	0.98106435
B3GNTL1	114.5791288	0.042713042	0.210393775	0.20301476	0.839123496	0.98106435
ARIH2OS	12.15651288	0.122700478	0.604435111	0.203000248	0.839134838	0.98106435
ADRA1B	2.316761022	0.291736743	1.439584316	0.20265346	0.839405901	0.981207733
MAPK3	294.0117801	-0.041939664	0.207009178	-0.202598091	0.839449182	0.981207733
ZNF281	1842.876337	0.034933266	0.172634149	0.20235432	0.839639737	0.981207733
PLCD1	106.6154128	-0.060090283	0.296982552	-0.202336073	0.839654001	0.981207733
SNORA70J	4.413234123	0.188532305	0.932030915	0.202281171	0.839696919	0.981207733
BABAM1	636.554027	0.024806422	0.122666158	0.202227104	0.839739185	0.981207733
RNVU1-14	24.70661884	0.120856171	0.597667371	0.202213099	0.839750133	0.981207733
GOLGA5	624.9266801	0.034348248	0.170034017	0.202008095	0.839910396	0.981207733
BOD1	658.9149037	0.024617864	0.121883271	0.201979024	0.839933122	0.981207733
MNS1	84.4785276	-0.063266974	0.31327746	-0.201951886	0.839954338	0.981207733
ARHGAP45	60.80947108	-0.062561921	0.309811881	-0.201935191	0.83996739	0.981207733
SNORD71	4.759571348	0.210103651	1.04081808	0.201863952	0.840023084	0.981207733
LINC01655	18.61771452	-0.088555716	0.438889268	-0.201772342	0.840094705	0.981207733
ZNF208	12.62385895	0.137597469	0.682157756	0.201709162	0.8401441	0.981207733
ANGEL2	605.4109074	0.029686992	0.147187365	0.201695244	0.840154981	0.981207733
URB1-AS1	9.701197494	-0.12488322	0.619348139	-0.201636547	0.840200872	0.981207733
PAFAH1B2	1874.136815	0.027466949	0.136220782	0.20163553	0.840201667	0.981207733
ARNT2	2.855807922	-0.243000537	1.205506292	-0.201575503	0.840248598	0.981207733
GXYLT2	998.1551054	-0.039624592	0.196791478	-0.201353191	0.840422414	0.981281156
C21orf62-AS1	13.10332354	0.113525205	0.564301757	0.201178188	0.840559246	0.981281156
DIO2-AS1	5.420542161	0.237593312	1.181138552	0.201156174	0.840576459	0.981281156
CAVIN1	7860.600034	-0.033834221	0.16828288	-0.201055635	0.840655072	0.981281156
MIR1915	7.390966407	-0.15100176	0.751109699	-0.201038223	0.840668686	0.981281156
CHCHD3	500.8127912	-0.039591289	0.196973668	-0.200997879	0.840700233	0.981281156
PEX12	227.5478794	0.038661372	0.192371025	0.20097295	0.840719726	0.981281156
MIR376A1	3.187363245	0.347493833	1.729412946	0.200931671	0.840752003	0.981281156
U2SURP	3197.588962	0.040226676	0.200410201	0.200721699	0.840916191	0.981408509
PML	830.2543513	0.027902807	0.139136551	0.20054261	0.841056237	0.981507672
AGO4	554.1829038	-0.029769817	0.148611562	-0.200319653	0.841230593	0.981566918
KIAA1841	401.3030043	0.040902444	0.204197818	0.200307937	0.841239756	0.981566918
DNAJC2	505.8847777	0.047543535	0.237401478	0.200266383	0.841272252	0.981566918
ARRB2	165.1828365	-0.044501641	0.222621328	-0.19989837	0.841560065	0.981673397
TIAF1	17.18374425	0.162852068	0.815230462	0.199762001	0.841666721	0.981673397
ZNF385B	6.883345071	0.150575809	0.753778168	0.199761435	0.841667163	0.981673397
HOTTIP	12.32873563	0.149093118	0.747548784	0.19944266	0.841916493	0.981673397
INTS14	806.2106728	-0.025284723	0.126788299	-0.199424739	0.84193051	0.981673397
MANEAL	11.50675896	0.139278083	0.69853926	0.19938476	0.841961781	0.981673397
TPGS2	1379.990368	0.016172232	0.081118503	0.199365519	0.841976831	0.981673397
PCOLCE2	683.4716013	0.029145211	0.146213874	0.199332735	0.842002474	0.981673397
LTBR	1532.90745	0.028887178	0.144969742	0.199263499	0.84205663	0.981673397
CYP2A13	2.770287442	-0.288733166	1.449361771	-0.199214007	0.842095343	0.981673397
EXOSC2	1361.667006	0.026370975	0.132398017	0.19917953	0.842122311	0.981673397
MFSD14C	211.9793618	0.048761042	0.245012166	0.199014776	0.842251186	0.981673397
TBC1D22B	244.3242385	0.032576657	0.163739692	0.198953941	0.842298774	0.981673397
KERA	192.4950051	-0.078271038	0.393633393	-0.198842474	0.84238597	0.981673397
ANAPC2	795.2191571	0.032754024	0.164728865	0.198835973	0.842391055	0.981673397
ZNF772	147.8724586	-0.041196422	0.207203826	-0.198820761	0.842402955	0.981673397
GJA9-MYCBP	6.885895496	-0.174119706	0.876231532	-0.198714266	0.842486264	0.981673397
LOC400499	8.025743021	-0.129195218	0.650684837	-0.19855268	0.842612672	0.981673397
DENND2A	34.90206976	-0.111699793	0.562614068	-0.198537149	0.842624823	0.981673397
GRWD1	1245.132821	0.031509004	0.158829122	0.198383036	0.842745389	0.981673397
GALNT13	409.6915151	-0.024285904	0.122420573	-0.198380902	0.842747058	0.981673397
EP400	2760.400362	0.026071042	0.13143514	0.198356711	0.842765984	0.981673397
UQCRHL	5.660636971	0.190046072	0.958397847	0.198295596	0.842813797	0.981673397
CTSW	15.41206644	0.120970193	0.610070511	0.198288872	0.842819058	0.981673397
RIF1	2849.006246	0.031851819	0.16082372	0.198054236	0.84300263	0.981673397

GRHPR	391.0501649	-0.026473441	0.133672593	-0.198046889	0.843008378	0.981673397
OSER1-DT	29.78728367	0.081498242	0.411634399	0.197986958	0.843055268	0.981673397
SAR1B	1054.491126	0.026730431	0.135113711	0.197836554	0.843172945	0.981673397
RAB9A	219.116733	-0.040407781	0.204296556	-0.19778983	0.843209504	0.981673397
TRERF1	670.758857	-0.028446669	0.143954367	-0.197608935	0.843351044	0.981673397
PSMA4	1688.18876	-0.025631192	0.129712675	-0.197599748	0.843358232	0.981673397
ZNF112	146.025336	-0.066034213	0.334280871	-0.197541106	0.843404117	0.981673397
BBS7	400.3368637	0.031057405	0.157274972	0.197472012	0.843458181	0.981673397
TRAM1	3992.967422	-0.021235797	0.107545436	-0.197458842	0.843468486	0.981673397
VSTM4	510.3007767	0.078577657	0.397951926	0.197455149	0.843471376	0.981673397
PPARD	1544.158681	-0.065640534	0.332482155	-0.197425736	0.843494392	0.981673397
CCDC74B	47.38856876	0.060554358	0.306784644	0.197383927	0.843527107	0.981673397
TRIM11	405.4898288	-0.03073804	0.155731842	-0.197378004	0.843531741	0.981673397
VIPR1	2.608614806	-0.239489203	1.213520592	-0.197350753	0.843553065	0.981673397
THAP1	120.6081579	-0.041920139	0.212524017	-0.19724895	0.843632726	0.981673397
NACAD	186.2154757	-0.040737755	0.206623636	-0.197159219	0.843702943	0.981673397
RBM25	1332.79362	-0.040961321	0.208231006	-0.196710957	0.844053735	0.981673397
DIO2	22.35346082	0.116333882	0.591466231	0.196687276	0.844072268	0.981673397
SPIRE2	51.46116011	0.066773579	0.339494031	0.196685575	0.844073599	0.981673397
UIMC1	383.4402305	-0.036251423	0.184316962	-0.196679801	0.844078118	0.981673397
LSM4	579.3957107	-0.029858184	0.151831926	-0.196652869	0.844099195	0.981673397
CDC14A	68.53329385	-0.059841869	0.304567422	-0.196481516	0.844233298	0.981673397
TMEM154	15.4656507	0.167814621	0.854107207	0.196479575	0.844234818	0.981673397
TMEM62	62.53133463	-0.059175212	0.301379411	-0.196347891	0.84433788	0.981673397
FBRS	632.8092798	0.024493002	0.124814765	0.196234813	0.844426381	0.981673397
ZNF543	189.5444369	-0.038372345	0.195657682	-0.196119796	0.844516403	0.981673397
JPH3	52.81937816	-0.138605263	0.706755534	-0.196114861	0.844520265	0.981673397
CRYBB2P1	100.4604457	0.061151451	0.312097588	0.195936956	0.844659511	0.981673397
PSMB3	1281.381502	0.0287066	0.146531771	0.195907006	0.844682954	0.981673397
RPP30	358.1823947	0.03502843	0.178825632	0.195880365	0.844703806	0.981673397
TLCD5	97.40812955	0.044934201	0.229482977	0.195806249	0.844761818	0.981673397
ZSWIM4	200.041994	-0.081986326	0.418788956	-0.19577003	0.844790169	0.981673397
CASP9	89.42231512	-0.043685132	0.22315045	-0.195765375	0.844793812	0.981673397
RAPH1	2401.713524	0.046950632	0.239872966	0.195731236	0.844820535	0.981673397
KALRN	146.6581724	0.057162981	0.292334024	0.195539952	0.844970264	0.981673397
TKT	3100.5907	-0.025993688	0.132962383	-0.195496556	0.845004234	0.981673397
COA4	261.0892203	0.037831004	0.193585064	0.195423157	0.845061689	0.981673397
F2R	2150.047788	-0.035380073	0.181191613	-0.195263302	0.845186825	0.981673397
TIGD2	192.716371	-0.036431164	0.186639078	-0.195195798	0.845239668	0.981673397
DUSP15	130.7580973	-0.161671818	0.828308006	-0.195183214	0.84524952	0.981673397
ZNF853	25.48924603	0.110402633	0.565765475	0.195138513	0.845284513	0.981673397
MXRA7	1132.722253	-0.030796881	0.157864923	-0.19508375	0.845327384	0.981673397
HACD3	3308.514226	-0.019879428	0.101938064	-0.195014772	0.845381383	0.981673397
MIR4482	3.097672484	-0.26723024	1.370386807	-0.195003512	0.845390199	0.981673397
ZSCAN25	191.1709249	0.033688954	0.172782892	0.194978531	0.845409755	0.981673397
SYNGAP1	560.4253151	-0.032391104	0.166224755	-0.194863299	0.845499967	0.981673397
CHRNA10	3.746977538	-0.278875631	1.431624261	-0.194796665	0.845552133	0.981673397
ZBTB1	1175.8371	0.041971194	0.215511009	0.194751974	0.845587122	0.981673397
SNORD13E	2.387393745	0.297487614	1.527529682	0.194750791	0.845588047	0.981673397
MIR1289-1	2.293172883	0.307285808	1.578246215	0.194700805	0.845627182	0.981673397
ICA1	16.79574751	0.116800868	0.599903747	0.194699348	0.845628323	0.981673397
TMEM127	1214.016721	-0.032299223	0.165935634	-0.194649105	0.845667658	0.981673397
TP53RK	235.0539834	0.036128102	0.185641408	0.194612303	0.845696471	0.981673397
STAG3L1	59.46313495	0.081752139	0.420129368	0.194588013	0.845715488	0.981673397
USP37	504.2141401	0.025175135	0.129496414	0.194407974	0.845856447	0.981773066
TOMM22	565.0278776	0.030351751	0.156278184	0.194216175	0.846006618	0.981786844
IFIT5	465.8654839	-0.031998219	0.164846665	-0.194108987	0.846090545	0.981786844
LOC101928682	3.023802371	0.280849937	1.447111273	0.194076255	0.846116174	0.981786844
B9D1	176.4051317	-0.034894387	0.179806	-0.194066869	0.846123523	0.981786844
PLCH1	92.81559116	0.054079916	0.27875151	0.194007615	0.846169919	0.981786844
LOC102724135	22.26164042	-0.089122024	0.459461456	-0.193970622	0.846198885	0.981786844
MED24	1096.242711	0.021824367	0.112587928	0.193842866	0.846298921	0.981838983
E2F8	53.92735685	0.069912739	0.361042051	0.19364154	0.846456569	0.981850779
XPO1	8887.606551	0.019582979	0.101231741	0.193447026	0.84660889	0.981850779
E2F7	855.7875821	-0.034395737	0.177832186	-0.193416828	0.846632538	0.981850779
SMG1P5	35.16480293	0.077582072	0.401436039	0.193261353	0.846754291	0.981850779
AUP1	1305.040655	0.021421509	0.110863119	0.193224849	0.846782879	0.981850779
SLC25A1	854.6685762	-0.019124794	0.098991218	-0.193196878	0.846804784	0.981850779
INO80E	331.9964395	-0.035742276	0.185012577	-0.19318836	0.846811454	0.981850779
FAM133B	129.5609983	0.060562424	0.313512703	0.193173748	0.846822898	0.981850779
FAM86JP	67.77547219	-0.050142278	0.259675813	-0.193095682	0.846884034	0.981850779
GPR39	4.653973269	0.198704673	1.030048075	0.192908154	0.847030899	0.981850779
TP53TG3D	18.47058934	0.140258125	0.727376257	0.192827472	0.847094088	0.981850779
GPSM2	752.9722393	0.035061523	0.181829587	0.192826279	0.847095022	0.981850779
FBN2	1838.355471	0.041298802	0.214180455	0.19282246	0.847098013	0.981850779
ARFRP1	380.329989	-0.045684304	0.2369656	-0.19278876	0.847124406	0.981850779
C5orf22	434.7193741	-0.030272381	0.157035179	-0.192774515	0.847135563	0.981850779
SNORD70B	10.20076461	-0.127588043	0.662551617	-0.192570722	0.847295176	0.981938375
DLG2	69.90965791	-0.047981695	0.249306383	-0.192460754	0.847381307	0.981938375
CARD8-AS1	19.96448899	-0.090260307	0.469154324	-0.192389374	0.847437215	0.981938375
CNTN1	1438.379963	0.064184248	0.333752001	0.192311201	0.847498445	0.981938375
ARR3	15.23739519	-0.122555232	0.637423035	-0.192266713	0.847533291	0.981938375
MRPS33	197.8139252	0.031824069	0.165529739	0.192255902	0.847541759	0.981938375
GNL3L	1568.013065	0.026932847	0.140176251	0.192135591	0.847635997	0.981973555
CERT1	516.5677266	-0.026964008	0.140381656	-0.192076434	0.847682334	0.981973555
MIR12114	6.650201156	0.16629073	0.86656682	0.191896027	0.84782365	0.982073416
STK4-AS1	3.621689773	-0.179195073	0.934219904	-0.191812519	0.847889064	0.982085351
MAZ	3292.901546	0.023731621	0.1238506	0.191614903	0.848043868	0.982189251
MYO9B	1938.156125	-0.031928775	0.166680031	-0.191557288	0.848089002	0.982189251
TXNL4A	568.8848221	0.026023823	0.136004263	0.191345641	0.848254805	0.982210183

BMPR2	2322.842699	0.026408052	0.138015603	0.191341061	0.848258393	0.982210183
TCTEX1D1	28.85121627	-0.085261503	0.445688119	-0.191303064	0.848288161	0.982210183
DAAM2-AS1	4.742713662	0.201464795	1.053395344	0.191252787	0.848327549	0.982210183
CCDC38	21.27769445	0.120703751	0.63167617	0.191084857	0.848459111	0.982298685
SLC49A4	68.02232428	-0.05098099	0.267102563	-0.190866719	0.848630015	0.982330877
NUDT13	74.26169224	0.071695207	0.375639228	0.190861873	0.848633812	0.982330877
TALDO1	825.9576329	0.021667901	0.113550654	0.190821455	0.848665478	0.982330877
NCLN	2079.450132	0.043159177	0.22625016	0.190758657	0.848714681	0.982330877
LOC392452	9.807983183	-0.15040544	0.788711885	-0.19069757	0.848762543	0.982330877
FAM149B1	240.5430153	0.034109761	0.178959629	0.190600311	0.848838747	0.982355271
TXN	3080.32922	0.023511559	0.123478871	0.190409572	0.848988197	0.982452819
MIR134	7.588018203	0.195991373	1.030499636	0.190190628	0.849159755	0.982452819
KCTD12	2088.459342	0.017898352	0.094123398	0.190158369	0.849185032	0.982452819
SLC4A5	40.94362111	0.104084401	0.547651789	0.190055803	0.849265403	0.982452819
SNORD114-5	3.743538787	-0.196900433	1.036178673	-0.190025561	0.849289101	0.982452819
RNF10	2196.896459	-0.026004578	0.136873511	-0.189989854	0.849317081	0.982452819
LCMT1-AS1	8.204496032	0.141549046	0.745904984	0.189768199	0.849490776	0.982452819
E2F1	526.7879178	-0.027802489	0.14653532	-0.18973234	0.849518877	0.982452819
LOC101927751	189.0770124	0.049285595	0.260008167	0.189554026	0.849658616	0.982452819
MTF2	602.8761363	0.031114218	0.164207693	0.189480881	0.849715938	0.982452819
SNHG10	193.0017143	0.061432322	0.324263013	0.18945214	0.849738463	0.982452819
ATE1-AS1	9.371756751	0.136210755	0.719106688	0.189416616	0.849766303	0.982452819
SLC18A2	7.532349301	0.136676854	0.721907867	0.189327281	0.849836315	0.982452819
SLC44A4	4.54543083	-0.197412125	1.042804215	-0.189308906	0.849850715	0.982452819
RNF141	294.8904965	-0.028445201	0.150277612	-0.189284355	0.849869957	0.982452819
LOC107986876	14.25982793	-0.13139096	0.694724489	-0.189126715	0.849993504	0.982452819
SNRNP40	1155.83413	-0.022021581	0.116439681	-0.189124367	0.849995344	0.982452819
GTF3A	682.1397789	-0.026701425	0.1412209	-0.189075591	0.850033572	0.982452819
C4orf33	76.35701533	-0.05353973	0.283259367	-0.189013095	0.850082554	0.982452819
ECI2	458.4594725	0.026581327	0.140693637	0.188930558	0.850147243	0.982452819
C8orf33	1330.775861	-0.029745105	0.15750465	-0.188852231	0.850208634	0.982452819
PRR16	64.73236239	0.061549424	0.325958862	0.188825741	0.850229396	0.982452819
LINC01241	88.88618359	0.089791947	0.47582234	0.188708977	0.850320915	0.982452819
LSM7	81.9334601	-0.042742444	0.226593373	-0.188630602	0.850382347	0.982452819
ATP1B1	290.3743347	0.048123136	0.255136034	0.18861756	0.850392569	0.982452819
TOR1AIP2	3639.806654	0.021371337	0.113321415	0.188590452	0.850413817	0.982452819
FAM72C	2.094232726	-0.263858944	1.399575496	-0.188527839	0.850462894	0.982452819
BTD	261.804969	-0.039307116	0.208554273	-0.188474276	0.850504879	0.982452819
IFT80	39.27456481	-0.070233893	0.372706953	-0.18844267	0.850529653	0.982452819
GNA13	2325.155735	0.025809479	0.13700588	0.18838227	0.850576997	0.982452819
ATP6V1E2	104.4087953	-0.037360466	0.198538035	-0.188177878	0.850737214	0.98250489
SART3	1475.209428	0.022876794	0.121614932	0.188108432	0.850791651	0.98250489
C2orf74	82.32484936	0.049020636	0.260668509	0.188057376	0.850831674	0.98250489
TRMT1L	602.0331757	-0.030685991	0.163392886	-0.187804942	0.851029561	0.98250489
CDH8	19.57958364	0.091275625	0.486186126	0.187738029	0.851082018	0.98250489
TRAIP	158.1891539	-0.047192098	0.251483724	-0.187654681	0.851147358	0.98250489
METTL26	141.988807	-0.037245132	0.198515175	-0.187618563	0.851175673	0.98250489
SMPD3	23.13843293	0.112586573	0.600324165	0.187542963	0.851234941	0.98250489
SNORA2B	7.082106503	0.164568223	0.877856345	0.187466006	0.851295274	0.98250489
ZNF250	266.3036022	0.031178752	0.166359413	0.187418019	0.851332896	0.98250489
INTS6	991.5575935	-0.037968198	0.202626326	-0.187380378	0.851362406	0.98250489
MIR487B	6.189418816	-0.235509589	1.257162504	-0.187334246	0.851398574	0.98250489
ZWILCH	1172.121086	0.020092725	0.107295606	0.187265128	0.851452763	0.98250489
NAT1	120.6185222	-0.037028712	0.197741579	-0.187258098	0.851458275	0.98250489
TXNDC9	648.0086827	-0.032079125	0.171334069	-0.187231444	0.851479172	0.98250489
ZNF286A	492.7673914	-0.021790315	0.11649412	-0.18705077	0.851620827	0.98250489
SYNPO2L	31.82134154	0.077272914	0.413164588	0.187026952	0.851639501	0.98250489
WASH8P	427.5925904	-0.022714604	0.121467669	-0.187001233	0.851659666	0.98250489
PCDH11X	3.214469704	0.229072174	1.225060165	0.186988509	0.851669643	0.98250489
IL1RAP	711.0417549	0.026260026	0.140542707	0.186847305	0.851780356	0.982519343
LOC100129484	8.962951021	0.154147435	0.825059552	0.186831889	0.851792443	0.982519343
DIRC3-AS1	7.486281156	0.133563381	0.715409518	0.186695002	0.851899775	0.982579546
TMEM167B	849.1046874	0.044118452	0.236669106	0.186414073	0.852120057	0.982704329
PPME1	380.7136999	-0.025097843	0.134649413	-0.186394	0.852135798	0.982704329
C19orf47	239.3571447	0.030839781	0.165497371	0.186346046	0.8521734	0.982704329
ISM1-AS1	2.673251999	-0.226825892	1.218021793	-0.186224822	0.852268459	0.982734393
SLC7A7	135.2485453	-0.073615975	0.395556174	-0.186107512	0.852360451	0.982734393
MEIOC	55.12312869	0.062040564	0.333368925	0.18610182	0.852364915	0.982734393
MSI1	79.80386169	0.044086828	0.23715389	0.185899663	0.852523446	0.982748559
P4HA3	95.34766728	0.057169381	0.307725617	0.185780376	0.852616994	0.982748559
DMD	114.3669754	-0.048419446	0.260847059	-0.185623892	0.852739716	0.982748559
MIR4512	4.735648068	0.199095895	1.072660302	0.185609456	0.852751037	0.982748559
ST3GAL2	635.9839196	-0.025736504	0.138689453	-0.185569292	0.852782537	0.982748559
GUCY1A2	47.48762731	0.080593892	0.434342612	0.18555373	0.852794741	0.982748559
CDH6	1470.237736	0.125095529	0.674243999	0.185534509	0.852809816	0.982748559
ADAM33	297.1878775	0.060992286	0.328889851	0.185448976	0.852876897	0.982748559
FGF7P6	3.65239354	-0.213416562	1.150940354	-0.185427995	0.852893352	0.982748559
SMPDL3B	5.383214961	0.162391759	0.87712695	0.185140542	0.853118804	0.982748559
LOC728554	395.28694	-0.026701485	0.144243574	-0.185113862	0.853139729	0.982748559
KCNJ10	5.681930063	0.158640764	0.857537351	0.184995748	0.85323237	0.982748559
HCCS	217.7908415	-0.037958931	0.205306282	-0.184889284	0.853315876	0.982748559
ADAM20	4.260591854	0.192218011	1.039756856	0.184868231	0.853332389	0.982748559
ABHD16B	66.89499101	0.065316746	0.353402434	0.184822569	0.853368205	0.982748559
PLAC8L1	23.60554668	0.094703981	0.512836467	0.184667017	0.853490217	0.982748559
SLCO6A1	24.84752383	0.071946505	0.389613073	0.184661424	0.853494605	0.982748559
EIF2B3	397.4695823	0.035914121	0.194645417	0.18451049	0.853612999	0.982748559
HYAL3	55.53712475	-0.054051558	0.293007985	-0.184471281	0.853643755	0.982748559
FBLL1	2.630497084	-0.254338331	1.379149946	-0.184416736	0.853686542	0.982748559
ARHGEF1	1747.931865	0.018127285	0.098328621	0.1843541	0.853735675	0.982748559
SNHG16	3702.819977	-0.026310322	0.142722211	-0.184346371	0.853741739	0.982748559

RNF31	844.7401417	-0.026882789	0.145860204	-0.184305169	0.853774059	0.982748559
C1QL1	4.922482697	-0.161108352	0.874319248	-0.184267191	0.853803851	0.982748559
MIR3176	20.97935864	0.141535943	0.768123844	0.184261879	0.853808018	0.982748559
NKAPP1	23.09758562	0.104817713	0.568863857	0.184257994	0.853811066	0.982748559
MIR5186	2.528281661	0.219410056	1.191560155	0.184136785	0.853906149	0.982774006
ZNF660	31.05992669	0.064710266	0.351515805	0.184089207	0.853943474	0.982774006
CDRT1	2.92267493	-0.215970763	1.173792174	-0.183994039	0.854018132	0.982791667
PPOX	204.9525468	-0.029873194	0.162436595	-0.183906795	0.854086574	0.982791667
ZSCAN26	293.0847496	0.024918543	0.135554667	0.183826521	0.85414955	0.982791667
CEP350	2007.846889	-0.018419281	0.100275428	-0.18368688	0.854259102	0.982791667
VPS13C	4070.634824	0.015881547	0.08648198	0.183639959	0.854295914	0.982791667
SVEP1	300.0297285	0.048517057	0.264323456	0.18355184	0.854365047	0.982791667
CEBPB	531.1656823	-0.056246902	0.306442925	-0.183547727	0.854368274	0.982791667
GBGT1	17.63079653	0.104223977	0.568096633	0.183461705	0.854435764	0.982791667
SPIN3	193.4070989	-0.047748317	0.260359495	-0.183393801	0.854489039	0.982791667
MIR6516	5.902430246	0.195968073	1.069443496	0.183243036	0.854607328	0.982791667
CCT2	3052.06835	-0.02966308	0.161889584	-0.183230319	0.854617306	0.982791667
ICAM3	152.4641141	0.047046758	0.256845829	0.183171196	0.854663694	0.982791667
PLA2R1	216.0024179	0.037647555	0.205705051	0.183017166	0.85478455	0.982791667
NDUFV2	517.1387201	0.023987162	0.131092551	0.182978829	0.85481463	0.982791667
SYCE2	25.75153564	0.075162928	0.41080389	0.182965472	0.85482511	0.982791667
DLX2	16.79834828	-0.084697567	0.462971339	-0.182943435	0.854842402	0.982791667
BHLHE40-AS1	2.083629643	0.278613285	1.523978416	0.182819705	0.854939487	0.982791667
FBXL17	363.4715177	0.046952918	0.257000376	0.182695911	0.855036624	0.982791667
SLCO1A2	4.605465651	0.168413285	0.921925503	0.18267559	0.85505257	0.982791667
GLIS2-AS1	5.807532929	0.180263412	0.986859119	0.182663775	0.855061841	0.982791667
LINC02367	3.81456628	-0.184778468	1.012337578	-0.182526533	0.855169534	0.982852054
FEM1B	2541.983953	-0.024774253	0.135956235	-0.182222263	0.855408302	0.982860669
RNASEH1-AS1	74.05099072	0.043937842	0.24138425	0.182024479	0.855563515	0.982860669
FLACC1	11.36681645	-0.119750429	0.657968532	-0.182000238	0.855582539	0.982860669
WDR20	319.817012	0.02686191	0.147645046	0.181935736	0.855633159	0.982860669
MSANTD1	2.196985846	-0.24802931	1.363533882	-0.181901832	0.855659767	0.982860669
SHMT1	436.1342632	-0.020329743	0.111785585	-0.181863727	0.855689671	0.982860669
CSPP1	502.7593798	0.04806505	0.264318445	0.181845235	0.855704184	0.982860669
TRMT44	133.0831629	0.042470904	0.233593656	0.181815313	0.855727667	0.982860669
POLR2K	574.7954098	0.022766101	0.125257082	0.181755	0.855775001	0.982860669
SPATA45	1.818949926	-0.323441431	1.779560868	-0.181753508	0.855776172	0.982860669
ZFPL1	121.7645869	-0.045640789	0.251126974	-0.181743872	0.855783734	0.982860669
MRPL41	237.0308908	0.033646958	0.185230376	0.181649244	0.855858	0.982882617
TRAFD1	791.4023219	0.025685655	0.141461361	0.181573646	0.855917332	0.982887412
ABCA10	5.943372549	-0.157919583	0.870109219	-0.181493978	0.85597986	0.982895876
IFTAP	58.96099556	-0.057289624	0.315889157	-0.181359895	0.856085096	0.982953377
SNORD116-24	2.69687422	0.226067741	1.247915359	0.181156309	0.856244887	0.982971859
SSU72	798.5059242	-0.026119161	0.144272745	-0.181040163	0.856336051	0.982971859
PITPNC1	136.0314623	0.06905194	0.381886561	0.180817938	0.856510483	0.982971859
PRDM2	898.4599662	0.020260107	0.112053265	0.180807822	0.856518423	0.982971859
DOCK9-DT	7.913619745	-0.129609773	0.717122135	-0.180735982	0.856574814	0.982971859
SEMA3E	230.546291	0.02855068	0.158002192	0.180697999	0.856604629	0.982971859
KIAA1614	199.1715142	-0.040525692	0.22435832	-0.180629326	0.856658535	0.982971859
YBX2	6.227106469	-0.137867557	0.763657107	-0.180535945	0.856731838	0.982971859
WDR37	248.3186193	0.026674519	0.147778834	0.180502972	0.856757721	0.982971859
MATN2	1208.735601	0.042183717	0.233766095	0.180452674	0.856797205	0.982971859
LOC101928424	4.319881713	0.15823687	0.876958736	0.180438216	0.856808555	0.982971859
METTL1	243.4570728	0.03054921	0.169376875	0.18036234	0.856868118	0.982971859
SNORD70	17.5870296	0.122130401	0.677513258	0.180262747	0.8569463	0.982971859
RBBP5	1090.230871	0.021960883	0.121861989	0.180211103	0.856986842	0.982971859
BCCIP	642.749071	0.033016447	0.183228728	0.180192521	0.85700143	0.982971859
TMUB2	482.3612512	-0.026414658	0.146638474	-0.180134568	0.857046925	0.982971859
TMEM250	1383.151799	-0.02543175	0.141233348	-0.180069019	0.857098384	0.982971859
LOC101927040	4.958709847	-0.159759211	0.887657481	-0.179978442	0.857169493	0.982971859
UST	243.2632577	-0.038953098	0.21651859	-0.179906485	0.857225984	0.982971859
CDK17	1039.529908	-0.017850081	0.099329015	-0.179706618	0.857382898	0.982971859
RDX	1601.410775	-0.033296966	0.185317203	-0.179675529	0.857407306	0.982971859
NEMP2	330.1231208	-0.030239889	0.168306706	-0.179671328	0.857410604	0.982971859
ARHGEF33	8.8744977	0.123560012	0.687785566	0.179649033	0.857428108	0.982971859
LINC00310	3.975538431	-0.208739044	1.162031659	-0.179632838	0.857440823	0.982971859
CCDC87	8.6500088	0.116701197	0.6498467	0.179582657	0.857480221	0.982971859
MYD88	104.7653755	0.053382269	0.297532982	0.179416309	0.857610827	0.983058339
PURB	1441.290135	-0.020773611	0.115883823	-0.179262391	0.857731676	0.983133625
DPY19L4	1802.308885	0.01744501	0.097361716	0.179177307	0.857798482	0.98314696
MRPS24	12.68001615	0.103557123	0.579158325	0.178806241	0.858089845	0.983147176
CRELD1	518.2329419	-0.037765872	0.211417794	-0.178631472	0.858227081	0.983147176
LOC102724579	8.049714461	0.203678463	1.140363186	0.178608416	0.858245186	0.983147176
PSMD6	539.8260335	0.025602732	0.143355397	0.178596218	0.858254764	0.983147176
UBE4A	1824.947336	-0.01578479	0.088385627	-0.178590008	0.858259641	0.983147176
RHOV	4.136067752	0.216500727	1.213019237	0.178480868	0.858345345	0.983147176
CMPK2	20.12024706	0.1177394	0.659783529	0.178451559	0.858368361	0.983147176
ZNF285	129.1867746	0.032369867	0.181422838	0.178422226	0.858391396	0.983147176
CACHD1	302.9823701	0.023952846	0.134249778	0.178420006	0.858393139	0.983147176
PET100	51.0675399	-0.050480226	0.282932477	-0.178417925	0.858394773	0.983147176
ADAMTS19-AS1	2.402448394	-0.239043174	1.339896553	-0.178404201	0.85840555	0.983147176
ADD3	747.6570216	0.059016897	0.331258386	0.178159706	0.858597554	0.983303882
LCT	10.60435821	0.11762242	0.661458449	0.177822839	0.85886211	0.983410436
ESRP1	90.69621122	0.041338216	0.232658104	0.177677954	0.8589759	0.983410436
LENG1	39.40330382	0.056894523	0.320645014	0.17743773	0.859164573	0.983410436
PMP2	3.920079691	0.172128132	0.970281522	0.177400196	0.859194053	0.983410436
TTC38	242.8188999	0.024336597	0.137185996	0.177398552	0.859195345	0.983410436
VN1R1	6.287186592	0.15461981	0.87170322	0.177376665	0.859212535	0.983410436
ERMARD	238.4224506	0.03508203	0.197920287	0.177253331	0.859309407	0.983410436
SMAD4	2677.101716	-0.031712984	0.178922094	-0.177244653	0.859316223	0.983410436

SNORD160	3.402574103	-0.198657455	1.121340536	-0.177160683	0.859382178	0.983410436
KANSL1-AS1	4.047852892	-0.173142939	0.977737318	-0.177085334	0.859441362	0.983410436
CORO2B	71.11659028	-0.118587779	0.670053376	-0.176982586	0.859522068	0.983410436
FAM74A3	2.658849051	-0.222967899	1.259978204	-0.176961711	0.859538465	0.983410436
ABCA13	13.74149147	-0.112674369	0.636742815	-0.176954284	0.859544299	0.983410436
LOC112267983	14.75009502	-0.089504632	0.50590512	-0.176919799	0.859571386	0.983410436
ANKFY1	2161.957596	-0.023007362	0.130124017	-0.17681103	0.859656825	0.983410436
MRAS	313.8643499	-0.032727003	0.185105324	-0.176802063	0.859663869	0.983410436
RNF167	926.9274193	0.020951673	0.118522006	0.176774541	0.859685487	0.983410436
TMEM147	499.5919545	0.027408285	0.155114244	0.176697407	0.859746077	0.983410436
FGF2	212.1262235	0.047314191	0.267967676	0.176566786	0.859848685	0.983410436
GHET1	6.628425939	0.152262105	0.862452865	0.176545422	0.859865467	0.983410436
SPSB3	386.7514763	0.027487282	0.155856586	0.176362657	0.860009041	0.983410436
C1RL	287.74576	-0.054248406	0.307631496	-0.176342172	0.860025132	0.983410436
FAXDC2	123.4541663	-0.045650735	0.258960752	-0.176284378	0.860070534	0.983410436
KIF13B	1358.302782	0.031377843	0.178008617	0.176271481	0.860080666	0.983410436
HEYL	13.79553621	-0.090808599	0.515365072	-0.176202471	0.860134879	0.983410436
KPNA1	1582.267844	-0.017534388	0.099538865	-0.176156202	0.860171228	0.983410436
PATZ1	673.0036659	-0.019146804	0.108728167	-0.176097921	0.860217014	0.983410436
POP1	1057.406419	0.055827549	0.317068647	0.176074014	0.860235796	0.983410436
RNF121	290.3309895	-0.027749799	0.157829254	-0.175821646	0.860434063	0.983478922
TNFRSF9	250.9169041	-0.138853376	0.789753346	-0.175818662	0.860436408	0.983478922
PROCR	337.5269005	-0.044617652	0.253816545	-0.175787013	0.860461273	0.983478922
SLC25A39	1052.910595	-0.024878863	0.141590123	-0.175710444	0.86052143	0.9834846
FLJ32255	4.347555999	0.167938527	0.958001768	0.175300852	0.860843242	0.983645348
LINC00601	1.931838884	-0.23634537	1.350026816	-0.175067167	0.861026857	0.983645348
TMEM203	522.8571834	-0.027423021	0.156647308	-0.175062191	0.861030767	0.983645348
PGR	179.8161732	0.034216413	0.195498994	0.175020918	0.861063197	0.983645348
DNASE1L1	381.8295605	0.057785183	0.330164933	0.175019141	0.861064593	0.983645348
SLC6A8	954.644883	-0.030596203	0.174957209	-0.174878208	0.861175334	0.983645348
NCAPH	995.5423465	0.021118601	0.120772762	0.174862285	0.861187846	0.983645348
FTSJ3	928.7958098	-0.026700465	0.152850128	-0.174683955	0.861327976	0.983645348
SPATA9	15.00556126	-0.102363915	0.58610111	-0.174652314	0.861352839	0.983645348
RALGAPA2	236.9863388	-0.026503172	0.151774437	-0.174622104	0.861376579	0.983645348
LOC728755	43.6855623	0.056838331	0.325824672	0.174444529	0.861516122	0.983645348
MAP3K20	1239.878136	-0.028395055	0.162872977	-0.174338653	0.861599323	0.983645348
PLEKHM1	270.7874094	-0.028062246	0.160969486	-0.174332703	0.861603999	0.983645348
ARMCX1	386.3836255	-0.04476977	0.25689236	-0.174274432	0.861649792	0.983645348
ZNF649-AS1	5.381191197	-0.167686711	0.962205089	-0.174273357	0.861650637	0.983645348
VCAN-AS1	4.275378103	-0.255928962	1.469608462	-0.174147719	0.861749372	0.983645348
CLIP1-AS1	7.532957834	-0.238885595	1.372032147	-0.174110786	0.861778397	0.983645348
GNG5	105.3560918	0.045835911	0.263328624	0.174063533	0.861815532	0.983645348
SIPA1L2	139.2266217	0.050624928	0.290919108	0.174017199	0.861851946	0.983645348
MRPL43	249.3758851	-0.027731142	0.159582163	-0.173773444	0.862043515	0.983645348
TMEM37	4.104530832	0.174472164	1.004272879	0.173729837	0.862077787	0.983645348
SYNE4	9.96621301	0.111530827	0.641982089	0.173728877	0.862078542	0.983645348
STON2	203.4893188	0.043119872	0.24824056	0.173701961	0.862099696	0.983645348
CELF3	5.585469083	0.173551195	0.999453218	0.173646141	0.862143567	0.983645348
CBY1	157.6417307	-0.040507769	0.233281739	-0.173643119	0.862145943	0.983645348
MSH2-OT1	3.021735092	-0.179879811	1.036815337	-0.173492621	0.862264227	0.983645348
MAPK13	27.80653119	-0.064867825	0.373948942	-0.173467064	0.862284314	0.983645348
ARNTL2	370.7451199	-0.037653555	0.217133773	-0.173411786	0.862327762	0.983645348
MICAL1	905.731973	0.036579901	0.210987905	0.173374399	0.862357147	0.983645348
NBPF4	59.8226209	0.048107447	0.277602275	0.173296299	0.862418533	0.983645348
TAPT1	232.8256892	-0.029855886	0.172342662	-0.17323561	0.862466234	0.983645348
TRMT9B	159.1079921	0.045451216	0.262680737	0.173028357	0.862629138	0.983645348
HOXC11	103.1148659	-0.035459045	0.204932844	-0.173027633	0.862629707	0.983645348
RNF217-AS1	6.230065075	0.150804623	0.872022416	0.172936636	0.862701234	0.983645348
CORO1A	31.56708667	-0.066874393	0.3869457	-0.172826297	0.862787966	0.983645348
HOMER3-AS1	9.035679969	0.118076992	0.683246362	0.172817594	0.862794807	0.983645348
ATG5	584.9321381	0.023096292	0.133663194	0.1727947	0.862812803	0.983645348
KIAA0319L	796.0946871	-0.023109287	0.133794053	-0.172722828	0.862869299	0.983645348
KCNQ5-AS1	10.69776959	0.138233688	0.800518053	0.172680288	0.862902739	0.983645348
LOC728485	5.560266442	0.160260624	0.928343867	0.17263067	0.862941743	0.983645348
ACSF2	195.6799122	-0.053489902	0.309930051	-0.172587013	0.862976061	0.983645348
ANKLE1	90.78164071	-0.036659935	0.212421066	-0.172581447	0.862980436	0.983645348
CHST7	231.5244469	0.033128801	0.192119813	0.172438232	0.863093018	0.983660528
INAVA	17.75891729	-0.075571954	0.43829122	-0.172424066	0.863104153	0.983660528
KIF18A	447.7378472	-0.024333378	0.141294353	-0.172217626	0.863266441	0.983782566
LINC02669	42.25244289	0.118914385	0.691275228	0.172021765	0.863420418	0.983797097
ZNF653	61.89413782	0.044347727	0.258016954	0.171879118	0.863532563	0.983797097
OAS2	37.25780648	0.135668551	0.790424884	0.17164003	0.863720535	0.983797097
SVBP	18.3218666	-0.075023598	0.437203796	-0.171598689	0.863753038	0.983797097
ARSD	789.113624	0.046727391	0.27236232	0.171563345	0.863780827	0.983797097
FBXO15	8.726884406	0.130668047	0.762567573	0.171352745	0.863946409	0.983797097
RASSF1	173.7137383	-0.033414644	0.195134154	-0.171239343	0.864035573	0.983797097
CYP1B1	4.407547275	0.249605097	1.457988552	0.171198256	0.864067879	0.983797097
DNAJC28	5.216196078	0.174853858	1.021489826	0.17117533	0.864085905	0.983797097
LOC441052	12.12043944	-0.09578205	0.559700288	-0.171130965	0.864120789	0.983797097
CLDN23	3.886171656	-0.158639105	0.927442307	-0.171050106	0.864184367	0.983797097
KLHL23	14.50787188	0.097199841	0.568300559	0.171035976	0.864195478	0.983797097
CRIP3	7.245864391	0.115970896	0.678201358	0.170997734	0.864225547	0.983797097
DNAJC9-AS1	32.92238785	0.064604449	0.377830454	0.170987935	0.864233253	0.983797097
PTBP2	1021.986723	0.049888975	0.291773146	0.170985492	0.864235173	0.983797097
ZDHHC11	12.6293993	0.088852024	0.5196962	0.170969162	0.864248013	0.983797097
ZNF773	131.333529	0.030899917	0.1808042	0.170902652	0.864300311	0.983797097
CCDC28B	99.56318981	0.052235961	0.305852267	0.17078821	0.8643903	0.983797097
PPP1R36	5.925192159	-0.130139598	0.762623505	-0.170647243	0.864501148	0.983797097
KIF3B	683.8465685	0.020774758	0.121764865	0.17061373	0.864527501	0.983797097
SMIM20	149.9278596	-0.04451572	0.26103046	-0.170538412	0.864586728	0.983797097

FAM53B-AS1	4.956858672	0.187046415	1.097531191	0.170424692	0.864676155	0.983797097
BAGE	6.273705916	-0.132216259	0.776460396	-0.17028075	0.864789349	0.983797097
MRPL14	553.3338165	0.021970381	0.129188915	0.17006398	0.86495982	0.983797097
UBQLN4	1007.16436	-0.021671663	0.127449202	-0.170041575	0.864977441	0.983797097
USP34	5614.611254	0.014198248	0.08353756	0.169962441	0.865039675	0.983797097
NKX3-2	42.39540725	0.100059042	0.588784196	0.169941793	0.865055913	0.983797097
DGCR5	6.316334412	0.14817335	0.87232645	0.169859976	0.865120258	0.983797097
STYK1	34.76015111	-0.073871002	0.43563269	-0.169571759	0.865346934	0.983797097
GALNT7	531.3275431	0.026733164	0.157759199	0.169455502	0.865438371	0.983797097
SERPIND1	9.61153548	-0.182525447	1.077211595	-0.16944252	0.865448581	0.983797097
AQP7P1	3.115908478	-0.194330643	1.147006598	-0.169424171	0.865463012	0.983797097
FNDC7	2.59143886	-0.238312728	1.406778855	-0.169403121	0.865479569	0.983797097
AKAP8L	627.0532415	0.021028191	0.124169809	0.16935027	0.865521137	0.983797097
SAGE1	3.818192095	-0.161931608	0.95670548	-0.169259622	0.865592435	0.983797097
WASH7P	165.9973945	0.030588283	0.180893036	0.169095969	0.865721156	0.983797097
GAS6-AS1	4.917137552	0.146607818	0.867115607	0.169075285	0.865737425	0.983797097
B3GALNT1	745.2088208	0.027466749	0.162453583	0.169074445	0.865738085	0.983797097
ACTG1P17	11.59232662	0.098995937	0.585568175	0.169059626	0.865749741	0.983797097
NOP56	2003.756272	-0.041082902	0.243039973	-0.169037634	0.865767039	0.983797097
CNOT6	1445.897257	-0.019242002	0.113843135	-0.169022066	0.865779285	0.983797097
TMLHE	153.3161802	0.043517393	0.257542047	0.168971994	0.86581867	0.983797097
PLEKHO1	456.7616667	-0.023243167	0.137573649	-0.168950718	0.865835406	0.983797097
SLC4A8	7.786569848	0.155828107	0.922734735	0.168876386	0.865893873	0.983797097
ABHD11	73.57935629	0.057421508	0.34008602	0.168844071	0.865919292	0.983797097
LOC105373311	14.34561538	0.088885835	0.527153213	0.168614803	0.866099636	0.983797097
TSPY26P	31.94305816	-0.067877372	0.402615745	-0.168590953	0.866118397	0.983797097
MARCHF7	1362.621008	-0.01996948	0.118486352	-0.168538228	0.866159872	0.983797097
NEDD8	637.3628854	-0.027081938	0.16072519	-0.168498407	0.866191197	0.983797097
COL6A6	18.48263138	0.14762056	0.876926369	0.168338603	0.866316906	0.983797097
PROSER1	574.9836984	-0.023603185	0.140238373	-0.168307611	0.866341287	0.983797097
C11orf80	85.58897753	-0.053188753	0.316027896	-0.168303982	0.866344142	0.983797097
MGC16025	3.006567806	-0.24104238	1.432226431	-0.168299073	0.866348003	0.983797097
SNX9	1236.074474	0.036710063	0.218168558	0.168264681	0.866375059	0.983797097
CDH15	6.259247348	0.162755315	0.96726996	0.168262556	0.86637673	0.983797097
SNORD17	81.92329177	-0.069051166	0.410507528	-0.168209255	0.86641866	0.983797097
LOC644634	130.1339681	0.040490986	0.240842659	0.168122152	0.866487183	0.983797097
TMEM190	3.195624707	0.197212759	1.173105766	0.168111661	0.866495435	0.983797097
SCMH1	450.1891282	-0.025770836	0.153343483	-0.168059548	0.866536433	0.983797097
TMEM92-AS1	9.971092094	0.112091947	0.668053779	0.167788807	0.866749428	0.983976225
ZNF607	236.0895214	-0.027606808	0.164711638	-0.167606908	0.866892536	0.984075996
MGAT1	1123.99692	0.020208895	0.120795055	0.167299027	0.86713477	0.984193893
ZNF830	178.4129486	0.030165947	0.180439943	0.167179985	0.867228433	0.984193893
ADAMTSL5	21.08441741	-0.079271527	0.47425816	-0.167148473	0.867253226	0.984193893
PCDHGA6	11.16534701	0.101587343	0.608081106	0.167062159	0.86732114	0.984193893
LEPR	80.40744353	-0.096030375	0.574878982	-0.167044504	0.867335031	0.984193893
SHFL	129.4247594	0.050459052	0.302115149	0.167019272	0.867354885	0.984193893
MADD-AS1	4.819687826	0.149799707	0.897332894	0.166938833	0.867418177	0.984193893
LINC01249	2.813097584	-0.188939462	1.132599867	-0.166819252	0.86751227	0.984193893
FOXP1-AS1	7.159161763	-0.176797535	1.05996415	-0.166795769	0.867530748	0.984193893
FLG	963.6261191	0.039448975	0.236670166	0.166683343	0.867619212	0.984193893
SPG21	1551.895664	0.028383998	0.170390178	0.166582362	0.867698673	0.984193893
AKR7L	10.51423196	-0.13118008	0.787756348	-0.166523672	0.867744856	0.984193893
CRTC2	225.9519947	-0.028945765	0.17398367	-0.166370583	0.867865323	0.984193893
CEP162	196.0696043	-0.049885181	0.300096111	-0.166230681	0.867975416	0.984193893
STAG3L3	15.27909012	0.093960722	0.565274066	0.166221533	0.867982615	0.984193893
MRPL51	366.3007328	-0.027591541	0.166004197	-0.166209899	0.86799177	0.984193893
ADCK1	148.1392821	-0.029740512	0.178981491	-0.166165295	0.86802687	0.984193893
NKX2-2	7.537318599	0.138858483	0.835696202	0.166159046	0.868031788	0.984193893
MBLAC2	232.8400844	-0.029456476	0.177303543	-0.166135858	0.868050036	0.984193893
TIMP4	7.687492713	-0.12619974	0.760741274	-0.165890486	0.868243135	0.984193893
MLLT1	1156.677045	-0.021120051	0.127353821	-0.165837592	0.868284761	0.984193893
RAB42	100.8704355	-0.043720158	0.263684648	-0.165804717	0.868310634	0.984193893
TSC2	1376.526615	-0.018679814	0.112669281	-0.165793317	0.868319606	0.984193893
PCDHB9	56.75209284	0.063043643	0.380311202	0.165768566	0.868339084	0.984193893
LINC00397	36.81593226	0.086074075	0.519964305	0.16553843	0.868520204	0.984193893
FAM222A	11.03054959	-0.103868045	0.627742154	-0.165462913	0.868579638	0.984193893
UBAC1	351.2085497	-0.022303973	0.134924996	-0.16530646	0.868702775	0.984193893
TMEM131L	339.0571206	0.027734029	0.16785898	0.165222195	0.868769096	0.984193893
PIP4P1	325.8861766	0.024379365	0.147556362	0.165220696	0.868770276	0.984193893
CLPB	678.2700604	0.017043763	0.103175231	0.165192394	0.868792552	0.984193893
MIR3175	8.283724235	0.137504304	0.832894851	0.165092032	0.868871544	0.984193893
NOX1	3.943448799	0.158911908	0.963420043	0.164945611	0.868986792	0.984193893
ARL6IP1	1896.101221	-0.021159531	0.128333632	-0.164879081	0.869039158	0.984193893
PPP1CC	5138.987756	0.023684787	0.143665749	0.164860356	0.869053896	0.984193893
TMBIM1	1286.188972	0.034459775	0.209026308	0.164858555	0.869055315	0.984193893
RGPD3	25.3109338	0.064256318	0.389971152	0.164771978	0.869123461	0.984193893
CNIH2	21.41896905	-0.085428012	0.518649934	-0.164712278	0.869170453	0.984193893
PRPF39	803.4948129	-0.037458175	0.227488091	-0.164659938	0.869211651	0.984193893
TSPAN7	51.94565383	0.070387563	0.427622608	0.164602062	0.869257208	0.984193893
GEMIN8P4	9.178573418	-0.124473185	0.756507391	-0.164536641	0.869308704	0.984193893
EGF	19.33946453	0.073954192	0.449571587	0.164499258	0.869338131	0.984193893
LOC101927557	3.192838881	0.202933601	1.235162697	0.164297061	0.869497295	0.984193893
ZNF700	263.8490993	0.035792695	0.217976752	0.164204187	0.869570405	0.984193893
ADCY1	74.01634677	-0.048667907	0.296411681	-0.164190245	0.86958138	0.984193893
RGS12	284.039571	0.041163703	0.250709172	0.164189061	0.869582312	0.984193893
IZUMO3	21.64985634	-0.129288532	0.788244117	-0.164020928	0.869714669	0.984193893
UCHL5	1121.411716	0.03186501	0.194299197	0.1639997	0.86973138	0.984193893
CTU1	220.9367622	0.028560312	0.174178722	0.1639713	0.869753737	0.984193893
VWA5A	2.736160731	-0.224721162	1.371070967	-0.163901918	0.869808357	0.984193893
LOC145694	30.56992797	0.078650984	0.479891779	0.163893167	0.869815246	0.984193893

WIPF2	790.3119489	0.027818901	0.169771355	0.163860985	0.869840582	0.984193893
LMF1	6.34023411	-0.139727472	0.852989733	-0.16380909	0.869881436	0.984193893
CHODL	15.2332535	0.075777263	0.462746171	0.16375557	0.869923569	0.984193893
HIVEP2	748.0548005	0.026363053	0.16110191	0.16364209	0.870012908	0.98423248
CSAG3	4.292920517	0.171117714	1.04627463	0.163549521	0.870085786	0.984252441
CNOT8	739.3404227	0.02955506	0.180835835	0.16343586	0.87017527	0.984253049
LOC102546299	13.77702663	0.167497791	1.025416779	0.163346061	0.87024597	0.984253049
SIRT1	414.3887649	-0.022014812	0.134833899	-0.163273567	0.870303045	0.984253049
DIABLO	502.2150768	0.024424855	0.149599569	0.163268219	0.870307256	0.984253049
WFIKKN1	15.37605937	0.101201974	0.620220753	0.163170892	0.870383884	0.984277244
NDUFB5	1908.937019	-0.024967429	0.153134511	-0.163042468	0.870484998	0.984278306
EMC8	336.7154827	0.025109818	0.154020187	0.163029393	0.870495292	0.984278306
CCDC110	8.508318876	-0.141776866	0.871209609	-0.162735654	0.870726573	0.984477351
OGFRP1	6.839508994	0.138293424	0.850993477	0.162508207	0.870905666	0.984529341
NEURL1B	81.93307388	0.099898845	0.615040148	0.162426543	0.87096997	0.984529341
LOC101927824	5.261568741	-0.158942922	0.979182161	-0.162322118	0.871052198	0.984529341
PIEZO1	3760.241801	-0.027886009	0.171806294	-0.162310752	0.871061148	0.984529341
HAGHL	412.8812056	0.034419469	0.212476757	0.16199169	0.871312398	0.984529341
CRYZ	447.7298982	0.029855966	0.184317414	0.161981256	0.871320614	0.984529341
FGR	4.549582468	0.223569793	1.380357524	0.161965135	0.871333309	0.984529341
ELOVL2	32.64919179	-0.056322773	0.34785066	-0.161916534	0.871371583	0.984529341
CEP95	488.8632364	-0.039618061	0.244767879	-0.161859721	0.871416322	0.984529341
RABEPK	431.0905129	-0.024756806	0.153043216	-0.161763496	0.8714921	0.984529341
CWC15	168.1603011	-0.028734852	0.177657702	-0.161742789	0.871508407	0.984529341
BMP8A	11.46139115	0.102424456	0.633866848	0.161586706	0.871631327	0.984529341
OR51E2	7.506787212	-0.174943101	1.083376171	-0.161479554	0.871715714	0.984529341
KCNK7	2.995663248	-0.192463918	1.192012286	-0.161461354	0.871730047	0.984529341
MTMR3	835.977737	-0.021508456	0.133284681	-0.161372304	0.871800179	0.984529341
NPR1	7.302973796	0.16438307	1.019575646	0.161226948	0.871914657	0.984529341
SPIN2B	32.73361774	0.055473772	0.34411673	0.161206264	0.871930948	0.984529341
ZC4H2	181.0248307	-0.029581666	0.183511347	-0.161198023	0.871937438	0.984529341
EXOSC4	286.7598226	0.027279879	0.169281505	0.161150972	0.871974495	0.984529341
MAGED1	4953.740762	-0.024837723	0.15415021	-0.161126755	0.871993568	0.984529341
LOC730234	2.040151009	0.245049645	1.520873497	0.161124279	0.871995518	0.984529341
NDUFAF6	310.3613182	0.035837293	0.222538922	0.161038314	0.872063224	0.984529341
PDCD5	219.1992513	-0.033054906	0.205356453	-0.160963562	0.872122099	0.984529341
ZNF875	328.3751019	0.030465389	0.189304226	0.160933487	0.872145787	0.984529341
RAD51AP1	327.8038639	-0.030304269	0.188314927	-0.160923352	0.87215377	0.984529341
RGPD4	18.53087486	-0.08225654	0.511433841	-0.160835152	0.872223238	0.984545392
ABI2	1551.488821	-0.015082445	0.093843245	-0.160719564	0.87231428	0.984585791
AKAP11	3235.078499	-0.033276492	0.207351656	-0.160483366	0.872500324	0.984658354
ZNF570	154.9397096	-0.035010668	0.218283944	-0.160390486	0.872573484	0.984658354
ZNF548	226.0716832	0.026241111	0.163642844	0.160355993	0.872600654	0.984658354
SRP68	1193.675665	-0.020012098	0.124847244	-0.160292671	0.872650532	0.984658354
TMEM274P	13.54142525	0.110261295	0.688083424	0.16024408	0.872688808	0.984658354
SYTL4	86.62166765	0.045960453	0.28686369	0.160217045	0.872710103	0.984658354
TOMM40L	526.7449289	-0.024500772	0.153098451	-0.160032788	0.872855246	0.984681886
KIAA0586	838.637442	-0.020344065	0.12719361	-0.15994565	0.872923888	0.984681886
CYP27C1	79.79455772	0.062525283	0.391053241	0.159889437	0.872968169	0.984681886
IPW	343.2213678	-0.051604054	0.322880082	-0.159824209	0.873019553	0.984681886
TRIP11	1110.936998	0.019857145	0.124414163	0.159605183	0.873192096	0.984681886
SDHD	1280.571876	-0.017554367	0.11001401	-0.159564834	0.873223882	0.984681886
MAPK8	340.0466812	-0.027006776	0.169418976	-0.159408213	0.873347268	0.984681886
LOC102723534	8.506611455	0.106326341	0.667199621	0.159362113	0.873383587	0.984681886
NR4A3	24.68957836	-0.074685998	0.468718079	-0.159340979	0.873400237	0.984681886
SUN2	2174.044824	-0.023190218	0.145661333	-0.159206408	0.873506255	0.984681886
RNF182	18.79293921	0.101085534	0.635116427	0.159160635	0.873542317	0.984681886
AADACL2	152.8307144	0.129900435	0.817270001	0.158944333	0.873712732	0.984681886
REV3L	1355.806187	0.017302943	0.109061849	0.158652572	0.873942607	0.984681886
MRPL2	491.8227429	-0.027345856	0.172407001	-0.15861221	0.873974408	0.984681886
BTRC	282.2652611	0.024293039	0.153181748	0.158589644	0.873992188	0.984681886
PCDHB8	137.1787374	0.031157697	0.19649135	0.158570324	0.874007411	0.984681886
MTG1	512.1450804	0.04233037	0.267234572	0.15840155	0.874140392	0.984681886
SUCLG2-AS1	11.30693462	0.105201988	0.66420496	0.158387839	0.874151196	0.984681886
PYGO2	758.8442521	-0.016715933	0.105562658	-0.15835082	0.874180365	0.984681886
HSPD1	9157.753061	0.02812606	0.177672631	0.158302714	0.874218269	0.984681886
PGM1	795.3077543	0.027132249	0.17148114	0.158222931	0.874281135	0.984681886
PRNCR1	12.81431768	0.140059425	0.885245727	0.158215308	0.874287142	0.984681886
MGAT5B	2.275339216	-0.217468485	1.375791927	-0.15806786	0.874403327	0.984681886
PANK2	677.5289036	-0.029848791	0.188844971	-0.158059761	0.874409708	0.984681886
RTCA-AS1	17.40200942	0.078839013	0.498799855	0.15805741	0.87441156	0.984681886
BOLA3	165.9878537	0.035700736	0.225903216	0.158035536	0.874428797	0.984681886
C16orf91	105.201869	0.035938713	0.227441004	0.158013343	0.874446285	0.984681886
HSD3BP4	23.72221471	0.080251395	0.508085404	0.157948632	0.874497276	0.984681886
ERO1B	39.36436894	-0.080606441	0.510438487	-0.157916072	0.874522933	0.984681886
KCNS1	17.37584172	-0.072053319	0.456511168	-0.157834734	0.874587028	0.984681886
HERC6	49.88420593	-0.051093664	0.323880019	-0.157754912	0.874649928	0.984681886
ARRDC2	178.9994574	-0.030983282	0.196509902	-0.15766779	0.874718583	0.984681886
IL10RB	419.5501664	-0.034034265	0.215910916	-0.157631051	0.874747534	0.984681886
SEC23A-AS1	6.074793326	-0.124243536	0.7882134	-0.157626775	0.874750904	0.984681886
MAN1A2	1679.719201	-0.015867254	0.100669834	-0.157616766	0.874758791	0.984681886
PTPRN	10.18106079	0.097364676	0.617740556	0.157614188	0.874760823	0.984681886
MAGED2	1881.889507	0.031094045	0.197383747	0.157530928	0.874826435	0.984681886
LYL1	6.387867371	-0.139952992	0.889011991	-0.157425314	0.874909664	0.984681886
LINC02860	2.649731009	0.208830414	1.326694158	0.157406598	0.874924414	0.984681886
ATP6V0E2-AS1	6.008266073	0.123371794	0.78465743	0.157230136	0.875063479	0.984681886
ARMC10	357.986994	-0.023633031	0.150390625	-0.157144311	0.875131116	0.984681886
KCNIP4	14.4628114	-0.122335992	0.778525727	-0.157138021	0.875136074	0.984681886
CDKL3	27.40430825	-0.056373607	0.358951024	-0.157050971	0.875204677	0.984681886
PWWP2B	61.88106883	-0.042199318	0.268702637	-0.157048395	0.875206708	0.984681886

PUS7	1021.448811	0.029737548	0.189504846	0.156922364	0.875306034	0.984681886
PPP1R10	1347.266096	-0.021915963	0.139798938	-0.156767736	0.875427901	0.984681886
LINC01569	27.57026253	0.076028204	0.485212088	0.156690664	0.875488645	0.984681886
MAP3K19	1.934431246	0.241303145	1.541000416	0.156588631	0.875569063	0.984681886
NPY2R	5.794853822	0.167233868	1.068211877	0.15655496	0.875595601	0.984681886
SIX3	4.428464883	-0.125931157	0.805270757	-0.156383621	0.875730647	0.984681886
DYRK3	731.184558	-0.031735983	0.20297592	-0.15635344	0.875754435	0.984681886
PRLR	9.106334771	0.109842731	0.702535532	0.156351851	0.875755688	0.984681886
DNAH17	32.64027955	-0.074718502	0.478180399	-0.156255886	0.875831327	0.984681886
ZNF487	9.173704778	0.141290413	0.904408352	0.156224135	0.875856353	0.984681886
TRMT112	274.5912744	-0.027084489	0.173396406	-0.15619983	0.875875511	0.984681886
S100A11	501.3063448	-0.022884644	0.146627975	-0.156072837	0.875975609	0.984681886
CYP24A1	94.28590345	-0.217016328	1.391004939	-0.15601406	0.876021939	0.984681886
GPR156	33.41790145	-0.071206207	0.456833788	-0.155868959	0.876136313	0.984681886
TMEM100	2.358295262	0.192539047	1.235391913	0.155852604	0.876149205	0.984681886
CRADD	112.501071	-0.040832886	0.262082427	-0.155801693	0.876189336	0.984681886
TYRO3	1464.49964	-0.019168709	0.123084477	-0.155736202	0.87624096	0.984681886
ADCK5	170.767214	-0.051508986	0.331755889	-0.155261708	0.876615002	0.984681886
LINC02568	2.276935668	-0.185342489	1.19543771	-0.155041528	0.876788579	0.984681886
RNF148	2.452839083	-0.212075643	1.368178179	-0.155005866	0.876816693	0.984681886
FOXN1	5.791800022	0.193462818	1.248426832	0.154965284	0.876848686	0.984681886
C11orf68	244.3364578	-0.045977782	0.296964845	-0.154825674	0.87695875	0.984681886
ARL17B	3.848967727	0.159222111	1.028716194	0.154777491	0.876996737	0.984681886
RAB27A	336.9444998	0.01877135	0.121318399	0.154727974	0.877035776	0.984681886
GAR1	212.9673619	0.037162742	0.240304602	0.154648482	0.877098447	0.984681886
HOXD13	425.6700941	0.023085195	0.14927584	0.154647896	0.877098909	0.984681886
MAN2A2	852.735382	-0.017693179	0.114558627	-0.154446503	0.877257689	0.984681886
SNORD142	6.300060735	-0.130287691	0.844029588	-0.154363891	0.877322823	0.984681886
SAPCD1-AS1	12.98105519	-0.109326032	0.708481165	-0.154310428	0.877364975	0.984681886
C12orf43	266.8582309	0.028126311	0.182350398	0.154243212	0.877417971	0.984681886
HOXA7	27.7449582	-0.068265351	0.442737111	-0.154189357	0.877460433	0.984681886
SPESP1	134.3791773	-0.034159579	0.221575828	-0.15416654	0.877478424	0.984681886
TTC39A	16.15748649	-0.097581323	0.633075534	-0.154138516	0.87750052	0.984681886
GNG14	6.304323837	-0.133275263	0.864936089	-0.154086834	0.877541269	0.984681886
MRPS11	377.3263939	-0.022067951	0.143444431	-0.153843201	0.87773337	0.984681886
TAS2R10	4.461386327	-0.17953601	1.16725191	-0.153810851	0.877758878	0.984681886
IFT57	186.396159	0.029781187	0.193638872	0.153797563	0.877769356	0.984681886
IFI44	226.313723	-0.024756594	0.161109417	-0.153663232	0.877875277	0.984681886
PCDHB14	41.23368797	-0.047584397	0.30972225	-0.153635709	0.87789698	0.984681886
ZNF444	277.8004873	0.028217176	0.183734842	0.153575533	0.87794443	0.984681886
B3GALT4	10.17902427	0.098540265	0.641720733	0.153556306	0.877959591	0.984681886
KDSR	999.2035095	0.039771323	0.259017938	0.153546599	0.877967245	0.984681886
MIR7161	24.69307209	0.072727088	0.47391121	0.153461421	0.878034412	0.984681886
MAMDC2	9.200542315	-0.117857493	0.768326926	-0.15339498	0.878086804	0.984681886
ABLIM3	121.1714944	-0.043803092	0.285680237	-0.153329096	0.878138757	0.984681886
GFOD1	827.9433749	0.017365893	0.113283682	0.153295626	0.87816515	0.984681886
APEH	897.5967727	-0.0153474	0.100149438	-0.153244993	0.878205077	0.984681886
ZNF561	599.7194923	0.020679401	0.135032013	0.153144433	0.878284377	0.984681886
ZNF460	1579.528837	-0.018996995	0.124122236	-0.153050703	0.878358291	0.984681886
PSMB1	977.6446609	0.019061915	0.124617649	0.152963205	0.878427292	0.984681886
TRMT13	287.208296	0.034998209	0.228873919	0.152914798	0.878465466	0.984681886
CHADL	5.082835085	-0.138653584	0.907066065	-0.15285941	0.878509146	0.984681886
CLEC18A	4.761859342	0.155655325	1.01855851	0.152819227	0.878540835	0.984681886
LOC103171574	3.693040323	-0.164757773	1.078494836	-0.1527664	0.878582496	0.984681886
PFDN2	132.2214675	0.027484253	0.180070327	0.152630662	0.878689543	0.984681886
PUSL1	268.7162289	-0.026786601	0.175536848	-0.152598165	0.878715172	0.984681886
IP6K2	438.0799546	0.035686821	0.233948352	0.152541453	0.878759899	0.984681886
SLITRK3	47.99774794	-0.045925434	0.301135529	-0.152507525	0.878786655	0.984681886
RFNG	634.1416611	0.030656258	0.201087787	0.152452113	0.878830357	0.984681886
SBSPON	41.54613026	-0.051670222	0.339514335	-0.152188632	0.87903816	0.984681886
MIR1185-1	4.70006221	0.202877254	1.333561446	0.152131913	0.879082894	0.984681886
IPO9-AS1	7.008849269	0.155239251	1.020459466	0.152126818	0.879086913	0.984681886
FAM166A	14.60881352	-0.08053132	0.529696715	-0.152032886	0.879160998	0.984681886
ANK3	415.1107805	-0.032704308	0.215176134	-0.151988549	0.879195967	0.984681886
PCYOX1	1630.015279	-0.021835966	0.143860968	-0.1517852	0.879356355	0.984681886
TAGLN	374.603962	-0.111196477	0.733261299	-0.151646455	0.879465791	0.984681886
NME6	268.0999274	0.020797136	0.1371739	0.151611465	0.87949339	0.984681886
MEPCE	356.9282504	0.021612019	0.142658263	0.151495038	0.879585225	0.984681886
PCDHGC4	15.63848657	-0.095746639	0.632078517	-0.151479027	0.879597854	0.984681886
PCGF2	265.2396057	-0.022869926	0.151052865	-0.151403455	0.879657464	0.984681886
HMBS	301.5993958	0.022431788	0.148162801	0.151399597	0.879660507	0.984681886
ARHGAP27P1-BPTFP1-KPNA2P3	141.861805	-0.039012802	0.258233434	-0.151075719	0.879915986	0.984681886
ZKSCAN7-AS1	9.373118737	-0.090369501	0.598359882	-0.151028677	0.879953094	0.984681886
SYT10	3.340147704	0.167178406	1.106941076	0.151027376	0.879954121	0.984681886
SCN5A	12.06144677	0.08146563	0.539538898	0.150991208	0.879982651	0.984681886
MUL1	590.5413234	-0.019202952	0.12718443	-0.150985083	0.879987483	0.984681886
LMF2	874.5737986	-0.023980672	0.158902541	-0.150914336	0.880043291	0.984681886
LINC02618	3.087121024	-0.175935481	1.165932393	-0.150896812	0.880057116	0.984681886
TMPRSS2	8.28996709	-0.102943799	0.682514012	-0.150830309	0.880109577	0.984681886
ZNF687	775.3759893	-0.01754836	0.11634832	-0.150826073	0.880112918	0.984681886
RASIP1	6.699287695	-0.124886193	0.828056769	-0.150818395	0.880118975	0.984681886
BATF3	26.23529841	0.077081551	0.511095598	0.150816308	0.880120621	0.984681886
FXR2	890.6062454	-0.016996205	0.112854067	-0.150603387	0.88028859	0.984681886
MCM10	416.0760964	-0.03862696	0.256573117	-0.150549522	0.880331083	0.984681886
STX5	399.7381623	0.03810909	0.253189563	0.15051604	0.880357497	0.984681886
BTC	5.530694881	-0.123493143	0.820511269	-0.150507553	0.880364192	0.984681886
SH2D7	5.767912494	0.129761825	0.862942626	0.150371324	0.880471664	0.984681886
PPP6R2	853.3173179	0.020729107	0.137872821	0.150349477	0.880488899	0.984681886
TNFRSF19	907.2420086	-0.041094523	0.273571193	-0.150215095	0.880594917	0.984681886
CKAP2	1030.098465	-0.017075983	0.113718649	-0.150159922	0.880638445	0.984681886

TAS2R5	11.50339071	0.121716726	0.811074767	0.150068441	0.880710618	0.984681886
IL1B	2.323794853	-0.231569282	1.543163556	-0.150061399	0.880716174	0.984681886
KIF5B	2881.969713	0.01721887	0.114752784	0.150051871	0.880723691	0.984681886
ZSWIM8-AS1	67.86180389	-0.054547637	0.363859563	-0.149913986	0.880832477	0.984681886
LRRC8E	81.72699333	0.034424547	0.229633762	0.149910654	0.880835106	0.984681886
RBM26	1024.164422	-0.027994295	0.186800527	-0.14986197	0.880873516	0.984681886
SURF1	317.5724193	-0.024102363	0.160975586	-0.149726824	0.880980144	0.984681886
RIC8B	492.228928	0.024242349	0.161942686	0.149697093	0.881003602	0.984681886
LOC100190986	44.49172008	0.088943526	0.594513255	0.149607305	0.881074444	0.984681886
PTGIS	205.8010714	-0.143735741	0.961030607	-0.149564166	0.881108482	0.984681886
WDR75	2051.193127	0.01952203	0.130612707	0.149465013	0.881186715	0.984681886
CD302	5.878935355	-0.115823562	0.775053368	-0.149439467	0.881206872	0.984681886
EVI2B	19.35964657	-0.129754818	0.868428841	-0.149413299	0.881227519	0.984681886
CDKN2AIPNL	222.5499549	0.027370549	0.183337334	0.149290647	0.881324295	0.984681886
PRCP	831.6287501	-0.029795497	0.199616238	-0.149263892	0.881345406	0.984681886
KLF3	1534.345035	0.040681865	0.272554102	0.149261613	0.881347205	0.984681886
PLSCR3	28.29825839	-0.05554037	0.372154922	-0.149239917	0.881364323	0.984681886
HERC3	297.4286162	-0.021002036	0.140889371	-0.14906757	0.881500316	0.984681886
IQSEC1	404.8464779	0.028403624	0.190703538	0.148941252	0.88159999	0.984681886
ECHDC2	65.35979208	0.038168436	0.256294052	0.148924393	0.881613293	0.984681886
CD37	43.41761319	-0.052244531	0.351152863	-0.148780023	0.881727215	0.984681886
TIPRL	700.2838739	-0.019900046	0.133765402	-0.14876826	0.881736496	0.984681886
FTL	2502.12401	0.034305779	0.230670898	0.148721748	0.8817732	0.984681886
MADCAM1	5.236624027	0.130337646	0.87689881	0.148634762	0.881841842	0.984681886
TMEM26	245.5677312	-0.125955213	0.847614138	-0.148599708	0.881869504	0.984681886
TMPPE	132.3692332	0.043535628	0.292996222	0.148587676	0.881878998	0.984681886
CDC42EP4	552.7542325	0.036959803	0.249179935	0.148325758	0.882085688	0.984681886
KBTBD4	129.6920496	0.028967883	0.195354569	0.148283623	0.88211894	0.984681886
STAM	258.2651351	0.025605442	0.172702285	0.148263482	0.882134834	0.984681886
RPIA	639.9889545	-0.026117241	0.176392633	-0.148063104	0.882292967	0.984681886
BTBD8	193.5745467	-0.045946749	0.310431825	-0.148009144	0.882335552	0.984681886
MID1IP1	1042.055573	-0.024957095	0.168868391	-0.147790212	0.882508334	0.984681886
CDK2	1103.151576	-0.017916187	0.121249841	-0.147762562	0.882530156	0.984681886
LINC01191	7.290860034	0.13049022	0.883321363	0.147726779	0.882558397	0.984681886
CARF	211.6935911	0.038039835	0.25751696	0.147717787	0.882565494	0.984681886
DAB1	2.828181532	-0.190135328	1.287426699	-0.147686333	0.882590318	0.984681886
IL17RE	7.262034072	0.141652305	0.959235002	0.147672161	0.882601503	0.984681886
PCSK9	58.84473358	0.215807067	1.461693259	0.14764183	0.882625442	0.984681886
SNORD21	16.08000526	-0.079891341	0.541588869	-0.147512894	0.882727203	0.984681886
TMEM185A	162.9731685	0.025456234	0.172662717	0.147433298	0.882790024	0.984681886
HSF2BP	21.02921495	0.074685567	0.50674141	0.147383983	0.882828947	0.984681886
FAAP20	400.5957786	0.018021613	0.122338663	0.147309221	0.882887954	0.984681886
CES4A	19.19022648	-0.072939339	0.495472919	-0.147211556	0.88296504	0.984681886
ASB7	458.0385702	0.023585069	0.160234538	0.147190917	0.882981329	0.984681886
LRRC57	701.3587746	-0.022688749	0.154163409	-0.147173372	0.882995178	0.984681886
CBR1	301.4596285	-0.025526853	0.173496321	-0.147131956	0.883027867	0.984681886
CD80	2.318123286	0.2120427	1.441602011	0.147088238	0.883062374	0.984681886
PUS1	645.7243066	0.030404827	0.206895443	0.14695745	0.883165605	0.984681886
ARL3	88.84164274	0.039106558	0.266262851	0.146872004	0.88323305	0.984681886
VEGFB	346.9503715	0.02856705	0.194551461	0.146835445	0.883261907	0.984681886
ATG4B	509.3495889	0.017954568	0.122277952	0.146834062	0.883262998	0.984681886
RPSAP52	26.06131273	0.079673029	0.542830744	0.146773244	0.883311004	0.984681886
GAS8	98.61224879	-0.038323856	0.261122156	-0.146766006	0.883316717	0.984681886
CRTC3-AS1	8.913564963	0.103861138	0.707740253	0.146750362	0.883329066	0.984681886
ATRIP-TREX1	6.734272307	0.154076884	1.050388308	0.146685642	0.883380152	0.984681886
KLHL33	18.31428539	-0.085914897	0.585734263	-0.146678967	0.883385421	0.984681886
ZRANB3	284.3764438	0.027005423	0.184126444	0.146667812	0.883394226	0.984681886
ARHGAP36	4.563848854	-0.137958127	0.940672325	-0.146659069	0.883401127	0.984681886
PELO	327.4831556	-0.021598661	0.147326683	-0.146603862	0.883444705	0.984681886
GALNT14	596.5202762	-0.018515772	0.126304682	-0.146596088	0.883450842	0.984681886
FAM131C	26.68781238	0.062145413	0.424681495	0.146334167	0.883657594	0.98477672
MFSD13B	2.520231942	0.207096319	1.41569567	0.146285903	0.883695694	0.98477672
ZDHHC20	1376.861431	-0.014755983	0.100915838	-0.146220682	0.883747179	0.98477672
AADACP1	16.14225473	-0.131165942	0.897367844	-0.146167419	0.883789225	0.98477672
TBC1D10B	1062.540475	0.016554193	0.113341012	0.14605651	0.883876778	0.98477672
LOC105371730	2.823021156	0.173271355	1.187289541	0.145938584	0.883969872	0.98477672
ZFAND6	899.6793446	-0.025321221	0.17354718	-0.145903963	0.883997203	0.98477672
MANBA	409.2235624	0.02531077	0.173493282	0.145889049	0.884008976	0.98477672
LINC01152	2.482477545	-0.19524902	1.338880957	-0.145830007	0.884055587	0.98477672
RALGAPA1P1	70.27288406	0.086383226	0.59252529	0.145788252	0.88408855	0.98477672
MRPS15	353.77158	-0.024031144	0.164970121	-0.145669677	0.88418216	0.984819432
GACAT2	10.4245128	-0.10434048	0.716964457	-0.145530896	0.884291724	0.984837846
LOC401021	116.5644686	0.047341314	0.32551212	0.145436408	0.884366321	0.984837846
THUMPD3	580.9828133	0.018787188	0.129239463	0.145367267	0.884420907	0.984837846
SNORA104	231.1420477	-0.023938654	0.164750092	-0.145302826	0.884471783	0.984837846
SLC7A6OS	102.3038287	0.029202889	0.200996561	0.145290493	0.884481521	0.984837846
PRKCQ	3.765155029	0.169124797	1.164951269	0.145177572	0.884570673	0.984837846
RPL37	1589.809323	-0.0200273	0.138012449	-0.145112269	0.884622231	0.984837846
SNORD116-21	143.2532798	0.038172464	0.26318602	0.14503986	0.884679401	0.984837846
GATA3	38.4839369	-0.052123428	0.359425494	-0.145018728	0.884696085	0.984837846
CSE1L	4550.264233	0.021287789	0.146927773	0.144886078	0.884800819	0.984876811
LINC02585	5.549669788	-0.133999677	0.92519236	-0.144834396	0.884841624	0.984876811
SUV39H1	267.9213425	-0.030061249	0.207944825	-0.144563584	0.885055451	0.985028482
DPY19L2	155.1294682	-0.062457299	0.432223298	-0.144502388	0.885103771	0.985028482
ZNF439	4.835511497	-0.160541991	1.111388003	-0.144451794	0.88514372	0.985028482
PIGS	1656.008747	0.019959164	0.138316661	0.144300502	0.885263182	0.985083214
GABPB1	457.8012531	-0.024951233	0.172972763	-0.144249492	0.885303461	0.985083214
ISCU	627.7976307	0.021667684	0.150300679	0.144162247	0.885372352	0.985098359
ZFP2	3.291274736	0.159450855	1.108433017	0.143852495	0.885616949	0.985308986
MOGAT1	2.469658219	0.205607961	1.430819635	0.143699426	0.885737824	0.985381947

FOXC2-AS1	1.948682847	-0.23322083	1.623832153	-0.143623729	0.885797602	0.985386932
C1orf189	2.699684982	-0.189345329	1.320897443	-0.143345973	0.88601695	0.985465586
N4BP2	616.5364637	-0.016356073	0.11413687	-0.143302276	0.886051459	0.985465586
ADAT3	68.25052877	-0.038543454	0.269014573	-0.143276453	0.886071851	0.985465586
SLC22A17	80.01405049	-0.052116957	0.363807805	-0.143254092	0.886089511	0.985465586
MED4	334.2111388	0.018605562	0.129975783	0.143146371	0.886174583	0.985498694
PRSS12	483.9879758	0.033144403	0.231894127	0.142929032	0.88634623	0.98562807
CDKN3	297.1797028	-0.028663244	0.200746706	-0.142783135	0.886461457	0.985687926
NEK2	500.3014652	-0.028492397	0.199637306	-0.142720807	0.886510684	0.985687926
LOC105375690	42.25939936	0.075757366	0.531526333	0.142527964	0.886662993	0.985795766
SLTM	552.8270977	-0.02479488	0.174057118	-0.142452551	0.886722556	0.985798486
LOC112543491	26.23744462	0.101223448	0.711141509	0.142339389	0.886811935	0.985798486
ABCC6P2	4.639303912	-0.124333112	0.873967469	-0.14226286	0.886872381	0.985798486
RASL10A	2.056382403	-0.175458791	1.233499606	-0.142244708	0.886886719	0.985798486
DZIP3	371.8578377	-0.022062309	0.155282477	-0.142078551	0.88701796	0.98588287
ZNF75D	211.5952628	0.021638311	0.152479605	0.141909545	0.887151455	0.985938487
ZNF609	1694.036893	-0.014883368	0.104904714	-0.141875109	0.887178655	0.985938487
FAM13A	293.5982016	-0.024989329	0.176304811	-0.141739345	0.887285896	0.985996175
IFT20	249.4443829	0.032796593	0.232063043	0.14132622	0.887612237	0.986297317
IAH1	505.2530775	-0.025634825	0.181501553	-0.141237498	0.887682324	0.986313693
COQ8B	285.3004882	0.023033761	0.163281642	0.141067674	0.887816481	0.986316894
TNNT3	2.593571402	-0.178566274	1.267215837	-0.140912281	0.88793924	0.986316894
LOC105369921	5.688016282	-0.115787747	0.822087184	-0.140846068	0.887991549	0.986316894
GPR160	74.99291237	0.034205977	0.242896978	0.140825043	0.888008159	0.986316894
EMSLR	33.01267503	0.062825581	0.446202698	0.14080054	0.888027516	0.986316894
AGRP	4.911557106	0.158609489	1.127155109	0.140716648	0.888093793	0.986316894
PLS3-AS1	16.91530549	0.070290139	0.499762877	0.140646979	0.888148833	0.986316894
ZNF616	306.3413083	0.02285621	0.162573304	0.140590181	0.888193706	0.986316894
BLOC1S6	2008.169538	0.017384925	0.123830132	0.140393332	0.888349226	0.986316894
ASRGL1	7.455658834	0.097825297	0.697437889	0.140263811	0.888451556	0.986316894
CPEB4	255.8022881	0.026968145	0.19254614	0.14006069	0.888612039	0.986316894
LOC100287837	3.231471937	0.162876963	1.163172602	0.140028197	0.888637712	0.986316894
VKORC1L1	963.5380297	0.015540116	0.111021679	0.139973711	0.888680762	0.986316894
NELFCD	1101.87256	0.021574648	0.154172721	0.139938165	0.888708847	0.986316894
SSPN	315.3373265	0.054994833	0.393373975	0.139802926	0.888815701	0.986316894
LIPG	1496.363829	-0.068724353	0.491613896	-0.139793348	0.888823269	0.986316894
ODF2	985.2888643	-0.016888806	0.120819553	-0.139785367	0.888829575	0.986316894
STMN3	5.472315789	-0.120256048	0.860864197	-0.139692239	0.888903158	0.986316894
C10orf55	16.76747168	-0.093477754	0.669399443	-0.139644206	0.888941111	0.986316894
ZCCHC9	175.9867803	-0.023947247	0.171493569	-0.139639331	0.888944964	0.986316894
ARCN1	2857.138301	0.024662155	0.176796677	0.139494451	0.889059441	0.986316894
TMX1	2280.160588	-0.018165848	0.13026911	-0.139448624	0.889095652	0.986316894
PDXP	444.1853342	-0.018603179	0.13344867	-0.139403253	0.889131502	0.986316894
ZNF706	1024.72824	0.021916136	0.157422278	0.139218768	0.889277279	0.986316894
VEZT	1402.633567	-0.020063015	0.144191333	-0.139141613	0.889338246	0.986316894
LINC-PINT	227.4274336	0.065399735	0.470413118	0.139026171	0.889429468	0.986316894
POTEF	8.451424214	0.145676295	1.048607334	0.138923589	0.88951053	0.986316894
LOC91450	9.901354662	0.099657857	0.717410221	0.13891335	0.889518621	0.986316894
KLF8	8.74481515	0.090643575	0.653645863	0.13867383	0.889707899	0.986316894
SYCP3	31.49542168	0.099861812	0.720214472	0.138655658	0.889722259	0.986316894
DAG1	703.6167784	0.02523288	0.181990458	0.138649468	0.889727151	0.986316894
SMUG1	268.6949583	-0.018847328	0.135999743	-0.138583558	0.889779237	0.986316894
RANBP10	380.6803668	-0.02100731	0.151642351	-0.138531943	0.889820026	0.986316894
MESD	1243.706678	-0.022518749	0.162576979	-0.138511303	0.889836337	0.986316894
SMNDC1	540.3503995	0.020740049	0.14977952	0.138470528	0.889868561	0.986316894
PTPRG-AS1	32.74921678	0.057865156	0.418192603	0.138369631	0.889948297	0.986316894
KANSL2	604.8008203	-0.030811285	0.222855242	-0.138256945	0.890037351	0.986316894
RTEL1	48.66855588	-0.055860714	0.40415343	-0.138216603	0.890069234	0.986316894
LOC91370	14.15350634	-0.088512389	0.640900278	-0.138106336	0.890156379	0.986316894
LINC01061	10.82548229	0.086937491	0.629588744	0.138086159	0.890172325	0.986316894
ERBB3	11.43499724	0.094699043	0.685842859	0.138076881	0.890179657	0.986316894
UBN1	1388.588676	0.020330872	0.14726166	0.138059506	0.890193389	0.986316894
KIAA0895L	326.2722095	0.03652102	0.26465109	0.137996864	0.890242896	0.986316894
ALAS1	357.8058315	0.019429709	0.140807692	0.137987553	0.890250255	0.986316894
CHD5	2.174393444	0.185170749	1.342320835	0.137948204	0.890281353	0.986316894
LSM11	708.9584885	0.019579575	0.141960541	0.137922657	0.890301544	0.986316894
SRI	578.6210615	0.019215776	0.139362934	0.137882974	0.890332907	0.986316894
MLF2	1142.327613	-0.01473356	0.106864408	-0.137871531	0.890341951	0.986316894
XXYLT1-AS1	4.435653988	0.209612755	1.52189698	0.137731238	0.89045283	0.986378407
ADNP2	1034.116226	-0.017889674	0.129985787	-0.137627926	0.890534484	0.98640754
FAM122C	79.17960518	0.037283405	0.271663504	0.137241124	0.890840206	0.98668209
MITD1	154.7490582	-0.024174684	0.176233436	-0.13717422	0.890893088	0.98668209
ANKRD6	53.94045281	0.040131203	0.292981126	0.136975386	0.891050251	0.986769385
WFDC21P	6.792991894	-0.109268538	0.798110015	-0.136909118	0.891102632	0.986769385
ARL2	365.7970938	-0.033338484	0.243654598	-0.136826819	0.891167684	0.986769385
MTRR	621.7909844	0.020965538	0.153263263	0.13679428	0.891193405	0.986769385
NOC4L	378.1740069	-0.022462257	0.164405199	-0.136627415	0.891325305	0.986849258
ALG2	505.1808899	0.015792922	0.11565178	0.136555807	0.89138191	0.986849258
NLK	279.2901796	0.022621272	0.165732291	0.136492848	0.891431678	0.986849258
CTDP1	442.3696723	0.018512601	0.135868984	0.136253326	0.89162102	0.986997551
TUBA3D	19.33580509	0.075514782	0.554862777	0.136096321	0.891745136	0.987030229
HDGFL3	807.6171893	-0.013827614	0.101616957	-0.136075851	0.891761318	0.987030229
EEA1	945.365639	-0.017107718	0.126069838	-0.135700322	0.892058193	0.987173959
KLHL35	11.08031431	0.079443511	0.586211452	0.135520231	0.89220057	0.987173959
SFT2D1	373.024836	0.022848567	0.168683947	0.135451936	0.892254564	0.987173959
NUP85	893.8432674	-0.019023508	0.140500648	-0.135398009	0.892297198	0.987173959
NMD3	1923.22141	-0.016487355	0.121788409	-0.135377042	0.892313775	0.987173959
TMEM230	1275.85988	0.017869651	0.132070271	0.135304116	0.892371431	0.987173959
WDR45B	1133.372364	-0.013600051	0.100520352	-0.135296495	0.892377456	0.987173959
RAB5B	1047.415599	-0.034496243	0.255332044	-0.135103463	0.892530072	0.987173959

GLS2	2.165305654	0.209037252	1.548368145	0.135004877	0.892608018	0.987173959
ERLEC1	1132.444675	0.023955081	0.177483431	0.134970803	0.892634959	0.987173959
HDX	103.673088	-0.030726439	0.22766532	-0.134963195	0.892640974	0.987173959
DCAF16	991.7513434	-0.018447391	0.136714333	-0.134933848	0.892664177	0.987173959
OPHN1	183.9074248	0.036216222	0.268535929	0.134865462	0.892718248	0.987173959
EML1	708.4203304	-0.025548631	0.18946529	-0.134845974	0.892733656	0.987173959
CKS1B	643.3492811	-0.016931979	0.125584549	-0.134825333	0.892749976	0.987173959
SNORA53	16.37118088	0.079077772	0.586829552	0.134754243	0.892806185	0.987173959
ZNF214	69.77318605	0.040101045	0.297682849	0.134710634	0.892840665	0.987173959
SPPL2B	826.2755726	0.018902738	0.140383861	0.134650363	0.892888321	0.987173959
LOC644135	10.90564266	-0.132126086	0.982302259	-0.134506548	0.893002034	0.987238429
SYT14	3.827060808	-0.150665272	1.121687884	-0.134320139	0.893149429	0.987277958
PCDHB17P	3.070563864	0.17987861	1.339620834	0.134275763	0.893184518	0.987277958
PON3	4.663372113	-0.124281555	0.926452777	-0.13414775	0.893285742	0.987277958
MTFP1	238.775378	0.027188716	0.20278757	0.134074863	0.893343377	0.987277958
TDRP	17.24431467	-0.083706545	0.624336529	-0.134072798	0.89334501	0.987277958
TTC39B	180.8443582	0.040859181	0.304875941	0.134019039	0.893387519	0.987277958
LOC101929767	31.23274829	-0.053865862	0.402071451	-0.13397087	0.893425609	0.987277958
LOC101927815	24.44067143	0.053947608	0.402913	0.133893936	0.893486445	0.987283962
HOXC9	55.89963436	-0.039990749	0.299036528	-0.133731985	0.893614512	0.987323363
TCEANC	40.7875653	-0.049510766	0.37028825	-0.133708715	0.893632913	0.987323363
STAT4	28.79808479	-0.067285472	0.503767243	-0.133564603	0.893746876	0.987335271
UFSP1	24.22950113	-0.058835763	0.440680268	-0.133511227	0.893789086	0.987335271
SOWAHC	257.1906791	0.020457052	0.153353276	0.133398206	0.893878465	0.987335271
MBOAT4	2.578425077	0.156336639	1.172488195	0.133337496	0.893926475	0.987335271
PRKG1-AS1	3.059250781	-0.148910557	1.117472837	-0.133256534	0.893990502	0.987335271
ABCG4	2.257875409	0.165148349	1.23988232	0.133196793	0.894037747	0.987335271
MAT1A	2.426712972	-0.162425584	1.219965115	-0.133139532	0.894083032	0.987335271
SLC44A5	11.69639589	-0.080154394	0.60228937	-0.133082863	0.894127848	0.987335271
JPX	94.50608724	0.040869974	0.307144015	0.133064531	0.894142346	0.987335271
ZNF737	17.16716198	0.075335807	0.568029929	0.132626475	0.894488794	0.98761201
CYP2D7	16.44716177	0.076384561	0.576019954	0.132607492	0.894503807	0.98761201
KAZALD1	330.1109765	0.037156087	0.280533015	0.132448179	0.894629809	0.987615746
TMED7	1627.651934	-0.018731556	0.14143894	-0.132435635	0.89463973	0.987615746
MAPRE1	2012.856935	-0.016612833	0.125613127	-0.132253957	0.894783425	0.987615746
NAALADL2	169.2203426	0.054612494	0.412999069	0.132233939	0.894799258	0.987615746
HHLA3	45.76372221	-0.065627195	0.496346577	-0.132220504	0.894809883	0.987615746
BCL2L11	337.943128	0.02657974	0.201083225	0.13218278	0.894839721	0.987615746
MEG9	65.89241551	0.075294694	0.570901388	0.131887391	0.895073362	0.987732888
BORCS8-MEF2B	4.522999763	-0.145640382	1.104414114	-0.13187117	0.895086192	0.987732888
UGP2	1497.234318	0.015408897	0.116990683	0.131710464	0.895213309	0.987732888
B4GALT6	618.607309	-0.022373771	0.169929466	-0.131665047	0.895249234	0.987732888
LCA5	121.5936471	0.027605659	0.20967162	0.131661398	0.89525212	0.987732888
RNF225	3.935449998	0.158081708	1.203375804	0.131365204	0.895486413	0.987732888
EPHB3	169.1902564	0.038752292	0.295068907	0.131333026	0.895511867	0.987732888
SNRNP48	580.5462002	-0.020881135	0.158994956	-0.131332056	0.895512634	0.987732888
ZCWPW2	11.81737267	0.079244162	0.603408149	0.131327629	0.895516136	0.987732888
OGDHL	281.5252213	0.021249702	0.16186223	0.131282645	0.89555172	0.987732888
PRKXP1	104.1529393	-0.053319302	0.406203019	-0.131262694	0.895567502	0.987732888
PHETA1	417.689638	0.023712742	0.180726747	0.131207708	0.895610999	0.987732888
SLC22A23	400.5345881	-0.023094559	0.176197499	-0.131072001	0.89571835	0.987790148
ZNF200	143.2801197	-0.025621656	0.19574432	-0.130893485	0.895859568	0.987834337
DUXAP9	357.4397486	-0.037875383	0.289387501	-0.130881199	0.895869288	0.987834337
PHF19	754.2016619	0.015178545	0.116043134	0.130800885	0.895932823	0.987843268
FAM71E1	4.968857119	0.126663817	0.968970668	0.130719971	0.895996834	0.987852724
LRP10	2356.177727	-0.0368428	0.282255217	-0.130530094	0.896147046	0.987947227
E2F6	432.3885398	0.027612365	0.211635287	0.130471463	0.89619343	0.987947227
LOC101929595	4.554351105	0.170893381	1.312717432	0.130182914	0.896421712	0.988137753
EGFL6	11.92296931	0.140909458	1.084070909	0.129981772	0.896580848	0.988181254
SDHAF1	181.9229575	0.024678283	0.189920659	0.129939962	0.896613927	0.988181254
DNAJA4	16.36490098	-0.063067118	0.485420086	-0.129922761	0.896627537	0.988181254
PIK3CD-AS2	8.383940988	-0.109367516	0.842804637	-0.129766154	0.896751442	0.988201492
IQCN	3.201813522	0.151366516	1.166517031	0.129759371	0.896756808	0.988201492
PKMYT1	364.8432926	-0.044143682	0.340813175	-0.129524576	0.89694258	0.98826775
FBXO42	400.6609969	0.01767624	0.13649703	0.129499078	0.896962754	0.98826775
FAM89B	133.4902741	0.025627867	0.198002254	0.129432199	0.897015671	0.98826775
LINC02453	4.501796262	-0.131752865	1.018446636	-0.129366489	0.897067663	0.98826775
RGPD1	4.199127841	-0.135395926	1.046984268	-0.129319924	0.897104507	0.98826775
WWC2-AS2	17.49759317	-0.076121955	0.589033708	-0.129231916	0.897174143	0.98826775
SH3GLB2	386.0998609	0.017454885	0.135108351	0.129191754	0.897205921	0.98826775
CAMK2B	2.932829212	-0.137064706	1.062815923	-0.12896373	0.897386348	0.98826775
ANKRD16	99.38074106	0.033982502	0.26353537	0.128948542	0.897398367	0.98826775
GTF3C2	1366.530344	0.017435659	0.135230702	0.128932699	0.897410903	0.98826775
IPO7	8931.588295	-0.021805054	0.169245176	-0.128837079	0.897486565	0.98826775
SH3BP2	937.2981244	0.022445143	0.174307632	0.128767412	0.897541692	0.98826775
ZNF433-AS1	35.2718272	0.048839252	0.379288764	0.128765355	0.89754332	0.98826775
GPS1	1048.655888	-0.01597413	0.124321279	-0.128490717	0.897760644	0.98826775
PRRX1	13932.50738	0.033968154	0.264458553	0.128444149	0.897797494	0.98826775
CEP70	132.4998515	-0.0281154	0.219001494	-0.128379948	0.897848299	0.98826775
TMX2-CTNND1	2.735599836	0.160513636	1.250771668	0.128331685	0.897886491	0.98826775
CDRT3	3.576217893	0.175185447	1.365345205	0.128308538	0.897904809	0.98826775
RILP	44.94774267	0.039788349	0.310129345	0.128295984	0.897914744	0.98826775
NDUFA6	172.2679813	0.02579436	0.201160298	0.12822789	0.89796863	0.98826775
LINC01771	2.908803364	-0.179877403	1.402973475	-0.12821155	0.89798156	0.98826775
GET4	356.9882904	0.017570117	0.137133143	0.128124512	0.898050438	0.988282517
LOC101929188	23.28866597	-0.053576182	0.418847394	-0.127913371	0.898217529	0.988296097
FAM222A-AS1	3.979090971	0.134431082	1.050967444	0.127911748	0.898218814	0.988296097
ZNF770	2989.324887	0.014882479	0.116392355	0.127864746	0.898256011	0.988296097
PKD1L1	5.869844211	0.106674408	0.835180044	0.127726241	0.898365623	0.988296097
CHN2-AS1	6.272156727	-0.128750629	1.008644645	-0.127647165	0.898428204	0.988296097

LAMC1-AS1	23.25022306	0.057684206	0.451944669	0.12763555	0.898437396	0.988296097
NCDN	908.186183	-0.019120762	0.149936768	-0.127525502	0.89852449	0.988296097
PRKCZ-AS1	4.408464725	0.122778238	0.963280684	0.127458424	0.898577578	0.988296097
SLITRK1	201.5553472	-0.028699486	0.225201438	-0.127439178	0.898592809	0.988296097
DNMT3B	97.99611499	0.031646032	0.248648364	0.127272231	0.898724938	0.988296097
EFL1P1	2.976173638	-0.16844812	1.324243448	-0.127203287	0.898779504	0.988296097
SNORD113-8	4.514177599	-0.137009967	1.077144988	-0.12719733	0.898784218	0.988296097
STK19	182.9411562	0.028452754	0.223709761	0.127186022	0.898793169	0.988296097
TAGLN3	2.719346205	0.172850672	1.359790999	0.127115617	0.898848891	0.988296097
STK25	1211.441993	-0.016040291	0.126369872	-0.126931293	0.898994778	0.988296097
NOSTRIN	22.2404661	0.093636369	0.737723794	0.126926053	0.898998926	0.988296097
SLC5A4-AS1	5.440301302	0.122835299	0.968170068	0.12687368	0.899040379	0.988296097
POT1-AS1	24.77009359	0.053422699	0.421156711	0.126847555	0.899061056	0.988296097
MIR222	15.20418382	0.083786365	0.661356812	0.126688594	0.899186873	0.988373432
LINC01560	36.29191007	0.069274425	0.547417282	0.126547749	0.899298355	0.988435002
POGK	1414.291356	-0.015957099	0.126291211	-0.126351616	0.8994536	0.988450255
ZNF34	135.0699815	0.026232557	0.207780829	0.126251092	0.89953317	0.988450255
LOC101927727	10.40797492	0.112693225	0.892907899	0.126209237	0.8995663	0.988450255
FMN2	442.1755653	-0.016507357	0.130844127	-0.126160473	0.899604899	0.988450255
NRN1L	2.860177891	0.167537812	1.329264874	0.126037944	0.899701889	0.988450255
MLYCD	94.85331826	0.027193943	0.215861088	0.125978904	0.899748624	0.988450255
HOXA13	189.3963389	0.025178628	0.200072516	0.12584751	0.899852633	0.988450255
PPP4R3C	11.13238694	0.075808936	0.602464661	0.12583134	0.899865433	0.988450255
LINC02126	5.064766837	-0.120385843	0.957996019	-0.125664241	0.899997709	0.988450255
PIK3CD	141.5881595	0.038731588	0.308449708	0.125568567	0.900073446	0.988450255
LRRC41	1139.094834	0.013584952	0.10830234	0.125435442	0.900178831	0.988450255
LOC101929415	11.53873219	0.12368146	0.986193657	0.125412954	0.900196633	0.988450255
NHSL2	31.1449928	0.065494329	0.522621634	0.125318824	0.90027115	0.988450255
WDR83	123.0322847	0.023000506	0.183686149	0.125216331	0.900352289	0.988450255
ATP6V1F	446.3016583	0.024826938	0.198425233	0.125119862	0.900428659	0.988450255
NES	152.5936252	0.052266948	0.417783606	0.12510531	0.900440179	0.988450255
TTC25	28.68669808	-0.047068176	0.376256481	-0.125095987	0.90044756	0.988450255
GPBP1L1	1395.746293	0.018824485	0.150507595	0.125073321	0.900465504	0.988450255
ZNF682	3.336341159	-0.132144174	1.05669084	-0.125054717	0.900480233	0.988450255
CD55	629.7716212	0.019399437	0.155180727	0.125011899	0.90051413	0.988450255
RPL23P8	3.604531017	0.148874239	1.190945211	0.125005112	0.900519503	0.988450255
NOXA1	11.9844517	0.112298787	0.898738135	0.124951621	0.900561851	0.988450255
NUDT12	231.0034182	-0.02466562	0.197716877	-0.124752221	0.900719714	0.988450255
CRB1	10.78954702	0.082411229	0.660686927	0.124735673	0.900732815	0.988450255
HSD17B4	1059.861139	-0.014695054	0.117862711	-0.124679412	0.900777357	0.988450255
VOPP1	1062.285998	0.015584362	0.125009854	0.124665068	0.900788714	0.988450255
RAD23A	604.8840257	0.014279365	0.114592554	0.124609889	0.900832399	0.988450255
MFF	524.2199624	0.013726771	0.110331129	0.124414303	0.900987249	0.988450255
CPEB2-DT	4.153385288	0.118856886	0.955473333	0.124395817	0.901001885	0.988450255
BEST1	36.61596594	0.049365516	0.396934134	0.124367023	0.901024683	0.988450255
DUS4L-BCAP29	28.90553163	-0.05386855	0.433172884	-0.124358085	0.901031759	0.988450255
RMDN2	58.546464	0.03689361	0.296892527	0.124265875	0.901104766	0.988469493
PDE6B	9.161714308	-0.08550571	0.690660379	-0.12380283	0.901471391	0.988810795
RBL2	2014.523787	0.020907998	0.170071891	0.122936236	0.902157591	0.989472929
FUNDC1	153.0211604	0.026504137	0.215655691	0.122900245	0.902186092	0.989472929
KLHDC2	709.2851044	-0.022565424	0.184199159	-0.122505575	0.902498631	0.989754791
BRIX1	472.9038436	-0.019822244	0.162189473	-0.122216588	0.902727489	0.989838351
MIR181B1	4.223583124	0.137160187	1.122548975	0.122186372	0.902751419	0.989838351
ZP3	30.4147841	0.088903822	0.727809236	0.122152644	0.90277813	0.989838351
DENND1A	561.0554672	0.014045213	0.115027948	0.122102616	0.90281775	0.989838351
KLHL5	639.3114543	0.022330197	0.182971864	0.1220417	0.902865993	0.989838351
ZNF511	97.13755339	-0.024948587	0.204515844	-0.121988529	0.902908103	0.989838351
RPL21P44	3.9301857	0.13047472	1.070463461	0.121886197	0.902989147	0.98986122
ODC1	1374.842221	-0.034892653	0.286423448	-0.121821914	0.903040059	0.98986122
ABCC6	42.70314736	0.040165042	0.330036955	0.121698621	0.903137706	0.989907363
KCTD20	2344.911593	-0.012300751	0.101177546	-0.121575897	0.903234904	0.989953009
LOC100507634	4.260939104	-0.115574754	0.952831925	-0.121296056	0.903456544	0.990094581
SCAP	1630.132847	0.012549889	0.103485032	0.121272507	0.903475196	0.990094581
CNTRL	492.9531671	-0.018807664	0.15537748	-0.12104498	0.903655408	0.990139376
MIR10396A	2407.022636	-0.060359158	0.499223037	-0.120906195	0.903765335	0.990139376
TRMT1	753.1982177	0.024262827	0.200860967	0.120794135	0.903854096	0.990139376
IL16	1006.605541	-0.077001977	0.637851573	-0.120720839	0.903912153	0.990139376
CNTF	4.041815261	0.171355213	1.419481761	0.120716742	0.903915398	0.990139376
MIR381	4.993386832	-0.132556317	1.098519552	-0.120668145	0.903953891	0.990139376
PAX6	5.474183333	0.120634957	0.999955855	0.120640282	0.903975961	0.990139376
SNORA98	9.921729866	-0.106050131	0.879087777	-0.120636566	0.903978905	0.990139376
EBLN2	51.3520416	0.043979355	0.364894322	0.120526279	0.904066264	0.990139376
NIN	1386.472658	0.01264837	0.104948818	0.12051941	0.904071705	0.990139376
GPSM1	1177.171337	-0.017773187	0.147560691	-0.120446625	0.904129359	0.990141665
LHPP	33.69700151	-0.055656091	0.462602018	-0.120310956	0.904236825	0.990198502
EPHB2	2495.230062	-0.023477403	0.195355996	-0.120177537	0.904342511	0.990253383
LAMC3	5.430574468	-0.112497033	0.937095438	-0.12004864	0.904444617	0.990304337
AIMP1	347.1193996	0.016020707	0.133873064	0.11967088	0.904743869	0.99042302
ZPLD1	10.61666025	0.083721425	0.699823135	0.119632262	0.904774462	0.99042302
SYTL3	18.71458641	0.055719145	0.46577794	0.119625986	0.904779434	0.99042302
CYP26C1	4.581052106	0.124724259	1.042740729	0.119611957	0.904790547	0.99042302
NCAPD2	4749.712552	0.018978252	0.158734189	0.11955995	0.904831747	0.99042302
KDM5C	3427.600481	0.011415614	0.095564542	0.119454494	0.904915291	0.99042302
ZNF829	108.4551465	-0.027128113	0.227164251	-0.119420696	0.904942065	0.99042302
DNAH6	96.43730462	-0.02717992	0.227751746	-0.119340116	0.905005903	0.990432057
LOC102724450	14.39236982	-0.063287817	0.53194237	-0.11897495	0.905295202	0.990654466
EPOP	597.4601775	-0.01729078	0.145717044	-0.118659968	0.905544754	0.990654466
CNNM2	194.3774738	0.023823156	0.200817595	0.118630819	0.905567848	0.990654466
ILK	132.1423925	-0.027993326	0.235973997	-0.118628858	0.905569401	0.990654466
VRK3	258.2227379	-0.016297275	0.137582335	-0.118454706	0.905707382	0.990654466

H3C12	3.49283264	0.147992873	1.250501687	0.1183468	0.905792877	0.990654466
DUSP8	3.340313443	-0.131400822	1.111698316	-0.118198273	0.905910558	0.990654466
BCR	59.06297468	-0.034697671	0.293884288	-0.118065756	0.906015556	0.990654466
LOC100506476	11.94188234	-0.094528323	0.80070851	-0.118055849	0.906023406	0.990654466
TMEM119	4968.707046	0.016006199	0.135672187	0.11797701	0.906085874	0.990654466
PPP1R16A	514.3298931	-0.027758495	0.235449555	-0.11789572	0.906150285	0.990654466
CADM1	3.824063292	0.173559469	1.472332605	0.117880613	0.906162255	0.990654466
MIR1469	5.338341089	0.110198016	0.936280569	0.117697643	0.906307234	0.990654466
TOR2A	159.2727323	0.024386749	0.2073977	0.117584471	0.90639691	0.990654466
DLL4	4.501705654	0.116947041	0.995246152	0.117505645	0.906459371	0.990654466
PIGX	466.4554796	-0.014636018	0.124662243	-0.117405377	0.906538823	0.990654466
CCDC113	34.39983022	-0.0462618	0.394075094	-0.117393363	0.906548343	0.990654466
PCDHB13	30.08023851	0.045733526	0.389704268	0.117354439	0.906579187	0.990654466
LOC650226	4.268684831	0.12951394	1.104185213	0.117293673	0.906627338	0.990654466
TNFAIP8L1	65.82794747	0.033989651	0.289801122	0.117286125	0.90663332	0.990654466
ARID5B	854.4891554	-0.013842591	0.118065566	-0.117244946	0.906665951	0.990654466
DUXAP8	281.5901167	0.028589389	0.243919068	0.117208502	0.90669483	0.990654466
SMAD2	2265.891596	-0.010530899	0.089929552	-0.117101653	0.906779499	0.990654466
PPM1A	1325.779378	0.013152601	0.112348104	0.117070079	0.90680452	0.990654466
RGS3	231.7099147	0.034426482	0.294226287	0.11700682	0.906854649	0.990654466
NSRP1	212.2567317	0.021200639	0.181213356	0.116992696	0.906865842	0.990654466
CCDC77	247.2353387	0.019899576	0.170335293	0.11682591	0.906998011	0.990654466
DUSP1	368.6470664	-0.046368168	0.396949629	-0.116811215	0.907009656	0.990654466
NNMT	408.5471611	0.046472666	0.39791198	0.116791321	0.907025422	0.990654466
ADAMTS15	24.3438147	-0.118604824	1.015696048	-0.116771966	0.90704076	0.990654466
DDX60L	185.6088519	-0.018024863	0.15437018	-0.116763893	0.907047157	0.990654466
PKN1	1150.156307	-0.02904956	0.248882877	-0.116719802	0.907082098	0.990654466
RADIL	74.61452519	-0.029314899	0.251296152	-0.116654788	0.907133619	0.990654466
ALG1L2	1.933848008	-0.15288501	1.31061868	-0.116651023	0.907136603	0.990654466
MORN1	124.2812242	-0.027579068	0.236475672	-0.116625392	0.907156915	0.990654466
MIR31HG	77.49601023	0.030911616	0.265307462	0.116512425	0.907246439	0.990654466
TNFRSF11B	6.735460861	-0.094613711	0.812296121	-0.116476872	0.907274615	0.990654466
SLC16A2	283.4265313	-0.018880289	0.162279273	-0.116344429	0.907379575	0.990654466
CCDC144CP	13.3474188	0.064587506	0.555480587	0.116273201	0.907436024	0.990654466
MRPS17	171.9619352	-0.021584306	0.185817734	-0.116158481	0.907526941	0.990654466
EPHB1	6.07782262	0.089535253	0.771067236	0.116118607	0.907558543	0.990654466
TOM1	212.4630458	-0.024187612	0.208372563	-0.116078682	0.907590184	0.990654466
LOC102723968	4.28336209	-0.110375478	0.95096508	-0.116066805	0.907599597	0.990654466
CALB1	3.752880688	0.141811251	1.224156885	0.115844017	0.907776165	0.99073276
ABHD13	252.3738654	-0.017027411	0.146995854	-0.115835998	0.90778252	0.99073276
WIPI1	296.900395	-0.046475924	0.401782217	-0.115674419	0.907910581	0.990736699
ATP5PF	199.3159699	-0.022113113	0.191231889	-0.115635069	0.907941769	0.990736699
CA5BP1-CA5B	28.77096956	0.040218633	0.348033507	0.115559657	0.908001538	0.990736699
KDM3A	1032.591373	-0.013520977	0.117017099	-0.115547017	0.908011556	0.990736699
TG	253.3608936	0.117628433	1.019028718	0.115431911	0.908102787	0.990736699
GLYATL2	6.325176707	-0.107189852	0.928769898	-0.115410558	0.908119711	0.990736699
ART5	2.153302775	0.148323068	1.289327096	0.115039131	0.908414106	0.990908829
CALHM2	55.91692831	-0.028571941	0.248510586	-0.114972732	0.908466736	0.990908829
PCDH9	612.4118001	0.027426763	0.238593247	0.114951968	0.908483194	0.990908829
FUT9	22.32895737	0.051858682	0.451578234	0.114838754	0.908572931	0.990908829
FAM13C	87.64156351	-0.02724067	0.237258892	-0.114814118	0.908592458	0.990908829
TSC22D1-AS1	90.00122805	0.028715026	0.250277136	0.114732918	0.908656821	0.990908829
GIMAP2	6.364650966	-0.091448793	0.797144898	-0.114720415	0.908666732	0.990908829
SORL1	2.31198266	-0.164012263	1.432367579	-0.114504311	0.908838029	0.99094715
ZFYVE19	378.8175085	-0.014706504	0.128440533	-0.114500492	0.908841056	0.99094715
TGOLN2	4339.783097	0.0145007	0.126889604	0.114278081	0.909017358	0.99094715
NDUFB7	274.9754605	-0.022241419	0.194807854	-0.114171059	0.909102193	0.99094715
MICB-DT	2.56362223	0.131798122	1.154472821	0.114163036	0.909108554	0.99094715
RBMX2	129.2190646	0.020958915	0.18379015	0.114037207	0.909208299	0.99094715
RFX5	1198.746347	-0.010553197	0.092628599	-0.113930222	0.909293108	0.99094715
LOC100289230	25.89549419	-0.064351832	0.5650613	-0.113884692	0.909329201	0.99094715
NEXN-AS1	15.93573399	-0.0568995	0.500012929	-0.113796057	0.909399464	0.99094715
PDCD6	631.7259343	0.015688664	0.137956715	0.113721643	0.909458455	0.99094715
MIATNB	7.845932835	-0.104535834	0.919929918	-0.113634563	0.909527487	0.99094715
C5orf63	139.1832987	-0.036312283	0.31958096	-0.113624676	0.909535326	0.99094715
MYO10	1409.491296	-0.020396742	0.179530068	-0.113611844	0.909545498	0.99094715
LCTL	29.97121563	0.05969955	0.525757712	0.113549547	0.909594884	0.99094715
RFFL	72.18150187	0.032654629	0.2877949	0.113464933	0.909661963	0.99094715
CALHM6	25.47828885	0.054168956	0.477604918	0.11341792	0.909699234	0.99094715
LSM6	86.41313156	0.035038468	0.309261877	0.113297081	0.909795031	0.99094715
COX7B	434.6244472	-0.019556977	0.172623656	-0.113292563	0.909798613	0.99094715
GSTCD	388.1366037	-0.015646063	0.138280361	-0.113147396	0.9099137	0.99094715
CD101	11.30560454	-0.074770222	0.661669075	-0.113002444	0.910028618	0.99094715
GPAA1	3517.453649	0.013213459	0.117216448	0.112727008	0.910246989	0.99094715
MSI2	1080.97177	-0.014577812	0.129426275	-0.112634102	0.910320648	0.99094715
IPO4	1971.496964	0.026719914	0.237481186	0.112513816	0.910416016	0.99094715
MAVS	2778.33789	0.021610355	0.192099211	0.112495801	0.910430299	0.99094715
MIR571	4.267016734	-0.144933187	1.289384698	-0.112404922	0.910502353	0.99094715
RPUSD1	271.9253394	-0.021460314	0.190928004	-0.112400033	0.910506229	0.99094715
BCAS4	38.81916515	-0.033609057	0.299105061	-0.112365391	0.910533695	0.99094715
PTGES2	836.9618403	0.022852773	0.203470542	0.112314896	0.910573731	0.99094715
LINC01535	13.94344439	-0.053785592	0.479170184	-0.112247367	0.910627273	0.99094715
DCAF10	919.3458969	0.018475778	0.164646815	0.112214609	0.910653246	0.99094715
MIR620	4.265747555	0.156185089	1.39214486	0.112190257	0.910672554	0.99094715
IFIH1	27.54448165	-0.046485883	0.41434894	-0.112190181	0.910672614	0.99094715
PLK1	1098.401993	0.015683771	0.139807991	0.112180789	0.910680061	0.99094715
CA8	7.055670223	0.098842866	0.88118292	0.112170656	0.910688095	0.99094715
CMAHP	10.23469423	-0.086968907	0.775821098	-0.112099178	0.910744769	0.99094715
LHFPL4	7.380656308	0.084486961	0.753956391	0.112058153	0.910777297	0.99094715
LOC729603	53.67602883	0.061362175	0.547647308	0.112046885	0.910786231	0.99094715

CCAR2	2036.320232	-0.017348313	0.154880997	-0.112010597	0.910815004	0.99094715
PLK5	3.97282153	0.125109749	1.117821028	0.11192288	0.910884555	0.990962318
TRIM41	614.2399446	-0.01885833	0.168639885	-0.111826036	0.910961343	0.990971745
LDB2	172.0725069	-0.022582671	0.202042864	-0.111771683	0.911004441	0.990971745
RAET1E-AS1	2.273026977	-0.1347733	1.208648381	-0.111507451	0.911213958	0.991050977
EVC2	30.46978093	-0.050183775	0.450833314	-0.11131337	0.911367854	0.991050977
CEP126	142.9066881	-0.020641727	0.185498627	-0.11127698	0.911396709	0.991050977
F11R	102.1571816	0.035843402	0.322248187	0.11122918	0.911434613	0.991050977
SPAG17	4.248277947	0.150738464	1.355437597	0.111210184	0.911449676	0.991050977
TEDC2	223.3244314	0.038700761	0.348144919	0.11116279	0.911487258	0.991050977
TMX2	872.9798469	-0.015386585	0.138464277	-0.111123135	0.911518703	0.991050977
C1QTNF1	5.754633555	-0.115822864	1.042385041	-0.111113322	0.911526485	0.991050977
PXMP4	144.6820663	-0.030518668	0.274822634	-0.111048598	0.911577809	0.991050977
TRMT10A	59.37282989	-0.029831104	0.269001016	-0.110895879	0.911698914	0.991112832
PTCH1	450.096133	0.020793932	0.18760894	0.110836576	0.911745941	0.991112832
PCP2	9.107555829	-0.07111646	0.643847931	-0.110455368	0.912048244	0.991202154
RBM18	563.3710226	-0.016918933	0.153189025	-0.110444809	0.912056618	0.991202154
RBM43	103.8404845	-0.035804322	0.324208886	-0.110435966	0.912063631	0.991202154
RNVU1-6	10.51346269	0.091722077	0.830936899	0.110383926	0.9121049	0.991202154
PDLIM2	319.6874143	-0.017915994	0.162308644	-0.110382257	0.912106224	0.991202154
LIMK2	195.6035969	-0.018031991	0.163746357	-0.110121479	0.912313034	0.991366441
TEFM	151.9137651	-0.024293527	0.220819464	-0.110015333	0.912397214	0.991385902
MIR4256	3.106994996	0.160366309	1.458424462	0.109958598	0.91244221	0.991385902
PRDX6	2240.555631	-0.014872865	0.135538725	-0.109731483	0.912622331	0.991438831
ZBTB24	448.6994831	0.018435855	0.168027689	0.109719147	0.912632115	0.991438831
LOC101927377	38.78001519	0.073419759	0.66935869	0.10968672	0.912657833	0.991438831
ATP5MG	128.8179335	0.021068997	0.192502213	0.109448075	0.912847104	0.991565733
PCDHGA4	31.70541318	0.042080817	0.384654102	0.109399112	0.912885938	0.991565733
RAD23B	3481.223158	-0.01295185	0.118592049	-0.109213481	0.913033168	0.991607959
APOLD1	136.3165986	0.046304538	0.424217235	0.109152892	0.913081224	0.991607959
WDR11	1198.94192	0.009359716	0.085759109	0.109139619	0.913091751	0.991607959
SNORA22B	9.093506508	0.072889995	0.669257737	0.108911696	0.91327253	0.991706785
ZNF763	2.381042372	-0.131770663	1.210186861	-0.108884559	0.913294054	0.991706785
PLA2G4C	2.375383739	0.149138659	1.37089144	0.108789548	0.913369414	0.991728185
A4GALT	73.30087446	0.059878501	0.550862721	0.108699498	0.91344084	0.99174531
FXYD7	4.165629151	-0.109643881	1.009603704	-0.10860091	0.913519039	0.991769787
DISC1	14.24769987	-0.07339707	0.676547715	-0.108487647	0.913608879	0.991802729
PTN	822.2095314	-0.013637883	0.125784828	-0.108422321	0.913660696	0.991802729
NDUFB8	351.4964395	-0.017127122	0.158320637	-0.108179974	0.91385293	0.9918931
SMIM10L2B	19.91488634	0.056078287	0.518393692	0.108177024	0.91385527	0.9918931
ORC3	637.455403	-0.015930669	0.147650306	-0.107894586	0.914079312	0.991995313
ZNF354C	413.8887331	0.014487551	0.134284613	0.107886904	0.914085406	0.991995313
ARHGAP25	4.698192516	0.111316086	1.033072147	0.10775248	0.914192039	0.991995313
DAGLA	53.19351787	-0.03694191	0.34309465	-0.107672649	0.914255366	0.991995313
SNORD113-6	5.083193568	-0.165507988	1.537238708	-0.107665769	0.914260824	0.991995313
STK32C	65.56270929	0.03389297	0.314881419	0.107637251	0.914283447	0.991995313
SDCBP2	11.28937407	0.066853934	0.622544562	0.107388191	0.914481022	0.992108913
TP53TG1	22.40813114	-0.048339389	0.450234549	-0.107364905	0.914499495	0.992108913
ZNF792	30.41632136	0.041110155	0.383744078	0.107129092	0.914686568	0.992155397
C3orf67-AS1	2.999251536	0.153504301	1.435482289	0.106935698	0.914839992	0.992155397
SREBF2	5862.095784	0.022214142	0.207801413	0.106900825	0.914867659	0.992155397
ANKRD24	9.411175983	0.08148917	0.762779262	0.106831916	0.914922327	0.992155397
CBSL	60.93911859	0.055231179	0.517020332	0.106825933	0.914927074	0.992155397
LRWD1	221.8585848	-0.019072051	0.178637661	-0.106763887	0.914976298	0.992155397
LINC02510	35.06167157	0.133992976	1.25504757	0.106763265	0.914976791	0.992155397
WDR66	23.3419572	-0.052330053	0.490670489	-0.106650093	0.915066577	0.992155397
PNLDC1	2.689897658	-0.118234975	1.1097576	-0.106541262	0.915152919	0.992155397
YTHDF3-AS1	16.91917495	-0.053430166	0.501498341	-0.106541063	0.915153077	0.992155397
NDE1	348.1058166	0.0233922	0.219564849	0.106538911	0.915154785	0.992155397
WDR1	2548.750093	0.01604506	0.15077708	0.106415778	0.915252475	0.992184682
CPOX	542.3509966	-0.017850555	0.167908427	-0.106311249	0.915335407	0.992184682
ANKS6	994.7574213	-0.012452756	0.117196454	-0.106255399	0.915379717	0.992184682
TNFRSF1B	9.608293917	-0.075259619	0.708498196	-0.106224151	0.91540451	0.992184682
BICD2	1447.800282	0.010854662	0.102322925	0.106082404	0.915516972	0.992197929
RFX3-AS1	4.822336751	-0.09152386	0.863755685	-0.105960356	0.915613807	0.992197929
UGT3A2	96.97279429	-0.026937433	0.254406166	-0.10588357	0.91567473	0.992197929
CHMP5	387.5585855	-0.027613504	0.260830976	-0.105867425	0.91568754	0.992197929
ARAP2	40.17649195	0.034288925	0.324080196	0.105803827	0.915738	0.992197929
NBPF9	107.2075443	0.026402448	0.249587193	0.105784465	0.915753363	0.992197929
TMED2-DT	61.88601114	0.050203046	0.475465106	0.105587235	0.915909853	0.992197929
ABHD12B	47.03542355	-0.031850701	0.302226796	-0.105386754	0.916068927	0.992197929
CACTIN	409.186182	0.019271265	0.182935151	0.105344788	0.916102225	0.992197929
ABHD14A	91.65627807	-0.023597491	0.224133665	-0.105283119	0.916151158	0.992197929
AGL	1100.039036	-0.012587057	0.119598527	-0.10524425	0.916181999	0.992197929
ADAMTS9-AS1	3.003424138	-0.144914357	1.37704205	-0.105235971	0.916188568	0.992197929
RBM15B	1543.379006	-0.016230313	0.154346969	-0.105154723	0.916253037	0.992197929
GAS8-AS1	14.53832176	0.072056291	0.68541783	0.10512754	0.916274606	0.992197929
FBXW11	1198.40519	0.01255092	0.119428352	0.105091626	0.916303104	0.992197929
LINC01515	54.47284194	-0.033351025	0.317632649	-0.104998731	0.916376816	0.992197929
MICALCL	10.98426344	0.060492607	0.576397928	0.104949383	0.916415973	0.992197929
UCP3	5.958524088	-0.087547768	0.834220232	-0.10494563	0.916418951	0.992197929
SMG9	418.2958965	-0.014958224	0.142834282	-0.104724329	0.916594557	0.992298844
PRDX4	1647.896905	0.014398157	0.13756723	0.104662692	0.916643466	0.992298844
TSSC2	3.086805656	0.123194477	1.177568828	0.104617645	0.916679213	0.992298844
APOL3	2.045596043	-0.14376677	1.375180059	-0.104543961	0.916737684	0.99230186
TMEM199	278.1495526	-0.020043047	0.19192225	-0.10443316	0.916825608	0.992336755
PMS2P7	5.313994697	0.106323941	1.019572059	0.10428291	0.91694484	0.99240553
PCAT6	7.738682235	0.08905609	0.855432047	0.104106563	0.917084783	0.992475759
PBDC1	188.6376286	0.0255423	0.245687254	0.103962657	0.917198983	0.992475759
PPA2	608.5308878	-0.014422196	0.13873467	-0.103955243	0.917204867	0.992475759

RASSF4	24.28643136	0.049022089	0.471727228	0.103920414	0.917232507	0.992475759
TNFRSF6B	11.84392823	-0.069206129	0.666788516	-0.103790223	0.917335825	0.992511248
LIX1L	1271.689243	0.024084564	0.232369684	0.103647619	0.917448997	0.992511248
MIR4322	5.333308984	-0.099259622	0.957940562	-0.103617725	0.917472721	0.992511248
BUD13	187.8027429	0.019580672	0.189114721	0.103538594	0.917535521	0.992511248
RNF215	266.4428029	0.019256632	0.186065394	0.103493892	0.917570997	0.992511248
ATP5MGL	7.7542028	-0.078106653	0.755672305	-0.103360482	0.917676875	0.992511248
HMGN3	326.749363	0.025202228	0.243939679	0.103313359	0.917714273	0.992511248
ZNF37A	604.5384822	0.017154528	0.166086216	0.103286888	0.917735282	0.992511248
DSCR9	6.508665152	-0.079425364	0.769271851	-0.10324746	0.917766574	0.992511248
ARL4C	654.1684606	-0.078290076	0.759343265	-0.103102351	0.917881739	0.992575556
HAUS8	90.36408547	0.024984013	0.242909109	0.102853338	0.918079372	0.992695457
SFMBT2	59.65765756	0.032861635	0.31961042	0.102817783	0.918107592	0.992695457
SPINT1	70.55054862	-0.042864621	0.417165477	-0.102752081	0.918159737	0.992695457
ETV6	319.6183068	-0.030884752	0.301053978	-0.102588752	0.91828937	0.99271978
NUP133	1824.611911	0.010769914	0.105197648	0.102377901	0.918456724	0.99271978
MTCL1	589.6540828	0.020004166	0.195472522	0.102337484	0.918488804	0.99271978
APOE	32.9742625	0.047190022	0.461311984	0.102295245	0.91852233	0.99271978
SLC46A3	28.31312464	-0.039916585	0.390890928	-0.102116938	0.918663857	0.99271978
C1GALT1C1	183.1680663	-0.01750325	0.171405588	-0.102115985	0.918664613	0.99271978
LINC01521	161.5842476	0.029195927	0.286087723	0.102052359	0.918715115	0.99271978
FAM118A	194.2193944	-0.015598942	0.152897163	-0.102022445	0.91873886	0.99271978
GLMN	313.7943929	-0.020190639	0.197964361	-0.101991283	0.918763594	0.99271978
CD160	6.811287746	-0.074302017	0.728578726	-0.101982139	0.918770852	0.99271978
MYBBP1A	3107.056971	0.017845065	0.175034526	0.101951686	0.918795024	0.99271978
ABHD12	677.9657824	0.014370491	0.141068955	0.101868558	0.918861007	0.992730881
C17orf49	47.32528824	0.030416044	0.298996933	0.101726943	0.918973416	0.992744147
ZNF717	302.1077739	0.013164136	0.12948391	0.101666195	0.919021636	0.992744147
LOC642361	40.01427976	0.032597129	0.321069781	0.101526617	0.91913243	0.992744147
HUS1B	4.486069957	-0.106011617	1.044363443	-0.101508357	0.919146924	0.992744147
YEATS2	3886.481189	0.01211166	0.119324143	0.101502171	0.919151834	0.992744147
RAD17	438.228235	-0.015171128	0.149958892	-0.101168577	0.919416641	0.992951761
LINC00337	2.949593867	0.12196614	1.206156967	0.101119625	0.9194555	0.992951761
GDNF-AS1	3.152020046	0.113894232	1.127337591	0.101029392	0.919527128	0.992968938
ZNF195	233.4404326	0.035621747	0.353129218	0.10087454	0.919650054	0.993041506
MRPL12	755.6603643	-0.016291519	0.161733913	-0.100730381	0.919764494	0.9931049
LINC01608	27.98244252	0.038548562	0.383300844	0.10056999	0.91989182	0.993176814
FBXO27	14.02793891	0.071619741	0.713006445	0.100447537	0.919989032	0.993176814
ABHD6	68.51492401	-0.029734184	0.296051458	-0.100435865	0.919998298	0.993176814
LOC728989	3.723854082	-0.09741318	0.972144587	-0.100204415	0.920182041	0.993297376
HCG14	2.53621206	0.130805254	1.306569092	0.100113537	0.920254188	0.993297376
LOC104968399	11.97112885	-0.056739985	0.567333142	-0.100011759	0.92033499	0.993297376
SMIM10	29.22690213	0.042366757	0.423865006	0.09995342	0.920381305	0.993297376
PKN3	973.9275092	0.015885329	0.159086251	0.09985356	0.920460585	0.993297376
ICE1	1821.738914	0.010856676	0.108837624	0.099751133	0.920541905	0.993297376
PLG	4.479275875	-0.126549518	1.26962371	-0.099674823	0.920602489	0.993297376
BBIP1	63.28612356	0.033969409	0.341000186	0.099616982	0.920648411	0.993297376
TUBG2	138.3875468	0.031759342	0.318859894	0.099602812	0.920659661	0.993297376
LOC107984035	13.53245101	0.064566628	0.649208994	0.099454303	0.920777569	0.993297376
MIR4479	3.356847074	-0.121799135	1.224714071	-0.099451078	0.920780129	0.993297376
GNG4	18.5595622	-0.052868898	0.532303526	-0.099320962	0.920883436	0.993297376
IBA57	243.902899	-0.020588914	0.20731216	-0.099313586	0.920889292	0.993297376
CST7	4.691056075	-0.093364026	0.940105748	-0.099312259	0.920890345	0.993297376
PABPN1	672.7328721	0.027732472	0.279926465	0.09907056	0.921082247	0.99332359
DNPEP	574.143506	0.012639044	0.127730384	0.098950958	0.921177208	0.99332359
PARP16	226.0505627	-0.027729853	0.280333359	-0.098917422	0.921203836	0.99332359
PIP5K1P1	6.242789252	-0.112606796	1.138855218	-0.098877183	0.921235785	0.99332359
ZNF726	170.0950796	0.026419013	0.267192407	0.098876363	0.921236437	0.99332359
IL4I1	3.976219903	0.122611181	1.240245478	0.098860413	0.9212491	0.99332359
MIR1193	6.096809	0.123666037	1.252283592	0.098752421	0.921334846	0.993338749
PXN	997.8172597	0.010833964	0.109764051	0.098702296	0.921374646	0.993338749
LGMN	1558.973153	-0.014322823	0.145290042	-0.098580899	0.921471037	0.993382569
SEMA6B	7.097125891	-0.081777606	0.832501908	-0.098231133	0.921748761	0.993557956
HOXA9	3.319011994	0.114488161	1.165824797	0.098203574	0.921770645	0.993557956
GTDC1	137.5960276	0.022538311	0.229595371	0.098165355	0.921800993	0.993557956
CDKL4	4.212919451	-0.089964434	0.918087143	-0.09799117	0.921939305	0.993624231
GCC2-AS1	6.422624175	-0.073654926	0.752719945	-0.0978517	0.922050054	0.993624231
NODAL	7.97255685	0.071224729	0.728154152	0.097815453	0.922078836	0.993624231
RECQL4	1369.200884	0.032402515	0.331455369	0.097758304	0.922124218	0.993624231
CCDC92	179.9253744	-0.030331834	0.310479174	-0.097693617	0.922175584	0.993624231
SENP3	204.4006925	-0.018913032	0.193991731	-0.097494009	0.922334092	0.993624231
GDPD3	21.71846414	0.058725704	0.60295185	0.097397005	0.922411123	0.993624231
SMIM14	356.8136874	-0.058815981	0.604277826	-0.097332682	0.922462203	0.993624231
GALNS	288.0555944	-0.019689277	0.202390641	-0.097283534	0.922501232	0.993624231
LINC01270	5.58011441	0.08792616	0.904206447	0.097241245	0.922534815	0.993624231
TSEN54	338.382965	0.018052893	0.185666657	0.097232822	0.922541503	0.993624231
WDR53	206.2519318	-0.015233565	0.156862869	-0.097113903	0.92263594	0.993624231
ATP6V0C	416.2372358	0.016088195	0.166083434	0.096868147	0.922831105	0.993624231
NGF	2.268195739	0.12199699	1.260306285	0.096799478	0.922885639	0.993624231
PDS5A	2616.675577	-0.011878025	0.123013161	-0.096558976	0.923076637	0.993624231
SAFB	555.9580546	0.017393823	0.18023324	0.096507296	0.92311768	0.993624231
KEL	2.5605761	-0.142034526	1.47198947	-0.096491537	0.923130195	0.993624231
SENP5	2445.733187	-0.009446979	0.098052176	-0.096346449	0.923245422	0.993624231
CDC42SE1	1215.847005	0.011659944	0.121075849	0.096302804	0.923280084	0.993624231
EFHC2	87.04367529	-0.027464201	0.285269279	-0.096274655	0.92330244	0.993624231
RPS24	1293.956466	-0.013363357	0.139132872	-0.096047449	0.923482888	0.993624231
MRPS21	410.7753751	-0.011863389	0.123586932	-0.095992261	0.923526719	0.993624231
DYM	1402.231742	0.021071666	0.219547537	0.095977694	0.923538288	0.993624231
TLCD2	210.9841364	0.021491585	0.223965932	0.095959172	0.923552999	0.993624231
RGP1	918.3887064	-0.015464	0.161224619	-0.095915874	0.923587387	0.993624231

KIAA1324	2.703624777	-0.120651967	1.258469477	-0.095871985	0.923622244	0.993624231
CPSF2	3305.022385	0.009209804	0.096072341	0.095863222	0.923629205	0.993624231
HRAS	202.6567734	-0.025002091	0.260946928	-0.095812933	0.923669146	0.993624231
PTK7	6059.345302	0.019869049	0.207550567	0.095731122	0.923734122	0.993624231
KXD1	470.6953197	-0.018992708	0.198465914	-0.095697581	0.923760762	0.993624231
IPO8	978.5996392	-0.014927781	0.156007475	-0.09568632	0.923769706	0.993624231
KIF5C	4.61488117	0.090102513	0.94255869	0.09559353	0.923843404	0.993624231
NCOA1	379.5239946	-0.012868953	0.134824599	-0.095449594	0.923957725	0.993624231
RPL23AP32	10.3971155	-0.109133221	1.14356965	-0.095432072	0.923971643	0.993624231
LINC01977	3.328887816	-0.122516103	1.284336896	-0.095392497	0.924003076	0.993624231
RBFADN	5.83281587	-0.086335037	0.906024967	-0.095289909	0.924084557	0.993624231
TBCD	1710.420045	-0.013123701	0.137773287	-0.095255774	0.92411167	0.993624231
TAF9B	316.9529765	-0.019399285	0.20396508	-0.095110813	0.92422681	0.993624231
TGFB2	699.1561313	0.014685596	0.154457841	0.095078343	0.9242526	0.993624231
DOCK9	476.8993659	0.011185696	0.117685233	0.095047574	0.92427704	0.993624231
TIMM17B	203.5356074	-0.016214851	0.170616987	-0.095036556	0.924285791	0.993624231
THEMIS2	65.04639829	-0.045591187	0.479962192	-0.094989121	0.924323468	0.993624231
LIG4	191.0895429	0.019740262	0.20825432	0.094789206	0.924482261	0.993624231
MIR659	2.627739175	0.13219938	1.394759905	0.094782894	0.924487275	0.993624231
ST3GAL3	294.7945447	-0.018136394	0.191606094	-0.094654577	0.924589198	0.993624231
C11orf94	3.298394577	-0.135409686	1.431144509	-0.094616361	0.924619554	0.993624231
LOC102724378	9.827784065	0.079073395	0.8366105	0.09451638	0.924698972	0.993624231
SNORD11B	4.411014235	0.086139159	0.911471049	0.094505645	0.924707499	0.993624231
PTPRVP	10.62026554	0.073443666	0.778048856	0.094394671	0.924795649	0.993624231
LINC00237	2.711276984	-0.129026859	1.369468165	-0.094216764	0.924936968	0.993624231
CIDECP1	20.41275988	-0.040073745	0.426652722	-0.09392591	0.925168011	0.993624231
ATG101	231.1587137	-0.014020014	0.149348264	-0.093874633	0.925208745	0.993624231
DCUN1D3	339.8239632	-0.012839229	0.136971032	-0.093736821	0.92531822	0.993624231
KIZ	235.6733381	0.017561413	0.187402094	0.093709799	0.925339686	0.993624231
SH3BGRL3	259.9006707	-0.024117571	0.257380745	-0.093703865	0.9253444	0.993624231
RAB5A	676.6629209	-0.019958318	0.213435864	-0.093509673	0.925498665	0.993624231
MMP17	550.5418257	0.014965917	0.160133084	0.093459241	0.925538729	0.993624231
GMDS-DT	25.37675098	-0.03827916	0.40979425	-0.093410682	0.925577304	0.993624231
TMEM243	236.6719293	0.015950115	0.170846787	0.093359176	0.925618222	0.993624231
TMOD4	7.560637075	-0.067606982	0.72416626	-0.09335837	0.925618862	0.993624231
FHL2	818.4780493	0.023992924	0.257102094	0.093320609	0.92564886	0.993624231
SOX15	17.39692476	0.045150702	0.483881071	0.093309502	0.925657684	0.993624231
PLCE1-AS2	6.940578027	-0.095817224	1.0273073	-0.093270265	0.925688854	0.993624231
LOC105371566	7.652518836	0.077768009	0.833801604	0.093269201	0.9256897	0.993624231
AFF4	2269.132032	-0.008723682	0.093551444	-0.093250108	0.925704868	0.993624231
HOXB3	131.2126417	-0.020117245	0.215929014	-0.09316601	0.925771677	0.993624231
LINC00968	2.707386135	-0.130274911	1.399140225	-0.093110689	0.925815626	0.993624231
MYO1F	6.28930384	0.069896355	0.751149871	0.093052475	0.925861873	0.993624231
ACVR1	376.2962473	-0.015860294	0.170492026	-0.093026605	0.925882425	0.993624231
USP4	617.1790699	0.01113616	0.119773814	0.092976579	0.925922167	0.993624231
SNORD95	44.5825352	0.031117829	0.334826296	0.092937233	0.925953426	0.993624231
AFG1L	134.5122012	-0.021239914	0.228549945	-0.092933358	0.925956504	0.993624231
TAOK3	412.6705691	0.012162928	0.131058976	0.092805001	0.926058478	0.993624231
FOXL2	425.8766272	0.010614432	0.114379246	0.092800332	0.926062187	0.993624231
ARSG	78.56702624	-0.028630479	0.308532398	-0.092795696	0.926065871	0.993624231
EYS	32.34542304	0.031515902	0.339824249	0.092741769	0.926108713	0.993624231
ABCC4	1060.825574	0.01146827	0.123825235	0.092616582	0.92620817	0.993624231
TESMIN	9.530259382	0.057679209	0.622803802	0.092612166	0.926211678	0.993624231
LOC100506207	15.46441285	-0.054480783	0.588942793	-0.09250607	0.926295969	0.993642741
LY96	4.28371925	-0.088833445	0.960925846	-0.092445682	0.926343946	0.993642741
ERVMER34-1	307.4282609	0.014855269	0.160908793	0.092321053	0.926442962	0.993642741
FLRT2	1347.399487	0.009596709	0.103962076	0.092309712	0.926451972	0.993642741
MSR1	4.642416943	0.090426988	0.980428028	0.092232153	0.926513592	0.993649026
ERLIN1	920.6820213	0.015083989	0.163788454	0.092094336	0.926623089	0.993706652
PLIN4	40.49731033	-0.0398613	0.433261078	-0.092002956	0.926695691	0.993714925
LOC102723604	2.835559501	0.125141051	1.367268394	0.091526325	0.92707439	0.993714925
POLDIP3	1015.976973	0.012763431	0.139514545	0.09148459	0.927107551	0.993714925
DNAH1	122.9061768	0.033460048	0.366322629	0.091340379	0.927222135	0.993714925
ALG3	1221.553557	0.01271779	0.139399359	0.091232774	0.927307633	0.993714925
ERG28	389.6094952	0.020538904	0.225202843	0.091201797	0.927332247	0.993714925
ZNF227	249.9335513	-0.014852486	0.162879039	-0.091187215	0.927343834	0.993714925
DUSP5P1	65.97112658	0.038708034	0.424540381	0.091176331	0.927352482	0.993714925
PROSER3	390.2814537	-0.012637009	0.138606331	-0.091171948	0.927355964	0.993714925
ANKRD7	30.8637782	0.035411021	0.388511489	0.091145363	0.927377088	0.993714925
KCTD2	696.7367483	0.015261743	0.167461242	0.091135974	0.927384548	0.993714925
EIF4E	1335.835986	0.013068982	0.143581117	0.091021591	0.927475435	0.993714925
ID2-AS1	20.39788631	-0.048393114	0.531677343	-0.091019703	0.927476936	0.993714925
DDX59-AS1	2.400554559	-0.127529685	1.401162117	-0.09101708	0.927479019	0.993714925
ATP2A3	97.25521287	0.024722266	0.272258054	0.090804536	0.927647906	0.993714925
KIAA2013	1072.408068	0.012063655	0.133090131	0.090642746	0.927776465	0.993714925
SNORD114-21	48.60677229	-0.033084406	0.365188217	-0.090595492	0.927814014	0.993714925
FAM185A	84.68113268	0.033204941	0.36664611	0.090564006	0.927839033	0.993714925
ZNF728	4.95804654	-0.102903204	1.138523962	-0.090382994	0.92798287	0.993714925
SNORD91B	9.40415962	0.091697826	1.014726665	0.090367021	0.927995563	0.993714925
RORA	335.3787636	0.02512059	0.278194723	0.090298586	0.928049944	0.993714925
ZNF710-AS1	16.40489153	-0.045849254	0.508385935	-0.090185922	0.928139472	0.993714925
FAM162A	103.2516098	0.021453797	0.238460507	0.089967927	0.928312702	0.993714925
GALNT16	24.89845884	0.036584983	0.406742012	0.089946407	0.928329803	0.993714925
INHBA-AS1	12.77365044	0.050146108	0.557633915	0.089926576	0.928345562	0.993714925
LOC101409256	3.505701257	0.114688806	1.275668935	0.089904836	0.928362838	0.993714925
QPRT	81.88671824	0.041740039	0.465340069	0.089697926	0.928527264	0.993714925
XPA	178.3640463	-0.022813305	0.254382718	-0.089681033	0.928540689	0.993714925
L3MBTL4	36.50808422	0.035548163	0.396549797	0.08964363	0.928570412	0.993714925
SP110	223.6447022	0.014939732	0.166669377	0.089636935	0.928575732	0.993714925
GFOD2	269.1235805	0.017118175	0.191032945	0.089608495	0.928598334	0.993714925

MAPK1IP1L	2453.501499	0.007536211	0.084131521	0.08957654	0.928623728	0.993714925
LOC100507437	48.44192877	-0.036890617	0.41215852	-0.089505895	0.928679869	0.993714925
EXOSC3	182.8743193	0.018367001	0.205351039	0.089441966	0.928730673	0.993714925
GMFB	2212.556566	0.014244731	0.159342538	0.089396916	0.928766475	0.993714925
FAM171A1	705.5037004	-0.010723264	0.119957033	-0.089392541	0.928769951	0.993714925
ZNF14	76.87949361	-0.023135215	0.258861412	-0.089372978	0.928785498	0.993714925
ADD2	23.08161075	0.090608239	1.014880287	0.089279731	0.928859602	0.993714925
EXOC3L1	10.05522688	-0.055520445	0.621931144	-0.089271048	0.928866502	0.993714925
HTD2	5.953697407	0.077981901	0.874506927	0.089172422	0.928944882	0.993714925
RALA	1222.250878	-0.014514265	0.162774518	-0.089167918	0.928948462	0.993714925
DHRS12	20.97873786	0.041020626	0.460196597	0.089137177	0.928972892	0.993714925
CSNK2B	680.2068024	-0.010980574	0.123473385	-0.088930696	0.929136988	0.993820858
ADPRS	330.1301654	-0.013898804	0.156607754	-0.088749145	0.929281275	0.993820858
LOC101928909	9.199724153	0.075805459	0.854306445	0.088733334	0.92929384	0.993820858
MBNL3	350.4767974	0.012814878	0.144447734	0.088716366	0.929307326	0.993820858
USP46-AS1	24.13740945	-0.042818278	0.483768755	-0.088509804	0.929471492	0.993820858
SMPD4	899.4015756	0.012189886	0.137825126	0.088444586	0.929523325	0.993820858
SFN	3.954203917	0.098402514	1.112692299	0.088436412	0.929529822	0.993820858
PARGP1-AGAP4	8.297654546	-0.064452422	0.729362923	-0.088368108	0.929584108	0.993820858
PTGES3	7189.340183	0.010954982	0.124196924	0.088206546	0.929712514	0.993820858
APOBEC3F	17.28566445	-0.058299366	0.662537928	-0.087994006	0.92988144	0.993820858
TDO2	31.68278709	-0.033940853	0.385719345	-0.087993649	0.929881724	0.993820858
CD58	266.3221426	0.014702722	0.167143733	0.087964542	0.929904858	0.993820858
MRPL58	216.6180618	-0.015225273	0.173178327	-0.087916735	0.929942856	0.993820858
CXCL3	2.545774161	0.132969065	1.514137564	0.087818351	0.930021052	0.993820858
PROZ	5.125702379	-0.094408554	1.075866754	-0.087751158	0.930074458	0.993820858
SGMS2	268.7917095	0.012502687	0.142507582	0.087733487	0.930088503	0.993820858
LINC02298	7.42837859	0.0695499	0.793077645	0.087696205	0.930118136	0.993820858
CDC42BPB	3085.181707	0.009275449	0.10577654	0.087689092	0.930123789	0.993820858
ABCC9	41.77040085	0.032270185	0.368097367	0.087667524	0.930140932	0.993820858
ZNF780A	580.621617	0.020067745	0.229059896	0.087609159	0.930187322	0.993820858
SLC35B4	1221.590649	-0.01139401	0.130324551	-0.087427962	0.930331344	0.993901151
L3MBTL2	549.7894612	-0.018706144	0.214712791	-0.087121701	0.930574776	0.993901151
SP9	12.06832021	-0.066802333	0.766943153	-0.087102065	0.930590384	0.993901151
MAPK6-DT	2.583181566	0.115891386	1.332511313	0.086972159	0.930693642	0.993901151
ZNF710	181.0066077	-0.016484143	0.189585572	-0.0869483	0.930712607	0.993901151
UFL1	1058.893593	0.011530875	0.132904887	0.086760357	0.930861999	0.993901151
HAND2	116.1943663	-0.017999381	0.20751412	-0.086738103	0.930879689	0.993901151
SLC25A34	3.093847617	-0.093003791	1.072329563	-0.086730604	0.93088565	0.993901151
CLEC11A	5.709297023	-0.073535121	0.848152326	-0.086700371	0.930909681	0.993901151
METTL23	349.9446723	0.014103316	0.162729109	0.086667446	0.930935853	0.993901151
CMTM6	955.587541	0.012511602	0.144386812	0.086653357	0.930947052	0.993901151
SNORD3K	7.793889361	0.068089517	0.786473124	0.086575771	0.931008725	0.993901151
LINC01372	10.98316822	-0.051023636	0.589448399	-0.086561666	0.931019937	0.993901151
SNORD99	14.22154097	-0.053969419	0.624292961	-0.086448867	0.931109602	0.993901151
ZFP62	474.198572	-0.010942946	0.126679063	-0.086383228	0.931161779	0.993901151
RCN2	1678.845006	0.009146793	0.105902832	0.086369669	0.931172557	0.993901151
PPIG	558.2154855	-0.009804831	0.113607474	-0.086304456	0.931224396	0.993901151
RIOX1	417.5923207	-0.014997674	0.17413436	-0.086127021	0.931365444	0.993901151
LZTR1	1368.484633	0.008764283	0.101762438	0.086124929	0.931367107	0.993901151
TBRG1	281.1542169	0.013388077	0.1555122	0.086090204	0.93139471	0.993901151
PRR14	200.0932244	0.016268747	0.189275326	0.085952812	0.931503929	0.993901151
LIN37	95.55289453	-0.02173301	0.253068656	-0.085877923	0.931563462	0.993901151
NPEPL1	33.95920651	-0.032653867	0.380388743	-0.085843409	0.931590898	0.993901151
LDHD	31.28561984	-0.031620303	0.368511876	-0.085805384	0.931621127	0.993901151
FILIP1L	91.00480307	0.087222533	1.018942446	0.08560104	0.931783572	0.993901151
ZNF806	2.101527649	-0.11372871	1.329947192	-0.085513704	0.931853002	0.993901151
PCYT2	886.7180094	-0.010446892	0.122226411	-0.085471642	0.931886439	0.993901151
RBM47	8.839388163	0.058396771	0.684105319	0.085362253	0.931973401	0.993901151
RAD54L	511.2188692	-0.016297431	0.191120202	-0.085273198	0.932044199	0.993901151
TNFRSF1A	1548.786763	0.020463445	0.239980273	0.085271363	0.932045657	0.993901151
PLTP	304.9406461	0.012214758	0.143436039	0.085158221	0.932135605	0.993901151
CPNE5	6.519074176	-0.078432788	0.921181718	-0.085143665	0.932147176	0.993901151
CIPC	466.4867996	-0.016023984	0.188221574	-0.08513362	0.932155162	0.993901151
COX6C	1722.551481	-0.011390096	0.133883333	-0.085074787	0.932201935	0.993901151
ZNF865	377.5659548	0.014578373	0.171480192	0.085014907	0.93224954	0.993901151
TAPBP	1194.486005	-0.018182241	0.213971229	-0.084975169	0.932281131	0.993901151
PRKAR2A-AS1	15.64274021	-0.050492224	0.59530865	-0.084816883	0.932406971	0.993901151
SUMO3	973.5391354	0.013104646	0.154718562	0.084699895	0.932499979	0.993901151
SLAIN1	4.17766127	-0.093539173	1.10632236	-0.084549654	0.932619426	0.993901151
CACNA1G-AS1	11.37789544	0.061256927	0.725075409	0.084483525	0.932672001	0.993901151
AFTPH	444.9805207	-0.011573037	0.137067921	-0.084432865	0.932712278	0.993901151
DACT2	22.40660512	0.055484723	0.657552411	0.084380685	0.932753763	0.993901151
EXOC3	574.4164934	-0.011297519	0.133910109	-0.08436644	0.932765089	0.993901151
SLC16A8	10.71358174	-0.050524377	0.598969081	-0.084352229	0.932776387	0.993901151
LRRC61	3.573251865	0.126330154	1.498066303	0.084328813	0.932795004	0.993901151
ARHGAP4	40.12111976	-0.031083194	0.368793732	-0.084283411	0.932831102	0.993901151
PALMD	3.98443119	0.081172511	0.963849081	0.084217034	0.932883875	0.993901151
ZNF180	221.5038214	-0.017800171	0.211848535	-0.084023101	0.933038065	0.994005997
PINK1	231.3767105	-0.032374518	0.386025183	-0.083866336	0.933162705	0.994023234
AVPR1A	1.867508812	0.134856004	1.608062105	0.083862435	0.933165807	0.994023234
SCN8A	9.915709432	-0.050608578	0.604371957	-0.083737469	0.933265166	0.994040395
BCL2L13	1027.856879	-0.01579494	0.188704782	-0.083701855	0.933293482	0.994040395
SELENOS	431.5788436	0.011300643	0.135561134	0.083361967	0.933563729	0.994128359
SDK2	3.320883665	0.091459742	1.098122656	0.083287364	0.933623047	0.994128359
DCAF13	2892.245453	-0.008486708	0.101968523	-0.083228706	0.933669688	0.994128359
MIR138-1	3.07472369	-0.121039977	1.45484397	-0.08319791	0.933694175	0.994128359
ZNF365	76.12668407	0.022380348	0.269043685	0.083184811	0.93370459	0.994128359
RTL10	558.7039502	0.011483592	0.138139775	0.083130234	0.933747986	0.994128359
DHX58	9.02867647	-0.085954715	1.035620904	-0.082998242	0.933852937	0.994128359

SHLD1	21.91570125	-0.038076255	0.458854884	-0.082981038	0.933866617	0.994128359
MAP7D1	2049.064053	0.014336524	0.172872889	0.082931016	0.933906391	0.994128359
LINC01341	9.351474409	0.066925145	0.807648665	0.082864181	0.933959536	0.994128359
SOS2	679.718391	-0.011517553	0.139056869	-0.082826208	0.93398973	0.994128359
LINC00205	762.7757815	0.010453809	0.126487294	0.08264711	0.934132141	0.994220555
CSNK1G1	1073.798466	-0.009806649	0.118836593	-0.082522131	0.93423152	0.994266943
SENP1	494.6363239	-0.011745811	0.142927448	-0.082180231	0.934503394	0.994327728
IRAK1	3215.386259	-0.009785555	0.119317802	-0.082012535	0.934636746	0.994327728
LMLN2	2.627695123	0.130565816	1.593578751	0.081932453	0.934700428	0.994327728
STAT2	978.2519206	0.017579297	0.214567068	0.081929149	0.934703055	0.994327728
DNAJB1	961.1690833	-0.013729965	0.167666333	-0.081888624	0.93473528	0.994327728
VCP	5378.646578	-0.009833482	0.120226708	-0.08179116	0.934812786	0.994327728
LMNTD1	6.641410304	-0.091214764	1.116646058	-0.081686371	0.934896117	0.994327728
DDN-AS1	94.7691854	-0.017278193	0.211539396	-0.081678369	0.93490248	0.994327728
SNORD164	3.603403739	0.086319311	1.059485873	0.081472829	0.935065932	0.994327728
TNRC18P1	4.623841213	-0.105323632	1.292796801	-0.081469595	0.935068504	0.994327728
AIFM3	17.70246737	0.040421933	0.49616776	0.081468278	0.935069551	0.994327728
ZSCAN29	899.9966601	-0.009360469	0.114957208	-0.081425685	0.935103423	0.994327728
SH3PXD2A	2952.472707	0.016868304	0.207696207	0.081216236	0.935269987	0.994327728
CRAT	410.838167	0.026620359	0.327848179	0.081197213	0.935285116	0.994327728
COL1A1	178898.2251	-0.00944635	0.116400962	-0.081153536	0.935319851	0.994327728
GPX3	70.63867179	0.021530704	0.265342124	0.08114318	0.935328086	0.994327728
STX19	8.262976657	-0.07305418	0.901175872	-0.081065397	0.935389944	0.994327728
FGF18	5.121134998	0.092694949	1.14370998	0.0810476	0.935404098	0.994327728
DIS3	1527.780113	0.007958764	0.098386716	0.080892668	0.935527311	0.994327728
GALC	891.5279996	0.013582167	0.16794311	0.080873619	0.93554246	0.994327728
OTUD1	64.27196394	0.040273509	0.498017893	0.080867595	0.935547251	0.994327728
CHMP4C	9.370192308	0.061061868	0.755152015	0.080860366	0.935553	0.994327728
TNIP2	331.0375313	-0.013520195	0.167280971	-0.080823267	0.935582504	0.994327728
HLA-J	39.19575661	0.031138356	0.385551779	0.080763097	0.935630356	0.994327728
TASP1	131.3464412	-0.022049007	0.274080895	-0.080447077	0.935881686	0.994327728
DHX57	792.5753429	-0.008373925	0.10419906	-0.080364691	0.935947208	0.994327728
MCL1	3012.808545	-0.011604933	0.144458478	-0.080334037	0.935971588	0.994327728
PRDM5	342.1061742	0.011660469	0.145236758	0.080285936	0.936009844	0.994327728
EMID1	35.43560402	-0.030579237	0.381239034	-0.080210142	0.936070124	0.994327728
MAOA	227.1197598	-0.026233622	0.327441872	-0.080116883	0.936144295	0.994327728
RBM15-AS1	5.198610286	-0.078513707	0.981682352	-0.079978729	0.936254173	0.994327728
GNG10	320.0567037	0.015337014	0.19181047	0.079959212	0.936269696	0.994327728
RBM22	581.4732407	0.010998133	0.137689237	0.079876493	0.936335486	0.994327728
POLR2C	856.5306949	0.010744062	0.134529996	0.079863692	0.936345667	0.994327728
TOE1	161.3445146	0.021334917	0.267390434	0.079789379	0.936404772	0.994327728
MFSD6	91.78975898	-0.016756086	0.210353976	-0.079656617	0.936510364	0.994327728
RADX	291.7462424	-0.019393068	0.243510805	-0.079639457	0.936524013	0.994327728
DFFA	2169.360309	-0.015941564	0.200223378	-0.079618894	0.936540368	0.994327728
OXLD1	272.9824433	0.011290118	0.141974669	0.07952206	0.936617386	0.994327728
MIR99AHG	262.5585406	0.049544774	0.623065624	0.079517746	0.936620817	0.994327728
CLSPN	915.7806584	-0.019020445	0.239251507	-0.079499793	0.936635097	0.994327728
TRDN	2.671199593	-0.114990308	1.448713026	-0.079374111	0.93673506	0.994327728
PATJ	185.3439376	-0.013507444	0.170390817	-0.079273309	0.936815236	0.994327728
SPART-AS1	3.421278373	-0.098066678	1.237316285	-0.079257567	0.936827757	0.994327728
LKAAEAR1	5.660665124	-0.075156653	0.949317646	-0.079169131	0.936898098	0.994327728
SNORD114-16	4.973862081	0.079877697	1.009310574	0.079140851	0.936920591	0.994327728
TMEM45A	485.9596866	-0.012751956	0.161257049	-0.07907844	0.936970232	0.994327728
CLCN3	1178.282343	0.012113507	0.153359123	0.078987846	0.937042291	0.994327728
NFKBIZ	249.0533861	-0.026522127	0.335822827	-0.078976545	0.937051279	0.994327728
AMPD3	10.29139333	-0.06120041	0.77845337	-0.078617952	0.937336508	0.994327728
IQUB	10.67038379	0.058714964	0.746887272	0.078612886	0.937340538	0.994327728
C16orf95	31.73465183	0.028743292	0.365718681	0.078593994	0.937355565	0.994327728
ENPP4	1145.710765	0.018438123	0.234667053	0.078571419	0.937373522	0.994327728
RNF24	409.0912871	-0.020038983	0.255074625	-0.078561256	0.937381606	0.994327728
SBNO1-AS1	2.998660545	-0.08970153	1.144275512	-0.078391549	0.937516596	0.994327728
PSMD9	366.3191064	-0.011607363	0.148135049	-0.078356626	0.937544375	0.994327728
WNT2	4.71889866	-0.074304887	0.949848676	-0.078228132	0.937646585	0.994327728
PYCR3	982.4634886	0.016466354	0.210572981	0.078197848	0.937670674	0.994327728
CDK2AP1	1061.671223	-0.010202008	0.13053941	-0.078152706	0.937706582	0.994327728
MIR6845	6.451462955	0.06958354	0.890664605	0.078125413	0.937728292	0.994327728
ATP1A3	12.29740605	0.046510533	0.595747447	0.07807089	0.937771663	0.994327728
RMND5A	2661.527752	0.00960913	0.123167767	0.078016595	0.937814852	0.994327728
ARHGEF18	792.2872257	-0.008170165	0.104762745	-0.077987308	0.937838149	0.994327728
GORAB	514.6590925	0.012544476	0.160933853	0.077948024	0.937869398	0.994327728
IP6K1	707.4248618	0.009298604	0.119487725	0.077820583	0.937970773	0.994327728
MYCBPAP	13.45991016	0.049481766	0.636790172	0.077704978	0.938062734	0.994327728
P4HTM	175.4534514	0.014878483	0.191520082	0.07768628	0.938077608	0.994327728
SIRT5	315.9588478	0.012533836	0.161353011	0.077679594	0.938082927	0.994327728
HIPK3	1625.094566	-0.014632937	0.188943003	-0.077446301	0.938268509	0.994369052
MIR626	2.446096517	0.125999469	1.629386749	0.077329381	0.938361518	0.994369052
PHF10	399.1761819	-0.009537319	0.123399674	-0.077288039	0.938394406	0.994369052
EPOR	21.74386729	0.034550824	0.447182429	0.077263375	0.938414026	0.994369052
NDRG3	474.1129827	0.012713276	0.164654145	0.077212	0.938454895	0.994369052
PDE2A	8.787913591	-0.064514314	0.835572555	-0.077209709	0.938456718	0.994369052
PJA2	1781.762663	0.013328644	0.172884494	0.077095656	0.938547449	0.994406061
PATL1	675.2786196	0.008999449	0.11697399	0.076935472	0.938674879	0.994481947
VAC14	580.2573152	-0.008965214	0.116829593	-0.076737525	0.938832352	0.994510198
LPGAT1	1677.087362	0.007796221	0.101656575	0.076691751	0.938868767	0.994510198
IDH1	887.0635574	0.01120521	0.146107591	0.076691495	0.938868971	0.994510198
LOC105377102	3.31057766	-0.084163352	1.099500382	-0.076546906	0.938983998	0.994516275
EEF1AKMT2	275.2892198	-0.011973415	0.156722396	-0.076398875	0.939101764	0.994516275
CNMD	7.190085689	0.054190764	0.709580298	0.076370165	0.939124605	0.994516275
GATC	1303.355872	0.008523258	0.111741963	0.076276253	0.939199318	0.994516275
PCMTD1	681.7810501	0.014251706	0.186887781	0.076258094	0.939213765	0.994516275

PPP5D1	4.246183903	-0.07142725	0.937676602	-0.076174717	0.939280097	0.994516275
GAREM1	286.3653329	0.01205257	0.158268895	0.07615249	0.939297781	0.994516275
ERGIC1	2507.113288	0.010872362	0.142933077	0.076066106	0.939366506	0.994516275
ISG15	170.6385515	-0.014392983	0.189557838	-0.075929244	0.93947539	0.994516275
TSPAN31	397.821251	-0.00916363	0.120804179	-0.075855243	0.939534265	0.994516275
RMND5B	244.7432901	-0.018824266	0.249243318	-0.075525658	0.939796484	0.994516275
FRG1BP	159.5997254	0.022628933	0.299705474	0.075503903	0.939813792	0.994516275
C21orf91	323.8304795	-0.011441636	0.15156027	-0.075492317	0.93982301	0.994516275
ISY1-RAB43	6.996582318	-0.051049408	0.676570656	-0.075453181	0.939854148	0.994516275
POU5F1	39.29344619	-0.023890716	0.316634007	-0.075452147	0.93985497	0.994516275
SFXN5	243.7818131	0.023938611	0.317821477	0.075320937	0.939959364	0.994516275
LINC01597	72.81877748	-0.021063504	0.279802569	-0.07527988	0.939992029	0.994516275
TOGARAM1	652.2399143	0.010937765	0.145323035	0.075265184	0.940003722	0.994516275
DCAF5	890.5497254	-0.009494902	0.126243289	-0.075211139	0.940046722	0.994516275
MPHOSPH8	486.006831	-0.009958098	0.132401978	-0.075211095	0.940046757	0.994516275
CGRRF1	133.6292427	0.015339306	0.204084648	0.075161488	0.940086226	0.994516275
PACS1	558.0773672	-0.015294913	0.203648427	-0.075104501	0.940131567	0.994516275
PPM1N	10.71182871	0.061462302	0.818723575	0.075070883	0.940158315	0.994516275
TMEM79	75.66773505	0.019874922	0.265084822	0.074975706	0.940234041	0.994533145
SNORA48	23.87944386	0.036794327	0.49117684	0.07491055	0.940285883	0.994533145
GAL	24.64221954	0.036950766	0.495676608	0.074546117	0.940575847	0.994535967
VANGL1	727.4518065	0.009210369	0.123557785	0.074543008	0.940578321	0.994535967
ATP2B1-AS1	35.17722197	-0.031932823	0.428974774	-0.074439862	0.940660392	0.994535967
CRYZL2P-SEC16B	9.265344255	-0.045395627	0.610054192	-0.07441245	0.940682203	0.994535967
USP15	1365.710106	0.007268716	0.097695717	0.07440158	0.940690852	0.994535967
BRSK2	6.076421098	0.06976271	0.937748722	0.074393821	0.940697025	0.994535967
RBM34	241.710854	-0.010805389	0.145348822	-0.074341082	0.940738989	0.994535967
HELQ	185.6310547	0.014741813	0.198448923	0.074285173	0.940783475	0.994535967
ZNF688	46.20549171	-0.023198267	0.312325569	-0.074275914	0.940790842	0.994535967
COX6B2	15.452023	-0.056129243	0.757298941	-0.074117683	0.940916745	0.99461006
CLIP4	429.114634	-0.010566041	0.142785993	-0.073999141	0.941011069	0.994625855
WDFY1	1273.364945	-0.01119551	0.151516386	-0.073889763	0.941098102	0.994625855
TENT2	515.8179539	-0.008697863	0.11771611	-0.073888467	0.941099133	0.994625855
HYOU1	3292.052023	0.007829679	0.106077087	0.073811218	0.9411606	0.994631829
DAPK1	46.00103522	0.025200471	0.342604183	0.073555642	0.941363968	0.99468597
DPM1	234.0384897	-0.01172219	0.159387169	-0.073545384	0.941372131	0.99468597
EEF1AKNMT	767.1277213	0.012930375	0.175836411	0.073536391	0.941379286	0.99468597
C1D	238.490379	-0.010524248	0.143643215	-0.073266586	0.941593981	0.994703355
KPNA3	1763.132547	-0.009952208	0.136113205	-0.07311714	0.941712902	0.994703355
TMEM170B	101.4454117	-0.025132889	0.343866965	-0.073088989	0.941735303	0.994703355
ANKAR	87.93378964	0.020466496	0.280035972	0.073085238	0.941738289	0.994703355
MBOAT1	450.2783413	0.010159168	0.139010643	0.073081944	0.94174091	0.994703355
ENOX2	162.932346	-0.016044358	0.219850121	-0.072978618	0.941823133	0.994703355
CMC1	44.59177385	0.030206639	0.414224152	0.072923414	0.941867062	0.994703355
SYT11	353.1932472	0.024494336	0.336101256	0.072877847	0.941903323	0.994703355
MIR4284	3.745660958	0.094247952	1.29370109	0.072851413	0.941924358	0.994703355
LOC100506100	25.20032504	-0.030838274	0.424548998	-0.072637726	0.942094405	0.994703355
MTHFS	35.97396159	-0.029879068	0.411428627	-0.072622726	0.942106342	0.994703355
SHC3	4.01139517	0.077909915	1.073507846	0.072575077	0.94214426	0.994703355
RELA	394.778706	-0.010385962	0.143178995	-0.072538307	0.942173521	0.994703355
FBLN7	83.31222516	0.035806245	0.494077321	0.072470934	0.942227136	0.994703355
RBMS2	773.0377433	0.016977394	0.234775737	0.07231324	0.942352628	0.994703355
ACAP2	3161.570216	0.01113495	0.154193765	0.07221401	0.942431596	0.994703355
SLC13A5	35.54371123	0.029011329	0.404361674	0.071745992	0.942804055	0.994703355
RAB8A	464.0134696	-0.008242826	0.115013586	-0.071668282	0.942865898	0.994703355
CYB561A3	342.4328397	0.010458018	0.14598806	0.071636122	0.942891493	0.994703355
RCN3	2650.434791	0.015146185	0.211890883	0.071481059	0.943014899	0.994703355
STRN3	1128.252466	-0.011580497	0.162093054	-0.071443511	0.943044781	0.994703355
MANBAL	341.6311794	0.011656762	0.163240604	0.071408474	0.943072665	0.994703355
PBLD	36.04463848	0.030584082	0.428402552	0.07139099	0.943086581	0.994703355
TAB1	661.6634914	-0.009885289	0.138870294	-0.071183613	0.943251623	0.994703355
CITED4	5.806609913	0.068554793	0.963903533	0.071122048	0.943300621	0.994703355
SEMA6A-AS1	7.353549127	0.080374935	1.130123657	0.071120478	0.94330187	0.994703355
LINC02067	21.77828357	-0.037848203	0.532245033	-0.071110485	0.943309823	0.994703355
DIP2A-IT1	12.08157447	0.051832203	0.729147074	0.071086075	0.943329251	0.994703355
C5orf51	798.3740925	0.012702724	0.178725763	0.071073827	0.943338998	0.994703355
ACKR3	29.28573239	0.052560555	0.739913383	0.071036092	0.943369031	0.994703355
NRAS	2614.121235	0.008572273	0.120679633	0.071033306	0.943371248	0.994703355
NSL1	508.2929083	-0.012613839	0.17786619	-0.070917575	0.943463356	0.994703355
FEZ2	538.8881191	0.008144546	0.114906605	0.0708797	0.9434935	0.994703355
DHRS4	128.9550166	-0.017114008	0.24148609	-0.07086954	0.943501586	0.994703355
SNORD13F	3.896462525	0.077638978	1.095656404	0.070860699	0.943508623	0.994703355
MEG3	2379.558139	-0.022759074	0.321616013	-0.07076474	0.943584995	0.994703355
SALL2	88.13547048	-0.018554902	0.262342762	-0.070727708	0.943614468	0.994703355
TYW3	565.0118147	0.011690713	0.165300993	0.070723789	0.943617588	0.994703355
USE1	101.6424999	0.019357737	0.274001624	0.070648256	0.943677703	0.994703355
REXO4	574.6561054	0.009780311	0.138487994	0.070622084	0.943698534	0.994703355
LOC105370943	8.643412885	-0.048607439	0.689020743	-0.070545682	0.943759342	0.994703355
PGAP3	383.9704777	-0.016083239	0.228425736	-0.070409049	0.943868089	0.994703355
IDH2-DT	2.754067048	0.102304894	1.453170023	0.070401187	0.943874347	0.994703355
RYR2	8.666796754	-0.054028337	0.767735668	-0.070373618	0.943896289	0.994703355
SPATA7	118.5065479	0.020760515	0.29509722	0.070351441	0.94391394	0.994703355
KRT81	6.218440198	0.100700272	1.432655337	0.070289252	0.943963437	0.994703355
KLHDC8B	79.20901717	0.018220133	0.260036431	0.070067618	0.94413984	0.994752905
HOXB13	22.68694247	0.033198993	0.47424239	0.070004272	0.94419026	0.994752905
ZNF385C	15.65215218	0.050180557	0.717257298	0.069961724	0.944224125	0.994752905
ZNF799	50.52736479	0.021398262	0.306018697	0.069924689	0.944253602	0.994752905
BNIP1	167.4492587	-0.015704879	0.225821219	-0.069545629	0.944555314	0.994752905
PPP2R5E	1472.509602	0.008243506	0.11862589	0.06949163	0.944598295	0.994752905
ZNF641	147.7600846	-0.011845955	0.170720285	-0.069388095	0.944680705	0.994752905

HMGCLL1	12.95913533	0.057566091	0.829753598	0.069377332	0.944689272	0.994752905
ZNF534	2.088021794	0.098705109	1.423221763	0.069353288	0.94470841	0.994752905
YIPF5	943.2805728	-0.012782432	0.184697646	-0.069207338	0.944824582	0.994752905
DRP2	142.2302359	0.012704575	0.183794357	0.069123858	0.944891031	0.994752905
SCAND2P	250.80691	0.017210539	0.249066993	0.069100041	0.944909989	0.994752905
ARHGEF25	364.4311166	-0.011464674	0.166038599	-0.069048244	0.944951218	0.994752905
IRF2BP2	4140.159418	0.021348903	0.309624467	0.068950957	0.945028657	0.994752905
NPHP1	48.98766409	-0.024091163	0.349669097	-0.068897032	0.945071581	0.994752905
MRPS34	563.9648728	0.008538089	0.124004078	0.068853293	0.945106397	0.994752905
ZNF280D	548.3540023	0.008677518	0.126044102	0.068845093	0.945112924	0.994752905
GIGYF1	1185.436249	-0.017398533	0.252721749	-0.068844622	0.945113299	0.994752905
MTMR1	552.5550611	-0.007377383	0.107471052	-0.068645304	0.945271956	0.994752905
PALD1	297.1553733	0.011702181	0.170513933	0.068628887	0.945285024	0.994752905
FANCI	2510.473685	-0.009726251	0.142017182	-0.068486441	0.945398413	0.994752905
VSIG8	10.07710417	0.051285797	0.749731828	0.068405522	0.945462826	0.994752905
ZNF668	193.6300406	-0.011707434	0.171406287	-0.068302244	0.945545038	0.994752905
FBXO34	805.5130503	0.008899163	0.130310381	0.068292048	0.945553154	0.994752905
TDRD7	57.81705465	-0.023434384	0.343430095	-0.068236255	0.945597567	0.994752905
NOXRED1	11.66905689	0.040296471	0.590763117	0.068210878	0.945617767	0.994752905
FAM200B	127.184315	0.014800538	0.217347919	0.068096067	0.945709162	0.994752905
NDFIP2	521.9659791	0.010431859	0.1533081	0.06804506	0.945749765	0.994752905
GRIN2D	156.9279401	-0.023294158	0.342353717	-0.068041201	0.945752837	0.994752905
TMEM147-AS1	100.0174666	-0.018954645	0.278647062	-0.068023848	0.94576665	0.994752905
SPIN1	1557.239738	0.007783152	0.11452675	0.06795925	0.945818073	0.994752905
UBAP1	336.3308637	0.012980907	0.191193799	0.067893977	0.945870033	0.994752905
CDC26	150.5097909	0.01413249	0.208609704	0.06774608	0.945987767	0.994752905
HOOK3	3717.096429	0.008082725	0.119679245	0.067536563	0.946154555	0.994752905
HPD	3.081069214	-0.078282307	1.159151683	-0.067534136	0.946156488	0.994752905
MAP3K9	3.919815502	-0.06494703	0.96394995	-0.067375935	0.946282426	0.994752905
NAA15	1715.862749	-0.013084453	0.195148842	-0.067048583	0.946543027	0.994752905
SNORD13	12.29766489	-0.033964351	0.507033999	-0.066986338	0.94659258	0.994752905
FOPNL	500.9452149	0.010161597	0.152269697	0.066734205	0.946793303	0.994752905
ZNF394	120.4912189	0.01595421	0.239423963	0.066635812	0.946871635	0.994752905
BLOC1S4	172.6460502	-0.011995354	0.180052633	-0.066621375	0.946883129	0.994752905
FAM230E	12.26567044	-0.038641576	0.581174354	-0.066488783	0.946988688	0.994752905
LOC101927950	10.79811805	0.065006894	0.977801167	0.066482733	0.946993504	0.994752905
NUP155	3287.894422	0.007996039	0.120288971	0.066473585	0.947000787	0.994752905
UBR5	12450.45745	0.006397628	0.096275889	0.066450988	0.947018777	0.994752905
EPG5	1186.09763	0.00772989	0.116437014	0.066386879	0.947069816	0.994752905
ANOS1	26.59744683	-0.04221641	0.636025735	-0.066375317	0.947079021	0.994752905
KLRG1	28.81119998	0.029198075	0.439926645	0.066370327	0.947082993	0.994752905
TFPI	628.3795934	-0.018633108	0.280755837	-0.066367659	0.947085118	0.994752905
ZNF589	107.6876538	0.017169013	0.258800824	0.066340644	0.947106625	0.994752905
CPEB3	58.02517995	0.022590844	0.3408559	0.066276817	0.94715744	0.994752905
GNA11	913.0708543	-0.006535037	0.098694252	-0.066214974	0.947206676	0.994752905
ZPR1	496.1568037	-0.009075314	0.137094178	-0.066197659	0.947220461	0.994752905
EDRF1-DT	3.757958314	-0.0710294	1.073590566	-0.066160604	0.947249962	0.994752905
FARSB	1669.965073	-0.00915283	0.138370279	-0.066147367	0.9472605	0.994752905
TMEM52B	1.92155666	0.093293271	1.411256184	0.066106546	0.947293	0.994752905
KIAA1549L	117.6183768	-0.029605812	0.448890631	-0.065953287	0.947415016	0.994752905
LOC100505774	2.915484068	-0.078054987	1.183693416	-0.065941895	0.947424086	0.994752905
ZNF45	323.3478933	-0.009106831	0.138391171	-0.065805003	0.947533073	0.994752905
RBPJ	1551.899087	0.014285814	0.217179076	0.065778963	0.947553805	0.994752905
PTPA	2213.671942	-0.009250564	0.140771175	-0.065713483	0.947605938	0.994752905
BCL6B	1.803496619	-0.09818608	1.494465627	-0.065699792	0.947616838	0.994752905
NAXE	550.0970027	-0.008650818	0.131724092	-0.065673776	0.947637551	0.994752905
LOC729867	27.334369	0.044772678	0.681830135	0.065665443	0.947644185	0.994752905
PTCD3	2219.247243	0.010026771	0.152743854	0.065644351	0.947660978	0.994752905
PDSS2	292.0054257	-0.013611345	0.207483814	-0.06560196	0.947694729	0.994752905
SAC3D1	151.0581962	0.012915803	0.197782164	0.065303171	0.947932617	0.994866585
WDR17	369.009678	-0.010620373	0.162911476	-0.065191068	0.948021873	0.994866585
PRKG2	3.508475559	-0.06539224	1.00348743	-0.065164982	0.948042642	0.994866585
HORMAD2-AS1	3.505054749	-0.069339486	1.065660103	-0.065067169	0.948120521	0.994866585
LOC101927365	51.44612833	0.021983814	0.337899373	0.065060239	0.948126038	0.994866585
DBP	47.13002209	-0.025440376	0.391226444	-0.065027239	0.948152312	0.994866585
SPRYD3	675.5391209	0.013765051	0.212305527	0.064836046	0.948304541	0.994866585
WASH3P	178.3841289	0.013830916	0.213514316	0.064777465	0.948351184	0.994866585
COL4A1	1105.431202	-0.009100701	0.140604333	-0.064725607	0.948392474	0.994866585
NEK1	398.4914428	-0.007729471	0.119634119	-0.064609253	0.948485118	0.994866585
RPL3L	2.11120429	-0.087263716	1.351685557	-0.064559183	0.948524984	0.994866585
SETD2	1278.482005	0.006221544	0.096390093	0.064545474	0.9485359	0.994866585
FXYD1	1.999653594	-0.090798255	1.406959003	-0.06453511	0.948544151	0.994866585
LOC100272217	12.52474614	-0.054695549	0.848199866	-0.06448427	0.948584632	0.994866585
FAS-AS1	57.24505028	0.021438671	0.334111775	0.064166165	0.948837918	0.995061937
PLAU	573.5666475	-0.047067169	0.734889679	-0.064046578	0.948933139	0.995061937
ZNF426	879.7839598	0.007783026	0.121584983	0.064013051	0.948959835	0.995061937
ZNF177	3.588295172	-0.066356755	1.037412066	-0.06396374	0.948999099	0.995061937
ZRANB2	1871.781784	0.00940937	0.147261292	0.063895742	0.949053242	0.995061937
CROCCP3	132.8752388	-0.022811778	0.357591906	-0.063792769	0.949135236	0.995061937
PORCN	117.9935692	-0.016441552	0.257868502	-0.063759441	0.949161774	0.995061937
GRAMD2B	23.70854838	0.026993407	0.424426739	0.063599685	0.949288983	0.995124947
CFAP410	155.8289517	0.011765585	0.185375836	0.063468819	0.949393188	0.995124947
SMURF1	365.0425229	0.008617154	0.135780632	0.063463792	0.949397191	0.995124947
LINC01391	48.7213103	0.022950414	0.362596837	0.063294579	0.949531932	0.995124947
NDUFA8	448.4943188	0.011795649	0.186456059	0.063262354	0.949557593	0.995124947
LUC7L	662.2198854	0.019013171	0.300763862	0.063216273	0.949594286	0.995124947
CEACAM19	72.8244111	0.021634367	0.342353585	0.06319305	0.949612779	0.995124947
NRBF2	228.4068238	0.010701263	0.169542096	0.063118616	0.94967205	0.995128539
LOC100288069	100.5375458	0.030309582	0.482263435	0.062848601	0.949887064	0.995269764
LOC102723322	3.424692824	0.064651978	1.029570191	0.062795114	0.949929656	0.995269764

KRT18P55	1.783476572	0.096381969	1.537372252	0.062692669	0.950011235	0.995269764
THSD4	174.1244313	-0.021211726	0.339473591	-0.062484172	0.950177267	0.995269764
RASA4DP	7.922936558	-0.048193452	0.771661088	-0.062454169	0.950201158	0.995269764
LOC102723439	7.834469641	0.056238942	0.900952067	0.06242168	0.95022703	0.995269764
MPST	678.108416	0.011705034	0.188259638	0.062174951	0.95042351	0.995269764
MAP3K5	241.8912752	-0.014241321	0.229290232	-0.062110457	0.95047487	0.995269764
BTBD19	88.21842606	-0.018181756	0.292784417	-0.062099465	0.950483623	0.995269764
TMEM87A	1179.559518	0.008017692	0.129145806	0.062082477	0.950497151	0.995269764
DPY30	234.8426134	0.014354484	0.231771533	0.061933765	0.950615578	0.995269764
PGAM1	2697.87637	-0.009080475	0.146620677	-0.061931751	0.950617183	0.995269764
C11orf1	17.02103319	-0.035990415	0.583296284	-0.061701774	0.950800327	0.995269764
NRXN1	388.7383251	0.010391353	0.168638085	0.061619256	0.950866042	0.995269764
POU5F2	30.26510486	0.059306218	0.962877611	0.061592686	0.950887202	0.995269764
GTF3C5	774.2288605	-0.006385454	0.10372962	-0.061558633	0.95091432	0.995269764
CREB5	74.23669989	0.016407288	0.266883319	0.061477384	0.950979025	0.995269764
SLC29A3	30.66380065	-0.02625569	0.427370703	-0.0614354	0.951012461	0.995269764
ROGDI	68.18338925	-0.020111639	0.327756022	-0.061361616	0.951071221	0.995269764
GRSF1	1178.311019	-0.007666071	0.125159965	-0.061250182	0.951159965	0.995269764
FBXO2	3.365959403	-0.073921731	1.207253819	-0.061231308	0.951174996	0.995269764
COQ5	412.1050371	-0.011206533	0.183399435	-0.06110451	0.951275977	0.995269764
HLA-DPA1	3.22687648	-0.063586444	1.040961752	-0.061084323	0.951292054	0.995269764
MAP1LC3B2	2.27346672	0.084225243	1.379473069	0.061056098	0.951314533	0.995269764
AK8	3.122878459	-0.069822558	1.143886713	-0.061039749	0.951327553	0.995269764
RCBTB1	685.9647831	0.007346749	0.12048169	0.060978136	0.951376621	0.995269764
HMCN1	10480.17765	0.017778156	0.291827792	0.060920024	0.951422902	0.995269764
MAGI2	65.41813126	-0.016146145	0.265431031	-0.060829908	0.951494672	0.995269764
SNORA24B	2.131205231	-0.081950262	1.347580128	-0.060812905	0.951508213	0.995269764
CLIP2	163.2897898	-0.03081809	0.506781784	-0.060811362	0.951509442	0.995269764
LOC730098	15.89392907	0.035474056	0.583749908	0.06076927	0.951542964	0.995269764
MUC5B	6.192064701	0.063841119	1.051659782	0.060705106	0.951594065	0.995269764
SNX18	1053.679425	0.007031307	0.116290898	0.060463088	0.951786813	0.995379107
LINC00680-GUSBP4	7.127664378	0.057503096	0.951509217	0.060433567	0.951810325	0.995379107
VAMP8	2.049926398	-0.082135186	1.360931353	-0.060352189	0.951875137	0.995388471
MDH2	1024.066488	-0.009321073	0.154773422	-0.060223992	0.951977238	0.995418891
NPIPB5	20.53520826	0.033428581	0.555519148	0.06017539	0.952015946	0.995418891
LMAN2	1128.877349	-0.006103146	0.101821663	-0.059939562	0.952203771	0.995518708
TSGA10	38.78691389	-0.023731332	0.39608173	-0.059915241	0.952223141	0.995518708
HACD2	720.9061739	0.011627707	0.194757188	0.059703608	0.952391698	0.995528063
TMEM63B	647.0719133	-0.008733001	0.146302043	-0.059691589	0.95240127	0.995528063
ADRM1	596.665337	0.011595164	0.194860092	0.059505072	0.952549826	0.995528063
LINC02589	2.394131467	-0.070723054	1.188858206	-0.059488216	0.952563251	0.995528063
RPRD2	897.3251663	0.006672242	0.112218305	0.059457699	0.952587557	0.995528063
RNF14	448.6025818	-0.00896499	0.150896098	-0.059411675	0.952624214	0.995528063
NUMBL	724.8655487	0.007221568	0.121622855	0.059376732	0.952652045	0.995528063
CSNKA2IP	2.690672195	0.074578782	1.256743758	0.05934287	0.952679016	0.995528063
TSEN34	256.8181758	0.009934193	0.167835323	0.059190123	0.952800677	0.995564336
LOC100144595	4.668477685	0.058315553	0.985742654	0.059159004	0.952825463	0.995564336
THAP10	80.51055435	-0.014700914	0.249210808	-0.058989873	0.952960174	0.995621195
CENPBD1P1	410.1004966	0.008840161	0.14999066	0.058938078	0.953001429	0.995621195
GABARAPL2	512.8834971	-0.009044026	0.153730609	-0.058830351	0.953087234	0.995621195
ACO1	752.3713839	0.007253455	0.123336901	0.058810098	0.953103366	0.995621195
RCCD1	398.7925084	-0.008871675	0.151432213	-0.058585124	0.95328256	0.995722537
TCTEX1D2	34.88401429	0.020991201	0.358902138	0.058487254	0.953360515	0.995722537
USP46	563.1192536	0.008525456	0.146290572	0.058277549	0.953527551	0.995722537
BRCA2	628.7620857	-0.009176706	0.157773123	-0.05816394	0.953618044	0.995722537
TOB1-AS1	9.767552156	-0.035999754	0.619211942	-0.058138017	0.953638692	0.995722537
OAS1	4.412099474	-0.075775756	1.30376626	-0.058120661	0.953652517	0.995722537
MROH1	626.0592868	-0.0103755	0.178586835	-0.058097789	0.953670735	0.995722537
DCBLD1	362.9127291	-0.013932473	0.240576066	-0.057912962	0.953817958	0.995722537
SCAF4	440.3144758	-0.007157607	0.123705108	-0.057860242	0.953859952	0.995722537
PDE6G	4.489145885	-0.052159054	0.901933952	-0.057830237	0.953883853	0.995722537
GPATCH11	559.9272867	-0.014849741	0.256890251	-0.05780578	0.953903334	0.995722537
SNHG14	2086.412026	0.015746603	0.272624894	0.057759227	0.953940416	0.995722537
TSNAXIP1	14.30662873	0.03831022	0.664293988	0.05767058	0.954011028	0.995722537
RNF144A-AS1	261.0629591	0.020310163	0.352243735	0.0576594	0.954019934	0.995722537
H2BE1	5.489079947	0.047896178	0.831798294	0.057581482	0.954082001	0.995722537
LOC105371849	8.978430711	-0.041878144	0.728878829	-0.057455564	0.954182302	0.995722537
ROR2	912.8358858	0.015340395	0.267022861	0.057449746	0.954186937	0.995722537
NSUN2	1473.112776	-0.011361146	0.19784109	-0.057425614	0.95420616	0.995722537
LINC02076	2.382397166	0.075017457	1.308740368	0.057320351	0.95429001	0.995751726
DHRS4-AS1	332.1548748	-0.009434575	0.164902611	-0.057213013	0.954375512	0.995782635
SLA	70.86832877	0.059579638	1.04285042	0.057131528	0.954440422	0.995782747
LINC00696	8.298304065	-0.036620513	0.641961617	-0.057044708	0.954509581	0.995782747
CERS5	404.304392	-0.007498225	0.131572794	-0.056989176	0.954553818	0.995782747
UTS2B	10.54189892	0.031577883	0.555661655	0.056829336	0.954681145	0.995782747
SLC25A14	90.53292126	-0.011397953	0.20057305	-0.056826941	0.954683053	0.995782747
PLXND1	2745.631448	-0.011270246	0.198542936	-0.056764782	0.954732569	0.995782747
C6orf47	263.410059	-0.008177182	0.14416285	-0.056721836	0.954766779	0.995782747
CTAGE7P	27.09684146	-0.038953382	0.688891446	-0.056545023	0.95490763	0.995830346
CCR10	6.107853841	-0.042787418	0.756974636	-0.056524243	0.954924183	0.995830346
ZNF695	103.8114459	-0.02371982	0.420460784	-0.056413871	0.955012107	0.995863757
ALPK1	128.2145254	-0.023824877	0.422871131	-0.056340751	0.955070356	0.995866222
WIPF1	726.6636584	-0.017337869	0.308465492	-0.056206836	0.955177035	0.995915912
TMEM254	242.9778077	0.012144631	0.21632521	0.05614062	0.955229785	0.995915912
DCAF7	3257.991543	-0.009268542	0.165781228	-0.055908272	0.955414881	0.995951851
MEDAG	2.712093839	-0.099355705	1.777167684	-0.05590677	0.955416078	0.995951851
LOC100507557	30.23162289	0.021732766	0.388870641	0.055886878	0.955431925	0.995951851
TOX	175.964268	-0.019517904	0.3499505	-0.055773329	0.955522382	0.995972332
CRLS1	427.3257026	-0.007611601	0.136851152	-0.055619562	0.95564488	0.995972332
LOC101927179	2.650197603	-0.083290535	1.499350853	-0.055551064	0.95569945	0.995972332

LINC01181	3.689978426	0.054164008	0.9762452	0.055481971	0.955754493	0.995972332
RNU12	10.69025915	-0.045066722	0.81266222	-0.055455663	0.955775452	0.995972332
NOP10	170.3155488	-0.010948575	0.197862029	-0.055334393	0.955872063	0.995972332
LOC100506083	8.595862935	-0.04452663	0.805400317	-0.055285091	0.955911339	0.995972332
MAGI3	372.5240754	0.008755699	0.158550179	0.055223519	0.955960392	0.995972332
RPS6KC1	922.172067	-0.006191103	0.112208089	-0.055175192	0.955998893	0.995972332
SRGAP2C	2.931717953	0.064494278	1.169685763	0.055138124	0.956028424	0.995972332
ATP2C1	2052.032815	-0.006553549	0.11938072	-0.05489621	0.956221151	0.995972332
RAD18	339.5800665	-0.00767661	0.139881744	-0.054879282	0.956234638	0.995972332
MYCBP2	3465.128412	-0.007509136	0.136873548	-0.054861852	0.956248523	0.995972332
ABHD14B	277.2719032	0.009155995	0.167010439	0.054822888	0.956279565	0.995972332
MED8	512.3550803	-0.009141613	0.166871305	-0.054782413	0.956311812	0.995972332
MCTP1	21.05094811	0.029410423	0.537918251	0.054674521	0.956397768	0.995972332
EFCAB10	19.52899434	-0.027700897	0.50669683	-0.054669568	0.956401714	0.995972332
FLJ39095	15.51548416	-0.034518946	0.633590599	-0.054481468	0.956551573	0.995990216
CRABP2	185.2526023	-0.019332	0.355352903	-0.054402258	0.95661468	0.995990216
C1orf122	154.4539951	-0.013811854	0.254065011	-0.054363463	0.956645588	0.995990216
SHROOM2	37.90337168	0.021996675	0.404631075	0.054362298	0.956646516	0.995990216
ZNF230	70.3162068	-0.012940194	0.23847537	-0.054262183	0.956726279	0.995990216
PEAK1	2259.788779	0.006091889	0.11247844	0.054160502	0.956807289	0.995990216
C10orf62	2.318056556	0.074354844	1.375804697	0.054044621	0.956899613	0.995990216
SNRNP70	897.5957431	0.007583361	0.14048994	0.053977967	0.956952718	0.995990216
GRB2	1594.365219	-0.006416725	0.119020951	-0.053912563	0.957004828	0.995990216
MACROD2	2.317936511	-0.070015511	1.298770736	-0.053909061	0.957007618	0.995990216
LINC01029	6.014803995	-0.043181901	0.801706547	-0.053862478	0.957044731	0.995990216
ING2	36.73895414	-0.017695981	0.329191648	-0.053755863	0.957129675	0.995990216
CRELD2	291.3057332	-0.008944342	0.166449647	-0.053736026	0.95714548	0.995990216
TMEM108	9.521543326	0.042003136	0.785534885	0.053470746	0.957356839	0.996125109
KLLN	7.72945081	0.037810962	0.707633097	0.053433003	0.95738691	0.996125109
YKT6	1267.481521	-0.005221597	0.098126694	-0.053212806	0.957562352	0.996183877
OSTC	1449.453751	0.008024055	0.150922831	0.053166605	0.957599163	0.996183877
MIR3138	2.025916732	0.071696971	1.34948601	0.053129096	0.957629049	0.996183877
P2RX6	8.975414004	0.060547722	1.1410406	0.0530636	0.957681234	0.996183877
MIR1299	2.637008798	0.070873122	1.336943716	0.053011298	0.957722906	0.996183877
MFGE8	781.42934	0.013207228	0.249685892	0.052895372	0.957815272	0.996192951
ANO4	9.664305199	-0.038669021	0.732304411	-0.052804572	0.957887618	0.996192951
DHRS2	5.159991791	-0.043767882	0.829096333	-0.052789863	0.957899338	0.996192951
SEC62	3907.720065	0.005630194	0.107316824	0.052463295	0.958159541	0.996402172
MRPL24	240.3736077	-0.011101895	0.211880258	-0.052397022	0.958212347	0.996402172
RINL	131.3447829	0.010596028	0.202889566	0.052225592	0.958348941	0.996452603
RHOT1	622.5705568	0.010402173	0.199291385	0.052195799	0.95837268	0.996452603
SH3GL1P2	6.977870901	0.040047343	0.769336063	0.05205442	0.958485331	0.996475988
LOC105371789	19.31449678	0.040398811	0.779498704	0.051826655	0.958666816	0.996475988
LOC102724720	2.908788604	-0.055894106	1.081300555	-0.051691554	0.958774467	0.996475988
ABCA5	284.6702673	-0.013652073	0.264347929	-0.051644333	0.958812094	0.996475988
VPS54	953.02818	0.004978124	0.096643446	0.051510205	0.95891897	0.996475988
FMO5	46.98818874	-0.014972583	0.290728067	-0.0515003	0.958926863	0.996475988
NAALADL1	5.063127004	-0.054355024	1.059280398	-0.05131316	0.959075982	0.996475988
TEAD4	194.7382203	-0.008331186	0.162459555	-0.051281602	0.959101128	0.996475988
CNTNAP1	193.6406848	-0.012169637	0.237334638	-0.051276279	0.95910537	0.996475988
RNASE4	79.58837472	0.024481502	0.479072904	0.051101829	0.959244378	0.996475988
PIPSL	11.44228255	-0.027865785	0.546679161	-0.050972831	0.95934717	0.996475988
SEPSECS	267.5126569	-0.011007114	0.216057997	-0.050945183	0.959369201	0.996475988
PKIA-AS1	10.95574947	-0.049280228	0.967754085	-0.050922263	0.959387465	0.996475988
FBXO17	390.2491762	-0.017332036	0.340826328	-0.050852985	0.95944267	0.996475988
LAYN	320.3429541	-0.008743723	0.172060374	-0.050817758	0.95947074	0.996475988
SNORD114-29	8.586576265	-0.051945397	1.022962255	-0.050779388	0.959501316	0.996475988
CCNB1	1355.699227	-0.00567164	0.111775019	-0.050741575	0.959531448	0.996475988
LOC100506885	8.566779644	-0.043830664	0.86458522	-0.050695598	0.959568085	0.996475988
TMEM88	6.404348359	-0.040500361	0.798933251	-0.050693047	0.959570117	0.996475988
MGARP	8.929248426	0.037221011	0.734551094	0.05067178	0.959587064	0.996475988
MRPL52	413.0538772	-0.006203506	0.122678494	-0.050567183	0.959670413	0.996475988
ACAP3	1078.596032	-0.010712326	0.212302391	-0.05045787	0.959757522	0.996475988
LOC105376306	11.11200006	-0.038036416	0.755716943	-0.050331565	0.95985817	0.996475988
NAA38	285.3178785	0.008734795	0.173824297	0.050250715	0.959922598	0.996475988
MARK2	492.5371991	0.00589944	0.117568444	0.050178772	0.959979928	0.996475988
CACNA2D2	12.85270596	0.036290488	0.723409137	0.050165924	0.959990166	0.996475988
P4HA1	2456.591799	0.00545349	0.108804236	0.050122033	0.960025142	0.996475988
SMIM38	4.42265584	-0.061998605	1.239346567	-0.050025236	0.960102278	0.996475988
FAM3A	350.9358647	-0.009719131	0.19434331	-0.050010115	0.960114328	0.996475988
CTNNAL1	949.043912	-0.005398671	0.107990517	-0.049992086	0.960128695	0.996475988
MISP3	3.672926387	-0.058840587	1.180809179	-0.049830733	0.960257275	0.996475988
PWAR1	6.306999509	0.050709757	1.018860442	0.049771053	0.960304834	0.996475988
FRG1JP	129.1588746	0.020842069	0.418962123	0.049746906	0.960324077	0.996475988
GRAMD1B	188.5472505	0.011328605	0.227897055	0.049709307	0.960354039	0.996475988
DEAF1	477.6780653	-0.006335416	0.127700945	-0.049611345	0.960432105	0.996475988
ZNF835	2.690598251	-0.06436391	1.298149585	-0.049581274	0.96045607	0.996475988
CCNL1	676.6259956	0.012539	0.25302965	0.049555458	0.960476643	0.996475988
LINC01023	6.348687988	0.049622806	1.002462002	0.049500934	0.960520093	0.996475988
COX8A	554.1653153	-0.007935457	0.161136031	-0.049246944	0.960722501	0.99653925
AIRN	7.975282435	0.045521382	0.925007603	0.0492119	0.960750428	0.99653925
PMF1	298.0923863	-0.007667456	0.155878375	-0.04918871	0.960768909	0.99653925
NDUFB1	54.5946704	-0.01385081	0.282246987	-0.049073366	0.960860828	0.99653925
COBLL1	183.4759464	0.010328852	0.211018044	0.048947721	0.960960959	0.99653925
ZNRD2	153.4696856	0.008677703	0.177303725	0.048942585	0.960965052	0.99653925
LOC100506497	3.660683139	-0.079019632	1.617729145	-0.048846021	0.961042006	0.99653925
ANKRD45	5.262789749	0.042413234	0.868611143	0.048828793	0.961055736	0.99653925
USP36	1448.456071	-0.005394677	0.110562846	-0.048792857	0.961084375	0.99653925
ZNF574	322.7328718	0.006892404	0.141667592	0.048651947	0.961196671	0.996553966
IFFO1	518.2792514	-0.010080872	0.207580221	-0.048563739	0.961266968	0.996553966

MTLN	29.44458	0.02109785	0.43464202	0.048540752	0.961285288	0.996553966
CEP120	564.7600012	0.006218432	0.128230005	0.048494359	0.96132226	0.996553966
NUDT11	233.4848428	-0.007756061	0.160378313	-0.048361035	0.961428513	0.996606137
TACR2	4.436539739	-0.045486861	0.942060916	-0.048284416	0.961489575	0.99661146
FAM184A	9.384649047	0.029380186	0.609470892	0.048206053	0.961552026	0.996618223
TMEM143	196.1936323	0.012481	0.259949322	0.048013205	0.961705719	0.996646546
NMT1	1963.144442	0.005183757	0.108131302	0.047939468	0.961764485	0.996646546
KIF25	14.15104435	-0.025300002	0.528308755	-0.047888667	0.961804971	0.996646546
LINC01572	104.7992378	0.021859191	0.45664717	0.047868885	0.961820738	0.996646546
CD226	14.4354675	-0.030404198	0.63682666	-0.047743287	0.961920835	0.996646546
BAAT	6.381108685	-0.040955449	0.859997684	-0.047622743	0.962016906	0.996646546
SOD2	1443.724629	-0.005902874	0.124059523	-0.04758098	0.962050191	0.996646546
LINC00346	33.30151351	0.024343561	0.511812678	0.047563419	0.962064187	0.996646546
NDUFB2	288.9276325	-0.006179745	0.130419847	-0.047383473	0.962207601	0.996646546
PMEL	7.130924556	0.037429686	0.790690132	0.047337995	0.962243846	0.996646546
LOC101928725	10.71034279	-0.029304102	0.619091229	-0.047334062	0.962246981	0.996646546
RNF168	1824.591612	0.009272808	0.195919636	0.047329653	0.962250495	0.996646546
RBM38	112.4227976	0.011604002	0.245847605	0.04719998	0.962353844	0.996695658
FOXF2	443.3762375	-0.0112427	0.239154179	-0.04701026	0.962505051	0.996744981
ZNF358	406.2600852	0.014409346	0.306627198	0.046993046	0.96251877	0.996744981
ZNF3	239.5408291	0.007956785	0.169546947	0.046929685	0.962569269	0.996744981
CCNF	807.2972903	-0.006753929	0.145180657	-0.046520858	0.96289511	0.996984143
PDHB	576.3867458	0.00666853	0.143431952	0.046492641	0.962917599	0.996984143
SLC37A4	460.4349712	-0.006139077	0.132229617	-0.0464274	0.962969598	0.996984143
CCM2	159.6167535	0.008420259	0.182034163	0.046256479	0.963105827	0.996984143
C3orf67	52.44580573	-0.014858634	0.321790922	-0.046174807	0.963170922	0.996984143
FDPS	867.6393359	0.010709477	0.232627576	0.046037006	0.963280754	0.996984143
ZNF33A	502.285222	0.005339529	0.116194727	0.045953282	0.963347486	0.996984143
ZNF649	146.3087987	-0.012434649	0.270807906	-0.045916862	0.963376514	0.996984143
RTN1	11.56473525	0.028731147	0.626080669	0.045890487	0.963397537	0.996984143
SNORA9	36.39136407	-0.01848493	0.403227556	-0.045842428	0.963435842	0.996984143
LINC00163	7.956838055	-0.035462729	0.773710884	-0.0458346	0.963442081	0.996984143
RNF19A	878.6925009	-0.005077969	0.110878592	-0.045797563	0.963471601	0.996984143
EME1	305.9614289	-0.009682728	0.21223502	-0.045622667	0.963611002	0.997013439
STEAP3	225.4641386	0.008695106	0.190591652	0.045621653	0.96361181	0.997013439
SNORA80E	6.19877691	0.04279026	0.941993592	0.045425214	0.963768384	0.997117545
ZNF827	468.3521164	-0.006296344	0.139703818	-0.045069234	0.964052124	0.997213377
DROSHA	1398.701152	-0.003733275	0.082868318	-0.045050687	0.964066907	0.997213377
SEMA6C	138.1590063	0.010442783	0.231939953	0.045023647	0.964088461	0.997213377
GPR37	122.9427502	-0.009954987	0.221451013	-0.044953449	0.964144414	0.997213377
TMEM198B	115.8668667	-0.012720933	0.283778829	-0.044826929	0.96424526	0.997213377
PPWD1	342.9360935	0.007187692	0.160602526	0.044754537	0.964302963	0.997213377
SIDT2	418.9195782	0.009472161	0.211817729	0.044718453	0.964331725	0.997213377
DIMT1	314.5650758	0.006393744	0.143109492	0.044677291	0.964364535	0.997213377
BBX	1878.821675	-0.004826593	0.108032716	-0.044677142	0.964364654	0.997213377
SSH3	200.2038299	-0.006838144	0.153333579	-0.044596517	0.964428919	0.997221965
C2orf15	12.12715043	-0.033624911	0.755204736	-0.044524232	0.964486537	0.997223678
ZNFX1	923.7900787	-0.005391322	0.121335618	-0.044433139	0.964559147	0.997240891
RPL31	2513.98231	-0.006834558	0.154380948	-0.044270732	0.964688601	0.997316869
TBX2-AS1	55.55776657	0.018592417	0.421388128	0.044121834	0.964807289	0.997331566
TFE3	343.131601	0.008171088	0.185342068	0.044086526	0.964835433	0.997331566
LOC100652999	3.169599054	-0.053871952	1.223187748	-0.044042259	0.964870718	0.997331566
PPP2R5C	2021.982994	-0.008605142	0.19631796	-0.043832676	0.96503778	0.997446392
RPL36A	153.5288399	-0.009378907	0.214922257	-0.043638602	0.965192481	0.997476527
MIR4659A	11.91146099	-0.037461527	0.859758167	-0.043572168	0.965245437	0.997476527
TGFBI	34152.62514	-0.013606488	0.312908224	-0.043483957	0.965315753	0.997476527
NOS1AP	14.05387452	0.025644168	0.59033273	0.043440193	0.965350638	0.997476527
SNORA13	6.722863149	0.034092055	0.786582179	0.043342013	0.965428901	0.997476527
DIXDC1	447.7610845	0.007417665	0.171466814	0.04326006	0.965494229	0.997476527
ZNF579	168.2938868	-0.008722778	0.20182053	-0.043220468	0.965525789	0.997476527
ARHGAP22	160.8183555	-0.009105647	0.211024964	-0.043149619	0.965582266	0.997476527
YDJC	411.3631731	-0.009302341	0.215702503	-0.043125792	0.96560126	0.997476527
PAN2	1099.76385	0.010749567	0.249485774	0.043086892	0.965632268	0.997476527
DCTN1-AS1	15.32015915	-0.029741312	0.691433858	-0.043013966	0.965690401	0.997476527
TCTEX1D4	9.388034955	0.029084078	0.679530965	0.042800225	0.965860784	0.997476527
CNN2	2204.195571	0.009298371	0.217347661	0.042781095	0.965876034	0.997476527
MED11	92.05582042	-0.010400514	0.24420083	-0.042590001	0.966028366	0.997476527
PTOV1-AS1	172.087297	0.008240903	0.194678516	0.04233083	0.966234968	0.997476527
BORCS5	279.1257476	-0.006293147	0.148764725	-0.042302685	0.966257405	0.997476527
SH3BP4	2157.479885	-0.008749914	0.206989351	-0.04227229	0.966281635	0.997476527
PARD3-AS1	2.378122814	0.062939109	1.492703387	0.042164511	0.966367553	0.997476527
LINC00942	2.343721846	0.05081432	1.210212533	0.04198793	0.96650832	0.997476527
OSBPL8	3189.895684	-0.006661274	0.15912866	-0.04186093	0.966609562	0.997476527
CENPQ	256.5205744	-0.006452754	0.154370573	-0.041800412	0.966657806	0.997476527
FOXN3	1593.435388	0.009421223	0.225428059	0.041792593	0.966664039	0.997476527
HOXD3	62.13525204	-0.015387192	0.368439284	-0.041763169	0.966687496	0.997476527
KCNJ13	36.72515662	-0.027748652	0.664736474	-0.041743838	0.966702907	0.997476527
LOC100287896	54.67451613	0.0181921	0.435826448	0.041741615	0.966704679	0.997476527
POTEC	13.5672607	0.035556282	0.859643556	0.041361657	0.96700758	0.997476527
PPIF	967.5307836	-0.006149848	0.148703955	-0.04135632	0.967011835	0.997476527
CPZ	3.769295847	0.043974287	1.063833608	0.041335682	0.967028287	0.997476527
ETS1	1465.651271	-0.010369347	0.25240892	-0.041081539	0.967230892	0.997476527
LOC101928809	4.529083372	-0.052310857	1.274056212	-0.041058515	0.967249247	0.997476527
RNASE10	29.30646195	0.02538502	0.61947583	0.040978224	0.967313256	0.997476527
PKN2	1158.274577	0.006456124	0.157641156	0.040954556	0.967332124	0.997476527
PLEK2	6.700517254	-0.029925946	0.730745632	-0.040952617	0.96733367	0.997476527
XXYLT1-AS2	11.37653731	0.047782769	1.16749309	0.040927667	0.967353561	0.997476527
WARS2-AS1	294.2820376	0.006681665	0.163659905	0.040826524	0.967434194	0.997476527
JHY	61.89401775	0.013029791	0.31937283	0.040798059	0.967456887	0.997476527
BICRAL	487.2691732	0.010659008	0.261829366	0.040709751	0.967527288	0.997476527

PCDH17	644.7332529	0.020868178	0.515514798	0.040480269	0.967710237	0.997476527
ISCA1	177.6312668	0.006765408	0.167575712	0.040372248	0.967796355	0.997476527
CSE1L-AS1	4.080133053	-0.042112903	1.044619742	-0.040314099	0.967842714	0.997476527
CLTRN	2.030079679	-0.065584149	1.629530728	-0.040247262	0.967895999	0.997476527
KCNIP2	22.09802014	-0.027356704	0.679967047	-0.040232397	0.967907849	0.997476527
SLC22A18	48.01905323	-0.017379995	0.433849814	-0.040059933	0.968045345	0.997476527
H2AZ1-DT	4.248790879	-0.037494213	0.938281549	-0.039960514	0.968124606	0.997476527
GCA	51.28202895	-0.013706069	0.344461173	-0.039789881	0.968260643	0.997476527
CTDNEP1	1525.793561	0.006372026	0.160636892	0.039667264	0.968358401	0.997476527
C1QTNF12	6.775227927	0.03415566	0.861198622	0.039660607	0.968363708	0.997476527
SEMA4D	16.06231401	-0.027980577	0.706656092	-0.039595749	0.968415417	0.997476527
GUCD1	844.7920608	-0.00559754	0.141542851	-0.039546611	0.968454592	0.997476527
SARDH	2.959809174	-0.046609223	1.178951341	-0.039534475	0.968464268	0.997476527
LOC728743	31.62910887	0.01842373	0.466665986	0.039479479	0.968508114	0.997476527
CASP16P	20.69149788	-0.018160314	0.460486859	-0.039437205	0.968541818	0.997476527
TCTN3	525.5454364	0.004996548	0.126864847	0.039384808	0.968583592	0.997476527
OCLN	6.5612909	-0.029265502	0.744179526	-0.039325862	0.968630587	0.997476527
TNFRSF10B	4090.570013	-0.010270757	0.261724473	-0.039242632	0.968696944	0.997476527
ZNF256	88.1254728	-0.012967039	0.331076543	-0.039166287	0.968757812	0.997476527
LINC01004	152.3754944	0.017486194	0.44806595	0.039025937	0.968869709	0.997476527
RAI14	2066.776226	0.006776993	0.174952312	0.038736232	0.969100686	0.997476527
LOC101927050	2.805779184	-0.046715019	1.209887756	-0.038611035	0.969200504	0.997476527
LGR5	5.970484416	0.039505478	1.023442024	0.038600601	0.969208823	0.997476527
SCO2	15.20262386	-0.024221649	0.628287897	-0.038551832	0.969247706	0.997476527
N4BP2L1	23.69852957	0.015246328	0.395505522	0.038548964	0.969249993	0.997476527
RELCH	613.0226637	-0.00839273	0.218603356	-0.038392505	0.969374737	0.997476527
SPRYD4	302.624265	0.006707313	0.175075208	0.038311037	0.969439691	0.997476527
YIF1A	648.9328595	0.008736588	0.228516755	0.038231717	0.969502933	0.997476527
ATP5MPL	405.7620767	-0.005798314	0.151754276	-0.038208569	0.969521389	0.997476527
PAPPA	1630.147517	-0.03663973	0.959393151	-0.038190527	0.969535774	0.997476527
NDUFS7	373.2015678	-0.005589406	0.146364772	-0.038188194	0.969537634	0.997476527
PNKD	186.5430034	-0.006612312	0.173630868	-0.038082583	0.969621838	0.997476527
SNORD114-30	7.503768287	0.028945672	0.762691032	0.037952029	0.96972593	0.997476527
UBE2E1-AS1	2.818689556	0.052937424	1.399915798	0.03781472	0.969835408	0.997476527
SLC25A21	34.10785149	-0.013234031	0.350026804	-0.037808621	0.969840271	0.997476527
MYOZ3	4.629000486	-0.03341276	0.885132341	-0.037748886	0.969887899	0.997476527
SQLE	4587.339974	-0.007167635	0.189957673	-0.037732801	0.969900723	0.997476527
SPANXB1	1.872452252	0.075719728	2.006961105	0.037728548	0.969904114	0.997476527
HTATIP2	24.54169509	-0.023802588	0.631909296	-0.037667729	0.969952606	0.997476527
BCL2L1	143.1416575	-0.007141071	0.189674672	-0.037649047	0.969967502	0.997476527
CORO6	85.01462583	0.011764595	0.312711508	0.03762124	0.969989673	0.997476527
TBC1D7	87.50898485	-0.00920568	0.245018088	-0.037571432	0.970029386	0.997476527
GPRC5A	11.47393922	-0.040838034	1.087792676	-0.037542112	0.970052764	0.997476527
RRP1B	1291.050421	0.004443569	0.11845895	0.037511467	0.970077197	0.997476527
ARL1	1600.157988	-0.006074842	0.162248613	-0.037441566	0.970132931	0.997476527
ASL	434.3053403	0.006555499	0.175160344	0.037425705	0.970145578	0.997476527
ZNF580	125.0108251	-0.007066979	0.189046201	-0.037382283	0.970180199	0.997476527
MTRES1	111.736672	0.008857026	0.237051268	0.037363337	0.970195305	0.997476527
POP5	423.2466103	0.00763576	0.204669391	0.037307775	0.970239606	0.997476527
CD99L2	1216.566067	0.007306271	0.195936031	0.037289064	0.970254525	0.997476527
HTATSF1P2	63.95705412	0.014205007	0.381065555	0.037277069	0.970264089	0.997476527
CNOT7	3717.257509	0.003798114	0.101973243	0.037246186	0.970288713	0.997476527
HCAR1	52.70996003	-0.012313763	0.331142613	-0.03718568	0.970336957	0.997476527
LOC101927051	11.35848389	0.02267287	0.611066568	0.037103764	0.970402271	0.997476527
MFSD5	299.0282694	-0.005871047	0.158556061	-0.037028211	0.970462512	0.997476527
RAI1-AS1	9.665091643	0.031186439	0.842237763	0.03702807	0.970462624	0.997476527
CDH23	9.427859345	0.02439268	0.659467884	0.036988427	0.970494233	0.997476527
PDZRN3	265.7578915	0.006394304	0.172899372	0.036982808	0.970498713	0.997476527
RPUSD2	306.0081649	0.005668085	0.153380534	0.036954395	0.970521368	0.997476527
CYFIP2	9.177178347	0.028448163	0.771864599	0.036856416	0.970599491	0.997476527
PRSS27	9.978107006	0.021340836	0.584277083	0.036525197	0.970863588	0.997476527
CUL5	705.1545346	0.004122959	0.112888495	0.0365224	0.970865818	0.997476527
SHLD2P3	7.215105763	0.026043677	0.714407464	0.036454934	0.970919612	0.997476527
LOC101927245	26.07908754	0.021693451	0.595579598	0.0364241	0.970944198	0.997476527
ZNF174	95.10045731	-0.009844963	0.270998816	-0.036328434	0.971020478	0.997476527
LOC105374952	27.31458415	0.01416386	0.391153681	0.036210474	0.971114535	0.997476527
ZSWIM5	3.484714318	0.041568662	1.14854688	0.036192395	0.97112895	0.997476527
RECK	563.0385902	-0.004960509	0.137639255	-0.036039932	0.971250519	0.997476527
PCBP2-OT1	21.5331748	0.021324326	0.591696412	0.036039303	0.97125102	0.997476527
LOC107985544	4.537006927	0.036097048	1.004937972	0.035919677	0.971346406	0.997476527
F8A1	17.62226447	-0.01887862	0.526868852	-0.035831725	0.971416537	0.997476527
SMARCA4	3256.382188	-0.004103313	0.115211827	-0.035615384	0.971589041	0.997476527
EHMT1	1304.101738	-0.003517696	0.099166664	-0.035472568	0.97170292	0.997476527
SCAF1	1415.696774	0.003592551	0.101423888	0.035421152	0.971743918	0.997476527
HYKK	149.3864573	0.007760629	0.220216844	0.035240852	0.971887688	0.997476527
TRIP10	718.251134	0.004302094	0.122954407	0.034989344	0.972088238	0.997476527
CACNA2D3	3.444444883	0.052664719	1.506485262	0.034958669	0.972112698	0.997476527
KDM4B	770.7299416	0.003701541	0.105989116	0.034923787	0.972140513	0.997476527
PTPN11	7306.030032	0.003860385	0.1106068	0.034901878	0.972157983	0.997476527
DIAPH3-AS1	4.5300667	-0.046594724	1.336965613	-0.034851101	0.972198472	0.997476527
RNASEH1	1306.381612	-0.004738282	0.13629848	-0.03476401	0.972267919	0.997476527
MIR5581	7.551373082	-0.031712418	0.920487343	-0.03445177	0.972516902	0.997476527
ZNF787	505.4193184	0.004114519	0.119434546	0.034449988	0.972518322	0.997476527
NDRG4	246.7938836	0.006561449	0.19174202	0.034220192	0.972701565	0.997476527
NLRP6	2.026673465	-0.0447048	1.313933016	-0.034023652	0.97285829	0.997476527
CBLN2	126.7053627	-0.017973988	0.528587827	-0.034003788	0.97287413	0.997476527
SNPH	11.41298836	0.027550703	0.810819047	0.033978856	0.972894012	0.997476527
AZIN2	30.15360026	0.015389493	0.454250759	0.033878849	0.97297376	0.997476527
SUPT7L	861.6757544	0.004278336	0.12629088	0.033876837	0.972975364	0.997476527
NPIPB12	4.642393121	-0.036382351	1.076447845	-0.033798527	0.973037811	0.997476527

LOC646030	2.206202199	-0.047689623	1.420008202	-0.033584048	0.973208843	0.997476527
BAIAP2-DT	271.3554767	0.011032854	0.328994875	0.033535034	0.973247928	0.997476527
XPO5	3181.944156	-0.00398141	0.119044824	-0.033444628	0.973320022	0.997476527
THAP11	750.5902862	-0.004687529	0.14046409	-0.033371729	0.973378154	0.997476527
GPSM3	4.872841469	-0.026309315	0.791869384	-0.033224311	0.973495711	0.997476527
RBM5-AS1	129.0810815	0.013416762	0.4044129	0.0331759	0.973534317	0.997476527
RIBC1	16.51833111	-0.017229284	0.520124344	-0.033125317	0.973574654	0.997476527
ZNF556	4.086067436	-0.038078145	1.156049006	-0.032938175	0.97372389	0.997476527
NMRAL2P	2.232361411	0.042736096	1.298930316	0.032900992	0.973753542	0.997476527
TRPS1	2869.644237	0.004962322	0.151392481	0.032777865	0.97385173	0.997476527
PLCG1-AS1	19.92170943	-0.019249317	0.589117539	-0.032674832	0.973933894	0.997476527
CTXN1	27.48348082	0.014253495	0.437680806	0.032565959	0.974020716	0.997476527
DAP	1206.977089	0.00890504	0.273904813	0.032511442	0.974064191	0.997476527
LONP2	1432.683513	-0.005402909	0.166266285	-0.032495516	0.974076892	0.997476527
TTLL5	704.7454815	-0.004389257	0.135313464	-0.032437694	0.974123003	0.997476527
RAET1G	5.329809469	0.029633302	0.914604524	0.032400126	0.974152962	0.997476527
SNORD114-19	5.78977322	0.033504708	1.036687595	0.032319002	0.974217656	0.997476527
PTPRZ1	9.06704763	0.024625587	0.762815307	0.032282502	0.974246763	0.997476527
MUS81	446.9807898	0.008123424	0.252531335	0.032167983	0.974338088	0.997476527
SH3D19	611.2192374	-0.004985409	0.155007777	-0.032162313	0.974342611	0.997476527
CDC45	361.5856593	-0.009858597	0.306807798	-0.032132812	0.974366137	0.997476527
FAAHP1	5.062263236	-0.028305595	0.88106352	-0.032126622	0.974371073	0.997476527
KRAS	1458.44945	0.004407057	0.137380909	0.032079109	0.974408964	0.997476527
SNX14	1038.707326	-0.003844376	0.119956431	-0.032048105	0.974433689	0.997476527
CHD7	1705.474939	0.006457422	0.202130528	0.031946792	0.974514483	0.997476527
ELOA-AS1	26.33728956	0.01222295	0.383040968	0.031910293	0.97454359	0.997476527
COLEC11	9.964722114	0.023291647	0.731236916	0.031852395	0.974589762	0.997476527
RGS5	62.7981284	0.0108847	0.34228705	0.031799917	0.974631613	0.997476527
TULP1	30.36062372	-0.015265353	0.480291965	-0.031783486	0.974644716	0.997476527
STXBP2	42.6117092	-0.011143436	0.351977404	-0.031659522	0.974743575	0.997476527
FNDC10	30.98205429	-0.012910356	0.408361509	-0.031615018	0.974779067	0.997476527
ATP6V1C2	68.0994775	0.011538601	0.366651743	0.031470192	0.974894564	0.997476527
SASS6	287.043106	-0.005260714	0.16773074	-0.031364045	0.974979215	0.997476527
MELTF	3791.134087	0.004618652	0.147437577	0.031326153	0.975009433	0.997476527
FOXC1	287.6941522	0.006045234	0.193465199	0.03124714	0.975072446	0.997476527
C9orf43	12.41899528	-0.018507665	0.592532895	-0.031234832	0.975082262	0.997476527
DHRS13	185.1647445	0.004986668	0.16018968	0.031129773	0.975166046	0.997476527
SNORD92	4.517715658	0.032310209	1.039618321	0.031078915	0.975206605	0.997476527
PIN4P1	36.09745828	0.023712148	0.768073445	0.030872241	0.975371428	0.997476527
TENT5B	4.725425241	-0.03656647	1.187811617	-0.030784738	0.975441212	0.997476527
KCND1	40.42514966	-0.014430218	0.468775799	-0.03078277	0.975442781	0.997476527
ZNF862	78.16825068	0.013593009	0.443308197	0.030662662	0.975538569	0.997476527
ZNRD1ASP	127.5834406	0.012055156	0.394068668	0.030591512	0.975595312	0.997476527
PHYHIPL	8.495575325	0.027263108	0.893529501	0.030511705	0.975658959	0.997476527
FBXO7	1051.607876	-0.005053262	0.16580001	-0.030478058	0.975685792	0.997476527
PARTICL	30.83803933	-0.010386537	0.344570778	-0.030143405	0.975952684	0.997476527
C7orf57	5.611246497	-0.034131105	1.136399074	-0.030034436	0.97603959	0.997476527
ZNF577	184.6216596	-0.006924369	0.230654432	-0.030020531	0.976050679	0.997476527
XPO7	3715.397687	0.003071611	0.102718286	0.029903256	0.976144209	0.997476527
SNORD53B	13.18167659	0.017059215	0.570545946	0.02989981	0.976146958	0.997476527
GPATCH3	172.9979362	0.005175075	0.17333684	0.0298556	0.976182216	0.997476527
CFI	129.2805106	-0.010048379	0.336804125	-0.029834489	0.976199052	0.997476527
CNKSR2	11.47554807	0.023760995	0.798080548	0.029772677	0.976248349	0.997476527
ZBTB33	955.0023616	0.004163153	0.140016606	0.029733282	0.976279769	0.997476527
RUFY1	597.1964481	0.004205113	0.142020471	0.029609203	0.976378726	0.997476527
ROBO1	1381.149728	-0.004118835	0.139432983	-0.029539893	0.976434003	0.997476527
LOC100507599	38.16773564	0.009589599	0.325211571	0.029487263	0.976475977	0.997476527
ARNTL	158.2169495	0.005679345	0.19279823	0.029457456	0.976499749	0.997476527
TSPO2	2.513728626	-0.043387651	1.493109191	-0.029058592	0.97681786	0.997476527
HSP90B2P	13.96458313	-0.020722916	0.715170169	-0.028976203	0.97688357	0.997476527
CBX6	2711.476784	-0.003039749	0.10509504	-0.028923807	0.976925359	0.997476527
LOC107984236	2.709662214	-0.034341203	1.18930233	-0.028875083	0.976964219	0.997476527
PIGB	207.649817	0.005596967	0.193866536	0.028870205	0.976968109	0.997476527
ERAL1	1060.063914	-0.003234831	0.112154026	-0.028842755	0.976990002	0.997476527
SEMA3C	34.04087116	0.011438246	0.397396622	0.028782947	0.977037702	0.997476527
NR6A1	128.4437529	0.006030082	0.209627506	0.028765698	0.977051459	0.997476527
TMEM272	7.357569279	-0.019315231	0.671826908	-0.028750309	0.977063732	0.997476527
ZNF549	189.6932692	-0.004853064	0.169026696	-0.028711818	0.97709443	0.997476527
MARCHF8	329.9390855	0.005776676	0.20129378	0.028697736	0.977105662	0.997476527
TADA1	304.8512882	0.005150721	0.179546649	0.02868737	0.977113929	0.997476527
WDR3	1402.233011	0.003306269	0.115392936	0.028652267	0.977141926	0.997476527
RP9	115.3927064	0.005752979	0.201013385	0.028619879	0.977167758	0.997476527
TICAM2	23.48926354	-0.011980135	0.42263533	-0.02834627	0.977385977	0.997476527
PEX13	514.6833984	0.00439074	0.154924267	0.028341205	0.977390017	0.997476527
METTL14	332.6178332	-0.004024153	0.14223579	-0.028292128	0.977429159	0.997476527
ADAMTS2	179.948817	-0.008441793	0.298526598	-0.028278192	0.977440274	0.997476527
SPTLC1	1352.436596	0.004340385	0.154656309	0.028064715	0.977610536	0.997476527
MIR628	3.459977603	0.037727199	1.34567412	0.02803591	0.97763351	0.997476527
EIF2AK4	2681.577841	0.00332182	0.118889975	0.027940284	0.977709779	0.997476527
MPC2	168.1995374	-0.006497841	0.232800932	-0.027911577	0.977732675	0.997476527
STS	221.9052612	-0.004148847	0.148965325	-0.02785109	0.977780917	0.997476527
UQCRB	534.9967299	0.004314848	0.15533127	0.02777836	0.977838926	0.997476527
MIR4639	2.095875364	-0.040116819	1.446781584	-0.027728318	0.977878838	0.997476527
IRF9	217.7749305	0.00630228	0.227651103	0.027683943	0.97791423	0.997476527
GUSBP2	46.75951628	0.008813924	0.320039555	0.02754011	0.978028949	0.997476527
LRRC23	71.56983028	-0.008647713	0.315735067	-0.027389144	0.978149357	0.997476527
SLC9A3R1	86.29358127	0.008509852	0.31097894	0.027364722	0.978168836	0.997476527
ZNF280B	143.2028983	-0.005448595	0.199240212	-0.027346865	0.978183078	0.997476527
MRGPRF	45.69859614	0.012485838	0.456931001	0.027325433	0.978200172	0.997476527
CRIP1	34.19861397	-0.009721512	0.357673045	-0.027179885	0.978316259	0.997476527

TMEM204	22.65110682	-0.015466798	0.570488053	-0.02711152	0.978370787	0.997476527
TBC1D1	845.1042225	0.007179868	0.265140804	0.027079455	0.978396361	0.997476527
NOMO3	304.1263751	-0.004074653	0.152155789	-0.026779479	0.978635621	0.997476527
HGFAC	1.95387522	0.043189941	1.616788414	0.026713416	0.978688313	0.997476527
TNIP1	521.5623291	-0.003899296	0.146026012	-0.026702749	0.97869682	0.997476527
TPD52L1	206.8949263	0.00538362	0.202147318	0.026632161	0.978753122	0.997476527
LSM2	541.9051715	-0.006438965	0.241912257	-0.026616942	0.97876526	0.997476527
GP1BA	67.10476654	-0.009748236	0.366444891	-0.026602188	0.978777028	0.997476527
SPCS1	326.9138011	0.00582733	0.219153469	0.026590177	0.978786608	0.997476527
ZNF212	148.8400734	-0.005253621	0.197812237	-0.026558623	0.978811775	0.997476527
TCHH	2.998866922	0.038876171	1.463886357	0.026556823	0.978813212	0.997476527
H3C6	12.94579766	-0.014490886	0.545866135	-0.026546592	0.978821371	0.997476527
MIR4504	2.072452163	0.038265512	1.448078439	0.026425027	0.978918333	0.997476527
ASB1	491.1234975	-0.003786069	0.14329689	-0.026421153	0.978921422	0.997476527
SMIM29	107.882124	-0.005313449	0.201130772	-0.026417884	0.97892403	0.997476527
PTRH2	351.0412819	-0.00406967	0.155194242	-0.026223076	0.97907941	0.997476527
DNAJB13	3.433510845	-0.033206069	1.266833512	-0.026211865	0.979088352	0.997476527
NDUFC1	97.592519	0.006433432	0.245962832	0.026156114	0.97913282	0.997476527
CFDP1	180.2173596	0.004527743	0.173366778	0.026116557	0.979164371	0.997476527
TMCC3	21.41292803	-0.012500197	0.482280446	-0.025918938	0.979321995	0.997476527
HSD3B7	20.97432216	0.014699292	0.56732064	0.025910024	0.979329104	0.997476527
ZNF610	6.871364627	-0.023047462	0.893757099	-0.025787165	0.979427099	0.997476527
LOC101928697	2.819181771	-0.030489368	1.1867712	-0.025691024	0.979503783	0.997476527
SLC2A11	80.88603884	0.0066458	0.258872539	0.025672094	0.979518882	0.997476527
RIOK2	275.209884	-0.00343531	0.133934057	-0.025649266	0.97953709	0.997476527
NKX3-1	113.5778743	0.009720186	0.378991819	0.025647481	0.979538514	0.997476527
CDHR3	15.76823062	-0.013029468	0.513157827	-0.025390761	0.979743281	0.997476527
LOC101927478	2.943390996	0.030360253	1.200019226	0.025299805	0.979815829	0.997476527
PECR	109.4815168	-0.004840463	0.192144847	-0.025191742	0.979902024	0.997476527
LMAN2L	741.8299085	-0.004623717	0.183744314	-0.025163863	0.979924261	0.997476527
BEST4	6.990121819	-0.020823255	0.829346654	-0.025108023	0.979968801	0.997476527
TBC1D16	2414.665906	-0.003748861	0.149688088	-0.025044486	0.98001948	0.997476527
LOC105376360	98.62715336	0.006336749	0.253071427	0.02503937	0.980023561	0.997476527
HTR6	2.017384313	0.034324669	1.373546485	0.024989813	0.980063089	0.997476527
EN2	3.77999952	0.025086128	1.006119727	0.024933541	0.980107973	0.997476527
TBC1D3L	3.635602327	0.022468212	0.903798079	0.02485977	0.980166816	0.997476527
SESN1	241.1829105	0.004656662	0.187380981	0.024851303	0.980173569	0.997476527
POGLUT2	545.1387427	-0.003598883	0.145021398	-0.02481622	0.980201553	0.997476527
SNAP91	8.434600754	-0.023846132	0.960909571	-0.024816209	0.980201563	0.997476527
LOC101928307	8.844291599	-0.01576136	0.635910602	-0.024785497	0.980226059	0.997476527
LINC01589	11.42560286	-0.016631857	0.672589779	-0.024728085	0.980271853	0.997476527
WDR73	450.0768104	-0.007419798	0.301035104	-0.024647616	0.980336039	0.997476527
MTMR6	601.7495362	0.003336744	0.136250263	0.024489818	0.980461905	0.997476527
PCSK4	7.233579256	0.017537348	0.716563715	0.024474234	0.980474336	0.997476527
LINC00470	69.55532473	0.013458704	0.552800033	0.024346424	0.980576283	0.997476527
MAMLD1	47.15218779	0.013709775	0.563450967	0.024331797	0.98058795	0.997476527
BACE1-AS	24.57125805	-0.012451185	0.513646197	-0.024240782	0.980660548	0.997476527
MIR616	7.816986824	0.017368549	0.720311272	0.02411256	0.980762825	0.997476527
OGFOD2	469.4409988	-0.004538363	0.188358955	-0.024094226	0.980777449	0.997476527
AR	160.8513784	-0.009424854	0.392851879	-0.023990858	0.980859901	0.997476527
PLPPR2	12.8141092	-0.013816715	0.576209881	-0.023978616	0.980869666	0.997476527
NDST1	2360.239221	-0.00260025	0.108495162	-0.023966508	0.980879323	0.997476527
RABL3	343.5120112	0.003314925	0.138788103	0.023884789	0.980944508	0.997476527
SLF2	457.0279579	0.005904036	0.248391932	0.023769031	0.981036843	0.997476527
CCNY	1341.34609	0.005142601	0.217352676	0.023660168	0.981123678	0.997476527
SYBU	308.7978341	0.005735101	0.242823136	0.02361843	0.981156971	0.997476527
CLK2	751.765688	0.005252217	0.22291471	0.023561552	0.98120234	0.997476527
DGCR2	1743.409155	-0.003201126	0.135981403	-0.023540914	0.981218803	0.997476527
CIDEA	3.974973316	0.035933652	1.527736691	0.023520841	0.981234814	0.997476527
CEP164	576.2645559	0.002357186	0.10048512	0.023458063	0.981284891	0.997476527
TNFAIP3	40.0749692	-0.009159698	0.390673995	-0.023445886	0.981294604	0.997476527
RPL17-C18orf32	5.491377037	0.018940808	0.809695376	0.023392512	0.981337178	0.997476527
CADM4	9.372113014	-0.015030327	0.64342211	-0.023359979	0.981363128	0.997476527
POLR1A	3384.839862	0.003427907	0.147003916	0.023318472	0.981396237	0.997476527
SNTA1	361.473781	0.004192358	0.179853677	0.023309826	0.981403134	0.997476527
EHD3	424.0528035	0.006225994	0.268728785	0.023168316	0.981516012	0.997476527
CASP2	422.0362046	0.004915682	0.212281766	0.023156402	0.981525516	0.997476527
RXFP2	7.865174507	0.014646839	0.635228657	0.023057586	0.981604339	0.997476527
FAM76A	277.9532267	-0.003624764	0.157287019	-0.02304554	0.981613947	0.997476527
SLC30A10	4.630931193	-0.025798134	1.124298196	-0.022945989	0.981693356	0.997476527
MED25	353.5835091	-0.00295422	0.128836888	-0.022929922	0.981706173	0.997476527
SNORD114-12	5.890689506	-0.020698806	0.90399518	-0.022897031	0.981732409	0.997476527
NRGN	17.57039833	0.01158136	0.509547447	0.022728717	0.981866669	0.997476527
PIP4K2C	429.2741009	-0.003313094	0.145856368	-0.022714774	0.981877791	0.997476527
LOC105274304	9.593385739	0.014399432	0.635861251	0.022645557	0.981933004	0.997476527
LINC00587	2.694712833	0.025485156	1.128154332	0.022590133	0.981977215	0.997476527
LMCD1	26.29519865	0.010174569	0.453742919	0.022423642	0.982110022	0.997476527
MTHFSD	226.8989191	0.003253003	0.145518117	0.022354625	0.982165075	0.997476527
FAM120C	443.1215145	0.003209917	0.144482859	0.022216596	0.98227518	0.997476527
DNAJC3	1266.333732	0.002676161	0.121128784	0.022093516	0.982373359	0.997476527
CCDC92B	6.039422984	0.017862767	0.81455495	0.02192948	0.982504209	0.997476527
TRIM56	407.2505634	0.003747288	0.170930696	0.021922853	0.982509495	0.997476527
ZNF620	29.65087331	-0.007317128	0.334536645	-0.021872425	0.982549721	0.997476527
STIM2-AS1	4.322731706	0.020159966	0.922274191	0.021858972	0.982560452	0.997476527
PIK3CA	2769.580316	0.002824515	0.129269245	0.021849863	0.982567719	0.997476527
MEAF6	541.8756001	-0.003061124	0.140758308	-0.02174738	0.982649469	0.997476527
TMEM169	36.31677068	-0.010196959	0.469644024	-0.021712103	0.982677609	0.997476527
MIR8087	2.235713308	-0.031182195	1.439536118	-0.02166128	0.982718151	0.997476527
LINC02143	7.44198456	0.035428371	1.638204004	0.021626348	0.982746015	0.997476527
LINC00173	23.99674807	0.010998217	0.509573725	0.021583171	0.982780458	0.997476527

CD83	49.56213057	0.007810228	0.363103463	0.021509648	0.982839107	0.997476527
KIF9	27.12298155	-0.009308118	0.432885369	-0.0215025	0.982844809	0.997476527
ERCC1	691.7952546	0.004887096	0.227339887	0.02149687	0.9828493	0.997476527
PXDN	1362.935309	-0.012105925	0.564145098	-0.021458886	0.9828796	0.997476527
MARK3	1499.443277	-0.002610396	0.122061323	-0.021385937	0.982937792	0.997476527
RPS2P32	3.484527728	0.024959747	1.167280852	0.021382812	0.982940284	0.997476527
ARHGDIA	3546.558993	0.003187465	0.149915997	0.021261673	0.983036918	0.997476527
SERINC2	148.5729883	-0.006921949	0.325569663	-0.02126104	0.983037423	0.997476527
ST20-AS1	58.70201665	-0.006181298	0.291047314	-0.02123812	0.983055706	0.997476527
RDH16	3.952839747	0.023285858	1.101760754	0.021135131	0.98313786	0.997476527
IDH2	1037.034839	0.004267755	0.202112727	0.021115717	0.983153348	0.997476527
PKD1P4-NPIPA8	59.80973285	0.009898972	0.4701122	0.021056617	0.983200492	0.997476527
LOC154761	4.638217965	-0.020240382	0.962131262	-0.021037027	0.983216119	0.997476527
DHODH	233.9557743	-0.004241256	0.201867184	-0.021010132	0.983237573	0.997476527
CTSH	27.94280293	0.009485404	0.453051456	0.020936703	0.983296148	0.997476527
AHSA1	1166.577268	-0.002786223	0.133406132	-0.020885267	0.983337179	0.997476527
CDCA8	612.7841773	-0.002323144	0.11134183	-0.020864971	0.98335337	0.997476527
CT45A10	4.637259721	0.018637523	0.896245387	0.020795112	0.983409097	0.997476527
MED28	1019.69001	-0.002223909	0.107339306	-0.020718493	0.983470217	0.997476527
H6PD	2368.748832	-0.006722485	0.324485091	-0.020717392	0.983471095	0.997476527
FMO4	35.20478921	-0.007460164	0.360718235	-0.020681416	0.983499794	0.997476527
DOCK3	12.19236135	-0.012880112	0.624262873	-0.020632514	0.983538804	0.997476527
NUTM2E	6.56448922	0.018397853	0.894761861	0.020561731	0.983595268	0.997476527
TPM3P9	101.1383164	-0.008917726	0.436969662	-0.020408113	0.983717812	0.997476527
LINC00886	10.88899594	-0.011654728	0.571724261	-0.020385226	0.983736069	0.997476527
TTC9C	99.3002812	0.005112602	0.251784635	0.020305456	0.983799703	0.997476527
GFI1	41.72426208	-0.009017988	0.444179219	-0.020302588	0.983801991	0.997476527
TOMM20L	5.078965736	0.024053193	1.189176046	0.020226773	0.983862471	0.997476527
USP6NL	244.5524629	0.003612803	0.178748972	0.0202116	0.983874574	0.997476527
PRELP	42.04459817	-0.013040709	0.656548081	-0.019862535	0.984153032	0.997524824
COQ9	337.7654324	0.003132433	0.157933034	0.019833935	0.984175847	0.997524824
WDR49	15.02208397	0.012471911	0.631985131	0.019734501	0.984255168	0.997524824
ZNF324	480.3517308	-0.004475128	0.226825846	-0.019729355	0.984259273	0.997524824
TNFAIP1	769.8165225	-0.004080442	0.20760208	-0.019655111	0.9843185	0.997524824
C1orf50	135.914748	0.004054499	0.208575194	0.01943903	0.984490875	0.997524824
DRD4	24.36376874	-0.009970846	0.513865948	-0.019403594	0.984519143	0.997524824
POLR2B	1988.767451	0.002337073	0.120704365	0.019361961	0.984552356	0.997524824
TTC12	93.61708781	-0.004790655	0.247559021	-0.019351569	0.984560646	0.997524824
ERCC5	74.26250476	-0.005991149	0.310268808	-0.019309542	0.984594172	0.997524824
DAZAP1	1903.039563	0.002086458	0.108100742	0.019301055	0.984600942	0.997524824
LMLN	475.2675072	-0.002587602	0.134151047	-0.019288721	0.984610782	0.997524824
MYL12A	1247.5029	0.002973918	0.155318092	0.019147276	0.984723618	0.997524824
CDKAL1	718.5071842	0.002168948	0.113509714	0.019108039	0.984754919	0.997524824
PRC1	2065.687512	-0.002265793	0.119358889	-0.018983025	0.984854647	0.997524824
FARSA-AS1	10.42533734	0.014148966	0.745928986	0.018968248	0.984866435	0.997524824
DQX1	2.289184595	-0.025693437	1.355212632	-0.018958971	0.984873836	0.997524824
SSTR1	17.22192582	-0.010327509	0.548183756	-0.018839502	0.984969141	0.997547177
EPB41L2	1384.079386	0.002321947	0.124195725	0.018695873	0.985083721	0.997547177
TTC41P	11.87243403	0.011961076	0.642333401	0.01862129	0.985143219	0.997547177
ATP6V1A	1522.060569	-0.002535604	0.137512479	-0.018439084	0.985288573	0.997547177
FUOM	174.7051546	0.003500111	0.189901232	0.018431218	0.985294848	0.997547177
NELFB	1718.852357	0.002613222	0.142132391	0.01838583	0.985331056	0.997547177
FBXW8	1070.135153	0.002715847	0.147788574	0.018376567	0.985338446	0.997547177
SFT2D2	2688.430158	-0.00190165	0.103632164	-0.018349997	0.985359642	0.997547177
FKSG29	2.898627952	0.021983468	1.203221659	0.018270506	0.985423056	0.997547177
RAPGEF3	21.63299176	-0.010008574	0.549028992	-0.018229591	0.985455696	0.997547177
BCS1L	569.185879	0.002768304	0.154529179	0.017914444	0.985707106	0.997744995
LINC02444	5.271538789	0.015329155	0.865894202	0.017703265	0.985875576	0.997781064
WDSUB1	95.41334486	-0.003607104	0.204012365	-0.017680811	0.985893489	0.997781064
UCP2	16.50486629	-0.008465056	0.481177023	-0.017592395	0.985964024	0.997781064
MTARC2	145.609237	0.003234961	0.183919241	0.017589028	0.985966709	0.997781064
CNOT4	387.4801077	-0.002068757	0.118438706	-0.017466901	0.986064138	0.997822995
GGCT	263.0483821	-0.002247139	0.130918078	-0.01716447	0.986305407	0.997916102
EXO5	190.8825443	-0.003897424	0.227440374	-0.017136027	0.986328097	0.997916102
MIA2	464.0599416	-0.00232355	0.136318963	-0.017044951	0.986400755	0.997916102
ZHX3	884.0750486	0.002336474	0.137323096	0.017014429	0.986425104	0.997916102
MIR563	3.613771159	-0.020109304	1.187716248	-0.016931067	0.986491608	0.997916102
ANO6	1542.348986	-0.00403554	0.238360676	-0.016930393	0.986492146	0.997916102
ZBTB20-AS4	3.369999289	0.027276863	1.624193886	0.016794093	0.986600882	0.997969445
TRAM1L1	62.10608159	-0.004780135	0.287877078	-0.016604779	0.986751912	0.997970182
CFAP161	9.615800389	-0.014693002	0.889804651	-0.016512615	0.986825438	0.997970182
ALDOC	304.6236168	-0.005477832	0.332526441	-0.016473372	0.986856746	0.997970182
MGA	1778.270282	-0.001649696	0.101141408	-0.016310784	0.986986454	0.997970182
RAB39A	31.22094088	-0.007225755	0.444444815	-0.016257936	0.987028615	0.997970182
TDRKH	141.9965455	0.004669801	0.287401645	0.016248345	0.987036267	0.997970182
LINC01134	3.543088138	-0.016946168	1.045885596	-0.016202698	0.987072683	0.997970182
OCEL1	84.9545587	-0.006082114	0.375581224	-0.01619387	0.987079726	0.997970182
RPAP3	279.3356134	-0.002791256	0.172736595	-0.016159033	0.987107518	0.997970182
ZNF415	3.593510684	-0.019624208	1.221411248	-0.016066831	0.987181075	0.997970182
MZT2A	196.1291665	0.003915442	0.245984667	0.015917425	0.987300269	0.997970182
WTIP	67.97160179	0.004898295	0.308124088	0.015897151	0.987316443	0.997970182
ZNF66	35.69105058	0.008108885	0.510615779	0.0158806	0.987329647	0.997970182
FLJ45513	11.470291	0.014488207	0.922152951	0.015711284	0.987464725	0.998050105
ZDHHC16	502.9946692	0.002175916	0.139513433	0.01559646	0.98755633	0.998065971
MRRF	658.339559	0.002827147	0.181796103	0.015551199	0.987592439	0.998065971
POLR2J2	3.978299666	-0.01506181	0.977163162	-0.015413813	0.987702044	0.998104023
SLC9A8	276.2049976	0.003598059	0.234840163	0.015321312	0.98777584	0.998104023
KIFAP3	5928.635781	0.003848118	0.251619832	0.015293382	0.987798122	0.998104023
IREB2	2503.086395	0.001508364	0.099240074	0.015199141	0.987873307	0.998123396
CAMTA2	1065.356242	-0.002475446	0.165501341	-0.014957254	0.988066283	0.998203727

STRN	1354.859554	-0.001882933	0.126763336	-0.014853923	0.98814872	0.998203727
SUCLA2	591.7466918	-0.002142003	0.144753649	-0.014797575	0.988193674	0.998203727
LINC00324	19.971413	0.00627163	0.429311393	0.014608581	0.988344453	0.998203727
SNX29P1	20.16814056	0.007323789	0.506060886	0.01447215	0.988453298	0.998203727
KIAA1586	462.2150002	0.001849186	0.128504298	0.014390073	0.98851878	0.998203727
LOC101929268	9.598547421	-0.010946209	0.762881228	-0.014348511	0.988551938	0.998203727
HAPLN4	3.184701497	0.022807431	1.591309232	0.014332495	0.988564715	0.998203727
VEGFC	214.6577545	-0.003534748	0.248449176	-0.014227249	0.98864868	0.998203727
MMP21	3.789484053	-0.015449827	1.095176528	-0.014107157	0.988744491	0.998203727
MAP7D2	19.96026946	-0.006433957	0.456251856	-0.014101766	0.988748792	0.998203727
CLDN4	9.565763785	-0.013764827	0.980943598	-0.014032231	0.988804267	0.998203727
ANXA4	395.2836618	0.003374142	0.241168134	0.013990827	0.988837299	0.998203727
DIRAS1	6.342782384	0.01138332	0.813706098	0.013989473	0.988838379	0.998203727
RNF122	258.2724808	0.002292129	0.163938647	0.013981625	0.988844641	0.998203727
KRT8	7.463513639	-0.010448091	0.747833792	-0.01397114	0.988853006	0.998203727
HEATR1	3855.066828	0.002152984	0.154835304	0.013904994	0.988905777	0.998203727
NR2C1	733.4221924	0.001914554	0.138378271	0.013835651	0.988961099	0.998203727
LINC01687	18.62703494	0.006519825	0.475443582	0.013713141	0.98905884	0.998245838
COLCA2	10.34880926	0.009052234	0.667608816	0.013559189	0.989181664	0.998246944
FMO2	6.703441593	0.015660685	1.155111937	0.013557721	0.989182835	0.998246944
GSC	32.84879006	-0.006020691	0.45034136	-0.013369171	0.989333263	0.998246944
UBE4B	1834.633779	-0.001961268	0.146903226	-0.01335075	0.989347959	0.998246944
FABP5	470.9664177	0.003947267	0.296397423	0.01331748	0.989374502	0.998246944
TMEM8B	185.9081789	0.005001805	0.37634501	0.013290478	0.989396045	0.998246944
SDSL	10.75191589	0.007728086	0.589965923	0.013099207	0.989548644	0.998262473
TLL2	15.011869	-0.007116645	0.549101029	-0.012960538	0.989659276	0.998262473
FBXO41	494.5709999	-0.002092075	0.161589479	-0.01294685	0.989670197	0.998262473
PRELID3B	585.2938469	-0.001617487	0.125786809	-0.012858959	0.989740318	0.998262473
RGS20	34.89713923	-0.005247267	0.409509873	-0.01281353	0.989776562	0.998262473
SNORD20	28.89326997	0.007672415	0.602550189	0.012733238	0.989840621	0.998262473
CEBPD	155.7608617	-0.002687417	0.2118227	-0.012687106	0.989877426	0.998262473
COQ2	141.5084298	-0.00349011	0.276437807	-0.0126253	0.989926735	0.998262473
EXD2	702.9735337	-0.001376827	0.109436447	-0.012581062	0.98996203	0.998262473
C1QL3	6.602588252	-0.0117088	0.931559521	-0.01256903	0.989971629	0.998262473
LINC02046	1.96888611	-0.029032018	2.371201994	-0.012243587	0.990231275	0.998467794
SRXN1	723.150502	0.001607031	0.132397579	0.01213792	0.990315579	0.9984963
NOL11	1236.067499	-0.002046152	0.170391945	-0.012008504	0.99041883	0.998507749
PSMA3-AS1	349.464373	0.002876895	0.240288207	0.011972686	0.990447407	0.998507749
RNASEH2B-AS1	31.83361575	-0.00453938	0.381044507	-0.011912992	0.990495032	0.998507749
NKAIN2	5.279141447	-0.012334234	1.051323415	-0.011732102	0.990639351	0.998568688
SLC15A4	648.2914921	-0.001535004	0.131233358	-0.011696752	0.990667555	0.998568688
MIR3145	2.126242848	0.015159093	1.304958763	0.01161653	0.990731558	0.998576718
HIVEP1	543.2655244	-0.00151211	0.13266808	-0.011397691	0.990906155	0.998655055
COTL1	779.4237805	-0.001691267	0.149376362	-0.011322186	0.990966395	0.998655055
R3HDM2	813.6062759	0.002603109	0.230192758	0.011308388	0.990977404	0.998655055
SEH1L	1302.52433	0.002068202	0.185108444	0.011172922	0.991085483	0.998688241
SAMD3	12.59225077	-0.006978966	0.631096465	-0.011058477	0.991176792	0.998688241
PIGW	329.7873526	-0.003340444	0.302770602	-0.011032921	0.991197182	0.998688241
NTAN1	248.1843491	-0.001900252	0.173698178	-0.010939966	0.991271344	0.998688241
DDX56	753.1150825	-0.002065595	0.189228234	-0.010915893	0.99129055	0.998688241
LOC101927932	2.548105243	0.012699093	1.179382935	0.010767574	0.991408885	0.998750994
TRIM7	235.6630627	0.001997366	0.186823933	0.010691167	0.991469845	0.998755944
ZFP91	1720.646293	0.001390248	0.132018232	0.010530729	0.991597849	0.998828425
EBF3	266.7626709	-0.002085563	0.199950385	-0.010430401	0.991677895	0.998829768
TRPM4	102.1533025	0.002818651	0.273145908	0.010319212	0.991766606	0.998829768
LINC01140	26.81825127	-0.003638772	0.352933132	-0.01031009	0.991773884	0.998829768
UCK2	1581.518552	-0.001154989	0.114106598	-0.010122013	0.99192394	0.998829768
CERNA3	8.137670834	0.00681143	0.673171137	0.010118423	0.991926804	0.998829768
LHFPL2	841.5724576	-0.001860483	0.184163104	-0.010102367	0.991939614	0.998829768
LINC00910	68.83227649	-0.003031215	0.301995509	-0.010037286	0.991991539	0.998829768
POLR2J3	9.105884542	0.006751445	0.680218984	0.009925399	0.992080807	0.998863212
AKR1C2	62.11164911	0.006367481	0.652379448	0.009760395	0.992212455	0.99887487
EXTL2	1183.900733	0.001266196	0.129959099	0.009743033	0.992226307	0.99887487
SNORD38B	15.06689597	0.006161887	0.635238332	0.009700118	0.992260547	0.99887487
YJEFN3	37.97857157	-0.003955889	0.414114719	-0.009552641	0.992378211	0.998911841
HOXA6	29.13681874	0.003853138	0.405015016	0.009513569	0.992409385	0.998911841
EFCAB7	121.2511047	-0.001927665	0.204661947	-0.009418775	0.992485016	0.998931544
C20orf27	1042.786166	0.001165118	0.12470731	0.00934282	0.992545617	0.998936117
LETM1	1759.601181	-0.000972122	0.107290459	-0.009060656	0.992770741	0.998945097
NRCAM	12.57711113	-0.005683584	0.629489667	-0.009028876	0.992796097	0.998945097
MATN3	197.7384513	0.00184109	0.204514764	0.009002237	0.992817351	0.998945097
GNB1L	56.00834203	0.002186567	0.244166827	0.008955218	0.992854866	0.998945097
ATP6V0A2	690.5534841	-0.001234371	0.141096723	-0.008748404	0.993019872	0.998945097
RNF216P1	296.3905787	0.001802019	0.20661587	0.008721591	0.993041265	0.998945097
CANT1	1426.67258	-0.000988082	0.113536126	-0.008702796	0.993056261	0.998945097
PEX10	318.5081879	-0.001302002	0.150263098	-0.008664813	0.993086566	0.998945097
SLC9A7	359.2285098	0.001865996	0.215461038	0.008660481	0.993090022	0.998945097
ENTPD3	7.904280143	0.007330793	0.849549934	0.008629031	0.993115115	0.998945097
EPS8L2	53.99350992	-0.003426794	0.403276049	-0.008497391	0.993220145	0.998988224
KNL1	1745.633511	0.000958866	0.115111096	0.008329917	0.993353764	0.998988224
SUMO1P3	30.04605499	-0.004777905	0.576805537	-0.00828339	0.993390887	0.998988224
FBXO38	651.2041149	0.001119678	0.135313591	0.008274692	0.993397826	0.998988224
ARL17A	106.7759546	0.003365591	0.411106356	0.008186667	0.993468058	0.998988224
ZNF765	360.2261355	0.001530477	0.191557481	0.007989651	0.993625249	0.998988224
EVC	623.2459121	0.00147984	0.185252992	0.007988213	0.993626396	0.998988224
CDIPT	645.5331197	-0.001552918	0.198722377	-0.007814508	0.993764988	0.998988224
FRY	12.76064992	0.004583403	0.606396439	0.007558426	0.993969306	0.998988224
TMSB15B	48.90671661	0.002506975	0.332502449	0.007539719	0.993984232	0.998988224
SNORD102	16.70078543	0.004073756	0.545887483	0.00746263	0.994045738	0.998988224
NCK2	408.330153	0.001759577	0.236562298	0.007438114	0.994065299	0.998988224

C1orf162	5.155059552	-0.007374932	0.997934558	-0.007390196	0.994103531	0.998988224
RAP1GDS1	843.1498347	-0.001309863	0.17824555	-0.007348645	0.994136682	0.998988224
CHURC1-FNTB	3.638457628	0.007781692	1.059107069	0.007347408	0.994137669	0.998988224
MRPL20-AS1	151.7472506	-0.001259852	0.172710607	-0.007294585	0.994179815	0.998988224
FLJ46906	160.3578184	0.001936714	0.266363523	0.007270941	0.994198679	0.998988224
MIR640	2.533432206	-0.010695236	1.471261453	-0.007269433	0.994199883	0.998988224
THOC1	386.2298186	-0.001852188	0.256959989	-0.007208078	0.994248836	0.998988224
SLA2	6.217956339	-0.007253777	1.01862605	-0.007121138	0.994318202	0.998988224
EMC7	923.9757679	0.000736541	0.103741493	0.007099773	0.994335248	0.998988224
MIR10396B	913.491179	0.002523643	0.373986532	0.006747951	0.994615955	0.9990744
BLZF1	424.5543578	-0.000770851	0.115372798	-0.006681389	0.994669063	0.9990744
CHD2	1665.030013	-0.001143736	0.172171456	-0.006643003	0.994699689	0.9990744
PLEKHA8P1	22.02377888	-0.003072268	0.46274275	-0.006639257	0.994702678	0.9990744
AMH	63.31003099	0.002083002	0.333234742	0.006250855	0.995012572	0.9990744
MIR4467	4.782280026	0.007169547	1.157690957	0.006192972	0.995058755	0.9990744
EIF2AK1	2114.461817	-0.001264771	0.204398675	-0.006187765	0.995062909	0.9990744
PDE4A	360.8842846	0.001247033	0.203129648	0.006139097	0.99510174	0.9990744
PIK3IP1	153.1214296	0.00218435	0.363901558	0.006002584	0.995210659	0.9990744
LOC101928402	2.872245352	0.007725332	1.287440478	0.006000535	0.995212294	0.9990744
GRK6	755.0479257	0.000861402	0.143941636	0.005984385	0.99522518	0.9990744
VDAC1	4035.703884	-0.000719746	0.120296113	-0.005983116	0.995226192	0.9990744
WNT4	18.07782426	0.005479662	0.921095681	0.00594907	0.995253357	0.9990744
RIMS1	32.3374218	0.002725595	0.45844797	0.005945266	0.995256392	0.9990744
PABPC1L	423.6476633	-0.002413818	0.414126265	-0.005828701	0.995349396	0.9990744
TRAPPC12	863.6372349	-0.000707141	0.122813543	-0.00575784	0.995405934	0.9990744
LOC101928834	3.145996245	-0.007441051	1.292850884	-0.005755537	0.995407771	0.9990744
RCAN2	286.867431	0.004388878	0.775164231	0.005661869	0.995482506	0.9990744
POU6F2	1.903082046	-0.009694382	1.7136444	-0.005657172	0.995486254	0.9990744
ZNF782	80.59526124	0.001937871	0.355685972	0.005448264	0.995652936	0.99907832
LAPTM4B	8402.951402	0.000628532	0.115741649	0.005430471	0.995667132	0.99907832
NCOR1P1	3.44519958	0.005786944	1.079682858	0.005359855	0.995723475	0.99907832
LRRC56	7.945011003	-0.004078629	0.765791539	-0.00532603	0.995750463	0.99907832
TMEM184C	379.1604464	0.000758386	0.143066376	0.005300936	0.995770485	0.99907832
CPNE7	10.55875476	0.003064818	0.597312539	0.005131013	0.995906062	0.999158092
LPAR3	374.8592953	-0.000649912	0.13185532	-0.004928977	0.996067261	0.999187011
RFX1	329.8154642	-0.000842841	0.171233458	-0.004922173	0.99607269	0.999187011
NRSN2-AS1	39.02187551	0.001504436	0.330180522	0.004556404	0.996364528	0.999187011
LMO7DN	2.645880835	-0.005672316	1.255256677	-0.00451885	0.996394492	0.999187011
LRRC37B	138.9338508	0.00112546	0.256138376	0.004393952	0.996494145	0.999187011
ANKS1B	38.80041404	-0.001610947	0.367450149	-0.004384124	0.996501986	0.999187011
WDR72	107.2463767	0.001715891	0.39232684	0.004373625	0.996510363	0.999187011
NPFF	6.681713803	0.003702559	0.847520231	0.004368697	0.996514295	0.999187011
PTPRM	5.570592421	-0.004146013	0.983198973	-0.00421686	0.996635442	0.999187011
NUP98	2375.926316	0.000459272	0.112888142	0.004068382	0.99675391	0.999187011
WDR6	1801.237001	0.000373449	0.093218898	0.004006148	0.996803565	0.999187011
RNASEK-C17orf49	15.20792325	0.002119702	0.547681155	0.003870322	0.996911938	0.999187011
AZIN1-AS1	35.80709264	-0.001352006	0.349369582	-0.003869845	0.996912318	0.999187011
CMPK1	2024.581156	-0.000671448	0.176450859	-0.003805296	0.99696382	0.999187011
NPR3	12.36281829	-0.002338466	0.625246581	-0.003740069	0.997015863	0.999187011
ERMP1	399.2364008	0.000589123	0.159912706	0.003684028	0.997060577	0.999187011
CNPPD1	517.4161049	-0.000524882	0.151295613	-0.003469249	0.997231945	0.999187011
FAM66A	7.374155367	-0.002671533	0.771319886	-0.003463587	0.997236463	0.999187011
SIGLEC10	5.00595709	0.002844334	0.82914988	0.003430422	0.997262925	0.999187011
CREM	173.8109807	0.000601137	0.175759929	0.003420215	0.997271068	0.999187011
BORA	183.3711714	-0.00068321	0.203638535	-0.003355013	0.997323092	0.999187011
ORC1	288.8553914	0.000708046	0.214911528	0.003294592	0.997371301	0.999187011
OIP5	324.7169175	0.00041564	0.131695791	0.003156062	0.997481831	0.999187011
CDKN2D	49.3238993	-0.000895838	0.285619647	-0.003136472	0.997497461	0.999187011
FUNDC2	373.986354	0.00054968	0.177144421	0.003103002	0.997524166	0.999187011
LINGO2	31.28670575	-0.001113235	0.365363628	-0.003046923	0.997568911	0.999187011
HHIPL2	476.072022	0.001768637	0.582217113	0.003037762	0.99757622	0.999187011
TCP1	3728.133197	-0.000496568	0.164072914	-0.003026506	0.997585201	0.999187011
INPP5A	135.7045304	0.000538799	0.180214133	0.002989772	0.997614511	0.999187011
NFU1	143.0893381	-0.000611309	0.20468205	-0.002986626	0.99761702	0.999187011
RALBP1	912.0507475	0.00046717	0.164500309	0.002839932	0.997734065	0.999225301
THOC3	443.3801967	-0.000373259	0.133702665	-0.002791707	0.997772543	0.999225301
RRP7A	986.6205942	-0.000495715	0.184413848	-0.002688056	0.997855244	0.999225301
EBP	433.7778912	0.000401324	0.151009673	0.002657602	0.997879543	0.999225301
ISYNA1	22.92288666	0.001098059	0.439982	0.002495691	0.998008728	0.999264644
ADAM20P1	4.145205031	0.002595254	1.051647545	0.002467798	0.998030984	0.999264644
DLX1	132.123553	0.000442962	0.203726578	0.002174297	0.998265163	0.999442958
MAGEE1	44.5432925	0.000602055	0.291357419	0.002066379	0.998351269	0.999473012
FOXO6	11.94454993	0.001154591	0.595933866	0.001937449	0.99845414	0.999519846
ZNF480	242.2699297	0.000377743	0.224984377	0.001678974	0.998660373	0.999641244
MIR553	3.92962324	0.001655599	1.043321556	0.001586854	0.998733875	0.999641244
TTC9B	1.971428579	0.002558581	1.624969613	0.001574541	0.998743699	0.999641244
TUBGCP5	517.638172	-0.00026485	0.184057022	-0.001438957	0.998851879	0.999693371
RHBDL2	14.8181121	0.00072054	0.58673733	0.001228045	0.999020162	0.999727585
SNAPC5	221.7763724	0.000250394	0.207069359	0.00120923	0.999035174	0.999727585
VAX2	30.30963851	-0.000419618	0.389192247	-0.001078177	0.999139739	0.999727585
LINC00299	21.35896424	0.001050764	1.006571832	0.001043904	0.999167085	0.999727585
NDUFAF8	251.4159471	0.000232792	0.235350409	0.000989128	0.99921079	0.999727585
LOC440346	2.023789023	-0.001650606	1.816568051	-0.00090864	0.99927501	0.999727585
SCN1B	37.34678565	0.000376195	0.425213902	0.00088472	0.999294096	0.999727585
CBR3-AS1	17.72465007	0.000504963	0.605754565	0.000833611	0.999334875	0.999727585
MYL5	52.52329266	0.000201732	0.381478213	0.000528817	0.999578065	0.999825044
CCNC	786.8305568	-5.02E-05	0.144132161	-0.00034842	0.999722001	0.999825044
HHIPL1	13.28744664	-0.000238454	0.815634972	-0.000292353	0.999766736	0.999825044
DENND5A	2310.851525	-4.33E-05	0.152912546	-0.000283187	0.99977405	0.999825044
RGMA	47.28061375	9.67E-05	0.344844091	0.000280429	0.99977625	0.999825044

NBR2	97.16905805	-7.98E-05	0.313372909	-0.000254671	0.999796802	0.999825044
SNORA2A	7.398410293	-0.000207643	0.946951947	-0.000219275	0.999825044	0.999825044
NSG1	124.5407943	-1.771842118	1.263126474	-1.402743237	NA	NA

Supplementary Table 11. Differentially expressed genes comparing pazopanib 10 μM and control						
**Gene**	**baseMean**	**log2FoldChange**	**lfcSE**	**stat**	**pvalue**	**padj**
MEGF10	1370.809837	-2.035652994	0.156459897	-13.01070132	1.06E-38	1.90E-34
FGFR3	1199.154428	2.06834952	0.165749955	12.47873356	9.75E-36	6.87E-32
TP63	1564.528244	-1.879077295	0.150746336	-12.46516068	1.16E-35	6.87E-32
EMP1	3891.672358	-1.322963798	0.110375982	-11.98597536	4.21E-33	1.87E-29
FGFR2	1256.82837	1.573200489	0.133448939	11.78878226	4.46E-32	1.59E-28
DAB2	899.6037513	-1.079862892	0.095517675	-11.30537243	1.23E-29	3.67E-26
ETV5	937.0777055	-2.095348301	0.189109506	-11.08007917	1.57E-28	3.99E-25
FZD5	701.8429258	1.345967157	0.145985475	9.219870355	2.97E-20	6.63E-17
SYNE3	175.8323816	-2.346989393	0.257998369	-9.096915613	9.29E-20	1.84E-16
ENPP1	6751.73869	-1.485683338	0.166187277	-8.939813965	3.90E-19	6.95E-16
ANKRD28	1255.30046	-0.88348235	0.101077959	-8.740603399	2.32E-18	3.76E-15
MDFI	512.0464592	1.327617837	0.159188153	8.339928628	7.43E-17	1.10E-13
GGT8P	67.41487044	5.379455802	0.670562051	8.022308746	1.04E-15	1.42E-12
ETV1	508.5627055	-1.065634692	0.134049406	-7.949566682	1.87E-15	2.38E-12
CACNA1G	1212.546436	-1.043091683	0.132703922	-7.860292782	3.83E-15	4.55E-12
LINC01945	105.4452877	2.126305395	0.276217047	7.697951371	1.38E-14	1.54E-11
ACACA	7817.710493	0.922137206	0.120043141	7.681715101	1.57E-14	1.65E-11
VEGFA	2681.437446	-1.022726377	0.133692385	-7.649847631	2.01E-14	1.99E-11
WNT16	1005.059189	1.859095436	0.244647451	7.599079514	2.98E-14	2.80E-11
FLRT1	148.7061083	1.837956926	0.242582286	7.576632895	3.55E-14	3.16E-11
MXRA5	2672.739673	1.809150029	0.239448478	7.555487703	4.17E-14	3.54E-11
SCUBE1	56.75381509	4.172946555	0.559053068	7.464312056	8.37E-14	6.78E-11
FAT3	476.5635213	1.020347297	0.14141762	7.215135538	5.39E-13	4.17E-10
RNF145	2079.511635	0.941248124	0.130562386	7.209182923	5.63E-13	4.18E-10
IGFN1	67.19427082	3.102234249	0.431009864	7.197594543	6.13E-13	4.37E-10
TEAD1	3036.650945	-0.667823217	0.093060052	-7.176260981	7.16E-13	4.91E-10
ASIC3	291.3354864	1.906320709	0.267473075	7.127149935	1.02E-12	6.76E-10
OGN	678.8917891	0.92601608	0.130053804	7.120253726	1.08E-12	6.86E-10
SERPINI1	220.668581	-1.941619377	0.278624155	-6.96859674	3.20E-12	1.97E-09
TRIB3	446.0245042	-1.757768417	0.257641396	-6.822538772	8.94E-12	5.31E-09
RGS16	128.7159726	-1.651798926	0.246818014	-6.6923759	2.20E-11	1.26E-08
PDE7B	588.4737113	-1.273455307	0.191527124	-6.64895541	2.95E-11	1.64E-08
TENM2	250.0358658	-1.453324668	0.22191729	-6.548947444	5.79E-11	3.13E-08
GJB2	384.0161695	-2.469133417	0.385139414	-6.411012028	1.45E-10	7.58E-08
SH2B3	1069.582903	-1.055886232	0.165245003	-6.389822479	1.66E-10	8.46E-08
ABCA1	1661.332255	3.375720925	0.529066552	6.380522286	1.76E-10	8.74E-08
RFLNB	1071.468013	-0.75105577	0.117834192	-6.373835627	1.84E-10	8.88E-08
EVA1A	29.20459275	-2.708933685	0.428232974	-6.325840962	2.52E-10	1.18E-07
CNIH3	94.77907642	-2.116948697	0.344238272	-6.149661062	7.76E-10	3.54E-07
EMB	613.3941487	-0.712025259	0.115852118	-6.14598395	7.95E-10	3.54E-07
TBX2	704.9718108	-1.312598308	0.216170375	-6.07205454	1.26E-09	5.49E-07
FLNB	4439.144482	0.905932138	0.149349754	6.065842844	1.31E-09	5.57E-07
ERRFI1	1293.11125	-1.517682207	0.250676765	-6.054339369	1.41E-09	5.77E-07
VSNL1	442.0194419	0.845371393	0.139667866	6.052726503	1.42E-09	5.77E-07
FASN	22164.87325	0.978931329	0.161829719	6.04914433	1.46E-09	5.77E-07
TNC	4007.305899	-1.080070145	0.178691203	-6.044338663	1.50E-09	5.81E-07
SRPX	491.4454388	-0.821086371	0.137024921	-5.992241159	2.07E-09	7.75E-07
LINC01614	4926.718314	-1.102732695	0.184071294	-5.990791241	2.09E-09	7.75E-07
CHAC1	284.0626018	-1.82002122	0.304728799	-5.972593427	2.34E-09	8.39E-07
LOC101929322	52.01762939	2.970231673	0.497421048	5.971262539	2.35E-09	8.39E-07
PCDH18	3744.86059	-0.581121535	0.097483745	-5.961214742	2.50E-09	8.75E-07
TMTC2	2385.01411	0.526093752	0.088702004	5.931024423	3.01E-09	1.03E-06
TRIB2	994.0410819	-1.000390739	0.169290904	-5.909300004	3.44E-09	1.16E-06
ENDOD1	484.6659608	0.768739913	0.130592611	5.886549825	3.94E-09	1.30E-06
FAM160A1	375.6116135	1.087094855	0.184894108	5.879553787	4.11E-09	1.33E-06
GASK1B	445.8661358	-0.777403207	0.134475058	-5.781021519	7.42E-09	2.36E-06
SMILR	132.6328091	-1.768905454	0.30708339	-5.760342337	8.39E-09	2.62E-06
PTPN14	8160.046404	-0.506239043	0.088247716	-5.736568241	9.66E-09	2.97E-06
TCF4	1542.050349	-0.731456849	0.127604213	-5.732231173	9.91E-09	2.99E-06
RNF152	365.6281935	-1.314222493	0.229665283	-5.722338513	1.05E-08	3.12E-06
KLF5	170.646536	-1.30465966	0.228818405	-5.701725176	1.19E-08	3.46E-06
CH25H	897.405334	-1.638682482	0.290656087	-5.637874306	1.72E-08	4.95E-06
IL17RD	567.9393484	-0.876707168	0.155758702	-5.62862399	1.82E-08	5.14E-06
TNRC18	2917.136574	-0.526535852	0.093611598	-5.624686082	1.86E-08	5.17E-06
LGR4	2336.71897	1.215098369	0.216848886	5.603433764	2.10E-08	5.69E-06
NT5DC2	2687.026336	-0.667987776	0.119219945	-5.60298678	2.11E-08	5.69E-06
TGFBR3	334.3579822	-1.177406749	0.210614834	-5.590331538	2.27E-08	6.03E-06
LPCAT1	2431.957346	0.945245319	0.169747766	5.56852879	2.57E-08	6.73E-06
PARVG	267.8867492	-1.167180544	0.210723466	-5.538920584	3.04E-08	7.86E-06
OMD	142.7932608	1.404592704	0.254880395	5.510791464	3.57E-08	9.09E-06
PRUNE2	1367.315534	1.360916541	0.252929057	5.38062552	7.42E-08	1.86E-05
SLC1A4	854.7501036	-1.006994979	0.187366011	-5.374480537	7.68E-08	1.90E-05
HTR1F	380.407911	-0.889953565	0.166969673	-5.330031191	9.82E-08	2.40E-05
PKDCC	310.6848366	1.355243815	0.254605872	5.322908723	1.02E-07	2.46E-05
ST6GAL2	242.0788132	-1.266218535	0.23938747	-5.28941024	1.23E-07	2.92E-05
SPTLC3	131.7782332	-1.274095848	0.241704491	-5.271295721	1.35E-07	3.18E-05
PNMA2	170.5524373	-1.028854731	0.195486359	-5.263051278	1.42E-07	3.28E-05
CAV1	1868.366729	-0.746962858	0.142164818	-5.254203315	1.49E-07	3.40E-05
CSPG4	7808.818335	-0.770687044	0.147143257	-5.23766469	1.63E-07	3.67E-05
EFHD1	80.46519182	1.171392983	0.225391874	5.197139377	2.02E-07	4.51E-05
DUSP7	1262.342581	-0.896689927	0.173935903	-5.155289444	2.53E-07	5.57E-05
ARHGEF2	2100.333163	-0.732579365	0.142196171	-5.151892349	2.58E-07	5.60E-05
DDR2	6647.2306	-0.745768866	0.145874597	-5.11239709	3.18E-07	6.83E-05
LGALS3	571.1511603	-0.993364845	0.194876946	-5.097395382	3.44E-07	7.31E-05
RIN1	524.9199692	-0.625236547	0.122945778	-5.085465796	3.67E-07	7.65E-05
C2orf27A	317.6805717	0.927741193	0.182479963	5.084071572	3.69E-07	7.65E-05
ITGA4	5332.991127	0.76993747	0.151687372	5.075817835	3.86E-07	7.90E-05
HHIP	93.82828911	-1.15436107	0.22808654	-5.061066166	4.17E-07	8.44E-05

SPRED1	2034.152742	-0.832392442	0.164576921	-5.057771381	4.24E-07	8.49E-05
PITPNM2	799.8747222	0.745524388	0.147825958	5.043257604	4.58E-07	9.06E-05
CA9	101.4493269	-1.282304035	0.255469813	-5.019395532	5.18E-07	0.000101504
NXPH3	181.5424473	1.809980484	0.362085334	4.998767731	5.77E-07	0.000111758
KLHL14	77.15071549	-1.44742508	0.290521301	-4.98216508	6.29E-07	0.000119475
TXNIP	1328.404114	-1.617764046	0.324740273	-4.981716715	6.30E-07	0.000119475
FAM210B	332.9014007	-0.930436777	0.187076421	-4.973565187	6.57E-07	0.000123301
CARS1	1189.601795	-0.854708136	0.173437956	-4.928033961	8.31E-07	0.000154182
ABCB6	330.0623204	0.734955084	0.149248502	4.924371604	8.46E-07	0.000155478
ENOX1	386.832367	-0.687362595	0.139932564	-4.9120989	9.01E-07	0.000163847
OSTM1	326.3998767	0.620126991	0.12644478	4.904330513	9.37E-07	0.000168744
DGKH	2305.934337	0.780855998	0.159320991	4.901149522	9.53E-07	0.000169785
PIH1D1	394.7265245	-0.663912961	0.136101089	-4.878087069	1.07E-06	0.000188997
GCNT1	435.8747173	0.707072359	0.14513331	4.871882005	1.11E-06	0.00019312
CHURC1	576.7836266	-0.585401219	0.12094989	-4.840030999	1.30E-06	0.000223059
SLC6A10P	109.587393	1.229577764	0.254072372	4.839478434	1.30E-06	0.000223059
MAPKBP1	1110.661473	0.635382376	0.131641168	4.826623655	1.39E-06	0.000235677
CRISPLD2	389.969977	3.016380819	0.625208629	4.824598829	1.40E-06	0.000235838
BAIAP2L1	224.6896647	-0.874011116	0.181524341	-4.814842516	1.47E-06	0.000245343
IVNS1ABP	2186.069128	0.896491284	0.186629433	4.80359003	1.56E-06	0.000257146
CUBN	1226.280583	-0.871966141	0.181867405	-4.794515779	1.63E-06	0.000266594
LOC105369147	297.2162382	-0.807886399	0.168807647	-4.785840057	1.70E-06	0.000275843
KIDINS220	3212.102421	0.406278803	0.084940196	4.78311592	1.73E-06	0.000277091
ETV4	77.72926568	-1.18455774	0.247782239	-4.780640223	1.75E-06	0.00027802
PLCE1	908.554713	0.673427559	0.141165009	4.770499183	1.84E-06	0.000289804
OTULINL	107.5133133	-0.892532707	0.187168042	-4.768617002	1.85E-06	0.000289958
PCK2	1114.806254	-1.22967971	0.258261829	-4.76136839	1.92E-06	0.000297958
PDCD4	438.8731818	-0.564618505	0.119634062	-4.719546384	2.36E-06	0.000363115
KIF21B	401.505248	-0.842687691	0.178630329	-4.717495044	2.39E-06	0.000363659
RBMS1	1446.53518	-0.424105402	0.090125797	-4.705704878	2.53E-06	0.000382058
MEX3B	1163.41249	1.001610565	0.213245739	4.696978097	2.64E-06	0.000393194
YOD1	809.9112752	0.541173772	0.115231436	4.696407436	2.65E-06	0.000393194
SH2B1	1095.97054	0.538731973	0.114909787	4.688303655	2.75E-06	0.000405706
SESN2	485.0802846	-1.207942072	0.258468307	-4.673463008	2.96E-06	0.000432592
EPHA4	159.0860034	1.122853848	0.240978342	4.659563347	3.17E-06	0.000459091
CALCB	374.5138914	1.958609062	0.420661026	4.656026926	3.22E-06	0.000463277
S100A4	113.6606694	-1.167710131	0.251689866	-4.639480119	3.49E-06	0.000497943
PDE8A	1184.10221	0.665472557	0.14423058	4.613949105	3.95E-06	0.000558769
KIF7	520.5179784	0.660808352	0.143344506	4.609931487	4.03E-06	0.000565191
KIF26B	1524.503285	-0.761315975	0.165375882	-4.603548987	4.15E-06	0.000574971
RSU1	1397.232943	-0.689745049	0.149843218	-4.603111566	4.16E-06	0.000574971
PDLIM7	1230.22989	0.631349871	0.137552198	4.589892996	4.43E-06	0.0006079
PRPSAP2	581.1128456	-0.537569104	0.117236572	-4.585336246	4.53E-06	0.000616567
TRPV2	1196.636605	-0.735885526	0.160587801	-4.582449737	4.60E-06	0.000620406
H2AC20	1402.113432	-0.767439129	0.167625307	-4.578301108	4.69E-06	0.000628078
LINC02593	162.1719556	1.386783548	0.303247909	4.573101769	4.81E-06	0.000639069
SLC4A7	1411.882539	0.556807114	0.121870284	4.568850538	4.90E-06	0.000647336
DHRS3	286.0663181	3.577860923	0.785124158	4.557063859	5.19E-06	0.000678983
S100A2	143.0908137	-1.310343915	0.287624494	-4.555745223	5.22E-06	0.000678983
HR	86.863478	-2.469910374	0.545137613	-4.530801611	5.88E-06	0.000758774
RGS13	26.68883798	-1.997658761	0.441416172	-4.525567681	6.02E-06	0.000772203
COL2A1	81.89778464	2.457949832	0.546922638	4.49414535	6.98E-06	0.00088909
USP3	1443.28335	-0.469982387	0.104882647	-4.481030933	7.43E-06	0.000936978
SGSH	317.5426664	-0.608856651	0.135907303	-4.479940648	7.47E-06	0.000936978
MIR1915HG	335.7482316	0.664660339	0.148421326	4.478199712	7.53E-06	0.000938044
IQGAP3	1145.142498	-0.555168899	0.124247638	-4.468245105	7.89E-06	0.000975941
PTX3	224.5853668	-1.590781981	0.356962583	-4.456439017	8.33E-06	0.001024125
MYLK	501.5711404	0.683713886	0.153635332	4.450238602	8.58E-06	0.001046924
MTHFD2	3744.058769	-0.755386523	0.17004681	-4.442226963	8.90E-06	0.001073507
GPC4	1088.579862	-0.462236549	0.104062219	-4.441924776	8.92E-06	0.001073507
MKNK2	817.8653136	-0.718199878	0.161969754	-4.434160461	9.24E-06	0.001105459
DIPK1A	298.7619803	0.632959549	0.142836368	4.431361277	9.36E-06	0.001112444
KIT	14.95510558	3.656300965	0.829665131	4.4069599	1.05E-05	0.001237152
EPSTI1	121.3536458	-1.407923107	0.319633858	-4.404799651	1.06E-05	0.001241323
FBXO32	448.8015592	-1.694728895	0.385759193	-4.393230093	1.12E-05	0.00130073
SLC1A1	377.0456199	0.671200272	0.153253228	4.379681135	1.19E-05	0.0013753
BEND4	280.7322132	0.732132529	0.167703605	4.365633804	1.27E-05	0.001448995
SLC2A3	181.3491771	-1.263392007	0.289405532	-4.365472892	1.27E-05	0.001448995
TK1	792.6307398	-0.476682906	0.109278722	-4.36208347	1.29E-05	0.001462258
RNF125	269.6067152	-0.652274127	0.150179924	-4.343284423	1.40E-05	0.001582324
PSPH	515.7850652	-1.007248844	0.231977443	-4.342012018	1.41E-05	0.001582324
COL11A2	32.56060258	2.302594346	0.530884332	4.337280662	1.44E-05	0.001600802
LOXL1	470.9982685	-0.749151764	0.172746438	-4.336713246	1.45E-05	0.001600802
KHDRBS1	5176.940296	-0.388191018	0.089765993	-4.32447754	1.53E-05	0.001681832
ANXA1	3273.458104	-0.533244792	0.123473876	-4.31868512	1.57E-05	0.001715984
CDH10	1282.773007	0.497701213	0.115341606	4.315018911	1.60E-05	0.001734073
LAMB1	9020.262989	-0.583619952	0.136321392	-4.281205946	1.86E-05	0.002007539
ARFGAP1	1782.99054	0.518406948	0.122256768	4.240312892	2.23E-05	0.002396129
INPP4B	509.6457161	-0.773910911	0.182727031	-4.235338947	2.28E-05	0.002435121
ZNF74	460.5028743	0.606343347	0.143225541	4.23348616	2.30E-05	0.002440665
SARS1	2492.479659	-0.62688346	0.148647698	-4.21724298	2.47E-05	0.002607703
UBASH3B	100.6810971	-1.02246628	0.242866946	-4.209985324	2.55E-05	0.00267706
LGALS1	3389.748428	-0.463408477	0.11014665	-4.207195376	2.59E-05	0.002694463
ZNF592	969.2089874	0.438157997	0.10466057	4.186466772	2.83E-05	0.002935435
FAM53C	812.4047109	-0.394438824	0.094372891	-4.179577627	2.92E-05	0.00299141
ADAM19	2501.013112	-0.644477857	0.154198021	-4.179546875	2.92E-05	0.00299141
DDIT3	112.6889206	-1.020473303	0.244341527	-4.176421896	2.96E-05	0.003008379
AP5B1	434.5406844	0.573482116	0.137369314	4.174746883	2.98E-05	0.003008379
STAT6	1841.601495	-0.348537742	0.083494697	-4.174369799	2.99E-05	0.003008379
SPRED2	641.9328851	-0.534402065	0.128295274	-4.165407267	3.11E-05	0.003111464

ATP2A1	89.96353491	1.225388676	0.294887522	4.155444311	3.25E-05	0.00323205
OLFML2A	938.3308291	-0.950779407	0.230942825	-4.116947155	3.84E-05	0.003800848
TMED8	1299.247249	0.473096574	0.115058328	4.111797733	3.93E-05	0.00386517
ARMT1	732.297584	-0.565682309	0.138126589	-4.095390425	4.21E-05	0.004126579
RBMS3	356.3422634	-0.58904454	0.143972527	-4.091367654	4.29E-05	0.004175885
FAM3C	1107.426557	0.56843534	0.139095393	4.0866583	4.38E-05	0.00423836
PDE3A	314.6681304	-0.864463914	0.211690042	-4.083630517	4.43E-05	0.004270781
SPRY2	203.9648315	-1.197041154	0.293935653	-4.07245988	4.65E-05	0.004456841
BNC2	1171.767133	-0.412357032	0.101477159	-4.063545284	4.83E-05	0.004605845
FBLN1	3088.919114	-0.799968286	0.197359056	-4.053364976	5.05E-05	0.004785442
DUSP6	32.67403046	-2.249057476	0.556532284	-4.04119858	5.32E-05	0.004997864
POSTN	114.8038642	-1.957500139	0.484443803	-4.040716649	5.33E-05	0.004997864
MAP4K4	3367.654805	-0.376537631	0.093329503	-4.034497324	5.47E-05	0.005087158
MXD4	1051.054406	-0.508345194	0.126011933	-4.034103616	5.48E-05	0.005087158
ZNF521	602.3492601	-0.484367783	0.120108238	-4.032760705	5.51E-05	0.005089817
EYA2	320.9452286	2.367980318	0.588135499	4.026249604	5.67E-05	0.005205785
CXCL14	127.6096961	1.847465507	0.460125856	4.015130821	5.94E-05	0.005422342
HMGA2	960.2706392	-0.794290804	0.197868686	-4.01423197	5.96E-05	0.005422342
H3C13	594.0920824	-0.902510177	0.225095696	-4.009451066	6.09E-05	0.00550521
CTH	409.4931994	-1.207663293	0.302084551	-3.997765825	6.39E-05	0.005754885
SH3BP1	132.8778792	-0.945296062	0.237061577	-3.98755493	6.68E-05	0.005978006
AARS1	5226.100532	-0.628484863	0.158012433	-3.977439319	6.97E-05	0.00620683
SMIM3	374.2654072	-1.104490035	0.277931283	-3.973968043	7.07E-05	0.006266705
SCARF2	1412.475989	0.442986787	0.111613408	3.968938813	7.22E-05	0.006368746
TES	851.8282849	-0.542596704	0.136919337	-3.962893169	7.40E-05	0.00650008
XPR1	5402.306926	1.886153825	0.476736601	3.956385606	7.61E-05	0.006646886
BNC1	202.0404297	0.683257332	0.172853775	3.952805379	7.72E-05	0.006714251
HSPG2	2691.317261	0.610991512	0.155107731	3.939142874	8.18E-05	0.007058483
NIBAN2	3421.597057	-0.509145415	0.12927393	-3.93850033	8.20E-05	0.007058483
ULBP1	38.8190568	-1.631697324	0.414826049	-3.933449521	8.37E-05	0.007173872
ITGA3	66.21679946	-1.335254345	0.3404831	-3.921646463	8.79E-05	0.007498551
ARRDC4	75.30794994	-1.498461714	0.382974454	-3.912693654	9.13E-05	0.007745106
PLPP1	206.7886559	0.646435222	0.165305896	3.910539421	9.21E-05	0.007777478
PPEF1	61.20594741	-1.081302783	0.276606322	-3.909175947	9.26E-05	0.007784608
SLCO5A1	456.4794914	0.514946836	0.131907597	3.903845164	9.47E-05	0.007920811
PLEKHG1	280.6409209	-0.878282709	0.225119662	-3.901403812	9.56E-05	0.007963747
TUBB2B	505.4954533	0.479453642	0.123027521	3.897125107	9.73E-05	0.008068012
ARSJ	450.7161989	-0.706640521	0.181412986	-3.895203623	9.81E-05	0.008094592
SEMA6A	234.1020627	-0.990620623	0.25453275	-3.891918125	9.95E-05	0.008167211
NUPR1	351.4683748	-0.806979199	0.207875805	-3.882025607	0.00010359	0.008467767
TRIOBP	500.0253158	-0.529823942	0.136609754	-3.878375637	0.000105156	0.00855655
ACSL3	2590.22767	0.683213926	0.176341351	3.874382955	0.000106895	0.008658512
IMPA1	453.6143608	0.445871172	0.115221384	3.86969118	0.000108973	0.008779304
H4C5	674.8993257	-0.675162176	0.174514573	-3.868801124	0.000109372	0.008779304
SERTAD4	485.264552	-0.658353724	0.170295294	-3.865953711	0.000110656	0.008823041
TMEM120B	963.4119186	0.471365461	0.121944782	3.86540081	0.000110907	0.008823041
STXBP5	1037.544882	0.338016799	0.087479793	3.863941449	0.000111572	0.008836501
INHBE	581.8869342	-1.200979708	0.311829497	-3.851398668	0.000117445	0.009260496
BCAT1	5170.885937	-0.489645525	0.127319384	-3.845805023	0.000120157	0.009432612
NRN1	297.5882713	-0.782984625	0.204060358	-3.837024651	0.000124534	0.009725718
NCALD	254.6390308	0.745561441	0.194351929	3.836141188	0.000124983	0.009725718
FGF7	37.88051612	-1.735791676	0.453113103	-3.830813242	0.00012772	0.009895556
CUTALP	195.2831688	-0.782569161	0.20504882	-3.816501652	0.000135357	0.010441839
GRIK3	237.0380291	-0.779402031	0.204285187	-3.815264545	0.000136037	0.010449058
PLCB1	378.3087814	-0.556076726	0.145909992	-3.811094214	0.000138353	0.010581334
PPFIBP2	17.13721498	3.289544726	0.863909274	3.807743272	0.000140241	0.010679873
ITGA10	1218.793545	-0.596345516	0.156683011	-3.806063676	0.000141196	0.010706866
DDHD1	891.2273202	0.448681094	0.117961025	3.803638481	0.000142586	0.010723881
SOX6	559.6461349	0.607476022	0.159711945	3.803572871	0.000142624	0.010723881
PKM	21732.30581	-0.636086592	0.167401495	-3.799766506	0.000144832	0.01084418
CILP2	109.7571428	1.334305274	0.352296621	3.787448405	0.000152202	0.011348304
TPCN1	2075.942784	-0.626117347	0.165511512	-3.78292326	0.000154997	0.011508543
ADM2	99.77572532	-0.9545239	0.252541716	-3.779668224	0.000157037	0.01161165
THOC2	1963.249919	0.382196919	0.101252564	3.774688794	0.000160208	0.011797101
CHSY3	922.0821479	0.470529002	0.124690194	3.773584659	0.000160919	0.011800696
STK17B	1585.138208	-0.68897621	0.183405855	-3.75656606	0.000172261	0.012580687
FGFR1	14803.61196	-0.722316047	0.192672338	-3.748934869	0.000177587	0.01291675
MCM2	2532.458743	-0.389181556	0.104024847	-3.741236534	0.000183117	0.013264822
H4-16	212.3001416	-0.719217755	0.192425302	-3.737646496	0.000185751	0.013380055
MLLT11	765.930871	0.429808294	0.115013455	3.737026208	0.00018621	0.013380055
TTC7A	411.8518357	0.520719437	0.13942598	3.734737501	0.000187911	0.013420128
SLC7A2	3779.857341	0.498649345	0.133533906	3.734252671	0.000188273	0.013420128
UHMK1	2206.804741	0.408858392	0.109544533	3.732348678	0.000189703	0.013468133
PPP1CA	688.109512	-0.554973382	0.149352201	-3.715870118	0.000202506	0.014320038
MMP13	120.7100209	-1.134987618	0.305830591	-3.711164451	0.000206308	0.01453126
TWF2	443.3685221	-0.576285915	0.155351117	-3.709570453	0.000207611	0.014565477
ZC3H4	1122.482013	-0.388503287	0.104828066	-3.706099923	0.000210475	0.014708508
IGF2BP3	625.944898	-0.54719642	0.147717219	-3.704350953	0.000211933	0.014752501
OLFML3	634.3179528	0.609549721	0.164613893	3.702905697	0.000213144	0.014779099
THOC5	820.8468142	-0.476253275	0.129038502	-3.690784296	0.000223564	0.01544149
SHMT2	2851.223466	-0.544647215	0.147709157	-3.687294846	0.000226651	0.015553901
VAV3	135.7947071	-1.277535775	0.346499837	-3.686973666	0.000226937	0.015553901
BBOX1-AS1	43.37246849	1.481462534	0.402048989	3.68478114	0.000228899	0.015628293
THG1L	334.6527155	-0.545298199	0.148230679	-3.678713491	0.000234413	0.015943692
STING1	35.94941389	-1.541421627	0.419631315	-3.673275975	0.000239461	0.01622505
SAMD11	850.5028747	1.91695431	0.52220624	3.670875918	0.000241721	0.016316145
GMIP	257.1053232	0.696302264	0.189880642	3.667052401	0.000245363	0.016499473
PLEKHA3	772.7889007	0.522820211	0.142793818	3.661364458	0.000250876	0.016785731
NRP2	1396.589798	0.936555034	0.255838732	3.660724187	0.000251503	0.016785731
LZTS1	346.9806601	0.596227598	0.163214315	3.653034961	0.000259159	0.017232135

CENPP	371.1518393	0.537074694	0.147120707	3.650571732	0.000261657	0.017333575
KIAA1549	506.7318749	0.435704623	0.119639494	3.64181266	0.000270725	0.017867854
GCLC	1021.90463	-0.433352082	0.11915816	-3.636780581	0.000276067	0.018153182
CRYBG1	659.0082135	-0.549199191	0.151362664	-3.628366303	0.00028522	0.01868613
PARVA	863.2764357	-0.570401515	0.157349269	-3.625066181	0.000288887	0.018763332
FIBIN	849.6524878	-1.048881764	0.289373951	-3.624658542	0.000289343	0.018763332
RGS1	57.56034586	-1.458000927	0.402308874	-3.62408344	0.000289988	0.018763332
CCDC171	357.9729953	-0.774457365	0.213730137	-3.623529073	0.000290611	0.018763332
C11orf24	364.5103693	0.480787861	0.132840463	3.619287742	0.000295415	0.019004677
CCND1	2816.033324	-0.437957755	0.121048878	-3.618024069	0.000296861	0.01902899
LETM2	278.7999534	-0.658987665	0.182218939	-3.61646088	0.000298658	0.019061206
TRIM8	318.191239	-0.732538392	0.20259766	-3.615729773	0.000299503	0.019061206
RSL24D1	1971.417726	-0.563511278	0.156034784	-3.611446527	0.000304494	0.019309896
PRKAB2	1287.813686	0.370300863	0.102570085	3.61022282	0.000305934	0.01933243
C1QTNF7	108.315581	-1.049300919	0.291022083	-3.605571475	0.000311467	0.0196125
BTBD3	567.0286033	-0.415656489	0.115325589	-3.604200005	0.000313116	0.019646919
ISLR	122.2306454	1.635260871	0.453887029	3.602792692	0.000314817	0.019684322
VCL	7802.592374	-0.332624692	0.092412163	-3.59936054	0.000319001	0.019855574
OAZ2	665.5008633	-0.497084546	0.138128049	-3.598722703	0.000319784	0.019855574
PIK3CB	645.3664574	0.424096794	0.118272284	3.58576649	0.00033609	0.020795544
CAMKK1	364.5410064	0.670352166	0.18704429	3.583922097	0.000338473	0.020807987
GNAO1	34.0368875	-2.251890733	0.628492013	-3.583006126	0.000339663	0.020807987
FAM78B	22.52770805	-2.296784624	0.64103971	-3.582905377	0.000339794	0.020807987
PRR12	846.0102848	0.362488311	0.101221385	3.581143547	0.000342094	0.020877084
LOC100289511	52.34117317	1.079582918	0.301606992	3.579435973	0.000344337	0.020942246
SLC25A30	219.6322441	0.633796191	0.177285264	3.575007743	0.000350218	0.021163207
TLR8	18.52179919	-1.701583326	0.475978998	-3.574912615	0.000350345	0.021163207
MAST2	1779.432068	0.64780363	0.181335322	3.57240731	0.000353715	0.021277408
WNK3	313.241886	0.682011212	0.190946729	3.571735503	0.000354623	0.021277408
COL21A1	57.5025247	-1.077400789	0.301845731	-3.569375603	0.000357833	0.021397938
PLCB4	414.5717141	0.694146884	0.194596482	3.567109112	0.000360941	0.021511614
GREM2	215.46277	0.632170619	0.177451699	3.562494032	0.000367348	0.021738989
GSN	2311.34055	-0.68156438	0.19133041	-3.562237599	0.000367707	0.021738989
ETS2	929.7543801	0.677594714	0.190255425	3.561500083	0.000368742	0.021738989
RBM33	993.3361511	0.33454524	0.093950571	3.560864355	0.000369636	0.021738989
TFAP2A	70.91699743	-1.288307058	0.36231029	-3.555811393	0.000376814	0.022071808
C2CD2	282.0059554	0.507519902	0.1427565	3.555143922	0.000377772	0.022071808
VWA8	988.9415783	0.373950732	0.105384201	3.548451561	0.000387503	0.022538335
ADAMTS14	114.3210482	-1.184325688	0.333808489	-3.547919623	0.000388287	0.022538335
IGFBP7	630.3741592	-0.74961091	0.21137329	-3.546384264	0.000390556	0.022596469
BTG1	2867.681428	-0.704743309	0.199018374	-3.541096704	0.000398468	0.022979582
TXNDC17	123.8875934	-0.636907619	0.18003615	-3.537665177	0.000403682	0.023205181
GARS1	4719.981164	-0.66427016	0.188069256	-3.532050768	0.00041235	0.023627268
SMG5	2832.32444	0.346505787	0.098293247	3.525224741	0.000423124	0.024099807
RUSC2	363.2147374	0.525522142	0.149079509	3.525113184	0.000423302	0.024099807
ARSL	903.8151893	0.458149516	0.130055486	3.522723484	0.000427137	0.024240691
CTDSP1	1047.490731	-0.44557436	0.126591065	-3.519793135	0.000431883	0.024432267
E2F2	187.1072087	-0.783459078	0.222885278	-3.515077736	0.000439625	0.024791527
CLDN11	43.70536461	-1.337744567	0.38098017	-3.511323352	0.000445882	0.025065021
RSPO2	866.70405	0.754063681	0.214928315	3.508442714	0.000450738	0.025258353
ZFP36L2	568.6323716	-0.566730334	0.161756883	-3.503593313	0.000459026	0.025642131
STC1	20.11290698	-2.819718045	0.805654084	-3.499911562	0.000465413	0.025917661
STX16	1215.816276	0.35461238	0.101574222	3.491165102	0.000480919	0.026597682
EBF2	379.7505912	-0.685777994	0.196451973	-3.490817553	0.000481545	0.026597682
FZD1	1853.888489	-0.541037636	0.155002511	-3.490508849	0.000482102	0.026597682
KHDRBS3	31.68373364	-1.488551591	0.426686415	-3.488631323	0.0004855	0.026702515
DIPK2A	2535.458514	0.720100424	0.206524667	3.486752615	0.000488923	0.026808046
CD81	2557.346218	-0.340540717	0.097841971	-3.480517759	0.000500446	0.027355648
TLN2	2015.443443	0.306298423	0.088032742	3.479369335	0.000502595	0.027389144
CNTNAP2	149.7931755	1.096551426	0.315387267	3.476841141	0.000507358	0.027564407
MYLIP	840.7969809	0.541827173	0.155896653	3.475553608	0.0005098	0.02761288
LOC107985911	284.0132524	0.582768815	0.167757576	3.473874799	0.000513	0.027702019
S100A6	3105.037912	-0.672466534	0.193644554	-3.472684987	0.00051528	0.027741044
ATP2B1	3701.62405	-0.432707237	0.12521215	-3.455792718	0.000548677	0.029450077
LINC00703	167.2881805	-0.831654567	0.240766612	-3.454193923	0.00055194	0.029536267
TPRG1	207.9066016	-1.181682172	0.342219438	-3.452995477	0.000554398	0.029562756
FKBP9P1	40.23713148	-1.041310098	0.301624701	-3.452336943	0.000555753	0.029562756
RASA1	2077.275874	-0.43349678	0.125684408	-3.449089557	0.00056248	0.029831535
NR2F2	732.8847715	-1.09897851	0.3191463	-3.443494444	0.000574248	0.030365301
LPCAT3	238.9420437	0.565846119	0.164625659	3.437168431	0.00058783	0.030991507
RGS2	753.5811576	-1.29141964	0.377028224	-3.425259857	0.000614212	0.0322642
IWS1	501.7355522	0.427997588	0.124975553	3.424650491	0.000615591	0.0322642
FAM241A	289.9879321	-0.616686744	0.180517121	-3.416223015	0.000634962	0.033181897
PACSIN2	992.385712	0.638298551	0.187023784	3.41292716	0.000642691	0.033487593
ECHS1	847.8611573	-0.438353827	0.128645855	-3.407446189	0.000655738	0.034067807
LPCAT4	728.7072973	0.501196686	0.147212049	3.404590112	0.000662634	0.034326
PTN	822.2095314	0.424619233	0.124906045	3.399509062	0.000675069	0.034868803
SNORA75	18.19778103	2.041060562	0.602490329	3.387706763	0.000704796	0.03605182
GPR180	1042.801306	0.334232469	0.098669711	3.387386719	0.000705618	0.03605182
CHN1	541.1079718	-0.796824829	0.23524098	-3.387270485	0.000705918	0.03605182
TSEN15	899.3800563	-0.543852903	0.160560584	-3.387213035	0.000706065	0.03605182
MICAL2	1746.031822	-0.857808312	0.253425975	-3.384847639	0.000712178	0.036166669
H3C1	259.721939	-0.715749423	0.21146163	-3.384772082	0.000712374	0.036166669
SNAI1	250.706038	1.172904666	0.347037872	3.379759852	0.000725492	0.036728028
SLIT2	1898.309619	-0.990373437	0.293249139	-3.377242433	0.000732165	0.036908361
LINC01583	123.9485451	1.235795859	0.365960573	3.376855192	0.000733196	0.036908361
ZNF692	669.9863501	0.541267219	0.160803574	3.366014859	0.000762626	0.038281677
ARF5	792.0163921	-0.490644217	0.1458814	-3.363308932	0.000770141	0.03855033
VPS33A	632.1441486	-0.437767674	0.130282425	-3.360143743	0.000779019	0.038885501
KLHL3	225.986074	0.874328351	0.260333971	3.35848736	0.000783703	0.039010024

APOL4	33.87368051	-1.546247619	0.461171202	-3.352871153	0.000799779	0.039556506
HECW1	349.8731413	0.571709375	0.170523455	3.352672944	0.000800352	0.039556506
CTHRC1	2241.122601	-0.583624794	0.174095211	-3.352331132	0.000801341	0.039556506
LINGO1	502.8512801	0.397073376	0.118521504	3.350222209	0.000807468	0.039748817
SLC6A9	214.5459559	-1.133995273	0.338671664	-3.348361827	0.000812908	0.039811452
RBM3	2916.182372	-0.580927879	0.173501447	-3.348259556	0.000813208	0.039811452
KRT18	16.63404246	-2.108203456	0.63061009	-3.343117229	0.000828429	0.04039873
GPT2	958.9596843	-0.842736297	0.252114037	-3.342678996	0.000829738	0.04039873
GRAMD1A	656.1205518	0.479935576	0.143691832	3.340033803	0.000837682	0.040674365
CDT1	658.5674928	-0.38785839	0.116222827	-3.337196307	0.000846282	0.040980263
CCDC34	277.7692899	0.574703627	0.172330199	3.334897942	0.000853307	0.04120849
ARHGAP18	172.4943227	-0.621965146	0.186938452	-3.327111895	0.000877511	0.041958118
GPR1	40.53230172	-1.15676507	0.34772218	-3.326693365	0.00087883	0.041958118
FGF5	332.2512165	-0.541022794	0.162670871	-3.325873837	0.000881418	0.041958118
LOC102723753	187.301438	0.77772752	0.233844371	3.325833832	0.000881545	0.041958118
NTRK1	61.36608569	-1.694304561	0.509492294	-3.325476328	0.000882676	0.041958118
H4C2	1652.991938	-0.600519014	0.180586173	-3.325387572	0.000882957	0.041958118
PAWR	1792.741928	0.521841386	0.15697856	3.324284443	0.000886457	0.042012419
LINC01426	37.82681691	-1.168022884	0.351789419	-3.320233131	0.000899423	0.042414744
PLP1	59.10765219	-0.982516547	0.295925785	-3.320145109	0.000899707	0.042414744
INPP5B	900.2445346	-0.393813634	0.118713786	-3.317336998	0.000908799	0.042730351
PTPRC	125.4331789	-0.727183694	0.219331346	-3.31545722	0.000914933	0.042905563
TMX3	1962.369245	-0.384287189	0.11603977	-3.311685198	0.000927358	0.04326367
ALS2	560.754653	0.401232635	0.121157376	3.31166495	0.000927425	0.04326367
KNOP1	840.1965355	0.324097022	0.097924179	3.309673125	0.00093405	0.043351054
S100A13	312.7046418	-0.575130829	0.17380878	-3.308986053	0.000936345	0.043351054
GNG2	463.8080589	-0.644758588	0.194894305	-3.308247453	0.000938818	0.043351054
THRB	246.6778653	0.927840267	0.280468122	3.308184404	0.00093903	0.043351054
NEMP1	1453.504393	-0.276470941	0.083630946	-3.30584495	0.000946905	0.043601658
DOCK5	3356.944962	0.284542118	0.086117444	3.304117091	0.00095276	0.043758218
LZTS1-AS1	5.310156048	4.903869705	1.484824907	3.302658571	0.000957729	0.043873354
MSRB3	1154.911701	-0.384707786	0.116536903	-3.301167049	0.000962835	0.04399417
IL31RA	94.11129661	-0.944393867	0.286624364	-3.294883433	0.000984625	0.044775165
RABGGTB	1001.18999	0.505598147	0.153453838	3.294789841	0.000984953	0.044775165
FAM216B	40.25076912	-1.524986871	0.464373653	-3.283965102	0.001023576	0.046412528
EPC2	321.2592615	-0.67735078	0.206525192	-3.279748939	0.001038995	0.046865919
INTS4P2	23.97314568	2.268680186	0.691862083	3.279093105	0.001041413	0.046865919
PHGDH	3666.170368	-0.614829765	0.187500749	-3.279078974	0.001041465	0.046865919
BORCS7	45.11378331	-1.070331669	0.326498126	-3.278216882	0.001044651	0.046890883
CXXC4	84.9921074	-0.725330501	0.221808379	-3.270077103	0.001075182	0.048140047
LOC101929130	18.6387578	-1.78674077	0.547319202	-3.264531489	0.001096453	0.048847734
NPAS2	381.686967	-0.492855555	0.150973034	-3.26452706	0.00109647	0.048847734
ASNSP1	104.0703474	-0.801188051	0.245683389	-3.261059104	0.001109969	0.049325805
DNAJB4	548.5918188	0.478121863	0.146725571	3.258613064	0.001119583	0.049629256
PLP2	428.1778671	-0.500332994	0.153609584	-3.257173027	0.001125278	0.04974547
EIF2S2	1590.594532	-0.473701834	0.145461662	-3.256540774	0.001127787	0.04974547
FAM102A	600.324259	-0.625062554	0.192191958	-3.252282574	0.001144821	0.050372144
INKA2	145.978821	-0.889643993	0.273731465	-3.250061124	0.001153802	0.050642248
PLPP2	81.90860403	-0.742483769	0.228698446	-3.246562368	0.001168079	0.051142897
PTPRG	1322.625367	-0.511790118	0.158089738	-3.237339276	0.001206499	0.052695609
ADAMTS7	141.5962832	0.793123952	0.245349019	3.232635519	0.001226539	0.053439934
BMP2	906.6486015	-0.95283101	0.295090061	-3.228949859	0.001242457	0.054001414
STAB1	58.95790931	-1.430537307	0.443329371	-3.226804719	0.001251809	0.054199428
SLC26A11	65.30037169	-0.836022811	0.259110506	-3.226510662	0.001253096	0.054199428
H2BC18	718.8054357	-0.786427826	0.243954994	-3.223659471	0.001265638	0.054609371
PYROXD1	240.3147164	-0.512334004	0.159019477	-3.221831779	0.001273739	0.054826155
MICA	925.6977148	0.420701558	0.130662055	3.219768413	0.001282942	0.055089218
H2AC14	166.8443933	-0.918394732	0.285495441	-3.21684553	0.001296084	0.055519735
GYG2	184.531581	0.518944256	0.16154598	3.21236254	0.001316481	0.056258266
RALGAPA1	787.183356	0.428493594	0.133596612	3.207368711	0.001339552	0.057030666
CLIC5	775.2727791	-0.84791227	0.264437032	-3.20648081	0.001343693	0.057030666
H4C9	595.735042	-0.978317125	0.305115646	-3.206381379	0.001344157	0.057030666
TWISTNB	3541.277636	-0.348756292	0.108831208	-3.204561443	0.001352684	0.057161255
LRP1	16103.40706	-0.427932382	0.133547083	-3.204355891	0.00135365	0.057161255
HAS2	2338.701007	-1.5719357	0.49070343	-3.203433283	0.001357995	0.057209151
NUDT3	3553.294397	-0.375938565	0.117528002	-3.198714862	0.001380416	0.057893084
PSAT1	2659.641316	-0.930351861	0.290883311	-3.198367951	0.001382078	0.057893084
GDI1	818.719722	0.358650209	0.112152815	3.197870776	0.001384463	0.057893084
ANTXR2	370.309266	-0.601423133	0.188126426	-3.196909359	0.001389086	0.057893084
ARHGAP24	221.5296029	-0.53800349	0.168303795	-3.19662127	0.001390474	0.057893084
DLX5	252.9905379	1.004470699	0.314376685	3.195118295	0.001397735	0.058059777
HCN1	45.48958635	-1.138409997	0.35688526	-3.189848745	0.001423473	0.058991359
DDX39A	1109.111866	-0.292386628	0.091762837	-3.186329438	0.001440904	0.059575204
TMCC2	111.5997189	0.762849861	0.239645204	3.183246933	0.001456334	0.06007376
GNAI1	516.339089	0.427019934	0.134273197	3.180232114	0.001471571	0.060562125
LARGE2	106.5225457	-0.684603316	0.215500365	-3.176808156	0.001489055	0.061140462
TMEM181	1821.583132	0.271467914	0.085507328	3.174791204	0.001499444	0.06142548
BPIFB4	347.6717101	1.079270759	0.340166196	3.172774872	0.001509896	0.061711786
ALDH1L2	6178.704291	-0.526270719	0.165917728	-3.171877573	0.001514568	0.061761119
METTL7A	45.11352191	-1.540491047	0.485861278	-3.170639679	0.001521037	0.061880879
RARB	101.4901105	0.828407382	0.261328208	3.169988371	0.00152445	0.061880879
SREBF1	2637.663204	0.489319188	0.154625564	3.164542635	0.001553269	0.062907396
JUP	20.46628283	-1.818367216	0.575042869	-3.162142016	0.001566132	0.063284503
IFFO2	382.8522564	0.758923533	0.2401091	3.160744562	0.001573664	0.06344502
JADE2	258.6242126	-0.571392487	0.181015017	-3.156602678	0.001596187	0.064207785
ST8SIA4	285.2168292	0.589318564	0.18678887	3.154998283	0.001604991	0.064416511
GRIA2	80.16336108	0.875667893	0.278247173	3.147086392	0.001649062	0.066036607
DOT1L	1501.132806	0.305268874	0.097021372	3.146408547	0.001652889	0.066041457
IL27RA	208.3186621	-0.573410286	0.182327648	-3.144944231	0.001661185	0.06622442
LHX8	126.4295598	-0.839335999	0.266966653	-3.143973183	0.001666707	0.066296252

ELF4	1033.149704	0.570013448	0.181579036	3.139202969	0.001694081	0.067235004
LOC101930085	223.9692617	-0.509118815	0.162254678	-3.137775879	0.00170235	0.067413056
ITGAV	10523.19112	-0.420029113	0.133900247	-3.136880787	0.001707555	0.067469263
NUFIP2	5333.274301	0.261529821	0.08356263	3.129746152	0.001749574	0.068899105
OSMR	820.658702	-0.554858171	0.177303437	-3.129427051	0.001751476	0.068899105
CLIC1	2710.711069	-0.381094038	0.121808583	-3.12863041	0.001756231	0.06890097
BICD1	288.66776	0.517218886	0.165344721	3.128124579	0.001759256	0.06890097
ANKRD50	862.2187207	0.430964069	0.137816504	3.127086075	0.001765482	0.06899319
WIZ	666.649961	0.30606472	0.097950211	3.124696892	0.001779884	0.069403777
ADCY7	2937.695761	0.61293214	0.196423915	3.120455779	0.001805714	0.070190733
LMNB1	1755.555424	-0.463964613	0.148702202	-3.12009241	0.001807943	0.070190733
EPHX4	42.54071335	-0.946950341	0.303717449	-3.11786611	0.001821655	0.070565606
PANK3	3826.11496	0.484451957	0.155420593	3.11703841	0.001826778	0.070565606
RBP1	16.478242	-1.591802527	0.510835229	-3.116078211	0.001832737	0.070565606
CMTM3	665.6158481	-0.625546192	0.200755187	-3.115965279	0.001833439	0.070565606
BUB1B	1499.48113	0.334391509	0.107437728	3.112421622	0.001855593	0.071264361
ABR	995.7804362	-0.491819966	0.158052888	-3.111742981	0.001859863	0.071274768
C15orf39	927.5311442	-0.437648661	0.140707131	-3.11035169	0.001868647	0.071457706
ENTPD4	2324.336485	0.439521197	0.141425467	3.107793856	0.001884895	0.071924685
PSTPIP2	110.3604038	-0.842161504	0.271296918	-3.104205937	0.001907905	0.072647145
SFSWAP	688.2612397	0.457606404	0.147483353	3.10276648	0.001917209	0.072750143
ZNF680	205.5936775	0.536452687	0.172908429	3.102524781	0.001918775	0.072750143
PRNP	1402.824968	-0.533598885	0.172593195	-3.091656567	0.00199043	0.075306709
IER2	564.7527046	-0.540164341	0.174866697	-3.089006378	0.002008271	0.07582075
GRIN3A	100.232694	0.802109648	0.259917617	3.086014936	0.002028586	0.076372834
CAPN5	38.40219895	-1.001710228	0.324709342	-3.08494428	0.002035903	0.076372834
SLC5A3	1433.163376	0.563987678	0.182852904	3.08437911	0.002039775	0.076372834
NDUFS1	1358.302665	-0.29183493	0.09461825	-3.084340807	0.002040038	0.076372834
DSE	1594.29273	0.293081164	0.095144241	3.080387835	0.002067312	0.077231657
MCTP2	876.3717887	-0.628198836	0.204426839	-3.072976331	0.002119353	0.079010188
TMSB4X	4946.897322	-0.707639357	0.230332186	-3.072255635	0.002124477	0.079035867
EMC2	1214.588204	0.40967707	0.133420634	3.070567552	0.002136523	0.079318435
PLXNA2	1245.387117	0.973445791	0.317707567	3.063967913	0.002184223	0.080853864
LTBP2	724.9520776	-0.966066974	0.315355737	-3.06341969	0.002188229	0.080853864
RAB27B	574.2984727	-0.750145697	0.244938083	-3.062593155	0.002194282	0.080853864
TRIM2	925.7951813	0.83800276	0.273646541	3.062354662	0.002196031	0.080853864
NSMF	1194.661182	-0.420883085	0.137481466	-3.061380539	0.002203189	0.080950157
AKNA	287.9461736	-1.054953902	0.345328339	-3.054929998	0.002251131	0.082370113
EVI2A	254.0997233	-0.569621123	0.186475683	-3.054667043	0.002253105	0.082370113
UNC119B	1618.253117	-0.359414038	0.117673939	-3.054321466	0.002255702	0.082370113
COMMD4	545.3146762	-0.417762499	0.136978453	-3.049840987	0.002289626	0.083414215
USP38	486.4369568	0.360129518	0.118116559	3.048933356	0.002296554	0.083414215
SNAPC3	468.504363	0.366736179	0.120292638	3.048700117	0.002298338	0.083414215
SLC5A12	10.88274437	3.162771876	1.038824215	3.044568879	0.002330141	0.084320687
ZFAND2B	148.75166	0.539983291	0.177379348	3.044228645	0.002332778	0.084320687
FBXO11	1852.738276	-0.382857452	0.125856327	-3.042019917	0.002349963	0.084687144
GK5	751.9966794	0.513508557	0.168822586	3.041705322	0.002352421	0.084687144
S100A16	214.5256801	-0.790936273	0.260082911	-3.041092815	0.002357212	0.084688528
ATF4	3309.241169	-0.502003609	0.16512283	-3.040182939	0.002364345	0.08472111
H2BC17	1389.948693	-0.558386983	0.183719232	-3.039349651	0.002370895	0.08472111
CRISPLD1	1019.426589	-0.384229123	0.126426056	-3.039160885	0.002372381	0.08472111
DYNC2H1	2050.86933	-0.628307929	0.20685694	-3.037403189	0.00238626	0.084952735
ENPP3	18.43910187	-1.532754789	0.504671634	-3.03713283	0.002388402	0.084952735
CAMK2N1	15.80373607	-1.795371929	0.591463419	-3.035474169	0.002401578	0.085251233
ANPEP	613.4731463	-0.978836133	0.322695939	-3.033307876	0.002418887	0.085688248
SLC20A2	674.0178445	0.397927623	0.131210935	3.032732156	0.002423506	0.085688248
BCL3	152.2869808	1.410800703	0.465558537	3.030340102	0.002442785	0.08601602
PTGS1	33.91539783	-1.282489319	0.423241467	-3.030159891	0.002444243	0.08601602
GTF2E2	1085.566117	0.32192084	0.106253204	3.029751845	0.002447547	0.08601602
CEBPG	1067.601958	-0.463444198	0.152992655	-3.029192474	0.002452084	0.08601602
STMN1	1551.331697	-0.409587273	0.1352418	-3.028555332	0.002457261	0.086028264
SNORA33	29.11257806	1.252491647	0.413860056	3.026365139	0.002475132	0.086484026
GABARAP	1136.496537	-0.60782824	0.200913453	-3.025323746	0.002483671	0.086612564
VSIR	59.91672311	1.784221817	0.590498955	3.021549493	0.002514845	0.087528403
MCMBP	1247.019938	-0.273711445	0.090684663	-3.018277117	0.002542163	0.088306723
LINC02709	14.06587464	-2.073900092	0.687447104	-3.016814063	0.002554464	0.088561395
SLC37A2	173.5656003	1.256644257	0.416728565	3.01549825	0.002565574	0.088773849
HERPUD1	948.7232052	-0.439534651	0.145812508	-3.014382354	0.002575031	0.088928381
SBNO2	1019.04851	0.296711645	0.098465633	3.013352322	0.002583788	0.089058211
PPRC1	1307.883874	0.420956083	0.139922817	3.008487773	0.002625514	0.090321725
UBXN2B	950.7126521	-0.346102182	0.115153969	-3.005560158	0.002650922	0.09102008
COL8A1	125.9377746	-1.304254799	0.434365953	-3.002663513	0.002676282	0.091714111
CEP68	633.3203252	-0.349916707	0.11661435	-3.000631632	0.002694203	0.092151043
FRMD4B	175.063822	0.814679142	0.271639083	2.999123437	0.002707576	0.092431039
ATP2B4	3957.172664	0.35629951	0.118883627	2.997044405	0.00272611	0.092885814
ADGRE5	284.6735974	-0.567415577	0.189437312	-2.995268303	0.002742035	0.093018051
ZFAND5	2389.435225	0.372751386	0.124455844	2.995049271	0.002744005	0.093018051
SLC25A44	417.7849407	0.381448608	0.127389202	2.994355893	0.002750249	0.093018051
HMGXB3	1822.167954	0.306847514	0.102477656	2.994287	0.002750871	0.093018051
RBMX	3811.92545	-0.254737335	0.085114928	-2.992863201	0.002763736	0.09327609
SDC1	2898.136945	0.383274684	0.128334945	2.986518471	0.002821738	0.094900221
WWC1	354.4003349	0.429335194	0.143761779	2.986434889	0.002822509	0.094900221
SCD	26100.65165	0.883010673	0.295755066	2.985614705	0.00283009	0.094975894
PTGES	1430.695635	2.75182714	0.92229198	2.983683257	0.002848014	0.095285498
PTPN13	3029.582206	-0.423701558	0.142016413	-2.983468944	0.00285001	0.095285498
RRBP1	4805.72602	0.697649967	0.233977478	2.981697102	0.002866555	0.095583661
ABCA2	149.2904216	-0.686488039	0.230315739	-2.980638845	0.002876478	0.095583661
NCR3LG1	501.7124265	0.480629476	0.161269869	2.980280687	0.002879844	0.095583661
AAK1	1768.505216	0.347906766	0.116738488	2.980223326	0.002880383	0.095583661
CD44	4400.546799	-0.687860919	0.231159211	-2.975701967	0.002923188	0.096688077

MON2	2723.610208	0.292523226	0.098308542	2.975562666	0.002924516	0.096688077
GPR183	172.0385821	0.896205733	0.30134038	2.974064527	0.002938833	0.096981478
REXO1	662.3499027	0.334754553	0.112698626	2.970351677	0.00297459	0.097980028
FAS	1380.320272	-0.619807606	0.208760679	-2.968986351	0.002987839	0.098234843
SH3RF2	170.2879297	0.877326654	0.295646892	2.967481402	0.003002504	0.098520012
VLDLR	409.075782	-0.90680917	0.305635461	-2.966963216	0.003007569	0.098520012
H2AZ2	802.8494161	-0.311531835	0.105078944	-2.964740831	0.00302938	0.099052396
MLIP	448.4788005	-0.712588268	0.240614907	-2.961530009	0.003061147	0.099907752
HTRA3	599.2062638	-1.172594789	0.396168728	-2.959836823	0.00307802	0.100121685
NR2F1	249.5606659	-0.542272824	0.183216066	-2.959744931	0.003078938	0.100121685
C18orf25	656.667122	0.287182501	0.097109838	2.957295645	0.003103504	0.100736684
WARS1	2508.283813	-0.707498679	0.239303069	-2.956496476	0.003111558	0.100814475
CDC42	4332.800155	-0.355751441	0.120383851	-2.955142564	0.003125246	0.101074199
TWIST1	4170.491253	-0.484575524	0.164018554	-2.954394571	0.003132832	0.101135982
FYN	1319.548862	-0.63181872	0.213968439	-2.952859414	0.003148453	0.101199407
CPN2	5.929256367	5.22307976	1.768977619	2.952597989	0.00315112	0.101199407
PPFIBP1	1525.13772	-0.42822467	0.145036609	-2.952528142	0.003151833	0.101199407
PRTFDC1	111.7334635	-0.648007736	0.219571143	-2.951242714	0.003164981	0.101438781
LTBP3	132.6056732	-0.854376779	0.28962006	-2.949991721	0.003177824	0.101667561
H2AC15	267.8649638	-0.717872483	0.243453271	-2.948707489	0.003191059	0.101907998
IBTK	3378.583405	0.23477226	0.079755754	2.943640422	0.003243766	0.103254085
SRSF4	762.6930293	0.490424079	0.166610182	2.94354206	0.003244797	0.103254085
TOP3B	356.0373286	0.648540772	0.220474194	2.941572256	0.003265506	0.103727853
APCDD1	226.6760803	0.919249169	0.31266862	2.940010957	0.003282006	0.104066467
FAM131A	718.1314833	0.280006949	0.095370044	2.936005234	0.003324687	0.105232548
NCAM2	333.1289896	0.351665377	0.119863081	2.933892352	0.003347403	0.105763685
H3P6	431.0406809	-0.672576547	0.22934107	-2.932647637	0.003360851	0.105809655
INPPL1	1766.849591	0.335102919	0.114291195	2.932009933	0.00336776	0.105809655
ZFPM2-AS1	91.47306651	-0.995426052	0.339512799	-2.931924962	0.003368681	0.105809655
HEATR5A	968.0536453	0.398964784	0.136132742	2.930704099	0.003381948	0.105809655
APH1A	1492.612972	-0.359826366	0.122787155	-2.930488665	0.003384293	0.105809655
SLC25A12	390.0530392	-0.461007836	0.15731526	-2.930471174	0.003384484	0.105809655
THY1	1497.685992	-0.506837883	0.173232028	-2.925774684	0.003435997	0.107231989
FLJ22447	10.52123629	-2.323026967	0.794832692	-2.922661575	0.003470535	0.107862312
LOC100507065	11.82900677	-2.46395244	0.843066641	-2.922606969	0.003471144	0.107862312
TPST1	496.9692497	0.687048903	0.235174954	2.921437394	0.003484203	0.107862312
HS3ST3B1	557.5689754	0.450774806	0.154335926	2.920738013	0.003492033	0.107862312
PLEKHM3	433.0120226	0.701511872	0.240232183	2.9201411	0.003498729	0.107862312
TUB	624.861844	0.623336917	0.213489739	2.919751175	0.00350311	0.107862312
DDIT4	1455.799523	-0.784141373	0.268569647	-2.919694696	0.003503745	0.107862312
PLEKHA1	684.413524	0.847412845	0.290247941	2.91961708	0.003504617	0.107862312
NAGLU	810.2596791	0.33361422	0.114387424	2.916528831	0.0035395	0.108748089
WDR45	289.3701463	-0.515417018	0.176849148	-2.914444443	0.003563222	0.109288503
SNAPC2	131.5699618	-0.667122504	0.22912752	-2.911577378	0.003596088	0.110107032
MID1	1223.528825	0.645741797	0.221906753	2.909969112	0.003614645	0.110467117
MCM5	1653.480431	-0.356948424	0.122684398	-2.909485077	0.003620247	0.110467117
MAP3K8	20.68810606	1.460826038	0.502231357	2.908671506	0.00362968	0.110565645
H2AZ1	5878.551362	-0.418888881	0.144055794	-2.907823897	0.003639532	0.110676562
ZNF217	1273.64042	-0.391551606	0.134699294	-2.906857158	0.003650799	0.110830038
PIK3C3	535.7753167	-0.401583288	0.138219657	-2.905399251	0.003667849	0.111158283
PCNX1	1997.085123	0.253943052	0.087421585	2.904809523	0.003674767	0.111178847
GBA2	665.5767477	0.433607519	0.149320187	2.903877421	0.003685724	0.111321369
NFIA	804.8146521	-0.4245257	0.146259208	-2.902557088	0.003701297	0.111602557
SPRY1	79.47203198	-1.020529776	0.351725237	-2.901497158	0.003713841	0.111791647
LOC101929829	2.64878008	4.503612516	1.552503125	2.900871789	0.003721261	0.11182609
PTAR1	1257.512734	0.381525385	0.131885322	2.892857065	0.00381755	0.114371475
ZADH2	1026.82459	0.36302849	0.125499361	2.892672031	0.003819799	0.114371475
SPRY4	172.8634843	-1.059458984	0.366355361	-2.891888851	0.003829334	0.114371475
SLC29A2	896.7329669	0.604378125	0.209004439	2.891699946	0.003831637	0.114371475
KYAT1	168.5161346	0.56407009	0.195199533	2.889710239	0.003855971	0.114905348
STRBP	213.284707	-0.529502927	0.183388226	-2.88733328	0.003885224	0.115583807
AHI1	592.661934	0.356346654	0.123568105	2.883807711	0.003928986	0.116690879
ADCY9	1056.881434	0.285757368	0.099111444	2.88319247	0.003936668	0.116724507
SPX	19.47732668	-1.386959874	0.48146659	-2.880698064	0.003967956	0.117280269
PIDD1	340.5600022	0.456588171	0.158564014	2.879519503	0.003982817	0.117280269
PLEKHA8	784.3174574	0.322548362	0.11201999	2.879382172	0.003984552	0.117280269
TRPV3	112.8907961	0.676955111	0.235126268	2.879113072	0.003987953	0.117280269
PTEN	2692.146959	-0.420613552	0.146112562	-2.878695348	0.003993239	0.117280269
TRPC6	3.629665463	-3.717295012	1.291371262	-2.878564144	0.0039949	0.117280269
PRIM1	644.9733214	-0.400270497	0.139259989	-2.874267755	0.004049655	0.118475914
EIF2S3	8355.471997	-0.322216691	0.1122047	-2.871686207	0.004082882	0.118475914
DHX40	1208.788068	-0.450876909	0.15700917	-2.871659724	0.004083224	0.118475914
IRS1	4010.517298	-0.268694692	0.093572001	-2.871528746	0.004084916	0.118475914
NIBAN1	265.5355598	-1.002787034	0.349231944	-2.87140696	0.004086491	0.118475914
EPS8	933.5141678	-0.426055017	0.148387586	-2.87123087	0.004088768	0.118475914
KANSL3	1977.626563	0.266973968	0.092992647	2.870914821	0.004092858	0.118475914
GOLGA4	652.3743653	0.441136926	0.153659173	2.870879207	0.004093319	0.118475914
CACNA2D4	35.19458495	-1.261641319	0.439614912	-2.869878348	0.004106297	0.118475914
AHR	989.283043	-1.048584876	0.365388123	-2.86978369	0.004107527	0.118475914
LIMD2	182.5908303	-0.593569294	0.206841003	-2.869688719	0.004108761	0.118475914
PCDHGB7	1094.986103	0.807039216	0.281385249	2.868093543	0.004129534	0.118637777
CCNG2	277.0839203	-0.473523432	0.165113347	-2.86786889	0.004132467	0.118637777
EPRS1	5918.541874	-0.403142651	0.140579256	-2.867725029	0.004134347	0.118637777
ADGRL4	100.2385788	-0.860813513	0.300758953	-2.862137622	0.004207941	0.120454157
CHD4	4991.576708	-0.261998462	0.091547196	-2.861895006	0.004211164	0.120454157
HNRNPA1	9542.761656	-0.185818057	0.064945619	-2.861133066	0.004221298	0.120550539
BLOC1S1	179.5551866	-0.560775845	0.196133536	-2.859153289	0.004247735	0.121111412
SOGA1	2220.677994	0.364590478	0.127544949	2.858525417	0.00425615	0.1211575
RETSAT	684.8748675	-0.320675827	0.112299396	-2.855543653	0.004296322	0.122013939
SPON2	36.15852746	1.632290387	0.571674957	2.855277053	0.00429993	0.122013939

FKBP14	2128.718726	0.346709541	0.121507368	2.853403443	0.004325367	0.122515931
AMMECR1	705.1077652	-0.397066131	0.139208692	-2.852308461	0.004340296	0.122515931
LINC02028	3.148936958	4.263042534	1.495096053	2.851350269	0.004353398	0.122515931
P4HA2	5635.027243	0.915945473	0.321260283	2.851100876	0.004356815	0.122515931
FPGS	764.2773907	0.28342169	0.09941193	2.850982665	0.004358435	0.122515931
LTBP4	548.4018118	-0.444613982	0.155952881	-2.850950749	0.004358872	0.122515931
MEST	1577.546548	-0.603044505	0.211760747	-2.847763398	0.004402764	0.123554742
USP32P3	23.85256454	1.323705053	0.465715853	2.8423019	0.004478906	0.125493865
FER1L4	254.9419183	0.985013431	0.34674153	2.840771432	0.004500456	0.125718739
ATP5F1D	616.8417829	-0.439708122	0.154787021	-2.84072993	0.004501041	0.125718739
UHRF1BP1L	2050.325183	0.420915982	0.148203312	2.840125331	0.004509581	0.125760153
BMF	544.3018504	1.60779012	0.56643801	2.838422021	0.00453372	0.126235754
ASF1B	540.7977675	-0.430517391	0.151725105	-2.837482904	0.004547078	0.126410192
RPA3	486.5649564	-0.336990765	0.118788914	-2.836887329	0.004555569	0.126448958
FLOT2	781.3359465	-0.407904366	0.143817702	-2.836259794	0.00456453	0.12650066
YPEL5	729.5760629	-0.544067836	0.191920623	-2.834858633	0.004584597	0.126859495
PRELID2	308.1287249	0.479833604	0.169297675	2.834259858	0.004593197	0.126900406
PLPP3	280.6404713	-0.572360148	0.202094067	-2.832147216	0.004623656	0.127486485
H2AC17	1739.330524	-0.542046256	0.191414214	-2.831797304	0.004628718	0.127486485
DGKD	835.2265609	0.307393404	0.108597787	2.830567855	0.004646545	0.127779998
ADAMTS6	287.900993	0.453207358	0.160190259	2.829181749	0.004666719	0.128137026
FN1	286811.2716	-0.715598117	0.25297981	-2.828676791	0.004674088	0.128141912
HTR7	79.36817899	-0.696950995	0.246534087	-2.82699647	0.004698685	0.12861837
LINC00607	609.7182131	-0.886139133	0.313539022	-2.826248319	0.004709674	0.128721453
TMEM200B	94.64697367	-0.653722436	0.231399177	-2.825085398	0.004726802	0.128991738
AP1S2	754.2818569	-0.340986726	0.120762192	-2.823621543	0.004748442	0.129384148
KIF3A	454.175978	0.388316519	0.137596364	2.822142306	0.0047704	0.12970075
TARS1	2298.96427	-0.315586456	0.11183637	-2.821858898	0.004774618	0.12970075
LINC01050	49.5485124	-1.448095393	0.513524124	-2.819916972	0.004803608	0.130151187
GPI	3760.451777	-0.290703363	0.103094705	-2.819770077	0.004805807	0.130151187
MLXIP	2063.918391	0.508125019	0.180254118	2.818937086	0.004818296	0.130291406
FAM107B	226.8682862	-0.495160852	0.175922167	-2.814658663	0.004882907	0.131838481
GLIS1	123.2183918	0.632721361	0.22485554	2.813901592	0.004894421	0.131949441
MYO5A	1478.286351	0.301239592	0.107111033	2.812404896	0.004917256	0.132364813
ARHGEF19	195.507321	0.897845439	0.319658057	2.808768365	0.004973141	0.133640475
RAP2C	860.5265942	-0.367988873	0.131045213	-2.808106178	0.004983379	0.133640475
DLX6-AS1	63.76910486	0.962439865	0.342765969	2.807862952	0.004987145	0.133640475
DUSP9	39.61543978	1.042777487	0.371483879	2.80705986	0.004999595	0.133772948
CLMAT3	106.4096463	0.926796705	0.330357881	2.805432409	0.005024912	0.134248771
TPM1-AS	670.9640049	0.770043801	0.274600814	2.804229858	0.005043693	0.134548826
PRDX2	153.6221299	-0.70758028	0.252490536	-2.802403176	0.005072344	0.135110866
IL20RB	16.84531316	-1.405406577	0.501981928	-2.799715487	0.005114767	0.136037523
PCDHGC5	139.6326437	1.199060398	0.42874468	2.796677028	0.005163112	0.137118704
SLITRK5	627.9534943	0.413354236	0.147940671	2.794054092	0.005205177	0.138030143
TTF2	1061.544313	0.387990633	0.13909384	2.789416368	0.005280313	0.139648436
NARS1	3267.746256	-0.437094414	0.15670283	-2.789320486	0.005281877	0.139648436
OLFML2B	1450.456878	-0.835294366	0.30004019	-2.783941595	0.00537027	0.141574283
CYP26B1	8.762906661	2.484376276	0.892401774	2.783921266	0.005370607	0.141574283
CYP26A1	3.843477698	5.143832972	1.849232613	2.781604076	0.005409098	0.142378336
PCBD1	133.9746553	-0.587305468	0.211413076	-2.777999734	0.005469466	0.143754999
BMP5	77.4786944	1.197645413	0.431522263	2.775396583	0.005513443	0.144697431
ADD1	1814.395028	-0.281680133	0.101569616	-2.773271629	0.005549578	0.14543158
CHST6	233.5443848	0.553346289	0.19977236	2.769884125	0.005607624	0.146736941
NT5DC3	1263.073895	0.271874613	0.098209932	2.768300579	0.005634946	0.147235683
ZIC5	311.6222704	0.562961089	0.203438347	2.767231929	0.005653452	0.147502948
AP2A2	1517.721479	-0.233921474	0.084559003	-2.766369839	0.005668421	0.147532839
FKBP1A	2511.255174	-0.308236081	0.11143717	-2.766007805	0.005674718	0.147532839
ATP2A1-AS1	14.60110487	1.532767818	0.554198722	2.765736833	0.005679435	0.147532839
DPAGT1	438.2857028	0.347926289	0.125824511	2.765171005	0.005689296	0.147573884
EML4	3473.08123	-0.33382228	0.120789716	-2.763664742	0.005715623	0.147820418
SRGAP2D	154.9929646	0.787644853	0.285064717	2.763038726	0.005726597	0.147820418
LOC101927854	4.014171916	4.044247951	1.463801638	2.762838793	0.005730106	0.147820418
MYO1B	3108.869309	-0.346894428	0.1255621	-2.762731973	0.005731981	0.147820418
FAM53B	730.3142341	0.412827172	0.149482005	2.761718181	0.005749809	0.148065878
SLC25A32	3070.287509	0.324453336	0.117571967	2.759614767	0.005786956	0.148617972
INSC	220.2332162	2.054697614	0.744574388	2.759559888	0.005787928	0.148617972
PCDH7	98.57581724	-0.65658279	0.238010311	-2.758631701	0.005804391	0.148665143
PPP3CC	299.7774809	0.460505526	0.166939601	2.758515788	0.00580645	0.148665143
TSPAN18	2521.272895	1.311768515	0.475837267	2.756758679	0.005837742	0.149251876
LINC01411	11.49203961	3.405325722	1.23678901	2.75336027	0.005898694	0.150476361
S100A10	2208.231057	-0.435704688	0.158256918	-2.753147816	0.005902524	0.150476361
PCBP2	9121.005679	-0.402272069	0.146148192	-2.752494327	0.005914317	0.150561616
AGO1	1468.703325	-0.270815679	0.098437427	-2.751145455	0.005938727	0.150813563
FGFRL1	310.7835048	0.439213643	0.159667719	2.750798013	0.005945029	0.150813563
HBP1	599.4999913	-0.559482586	0.203410874	-2.750504802	0.005950352	0.150813563
H2BC8	313.1164133	-0.684986191	0.249078617	-2.750080273	0.005958067	0.150813563
EFL1	617.494277	0.61634656	0.224180074	2.749336945	0.005971597	0.150817043
MFSD2A	81.01786018	0.645128284	0.234665232	2.749143018	0.005975131	0.150817043
ARHGAP32	751.0208632	0.370019333	0.134674768	2.747503026	0.006005095	0.151358983
KLHL13	240.3599835	0.499883675	0.182066992	2.745602977	0.006039981	0.152023247
ENTPD7	848.337753	0.396230056	0.144398916	2.743996061	0.006069627	0.152553944
CAPG	652.2981902	-0.873332031	0.318324087	-2.743531098	0.006078229	0.152554989
EPHA7	566.4463113	-0.624825227	0.227827214	-2.742539903	0.006096604	0.152800966
RAVER1	1283.074598	0.259701665	0.094774642	2.740202021	0.006140143	0.153676052
ZNF708	129.5064533	0.637694964	0.233140583	2.735237924	0.00623352	0.155794292
PIAS1	743.6214975	0.323492556	0.118304626	2.734403271	0.006249345	0.155967653
ZNF385D	66.30643956	-1.067242256	0.390371066	-2.733917412	0.006258574	0.155967653
BBOX1	18.71485903	1.573256369	0.575627963	2.733113174	0.006273877	0.155967653
TMTC3	1788.770745	0.236977986	0.086711715	2.732940831	0.006277161	0.155967653
VPS26A	557.7013786	-0.315305554	0.115413447	-2.731965486	0.006295774	0.155967653

TUBB	13666.05356	0.318094265	0.116449617	2.731604227	0.00630268	0.155967653
TRAF5	926.1492484	0.262377156	0.09606604	2.731216518	0.0063101	0.155967653
MGP	95.82892141	2.253000545	0.825049604	2.730745562	0.006319124	0.155967653
KAT6B	491.6346123	-0.318910769	0.11678547	-2.730740114	0.006319228	0.155967653
TFRC	13443.19377	0.558745552	0.204652974	2.730209787	0.006329403	0.156002722
ANXA6	4447.617996	-0.274053555	0.100493242	-2.727084439	0.006389669	0.157270571
GNAS	6321.963079	-0.453090864	0.166236036	-2.725587517	0.006418716	0.157767606
BMP6	370.4714704	0.750643448	0.275794803	2.721746162	0.0064938	0.159393275
ITGA7	89.31615104	-0.708826925	0.26053424	-2.720667055	0.006515034	0.159669698
CREG1	544.071306	-0.322113138	0.118412456	-2.720263975	0.006522982	0.159669698
LINC02614	24.3532505	1.190693405	0.437950068	2.718788034	0.006552158	0.160163865
SLC38A2	5060.714078	-0.522326426	0.192208959	-2.717492618	0.006577862	0.160571925
ARIH1	2224.557971	-0.314714637	0.115847214	-2.716635351	0.006594922	0.160606302
UROD	475.3531406	-0.369769187	0.136118895	-2.716516237	0.006597296	0.160606302
MEIS3P1	255.6681883	-0.468510596	0.172588556	-2.714609863	0.006635389	0.16118242
CDK4	1934.439941	-0.317134077	0.116842244	-2.71420734	0.006643457	0.16118242
VIM	51302.93921	-0.550886831	0.202981461	-2.713976089	0.006648096	0.16118242
PLEKHO2	242.1862305	-0.380471562	0.140268952	-2.712443174	0.006678923	0.161709798
RALGPS2	704.6561652	0.526719201	0.194275438	2.711198112	0.006704056	0.1617885
MRPL30	1667.810956	-0.271914556	0.100300494	-2.710999164	0.00670808	0.1617885
RANBP9	1132.242207	-0.306694517	0.113132444	-2.710933363	0.006709411	0.1617885
COL9A2	593.9313424	0.920387079	0.33986651	2.708084064	0.006767288	0.162963608
THSD7A	73.52060191	1.00622563	0.372054219	2.704513429	0.00684045	0.164401731
PLAC1	58.57106021	-1.188412679	0.439457811	-2.704270236	0.006845459	0.164401731
KIAA1217	198.1479524	-0.558986612	0.206778193	-2.7033151	0.006865163	0.164653038
MIR4292	25.08256293	1.214000462	0.449407323	2.701336624	0.00690614	0.165413194
PHEX	16.46436401	-1.599006284	0.59217338	-2.700233307	0.006929087	0.165677771
CLCN4	444.745731	0.54006645	0.200031127	2.699912044	0.006935781	0.165677771
THBS4	23.52933283	-1.195563374	0.442947588	-2.699107988	0.006952561	0.165856279
ADGRL2	1204.484255	0.326368481	0.121010097	2.697035113	0.006995989	0.166291799
EIF2S3B	45.62656248	-1.371403183	0.508524329	-2.696829052	0.00700032	0.166291799
COPS2	2240.408456	-0.343962174	0.127548403	-2.696718784	0.007002638	0.166291799
CFAP54	24.80509861	-1.006057391	0.373103445	-2.696456985	0.007008145	0.166291799
KLRC2	17.77558281	-1.837188195	0.681612621	-2.695355306	0.007031361	0.166620813
ANKRD11	1201.381305	-0.423863582	0.157357723	-2.693630624	0.007067844	0.167262932
SEMA4C	1684.312973	0.494716331	0.183901081	2.690121926	0.007142592	0.168807672
CALCOCO2	708.9607858	-0.417987788	0.155441503	-2.689035942	0.00716587	0.169116839
KATNBL1	598.6181702	0.334640307	0.124485076	2.688196198	0.007183917	0.169116839
TRIL	14.96997886	-1.672950914	0.622334588	-2.688185659	0.007184144	0.169116839
BOC	182.9690664	-0.489967505	0.182453063	-2.685444113	0.007243349	0.170193966
AGBL3	85.2285291	0.740682276	0.275863987	2.684954585	0.007253966	0.170193966
MIIP	307.1207985	0.350996035	0.130737279	2.684743307	0.007258553	0.170193966
H2AC4	358.9226143	-0.580080784	0.21626267	-2.68229734	0.007311844	0.17121821
POC1A	180.5774101	-0.446160924	0.166394146	-2.681349882	0.007332581	0.171407812
PRDX1	2806.592916	-0.301719956	0.112538027	-2.681048925	0.007339178	0.171407812
WNT10B	317.7077265	0.34471844	0.128611424	2.680309636	0.007355409	0.171562021
KCTD15	892.3418966	-0.386517633	0.144344104	-2.677751446	0.00741182	0.1726518
F3	376.5207594	-0.770908099	0.288216959	-2.674749267	0.007478516	0.173978005
ACAN	78.49401559	2.335013366	0.873781363	2.672308503	0.007533136	0.17502018
BACH2	155.185147	-0.700301533	0.262175024	-2.671122227	0.007559811	0.175411248
HPSE	225.6380845	0.453875009	0.169951829	2.670609731	0.007571362	0.175450807
TSHZ3	14.11452132	-1.430997119	0.536017198	-2.669685084	0.007592242	0.175528671
EPHA6	89.62256474	0.789494483	0.29573638	2.669588648	0.007594422	0.175528671
ANO8	252.6971899	0.421957351	0.158178129	2.66760868	0.007639318	0.176337619
SDC3	728.8232052	-0.369629638	0.138601935	-2.666843275	0.007656737	0.176511061
GOLGA8N	119.3188272	0.739211126	0.277337251	2.665387077	0.007689975	0.177033843
HS3ST3A1	403.0641183	0.393930158	0.147817287	2.664980299	0.007699283	0.177033843
CLVS2	94.69705902	0.628504309	0.23601394	2.662996559	0.007744821	0.177751114
PLEKHG3	1482.173703	0.725378309	0.272500367	2.661935159	0.007769284	0.177751114
NEFL	764.3128026	-0.487205696	0.183036088	-2.661801292	0.007772375	0.177751114
ISM1	38.21376439	-1.062909362	0.399357374	-2.66154936	0.007778194	0.177751114
CASK	919.2687054	-0.707894354	0.265980112	-2.661455962	0.007780352	0.177751114
TMX4	1184.258715	-0.347368765	0.130617719	-2.659430653	0.007827284	0.178594371
SRRM2	8839.649887	0.458768836	0.172563464	2.658551385	0.007847738	0.178604927
PRDM6	229.3412803	0.487124257	0.183229365	2.658549066	0.007847792	0.178604927
CALCA	4.063669347	4.080794331	1.536837131	2.655319974	0.007923321	0.180093858
WSB1	1857.747137	0.500811262	0.188708188	2.653892594	0.007956915	0.180450034
CARMN	128.7453564	0.89894236	0.338738581	2.653793837	0.007959244	0.180450034
LARS1	4734.020667	-0.269177454	0.101460211	-2.653034642	0.007977168	0.180552287
OGFRL1	564.1404282	-0.429052561	0.161739094	-2.652744923	0.007984018	0.180552287
CLIP1	612.1831133	0.354770148	0.13378256	2.651841516	0.008005411	0.180806617
GFRA2	6.766163118	-3.240394228	1.223823832	-2.647761993	0.008102655	0.182651888
ACP1	1191.742638	-0.297322874	0.112300915	-2.647555214	0.008107612	0.182651888
SMG1P7	100.691	0.811183751	0.306470707	2.646855746	0.0081244	0.182799004
HOMER1	389.132069	0.316368607	0.11954736	2.646387236	0.008135662	0.182800262
PMAIP1	376.1919584	-0.561242123	0.212109615	-2.646000385	0.008144972	0.182800262
NOTUM	41.21383618	2.232272996	0.844548456	2.643155618	0.008213727	0.184111476
MIR12136	239.6148212	1.13152727	0.428269719	2.642090299	0.008239608	0.184459577
KIF21A	463.2312317	0.402899911	0.15254721	2.641149001	0.008262537	0.184740795
CREB3L1	1031.523093	0.885941146	0.335566331	2.640137176	0.008287248	0.184970377
SLC25A37	1823.402885	0.356802228	0.13516891	2.639676747	0.008298514	0.184970377
GALNT10	2568.573987	0.763429153	0.289237421	2.639454983	0.008303945	0.184970377
WNT5B	540.3821169	-0.53507776	0.202770142	-2.638839008	0.008319048	0.185075443
LTBP1	1587.906155	-0.673079925	0.255167745	-2.637793912	0.008344728	0.185141556
SPICE1	314.4746119	0.50907143	0.193022008	2.637375059	0.00835504	0.185141556
PNCK	5.332624893	3.437128085	1.303492592	2.636860468	0.008367724	0.185141556
NONO	3869.018038	-0.223031669	0.084596103	-2.636429593	0.008378359	0.185141556
DLD	1547.450792	-0.336608579	0.127680792	-2.636329035	0.008380842	0.185141556
IGHMBP2	375.4623002	0.394134857	0.149509458	2.636186778	0.008384357	0.185141556
LZTFL1	189.5663425	-0.559430958	0.212449543	-2.633241528	0.008457418	0.18652375

OTUD7B	1246.389275	0.492236333	0.187055643	2.631496836	0.008500966	0.187252425
ANKRD61	59.35908303	1.271330569	0.483563746	2.629085783	0.008561476	0.18832485
SLC17A9	905.5437617	0.597482027	0.227336828	2.628179662	0.008584316	0.18832485
SOX11	21.80898367	-1.145795387	0.43605443	-2.62764304	0.008597869	0.18832485
AXL	1730.06106	-0.617767378	0.235115333	-2.627507831	0.008601286	0.18832485
HOXB8	41.47706364	-1.248560626	0.475220578	-2.627328623	0.008605818	0.18832485
HNMT	113.3998173	-0.508266156	0.193474671	-2.627042353	0.008613061	0.18832485
H3C10	902.3642591	-0.572835246	0.218254112	-2.624625222	0.008674439	0.189434438
KCNJ2	71.54834464	0.99575779	0.380182175	2.619159592	0.00881467	0.19226122
NANP	471.679797	0.341211578	0.130361248	2.617431042	0.008859439	0.192809015
PDK3	395.8153378	-0.40338681	0.154143254	-2.616960513	0.00887166	0.192809015
VASP	706.1420397	-0.382714969	0.14624533	-2.616938049	0.008872244	0.192809015
AKR1B1	1143.905613	-0.337624257	0.129090967	-2.615398005	0.008912354	0.19344475
PTGFR	385.7532271	-0.52119719	0.199399501	-2.613833971	0.008953253	0.194096075
KHDC1	114.4835455	0.588246235	0.225121627	2.613015203	0.008974731	0.194325281
MILR1	21.16836301	-1.086701841	0.416194991	-2.611040174	0.009026729	0.195148485
ST8SIA5	20.11419439	-1.516875787	0.581077596	-2.610453057	0.009042238	0.195148485
LINC02454	5.37737427	-2.67818967	1.025998242	-2.610325787	0.009045603	0.195148485
NECTIN4	34.26597756	2.353137251	0.90193754	2.608980276	0.009081248	0.195680572
COPS6	1004.391739	-0.396216581	0.152051596	-2.605803499	0.009165903	0.197159828
CASC15	32.42958038	1.176805575	0.451686535	2.605358992	0.009177804	0.197159828
UNC79	11.69854807	1.798933724	0.690526691	2.605161751	0.00918309	0.197159828
NDST4	33.65643634	-1.191738883	0.457696037	-2.603778022	0.009220245	0.197719338
ASXL2	1317.771301	-0.245030679	0.094251007	-2.599767231	0.009328701	0.199796111
ZDHHC15	4.077181216	-2.964014754	1.140282144	-2.599369612	0.009339515	0.199796111
TBKBP1	359.8841995	0.375943842	0.14471839	2.597761359	0.009383368	0.200493549
ZNF684	52.58973927	0.755121268	0.290784304	2.596843289	0.009408484	0.200789439
LOC100288203	108.9276676	0.568091217	0.218961653	2.594478112	0.009473465	0.201850511
UBR7	1157.56185	-0.298214275	0.114953794	-2.594209946	0.009480857	0.201850511
LINC02060	3.016209601	4.321356215	1.667168088	2.592033908	0.009541037	0.202889349
TATDN2	1230.888345	0.263892151	0.101828997	2.591522639	0.009555225	0.202939196
ZNF512	301.1105861	-0.478622284	0.184715717	-2.591129185	0.009566157	0.202939196
SUMO1	1023.92342	-0.367591785	0.141904299	-2.590420349	0.00958588	0.203115797
AP2A1	1489.656845	-0.308939269	0.119361032	-2.588275778	0.009645772	0.204052771
DECR1	631.8111303	-0.2999408	0.115895961	-2.58801772	0.009653001	0.204052771
FRMD4A	298.652629	-0.356931161	0.138073749	-2.585076198	0.009735748	0.205408091
ROR1	269.1299885	0.435986558	0.168665423	2.584919611	0.00974017	0.205408091
HS6ST2	704.1071789	-0.586137008	0.226796695	-2.584415999	0.009754406	0.205465152
COL7A1	515.828824	-0.544472349	0.210725447	-2.583799714	0.009771852	0.205589619
FYCO1	842.3969133	-0.591117616	0.228826403	-2.583257908	0.009787213	0.205669968
PRDM10	264.9130352	0.403298896	0.156204881	2.581858478	0.009826987	0.206262561
KLF10	1560.979447	-0.473397433	0.183445387	-2.580590547	0.009863149	0.206778012
NFATC3	836.720285	0.327785611	0.127108615	2.578783597	0.009914888	0.207618448
GBE1	843.6994672	-0.27710326	0.107514235	-2.577363459	0.009955721	0.208228804
HAUS1	244.6935524	-0.363227996	0.141177842	-2.572839984	0.010086783	0.210722714
LOC646903	40.72358523	0.994939507	0.386810443	2.572162993	0.01010653	0.210818662
CCSAP	336.1430403	0.342533376	0.133184472	2.571871703	0.010115037	0.210818662
ABHD10	393.2809146	0.336241681	0.130774175	2.571162699	0.01013577	0.21100399
NHS	183.6876719	0.494118506	0.19245183	2.567491851	0.01024372	0.213002433
CCDC152	218.8703233	-0.405793016	0.158097439	-2.566727328	0.010266331	0.213013185
C12orf56	31.68698424	-0.930818979	0.362656828	-2.566666082	0.010268144	0.213013185
A1BG-AS1	95.29381641	0.699810917	0.272849937	2.564819788	0.01032294	0.213620045
FOXP4	554.6364918	-0.376833277	0.146953663	-2.564299986	0.010338415	0.213620045
PRKX	801.8872549	0.285259326	0.111245607	2.564230025	0.010340499	0.213620045
CEP85L	454.4978435	0.340475326	0.132787203	2.564067313	0.010345348	0.213620045
FEZ1	18.97266973	-1.395774049	0.54451766	-2.563321913	0.010367587	0.213831487
PRRT2	95.00476461	0.805686998	0.314610896	2.560899858	0.010440144	0.215051862
HYAL2	338.3183274	0.43799346	0.171054957	2.560542335	0.010450893	0.215051862
FGF10	12.33978339	-1.656089062	0.647251728	-2.558647571	0.010508021	0.215885693
MIER2	431.3336931	0.321756823	0.125765087	2.558395424	0.010515644	0.215885693
DPY19L3	929.9278162	0.255795945	0.100166975	2.55369543	0.010658645	0.218569672
SLC3A2	1611.156383	-0.539071181	0.211143018	-2.5531092	0.010676602	0.218686255
BPHL	480.6595142	-0.390898523	0.153193994	-2.551656978	0.010721201	0.219347658
KTN1	2412.875927	0.224274619	0.087956304	2.549841336	0.010777195	0.220240392
LOC101928034	6.614049598	-1.887550061	0.740416646	-2.549307975	0.010793693	0.220311932
PAIP2	1100.544754	-0.33094434	0.12984747	-2.548716129	0.010812027	0.220311932
ST8SIA6	86.10200525	-0.579879197	0.227534738	-2.548530405	0.010817786	0.220311932
TANC2	2275.786691	0.3974948	0.156021463	2.547693072	0.010843783	0.220454997
BAMBI	86.0390286	0.740203512	0.290559898	2.547507475	0.010849553	0.220454997
ANKRD33B	226.2090095	-0.469325777	0.184294535	-2.546607127	0.010877582	0.220772789
TMEFF2	14.693007	-2.038177667	0.801149947	-2.544065159	0.010957064	0.221454337
SCAT8	8.787711516	-1.930802216	0.759005649	-2.543857504	0.01096358	0.221454337
WEE1	1013.660191	-0.297662027	0.11702541	-2.543567471	0.010972686	0.221454337
ARPC1B	897.2330022	-0.33120879	0.130218328	-2.543488263	0.010975174	0.221454337
APOBEC3C	196.5722036	-0.578064367	0.227282899	-2.54336938	0.01097891	0.221454337
MYO18A	1292.671059	0.414780379	0.163096933	2.54315254	0.010985726	0.221454337
PHC2	1119.503212	-0.375850494	0.147850795	-2.542093151	0.01101908	0.221495332
SPAG4	130.8429546	0.734678596	0.289016182	2.541998136	0.011022076	0.221495332
LOC101928389	4.655342301	4.32546952	1.701665244	2.541903899	0.011025048	0.221495332
RPRM	17.60970615	1.259879066	0.495746114	2.541379612	0.011041596	0.221577982
AGTR1	393.9007223	-0.686361628	0.27014104	-2.540752891	0.011061407	0.221725835
GPD2	1247.292956	0.407395379	0.160369895	2.540348232	0.011074215	0.221733149
GATA6	217.7265523	-0.449299013	0.176947589	-2.539164367	0.011111761	0.222235211
CHST2	324.2799543	-0.425935183	0.167903024	-2.536792806	0.011187314	0.223495451
STARD4-AS1	223.2059093	0.799820728	0.31543421	2.535618215	0.011224904	0.223771481
LOC100996724	73.47816506	0.616292829	0.243058283	2.535576329	0.011226246	0.223771481
PRKACA	778.9284965	-0.29869033	0.117842121	-2.534665248	0.011255483	0.224103576
THRA	746.788229	-0.584649716	0.230748364	-2.533711205	0.01128617	0.22446379
SWAP70	1071.710054	-0.316996022	0.125146759	-2.53299426	0.01130928	0.224672663
ZNF699	293.6609452	0.338401818	0.133620469	2.532559719	0.011323308	0.224700833

CD276	2189.907383	-0.506087764	0.199915894	-2.531503391	0.011357472	0.225128087
VPS37A	691.5540802	0.334273012	0.132122209	2.530028943	0.011405312	0.225825174
EPDR1	1862.905119	0.356715472	0.141083934	2.528391871	0.011458638	0.226394082
SNORD134	30.11079667	1.559397245	0.616792359	2.528236969	0.011463695	0.226394082
SLC7A5	3856.887198	-0.745923658	0.295107441	-2.527634196	0.011483393	0.226394082
PHTF2	613.662691	0.334512602	0.132344526	2.527589262	0.011484863	0.226394082
UBE2E3	1309.191544	-0.358155038	0.141750279	-2.526661972	0.011515228	0.22674183
SLC34A3	66.89737105	0.857912888	0.339880593	2.524159676	0.011597524	0.227916103
CNIH1	2929.308477	-0.329602537	0.130583693	-2.524071184	0.011600444	0.227916103
LINC00977	123.9803323	-0.925271755	0.366654204	-2.523554192	0.011617515	0.227993605
PITPNA	715.0358586	-0.314456056	0.124641273	-2.522888669	0.011639524	0.227993605
ATF6B	1258.472272	-0.309965083	0.122865958	-2.522790591	0.011642771	0.227993605
SOX13	326.7102832	0.408009486	0.161773062	2.522110169	0.011665317	0.228184352
LOC105373878	4.533815757	-2.836449368	1.124863453	-2.521594387	0.011682433	0.228268591
PNMA8A	5.83650463	-2.329201602	0.924172912	-2.520309318	0.011725175	0.228852808
PDZD8	1292.436231	0.386529601	0.153396777	2.519802615	0.011742066	0.228931745
FBLN5	257.511706	-0.523396873	0.207776138	-2.519042263	0.011767453	0.229175973
RBM4B	151.2283975	-0.515183494	0.204616721	-2.51779762	0.011809115	0.229736282
GLIPR2	106.3761768	-0.551947963	0.219295782	-2.516910989	0.011838873	0.230063149
CYGB	89.21983663	-1.552418576	0.616926668	-2.516374565	0.01185691	0.230063149
ST6GALNAC3	128.6525734	0.633414858	0.251740242	2.51614463	0.011864648	0.230063149
MLEC	8451.97865	0.277989466	0.110500785	2.5157239	0.01187882	0.230087577
KDM4A	1243.516659	-0.352861594	0.140298685	-2.515074138	0.011900736	0.230261788
MTURN	366.7930654	-0.69421203	0.276227061	-2.513193413	0.011964372	0.231096772
CTDSPL	320.9092454	-0.369011044	0.146878955	-2.512347974	0.011993077	0.231096772
MATN1-AS1	44.11192816	0.823622987	0.327830931	2.512340685	0.011993324	0.231096772
MPC1	161.1435883	0.452623616	0.180165273	2.512268915	0.011995764	0.231096772
ADAMTS7P1	2.038255888	4.122994916	1.642513455	2.510174211	0.012067161	0.232221182
MCM7	2493.048243	-0.341697515	0.136159165	-2.509544729	0.01208869	0.232384535
CD109	4712.069088	-0.477006705	0.190143413	-2.508668043	0.012118731	0.232523513
TGIF1	278.5062939	-0.386865392	0.154233002	-2.508317856	0.012130749	0.232523513
AGPAT2	87.89627795	-0.584208754	0.232920254	-2.508192158	0.012135066	0.232523513
STRA6	144.2939175	0.835883964	0.333323543	2.507725547	0.012151101	0.232580679
SERPINE1	89.4576001	1.220004545	0.486889378	2.505711975	0.012220512	0.233658292
TOP1	922.792555	-0.449821661	0.179603553	-2.504525397	0.01226158	0.233974994
KIAA1958	299.4704467	0.533435904	0.212993125	2.50447475	0.012263336	0.233974994
SMAGP	202.6816553	-0.46552891	0.185924322	-2.503862355	0.012284582	0.234129672
ACP3	22.53716923	-1.194201848	0.477268703	-2.502158303	0.012343872	0.235008326
CDR2	553.9391974	0.539541189	0.215700856	2.501340047	0.012372432	0.235273122
WRN	1707.459195	0.261230407	0.104450225	2.50100378	0.012384186	0.235273122
KIAA1143	341.7168038	-0.373351629	0.149327364	-2.500222461	0.012411534	0.235315954
ACOT2	303.4767267	-0.334367395	0.133749662	-2.499949451	0.012421103	0.235315954
JAGN1	362.2514289	-0.356559004	0.142634544	-2.499808211	0.012426056	0.235315954
CDK14	387.9238499	-0.374090784	0.149673615	-2.499376962	0.012441189	0.235352434
AOC3	24.81625196	1.660436502	0.664703901	2.498009263	0.012489293	0.235685414
SESN3	3346.642427	0.75893167	0.303827575	2.497902535	0.012493054	0.235685414
TRAK1	456.8067538	0.383745554	0.153636562	2.497748902	0.012498469	0.235685414
RAB35	877.0690265	-0.279463346	0.111938197	-2.496586087	0.012539523	0.236209615
YTHDF2	1567.231361	-0.299825128	0.120147832	-2.495468485	0.012579093	0.236704789
ZBTB25	1155.447224	0.35143158	0.140933718	2.493594749	0.012645683	0.237706824
ASAP2	624.5860733	0.475813963	0.190980255	2.491430131	0.012722999	0.238808108
JAM3	951.4343692	-0.243561131	0.09776841	-2.491204779	0.012731072	0.238808108
GTF2H3	1643.606542	-0.325586957	0.130745776	-2.490229261	0.012766072	0.239212827
SIK3	617.3051241	-0.336843284	0.135364718	-2.488412704	0.012831473	0.240147075
AP1B1	1153.168585	-0.243447329	0.097844804	-2.488096658	0.012842882	0.240147075
PCDH20	112.058691	0.619982973	0.249243578	2.487458168	0.012865958	0.240326392
RALGDS	588.9765625	0.294823342	0.118556184	2.486781646	0.012890449	0.240531729
MINAR1	210.8732251	0.599858696	0.241431979	2.484586748	0.01297019	0.241766518
LINC01139	72.71871704	-0.612759244	0.246713958	-2.483682919	0.013003153	0.241803066
SYNGR2	62.87553294	-0.642880179	0.258852067	-2.483581404	0.01300686	0.241803066
NYAP1	23.39174489	1.309633784	0.527351501	2.483417191	0.013012859	0.241803066
GCH1	116.8474125	0.522056397	0.210512517	2.479930433	0.013140802	0.243171829
DZIP1	1261.483511	0.315004993	0.127053533	2.479309198	0.013163714	0.243171829
ERG	425.0228479	0.334952171	0.135104679	2.479204826	0.013167567	0.243171829
CEP250	642.1553947	0.27582897	0.111291916	2.478427728	0.013196284	0.243171829
CRLF3	464.467413	0.294687767	0.11890204	2.478408011	0.013197014	0.243171829
LMNTD2-AS1	3.776693785	-2.925017682	1.180229453	-2.478346624	0.013199285	0.243171829
HADHA	3556.55036	-0.300149408	0.121132142	-2.477867578	0.013217019	0.243171829
ALPL	1121.894175	1.985513504	0.801399225	2.477558552	0.01322847	0.243171829
YWHAB	2774.140031	-0.33070035	0.133482166	-2.477487151	0.013231117	0.243171829
SEMA3A	1643.056044	-0.328164422	0.132459367	-2.477472369	0.013231665	0.243171829
IMPAD1	7187.536185	0.236057255	0.095286633	2.477338604	0.013236626	0.243171829
NEK4	431.8811387	-0.272329164	0.109998479	-2.475753911	0.013295518	0.244002203
RGCC	1242.92137	1.697029517	0.685693592	2.4749094	0.013326998	0.244252518
GLUD1	2306.781764	-0.367099382	0.148343785	-2.47465292	0.013336571	0.244252518
MAST4	185.3491098	-0.683766125	0.276424325	-2.473610547	0.013375542	0.244714734
THEM4	221.7282256	-0.520338493	0.210407013	-2.473009268	0.013398067	0.244875437
KIFBP	407.6441447	-0.309556625	0.125198083	-2.472534865	0.013415863	0.24494946
SNRPD3	811.2542085	-0.298506281	0.120773795	-2.471614646	0.013450442	0.245329446
CARNS1	37.94620322	1.047277442	0.423894492	2.470608753	0.01348833	0.245768956
LNPK	1982.979347	0.313286156	0.126851832	2.469701478	0.013522585	0.246141426
ESPNP	3.145084436	3.795673793	1.537514659	2.468707384	0.013560205	0.246574345
LMNA	9665.518747	-0.411886022	0.166898656	-2.467880998	0.01359155	0.24685757
LOC100129617	16.5834113	1.54498772	0.626117911	2.467566719	0.013603487	0.24685757
KCTD13	151.4392319	0.451098551	0.18284402	2.467122251	0.013620385	0.246912773
SP4	388.4786675	0.305632223	0.12399139	2.464947135	0.013703347	0.247255508
SHC2	2.277960077	-4.032104187	1.635785131	-2.464935101	0.013703807	0.247255508
LRRC8D	740.0984415	-0.277701489	0.112665416	-2.464833464	0.013707695	0.247255508
RASSF7	27.56822878	-0.938304081	0.380693193	-2.464725135	0.013711839	0.247255508
FREM1	21.12366275	-1.199471533	0.486759092	-2.464199542	0.013731964	0.247255508

TRIM14	445.6487893	-0.405697833	0.16466811	-2.463730431	0.013749948	0.247255508
DLG3	123.7751421	-0.512169328	0.207919632	-2.46330432	0.013766302	0.247255508
NT5C3B	433.4925788	-0.327733998	0.1330703	-2.462863595	0.013783234	0.247255508
OLFM1	3.903164183	-3.214760961	1.305378843	-2.462703435	0.013789392	0.247255508
CEP290	696.0444713	0.441300198	0.179195702	2.462671778	0.013790609	0.247255508
PHF8	645.686331	0.296305212	0.120320259	2.462637761	0.013791918	0.247255508
CCDC14	959.9351916	0.409016044	0.166128185	2.462050876	0.013814507	0.247411571
ZBTB4	1219.410179	0.54245884	0.22061119	2.458890869	0.013936697	0.249323241
TAF1D	1365.600784	0.435497977	0.177134799	2.458568158	0.01394923	0.249323241
TMEM209	903.806185	-0.261833124	0.106531498	-2.45780008	0.013979097	0.249393158
SNHG3	1068.005054	0.677344382	0.275595559	2.457747808	0.013981132	0.249393158
B3GNT4	56.6877002	0.93147047	0.379135452	2.456827674	0.014016991	0.249782774
MTMR2	969.0923557	0.311788642	0.126981961	2.455377446	0.014073673	0.250542313
C2CD4D-AS1	8.453607054	-1.622084211	0.662583049	-2.448122108	0.014360298	0.255389729
CCSER2	922.2088017	0.331918519	0.135623566	2.447351354	0.014391047	0.25546011
LINC01852	15.24116131	-1.38020793	0.563970705	-2.447304297	0.014392927	0.25546011
DNTTIP1	163.9806751	-0.428759038	0.175261653	-2.446393904	0.014429326	0.255727804
EPHA5	231.0992335	1.12115554	0.458323604	2.446209466	0.01443671	0.255727804
ACYP2	53.26582365	0.733149982	0.300088491	2.443112632	0.014561192	0.257588379
CALM3	4737.889189	-0.263290135	0.107778661	-2.442878152	0.014570656	0.257588379
PRRG1	364.6590524	0.348922751	0.142879193	2.442082327	0.014602816	0.257901074
EWSR1	2589.076006	-0.295682856	0.12115265	-2.44058101	0.014663657	0.258094235
LRRC1	577.6289964	0.54786865	0.224501025	2.440383735	0.014671668	0.258094235
DNMT3A	403.9427433	-0.342875199	0.140502117	-2.440356096	0.01467279	0.258094235
FASTKD1	495.8719046	0.370868572	0.151992542	2.440044534	0.014685451	0.258094235
EFNB3	69.54779164	-0.990196319	0.405813698	-2.44002685	0.01468617	0.258094235
GLI1	34.95811911	-0.89503388	0.367144761	-2.437822827	0.014776014	0.258923384
TMEM33	1972.293172	0.237421454	0.097391099	2.437814705	0.014776346	0.258923384
QPCTL	365.3050401	0.401792448	0.164817631	2.43780016	0.014776941	0.258923384
LPAR2	65.45921184	0.802102899	0.329256904	2.436100471	0.01484656	0.259518833
LOC101928882	69.3632931	-0.863467069	0.354518438	-2.43560553	0.014866887	0.259518833
HES4	82.04179515	0.591223107	0.242751415	2.435508386	0.01487088	0.259518833
SUGCT	1824.44711	-0.541208235	0.222216941	-2.435494941	0.014871433	0.259518833
THEM6	378.8749521	0.378875884	0.15559709	2.434980517	0.014892592	0.259518833
EIF4EBP1	2113.716083	-0.5013936	0.205973471	-2.434262999	0.014922148	0.259518833
ARHGEF9-IT1	2.162889447	4.124945874	1.694684662	2.434049217	0.014930965	0.259518833
ING4	206.1597317	-0.543096595	0.223145523	-2.4338225	0.01494032	0.259518833
ZNF678	420.6413065	0.310293751	0.127494475	2.433781946	0.014941993	0.259518833
STK26	900.3981663	0.311049689	0.1278854	2.432253315	0.015005209	0.260248008
PRKCH	82.804958	-0.608632401	0.250274934	-2.431855206	0.015021711	0.260248008
COG4	1105.898262	-0.289613031	0.119098928	-2.431701409	0.01502809	0.260248008
WDR76	682.4623982	-0.26472964	0.108883471	-2.431311547	0.015044272	0.260248008
MARS1	3622.592781	-0.515622631	0.212131691	-2.430672321	0.015070838	0.260248008
SHC4	23.72365822	-0.987575773	0.406300442	-2.430653946	0.015071602	0.260248008
RMI2	185.8747861	-0.427593804	0.176020505	-2.42922723	0.015131046	0.261021525
GGA3	800.6277946	0.258817705	0.10658141	2.428356925	0.015167408	0.26121036
LY6E	1777.868393	-0.268480822	0.110570088	-2.42815057	0.015176041	0.26121036
MAP1LC3B	769.2339847	-0.406088876	0.167280193	-2.427596891	0.015199226	0.26121036
SORT1	622.6668651	-0.552483013	0.22758746	-2.427563507	0.015200625	0.26121036
EMX2	32.8381595	-1.96183007	0.808449363	-2.426657944	0.015238616	0.261394853
TMEM106A	248.1608529	0.530632977	0.218672691	2.426608347	0.015240699	0.261394853
ACOT7	246.1097036	-0.540600437	0.222951563	-2.424743876	0.01531919	0.262294134
MCFD2	2039.562401	-0.316004129	0.13034177	-2.424427179	0.015332558	0.262294134
ZDHHC3	514.8486787	-0.342988359	0.141478453	-2.424315158	0.015337289	0.262294134
MBOAT2	3127.578833	0.623313232	0.257172649	2.423715092	0.015362653	0.262476003
TPBG	653.7761539	-0.313589021	0.129436722	-2.422720674	0.015404766	0.262943427
HSDL2	748.9684596	-0.318633941	0.131568127	-2.421817111	0.015443121	0.263345845
XAF1	3.620062004	-3.262157643	1.347924335	-2.420134097	0.015514785	0.264314972
AREL1	1300.02078	0.243582074	0.100721225	2.418378794	0.015589838	0.265339942
HCFC1R1	270.8272544	0.401197953	0.166017832	2.416595537	0.015666414	0.26638884
SLC25A19	169.2729121	0.576431009	0.238678528	2.415093697	0.015731162	0.267234808
LOC101448202	88.32458995	1.061184171	0.439673083	2.413575478	0.015796856	0.268095207
AKAP5	154.8081726	0.766733363	0.317754816	2.412971653	0.01582305	0.26827836
PDE3B	473.1823659	0.331405149	0.137362436	2.412633015	0.015837757	0.26827836
PTPRR	22.28516319	1.415408882	0.586850456	2.411873193	0.0158708	0.268362974
OTUD6B-AS1	300.2296904	0.400287229	0.165968563	2.411825606	0.015872872	0.268362974
CBR3	33.89844737	-0.939827766	0.389922054	-2.410296508	0.01593956	0.269115741
YRDC	290.9147838	0.410445865	0.170302634	2.410096987	0.01594828	0.269115741
GREM1	213.8684929	0.721457075	0.299430743	2.409428862	0.015977511	0.269115741
COL15A1	13.50696273	-2.255061251	0.935936907	-2.409415886	0.015978079	0.269115741
VPS37B	652.110094	-0.379479315	0.157520598	-2.409077415	0.015992905	0.269115741
EIF1	5325.214306	-0.380099856	0.157863254	-2.407779171	0.016049886	0.26981978
LINC01787	14.69512734	-1.642479251	0.683328721	-2.403644396	0.016232555	0.272633485
MYNN	1129.611107	0.235281536	0.097924086	2.402693212	0.016274835	0.273086207
OSBPL10	39.07936105	0.987979344	0.41139733	2.401521041	0.01632707	0.273559048
H2AC21	561.0355333	-0.463468515	0.193001538	-2.401372132	0.016333716	0.273559048
DDX31	680.4979521	0.4593677	0.191365593	2.400471756	0.016373954	0.273702084
H2BC13	327.8027213	-0.438291639	0.182587792	-2.400443283	0.016375228	0.273702084
GALK1	323.0127596	-0.397984444	0.165824508	-2.40003392	0.016393553	0.273702084
GCN1	10185.01896	0.204110044	0.085052672	2.39980754	0.016403694	0.273702084
NAPB	161.80277	0.677250849	0.282267317	2.399324352	0.016425358	0.273807184
MED21	320.5878979	-0.322905667	0.134689156	-2.39741399	0.016511257	0.274981867
ASNS	92.86641594	-0.699111882	0.291884374	-2.395167213	0.016612787	0.276157906
LRP4	3068.547081	0.749947965	0.313149508	2.394855961	0.016626896	0.276157906
ROBO2	248.2908001	0.755261937	0.315372681	2.394823595	0.016628363	0.276157906
ATP10D	372.6289547	0.358193908	0.149614889	2.394106027	0.016660932	0.276441158
SAP30L-AS1	58.2778855	0.706065053	0.295145298	2.392262585	0.016744858	0.277575221
TRPT1	60.41963593	0.656419393	0.274520599	2.391148046	0.016795779	0.278160573
ALDH1B1	2483.213167	0.422790547	0.177031052	2.38822818	0.016929827	0.279266812
LOC105369187	3.064449263	-2.808969662	1.176366469	-2.387835539	0.016947924	0.279266812

ZIC2	273.819813	0.565275162	0.236738938	2.387757446	0.016951525	0.279266812
PRSS23	805.2969602	-0.753276308	0.315505456	-2.387522284	0.016962374	0.279266812
TNRC6B	1804.511599	-0.363863291	0.152406852	-2.387447055	0.016965846	0.279266812
ZZEF1	1724.649473	0.220390215	0.092332641	2.386915528	0.016990395	0.279266812
SNX2	1066.454715	-0.289743658	0.121394188	-2.386800088	0.01699573	0.279266812
TMEM102	19.49109814	1.10912496	0.464710314	2.386701835	0.017000273	0.279266812
CRTC3	640.1025505	0.3194596	0.133865001	2.386431079	0.017012796	0.279266812
COCH	1969.952463	-0.286013966	0.119857133	-2.38629073	0.017019291	0.279266812
LOC102723885	13.01126854	1.409398657	0.590722422	2.385889894	0.017037851	0.279314177
FAM131C	26.68781238	0.97331384	0.408054471	2.385254686	0.017067301	0.279364972
PTER	377.6381666	-0.503619523	0.211148223	-2.385146874	0.017072304	0.279364972
TIMP3	2611.288017	-0.738659778	0.309844736	-2.383967493	0.017127115	0.280004764
SH3KBP1	693.2882385	-0.337495487	0.141672507	-2.382222873	0.017208479	0.281043349
CNOT1	6011.798617	-0.26068878	0.109445442	-2.381906228	0.017223283	0.281043349
LRP8	1131.990132	0.568885486	0.238920504	2.381065991	0.017262619	0.281043349
CBWD5	109.8684455	0.542567721	0.227888848	2.380843671	0.017273041	0.281043349
GART	2272.067023	-0.31942799	0.134169122	-2.380786165	0.017275737	0.281043349
ADAMTS5	2065.224383	-0.391594407	0.16449926	-2.380523815	0.017288044	0.281043349
BLMH	1072.559107	-0.254858374	0.107072242	-2.380246909	0.017301041	0.281043349
SIRT3	217.8149371	-0.353721798	0.148713663	-2.378542694	0.017381224	0.282088718
APLP2	2542.540994	-0.350966653	0.147649122	-2.377031768	0.017452585	0.282853676
GLT8D2	746.2038255	-0.315474502	0.132726703	-2.376872895	0.017460103	0.282853676
MCRS1	793.4690003	-0.30641402	0.12895965	-2.376045688	0.017499296	0.28323111
SERTAD2	1234.758293	0.428984529	0.180610521	2.375191247	0.01753986	0.283630036
LOC729652	61.55261547	0.993338558	0.418393843	2.374171068	0.0175884	0.284153087
LOC100268168	20.4439317	1.181437879	0.497690258	2.37384168	0.017604097	0.284153087
FAM72B	144.9804755	0.617965851	0.260411227	2.373038437	0.017642428	0.284514086
MIR7-1	26.98494491	0.930808685	0.392365375	2.37230078	0.017677693	0.284675609
ST6GALNAC4	272.4934082	0.464290178	0.195735151	2.3720327	0.017690525	0.284675609
H1-5	1329.942454	-0.492182423	0.207511927	-2.371827153	0.017700369	0.284675609
YIPF4	1218.449058	-0.26011756	0.109728336	-2.370559595	0.01776118	0.285297931
NIM1K	92.21912144	0.849286194	0.358295157	2.370353538	0.017771083	0.285297931
FKBPL	71.39578147	-0.593232142	0.250424109	-2.368909868	0.017840601	0.286156168
CAPN6	26.23248673	-1.14401146	0.48345253	-2.366336689	0.017965098	0.287893931
TLR8-AS1	47.76935728	-0.891017349	0.376876516	-2.364215626	0.018068293	0.288889045
PTGER1	9.279651756	-2.142864889	0.906389665	-2.364176216	0.018070215	0.288889045
A1BG	112.9429567	0.59934631	0.253552118	2.363799265	0.018088611	0.288889045
PYGO1	1121.580197	0.293003806	0.123972634	2.363455528	0.0181054	0.288889045
ZNF850	304.7676384	0.339768802	0.14376289	2.363397139	0.018108253	0.288889045
CHL1	1.888004663	3.960312196	1.676538824	2.36219534	0.018167067	0.289447127
TNK2	1172.151202	0.422837476	0.179015288	2.362018804	0.01817572	0.289447127
MAN2A1	2578.777833	-0.263269182	0.111483381	-2.361510559	0.018200654	0.289567191
PLRG1	830.9149084	-0.36507823	0.154633017	-2.360933245	0.018229012	0.289567191
RADIL	74.61452519	-0.610842214	0.258735815	-2.360872285	0.018232008	0.289567191
ASS1	150.2692757	-0.701756133	0.297498162	-2.358858717	0.018331233	0.290883863
MIR329-1	3.507581449	-3.113179914	1.320132325	-2.358233229	0.018362152	0.290978516
CNTN6	8.056236325	-2.02283725	0.857909594	-2.357867615	0.018380246	0.290978516
MIR5692C2	3.305583874	3.633706706	1.541177079	2.357747696	0.018386185	0.290978516
GLI4	238.6324555	0.448414477	0.190297483	2.356386799	0.018453691	0.291565781
CHIC2	122.2606197	-0.620853894	0.263517396	-2.356026221	0.018471614	0.291565781
LRRC27	42.96707833	0.757890548	0.321756946	2.355475325	0.018499026	0.291565781
YBX3	1966.555392	-0.27763547	0.117872323	-2.355391514	0.018503199	0.291565781
TTC34	7.401726421	1.945812622	0.826123453	2.355353325	0.018505101	0.291565781
BBS5	79.43063599	-0.511120322	0.217070283	-2.354630557	0.018541129	0.291875373
CC2D1B	708.3378481	0.301386481	0.128064132	2.35340276	0.018602473	0.292062802
SLC2A13	376.3427101	0.381646822	0.162168964	2.35339002	0.01860311	0.292062802
MCC	562.061212	0.393263492	0.167118554	2.353200663	0.018612587	0.292062802
DIPK1C	112.8096913	0.563199568	0.239349919	2.353038479	0.018620707	0.292062802
CHST3	959.5980752	0.421495083	0.17916097	2.352605492	0.018642401	0.292062802
MARCHF9	156.9889529	-0.431723471	0.183534924	-2.35226878	0.018659287	0.292062802
AMOT	1344.973569	0.365825203	0.155540263	2.351964665	0.018674549	0.292062802
PHLDA1	1755.520976	-1.270601159	0.540273629	-2.351773419	0.018684152	0.292062802
DYRK2	1656.441787	0.204831587	0.087133288	2.350784548	0.018733878	0.292284501
NTN3	127.3503822	1.049907845	0.446635271	2.350705183	0.018737874	0.292284501
GSTO1	497.0095184	-0.428235504	0.182189302	-2.350497521	0.018748333	0.292284501
FN3KRP	459.6128479	-0.34223838	0.145621719	-2.350187758	0.018763943	0.292284501
PTPRO	2.809057249	-3.739067498	1.592806671	-2.347471019	0.018901344	0.294167635
FBXO10	235.8156821	0.357028108	0.152225487	2.34538982	0.019007195	0.295386371
TP53BP2	669.3749558	0.262855183	0.112081068	2.345223745	0.019015664	0.295386371
TRIM37	722.511706	0.290207427	0.123761031	2.344901491	0.019032107	0.295386371
SNW1	941.743185	-0.25593331	0.109157215	-2.344630261	0.019045956	0.295386371
TNFRSF21	381.5142439	0.396904497	0.169359035	2.343568477	0.019100256	0.295754816
ZBTB20	663.4730529	-0.332885481	0.142048134	-2.343469576	0.01910532	0.295754816
BDP1	1165.141469	0.316130104	0.134948058	2.342605803	0.019149604	0.295754816
ADGRV1	76.24098613	-0.593222294	0.25323732	-2.342554777	0.019152222	0.295754816
VANGL2	18.19848989	-1.094578281	0.467260194	-2.342545533	0.019152697	0.295754816
GLUD2	48.68563511	-0.769634922	0.328671356	-2.341654995	0.019198453	0.295934047
SF3A1	1793.754424	-0.285686757	0.122011337	-2.341477144	0.019207602	0.295934047
MSRB1	371.2319459	0.317793082	0.135738354	2.341218034	0.019220939	0.295934047
INHBB	4.605548129	-2.780872909	1.187885429	-2.34102788	0.019230731	0.295934047
QSOX1	1600.286146	-0.444961082	0.190107558	-2.340575441	0.019254048	0.296037218
GTPBP2	1330.858211	-0.318071465	0.135979463	-2.339113989	0.019329535	0.296941648
RMND1	414.0190709	-0.371288411	0.158864062	-2.33714539	0.019431626	0.298210884
GET3	567.6267111	-0.269818396	0.115461146	-2.336876135	0.019445626	0.298210884
COLEC12	915.4704131	0.536673346	0.229802862	2.335364066	0.01952441	0.299161638
ALG14-AS1	4.129353184	2.973427538	1.273565473	2.334726875	0.019557693	0.299414172
B4GALT3	549.2301867	0.31238376	0.133853051	2.333781395	0.019607171	0.299818971
SLC25A23	527.9934259	-0.299864906	0.128513831	-2.333327882	0.019630943	0.299818971
GNAQ	1517.257691	0.256989147	0.110152019	2.333040741	0.019646007	0.299818971
SLC31A2	22.89123956	-0.968866085	0.415298803	-2.332937339	0.019651434	0.299818971

BEND5	13.69847778	-1.856049273	0.796272823	-2.330921285	0.019757511	0.300948737
UBE2S	813.8456019	0.360727835	0.154759825	2.330888108	0.019759261	0.300948737
CCND2	7953.929828	-0.337588333	0.144901491	-2.329778181	0.019817878	0.301445817
H2AC11	3429.793492	-0.536142273	0.230140553	-2.329629731	0.019825729	0.301445817
PAFAH1B1	2832.576333	-0.340313273	0.146127729	-2.328875405	0.019865667	0.301795558
OTUD7A	13.87138881	3.046467332	1.308339868	2.328498433	0.019885652	0.301841844
PMPCB	1304.64683	-0.247589727	0.106351198	-2.328038903	0.019910038	0.30195479
RNU4ATAC	2.528814913	-3.634789769	1.561691644	-2.327469563	0.019940287	0.302156391
CES2	481.1757853	-0.358944252	0.154283359	-2.326526037	0.019990505	0.30250223
NOL6	984.3619144	0.38655133	0.166158369	2.326403014	0.019997061	0.30250223
CCDC186	269.0170583	0.345702056	0.148652681	2.325568924	0.020041559	0.302768271
CC2D2A	425.8029748	-0.261317136	0.112376127	-2.325379439	0.02005168	0.302768271
UCHL1	2917.225898	-0.271201932	0.116640042	-2.325118609	0.020065619	0.302768271
ZMYM4	1701.64178	-0.331035934	0.142425363	-2.324276567	0.020110676	0.303191409
DNM3	269.5280081	-0.65444386	0.281699466	-2.323198797	0.020168475	0.303592683
PDGFRA	2152.814889	-0.37183276	0.160065859	-2.322998553	0.02017923	0.303592683
FLCN	984.431095	-0.304587158	0.131151862	-2.322400557	0.020211377	0.303592683
SGCB	3987.470647	-0.225233638	0.097000754	-2.321978223	0.020234108	0.303592683
ATP6V1B2	1943.293186	-0.239006862	0.102936376	-2.321889227	0.020238901	0.303592683
RFTN1	467.9671695	-0.892598643	0.384429625	-2.32187788	0.020239512	0.303592683
IFNAR2	162.0138222	0.614387784	0.2647121	2.320965999	0.020288679	0.303836004
CDC42EP3	1680.427872	-0.64606055	0.278384312	-2.320750567	0.02030031	0.303836004
TFDP2	1050.447335	-0.425416761	0.183319604	-2.320628837	0.020306884	0.303836004
C17orf67	51.21681105	0.76780322	0.331008934	2.319584584	0.020363361	0.304176588
RND2	12.73570747	-1.221215138	0.52648189	-2.319576725	0.020363786	0.304176588
PSME2	527.9375951	-0.353377813	0.152389639	-2.318909711	0.020399932	0.304433836
POMK	1444.799171	0.231562356	0.09987039	2.318628731	0.020415176	0.304433836
PRAME	625.8212278	-0.257140743	0.110958418	-2.317451413	0.020479154	0.305132552
EIF5AL1	54.90087781	-1.393432923	0.601549086	-2.316407681	0.02053602	0.305724204
LINC02208	8.427232713	-1.828527602	0.789527	-2.315978558	0.020559439	0.305817371
H4C8	1405.937928	-0.523487099	0.22616075	-2.314668209	0.020631097	0.306519783
UGCG	741.1685641	0.436024351	0.188407813	2.314258324	0.020653556	0.306519783
MPP4	9.8419818	1.545830494	0.667984136	2.314172465	0.020658264	0.306519783
AKAP13	3002.385805	-0.287102375	0.124103264	-2.313415172	0.020699824	0.306880914
GNL3	1164.931448	0.229806309	0.099358323	2.312904454	0.020727893	0.306940127
ACTR3-AS1	12.29249598	1.293741776	0.559445578	2.31254268	0.020747796	0.306940127
ZFYVE16	1115.097674	0.274566157	0.11874363	2.312260084	0.020763355	0.306940127
TARS3	183.6214763	0.407630037	0.176303695	2.312090153	0.020772716	0.306940127
BAG1	173.578788	-0.444852346	0.192437583	-2.311670822	0.020795831	0.307027092
CLK3	653.3951856	0.251673529	0.108909006	2.310860575	0.020840557	0.307432725
UHRF1BP1	1732.603344	-0.446485259	0.193512261	-2.307271165	0.021039708	0.310095951
SEPTIN2	5133.305687	-0.228713635	0.099139814	-2.306980667	0.021055898	0.310095951
TEF	150.7302428	-0.611391418	0.265055432	-2.306654929	0.021074065	0.310107212
PTDSS2	743.5711613	-0.299165738	0.129739032	-2.305903885	0.021116003	0.310467972
HDAC9	10731.81914	-0.50138947	0.217467489	-2.30558357	0.021133912	0.310475116
ZFAND3	1014.607459	-0.315489152	0.136868468	-2.305053572	0.021163573	0.310507517
SEMA3G	167.2581458	-0.483117966	0.20960278	-2.304921557	0.021170967	0.310507517
IDUA	156.8603878	0.651089176	0.282543148	2.304388484	0.021200846	0.310690027
RHOJ	134.926763	-0.457501838	0.198569455	-2.303988984	0.021223262	0.310762965
ELMOD1	45.22241075	0.718036764	0.311834252	2.302623137	0.021300056	0.311631364
H2BC7	808.0302366	-0.440448225	0.191309763	-2.30227782	0.02131951	0.311660102
PCBP1	4245.221323	-0.296602394	0.128865872	-2.301636495	0.02135568	0.311876906
AAMP	756.4280255	-0.327211708	0.142179757	-2.301394482	0.021369344	0.311876906
MCM6	1285.001588	-0.322060478	0.139961394	-2.301066524	0.021387871	0.311891869
SDC4	182.0220817	-0.464336984	0.201906304	-2.299764665	0.021461556	0.312201724
H2BC15	790.8732975	-0.51984926	0.226047925	-2.299730291	0.021463505	0.312201724
GALNT1	2738.703705	-0.252259081	0.109693271	-2.299676908	0.021466531	0.312201724
ZEB2	1328.407794	-0.396242889	0.172320458	-2.299453547	0.021479198	0.312201724
ZNF587	1442.615169	0.351392651	0.152868668	2.29865711	0.021524418	0.312558345
KLHL17	824.2358069	0.557502193	0.242583102	2.298190549	0.021550946	0.312558345
TRIM52-AS1	30.87079768	0.973500988	0.423699633	2.297620563	0.021583394	0.312558345
KANK1	448.9023713	-0.396808358	0.172712603	-2.297506674	0.021589883	0.312558345
RAB6B	16.33979944	-1.233207958	0.536802274	-2.29732253	0.021600377	0.312558345
UBE2O	1631.870179	0.198335414	0.086338956	2.297171785	0.021608972	0.312558345
EMP2	501.5849797	-0.395643648	0.17226236	-2.296750425	0.021633011	0.312652277
ANXA5	5834.228392	-0.319827857	0.139271555	-2.296433436	0.021651111	0.31266029
DGUOK	599.7197815	-0.299950345	0.130654615	-2.295750089	0.021690175	0.312970779
TINF2	900.0706952	0.326408876	0.142227086	2.29498392	0.021734046	0.313350078
PUDP	557.8855408	-0.308387235	0.134398661	-2.294570737	0.021757737	0.313438053
LRP12	1098.74582	0.257946378	0.112467584	2.29351755	0.021818226	0.313799631
SLC7A11	1270.91535	-1.128285759	0.491982133	-2.293347022	0.021828034	0.313799631
NRP1	1069.087553	-0.301417286	0.131440222	-2.293189105	0.02183712	0.313799631
SLC4A4	78.60804957	-0.602961271	0.262970714	-2.29288373	0.0218547	0.313799631
PDCD4-AS1	30.81082248	-0.838799737	0.365872247	-2.292602794	0.021870883	0.313799631
CMKLR1	88.8109056	-1.297893755	0.566269822	-2.292005869	0.021905305	0.314040649
SLC45A1	3.818984353	-2.670962386	1.165499675	-2.291688659	0.021923615	0.314050503
ETF1	3277.688425	-0.429492267	0.187486436	-2.290791143	0.021975496	0.314540837
BARX1-DT	13.41888252	1.650614534	0.720660467	2.290419149	0.021997031	0.314596376
CBR4	191.0367231	-0.372921437	0.162876748	-2.289592845	0.02204493	0.315028593
ISLR2	27.51521152	1.562185605	0.682559414	2.288717396	0.022095778	0.315240884
APLN	20.21623702	1.125343319	0.491726523	2.288555257	0.022105206	0.315240884
SGMS1	228.2722726	-0.395291663	0.172743195	-2.288319737	0.022118908	0.315240884
RUFY3	324.517497	0.482100554	0.210697254	2.288119775	0.022130547	0.315240884
ZRANB2-AS2	5.628549755	-2.118218226	0.926094136	-2.287260165	0.022180642	0.315702111
ALDH9A1	816.4373575	-0.402364186	0.175954075	-2.286756844	0.02221002	0.315843437
NUDT21	2283.356159	-0.268837824	0.117590363	-2.28622326	0.022241201	0.315843437
FAP	483.6542261	0.30308668	0.132573424	2.286179771	0.022243744	0.315843437
SGK1	208.3322421	-1.00274947	0.438682516	-2.285820456	0.022264764	0.315890209
GAB1	237.641718	0.489978588	0.214439997	2.284921634	0.022317423	0.316385428
SHC1	1690.49152	-0.229962375	0.100657584	-2.284600576	0.022336259	0.316400745

PRKD1	577.7513823	-0.269004737	0.117764625	-2.284257574	0.022356398	0.316434477
RASL10B	335.1690661	-0.333362045	0.145983913	-2.283553292	0.022397797	0.316686848
TMEM64	1676.383571	0.245536236	0.107533342	2.283349817	0.022409771	0.316686848
MIR503HG	22.94417593	-1.289554327	0.56492653	-2.28269387	0.022448407	0.316818186
ZBTB37	969.9493214	0.410079457	0.179667103	2.282440423	0.022463351	0.316818186
FRMD6	2559.50798	-0.494755421	0.216780545	-2.282287009	0.022472401	0.316818186
RAB1A	2600.471708	-0.333839	0.146363804	-2.280884962	0.022555255	0.317734897
AKAP6	283.8141879	-0.360378037	0.158095513	-2.279495663	0.022637617	0.318643241
LINC01291	15.08811169	1.287843864	0.565208824	2.278527526	0.022695166	0.319201157
PRKAA1	1193.274099	-0.205839907	0.090359638	-2.278007205	0.022726148	0.319249007
H3C4	2850.144466	-0.490885533	0.215502115	-2.277868745	0.022734399	0.319249007
IARS1	13962.82168	-0.306274447	0.134502616	-2.277089142	0.022780903	0.31965015
NYNRIN	31.02769887	-0.829691865	0.364587799	-2.275698384	0.022864068	0.320164931
ZNF442	11.46153949	1.647437608	0.723955066	2.275607541	0.02286951	0.320164931
BRWD3	972.7036297	0.30283144	0.133079116	2.275574478	0.02287149	0.320164931
TUBGCP4	775.6299486	0.302766855	0.133088891	2.274922063	0.022910606	0.320460747
PPM1L	109.7619191	-0.467752474	0.205699911	-2.273955643	0.022968654	0.320970854
HDGF	3682.17812	-0.287341852	0.126375465	-2.273715491	0.022983098	0.320970854
ZBTB39	399.8733181	0.269764463	0.118677865	2.273081526	0.023021267	0.321252143
MEX3C	935.6973454	-0.354814046	0.156235283	-2.271023797	0.023145537	0.322495452
GRAMD4	603.7709049	0.416654149	0.183466702	2.271006919	0.023146559	0.322495452
CREBL2	276.0605556	-0.521817756	0.229814424	-2.270604896	0.023170907	0.322559927
FICD	115.843928	0.539405096	0.237588541	2.27033296	0.023187389	0.322559927
SAMD4A	714.3573342	-0.249947869	0.110247459	-2.267153097	0.023380875	0.324997809
UAP1	1910.028286	0.255123813	0.112548078	2.266798483	0.023402539	0.325045395
PIWIL4	44.95890761	0.679229687	0.299709699	2.266291979	0.023433512	0.325101306
UBE2D3	2251.362429	-0.34301574	0.151365904	-2.2661361	0.023443052	0.325101306
KCTD10	1745.646772	-0.22812154	0.100701527	-2.265323554	0.023492832	0.325538305
B4GALT5	1408.226596	0.382941008	0.169145482	2.263974199	0.023575702	0.326369665
TSHR	190.2403839	0.97305222	0.429840731	2.263750619	0.023589457	0.326369665
H2AC8	525.1511552	-0.474349642	0.209598788	-2.263131603	0.023627578	0.326581902
WT1-AS	61.4736668	0.640895509	0.283236271	2.262759312	0.02365053	0.326581902
HNRNPK	8630.327178	-0.338649645	0.149690849	-2.262326968	0.023677209	0.326581902
EME2	194.6606433	0.873194365	0.385986179	2.262242567	0.023682421	0.326581902
H2BU1	5.185151568	-2.283475198	1.009486875	-2.262015738	0.023696431	0.326581902
RRAGC	557.5464278	-0.421337739	0.186343454	-2.261081519	0.02375421	0.327125204
FAM162B	37.88579847	-0.868121978	0.384117978	-2.260039952	0.023818771	0.327761008
FUT1	3.280690437	-2.875854312	1.272984861	-2.259142588	0.023874517	0.328274605
MMP16	1757.340069	0.230485675	0.102065717	2.258208564	0.02393266	0.32882035
VIM-AS1	55.128129	-0.784184667	0.34754522	-2.256352908	0.024048538	0.3301579
MFSD14B	1635.041443	-0.268483243	0.119021758	-2.255749264	0.024086338	0.330218297
SLFN11	1268.507378	0.20553494	0.091122766	2.255582758	0.024096774	0.330218297
CAPZA2	1644.567672	-0.276190251	0.122457583	-2.255395261	0.02410853	0.330218297
C16orf72	1322.799126	-0.320738603	0.142239377	-2.254921311	0.024138268	0.330239178
LOC730338	2.54260861	-3.375855205	1.497358116	-2.25454096	0.024162157	0.330239178
HTRA1	1316.867914	0.775266306	0.343877285	2.254485366	0.02416565	0.330239178
SERINC4	91.66715953	1.03455531	0.459017087	2.253849235	0.024205654	0.330332925
TRPM2	3.578705687	3.101067645	1.375936566	2.253786782	0.024209585	0.330332925
SPTLC2	1871.899045	0.334760753	0.148566786	2.253267782	0.02424227	0.330383042
MINK1	1689.8834	0.217045811	0.09633038	2.253139767	0.024250338	0.330383042
TRIAP1	401.71627	-0.324653358	0.144212516	-2.251214855	0.024371932	0.331655391
METRNL	1697.247157	0.682249842	0.303077698	2.251072406	0.024380952	0.331655391
ITPA	548.7894657	-0.327185883	0.145411648	-2.25006653	0.024444722	0.332269223
KITLG	1159.960503	0.448737451	0.199523528	2.24904529	0.024509615	0.33261603
PABPC1	40165.33704	-0.299795974	0.133311805	-2.248832898	0.024523129	0.33261603
LINC00972	57.72840026	-0.665173642	0.295849083	-2.248354583	0.024553589	0.33261603
BUD31	268.3227995	-0.419053987	0.186394501	-2.248210026	0.024562801	0.33261603
ABL2	2335.760676	-0.304083999	0.135256766	-2.248198062	0.024563563	0.33261603
ELK3	1982.866089	-0.517111038	0.23018272	-2.24652414	0.024670455	0.333809802
EIF2AK3	572.5989483	0.456206333	0.203109718	2.246107858	0.0246971	0.333916787
ANGEL1	979.0417416	0.241151704	0.107379179	2.245795748	0.024717094	0.333933747
ATXN1	1003.275045	-0.267921959	0.119324804	-2.245316576	0.024747817	0.334095525
B4GALNT3	303.5218945	0.361802586	0.16120412	2.244375561	0.024808248	0.334310551
RNF185	438.627509	-0.260244192	0.115955193	-2.244351337	0.024809805	0.334310551
EGR3	57.44336366	-1.272357338	0.566955554	-2.244192385	0.024820026	0.334310551
CFL2	669.3147231	0.311446181	0.138803784	2.243787401	0.024846083	0.33438452
ADRB1	4.423164227	2.915656543	1.299587878	2.243523961	0.024863047	0.33438452
DARS1	1062.262809	-0.252840583	0.11273761	-2.242735005	0.024913908	0.334418381
LRIG2	1164.306191	0.233084838	0.103947465	2.242333053	0.024939855	0.334418381
STOM	1084.613888	-0.332663907	0.148365258	-2.242195453	0.024948743	0.334418381
ARHGAP11A	1990.116449	0.286581524	0.127819167	2.242085677	0.024955835	0.334418381
SGCD	406.5706216	-0.325862497	0.145342575	-2.242030571	0.024959397	0.334418381
NPNT	125.3538261	0.618304122	0.275877101	2.241230315	0.02501116	0.334664617
NACC1	1204.381998	0.211780968	0.094509513	2.240842858	0.025036256	0.334664617
GAB2	224.2213967	0.647135995	0.288810582	2.240693502	0.025045935	0.334664617
MBOAT7	552.0752347	0.280643749	0.125257278	2.240538456	0.025055987	0.334664617
DOK6	226.207997	0.67358353	0.300688775	2.240135273	0.025082142	0.334664617
TSPAN6	599.9133249	-0.313951526	0.140156481	-2.240007189	0.025090456	0.334664617
IL17RA	289.5981237	-0.363555858	0.162486871	-2.237447593	0.025257101	0.335264433
STK10	634.8952482	-0.254279143	0.113647409	-2.237438978	0.025257663	0.335264433
SLC35F1	7.404687531	1.62606054	0.726844323	2.237151051	0.025276469	0.335264433
H1-4	3360.085748	-0.322215757	0.14404545	-2.236903403	0.025292654	0.335264433
ARHGEF9	291.1775783	-0.333783756	0.149222058	-2.236825841	0.025297725	0.335264433
ZFAND1	734.0646262	-0.254868998	0.113950831	-2.236657649	0.025308724	0.335264433
POLG	1840.695885	0.227521415	0.101724711	2.236638595	0.02530997	0.335264433
SEC16B	5.459699684	2.269497141	1.014704331	2.236609298	0.025311887	0.335264433
MRTFA-AS1	24.94455683	1.535741696	0.686661003	2.236535479	0.025316716	0.335264433
IPO5	5177.484531	-0.218343627	0.097630397	-2.236430809	0.025323565	0.335264433
ERH	1123.360844	-0.312504285	0.139830787	-2.234874682	0.025425577	0.336235865
PHF13	740.617338	-0.277755942	0.124290263	-2.23473613	0.025434677	0.336235865

LMO7	932.27668	0.523815938	0.2344411	2.234317863	0.025462166	0.336349736
ELAC1	56.99137898	-0.536806639	0.24035377	-2.233402205	0.025522433	0.336896114
HSD17B12	1495.37142	-0.294085952	0.13170128	-2.232977182	0.025550449	0.337016287
UBE2B	500.597396	-0.487781792	0.218519723	-2.232209458	0.025601123	0.337342579
PCA3	13.57723734	2.068581555	0.926771818	2.232028979	0.025613048	0.337342579
PDZD4	4.122488969	-2.480889581	1.113024285	-2.228962669	0.025816389	0.339769606
PREB	1122.352877	0.202979766	0.091095244	2.228214759	0.025866197	0.339940967
DDX50	902.3283849	-0.313187787	0.140556768	-2.228194289	0.025867562	0.339940967
MIR4784	7.385950587	2.153583915	0.966753401	2.227645553	0.02590416	0.340171068
HSPA1B	796.0142811	0.598681062	0.26888945	2.226495175	0.025981032	0.340929298
ATP5MC3	1878.938758	-0.346354848	0.155711891	-2.224331394	0.026126156	0.342538613
RUBCN	1310.6743	0.337945895	0.151947663	2.224094062	0.026142116	0.342538613
SAMHD1	286.0251297	-0.326733801	0.146970903	-2.223118964	0.026207779	0.342863317
SGO2	795.4835208	0.256955443	0.115587644	2.223035552	0.026213403	0.342863317
MKLN1-AS	53.34427845	-0.787705645	0.354404863	-2.222615226	0.026241757	0.342863317
PDIA3P1	163.6084437	-0.883450919	0.397498456	-2.222526669	0.026247734	0.342863317
PRB4	8.114102444	2.117051763	0.952640338	2.222299097	0.026263099	0.342863317
UBE2H	1627.7458	-0.335589343	0.151100129	-2.220973236	0.026352774	0.343672151
RUNX1T1	718.8075641	-0.502440296	0.226253026	-2.220700889	0.026371227	0.343672151
SFXN3	281.4567154	-0.670290496	0.301860793	-2.220528509	0.026382913	0.343672151
LRP4-AS1	36.40732927	1.16391485	0.524591392	2.21870749	0.026506631	0.345009508
LDLRAD3	290.103086	0.293897279	0.132478776	2.218448021	0.0265243	0.345009508
SNHG6	242.8191446	0.395842094	0.178548513	2.217000234	0.026623076	0.346041726
ZNF652	1089.951307	0.416863867	0.188166989	2.215393192	0.026733088	0.346493674
JMJD8	826.9775287	0.231673402	0.104580023	2.215273963	0.026741266	0.346493674
EDA2R	2.656836062	-3.14766346	1.420930721	-2.215212476	0.026745484	0.346493674
TOM1L1	92.44609196	-0.655367003	0.295858228	-2.215138672	0.026750548	0.346493674
BUB1	1166.95636	0.267731246	0.120881482	2.214824322	0.026772125	0.346493674
PNPLA4	106.8856384	-0.56564442	0.255394204	-2.214789574	0.026774511	0.346493674
PPP2R5D	892.6919311	-0.241395275	0.109007766	-2.214477762	0.026795931	0.346519222
CPT1C	39.4485493	-0.947791573	0.428199417	-2.213434991	0.026867671	0.34691508
PTPRS	1266.067023	-0.315806432	0.142699847	-2.213081782	0.026892008	0.34691508
TSPAN14	593.0709475	0.278238829	0.125728976	2.213004817	0.026897314	0.34691508
LGALS2	16.37579192	1.51966112	0.686727755	2.212901851	0.026904413	0.34691508
PLS3	4197.127697	-0.308091904	0.13924306	-2.212619461	0.026923892	0.346915226
ASB6	594.8793862	0.306034226	0.13834011	2.212187236	0.02695373	0.347048754
KLF4	294.0127706	-0.734163184	0.332135932	-2.210429867	0.027075343	0.348142104
DTWD2	200.8301778	-0.425875477	0.192669347	-2.210395596	0.027077719	0.348142104
EXOSC5	339.8145216	-0.374269945	0.169357097	-2.209945438	0.027108949	0.34829234
WNT11	57.85248947	0.952350023	0.431027002	2.209490398	0.02714055	0.348447113
PPP1R3D	188.1213372	0.367487615	0.166360518	2.208983357	0.027175799	0.348469923
ATP7B	491.4827212	0.276850778	0.12534285	2.208748069	0.027192169	0.348469923
TGM4	2.308760609	-3.368888752	1.525335612	-2.20862132	0.027200991	0.348469923
H2BC11	2069.936293	-0.468317913	0.212078323	-2.208230936	0.027228179	0.348567635
UBALD2	141.8195809	-0.801368922	0.362989257	-2.207693223	0.027265666	0.348796957
LMO4	540.9916587	-0.359382278	0.162871621	-2.206537132	0.027346413	0.34917777
SEPTIN7	3065.133245	-0.357913696	0.162208995	-2.206497218	0.027349205	0.34917777
DST	9074.072144	0.266339565	0.120710878	2.206425545	0.027354218	0.34917777
RASSF9	56.06670822	-0.60211024	0.273012256	-2.205433008	0.027423724	0.349814431
STAG1	804.0517664	-0.380615169	0.172644105	-2.204623027	0.027480559	0.350288665
MROCKI	13.65407938	1.65071872	0.7491884	2.203342603	0.02757061	0.35118533
IL6R	97.3766316	0.659953723	0.299624618	2.202601798	0.027622827	0.351352494
PLB1	123.7056033	0.940179378	0.426850391	2.202596973	0.027623167	0.351352494
SMARCA1	717.1137106	-0.362090759	0.164431723	-2.202073622	0.027660108	0.351356333
PCSK5	214.2738603	-0.577697295	0.262402809	-2.201566736	0.027695928	0.351356333
MED15	721.1389238	0.277536466	0.126092738	2.20105036	0.027732459	0.351356333
FAM9C	586.6767834	-0.49181513	0.223457262	-2.200935986	0.027740556	0.351356333
ATF1	271.1081298	-0.427563107	0.194266054	-2.200915177	0.027742029	0.351356333
DNHD1	859.5210131	0.688505756	0.312883416	2.200518535	0.027770126	0.351356333
TCF7L2	345.5159464	-0.322994151	0.146793172	-2.200334975	0.027783138	0.351356333
ALAD	556.9765931	-0.292025483	0.132729194	-2.200160149	0.027795535	0.351356333
ATXN2	1119.780585	0.220259995	0.100114348	2.200084196	0.027800922	0.351356333
DHFR	432.4242841	-0.466399761	0.212112939	-2.198827491	0.027890191	0.352234729
TSPYL5	126.9840308	-0.464392044	0.21141822	-2.196556404	0.028052143	0.354029166
LUM	56818.33179	0.238098533	0.108435506	2.195761726	0.028109003	0.354387781
BTN3A2	617.0269361	0.382454297	0.174208451	2.195383145	0.028136125	0.354387781
ZMYND8	646.4690294	-0.402268885	0.183249327	-2.195199794	0.028149269	0.354387781
RBM24	18.50297199	1.192548545	0.543353667	2.194792487	0.028178487	0.354387781
KLHL15	430.3252079	0.314956228	0.14350297	2.194771489	0.028179994	0.354387781
RNF138P1	45.30067508	1.053526961	0.480167163	2.194083733	0.02822939	0.354733638
ERI3-IT1	47.29427319	1.145247612	0.522030081	2.19383452	0.028247308	0.354733638
LRRTM3	79.21091745	0.815674103	0.372198363	2.191503737	0.02841536	0.356459482
ARHGAP28	1203.745686	-0.394520365	0.180033338	-2.191373942	0.028424743	0.356459482
PRPS2	929.3328476	-0.286403502	0.13081237	-2.189422173	0.02856617	0.357792789
DNAH11	71.72507045	-0.755259903	0.345012297	-2.189081117	0.028590945	0.357792789
VAPB	1270.392628	-0.233659495	0.10674812	-2.188886288	0.028605106	0.357792789
INTS8	1451.709691	0.249958663	0.114213128	2.188528292	0.028631143	0.357792789
EGR2	11.89663513	-1.311187091	0.599119354	-2.188524009	0.028631454	0.357792789
EVA1B	206.3644311	-0.439523123	0.200969091	-2.187018521	0.028741172	0.358442257
POFUT2	1646.063563	0.33533025	0.153337651	2.186874835	0.028751662	0.358442257
LDHB	5564.489507	-0.255768479	0.116956928	-2.186860433	0.028752714	0.358442257
LOC101927402	1.865669489	3.656776027	1.672274894	2.186707485	0.028763885	0.358442257
UFSP2	241.8878338	-0.359545005	0.164470671	-2.186073678	0.028810215	0.35876872
NOX5	19.31103058	1.14237688	0.522659409	2.185700403	0.028837531	0.358858107
ARHGEF17	638.3710648	0.317207198	0.145225531	2.184238517	0.028944726	0.359565894
OPLAH	51.31925167	-0.795303947	0.364129522	-2.184123778	0.028953154	0.359565894
RSRP1	705.5325954	0.427843365	0.195892673	2.184070274	0.028957085	0.359565894
DENR	1710.666463	-0.3148745	0.144184867	-2.183824872	0.028975119	0.359565894
SNORD114-20	406.2852043	-0.485840602	0.222509022	-2.183464733	0.029001603	0.3596441
RBBP6	674.6714681	0.252955624	0.115887616	2.182766647	0.029052999	0.359701969

PLXNA1	3322.309684	-0.243525912	0.111576124	-2.182598771	0.02906537	0.359701969
ODF3B	66.87485909	0.848293033	0.388665438	2.182579025	0.029066826	0.359701969
BRD3OS	408.5105277	-0.282471824	0.129446594	-2.18214952	0.029098499	0.359844039
SNORD32A	16.11694363	1.433671872	0.657125633	2.181731771	0.029129335	0.35997555
LINC01546	4.671678271	2.181899097	1.001067022	2.179573444	0.029289095	0.36106143
POLR3A	873.2333981	0.206678546	0.094826778	2.179537794	0.02929174	0.36106143
SNORA72	54.9318138	0.725173649	0.332733516	2.17944275	0.029298793	0.36106143
COX19	403.8983544	-0.335466761	0.153929162	-2.179358073	0.029305078	0.36106143
TAZ	418.219851	0.253769783	0.116452114	2.179177129	0.029318512	0.36106143
HAO1	9.571816189	-1.865192065	0.856415116	-2.17790652	0.029412997	0.361974866
SPATA6	41.33262119	-0.751072574	0.344993809	-2.17706102	0.029476015	0.362091476
GBP5	218.2190623	-0.540198115	0.248147164	-2.176926409	0.029486059	0.362091476
UNG	857.1885049	-0.281596308	0.1293636	-2.176781621	0.029496865	0.362091476
NUTM2B	3.908222495	2.89331917	1.329544232	2.176173685	0.029542276	0.362091476
CLCC1	317.76307	0.27271243	0.125317959	2.176163991	0.029543	0.362091476
RPL7L1	5311.913923	-0.222695331	0.102334765	-2.176145419	0.029544389	0.362091476
LRRC8C	347.5248303	-0.34842701	0.160273403	-2.17395403	0.029708588	0.363853631
CTAG2	683.6270731	-0.3188297	0.146681461	-2.173619606	0.029733715	0.363911262
CHRAC1	498.0892578	-0.271162442	0.124795255	-2.172858589	0.029790962	0.364361664
SYAP1	847.6968505	-0.258587276	0.119024087	-2.172562573	0.029813255	0.364384232
UBE2Q2	1207.760302	-0.212360769	0.097775001	-2.171933182	0.029860703	0.364527332
LRATD1	240.8551426	0.598066785	0.275415827	2.171504776	0.029893037	0.364527332
FDX1	255.5766512	-0.315709061	0.145396185	-2.171371006	0.029903139	0.364527332
MCM4	7549.735798	-0.19630197	0.090414266	-2.171139341	0.029920641	0.364527332
LOC100129203	12.62677246	1.503138202	0.692354776	2.171051972	0.029927244	0.364527332
NME1-NME2	42.70624495	0.69147673	0.318662418	2.16993499	0.030011771	0.365307215
RAB9B	16.06795882	-1.07485663	0.495434603	-2.169522723	0.030043021	0.365437977
FBXL8	164.5274422	0.666411316	0.307338588	2.168329466	0.030133628	0.366042107
NRIP1	1023.330523	0.243610867	0.112349659	2.168327607	0.030133769	0.366042107
SERTAD4-AS1	21.94619547	-1.079612721	0.498154511	-2.167224618	0.030217731	0.366811968
NR1D1	163.6942888	-0.486025659	0.224393464	-2.165952829	0.030314792	0.367518525
RELB	42.57735286	-0.927223746	0.42809665	-2.165921518	0.030317185	0.367518525
RMDN1	783.9890689	-0.261211556	0.120620436	-2.165566344	0.030344341	0.367597654
BARD1	640.6025252	-0.240477382	0.111088473	-2.164737481	0.030407795	0.368116105
FSTL3	85.80840569	0.546413466	0.252501919	2.163997284	0.030464558	0.368416836
LMCD1-AS1	5.024636999	-1.9301833	0.892003363	-2.163874465	0.030473985	0.368416836
SYNPO	53.37028368	-0.788734948	0.364692221	-2.162741358	0.030561078	0.369219264
SLC39A4	154.2215384	0.457870029	0.211849944	2.161294073	0.03067263	0.370069336
GRB14	23.75709425	0.961800054	0.445017184	2.161264978	0.030674877	0.370069336
PARN	780.7367589	-0.305282326	0.141288392	-2.160703524	0.030718249	0.370069336
QRSL1	376.3684941	0.312440129	0.144602718	2.160679504	0.030720105	0.370069336
[truncated: 1,569,381 more chars]
